# Voluminous continental growth of the Altaids and its control on metallogeny

**DOI:** 10.1093/nsr/nwac283

**Published:** 2022-12-19

**Authors:** Tao Wang, He Huang, Jianjun Zhang, Chaoyang Wang, Guangyue Cao, Wenjiao Xiao, Qidi Yang, Xuewei Bao

**Affiliations:** Beijing SHRIMP Center, Institute of Geology, Chinese Academy of Geological Sciences, Beijing 100037, China; Key Laboratory of Earth Probe and Geodynamics, Chinese Academy of Geological Sciences, Beijing 100037, China; Key Laboratory of Earth Probe and Geodynamics, Chinese Academy of Geological Sciences, Beijing 100037, China; Key Laboratory of Earth Probe and Geodynamics, Chinese Academy of Geological Sciences, Beijing 100037, China; Beijing SHRIMP Center, Institute of Geology, Chinese Academy of Geological Sciences, Beijing 100037, China; Key Laboratory of Earth Probe and Geodynamics, Chinese Academy of Geological Sciences, Beijing 100037, China; Beijing SHRIMP Center, Institute of Geology, Chinese Academy of Geological Sciences, Beijing 100037, China; National Key Laboratory of Arid Area Ecological Security and Sustainable Development, Xinjiang Institute of Ecology and Geography, Chinese Academy of Sciences, Urumqi 830011, China; Wuhan Center of China Geological Survey, Wuhan 430205, China; School of Earth Sciences, Zhejiang University, Hangzhou 310058, China

**Keywords:** 3D lithospheric architecture, architecture evolution, juvenile crust, igneous rocks, isotopic mapping, metallogeny, Central Asian Orogenic Belt (CAOB)

## Abstract

The Altaids is generally considered to be the largest Phanerozoic accretionary orogen on Earth, but it is unclear whether it was associated with extensive continental crustal growth and whether there is a link between the crustal growth and ore mineralization. This paper reviews whole-rock Nd and zircon Hf isotope data for felsic–intermediate–mafic igneous rocks in the Altaids and presents Nd + Hf isotopic contour maps for this region. The maps highlight the 3D lithospheric compositional architecture of the Altaids and make it possible to quantitatively evaluate the crustal growth and its relationship with ore deposits. The Altaids hosts ∼4 107 350 km^2^ and ∼184 830 750 km^3^ (assuming a crustal thickness of 40–50 km) juvenile crust (*ϵ*_Nd_(*t*) > 0), accounting for 58% by isotope-mapped area (∼7 010 375 km^2^) of almost all outcrops of the Altaids (∼8 745 000 km^2^) and formed during 1000–150 Ma (mainly 600–150 Ma). The juvenile crustal, slightly juvenile-reworked crustal and slightly reworked crustal provinces controlled the Cu–Au, the Pb–Zn–Ag and the Li–Be, Nb–Ta and W–Sn ore deposits. According to the crustal architecture and background of deep compositions, we propose that the ore deposits can be grouped into three types: juvenile crust-related, mixed-source (or slightly juvenile crust)-related and reworked crust-related. This highlights the close relationship between accretion, continental growth and mineralization, and will facilitate exploration for specific ore-deposit types in the Altaids.

## Teaser

Nd + Hf isotopic mapping of igneous rocks demonstrates voluminous Phanerozoic continental growth and its crustal architecture in the Altaids, which control the distribution of the ore deposits and allow the deposits to be grouped into three types: juvenile crust-, mixed source-, and reworked crust-related. This highlights the relationship between continental growth and mineralization.

## INTRODUCTION

Earth is the only planet in the Solar System known to have a granitic (felsic) continental crust [1]. The continental crust records Earth's history and hosts mineral resources [2–4]. The growth and reworking of the continental crust, and its link to ore resources are some of the most contentious areas of Earth Sciences and essential to understanding the origins of the metallic resources [5–13]. Continental crustal growth refers to the addition of mantle-derived materials (e.g. magmas) and their differentiation products to the continental crust via a range of geological processes, which increase the crustal area and volume [14,15]. It is generally considered that converging plate margins where ocean crust is subducted beneath continents are the most important sites for crustal growth, such as island arc accretion belts in the West Pacific (e.g. the Japan arc), the Cordilleras in North America and multiple oceanic subduction systems in Indonesia [13,16,17]. However, the onset time of plate tectonics is controversial, ranging from Hadean to Phanerozoic (e.g. [18]). Some models suggested that there may have been huge crustal growth early on [13,19,20].

In addition, numerous studies have argued that subduction can also cause crustal consumption [4,21,22]. At denudation- or advancing-type subduction zones, crustal materials can be transported into the mantle. Therefore, it is generally thought that continental growth has been <10% of the entire continental crust or almost zero since the Phanerozoic during which modern plate tectonics was dominated and ∼80%–90% of the continental crust had formed by the early Precambrian prior to 1.8 Ga [2,4,23,24].

The identification of significant crustal growth in the Phanerozoic Altaids [25–27], or the Central Asian Orogenic Belt (CAOB, [28–30]), challenges this concept [28,31–33]. Şengör *et al.* [25] speculated that nearly 50% of the Altaids may be juvenile. This was evidenced by the study of voluminous granites and their primitive isotopes [28,31,34–41]. Thus, the Altaids is considered to be Earth's largest Phanerozoic accretionary orogen and also the most important site of Phanerozoic crustal growth [25–29,31,42,43]. More recently, Kusky and Şengör [44] took the Altaids as the largest preserved Phanerozoic juvenile crustal region to compare it with the Archean Superior Province of the North American Craton to test for similarities or differences in the formation of continents through time and to confirm the onset time of accretionary orogenic systems and plate tectonics in the Archean Eon. On the other hand, the Altaids also comprises numerous ancient crustal blocks and microcontinents [45–49]. Even in the juvenile crustal regions, an increasing number of ancient materials have been identified. Therefore, some previous studies proposed that the volume of the crustal growth in the Altaids may have been overestimated and the Altaids is not different from other orogens [46,47]. Consequently, whether or not voluminous crustal growth occurred in the Altaids remains controversial. In addition, the Altaids contains numerous giant ore deposits [9,32,49–52], which are unlike those in Cenozoic subduction zones (the North American Cordillera and the Andes) and collisional settings (the Tethyan Himalaya). Many studies have been carried out on the ore deposits in the Altaids, but most have focused on porphyry (Cu) deposits [36,50–54]. The complex accretionary architecture of the Altaids, particularly the variable types of crust and their link to the ore deposits, remains unclear.

In this paper, we review 5507 whole-rock Sm–Nd and 39 514 (2443 samples) zircon Lu–Hf isotope analyses of igneous rocks as well as 1830 magma-related metallogenic data from the Altaids. We generate isotopic maps for felsic–intermediate–mafic igneous rocks in different periods. We use the maps to define the spatio-temporal distribution and relative proportions of juvenile and ancient crust provinces, and to delineate the 3D lithospheric architecture of the Altaids and how it has evolved through time. Our results indicate that extensive Neoprotozoic–Phanerozoic crustal growth occurred in the Altaids. Based on this, the relationship between the (juvenile and reworked ancient) crust and ore-deposit formation is discussed.

## TECTONICS AND IGNEOUS ROCKS OF THE ALTAIDS

**Figure 1.**
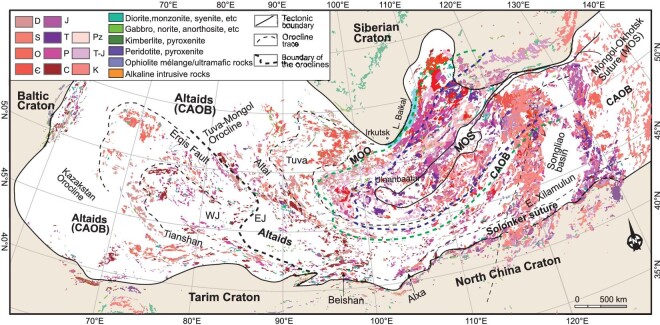
Tectono-magmatic map of the Altaids showing igneous rocks (modified from Ref. [61]). The major tectonic units are modified after Refs [26,43]. MOO, Mongol-Okhotsk orogen; MOS, Mongol-Okhotsk suture; WJ, West Junggar; EJ, East Junggar; Є, Cambrian; O, Ordovician; S, Silurian; D, Devonian; C, Carboniferous; P, Permian; T, Triassic; J, Jurassic; Pz, Paleozoic. The numbers 1, 2 and 3 that follow P, C, T and J represent the early, middle and late phases, respectively. Green and violet dotted lines denote the margins of the Mongol-Okhotsk orogen in the late Paleozoic and early Mesozoic, respectively.

The Altaids (Altaid tectonic collage [25–27,55], Central Asian Orogenic Supercollage [56], Central Asian Fold Belt [57], the Central Asian Orogenic Belt (CAOB; [28,29,30]) or the Phanerozoic Altaid orogenic systems [44]) is located between the Siberian, Baltic (the East European Cratons) and Tarim-North China cratons and occupies ∼8 745 000 km^2^ [26,27]. The Altaids evolved from the late Precambrian (Ediacaran: 635 Ma) to the Early Cretaceous (∼145 Ma) [27]. It was mainly formed by the subduction/accretion of the Paleo-Asian Ocean and the subsequence collision of ancient terranes, microcontinents and continental fragments as well as island arcs and accretionary complexes between the Siberia and Tarim-North China Cratons [25,27,30,43]. The accretion started mainly at ∼600 Ma (locally ∼1000 Ma) and came to an end with the late-Permian-to-Triassic collision [25–27,30,43,58,59]. Thus, the orogenic system is a complex collage of diverse terranes, including micro-cratons, island arcs, continental-margin arcs, forearc/back-arc regimes, accretionary complexes, and ophiolites and microcontinent fragments. The system is characterized by a series of oroclines, including the Kazakhstan orocline in the western Altaids and the Tuva-Mongolian orocline in the eastern Altaids [25,30,43,55,60]. In addition, the eastern Altaids was formed by the subduction/accretion (350–150 Ma) and closure (∼150 Ma) of the Mongol-Okhotsk Ocean, forming the Tuva-Mongol orocline [25,55,58,61] (Fig. 1).

The long-lived accretionary and post-accretionary orogeny produced voluminous igneous rocks (mostly granitoids), which account for ∼50%–70% of the present outcrop of the Altaids [28,31,62] (Fig. 1). All these igneous rocks in the western and southern Altaids were mostly generated during the syn-orogenic period (600–250 Ma) and a few during post-orogenic (240–200 Ma) [62,63] in the Paleo-Asian Ocean regime. Many late Paleozoic–Mesozoic igneous rocks in the eastern Altaids were formed by the subduction/accretion (350–150 Ma) of the Mongol-Okhotsk Ocean. A few of the rocks (150–120 Ma) were produced in a post-collisional setting.

## SCALE OF CONTINENTAL GROWTH IN THE ALTAIDS

### Nd + Hf isotopic mapping

A database of whole-rock Sm–Nd (*n* = 5507 with ages) and zircon Lu–Hf (*n* = 2443 samples and 39 514 individual data) isotope data for felsic–intermediate–mafic rocks from the Altaids was compiled from the literature (Supplementary Tables S1 and S2). These data are available and were used to conduct a series of isotopic maps. The isotopic mapping covers ∼7 010 375 km^2^, including almost all the outcrops of the Altaids, except the largest Cenozoic basin of the northwestern Altaids.

The major orogeny, particularly major magmatism, in the Altaids occurred from ∼600 to 150 Ma (see above). Therefore, Nd and Hf isotopic data of this period were selected and compiled to construct isotopic maps. To obtain more isotope data for mapping, isotope data for igneous rocks older than 600 Ma and younger than 150 Ma were also used, but the isotopic parameters (e.g. *ϵ*_Nd_(*t*) and model ages (*T_DM_*)) were recalculated to ages of 600 and 150 Ma, respectively (Supplementary Tables S1 and S2).

**Figure 2.**
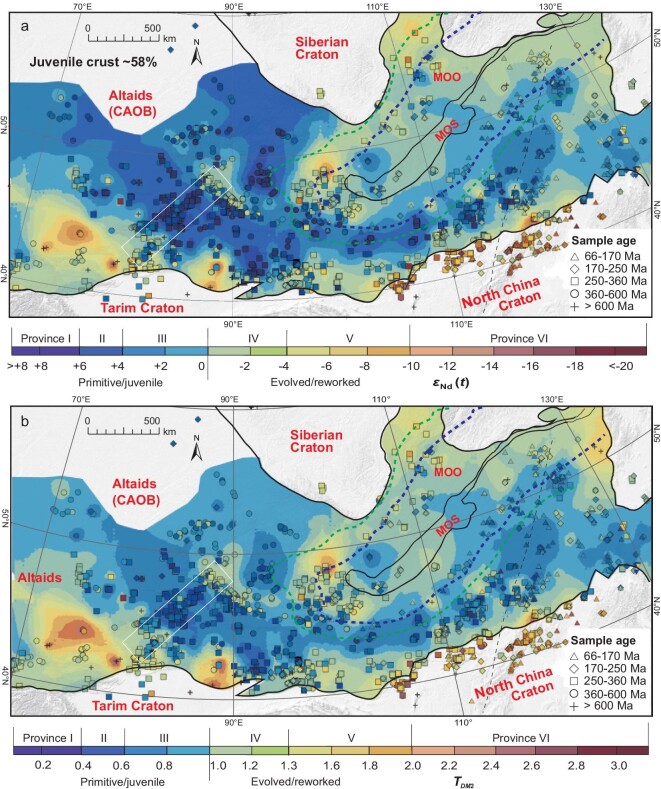
Contour maps of (a) *ϵ*_Nd_(*t*) values and (b) two-stage Nd depleted mantle model ages (*T_DM_*_2_; Ga) for felsic–intermediate igneous rocks in the Altaids, showing spatial variations of *ϵ*_Nd_(*t*) and *T_DM_*_2_ values, and the distribution of Nd isotope provinces. See the method and explanation in the text. The data set is presented in Supplementary Table S1. For abbreviations and other legends, see Fig. 1.

Neodymium isotope contour maps of *ϵ*_Nd_(*t*) (Fig. 2a) and two-stage Nd depleted mantle model ages (*T_DM_*_2_; Fig. 2b) were conducted for the Altaids. The *ϵ*_Nd_(*t*) and *T_DM_*_2_ values exhibit large variations (Supplementary Table S1) but a good linear correlation (Supplementary Fig. S1). So both maps have a similar pattern.

We also produced zircon Hf isotope (*ϵ*_Hf_(*t*)) and Hf depleted mantle model age (*T_DM_*_2_; Ga) maps (Supplementary Table S2 and Supplementary Fig. S2). As the *ϵ*_Hf_(*t*) and Hf *T_DM_*_2_ values have a good linear correlation: *y* = –60.702*x* + 1228.4, *R*² = 0.988 (Supplementary Fig. S3), the maps of the *ϵ*_Hf_(*t*) and Hf *T_DM_*_2_ values are almost the same in an isotopic province pattern (Supplementary Fig. S2a and b).

From a statistical analysis of 220 samples, we obtained good linear correlations between *ϵ*_Hf_(*t*) and *ϵ*_Nd_(*t*) values, with *ϵ*_Hf_(*t*) = 1.0652 × *ϵ*_Nd_(*t*) + 5.9766 for the intermediate–felsic igneous rocks (Supplementary Fig. S4) and *ϵ*_Hf_(*t*) = 1.0132 × *ϵ*_Nd_(*t*) + 7.1354 mafic rocks (Supplementary Fig. S5), which are consistent with those of Huang *et al.* [64]. These correlations have a similar slope to that of the proposed terrestrial array (*y* = 1.36*x* + 2.95; Vervoort *et al.* [65]) despite the small difference in the intercept. Based on these equations, the *ϵ*_Hf_(*t*) values can be converted into *ϵ*_Nd_(*t*) and viewed as equivalent to corresponding *ϵ*_Nd_(*t*) values for isotopic mapping.

**Figure 3.**
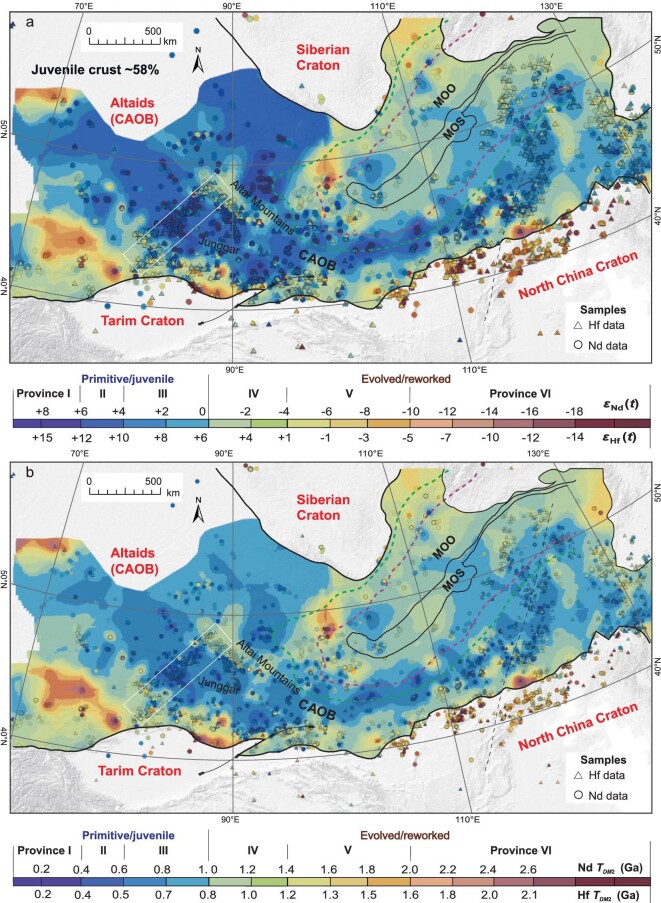
Contour maps made by a combination of (a) *ϵ*_Nd_(*t*) values and zircon median *ϵ*_Hf_(*t*) values and (b) Nd and Hf depleted two-stage Hf depleted mantle model ages (*T_DM_*_2_, Ga) of felsic–intermediate rocks of the Altaids. *t* = 600–150 Ma. See the explanations in the text and data set in Supplementary Tables S2a and S3a. For abbreviations and other legends, see Fig. 1.

**Figure 4.**
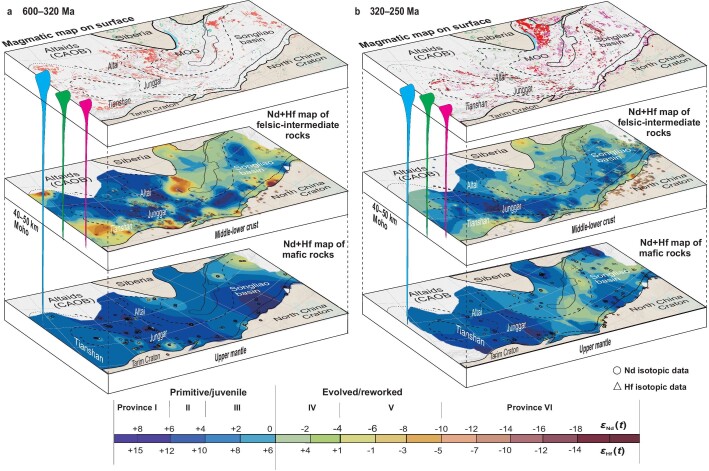
Contour maps of (a) whole-rock *ϵ*_Nd_(*t*) and zircon *ϵ*_Hf_(*t*) values of felsic–intermediate and mafic igneous rocks at (a) 600–320 and (b) 320–250 Ma, showing the deep crust and mantle architecture (3D) and its evolution (4D). For abbreviations and other legends, see Fig. 1.

In general, an *ϵ*_Nd_(*t*) value of 0 separates juvenile and evolved crust [14,66]. Based on the equations, an *ϵ*_Hf_(*t*) value of +6 is equivalent to an *ϵ*_Nd_(*t*) value of 0 and distinguishes juvenile and evolved crust. As a result, the whole-rock Nd and zircon Hf isotopic maps show the same or similar isotopic province patterns (Fig. 2 and Supplementary Fig. S2). Both the Nd and Hf isotopic maps are similar to our maps for xenocrysts and inherited zircons in igneous rocks, revealing the same patterns of juvenile ancient crustal distribution in depth [67]. In this way, we combined Nd and Hf data to produce Nd + Hf isotopic mapping to ensure enough sample data for the isotopic mapping (Fig. 3), particularly for the 3D and 4D mapping (Fig. 4).

The Nd and Hf isotopic mapping of felsic–intermediate rocks revealed six types of isotopic provinces: (I) a highly primitive (i.e. juvenile) province characterized by *ϵ*_Nd_(*t*) value > +6, *ϵ*_Hf_(*t*) > +11, and with young Nd (*T_DM_*_2_ < 0.4 Ga) and Hf (*T_DM_*_2_ < 0.3 Ga) mode ages; (II) primitive provinces with *ϵ*_Nd_(*t*) = +4 to +6, relatively young *T_DM_*_2_ values (0.4–0.8 Ga), *ϵ*_Hf_(*t*) = +10 to +16 and relatively young Hf model ages (0.3–0.7 Ga); (III) slightly primitive provinces (i.e. slightly primitive to mixed sources) characterized by *ϵ*_Nd_(*t*) value = +4 to 0, *T_DM_*_2_ = 0.8–1.0 Ga, *ϵ*_Hf_(*t*) = +10 to +6 and *T_DM_*_2_ = 0.7–0.9 Ga;(IV) slightly evolved (i.e. mixed-source) provinces, with *ϵ*_Nd_(*t*) = –4 to 0, *T_DM_*_2_ = 1.0–1.2 Ga, *ϵ*_Hf_(*t*) = +2 to +6 and *T_DM_*_2_ = 0.9–1.1 Ga; (V) evolved (i.e. mixed-source) provinces with *ϵ*_Nd_(*t*) = –10 to –4, *T_DM_*_2_ = 1.2–1.6 Ga, *ϵ*_Hf_(*t*) = –4 to +2 and *T_DM_*_2_ = 1.1–1.5 Ga; (VI) highly evolved provinces with *ϵ*_Nd_(*t*) < –10, *T_DM_*_2_ = 1.6–2.8 Ga, *ϵ*_Hf_(*t*) < –4 and *T_DM_*_2_ > 1.5 Ga.

### Crustal architecture and its evolution

The primitive and evolved isotopic provinces reflect the distribution of juvenile and reworked ancient crust. Province I is characterized by juvenile accretionary complex (i.e. mélange), including intra-oceanic arcs (most contain boninites), accreted oceanic islands and oceanic basalts. This highly juvenile crust has been recognized in many areas (e.g. Western and Eastern Junggar, Inner Mongolia in China, southern Mongolia, the Lake zone of northwestern Mongolia and the southern Great Xing’an Range) [46,47,60,68–70].

Provinces II and III include juvenile and slightly juvenile crustal regions such as north Tianshan and southern Mongolia. Province IV represents intact or reworked microcontinents or old terranes, such as the central Altai. Province V is mainly old microcontinents or blocks, such as the Precambrian Erguna, South Tianshan, Central Mongolian and South Mongolian microcontinents. Province VI corresponds to cratons and microcontinents (e.g. the North China Craton and Tuva microcontinents [48]).

The isotopic mapping of igneous rocks is an approach that allows basement terrane mapping [38,71]. In general, intermediate–felsic igneous rocks are derived from the middle-lower crust and mafic igneous rocks are derived from the mantle. Therefore, isotopic mapping of intermediate–felsic and mafic igneous rocks reflects the composition of the middle-lower crust and mantle, respectively. As such, we examined the crustal compositional architecture and its evolution using these two types of rocks and their different ages (Fig. 4).

The juvenile crustal provinces correspond to depleted mantle regions (Fig. 4). This suggests that the depleted mantle provided the material for crustal growth. The evolution from the syn-accretionary (600–320 Ma) to post-accretionary (320–250 and 250–150 Ma) periods indicates that the juvenile crust formed mainly in the accretionary periods (Fig. 4a). This indicates that horizontal crustal growth during accretion was significant and that it did not result in the destruction of the overall crustal architecture. Figure 4b shows a similar pattern to Fig. 3a because the post-accretionary igneous rocks have similar primitive isotopic signatures as the syn-accretionary igneous rocks. The primitive isotopic signatures of the post-accretionary and/or intraplate igneous rocks were either inherited from the earlier syn-accretionary juvenile crustal sources (i.e. there was no new crustal growth) or derived from new mantle-sourced materials (i.e. there was new vertical crustal growth). The latter possibility has been confirmed by numerous previous studies [34,72]. For example, in the eastern Tianshan, the post-accretionary or intraplate (220–200 Ma) granitoids exhibit more primitive signatures (*ϵ*_Nd_(*t*) = –3 to +0.8) than the syn-accretionary (450–430 Ma) granitoids (*ϵ*_Nd_(*t*) = –5.8 to –15.8) in the same region [73]. This indicates that new juvenile crustal material was added to the deep crust (i.e. vertical crustal growth) by underplating of mantle-derived magma in a post-accretionary and intraplate setting [73].

### Geophysical evidence

**Figure 5.**
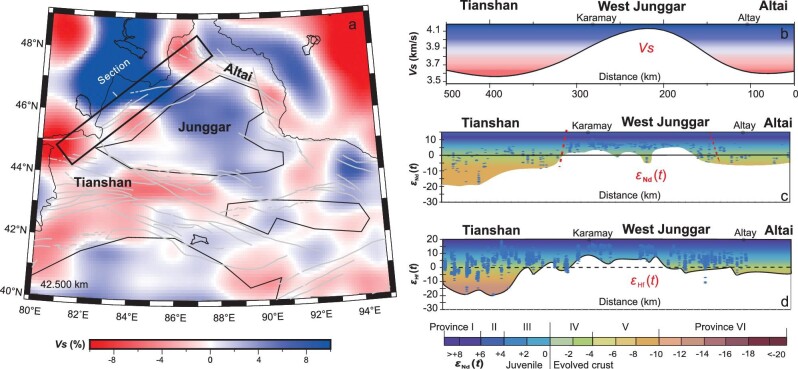
(a–d) Sections of *ϵ*_Nd_(*t*) (c) and zircon *ϵ*_Hf_(*t*) (d) values of felsic–intermediate and mafic igneous rocks, and ages of xenocrystic and inherited zircons across the Altai-West Junggar-Tianshan orogens, and a comparison with the *Vs* velocity profile (b). The *Vs* velocity profile (a) is from the unpublished data of Bao *et al*. The location of the sections is shown in Fig. 2a.

The results of the isotopic mapping are consistent with geophysical data. For example, shear wave (*Vs*) seismic tomography at the depths of 30–40 km, based on >200 stations, identified a high-velocity body in the western Junggar orogen and low-velocity bodies in the Altai and Tianshan mountains (Fig. 5). These coincide with Provinces I and II in the western Junggar orogen and evolved Provinces V and IV in the Tianshan and Altai mountains (Figs 2–4). Furthermore, magnetotelluric imaging identified deep-seated Paleozoic paleo-oceanic crust in the western Junggar orogen [74,75]. These features reveal that juvenile oceanic crust-like (i.e. mafic) materials occur in the western Junggar orogen and reworked crust occurs in the Altai and Tianshan mountains.

### Nature of the basement of the Junggar Basin: ancient or juvenile?

The Junggar Basin is the second largest basin of the Altaids. The nature of the basement of the basin is controversial, although the basement of the Western and Eastern Junggar orogens is considered to be juvenile (i.e. not Archean–Proterozoic basement [34,67,76,77]). It is generally thought that the basin has a Precambrian (i.e. Archean–Proterozoic) basement [78]. However, others have suggested that the basement could be young and possibly comprises Paleozoic orogenic systems [77,79]. Resolving this controversy requires an evaluation of the area of juvenile crust and the extent of continental growth in the Altaids.

The isotope mapping and geophysical data demonstrate that the basement of the Junggar Basin is mainly juvenile crust, which is similar to that of the peripheral Paleozoic orogen. First, igneous rocks from numerous drill holes in the basin have primitive isotopic characteristics (*ϵ*_Nd_(*t*) = +2 to +6; median zircon *ϵ*_Hf_(*t*) = +4 to +9; Figs 2 and 3), similar to those of igneous rocks around the basin. Second, *Vs* values in the basin at the depths of 30–40 km have revealed a high-velocity body, which is the same or similar to the *Vs* values at the same depth in the peripheral (i.e. Junggar) Paleozoic orogens. In addition, Zhu *et al.* [80] used magnetic data to identify numerous strong positive anomalies that trend NNE–SSW in the northwest, NW–SE in the southwest and NE–SW in the center of the Junggar Basin. They interpreted these to be paleo-suture zones consisting of faults, linear tectonics belts or igneous bodies [80]. These features, together with gravity and crustal-thickness data, strongly suggest that the basement has similar geophysical properties to oceanic and mafic lower

crust. All these geophysical studies are consistent with the presence of subducted oceanic crust in the West Junggar orogen [74,75]. Third, our Nd and Hf isotopic mapping, together with xenocrystal zircon mapping of igneous rocks, in the eastern and western Junggar orogens shows no evidence of pervasive ancient continental crustal basement [67,76].

Based on the above discussion, the basement of the Junggar Basin is likely juvenile accretive complexes (e.g. ophiolite mélanges and oceanic crust), which are dense and highly magnetic, similar to the surrounding orogenic belts and their basement. Even if the basin once had ancient Precambrian basement rocks, this basement has been destroyed and only remains as numerous small fragments.

### The growth and preservation of the voluminous continent

Based on our isotopic maps, we obtained the relative areal proportions of primitive and evolved isotopic compositions and used these to determine the juvenile and reworked crustal provinces, respectively. This allowed us to quantitatively estimate the volume and extent of continental growth.

Provinces I–III reflect juvenile crust (*ϵ*_Nd_(*t*) > 0; *T_DM_*_2_ < 1.0 Ga). Its total area is 4 107 350 km^2^, which accounts for 58% of the surface area of the Altaids (all isotopic provinces; Figs 2 and 3). Assuming that the deep-seated juvenile crust represents the entire crust (i.e. there is little significant over-thrusting) and based on a crustal thickness of 40–50 km in the Altaids [81], we estimated the juvenile crust has a volume of 184 830 750 km^3^. If 1000–150 Ma is assumed as the duration for the formation of the juvenile crust, the crustal growth rate is estimated to be ∼21.74 M km^3^/100 Ma (0.22 km^3^ per year); and if 600–150 Ma of the timing of the major orogeny is considered, the rate is estimated to be 41.07 M km^3^/100Ma (0.41 km^3^ per year). The rate is higher than those estimated in other regions and orogens such as 0.15 km^3^ per year of the central Altaids (Mongolia; [37]), 0.016 km^3^ per year of the Chinese Tianshan (northern Xinjiang; [41]) and 0.234 km^3^ per year of the part of the North American Cordillera (five of the major terranes plus the Coast Mountains batholith [82]). All these appear to be <1.0 km^3^ per year—the rate commonly assumed for the worldwide Mesozoic–Cenozoic arc addition rate [83]. All these parameters reflect overall crustal growth.

Moreover, from Fig. 4a, we obtained 3 156 900 km^2^ and 142 060 500 km^3^ (with the crustal thickness of 40–50 km) of the juvenile crust formed during 600–320 Ma, which accounts for 45% of the surface area of the Altaids (all isotopic provinces). The crustal growth rate is then estimated to be ∼50.74 M km^3^/100 Ma (0.51 km^3^ per year). These largely reflect the syn-accretionary (horizontal) crustal growth. Subtracting these from the overall crustal growth, we can get the parameters of the left post-accretionary (320–150 Ma) vertical crustal-growth parameters: 950 450 km^2^ and 42 770 250 km^3^ (crustal thickness of 40–50 km) of the juvenile, accounting for 14% of the surface area of the Altaids (all isotopic provinces; Fig. 4b), which are the largest estimations of the post-accretionary juvenile crust since accretion probably occurred in some regions (e.g. the southeastern Altaids) during this period. The crustal growth rate is then estimated to be ∼61.10 M km^3^/100 Ma (0.61 km^3^ per year). These results demonstrate that the syn-accretionary (horizontal) crustal growth dominated in the overall crustal growth in the Altaids.

**Figure 6.**
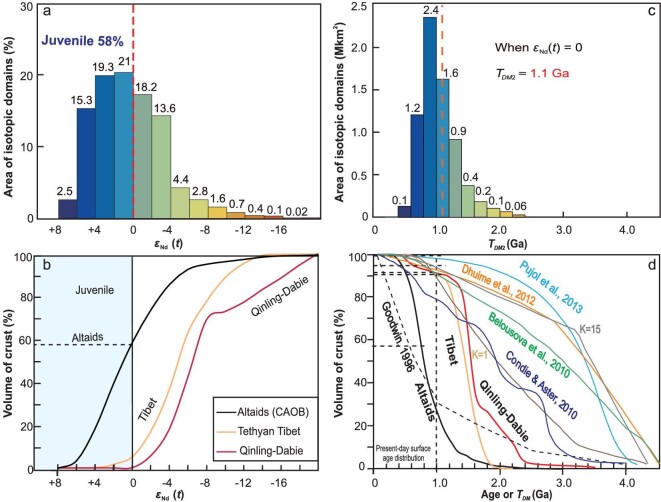
Plots of (a) *ϵ*_Nd_(*t*) value vs. the area % of the isotopic provinces, showing the total area % of the primitive isotopic provinces or juvenile crust (*ϵ*_Nd_(*t*) > 0; blue area) and evolved isotopic provinces or reworked crust (*ϵ*_Nd_(*t*) <0; warmer colors) for the Altaids; and (b) the corresponding crustal growth curves, showing the percentage areas of crust produced per two *ϵ*_Nd_(*t*) value intervals. (c) Histograms of *T_DM_*_2_ values, which reflect increasing amounts of primitive isotopic provinces or juvenile crust represented (blue areas; *ϵ*_Nd_(*t*) > 0) and ancient crust (warmer colors). (d) The corresponding crustal growth curves, showing the percentage of crust produced every 200 Myr (based on *T_DM_*_2_ values). The vertical dashed lines (b) represent *T_DM_*_2_ values corresponding to *ϵ*_Nd_(*t*) = 0; and the dotted horizontal lines denote the percentages of the crust when *ϵ*_Nd_(*t*) = 0 (e.g. ∼58% of the juvenile crust had been generated at ∼1000 Ma). (d) Other commonly used crustal growth curves are based on a summary by Hawkesworth *et al.* [2]. The data are listed in Supplementary Tables S1 and S2.

Using the same method, we also estimated the overall proportions of juvenile crust in other typical accretionary orogens: the North American Cordillera contains ∼54% juvenile crust; the Newfoundland Appalachians contains ∼40% juvenile crust; the Lachlan fold belt contains ∼31% juvenile crust. Collisional orogens contain much less juvenile crust, such as the Tethyan Tibet (3%), Caledonides Variscides (<2%) and Qinling-Dabie (<2%). Compared with these eight orogens, the Altaids shows the highest percentage (∼58%) and largest volume of juvenile crust. Moreover, the Altaids has the highest percentage of highly juvenile provinces with *ϵ*_Nd_(*t*) value > +4 that formed during the Phanerozoic time. Therefore, the Altaids is the most important site of Phanerozoic crustal growth and the largest preserved Phanerozoic juvenile crustal region. Kusky and Şengör [44] compared the map patterns, lithological contents, and structural and metamorphic evolution of the Altaids with those of the Archean Superior Province of the North American Craton to test for similarities or differences in the formation of continents through time. In addition, the comparison of crustal patterns and volumes of the two tectonic units is also significant. Isotopic mapping is a powerful tool to accomplish this. We use the areas of the isotopic provinces and domains to make a new crustal-growth curve (Fig. 6) and the curve directly and quantitatively denotes the proportions of juvenile and ancient crust and crustal growth. This method is different from other commonly used methods and crustal-growth curves are constructed from cumulative frequency plots of global isotopic data sets [2,4,23,83–85] or by analysis of igneous rock volumes and isotopic data [41,86].

The crustal curve of the Altaids is distinct from that of other orogens and the global curve, indicating a large volume and rapid crustal growth occurred during the Neoproterozoic–Phanerozoic (1000–100 Ma), particularly from 600 to 150 Ma. The growth rate is higher than any other Phanerozoic orogens (see above). As such, the accretionary orogen of the Altaids resulted in voluminous crustal growth from 1000 to 100 Ma, particularly from 600 to 150 Ma. This is consistent with the fact that slab rollback accretion and/or subduction was dominant in the Paleo-Asian Ocean [25,30,43] and Mongol-Okhotsk Ocean domains (e.g. [61]). This also confirms that slab rollback or subduction retreat and accretion are major mechanisms for continental crustal growth [25,87].

More significantly, the preservation of juvenile crust is direct evidence for the voluminous crustal growth [88–90]. Numerous oroclines with different sizes have been identified in the Altaids [25,26,43,91]. These oroclines had a key role in the preservation of the juvenile crust, such as the Mongolian orocline [61]. For example, the oroclines contain many trapped structures that preserve a large proportion of the oceanic crustal systems and other types of juvenile continental crust, such as the Junggar region of NW China [69,77,92] and the Lake zone of Mongolia [70] of the western Altaids.

## CONTROLS ON MINERALIZATION

The Altaids hosts many world-class, giant mineralization belts, including a porphyry Cu–(Au)–(Mo) ore system and other polymetallic magmatic–hydrothermal ore deposits, such as Li–Be, Nb–Ta, W–Sn and Pb–Zn deposits [32,36,49,52,53]. The ore-deposit belts were formed during several periods, including the early (490–440 Ma) and late (330–300 Ma) Paleozoic, and Mesozoic (220–190 and 160–120 Ma), in the syn-accretionary, post-accretionary and intraplate settings related to the Paleo-Asian Ocean and Mongol-Okhotsk Ocean tectonic domains [30,43,52]. As such, the mineralization domains in the Altaids can be divided into two parts influenced by the two dynamics. Their extent and boundaries during different periods have been recognized (Fig. 1; [61]). In addition, the far-field effects of the Paleo-Pacific (or Izanagi) Plate subduction were also superimposed on the eastern Altaids during the Mesozoic.

**Figure 7.**
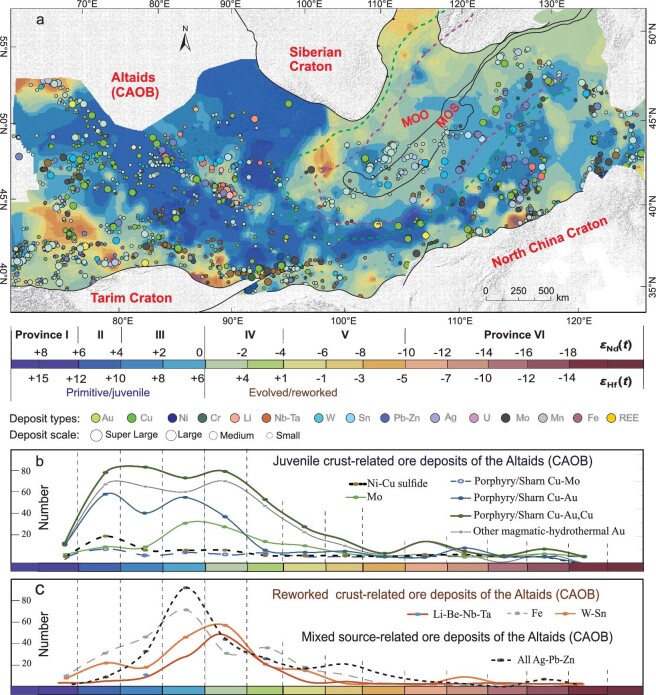
(a) Isotopic provinces and their definition of the spatial distribution of diversified ore deposits of the Altaids; (b) and (c) isotopic provinces vs. numbers of major ore deposits within each isotopic province. For abbreviations and other legends, see Fig. 1.

Our isotope mapping indicates that most of these ore deposits were controlled by the lithospheric architecture and specific crustal types, regardless of where they occur. Figure 7 quantitatively demonstrates the controls of highly juvenile and slightly juvenile-mixed lithospheric architecture on deposit systems, by showing the density of each type of ore deposit in each isotopic province. Most of the deposits are distributed in primitive–slightly evolved isotopic provinces (Provinces I–IV) and at least three peaks in number are prominent (Fig. 7).

### Ore-deposit systems associated with juvenile crustal and depleted mantle

A suite of porphyry-type Cu–(Au)–(Mo) deposits, including world-class deposits such as those at Kounrad, Aktogai, Kal’makyr, Oru lolhai and Chalukou, occur in the southern Altaids (Fig. 7a and b; [50–52]). Based on their ages, locations and dominant ore minerals, these deposits can be further grouped into the western, central and eastern mineralization belts or provinces: (1) the Kazakhstan Cu–Au–Mo belt in the west; (2) the Mongolia Cu–Au in the central area; and (3) the northeast China Mo–Cu in the east, respectively, displaying more Cu deposits in the west and more Mo deposits in the east [52]. Our isotopic mapping demonstrates that the regions of the western and central belts are dominated by large, highly primitive isotopic provinces (Provinces I and II) and the eastern belt is dominated by primitive isotopic provinces (Provinces II, III and some IV). Most porphyry-type Cu–(Au)–(Mo) deposits occur in the Paleo-Asian Ocean domain, which contains more juvenile crust province (Fig. 3). This deep crustal architecture controls the nature of the three mineralization belts.

Moreover, the porphyry Cu and Au deposits are clustered almost exclusively in the primitive isotopic provinces (Province II; i.e. juvenile terranes or crust; Fig. 7a and b). This suggests that the porphyry Cu–Au deposits were mostly related to the juvenile crust, such as the Early and Late Paleozoic (Late Carboniferous) porphyry Cu–Au–Mo deposits in Balkhash in the western Altaids and the Early Paleozoic Cu mineralization in the Duobaoshan (∼480 Ma) and Bainaimiao (∼440 Ma) in the eastern Altaids [36,50–53]. This is particularly the case for the largest porphyry Cu system (i.e. the Oyu Tolgoi Cu deposit in southern Mongolia), which was genetically associated with the ∼375-Ma intra-oceanic arcs within the Paleo-Asian Ocean (PAO; [51,53]). Some porphyry Cu and Au deposits around some regions of the Mongol-Okhotsk suture were generated in a continental arc setting (e.g. the Erguna terrane) and they are located in slightly evolved isotopic provinces (Provinces IV; Fig. 7b).

Whether the ore-forming magmas of (porphyry Cu–) Mo deposits in the Altaids have a genetic link with specific deep crustal component(s) is still unclear. Some researchers proposed that relatively ancient crust sources facilitated the formation of the porphyry Mo ore systems (e.g. [36]); others argued for the importance of magma volume (e.g. [93]). Figure 7 shows that the (porphyry Cu–) Mo deposits show more evolved isotopic provinces (in Province IV) and reworked crust than the porphyry Cu–Au deposits (mainly in Province II; Fig. 7a and b), although the Mo deposits have relatively primitive isotopic signatures compared with the Mo deposits in collisional orogenic belts [10]. This indicates that the (porphyry Cu–) Mo deposits in the Altaids have closer relationships to the more reworked ancient crust.

Our Nd–Hf isotopic mapping in the Qinling orogen, central China, also demonstrated a similar correlation [94]. The northern part of the Qinling orogen has a very old basement that is propitious to the development of large and numerous Mo deposits [94]. So, these relationships between the reworked crust and porphyry Mo deposits are the same or similar to previous reports that porphyry Cu–(Mo) deposits commonly occur in more mature continental arc environments compared with gold-rich porphyry Cu deposits in more primitive intra-oceanic arc settings (e.g. SW Pacific; Central and Northern Chile; [95]).

These observations suggest that the deep lithospheric architecture had a key control on these ore systems, despite the role of other factors (e.g. water content, intra-crustal magmatic differentiation, oxidation state and magmatic flux). These support the conclusion that (porphyry) Cu–Au deposits are closer to magmatic arcs (particularly to intra-oceanic arcs) and (porphyry) Cu–Mo to reworked continental margins. Our observations are consistent with those of previous studies of the Altaids [36,52] and other orogens, such as the central Andes [96] and Tethyan Tibet [97].

Most porphyry Cu and Cu–Au deposits in the Altaids were formed in subduction-related settings and close to the arc systems. Four representative genetic models for porphyry magmas associated with porphyry Cu–Mo–Au mineralization have been used to interpret the origin of these deposits [52], i.e. MASH model, ‘slab-melting’ model, ‘pre-enriched mafic lower crust and subduction of relict mid-oceanic ridges’ model and ‘melting of juvenile lower crust’ model. All the models mentioned above invoke pre- or syn-mineralization injections of juvenile, water-rich materials to the lower crustal levels and explain the dominant occurrence of porphyry Cu–Mo and Cu–Au deposits in juvenile domains.

In addition, mantle-derived deposits such as ophiolite-related Cr and mafic intrusion-related Ni–Cu sulfide deposits are concentrated in the juvenile crustal region (Provinces I and II; Fig. 7a and b), which is underlain by extremely depleted mantle provinces. The Ni–Cu and Cr deposits in the Altai and Junggar orogens are typical for these deposits.

### Ore-deposit systems associated with reworked crust

**Figure 8.**
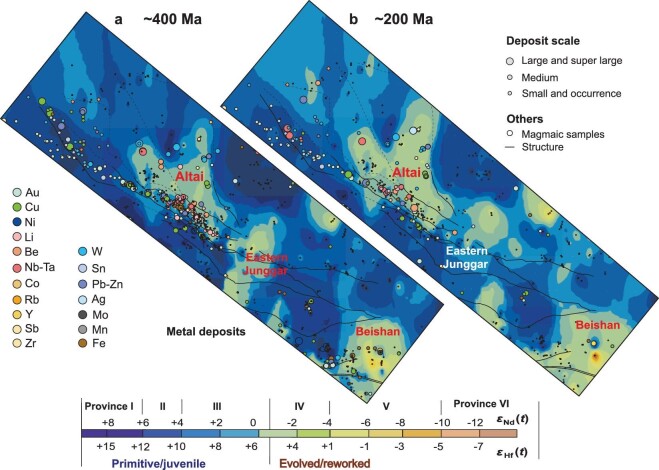
Contour isotopic maps made by a combination of *ϵ*_Nd_(*t*) values and zircon median *ϵ*_Hf_(*t*) values of felsic and intermediate igneous rocks along the Great Altai–Junggar–Beishan section, the western Altaids, at time slices of (a) ∼ 400 and (b) ∼ 200 Ma, and their control on the igneous-rock-related ore deposits. See the method and explanation in the text. The data set is presented in Supplementary Tables S1 and S3.

Many large-scale critical deposits with Li–Be, Nb–Ta and W–Sn deposits also occur in the Altaids. These deposits are mainly localized in evolved isotopic provinces (i.e. revoked crust or terranes; Provinces III, IV and V; *ϵ*_Nd_(*t*) = –4 to +2, peaks at *ϵ*_Nd_(*t*) = –1; Fig. 7a and c). For example, a world-class rare metal mineral (Li–Be, Nb–Ta) province occurs in the Great Altai orogen (Mountains) across China, Mongolia, Russia and the border areas of Kazakhstan. This includes the Keketokay No. 3 pegmatite deposit in Chinese Altai, which is the largest pegmatite rare metal deposit worldwide. All these critical metal deposits in the Great Altai orogen, regarding less in the Paleozoic (400–360 Ma) syn-accretionary, post-accretionary late Paleozoic and the early Mesozoic (252–180 Ma) intraplate settings, are almost distributed in the central Altai tectonic unit, i.e. reworked ancient continental fragments, characterized by relative evolved isotopic province with *ϵ*_Nd_(*t*) values of –2 to –4 (Figs 7a and c, and 8; Province IV). The major reason is that all these rare earth metal deposits are related to the S-type granites and pegmatites that are mainly derived from sedimentary crustal materials characterized by evolved isotopic signatures. Accordingly, the more evolved ancient crustal region is more beneficial for the generation of these deposits. We predict that the Qinghe-Halong region in the southeastern Altaids could be a highly prospective region for such deposits, based on our xenocrystic zircon mapping that identified abundant ancient crustal materials amongst the basement rocks [67,98]. It should be mentioned that all these rare metal mineral deposits in the Altai show a more juvenile crustal background than the world-class, ancient crust-derived metal mineralization province (*ϵ*_Nd_(*t*) = –10 to –22) in South China. This suggests that rare metal mineral deposits are closely related to the evolved and reworked crust, no matter the age (young or ancient) of the crust.

In terms of magmatic–hydrothermal Pb–Zn–Ag ore deposits, their high spatial density in both the slight juvenile and slight ancient crustal provinces (Provinces III and IV, mainly *ϵ*_Nd_(*t*) = –3 to +3 with a peak at +1; Fig. 8) indicate that two factors, i.e. the ancient crust and multiple phases of reworking triggered by episodic enhancement of mantle activity, played a role in their formation. These reflect that many giant Mesozoic epithermal Pb–Zn–(Ag) deposits are distributed in the southern Great Xing’an Range of the eastern Altaids where more reworked crust occurs and some Late Paleozoic MVT-type Pb–Zn deposits are located on margins of cratons in the western Altaids. Previous studies have revealed that base metals (i.e. Pb, Zn and Ag) were readily exsolved during crystallization of the final phase of a composite granitic magma and the fluid transported metals to the distal parts of the ore-forming system (e.g. [99]). In such a condition, old crustal materials contribute metals to the granitic magmas. This is a prerequisite for regional Pb–Zn–(Ag) mineralization.

Several studies of both orogens and cratons also revealed that lithospheric architecture exerts crucial control on the formation of magmatic–hydrothermal ore systems [10–12,94,100,101]. Isotopic mapping reveals how the lithospheric architecture and crustal type (juvenile, ancient and reworked) control the type and distribution of ore deposits. Here, according to their deep material background or architecture, we propose that metallic ore mineralization or deposits can be grouped into at least three types: juvenile, mixed and ancient crustal architecture-related deposits, represented by the Li–Be, Ni–Ta, W–Sn and Pb–Zn–Ag, as well as (porphyry) Cu–Au deposits as three end members, respectively.

Orogens generally evolve from subduction/accretionary to collisional, and to intra-cratonic stages [66,87]. Recent studies revealed that collisional orogens can also produce world-class mineralization belts in addition to subductional/accretionary orogens [97]; for example, in the Tethyan belt, remelting of sulfide-bearing, lower crustal cumulates of arc magmas associated with subduction-related porphyry Cu deposits produced giant collision-related porphyry Cu deposits [97]. The Altaids grew during long-term slab rollback in a retreating accretionary orogen, which formed giant extensional accretionary systems [25,26,30]. For example, the (south)westward rollback subduction/accretion in the Paleo-Asian Ocean formed the giant extensional accretionary systems (such as the Kazakhstan orocline) of the western Altaids, and eastward rollback subduction/accretion within the Mongol-Okhotsk Ocean formed the Tuva-Mongol accretionary systems (e.g. the Tuva-Mongol-Okhotsk orocline) in the eastern Altaids [27,43,61]. These extensional accretionary events produced a large-scale massive juvenile crust (or arc systems) and, significantly, this massive juvenile crust has been preserved through the development of oroclines [43,61]. Therefore, the Altaids can be viewed as the largest storage area and most typical ‘fossils’ of the juvenile crust in orogens worldwide. Our results are applicable to other types of orogens, particularly to the final continental collision and its control on mineralization.

Significantly, the preservation of large amounts of juvenile crust in accretionary systems may indicate that more giant ore deposits related to early subduction and accretion are present, such as in the Altaids.

## CONCLUDING REMARKS

Isotopic mapping, based on 5507 whole-rock Nd and 39 514 (2443 samples) zircon Hf isotope data of felsic–intermediate–mafic igneous rocks as well as associated 1830 ore-deposit data for the Altaids has revealed the 3D lithospheric architecture characterized by the middle-lower crust and mantle. The juvenile crust (defined by *ϵ*_Nd_(*t*) > 0) comprises an area of ∼4 107 350 km^2^ and a volume of ∼184 830 750 km^3^ (assuming a crustal thickness of 40–50 km), accounting for ∼58% of the isotope-mapped area (∼7 010 375 km^2^) of almost all the outcrops of the Altaids (∼8 745 000 km^2^). The crustal growth rate is at least ∼0.22 km^3^ per year. Compared with other global examples of Phanerozoic orogens, the Altaids hosts the largest volume, highest proportions and most rapidly produced juvenile crust, and it can be viewed as a ‘fossiled’ orogen with the largest preservation of juvenile crust in the world.

Isotopic mapping of the igneous rocks of 600–250 and 250–200 Ma revealed the 4D evolution of the crustal architecture. This indicates that the crustal architecture was mainly constructed during the syn-accretionary orogeny (600–250 Ma), which is indicative of a horizontal growth mechanism. Late Paleozoic–Mesozoic post-accretionary or intraplate igneous rocks (i.e. newly derived-mantle materials) record vertical crustal growth, which occurred in the eastern Altaids.

The crustal architecture of the Altaids reflects both the production and the preservation of massive juvenile crust, formed in the Paleo-Asian Ocean and the Mongol-Okhotsk Ocean tectonic domains. The juvenile crustal, slightly juvenile-reworked crustal and the slightly reworked crustal provinces controlled the Cu–Au, the Pb–Zn–Ag and the Li–Be, Nb–Ta, W–Sn ore deposits. A quantitative correlation between the isotopic provinces and ore-deposit types is proposed. According to the crustal architecture and background of deep compositions, we propose that the ore deposits can be grouped into three types: juvenile-crust-related, mixed-source-related and reworked-crust-related.

## Supplementary Material

nwac283_Supplemental_File

## References

[1] Campbell IH , Taylor SR. No water, no granites-no oceans, no continents. Geophys Res Lett 1983; 10: 1061–4. 10.1029/GL010i011p01061

[2] Hawkesworth C , Cawood P, Dhuime B. Continental growth and the crustal record. Tectonophysics 2013; 609: 651–60. 10.1016/j.tecto.2013.08.013

[3] Hawkesworth C , Cawood P, Dhuime B. Rates of generation and growth of the continental crust. Geosc Front 2019; 10: 165–73. 10.1016/j.gsf.2018.02.004

[4] Spencer CJ , Roberts NMW, Santosh M. Growth, destruction, and preservation of Earth's continental crust. Earth-Sci Rev 2017; 172: 87–106. 10.1016/j.earscirev.2017.07.013

[5] Dhuime B , Hawkesworth C, Cawood P. When continents formed. Science 2011; 331: 154–5. 10.1126/science.1201245 21233372

[6] Champion DC , Huston DL. Radiogenic isotopes, ore deposits and metallogenic terranes: novel approaches based on regional isotopic maps and the mineral systems concept. Ore Geol Rev 2016; 76: 229–56. 10.1016/j.oregeorev.2015.09.025

[7] Hacker BR , Kelemen PB, Behn MD. Continental lower crust. Annu Rev Earth Planet Sci 2015; 43: 167–205. 10.1146/annurev-earth-050212-124117

[8] Richards JP . Tectono-magmatic precursors for porphyry Cu-(Mo-Au) deposit formation. Econ Geol 2003; 98: 1515–33. 10.2113/gsecongeo.98.8.1515

[9] Goldfarb R , Taylor RD, Collins GS et al. Phanerozoic continental growth and gold metallogeny of Asia. Gondwana Res 2014; 25: 48–102. 10.1016/j.gr.2013.03.002

[10] Hou Z , Duan L, Lu Y et al. Lithospheric architecture of the Lhasa Terrane and its control on ore deposits in the Himalayan-Tibetan orogen. Econ Geol 2015; 110: 1541–75. 10.2113/econgeo.110.6.1541

[11] Mole DR , Fiorentini ML, Cassidy KF et al. Crustal evolution, intra-cratonic architecture and the metallogeny of an Archaean craton. Geol Soc London Spec Publ 2015; 393: 23–80. 10.1144/SP393.8

[12] Mole DR , Kirkland CL, Fiorentini ML et al. Time-space evolution of an Archean craton: a Hf-isotope window into continent formation. Earth-Sci Rev 2019; 196: 102831. 10.1016/j.earscirev.2019.04.003

[13] Kusky TM , Wang L. Growth of continental crust in intra-oceanic and continental margin arc systems: analogs for Archean systems. Sci China Earth Sci 2022; 65: 1615–45. 10.1007/s11430-021-9964-1

[14] Rudnick RL . Making continental crust. Nature 1995; 378: 571–8. 10.1038/378571a0

[15] Hawkesworth C , Kemp A. Evolution of the continental crust. Nature 2006; 443: 811–7. 10.1038/nature05191 17051208

[16] Maruyama S . Pacific-type orogeny revisited: Miyashiro-type orogeny proposed. Isl Arc 1997; 6: 91–120. 10.1111/j.1440-1738.1997.tb00042.x

[17] Hall R . Southeast Asia: new views of the geology of the Malay Archipelago. Annu Rev Earth Planet Sci 2017; 45: 331–58. 10.1146/annurev-earth-063016-020633

[18] Windley BF , Kusky TM, Polat A. Onset of plate tectonics by the early Archean. Precambrian Res 2021; 352: 105980. 10.1016/j.precamres.2020.105980

[19] Korenaga J . Hadean geodynamics and the nature of early continental crust. Precambrian Res 2021; 359: 106178 10.1016/j.precamres.2021.106178

[20] Harrison TM . Hadean Earth. Cham: Springer Nature Switzerland, 2020.

[21] von Huene R , Scholl DW. Observations at convergent margins concerning sediment subduction, subduction erosion, and the growth of continental crust. Rev Geophys 1991; 29: 279–316. 10.1029/91RG00969

[22] Scholl DW , von Huene R. Implications of estimated magmatic additions and recycling losses at the subduction zones of accretionary (non-collisional) and collisional (suturing) orogens. Geol Soc London Spec Pub 2009; 318: 105–25. 10.1144/SP318.4

[23] Belousova EA , Kostitsyn YA, Griffin WL et al. The growth of the continental crust: constraints from zircon Hf-isotope data. Lithos 2010; 119: 457–66. 10.1016/j.lithos.2010.07.024

[24] Stern RJ , Scholl DW. Yin and yang of continental crust creation and destruction by plate tectonic processes. Int Geol Rev 2010; 52: 1–31. 10.1080/00206810903332322

[25] Şengör AMC , Natal’in BA, Burtman VS. Evolution of the Altaid tectonic collage and Palaeozoic crustal growth in Eurasia. Nature 1993; 364: 299–307. 10.1038/364299a0

[26] Şengör AMC , Boris N, Grsel S et al. The tectonics of the Altaids: crustal growth during the construction of the continental lithosphere of Central Asia between 750 and 130 Ma ago. Annu Rev Earth Planet Sci 2018; 46: 439–94. 10.1146/annurev-earth-060313-054826

[27] Şengör AMC , Suna G, Natal’in BA et al. The Altaids: a review of twenty-five years of knowledge accumulation. Earth-Sci Rev 2022; 228: 104013. 10.1016/j.earscirev.2022.104013

[28] Jahn BM , Wu FY, Chen B. Granitoids of the Central Asian Orogenic Belt and continental growth in the Phanerozoic. T Roy Soc Edin-Earth 2000; 91: 181–93. 10.1017/S0263593300007367

[29] Windley BF , Alexeiev D, Xiao WJ. Tectonic models for accretion of the Central Asian Orogenic Belt. J Geol Soc 2007; 164: 31–47. 10.1144/0016-76492006-022

[30] Xiao WJ , Windley BF, Sun S et al. A tale of amalgamation of three Permo-Triassic collage systems in Central Asia: oroclines, sutures, and terminal accretion. Annu Rev Earth Planet Sci 2015; 43: 477–507. 10.1146/annurev-earth-060614-105254

[31] Jahn BM . The Central Asian Orogenic Belt and growth of the continental crust in the Phanerozoic. Geol Soc London Spec Pub 2004; 226: 73–100. 10.1144/GSL.SP.2004.226.01.05

[32] Hong DW , Wang SG, Xie XL et al. Metallogenic province derived from mantle sources: Nd, Sr, S and Pb isotope evidence from the Central Asian Orogenic Belt. Gondwana Res 2003; 4: 711–28. 10.1016/S1342-937X(05)71019-4

[33] Hong DW , Wang SG, Xie XL et al. Continental crustal growth and the supercontinental cycle: evidence from the Central Asian Orogenic Belt. J Asian Earth Sci 2004; 23: 799–813. 10.1016/S1367-9120(03)00134-2

[34] Han BF , Wang SG, Jahn BM et al. Depleted mantle magma source for the Ulungur River A-type granites from North Xinjiang China: geochemistry and Nd-Sr isotopic evidence, and implication for Phanerozoic crust al growth. Chem Geol 1997; 138: 135–59. 10.1016/S0009-2541(97)00003-X

[35] Wu FY , Jahn B, Wilde S et al. Phanerozoic crustal growth: U-Pb and Sr-Nd isotopic evidence from the granites in northeastern China. Tectonophysics 2000; 328: 89–113. 10.1016/S0040-1951(00)00179-7

[36] Wu C , Chen HY, Lu YJ. Crustal structure control on porphyry copper systems in accretionary orogens: insights from Nd isotopic mapping in the Central Asian Orogenic Belt. Miner Depos 2022; 57: 631–41. 10.1007/s00126-021-01074-z

[37] Kovalenko VI , Yarmolyuk VV, Kovach VP et al. Isotope provinces, mechanisms of generation and sources of the continental crust in the Central Asian mobile belt: geological and isotopic evidence. J Asian Earth Sci 2004; 23: 605–27. 10.1016/S1367-9120(03)00130-5

[38] Wang T , Jahn BM, Kovach VP et al. Nd-Sr isotopic mapping of the Chinese Altai and implications for continental growth in the Central Asian Orogenic Belt. Lithos 2009; 110: 359–72. 10.1016/j.lithos.2009.02.001

[39] Sun M , Yuan C, Xiao W et al. Zircon U-Pb and Hf isotopic study of gneissic rocks from the Chinese Altai: progressive accretionary history in the early to middle Palaeozoic. Chem Geol 2008; 247: 352–83. 10.1016/j.chemgeo.2007.10.026

[40] Seltmann R , Konopelko D, Biske G et al. Hercynian post-collisional magmatism in the context of Paleozoic magmatic evolution of the Tien Shan orogenic belt. J Asian Earth Sci 2011; 42: 821–38. 10.1016/j.jseaes.2010.08.016

[41] Tang GJ , Chung SL, Hawkesworth CJ et al. Short episodes of crust generation during protracted accretionary processes: evidence from Central Asian Orogenic Belt, NW China. Earth Planet Sci Lett 2017; 464: 142–54. 10.1016/j.epsl.2017.02.022

[42] Patchett PJ , Samson SD. Ages and growth of the continental crust from radiogenic isotopes. Treatise Geochem 2003; 3: 321–48. 10.1016/B0-08-043751-6/03026-7

[43] Xiao WJ , Windley BF, Han CM. Late Paleozoic to early Triassic multiple roll-back and oroclinal bending of the Mongolia collage in Central Asia. Earth-Sci Rev 2018; 186: 94–128. 10.1016/j.earscirev.2017.09.020

[44] Kusky TM , Şengör AMC. Comparative orotomy of the Archean Superior and Phanerozoic Altaid orogenic systems. Natl Sci Rev 2022; 10: nwac235. 10.1093/nsr/nwac235 PMC9933457 36817838

[45] Dobretsov NL , Buslov MM. Problems of geodynamics, tectonics, and metallogeny of orogens. Russ Geol Geophys 2011; 52: 1505–15. 10.1016/j.rgg.2011.11.012

[46] Kröner A , Kovach V, Belousova E et al. Reassessment of continental growth during the accretionary history of the Central Asian Orogenic Belt. Gondwana Res 2014; 25: 103–25. 10.1016/j.gr.2012.12.023

[47] Kröner A , Kovach V, Alexeiev D et al. No excessive crustal growth in the Central Asian Orogenic Belt: further evidence from field relationships and isotopic data. Gondwana Res 2017; 50: 135–66. 10.1016/j.gr.2017.04.006

[48] Zhou JB , Wilde SA, Zhao GC et al. Nature and assembly of microcontinental blocks within the Paleo-Asian Ocean. Earth-Sci Rev 2018; 186: 76–93. 10.1016/j.earscirev.2017.01.012

[49] Heinhorst J , Lehmann B, Ermolov P et al. Paleozoic crustal growth and metallogeny of Central Asia: evidence from magmatic-hydrothermal ore systems of Central Kazakhstan. Tectonophysics 2000; 328: 69–87. 10.1016/S0040-1951(00)00178-5

[50] Seltmann R , Porter TM. The porphyry Cu-Au/Mo deposits of central Eurasia: 1. tectonic, geologic & metallogenic setting and significant deposits. In: Porter TM (ed.). Super Porphyry Copper & Gold Deposits: A Global Perspective, Vol. 2. Adelaide: PGC Publishing, 2005, 467–512.

[51] Seltmann R , Porter TM, Pirajno F. Geodynamics and metallogeny of the central Eurasian porphyry and related epithermal mineral systems: a review. J Asian Earth Sci 2014; 79: 810–41. 10.1016/j.jseaes.2013.03.030

[52] Gao J , Qin K, Zhou M et al. Large-scale porphyry-type mineralization in the Central Asian Metallogenic Domain: geodynamic background, magmatism, fluid activity and metallogenesis. J Asian Earth Sci 2018; 165: 7–36. 10.1016/j.jseaes.2017.10.002

[53] Yakubchuk AS , Shatov VV, Kirwin D et al. Gold and base metal metallogeny of the Central Asian orogenic supercollage. Soc Econ Geol 2005; 100: 1035–68.

[54] Xue CJ , Zhao XB, Mo XX. Problem on porphyry Cu-Au metallogenic environment in Central Asian: an overview (in Chinese with English abstract). Acta Petrol Sin 2016; 32: 1249–61.

[55] Şengör AMC , Natal’in B. Turkic-type orogeny and its role in the making of the continental crust. Annu Rev Earth Planet Sci 1996; 24: 263–337. 10.1146/annurev.earth.24.1.263

[56] Yakubchuk A . Evolution of the Central Asian Orogenic Supercollage since Late Neoproterozoic revised again. Gondwana Res 2017; 47: 372–98. 10.1016/j.gr.2016.12.010

[57] Mossakovsky AA . Central Asian fold belt: geodynamic evolution and formation history. Geotectonics 1994; 24: 445–74.

[58] Han Y , Zhao G. Final amalgamation of the Tianshan and Junggar orogenic collage in the southwestern Central Asian Orogenic Belt: constraints on the closure of the Paleo-Asian Ocean. Earth-Sci Rev 2018; 186: 129–52. 10.1016/j.earscirev.2017.09.012

[59] Tan Z , Xiao W, Mao Q et al. Final closure of the Paleo Asian Ocean basin in the early Triassic. Commun Earth Environ 2022; 3: 259. 10.1038/s43247-022-00578-4

[60] Safonova I . Juvenile versus recycled crust in the Central Asian Orogenic Belt: implications from ocean plate stratigraphy, blueschist belts and intra-oceanic arcs. Gondwana Res 2017; 47: 6–27. 10.1016/j.gr.2016.09.003

[61] Wang T , Tong Y, Xiao WJ et al. Rollback, scissor-like closure of the Mongol-Okhotsk Ocean and formation of an orocline: magmatic migration based on a large archive of age-data. Natl Sci Rev 2022; 9: nwab210. 10.1093/nsr/nwab210 35547957 PMC9084359

[62] Wang T , Tong Y, Zhang L et al. Phanerozoic granitoids in the central and eastern parts of Central Asia and their tectonic significance. J Asian Earth Sci 2017; 145: 368–92. 10.1016/j.jseaes.2017.06.029

[63] Li S , Wang T, Wilde SA et al. Evolution, source, and tectonic significance of Early Mesozoic granitoid magmatism in the Central Asian Orogenic Belt (central segment). Earth-Sci Rev 2013; 126: 206–34. 10.1016/j.earscirev.2013.06.001

[64] Huang H , Wang T, Tong Y et al. Rejuvenation of ancient micro-continents during accretionary orogenesis: insights from the Yili block and adjacent regions of the SW central Asian orogenic belt. Earth-Sci Rev 2020; 208: 103255. 10.1016/j.earscirev.2020.103255

[65] Vervoort JD , Patchett PJ, Blichert-Toft J et al. Relationships between Lu-Hf and Sm-Nd isotopic systems in the global sedimentary system. Earth Planet Sci Lett 1999; 168: 79–99. 10.1016/S0012-821X(99)00047-3

[66] Condie K . Preservation and recycling of crust during accretionary and collisional phases of Proterozoic orogens: a bumpy road from Nuna to Rodinia. Geosciences 2013; 3: 240–61. 10.3390/geosciences3020240

[67] Zhang J , Wang T, Tong Y et al. Tracking deep ancient crustal components by xenocrystic/inherited zircons of Palaeozoic felsic igneous rocks from the Altai-East Junggar terrane and adjacent regions, western Central Asian Orogenic Belt and its tectonic significance. Int Geol Rev 2017; 59: 2021–40. 10.1080/00206814.2017.1308841

[68] Yang QD , Wang T, Guo L et al. Nd isotopic variation of Paleozoic-Mesozoic granitoids from the Da Hinggan Mountains and adjacent areas, NE Asia: implications for the architecture and growth of continental crust. Lithos 2017; 272–273: 164–84. 10.1016/j.lithos.2016.11.015

[69] Zheng B , Han BF, Wang ZZ et al. An example of Phanerozoic continental crustal growth: the West Junggar Orogenic Belt, Northwest China. Lithos 2020; 376–377: 105745. 10.1016/j.lithos.2020.105745

[70] Khukhuudei U , Kusky T, Windley BF et al. Ophiolites and ocean plate stratigraphy (OPS) of the Central Mongolian Microcontinent: an archive of data for the tectonic evolution of the Paleo-Asian Ocean. Gondwana Res 2022; 105: 51–83. 10.1016/j.gr.2021.12.008

[71] Champion DC , Sheraton JW. Geochemistry and Nd isotope systematics of Archaean granites of the Eastern Goldfields, Yilgarn Craton, Australia: implications for crustal growth processes. Precambrian Res 1997; 83: 109–32. 10.1016/S0301-9268(97)00007-7

[72] Han BF , Ji JQ, Song B et al. Late Paleozoic vertical growth of continental crust around the Junggar Basin, Xinjiang, China (Part I): timing of post collisional plutonism (in Chinese with English abstract). Acta Petrol Sin 2006; 22: 1077–86.

[73] Wang T , Li WP, Li JB et al. Increase of juvenal mantle-derived composition from syn-orogenic to post-orogenic granites of the east part of the eastern Tianshan (China) and implications for continental vertical growth: Sr and Nd isotopic evidence (in Chinese with English abstract). Acta Petrol Sin 2008; 24: 762–72.

[74] Xu YX , Yang B, Zhang S et al. Magnetotelluric imaging of a fossil Paleozoic intraoceanic subduction zone in western Junggar, NW China. J Geophys Res Solid Earth 2016; 121: 4103–17. 10.1002/2015JB012394

[75] Xu YX , Yang B, Zhang AQ et al. Magnetotelluric imaging of a fossil oceanic plate in northwestern Xinjiang, China. Geology 2020; 48: 385–9. 10.1130/G47053.1

[76] Song P , Wang T, Tong Y et al. Contrasting deep crustal compositions between the Altai and East Junggar orogens, SW Central Asian Orogenic Belt: evidence from zircon Hf isotopic mapping. Lithos 2019; 328–329: 297–311. 10.1016/j.lithos.2018.12.039

[77] Yang X , Tian X, Windley BF et al. The role of multiple trapped oceanic basins in continental growth: seismic evidence from the southern Altaids. Geophys Res Lett 2022; 49: e2022GL098548. 10.1029/2022GL098548

[78] Wang J , Su YP, Zheng JP et al. Hidden Eoarchean crust in the southwestern Central Asian Orogenic Belt. Lithos 2020; 360–361: 105437. 10.1016/j.lithos.2020.105437

[79] Chen B , Jahn BM. Genesis of post-collisional granitoids and basement nature of the Junggar Terrane, NW China: Nd-Sr isotope and trace element evidence. J Asian Earth Sci 2004; 23: 691–703. 10.1016/S1367-9120(03)00118-4

[80] Zhu XS , Wang T, Huang H et al. An aeromagnetic study of fault structures underneath the region across the Chinese Altai orogen, Junggar Basin, Tianshan orogen, and Tarim Basin. J Asian Earth Sci 2022; 239: 105418. 10.1016/j.jseaes.2022.105418

[81] Bao XW , Song XD, Li JT. High-resolution lithospheric structure beneath Mainland China from ambient noise and earthquake surface-wave tomography. Earth Planet Sci Lett 2015; 417: 132–41. 10.1016/j.epsl.2015.02.024

[82] Samson SD , Patchett PJ. The Canadian Cordillera, as a modern analogue of Proterozoic crustal growth. Aust J Earth Sci 1991; 38: 595–611. 10.1080/08120099108727994

[83] Reymer A , Schubert G. Phanerozoic addition rates to the continental crust and crustal growth. Tectonics 1984; 3: 63–77. 10.1029/TC003i001p00063

[84] Dhuime B , Wuestefeld A, Hawkesworth CJ. Emergence of modern continental crust about 3 billion years ago. Nat Geosci 2015; 8: 552–5. 10.1038/ngeo2466

[85] Dhuime B , Hawkesworth C, Delavault H et al. Rates of generation and destruction of the continental crust: implications for continental growth. Phil Trans R Soc A 2018; 376: 20170403. 10.1098/rsta.2017.0403 30275156 PMC6189557

[86] Sun C , Xu W, Cawood PA et al. Crustal growth and reworking: a case study from the Erguna Massif, eastern Central Asian Orogenic Belt. Sci Rep 2019; 9: 17671. 10.1038/s41598-019-54230-x 31776438 PMC6881325

[87] Cawood PA , Kroner A, Collins WJ et al. Accretionary orogens through Earth history. Geol Soc London Spec Pub 2009; 318: 1–36. 10.1144/SP318.1

[88] Cawood PA , Hawkesworth CJ, Dhuime B. The continental record and the generation of continental crust. Geol Soc Am Bull 2013; 125: 14–32. 10.1130/B30722.1

[89] Condie KC , Bickford ME, Aster RC et al. Episodic zircon ages, Hf isotopic composition, and the preservation rate of continental crust. Geol Soc Am Bull 2011; 123: 951–7. 10.1130/B30344.1

[90] Mulder JA , Cawood PA. Evaluating preservation bias in the continental growth record against the monazite archive. Geology 2022; 50: 243–7. 10.1130/G49416.1

[91] Liu YJ , Li WM, Ma YF et al. An orocline in the eastern Central Asian Orogenic Belt. Earth-Sci Rev 2021; 221: 103808. 10.1016/j.earscirev.2021.103808

[92] Carroll AR , Yunhai L, Graham SA et al. Junggar basin, northwest China: trapped Late Paleozoic ocean. Tectonophysics 1990; 181: 1–14. 10.1016/0040-1951(90)90004-R

[93] Shu Q , Chiaradia M. Mesozoic Mo mineralization in northeastern China did not require regional-scale pre-enrichment. Econ Geol 2021; 116: 1227–37. 10.5382/econgeo.4823

[94] Wang X , Wang T, Ke C et al. Nd-Hf isotopic mapping of Late Mesozoic granitoids in the East Qinling orogen, central China: constraint on the basements of terranes and distribution of Mo mineralization. J Asian Earth Sci 2015; 103: 169–83. 10.1016/j.jseaes.2014.07.002

[95] Cooke DR , Hollings P, Walshe JL. Giant porphyry deposits: characteristics, distribution, and tectonic controls. Econ Geol 2005; 100: 801–18. 10.2113/gsecongeo.100.5.801

[96] Muñoz M , Charrier R, Fanning CM et al. Zircon trace element and O-Hf isotope analyses of mineralized intrusions from El Teniente ore deposit, Chilean Andes: constraints on the source and magmatic evolution of porphyry Cu-Mo related magmas. J Petrol 2012; 53: 1091–122. 10.1093/petrology/egs010

[97] Hou Z , Yang Z, Lu Y et al. A genetic linkage between subduction- and collision-related porphyry Cu deposits in continental collision zones. Geology 2015; 43: 247–50. 10.1130/G36362.1

[98] Shen P , Pan HD, Li CH et al. Temporal-spatial distribution, genesis and metallogenic regularity of the rare metal deposits in Altay of China, Kazakhstan, and Russia (in Chinese with English abstract). J Earth Sci Environ 2021; 43: 487–505.

[99] Zhai DG , Williams-Jones AE, Liu JJ et al. The genesis of the giant Shuangjianzishan Epithermal Ag-Pb-Zn deposit, Inner Mongolia, Northeastern China. Econ Geol 2020; 115: 101–28. 10.5382/econgeo.4695

[100] Champion DC , Huston DL. Radiogenic isotopes, ore deposits and metallogenic terranes: novel approaches based on regional isotopic maps and the mineral systems concept. Ore Geol Rev 2016; 76: 229–56. 10.1016/j.oregeorev.2015.09.025

[101] Deng J , Wang CM, Bagas Leon et al. Crustal architecture and metallogenesis in the south-eastern North China Craton. Earth-Sci Rev 2018; 182: 251–72. 10.1016/j.earscirev.2018.05.001

