# Supplementary material for: Voluminous continental growth of the Altaids and its control on metallogeny
Source: Natl Sci Rev. 2022 Dec 19;10(2):nwac283. doi: 10.1093/nsr/nwac283 (PMC9942667; doi:10.1093/nsr/nwac283)
Supplement: nwac283_Supplemental_File [file nwac283_supplemental_file.pdf]

Appendix: Supporting Supplementary Materials

Table S1a. Whole-rock Sm-Nd isotope data of the felsic-intermediate igneous rocks of the Altaids. In this paper, $(^{143}Nd/^{144}Nd)_{CHUR}$ = 0.512638, $(^{147}Sm/^{144}Nd)_{CHUR}$ = 0.1967 (Jacobsen & Wasserburg, 1984, II. *Earth Planet. Sci. Lett.*). Parameters are calculated as below (DePaolo & Wasserburg, 1976, *Geophysical Research Letters*): ε_{Nd} = $((^{143}Nd/^{144}Nd)_{Sample}(T)/(^{143}Nd/^{144}Nd)_{CHUR}(T)-1) \times 10000$, The one-stage model ages (T_{DM1}) = 1/λx ln(1+ $((^{143Nd}/^{144}Nd)_{Sample(T)}$ - 0.51315)/ $((^{147}Sm/^{144}Nd)_{Sample(T)}$ - 0.2137)); The two-stage model age (T_{DM2}) = (T_{DM1}-(T_{DM1}-t)(fcc-fs))/ (fcc-fdm); where $f_{Sm/Nd}=(^{147}Sm/^{144}Nd)s/(^{147}Sm/^{144}Nd)_{CHUR}-1$; fcc, fs, fdm = $f_{Sm/Nd}$ values of the average continental crust, the sample, and the depleted mantle, respectively. fcc = 0.4, fdm = 0.08592, t = the intrusive age of felsic-intermediate igneous rocks.

No.	Sample	GeoTectonic Unit	Tectonic unit/Locationh	Longitude	Latitude	Lithology	Pluton	Sample Age (Ma)	Revised Age (Ma)	Sm	Nd	$^{147}Sm/^{144}Nd$	$^{143}Nd/^{144}Nd$	2σ	$f_{Sm/Nd}$	Calcul.$\varepsilon_{Nd}(0)$	Orogi. $\varepsilon_{Nd}(t)$	Recal. $\varepsilon_{Nd}(t)$	Mapped $\varepsilon_{Nd}(t)$	Mapped T_{DM} (Ga)	Recal. T_{DM1} (Ga)	Recal. T_{DM2} (Ga)	Origi. T_{DM1} (Ga)	Origi. T_{DM2} (Ga)	Ref. Author	Ref. year	Ref_Journal	Volume and Number	Page
1	GC-205	CAOB	Western Mongolia	92.3812	47.0816	Quartz metadiorite		460	460	5.96	18.90	0.1906	0.512582		-0.03	-1.09	-0.70	-0.73	-0.73	1.24	3.71	1.24	3.04	1.20	Soejono I et al.	2017	Lithos	294-295	112-132
2	GB-475	CAOB	Western Mongolia	92.0635	47.1130	Quartz metadiorite		460	460	4.63	20.80	0.1346	0.512618		-0.32	-0.39	3.30	3.26	3.26	0.93	1.02	0.93	0.96	0.89	Soejono I et al.	2017	Lithos	294-295	112-132
3	GB-363A	CAOB	Western Mongolia	92.2553	47.0152	Metagranodiorite		460	460	3.82	21.90	0.1055	0.512544		-0.46	-1.83	3.50	3.52	3.52	0.91	0.85	0.91	0.82	0.87	Soejono I et al.	2017	Lithos	294-295	112-132
4	GB-331	CAOB	Western Mongolia	92.3148	47.1320	Quartz metadiorite		460	460	4.81	22.80	0.1275	0.512619		-0.35	-0.37	3.70	3.69	3.69	0.89	0.94	0.89	0.89	0.85	Soejono I et al.	2017	Lithos	294-295	112-132
5	306	CAOB	stern margin of the Siberian Cr	91.5833	60.3667	Granite		1380	600	5.00	35.00	0.0869	0.511344		-0.56	-25.24	-5.80	-17	-17	2.67	2.16	2.67	2.17	2.44	Likhanov II et al.	2019	Precambrian Research	328	128-145
6	S-n8	CAOB	Uzbekista	69.7667	40.7333	Monzonite dyke	Sarycheku	313	313	5.30	28.80	0.1038	0.512440		-0.47	-3.86	0.10	-0.15	-0.15	1.08	0.98	1.08		1.08	Cheng ZG et al.	2017	International Geology Review	60(1)	1-20
7	S-n6	CAOB	Uzbekista	69.7667	40.7333	Monzonite dyke	Sarycheku	313	313	5.00	31.10	0.1085	0.512450		-0.45	-3.67	0.00	-0.14	-0.14	1.08	1.01	1.08		1.09	Cheng ZG et al.	2017	International Geology Review	60(1)	1-20
8	S-n2	CAOB	Uzbekista	69.7667	40.7333	Monzonite dyke	Sarycheku	313	313	5.40	29.90	0.1736	0.512470		-0.12	-3.28	-2.30	-2.35	-2.35	1.26	2.57	1.26		1.27	Cheng ZG et al.	2017	International Geology Review	60(1)	1-20
9	S-n17	CAOB	Uzbekista	69.7833	40.7667	Granodiorite	Sarycheku	338	338	4.00	22.40	0.1106	0.512460		-0.44	-3.47	0.00	0.24	0.24	1.07	1.02	1.07		1.08	Cheng ZG et al.	2017	International Geology Review	60(1)	1-20
10	S-n14	CAOB	Uzbekista	69.7833	40.7667	Granodiorite	Sarycheku	338	338	3.00	16.70	0.1128	0.512470		-0.43	-3.28	0.20	0.34	0.34	1.06	1.03	1.06		1.07	Cheng ZG et al.	2017	International Geology Review	60(1)	1-20
11	S-n10	CAOB	Uzbekista	69.7667	40.7333	Monzonite dyke	Sarycheku	313	313	5.50	30.90	0.1063	0.512450		-0.46	-3.67	0.00	-0.06	-0.06	1.08	0.99	1.08		1.08	Cheng ZG et al.	2017	International Geology Review	60(1)	1-20
12	S-n1	CAOB	Uzbekista	69.7833	40.7667	Tonalite	Sarycheku	338	338	4.30	23.20	0.1109	0.512470		-0.44	-3.28	0.20	0.42	0.42	1.06	1.01	1.06		1.07	Cheng ZG et al.	2017	International Geology Review	60(1)	1-20
13	K-10	CAOB	Uzbekista	69.6500	40.8167	Granodiorite	Kalmakyr	315	315	3.20	15.30	0.1172	0.512450		-0.40	-3.67	-0.30	-0.47	-0.47	1.11	1.11	1.11		1.11	Cheng ZG et al.	2017	International Geology Review	60(1)	1-20
14	K-9	CAOB	Uzbekista	69.6500	40.8167	Granodiorite	Kalmakyr	315	315	2.90	13.00	0.1297	0.512470		-0.34	-3.28	-0.50	-0.58	-0.58	1.12	1.23	1.12		1.12	Cheng ZG et al.	2017	International Geology Review	60(1)	1-20
15	K-5	CAOB	Uzbekista	69.6333	40.8333	Quartz monzonite	Kalmakyr	326	326	3.30	18.70	0.1143	0.512460		-0.42	-3.47	-0.10	-0.04	-0.04	1.09	1.06	1.09		1.09	Cheng ZG et al.	2017	International Geology Review	60(1)	1-20
16	K-4	CAOB	Uzbekista	69.6333	40.8333	Quartz monzonite	Kalmakyr	326	326	2.30	10.70	0.1344	0.512470		-0.32	-3.28	-0.70	-0.69	-0.69	1.14	1.31	1.14		1.14	Cheng ZG et al.	2017	International Geology Review	60(1)	1-20
17	K-3-1	CAOB	Uzbekista	69.6500	40.8167	Granodiorite	Kalmakyr	315	315	2.50	13.10	0.1256	0.512460		-0.36	-3.47	-0.50	-0.61	-0.61	1.12	1.19	1.12		1.13	Cheng ZG et al.	2017	International Geology Review	60(1)	1-20
18	K-1	CAOB	Uzbekista	69.6333	40.8333	Quartz monzonite	Kalmakyr	326	326	1.70	6.80	0.1435	0.512470		-0.27	-3.28	-1.00	-1.06	-1.06	1.17	1.47	1.17		1.17	Cheng ZG et al.	2017	International Geology Review	60(1)	1-20
19	K3*	CAOB	Junggar	88.5471	45.6120	Basaltic andesite		360	360	4.50	18.30	0.1487	0.512860		-0.24	4.33	6.60	6.54	6.54	0.58	0.68	0.58	0.68		Li D et al.	2016	Chinese University of Geosciences (Beijing).		
20	K4*	CAOB	Junggar	88.5471	45.6120	Basaltic andesite		360	360	2.93	12.10	0.1464	0.512903		-0.26	5.17	7.60	7.48	7.48	0.50	0.56	0.50	0.56		Li D et al.	2016	Chinese University of Geosciences (Beijing).		
21	L8-3	CAOB	Junggar	87.8151	45.9904	Dacite		360	360	4.30	20.60	0.1262	0.512760		-0.36	2.38	5.30	5.62	5.62	0.65	0.68	0.65	0.68		Li D et al.	2016	Chinese University of Geosciences (Beijing).		
22	L8-5	CAOB	Junggar	87.8151	45.9904	Dacite		360	360	4.71	22.30	0.1277	0.512744		-0.35	2.07	4.90	5.24	5.24	0.68	0.72	0.68	0.72		Li D et al.	2016	Chinese University of Geosciences (Beijing).		
23	K7*	CAOB	Junggar	88.0818	46.0649	Andesite		360	360	6.97	30.00	0.1405	0.512953		-0.29	6.14	8.40	8.73	8.73	0.40	0.41	0.40	0.41		Li D et al.	2016	Chinese University of Geosciences (Beijing).		
24	K8*	CAOB	Junggar	88.0818	46.0649	Andesite		360	360	7.23	30.70	0.1424	0.512961		-0.28	6.30	8.50	8.80	8.80	0.39	0.40	0.39	0.41		Li D et al.	2016	Chinese University of Geosciences (Beijing).		
25	JL14-8	CAOB	Junggar	85.0959	45.1347	Andesite		310	310	1.95	8.20	0.1433	0.512881		-0.27	4.74	6.90	6.85	6.85	0.51	0.58	0.51	0.58	0.48	Li D et al.	2016	Chinese University of Geosciences (Beijing).		
26	JL14-10	CAOB	Junggar	85.0959	45.1347	Andesite		310	310	1.70	6.70	0.1525	0.512919		-0.22	5.48	7.20	7.23	7.23	0.48	0.58	0.48	0.58	0.45	Li D et al.	2016	Chinese University of Geosciences (Beijing).		
27	JL14-12	CAOB	Junggar	85.0959	45.1347	Andesite		310	310	1.89	7.70	0.1494	0.512812		-0.24	3.39	5.30	5.27	5.27	0.64	0.80	0.64	0.80	0.59	Li D et al.	2016	Chinese University of Geosciences (Beijing).		
28	JL14-1	CAOB	Junggar	85.0959	45.1347	Dacite		310	310	7.15	30.90	0.1399	0.512860		-0.29	4.33	6.60	6.58	6.58	0.53	0.60	0.53	0.60	0.50	Li D et al.	2016	Chinese University of Geosciences (Beijing).		
29	JL14-3	CAOB	Junggar	85.0959	45.1347	Dacite		310	310	7.60	33.70	0.1363	0.512855		-0.31	4.23	6.60	6.62	6.62	0.53	0.58	0.53	0.58	0.50	Li D et al.	2016	Chinese University of Geosciences (Beijing).		
30	JL14-5	CAOB	Junggar	85.0959	45.1347	Dacite		310	310	4.46	18.10	0.1490	0.512831		-0.24	3.76	5.60	5.65	5.65	0.61	0.75	0.61	0.75	0.56	Li D et al.	2016	Chinese University of Geosciences (Beijing).		
31	JL14-6	CAOB	Junggar	85.0959	45.1347	Dacite		310	310	4.16	17.10	0.1471	0.512826		-0.25	3.67	5.60	5.63	5.63	0.61	0.74	0.61	0.74	0.56	Li D et al.	2016	Chinese University of Geosciences (Beijing).		
32	J213-15	CAOB	Junggar	85.1539	45.0380	Rhyolite		310	310	7.63	31.70	0.1455	0.512843		-0.26	4.00	6.00	6.02	6.02	0.58	0.69	0.58	0.69	0.54	Li D et al.	2016	Chinese University of Geosciences (Beijing).		
33	J213-18	CAOB	Junggar	85.1539	45.0380	Rhyolite		310	310	6.37	27.70	0.1390	0.512884		-0.29	4.80	7.10	7.08	7.08	0.49	0.54	0.49	0.54	0.46	Li D et al.	2016	Chinese University of Geosciences (Beijing).		
34	J213-21	CAOB	Junggar	85.1539	45.0380	Rhyolite		310	310	6.09	27.20	0.1354	0.512877		-0.31	4.66	7.10	7.09	7.09	0.49	0.53	0.49	0.53	0.46	Li D et al.	2016	Chinese University of Geosciences (Beijing).		
35	JL2-2	CAOB	Junggar	84.9984	45.2001	Rhyolite		310	310	9.90	42.00	0.1425	0.512823		-0.28	3.61	5.70	5.75	5.75	0.60	0.70	0.60	0.70	0.56	Li D et al.	2016	Chinese University of Geosciences (Beijing).		
36	JL2-4	CAOB	Junggar	84.9984	45.2001	Rhyolite		310	310	10.40	46.80	0.1344	0.512820		-0.32	3.55	6.00	6.01	6.01	0.58	0.63	0.58	0.64	0.54	Li D et al.	2016	Chinese University of Geosciences (Beijing).		
37	J11-8-1	CAOB	Junggar	88.0818	46.0649	Andesite		360	360	7.92	28.10	0.1704	0.512952		-0.13	6.13	7.20	7.33	7.33	0.51	0.70	0.51	0.70		Li D et al.	2016	Chinese University of Geosciences (Beijing).		
38	J11-8-2	CAOB	Junggar	88.0818	46.0649	Andesite		360	360	7.54	29.10	0.1567	0.512788		-0.20	2.93	4.50	4.76	4.76	0.72	0.97	0.72	0.97		Li D et al.	2016	Chinese University of Geosciences (Beijing).		
39	J11-5-2	CAOB	Junggar	86.4469	45.5937	Dacite		350	350	4.26	19.60	0.1314	0.512750		-0.33	2.18	4.80	5.10	5.10	0.69	0.74	0.69	0.76		Li D et al.	2016	Chinese University of Geosciences (Beijing).		
40	5041	CAOB	Dzabkhan microcontinent	92.2332	46.0785	Granite		240	240	3.96	25.60	0.0926	0.512610		-0.53	-0.55	2.60	2.64	2.64	0.80	0.68	0.80	0.68	0.80	Kozakov IK et al.	2007	Stratigraphy and Geological Correlation	15	121-140
41	BH003-2	CAOB	Altai	86.5506	48.2269	Tonalite	Habahe	372	372	2.66	8.88	0.1810	0.512842		-0.08	3.98	4.70	4.72	4.72	0.73	1.43	0.73			Yang Y et al.	2019	Journal of Asian Earth Sciences	175	230-246
42	BH003-3	CAOB	Altai	86.5506	48.2269	Tonalite	Habahe	372	372	1.83	6.48	0.1702	0.512820		-0.13	3.55	4.80	4.81	4.81	0.73	1.16	0.73			Yang Y et al.	2019	Journal of Asian Earth Sciences	175	230-246
43	ALT14-85	CAOB	Altai	86.6135	48.1533	Biotite granite	Habahe	381	381	2.13	9.10	0.1414	0.512596		-0.28	-0.82	1.90	1.87	1.87	0.97	1.17	0.97			Yang Y et al.	2019	Journal of Asian Earth Sciences	175	230-246
44	ALT14-86	CAOB	Altai	86.6135	48.1533	Biotite granite	Habahe	381	381	3.10	13.40	0.1397	0.512600		-0.29	-0.74	2.00	2.03	2.03	0.96	1.13	0.96			Yang Y et al.	2019	Journal of Asian Earth Sciences	175	230-246
45	ALT14-54	CAOB	Altai	86.5817	48.2628	Biotite granite	Habahe	359	359	3.56	20.20	0.1064	0.512708		-0.46	1.37	5.50	5.50	5.50	0.66	0.63	0.66			Yang Y et al.	2019	Journal of Asian Earth Sciences	175	230-246
46	ALT14-55	CAOB	Altai	86.5817	48.2628	Biotite granite	Habahe	359	359	4.50	24.60	0.1104	0.512698		-0.44	1.17	5.10	5.12	5.12	0.69	0.67	0.69			Yang Y et al.	2019	Journal of Asian Earth Sciences	175	230-246
47	K254/2	CAOB	orth Mongolia-Transbaikal rift b	106.9987	50.9600	Monzonite		230	230	2.95	18.10	0.0993	0.512289		-0.50	-6.81	-3.95	-3.95	-3.95	1.33	1.15	1.33			Yarmolyuk VV et al.	2001	Petrologiya	9(4)	302-328
48	B163/3	CAOB	orth Mongolia-Transbaikal rift b	106.9312	51.0470	Granite		229	229	27.50	152.00	0.1102	0.512460		-0.44	-3.47	-0.94	-0.94	-0.94	1.08	1.02	1.08			Yarmolyuk VV et al.	2001	Petrologiya	9(4)	302-328
49	MG59	CAOB	Western Junggar	83.8333	45.6333	Dacite		340	340	5.22	24.60	0.1285	0.512733		-0.35	1.85	4.81	4.81	4.81	0.70	0.75	0.70	0.75	0.70	Geng HY et al.	2011	Journal of Asian Earth Sciences	42(5)	854-866
50	MG40	CAOB	Western Junggar	83.9167	45.5000	Andesite		340	340	4.61	18.20	0.1534	0.512898		-0.22	5.07	6.95	6.95	6.95	0.53	0.64	0.53	0.64	0.53	Geng HY et al.	2011	Journal of Asian Earth Sciences	42(5)	854-866
51	MG36	CAOB	Western Junggar	83.9167	45.5000	Andesite		340	340	11.40	48.90	0.1409	0.512858		-0.28	4.29	6.71	6.71	6.71	0.55	0.61	0.55	0.61	0.55	Geng HY et al.	2011	Journal of Asian Earth Sciences	42(5)	854-866
52	HS28	CAOB	Western Junggar	85.0333	45.9917	Dacite		340	340	4.28	15.30	0.1695	0.512850		-0.14	4.14	5.31	5.31	5.31	0.66	1.03	0.66	1.03	0.66	Geng HY et al.	2011	Journal of Asian Earth Sciences	42(5)	854-866
53	HS27	CAOB	Western Junggar	85.0333	45.9917	Dacite		340	340	3.99	17.40	0.1386	0.512734		-0.30	1.87	4.39	4.39	4.39	0.74	0.84	0.74	0.84	0.74	Geng HY et al.	2011	Journal of Asian Earth Sciences	42(5)	854-866
54	HS13	CAOB	Western Junggar	85.1333	46.0000	Andesite		340	340	5.36	24.20	0.1340	0.512733		-0.32	1.85	4.57	4.57	4.57	0.72	0.80	0.72	0.80	0.72	Geng HY et al.	2011	Journal of Asian Earth Sciences	42(5)	854-866
55	FYD1	CAOB	Western Junggar	84.4250	45.6167	Andesite		340	340	5.85	26.30	0.1348	0.512756		-0.31	2.30	4.99	4.99	4.99	0.69	0.76	0.69	0.76	0.69	Geng HY et al.	2011	Journal of Asian Earth Sciences	42(5)	854-866
56	R-15-SUM	CAOB	Eastern Siberia	113.0000	57.0000	Syenite		290	290	0.34	2.08	0.1001	0.511891		-0.49	-14.57	-11.0	-11.0	-11.0	1.95	1.69	1.95	1.69	1.98	Rytsk EY et al.	2017	Doklady Earth Sciences	476	1043-1047
57	P1-030	CAOB	Eastern Siberia	113.0000	57.0000	Syenite		290	290	19.40	111.80	0.1047	0.511917		-0.47	-14.06	-10.7	-10.7	-10.7	1.92	1.72	1.92	1.72	1.96	Rytsk EY et al.	2017	Doklady Earth Sciences	476	1043-1047
58	P1-026	CAOB	Eastern Siberia	113.0000	57.0000	Syenite		290	290	0.82	5.06	0.0978	0.511899		-0.50	-14.42	-10.8	-10.8	-10.8	1.93	1.64	1.93	1.64	1.96	Rytsk EY et al.	2017	Doklady Earth Sciences	476	1043-1047
59	9186ZH	CAOB	Eastern Siberia	122.0000	57.0000	Syenite		290	290	0.80	2.94	0.1640	0.510945		-0.17	-33.03	-31.9	-31.8	-31.8	3.58	6.64	3.58		3.70	Rytsk EY et al.	2017	Doklady Earth Sciences	476	1043-1047
60	9178A	CAOB	Eastern Siberia	122.0000	57.0000	Syenite		290	290	0.51	3.39	0.0908	0.511096		-0.54	-30.08	-26.2	-26.2	-26.2	3.17	2.53	3.17	2.54	3.23	Rytsk EY et al.	2017	Doklady Earth Sciences	476	1043-1047
61	9178	CAOB	Eastern Siberia	122.0000	57.0000	Syenite		290	290	0.70	3.59	0.1183	0.511118		-0.40	-29.65	-26.8	-26.7	-26.7	3.21	3.22	3.21	3.23	3.28	Rytsk EY et al.	2017	Doklady Earth Sciences	476	1043-1047
62	V439	CAOB	SW Mongolia	95.6628	44.5294	Dacite		350	350	4.74	23.90	0.1199	0.512728		-0.39	1.76	5.20	5.19	5.19	0.68	0.69	0.68	0.65		Nguyen H et al.	2018	Journal of Asian Earth Sciences	164	322-343
63	V436	CAOB	SW Mongolia	95.6453	44.5003	Dacite		350	350	8.55	38.30	0.1350	0.512792		-0.31	3.00	5.80	5.76	5.76	0.63	0.69	0.63	0.60		Nguyen H et al.	2018	Journal of Asian Earth Sciences	164	322-343
64	W8053	CAOB	Tianshan	80.8240	42.4942	S-type granites		275	275	1.43	5.24	0.1649	0.512208		-0.16	-8.39	-7.30	-7.27	-7.27	1.62	2.92	1.62	2.92	1.62	Gou LL et al.	2015	Journal of Asian Earth Sciences	107	151-171
65	W8020	CAOB	Tianshan	80.7800	42.4925	S-type granites		275	275	6.12	30.54	0.1213	0.512078		-0.38	-10.92	-8.30	-8.28	-8.28	1.71	1.76	1.71	1.76	1.71	Gou LL et al.	2015	Journal of Asian Earth Sciences	107	151-171
66	W8015	CAOB	Tianshan	80.7800	42.4925	S-type granites		275	275	14.83	76.31	0.1176	0.512180		-0.40	-8.93	-6.20	-6.16	-6.16	1.54	1.54	1.54	1.54	1.54	Gou LL et al.	2015	Journal of Asian Earth Sciences	107	151-171
67	W8011	CAOB	Tianshan	80.8240	42.4942	S-type granites		275	275	1.86	5.38	0.2090	0.512394		0.06	-4.76	-5.20	-5.19	-5.19	1.36	22.81	1.36	22.62	1.36	Gou LL et al.	2015	Journal of Asian Earth Sciences	107	151-171
68	ZLT04-6	CAOB	NE China	122.7719	48.0033	Diorite		123	150	6.23	29.91	0.1169	0.512722		-0.41	1.64	3.06	3.17	3.17	0.68	0.67	0.68			Zhang Y.-T.et al	2006	Acta Petrologica Sinica	34(7)	923-926
69	JDY15	CAOB	Altai	87.1500	48.4800	Rhyolite		443	443	8.10	38.20	0.1282	0.512400		-0.35	-4.64	-0.80	-0.77	-0.77	1.24	1.34	1.24			Wang YJ et al.	2011	Journal of Asian Earth Sciences	42(5)	969-985
70	JDY13	CAOB	Altai	87.1500	48.4800	Rhyolite		443	443	5.14	26.30	0.1182	0.512378		-0.40	-5.07	-0.60	-0.63	-0.63	1.23	1.23	1.23			Wang YJ et al.	2011	Journal of Asian Earth Sciences	42(5)	969-985
71	HN18	CAOB	Altai	87.1500	48.4800	Rhyolite		443	443	7.74	35.10	0.1333	0.512419		-0.32	-4.27	-0.70	-0.69	-0.69	1.23	1.38	1.23			Wang YJ et al.	2011	Journal of Asian Earth Sciences	42(5)	969-985
72	HN17	CAOB	Altai	87.1500	48.4800	Rhyolite		443	443	7.12	28.30	0.1521	0.512433		-0.23	-4.00	-1.40	-1.48	-1.48	1.30	1.77	1.30			Wang YJ et al.	2011	Journal of Asian Earth Sciences	42(5)	969-985
73	HN08	CAOB	Altai	87.1000	48.7000	Rhyolite		443	443	7.75	36.40	0.1287	0.512408		-0.35	-4.49	-0.60	-0.64	-0.64	1.23	1.33	1.23			Wang YJ et al.	2011	Journal of Asian Earth Sciences	42(5)	969-985
74	HN06	CAOB	Altai	87.1000	48.7000	Rhyolite		443	443	8.22	38.20	0.1301	0.512384		-0.34	-4.95	-1.20	-1.19	-1.19	1.27	1.39	1.27			Wang YJ et al.	2011	Journal of Asian Earth Sciences	42(5)	969-985
75	HN03	CAOB	Altai	87.1000	48.7000	Rhyolite		443	443	8.25	39.00	0.1279	0.512351		-0.35	-5.60	-1.70	-1.71	-1.71	1.32	1.42	1.32			Wang YJ et al.	2011	Journal of Asian Earth Sciences	42(5)	969-985
76	BH42	CAOB	Altai	87.1500	48.4800	Rhyolite		410	410	6.14	28.60	0.1298	0.512377		-0.34	-5.09	-1.60	-1.59	-1.59	1.28	1.40	1.28			Wang YJ et al.	2011	Journal of Asian Earth Sciences	42(5)	969-985
77	BH40	CAOB	Altai	87.1500	48.4800	Rhyolite		410	410	6.41	29.10	0.1332	0.512379		-0.32	-5.05	-1.70	-1.73	-1.73	1.29	1.46	1.29			Wang YJ et al.	2011	Journal of Asian Earth Sciences	42(5)	969-985
78	BH38	CAOB	Altai	87.1500	48.4800	Dacite		410	410	5.19	26.80	0.1171	0.512210		-0.40	-8.35	-4.20	-4.18	-4.18	1.49	1.48	1.49			Wang YJ et al.	2011	Journal of Asian Earth Sciences	42(5)	969-985
79	BH36	CAOB	Altai	87.1500	48.4800	Dacite		410	410	5.57	28.50	0.1182	0.512258		-0.40	-7.41	-3.30	-3.30	-3.30	1.42	1.42	1.42			Wang YJ et al.	2011	Journal of Asian Earth Sciences	42(5)	969-985
80	BH34	CAOB	Altai	87.1500	48.4800	Dacite		410	410	4.25	19.30	0.1331	0.512231		-0.32	-7.94	-4.60	-4.61	-4.61	1.52	1.73	1.52			Wang YJ et al.	2011	Journal of Asian Earth Sciences	42(5)	969-985
81	BH12	CAOB	Altai	86.8500	48.7000	Rhyolite		410	410	6.98	26.90	0.1569	0.512465		-0.20	-3.37	-1.30	-1.29	-1.29	1.25	1.83	1.25			Wang YJ et al.	2011	Journal of Asian Earth Sciences	42(5)	969-985
82	06XJ-017	CAOB	Tianshan	80.9750	44.7100	Granodiorite	Lamasu	366	366	2.08	10.60	0.1195	0.512266		-0.39	-7.25	-3.65	-3.65	-3.65	1.41	1.43	1.41		1.42	Tang GJ et al.	2010	Lithos	119(3-4)	393-411
83	06XJ19-2	CAOB	Tianshan	80.9750	44.7100	Diorite	Lamasu	366	366	6.05	33.48	0.1101	0.512502		-0.44	-2.66	1.39	1.39	1.39	1.00	0.95	1.00		1.01	Tang GJ et al.	2010	Lithos	119(3-4)	393-411
84	06XJ20	CAOB	Tianshan	80.9750	44.7100	Granite	Lamasu	366	366	3.01	14.69	0.1248	0.512177		-0.37	-8.99	-5.63	-5.63	-5.63	1.57	1.66	1.57		1.59	Tang GJ et al.	2010	Lithos	119(3-4)	393-411
85	06XJ22	CAOB	Tianshan	80.9750	44.7100	Granodiorite	Lamasu	366	366	3.07	17.64	0.1060	0.512379		-0.46	-5.04	-0.80	-0.81	-0.81	1.18	1.09	1.18		1.19	Tang GJ et al.	2010	Lithos	119(3-4)	393-411
86	06XJ23-2	CAOB	Tianshan	80.9750	44.7100	Granite	Lamasu	366	366	2.94	16.29	0.1098	0.512239		-0.44	-7.78	-3.72	-3.72	-3.72	1.42	1.33	1.42		1.43	Tang GJ et al.	2010	Lithos	119(3-4)	393-411
87	06XJ-04	CAOB	Tianshan	81.4194	44.7403	Dacite	Dabate	316	316	5.80	26.80	0.1305	0.512672		-0.34	0.67	3.29	3.33	3.33	0.80	0.88	0.80		0.81	Tang GJ et al.	2010	Lithos	119(3-4)	393-411
88	06XJ-06	CAOB	Tianshan	81.4194	44.7403	Dacite	Dabate	316	316	3.16	14.44	0.1374	0.512510		-0.30	-2.50	0.05	-0.11	-0.11	1.08	1.28	1.08		1.08	Tang GJ et al.	2010	Lithos	119(3-4)	393-411
89	06XJ-010	CAOB	Tianshan	81.4194	44.7403	Granite	Dabate	290	290	3.35	10.46	0.1950	0.512635		-0.01	-0.06	0.00	0.00	0.00	1.04	4.16	1.04		1.06	Tang GJ et al.	2010	Lithos	119(3-4)	393-411
90	06XJ-13	CAOB	Tianshan	81.4194	44.7403	Granite	Dabate	290	290	5.12	18.47	0.1686	0.512534		-0.14	-2.02	-0.99	-0.99	-0.99	1.13	2.07	1.13		1.14	Tang GJ et al.	2010	Lithos	119(3-4)	393-411
91	06XJ-15	CAOB	Tianshan	81.4194	44.7403	Granite	Dabate	290	290	4.64	15.86	0.1780	0.512514		-0.10	-2.42	-1.72	-1.72	-1.72	1.19	2.70	1.19		1.20	Tang GJ et al.	2010	Lithos	119(3-4)	393-411
92	ZCZ1-3	NCC	NCC	121.3200	41.7094	Andesite		160	160	4.63	28.70	0.1012	0.511900		-0.49	-14.40	-12.9	-12.4	-12.4	1.96	1.69	1.96			Yang W et al.	2008	Lithos	102(1-2)	88-117
93	ZCZ1-2	NCC	NCC	121.3200	41.7094	Andesite		160	160	4.65	29.40	0.1005	0.511918		-0.49	-14.04	-12.5	-12.1	-12.1	1.93	1.66	1.93			Yang W et al.	2008	Lithos	102(1-2)	88-117
94	ZCZ1-1	NCC	NCC	121.3200	41.7094	Andesite		160	160	4.68	28.80	0.1001	0.511938		-0.49	-13.65	-12.1	-11.7	-11.7	1.90	1.62	1.90			Yang W et al.	2008	Lithos	102(1-2)	88-117
95	XLG-1	NCC	NCC	120.6428	41.7756	Andesite		177	177	3.82	20.50	0.0971	0.512459		-0.51	-3.49	-1.50	-1.24	-1.24	1.06	0.90	1.06			Yang W et al.	2008	Lithos	102(1-2)	88-117
96	LQ-29	NCC	NCC	120.7668	41.8341	Rhyolite		160	160	6.04	33.80	0.0987	0.511847		-0.50	-15.43	-13.4	-13.4	-13.4	2.04	1.72	2.04			Yang W et al.	2008	Lithos	102(1-2)	88-117

97	LQ-28	NCC	NCC	120.7668	41.8341	Rhyolite		160	160	6.31	35.10	0.1145	0.511847		-0.42	-15.43	-13.8	-13.8	-13.8	2.06	2.00	2.06			Yang W et al.	2008	Lithos	102(1-2)	88-117
98	LQ-27	NCC	NCC	120.7668	41.8341	Rhyolite		160	160	6.73	37.00	0.1172	0.511852		-0.40	-15.33	-13.7	-13.7	-13.7	2.06	2.04	2.06			Yang W et al.	2008	Lithos	102(1-2)	88-117
99	LQ-22	NCC	NCC	120.7668	41.8341	Andesite		160	160	12.01	62.30	0.0953	0.511981		-0.52	-12.82	-10.7	-10.7	-10.7	1.82	1.50	1.82			Yang W et al.	2008	Lithos	102(1-2)	88-117
100	LQ-21	NCC	NCC	120.7668	41.8341	Andesite		160	160	11.40	58.90	0.0950	0.511935		-0.52	-13.71	-11.6	-11.6	-11.6	1.89	1.56	1.89			Yang W et al.	2008	Lithos	102(1-2)	88-117
101	LQ-20	NCC	NCC	120.7668	41.8341	Andesite		160	160	11.10	58.00	0.1050	0.511958		-0.47	-13.26	-11.4	-11.4	-11.4	1.87	1.67	1.87			Yang W et al.	2008	Lithos	102(1-2)	88-117
102	LQ-19	NCC	NCC	120.7668	41.8341	Andesite		160	160	11.20	58.20	0.0924	0.511967		-0.53	-13.09	-11.0	-11.0	-11.0	1.84	1.48	1.84			Yang W et al.	2008	Lithos	102(1-2)	88-117
103	LQ-18	NCC	NCC	120.7668	41.8341	Andesite		160	160	11.40	58.90	0.1002	0.511956		-0.49	-13.30	-11.3	-11.3	-11.3	1.87	1.60	1.87			Yang W et al.	2008	Lithos	102(1-2)	88-117
104	LQ-17	NCC	NCC	120.7668	41.8341	Andesite		160	160	11.40	59.00	0.0924	0.511934		-0.53	-13.73	-11.6	-11.6	-11.6	1.89	1.53	1.89			Yang W et al.	2008	Lithos	102(1-2)	88-117
105	LQ-16	NCC	NCC	120.7668	41.8341	Andesite		160	160	11.00	57.60	0.1026	0.511947		-0.48	-13.48	-11.6	-11.6	-11.6	1.89	1.65	1.89			Yang W et al.	2008	Lithos	102(1-2)	88-117
106	LQ-15	NCC	NCC	120.7668	41.8341	Andesite		160	160	11.60	60.50	0.1035	0.511965		-0.47	-13.13	-11.2	-11.2	-11.2	1.86	1.64	1.86			Yang W et al.	2008	Lithos	102(1-2)	88-117
107	LQ-10	NCC	NCC	120.7668	41.8341	Rhyolite		160	160	9.58	54.20	0.1076	0.511930		-0.45	-13.81	-12.0	-12.0	-12.0	1.92	1.75	1.92			Yang W et al.	2008	Lithos	102(1-2)	88-117
108	LQ-9	NCC	NCC	120.7668	41.8341	Rhyolite		160	160	9.58	53.70	0.1068	0.511921		-0.46	-13.99	-12.1	-12.2	-12.2	1.93	1.75	1.93			Yang W et al.	2008	Lithos	102(1-2)	88-117
109	T6-007	CAOB	Tianshan	60.2878	42.1211	Granite cataclastic	Sheihdzheily	382	382	2.70	10.90	0.1492	0.512879		-0.24	4.70	7.00	7.02	7.02	0.56	0.64	0.56	0.49	0.47	Dolgopolova A et al.	2017	Gondwana Research	47	76-109
110	T6-010	CAOB	Tianshan	64.1050	42.1477	Quartz monzonite	Sautbai	288	288	6.80	38.60	0.1060	0.512373		-0.46	-5.17	-1.80	-1.84	-1.84	1.20	1.10	1.20	0.97	1.22	Dolgopolova A et al.	2017	Gondwana Research	47	76-109
111	T6-013	CAOB	Tianshan	63.3885	42.6713	cocratic tonalite (trondhjem	Bokaly	282	282	1.70	9.60	0.1069	0.512619		-0.46	-0.37	2.90	2.86	2.86	0.81	0.76	0.81	0.66	0.81	Dolgopolova A et al.	2017	Gondwana Research	47	76-109
112	T6-017	CAOB	Tianshan	63.5706	42.1818	anite two-mica, K-feldspatl	Altyntau	286	286	4.80	23.00	0.1261	0.512399		-0.36	-4.66	-2.10	-2.09	-2.09	1.22	1.31	1.22	1.14	1.24	Dolgopolova A et al.	2017	Gondwana Research	47	76-109
113	T6-021	CAOB	Tianshan	64.2906	41.6813	Granite leucocratic	eskuduk granite (North Tamd	274	274	9.10	45.20	0.1223	0.512361		-0.38	-5.40	-2.80	-2.80	-2.80	1.27	1.31	1.27	1.15	1.29	Dolgopolova A et al.	2017	Gondwana Research	47	76-109
114	T6-022	CAOB	Tianshan	63.5397	41.1996	monzonite (adamellite) cat	Auminzatau	286	286	5.90	31.10	0.1145	0.512338		-0.42	-5.85	-2.90	-2.85	-2.85	1.28	1.25	1.28	1.10	1.30	Dolgopolova A et al.	2017	Gondwana Research	47	76-109
115	T6-026	CAOB	Tianshan	66.2353	40.6572	Monzodiorite-porphyry	Kyngir	294	294	5.50	29.80	0.1124	0.512373		-0.43	-5.17	-2.00	-2.01	-2.01	1.22	1.17	1.22	1.03	1.24	Dolgopolova A et al.	2017	Gondwana Research	47	76-109
116	T6-040	CAOB	Tianshan	66.2315	40.6425	phyritic quartz monzonite gr	Madavat (Kattaich complex)	273	273	4.20	24.40	0.1047	0.512202		-0.47	-8.51	-5.30	-5.30	-5.30	1.47	1.32	1.47	1.18	1.47	Dolgopolova A et al.	2017	Gondwana Research	47	76-109
117	T6-042	CAOB	Tianshan	66.6949	40.3839	Quartz monzonite	Koshrabad	283	283	7.80	38.20	0.1243	0.512319		-0.37	-6.22	-3.60	-3.61	-3.61	1.34	1.41	1.34	1.24	1.35	Dolgopolova A et al.	2017	Gondwana Research	47	76-109
118	T6-047	CAOB	Tianshan	66.9227	39.3023	porphyritic granite K-feldspa	Lolabulaqsky	269	269	6.60	36.40	0.1101	0.512215		-0.44	-8.25	-5.30	-5.28	-5.28	1.47	1.37	1.47	1.22	1.47	Dolgopolova A et al.	2017	Gondwana Research	47	76-109
119	T6-050	CAOB	Tianshan	67.3376	38.9506	pporphyritic granite catacla	Western Gyssar batholith	321	321	6.50	39.90	0.0989	0.512320		-0.50	-6.20	-2.20	-2.20	-2.20	1.26	1.10	1.26	0.98	1.27	Dolgopolova A et al.	2017	Gondwana Research	47	76-109
120	T6-051	CAOB	Tianshan	67.3540	38.9504	pporphyritic granite catacla	Western Gyssar batholith	298	298	5.10	29.70	0.1041	0.512358		-0.47	-5.46	-1.90	-1.94	-1.94	1.22	1.10	1.22	0.98	1.23	Dolgopolova A et al.	2017	Gondwana Research	47	76-109
121	T6-057	CAOB	Tianshan	69.6488	40.8125	Quartz syenite	Kal'makyr	315	315	4.80	20.90	0.1382	0.512483		-0.30	-3.02	-0.70	-0.67	-0.67	1.13	1.34	1.13	1.15	1.14	Dolgopolova A et al.	2017	Gondwana Research	47	76-109
122	T6-061	CAOB	Tianshan	69.6485	40.8122	Granosyenite porphyry	Kal'makyr	422	422	2.60	13.20	0.1195	0.512297		-0.39	-6.65	-2.50	-2.49	-2.49	1.36	1.38	1.36	1.21	1.36	Dolgopolova A et al.	2017	Gondwana Research	47	76-109
123	T6-064	CAOB	Tianshan	69.7167	41.0527	Granodiorite	Karabash	420	420	4.10	21.80	0.1138	0.512764		-0.42	2.46	6.90	6.90	6.90	0.60	0.59	0.60	0.49	0.52	Dolgopolova A et al.	2017	Gondwana Research	47	76-109
124	T6-067	CAOB	Tianshan	69.7780	40.7756	Microgranite-porphyry	Sarycheku	304	304	3.80	22.80	0.1017	0.512346		-0.48	-5.70	-2.00	-2.01	-2.01	1.23	1.09	1.23	0.97	1.24	Dolgopolova A et al.	2017	Gondwana Research	47	76-109
125	T6-069	CAOB	Tianshan	69.7782	40.7763	Andesite-dacite	Sarycheku	311	311	3.90	24.20	0.0969	0.512338		-0.51	-5.85	-1.90	-1.89	-1.89	1.22	1.06	1.22	0.95	1.24	Dolgopolova A et al.	2017	Gondwana Research	47	76-109
126	T6-075	CAOB	Tianshan	70.1578	40.9710	Granite subporphyritic	Karasai/Kalkanata	429	429	6.60	32.30	0.1244	0.512176		-0.37	-9.01	-5.10	-5.05	-5.05	1.58	1.66	1.58	1.46	1.56	Dolgopolova A et al.	2017	Gondwana Research	47	76-109
127	T6-076	CAOB	Tianshan	69.9924	41.0172	Microgranite-porphyry	Karasai/Kalkanata	416	416	6.40	35.40	0.1098	0.512201		-0.44	-8.52	-3.90	-3.91	-3.91	1.47	1.39	1.47	1.24	1.47	Dolgopolova A et al.	2017	Gondwana Research	47	76-109
128	T6-077	CAOB	Tianshan	69.9817	41.0246	Quartz diorite-porphyry	Kyzylalmasai	314	314	4.40	24.10	0.1104	0.512425		-0.44	-4.15	-0.70	-0.70	-0.70	1.13	1.07	1.13	0.94	1.14	Dolgopolova A et al.	2017	Gondwana Research	47	76-109
129	T6-079	CAOB	Tianshan	69.9741	41.0265	anodiorite-porphyry catacla	Kyzylalmasai	429	429	4.70	23.50	0.1205	0.512382		-0.39	-4.99	-0.80	-0.82	-0.82	1.23	1.25	1.23	1.10	1.24	Dolgopolova A et al.	2017	Gondwana Research	47	76-109
130	T6-082	CAOB	Tianshan	69.9446	41.0150	Alkali feldspar granite	Nausalysai	318	318	2.70	15.40	0.1050	0.512410		-0.47	-4.45	-0.70	-0.73	-0.73	1.14	1.04	1.14	0.92	1.15	Dolgopolova A et al.	2017	Gondwana Research	47	76-109
131	T6-084	CAOB	Tianshan	69.8707	40.9606	Granite subporphyritic (pink	Akcha	401	401	4.90	31.00	0.0962	0.512132		-0.51	-9.87	-4.70	-4.73	-4.73	1.53	1.32	1.53	1.19	1.52	Dolgopolova A et al.	2017	Gondwana Research	47	76-109
132	T6-086	CAOB	Tianshan	69.9450	41.0469	Granite	Kyzylalmasai (Mezhdurech'e)	428	428	5.00	26.20	0.1157	0.512149		-0.41	-9.54	-5.10	-5.12	-5.12	1.58	1.55	1.58	1.38	1.57	Dolgopolova A et al.	2017	Gondwana Research	47	76-109
133	T6-089	CAOB	Tianshan	70.1579	40.9710	Andesite-dacite	Kairagach	308	308	3.90	21.00	0.1117	0.512422		-0.43	-4.21	-0.90	-0.87	-0.87	1.14	1.09	1.14	0.96	1.15	Dolgopolova A et al.	2017	Gondwana Research	47	76-109
134	T6-092	CAOB	Tianshan	70.4377	41.2914	Micropegmatite	Akchasai	397	397	5.80	34.90	0.1006	0.512384		-0.49	-4.95	-0.10	-0.09	-0.09	1.15	1.03	1.15	0.92	1.15	Dolgopolova A et al.	2017	Gondwana Research	47	76-109
135	T6-096	CAOB	Tianshan	70.4364	41.2906	Granite subporphyritic	Arashan	289	289	7.00	35.40	0.1197	0.512301		-0.39	-6.57	-3.70	-3.73	-3.73	1.36	1.37	1.36	1.21	1.37	Dolgopolova A et al.	2017	Gondwana Research	47	76-109
136	T6-101	CAOB	Tianshan	70.4448	41.0022	Granite-porphyry	Pirmirab	302	302	3.60	21.20	0.1041	0.512279		-0.47	-7.00	-3.40	-3.43	-3.43	1.34	1.21	1.34	1.08	1.35	Dolgopolova A et al.	2017	Gondwana Research	47	76-109
137	T6-104	CAOB	Tianshan	70.1425	40.9320	Andesite-dacite	Kochbulak, Nadak suite	301	301	4.40	24.00	0.1097	0.512328		-0.44	-6.05	-2.70	-2.71	-2.71	1.28	1.20	1.28	1.07	1.30	Dolgopolova A et al.	2017	Gondwana Research	47	76-109
138	T6-106	CAOB	Tianshan	70.1425	40.5545	Dacite	Kochbulak, Nadak suite	299	299	5.80	35.50	0.0983	0.512279		-0.50	-7.00	-3.20	-3.25	-3.25	1.33	1.15	1.33	1.03	1.34	Dolgopolova A et al.	2017	Gondwana Research	47	76-109
139	H5	CAOB	Tianshan	94.4813	41.7342	Granite	Hongliuhe	222	222	4.01	17.86	0.1356	0.512359		-0.31	-5.44	3.70	-3.71	-3.71	1.30	1.54	1.30	1.54		Wang T et al.	2008	Acta Petrologica Sinica	206(1-4)	42-57
140	1505-1	CAOB	Tianshan	94.4703	41.7801	Granite	Hongliuhe	222	222	4.50	25.97	0.1047	0.512328		-0.47	-6.05	-3.40	-3.44	-3.44	1.28	1.15	1.28	1.15		Wang T et al.	2008	Acta Petrologica Sinica	206(1-4)	42-57
141	1504-2	CAOB	Tianshan	94.4485	41.8129	Granite	Hongliuhe	222	222	4.69	21.86	0.1298	0.512373		-0.34	-5.17	-3.30	-3.27	-3.27	1.26	1.41	1.26	1.41		Wang T et al.	2008	Acta Petrologica Sinica	206(1-4)	42-57
142	1503/1-2(?)	CAOB	Tianshan	94.4092	41.8249	Granite	Hongliuhe	222	222	3.22	17.35	0.1122	0.512521		-0.43	-2.28	0.10	0.11	0.11	0.99	0.94	0.99	0.94		Wang T et al.	2008	Acta Petrologica Sinica	206(1-4)	42-57
143	1503/1-1(e)	CAOB	Tianshan	94.4092	41.8249	Granite	Hongliuhe	222	222	3.12	13.24	0.1423	0.512484		-0.28	-3.00	-1.50	-1.46	-1.46	1.12	1.42	1.12	1.42		Wang T et al.	2008	Acta Petrologica Sinica	206(1-4)	42-57
144	1501-2	CAOB	Tianshan	94.4703	41.7801	Granite	Hongliuhe	222	222	4.62	24.96	0.1119	0.512365		-0.43	-5.33	-2.90	-2.92	-2.92	1.24	1.17	1.24	1.18		Wang T et al.	2008	Acta Petrologica Sinica	206(1-4)	42-57
145	0601A01-2	CAOB	Altai	89.5773	46.5258	Adakite		389	389	3.99	19.11	0.1261	0.512683		-0.36	0.88	4.30	4.38	4.38	0.78	0.81	0.78			Niu HC et al.	2006	Journal of Asian Earth Sciences	28(4-6)	439-456
146	0601A010-2	CAOB	Altai	89.6912	46.5000	Adakite		389	389	3.51	16.76	0.1265	0.512644		-0.36	0.12	3.50	3.60	3.60	0.84	0.88	0.84			Niu HC et al.	2006	Journal of Asian Earth Sciences	28(4-6)	439-456
147	0909BB10-3	CAOB	Altai	89.2672	46.9203	Boninite		389	389	0.95	3.40	0.1681	0.512905		-0.15	5.21	6.60	6.63	6.63	0.59	0.82	0.59			Niu HC et al.	2006	Journal of Asian Earth Sciences	28(4-6)	439-456
148	M2	CAOB	Kuluketage block	90.5667	41.3000	Granite		816	600	4.71	27.60	0.1033	0.511749		-0.47	-17.34	-7.60	-10.2	-10.2	2.13	1.93	2.13			Cao XF et al.	2011	Precambrian Research	186(1-4)	205-219
149	K1	CAOB	Kuluketage block	90.5583	41.3000	Granite		816	600	7.89	46.41	0.1029	0.511802		-0.48	-16.31	-6.50	-9.13	-9.13	2.05	1.85	2.05			Cao XF et al.	2011	Precambrian Research	186(1-4)	205-219
150	ME4-2	CAOB	CAOB	104.1333	49.0167	Tonalite		242	242	2.19	15.25	0.0869	0.512661		-0.56	0.45	3.80	3.84	3.84	0.70	0.59	0.70	0.59	0.70	Shen P et al.	2018	Gondwana Research	59	161-194
151	ME2-7	CAOB	CAOB	104.1333	49.0167	Tonalite		242	242	0.57	3.81	0.0905	0.512650		-0.54	0.23	3.50	3.51	3.51	0.73	0.62	0.73	0.62	0.73	Shen P et al.	2018	Gondwana Research	59	161-194
152	ME2-5	CAOB	CAOB	104.1333	49.0167	Tonalite		242	242	1.99	13.18	0.0914	0.512620		-0.54	-0.35	2.90	2.90	2.90	0.78	0.66	0.78	0.66	0.78	Shen P et al.	2018	Gondwana Research	59	161-194
153	YUKH-29/3	CAOB	Mongolia	103.4468	44.7496	Basaltic trachyandesite		127	150	5.45	31.76	0.1037	0.512552		-0.47	-1.68	-0.17	0.10	0.10	0.93	0.83	0.93			Yarmolyuk VV et al.	2015	Journal of Asian Earth Sciences	111	604-623
154	YUKH-25/3	CAOB	Mongolia	99.1655	44.7310	Trachyandesite		123	150	5.01	31.42	0.0964	0.512548		-0.51	-1.76	-0.36	0.16	0.16	0.93	0.78	0.93			Yarmolyuk VV et al.	2015	Journal of Asian Earth Sciences	111	604-623
155	YUKH-22/26	CAOB	Mongolia	99.4186	45.2386	Trachyandesite		116	150	3.11	18.83	0.0999	0.512498		-0.49	-2.73	-1.30	-0.88	-0.88	1.01	0.87	1.01			Yarmolyuk VV et al.	2015	Journal of Asian Earth Sciences	111	604-623
156	YUKH-22/22	CAOB	Mongolia	99.0365	45.3829	Basaltic andesite		113	150	4.52	21.13	0.1292	0.512694		-0.34	1.09	2.07	2.38	2.38	0.74	0.82	0.74			Yarmolyuk VV et al.	2015	Journal of Asian Earth Sciences	111	604-623
157	YUKH-19/65	CAOB	Mongolia	103.2094	44.3828	Basaltic trachyandesite		123	150	12.48	68.74	0.1098	0.512605		-0.44	-0.64	0.65	1.02	1.02	0.86	0.80	0.86			Yarmolyuk VV et al.	2015	Journal of Asian Earth Sciences	111	604-623
158	YUKH-19/26	CAOB	Mongolia	102.3105	44.3248	Basaltic trachyandesite		94	150	4.90	23.15	0.1278	0.512750		-0.35	2.18	3.01	3.50	3.50	0.65	0.71	0.65			Yarmolyuk VV et al.	2015	Journal of Asian Earth Sciences	111	604-623
159	YUKH-16/9	CAOB	Mongolia	103.8093	44.4150	Trachyandesite		94	150	4.35	19.42	0.1355	0.512720		-0.31	1.60	2.33	2.77	2.77	0.71	0.84	0.71			Yarmolyuk VV et al.	2015	Journal of Asian Earth Sciences	111	604-623
160	YUKH-16/6	CAOB	Mongolia	103.8237	44.4458	Trachyandesite		99	150	4.52	20.75	0.1315	0.512710		-0.33	1.40	2.23	2.65	2.65	0.72	0.82	0.72			Yarmolyuk VV et al.	2015	Journal of Asian Earth Sciences	111	604-623
161	YUKH-16/3	CAOB	Mongolia	103.8251	44.4772	Basaltic trachyandesite		102	150	4.05	17.37	0.1408	0.512688		-0.28	0.98	1.70	2.04	2.04	0.77	0.97	0.77			Yarmolyuk VV et al.	2015	Journal of Asian Earth Sciences	111	604-623
162	15T92	CAOB	alkhash - Yili continental marg	81.0800	43.1200	Andesite		329	329	4.77	21.90	0.1317	0.512723		-0.33	1.66	4.42	4.39	4.39	0.73	0.79	0.73	0.80		Cao YC et al.	2017	Lithos	278-281	111-125
163	15T60	CAOB	alkhash - Yili continental marg	81.7800	43.1900	Granite		329	329	5.70	30.30	0.1137	0.512632		-0.42	-0.12	3.23	3.37	3.37	0.81	0.79	0.81	0.79		Cao YC et al.	2017	Lithos	278-281	111-125
164	15T56A	CAOB	alkhash - Yili continental marg	81.7800	43.1900	Rhyolite		329	329	3.56	21.00	0.1025	0.512527		-0.48	-2.17	1.63	1.79	1.79	0.94	0.85	0.94	0.86		Cao YC et al.	2017	Lithos	278-281	111-125
165	GFK09	CAOB	NE China	122.0000	52.6700	Qtz diorite porphyry		149	150	5.20	30.60	0.1027	0.512461		-0.48	-3.45	-1.67	-1.65	-1.65	1.07	0.95	1.07	0.95		Deng CZ et al.	2019	Lithos	326-327	341-357
166	GFK08	CAOB	NE China	122.0000	52.6700	Qtz diorite porphyry		149	150	5.29	31.40	0.1018	0.512448		-0.48	-3.71	-1.91	-1.89	-1.89	1.09	0.96	1.09	0.96		Deng CZ et al.	2019	Lithos	326-327	341-357
167	GFK07	CAOB	NE China	122.0000	52.6700	Qtz diorite porphyry		149	150	6.09	34.10	0.1080	0.512450		-0.45	-3.67	-1.98	-1.97	-1.97	1.10	1.01	1.10	1.01		Deng CZ et al.	2019	Lithos	326-327	341-357
168	GFK06	CAOB	NE China	122.0000	52.6700	Qtz diorite porphyry		149	150	5.56	36.20	0.0929	0.512452		-0.53	-3.63	-1.66	-1.64	-1.64	1.07	0.88	1.07	0.88		Deng CZ et al.	2019	Lithos	326-327	341-357
169	GFK05	CAOB	NE China	122.0000	52.6700	Qtz diorite porphyry		149	150	3.67	21.90	0.1013	0.512423		-0.48	-4.19	-2.38	-2.37	-2.37	1.13	0.99	1.13	0.99		Deng CZ et al.	2019	Lithos	326-327	341-357
170	HL-028	CAOB	NW China	90.0459	46.5802	Diorite porphyry		372	372	3.37	11.90	0.1725	0.512818		-0.12	3.52	4.70	4.67	4.67	0.74	1.23	0.74		0.74	Wu C et al.	2015	Ore Geology Reviews	71	373-404
171	HL-017	CAOB	NW China	90.0655	46.5492	Granodiorite		378	378	2.23	8.50	0.1598	0.512826		-0.19	3.67	5.50	5.45	5.45	0.68	0.92	0.68		0.68	Wu C et al.	2015	Ore Geology Reviews	71	373-404
172	HL-016	CAOB	NW China	90.0660	46.5497	Quartz diorite		379	379	4.54	16.40	0.1686	0.512853		-0.14	4.20	5.60	5.56	5.56	0.67	1.00	0.67		0.68	Wu C et al.	2015	Ore Geology Reviews	71	373-404
173	HL-012	CAOB	NW China	90.0640	46.5520	Quartz diorite		379	379	3.60	13.20	0.1661	0.512832		-0.16	3.78	5.30	5.26	5.26	0.70	1.02	0.70		0.70	Wu C et al.	2015	Ore Geology Reviews	71	373-404
174	HL-011	CAOB	NW China	90.0920	46.5220	Alkali granite porphyry		327	327	6.42	32.40	0.1207	0.512754		-0.39	2.26	5.40	5.43	5.43	0.64	0.65	0.64		0.64	Wu C et al.	2015	Ore Geology Reviews	71	373-404
175	N07	CAOB	Mongolian border in China	103.7500	41.8000	Q-Monzonite		228	228	17.38	108.00	0.0974	0.512384		-0.50	-4.95	-2.10	-2.07	-2.07	1.17	1.00	1.17	1.00		Wang T et al.	2004	Journal of Asian Earth Sciences	23（5）	715-729
176	I09	CAOB	Mongolian border in China	103.7500	41.8000	Q-monzonite		228	228	12.52	73.22	0.1034	0.512385		-0.47	-4.94	-2.20	-2.22	-2.22	1.18	1.06	1.18	1.06		Wang T et al.	2004	Journal of Asian Earth Sciences	23（5）	715-729
177	I32	CAOB	Mongolian border in China	103.7500	41.8000	Garnet monzogranit		228	228	1.66	5.77	0.1740	0.512560		-0.12	-1.52	-0.80	-0.86	-0.86	1.07	2.26	1.07	2.25		Wang T et al.	2004	Journal of Asian Earth Sciences	23（5）	715-729
178	I13	CAOB	Mongolian border in China	103.7500	41.8000	Q-monzonite		228	228	17.23	106.58	0.0978	0.512422		-0.50	-4.21	-1.30	-1.34	-1.34	1.11	0.96	1.11	0.96		Wang T et al.	2004	Journal of Asian Earth Sciences	23（5）	715-729
179	7	CAOB	Mongolian border in China	103.7500	41.8000	Bi-Monzogranite		228	228	7.65	44.58	0.1037	0.512381		-0.47	-5.01	-2.30	-2.31	-2.31	1.19	1.07	1.19	1.07		Wang T et al.	2004	Journal of Asian Earth Sciences	23（5）	715-729
180	20YJ-140	CAOB	NE China	129.0000	42.4600	Andesite		57	150	4.66	30.64	0.0936	0.512853		-0.52	4.19	4.92	6.17	6.17	0.44	0.38	0.44			Guo F et al.	2009	Lithos	112(3-4)	321-341
181	20YJ-139	CAOB	NE China	129.0000	42.4600	Andesite		57	150	4.94	29.16	0.1041	0.512745		-0.47	2.09	2.74	3.86	3.86	0.62	0.56	0.62			Guo F et al.	2009	Lithos	112(3-4)	321-341
182	20YJ-134	CAOB	NE China	129.0000	42.4600	Andesite		57	150	6.03	40.60	0.0913	0.512580		-0.54	-1.13	-0.39	0.89	0.89	0.87	0.71	0.87			Guo F et al.	2009	Lithos	112(3-4)	321-341
183	20YJ-133	CAOB	NE China	129.0000	42.4600	Andesite		57	150	6.76	42.16	0.0986	0.512783		-0.50	2.83	3.52	4.71	4.71	0.56	0.49	0.56			Guo F et al.	2009	Lithos	112(3-4)	321-341
184	20YJ-132	CAOB	NE China	129.0000	42.4600	Andesite		57	150	6.60	42.40	0.0957	0.512652		-0.51	0.27	0.98	2.21	2.21	0.76	0.64	0.76			Guo F et al.	2009	Lithos	112(3-4)	321-341
185	20YJ-127	CAOB	NE China	129.0000	42.4600	Andesite		57	150	6.91	44.62	0.0953	0.512785		-0.52	2.87	3.58	4.81	4.81	0.55	0.47	0.55			Guo F et al.	2009	Lithos	112(3-4)	321-341
186	20YJ-125	CAOB	NE China	129.0000	42.4600	Andesite		57	150	6.35	41.17	0.0949	0.512776		-0.52	2.69	3.41	4.64	4.64	0.56	0.48	0.56			Guo F et al.	2009	Lithos	112(3-4)	321-341
187	20YJ-121	CAOB	NE China	129.0000	42.4600	Andesite		57	150	6.01	40.87	0.0904	0.512818		-0.54	3.51	4.26	5.55	5.55	0.49	0.41	0.49			Guo F et al.	2009	Lithos	112(3-4)	321-341
188	05YJ-73	CAOB	NE China	129.0000	42.4600	Andesite		57	150	6.09	42.76	0.0876	0.512762		-0.55	2.42	3.19	4.51	4.51	0.57	0.47	0.57			Guo F et al.	2009	Lithos	112(3-4)	321-341
189	05YJ-65	CAOB	NE China	129.2200	42.8500	Andesite		57	150	4.84	34.14	0.0872	0.512935		-0.56	5.79	6.56	7.89	7.89	0.30	0.26	0.30			Guo F et al.	2009	Lithos	112(3-4)	321-341
190	05YJ-64	CAOB	NE China	129.2200	42.8500	Andesite		57	150	4.56	32.01	0.0875	0.512940		-0.56	5.89	6.67	7.98	7.98	0.29	0.25	0.29			Guo F et al.	2009	Lithos	112(3-4)	321-341
191	05YJ-63	CAOB	NE China	129.2200	42.8500	Andesite		57	150	4.47	31.51	0.0871	0.512954		-0.56	6.16	6.93	8.26	8.26	0.26	0.24	0.26			Guo F et al.	2009	Lithos	112(3-4)	321-341
192	05YJ-61	CAOB	NE China	129.2200	42.8500	Andesite		57	150	4.97	34.52	0.0885	0.512945		-0.55	5.99	6.75	8.06	8.06	0.28	0.25	0.28			Guo F et al.	2009	Lithos	112(3-4)	321-341
193	05YJ-21	CAOB	NE China	129.4400	42.8400	Andesite		57	150	6.66	46.84	0.0875	0.512774		-0.56	2.65	3.42	4.74	4.74	0.55	0.45	0.55			Guo F et al.	2009	Lithos	112(3-4)	321-341
194	05YJ-20	CAOB	NE China	129.4400	42.8400	Andesite		57	150	6.67	47.29	0.0867	0.512777		-0.56	2.71	3.49	4.82	4.82	0.55	0.45	0.55			Guo F et al.	2009	Lithos	112(3-4)	321-341

195	05YJ-18	CAOB	NE China	129.3300	42.8500	Andesite		57	150	4.61	32.95	0.0860	0.512907		-0.56	5.25	6.03	7.37	7.37	0.34	0.29	0.34			Guo F et al.	2009	Lithos	112(3-4)	321-341
196	05YJ-16	CAOB	NE China	129.3300	42.8500	Andesite		57	150	4.67	33.82	0.0849	0.512917		-0.57	5.44	6.23	7.58	7.58	0.32	0.28	0.32			Guo F et al.	2009	Lithos	112(3-4)	321-341
197	05YJ-14	CAOB	NE China	129.3300	42.8500	Andesite		57	150	6.00	44.34	0.0831	0.512826		-0.58	3.67	4.47	5.84	5.84	0.46	0.38	0.46			Guo F et al.	2009	Lithos	112(3-4)	321-341
198	05YJ-13	CAOB	NE China	129.3300	42.8500	Andesite		57	150	5.74	41.45	0.0851	0.512844		-0.57	4.02	4.81	6.15	6.15	0.44	0.36	0.44			Guo F et al.	2009	Lithos	112(3-4)	321-341
199	05YJ-1	CAOB	NE China	129.3300	42.8500	Andesite		57	150	5.53	41.30	0.0823	0.512850		-0.58	4.14	4.93	6.33	6.33	0.42	0.35	0.42			Guo F et al.	2009	Lithos	112(3-4)	321-341
200	ZH9	CAOB	Kazakhstan	72.0000	51.6967	Granodiorite		447	447	2.74	12.98	0.1276	0.512540		-0.35	-1.91	2.00	2.03	2.03	1.02	1.08	1.02	0.89		Kroner A et al.	2008	Journal of Asian Earth Sciences	32(2-4)	118-130
201	ZH4	CAOB	Kazakhstan	71.5000	51.6667	Granodiorite		453	453	2.78	13.20	0.1276	0.512561		-0.35	-1.50	2.50	2.49	2.49	0.98	1.04	0.98	0.86		Kroner A et al.	2008	Journal of Asian Earth Sciences	32(2-4)	118-130
202	VE9	CAOB	Kazakhstan	82.5467	48.3150	Granodiorite		250	250	28.79	106.30	0.1637	0.512854		-0.17	4.21	5.30	5.27	5.27	0.59	0.90	0.59	0.60		Kroner A et al.	2008	Journal of Asian Earth Sciences	32(2-4)	118-130
203	St11	CAOB	Kazakhstan	70.5617	52.8783	Monzonite		481	481	3.02	15.80	0.1155	0.512330		-0.41	-6.01	-1.00	-1.02	-1.02	1.29	1.27	1.29	1.10		Kroner A et al.	2008	Journal of Asian Earth Sciences	32(2-4)	118-130
204	AK21	CAOB	Kazakhstan	72.0917	53.1017	Monzonite		457	457	2.35	9.91	0.1435	0.512696		-0.27	1.13	4.20	4.23	4.23	0.84	0.99	0.84	0.76		Kroner A et al.	2008	Journal of Asian Earth Sciences	32(2-4)	118-130
205	13ts15D	CAOB	Tianshan	83.9730	43.1006	Foliated diorite		421	421	5.20	22.98	0.1367	0.512384		-0.30	-4.95	-1.73	-1.73	-1.73	1.30	1.51	1.30	1.52		Zhong LL et al.	2017	Gondwana Research	45	254-274
206	13ts14I	CAOB	Tianshan	83.9701	43.1029	Coarse-grained diorite		353	353	3.79	17.55	0.1307	0.512584		-0.34	-1.05	1.92	1.92	1.92	0.95	1.04	0.95	1.04		Zhong LL et al.	2017	Gondwana Research	45	254-274
207	13ts14B	CAOB	Tianshan	83.9701	43.1029	Undeformed granite stock		292	292	6.76	34.58	0.1182	0.512262		-0.40	-7.33	-4.41	-4.41	-4.41	1.41	1.42	1.41	1.42		Zhong LL et al.	2017	Gondwana Research	45	254-274
208	TC-10	CAOB	Tianshan	90.5000	43.5000	Rhyolite	Bogda	311	311	4.15	23.30	0.1077	0.512801		-0.45	3.18	6.76	6.71	6.76	0.50	0.50	0.52	0.50		Xie W et al.	2016	Gondwana Research	39	165-179
209	DST-06	CAOB	Tianshan	90.5000	43.5000	Rhyolite	Bogda	311	311	5.04	26.90	0.1133	0.512781		-0.42	2.79	6.20	6.10	6.20	0.56	0.56	0.57	0.56		Xie W et al.	2016	Gondwana Research	39	165-179
210	DST-05	CAOB	Tianshan	90.5000	43.5000	Rhyolite	Bogda	311	311	6.15	33.00	0.1127	0.512788		-0.43	2.93	6.36	6.26	6.36	0.55	0.55	0.56	0.55		Xie W et al.	2016	Gondwana Research	39	165-179
211	X-617	CAOB	Altai, unit 8	95.3708	43.0181	Granite	Omrtag	288	288	14.57	59.87	0.1500	0.512887	11	-0.24	4.86	4.96	6.57	6.57	0.52	0.63	0.52		0.63	Wang CS et al.	2009	Acta Petrologica Sinica	25(12)	3182-3196
212	KCA3-232	CAOB	NE China	125.7867	50.2489	Granodiorite		476	476	1.13	5.16	0.1443	0.512699		-0.27	1.19	4.40	4.37	4.37	0.85	0.99	0.85	0.99	0.85	Zhao C et al.	2018	Journal of Asian Earth Sciences	165	352-370
213	DW-06	CAOB	NE China	125.7903	50.2436	Granodiorite		476	476	0.97	5.09	0.0977	0.512658		-0.50	0.39	6.40	6.40	6.40	0.68	0.65	0.68	0.65	0.69	Zhao C et al.	2018	Journal of Asian Earth Sciences	165	352-370
214	5401-274	CAOB	NE China	125.7872	50.2450	Granodiorite		476	476	1.26	7.26	0.1578	0.512672		-0.20	0.66	3.00	3.02	3.02	0.96	1.30	0.96	1.31	0.96	Zhao C et al.	2018	Journal of Asian Earth Sciences	165	352-370
215	5001-451	CAOB	NE China	125.7872	50.2428	Granodiorite		476	476	1.00	5.49	0.1385	0.512660		-0.30	0.43	4.00	3.97	3.97	0.88	0.99	0.88	1.00	0.89	Zhao C et al.	2018	Journal of Asian Earth Sciences	165	352-370
216	14DB3-28	CAOB	NE China	125.7861	50.2494	Granodiorite		476	476	1.41	7.64	0.1124	0.512688		-0.43	0.98	5.70	6.10	6.10	0.71	0.70	0.71	0.75	0.75	Zhao C et al.	2018	Journal of Asian Earth Sciences	165	352-370
217	06XJ79-1	CAOB	Western Tianshan	82.5833	43.6167	Granitic instrusive	Zhongyangchang	317	317			0.1470	0.512681		-0.25	0.84	2.85	2.85	2.85	0.84	1.07	0.84	1.07	0.85	Tang GJ et al.	2014	Lithos	202-203	85-99
218	06XJ-78	CAOB	Western Tianshan	82.5833	43.6167	Granitic instrusive	Zhongyangchang	317	317			0.1720	0.512598		-0.13	-0.78	0.22	0.22	0.22	1.05	2.01	1.05	2.01	1.06	Tang GJ et al.	2014	Lithos	202-203	85-99
219	06XJ-74	CAOB	Western Tianshan	82.5833	43.6167	Granitic dike	Zhongyangchang	310	310			0.1560	0.512668		-0.21	0.59	2.20	2.20	2.20	0.89	1.27	0.89	1.27	0.89	Tang GJ et al.	2014	Lithos	202-203	85-99
220	06XJ72	CAOB	Western Tianshan	82.5833	43.6167	Granitic instrusive	Zhongyangchang	317	317			0.1340	0.512635		-0.32	-0.06	2.48	2.48	2.48	0.87	0.98	0.87	0.99	0.88	Tang GJ et al.	2014	Lithos	202-203	85-99
221	06XJ71	CAOB	Western Tianshan	82.5833	43.6167	Granitic instrusive	Zhongyangchang	317	317			0.1340	0.512631		-0.32	-0.14	2.40	2.40	2.40	0.88	0.99	0.88	0.99	0.88	Tang GJ et al.	2014	Lithos	202-203	85-99
222	06XJ69	CAOB	Western Tianshan	82.5833	43.6167	Granitic instrusive	Zhongyangchang	317	317			0.1250	0.512619		-0.36	-0.37	2.53	2.53	2.53	0.87	0.91	0.87	0.91	0.87	Tang GJ et al.	2014	Lithos	202-203	85-99
223	FW04-334	CAOB	NE China	124.5491	40.9110	Syenite		227	227	18.50	128.00	0.0889	0.511887		-0.55	-14.65	-11.6	-11.5	-11.5	1.94	1.54	1.94			Zhu YS et al.	2015	Journal of Asian Earth Sciences	117	184-207
224	FW04-333	CAOB	NE China	124.5491	40.9110	Syenite		227	227	20.10	138.00	0.0884	0.511900		-0.55	-14.40	-11.3	-11.3	-11.3	1.92	1.52	1.92			Zhu YS et al.	2015	Journal of Asian Earth Sciences	117	184-207
225	FW04-332	CAOB	NE China	124.5491	40.9110	Syenite		227	227	18.10	124.00	0.0891	0.511903		-0.55	-14.34	-11.3	-11.2	-11.2	1.91	1.52	1.91			Zhu YS et al.	2015	Journal of Asian Earth Sciences	117	184-207
226	11SM61	CAOB	NE China	124.6131	40.9133	Syenite		227	227	12.30	82.00	0.0904	0.511855		-0.54	-15.27	-12.2	-12.2	-12.2	1.99	1.60	1.99			Zhu YS et al.	2015	Journal of Asian Earth Sciences	117	184-207
227	11SM50	CAOB	NE China	124.6131	40.9133	Syenite		227	227	25.10	168.00	0.0901	0.511850		-0.54	-15.37	-12.3	-12.3	-12.3	2.00	1.60	2.00			Zhu YS et al.	2015	Journal of Asian Earth Sciences	117	184-207
228	11SM47	CAOB	NE China	124.6131	40.9133	Syenite		227	227	14.00	95.10	0.0890	0.511862		-0.55	-15.14	-12.1	-12.0	-12.0	1.98	1.57	1.98			Zhu YS et al.	2015	Journal of Asian Earth Sciences	117	184-207
229	11SM46	CAOB	NE China	124.6131	40.9133	Syenite		227	227	14.20	96.80	0.0886	0.511889		-0.55	-14.61	-11.5	-11.5	-11.5	1.94	1.53	1.94			Zhu YS et al.	2015	Journal of Asian Earth Sciences	117	184-207
230	11SM18	CAOB	NE China	124.3907	40.9949	Syenite		227	227	7.21	46.30	0.0940	0.511832		-0.52	-15.72	-12.8	-12.8	-12.8	2.04	1.67	2.04			Zhu YS et al.	2015	Journal of Asian Earth Sciences	117	184-207
231	11SM15	CAOB	NE China	124.3907	40.9949	Syenite		227	227	41.20	178.00	0.1400	0.511890		-0.29	-14.59	-13.0	-13.0	-13.0	2.05	2.59	2.05			Zhu YS et al.	2015	Journal of Asian Earth Sciences	117	184-207
232	11SM14	CAOB	NE China	124.3907	40.9949	Syenite		227	227	13.90	82.30	0.1023	0.511859		-0.48	-15.20	-12.5	-12.5	-12.5	2.01	1.76	2.01			Zhu YS et al.	2015	Journal of Asian Earth Sciences	117	184-207
233	09JH89	CAOB	NE China	124.3907	40.9949	Syenite		227	227	1.88	20.60	0.0552	0.511785		-0.72	-16.64	-12.6	-12.5	-12.5	2.02	1.31	2.02			Zhu YS et al.	2015	Journal of Asian Earth Sciences	117	184-207
234	09JH83	CAOB	NE China	124.3907	40.9949	Syenite		227	227	2.77	18.90	0.0887	0.511855		-0.55	-15.27	-12.2	-12.2	-12.2	1.99	1.58	1.99			Zhu YS et al.	2015	Journal of Asian Earth Sciences	117	184-207
235	09JH81	CAOB	NE China	124.3907	40.9949	Syenite		227	227	2.87	14.60	0.1192	0.511901		-0.39	-14.38	-12.2	-12.1	-12.1	1.99	2.01	1.99			Zhu YS et al.	2015	Journal of Asian Earth Sciences	117	184-207
236	09JH80	CAOB	NE China	124.3907	40.9949	Syenite		227	227	2.82	17.70	0.0964	0.511844		-0.51	-15.49	-12.6	-12.6	-12.6	2.02	1.69	2.02			Zhu YS et al.	2015	Journal of Asian Earth Sciences	117	184-207
237	09JH79	CAOB	NE China	124.3907	40.9949	Syenite		227	227	30.40	198.00	0.0925	0.511832		-0.53	-15.72	-12.7	-12.7	-12.7	2.03	1.65	2.03			Zhu YS et al.	2015	Journal of Asian Earth Sciences	117	184-207
238	09JH65	CAOB	NE China	124.5491	40.9110	Syenite		227	227	19.20	126.00	0.0923	0.511881		-0.53	-14.77	-11.8	-11.7	-11.7	1.96	1.59	1.96			Zhu YS et al.	2015	Journal of Asian Earth Sciences	117	184-207
239	09JH58	CAOB	NE China	124.6131	40.9133	Syenite		227	227	19.30	131.00	0.0895	0.511830		-0.54	-15.76	-12.7	-12.7	-12.7	2.03	1.62	2.03			Zhu YS et al.	2015	Journal of Asian Earth Sciences	117	184-207
240	S-B	CAOB	Central Kazakhstan	77.3200	47.0000	Granodiorite		311	311	2.36	11.81	0.1211	0.512757		-0.38	2.32	5.30	5.33	5.33	0.64	0.65	0.64	0.65	0.64	Li GM et al.	2015	Gondwana Research	37	408-425
241	S-A	CAOB	Central Kazakhstan	77.3300	47.0000	Granodiorite		310	310	2.32	12.40	0.1131	0.512722		-0.42	1.64	4.90	4.94	4.94	0.67	0.65	0.67	0.65	0.67	Li GM et al.	2015	Gondwana Research	37	408-425
242	S1-1	CAOB	Central Kazakhstan	77.4300	47.0000	Granodiorite		311	311	3.04	16.11	0.1144	0.512781		-0.42	2.79	6.00	6.06	6.06	0.58	0.57	0.58	0.57	0.58	Li GM et al.	2015	Gondwana Research	37	408-425
243	L-1	CAOB	Central Kazakhstan	74.6000	47.3833	Monzonite		305	305	6.84	36.95	0.1118	0.512624		-0.43	-0.27	3.00	3.03	3.03	0.82	0.79	0.82	0.79	0.82	Li GM et al.	2015	Gondwana Research	37	408-425
244	Kzh-1	CAOB	Central Kazakhstan	74.3833	47.4667	Monzonite		300	300	2.98	9.69	0.1860	0.512485		-0.05	-2.98	-2.60	-2.57	-2.57	1.26	3.63	1.26	3.65	1.27	Li GM et al.	2015	Gondwana Research	37	408-425
245	Kt-1	CAOB	Central Kazakhstan	77.3667	47.8500	Monzonite		300	300	1.83	10.40	0.1068	0.512062		-0.46	-11.24	-7.80	-7.79	-7.79	1.69	1.55	1.69	1.55	1.70	Li GM et al.	2015	Gondwana Research	37	408-425
246	K-3(Li)	CAOB	Central Kazakhstan	74.9667	46.9833	Granodiorite		325	325	3.37	18.85	0.1082	0.512508		-0.45	-2.54	1.10	1.14	1.14	0.99	0.93	0.99	0.93	0.99	Li GM et al.	2015	Gondwana Research	37	408-425
247	H-2	CAOB	Central Kazakhstan	74.4500	47.3000	Granodiorite		300	300	2.96	15.14	0.1183	0.512738		-0.40	1.95	5.00	4.95	4.95	0.66	0.66	0.66	0.66	0.66	Li GM et al.	2015	Gondwana Research	37	408-425
248	B-2(Li)	CAOB	Central Kazakhstan	74.6833	47.0833	Granodiorite		312	312	3.39	16.21	0.1264	0.512494		-0.36	-2.81	0.00	-0.01	-0.01	1.07	1.14	1.07	1.15	1.07	Li GM et al.	2015	Gondwana Research	37	408-425
249	EL15-8-1	CAOB	NE China	111.3972	43.3978	Quartz-monzonite enclave		242	242	8.44	40.30	0.1268	0.512523		-0.36	-2.24	-0.10	-0.08	-0.08	1.02	1.10	1.02	1.10	1.02	Yuan LL et al.	2017	Journal of Asian Earth Sciences	165	37-58
250	EL14-20-1	CAOB	NE China	111.2261	42.8650	Diorite porphyrite		242	242	3.38	15.16	0.1348	0.512427		-0.31	-4.12	-2.30	-2.20	-2.20	1.19	1.39	1.19	1.40	1.19	Yuan LL et al.	2017	Journal of Asian Earth Sciences	165	37-58
251	EL14-14-1	CAOB	NE China	111.3342	43.2536	Monzodiorite porphyrite		242	242	10.28	60.87	0.1022	0.512473		-0.48	-3.22	-0.40	-0.30	-0.30	1.04	0.93	1.04	0.93	1.04	Yuan LL et al.	2017	Journal of Asian Earth Sciences	165	37-58
252	EL14-13-1	CAOB	NE China	111.3381	43.2622	Monzogranite		242	242	2.52	14.29	0.1066	0.512478		-0.46	-3.12	-0.40	-0.34	-0.34	1.04	0.96	1.04	0.96	1.05	Yuan LL et al.	2017	Journal of Asian Earth Sciences	165	37-58
253	EL14-11-2	CAOB	NE China	111.4036	43.2294	Alkali-feldspar granite		242	242	0.89	4.94	0.1085	0.512496		-0.45	-2.77	-0.10	-0.05	-0.05	1.02	0.95	1.02	0.95	1.02	Yuan LL et al.	2017	Journal of Asian Earth Sciences	165	37-58
254	EL13-9-4	CAOB	NE China	111.4597	43.3836	Syenogranite		242	242	2.10	10.50	0.1211	0.512542		-0.38	-1.87	0.40	0.46	0.46	0.98	1.00	0.98	1.00	0.98	Yuan LL et al.	2017	Journal of Asian Earth Sciences	165	37-58
255	EL13-9-2	CAOB	NE China	111.4597	43.3836	Sodium-rich granite		242	242	2.33	12.33	0.1142	0.512538		-0.42	-1.95	0.50	0.60	0.60	0.97	0.94	0.97	0.94	0.97	Yuan LL et al.	2017	Journal of Asian Earth Sciences	165	37-58
256	EL13-10-3	CAOB	NE China	111.5083	43.3186	Graphic granite		242	242	0.57	4.03	0.0852	0.512431		-0.57	-4.04	-0.70	-0.59	-0.59	1.06	0.85	1.06	0.85	1.07	Yuan LL et al.	2017	Journal of Asian Earth Sciences	165	37-58
257	EL13-10-2	CAOB	NE China	111.5083	43.3186	Graphic granite		242	242	0.49	3.75	0.0791	0.512447		-0.60	-3.73	-0.20	-0.09	-0.09	1.02	0.80	1.02	0.80	1.03	Yuan LL et al.	2017	Journal of Asian Earth Sciences	165	37-58
258	EL10-5-1	CAOB	NE China	111.3042	43.1942	Sodium-rich granite		242	242	2.37	15.13	0.0947	0.512375		-0.52	-5.13	-1.90	-1.98	-1.98	1.18	0.99	1.18	1.00	1.18	Yuan LL et al.	2017	Journal of Asian Earth Sciences	165	37-58
259	EL10-23-2	CAOB	NE China	111.4992	43.3683	Granite porphyry		242	242	10.33	31.12	0.2009	0.512715		0.02	1.50	1.40	1.37	1.37	0.89	5.11	0.89	5.11	0.88	Yuan LL et al.	2017	Journal of Asian Earth Sciences	165	37-58
260	NJ11-45	CAOB	Tianshan	81.5000	42.1000	Quartz-albite porphyry	Xiaotikanlike Formation	290	290	11.90	73.60	0.0900	0.512238		-0.54	-7.80	-3.79	-3.85	-3.85	1.37	1.12	1.37	1.12		Liu DD.et al,	2014	International Journal of Earth Sciences	103（3）	737-756
261	NJ11-40	CAOB	Tianshan	81.5000	42.1000	Quartz-albite porphyry	Xiaotikanlike Formation	290	290	3.01	19.50	0.0960	0.512262		-0.51	-7.33	-3.54	-3.61	-3.61	1.35	1.15	1.35	1.15		Liu DD.et al,	2014	International Journal of Earth Sciences	103（3）	737-756
262	NJ11-39	CAOB	Tianshan	81.5000	42.1000	Quartz-albite porphyry	Xiaotikanlike Formation	290	290	4.97	25.50	0.1164	0.512289		-0.41	-6.81	-3.79	-3.84	-3.84	1.37	1.35	1.37	1.35		Liu DD.et al,	2014	International Journal of Earth Sciences	103（3）	737-756
263	NJ11-05	CAOB	Tianshan	81.5000	42.1000	Quartz-albite porphyry	Xiaotikanlike Formation	290	290	9.02	49.60	0.0996	0.512130		-0.49	-9.91	-6.26	-6.32	-6.32	1.57	1.36	1.57	1.36		Liu DD.et al,	2014	International Journal of Earth Sciences	103（3）	737-756
264	NJ11-02	CAOB	Tianshan	81.5000	42.1000	Quartz-albite porphyry	Xiaotikanlike Formation	290	290	14.30	73.40	0.1087	0.512143		-0.45	-9.66	-6.35	-6.40	-6.40	1.57	1.46	1.57	1.46		Liu DD.et al,	2014	International Journal of Earth Sciences	103（3）	737-756
265	FS-28	NCC	NNCC	124.5000	42.0000	Andesite		123	150	7.07	50.46	0.0853	0.511770		-0.57	-16.93	-15.4	-14.8	-14.8	2.14	1.63	2.14	1.64		Pang CJ et al.	2015	Lithos	227	77-93
266	T5-029	CAOB	CAOB	75.8947	42.3056	Diorite		440	440	6.31	30.61	0.1246	0.512282		-0.37	-6.94	-2.90	-2.90	-2.90	1.41	1.48	1.41	1.29		Kröner A et al.	2014	Gondwana Research	25（1）	103-125
267	MV185/6	CAOB	CAOB	72.3380	41.3440	Granodiorite		445	445	6.50	33.40	0.1177	0.512276		-0.40	-7.06	-2.60	-2.58	-2.58	1.39	1.39	1.39	1.21		Kröner A et al.	2014	Gondwana Research	25（1）	103-125
268	KG-95	CAOB	CAOB	72.0420	41.3910	Granodiorite		467	467	4.09	22.45	0.1102	0.512244		-0.44	-7.69	-2.50	-2.53	-2.53	1.40	1.33	1.40	1.17		Kröner A et al.	2014	Gondwana Research	25（1）	103-125
269	KG-83	CAOB	CAOB	71.3973	42.6748	Granodiorite		456	456	9.96	61.94	0.0972	0.512057		-0.51	-11.33	-5.50	-5.54	-5.54	1.64	1.43	1.64	1.28		Kröner A et al.	2014	Gondwana Research	25（1）	103-125
270	KG-63	CAOB	CAOB	75.2816	41.9615	Granodiorite		451	451	4.86	26.38	0.1113	0.512136		-0.43	-9.79	-4.90	-4.88	-4.88	1.58	1.51	1.58	1.34		Kröner A et al.	2014	Gondwana Research	25（1）	103-125
271	KG-61	CAOB	CAOB	75.5333	41.9170	Granodiorite		490	490	2.39	12.86	0.1125	0.512300		-0.43	-6.59	-1.30	-1.33	-1.33	1.32	1.28	1.32	1.12		Kröner A et al.	2014	Gondwana Research	25（1）	103-125
272	KG-22	CAOB	CAOB	76.8250	41.5590	Granodiorite		764	600	12.84	59.89	0.1296	0.512195		-0.34	-8.64	-2.10	-3.50	-3.50	1.59	1.73	1.59	1.53		Kröner A et al.	2014	Gondwana Research	25（1）	103-125
273	KG-128	CAOB	CAOB	76.1470	42.1320	Diorite		483	483	4.11	21.71	0.1143	0.512255		-0.42	-7.47	-2.40	-2.39	-2.39	1.41	1.37	1.41	1.2		Kröner A et al.	2014	Gondwana Research	25（1）	103-125
274	KG-119	CAOB	CAOB	79.3900	42.3970	Granodiorite		746	600	11.20	58.21	0.1164	0.512300		-0.41	-6.59	1.10	-0.45	-0.45	1.34	1.33	1.34	1.16		Kröner A et al.	2014	Gondwana Research	25（1）	103-125
275	KG-116	CAOB	CAOB	75.6814	42.6928	Granodiorite		466	466	2.23	13.34	0.1009	0.511993		-0.49	-12.58	-6.90	-6.89	-6.89	1.76	1.56	1.76	1.41		Kröner A et al.	2014	Gondwana Research	25（1）	103-125
276	KG-102	CAOB	CAOB	71.3020	41.0180	Granodiorite		420	420	6.19	30.21	0.1239	0.512378		-0.37	-5.07	-1.20	-1.17	-1.17	1.25	1.31	1.25	1.13		Kröner A et al.	2014	Gondwana Research	25（1）	103-125
277	GCS-06	NCC	NNCC	121.8500	42.3000	Andesite		138	150	4.86	25.52	0.1150	0.512105		-0.42	-10.40	-9.00	-8.83	-8.83	1.66	1.61	1.66			Zhang HF et al.	2003	Geochimica Et Cosmochimica Acta	67（22）	4373-4387
278	GCS-05	NCC	NNCC	121.8500	42.3000	Andesite		138	150	4.38	24.38	0.1086	0.512113		-0.45	-10.24	-8.73	-8.55	-8.55	1.63	1.50	1.63			Zhang HF et al.	2003	Geochimica Et Cosmochimica Acta	67（22）	4373-4387
279	GCS-04	NCC	NNCC	121.8500	42.3000	Andesite		138	150	4.60	25.59	0.1088	0.512115		-0.45	-10.20	-8.69	-8.52	-8.52	1.63	1.50	1.63			Zhang HF et al.	2003	Geochimica Et Cosmochimica Acta	67（22）	4373-4387
280	GCS-03	NCC	NNCC	121.8500	42.3000	Andesite		138	150	4.90	25.52	0.1161	0.512118		-0.41	-10.14	-8.76	-8.60	-8.60	1.64	1.61	1.64			Zhang HF et al.	2003	Geochimica Et Cosmochimica Acta	67（22）	4373-4387
281	GCS-02	NCC	NNCC	121.8500	42.3000	Andesite		138	150	4.66	25.29	0.1115	0.512132		-0.43	-9.87	-8.41	-8.24	-8.24	1.61	1.52	1.61			Zhang HF et al.	2003	Geochimica Et Cosmochimica Acta	67（22）	4373-4387
282	GCS-01	NCC	NNCC	121.8500	42.3000	Andesite		138	150	4.59	24.91	0.1114	0.512128		-0.43	-9.95	-8.49	-8.32	-8.32	1.61	1.52	1.61			Zhang HF et al.	2003	Geochimica Et Cosmochimica Acta	67（22）	4373-4387
283	SED04	CAOB	Junggar	89.0000	46.8000	Rhyolite		389	389	7.21	33.60	0.1297	0.512764		-0.34	2.46	6.00	5.78	5.78	0.66	0.70	0.66			Liu X et al.	2014	Lithos	184-187	27-41
284	SED02	CAOB	Junggar	89.0000	46.8000	Rhyolite		389	389	4.58	22.10	0.1257	0.512743		-0.36	2.05	5.80	5.57	5.57	0.68	0.71	0.68			Liu X et al.	2014	Lithos	184-187	27-41
285	SEAN01	CAOB	Junggar	89.0000	46.8000	Adakite		389	389	4.00	19.20	0.1263	0.512815		-0.36	3.45	7.20	6.95	6.95	0.57	0.58	0.57			Liu X et al.	2014	Lithos	184-187	27-41
286	D1T12	CAOB	Junggar	89.0000	46.8000	Adakite		389	389	4.36	21.30	0.1237	0.512723		-0.37	1.66	5.50	5.28	5.28	0.70	0.72	0.70			Liu X et al.	2014	Lithos	184-187	27-41
287	D1T09	CAOB	Junggar	89.0000	46.8000	Adakite		389	389	4.03	20.10	0.1215	0.512736		-0.38	1.91	5.90	5.64	5.64	0.67	0.69	0.67			Liu X et al.	2014	Lithos	184-187	27-41
288	D1T02	CAOB	Junggar	89.0000	46.8000	Adakite		389	389	4.18	19.50	0.1298	0.512771		-0.34	2.59	6.10	5.92	5.92	0.65	0.69	0.65			Liu X et al.	2014	Lithos	184-187	27-41
289	NT-20/36	CAOB	Southern Mongolia	101.0850	43.1000	Andesite		318	318	4.16	18.88	0.1319	0.512759		-0.33	2.36	4.99	4.99	4.99	0.67	0.73	0.67			Kozlovsky AM et al.	2006	Petrology	14（4）	337-360
290	NT-20/21	CAOB	Southern Mongolia	101.0850	43.1000	Granite		318	318	2.22	13.16	0.1009	0.512678		-0.49	0.78	4.68	4.67	4.67	0.70	0.64	0.70			Kozlovsky AM et al.	2006	Petrology	14（4）	337-360
291	M99-24	CAOB	Transbaikalia and Mongolia	105.1048	46.9938	Aplite		120	150	0.67	3.30	0.1233	0.512540		-0.37	-1.91	-0.80	-0.51	-0.51	0.98	1.03	0.98	1.03	0.98	Jahn BM et al.	2004	Journal of Asian Earth Sciences	23（5）	629-653
292	Lzk1-285-1	CAOB	NE China	125.8742	50.2542	Granite	Duobaoshan	244	244	3.43	20.80	0.1038	0.512736		-0.47	1.91	4.95	4.80	4.80	0.62	0.57	0.62	0.55	0.61	Zhao C et al.	2019	Lithos	336-337	202-220

293	14DB18-1-1	CAOB	NE China	125.7875	50.2492	Granite	Duobaoshan	223	223	4.54	28.69	0.0997	0.512710		-0.49	1.40	4.29	4.16	4.16	0.66	0.59	0.66	0.57	0.65	Zhao C et al.	2019	Lithos	336-337	202-220
294	ZF8	CAOB	Junggar	91.8047	44.6011	Rhyolite	Taheir area	398	398	7.58	31.99	0.1432	0.512682		-0.27	0.86	3.58	3.57	3.57	0.85	1.01	0.85	1.01		Xu XW et al.	2013	Gondwana Research	24（2）	578-600
295	ZF5	CAOB	Junggar	91.7970	44.6127	Andesite	Taheir area	390	390	4.70	17.63	0.1612	0.512927		-0.18	5.64	7.42	7.40	7.40	0.53	0.65	0.53	0.65		Xu XW et al.	2013	Gondwana Research	24（2）	578-600
296	ZF10-1	CAOB	Junggar	91.7993	44.6021	Granite	Taheir area	455	455	6.32	32.80	0.1165	0.512689		-0.41	0.99	5.67	5.65	5.65	0.73	0.72	0.73	0.72		Xu XW et al.	2013	Gondwana Research	24（2）	578-600
297	TH9	CAOB	Junggar	91.7947	44.5995	Diorite	Taheir area	432	432	3.21	12.57	0.1267	0.512817		-0.36	3.49	7.37	7.35	7.35	0.57	0.58	0.57	0.58		Xu XW et al.	2013	Gondwana Research	24（2）	578-600
298	TH8	CAOB	Junggar	91.8001	44.6014	Granite	Taheir area	432	432	4.96	24.22	0.1240	0.512671		-0.37	0.64	4.66	4.65	4.65	0.79	0.81	0.79	0.81		Xu XW et al.	2013	Gondwana Research	24（2）	578-600
299	TH6	CAOB	Junggar	91.7984	44.6020	Granite	Taheir area	443	443	5.33	26.17	0.1156	0.512664		-0.41	0.51	5.10	5.09	5.09	0.76	0.76	0.76	0.76		Xu XW et al.	2013	Gondwana Research	24（2）	578-600
300	TH5	CAOB	Junggar	91.7904	44.6073	Granite	Taheir area	390	390	3.12	11.16	0.1439	0.512818		-0.27	3.51	6.53	6.14	6.14	0.63	0.73	0.63	0.73		Xu XW et al.	2013	Gondwana Research	24（2）	578-600
301	TH1	CAOB	Junggar	91.7746	44.6096	Diorite	Taheir area	432	432	1.95	9.83	0.1199	0.512747		-0.39	2.13	6.36	6.36	6.36	0.65	0.66	0.65	0.66		Xu XW et al.	2013	Gondwana Research	24（2）	578-600
302	TH05-3	CAOB	Junggar	91.7963	44.6015	Dacite	Taheir area	453	453	5.48	26.99	0.1230	0.512672		-0.37	0.66	4.93	4.92	4.92	0.79	0.80	0.79	0.80		Xu XW et al.	2013	Gondwana Research	24（2）	578-600
303	TH05-2	CAOB	Junggar	91.7963	44.6015	Rhyolite	Taheir area	454	454	5.37	27.84	0.1167	0.512668		-0.41	0.59	5.23	5.22	5.22	0.76	0.76	0.76	0.76		Xu XW et al.	2013	Gondwana Research	24（2）	578-600
304	03T-96	CAOB	Tianhsan	81.1059	43.2388	Granite		348	348	3.49	19.40	0.1088	0.512329		-0.45	-6.03	-2.12	-2.12	-2.12	1.27	1.19	1.27		1.19	Xu X et al.	2006	Northwestern Geology	39（1）	50-75
305	03T-57	CAOB	Tianhsan	84.4522	43.7061	Granite		287	287	1.63	5.20	0.1896	0.512514		-0.04	-2.42	-2.16	-2.16	-2.16	1.22	3.98	1.22		1.25	Xu X et al.	2006	Northwestern Geology	39（1）	50-75
306	02TB-19	CAOB	Tianhsan	83.2652	42.2983	Granite		425	425	3.21	14.10	0.1578	0.512387		-0.20	-4.90	-2.81	-2.79	-2.79	1.39	2.07	1.39		1.41	Xu X et al.	2006	Northwestern Geology	39（1）	50-75
307	02TB-18	CAOB	Tianhsan	83.2617	42.3002	Diorite		426	426	5.89	25.90	0.1375	0.512394		-0.30	-4.76	-1.54	-1.54	-1.54	1.29	1.51	1.29		1.51	Xu X et al.	2006	Northwestern Geology	39（1）	50-75
308	YB1-3-3	CAOB	NE China	129.7000	43.5000	Rhyolite		227	227	11.30	54.80	0.1245	0.512728		-0.37	1.76	3.60	3.84	3.84	0.69	0.72	0.69	0.72	0.68	Xu WL et al.	2009	Journal of Asian Earth Sciences	34(3)	392-402
309	YB1-3-2	CAOB	NE China	129.7000	43.5000	Rhyolite		227	227	9.09	44.60	0.1232	0.512731		-0.37	1.81	3.70	3.94	3.94	0.68	0.71	0.68	0.71	0.67	Xu WL et al.	2009	Journal of Asian Earth Sciences	34(3)	392-402
310	YB1-3-1	CAOB	NE China	129.7000	43.5000	Rhyolite		227	227	3.88	17.90	0.1311	0.512728		-0.33	1.76	3.40	3.65	3.65	0.70	0.78	0.70	0.78	0.69	Xu WL et al.	2009	Journal of Asian Earth Sciences	34(3)	392-402
311	HS9-1	CAOB	NE China	131.1250	44.3000	Dacite		227	227	7.04	32.20	0.1322	0.512751		-0.33	2.20	3.90	4.07	4.07	0.67	0.75	0.67	0.75	0.65	Xu WL et al.	2009	Journal of Asian Earth Sciences	34(3)	392-402
312	HS15-1	CAOB	NE China	131.1250	44.3000	Dacite		227	227	6.51	30.30	0.1300	0.512674		-0.34	0.70	2.50	2.63	2.63	0.79	0.87	0.79	0.87	0.77	Xu WL et al.	2009	Journal of Asian Earth Sciences	34(3)	392-402
313	HS14-3	CAOB	NE China	131.1250	44.3000	Rhyolite		227	227	6.22	28.80	0.1307	0.512643		-0.34	0.10	1.90	2.01	2.01	0.84	0.93	0.84	0.93	0.82	Xu WL et al.	2009	Journal of Asian Earth Sciences	34(3)	392-402
314	HS14-1	CAOB	NE China	131.1250	44.3000	Rhyolite		227	227	2.29	12.40	0.1119	0.512676		-0.43	0.74	3.00	3.19	3.19	0.74	0.71	0.74	0.71	0.75	Xu WL et al.	2009	Journal of Asian Earth Sciences	34(3)	392-402
315	HS13-1	CAOB	NE China	131.1250	44.3000	Dacite		227	227	6.58	29.40	0.1356	0.512663		-0.31	0.49	2.20	2.25	2.25	0.82	0.95	0.82	0.95	0.79	Xu WL et al.	2009	Journal of Asian Earth Sciences	34(3)	392-402
316	DD66	CAOB	Tianhsan	85.0000	45.0000	Rhyolite		329	329	2.94	14.50	0.1221	0.512600		-0.38	-0.74	2.60	2.39	2.39	0.89	0.92	0.89			Ge SS et al.	2015	Lithos	228-229	62-77
317	DD58	CAOB	Tianhsan	85.0000	45.0000	Rhyolite		329	329	2.80	14.40	0.1173	0.512600		-0.40	-0.74	3.40	2.59	2.59	0.87	0.87	0.87			Ge SS et al.	2015	Lithos	228-229	62-77
318	BZ28	CAOB	Tianhsan	85.0000	45.0000	Rhyolite		329	329	2.88	15.10	0.1156	0.512600		-0.41	-0.74	3.20	2.66	2.66	0.87	0.85	0.87			Ge SS et al.	2015	Lithos	228-229	62-77
319	BZ02	CAOB	Tianhsan	85.0000	45.0000	Andesite		329	329	6.13	28.00	0.1324	0.512700		-0.33	1.21	3.50	3.91	3.91	0.77	0.84	0.77			Ge SS et al.	2015	Lithos	228-229	62-77
320	D1336	CAOB	NE China	120.1275	44.0486	Rhyolite		173	173	11.20	68.20	0.1042	0.512430		-0.47	-4.06	-2.10	-2.02	-2.02	1.12	1.00	1.12	1.00		Gong MY et al.	2018	Lithos	308-309	412-427
321	D1335	CAOB	NE China	120.1308	44.0442	Rhyolite		173	173	10.70	55.20	0.1230	0.512430		-0.37	-4.06	-2.90	-2.43	-2.43	1.15	1.21	1.15	1.21		Gong MY et al.	2018	Lithos	308-309	412-427
322	D1332	CAOB	NE China	120.9414	46.3625	Rhyolite		106	150	6.09	31.80	0.1215	0.512700		-0.38	1.21	2.50	2.65	2.65	0.72	0.74	0.72	0.74		Gong MY et al.	2018	Lithos	308-309	412-427
323	D1310	CAOB	NE China	121.3428	46.2853	Dacite		106	150	6.72	32.40	0.1316	0.512620		-0.33	-0.35	0.60	0.90	0.90	0.87	0.98	0.87	0.98		Gong MY et al.	2018	Lithos	308-309	412-427
324	A-509	CAOB	Vitim alkaline province	111.5833	54.2000	arbonate, Nepheline syeni	Amalat	300	300	4.49	24.60	0.1105	0.512557		-0.44	-1.58	1.72	1.72	1.72	0.92	0.88	0.92	0.88	0.92	Doroshkevich AG et al.	2012	Lithos	152	157-172
325	725/5	CAOB	Vitim alkaline province	111.8333	54.1333	arbonate, Nepheline syeni	Verkhne-Burulzayski	300	300	4.31	25.60	0.1017	0.512531		-0.48	-2.09	1.55	1.55	1.55	0.94	0.84	0.94	0.84	0.94	Doroshkevich AG et al.	2012	Lithos	152	157-172
326	723/4	CAOB	Vitim alkaline province	111.8333	54.1333	Nepheline syenite	Verkhne-Burulzayski	300	300	6.13	29.70	0.1247	0.512585		-0.37	-1.03	1.72	1.72	1.72	0.92	0.97	0.92	0.97	0.92	Doroshkevich AG et al.	2012	Lithos	152	157-172
327	44-9a	CAOB	Vitim alkaline province	111.8667	54.1333	Carbonate, urtite	Mukhalski	300	300	4.75	20.50	0.1403	0.512545		-0.29	-1.81	0.34	0.34	0.34	1.03	1.26	1.03	1.26	1.03	Doroshkevich AG et al.	2012	Lithos	152	157-172
328	22-MK	CAOB	Vitim alkaline province	111.8667	54.1333	Apatite, urtite	Mukhalski	300	300	103.00	585.00	0.1058	0.512496		-0.46	-2.77	0.71	0.71	0.71	1.00	0.92	1.00	0.92	1.00	Doroshkevich AG et al.	2012	Lithos	152	157-172
329	178B	CAOB	Vitim alkaline province	111.5000	54.0833	arbonate, Nepheline syeni	Snezhny	480	480	25.80	150.00	0.0998	0.512237		-0.49	-7.82	-1.89	-1.89	-1.89	1.36	1.22	1.36	1.22	1.36	Doroshkevich AG et al.	2012	Lithos	152	157-172
330	126-24	CAOB	Vitim alkaline province	111.8667	54.1333	Ijolite	Mukhalski	300	300	2.19	9.36	0.1415	0.512642		-0.28	0.08	2.19	2.19	2.19	0.88	1.07	0.88	1.07	0.88	Doroshkevich AG et al.	2012	Lithos	152	157-172
331	59-7	CAOB	Vitim alkaline province	111.8667	54.1333	Nepheline syenite	Mukhalski	300	300	0.79	4.47	0.1073	0.512491		-0.45	-2.87	0.55	0.55	0.55	1.02	0.94	1.02	0.94	1.02	Doroshkevich AG et al.	2012	Lithos	152	157-172
332	8099	CAOB	Vitim alkaline province	111.5000	54.0833	Apatite, Nepheline syenite	Snezhny	480	480	357.00	2202.00	0.0981	0.512260		-0.50	-7.37	-1.33	-1.33	-1.33	1.32	1.17	1.32	1.17	1.32	Doroshkevich AG et al.	2012	Lithos	152	157-172
333	7931	CAOB	Vitim alkaline province	111.8500	54.1167	Nepheline syenite	Sayzhenski	500	500	2.27	14.70	0.0935	0.512495		-0.52	-2.79	3.79	3.79	3.79	0.92	0.83	0.92	0.83	0.92	Doroshkevich AG et al.	2012	Lithos	152	157-172
334	7922	CAOB	Vitim alkaline province	111.8500	54.1167	Pyroxenite	Sayzhenski	500	500	8.54	37.50	0.1375	0.512643		-0.30	0.10	3.87	3.87	3.87	0.91	1.01	0.91	1.01	0.91	Doroshkevich AG et al.	2012	Lithos	152	157-172
335	7892	CAOB	Vitim alkaline province	111.8500	54.1167	Ijolite	Sayzhenski	500	500	4.77	23.30	0.1237	0.512647		-0.37	0.18	4.83	4.83	4.83	0.83	0.85	0.83	0.85	0.83	Doroshkevich AG et al.	2012	Lithos	152	157-172
336	7871	CAOB	Vitim alkaline province	111.8500	54.1167	Pyroxenite	Sayzhenski	500	500	8.77	41.00	0.1293	0.512461		-0.34	-3.45	0.85	0.85	0.85	1.16	1.24	1.16	1.24	1.16	Doroshkevich AG et al.	2012	Lithos	152	157-172
337	509	CAOB	Vitim alkaline province	111.5833	54.2000	Ijolite	Amalat	300	300	7.32	42.80	0.1035	0.512561		-0.47	-1.50	2.07	2.07	2.07	0.89	0.82	0.89	0.82	0.89	Doroshkevich AG et al.	2012	Lithos	152	157-172
338	506	CAOB	Vitim alkaline province	111.5833	54.2000	Alkaline syenite	Amalat	300	300	2.66	14.70	0.1095	0.512573		-0.44	-1.27	2.07	2.07	2.07	0.89	0.84	0.89	0.84	0.89	Doroshkevich AG et al.	2012	Lithos	152	157-172
339	369	CAOB	Vitim alkaline province	111.8000	54.0833	Ijolite	Nizhne-Burulzayski	500	500	3.13	12.00	0.1577	0.512658		-0.20	0.39	2.88	2.88	2.88	0.99	1.34	0.99	1.34	0.99	Doroshkevich AG et al.	2012	Lithos	152	157-172
340	367	CAOB	Vitim alkaline province	111.8000	54.0833	Pyroxenite	Nizhne-Burulzayski	500	500	3.34	13.60	0.1488	0.512642		-0.24	0.08	3.13	3.13	3.13	0.97	1.19	0.97	1.19	0.97	Doroshkevich AG et al.	2012	Lithos	152	157-172
341	178	CAOB	Vitim alkaline province	111.5000	54.0833	Nepheline syenite	Snezhny	480	480	7.80	45.90	0.1027	0.512244		-0.48	-7.69	-1.93	-1.93	-1.93	1.37	1.24	1.37	1.24	1.37	Doroshkevich AG et al.	2012	Lithos	152	157-172
342	175	CAOB	Vitim alkaline province	111.8500	54.1167	Ijolite	Sayzhenski	500	500	2.37	13.80	0.1040	0.512343		-0.47	-5.75	0.16	0.16	0.16	1.21	1.12	1.21	1.12	1.21	Doroshkevich AG et al.	2012	Lithos	152	157-172
343	T5-140a	CAOB	Kyrgyz North Tianshan	72.6814	42.5914	Granite	Talas area	1131	600	5.92	44.46	0.0805	0.511456	9	-0.59	-23.06	-6.30	-14.16	-14.16	2.46	1.93	2.46			Kröner A et al.	2013	Gondwana Research	23(1)	272-195
344	KG 16	CAOB	Kyrgyz North Tianshan	75.6814	42.0581	Granite	Kochkorka	441	441	4.65	27.07	0.1038	0.512199	8	-0.47	-8.56	-3.30	-3.34	-3.34	1.45	1.32	1.45			Kröner A et al.	2013	Gondwana Research	23(1)	272-195
345	KG 15	CAOB	Kyrgyz North Tianshan	75.8758	42.2969	Diorite	Kochkorka	451	451	6.64	32.14	0.1248	0.512224	11	-0.37	-8.08	-3.90	-3.94	-3.94	1.50	1.58	1.50			Kröner A et al.	2013	Gondwana Research	23(1)	272-195
346	KG 14	CAOB	Kyrgyz North Tianshan	75.8819	42.3028	Granite gneiss	Kochkorka	1153	600	10.93	70.58	0.0936	0.511435	11	-0.52	-23.47	-8.30	-15.57	-15.57	2.57	2.17	2.57			Kröner A et al.	2013	Gondwana Research	23(1)	272-195
347	KG 13	CAOB	Kyrgyz North Tianshan	75.8819	42.3028	Diorite	Kochkorka	461	461	5.72	29.56	0.1169	0.512177	10	-0.41	-8.99	-4.30	-4.30	-4.30	1.54	1.53	1.54			Kröner A et al.	2013	Gondwana Research	23(1)	272-195
348	SD-1	CAOB	Northern margin of NCC	119.0303	43.5142	Granite porphyry	Yangchang	132	150			0.0951	0.512364		-0.52	-5.34	-3.63	-3.40	-3.40	1.22	1.01	1.22	1.01	1.22	Zeng QD et al.	2014	Geological Journal	49(1)	1-14
349	2-32	CAOB	Northern margin of NCC	119.0303	43.5142	Monzogranite	Yangchang	138	150			0.1007	0.512461		-0.49	-3.45	-1.76	-1.62	-1.62	1.07	0.93	1.07	0.93	1.08	Zeng QD et al.	2014	Geological Journal	49(1)	1-14
350	2-31	CAOB	Northern margin of NCC	119.0303	43.5142	Monzogranite	Yangchang	138	150			0.1007	0.512476		-0.49	-3.16	-1.47	-1.32	-1.32	1.05	0.91	1.05	0.91	1.05	Zeng QD et al.	2014	Geological Journal	49(1)	1-14
351	8-7	CAOB	Northern margin of NCC	119.0303	43.5142	Monzogranite	Yangchang	138	150			0.1006	0.512460		-0.49	-3.47	-1.78	-1.63	-1.63	1.07	0.93	1.07	0.93	1.08	Zeng QD et al.	2014	Geological Journal	49(1)	1-14
352	6-7	CAOB	Northern margin of NCC	119.0303	43.5142	Monzogranite	Yangchang	138	150			0.1021	0.512452		-0.48	-3.63	-1.96	-1.82	-1.82	1.09	0.95	1.09	0.96	1.09	Zeng QD et al.	2014	Geological Journal	49(1)	1-14
353	5-8	CAOB	Northern margin of NCC	119.0303	43.5142	Monzogranite	Yangchang	138	150			0.0990	0.512454		-0.50	-3.59	-1.86	-1.72	-1.72	1.08	0.93	1.08	0.93	1.08	Zeng QD et al.	2014	Geological Journal	49(1)	1-14
354	4-23	CAOB	Northern margin of NCC	119.0303	43.5142	Monzogranite	Yangchang	138	150			0.0998	0.512447		-0.49	-3.73	-2.02	-1.87	-1.87	1.09	0.94	1.09	0.94	1.10	Zeng QD et al.	2014	Geological Journal	49(1)	1-14
355	3-5	CAOB	Northern margin of NCC	119.0303	43.5142	Monzogranite	Yangchang	138	150			0.0999	0.512457		-0.49	-3.53	-1.82	-1.68	-1.68	1.08	0.93	1.08	0.93	1.08	Zeng QD et al.	2014	Geological Journal	49(1)	1-14
356	3-4	CAOB	Northern margin of NCC	119.0303	43.5142	Monzogranite	Yangchang	138	150			0.0964	0.512457		-0.51	-3.53	-1.76	-1.61	-1.61	1.07	0.90	1.07	0.90	1.08	Zeng QD et al.	2014	Geological Journal	49(1)	1-14
357	3-3	CAOB	Northern margin of NCC	119.0303	43.5142	Monzogranite	Yangchang	138	150			0.1010	0.512453		-0.49	-3.61	-1.92	-1.78	-1.78	1.08	0.94	1.08	0.95	1.09	Zeng QD et al.	2014	Geological Journal	49(1)	1-14
358	AJW-06-476 dup	CAOB	Southern Mongolia, Oyu Tolgo	106.8450	43.0500	granodiorite porphyry	Oyu Tolgoi	366	362	3.16	15.80	0.1209	0.512635	0	-0.39	-0.06	0.10	3.44	3.44	0.83	0.85	0.83	0.85	0.82	Wainwright et al.	2011	Gondwana Research	19(3)	764-787
359	AJW-04-272	CAOB	Southern Mongolia, Oyu Tolgo	106.8450	43.0500	Augite basalt	Oyu Tolgoi	390	362	4.25	13.80	0.1862	0.512841	0	-0.05	3.96	4.12	4.44	4.44	0.75	1.71	0.75	1.71	0.48	Wainwright et al.	2011	Gondwana Research	19(3)	764-787
360	AJW-03-204 dup	CAOB	Southern Mongolia, Oyu Tolgo	106.8450	43.0500	Augite basalt	Oyu Tolgoi	390	362	3.00	9.10	0.1993	0.512948	0	0.01	6.05	6.20	5.93	5.93	0.63	2.13	0.63	2.13	0.30	Wainwright et al.	2011	Gondwana Research	19(3)	764-787
361	AJW-03-204	CAOB	Southern Mongolia, Oyu Tolgo	106.8450	43.0500	Augite basalt	Oyu Tolgoi	390	362	3.00	9.10	0.1993	0.512949	0	0.01	6.07	6.22	5.95	5.95	0.63	2.12	0.63	2.12	0.30	Wainwright et al.	2011	Gondwana Research	19(3)	764-787
362	AJW-03-183 dup	CAOB	Southern Mongolia, Oyu Tolgo	106.8450	43.0500	Hb–Bi andesite	Oyu Tolgoi	345	362	4.40	20.50	0.1298	0.512805	0	-0.34	3.26	3.41	6.35	6.35	0.59	0.63	0.59	0.63	0.55	Wainwright et al.	2011	Gondwana Research	19(3)	764-787
363	AJW-03-116	CAOB	Southern Mongolia, Oyu Tolgo	106.8450	43.0500	Granite	Oyu Tolgoi	321	362	4.70	23.90	0.1189	0.512811	0	-0.40	3.37	3.53	6.97	6.97	0.54	0.55	0.54	0.55	0.54	Wainwright et al.	2011	Gondwana Research	19(3)	764-787
364	AJW-03-091 dup	CAOB	Southern Mongolia, Oyu Tolgo	106.8450	43.0500	LVS-dacite	Oyu Tolgoi	347	362	5.00	24.50	0.1234	0.512815	0	-0.37	3.45	3.61	6.84	6.84	0.55	0.57	0.55	0.57	0.53	Wainwright et al.	2011	Gondwana Research	19(3)	764-787
365	AJW-03-074	CAOB	Southern Mongolia, Oyu Tolgo	106.8450	43.0500	Granodiorite	Oyu Tolgoi	350	362	3.10	15.90	0.1179	0.512755	0	-0.40	2.28	2.44	5.92	5.92	0.63	0.63	0.63	0.63	0.63	Wainwright et al.	2011	Gondwana Research	19(3)	764-787
366	AJW-03-068 dup	CAOB	Southern Mongolia, Oyu Tolgo	106.8450	43.0500	Qtz monzodiorite	Oyu Tolgoi	372	362	5.30	23.70	0.1352	0.512821	0	-0.31	3.57	3.73	6.41	6.41	0.59	0.64	0.59	0.64	0.52	Wainwright et al.	2011	Gondwana Research	19(3)	764-787
367	XA-6	CAOB	Kazakhstan	85.7150	46.6150	Granite		431	431	3.19	17.99	0.1080	0.512731		-0.45	1.81	6.71	6.69	6.69	0.62	0.60	0.62			Shen P et al.	2015	Lithos	224	61-77
368	XA-5	CAOB	Kazakhstan	85.7150	46.6150	Rhyolite		431	431	2.97	13.72	0.1318	0.512733		-0.33	1.85	5.43	5.42	5.42	0.73	0.78	0.73			Shen P et al.	2015	Lithos	224	61-77
369	XA-2	CAOB	Kazakhstan	85.7150	46.6150	Rhyolite		431	431	1.98	10.93	0.1103	0.512654		-0.44	0.31	5.07	5.06	5.06	0.76	0.73	0.76			Shen P et al.	2015	Lithos	224	61-77
370	XA-1	CAOB	Kazakhstan	85.7150	46.6150	Rhyolite		431	431	2.90	14.36	0.1230	0.512718		-0.37	1.56	5.62	5.61	5.61	0.71	0.73	0.71			Shen P et al.	2015	Lithos	224	61-77
371	S24-C1	CAOB	Kazakhstan	85.7150	46.6150	Rhyolite		431	431	1.62	8.26	0.1195	0.512648		-0.39	0.20	4.45	4.44	4.44	0.81	0.81	0.81			Shen P et al.	2015	Lithos	224	61-77
372	S24-5	CAOB	Kazakhstan	85.7150	46.6150	Andesite		431	431	3.42	16.93	0.1230	0.512686		-0.37	0.94	5.00	4.99	4.99	0.76	0.78	0.76			Shen P et al.	2015	Lithos	224	61-77
373	S12+51	CAOB	Kazakhstan	85.7150	46.6150	Andesite		431	431	4.83	23.01	0.1279	0.512704		-0.35	1.29	5.08	5.07	5.07	0.76	0.79	0.76			Shen P et al.	2015	Lithos	224	61-77
374	GE+360	CAOB	Kazakhstan	85.7150	46.6150	Granite		431	431	3.57	18.69	0.1163	0.512715		-0.41	1.50	5.93	5.92	5.92	0.69	0.68	0.69			Shen P et al.	2015	Lithos	224	61-77
375	GE+280	CAOB	Kazakhstan	85.7150	46.6150	Granite		431	431	3.68	18.99	0.1180	0.512735		-0.40	1.89	6.23	6.22	6.22	0.66	0.66	0.66			Shen P et al.	2015	Lithos	224	61-77
376	12BP9	CAOB	Kazakhstan	74.0000	52.0000	Dacite		501	501	2.24	11.45	0.1192	0.512698		-0.39	1.17	6.14	6.13	6.13	0.73	0.73	0.73			Shen P et al.	2015	Lithos	224	61-77
377	12BP19	CAOB	Kazakhstan	74.0000	52.0000	Andesite		501	501	3.71	15.47	0.1461	0.512771		-0.26	2.59	5.84	5.83	5.83	0.75	0.85	0.75			Shen P et al.	2015	Lithos	224	61-77
378	04XJ-86	CAOB	Northern margin of Tarim	78.7833	39.7667	Syenite		282	282			0.1000	0.512629		-0.49	-0.18	3.31	3.30	3.30	0.78	0.70	0.78	0.70	0.78	Sun LH et al.	2008	ournal of Jilin University(Earth Science Edition	38(1)	8-19
379	04XJ-82	CAOB	Northern margin of Tarim	78.7833	39.7667	Syenite		282	282			0.0929	0.512664		-0.53	0.51	4.25	4.24	4.24	0.70	0.61	0.70	0.61	0.70	Sun LH et al.	2008	ournal of Jilin University(Earth Science Edition	38(1)	8-19
380	04XJ-77	CAOB	Northern margin of Tarim	78.7833	39.7667	Syenite		282	282			0.1017	0.512652		-0.48	0.27	3.70	3.69	3.69	0.75	0.68	0.75	0.68	0.75	Sun LH et al.	2008	ournal of Jilin University(Earth Science Edition	38(1)	8-19
381	04XJ-73	CAOB	Northern margin of Tarim	78.7833	39.7667	Syenite		282	282			0.0987	0.512641		-0.50	0.06	3.59	3.59	3.59	0.75	0.68	0.75	0.68	0.76	Sun LH et al.	2008	ournal of Jilin University(Earth Science Edition	38(1)	8-19
382	W990	CAOB	Southwest Tianshan	80.7833	42.5000	Granitoid	Alasan	294	294	8.38	50.02	0.1014	0.512159		-0.48	-9.34	-5.77	-5.77	-5.77	1.53	1.34	1.53	1.34	1.53	Gou LL et al.	2012	Lithos	136-139	201-224
383	W983	CAOB	Southwest Tianshan	80.7833	42.5000	Granitoid	Alasan	294	294	8.09	47.15	0.1038	0.512305		-0.47	-6.50	-3.01	-3.01	-3.01	1.30	1.17	1.30	1.17	1.30	Gou LL et al.	2012	Lithos	136-139	201-224
384	W980	CAOB	Southwest Tianshan	80.7833	42.5000	Granitoid	Alasan	294	294	6.56	37.71	0.1053	0.512185		-0.46	-8.84	-5.41	-5.41	-5.41	1.50	1.36	1.50	1.36	1.50	Gou LL et al.	2012	Lithos	136-139	201-224
385	W917	CAOB	Southwest Tianshan	80.7833	42.5000	Garnet	Changawuzi	330	330	1.17	5.11	0.1390	0.512594		-0.29	-0.86	1.57	1.57	1.57	0.96	1.13	0.96	1.13	0.96	Gou LL et al.	2012	Lithos	136-139	201-224
386	W912	CAOB	Southwest Tianshan	80.7833	42.5000	Tonalite	Changawuzi	330	330	2.05	8.46	0.1467	0.512577		-0.25	-1.19	0.92	0.92	0.92	1.01	1.30	1.01	1.30	1.01	Gou LL et al.	2012	Lithos	136-139	201-224
387	W8106	CAOB	Southwest Tianshan	80.7833	42.5000	Granitoid	Alasan	294	294	8.96	52.75	0.1028	0.512168		-0.48	-9.17	-5.65	-5.65	-5.65	1.52	1.35	1.52	1.35	1.52	Gou LL et al.	2012	Lithos	136-139	201-224
388	W8092	CAOB	Southwest Tianshan	80.7833	42.5000	Granitoid	Alasan	294	294	3.04	16.76	0.1097	0.512163		-0.44	-9.27	-6.00	-6.00	-6.00	1.54	1.44	1.54	1.44	1.54	Gou LL et al.	2012	Lithos	136-139	201-224
389	W8085	CAOB	Southwest Tianshan	80.7833	42.5000	Granitoid	Alasan	294	294	1.82	11.03	0.0996	0.512148		-0.49	-9.56	-5.92	-5.92	-5.92	1.54	1.34	1.54	1.34	1.54	Gou LL et al.	2012	Lithos	136-139	201-224
390	W8039	CAOB	Southwest Tianshan	80.7833	42.5000	Tonalite	Changawuzi	330	330	1.98	8.88	0.1348	0.512546		-0.31	-1.79	0.81	0.81	0.81	1.02	1.17	1.02	1.17	1.02	Gou LL et al.	2012	Lithos	136-139	201-224

391	W8037	CAOB	Southwest Tianshan	80.7833	42.5000	Tonalite	Changawuzi	330	330	2.15	9.38	0.1384	0.512434		-0.30	-3.98	-1.52	-1.52	-1.52	1.21	1.45	1.21	1.45	1.21	Gou LL et al.	2012	Lithos	136-139	201-224
392	W8028	CAOB	Southwest Tianshan	80.7833	42.5000	Garnet	Changawuzi	330	330	0.80	3.61	0.1338	0.512508		-0.32	-2.54	0.11	0.11	0.11	1.08	1.22	1.08	1.22	1.08	Gou LL et al.	2012	Lithos	136-139	201-224
393	W8027	CAOB	Southwest Tianshan	80.7833	42.5000	Granitoid	Alasan	294	294	8.15	49.11	0.1005	0.512150		-0.49	-9.52	-5.91	-5.91	-5.91	1.54	1.34	1.54	1.34	1.54	Gou LL et al.	2012	Lithos	136-139	201-224
394	W8026	CAOB	Southwest Tianshan	80.7833	42.5000	Granitoid	Alasan	294	294	6.95	39.18	0.1073	0.512440		-0.45	-3.86	-0.51	-0.51	-0.51	1.10	1.02	1.10	1.02	1.10	Gou LL et al.	2012	Lithos	136-139	201-224
395	K2012-3	Tajikistan	Tajikistan	68.8031	38.9313	Granite		312	312	4.19	19.00	0.1333	0.512302		-0.32	-6.55	-3.00	-4.03	-4.03	1.14	1.60	1.40		1.14	Konopelko D et al.	2017	Gondwana Research	47	110-130
396	K2012-10	Tajikistan	Tajikistan	68.8789	38.6978	Plagioclase granite		315	315	2.63	11.00	0.1445	0.512610		-0.27	-0.55	-1.50	1.55	1.55	1.14	1.19	0.95		1.14	Konopelko D et al.	2017	Gondwana Research	47	110-130
397	2012-49	Tajikistan	Tajikistan	70.4264	40.7461	Rhyolite		305	305	3.72	19.10	0.1177	0.512259		-0.40	-7.39	-5.00	-4.32	-4.32	1.14	1.41	1.42		1.14	Konopelko D et al.	2017	Gondwana Research	47	110-130
398	2012-35	Tajikistan	Tajikistan	68.8789	38.6978	Granite		315	315	2.63	11.00	0.1445	0.512362		-0.27	-5.38	-2.10	-3.29	-3.29	1.14	1.73	1.34		1.14	Konopelko D et al.	2017	Gondwana Research	47	110-130
399	2012-33	Tajikistan	Tajikistan	69.5044	40.2704	Granodiorite		311	311	3.90	20.80	0.1134	0.512263		-0.42	-7.32	-4.20	-4.01	-4.01	1.14	1.35	1.40		1.14	Konopelko D et al.	2017	Gondwana Research	47	110-130
400	2012-25	Tajikistan	Tajikistan	68.8789	38.6978	Granite		315	315	2.63	11.00	0.1445	0.512663		-0.27	0.49	2.30	2.58	2.58	1.14	1.07	0.86		1.14	Konopelko D et al.	2017	Gondwana Research	47	110-130
401	GX-7	CAOB	Tianhsan	70.7153	39.4414	Granite	Tajikistan	267	267	0.94	4.90	0.1160	0.512104		-0.41	-10.42	-7.40	-7.67	-7.67	1.53	1.63	1.66	1.53		Konopelko D et al.	2017	Gondwana Research	47	110-130
402	GX-4	CAOB	Tianhsan	67.7208	39.3508	Granite	Tajikistan	288	288	4.59	21.20	0.1309	0.512237		-0.33	-7.82	-5.50	-5.40	-5.40	1.58	1.68	1.49	1.58		Konopelko D et al.	2017	Gondwana Research	47	110-130
403	2012-49	CAOB	Tianhsan	70.4261	40.7461	Granite	Tajikistan	305	305	4.19	19.00	0.1333	0.512259		-0.32	-7.39	-5.00	-4.93	-4.93	1.59	1.69	1.46	1.59		Konopelko D et al.	2017	Gondwana Research	47	110-130
404	2012-47	CAOB	Tianhsan	70.0625	40.6175	Granite	Tajikistan	290	290	5.75	33.10	0.1050	0.512228		-0.47	-8.00	-4.60	-4.61	-4.61	1.23	1.29	1.43	1.23		Konopelko D et al.	2017	Gondwana Research	47	110-130
405	2012-45	CAOB	Tianhsan	69.7842	40.4203	Granite	Tajikistan	286	286	6.68	34.30	0.1177	0.512291		-0.40	-6.77	-3.90	-3.89	-3.89	1.29	1.36	1.37	1.29		Konopelko D et al.	2017	Gondwana Research	47	110-130
406	2012-40	CAOB	Tianhsan	69.6597	40.4056	Granite	Tajikistan	290	290	6.63	36.30	0.1104	0.512271		-0.44	-7.16	-4.00	-3.97	-3.97	1.24	1.30	1.38	1.24		Konopelko D et al.	2017	Gondwana Research	47	110-130
407	2012-35	CAOB	Tianhsan	69.5825	40.6761	Granite	Tajikistan	315	315	3.90	20.80	0.1134	0.512362		-0.42	-5.38	-2.10	-2.03	-2.03	1.14	1.20	1.24	1.14		Konopelko D et al.	2017	Gondwana Research	47	110-130
408	2012-33	CAOB	Tianhsan	69.5042	40.2703	Granite	Tajikistan	311	311	3.72	19.10	0.1177	0.512263		-0.40	-7.32	-4.20	-4.18	-4.18	1.34	1.41	1.41	1.34		Konopelko D et al.	2017	Gondwana Research	47	110-130
409	2012-29	CAOB	Tianhsan	69.3942	40.2419	Granite	Tajikistan	425	425	3.66	18.30	0.1209	0.512199		-0.39	-8.56	-4.50	-4.45	-4.45	1.48	1.56	1.52	1.48		Konopelko D et al.	2017	Gondwana Research	47	110-130
410	2012-25	CAOB	Tianhsan	68.6189	38.7700	Granodiorite	Tajikistan	315	315	4.98	20.20	0.1490	0.512663		-0.24	0.49	2.30	2.40	2.40	1.06	1.15	0.88	1.06		Konopelko D et al.	2017	Gondwana Research	47	110-130
411	2012-10	CAOB	Tianhsan	68.8789	38.6978	Granite	Tajikistan	315	315	2.63	11.00	0.1445	0.512610		-0.27	-0.55	1.50	1.55	1.55	1.11	1.19	0.95	1.11		Konopelko D et al.	2017	Gondwana Research	47	110-130
412	2012-3	CAOB	Tianhsan	68.8031	38.9311	Granite	Tajikistan	312	312	3.23	18.50	0.1056	0.512302		-0.46	-6.55	-3.00	-2.93	-2.93	1.14	1.19	1.31	1.14		Konopelko D et al.	2017	Gondwana Research	47	110-130
413	FX20	CAOB	Northern margin of NCC	121.8233	41.7086	Leucogranites	Gangjia	153	153	0.70	3.99	0.1602	0.511838	17	-0.19	-15.61	-14.9	-14.9	-14.9	2.14	3.70	2.14	3.71	2.15	Zhang XH et al.	2008	Geological Magazine	145(3)	305-320
414	FX18	CAOB	Northern margin of NCC	121.8233	41.7086	Leucogranites	Gangjia	153	153	1.72	5.91	0.1766	0.511684	14	-0.10	-18.61	-18.2	-18.2	-18.2	2.39	5.93	2.39	5.95	2.42	Zhang XH et al.	2008	Geological Magazine	145(3)	305-320
415	FX11-8	CAOB	Northern margin of NCC	121.8233	41.7086	Leucogranites	Gangjia	153	153	1.21	3.56	0.2061	0.511648	13	0.05	-19.31	-19.5	-19.5	-19.5	2.30	27.58	2.30	27.93	2.52	Zhang XH et al.	2008	Geological Magazine	145(3)	305-320
416	FX11-4	CAOB	Northern margin of NCC	121.8233	41.7086	Leucogranites	Gangjia	153	153	2.30	8.22	0.1694	0.511659	18	-0.14	-19.10	-18.6	-18.6	-18.6	2.42	5.06	2.42	5.08	2.45	Zhang XH et al.	2008	Geological Magazine	145(3)	305-320
417	FX11-2	CAOB	Northern margin of NCC	121.8233	41.7086	Leucogranites	Gangjia	153	153	1.17	3.25	0.2166	0.511662	13	0.10	-19.04	-19.4	-19.4	-19.4	3.61	#####	3.61		2.52	Zhang XH et al.	2008	Geological Magazine	145(3)	305-320
418	FX11-1	CAOB	Northern margin of NCC	121.8233	41.7086	Leucogranites	Gangjia	153	153	0.17	0.57	0.1809	0.511623	15	-0.08	-19.80	-19.5	-19.5	-19.5	2.48	6.96	2.48	6.98	2.52	Zhang XH et al.	2008	Geological Magazine	145(3)	305-320
419	SM3-22	CAOB	SMXAB	116.7461	46.0272	Middle-fine grain granite	Shamai	115	150	16.62	39.89	0.2519	0.512594	7	0.28	-0.86	-1.68	-1.91	-1.91	1.11	-2.24	1.11		1.05	Hu P et al.	2006	Acta Petrologica Sinica	22(11)	2781-2790
420	SM3-1	CAOB	SMXAB	116.7461	46.0272	Middle-fine grain granite	Shamai	115	150	27.74	63.45	0.2644	0.513082	3	0.34	8.66	7.67	7.37	7.37	0.34	-0.21	0.34		0.29	Hu P et al.	2006	Acta Petrologica Sinica	22(11)	2781-2790
421	SM18	CAOB	SMXAB	116.7461	46.0272	Middle-fine grain granite	Shamai	115	150	13.51	35.15	0.2327	0.512772	12	0.18	2.61	2.09	1.92	1.92	0.79	-3.07	0.79		0.74	Hu P et al.	2006	Acta Petrologica Sinica	22(11)	2781-2790
422	SM10	CAOB	SMXAB	116.7461	46.0272	Porphyritic granite	Shamai	115	150	18.64	81.30	0.1388	0.512896	15	-0.29	5.03	5.89	6.14	6.14	0.44	0.52	0.44	0.52	0.43	Hu P et al.	2006	Acta Petrologica Sinica	22(11)	2781-2790
423	SM06	CAOB	SMXAB	116.7461	46.0272	Middle-fine grain granite	Shamai	115	150	14.04	30.37	0.2796	0.513100	3	0.42	9.01	7.80	7.43	7.43	0.33	-0.12	0.33		0.28	Hu P et al.	2006	Acta Petrologica Sinica	22(11)	2781-2790
424	48yQ-1	CAOB	Alashan	88.3597	46.5194	Granite	Chaergan	277	277	10.90	41.50	0.1588	0.512825	2	-0.19	3.65	5.00	4.99	4.99	0.64	0.90	0.64			Zhang CL et al.	2010	Geological Society of America Bulletin	122(11)	2020-2040
425	D2374	CAOB	Alashan	88.3597	46.5194	Granite	Chaergan	277	277	5.10	28.90	0.1067	0.512562	3	-0.46	-1.48	1.70	1.70	1.70	0.90	0.84	0.90			Zhang CL et al.	2010	Geological Society of America Bulletin	122(11)	2020-2040
426	LM-0201	CAOB	NE China	128.5667	47.3667	Granite	Luming	181	181	5.00	29.90	0.1011	0.512345		-0.49	-5.72	3.50	-3.51	-3.51	1.25	1.09	1.25		1.25	Hu XL et al.	2014	Journal of Asian Earth Sciences	90	88-100
427	LM-0181	CAOB	NE China	128.5667	47.3667	Granite	Luming	181	181	5.22	31.20	0.1014	0.512362		-0.48	-5.38	3.20	-3.19	-3.19	1.22	1.07	1.22		1.23	Hu XL et al.	2014	Journal of Asian Earth Sciences	90	88-100
428	LM-0101	CAOB	NE China	128.5667	47.3667	Granite	Luming	181	181	5.13	28.10	0.1106	0.512420		-0.44	-4.25	2.30	-2.27	-2.27	1.15	1.08	1.15		1.15	Hu XL et al.	2014	Journal of Asian Earth Sciences	90	88-100
429	HQ0799	CAOB	SM	105.4342	41.6839	Granite	Yilian	362	362				0.512550	0			0.98		0.98				0.43	0.96	Han BF et al.	2010	Acta Petrologica et Mineralogica	29(6)	741-749
430	HQ0798	CAOB	SM	105.4342	41.6839	Granite	Yilian	362	362				0.512598	0			1.83		1.83					0.88	Han BF et al.	2010	Acta Petrologica et Mineralogica	29(6)	741-749
431	HQ0794	CAOB	SM	105.7450	41.6497	Granite	Kuchuwula pluton	362	362				0.512565	0			0.90		0.90					0.93	Han BF et al.	2010	Acta Petrologica et Mineralogica	29(6)	741-749
432	HQ0793	CAOB	SM	105.7450	41.6497	Granite	Kuchuwula pluton	362	362				0.512565	0			0.90		0.90					0.93	Han BF et al.	2010	Acta Petrologica et Mineralogica	29(6)	741-749
433	HQ0781	CAOB	SM	105.7425	41.5353	Granite	Yinggete-Bagemaode pluton	362	362				0.512595	0			3.43		3.43					0.89	Han BF et al.	2010	Acta Petrologica et Mineralogica	29(6)	741-749
434	HQ0780	CAOB	SM	105.7425	41.5353	Granite	Yinggete-Bagemaode pluton	362	362				0.512549	0			2.55		2.55					0.96	Han BF et al.	2010	Acta Petrologica et Mineralogica	29(6)	741-749
435	HQ0779	CAOB	SM	105.7425	41.5353	Granite	Yinggete-Bagemaode pluton	362	362				0.512652	0			3.28		3.28					0.80	Han BF et al.	2010	Acta Petrologica et Mineralogica	29(6)	741-749
436	HQ0778	CAOB	SM	105.7425	41.5353	Granite	Yinggete-Bagemaode pluton	362	362				0.512692	0			3.14		3.14					0.73	Han BF et al.	2010	Acta Petrologica et Mineralogica	29(6)	741-749
437	BA11-119	CAOB	Beishan	96.8000	41.7000	Granodiorite	Bagelengtai	434	434	1.71	9.53	0.1089	0.512441		-0.45	-3.84	1.03	1.02	1.02	1.09	1.03	1.09			Zheng RG et al.	2016	Journal of Asian Earth Sciences	153	75-99
438	BA11-118	CAOB	Beishan	96.8000	41.7000	Granodiorite	Bagelengtai	434	434	1.19	4.50	0.1599	0.512563		-0.19	-1.46	0.58	0.58	0.58	1.12	1.66	1.12			Zheng RG et al.	2016	Journal of Asian Earth Sciences	153	75-99
439	BA11-115	CAOB	Beishan	96.8000	41.7000	Granodiorite	Bagelengtai	434	434	1.12	5.47	0.1234	0.512427		-0.37	-4.12	-0.04	-0.05	-0.05	1.18	1.22	1.18			Zheng RG et al.	2016	Journal of Asian Earth Sciences	153	75-99
440	0947-4	CAOB	Yili	84.0261	43.3322	Granite		304	304	4.65	21.50	0.1313	0.512738		-0.33	1.95	4.50	4.49	4.49	0.70	0.76	0.70	0.77	0.70	Wang XS et al.	2017	Ore Geology Reviews	92	144-160
441	FY-74	CAOB	Altai, unit 3	89.8417	47.0500	Bi granite	Koktokay	359	359				0.512439				-1.80		-1.80					1.80	Yuan C et al.	2007	Chemical Geology	242(1-2)	22-39
442	FY-68	CAOB	Altai, unit 3	89.8417	47.0500	Bi granite	Koktokay	359	359				0.512376				-1.90		-1.90					1.34	Yuan C et al.	2007	Chemical Geology	242(1-2)	22-39
443	FY-54	CAOB	Altai, unit 3	89.8667	47.1333	Two-Mica Granite	Koktokay	359	359				0.512323				-2.50		-2.50					1.39	Yuan C et al.	2007	Chemical Geology	242(1-2)	22-39
444	FY-50	CAOB	Altai, unit 3	89.8667	47.1333	Two-Mica Granite	Koktokay	359	359				0.512252				-4.00		-4.00					1.56	Yuan C et al.	2007	Chemical Geology	242(1-2)	22-39
445	ALT-8	CAOB	Altai, unit 4	86.5028	48.3153	Granodiorite	Ashile	318	318				0.512684				3.30		3.30					0.95	Yuan C et al.	2007	Chemical Geology	242(1-2)	22-39
446	ALT-18	CAOB	Altai, unit 4	86.5028	48.3153	Granodiorite	Ashile	318	318				0.512643				2.80		2.80					0.91	Yuan C et al.	2007	Chemical Geology	242(1-2)	22-39
447	ALT-128	CAOB	Altai, unit 4	86.9761	47.9861	Granodiorite	Halatas	267	267				0.512282				-4.40		-4.40					1.46	Yuan C et al.	2007	Chemical Geology	242(1-2)	22-39
448	ALT-126	CAOB	Altai, unit 4	86.9761	47.9861	Granodiorite	Halatas	267	267				0.512383				-2.70		-2.70					1.39	Yuan C et al.	2007	Chemical Geology	242(1-2)	22-39
449	ALT-12	CAOB	Altai, unit 4	86.5000	48.3333	Granodiorite	Ashile	318	318				0.512384				3.00		3.00					1.00	Yuan C et al.	2007	Chemical Geology	242(1-2)	22-39
450	ALT-12	CAOB	Altai, unit 4	86.5028	48.3153	Granodiorite	Ashile	318	318				0.512384				3.00		3.00					0.93	Yuan C et al.	2007	Chemical Geology	242(1-2)	22-39
451	B68	CAOB	West Junggar	83.9167	45.0333	Alkali feldspar granite	Burksdale	314	314	5.06	23.92	0.1280	0.512779		-0.35	2.75	5.50	5.50	5.50	0.62	0.66	0.62	0.66	0.62	He BC et al.	1994	Geotectonica et Metallogenia	(03)	219-228
452	B66	CAOB	West Junggar	83.9167	45.0333	Diabase porphyrite	Burksdale	329	329	0.28	6.77	0.1144	0.512779		-0.42	2.75	6.21	6.21	6.21	0.58	0.57	0.58	0.57	0.58	He BC et al.	1994	Geotectonica et Metallogenia	(03)	219-228
453	HY-N2	CAOB	Tianshan	94.0000	42.0000	Granodiorite	Hongyuan	329	329	2.78	13.20	0.1300	0.512521		-0.34	-2.28	1.86	0.52	0.52	1.04	1.14	1.04	0.97		Lu WJ et al.	2017	Lithos	294	97-111
454	HY-N19	CAOB	Tianshan	94.0000	42.0000	Granodiorite	Hongyuan	329	329	3.02	15.00	0.1200	0.512512		-0.39	-2.46	0.43	0.76	0.76	1.02	1.04	1.02	1.06		Lu WJ et al.	2017	Lithos	294	97-111
455	HY-N1	CAOB	Tianshan	94.0000	42.0000	Granodiorite	Hongyuan	329	329	2.80	13.34	0.1300	0.512595		-0.34	-0.84	0.38	1.96	1.96	0.93	1.01	0.93	1.11		Lu WJ et al.	2017	Lithos	294	97-111
456	CHI-7	CAOB	Western Transbaikalia	113.0000	55.0000	Syenite		310	310	11.90	73.43	0.0978	0.512071		-0.50	-11.06	-7.15	-7.15	-7.15	1.65	1.42	1.65	1.42	1.68	Izbrodin IA et al.	2017	Russian Geology and Geophysics	58（8）	903-921
457	CHI-505/14	CAOB	Western Transbaikalia	113.0000	55.0000	Syenite		310	310	10.20	61.10	0.1004	0.511996		-0.49	-12.52	-8.72	-8.71	-8.71	1.78	1.55	1.78	1.552	1.811	Izbrodin IA et al.	2017	Russian Geology and Geophysics	58（8）	903-921
458	CHI-16	CAOB	Western Transbaikalia	113.0000	55.0000	Syenite		310	310	7.80	46.30	0.1018	0.512176		-0.48	-9.01	-9.01	-5.26	-5.26	1.50	1.32	1.50	1.33	1.52	Izbrodin IA et al.	2017	Russian Geology and Geophysics	58（8）	903-921
459	Bi7	NCC	NCC	118.4494	41.2939	Alkali granite	Guangtoushan	220	220	18.00	144.00	0.0757	0.512014	9	-0.62	-12.17	-8.78	-8.78	-8.78	1.71	1.25	1.71	1.26	1.71	Han BF et al.	2004	Acta Petrologica Sinica	20（6）	1375-1388
460	Bi6	NCC	NCC	118.4494	41.2939	Alkali granite	Guangtoushan	220	220	32.90	271.00	0.0733	0.512019	6	-0.63	-12.07	-8.62	-8.61	-8.61	1.70	1.23	1.70	1.23	1.70	Han BF et al.	2004	Acta Petrologica Sinica	20（6）	1375-1388
461	Bi5	NCC	NCC	118.4494	41.2939	Alkali granite	Guangtoushan	220	220	12.90	89.60	0.0870	0.512031	9	-0.56	-11.84	-8.76	-8.76	-8.76	1.71	1.34	1.71	1.35	1.71	Han BF et al.	2004	Acta Petrologica Sinica	20（6）	1375-1388
462	Bi4	NCC	NCC	118.4494	41.2939	Alkali granite	Guangtoushan	220	220	16.20	85.80	0.1142	0.512012	13	-0.42	-12.21	-9.89	-9.90	-9.90	1.80	1.74	1.80	1.74	1.80	Han BF et al.	2004	Acta Petrologica Sinica	20（6）	1375-1388
463	Bi2	NCC	NCC	118.4494	41.2939	Alkali granite	Guangtoushan	220	220	18.50	103.00	0.1082	0.512054	9	-0.45	-11.39	-8.92	-8.91	-8.91	1.72	1.58	1.72	1.58	1.72	Han BF et al.	2004	Acta Petrologica Sinica	20（6）	1375-1388
464	Bi10	NCC	NCC	118.4494	41.2939	Alkali granite	Guangtoushan	220	220	10.30	70.60	0.0882	0.512038	5	-0.55	-11.70	-8.66	-8.66	-8.66	1.70	1.35	1.70	1.35	1.70	Han BF et al.	2004	Acta Petrologica Sinica	20（6）	1375-1388
465	380101-2	CAOB	Altai, unit 6	90.9039	46.0786	Monzogranite	Takshiken	286	286	7.76	40.38	0.1161	0.512805	6	-0.41	3.26	6.20	6.20	6.20	0.54	0.54	0.54	0.54	0.54	Tong Y et al.	2006	Acta Petrologica Sinica	22(5)	1267-1278
466	380105	CAOB	Altai, unit 6	90.9039	46.0786	Monzogranite	Takshiken	286	286	8.72	43.93	0.1200	0.512816	5	-0.39	3.47	6.30	6.27	6.27	0.54	0.54	0.54	0.54	0.54	Tong Y et al.	2006	Acta Petrologica Sinica	22(5)	1267-1278
467	13Un-74	CAOB	Tianshan	70.3842	41.6517	Granodiorite	Andagul	307	307	6.92	39.64	0.1061	0.512671		-0.46	0.64	4.30	4.19	4.19	0.73	0.68	0.73	0.68	0.73	Zu B et al.	2018	Ore Geology Reviews	101	54-73
468	13Un-73	CAOB	Tianshan	70.3842	41.6517	Granodiorite	Andagul	307	307	8.50	48.63	0.1062	0.512568		-0.46	-1.37	2.30	2.18	2.18	0.89	0.83	0.89	0.83	0.89	Zu B et al.	2018	Ore Geology Reviews	101	54-73
469	13Un-72	CAOB	Tianshan	70.3842	41.6517	Granodiorite	Andagul	307	307	8.81	49.48	0.1082	0.512658		-0.45	0.39	4.00	3.86	3.86	0.75	0.71	0.75	0.71	0.76	Zu B et al.	2018	Ore Geology Reviews	101	54-73
470	13Un-71	CAOB	Tianshan	70.3842	41.6517	Granodiorite	Andagul	307	307	8.72	49.11	0.1079	0.512579		-0.45	-1.15	2.50	2.33	2.33	0.88	0.82	0.88	0.83	0.88	Zu B et al.	2018	Ore Geology Reviews	101	54-73
471	13Un-70	CAOB	Tianshan	70.3842	41.6517	Granodiorite	Andagul	307	307	8.09	46.27	0.1063	0.512559		-0.46	-1.54	2.10	2.00	2.00	0.90	0.84	0.90	0.84	0.91	Zu B et al.	2018	Ore Geology Reviews	101	54-73
472	13Un-69	CAOB	Tianshan	70.3842	41.6517	Granodiorite	Andagul	307	307	8.83	47.96	0.1119	0.512586		-0.43	-1.01	2.40	2.31	2.31	0.88	0.84	0.88	0.85	0.88	Zu B et al.	2018	Ore Geology Reviews	101	54-73
473	13Un-68	CAOB	Tianshan	70.3842	41.6517	Granodiorite	Andagul	307	307	8.75	49.37	0.1078	0.512571		-0.45	-1.31	2.30	2.18	2.18	0.89	0.83	0.89	0.84	0.89	Zu B et al.	2018	Ore Geology Reviews	101	54-73
474	13Un-22	CAOB	Tianshan	70.3342	41.6517	Granodiorite	Andagul	307	307	8.56	49.13	0.1060	0.512619		-0.46	-0.37	3.30	3.18	3.18	0.81	0.75	0.81	0.75	0.81	Zu B et al.	2018	Ore Geology Reviews	101	54-73
475	13Un-21	CAOB	Tianshan	70.3342	41.6517	Granodiorite	Andagul	307	307	8.20	49.66	0.1004	0.512625		-0.49	-0.25	3.70	3.52	3.52	0.78	0.71	0.78	0.71	0.78	Zu B et al.	2018	Ore Geology Reviews	101	54-73
476	13Un-20	CAOB	Tianshan	70.3342	41.6517	Granodiorite	Andagul	307	307	8.81	51.61	0.1038	0.512634		-0.47	-0.08	3.70	3.56	3.56	0.78	0.72	0.78	0.72	0.78	Zu B et al.	2018	Ore Geology Reviews	101	54-73
477	13Un-18	CAOB	Tianshan	70.3342	41.6517	Granodiorite	Andagul	307	307	10.31	58.68	0.1068	0.512621		-0.46	-0.33	3.30	3.19	3.19	0.81	0.75	0.81	0.76	0.81	Zu B et al.	2018	Ore Geology Reviews	101	54-73
478	13Un-17	CAOB	Tianshan	70.3342	41.6517	Granodiorite	Andagul	307	307	9.02	53.82	0.1018	0.512648		-0.48	0.20	4.00	3.91	3.91	0.75	0.68	0.75	0.69	0.75	Zu B et al.	2018	Ore Geology Reviews	101	54-73
479	13Un-16	CAOB	Tianshan	70.3342	41.6517	Granodiorite	Andagul	307	307	9.02	49.83	0.1101	0.512623		-0.44	-0.29	3.20	3.10	3.10	0.81	0.78	0.81	0.78	0.82	Zu B et al.	2018	Ore Geology Reviews	101	54-73
480	SKH-3	CAOB	Transbaikalia and Mongolia	100.4167	46.8333	Granitoid		270	270	3.73	18.34	0.1218	0.512675		-0.38	0.72	3.30	3.30	3.30	0.77	0.79	0.77	0.79	0.77	Kozakov IK et al.	2007	Petrology	15（2）	126-150
481	4467	CAOB	Transbaikalia and Mongolia	100.3833	46.9167	Granitoid		330	330	5.62	26.80	0.1265	0.512693		-0.36	1.07	4.03	4.03	4.03	0.76	0.80	0.76	0.80	0.76	Kozakov IK et al.	2007	Petrology	15（2）	126-150
482	4466	CAOB	Transbaikalia and Mongolia	100.4333	46.6667	Granitoid		330	330	9.02	26.00	0.2091	0.512886		0.06	4.84	4.32	4.32	4.32	0.72	8.53	0.72	8.53	0.72	Kozakov IK et al.	2007	Petrology	15（2）	126-150
483	4427-1	CAOB	Transbaikalia and Mongolia	99.3333	46.6000	Granitoid		280	280	13.20	65.50	0.1218	0.512788		-0.38	2.93	5.60	5.60	5.60	0.59	0.60	0.59	0.60	0.59	Kozakov IK et al.	2007	Petrology	15（2）	126-150
484	4421-1	CAOB	Transbaikalia and Mongolia	98.1667	46.3333	Granitoid		280	280	3.56	22.50	0.0957	0.512636		-0.51	-0.04	3.57	3.57	3.57	0.75	0.66	0.75	0.66	0.75	Kozakov IK et al.	2007	Petrology	15（2）	126-150
485	5603	CAOB	Transbaikalia and Mongolia	97.2500	46.5833	Granitoid		270	270	8.42	43.30	0.1176	0.512553		-0.40	-1.66	1.07	1.07	1.07	0.95	0.95	0.95	0.95	0.95	Kozakov IK et al.	2007	Petrology	15（2）	126-150
486	5803	CAOB	Transbaikalia and Mongolia	97.2167	46.5000	Granitoid		350	350	9.24	51.10	0.1083	0.512655		-0.45	0.33	4.28	4.28	4.28	0.75	0.72	0.75	0.72	0.75	Kozakov IK et al.	2007	Petrology	15（2）	126-150
487	Ga-1	CAOB	Transbaikalia and Mongolia	98.1000	46.4833	Granitoid		270	270	9.91	49.50	0.1200	0.512632		-0.39	-0.12	2.53	2.53	2.53	0.83	0.84	0.83	0.84	0.83	Kozakov IK et al.	2007	Petrology	15（2）	126-150
488	5041-1	CAOB	Transbaikalia and Mongolia	97.1667	46.4333	Granitoid		240	240	3.96	25.60	0.0926	0.512610		-0.53	-0.55	2.64	2.64	2.64	0.80	0.68	0.80	0.68	0.80	Kozakov IK et al.	2007	Petrology	15（2）	126-150

489	4469	CAOB	Transbaikalia and Mongolia	97.1667	46.3833	Granitoid		280	280	10.90	42.90	0.1532	0.512675		-0.22	0.72	2.28	2.28	2.28	0.86	1.20	0.86	1.20	0.86	Kozakov IK et al.	2007	Petrology	15（2）	126-150
490	4422-1	CAOB	Transbaikalia and Mongolia	97.0833	46.3333	Granitoid		280	280	2.50	14.20	0.1066	0.512587		-0.46	-0.99	2.22	2.22	2.22	0.86	0.80	0.86	0.80	0.86	Kozakov IK et al.	2007	Petrology	15（2）	126-150
491	4464-4	CAOB	Transbaikalia and Mongolia	100.9667	45.6667	Ultrametamorphic granitoid		360	360	2.64	9.73	0.1635	0.512454		-0.17	-3.59	-2.06	-2.06	-2.06	1.27	2.11	1.27	2.11	1.28	Kozakov IK et al.	2007	Petrology	15（2）	126-150
492	4622	CAOB	Transbaikalia and Mongolia	100.8333	45.3333	Ultrametamorphic granitoid		355	355	3.67	13.18	0.1685	0.512514		-0.14	-2.42	-1.14	-1.14	-1.14	1.20	2.14	1.20	2.14	1.20	Kozakov IK et al.	2007	Petrology	15（2）	126-150
493	3750	CAOB	Transbaikalia and Mongolia	100.9167	45.2500	Ultrametamorphic granitoid		360	360	4.86	24.90	0.1180	0.512538		-0.40	-1.95	1.66	1.66	1.66	0.97	0.97	0.97	0.97	0.97	Kozakov IK et al.	2007	Petrology	15（2）	126-150
494	3749	CAOB	Transbaikalia and Mongolia	100.5833	45.4167	Ultrametamorphic granitoid		360	360	4.89	24.20	0.1222	0.512535		-0.38	-2.01	1.41	1.41	1.41	1.00	1.02	1.00	1.02	1.00	Kozakov IK et al.	2007	Petrology	15（2）	126-150
495	3746	CAOB	Transbaikalia and Mongolia	100.5000	45.6667	Ultrametamorphic granitoid		360	360	5.86	35.10	0.1010	0.512607		-0.49	-0.60	3.79	3.79	3.79	0.80	0.73	0.80	0.73	0.80	Kozakov IK et al.	2007	Petrology	15（2）	126-150
496	3518	CAOB	Transbaikalia and Mongolia	100.3333	45.9167	Ultrametamorphic granitoid		360	360	2.23	8.26	0.1634	0.512571		-0.17	-1.31	0.22	0.22	0.22	1.09	1.75	1.09	1.75	1.09	Kozakov IK et al.	2007	Petrology	15（2）	126-150
497	3511	CAOB	Transbaikalia and Mongolia	100.3667	45.5000	Ultrametamorphic granitoid		360	360	21.75	82.30	0.1597	0.512842		-0.19	3.98	5.68	5.68	5.68	0.65	0.87	0.65	0.87	0.65	Kozakov IK et al.	2007	Petrology	15（2）	126-150
498	3684-1	CAOB	Transbaikalia and Mongolia	100.3333	45.0000	Granitoid		370	370	2.79	12.94	0.1302	0.512842		-0.34	3.98	7.12	7.12	7.12	0.54	0.56	0.54	0.56	0.54	Kozakov IK et al.	2007	Petrology	15（2）	126-150
499	3663	CAOB	Transbaikalia and Mongolia	99.1667	44.6667	Granitoid		370	370	3.86	16.89	0.1370	0.512807		-0.30	3.30	6.12	6.12	6.12	0.62	0.68	0.62	0.68	0.62	Kozakov IK et al.	2007	Petrology	15（2）	126-150
500	3660-1	CAOB	Transbaikalia and Mongolia	98.6667	45.2500	Granitoid		370	370	2.99	25.10	0.0714	0.512647		-0.64	0.18	6.09	6.09	6.09	0.62	0.54	0.62	0.54	0.62	Kozakov IK et al.	2007	Petrology	15（2）	126-150
501	5188	CAOB	Transbaikalia and Mongolia	96.3333	45.2500	Granitoid		385	385	7.99	47.70	0.1003	0.512669		-0.49	0.60	5.34	5.34	5.34	0.70	0.65	0.70	0.65	0.70	Kozakov IK et al.	2007	Petrology	15（2）	126-150
502	4131	CAOB	Transbaikalia and Mongolia	97.1167	45.3833	Granitoid		380	380	4.04	19.70	0.1240	0.512604		-0.37	-0.66	2.86	2.86	2.86	0.89	0.93	0.89	0.93	0.89	Kozakov IK et al.	2007	Petrology	15（2）	126-150
503	3995	CAOB	Transbaikalia and Mongolia	97.0833	45.4167	Granitoid		380	380	2.86	17.10	0.1012	0.512618		-0.49	-0.39	4.24	4.24	4.24	0.78	0.72	0.78	0.72	0.78	Kozakov IK et al.	2007	Petrology	15（2）	126-150
504	FK10-91	CAOB	NE China	117.1310	49.3967	Rhyolite		125	150	10.94	50.03	0.1322	0.512735		-0.33	1.89	3.10	3.13	3.13	0.68	0.78	0.68	0.59		Li SQ et al.	2014	Lithos	190-191	204-219
505	FK10-90	CAOB	NE China	117.1310	49.3967	Rhyolite		125	150	10.04	46.20	0.1313	0.512731		-0.33	1.81	3.00	3.07	3.07	0.69	0.78	0.69	0.59		Li SQ et al.	2014	Lithos	190-191	204-219
506	FK10-89	CAOB	NE China	117.5680	49.3160	Rhyolite		125	150	8.16	34.93	0.1413	0.512730		-0.28	1.79	2.80	2.86	2.86	0.71	0.88	0.71	0.67		Li SQ et al.	2014	Lithos	190-191	204-219
507	FK10-88	CAOB	NE China	117.5604	49.3131	Rhyolite		125	150	6.74	32.73	0.1246	0.512711		-0.37	1.42	2.70	2.80	2.80	0.71	0.75	0.71	0.58		Li SQ et al.	2014	Lithos	190-191	204-219
508	FK10-87	CAOB	NE China	117.5536	49.3097	Rhyolite		125	150	9.72	41.17	0.1423	0.512756		-0.28	2.30	3.30	3.34	3.34	0.67	0.84	0.67	0.62		Li SQ et al.	2014	Lithos	190-191	204-219
509	FK10-86	CAOB	NE China	117.5503	49.3069	Rhyolite		125	150	10.63	45.80	0.1404	0.512734		-0.29	1.87	2.90	2.95	2.95	0.70	0.87	0.70	0.65		Li SQ et al.	2014	Lithos	190-191	204-219
510	FK10-85	CAOB	NE China	117.5461	49.3012	Rhyolite		125	150	9.81	41.12	0.1442	0.512732		-0.27	1.83	2.80	2.84	2.84	0.71	0.92	0.71	0.69		Li SQ et al.	2014	Lithos	190-191	204-219
511	FK10-84	CAOB	NE China	117.5461	49.3012	Rhyolite		125	150	8.84	37.90	0.1411	0.512726		-0.28	1.72	2.70	2.78	2.78	0.71	0.89	0.71	0.67		Li SQ et al.	2014	Lithos	190-191	204-219
512	FK10-83	CAOB	NE China	117.5416	49.2932	Rhyolite		125	150	9.73	40.38	0.1457	0.512742		-0.26	2.03	3.00	3.00	3.00	0.69	0.91	0.69	0.68		Li SQ et al.	2014	Lithos	190-191	204-219
513	FK10-108	CAOB	NE China	120.4678	50.2455	Rhyolite		125	150	4.90	28.85	0.1027	0.512701		-0.48	1.23	2.90	3.03	3.03	0.69	0.62	0.69	0.48		Li SQ et al.	2014	Lithos	190-191	204-219
514	FK10-107	CAOB	NE China	120.4678	50.2455	Dacite		125	150	5.48	30.29	0.1093	0.512717		-0.44	1.54	3.10	3.21	3.21	0.68	0.63	0.68	0.49		Li SQ et al.	2014	Lithos	190-191	204-219
515	FK10-105	CAOB	NE China	116.8191	48.6219	Rhyolite		125	150	3.53	21.99	0.0971	0.512553		-0.51	-1.66	0.10	0.25	0.25	0.92	0.78	0.92	0.65		Li SQ et al.	2014	Lithos	190-191	204-219
516	FK10-104	CAOB	NE China	116.8194	48.6220	Dacite		125	150	7.34	37.61	0.1180	0.512627		-0.40	-0.21	1.20	1.29	1.29	0.83	0.83	0.83	0.67		Li SQ et al.	2014	Lithos	190-191	204-219
517	FK10-102	CAOB	NE China	116.2848	48.7756	Dacite		125	150	5.09	25.00	0.1232	0.512713		-0.37	1.46	2.80	2.87	2.87	0.70	0.74	0.70	0.56		Li SQ et al.	2014	Lithos	190-191	204-219
518	TM07	CAOB	Altai, unit 3	88.1083	47.8417	Gneissic Bi granite	Timulus	459	459	6.43	27.39	0.1420	0.512393	15	-0.28	-4.78	-1.55	-1.58	-1.58	1.32	1.61	1.32	1.61	1.32	Chai FM et al.	2010	Acta Petrologica Sinica	26(2)	377-386
519	TM06	CAOB	Altai, unit 3	88.1083	47.8417	Gneissic Bi granite	Timulus	459	459	5.73	23.43	0.1479	0.512422	11	-0.25	-4.21	-1.33	-1.36	-1.36	1.30	1.68	1.30	1.68	1.31	Chai FM et al.	2010	Acta Petrologica Sinica	26(2)	377-386
520	TM05	CAOB	Altai, unit 3	88.1083	47.8417	Gneissic Bi granite	Timulus	459	459	4.62	20.30	0.1375	0.512387	12	-0.30	-4.90	-1.44	-1.43	-1.43	1.31	1.52	1.31	1.52	1.30	Chai FM et al.	2010	Acta Petrologica Sinica	26(2)	377-386
521	TM04	CAOB	Altai, unit 3	88.1083	47.8417	Gneissic Bi granite	Timulus	459	459	5.82	25.00	0.1407	0.512396	12	-0.28	-4.72	-1.49	-1.44	-1.44	1.31	1.57	1.31	1.57	1.30	Chai FM et al.	2010	Acta Petrologica Sinica	26(2)	377-386
522	TM03	CAOB	Altai, unit 3	88.1083	47.8417	Gneissic Bi granite	Timulus	459	459	7.51	32.62	0.1391	0.512415	11	-0.29	-4.35	-1.01	-0.98	-0.98	1.27	1.50	1.27	1.50	1.26	Chai FM et al.	2010	Acta Petrologica Sinica	26(2)	377-386
523	TM01	CAOB	Altai, unit 3	88.1083	47.8417	Gneissic Bi granite	Timulus	459	459	8.62	37.34	0.1396	0.512406	1	-0.29	-4.53	-1.18	-1.18	-1.18	1.29	1.53	1.29	1.53	1.29	Chai FM et al.	2010	Acta Petrologica Sinica	26(2)	377-386
524	DK8-2	CAOB	Tianshan	84.3936	44.0981	Granodiorite	Sikeshu	316	316	7.86	37.60	0.1265	0.512841		-0.36	3.96	6.80	6.79	6.79	0.52	0.54	0.52	0.54	0.52	Han BF et al.	2010	Geological Society of America Bulletin	122 (3-4)	627-640
525	07ws06	CAOB	Tianshan	84.3936	44.0981	Granodiorite	Sikeshu	316	316	6.95	30.60	0.1397	0.512885		-0.29	4.82	7.12	7.12	7.12	0.49	0.55	0.49	0.55	0.50	Han BF et al.	2010	Geological Society of America Bulletin	122 (3-4)	627-640
526	07ws05	CAOB	Tianshan	84.3936	44.0981	Granodiorite	Sikeshu	316	316	6.70	29.90	0.1355	0.512888		-0.31	4.88	7.35	7.34	7.34	0.48	0.51	0.48	0.51	0.48	Han BF et al.	2010	Geological Society of America Bulletin	122 (3-4)	627-640
527	07ws04	CAOB	Tianshan	84.3936	44.0981	Granodiorite	Sikeshu	316	316	4.57	19.50	0.1418	0.512879		-0.28	4.70	6.92	6.91	6.91	0.51	0.58	0.51	0.58	0.51	Han BF et al.	2010	Geological Society of America Bulletin	122 (3-4)	627-640
528	BLH08-4	NCC	NMNCC	113.5558	42.4111	Syenogranite porphyry	Bilihe	253	253	3.66	20.76	0.1067	0.512558	8	-0.46	-1.56	1.34	1.35	1.35	0.91	0.84	0.91	0.85	0.92	Yang ZM et al.	2016	Gondwana Research	34	296-314
529	BLH08-3	NCC	NMNCC	113.5569	42.4106	Syenogranite porphyry	Bilihe	253	253	5.06	28.91	0.1058	0.512564	9	-0.46	-1.44	1.50	1.49	1.49	0.90	0.83	0.90	0.83	0.90	Yang ZM et al.	2016	Gondwana Research	34	296-314
530	BLH08-2	NCC	NMNCC	113.5569	42.4097	Syenogranite porphyry	Bilihe	253	253	4.03	22.94	0.1063	0.512560	10	-0.46	-1.52	1.40	1.40	1.40	0.91	0.84	0.91	0.84	0.91	Yang ZM et al.	2016	Gondwana Research	34	296-314
531	BLH08-1	NCC	NMNCC	113.5564	42.4089	Syenogranite porphyry	Bilihe	253	253	1.83	10.24	0.1080	0.512561	10	-0.45	-1.50	1.36	1.36	1.36	0.91	0.85	0.91	0.85	0.92	Yang ZM et al.	2016	Gondwana Research	34	296-314
532	8-6-184	NCC	NMNCC	113.5461	42.3944	Qtz diorite porphyry	Bilihe	261	261	3.82	19.43	0.1189	0.512429	6	-0.40	-4.08	-1.48	-1.49	-1.49	1.15	1.16	1.15	1.16	1.15	Yang ZM et al.	2016	Gondwana Research	34	296-314
533	48-2-697	NCC	NMNCC	113.5461	42.3944	Qtz diorite porphyry	Bilihe	261	261	3.92	19.92	0.1189	0.512429	6	-0.40	-4.08	-1.48	-1.49	-1.49	1.15	1.16	1.15	1.16	1.15	Yang ZM et al.	2016	Gondwana Research	34	296-314
534	48-2-484	NCC	NMNCC	113.5461	42.3944	Qtz diorite porphyry	Bilihe	261	261	3.84	19.32	0.1202	0.512425	8	-0.39	-4.15	-1.60	-1.61	-1.61	1.16	1.18	1.16	1.18	1.16	Yang ZM et al.	2016	Gondwana Research	34	296-314
535	48-2-381	NCC	NMNCC	113.5461	42.3944	Qtz diorite porphyry	Bilihe	261	261	3.71	17.84	0.1259	0.512429	9	-0.36	-4.08	-1.72	-1.72	-1.72	1.17	1.25	1.17	1.25	1.17	Yang ZM et al.	2016	Gondwana Research	34	296-314
536	007-217	NCC	NMNCC	113.5417	42.3856	Qtz diorite porphyry	Bilihe	261	261	3.62	18.13	0.1208	0.512383	10	-0.39	-4.97	-2.44	-2.45	-2.45	1.23	1.26	1.23	1.26	1.23	Yang ZM et al.	2016	Gondwana Research	34	296-314
537	007-146	NCC	NMNCC	113.5417	42.3856	Qtz diorite porphyry	Bilihe	261	261	3.98	16.31	0.1260	0.512426	7	-0.36	-4.14	-1.78	-1.78	-1.78	1.17	1.26	1.17	1.26	1.18	Yang ZM et al.	2016	Gondwana Research	34	296-314
538	007-136	NCC	NMNCC	113.5417	42.3856	Qtz diorite porphyry	Bilihe	261	261	4.02	19.95	0.1218	0.512516	8	-0.38	-2.38	0.12	0.11	0.11	1.02	1.05	1.02	1.05	1.02	Yang ZM et al.	2016	Gondwana Research	34	296-314
539	14GW532	CAOB	Jiamusi	133.0165	47.9917	Rhyolite		276	276	3.22	16.90	0.1152	0.512437		-0.41	-3.92	-1.02	-1.06	-1.06	1.13	1.10	1.13	1.10	1.13	Bi JH et al.	2016	Journal of Asian Earth Sciences	134	160-175
540	14GW518	CAOB	Jiamusi	133.0165	47.9917	Rhyolite		276	276	4.25	23.40	0.1098	0.512390		-0.44	-4.84	-1.74	-1.78	-1.78	1.19	1.11	1.19	1.11	1.19	Bi JH et al.	2016	Journal of Asian Earth Sciences	134	160-175
541	14GW177	CAOB	Jiamusi	132.9149	47.8580	Rhyolite		276	276	8.55	43.70	0.1160	0.512395		-0.41	-4.74	-2.16	-1.90	-1.90	1.20	1.18	1.20	1.20	1.22	Bi JH et al.	2016	Journal of Asian Earth Sciences	134	160-175
542	13GW186	CAOB	Jiamusi	133.0165	47.9917	Rhyolite		276	276	6.18	32.20	0.1183	0.512380		-0.40	-5.03	-1.95	-2.28	-2.28	1.23	1.23	1.23	1.21	1.20	Bi JH et al.	2016	Journal of Asian Earth Sciences	134	160-175
543	KWSY8	CAOB	tau-borokonu mountain, Tians	82.3333	45.0000	Granite	Kongwusayi	298	298	7.63	33.73	0.1369	0.512707		-0.30	1.35	7.44	3.62	3.62	0.76	0.88	0.76		0.86	Liu ZQ et al.	2005	Acta Petrologica Sinica	21(3)	623-639
544	KWSY5	CAOB	tau-borokonu mountain, Tians	82.3333	45.0000	Granite	Kongwusayi	298	298	7.02	31.45	0.1352	0.512702		-0.31	1.25	7.51	3.59	3.59	0.77	0.87	0.77		0.86	Liu ZQ et al.	2005	Acta Petrologica Sinica	21(3)	623-639
545	KWSY28	CAOB	tau-borokonu mountain, Tians	82.3333	45.0000	Granite vein	Kasangbulake	285	285	8.89	25.45	0.2114	0.512931		0.07	5.72	5.18	5.18	5.18	0.60	13.91	0.60		0.60	Liu ZQ et al.	2005	Acta Petrologica Sinica	21(3)	623-639
546	KWSY27	CAOB	North Tianshan	82.3333	45.0000	Granite	Kaqinbulake	292	271			0.1529	0.512804		-0.22	3.24	4.76	4.75	4.75	0.65	0.87	0.65	0.87	0.65	Liu ZQ et al.	2005	Acta Petrologica Sinica	21(3)	623-639
547	KWSY24*	CAOB	North Tianshan	82.3333	45.0000	Granite	Kaqinbulake	292	271			0.1524	0.512808		-0.23	3.32	4.85	4.85	4.85	0.64	0.85	0.64	0.85	0.65	Liu ZQ et al.	2005	Acta Petrologica Sinica	21(3)	623-639
548	KWSY22	CAOB	North Tianshan	82.3333	45.0000	Rhyolite	Alataoshandongbu	271	271			0.1379	0.512659		-0.30	0.41	2.45	2.44	2.44	0.84	0.99	0.84	0.99	0.84	Liu ZQ et al.	2005	Acta Petrologica Sinica	21(3)	623-639
549	KWSY17*	CAOB	North Tianshan	82.3333	45.0000	Rhyolite	Alataoshandongbu	271	271			0.1385	0.512643		-0.30	0.10	2.11	2.11	2.11	0.87	1.03	0.87	1.03	0.87	Liu ZQ et al.	2005	Acta Petrologica Sinica	21(3)	623-639
550	KWSY14	CAOB	North Tianshan	82.3333	45.0000	Rhyolite	Alataoshandongbu	271	271			0.1294	0.512627		-0.34	-0.21	2.12	2.11	2.11	0.87	0.95	0.87	0.95	0.87	Liu ZQ et al.	2005	Acta Petrologica Sinica	21(3)	623-639
551	KWSY10	CAOB	tau-borokonu mountain, Tians	82.3333	45.0000	Granite vein	Kasangbulake	285	285	5.00	12.96	0.2334	0.512859		0.19	4.31	2.98	2.98	2.98	0.82	-2.28	0.82		0.82	Liu ZQ et al.	2005	Acta Petrologica Sinica	21(3)	623-639
552	KWSY1	CAOB	tau-borokonu mountain, Tians	82.3333	45.0000	Granite	Kongwusayi	298	298	7.11	30.71	0.1402	0.512705		-0.29	1.31	3.46	3.46	3.46	0.78	0.92	0.78		0.78	Liu ZQ et al.	2005	Acta Petrologica Sinica	21(3)	623-639
553	KWSY04	CAOB	tau-borokonu mountain, Tians	82.3333	45.0000	Granite vein	Kasangbulake	285	285	6.62	27.58	0.1454	0.512710		-0.26	1.40	3.27	3.27	3.27	0.78	0.98	0.78		0.78	Liu ZQ et al.	2005	Acta Petrologica Sinica	21(3)	623-639
554	KWSY03	CAOB	tau-borokonu mountain, Tians	82.3333	45.0000	Granite vein	Kasangbulake	285	285	7.80	26.49	0.1782	0.512823		-0.09	3.61	4.28	4.28	4.28	0.70	1.40	0.70		0.70	Liu ZQ et al.	2005	Acta Petrologica Sinica	21(3)	623-639
555	4124	CAOB	Altai, unit 2	87.0369	48.7828	Bi monzogranite	Kanasi	398	398	10.68	49.10	0.1317	0.512444	15	-0.33	-3.78	-0.50	-0.48	-0.48	1.18	1.31	1.18	1.31	1.18	Tong Y et al.	2007	Acta Petrologica Sinica	23(8)	1933-1944
556	4094	CAOB	Altai, unit 3	88.8997	47.6406	Gneissic Bi granite	Jonkul	399	399	12.09	66.82	0.1095	0.512376	16	-0.44	-5.11	-0.70	-0.67	-0.67	1.20	1.13	1.20	1.13	1.19	Tong Y et al.	2007	Acta Petrologica Sinica	23(8)	1933-1944
557	XLG-40	CAOB	NE China	124.7500	51.6670	Granodiorite porphyry	Xiaokele	150	150	4.63	28.00	0.1000	0.512529		-0.49	-2.13	-0.27	-0.27	-0.27	0.96	0.83	0.96	0.83		Deng CZ et al.	2019	Ore Geology Reviews	107	417-433
558	XLG-4	CAOB	NE China	124.7500	51.6670	Granodiorite porphyry	Xiaokele	150	150	2.08	14.60	0.0861	0.512474		-0.56	-3.20	-1.08	-1.08	-1.08	1.03	0.81	1.03	0.81		Deng CZ et al.	2019	Ore Geology Reviews	107	417-433
559	XLG-34	CAOB	NE China	124.7500	51.6670	Granodiorite porphyry	Xiaokele	150	150	4.32	25.60	0.1020	0.512488		-0.48	-2.93	-1.11	-1.11	-1.11	1.03	0.90	1.03	0.90		Deng CZ et al.	2019	Ore Geology Reviews	107	417-433
560	XLG-30	CAOB	NE China	124.7500	51.6670	Granodiorite porphyry	Xiaokele	148	150	3.43	21.40	0.0969	0.512505		-0.51	-2.59	-0.71	-0.68	-0.68	0.99	0.84	0.99	0.84		Deng CZ et al.	2019	Ore Geology Reviews	107	417-433
561	XLG-2	CAOB	NE China	124.7500	51.6670	Granodiorite porphyry	Xiaokele	150	150	3.74	23.30	0.0970	0.512480		-0.51	-3.08	-1.17	-1.17	-1.17	1.03	0.88	1.03	0.88		Deng CZ et al.	2019	Ore Geology Reviews	107	417-433
562	ZKIV901-486	CAOB	South Erguna Block	116.6875	49.4722	Qtz monzonite		131	150	10.30	57.90	0.1131	0.512588		-0.43	-0.98	0.42	0.62	0.62	0.89	0.85	0.89			Han R et al.	2020	Ore Geology Reviews	122	103526
563	ZKIV2703-663	CAOB	South Erguna Block	116.6875	49.4700	Qtz monzonite		132	150	10.40	59.10	0.1118	0.512596		-0.43	-0.82	0.61	0.81	0.81	0.87	0.83	0.87			Han R et al.	2020	Ore Geology Reviews	122	103526
564	ZKI-10301-378	CAOB	South Erguna Block	116.7111	49.4583	Qtz monzonite		128	150	10.30	59.40	0.1103	0.512591		-0.44	-0.92	0.50	0.74	0.74	0.88	0.82	0.88			Han R et al.	2020	Ore Geology Reviews	122	103526
565	YM-4	CAOB	North Tianshan	82.3333	44.0000	Andesite		390	390	4.69	20.98	0.1351	0.512237		-0.31	-7.82	-4.76	-4.76	-4.76	1.52	1.77	1.52	1.77	1.53	An F et al.	2013	Journal of Asian Earth Sciences	78	100-113
566	YM2-1	CAOB	North Tianshan	82.3333	44.0000	Andesite		390	390	4.55	20.73	0.1328	0.512244		-0.32	-7.69	-4.50	-4.51	-4.51	1.50	1.70	1.50	1.71	1.51	An F et al.	2013	Journal of Asian Earth Sciences	78	100-113
567	JX3-1	CAOB	North Tianshan	82.3333	44.0000	Andesite		375	375	4.53	20.78	0.1318	0.512251		-0.33	-7.55	-4.45	-4.44	-4.44	1.48	1.67	1.48	1.67	1.49	An F et al.	2013	Journal of Asian Earth Sciences	78	100-113
568	JX1-1	CAOB	North Tianshan	82.3333	44.0000	Andesite		375	375	4.61	21.15	0.1319	0.512233		-0.33	-7.90	-4.79	-4.80	-4.80	1.51	1.70	1.51	1.71	1.52	An F et al.	2013	Journal of Asian Earth Sciences	78	100-113
569	09AL76	CAOB	Alashan	104.3794	40.3981	Qtz diorite		282	282	2.34	9.30	0.1522	0.512010	10	-0.23	-12.25	-10.5	-10.6	-10.6	1.90	2.81	1.90	2.81	1.89	Dan W et al.	2014	Lithos	204	144-158
570	09AL75	CAOB	Alashan	104.3794	40.3981	Qtz diorite		282	282	1.20	7.18	0.1091	0.511986	11	-0.45	-12.72	-9.4	-9.6	-9.6	1.82	1.69	1.82	1.69	1.82	Dan W et al.	2014	Lithos	204	144-158
571	09AL73	CAOB	Alashan, NH zone	104.3794	40.3981	Qtz diorite		282	282	1.72	8.04	0.1291	0.511994	9	-0.34	-12.56	-10.0	-10.1	-10.1	1.87	2.08	1.87	2.08	1.86	Dan W et al.	2014	Lithos	204	144-158
572	09AL70	CAOB	Alashan, NH zone	104.3842	40.3919	Diorite		280	280	5.12	22.30	0.1390	0.511920	9	-0.29	-14.01	-11.8	-11.9	-11.9	2.01	2.50	2.01	2.50	2.00	Dan W et al.	2014	Lithos	204	144-158
573	09AL42	CAOB	Alashan, NH zone	104.7692	40.1583	Monzogranite		280	280	3.57	16.90	0.1277	0.511798	6	-0.35	-16.39	-13.8	-13.9	-13.9	2.17	2.39	2.17	2.39	2.16	Dan W et al.	2014	Lithos	204	144-158
574	09AL38	CAOB	Alashan, NH zone	104.7692	40.1583	Granodiorite		283	283	2.86	23.20	0.0746	0.511685	8	-0.62	-18.59	-14.0	-14.2	-14.2	2.20	1.60	2.20	1.60	2.20	Dan W et al.	2014	Lithos	204	144-158
575	09AL37	CAOB	Alashan, NH zone	104.7114	40.1719	Granodiorite		283	283	3.48	20.10	0.1046	0.511739	7	-0.47	-17.54	-14.1	-14.2	-14.2	2.20	1.96	2.20	1.96	2.19	Dan W et al.	2014	Lithos	204	144-158
576	09AL33	CAOB	Alashan, NH zone	104.7692	40.1583	Granodiorite		281	281	3.44	18.00	0.1157	0.511781	6	-0.41	-16.72	-13.7	-13.8	-13.8	2.17	2.12	2.17	2.12	2.16	Dan W et al.	2014	Lithos	204	144-158
577	09AL27	CAOB	Alashan, NH zone	104.8881	40.0953	Monzogranite		284	284	1.93	8.10	0.1441	0.511770	7	-0.27	-16.93	-14.9	-15.0	-15.0	2.26	3.00	2.26	3.00	2.25	Dan W et al.	2014	Lithos	204	144-158
578	09AL22-1	CAOB	Alashan, NH zone	104.8300	39.8881	Monzogranite		278	278	3.85	20.00	0.1161	0.511890	6	-0.41	-14.59	-11.6	-11.7	-11.7	2.00	1.96	2.00	1.96	1.99	Dan W et al.	2014	Lithos	204	144-158
579	09AL17-1	CAOB	Alashan	104.9406	39.9411	Monzogranite		280	280	3.41	16.70	0.1237	0.511900	7	-0.37	-14.40	-11.6	-11.8	-11.8	2.00	2.11	2.00	2.11	1.99	Dan W et al.	2014	Lithos	204	144-158
580	09AL10-1	CAOB	Alashan	105.0042	39.9114	Monzogranite		280	280	5.35	27.60	0.1174	0.512071	6	-0.40	-11.06	-8.09	-8.23	-8.23	1.71	1.70	1.71	1.70	1.71	Dan W et al.	2014	Lithos	204	144-158
581	Wukuli-4	CAOB	Eastern Junggar	90.8200	46.7011	Granitic rock		305	305	3.51	15.50	0.1369	0.512345	10	-0.30	-5.72	-3.60	-3.39	-3.39	1.34	1.59	1.34	1.59	1.34	Zhang C et al.	2017	Journal of Asian Earth Sciences	159	185-208
582	Wukuli-3	CAOB	Eastern Junggar	90.8200	46.7011	Granitic rock		305	305	4.43	19.80	0.1353	0.512341	9	-0.31	-5.79	-3.60	-3.40	-3.40	1.34	1.57	1.34	1.57	1.34	Zhang C et al.	2017	Journal of Asian Earth Sciences	159	185-208
583	Wukuli-2	CAOB	Eastern Junggar	90.8200	46.7011	Granitic rock		305	305	4.48	20.50	0.1321	0.512346	9	-0.33	-5.70	-3.40	-3.18	-3.18	1.32	1.50	1.32	1.50	1.32	Zhang C et al.	2017	Journal of Asian Earth Sciences	159	185-208
584	Wukuli-1	CAOB	Eastern Junggar	90.8200	46.7011	Granitic rock		305	305	3.80	16.90	0.1359	0.512343	9	-0.31	-5.75	-3.60	-3.39	-3.39	1.34	1.58	1.34	1.58	1.34	Zhang C et al.	2017	Journal of Asian Earth Sciences	159	185-208
585	Aketas-6	CAOB	Eastern Junggar	89.1792	46.8747	Granitic rock		308	308	2.89	14.60	0.1197	0.512742	5	-0.39	2.03	5.10	5.06	5.06	0.66	0.66	0.66	0.66	0.66	Zhang C et al.	2017	Journal of Asian Earth Sciences	159	185-208
586	Aketas-5	CAOB	Eastern Junggar	89.6386	46.8261	Granitic rock		308	308	2.84	15.40	0.1115	0.512732	5	-0.43	1.83	5.20	5.18	5.18	0.65	0.62	0.65	0.62	0.65	Zhang C et al.	2017	Journal of Asian Earth Sciences	159	185-208

587	Aketas-4	CAOB	Eastern Junggar	89.6386	46.8261	Granitic rock		308	308	2.83	18.60	0.0920	0.512754	5	-0.53	2.26	6.40	6.38	6.38	0.55	0.50	0.55	0.50	0.55	Zhang C et al.	2017	Journal of Asian Earth Sciences	159	185-208
588	Aketas-3	CAOB	Eastern Junggar	89.6386	46.8261	Granitic rock		308	308	3.04	14.90	0.1233	0.512735	6	-0.37	1.89	4.80	4.78	4.78	0.68	0.70	0.68	0.70	0.68	Zhang C et al.	2017	Journal of Asian Earth Sciences	159	185-208
589	Aketas-2	CAOB	Eastern Junggar	89.6386	46.8261	Granitic rock		308	308	2.91	13.80	0.1275	0.512745	7	-0.35	2.09	4.80	4.81	4.81	0.68	0.72	0.68	0.72	0.68	Zhang C et al.	2017	Journal of Asian Earth Sciences	159	185-208
590	Aketas-1	CAOB	Eastern Junggar	89.6386	46.8261	Granitic rock		308	308	3.06	14.10	0.1312	0.512766	7	-0.33	2.50	5.10	5.07	5.07	0.65	0.71	0.65	0.71	0.65	Zhang C et al.	2017	Journal of Asian Earth Sciences	159	185-208
591	TC11~1	CAOB	Kangur-juerotage,Tianshan	92.7000	42.0500	Plagiogranite porphyry	Tuwu	369	369	3.75	21.84	0.1038	0.512730		-0.47	1.79	6.20	6.17	6.17	0.62	0.58	0.62			Rui ZY et al.	2004	Acta Petrologica Sinica	(02)	229-238
592	705~846.2	CAOB	Kangur-juerotage,Tianshan	92.7000	42.0500	Plagiogranite porphyry	Tuwu	369	369	0.70	3.62	0.1175	0.512870		-0.40	4.53	8.00	8.25	8.25	0.44	0.44	0.44			Rui ZY et al.	2004	Acta Petrologica Sinica	(02)	229-238
593	705~519	CAOB	Kangur-juerotage,Tianshan	92.7000	42.0500	Plagiogranite porphyry	Tuwu	369	369	1.52	8.17	0.1126	0.512806		-0.43	3.28	7.20	7.24	7.24	0.53	0.52	0.53			Rui ZY et al.	2004	Acta Petrologica Sinica	(02)	229-238
594	004~396	CAOB	Kangur-juerotage,Tianshan	92.7000	42.0500	Plagiogranite porphyry	Tuwu	369	369	1.22	5.39	0.1368	0.512421		-0.30	-4.23	-1.40	-1.41	-1.41	1.23	1.44	1.23			Rui ZY et al.	2004	Acta Petrologica Sinica	(02)	229-238
595	002~367	CAOB	Kangur-juerotage,Tianshan	92.7000	42.0500	Plagiogranite porphyry	Tuwu	369	369	2.05	10.04	0.1235	0.512944		-0.37	5.97	9.40	9.42	9.42	0.37	0.35	0.35			Rui ZY et al.	2004	Acta Petrologica Sinica	(02)	229-238
596	001~637	CAOB	Kangur-juerotage,Tianshan	92.7000	42.0500	Plagiogranite porphyry	Tuwu	369	369	1.89	10.22	0.1119	0.512812		-0.43	3.39	7.40	7.39	7.39	0.52	0.51	0.52			Rui ZY et al.	2004	Acta Petrologica Sinica	(02)	229-238
597	001~462	CAOB	Kangur-juerotage,Tianshan	92.7000	42.0500	Plagiogranite porphyry	Tuwu	369	369	2.49	14.86	0.1012	0.512767		-0.49	2.52	7.00	7.01	7.01	0.55	0.52	0.55			Rui ZY et al.	2004	Acta Petrologica Sinica	(02)	229-238
598	A2-7-9	CAOB	Central Kazakhstan	79.9846	46.9685	Granodiorite Porphyry		331	331	3.09	17.76	0.1052	0.512700		-0.47	1.21	5.00	5.07	5.07	0.67	0.63	0.67	0.64	0.68	Cao MJ et al.	2016	American Journal of Science	316（7）	614-668
599	A2-7-5	CAOB	Central Kazakhstan	79.9846	46.9685	Granodiorite Porphyry		331	331	2.48	15.36	0.1095	0.512718		-0.44	1.56	5.20	5.24	5.24	0.66	0.63	0.66	0.64	0.66	Cao MJ et al.	2016	American Journal of Science	316（7）	614-668
600	A2-7-12	CAOB	Central Kazakhstan	79.9846	46.9685	Granodiorite Porphyry		331	331	2.36	13.18	0.1086	0.512727		-0.45	1.74	5.40	5.46	5.46	0.64	0.61	0.64	0.62	0.64	Cao MJ et al.	2016	American Journal of Science	316（7）	614-668
601	A2-6	CAOB	Central Kazakhstan	79.9846	46.9685	Granodiorite Porphyry		331	331	2.85	16.32	0.1057	0.512726		-0.46	1.72	5.60	5.56	5.56	0.63	0.60	0.63	0.60	0.64	Cao MJ et al.	2016	American Journal of Science	316（7）	614-668
602	A2-3-3	CAOB	Central Kazakhstan	79.9846	46.9685	Granodiorite Porphyry		331	331	2.94	15.96	0.1115	0.512635		-0.43	-0.06	3.60	3.54	3.54	0.80	0.77	0.80	0.77	0.80	Cao MJ et al.	2016	American Journal of Science	316（7）	614-668
603	9849-1	CAOB	SHB	126.8333	49.5556	Alkali granite	Songmushan	260	260	8.68	35.73	0.1469	0.512660	6	-0.25	0.43	2.08	2.08	2.08	0.86	1.12	0.86	1.12	0.86	Wu FY et al.	2002	Chemical Geology	187(1-2)	143-173
604	9843-6	CAOB	SMXAB	126.4694	50.2347	Alkali granite	Daheishan	292	292	11.36	65.40	0.1050	0.512680	6	-0.47	0.82	4.24	4.24	4.24	0.71	0.66	0.71	0.66	0.71	Wu FY et al.	2002	Chemical Geology	187(1-2)	143-173
605	9843-1	CAOB	SMXAB	126.4694	50.2347	Alkali granite	Daheishan	292	292	12.42	68.66	0.1094	0.512716	9	-0.44	1.52	4.78	4.77	4.77	0.67	0.63	0.67	0.64	0.67	Wu FY et al.	2002	Chemical Geology	187(1-2)	143-173
606	9832-3	CAOB	SMXAB	127.3042	50.3583	Alkali-feldspar granite	Shangmachang	106	150	3.98	19.89	0.1208	0.512595	7	-0.39	-0.84	0.18	0.61	0.61	0.89	0.91	0.89	0.91	0.89	Wu FY et al.	2002	Chemical Geology	187(1-2)	143-173
607	9832-1	CAOB	SMXAB	127.3042	50.3583	Alkali-feldspar granite	Shangmachang	106	150	4.76	25.00	0.1152	0.512584	8	-0.41	-1.05	0.05	0.51	0.51	0.90	0.88	0.90	0.88	0.90	Wu FY et al.	2002	Chemical Geology	187(1-2)	143-173
608	9806-3	CAOB	SMXAB	125.4139	49.9750	Alkali-feldspar granite	Guguhe	264	264	11.93	53.70	0.1343	0.512631	9	-0.32	-0.14	1.97	1.97	1.97	0.87	1.00	0.87	1.00	0.87	Wu FY et al.	2002	Chemical Geology	187(1-2)	143-173
609	9801-2	CAOB	SMXAB	125.2028	49.0917	Alkali-feldspar granite	Xiaoshantun	285	285	12.71	47.18	0.1629	0.512709	10	-0.17	1.38	2.62	2.61	2.61	0.83	1.32	0.83	1.33	0.84	Wu FY et al.	2002	Chemical Geology	187(1-2)	143-173
610	JN07-40	NCC	NCC	111.4592	41.4792	Appinitic	SouthInnerMongolia	319	319	5.71	26.93	0.1282	0.511932	13	-0.35	-13.77	-11.0	-11.0	-11.0	1.97	2.16	1.97	2.17	1.97	Zhang XH et al.	2013	Journal of the Geological Society	169(3)	337-351
611	GTS09-06	NCC	NCC	118.4514	41.2875	Peraluminous granite	Guangtoushan	207	207			0.0912	0.511616	12	-0.54	-19.94	-17.2	-17.1	-17.1	2.38	1.90	2.38	1.91	2.38	Zhang XH et al.	2013	Journal of the Geological Society	169(3)	337-351
612	SZ07-31	NCC	NCC	111.8619	41.3633	Appinite	South Inner Mongolia	317	317	5.56	25.58	0.1314	0.511815	14	-0.33	-16.05	-13.4	-13.4	-13.4	2.16	2.46	2.16	2.47	2.17	Zhang XH et al.	2013	Journal of the Geological Society	169(3)	337-351
613	SZ07-30	NCC	NCC	111.8619	41.3633	Appinite	South Inner Mongolia	317	317	5.09	24.31	0.1266	0.511936	15	-0.36	-13.69	-10.9	-10.9	-10.9	1.95	2.12	1.95	2.12	1.96	Zhang XH et al.	2013	Journal of the Geological Society	169(3)	337-351
614	SZ07-28	NCC	NCC	111.8619	41.3633	Appinite	South Inner Mongolia	317	317	4.68	21.13	0.1338	0.511834	11	-0.32	-15.68	-13.2	-13.1	-13.1	2.14	2.50	2.14	2.50	2.14	Zhang XH et al.	2013	Journal of the Geological Society	169(3)	337-351
615	SZ07-26	NCC	NCC	111.9617	41.4375	Appinite	South Inner Mongolia	317	317	5.02	25.85	0.1174	0.511844	12	-0.40	-15.49	-12.3	-12.3	-12.3	2.07	2.06	2.07	2.06	2.07	Zhang XH et al.	2013	Journal of the Geological Society	169(3)	337-351
616	SZ07-25	NCC	NCC	111.9617	41.4375	Appinite	South Inner Mongolia	320	320	5.46	29.07	0.1135	0.511954	21	-0.42	-13.34	-10.0	-9.95	-9.95	1.88	1.81	1.88	1.82	1.89	Zhang XH et al.	2013	Journal of the Geological Society	169(3)	337-351
617	SZ07-15	NCC	NCC	111.9372	41.4231	Appinite	South Inner Mongolia	320	320	8.38	48.14	0.1052	0.511746	13	-0.47	-17.40	-13.7	-13.7	-13.7	2.19	1.97	2.19	1.97	2.19	Zhang XH et al.	2013	Journal of the Geological Society	169(3)	337-351
618	SZ07-11	NCC	NCC	111.9372	41.4231	Appinite	South Inner Mongolia	320	320	10.78	64.99	0.1002	0.511979	14	-0.49	-12.86	-8.9	-8.9	-8.9	1.80	1.57	1.80	1.57	1.80	Zhang XH et al.	2013	Journal of the Geological Society	169(3)	337-351
619	JN07-39	NCC	NCC	111.4592	41.4792	Appinite	South Inner Mongolia	319	319	5.51	25.83	0.1289	0.511961	12	-0.34	-13.21	-10.5	-10.4	-10.4	1.92	2.13	1.92	2.13	1.93	Zhang XH et al.	2013	Journal of the Geological Society	169(3)	337-351
620	JN07-37	NCC	NCC	111.4592	41.4792	Appinite	South Inner Mongolia	319	319	4.48	22.91	0.1183	0.511932	14	-0.40	-13.77	-10.6	-10.6	-10.6	1.94	1.94	1.94	1.94	1.94	Zhang XH et al.	2013	Journal of the Geological Society	169(3)	337-351
621	ET4QJ27	CAOB	East Tianshan	91.2500	43.7500	Rhyolite		330	330	9.36	47.50	0.1190	0.512796	0	-0.40	3.08	6.37	6.35	6.35	0.57	0.57	0.57			Zhang YY et al.	2017	Gondwana Research	43	92-106
622	ET4QJ26	CAOB	East Tianshan	91.2500	43.7500	Rhyolite		330	330	9.07	45.40	0.1208	0.512806	0	-0.39	3.28	6.47	6.47	6.47	0.56	0.57	0.56			Zhang YY et al.	2017	Gondwana Research	43	92-106
623	ET4QJ25	CAOB	East Tianshan	91.2500	43.7500	Rhyolite		330	330	7.34	38.10	0.1164	0.512783	0	-0.41	2.83	6.21	6.21	6.21	0.58	0.58	0.58			Zhang YY et al.	2017	Gondwana Research	43	92-106
624	P012-32-4	CAOB	NE China	113.0000	44.0000	Granite		329	329			0.1176	0.512509		-0.40	-2.51	0.06	0.81	0.81	1.02	1.02	1.02	1.07		Wei RH et al.	2018	Lithos	308-309	242-261
625	P012-24-2	CAOB	NE China	113.0000	44.0000	Granodiorite		329	329	5.34	25.50	0.1260	0.512689		-0.36	0.99	3.22	3.95	3.95	0.76	0.80	0.76	0.86		Wei RH et al.	2018	Lithos	308-309	242-261
626	P012-24-1	CAOB	NE China	113.0000	44.0000	Granodiorite		329	329	3.20	14.50	0.1094	0.512570		-0.44	-1.33	2.21	2.34	2.34	0.89	0.85	0.89	0.85		Wei RH et al.	2018	Lithos	308-309	242-261
627	P011-35-2	CAOB	NE China	113.0000	44.0000	Diorite		329	329	4.83	22.00	0.1321	0.512617		-0.33	-0.41	2.08	2.30	2.30	0.90	1.00	0.90	1.00		Wei RH et al.	2018	Lithos	308-309	242-261
628	P011-31-2	CAOB	NE China	113.0000	44.0000	Diorite		329	329	5.06	24.30	0.1253	0.512593		-0.36	-0.88	1.88	2.12	2.12	0.91	0.96	0.91	0.96		Wei RH et al.	2018	Lithos	308-309	242-261
629	P011-31-1	CAOB	NE China	113.0000	44.0000	Diorite		329	329	5.06	22.00	0.1384	0.512686		-0.30	0.94	3.19	3.38	3.38	0.81	0.94	0.81	0.94		Wei RH et al.	2018	Lithos	308-309	242-261
630	P0021-6-3	CAOB	NE China	113.0000	44.0000	Granite		329	329	5.57	27.30	0.1228	0.512547		-0.38	-1.78	1.09	1.33	1.33	0.98	1.01	0.98	1.01		Wei RH et al.	2018	Lithos	308-309	242-261
631	P0021-5-3	CAOB	NE China	113.0000	44.0000	Granite		329	329	3.52	12.10	0.1751	0.512594		-0.11	-0.86	-0.02	0.05	0.05	1.08	2.19	1.08	2.20		Wei RH et al.	2018	Lithos	308-309	242-261
632	D7240-0-1	CAOB	NE China	113.0000	44.0000	Granite		329	329	8.39	36.80	0.1372	0.512552		-0.30	-1.68	0.63	0.82	0.82	1.02	1.19	1.02	1.19		Wei RH et al.	2018	Lithos	308-309	242-261
633	D5304-0-1	CAOB	NE China	113.0000	44.0000	Granite		329	329	6.95	35.30	0.1185	0.512563		-0.40	-1.46	1.57	1.82	1.82	0.94	0.94	0.94	0.94		Wei RH et al.	2018	Lithos	308-309	242-261
634	D2307-0-1	CAOB	NE China	113.0000	44.0000	Granite		329	329	4.06	17.70	0.1381	0.512616		-0.30	-0.43	1.85	2.03	2.03	0.92	1.08	0.92	1.08		Wei RH et al.	2018	Lithos	308-309	242-261
635	D040-2	CAOB	NE China	113.0000	44.0000	Diorite		329	329	3.42	16.00	0.1287	0.512663		-0.35	0.49	3.11	3.34	3.34	0.81	0.87	0.81	0.88		Wei RH et al.	2018	Lithos	308-309	242-261
636	15FX-94	CAOB	Tianshan	92.0000	42.0000	Plagiogranite		341	341	1.17	7.05	0.1003	0.512703		-0.49	1.27	5.40	5.46	5.46	0.65	0.60	0.65	0.61	0.66	Wang YH et al.	2016	Gondwana Research	34	109-128
637	15FX-93	CAOB	Tianshan	92.0000	42.0000	Dacite		341	341	4.09	17.60	0.1405	0.512751		-0.29	2.20	4.60	4.65	4.65	0.72	0.83	0.72	0.84	0.72	Wang YH et al.	2016	Gondwana Research	34	109-128
638	15FX-92	CAOB	Tianshan	92.0000	42.0000	Plagiogranite		341	341	1.22	6.91	0.1067	0.512785		-0.46	2.87	6.70	6.78	6.78	0.54	0.52	0.54	0.52	0.55	Wang YH et al.	2016	Gondwana Research	34	109-128
639	15FX-91	CAOB	Tianshan	92.0000	42.0000	Plagiogranite		341	341	0.79	5.36	0.0887	0.512692		-0.55	1.05	5.60	5.75	5.75	0.63	0.56	0.63	0.56	0.63	Wang YH et al.	2016	Gondwana Research	34	109-128
640	15FX-90	CAOB	Tianshan	92.0000	42.0000	Plagiogranite		341	341	0.43	2.60	0.1000	0.512707		-0.49	1.35	5.50	5.55	5.55	0.64	0.59	0.64	0.60	0.65	Wang YH et al.	2016	Gondwana Research	34	109-128
641	15FX-89	CAOB	Tianshan	92.0000	42.0000	Monzogranite		341	341	0.91	5.76	0.0954	0.512922		-0.51	5.54	9.80	9.95	9.95	0.34	0.29	0.28	0.30	0.29	Wang YH et al.	2016	Gondwana Research	34	109-128
642	HB30	CAOB	Altai, unit 4	86.0833	48.3167	Granodiorite	Tuolesalei	313	313	4.47	18.60	0.1465	0.512654	7	-0.26	0.31	2.30	2.32	2.32	0.88	1.12	0.88	0.72	0.88	Cai KD et al.	2012	Gondwana Research	22(2)	681-698
643	HB20	CAOB	Altai, unit 4	86.0833	48.3167	Granodiorite	Tuolesalei	313	313	4.53	23.20	0.1187	0.512698	7	-0.40	1.17	4.30	4.29	4.29	0.72	0.73	0.72	0.72	0.72	Cai KD et al.	2012	Gondwana Research	22(2)	681-698
644	HB18	CAOB	Altai, unit 4	86.0833	48.3167	Granodiorite	Tuolesalei	313	313	4.80	24.50	0.1189	0.512684	7	-0.40	0.90	4.00	4.00	4.00	0.75	0.75	0.75	0.72	0.75	Cai KD et al.	2012	Gondwana Research	22(2)	681-698
645	HB14	CAOB	Altai, unit 4	86.0833	48.3167	Granodiorite	Tuolesalei	313	313	4.49	19.70	0.1387	0.512652	13	-0.29	0.27	2.60	2.59	2.59	0.86	1.01	0.86	0.72	0.86	Cai KD et al.	2012	Gondwana Research	22(2)	681-698
646	HB12	CAOB	Altai, unit 4	86.0833	48.3167	Granodiorite	Tuolesalei	313	313	4.74	21.80	0.1323	0.512660	12	-0.33	0.43	3.00	3.00	3.00	0.83	0.92	0.83	0.72	0.83	Cai KD et al.	2012	Gondwana Research	22(2)	681-698
647	C15ZS08	CAOB	Western Tianshan	81.3123	43.0073	Andesites	Wusun	355	355	4.73	25.40	0.1133	0.5124583	##	-0.42	-3.51	0.27	0.27	0.27	1.08	1.05	1.08	1.05	1.09	Su W et al.	2018	Lithos	310–311	241-254
648	C15ZS10	CAOB	Western Tianshan	81.3123	43.0073	Andesites	Wusun	355	355	4.53	25.10	0.1098	0.5124602	##	-0.44	-3.47	0.47	0.47	0.47	1.07	1.01	1.07	1.01	1.07	Su W et al.	2018	Lithos	310–311	241-254
649	C13TKS05	CAOB	Western Tianshan	81.9395	43.4307	Ryholites	Wusun	351	351	3.73	20.02	0.1133	0.51250685	##	-0.42	-2.56	1.23	1.18	1.18	1.01	0.98	1.01	0.98	1.01	Su W et al.	2018	Lithos	310–311	241-254
650	C13TKS10	CAOB	Western Tianshan	81.9395	43.4307	Ryholites	Wusun	351	351	5.70	25.53	0.1359	0.51257369	##	-0.31	-1.25	1.50	1.47	1.47	0.98	1.13	0.98	1.13	0.98	Su W et al.	2018	Lithos	310–311	241-254
651	C13TKS15	CAOB	Western Tianshan	81.9395	43.4307	Ryholites	Wusun	325	325	6.28	29.67	0.1289	0.51255809	##	-0.34	-1.56	3.34	1.25	1.25	0.98	1.06	0.98	0.82	0.81	Su W et al.	2018	Lithos	310–311	241-254
652	C13TKS12	CAOB	Western Tianshan	81.9395	43.4307	Ryholites	Wusun	351	351	5.28	26.99	0.1191	0.5126442	##	-0.39	0.12	1.52	3.60	3.60	0.81	0.82	0.81	1.07	0.98	Su W et al.	2018	Lithos	310–311	241-254
653	C13TKS17	CAOB	Western Tianshan	81.9395	43.4307	Ryholites	Wusun	325	325	5.12	26.51	0.1177	0.51265026	##	-0.40	0.24	3.52	3.52	3.52	0.80	0.79	0.80	0.80	0.80	Su W et al.	2018	Lithos	310–311	241-254
654	HTP7-67-1	CAOB	West Junggar	84.4214	46.0393	Granodiorite		305	305	2.99	14.90	0.1213	0.512800	5	-0.38	3.16	6.48	6.09	6.09	0.57	0.58	0.57	0.56	0.57	Gao R et al.	2014	Lithos	204	125-143
655	HTP7-17-1	CAOB	West Junggar	84.3743	46.0293	Granodiorite		319	319	2.87	13.70	0.1267	0.512800	4	-0.36	3.16	5.74	6.01	6.01	0.59	0.61	0.59	0.64	0.59	Gao R et al.	2014	Lithos	204	125-143
656	h2576-1	CAOB	West Junggar	84.2458	46.0265	Granite porphyry		305	305	13.40	52.90	0.1531	0.512500	3	-0.22	-2.69	5.96	-1.00	-1.00	1.14	1.63	1.14	0.75	1.14	Gao R et al.	2014	Lithos	204	125-143
657	h0423-5	CAOB	West Junggar	84.5778	46.3923	K-feldspar granite		321	321	11.20	54.80	0.1236	0.512600	5	-0.37	-0.74	7.76	2.25	2.25	0.89	0.93	0.89	0.46	0.89	Gao R et al.	2014	Lithos	204	125-143
658	h0408-1	CAOB	West Junggar	84.5230	46.8841	Granodiorite		342	342	11.50	52.00	0.1337	0.512500	2	-0.32	-2.69	5.92	0.06	0.06	1.09	1.24	1.09	0.66	1.09	Gao R et al.	2014	Lithos	204	125-143
659	BGT48	CAOB	western Junggar	84.4736	45.4833	Adakite		320	320	3.21	15.75	0.1175	0.512800		-0.40	3.16	6.39	6.39	6.39	0.56	0.56	0.56	0.56	0.56	Zhang LC et al.	2006	Geology in China	33(3)	626-631
660	BGT40	CAOB	western Junggar	84.4736	45.4833	Adakite		320	320	1.35	5.02	0.1452	0.512900		-0.26	5.11	7.21	7.21	7.21	0.49	0.56	0.49	0.56	0.49	Zhang LC et al.	2006	Geology in China	33(3)	626-631
661	BGT39	CAOB	western Junggar	84.4736	45.4833	Adakite		320	320	2.56	11.88	0.1230	0.512800		-0.37	3.16	6.17	6.17	6.17	0.57	0.59	0.57	0.59	0.57	Zhang LC et al.	2006	Geology in China	33(3)	626-631
662	BGT33	CAOB	western Junggar	84.4736	45.4833	Adakite		320	320			0.0836	0.512800		-0.57	3.16	7.78	7.78	7.78	0.44	0.41	0.44	0.41	0.44	Zhang LC et al.	2006	Geology in China	33(3)	626-631
663	BGT23	CAOB	western Junggar	84.4736	45.4833	Adakite		320	320	3.29	19.70	0.0988	0.512800		-0.50	3.16	7.16	7.16	7.16	0.49	0.47	0.49	0.47	0.49	Zhang LC et al.	2006	Geology in China	33(3)	626-631
664	X-473	CAOB	East Tianshan	95.9667	42.7000	Gneissic granite	Xianshuiquan	254	254	3.11	16.27	0.1180	0.512666	13	-0.40	0.55	3.10	3.10	3.10	0.77	0.77	0.77	0.77	0.77	Tang JH et al.	2007	Acta Petrologica Sinica	23(8)	1803-1820
665	X-472	CAOB	East Tianshan	95.9500	42.6833	Gneissic granite	Xianshuiquan	254	254	4.39	23.83	0.1188	0.512779	11	-0.40	2.75	5.27	5.27	5.27	0.59	0.60	0.59	0.60	0.59	Tang JH et al.	2007	Acta Petrologica Sinica	23(8)	1803-1820
666	X-471	CAOB	East Tianshan	95.9333	42.6667	Gneissic granite	Xianshuiquan	254	254	2.72	12.04	0.1336	0.512785	12	-0.32	2.87	4.91	4.91	4.91	0.62	0.69	0.62	0.69	0.58	Tang JH et al.	2007	Acta Petrologica Sinica	23(8)	1803-1820
667	X-469	CAOB	East Tianshan	95.9167	42.6500	Gneissic granite	Xianshuiquan	254	254	2.56	12.49	0.0841	0.512765	12	-0.57	2.48	6.13	6.13	6.13	0.52	0.45	0.52	0.45	0.61	Tang JH et al.	2007	Acta Petrologica Sinica	23(8)	1803-1820
668	X-468	CAOB	East Tianshan	95.9000	42.6333	Gneissic granite	Xianshuiquan	254	254	2.58	17.01	0.1347	0.512695	13	-0.32	1.11	3.12	3.12	3.12	0.77	0.88	0.77	0.88	0.72	Tang JH et al.	2007	Acta Petrologica Sinica	23(8)	1803-1820
669	X-466	CAOB	East Tianshan	95.8833	42.6167	Gneissic granite	Xianshuiquan	254	254	2.76	15.38	0.1180	0.512743	11	-0.40	2.05	4.60	4.60	4.60	0.65	0.65	0.65	0.65	0.65	Tang JH et al.	2007	Acta Petrologica Sinica	23(8)	1803-1820
670	X-462	CAOB	Kangur-juerotage,Tianshan	95.8667	42.6000	Mylonitized granodiorite	Xianshuquan	370	370	3.07	12.85	0.1385	0.512695	13	-0.30	1.11	3.86	3.86	3.86	0.80	0.92	0.80		0.80	Tang JH et al.	2007	Acta Petrologica Sinica	23(8)	1803-1820
671	X-461	CAOB	East Tianshan	95.8667	42.6000	Mylonitized granodiorite	Xianshuquan	370	370	3.07	12.85	0.1385	0.512695	13	-0.30	1.11	3.86	3.86	3.86	0.80	0.92	0.80	0.92	0.72	Tang JH et al.	2007	Acta Petrologica Sinica	23(8)	1803-1820
672	LY-9-19-2	CAOB	Beishan	95.0380	40.9878	Lamprophyre	Huitongshan	230	230	96.24	748.60	0.0777	0.512271	10	-0.60	-7.16	-3.67	-3.67	-3.67	1.30	0.99	1.30	0.99	1.40	Liu C et al.	2006	Acta Petrologica Sinica	5	1294-1306
673	LY-9-19-1	CAOB	Beishan	95.0380	40.9878	Lamprophyre	Huitongshan	230	230	91.99	720.50	0.0772	0.512322	10	-0.61	-6.16	-2.66	-2.66	-2.66	1.22	0.92	1.22	0.92	1.32	Liu C et al.	2006	Acta Petrologica Sinica	5	1294-1306
674	LY-9-18-1	CAOB	Beishan	95.0380	40.9878	Lamprophyre	Huitongshan	230	230	83.29	519.30	0.0970	0.512390	11	-0.51	-4.84	-1.91	-1.91	-1.91	1.16	0.99	1.16	0.99	1.21	Liu C et al.	2006	Acta Petrologica Sinica	5	1294-1306
675	TH7-7	NCC	NNCC	127.0000	42.0000	Andesite		123	150	5.98	29.20	0.1238	0.512290		-0.37	-6.79	-4.16	-5.39	-5.39	1.19	1.46	1.38	1.19		Yu Y et al.	2009	Acta Geologica Sinica-English Edition	83（2）	245-257
676	TH7-23	NCC	NNCC	127.0000	42.0000	Andesite		219	219	7.61	34.60	0.1330	0.512308		-0.32	-6.44	-4.23	-4.66	-4.66	1.35	1.59	1.37	1.35		Yu Y et al.	2009	Acta Geologica Sinica-English Edition	83（2）	245-257
677	TH7-10	NCC	NNCC	127.0000	42.0000	Rhyolite		219	219	5.41	30.00	0.1090	0.512313		-0.45	-6.34	-4.49	-3.89	-3.89	1.00	1.22	1.31	1.00		Yu Y et al.	2009	Acta Geologica Sinica-English Edition	83（2）	245-257
678	LJ21-1	NCC	NNCC	127.0000	42.0000	Rhyolite		123	150	6.03	36.79	0.0991	0.512135		-0.50	-9.81	-8.38	-7.94	-7.94	1.35	1.35	1.59	1.35		Yu Y et al.	2009	Acta Geologica Sinica-English Edition	83（2）	245-257
679	LJ17-1	NCC	NNCC	127.0000	42.0000	Rhyolite		123	150	2.76	17.77	0.0939	0.512392		-0.52	-4.80	-3.29	-2.83	-2.83	0.96	0.96	1.17	0.96		Yu Y et al.	2009	Acta Geologica Sinica-English Edition	83（2）	245-257
680	LJ16-1	NCC	NNCC	127.0000	42.0000	Rhyolite		123	150	3.68	22.52	0.0988	0.512383		-0.50	-4.97	-3.54	-3.10	-3.10	1.02	1.02	1.19	1.02		Yu Y et al.	2009	Acta Geologica Sinica-English Edition	83（2）	245-257
681	EHW02	CAOB	East Tianshan	94.2000	42.1333	Diorite		274	274	5.34	22.40	0.1440	0.512794		-0.27	3.04	4.90	4.89	4.89	0.64	0.78	0.64			Li DD et al.	2012	Geological Reviev	58(06)	1145-1160
682	SF03-3	CAOB	NE China	131.0478	44.3608	Andesite		106	150	3.07	18.71	0.0992	0.512639		-0.50	0.02	1.26	1.89	1.89	0.68	0.68	0.79	0.68		Ji W Q et al.	2007	Acta Geologica Sinica-English Edition	81（2）	266-277
683	SF03-2	CAOB	NE China	131.0478	44.3608	Andesite		106	150	3.09	18.40	0.1015	0.512616		-0.48	-0.43	0.79	1.39	1.39	0.73	0.73	0.83	0.73		Ji W Q et al.	2007	Acta Geologica Sinica-English Edition	81（2）	266-277
684	SF03-1	CAOB	NE China	131.0478	44.3608	Andesite		106	150	2.95	17.82	0.1001	0.512626		-0.49	-0.23	1.01	1.62	1.62	0.70	0.70	0.81	0.70		Ji W Q et al.	2007	Acta Geologica Sinica-English Edition	81（2）	266-277

685	SF01-5	CAOB	NE China	131.0478	44.3608	Dacite		106	150	2.61	15.88	0.0994	0.512596		-0.49	-0.82	0.39	1.04	1.04	0.76	0.74	0.85	0.76		Ji W Q et al.	2007	Acta Geologica Sinica-English Edition	81（2）	266-277
686	SF01-4	CAOB	NE China	131.0478	44.3608	Dacite		106	150	2.80	16.69	0.1014	0.512619		-0.48	-0.37	0.85	1.45	1.45	0.72	0.72	0.82	0.72		Ji W Q et al.	2007	Acta Geologica Sinica-English Edition	81（2）	266-277
687	SF01-1	CAOB	NE China	131.0478	44.3608	Dacite		106	150	2.73	16.22	0.1018	0.512603		-0.48	-0.68	0.53	1.13	1.13	0.75	0.75	0.85	0.75		Ji W Q et al.	2007	Acta Geologica Sinica-English Edition	81（2）	266-277
688	WL-94	CAOB	SMXAB	117.5000	44.0833	Biotitic monzonite-granite	Weilasituo	323	323	2.91	24.62	0.0716	0.512153	5	-0.64	-9.46	-4.30	-4.30	-4.30	1.43	1.07	1.43	1.07	1.43	Wang J.	2009	hina University of Geosciences for Master degre		1-68
689	WI-73	CAOB	SMXAB	117.5000	44.0833	Bi granite	Weilasituo	323	323	2.75	29.85	0.0557	0.512200	5	-0.72	-8.54	-2.73	-2.73	-2.73	1.30	0.92	1.30	0.92	1.31	Wang J.	2009	hina University of Geosciences for Master degre		1-68
690	WL-69	CAOB	SMXAB	117.5000	44.0833	Granodiorite	Weilasituo	298	298	9.37	37.22	0.1522	0.512475	5	-0.23	-3.18	-1.49	-1.49	-1.49	1.18	1.67	1.18	1.67	1.18	Wang J.	2009	hina University of Geosciences for Master degre		1-68
691	WL-60	CAOB	SMXAB	117.5000	44.0833	Biotitic monzonite-granite	Weilasituo	323	323	5.70	63.60	0.0542	0.512233	5	-0.72	-7.90	-2.03	-2.03	-2.03	1.25	0.88	1.25	0.88	1.25	Wang J.	2009	hina University of Geosciences for Master degre		1-68
692	WL-58	CAOB	SMXAB	117.5000	44.0833	Qtz diorite	Weilasituo	298	298	3.98	28.89	0.0833	0.512368	6	-0.58	-5.27	-0.96	-0.95	-0.95	1.14	0.91	1.14	0.92	1.14	Wang J.	2009	hina University of Geosciences for Master degre		1-68
693	WL-51	CAOB	SMXAB	117.5000	44.0833	Granodiorite	Weilasituo	298	298	2.86	18.87	0.0917	0.512479	6	-0.53	-3.10	0.89	0.89	0.89	0.99	0.84	0.99	0.84	0.99	Wang J.	2009	hina University of Geosciences for Master degre		1-68
694	WL-48	CAOB	SMXAB	117.5000	44.0833	Biotitic monzonite-granite	Weilasituo	323	323	2.24	16.81	0.0807	0.512230	6	-0.59	-7.96	-3.18	-3.18	-3.18	1.34	1.05	1.34	1.06	1.34	Wang J.	2009	hina University of Geosciences for Master degre		1-68
695	WL-46	CAOB	SMXAB	117.5000	44.0833	Diorite	Weilasituo	298	298	2.49	15.94	0.1045	0.512258	5	-0.47	-7.41	-3.91	-3.91	-3.91	1.38	1.24	1.38	1.25	1.38	Wang J.	2009	hina University of Geosciences for Master degre		1-68
696	WL-28	CAOB	SMXAB	117.5000	44.0833	Granodiorite	Weilasituo	298	298	3.60	20.83	0.1045	0.512444	7	-0.47	-3.78	-0.28	-0.28	-0.28	1.08	0.99	1.08	0.99	1.09	Wang J.	2009	hina University of Geosciences for Master degre		1-68
697	WL-183	CAOB	SMXAB	117.5000	44.0833	Diorite	Weilasituo	298	298	3.16	18.61	0.1026	0.512403	8	-0.48	-4.58	-1.00	-1.01	-1.01	1.14	1.02	1.14	1.03	1.14	Wang J.	2009	hina University of Geosciences for Master degre		1-68
698	WL-130	CAOB	SMXAB	117.5000	44.0833	Qtz diorite	Weilasituo	298	298	2.99	15.32	0.1181	0.512441	7	-0.40	-3.84	-0.84	-0.85	-0.85	1.13	1.13	1.13	1.13	1.13	Wang J.	2009	hina University of Geosciences for Master degre		1-68
699	WL-127	CAOB	SMXAB	117.5000	44.0833	Qtz diorite	Weilasituo	298	298	2.85	17.98	0.0959	0.512435	7	-0.51	-3.96	-0.12	-0.13	-0.13	1.07	0.93	1.07	0.93	1.07	Wang J.	2009	hina University of Geosciences for Master degre		1-68
700	WL-11	CAOB	SMXAB	117.5000	44.0833	Bi granite	Weilasituo	323	323	2.56	21.50	0.0719	0.512200	5	-0.63	-8.54	-3.40	-3.40	-3.40	1.36	1.02	1.36	1.02	1.36	Wang J.	2009	hina University of Geosciences for Master degre		1-68
701	WL-106	CAOB	SMXAB	117.5000	44.0833	Bi granite	Weilasituo	323	323	1.36	11.25	0.0733	0.512183	7	-0.63	-8.88	-3.79	-3.79	-3.79	1.39	1.05	1.39	1.05	1.39	Wang J.	2009	hina University of Geosciences for Master degre		1-68
702	WL-104	CAOB	SMXAB	117.5000	44.0833	Bi granite	Weilasituo	323	323	3.67	40.69	0.0545	0.512189	7	-0.72	-8.76	-2.89	-2.90	-2.90	1.32	0.92	1.32	0.92	1.32	Wang J.	2009	hina University of Geosciences for Master degre		1-68
703	WL-09	CAOB	SMXAB	117.5000	44.0833	Bi granite	Weilasituo	323	323	4.45	41.29	0.0652	0.512193	6	-0.67	-8.68	-3.26	-3.26	-3.26	1.35	0.98	1.35	0.98	1.35	Wang J.	2009	hina University of Geosciences for Master degre		1-68
704	WL-08	CAOB	SMXAB	117.5000	44.0833	Bi granite	Weilasituo	323	323	0.58	4.66	0.0748	0.512117	6	-0.62	-10.16	-5.14	-5.14	-5.14	1.50	1.13	1.50	1.14	1.50	Wang J.	2009	hina University of Geosciences for Master degre		1-68
705	WL-04	CAOB	SMXAB	117.5000	44.0833	Granite	Weilasituo	323	323	2.36	23.63	0.0603	0.512190	9	-0.69	-8.74	-3.12	-3.12	-3.12	1.33	0.95	1.33	0.96	1.34	Wang J.	2009	hina University of Geosciences for Master degre		1-68
706	Yaojiazhuang	NCC	NCC	114.5778	40.3411	Syenite	Yaojiazhuang	236	236	14.27	88.24	0.0978	0.511989	6	-0.50	-12.66	-9.68	-9.68	-9.68	1.80	1.52	1.80	1.53	1.80	Yan GH et al.	2000	Science in China	30(4)	383-387
707	Sungezhuang	NCC	NCC	117.5989	40.1422	Syenite	Sungezhuang	240	240	6.89	55.04	0.0757	0.512169	6	-0.62	-9.15	-5.45	-5.44	-5.44	1.46	1.08	1.46	1.09	1.46	Yan GH et al.	2000	Science in China	30(4)	383-387
708	Qiongcheng	NCC	NCC	112.5211	40.5481	Syenite	Qiongcheng	190	190	2.48	43.36	0.0346	0.511869	33	-0.82	-15.00	-11.1	-11.1	-11.1	1.9	1.09	1.88	1.09	1.87	Yan GH et al.	2000	Science in China	30(4)	383-387
709	Huangheshao	NCC	NCC	111.1219	41.5644	Syenite	Huangheshao	203	203	0.19	1.21	0.0934	0.511496	30	-0.53	-22.28	-19.6	-19.6	-19.6	2.6	2.09	2.57	2.09	2.57	Yan GH et al.	2000	Science in China	30(4)	383-387
710	Hekanzi	NCC	NCC	119.2400	40.8919	Syenite	Hekanzi	234	234	7.65	45.82	0.1010	0.512176	6	-0.49	-9.01	-6.16	-6.15	-6.15	1.51	1.32	1.51	1.32	1.51	Yan GH et al.	2000	Science in China	30(4)	383-387
711	Fanshan	NCC	NCC	115.4306	40.1586	Syenite	Fanshan	218	218	32.94	17.90	0.1159	0.512230	18	-0.41	-7.96	-5.71	-5.71	-5.71	1.46	1.43	1.46	1.44	1.46	Yan GH et al.	2000	Science in China	30(4)	383-387
712	Dongcheng	NCC	NCC	114.4117	40.5622	Syenite	Dongcheng	268	268	8.48	74.74	0.0686	0.512249	7	-0.65	-7.59	-3.20	-3.21	-3.21	1.30	0.95	1.30	0.95	1.30	Yan GH et al.	2000	Science in China	30(4)	383-387
713	Baotoudong	NCC	NCC	110.0211	40.7097	Syenite	East Baotou	197	197	5.54	31.36	0.1069	0.512183	22	-0.46	-8.88	-6.62	-6.62	-6.62	1.52	1.38	1.52	1.38	1.52	Yan GH et al.	2000	Science in China	30(4)	383-387
714	MX-19-1	CAOB	Altai, unit 7	95.3250	44.2000	Bi granite	Mengxi Mining Area	430	430	2.43	12.19	0.1204	0.512801		-0.39	3.18	7.40	7.37	7.37	0.57	0.57	0.57	0.57	0.57	Du SJ et al.	2010	Acta Petrologica Sinica	26(10)	2981-2996
715	MX-18-1	CAOB	Altai, unit 7	95.3250	44.2000	Alkali feldspar granite	Mengxi Mining Area	410	410	2.85	14.19	0.1216	0.512749		-0.38	2.17	6.10	6.09	6.09	0.65	0.66	0.65	0.67	0.68	Du SJ et al.	2010	Acta Petrologica Sinica	26(10)	2981-2996
716	MX-14-1	CAOB	Altai, unit 7	95.3250	44.2000	Monzogranite	Mengxi Mining Area	410	410	1.88	7.99	0.1426	0.512834		-0.28	3.82	6.70	6.65	6.65	0.61	0.68	0.61	0.68	0.68	Du SJ et al.	2010	Acta Petrologica Sinica	26(10)	2981-2996
717	HES2-1	CAOB	Altai, unit 7	95.2333	43.8000	Granodiorite porphyry	Hersai	410	410	2.14	10.46	0.1238	0.512772		-0.37	2.61	6.40	6.43	6.43	0.63	0.64	0.63	0.64	0.65	Du SJ et al.	2010	Acta Petrologica Sinica	26(10)	2981-2996
718	D11-1	CAOB	Altai, unit 7	95.2333	43.8000	Potash feldspar granite	Hersai	410	410	2.20	11.20	0.1190	0.512782		-0.39	2.81	6.90	6.87	6.87	0.59	0.59	0.59	0.60	0.60	Du SJ et al.	2010	Acta Petrologica Sinica	26(10)	2981-2996
719	D1-1	CAOB	Altai, unit 7	95.2333	43.8000	Granodiorite	Hersai	430	430	2.15	9.82	0.1321	0.512795		-0.33	3.06	6.60	6.61	6.61	0.63	0.66	0.63	0.67	0.67	Du SJ et al.	2010	Acta Petrologica Sinica	26(10)	2981-2996
720	ZK9701-21	CAOB	SMXAB	118.3333	46.0167	Porphyritic granite	Diyanqinamu	156	156	4.74	26.80	0.1065	0.512733	6	-0.46	1.85	3.64	3.65	3.65	0.65	0.59	0.65	0.60	0.65	Sun H R et al.	2014	Journal of Asian Earth Sciences	88	85-97
721	ZK9701-20	CAOB	SMXAB	118.3333	46.0167	Porphyritic granite	Diyanqinamu	156	156	3.95	23.50	0.1015	0.512734	8	-0.48	1.87	3.76	3.77	3.77	0.64	0.57	0.64	0.57	0.64	Sun H R et al.	2014	Journal of Asian Earth Sciences	88	85-97
722	ZK9701-18	CAOB	SMXAB	118.3333	46.0167	Porphyritic granite	Diyanqinamu	156	156	3.08	19.00	0.0978	0.512724	6	-0.50	1.68	3.64	3.65	3.65	0.65	0.56	0.65	0.56	0.65	Sun H R et al.	2014	Journal of Asian Earth Sciences	88	85-97
723	ZK9701-17	CAOB	SMXAB	118.3333	46.0167	Porphyritic granite	Diyanqinamu	156	156	3.67	21.60	0.1024	0.512725	4	-0.48	1.70	3.57	3.57	3.57	0.65	0.58	0.65	0.59	0.65	Sun H R et al.	2014	Journal of Asian Earth Sciences	88	85-97
724	ZK9701-16	CAOB	SMXAB	118.3333	46.0167	Porphyritic granite	Diyanqinamu	156	156	3.78	22.60	0.1006	0.512711	12	-0.49	1.42	3.33	3.34	3.34	0.67	0.59	0.67	0.59	0.67	Sun H R et al.	2014	Journal of Asian Earth Sciences	88	85-97
725	ZKp2104-5	CAOB	SMXAB	118.2333	46.0333	Aplitic grantie	Diyanqinamu	156	156	1.20	7.66	0.0942	0.512698	10	-0.52	1.17	3.22	3.21	3.21	0.68	0.58	0.68	0.58	0.68	Sun H R et al.	2014	Journal of Asian Earth Sciences	88	85-97
726	ZKp2104-4	CAOB	SMXAB	118.2333	46.0333	Aplitic grantie	Diyanqinamu	156	156	1.50	9.56	0.0946	0.512699	10	-0.52	1.19	3.22	3.22	3.22	0.68	0.58	0.68	0.58	0.68	Sun H R et al.	2014	Journal of Asian Earth Sciences	88	85-97
727	ZKp2104-3	CAOB	SMXAB	118.2333	46.0333	Aplitic grantie	Diyanqinamu	156	156	1.10	7.37	0.0899	0.512650	6	-0.54	0.23	2.36	2.36	2.36	0.75	0.62	0.75	0.62	0.75	Sun H R et al.	2014	Journal of Asian Earth Sciences	88	85-97
728	ZKp2104-2	CAOB	SMXAB	118.2333	46.0333	Aplitic grantie	Diyanqinamu	156	156	0.96	5.97	0.0970	0.512670	10	-0.51	0.62	2.61	2.61	2.61	0.73	0.63	0.73	0.63	0.73	Sun H R et al.	2014	Journal of Asian Earth Sciences	88	85-97
729	c	CAOB	Western Junggar	82.4750	45.7875	Granitoid		295	295	2.44	11.06	0.1334	0.512822		-0.32	3.59	6.00	5.97	5.97	0.57	0.62	0.57		0.57	Liu B et al.	2017	Tectonics	36(11-12)	2823–2845
730	14ALT-84	CAOB	Western Junggar	86.6083	47.6583	Granite		394	394	4.87	24.00	0.1227	0.512820	0	-0.38	3.55	7.30	7.27	7.27	0.55	0.55	0.55		0.56	Liu B et al.	2017	Tectonics	36(11-12)	2823–2845
731	14ALT-78	CAOB	Western Junggar	86.6083	47.6583	Andesite		362	362	3.76	18.10	0.1256	0.512823	0	-0.36	3.61	7.20	6.89	6.89	0.55	0.57	0.55		0.57	Liu B et al.	2017	Tectonics	36(11-12)	2823–2845
732	14ALT-77	CAOB	Western Junggar	86.6083	47.6583	Andesite		392	392	3.48	15.30	0.1375	0.512816	0	-0.30	3.47	6.40	6.43	6.43	0.61	0.67	0.61		0.67	Liu B et al.	2017	Tectonics	36(11-12)	2823–2845
733	14ALT-44-1	CAOB	Western Junggar	86.6083	47.6583	Monzonite		335	335	3.74	18.70	0.1209	0.512792	0	-0.39	3.00	6.30	6.24	6.24	0.58	0.59	0.58		0.59	Liu B et al.	2017	Tectonics	36(11-12)	2823–2845
734	14ALT-39	CAOB	Western Junggar	86.6083	47.6583	Trachydacite		326	326	5.77	28.80	0.1211	0.512752	0	-0.38	2.22	5.40	5.37	5.37	0.65	0.66	0.65		0.66	Liu B et al.	2017	Tectonics	36(11-12)	2823–2845
735	14ALT-38	CAOB	Western Junggar	86.6083	47.6583	Granite		403	403	5.28	25.70	0.1242	0.512784	0	-0.37	2.85	6.60	6.58	6.58	0.61	0.62	0.61		0.63	Liu B et al.	2017	Tectonics	36(11-12)	2823–2845
736	KL08-4	CAOB	Junggar	84.8406	45.6736	Granite		270	270	3.24	14.30	0.1370	0.512888		-0.30	4.88	6.94	6.93	6.93	0.47	0.52	0.47	0.52		Zhang C L et al.	2013	Lithos	174	15-27
737	KL08-2	CAOB	Junggar	84.8406	45.6736	Granite		270	270	1.99	8.05	0.1495	0.512714		-0.24	1.48	3.11	3.11	3.11	0.78	1.03	0.78	1.04		Zhang C L et al.	2013	Lithos	174	15-27
738	KL04-3	CAOB	Junggar	84.8000	45.7000	Granite		270	270	5.00	22.25	0.1359	0.512933		-0.31	5.75	7.86	7.85	7.85	0.40	0.43	0.40	0.43		Zhang C L et al.	2013	Lithos	174	15-27
739	ZHU-4	CAOB	Altai	89.1000	49.7000	Rhyolite		370	370	5.24	24.40	0.1300	0.512453		-0.34	-3.61	-0.49	-0.46	-0.46	1.16	1.27	1.16	1.27		Vrublevsky VV et al.	2007	Doklady Earth Sciences	416（1）	1090-1095
740	ZHU-1	CAOB	Altai	89.1000	49.7000	Dacite		370	370	11.50	53.70	0.1295	0.512445		-0.34	-3.76	-0.63	-0.59	-0.59	1.17	1.27	1.17	1.28		Vrublevsky VV et al.	2007	Doklady Earth Sciences	416（1）	1090-1095
741	UI-7	CAOB	Altai	89.1000	49.7000	Andesite		370	370	8.16	36.60	0.1348	0.512409		-0.31	-4.47	-1.54	-1.54	-1.54	1.24	1.43	1.24	1.43		Vrublevsky VV et al.	2007	Doklady Earth Sciences	416（1）	1090-1095
742	OYU-5	CAOB	Gorny Altai	89.1000	49.7000	Basaltic trachyandesite	Aksai Complex	370	370	6.82	37.7	0.1094	0.51251		-0.44	-2.50	1.58	1.62	1.64	0.99	0.94	0.99	0.94		Vrublevsky V V et al.	2007	Doklady Earth Sciences	416(7)	1090-1095
743	UI-1	CAOB	Gorny Altai	89.1000	49.7000	Trachyandesite	Aksai Complex	370	370	6.92	30.3	0.1382	0.512517		-0.30	-2.36	0.37	0.40	0.42	1.09	1.28	1.09	1.28		Vrublevsky V V et al.	2007	Doklady Earth Sciences	416(7)	1090-1095
744	BSH-3	CAOB	Gorny Altai	89.1000	49.7000	Trachyrhyolite	Aksai Complex	370	370	7.12	30.8	0.1395	0.512524		-0.29	-2.22	0.45	0.48	0.48	1.08	1.28	1.08	1.28		Vrublevsky V V et al.	2007	Doklady Earth Sciences	416(7)	1090-1095
745	503	CAOB	Gorny Altai	89.1000	49.7000	Basaltic trachyandesite	Aksai Complex	370	370	10.95	44.2	0.1498	0.512523		-0.24	-2.24	-0.04	-0.03	-0.02	1.12	1.49	1.12	1.49		Vrublevsky V V et al.	2007	Doklady Earth Sciences	416(7)	1090-1095
746	ZKH-5023	CAOB	Khangai zonal magmatic area	101.0500	47.6100	Granite		260	260	4.37	25.30	0.1048	0.512393		-0.47	-4.78	-1.70	-1.73	-1.73	1.17	1.06	1.17		1.19	Yarmolyuk VV et al.	2016	Petrology	24（5）	433-461
747	TT-21	CAOB	Khangai zonal magmatic area	101.3000	45.9500	Granite		260	260	5.06	32.70	0.0936	0.512302		-0.52	-6.55	-3.10	-3.13	-3.13	1.28	1.08	1.28		1.31	Yarmolyuk VV et al.	2016	Petrology	24（5）	433-461
748	TSTS-4455	CAOB	Khangai zonal magmatic area	101.0500	47.6100	Granite		260	260	5.12	28.80	0.1081	0.512399		-0.45	-4.66	-1.70	-1.72	-1.72	1.17	1.08	1.17		1.19	Yarmolyuk VV et al.	2016	Petrology	24（5）	433-461
749	TSTS-4454	CAOB	Khangai zonal magmatic area	101.3400	47.5600	Granite		260	260	6.03	35.20	0.1039	0.512406		-0.47	-4.53	-1.40	-1.45	-1.45	1.15	1.03	1.15		1.17	Yarmolyuk VV et al.	2016	Petrology	24（5）	433-461
750	KHAR-4487	CAOB	Khangai zonal magmatic area	102.7200	46.8600	Granite		260	260	4.82	27.40	0.1068	0.512461		-0.46	-3.45	-0.50	-0.47	-0.47	1.07	0.98	1.07		1.09	Yarmolyuk VV et al.	2016	Petrology	24（5）	433-461
751	KHAN-11/18	CAOB	Khangai zonal magmatic area	99.3260	47.9900	Rhyolite		800	600	10.40	48.50	0.1293	0.512246		-0.34	-7.65	-5.40	-2.49	-2.49	1.51	1.63	1.51		1.55	Yarmolyuk VV et al.	2016	Petrology	24（5）	433-461
752	KHAN-11/13	CAOB	Khangai zonal magmatic area	99.3674	47.7929	Granite		260	260	5.13	31.30	0.0993	0.512486		-0.50	-2.97	0.30	0.27	0.27	1.01	0.88	1.01		1.03	Yarmolyuk VV et al.	2016	Petrology	24（5）	433-461
753	KHAN-09/49	CAOB	Khangai zonal magmatic area	98.9100	48.0830	Granite		260	260	1.95	10.90	0.1084	0.512249		-0.45	-7.59	-4.70	-4.66	-4.66	1.41	1.30	1.41		1.44	Yarmolyuk VV et al.	2016	Petrology	24（5）	433-461
754	KHAN-09/47	CAOB	Khangai zonal magmatic area	100.6972	47.3671	Granite		260	260	4.53	24.80	0.1104	0.512519		-0.44	-2.32	0.50	0.54	0.54	0.98	0.93	0.98		1.00	Yarmolyuk VV et al.	2016	Petrology	24（5）	433-461
755	KHAN-09/46	CAOB	Khangai zonal magmatic area	101.7575	46.3236	Granite		260	260	3.46	18.80	0.1112	0.512396		-0.43	-4.72	-1.90	-1.88	-1.88	1.18	1.12	1.18		1.20	Yarmolyuk VV et al.	2016	Petrology	24（5）	433-461
756	KHAN-09/42	CAOB	Khangai zonal magmatic area	98.2192	46.6983	Granite		260	260	1.29	9.30	0.0837	0.512162		-0.57	-9.29	-5.50	-5.54	-5.54	1.48	1.16	1.48		1.51	Yarmolyuk VV et al.	2016	Petrology	24（5）	433-461
757	KHAN-09/41	CAOB	Khangai zonal magmatic area	97.6820	47.3590	Granite		260	260	16.60	129.00	0.0778	0.512359		-0.60	-5.44	-1.50	-1.50	-1.50	1.15	0.89	1.15		1.17	Yarmolyuk VV et al.	2016	Petrology	24（5）	433-461
758	KHAN-09/40	CAOB	Khangai zonal magmatic area	97.7634	47.4699	Granite		260	260	3.25	17.40	0.1133	0.512327		-0.42	-6.07	-3.30	-3.30	-3.30	1.30	1.25	1.30		1.32	Yarmolyuk VV et al.	2016	Petrology	24（5）	433-461
759	KHAN-09/39	CAOB	Khangai zonal magmatic area	97.8400	47.5300	Granite		260	260	5.55	18.70	0.1796	0.512493		-0.09	-2.83	-2.30	-2.26	-2.26	1.21	2.92	1.21		1.24	Yarmolyuk VV et al.	2016	Petrology	24（5）	433-461
760	KHAN-09/35	CAOB	Khangai zonal magmatic area	96.8349	47.4222	Granite		260	260	4.67	27.10	0.1044	0.512348		-0.51	-5.66	-2.60	-2.32	-2.32	1.22	1.04	1.22		1.26	Yarmolyuk VV et al.	2016	Petrology	24（5）	433-461
761	KHAN-09/33	CAOB	Khangai zonal magmatic area	96.5570	47.7540	Granite		260	260	3.70	23.30	0.0960	0.512482		-0.52	-3.04	0.30	0.34	0.34	1.00	0.86	1.00		1.02	Yarmolyuk VV et al.	2016	Petrology	24（5）	433-461
762	KHAN-09/30	CAOB	Khangai zonal magmatic area	96.2710	47.9250	Granite		260	260	2.97	18.90	0.0947	0.512427		-0.48	-4.12	-0.70	-0.99	-0.99	1.11	0.99	1.11		1.11	Yarmolyuk VV et al.	2016	Petrology	24（5）	433-461
763	KHAN-08/8	CAOB	Khangai zonal magmatic area	96.8060	47.2430	Granite		260	260	9.46	55.80	0.1025	0.512340		-0.48	-5.81	-2.70	-2.66	-2.66	1.25	1.10	1.25		1.27	Yarmolyuk VV et al.	2016	Petrology	24（5）	433-461
764	KHAN-08/7	CAOB	Khangai zonal magmatic area	96.7980	47.2150	Granite		260	260	10.40	61.90	0.1016	0.512361		-0.48	-5.40	-2.20	-2.27	-2.27	1.21	1.08	1.21		1.23	Yarmolyuk VV et al.	2016	Petrology	24（5）	433-461
765	KHAN-08/34	CAOB	Khangai zonal magmatic area	96.6540	47.9160	Granite		260	260	4.16	24.60	0.1022	0.512451		-0.54	-3.65	-0.50	-0.14	-0.14	1.04	0.87	1.04		1.09	Yarmolyuk VV et al.	2016	Petrology	24（5）	433-461
766	KHAN-08/24	CAOB	Khangai zonal magmatic area	100.5830	48.1670	Granite		260	260	4.87	32.20	0.0910	0.512337		-0.48	-5.87	-2.40	-2.72	-2.72	1.25	1.11	1.25		1.24	Yarmolyuk VV et al.	2016	Petrology	24（5）	433-461
767	KHAN-08/20	CAOB	Khangai zonal magmatic area	101.0830	48.1720	Granite		260	260	4.02	23.90	0.1017	0.512420		-0.52	-4.25	-1.10	-0.87	-0.87	1.10	0.93	1.10		1.14	Yarmolyuk VV et al.	2016	Petrology	24（5）	433-461
768	KHAN-08/2	CAOB	Khangai zonal magmatic area	96.7300	46.9770	Granite		260	260	3.41	21.80	0.0946	0.512228		-0.55	-8.00	-4.60	-4.40	-4.40	1.39	1.12	1.39		1.43	Yarmolyuk VV et al.	2016	Petrology	24（5）	433-461
769	KHAN-08/17	CAOB	Khangai zonal magmatic area	101.9400	46.7380	Granite		260	260	6.49	44.40	0.0883	0.512423		-0.43	-4.19	-0.60	-1.39	-1.39	1.14	1.09	1.14		1.10	Yarmolyuk VV et al.	2016	Petrology	24（5）	433-461
770	KHAN-08/15	CAOB	Khangai zonal magmatic area	102.1550	46.8670	Granite		260	260	7.71	41.50	0.1123	0.512549		-0.48	-1.74	1.10	1.37	1.37	0.92	0.83	0.92		0.96	Yarmolyuk VV et al.	2016	Petrology	24（5）	433-461
771	KHAN-08/13	CAOB	Khangai zonal magmatic area	96.8700	47.3980	Granite		260	260	5.11	30.00	0.1032	0.512341		-0.48	-5.79	-2.70	-2.68	-2.68	1.25	1.11	1.25		1.27	Yarmolyuk VV et al.	2016	Petrology	24（5）	433-461
772	KHAN-08/10	CAOB	Khangai zonal magmatic area	96.7700	47.3160	Granite		260	260	7.04	41.40	0.1028	0.512361		-0.43	-5.40	-2.30	-2.61	-2.61	1.24	1.19	1.24		1.24	Yarmolyuk VV et al.	2016	Petrology	24（5）	433-461
773	KHAN-08/1	CAOB	Khangai zonal magmatic area	96.6530	46.9160	Granite		260	260	8.34	44.80	0.1124	0.511851		-0.40	-15.35	-12.6	-12.7	-12.7	2.06	2.05	2.06		2.09	Yarmolyuk VV et al.	2016	Petrology	24（5）	433-461
774	KHAN-07/8	CAOB	Khangai zonal magmatic area	98.4388	46.4674	Granite		260	260	3.38	17.40	0.1175	0.512471		-0.41	-3.26	-0.60	-0.61	-0.61	1.08	1.07	1.08		1.10	Yarmolyuk VV et al.	2016	Petrology	24（5）	433-461
775	KHAN-07/6	CAOB	Khangai zonal magmatic area	98.3790	46.1040	Granite		260	260	6.14	31.80	0.1169	0.512520		-0.49	-2.30	0.30	0.90	0.90	0.96	0.85	0.96		1.02	Yarmolyuk VV et al.	2016	Petrology	24（5）	433-461
776	KHAN-07/27	CAOB	Khangai zonal magmatic area	97.2074	48.1658	Granite		260	260	4.43	26.70	0.1003	0.512553		-0.55	-1.66	1.50	1.93	1.93	0.87	0.73	0.87		0.92	Yarmolyuk VV et al.	2016	Petrology	24（5）	433-461
777	KHAN-07/20	CAOB	Khangai zonal magmatic area	97.1650	48.0830	Granite		260	260	1.71	11.60	0.0887	0.512358		-0.55	-5.46	-1.90	-1.87	-1.87	1.18	0.96	1.18		1.20	Yarmolyuk VV et al.	2016	Petrology	24（5）	433-461
778	KHAN-07/14	CAOB	Khangai zonal magmatic area	102.4030	46.0210	Granite		260	260	3.32	18.20	0.1107	0.512422		-0.48	-4.21	-1.40	-1.10	-1.10	1.12	1.00	1.12		1.16	Yarmolyuk VV et al.	2016	Petrology	24（5）	433-461
779	KHAN-07/10	CAOB	Khangai zonal magmatic area	98.8610	46.3360	Granite		260	260	3.38	23.10	0.0883	0.512036		-0.45	-11.74	-8.10	-8.81	-8.81	1.74	1.61	1.74		1.72	Yarmolyuk VV et al.	2016	Petrology	24（5）	433-461
780	KHAN-07/1	CAOB	Khangai zonal magmatic area	97.8820	46.0960	Granite		260	260	3.39	19.90	0.1028	0.512395		-0.49	-4.74	-1.60	-1.52	-1.52	1.15	1.01	1.15		1.18	Yarmolyuk VV et al.	2016	Petrology	24（5）	433-461
781	KHAN-06/6	CAOB	Khangai zonal magmatic area	99.4689	48.1235	Granite		260	260	9.01	50.20	0.1084	0.512149		-0.43	-9.54	-6.60	-6.73	-6.73	1.58	1.50	1.58		1.60	Yarmolyuk VV et al.	2016	Petrology	24（5）	433-461
782	KHAN-06/48	CAOB	Khangai zonal magmatic area	98.5890	48.7260	Granite		260	260	2.04	12.40	0.0997	0.512371		-0.40	-5.21	-2.00	-2.58	-2.58	1.24	1.23	1.24		1.21	Yarmolyuk VV et al.	2016	Petrology	24（5）	433-461

783	KHAN-06/45	CAOB	Khangai zonal magmatic area	99.3800	48.7660	Gneiss		800	600	9.13	49.30	0.1119	0.512138		-0.48	-9.75	-6.90	-2.48	-2.48	1.51	1.38	1.51		1.58	Yarmolyuk VV et al.	2016	Petrology	24（5）	433-461
784	KHAN-06/43	CAOB	Khangai zonal magmatic area	99.4070	48.8250	Gneiss		800	600	4.78	24.60	0.1175	0.512202		-0.44	-8.51	-5.90	-1.90	-1.90	1.46	1.40	1.46		1.52	Yarmolyuk VV et al.	2016	Petrology	24（5）	433-461
785	KHAN-06/42	CAOB	Khangai zonal magmatic area	99.4340	48.8580	Granite		260	260	2.82	16.80	0.1017	0.512348		-0.45	-5.66	-2.50	-2.73	-2.73	1.25	1.16	1.25		1.26	Yarmolyuk VV et al.	2016	Petrology	24（5）	433-461
786	KHAN-06/41	CAOB	Khangai zonal magmatic area	99.4510	48.8690	Granite		260	260	4.00	21.90	0.1104	0.512207		-0.52	-8.41	-5.50	-5.01	-5.01	1.44	1.20	1.44		1.51	Yarmolyuk VV et al.	2016	Petrology	24（5）	433-461
787	KHAN-06/27	CAOB	Khangai zonal magmatic area	99.3170	48.5940	Granite		260	260	4.53	25.30	0.1085	0.512122		-0.51	-10.07	-7.10	-6.71	-6.71	1.57	1.32	1.57		1.64	Yarmolyuk VV et al.	2016	Petrology	24（5）	433-461
788	KHAN-06/25	CAOB	Khangai zonal magmatic area	99.3660	48.5540	Granite		260	260	11.00	70.40	0.0943	0.512205		-0.55	-8.45	-5.10	-4.85	-4.85	1.42	1.15	1.42		1.47	Yarmolyuk VV et al.	2016	Petrology	24（5）	433-461
789	KHAN-06/23	CAOB	Khangai zonal magmatic area	99.3681	48.4837	Granite		260	260	11.60	73.50	0.0955	0.512213		-0.44	-8.29	-4.90	-5.44	-5.44	1.47	1.38	1.47		1.46	Yarmolyuk VV et al.	2016	Petrology	24（5）	433-461
790	KHAN-06/19	CAOB	Khangai zonal magmatic area	99.4447	48.3946	Granite		260	260	2.31	15.80	0.0884	0.511681		-0.50	-18.67	-15.1	-15.4	-15.4	2.28	1.95	2.28		2.30	Yarmolyuk VV et al.	2016	Petrology	24（5）	433-461
791	KHAN-06/16	CAOB	Khangai zonal magmatic area	99.3866	48.2839	Granite		260	260	3.19	17.40	0.1107	0.512407		-0.46	-4.51	-1.70	-1.48	-1.48	1.15	1.05	1.15		1.19	Yarmolyuk VV et al.	2016	Petrology	24（5）	433-461
792	KHAN-06/14	CAOB	Khangai zonal magmatic area	99.4300	48.1860	Granite		260	260	3.10	18.90	0.0993	0.512280		-0.22	-6.98	-3.80	-5.55	-5.55	1.48	2.20	1.48		1.36	Yarmolyuk VV et al.	2016	Petrology	24（5）	433-461
793	KHAN-06/1	CAOB	Khangai zonal magmatic area	99.8350	47.1903	Granite		260	260	11.60	66.60	0.1055	0.512407		-0.53	-4.51	-1.50	-1.08	-1.08	1.12	0.94	1.12		1.17	Yarmolyuk VV et al.	2016	Petrology	24（5）	433-461
794	DZ-3/1	CAOB	Khangai zonal magmatic area	96.5700	46.8840	Granite		755	600	6.59	25.90	0.1536	0.512179		-0.39	-8.95	-7.50	-3.14	-3.14	1.56	1.59	1.56		1.85	Yarmolyuk VV et al.	2016	Petrology	24（5）	433-461
795	7029	CAOB	Khangai zonal magmatic area	98.7860	48.1220	Gneiss		800	600	6.52	42.20	0.0934	0.511824		-0.54	-15.88	-12.50	-7.74	-7.74	1.93	1.64	1.93		1.93	Yarmolyuk VV et al.	2016	Petrology	24（5）	433-461
796	7028	CAOB	Khangai zonal magmatic area	98.8550	48.0780	Gneiss		800	600	6.25	31.30	0.1208	0.512029		-0.51	-11.88	-9.40	-4.24	-4.24	1.65	1.46	1.65		1.83	Yarmolyuk VV et al.	2016	Petrology	24（5）	433-461
797	7026	CAOB	Khangai zonal magmatic area	99.0510	48.1120	Gneiss		800	600	3.89	26.00	0.0904	0.511952		-0.48	-13.38	-9.90	-6.17	-6.17	1.81	1.64	1.81		1.70	Yarmolyuk VV et al.	2016	Petrology	24（5）	433-461
798	1716	CAOB	Khangai zonal magmatic area	102.0700	46.0800	Granite		260	260	8.39	52.50	0.0969	0.512294		-0.06	-6.71	-3.40	-6.34	-6.34	1.53	4.59	1.53		1.33	Yarmolyuk VV et al.	2016	Petrology	24（5）	433-461
799	1715	CAOB	Khangai zonal magmatic area	101.4200	47.9500	Granite		260	260	6.04	35.80	0.1025	0.512394		-0.18	-4.76	-1.60	-3.59	-3.59	1.32	2.19	1.32		1.18	Yarmolyuk VV et al.	2016	Petrology	24（5）	433-461
800	CH-4931/10	CAOB	Lake zone	92.7500	48.4500	Basalt andesite	Seri	570	570	3.15	10.26	0.1856	0.513043		-0.06	7.90	8.70	8.71	8.71	0.57	0.58	0.57			Kovach VP et al.	2011	Petrology	19(4)	399-425
801	PM-72-06	CAOB	Lake zone	93.1000	48.3000	Granitoids		529	529	1.66	6.23	0.1614	0.512921		-0.18	5.52	7.90	7.90	7.90	0.60	0.67	0.60			Kovach VP et al.	2011	Petrology	19(4)	399-425
802	PM-7-05	CAOB	Lake zone	93.7000	49.0000	Granitoids		494	494	3.52	14.18	0.1501	0.512823		-0.24	3.61	6.60	6.55	6.55	0.69	0.78	0.69			Kovach VP et al.	2011	Petrology	19(4)	399-425
803	PM-51-05	CAOB	Lake zone	93.1000	48.3000	Granitoids		459	459	2.79	15.46	0.1091	0.512640		-0.45	0.04	5.20	5.17	5.17	0.77	0.74	0.77	0.75	0.78	Kovach VP et al.	2011	Petrology	19(4)	399-425
804	PM-46-05	CAOB	Lake zone	93.1000	48.3000	Granitoids		531	531	0.70	3.40	0.1243	0.512768		-0.37	2.54	7.40	7.44	7.44	0.64	0.65	0.64	0.65		Kovach VP et al.	2011	Petrology	19(4)	399-425
805	PM-21-05	CAOB	Lake zone	93.7000	49.0000	Granitoids		519	519	2.76	13.02	0.1283	0.512742		-0.35	2.03	6.60	6.56	6.56	0.71	0.73	0.71	0.73		Kovach VP et al.	2011	Petrology	19(4)	399-425
806	PM-12-05	CAOB	Lake zone	93.7000	49.0000	Granitoids		519	519	1.83	9.52	0.1162	0.512701		-0.41	1.23	6.60	6.56	6.56	0.71	0.70	0.71	0.71		Kovach VP et al.	2011	Petrology	19(4)	399-425
807	Xp-1/7	CAOB	Lake zone	92.6000	49.0000	Basalt andesite		570	570	8.97	38.12	0.1422	0.512877		-0.28	4.66	8.60	8.63	8.63	0.58	0.58	0.58	0.59		Kovach VP et al.	2011	Petrology	19(4)	399-425
808	Xp-1/12	CAOB	Lake zone	93.0000	49.0000	Granitoids		465	465	2.74	15.71	0.1053	0.512688		-0.46	0.98	6.40	6.40	6.40	0.67	0.65	0.67	0.65		Kovach VP et al.	2011	Petrology	19(4)	399-425
809	Xp-1/10	CAOB	Lake zone	92.8000	49.0000	Granitoids		495	495	3.88	18.18	0.1291	0.512811		-0.34	3.37	7.70	7.64	7.64	0.60	0.61	0.60	0.61		Kovach VP et al.	2011	Petrology	19(4)	399-425
810	ХБУ-7/8	CAOB	Lake zone	92.0000	48.6000	Andesite		545	545	6.87	38.37	0.1073	0.512734		-0.45	1.87	8.10	8.09	8.09	0.60	0.60	0.60	0.598		Kovach VP et al.	2011	Petrology	19(4)	399-425
811	ХБУ-7/6	CAOB	Lake zone	92.0000	48.6000	Andesite		545	545	1.97	7.81	0.1059	0.512847		-0.46	4.08	7.30	10.4	10.4	0.55	0.43	0.41	0.76		Kovach VP et al.	2011	Petrology	19(4)	399-425
812	ХБУ-7/3	CAOB	Lake zone	92.0000	48.6000	Andesite		545	545	4.54	21.88	0.1254	0.512756		-0.36	2.30	7.30	7.26	7.26	0.67	0.68	0.67	0.68		Kovach VP et al.	2011	Petrology	19(4)	399-425
813	ХБУ-7/1	CAOB	Lake zone	92.0000	48.6000	Andesite		545	545	7.45	35.73	0.1260	0.512789		-0.36	2.95	7.90	7.86	7.86	0.62	0.63	0.62	0.63		Kovach VP et al.	2011	Petrology	19(4)	399-425
814	Xp-2	CAOB	Transbaikalia and Mongolia	93.9167	49.3333	Granitoid		220	220	5.51	38.00	0.0877	0.512549		-0.55	-1.74	1.32	1.32	1.32	0.89	0.73	0.89	0.73	0.89	Kovacha VP et al.	2012	Petrology	19（4）	399-425
815	Xp-1/12	CAOB	Transbaikalia and Mongolia	93.2500	48.9833	Granitoid		465	465	2.74	15.71	0.1053	0.512688		-0.46	0.98	6.40	6.40	6.40	0.67	0.65	0.67	0.65	0.67	Kovacha VP et al.	2012	Petrology	19（4）	399-425
816	Xp-1/10	CAOB	Transbaikalia and Mongolia	92.9333	49.0833	Granitoid		495	495	3.88	18.18	0.1291	0.512811		-0.34	3.37	7.64	7.64	7.64	0.60	0.61	0.60	0.61	0.60	Kovacha VP et al.	2012	Petrology	19（4）	399-425
817	ХБУ-IX	CAOB	Transbaikalia and Mongolia	91.9667	48.3167	Granitoid		490	490	7.00	28.12	0.1506	0.512891		-0.23	4.94	7.82	7.82	7.82	0.58	0.63	0.58	0.63	0.58	Kovacha VP et al.	2012	Petrology	19（4）	399-425
818	ХБУ-5006	CAOB	Transbaikalia and Mongolia	91.6667	47.9833	Granitoid		440	440	5.44	31.01	0.1063	0.512668		-0.46	0.59	5.66	5.66	5.66	0.71	0.68	0.71	0.68	0.71	Kovacha VP et al.	2012	Petrology	19（4）	399-425
819	ХБУ-5003	CAOB	Transbaikalia and Mongolia	92.0000	48.3667	Granitoid		441	441	3.01	15.90	0.1148	0.512693		-0.42	1.07	5.68	5.68	5.68	0.71	0.70	0.71	0.70	0.71	Kovacha VP et al.	2012	Petrology	19（4）	399-425
820	EX-6/3	CAOB	Transbaikalia and Mongolia	93.0000	48.3667	Granitoid		449	449	4.15	24.07	0.1042	0.512657		-0.47	0.38	5.68	5.68	5.68	0.72	0.69	0.72	0.69	0.72	Kovacha VP et al.	2012	Petrology	19（4）	399-425
821	EX-6/1	CAOB	Transbaikalia and Mongolia	93.0000	48.3500	Granitoid		514	514	2.22	10.14	0.1324	0.512884		-0.33	4.80	9.02	9.02	9.02	0.45	0.50	0.50	0.50	0.50	Kovacha VP et al.	2012	Petrology	19（4）	399-425
822	EX-3/8	CAOB	Transbaikalia and Mongolia	93.0000	48.3500	Granitoid		514	514	2.20	10.34	0.1288	0.512865		-0.35	4.43	8.88	8.88	8.88	0.51	0.51	0.51	0.51	0.51	Kovacha VP et al.	2012	Petrology	19（4）	399-425
823	EX-3/5	CAOB	Transbaikalia and Mongolia	93.0000	48.3500	Granitoid		514	514	6.78	23.61	0.1736	0.512966		-0.12	6.40	7.91	7.91	7.91	0.59	0.70	0.59	0.70	0.59	Kovacha VP et al.	2012	Petrology	19（4）	399-425
824	EX-3/2	CAOB	Transbaikalia and Mongolia	93.0000	48.3500	Granitoid		514	514	2.05	9.51	0.1300	0.512854		-0.34	4.21	8.59	8.59	8.59	0.54	0.54	0.54	0.54	0.54	Kovacha VP et al.	2012	Petrology	19（4）	399-425
825	БМХ-06/9	CAOB	Transbaikalia and Mongolia	92.8333	49.5000	Granitoid		511	511	5.43	24.26	0.1353	0.512740		-0.31	1.99	6.00	6.00	6.00	0.75	0.80	0.75	0.80	0.75	Kovacha VP et al.	2012	Petrology	19（4）	399-425
826	БМХ-04/10	CAOB	Transbaikalia and Mongolia	92.8333	49.5000	Granitoid		511	511	10.30	40.43	0.1540	0.512862		-0.22	4.37	7.15	7.15	7.15	0.65	0.74	0.65	0.74	0.65	Kovacha VP et al.	2012	Petrology	19（4）	399-425
827	Zo 47	CAOB	Transbaikalia and Mongolia	102.7667	43.5500	Metadacite		420	420	5.27	20.20	0.1576	0.512891		-0.20	4.94	7.03	7.03	7.03	0.59	0.70	0.59	0.70	0.59	Kovacha VP et al.	2012	Petrology	19（4）	399-425
828	Ts 96	CAOB	Transbaikalia and Mongolia	97.1000	45.1167	Felsic metatuffit		400	400	9.45	46.70	0.1225	0.512776		-0.38	2.69	6.48	6.48	6.48	0.62	0.63	0.62	0.63	0.62	Kovacha VP et al.	2012	Petrology	19（4）	399-425
829	Ts 94	CAOB	Transbaikalia and Mongolia	97.1000	45.1167	Metavolcaniclast.		400	400	6.73	34.10	0.1191	0.512314		-0.39	-6.32	-2.36	-2.36	-2.36	1.33	1.35	1.33	1.35	1.33	Kovacha VP et al.	2012	Petrology	19（4）	399-425
830	Ts 92	CAOB	Transbaikalia and Mongolia	97.1000	45.1167	Metaandesite		360	360	3.52	16.60	0.1278	0.512750		-0.35	2.18	5.35	5.35	5.35	0.67	0.71	0.67	0.71	0.67	Kovacha VP et al.	2012	Petrology	19（4）	399-425
831	Ts 91	CAOB	Transbaikalia and Mongolia	97.1500	45.0667	Gneiss, granitic		361	361	5.23	35.40	0.0892	0.512410		-0.55	-4.45	0.50	0.50	0.50	1.07	0.91	1.07	0.91	1.07	Kovacha VP et al.	2012	Petrology	19（4）	399-425
832	Ts 90	CAOB	Transbaikalia and Mongolia	97.1667	45.0167	Metadacite		360	360	5.41	26.20	0.1246	0.512162		-0.37	-9.29	-5.97	-5.97	-5.97	1.59	1.69	1.59	1.69	1.59	Kovacha VP et al.	2012	Petrology	19（4）	399-425
833	Ts 89	CAOB	Transbaikalia and Mongolia	97.1667	45.0333	Metagranodiorite		290	290	8.87	55.30	0.0969	0.512639		-0.51	0.02	3.71	3.71	3.71	0.75	0.67	0.75	0.67	0.75	Kovacha VP et al.	2012	Petrology	19（4）	399-425
834	Ts 113	CAOB	Transbaikalia and Mongolia	97.9667	45.4333	Granitic Gneiss		397	397	3.54	24.90	0.0860	0.512683		-0.56	0.88	6.49	6.49	6.49	0.61	0.56	0.61	0.56	0.61	Kovacha VP et al.	2012	Petrology	19（4）	399-425
835	Ts 111	CAOB	Transbaikalia and Mongolia	97.9667	45.4333	Granodioritic Gneiss		396	396	5.61	28.70	0.1181	0.512638		-0.40	0.00	3.97	3.97	3.97	0.82	0.82	0.82	0.82	0.82	Kovacha VP et al.	2012	Petrology	19（4）	399-425
836	Sh-13	CAOB	Transbaikalia and Mongolia	107.6333	44.0833	Adakitic		336	336	3.50	16.80	0.1270	0.512878		-0.35	4.68	7.67	7.67	7.67	0.47	0.48	0.47	0.48	0.47	Kovacha VP et al.	2012	Petrology	19（4）	399-425
837	Sh-0144	CAOB	Transbaikalia and Mongolia	107.7333	44.3333	Adakitic		336	336	2.70	10.20	0.1600	0.512867		-0.19	4.47	6.04	6.04	6.04	0.60	0.80	0.60	0.80	0.60	Kovacha VP et al.	2012	Petrology	19（4）	399-425
838	Sh-0143	CAOB	Transbaikalia and Mongolia	107.7333	44.3333	Adakitic		336	336	3.10	16.80	0.1120	0.512804		-0.43	3.24	6.87	6.87	6.87	0.53	0.52	0.53	0.52	0.53	Kovacha VP et al.	2012	Petrology	19（4）	399-425
839	Sh-0142	CAOB	Transbaikalia and Mongolia	107.7333	44.2167	Adakitic		336	336	3.10	17.10	0.1100	0.512844		-0.44	4.02	7.74	7.74	7.74	0.46	0.45	0.46	0.45	0.46	Kovacha VP et al.	2012	Petrology	19（4）	399-425
840	Sh-0125	CAOB	Transbaikalia and Mongolia	107.7333	44.1667	Adakitic		336	336	3.40	17.00	0.1210	0.512783		-0.38	2.83	6.07	6.07	6.07	0.60	0.60	0.60	0.60	0.60	Kovacha VP et al.	2012	Petrology	19（4）	399-425
841	Sh-0122	CAOB	Transbaikalia and Mongolia	107.6500	44.1333	Adakitic		336	336	3.40	16.60	0.1240	0.512795		-0.37	3.06	6.18	6.18	6.18	0.59	0.60	0.59	0.60	0.59	Kovacha VP et al.	2012	Petrology	19（4）	399-425
842	Sh-0117	CAOB	Transbaikalia and Mongolia	107.6333	44.0833	Adakitic		336	336	3.90	17.50	0.1350	0.512850		-0.31	4.14	6.78	6.78	6.78	0.54	0.58	0.54	0.58	0.54	Kovacha VP et al.	2012	Petrology	19（4）	399-425
843	NG 66	CAOB	Transbaikalia and Mongolia	101.5667	43.6000	Metaplagiogranite		420	420	3.27	10.10	0.1954	0.513113		-0.01	9.27	9.34	9.34	9.34	0.34	0.31	0.40	0.31	0.40	Kovacha VP et al.	2012	Petrology	19（4）	399-425
844	GS 9	CAOB	Transbaikalia and Mongolia	103.0667	43.7833	MetaGranodiorite		420	420	2.02	12.30	0.0992	0.512848		-0.50	4.10	9.32	9.32	9.32	0.42	0.40	0.40	0.40	0.40	Kovacha VP et al.	2012	Petrology	19（4）	399-425
845	GS 7	CAOB	Transbaikalia and Mongolia	103.1833	43.7667	Metarhyolite		420	420	1.58	9.25	0.1034	0.512752		-0.47	2.22	7.22	7.22	7.22	0.57	0.55	0.57	0.55	0.57	Kovacha VP et al.	2012	Petrology	19（4）	399-425
846	GS 6	CAOB	Transbaikalia and Mongolia	103.1167	43.7667	Metadacite		420	420	1.90	9.36	0.1229	0.512815		-0.38	3.45	7.41	7.41	7.41	0.56	0.56	0.56	0.56	0.56	Kovacha VP et al.	2012	Petrology	19（4）	399-425
847	BH 81	CAOB	Transbaikalia and Mongolia	100.7667	44.4333	Metagranite		420	420	5.59	29.00	0.1165	0.512509		-0.41	-2.52	1.78	1.78	1.78	1.01	1.01	1.01	1.01	1.01	Kovacha VP et al.	2012	Petrology	19（4）	399-425
848	BH 80	CAOB	Transbaikalia and Mongolia	100.7667	44.4500	Metagranite		426	426	7.19	34.70	0.1253	0.512496		-0.36	-2.77	1.11	1.11	1.11	1.07	1.13	1.07	1.13	1.07	Kovacha VP et al.	2012	Petrology	19（4）	399-425
849	BH 79	CAOB	Transbaikalia and Mongolia	100.7667	44.4500	Metarhyolite		420	420	1.87	10.60	0.1067	0.512664		-0.46	0.51	5.33	5.33	5.33	0.73	0.69	0.73	0.69	0.73	Kovacha VP et al.	2012	Petrology	19（4）	399-425
850	BH 78	CAOB	Transbaikalia and Mongolia	100.7833	44.4500	Metagranodiorite		420	420	3.38	19.00	0.1076	0.512542		-0.45	-1.87	2.90	2.90	2.90	0.92	0.87	0.92	0.87	0.92	Kovacha VP et al.	2012	Petrology	19（4）	399-425
851	BB1007	CAOB	Transbaikalia and Mongolia	107.7333	44.4167	Adakitic		336	336	1.80	10.40	0.1050	0.512807		-0.47	3.30	7.23	7.23	7.23	0.50	0.48	0.50	0.48	0.50	Kovacha VP et al.	2012	Petrology	19（4）	399-425
852	99g/7	CAOB	Transbaikalia and Mongolia	93.7167	46.9333	Granite		450	450	2.01	11.78	0.1038	0.512324		-0.47	-6.13	-0.79	-0.79	-0.79	1.25	1.14	1.25	1.14	1.25	Kovacha VP et al.	2012	Petrology	19（4）	399-425
853	28/07	CAOB	Transbaikalia and Mongolia	93.7000	47.0083	Tonalite		460	460	2.14	12.43	0.1034	0.512188		-0.47	-8.78	-3.30	-3.30	-3.30	1.46	1.33	1.46	1.33	1.46	Kovacha VP et al.	2012	Petrology	19（4）	399-425
854	42/07	CAOB	Transbaikalia and Mongolia	93.7167	46.9333	Granite		450	450	0.92	4.39	0.1245	0.512500		-0.37	-2.69	1.45	1.45	1.45	1.07	1.11	1.07	1.11	1.07	Kovacha VP et al.	2012	Petrology	19（4）	399-425
855	40/07	CAOB	Transbaikalia and Mongolia	93.7000	47.0083	Diorite		460	460	2.81	13.74	0.1277	0.512436		-0.35	-3.94	0.11	0.11	0.11	1.18	1.26	1.18	1.26	1.18	Kovacha VP et al.	2012	Petrology	19（4）	399-425
856	YQ-7	CAOB	Altai, unit 4	90.3667	46.4833	Gneissic Bi granodiorite	Mainobo	283	283	5.91	26.93	0.1326	0.512462	10	-0.33	-3.43	-1.12	-1.12	-1.12	1.14	1.29	1.14	1.29	1.14	Zhou G et al.	2007	Acta Petrologica Sinica	23(8)	1909-1920
857	YQ-4	CAOB	Altai, unit 4	90.3667	46.4833	Gneissic Bi granodiorite	Mainobo	283	283	4.62	21.87	0.1276	0.512359	12	-0.35	-5.44	-2.95	-2.95	-2.95	1.29	1.40	1.29	1.40	1.29	Zhou G et al.	2007	Acta Petrologica Sinica	23(8)	1909-1920
858	YQ-2	CAOB	Altai, unit 4	90.1300	46.6100	Mica granite	Mainobo	283	283	3.05	10.81	0.1707	0.512541	13	-0.13	-1.89	-0.95	-0.95	-0.95	1.12	2.15	1.12	2.15	1.12	Zhou G et al.	2007	Acta Petrologica Sinica	23(8)	1909-1920
859	YQ-17	CAOB	Altai, unit 4	90.3667	46.4833	Gneissic tonalite	Mainobo	283	283	5.39	26.40	0.1235	0.512321	12	-0.37	-6.18	-3.54	-3.54	-3.54	1.33	1.40	1.33	1.40	1.33	Zhou G et al.	2007	Acta Petrologica Sinica	23(8)	1909-1920
860	YQ-13	CAOB	Altai, unit 4	90.3667	46.4833	Gneissic Bi monzogranite	Mainobo	283	283	5.77	26.56	0.1313	0.512499	12	-0.33	-2.71	-0.35	-0.35	-0.35	1.08	1.20	1.08	1.20	1.08	Zhou G et al.	2007	Acta Petrologica Sinica	23(8)	1909-1920
861	ZS41	CAOB	Tianshan	81.0000	43.0000	Granite		360	360	1.14	7.12	0.0974	0.512237		-0.50	-7.82	-3.30	-3.26	-3.26	1.38	1.20	1.38	1.20	1.49	Bao ZH et al.	2017	Journal of Asian Earth Sciences	121(1-2)	100-117
862	ZS38	CAOB	Tianshan	81.0000	43.0000	Granite		360	360	1.41	7.36	0.1166	0.512283		-0.41	-6.92	-3.20	-3.25	-3.25	1.37	1.36	1.37	1.36	1.48	Bao ZH et al.	2017	Journal of Asian Earth Sciences	121(1-2)	100-117
863	ZS32	CAOB	Tianshan	81.0000	43.0000	Monzogranite		362	362	7.25	37.81	0.1167	0.512296		-0.41	-6.67	-3.00	-2.98	-2.98	1.35	1.34	1.35	1.34	1.47	Bao ZH et al.	2017	Journal of Asian Earth Sciences	121(1-2)	100-117
864	ZS29	CAOB	Tianshan	81.0000	43.0000	Monzogranite		362	362	3.39	17.29	0.1193	0.512302		-0.39	-6.55	-3.00	-2.98	-2.98	1.35	1.37	1.35	1.37	1.47	Bao ZH et al.	2017	Journal of Asian Earth Sciences	121(1-2)	100-117
865	ZS25	CAOB	Tianshan	81.0000	43.0000	Monzogranite		362	362	2.31	12.02	0.1170	0.512274		-0.41	-7.10	-3.40	-3.42	-3.42	1.39	1.38	1.39	1.38	1.50	Bao ZH et al.	2017	Journal of Asian Earth Sciences	121(1-2)	100-117
866	ZS23	CAOB	Tianshan	81.0000	43.0000	Monzogranite		362	362	2.90	14.73	0.1198	0.512276		-0.39	-7.06	-3.50	-3.51	-3.51	1.40	1.42	1.40	1.42	1.51	Bao ZH et al.	2017	Journal of Asian Earth Sciences	121(1-2)	100-117
867	40	CAOB	Russia Altai	86.8889	49.4472	Two-mica granite	Rakhmanovka	375	375	47.00	9.50	0.1162	0.512325	9	-0.41	-6.11	-2.25	-2.25	-2.25	1.31	1.29	1.31	0.72	1.31	Kruk NN.	2015	Russian Geology & Geophysics	56(8)	1097-1113
868	39	CAOB	Russia Altai	86.8889	49.4472	Bi granodiorite	Rakhmanovka	375	375	27.00	5.40	0.1259	0.512376	8	-0.36	-5.11	-1.72	-1.72	-1.72	1.26	1.34	1.26	0.72	1.27	Kruk NN.	2015	Russian Geology & Geophysics	56(8)	1097-1113
869	38	CAOB	Russia Altai	87.9528	50.4639	Bi granite	Kubadra	373	373	21.10	5.29	0.1408	0.512680	1	-0.28	0.82	3.48	3.48	3.48	0.84	0.98	0.84	0.72	0.84	Kruk NN.	2015	Russian Geology & Geophysics	56(8)	1097-1113
870	35	CAOB	Russia Altai	83.9847	51.0611	Two-mica leucogranite	Aba	370	370	17.20	3.50	0.1136	0.512490	1	-0.42	-2.89	1.05	1.04	1.04	1.03	1.00	1.03	0.72	1.04	Kruk NN.	2015	Russian Geology & Geophysics	56(8)	1097-1113
871	34	CAOB	Russia Altai	83.9847	51.0611	Bi cordierite granite	Aba	367	367	24.28	5.68	0.1358	0.512547	6	-0.31	-1.78	1.09	1.08	1.08	1.03	1.18	1.03	0.72	1.03	Kruk NN.	2015	Russian Geology & Geophysics	56(8)	1097-1113
872	33	CAOB	Russia Altai	84.0319	51.1417	Bi melanogranite	Borovlyanka	367	367	28.65	6.28	0.1269	0.512458	8	-0.35	-3.51	-0.24	-0.24	-0.24	1.14	1.21	1.14	0.72	1.14	Kruk NN.	2015	Russian Geology & Geophysics	56(8)	1097-1113
873	31	CAOB	Russia Altai	86.0889	51.7528	Bi granite	Chike–Taman	374	374	3.25	16.60	0.1246	0.512600	2	-0.37	-0.74	2.71	2.70	2.70	0.90	0.94	0.90	0.72	0.90	Kruk NN.	2015	Russian Geology & Geophysics	56(8)	1097-1113
874	29	CAOB	Russia Altai	82.9694	51.2528	Bi-Hb granodiorite	Kolyvan	374	374	4.12	20.64	0.1241	0.512453	7	-0.37	-3.61	-0.14	-0.14	-0.14	1.13	1.18	1.13	0.72	1.14	Kruk NN.	2015	Russian Geology & Geophysics	56(8)	1097-1113
875	28	CAOB	Russia Altai	82.9694	51.2528	Hb–Bi tonalite	Kolyvan	374	374	3.35	16.58	0.1210	0.512600	1	-0.38	-0.74	2.88	2.87	2.87	0.89	0.90	0.89	0.72	0.89	Kruk NN.	2015	Russian Geology & Geophysics	56(8)	1097-1113
876	27	CAOB	Russia Altai	83.9847	51.3347	Bi-Hb granodiorite	Bashchelak	374	374	7.32	30.48	0.1307	0.512444	8	-0.34	-3.78	-0.63	-0.63	-0.63	1.17	1.30	1.17	0.72	1.18	Kruk NN.	2015	Russian Geology & Geophysics	56(8)	1097-1113
877	26	CAOB	Russia Altai	85.2542	52.0778	Bi leucogranite	Biryuksa	380	380	3.64	15.93	0.1243	0.512669	3	-0.37	0.60	4.13	4.12	4.12	0.79	0.82	0.79	0.72	0.79	Kruk NN.	2015	Russian Geology & Geophysics	56(8)	1097-1113
878	23	CAOB	Russia Altai	85.5222	51.4806	lkali riebeckite leucogranit	Elinovo	380	380	9.32	41.80	0.1341	0.512674	9	-0.32	0.70	3.74	3.74	3.74	0.82	0.91	0.82	0.72	0.83	Kruk NN.	2015	Russian Geology & Geophysics	56(8)	1097-1113
879	22	CAOB	Russia Altai	83.6653	51.1083	lkali riebeckite leucogranit	Maiorka	381	381	23.42	122.12	0.1014	0.512633	3	-0.48	-0.10	4.54	4.54	4.54	0.76	0.70	0.76	0.72	0.76	Kruk NN.	2015	Russian Geology & Geophysics	56(8)	1097-1113
880	20	CAOB	Russia Altai	85.6014	51.6389	Bi-Hb granodiorite	Kinderla	384	384	4.36	21.60	0.1231	0.512626	8	-0.37	-0.23	3.37	3.37	3.37	0.86	0.88	0.86	0.72	0.86	Kruk NN.	2015	Russian Geology & Geophysics	56(8)	1097-1113

881	19	CAOB	Russia Altai	87.5861	52.0972	Miarolic Bi granite	Kyzyltash	395	395	5.00	26.67	0.1153	0.512648	5	-0.41	0.20	4.31	4.30	4.30	0.79	0.78	0.79	0.72	0.79	Kruk NN.	2015	Russian Geology & Geophysics	56(8)	1097-1113
882	16	CAOB	Russia Altai	84.5958	51.4694	Bi-Hb melanogranite	Oreshenka–Askat	395	395	6.55	29.11	0.1324	0.512754	6	-0.33	2.26	5.52	5.50	5.50	0.69	0.74	0.69	0.72	0.69	Kruk NN.	2015	Russian Geology & Geophysics	56(8)	1097-1113
883	15	CAOB	Russia Altai	84.5228	51.5367	Bi-Hb granodiorite	Topol'noe	395	395	5.12	25.49	0.1374	0.512696	6	-0.30	1.13	4.14	4.12	4.12	0.80	0.91	0.80	0.72	0.81	Kruk NN.	2015	Russian Geology & Geophysics	56(8)	1097-1113
884	14	CAOB	Russia Altai	86.5644	50.0822	Bi-Hb monzogranite	Turgunda	407	407	5.36	30.75	0.1055	0.512605	3	-0.46	-0.64	4.11	4.09	4.09	0.82	0.77	0.82	0.72	0.82	Kruk NN.	2015	Russian Geology & Geophysics	56(8)	1097-1113
885	11	CAOB	Russia Altai	86.4189	50.1644	Bi-Hb granodiorite	Katanda	404	404	5.70	26.80	0.1104	0.512499	5	-0.44	-2.71	1.75	1.74	1.74	1.00	0.96	1.00	0.72	1.01	Kruk NN.	2015	Russian Geology & Geophysics	56(8)	1097-1113
886	9	CAOB	Russia Altai	87.9194	50.7139	Bi-Hb granodiorite	Karakudyur	406	406	6.20	31.68	0.1490	0.512692	5	-0.24	1.05	3.53	3.52	3.52	0.86	1.08	0.86	0.72	0.86	Kruk NN.	2015	Russian Geology & Geophysics	56(8)	1097-1113
887	8	CAOB	Russia Altai	86.7889	52.1625	Porphyritic Bi granite	Turochak	390	390	4.86	23.92	0.1244	0.512650	9	-0.37	0.23	3.83	3.83	3.83	0.82	0.85	0.82	0.72	0.83	Kruk NN.	2015	Russian Geology & Geophysics	56(8)	1097-1113
888	6	CAOB	Russia Altai	86.9306	52.0306	Hb–Bi granodiorite	Yugala	400	400	4.02	19.39	0.1223	0.512499	5	-0.38	-2.71	1.10	1.09	1.09	1.05	1.09	1.05	0.72	1.06	Kruk NN.	2015	Russian Geology & Geophysics	56(8)	1097-1113
889	5	CAOB	Russia Altai	85.7528	51.7722	Hb tonalite	Barangol	510	510	2.04	8.95	0.1315	0.512769	4	-0.33	2.56	6.82	6.80	6.80	0.68	0.71	0.68	0.72	0.68	Kruk NN.	2015	Russian Geology & Geophysics	56(8)	1097-1113
890	4	CAOB	Russia Altai	86.6806	51.9111	Bi plagiogranite	Sarakoksha	512	512	0.97	5.94	0.1180	0.512715	12	-0.40	1.50	6.66	6.64	6.64	0.69	0.69	0.69	0.72	0.70	Kruk NN.	2015	Russian Geology & Geophysics	56(8)	1097-1113
891	1	CAOB	Russia Altai	88.4417	50.0583	Hb Qtz diorite	Dzheganterek	509	509	2.77	9.00	0.1862	0.512953	3	-0.05	6.14	6.83	6.83	6.83	0.68	1.09	0.68	0.72	0.68	Kruk NN.	2015	Russian Geology & Geophysics	56(8)	1097-1113
892	X-1052	CAOB	Kazakhstan	83.8854	48.6868	Qtz-monzonite		300	300	7.26	34.50	0.1273	0.512672	7	-0.35	0.66	3.30	3.32	3.32	0.79	0.84	0.79		0.80	Kuibida M L et al.	2019	Journal of Asian earth sciences	175	178-198
893	X-1047	CAOB	Kazakhstan	83.9375	48.7268	Granodiorite		300	300	5.40	22.28	0.1465	0.512682	8	-0.25	0.86	2.80	2.78	2.78	0.83	1.06	0.83		0.85	Kuibida M L et al.	2019	Journal of Asian earth sciences	175	178-198
894	MX-841	CAOB	Kazakhstan	83.4963	49.0639	Porphyritic granite		300	300	1.01	4.37	0.1401	0.512982	1	-0.29	6.71	8.90	8.88	8.88	0.34	0.35	0.34		0.34	Kuibida M L et al.	2019	Journal of Asian earth sciences	175	178-198
895	KT-34/1	CAOB	Kazakhstan	83.2318	49.4000	Qtz-monzonite		300	300	7.29	34.13	0.1291	0.512681	15	-0.34	0.84	3.40	3.43	3.43	0.78	0.85	0.78		0.80	Kuibida M L et al.	2019	Journal of Asian earth sciences	175	178-198
896	K-14-105/1	CAOB	Kazakhstan	82.6887	49.6306	Granite-porphyry		300	300	2.26	11.05	0.1239	0.512905	9	-0.37	5.21	8.00	8.00	8.00	0.41	0.42	0.41		0.41	Kuibida M L et al.	2019	Journal of Asian earth sciences	175	178-198
897	7-694	CAOB	Kazakhstan	83.9913	48.8325	Massive granite		300	300	1.81	8.85	0.1226	0.512834	6	-0.38	3.82	6.70	6.66	6.66	0.52	0.53	0.52		0.53	Kuibida M L et al.	2019	Journal of Asian earth sciences	175	178-198
898	SD020-3	NCC	NCC	117.7778	41.3333	Qtz diorite	Longhua	311	311	5.44	34.23	0.0961	0.511790	9	-0.51	-16.54	-12.56	-12.55	-12.55	2.09	1.76	2.09	1.76	2.09	Zhang SH et al.	2009	Geological Society of America Bulletin	121(1-2)	181-200
899	SD020-1	NCC	NCC	117.7778	41.3333	Qtz diorite	Longhua	311	311	5.83	33.88	0.1040	0.511788	11	-0.47	-16.58	-12.91	-12.90	-12.90	2.12	1.89	2.12	1.89	2.12	Zhang SH et al.	2009	Geological Society of America Bulletin	121(1-2)	181-200
900	HLB-G	NCC	NCC	117.3403	41.0650	Qtz diorite	Boluonuo	302	302	1.55	9.88	0.0947	0.511623	12	-0.52	-19.80	-15.87	-15.87	-15.87	2.35	1.95	2.35	1.95	2.35	Zhang SH et al.	2009	Geological Society of America Bulletin	121(1-2)	181-200
901	HFG-2	NCC	NCC	117.2667	41.1744	Granodiorite	Guanglingshan	254	254	2.16	14.10	0.0926	0.512016	10	-0.53	-12.13	-8.76	-8.76	-8.76	1.74	1.43	1.74	1.43	1.74	Zhang SH et al.	2009	Geological Society of America Bulletin	121(1-2)	181-200
902	FP3	NCC	NCC	116.9836	40.9881	Granodiorite	Hushiha	310	310	0.28	1.88	0.0896	0.511545	11	-0.54	-21.32	-17.1	-17.1	-17.1	2.46	1.96	2.46	1.97	2.46	Zhang SH et al.	2009	Geological Society of America Bulletin	121(1-2)	181-200
903	FP2	NCC	NCC	116.9836	40.9881	Granodiorite	Hushiha	310	310	0.76	5.22	0.0882	0.511829	12	-0.55	-15.78	-11.5	-11.5	-11.5	2.00	1.60	2.00	1.60	2.01	Zhang SH et al.	2009	Geological Society of America Bulletin	121(1-2)	181-200
904	D374	NCC	NCC	119.6303	41.8825	Monzogranite	Jianping	240	240	9.02	60.27	0.0905	0.511928	13	-0.54	-13.85	-10.6	-10.6	-10.6	1.87	1.51	1.87	1.51	1.88	Zhang SH et al.	2009	Geological Society of America Bulletin	121(1-2)	181-200
905	D322-1	NCC	NCC	119.6303	41.8825	Granodiorite	Jianping	304	304	7.05	37.14	0.1147	0.511692	12	-0.42	-18.45	-15.3	-15.3	-15.3	2.30	2.24	2.30	2.24	2.31	Zhang SH et al.	2009	Geological Society of America Bulletin	121(1-2)	181-200
906	D315-3	NCC	NCC	119.6303	41.8825	Monzogranite	Jianping	240	240	9.92	50.45	0.1189	0.512051	12	-0.40	-11.45	-9.1	-9.1	-9.1	1.75	1.76	1.75	1.77	1.75	Zhang SH et al.	2009	Geological Society of America Bulletin	121(1-2)	181-200
907	D315	NCC	NCC	119.6300	41.8825	Granodiorite	Jianping	304	304	4.01	23.44	0.1034	0.511831	12	-0.47	-15.74	-12.1	-12.1	-12.1	2.05	1.82	2.05	1.82	2.05	Zhang SH et al.	2009	Geological Society of America Bulletin	121(1-2)	181-200
908	D169-2	NCC	NCC	117.4453	41.2353	Qtz diorite	Daguangding	318	318	3.09	17.27	0.1081	0.511888	10	-0.45	-14.63	-11.0	-11.0	-11.0	1.97	1.82	1.97	1.82	1.97	Zhang SH et al.	2009	Geological Society of America Bulletin	121(1-2)	181-200
909	D159-1	NCC	NCC	117.4453	41.2353	Qtz diorite	Daguangding	318	318	2.94	18.63	0.0953	0.511836	11	-0.52	-15.64	-11.5	-11.5	-11.5	2.01	1.69	2.01	1.69	2.01	Zhang SH et al.	2009	Geological Society of America Bulletin	121(1-2)	181-200
910	D126-1	NCC	NCC	117.2667	41.1744	Granodiorite	Guanglingshan	254	254	0.75	3.71	0.1217	0.512001	13	-0.38	-12.43	-10.0	-10.0	-10.0	1.83	1.90	1.83	1.90	1.84	Zhang SH et al.	2009	Geological Society of America Bulletin	121(1-2)	181-200
911	D097-1	NCC	NCC	117.3403	41.0650	Qtz diorite	Boluonuo	302	302	1.87	10.92	0.1034	0.511656	12	-0.47	-19.16	-15.6	-15.6	-15.6	2.33	2.06	2.33	2.06	2.33	Zhang SH et al.	2009	Geological Society of America Bulletin	121(1-2)	181-200
912	D018-1	NCC	NCC	117.6350	41.2694	Qtz diorite	Daguangding	318	318	1.42	8.46	0.1012	0.511934	9	-0.49	-13.73	-9.87	-9.86	-9.86	1.88	1.64	1.88	1.65	1.88	Zhang SH et al.	2009	Geological Society of America Bulletin	121(1-2)	181-200
913	FX09-11	NCC	NCC	121.8469	42.1903	Monzodiorite	Sijiazi	220	220	0.69	5.72	0.0731	0.511985	14	-0.63	-12.74	-9.27	-9.27	-9.27	1.75	1.26	1.75	1.26	1.75	Zhang XH et al.	2012	Lithos	144-145	12-23
914	FX09-1	NCC	NCC	121.8469	42.1903	Monzodiorite	Sijiazi	220	220	3.58	18.20	0.1191	0.512553	11	-0.39	-1.66	0.53	0.52	0.52	0.95	0.96	0.95	0.97	0.96	Zhang XH et al.	2012	Lithos	144-145	12-23
915	FX06-9	NCC	NCC	121.8469	42.1903	Granodiorite	Sijiazi	220	220	6.12	38.60	0.0958	0.511986	12	-0.51	-12.72	-9.89	-9.89	-9.89	1.80	1.50	1.80	1.51	1.80	Zhang XH et al.	2012	Lithos	144-145	12-23
916	FX06-14	NCC	NCC	121.8469	42.1903	Granite	Sijiazi	220	220	5.64	35.57	0.0959	0.512014	13	-0.51	-12.17	-9.34	-9.34	-9.34	1.76	1.47	1.76	1.47	1.76	Zhang XH et al.	2012	Lithos	144-145	12-23
917	FX06-12	NCC	NCC	121.8469	42.1903	Granodiorite	Sijiazi	220	220	5.97	40.88	0.0883	0.511970	13	-0.55	-13.03	-9.99	-9.99	-9.99	1.81	1.43	1.81	1.43	1.81	Zhang XH et al.	2012	Lithos	144-145	12-23
918	FX06-11	NCC	NCC	121.8469	42.1903	Granite	Sijiazi	220	220	7.05	37.37	0.1141	0.512056	13	-0.42	-11.35	-9.03	-9.03	-9.03	1.73	1.67	1.73	1.67	1.73	Zhang XH et al.	2012	Lithos	144-145	12-23
919	DSL09-7	NCC	NMNCC	121.8869	42.3919	Monzodiorite	Dashaoleng	220	220	5.34	30.80	0.1049	0.512260	12	-0.47	-7.37	-4.80	-4.80	-4.80	1.39	1.25	1.39	1.25	1.39	Zhang XH et al.	2012	Lithos	144-145	12-23
920	DSL09-5	NCC	NMNCC	121.8869	42.3919	Monzodiorite	Dashaoleng	220	220	4.81	29.90	0.0972	0.512195	15	-0.51	-8.64	-5.85	-5.85	-5.85	1.47	1.25	1.47	1.25	1.47	Zhang XH et al.	2012	Lithos	144-145	12-23
921	DSL09-4	NCC	NMNCC	121.8869	42.3919	Monzodiorite	Dashaoleng	220	220	6.08	40.00	0.0920	0.512238	15	-0.53	-7.80	-4.86	-4.86	-4.86	1.39	1.14	1.39	1.14	1.39	Zhang XH et al.	2012	Lithos	144-145	12-23
922	DSL09-13	NCC	NMNCC	121.8869	42.3919	Monzodiorite	Dashaoleng	220	220	4.68	29.50	0.0961	0.512168	12	-0.51	-9.17	-6.34	-6.34	-6.34	1.51	1.27	1.51	1.27	1.51	Zhang XH et al.	2012	Lithos	144-145	12-23
923	ON-3	CAOB	Transbaikalia and Mongolia	107.5000	45.5000	Ongonite		120	150	6.20	15.11	0.2481	0.512627		0.26	-0.21	-1.00	-1.20	-1.20	1.05	-2.34	1.05	2.35	0.85	Dostal J et al.	2015	Lithos	236	173-189
924	ON-21	CAOB	Transbaikalia and Mongolia	107.5000	45.5000	Ongonite		120	150	3.43	8.19	0.2532	0.512625		0.29	-0.25	-1.12	-1.34	-1.34	1.06	-2.05	1.06	2.41	0.848	Dostal J et al.	2015	Lithos	236	173-189
925	ON-13	CAOB	Transbaikalia and Mongolia	107.5000	45.5000	Ongonite		120	150	4.96	12.34	0.2430	0.512626		0.24	-0.23	-0.94	-1.12	-1.12	1.04	-2.76	1.04	2.76	0.85	Dostal J et al.	2015	Lithos	236	173-189
926	TS64	CAOB	Western China	94.8333	42.0000	Gneiss		700	600	10.52	60.72	0.1047	0.511724	42	-0.47	-17.83	-9.30	-10.8	-10.8	2.18	1.99	2.18		2.18	Hu AQ et al.	2000	Tectonophysics	328（1-2）	15-51
927	TS63	CAOB	Western China	94.8333	42.0000	Gneiss		1300	600	3.65	22.11	0.0997	0.511690	34	-0.49	-18.49	-1.80	-11.1	-11.1	2.20	1.95	2.20		2.08	Hu AQ et al.	2000	Tectonophysics	328（1-2）	15-51
928	TS62	CAOB	Western China	94.8333	42.0000	Gneiss		1400	600	3.66	23.80	0.0930	0.511631	6	-0.53	-19.64	-1.10	-11.7	-11.7	2.26	1.91	2.26		2.06	Hu AQ et al.	2000	Tectonophysics	328（1-2）	15-51
929	TS61	CAOB	Western China	94.8333	42.0000	Gneiss		1300	600	12.72	70.15	0.1096	0.511850	30	-0.44	-15.37	-0.80	-8.70	-8.70	2.01	1.90	2.01		1.96	Hu AQ et al.	2000	Tectonophysics	328（1-2）	15-51
930	TS60	CAOB	Western China	94.8333	42.0000	Granitic		1300	600	6.53	31.86	0.1238	0.511980	26	-0.37	-12.84	-1.00	-7.25	-7.25	1.89	1.98	1.89		1.95	Hu AQ et al.	2000	Tectonophysics	328（1-2）	15-51
931	TS59	CAOB	Western China	94.0000	42.0000	Granulite		800	600	6.05	35.02	0.1044	0.511650	8	-0.47	-19.27	-9.90	-12.21	-12.21	2.30	2.08	2.30		2.29	Hu AQ et al.	2000	Tectonophysics	328（1-2）	15-51
932	TS58	CAOB	Western China	94.0000	42.0000	Granulite		1600	600	3.99	22.17	0.1089	0.511976	17	-0.45	-12.91	5.00	-6.19	-6.19	1.81	1.70	1.81		1.73	Hu AQ et al.	2000	Tectonophysics	328（1-2）	15-51
933	TS57	CAOB	Western China	94.0000	42.0000	Granulite		800	600	5.60	34.53	0.0981	0.511786	7	-0.50	-16.62	-6.60	-9.07	-9.07	2.04	1.79	2.04		2.02	Hu AQ et al.	2000	Tectonophysics	328（1-2）	15-51
934	TS56	CAOB	Western China	94.0000	42.0000	Schist		800	600	5.28	23.14	0.1380	0.511886	19	-0.30	-14.67	-8.70	-10.17	-10.17	2.13	2.53	2.13			Hu AQ et al.	2000	Tectonophysics	328（1-2）	15-51
935	TS54	CAOB	Western China	94.0000	42.0000	Gar-Bi-Qtz-Schi		800	600	10.05	55.67	0.1091	0.511871	10	-0.45	-14.96	-6.00	-8.25	-8.25	1.98	1.86	1.98		1.97	Hu AQ et al.	2000	Tectonophysics	328（1-2）	15-51
936	TS53	CAOB	Western China	94.0000	42.0000	Granulite		800	600	4.74	28.43	0.1008	0.512012	21	-0.49	-12.21	-2.40	-4.87	-4.87	1.70	1.53	1.70		1.68	Hu AQ et al.	2000	Tectonophysics	328（1-2）	15-51
937	TS49	CAOB	Western China	94.0000	42.0000	Granulite		800	600	5.34	25.86	0.1248	0.511918	10	-0.37	-14.04	-6.70	-8.54	-8.54	2.00	2.10	2.00		2.03	Hu AQ et al.	2000	Tectonophysics	328（1-2）	15-51
938	TS48	CAOB	Western China	93.8570	43.2130	Gneiss		800	600	2.60	11.32	0.1389	0.512316	21	-0.29	-6.28	-0.40	-1.86	-1.86	1.46	1.70	1.46		1.51	Hu AQ et al.	2000	Tectonophysics	328（1-2）	15-51
939	TS47	CAOB	Western China	93.8560	43.2110	Schist		800	600	15.91	100.40	0.0958	0.512246	35	-0.51	-7.65	2.70	0.08	0.08	1.30	1.17	1.30		1.26	Hu AQ et al.	2000	Tectonophysics	328（1-2）	15-51
940	TS46	CAOB	Western China	93.8550	43.2090	Gneiss		800	600	11.70	73.34	0.0964	0.512277	12	-0.51	-7.04	3.20	0.64	0.64	1.25	1.13	1.25		1.22	Hu AQ et al.	2000	Tectonophysics	328（1-2）	15-51
941	TS45	CAOB	Western China	93.8540	43.2070	Amphibolite		800	600	4.75	18.54	0.1547	0.512424	23	-0.21	-4.17	0.10	-0.96	-0.96	1.38	1.87	1.38			Hu AQ et al.	2000	Tectonophysics	328（1-2）	15-51
942	TS44	CAOB	Western China	93.8530	43.2050	Granitic		800	600	6.64	54.38	0.0739	0.511991	12	-0.62	-12.62	-0.10	-3.22	-3.22	1.57	1.26	1.57		1.48	Hu AQ et al.	2000	Tectonophysics	328（1-2）	15-51
943	TS40b	CAOB	Western China	93.8520	43.2030	Dioritic		700	600	4.76	26.15	0.1101	0.511795	8	-0.44	-16.44	-8.70	-9.81	-9.81	2.10	1.99	2.10		2.11	Hu AQ et al.	2000	Tectonophysics	328（1-2）	15-51
944	TS39	CAOB	Western China	93.8510	43.2010	Granitic GN		700	600	6.46	38.29	0.1019	0.511541	23	-0.48	-21.40	-12.90	-14.14	-14.14	2.45	2.18	2.45		2.46	Hu AQ et al.	2000	Tectonophysics	328（1-2）	15-51
945	TS35	CAOB	Western China	83.5330	44.3840	Schist		1000	600	1.44	4.67	0.1871	0.512387	34	-0.05	-4.90	-3.70	-4.16	-4.16	1.63	4.32	1.63		1.94	Hu AQ et al.	2000	Tectonophysics	328（1-2）	15-51
946	TS27	CAOB	Western China	83.5260	44.3770	Schist		1000	600	6.67	36.87	0.1093	0.511857	15	-0.44	-15.23	-4.10	-8.54	-8.54	2.00	1.88	2.00		1.98	Hu AQ et al.	2000	Tectonophysics	328（1-2）	15-51
947	TS26	CAOB	Western China	83.5250	44.3760	Gneiss		1000	600	3.03	17.00	0.1077	0.512172	10	-0.45	-9.09	2.30	-2.28	-2.28	1.49	1.40	1.49		1.46	Hu AQ et al.	2000	Tectonophysics	328（1-2）	15-51
948	TS25	CAOB	Western China	83.5240	44.3750	Gneiss		1000	600	4.67	24.17	0.1169	0.512189	7	-0.41	-8.76	1.40	-2.65	-2.65	1.52	1.51	1.52		1.53	Hu AQ et al.	2000	Tectonophysics	328（1-2）	15-51
949	TS24	CAOB	Western China	83.5230	44.3740	Gneiss		1000	600	3.44	16.60	0.1251	0.512421	9	-0.36	-4.23	4.90	1.25	1.25	1.20	1.25	1.20		1.24	Hu AQ et al.	2000	Tectonophysics	328（1-2）	15-51
950	TS23	CAOB	Western China	83.5220	44.3730	Gneiss		1000	600	8.20	39.41	0.1257	0.512292	18	-0.36	-6.75	2.30	-1.31	-1.31	1.41	1.48	1.41		1.45	Hu AQ et al.	2000	Tectonophysics	328（1-2）	15-51
951	TS22	CAOB	Western China	83.5210	44.3720	Amphibolite		1000	600	11.52	53.19	0.1309	0.512465	11	-0.33	-3.37	5.00	1.66	1.66	1.17	1.26	1.17			Hu AQ et al.	2000	Tectonophysics	328（1-2）	15-51
952	TS21	CAOB	Western China	81.6250	44.4350	Gneiss		700	600	6.35	36.61	0.1048	0.511953	11	-0.47	-13.36	-5.20	-6.33	-6.33	1.82	1.67	1.82		1.82	Hu AQ et al.	2000	Tectonophysics	328（1-2）	15-51
953	TS20	CAOB	Western China	81.6240	44.4340	Granitic		900	600	3.61	16.11	0.1354	0.512288	23	-0.31	-6.83	-0.10	-2.13	-2.13	1.48	1.67	1.48		1.54	Hu AQ et al.	2000	Tectonophysics	328（1-2）	15-51
954	TS19	CAOB	Western China	81.6230	44.4330	Mig-Gneiss		900	600	6.70	32.01	0.1265	0.512040	7	-0.36	-11.67	-3.70	-6.29	-6.29	1.82	1.93	1.82		1.86	Hu AQ et al.	2000	Tectonophysics	328（1-2）	15-51
955	TS18	CAOB	Western China	81.6220	44.4320	Gneiss		900	600	8.36	39.72	0.1272	0.512048	7	-0.35	-11.51	-3.50	-6.19	-6.19	1.81	1.94	1.81		1.85	Hu AQ et al.	2000	Tectonophysics	328（1-2）	15-51
956	TS17b	CAOB	Western China	81.6210	44.4310	Gneiss		800	600	6.85	29.38	0.1409	0.512202	20	-0.28	-8.51	-2.70	-4.23	-4.23	1.65	1.98	1.65		1.71	Hu AQ et al.	2000	Tectonophysics	328（1-2）	15-51
957	TS4b	CAOB	Western China	80.8333	45.8333	Granitic		1000	600	4.52	23.29	0.1174	0.512181	12	-0.40	-8.91	1.20	-2.84	-2.84	1.54	1.53	1.54		1.54	Hu AQ et al.	2000	Tectonophysics	328（1-2）	15-51
958	TS1	CAOB	Western China	80.8333	45.8333	Augen GN		800	600	19.56	95.10	0.1243	0.512104	36	-0.37	-10.42	-3.40	-4.87	-4.87	1.70	1.78	1.70		1.72	Hu AQ et al.	2000	Tectonophysics	328（1-2）	15-51
962	NT47	CAOB	Western China	86.0070	40.5360	Gneiss		2300	600	1.24	7.81	0.0958	0.511124	46	-0.51	-29.53	0.10	-21.8	-21.8	3.07	2.61	3.07		2.71	Hu AQ et al.	2000	Tectonophysics	328（1-2）	15-51
963	NT46	CAOB	Western China	86.0060	40.5350	Migmatite		2300	600	2.23	15.06	0.0895	0.511043	25	-0.54	-31.11	0.40	-22.9	-22.9	3.16	2.57	3.16		2.68	Hu AQ et al.	2000	Tectonophysics	328（1-2）	15-51
964	NT42	CAOB	Western China	86.0020	40.5310	Gneiss		2100	600	6.09	32.57	0.1131	0.511298	29	-0.43	-26.14	-3.70	-19.7	-19.7	2.90	2.79	2.90		2.85	Hu AQ et al.	2000	Tectonophysics	328（1-2）	15-51
965	NT41	CAOB	Western China	82.0620	43.0550	Gneiss		2500	600	6.01	34.41	0.1056	0.511438	16	-0.46	-23.41	5.60	-16.4	-16.4	2.64	2.40	2.64		2.41	Hu AQ et al.	2000	Tectonophysics	328（1-2）	15-51
966	NT40	CAOB	Western China	82.0610	43.0540	Gneiss		1300	600	4.55	31.10	0.0884	0.511335	22	-0.55	-25.42	-7.50	-17.1	-17.1	2.69	2.20	2.69		2.50	Hu AQ et al.	2000	Tectonophysics	328（1-2）	15-51
967	NT39	CAOB	Western China	82.0600	43.0530	Gneiss		1300	600	5.09	33.50	0.0919	0.511343	20	-0.53	-25.26	-7.90	-17.2	-17.2	2.70	2.25	2.70		2.53	Hu AQ et al.	2000	Tectonophysics	328（1-2）	15-51
968	NT38	CAOB	Western China	82.0590	43.0520	Gneiss		1300	600	5.93	46.27	0.0775	0.511223	22	-0.61	-27.60	-7.80	-18.5	-18.5	2.80	2.15	2.80		2.53	Hu AQ et al.	2000	Tectonophysics	328（1-2）	15-51
969	NT37	CAOB	Western China	82.0580	43.0510	Gneiss		1300	600	6.13	31.00	0.1196	0.511583	12	-0.39	-20.58	-7.80	-14.7	-14.7	2.49	2.53	2.49		2.53	Hu AQ et al.	2000	Tectonophysics	328（1-2）	15-51
971	NT35	CAOB	Western China	82.0560	43.0490	Dioritic		900	600	11.27	66.31	0.1028	0.511481	18	-0.48	-22.57	-12.2	-15.4	-15.4	2.55	2.28	2.55		2.53	Hu AQ et al.	2000	Tectonophysics	328（1-2）	15-51
972	NT34	CAOB	Western China	82.0550	43.0480	Dioritic		900	600	5.95	37.18	0.0968	0.511454	14	-0.51	-23.10	-12.0	-15.4	-15.4	2.56	2.20	2.56		2.52	Hu AQ et al.	2000	Tectonophysics	328（1-2）	15-51
973	NT33	CAOB	Western China	82.0540	43.0470	Dioritic		900	600	7.52	44.93	0.1012	0.511417	10	-0.49	-23.82	-13.2	-16.5	-16.5	2.64	2.34	2.64		2.62	Hu AQ et al.	2000	Tectonophysics	328（1-2）	15-51
974	NT32	CAOB	Western China	82.0530	43.0460	Gneiss		2200	600	4.86	26.57	0.1105	0.511560	20	-0.44	-21.03	3.60	-14.4	-14.4	2.47	2.34	2.47		2.37	Hu AQ et al.	2000	Tectonophysics	328（1-2）	15-51
975	NT31	CAOB	Western China	82.0520	43.0450	Gneiss		2200	600	3.74	24.35	0.0929	0.511349	12	-0.53	-25.14	4.50	-17.2	-17.2	2.70	2.26	2.70		2.29	Hu AQ et al.	2000	Tectonophysics	328（1-2）	15-51
976	NT24	CAOB	Western China	82.0510	43.0440	Gneiss		2200	600	5.97	32.44	0.1113	0.511586	12	-0.43	-20.52	3.90	-14.0	-14.0	2.44	2.32	2.44		2.34	Hu AQ et al.	2000	Tectonophysics	328（1-2）	15-51
977	NT23	CAOB	Western China	82.0500	43.0430	Gneiss		2200	600	5.42	28.40	0.1153	0.511653	16	-0.41	-19.21	4.00	-13.0	-13.0	2.36	2.31	2.36		2.33	Hu AQ et al.	2000	Tectonophysics	328（1-2）	15-51
978	NT28	CAOB	Western China	82.0490	43.0420	Gneiss		2200	600	6.93	35.03	0.1197	0.511721	16	-0.39	-17.89	4.10	-12.0	-12.0	2.28	2.31	2.28		2.32	Hu AQ et al.	2000	Tectonophysics	328（1-2）	15-51
979	NT27	CAOB	Western China	82.0480	43.0410	Granitic		800	600	7.47	38.94	0.1160	0.511459	25	-0.41	-23.00	-14.80	-16.8	-16.8	2.67	2.62	2.67		2.69	Hu AQ et al.	2000	Tectonophysics	328（1-2）	15-51
980	NT26	CAOB	Western China	82.0470	43.0400	Granitic		900	600	4.25	28.15	0.0912	0.511361	30	-0.54	-24.91	-13.20	-16.8	-16.8	2.67	2.22	2.67		2.62	Hu AQ et al.	2000	Tectonophysics	328（1-2）	15-51
981	NT25	CAOB	Western China	88.5833	42.5000	Schist		2400	600	2.84	15.81	0.1087	0.511250	36	-0.45	-27.08	-0.10	-20.3	-20.3	2.95	2.74	2.95		2.80	Hu AQ et al.	2000	Tectonophysics	328（1-2）	15-51
982	NT22	CAOB	Western China	88.5833	42.5000	Granitic GN		2100	600	6.78	36.66	0.1119	0.511158	45	-0.43	-28.87	-6.40	-22.4	-22.4	3.11	2.96	3.11		3.05	Hu AQ et al.	2000	Tectonophysics	328（1-2）	15-51

983	NT21	CAOB	Western China	88.5833	42.5000	Granitic GN		2100	600	1.79	8.66	0.1247	0.511378	43	-0.37	-24.58	-5.50	-19.1	-19.1	2.85	3.01	2.85		2.98	Hu AQ et al.	2000	Tectonophysics	328（1-2）	15-51
984	NT20	CAOB	Western China	88.5833	42.5000	Granitic GN		2100	600	8.97	48.70	0.1114	0.511124	25	-0.43	-29.53	-7.00	-23.0	-23.0	3.16	3.00	3.16		3.10	Hu AQ et al.	2000	Tectonophysics	328（1-2）	15-51
985	NT15	CAOB	Western China	88.5833	42.5000	Gneiss		2600	600	15.54	92.17	0.1019	0.510954	36	-0.48	-32.85	-1.40	-25.6	-25.6	3.37	2.97	3.37		3.08	Hu AQ et al.	2000	Tectonophysics	328（1-2）	15-51
986	NT14	CAOB	Western China	88.5833	42.5000	Gneiss		2600	600	11.59	82.76	0.0847	0.510566	28	-0.57	-40.42	-3.20	-31.8	-31.8	3.88	3.03	3.88		3.23	Hu AQ et al.	2000	Tectonophysics	328（1-2）	15-51
987	NT13	CAOB	Western China	88.5833	42.5000	Gneiss		2600	600	8.43	57.63	0.0885	0.510655	29	-0.55	-38.68	-2.80	-30.4	-30.4	3.76	3.02	3.76		3.19	Hu AQ et al.	2000	Tectonophysics	328（1-2）	15-51
988	NT12	CAOB	Western China	88.5833	42.5000	Gneiss		2600	600	15.53	95.71	0.0981	0.510839	38	-0.50	-35.09	-2.40	-27.5	-27.5	3.53	3.03	3.53		3.15	Hu AQ et al.	2000	Tectonophysics	328（1-2）	15-51
989	NT11	CAOB	Western China	88.5833	42.5000	Gneiss		2600	600	16.36	105.50	0.0937	0.510818	28	-0.52	-35.50	-1.30	-27.6	-27.6	3.54	2.94	3.54		3.07	Hu AQ et al.	2000	Tectonophysics	328（1-2）	15-51
990	TS68	CAOB	Western China	94.8333	42.0000	Gneiss		800	600	2.95	15.31	0.1166	0.512254	10	-0.41	-7.49	0.70	-1.36	-1.36	1.42	1.40	1.42		1.42	Hu AQ et al.	2000	Tectonophysics	328（1-2）	15-51
991	TS67	CAOB	Western China	94.8333	42.0000	Gneiss		1200	600	5.58	32.21	0.1047	0.511812	8	-0.47	-16.11	-2.50	-9.07	-9.07	2.04	1.87	2.04		1.98	Hu AQ et al.	2000	Tectonophysics	328（1-2）	15-51
992	K09-13	CAOB	NW China	83.0000	42.2000	Granite	Kuchehe	293	293	6.93	41.32	0.1031	0.512052		-0.48	-11.43	-7.90	-7.93	-7.93	1.70	1.51	1.70	1.52	1.71	Liu HQ et al.	2018	Lithos	326-327	370-383
993	K09-12	CAOB	NW China	83.0000	42.2000	Granite	Kuchehe	293	293	7.92	45.16	0.1078	0.512039		-0.45	-11.68	-8.40	-8.36	-8.36	1.74	1.60	1.74	1.61	1.75	Liu HQ et al.	2018	Lithos	326-327	370-383
994	K07-2	CAOB	NW China	83.0000	42.2000	Granite	Kuchehe	293	293	7.37	41.43	0.1093	0.512065		-0.44	-11.18	-7.90	-7.91	-7.91	1.70	1.58	1.70	1.59	1.71	Liu HQ et al.	2018	Lithos	326-327	370-383
995	K05-1	CAOB	NW China	83.0000	42.2000	Granite	Kuchehe	293	293	6.78	37.87	0.1101	0.512055		-0.44	-11.37	-8.10	-8.13	-8.13	1.72	1.61	1.72	1.62	1.73	Liu HQ et al.	2018	Lithos	326-327	370-383
996	K04-2	CAOB	NW China	83.0000	42.2000	Granite	Kuchehe	293	293	7.24	40.72	0.1094	0.512037		-0.44	-11.72	-8.50	-8.46	-8.46	1.74	1.62	1.74	1.63	1.76	Liu HQ et al.	2018	Lithos	326-327	370-383
997	K04-1	CAOB	NW China	83.0000	42.2000	Granite	Kuchehe	293	293	7.77	42.88	0.1114	0.512056		-0.43	-11.35	-8.20	-8.16	-8.16	1.72	1.63	1.72	1.64	1.73	Liu HQ et al.	2018	Lithos	326-327	370-383
998	K03-2	CAOB	NW China	83.0000	42.2000	Granite	Kuchehe	293	293	8.48	48.98	0.1065	0.512059		-0.46	-11.29	-7.90	-7.92	-7.92	1.70	1.55	1.70	1.56	1.71	Liu HQ et al.	2018	Lithos	326-327	370-383
999	DWQ09-6	CAOB	NW China	82.0000	42.1000	Granite	Dawanqi	274	274	48.55	239.60	0.1246	0.512241		-0.37	-7.74	-5.20	-5.22	-5.22	1.46	1.55	1.46	1.56	1.47	Liu HQ et al.	2018	Lithos	326-327	370-383
1000	DWQ09-5	CAOB	NW China	82.0000	42.1000	Granite	Dawanqi	274	274	4.03	23.47	0.1055	0.512199		-0.46	-8.56	-5.40	-5.38	-5.38	1.48	1.34	1.48	1.35	1.49	Liu HQ et al.	2018	Lithos	326-327	370-383
1001	ZJS15	NCC	NCC	110.8803	38.1203	Nepheline syenite	Zijinshan	127	150			0.1796	0.512481		-0.09	-3.06	-2.78	-2.74	-2.74	1.15	2.97	1.15	2.98	1.14	Yin JF et al.	2011	Lithos	125(1-2)	449-462
1002	ZJS08	NCC	NCC	110.8803	38.1203	Nepheline syenite	Zijinshan	127	150			0.1834	0.512490		-0.07	-2.89	-2.66	-2.63	-2.63	1.14	3.29	1.14	3.31	1.13	Yin JF et al.	2011	Lithos	125(1-2)	449-462
1003	ZJS03	NCC	NCC	110.8803	38.1203	Diopside syenite	Zijinshan	127	150			0.1285	0.512282		-0.35	-6.94	-5.85	-5.64	-5.64	1.40	1.55	1.40	1.55	1.40	Yin JF et al.	2011	Lithos	125(1-2)	449-462
1004	ZJS02	NCC	NCC	110.8803	38.1203	Monzonite	Zijinshan	127	150			0.1216	0.512114		-0.38	-10.22	-9.01	-8.78	-8.78	1.65	1.71	1.65	1.71	1.65	Yin JF et al.	2011	Lithos	125(1-2)	449-462
1005	ZJS01	NCC	NCC	110.8803	38.1203	Monzonite	Zijinshan	127	150			0.1044	0.512036		-0.47	-11.74	-10.26	-9.98	-9.98	1.75	1.55	1.75	1.55	1.76	Yin JF et al.	2011	Lithos	125(1-2)	449-462
1006	03ZJS13	NCC	NCC	110.8803	38.1203	Malignite	Zijinshan	127	150			0.1262	0.512177		-0.36	-8.99	-7.86	-7.64	-7.64	1.56	1.69	1.56	1.70	1.56	Yin JF et al.	2011	Lithos	125(1-2)	449-462
1007	03ZJS12-dupl	NCC	NCC	110.8803	38.1203	Nepheline syenite	Zijinshan	127	150			0.1694	0.512423		-0.14	-4.19	-3.76	-3.67	-3.67	1.23	2.49	1.23	2.50	1.22	Yin JF et al.	2011	Lithos	125(1-2)	449-462
1008	03ZJS12	NCC	NCC	110.8803	38.1203	Nepheline syenite	Zijinshan	127	150			0.1717	0.512464		-0.13	-3.39	-3.00	-2.92	-2.92	1.17	2.48	1.17	2.49	1.16	Yin JF et al.	2011	Lithos	125(1-2)	449-462
1009	03ZJS09	NCC	NCC	110.8803	38.1203	Malignite	Zijinshan	127	150			0.1316	0.512294		-0.33	-6.71	-5.65	-5.46	-5.46	1.38	1.59	1.38	1.59	1.38	Yin JF et al.	2011	Lithos	125(1-2)	449-462
1010	03ZJS07	NCC	NCC	110.8803	38.1203	Diopside syenite	Zijinshan	127	150			0.1304	0.512287		-0.34	-6.85	-5.77	-5.58	-5.58	1.39	1.58	1.39	1.58	1.39	Yin JF et al.	2011	Lithos	125(1-2)	449-462
1011	03ZJS06-dupl	NCC	NCC	110.8803	38.1203	Diopside syenite	Zijinshan	127	150			0.1332	0.512240		-0.32	-7.76	-6.74	-6.55	-6.55	1.47	1.72	1.47	1.72	1.47	Yin JF et al.	2011	Lithos	125(1-2)	449-462
1012	03ZJS01	NCC	NCC	110.8803	38.1203	Monzonite	Zijinshan	127	150			0.1065	0.511970		-0.46	-13.03	-11.6	-11.3	-11.3	1.86	1.67	1.86	1.68	1.86	Yin JF et al.	2011	Lithos	125(1-2)	449-462
1013	003ZJS06	NCC	NCC	110.8803	38.1203	Diopside syenite	Zijinshan	127	150			0.1346	0.512407		-0.32	-4.51	-3.50	-3.32	-3.32	1.21	1.43	1.21	1.43	1.21	Yin JF et al.	2011	Lithos	125(1-2)	449-462
1014		NCC	NCC	110.8803	38.1203	Diopside syenite	Zijinshan	127	150			0.1346	0.512296		-0.32	-6.67	-5.67	-5.48	-5.48	1.38	1.64	1.38	1.65	1.38	Yin JF et al.	2011	Lithos	125(1-2)	449-462
1015		NCC	NCC	110.8803	38.1203	Diopside syenite	Zijinshan	127	150			0.1285	0.512176		-0.35	-9.01	-7.91	-7.71	-7.71	1.56	1.74	1.56	1.74	1.57	Yin JF et al.	2011	Lithos	125(1-2)	449-462
1016	N62	NCC	NCC	115.3847	40.9225	Granite	Honghualiang	235	235	3.17	22.27	0.0861	0.511788	6	-0.56	-16.58	-13.3	-13.3	-13.3	2.09	1.62	2.09	1.63	2.09	Jiang N et al.	2007	Geochimica et Cosmochimica Acta	71(10)	2591-2608
1017	N61	NCC	NCC	115.4308	40.9406	Granite	Honghualiang	235	235	3.60	25.78	0.0846	0.511786	11	-0.57	-16.62	-13.3	-13.3	-13.3	2.09	1.61	2.09	1.61	2.09	Jiang N et al.	2007	Geochimica et Cosmochimica Acta	71(10)	2591-2608
1018	N54	NCC	NCC	115.4383	40.8869	Diorite	Zhuanzhilian	140	150	10.94	63.69	0.1039	0.511845	6	-0.47	-15.47	-13.8	-13.7	-13.7	2.05	1.81	2.05	1.81	2.06	Jiang N et al.	2007	Geochimica et Cosmochimica Acta	71(10)	2591-2608
1019	N51	NCC	NCC	115.4383	40.9106	Granite	Honghualiang	235	235	3.18	22.03	0.0873	0.511779	9	-0.56	-16.76	-13.5	-13.5	-13.5	2.10	1.65	2.10	1.65	2.11	Jiang N et al.	2007	Geochimica et Cosmochimica Acta	71(10)	2591-2608
1020	N302	NCC	NCC	115.4075	40.8989	Diorite	Zhuanzhilian	140	150	8.58	50.15	0.1035	0.511790	13	-0.47	-16.54	-14.9	-14.8	-14.8	2.14	1.88	2.14	1.88	2.14	Jiang N et al.	2007	Geochimica et Cosmochimica Acta	71(10)	2591-2608
1021	N301	NCC	NCC	115.3461	40.9225	Granite	Honghualiang	235	235	2.75	18.84	0.0885	0.511841	14	-0.55	-15.55	-12.3	-12.3	-12.3	2.01	1.59	2.01	1.59	2.01	Jiang N et al.	2007	Geochimica et Cosmochimica Acta	71(10)	2591-2608
1022	N15	NCC	NCC	115.4075	40.8989	Diorite	Zhuanzhilian	140	150	8.01	49.65	0.0976	0.511783	4	-0.50	-16.68	-14.9	-14.8	-14.8	2.14	1.79	2.14	1.79	2.15	Jiang N et al.	2007	Geochimica et Cosmochimica Acta	71(10)	2591-2608
1023	N14	NCC	NCC	115.4075	40.8989	Diorite	Zhuanzhilian	140	150	7.58	43.56	0.1053	0.511793	9	-0.46	-16.48	-14.9	-14.7	-14.7	2.14	1.90	2.14	1.91	2.14	Jiang N et al.	2007	Geochimica et Cosmochimica Acta	71(10)	2591-2608
1024	JN02	NCC	NCC	115.1769	40.8511	Granite	Guzuizi	236	236	1.90	10.67	0.1076	0.511673	9	-0.45	-18.82	-16.2	-16.1	-16.1	2.32	2.11	2.32	2.12	2.32	Jiang N et al.	2007	Geochimica et Cosmochimica Acta	71(10)	2591-2608
1025	JN01	NCC	NCC	115.1922	40.8333	Granite	Guzuizi	236	236	1.50	8.52	0.1067	0.511654	6	-0.46	-19.19	-16.5	-16.5	-16.5	2.35	2.12	2.35	2.13	2.35	Jiang N et al.	2007	Geochimica et Cosmochimica Acta	71(10)	2591-2608
1026	16206-1	NCC	NNCC	112.9617	41.5497	Monzonite		389	389	5.75	54.20	0.0642	0.511815		-0.67	-16.05	-9.30	-9.48	-9.48	1.91	1.36	1.91	1.36		Zhang QQ et al.	2018	Lithos	302-303	496-518
1027	16201-1	NCC	NNCC	113.1744	41.5925	Monzonite		389	389	5.23	28.20	0.1119	0.511801		-0.43	-16.33	-12.2	-12.1	-12.1	2.12	2.01	2.12	2.01		Zhang QQ et al.	2018	Lithos	302-303	496-518
1028	16200-1	NCC	NNCC	113.1744	41.5925	Monzonite		389	389	5.44	28.90	0.1139	0.511882		-0.42	-14.75	-10.7	-10.6	-10.6	2.00	1.93	2.00	1.93		Zhang QQ et al.	2018	Lithos	302-303	496-518
1029	08587-1	NCC	NNCC	110.4214	41.0431	Syenite		389	389	5.12	28.90	0.1073	0.511667		-0.45	-18.94	-14.4	-14.5	-14.5	2.31	2.12	2.31	2.12		Zhang QQ et al.	2018	Lithos	302-303	496-518
1030	08586-1	NCC	NNCC	110.4214	41.0431	Monzonite		389	389	5.96	32.70	0.1104	0.511684		-0.44	-18.61	-14.3	-14.3	-14.3	2.30	2.15	2.30	2.15		Zhang QQ et al.	2018	Lithos	302-303	496-518
1031	07169-1	NCC	NNCC	110.5483	40.9381	Monzonite		389	389	5.79	33.04	0.1061	0.511435		-0.46	-23.47	-18.9	-19.0	-19.0	2.67	2.42	2.67	2.42		Zhang QQ et al.	2018	Lithos	302-303	496-518
1032	07053-1	NCC	NNCC	113.1383	41.5969	Monzonite		389	389	5.46	28.41	0.1164	0.511833		-0.41	-15.70	-11.8	-11.7	-11.7	2.08	2.06	2.08	2.06		Zhang QQ et al.	2018	Lithos	302-303	496-518
1033	07052-1	NCC	NNCC	112.8917	41.5564	Syenite		389	389	5.14	30.50	0.1021	0.511522		-0.48	-21.77	-16.9	-17.1	-17.1	2.52	2.21	2.52	2.21		Zhang QQ et al.	2018	Lithos	302-303	496-518
1034	07051-1	NCC	NNCC	112.8917	41.5564	Syenite		389	389	5.30	30.00	0.1070	0.511618		-0.46	-19.90	-15.3	-15.4	-15.4	2.39	2.18	2.39	2.18		Zhang QQ et al.	2018	Lithos	302-303	496-518
1035	07049-1	NCC	NNCC	112.8917	41.5564	Syenite		389	389	5.46	32.30	0.1022	0.511484		-0.48	-22.51	-17.7	-17.8	-17.8	2.58	2.27	2.58	2.27		Zhang QQ et al.	2018	Lithos	302-303	496-518
1036	08550-1	NCC	NNCC	109.9873	41.8827	Andesite		273	273	5.21	27.24	0.1158	0.511870		-0.41	-14.98	-12.2	-12.2	-12.2	2.03	1.99	2.03	1.99		Zhang SH et al.	2016	Lithos	240-243	382-401
1037	08543-2	NCC	NNCC	110.1332	41.8435	Rhyolite		269	269	9.14	52.34	0.1057	0.512018		-0.46	-12.09	-9.00	-8.97	-8.97	1.77	1.59	1.77	1.59		Zhang SH et al.	2016	Lithos	240-243	382-401
1038	08485-1	NCC	NNCC	110.7320	41.7647	Rhyolite		264	264	9.10	48.41	0.1138	0.512068		-0.42	-11.12	-8.30	-8.33	-8.33	1.71	1.65	1.71	1.65		Zhang SH et al.	2016	Lithos	240-243	382-401
1039	08358-1	NCC	NNCC	113.8612	42.0063	Rhyolite		270	270	2.55	8.69	0.1779	0.512405		-0.10	-4.55	-3.90	-3.90	-3.90	1.35	3.15	1.35	3.15		Zhang SH et al.	2016	Lithos	240-243	382-401
1040	07D037-1	NCC	NNCC	118.6462	42.6293	Rhyolite		273	273	4.98	31.66	0.0952	0.511950		-0.52	-13.42	-9.90	-9.88	-9.88	1.84	1.54	1.84	1.54		Zhang SH et al.	2016	Lithos	240-243	382-401
1041	07D024-1	NCC	NNCC	119.6930	42.4067	Andesite		273	273	4.09	18.77	0.1318	0.512472		-0.33	-3.24	-1.00	-0.98	-0.98	1.12	1.26	1.12	1.26		Zhang SH et al.	2016	Lithos	240-243	382-401
1042	07D023-2	NCC	NNCC	119.6960	42.4027	Dacite		344	344	7.89	39.79	0.1200	0.512406		-0.39	-4.53	-1.20	-1.16	-1.16	1.19	1.21	1.19	1.21		Zhang SH et al.	2016	Lithos	240-243	382-401
1043	07D023-1	NCC	NNCC	119.6960	42.4027	Rhyolite		344	344	10.00	56.60	0.1072	0.512369		-0.46	-5.25	-1.30	-1.32	-1.32	1.20	1.12	1.20	1.12		Zhang SH et al.	2016	Lithos	240-243	382-401
1044	07D016-1	NCC	NNCC	119.8103	42.5090	Dacite		258	258	7.89	42.55	0.1123	0.512458		-0.43	-3.51	-0.70	-0.73	-0.73	1.09	1.04	1.09	1.04		Zhang SH et al.	2016	Lithos	240-243	382-401
1045	07057-1	NCC	NNCC	112.8530	41.8437	Rhyolite		276	276	7.99	38.83	0.1245	0.511885		-0.37	-14.69	-12.1	-12.1	-12.1	2.03	2.15	2.03	2.15		Zhang SH et al.	2016	Lithos	240-243	382-401
1046	W08-298	CAOB	Beishan	99.7592	41.4790	Granite	Dongqiyishan	356	356	2.78	16.40	0.0744	0.512383		-0.62	-4.97	0.60	0.58	0.58	1.06	0.84	1.06	0.84	1.06	Zhang W et al.	2012	Lithos	142	148-160
1047	W08-295	CAOB	Beishan	99.7392	41.4666	Granite	Dongqiyishan	356	356	2.78	15.30	0.0752	0.512319		-0.62	-6.22	-0.70	-0.70	-0.70	1.16	0.91	1.16	0.91	1.16	Zhang W et al.	2012	Lithos	142	148-160
1048	W08-294	CAOB	Beishan	99.7492	41.4704	Granite	Dongqiyishan	356	356	1.14	10.40	0.0828	0.512226		-0.58	-8.04	-2.90	-2.86	-2.86	1.34	1.08	1.34	1.08	1.34	Zhang W et al.	2012	Lithos	142	148-160
1049	G-9	CAOB	SMXAB	115.3372	45.6600	Adamellite	Southeast Mongolia	453	453	9.78	47.83	0.1238	0.512449	11	-0.37	-3.69	0.54	0.53	0.53	1.14	1.19	1.14	1.19	1.15	Guo ZH et al.	2013	ournal of Jilin University (Earth Science Edition	43(3)	776-787
1050	G-8	CAOB	SMXAB	115.3372	45.6600	Adamellite	Southeast Mongolia	453	453	5.44	24.99	0.1318	0.512476	13	-0.33	-3.16	0.60	0.59	0.59	1.14	1.25	1.14	1.26	1.14	Guo ZH et al.	2013	ournal of Jilin University (Earth Science Edition	43(3)	776-787
1051	G-7	CAOB	SMXAB	115.3372	45.6600	Adamellite	Southeast Mongolia	453	453	4.18	17.10	0.1480	0.512507	11	-0.25	-2.56	0.27	0.26	0.26	1.16	1.49	1.16	1.49	1.17	Guo ZH et al.	2013	ournal of Jilin University (Earth Science Edition	43(3)	776-787
1052	G-5	CAOB	SMXAB	115.3372	45.6600	Adamellite	Southeast Mongolia	453	453	6.06	28.28	0.1297	0.512486	12	-0.34	-2.97	0.91	0.91	0.91	1.11	1.20	1.11	1.21	1.12	Guo ZH et al.	2013	ournal of Jilin University (Earth Science Edition	43(3)	776-787
1053	G-4	CAOB	SMXAB	115.3372	45.6600	Adamellite	Southeast Mongolia	453	453	2.78	13.81	0.1219	0.512480	20	-0.38	-3.08	1.24	1.24	1.24	1.09	1.11	1.09	1.12	1.09	Guo ZH et al.	2013	ournal of Jilin University (Earth Science Edition	43(3)	776-787
1054	G-10	CAOB	SMXAB	115.3372	45.6600	Adamellite	Southeast Mongolia	453	453	8.07	36.46	0.1339	0.512463	11	-0.32	-3.41	0.23	0.22	0.22	1.17	1.31	1.17	1.31	1.17	Guo ZH et al.	2013	ournal of Jilin University (Earth Science Edition	43(3)	776-787
1055	08KT03-7	Tarim	Tarim	90.1992	46.5856	Granite		275	275	10.90	48.20	0.1175	0.512479		-0.40	-3.10	-0.32	-0.32	-0.32	1.07	1.06	1.07	1.10	1.10	Zhang CL et al.	2010	Lithos	119(3-4)	537-552
1056	08KT03-3	Tarim	Tarim	90.1992	46.5856	Granite		275	275	11.70	54.90	0.0973	0.512439		-0.51	-3.88	-0.39	-0.39	-0.39	1.07	0.93	1.07	0.90	1.10	Zhang CL et al.	2010	Lithos	119(3-4)	537-552
1057	08KT03-1	Tarim	Tarim	90.1992	46.5856	Granite		275	275	10.30	48.90	0.1240	0.512496		-0.37	-2.77	-0.22	-0.22	-0.22	1.06	1.11	1.06	1.10	1.10	Zhang CL et al.	2010	Lithos	119(3-4)	537-552
1058	08KT02-7	Tarim	Tarim	90.1992	46.5856	Granite		275	275	16.70	85.40	0.1357	0.512422		-0.31	-4.21	-2.07	-2.07	-2.07	1.21	1.42	1.21	1.40	1.20	Zhang CL et al.	2010	Lithos	119(3-4)	537-552
1059	08KT02-5	Tarim	Tarim	90.1992	46.5856	Granite		275	275	9.17	45.40	0.1331	0.512431		-0.32	-4.04	-1.81	-1.81	-1.81	1.19	1.36	1.19	1.40	1.20	Zhang CL et al.	2010	Lithos	119(3-4)	537-552
1060	08KT02-4	Tarim	Tarim	90.1992	46.5856	Granite		275	275	14.30	85.70	0.1448	0.512438		-0.26	-3.90	-2.08	-2.08	-2.08	1.21	1.57	1.21	1.60	1.20	Zhang CL et al.	2010	Lithos	119(3-4)	537-552
1061	HLH-6	CAOB	NE China	120.0000	45.0000	Dacite	Huolinhe suite	135	150	5.39	27.62	0.1199	0.512852		-0.39	4.17	5.55	5.64	5.64	0.48	0.49	0.48	0.49		Guo F et al.	2009	International Journal of Earth Sciences	98(6)	1395-1411
1062	HLH-24	CAOB	NE China	120.0000	45.0000	Rhyolite	Huolinhe suite	135	150	2.92	17.16	0.1046	0.512825		-0.47	3.65	5.30	5.41	5.41	0.50	0.45	0.50	0.45		Guo F et al.	2009	International Journal of Earth Sciences	98(6)	1395-1411
1063	HLH-23	CAOB	NE China	120.0000	45.0000	Rhyolite	Huolinhe suite	135	150	2.61	15.63	0.1028	0.512801		-0.48	3.18	4.87	4.98	4.98	0.53	0.48	0.53	0.48		Guo F et al.	2009	International Journal of Earth Sciences	98(6)	1395-1411
1064	HLH-22	CAOB	NE China	120.0000	45.0000	Rhyolite	Huolinhe suite	135	150	3.70	20.81	0.1092	0.512862		-0.44	4.37	5.94	6.04	6.04	0.45	0.42	0.45	0.42		Guo F et al.	2009	International Journal of Earth Sciences	98(6)	1395-1411
1065	HLH-21	CAOB	NE China	120.0000	45.0000	Rhyolite	Huolinhe suite	135	150	3.88	22.04	0.1083	0.512773		-0.45	2.63	4.22	4.33	4.33	0.59	0.55	0.59	0.55		Guo F et al.	2009	International Journal of Earth Sciences	98(6)	1395-1411
1066	HLH-15	CAOB	NE China	120.0000	45.0000	Dacite	Huolinhe suite	135	150	5.63	26.69	0.1297	0.512878		-0.34	4.68	5.88	5.96	5.96	0.45	0.49	0.45	0.49		Guo F et al.	2009	International Journal of Earth Sciences	98(6)	1395-1411
1067	HLH-14	CAOB	NE China	120.0000	45.0000	Dacite	Huolinhe suite	135	150	5.51	26.97	0.1255	0.512779		-0.36	2.75	4.02	4.11	4.11	0.60	0.64	0.60	0.64		Guo F et al.	2009	International Journal of Earth Sciences	98(6)	1395-1411
1068	HLH-13	CAOB	NE China	120.0000	45.0000	Dacite	Huolinhe suite	135	150	5.33	26.96	0.1215	0.512845		-0.38	4.04	5.37	5.48	5.48	0.49	0.50	0.49	0.51		Guo F et al.	2009	International Journal of Earth Sciences	98(6)	1395-1411
1069	JN07-9	NCC	NCC	112.9203	41.5519	Monzonite	Sandaogou	409	409	6.31	29.60	0.1289	0.511808	11	-0.34	-16.19	-12.7	-12.7	-12.7	2.17	2.40	2.17	2.41	2.18	Zhang XH et al.	2010	Journal of the Geological Society	167(4)	717-730
1070	JN07-8	NCC	NCC	112.9203	41.5519	Monzonite	Sandaogou	409	409	4.68	20.11	0.1407	0.511825	10	-0.28	-15.86	-13.0	-12.9	-12.9	2.19	2.75	2.19	2.76	2.20	Zhang XH et al.	2010	Journal of the Geological Society	167(4)	717-730
1071	JN07-53	NCC	NCC	112.8372	41.5444	Pyroxene syenite	Sandaogou	409	409	4.54	27.18	0.1010	0.511466	10	-0.49	-22.86	-17.9	-17.9	-17.9	2.60	2.27	2.60	2.27	2.60	Zhang XH et al.	2010	Journal of the Geological Society	167(4)	717-730
1072	JN07-52	NCC	NCC	112.8372	41.5444	Pyroxene syenite	Sandaogou	409	409	6.71	38.97	0.1040	0.511491	11	-0.47	-22.37	-17.6	-17.5	-17.5	2.57	2.30	2.57	2.30	2.58	Zhang XH et al.	2010	Journal of the Geological Society	167(4)	717-730
1073	JN07-3	NCC	NCC	112.9550	41.5203	Qtz syenite	Sandaogou	409	409	4.15	26.66	0.0941	0.511535	12	-0.52	-21.52	-16.2	-16.2	-16.2	2.46	2.05	2.46	2.05	2.47	Zhang XH et al.	2010	Journal of the Geological Society	167(4)	717-730
1074	JN07-25	NCC	NCC	112.9156	41.5781	Pyroxene syenite	Sandaogou	409	409	5.87	34.05	0.1042	0.511618	11	-0.47	-19.90	-15.1	-15.1	-15.1	2.37	2.12	2.37	2.13	2.38	Zhang XH et al.	2010	Journal of the Geological Society	167(4)	717-730
1075	JN07-23	NCC	NCC	112.9156	41.5781	Pyroxene syenite	Sandaogou	409	409	5.74	33.10	0.1047	0.511617	10	-0.47	-19.92	-15.1	-15.1	-15.1	2.38	2.14	2.38	2.14	2.38	Zhang XH et al.	2010	Journal of the Geological Society	167(4)	717-730
1076	JN07-19	NCC	NCC	112.9217	41.5531	Qtz syenite	Sandaogou	409	409	1.09	4.66	0.1410	0.511621	11	-0.28	-19.84	-17.0	-16.9	-16.9	2.52	3.18	2.52	3.19	2.52	Zhang XH et al.	2010	Journal of the Geological Society	167(4)	717-730
1077	JN07-18	NCC	NCC	112.9217	41.5531	Qtz syenite	Sandaogou	409	409	1.81	8.54	0.1282	0.511632	11	-0.35	-19.62	-16.1	-16.0	-16.0	2.45	2.69	2.45	2.70	2.45	Zhang XH et al.	2010	Journal of the Geological Society	167(4)	717-730
1078	05-05	CAOB	NE China	116.0000	44.0000	Rhyolite		407	407	4.29	20.20	0.1282	0.512112		-0.35	-10.26	-6.7	-6.7	-6.7	1.69	1.85	1.69	1.85	1.70	Liao W et al.	2017	Journal of Asian Earth Sciences	144	40-53
1079	05-04	CAOB	NE China	116.0000	44.0000	Rhyolite		407	407	3.39	16.70	0.1224	0.512089		-0.38	-10.71	-6.86	-6.85	-6.85	1.70	1.77	1.70	1.77	1.71	Liao W et al.	2017	Journal of Asian Earth Sciences	144	40-53
1080	05-03	CAOB	NE China	116.0000	44.0000	Rhyolite		407	407	4.88	26.10	0.1129	0.512043		-0.43	-11.61	-7.26	-7.25	-7.25	1.74	1.67	1.74	1.67	1.74	Liao W et al.	2017	Journal of Asian Earth Sciences	144	40-53

1081	05-02	CAOB	NE China	116.0000	44.0000	Rhyolite		407	407	3.82	21.10	0.1093	0.512018		-0.44	-12.09	-7.56	-7.56	-7.56	1.76	1.65	1.76	1.65	1.77	Liao W et al.	2017	Journal of Asian Earth Sciences	144	40-53
1082	05-01	CAOB	NE China	116.0000	44.0000	Rhyolite		407	407	4.29	23.20	0.1116	0.512020		-0.43	-12.06	-7.64	-7.64	-7.64	1.77	1.68	1.77	1.69	1.77	Liao W et al.	2017	Journal of Asian Earth Sciences	144	40-53
1083	UL-1	CAOB	Altai	89.1000	49.7000	Dacite		389	389	6.90	30.30	0.1382	0.512517		-0.30	-2.36	0.67	0.54	0.54	1.09	1.28	1.09			Krupchatnikov VI et al.	2018	Russian Geology and Geophysics	59（8）	905-924
1084	R-1/10	CAOB	Altai	89.1000	49.7000	Andesite		389	389	3.50	16.30	0.1311	0.512550		-0.33	-1.72	1.68	1.54	1.54	1.01	1.11	1.01			Krupchatnikov VI et al.	2018	Russian Geology and Geophysics	59（8）	905-924
1085	30061	CAOB	Altai	89.1000	49.7000	Dacite		389	389	7.10	29.40	0.1470	0.512480		-0.25	-3.08	-0.51	-0.62	-0.62	1.18	1.53	1.18			Krupchatnikov VI et al.	2018	Russian Geology and Geophysics	59（8）	905-924
1086	30002	CAOB	Altai	89.1000	49.7000	Dacite		389	389	4.60	14.60	0.1887	0.512641		-0.04	0.06	0.47	0.46	0.46	1.09	3.08	1.09			Krupchatnikov VI et al.	2018	Russian Geology and Geophysics	59（8）	905-924
1087	07236-1	NCC	NCC	110.2355	40.5697	Syenite	Yongfucun	230	230	14.96	74.92	0.1209	0.512029	14	-0.39	-11.88	-9.65	-9.66	-9.66	1.79	1.84	1.79	1.84	1.79	Zhang SH et al.	2012	Lithos	155	1-18
1088	07235-1	NCC	NCC	110.2246	40.5708	Syenite	Yongfucun	230	230	16.72	85.89	0.1179	0.512031	12	-0.40	-11.84	-9.52	-9.53	-9.53	1.78	1.78	1.78	1.78	1.78	Zhang SH et al.	2012	Lithos	155	1-18
1089	07-1a	CAOB	Kazakhstan	80.8333	44.8667	Ms-Chl schist		500	500	5.74	28.90	0.1204	0.511869		-0.39	-15.00	-10.1	-10.1	-10.1	2.05	2.09	2.05		2.09	Pilitsyna AV et al.	2018	Lithos	324	115-140
1090	Z 12371/2	CAOB	Russia	75.4950	44.0006	Hb-Bi orthogneiss		1840	600	7.60	40.60	0.1131	0.511619		-0.43	-19.88	-0.1	-13.5	-13.5	2.40	2.31	2.40		2.31	Pilitsyna AV et al.	2018	Lithos	324-324	115-140
1091	Z 12371	CAOB	Russia	75.4950	44.0006	Hb-Bi orthogneiss		1840	600	18.73	92.10	0.1230	0.511547		-0.37	-21.28	-3.9	-15.6	-15.6	2.57	2.68	2.57		2.68	Pilitsyna AV et al.	2018	Lithos	324-324	115-140
1092	TS 1193/1	CAOB	Russia	75.4508	43.9714	Hb-Bi orthogneiss		1840	600	12.13	61.90	0.1186	0.511474		-0.40	-22.71	-4.3	-16.7	-16.7	2.66	2.67	2.66			Pilitsyna AV et al.	2018	Lithos	324-325	115-140
1093	TS 1191/1	CAOB	Russia	75.4508	43.9714	Hb-Bi orthogneiss		1840	600	16.60	83.90	0.1196	0.511552		-0.39	-21.18	-3.0	-15.3	-15.3	2.54	2.57	2.54			Pilitsyna AV et al.	2018	Lithos	324-325	115-140
1094	TS 1185	CAOB	Russia	75.4508	43.9714	Ms-Chl orthogneiss		800	600	4.60	27.07	0.1026	0.511568		-0.48	-20.88	-11.3	-13.7	-13.7	2.41	2.16	2.41			Pilitsyna AV et al.	2018	Lithos	324-325	115-140
1095	P 10092	CAOB	Russia	75.4950	44.0006	Ms-Chl orthogneiss		800	600	4.69	30.10	0.0942	0.511531		-0.52	-21.59	-11.1	-13.7	-13.7	2.42	2.06	2.42			Pilitsyna AV et al.	2018	Lithos	324-325	115-140
1096	P 10091/1	CAOB	Russia	75.4950	44.0006	Ms-Chl orthogneiss		800	600	3.67	19.22	0.1154	0.511682		-0.41	-18.65	-10.4	-12.4	-12.4	2.31	2.27	2.31			Pilitsyna AV et al.	2018	Lithos	324-325	115-140
1097	AH 1514	CAOB	Russia	75.6125	43.9144	Hb-Bi orthogneiss		1840	600	18.01	89.50	0.1216	0.511552		-0.38	-21.18	-3.5	-15.4	-15.4	2.55	2.63	2.55			Pilitsyna AV et al.	2018	Lithos	324-325	115-140
1098	AH 1470	CAOB	Russia	75.4911	44.0128	Grt-mica schist		500	500	6.99	35.90	0.1178	0.511658		-0.40	-19.12	-14.1	-14.1	-14.1	2.36	2.36	2.36			Pilitsyna AV et al.	2018	Lithos	324-325	115-140
1099	AH 1320	CAOB	Russia	75.6125	43.9144	Ms-Chl schist		500	500	5.74	28.90	0.1204	0.511869		-0.39	-15.00	-10.1	-10.1	-10.1	2.05	2.09	2.05			Pilitsyna AV et al.	2018	Lithos	324-325	115-140
1100	AH 1319	CAOB	Russia	75.6125	43.9144	Hb-Bi orthogneiss		1840	600	1.21	6.63	0.1105	0.511585		-0.44	-20.54	-0.2	-13.9	-13.9	2.44	2.30	2.44			Pilitsyna AV et al.	2018	Lithos	324-325	115-140
1101	AH 1317/1	CAOB	Russia	75.6125	43.9144	Hb-Bi orthogneiss		1840	600	14.30	84.70	0.1021	0.511413		-0.48	-23.90	-1.6	-16.7	-16.7	2.65	2.36	2.65			Pilitsyna AV et al.	2018	Lithos	324-325	115-140
1102	AH 1316	CAOB	Russia	75.6125	43.9144	Hb-Bi orthogneiss		1840	600	18.66	94.00	0.1200	0.511588		-0.39	-20.48	-2.4	-14.6	-14.6	2.49	2.53	2.49			Pilitsyna AV et al.	2018	Lithos	324-325	115-140
1103	Z12371	CAOB	Southern Kazakhsta	75.4545	43.8801	Granite		1840	600	18.73	92.10	0.1230	0.511547		-0.37	-21.28	-3.9	-15.6	-15.6	2.57	2.68	2.57	2.68		Pilitsyna AV et al.	2019	Lithos	324	115-140
1104	TS1185	CAOB	Southern Kazakhsta	75.4542	43.8922	Granite		800	600	4.60	27.07	0.1026	0.511568		-0.48	-20.88	-11.3	-13.7	-13.7	2.41	2.16	2.41	2.17		Pilitsyna AV et al.	2019	Lithos	324	115-140
1105	X3ET347	CAOB	East Tianshan	91.7808	42.6167	Granite		447	447	3.98	16.50	0.1460	0.512845	0	-0.26	4.04	6.95	6.93	6.93	0.62	0.69	0.62		0.69	Du L et al.	2017	Journal of Asian Earth Sciences	153	139-153
1106	X3ET344	CAOB	East Tianshan	91.7808	42.6167	Granite		447	447	3.44	12.50	0.1670	0.512883	0	-0.15	4.78	6.49	6.47	6.47	0.65	0.87	0.65		0.87	Du L et al.	2017	Journal of Asian Earth Sciences	153	139-153
1107	X3ET341	CAOB	East Tianshan	91.7808	42.6167	Granite		447	447	3.93	15.80	0.1510	0.512855	0	-0.23	4.23	6.88	6.84	6.84	0.62	0.72	0.62		0.72	Du L et al.	2017	Journal of Asian Earth Sciences	153	139-153
1108	X3ET-408	CAOB	Tianshan	93.7168	43.2391	Granite		452	452	3.67	14.40	0.1540	0.512771		-0.22	2.59	5.08	5.06	5.06	0.77	0.97	0.77	0.97		Du L et al.	2017	Journal of Asian Earth Sciences	153	139-153
1109	X3ET-406	CAOB	Tianshan	93.7168	43.2391	Granite		452	452	2.90	10.60	0.1650	0.512804		-0.16	3.24	5.09	5.07	5.07	0.77	1.08	0.77	1.08		Du L et al.	2017	Journal of Asian Earth Sciences	153	139-153
1110	X3ET-359	CAOB	Tianshan	91.9550	42.5837	Diorite		442	442	4.08	17.50	0.1420	0.512815		-0.28	3.45	6.58	6.54	6.54	0.64	0.71	0.64	0.71		Du L et al.	2017	Journal of Asian Earth Sciences	153	139-153
1111	X3ET-356	CAOB	Tianshan	91.9550	42.5837	Diorite		442	442	4.38	19.80	0.1340	0.512770		-0.32	2.57	6.14	6.11	6.11	0.68	0.73	0.68	0.73		Du L et al.	2017	Journal of Asian Earth Sciences	153	139-153
1112	X3ET-347	CAOB	Tianshan	91.7808	42.6167	Granite		447	447	3.98	16.50	0.1460	0.512845		-0.26	4.04	6.95	6.93	6.93	0.62	0.69	0.62	0.69		Du L et al.	2017	Journal of Asian Earth Sciences	153	139-153
1113	X3ET-344	CAOB	Tianshan	91.7808	42.6167	Granite		447	447	3.44	12.50	0.1670	0.512883		-0.15	4.78	6.49	6.47	6.47	0.65	0.87	0.65	0.87		Du L et al.	2017	Journal of Asian Earth Sciences	153	139-153
1114	X3ET-341	CAOB	Tianshan	91.7808	42.6167	Granite		447	447	3.93	15.80	0.1510	0.512855		-0.23	4.23	6.88	6.84	6.84	0.62	0.72	0.62	0.72		Du L et al.	2017	Journal of Asian Earth Sciences	153	139-153
1115	HK-65	CAOB	Altai, unit 8	93.8333	43.1833	Diorite	Orda udek	450	450	2.95	11.55	0.1620	0.512800	3	-0.18	3.16	5.10	5.15	5.15	0.76	1.03	0.76		0.77	Ma XH et al.	2015	Acta Petrologica Sinica	31(1)	89-104
1116	HK-64	CAOB	Altai, unit 8	93.8333	43.1833	Dioritic inclusion	Orda udek	450	450	2.73	9.88	0.1754	0.512800	3	-0.11	3.16	5.30	4.38	4.38	0.83	1.39	0.83		0.75	Ma XH et al.	2015	Acta Petrologica Sinica	31(1)	89-104
1117	HK-63	CAOB	Altai, unit 8	93.8333	43.1833	Dioritic inclusion	Orda udek	450	450	9.68	45.30	0.1356	0.512700	5	-0.31	1.21	5.40	4.72	4.72	0.80	0.88	0.80		0.75	Ma XH et al.	2015	Acta Petrologica Sinica	31(1)	89-104
1118	HK-62	CAOB	Altai, unit 8	93.8333	43.1833	Diorite	Orda udek	450	450	5.63	22.02	0.1622	0.513000	4	-0.18	7.06	8.20	9.04	9.04	0.45	0.44	0.45		0.52	Ma XH et al.	2015	Acta Petrologica Sinica	31(1)	89-104
1119	HK-61	CAOB	Altai, unit 8	93.8333	43.1833	Granodiorite	Orda udek	450	450	2.61	9.56	0.1729	0.512800	4	-0.12	3.16	4.40	4.53	4.53	0.81	1.31	0.81		0.83	Ma XH et al.	2015	Acta Petrologica Sinica	31(1)	89-104
1120	HK-60	CAOB	Altai, unit 8	93.8333	43.1833	Dioritic inclusion	Orda udek	450	450	3.24	11.53	0.1786	0.512900	5	-0.09	5.11	5.50	6.15	6.15	0.68	1.09	0.68		0.74	Ma XH et al.	2015	Acta Petrologica Sinica	31(1)	89-104
1121	XT26	CAOB	Tianshan	81.0000	42.0000	Granodiorite		470	470	2.57	13.40	0.1177	0.512194		-0.40	-8.66	-4.46	-3.92	-3.92	1.52	1.52	1.52	1.44		Qian Q et al.	2009	International Journal of Earth Sciences	98（3）	551-569
1122	XT24	CAOB	Tianshan	81.0000	42.0000	Monzodiorite		513	513	4.76	15.50	0.1164	0.512855		-0.41	4.23	-3.85	9.49	9.49	0.47	0.46	0.46	1.38		Qian Q et al.	2009	International Journal of Earth Sciences	98（3）	551-569
1123	XT28	CAOB	Southern margin of Yili block	80.6000	42.6000	Granite		470	470	3.41	15.90	0.1298	0.512365		-0.34	-5.33	-1.31	-1.31	-1.31	1.31	1.42	1.31	1.43	1.31	Wang Q et al.	2009	International Journal of Earth Sciences	98(3)	551-569
1124	DV49-5	CAOB	Southern margin of Yili block	80.5833	42.6000	Granite		470	470			0.1176	0.512212		-0.40	-8.31	-3.56	-3.57	-3.57	1.49	1.49	1.49	1.49	1.50	Qian Q et al.	2009	International Journal of Earth Sciences	98(3)	551-569
1125	DV49-4	CAOB	Southern margin of Yili block	80.5833	42.6000	Granite		470	470			0.1198	0.512221		-0.39	-8.13	-3.52	-3.52	-3.52	1.49	1.50	1.49	1.51	1.50	Qian Q et al.	2009	International Journal of Earth Sciences	98(3)	551-569
1126	SN10-9-1	CAOB	SMXAB	113.6831	44.4753	Alkaline granite	Baiyinwula	290	290	9.81	46.37	0.1280	0.512767	12	-0.35	2.52	5.06	5.06	5.06	0.64	0.68	0.64	0.68	0.64	Zhang XH et al.	2015	Gondwana Research	28(1)	311-327
1127	SN10-8-1	CAOB	SMXAB	113.6831	44.4753	Alkaline granite	Baiyinwula	290	290	10.82	45.63	0.1435	0.512755	14	-0.27	2.28	4.26	4.25	4.25	0.71	0.86	0.71	0.86	0.71	Zhang XH et al.	2015	Gondwana Research	28(1)	311-327
1128	SN10-7-4	CAOB	SMXAB	113.7300	44.4892	Alkaline granite	Baiyinwula	288	288	11.85	52.87	0.1356	0.512779	15	-0.31	2.75	4.99	5.00	5.00	0.64	0.72	0.64	0.73	0.65	Zhang XH et al.	2015	Gondwana Research	28(1)	311-327
1129	SN10-7-3	CAOB	SMXAB	113.7300	44.4892	Alkaline granite	Baiyinwula	288	288	8.37	40.14	0.1263	0.512786	14	-0.36	2.89	5.48	5.47	5.47	0.61	0.64	0.61	0.64	0.61	Zhang XH et al.	2015	Gondwana Research	28(1)	311-327
1130	SN10-7-1	CAOB	SMXAB	113.7300	44.4892	Alkaline granite	Baiyinwula	288	288	8.95	37.00	0.1465	0.512794	12	-0.26	3.04	4.90	4.89	4.89	0.65	0.81	0.65	0.81	0.66	Zhang XH et al.	2015	Gondwana Research	28(1)	311-327
1131	SN10-3-1	CAOB	SMXAB	113.7300	44.4892	Alkaline granite	Baiyinwula	288	288	12.51	63.18	0.1199	0.512795	12	-0.39	3.06	5.89	5.89	5.89	0.57	0.58	0.57	0.58	0.57	Zhang XH et al.	2015	Gondwana Research	28(1)	311-327
1132	SN10-2-3	CAOB	SMXAB	113.5597	44.4544	Alkaline granite	Baiyinwula	289	289	11.11	56.19	0.1197	0.512775	14	-0.39	2.67	5.53	5.51	5.51	0.60	0.61	0.60	0.61	0.61	Zhang XH et al.	2015	Gondwana Research	28(1)	311-327
1133	SN10-2-1	CAOB	SMXAB	113.5597	44.4544	Alkaline granite	Baiyinwula	289	289	12.93	67.58	0.1158	0.512795	10	-0.41	3.06	6.05	6.05	6.05	0.56	0.55	0.56	0.56	0.56	Zhang XH et al.	2015	Gondwana Research	28(1)	311-327
1134	SN10-12-1	CAOB	SMXAB	113.2703	44.2981	Alkaline granite	Baiyinwula	290	290	7.96	35.81	0.1345	0.512788	12	-0.32	2.93	5.24	5.23	5.23	0.63	0.70	0.63	0.70	0.63	Zhang XH et al.	2015	Gondwana Research	28(1)	311-327
1135	SN10-11-1	CAOB	SMXAB	113.3336	44.3414	Alkaline granite	Baiyinwula	290	290	8.48	34.47	0.1490	0.512824	12	-0.24	3.63	5.40	5.39	5.39	0.61	0.77	0.61	0.77	0.62	Zhang XH et al.	2015	Gondwana Research	28(1)	311-327
1136	SN10-10-2	CAOB	SMXAB	113.3336	44.3414	Alkaline granite	Baiyinwula	290	290	9.51	41.53	0.1386	0.512762	12	-0.30	2.42	4.58	4.57	4.57	0.68	0.79	0.68	0.79	0.68	Zhang XH et al.	2015	Gondwana Research	28(1)	311-327
1137	SN10-10-1	CAOB	SMXAB	113.3336	44.3414	Alkaline granite	Baiyinwula	290	290	9.65	41.69	0.1402	0.512806	13	-0.29	3.28	5.38	5.37	5.37	0.62	0.71	0.62	0.72	0.62	Zhang XH et al.	2015	Gondwana Research	28(1)	311-327
1138	SN07-22	CAOB	NE China	113.3000	44.3000	Rhyolite		287	287	14.60	85.40	0.1037	0.512760		-0.47	2.38	5.81	5.79	5.79	0.58	0.54	0.58	0.54		Zhang XH et al.	2012	Journal of the Geological Society	168（2）	525-543
1139	SN07-7	CAOB	NE China	113.3000	44.3000	Rhyolite		287	287	4.23	23.90	0.1071	0.512870		-0.46	4.53	5.43	7.81	7.81	0.41	0.40	0.41	0.58		Zhang XH et al.	2012	Journal of the Geological Society	168（2）	525-543
1140	BY04-5	CAOB	NE China	113.2000	44.4000	Rhyolite		287	287	2.70	14.00	0.1168	0.512910		-0.41	5.31	5.79	8.23	8.23	0.38	0.38	0.38	0.55		Zhang XH et al.	2012	Journal of the Geological Society	168（2）	525-543
1141	BY04-4	CAOB	NE China	113.2000	44.4000	Rhyolite		287	287	2.82	14.40	0.1184	0.512820		-0.40	3.55	6.05	6.42	6.42	0.53	0.53	0.53	0.54		Zhang XH et al.	2012	Journal of the Geological Society	168（2）	525-543
1142	BY04-1	CAOB	NE China	113.2000	44.4000	Rhyolite		287	287	2.84	14.70	0.1167	0.512800		-0.41	3.16	5.87	6.09	6.09	0.55	0.55	0.55	0.55		Zhang XH et al.	2012	Journal of the Geological Society	168（2）	525-543
1143	BY04-2	CAOB	NE China	113.0000	44.0000	Basaltic andesite	Sonidquozi	290	290	8.294	35.1	0.1432	0.5129		-0.27	5.11	6.79	7.09	7.09	0.48	0.54	0.48	0.55		Zhang XH et al.	2011	Journal of the Geological Society	168(2)	525-543
1144	SN07-30	CAOB	NE China	113.0000	44.0000	Andesite	Sonidquozi	290	290	3.96	20.1	0.1190	0.51275		-0.40	2.18	5.01	5.06	5.06	0.64	0.64	0.64	0.65		Zhang XH et al.	2011	Journal of the Geological Society	168(2)	525-543
1145	SN07-10	CAOB	NE China	113.0000	44.0000	Basaltic andesite	Sonidquozi	290	290	5.01	26.1	0.1160	0.51273		-0.41	1.79	4.67	4.78	4.78	0.66	0.66	0.66	0.66		Zhang XH et al.	2011	Journal of the Geological Society	168(2)	525-543
1146	D467	NCC	NCC	116.9611	40.9864	Diorite	Tianqiao pluton	285	285	6.47	30.59	0.1279	0.511781	13	-0.35	-16.72	-14.2	-14.2	-14.2	2.20	2.42	2.20	2.43	2.21	Zhang SH et al.	2009	International Journal of Earth Sciences	98(6)	1441-1469
1147	D464	NCC	NCC	116.7522	40.9581	Diorite	XianghuangqiWudaoyingzi	280	280	3.74	19.88	0.1138	0.511817	13	-0.42	-16.02	-13.1	-13.1	-13.1	2.10	2.03	2.10	2.03	2.11	Zhang SH et al.	2009	International Journal of Earth Sciences	98(6)	1441-1469
1148	D461	NCC	NCC	116.7769	40.9414	Diorite	XianghuangqiWudaoyingzi	280	280	5.40	31.21	0.1046	0.511794	12	-0.47	-16.46	-13.2	-13.2	-13.2	2.11	1.89	2.11	1.89	2.12	Zhang SH et al.	2009	International Journal of Earth Sciences	98(6)	1441-1469
1149	D215-1	NCC	NCC	117.5583	40.9847	Qtz diorite	Zhoutaizi	291	291	3.37	20.27	0.1004	0.511889	11	-0.49	-14.61	-11.0	-11.0	-11.0	1.95	1.69	1.95	1.70	1.95	Zhang SH et al.	2009	International Journal of Earth Sciences	98(6)	1441-1469
1150	D079-1	NCC	NCC	117.1047	41.0269	Granite	Lingying	288	288	2.72	20.46	0.0803	0.511526	14	-0.59	-21.69	-17.4	-17.4	-17.4	2.47	1.85	2.47	1.85	2.47	Zhang SH et al.	2009	International Journal of Earth Sciences	98(6)	1441-1469
1151	D077-1	NCC	NCC	116.9744	40.9942	Diorite	Tianqiao	285	285	6.00	32.83	0.1105	0.511903	13	-0.44	-14.34	-11.2	-11.2	-11.2	1.96	1.84	1.96	1.84	1.96	Zhang SH et al.	2009	International Journal of Earth Sciences	98(6)	1441-1469
1152	B80628-8.2	CAOB	Beishan	95.8986	41.2472	Syenogranite	Wufengshan	275	275	5.56	24.1	0.1395	0.512598	14	-0.29	-0.78	1.20	1.23	1.23	0.94	1.13	0.94	1.13		Li S et al.	2013	Lithos	179	99-119
1153	DDQ09824-2.1	CAOB	Beishan	96.1597	41.2103	Mylonitic granodiorite	Dongdaquan	279	279	6.25	31.8	0.1190	0.51237	10	-0.40	-5.23	-2.50	-2.46	-2.46	1.24	1.25	1.24	1.25		Li S et al.	2013	Lithos	179	99-119
1154	DDQ09824-1.1	CAOB	Beishan	96.0961	41.1683	Syenogranite	Dongdaquan	279	279	14.2	77.5	0.1108	0.512436	5	-0.44	-3.94	-0.90	-0.88	-0.88	1.12	1.06	1.12	1.06		Li S et al.	2013	Lithos	179	99-119
1155	08FY-05	CAOB	Altai	89.6500	46.5000	Dacite		440	440	3.72	17.80	0.1263	0.512770		-0.36	2.57	6.50	6.53	6.53	0.64	0.66	0.64	0.67		Shen XM et al.	2021	Lithos	206-207	234-251
1156	WuerkashierN-04	CAOB	Western Junggar	84.3917	46.6278	Granite		416	416	6.95	31.20	0.1347	0.512870	7	-0.32	4.53	7.53	7.82	7.82	0.52	0.54	0.52	0.59	0.55	Zhang C et al.	2018	Lithos	300(13)	314-329
1157	WuerkashierN-03	CAOB	Western Junggar	84.3917	46.6278	Granite		416	416	8.07	39.70	0.1229	0.512872	4	-0.38	4.56	8.21	8.48	8.48	0.46	0.47	0.46	0.50	0.49	Zhang C et al.	2018	Lithos	300(13)	314-329
1158	WuerkashierN-02	CAOB	Western Junggar	84.3917	46.6278	Granite		416	416	7.75	34.90	0.1343	0.512869	7	-0.32	4.51	7.53	7.82	7.82	0.52	0.54	0.52	0.58	0.55	Zhang C et al.	2018	Lithos	300(13)	314-329
1159	WuerkashierN-01	CAOB	Western Junggar	84.3917	46.6278	Granite		416	416	7.78	35.20	0.1336	0.512873	5	-0.32	4.58	7.65	7.93	7.93	0.51	0.53	0.51	0.57	0.54	Zhang C et al.	2018	Lithos	300(13)	314-329
1160	WuerkashierE-04	CAOB	Western Junggar	84.1250	46.5111	Granite		380	380	5.82	24.30	0.1448	0.512792	8	-0.26	3.00	5.24	5.52	5.52	0.68	0.79	0.68	0.87	0.70	Zhang C et al.	2018	Lithos	300(13)	314-329
1161	WuerkashierE-03	CAOB	Western Junggar	84.1250	46.5111	Granite		380	380	6.79	30.30	0.1355	0.512791	8	-0.31	2.98	5.68	5.95	5.95	0.64	0.70	0.64	0.76	0.67	Zhang C et al.	2018	Lithos	300(13)	314-329
1162	WuerkashierE-02	CAOB	Western Junggar	84.1250	46.5111	Granite		380	380	6.87	30.50	0.1362	0.512794	9	-0.31	3.04	5.71	5.98	5.98	0.64	0.70	0.64	0.76	0.66	Zhang C et al.	2018	Lithos	300(13)	314-329
1163	WuerkashierE-01	CAOB	Western Junggar	84.1250	46.5111	Granite		380	380	6.35	28.10	0.1366	0.512795	8	-0.31	3.06	5.70	5.97	5.97	0.64	0.70	0.64	0.76	0.67	Zhang C et al.	2018	Lithos	300(13)	314-329
1164	WJ1125-1	CAOB	Western Junggar	85.5056	46.7106	K-feldspar granite		420	420	11.61	61.76	0.1136	0.512702	0	-0.42	1.25	5.71	5.70	5.70	0.69	0.68	0.69		0.70	Zhang C et al.	2018	Lithos	300(13)	314-329
1165	Taerbaha-04	CAOB	Western Junggar	86.5564	50.2258	Granite		328	328	6.78	26.00	0.1577	0.512812	5	-0.20	3.39	4.76	5.03	5.03	0.67	0.92	0.67	1.05	0.70	Zhang C et al.	2018	Lithos	300(13)	314-329
1166	Taerbaha-03	CAOB	Western Junggar	85.5394	49.2089	Granite		328	328	8.33	31.60	0.1594	0.512815	8	-0.19	3.45	4.75	5.01	5.01	0.68	0.94	0.68	1.08	0.70	Zhang C et al.	2018	Lithos	300(13)	314-329
1167	Taerbaha-02	CAOB	Western Junggar	84.5225	48.1919	Granite		328	328	7.60	28.40	0.1618	0.512813	7	-0.18	3.41	4.59	4.87	4.87	0.69	0.99	0.69	1.14	0.71	Zhang C et al.	2018	Lithos	300(13)	314-329
1168	Qibaertie-04	CAOB	Western Junggar	84.5111	46.6667	Granite		339	339	5.35	24.40	0.1326	0.512762	4	-0.33	2.42	4.97	5.20	5.20	0.67	0.73	0.67	0.79	0.69	Zhang C et al.	2018	Lithos	300(13)	314-329
1169	Qibaertie-03	CAOB	Western Junggar	84.5111	46.6667	Granite		339	339	5.73	26.80	0.1293	0.512763	7	-0.34	2.44	5.13	5.36	5.36	0.66	0.70	0.66	0.75	0.68	Zhang C et al.	2018	Lithos	300(13)	314-329
1170	Qibaertie-02	CAOB	Western Junggar	84.5111	46.6667	Granite		339	339	4.92	23.40	0.1271	0.512765	5	-0.35	2.48	5.26	5.49	5.49	0.65	0.68	0.65	0.73	0.67	Zhang C et al.	2018	Lithos	300(13)	314-329
1171	Qibaertie-01	CAOB	Western Junggar	84.5111	46.6667	Granite		339	339	5.15	25.20	0.1236	0.512766	8	-0.37	2.50	5.44	5.66	5.66	0.63	0.65	0.63	0.69	0.65	Zhang C et al.	2018	Lithos	300(13)	314-329
1172	KujibeiEc-04	CAOB	Western Junggar	84.3056	46.8389	Granite		330	330	9.87	47.60	0.1520	0.512804	8	-0.23	3.24	4.82	5.12	5.12	0.67	0.86	0.67	0.96	0.70	Zhang C et al.	2018	Lithos	300(13)	314-329
1173	KujibeiEc-03	CAOB	Western Junggar	84.3056	46.8389	Granite		330	330	7.51	34.90	0.1587	0.512802	4	-0.19	3.20	4.57	4.80	4.80	0.69	0.96	0.69	1.10	0.72	Zhang C et al.	2018	Lithos	300(13)	314-329
1174	KujibeiEc-02	CAOB	Western Junggar	84.3056	46.8389	Granite		330	330	8.20	35.60	0.1594	0.512801	7	-0.19	3.18	4.47	4.75	4.75	0.70	0.98	0.70	1.12	0.72	Zhang C et al.	2018	Lithos	300(13)	314-329
1175	KujibeiEc-01	CAOB	Western Junggar	84.3056	46.8389	Granite		330	330	8.10	38.00	0.1554	0.512805	9	-0.21	3.26	4.72	5.00	5.00	0.68	0.90	0.68	1.02	0.70	Zhang C et al.	2018	Lithos	300(13)	314-329
1176	KujibeiEb-04	CAOB	Western Junggar	84.0083	46.8417	Granite		341	341	6.78	26.00	0.1357	0.512748	8	-0.31	2.15	4.55	4.80	4.80	0.70	0.79	0.70	0.85	0.73	Zhang C et al.	2018	Lithos	300(13)	314-329
1177	KujibeiEb-03	CAOB	Western Junggar	84.0083	46.8417	Granite		341	341	8.33	31.60	0.1403	0.512751	7	-0.29	2.20	4.41	4.66	4.66	0.72	0.83	0.72	0.90	0.74	Zhang C et al.	2018	Lithos	300(13)	314-329
1178	KujibeiEb-02	CAOB	Western Junggar	84.0083	46.8417	Granite		341	341	7.60	28.40	0.1340	0.512749	4	-0.32	2.17	4.64	4.89	4.89	0.70	0.77	0.70	0.83	0.72	Zhang C et al.	2018	Lithos	300(13)	314-329

1179	KujibeiEb-01	CAOB	Western Junggar	84.0083	46.8417	Granite		341	341	7.51	29.90	0.1302	0.512752	7	-0.34	2.22	4.88	5.12	5.12	0.68	0.73	0.68	0.78	0.70	Zhang C et al.	2018	Lithos	300(13)	314-329
1180	KujibeiEa-04	CAOB	Western Junggar	83.8417	46.8417	Granite		329	329	9.87	47.60	0.1254	0.512744	8	-0.36	2.07	4.85	5.06	5.06	0.67	0.70	0.67	0.75	0.69	Zhang C et al.	2018	Lithos	300(13)	314-329
1181	KujibeiEa-03	CAOB	Western Junggar	83.8417	46.8417	Granite		329	329	7.51	34.90	0.1301	0.512747	7	-0.34	2.13	4.69	4.92	4.92	0.68	0.74	0.68	0.79	0.70	Zhang C et al.	2018	Lithos	300(13)	314-329
1182	KujibeiEa-02	CAOB	Western Junggar	83.8417	46.8417	Granite		329	329	8.20	35.60	0.1393	0.512745	5	-0.29	2.09	4.26	4.50	4.50	0.72	0.83	0.72	0.90	0.74	Zhang C et al.	2018	Lithos	300(13)	314-329
1183	KujibeiEa-01	CAOB	Western Junggar	83.8417	46.8417	Granite		329	329	8.10	38.00	0.1289	0.512748	4	-0.34	2.15	4.77	4.99	4.99	0.68	0.72	0.68	0.78	0.70	Zhang C et al.	2018	Lithos	300(13)	314-329
1184	HebukesairSW-04	CAOB	Western Junggar	84.7556	46.5639	Granite		434	434	3.63	18.60	0.1180	0.512844	9	-0.40	4.02	8.12	8.37	8.37	0.49	0.49	0.49	0.52	0.51	Zhang C et al.	2018	Lithos	300(13)	314-329
1185	HebukesairSW-03	CAOB	Western Junggar	84.7556	46.5639	Granite		434	434	3.42	16.90	0.1224	0.512843	8	-0.38	4.00	7.85	8.11	8.11	0.51	0.51	0.51	0.55	0.54	Zhang C et al.	2018	Lithos	300(13)	314-329
1186	HebukesairSW-02	CAOB	Western Junggar	84.7556	46.5639	Granite		434	434	3.77	19.40	0.1175	0.512846	7	-0.40	4.06	8.18	8.44	8.44	0.48	0.48	0.48	0.51	0.51	Zhang C et al.	2018	Lithos	300(13)	314-329
1187	HebukesairSW-01	CAOB	Western Junggar	84.7556	46.5639	Granite		434	434	3.87	19.30	0.1212	0.512847	4	-0.38	4.08	7.98	8.25	8.25	0.50	0.50	0.50	0.53	0.52	Zhang C et al.	2018	Lithos	300(13)	314-329
1188	HebukesairN-04	CAOB	Western Junggar	85.0083	46.5278	Granite		346	346	4.70	25.10	0.1132	0.512878	8	-0.42	4.68	8.17	8.37	8.37	0.42	0.41	0.42	0.44	0.44	Zhang C et al.	2018	Lithos	300(13)	314-329
1189	HebukesairN-03	CAOB	Western Junggar	85.0083	46.5278	Granite		346	346	5.06	25.60	0.1195	0.512881	8	-0.39	4.74	7.94	8.15	8.15	0.43	0.44	0.43	0.46	0.46	Zhang C et al.	2018	Lithos	300(13)	314-329
1190	HebukesairN-02	CAOB	Western Junggar	85.0083	46.5278	Granite		346	346	4.82	25.00	0.1166	0.512880	4	-0.41	4.72	8.06	8.26	8.26	0.43	0.42	0.43	0.45	0.45	Zhang C et al.	2018	Lithos	300(13)	314-329
1191	HebukesairN-01	CAOB	Western Junggar	85.0083	46.5278	Granite		346	346	4.73	25.30	0.1130	0.512882	7	-0.43	4.76	8.25	8.46	8.46	0.41	0.41	0.41	0.43	0.43	Zhang C et al.	2018	Lithos	300(13)	314-329
1192	SH-986	CAOB	Russia	86.0500	51.0667	Granite		512	512	1.58	8.00	0.1180	0.512715	12	-0.40	1.50	6.70	6.64	6.64	0.69	0.69	0.69		0.86	Kruk NN et al.	2011	Journal of Asian Earth Sciences	42(5)	928-948
1193	SH-664	CAOB	Russia	87.0667	52.0833	Granite		400	400	9.29	41.79	0.1335	0.512372	9	-0.32	-5.19	8.66	-1.96	-1.96	1.30	1.48	1.30		0.99	Kruk NN et al.	2011	Journal of Asian Earth Sciences	42(5)	928-948
1194	SH-1722	CAOB	Russia	86.0500	51.0833	Granite		402	402	5.38	28.00	0.1153	0.512684	5	-0.41	0.90	4.40	5.07	5.07	0.73	0.72	0.73		1.08	Kruk NN et al.	2011	Journal of Asian Earth Sciences	42(5)	928-948
1195	RA-42/3	CAOB	Russia	86.0500	52.0500	Granite		374	374	4.15	19.18	0.1307	0.512444	8	-0.34	-3.78	7.87	-0.63	-0.63	1.17	1.29	1.17		0.94	Kruk NN et al.	2011	Journal of Asian Earth Sciences	42(5)	928-948
1196	RA-3/3	CAOB	Russia	87.0833	52.0667	Granodiorite		364	364	5.06	23.40	0.1309	0.512571	9	-0.33	-1.31	1.70	1.75	1.75	0.97	1.07	0.97		1.35	Kruk NN et al.	2011	Journal of Asian Earth Sciences	42(5)	928-948
1197	9-04-8/4	CAOB	Russia	86.0500	51.1167	Granodiorite		380	380	4.01	19.36	0.1252	0.512572	20	-0.36	-1.29	2.20	2.18	2.18	0.95	1.00	0.95		0.92	Kruk NN et al.	2011	Journal of Asian Earth Sciences	42(5)	928-948
1198	8-749	CAOB	Russia	86.0500	52.0667	Granite		390	390	3.60	17.30	0.1244	0.512650	9	-0.37	0.23	3.80	3.83	3.83	0.82	0.85	0.82		0.73	Kruk NN et al.	2011	Journal of Asian Earth Sciences	42(5)	928-948
1199	8-729	CAOB	Russia	86.0500	52.0500	Qtz diorite		509	509	1.29	3.94	0.1983	0.512490	16	0.01	-2.89	5.80	-2.99	-2.99	1.45	6.41	1.45		0.70	Kruk NN et al.	2011	Journal of Asian Earth Sciences	42(5)	928-948
1200	8-724	CAOB	Russia	86.0500	51.1000	Granodiorite		390	390	7.00	38.00	0.1104	0.512499	5	-0.44	-2.71	1.70	1.58	1.58	1.01	0.96	1.01		0.88	Kruk NN et al.	2011	Journal of Asian Earth Sciences	42(5)	928-948
1201	8-717_	CAOB	Russia	86.0500	52.1000	Granite		374	374	4.65	22.60	0.1231	0.512626	8	-0.37	-0.23	3.30	3.28	3.28	0.85	0.88	0.85		1.00	Kruk NN et al.	2011	Journal of Asian Earth Sciences	42(5)	928-948
1202	8-648/1	CAOB	Russia	86.0500	52.0833	Granodiorite		406	406	6.43	25.90	0.1490	0.512692	5	-0.24	1.05	3.50	3.52	3.52	0.86	1.08	0.86		0.96	Kruk NN et al.	2011	Journal of Asian Earth Sciences	42(5)	928-948
1203	8-602	CAOB	Russia	86.0500	52.1167	Granite		380	380	6.33	28.51	0.1341	0.512674	23	-0.32	0.70	3.70	3.74	3.74	0.82	0.91	0.82		1.19	Kruk NN et al.	2011	Journal of Asian Earth Sciences	42(5)	928-948
1204	7-181	CAOB	Russia	82.4000	49.5000	Granite		373	373	6.73	28.60	0.1408	0.512677	11	-0.28	0.76	3.40	3.42	3.42	0.84	0.99	0.84			Kruk NN et al.	2011	Journal of Asian Earth Sciences	42(5)	928-948
1205	5-410	CAOB	Russia	87.0500	52.0667	Granite		400	400	8.03	41.78	0.1162	0.512325	9	-0.41	-6.11	8.74	-2.00	-2.00	1.31	1.29	1.31		1.48	Kruk NN et al.	2011	Journal of Asian Earth Sciences	42(5)	928-948
1206	5-401	CAOB	Russia	87.0500	52.0667	Granodiorite		380	380	5.58	26.78	0.1259	0.512376	8	-0.36	-5.11	7.81	-1.68	-1.68	1.26	1.34	1.26		1.29	Kruk NN et al.	2011	Journal of Asian Earth Sciences	42(5)	928-948
1207	5-331	CAOB	Russia	87.0500	52.0667	Granite		367	367	5.60	24.94	0.1358	0.512547	6	-0.31	-1.78	1.10	1.08	1.08	1.03	1.18	1.03		0.98	Kruk NN et al.	2011	Journal of Asian Earth Sciences	42(5)	928-948
1208	5-330v	CAOB	Russia	87.0500	52.0667	Granodiorite		369	369	2.91	14.31	0.1230	0.512566	7	-0.37	-1.40	2.10	2.07	2.07	0.95	0.98	0.95		0.79	Kruk NN et al.	2011	Journal of Asian Earth Sciences	42(5)	928-948
1209	4-404	CAOB	Russia	87.0667	52.0667	Granite		369	369	0.97	6.47	0.0910	0.512516	7	-0.54	-2.38	2.60	2.60	2.60	0.91	0.79	0.91		1.07	Kruk NN et al.	2011	Journal of Asian Earth Sciences	42(5)	928-948
1210	146-1	CAOB	Russia	86.0500	52.0500	Granite		360	360	2.49	13.30	0.1136	0.512490	10	-0.42	-2.89	0.90	0.93	0.93	1.03	1.00	1.03		1.18	Kruk NN et al.	2011	Journal of Asian Earth Sciences	42(5)	928-948
1211	22801	CAOB	Russia	86.0500	52.0500	Granodiorite		374	374	3.38	16.40	0.1246	0.512603	32	-0.37	-0.68	2.80	2.76	2.76	0.90	0.94	0.90		1.01	Kruk NN et al.	2011	Journal of Asian Earth Sciences	42(5)	928-948
1212	144	CAOB	Russia	86.0500	52.0500	Granodiorite		380	380	2.98	14.38	0.1241	0.512453	7	-0.37	-3.61	6.91	-0.09	-0.09	1.13	1.18	1.13		1.30	Kruk NN et al.	2011	Journal of Asian Earth Sciences	42(5)	928-948
1213	8-717	CAOB	Altai	86.2390	51.4450	Granodiorite	Elekmonar	380	380	4.01	19.36	0.1252	0.512572		-0.36	-1.29	2.20	2.18	2.18	0.95	1.00	0.95	1.00	0.96	Kruk NN et al.	2011	Journal of Asian Earth Sciences	42（5）	928-948
1214	O11-14	CAOB	NE Mongolia	110.4612	47.4443	Granite		455	455	5.84	28.30		0.512273			-7.12	-3.06		-3.06	1.44			1.55	1.44	Narantsetseg T et al.	2019	Journal of Asian Earth Sciences	175	138-157
1215	HN93	CAOB	NE Mongolia	110.3556	47.4040	Granite		461	461	4.50	24.10		0.512475			-3.18	1.65		1.65	1.06			1.04	1.06	Narantsetseg T et al.	2019	Journal of Asian Earth Sciences	175	138-157
1216	HN92	CAOB	NE Mongolia	110.3533	47.4179	Granite		461	461	6.60	34.50		0.512468			-3.32	1.35		1.35	1.09			1.09	1.09	Narantsetseg T et al.	2019	Journal of Asian Earth Sciences	175	138-157
1217	HN-48	CAOB	NE Mongolia	110.5926	47.5041	Granite		418	418	15.30	52.90		0.512557			-1.58	-0.57		-0.57	1.21			2.53	1.21	Narantsetseg T et al.	2019	Journal of Asian Earth Sciences	175	138-157
1218	HN45	CAOB	NE Mongolia	110.6835	47.5214	Granite		418	418	2.70	16.90		0.512304			-6.52	-1.26		-1.26	1.27			1.12	1.27	Narantsetseg T et al.	2019	Journal of Asian Earth Sciences	175	138-157
1219	HN-32-1	CAOB	NE Mongolia	110.6684	47.4509	Granite		418	418	4.90	23.70		0.512338			-5.85	-2.14		-2.14	1.34			1.44	1.34	Narantsetseg T et al.	2019	Journal of Asian Earth Sciences	175	138-157
1220	HN32	CAOB	NE Mongolia	110.6799	47.4571	Granite		418	418	11.10	57.20		0.512319			-6.22	-2.10		-2.10	1.34			1.35	1.34	Narantsetseg T et al.	2019	Journal of Asian Earth Sciences	175	138-157
1221	HN16	CAOB	NE Mongolia	110.4869	47.6001	Granite		461	461	4.10	26.30		0.512123			-10.05	-4.10		-4.10	1.54			1.33	1.54	Narantsetseg T et al.	2019	Journal of Asian Earth Sciences	175	138-157
1222	HN1	CAOB	NE Mongolia	110.4917	47.5952	Granite		461	461	5.60	30.50		0.512346			-5.70	-0.75		-0.75	1.26			1.22	1.26	Narantsetseg T et al.	2019	Journal of Asian Earth Sciences	175	138-157
1223	MG-26	CAOB	Northwestern Junggar Basin	83.9167	45.0333	Granitoid		300	300	5.83	28.07	0.1257	0.512911		-0.36	5.33	8.04	8.04	8.04	0.41	0.41	0.41	0.41	0.41	Chen B and Arakawa Y.	2005	Geochimica et Cosmochimica Acta	69(5)	1307-1320
1224	MG-24	CAOB	Northwestern Junggar Basin	83.9167	45.0333	Granitoid		300	300	6.63	30.51	0.1314	0.512890		-0.33	4.92	7.42	7.42	7.42	0.46	0.48	0.46	0.48	0.46	Chen B and Arakawa Y.	2005	Geochimica et Cosmochimica Acta	69(5)	1307-1320
1225	KY-5	CAOB	Northwestern Junggar Basin	86.2167	47.2000	Alkali granite		298	298	4.07	21.34	0.1154	0.512802		-0.41	3.20	6.29	6.29	6.29	0.55	0.54	0.55	0.54	0.55	Chen B and Arakawa Y.	2005	Geochimica et Cosmochimica Acta	69(5)	1307-1320
1226	KM-74	CAOB	Northwestern Junggar Basin	84.8167	45.6167	Granitoid		300	300	3.02	14.11	0.1295	0.512904		-0.34	5.19	7.76	7.76	7.76	0.43	0.45	0.43	0.45	0.43	Chen B and Arakawa Y.	2005	Geochimica et Cosmochimica Acta	69(5)	1307-1320
1227	KM-72	CAOB	Northwestern Junggar Basin	84.8167	45.6167	Granitoid		300	300	3.03	12.17	0.1503	0.512974		-0.24	6.55	8.33	8.33	8.33	0.38	0.42	0.38	0.42	0.38	Chen B and Arakawa Y.	2005	Geochimica et Cosmochimica Acta	69(5)	1307-1320
1228	KM-71	CAOB	Northwestern Junggar Basin	84.8167	45.6167	Granitoid		300	300	2.83	11.40	0.1504	0.512949		-0.24	6.07	7.84	7.84	7.84	0.42	0.48	0.42	0.48	0.42	Chen B and Arakawa Y.	2005	Geochimica et Cosmochimica Acta	69(5)	1307-1320
1229	KM-70	CAOB	Northwestern Junggar Basin	84.8167	45.6167	Granitoid		300	300	3.34	13.60	0.1485	0.512987		-0.25	6.81	8.65	8.65	8.65	0.36	0.38	0.36	0.38	0.36	Chen B and Arakawa Y.	2005	Geochimica et Cosmochimica Acta	69(5)	1307-1320
1230	KM-67	CAOB	Northwestern Junggar Basin	84.8167	45.6167	Granitoid		300	300	4.65	19.66	0.1429	0.512912		-0.27	5.34	7.40	7.40	7.40	0.46	0.51	0.46	0.51	0.46	Chen B and Arakawa Y.	2005	Geochimica et Cosmochimica Acta	69(5)	1307-1320
1231	KM-66	CAOB	Northwestern Junggar Basin	84.8167	45.6167	Granitoid		300	300	3.12	15.10	0.1249	0.512967		-0.37	6.42	9.17	9.17	9.17	0.31	0.31	0.31	0.31	0.31	Chen B and Arakawa Y.	2005	Geochimica et Cosmochimica Acta	69(5)	1307-1320
1232	KM-54	CAOB	Northwestern Junggar Basin	84.8167	45.6167	Granitoid		300	300	3.58	15.52	0.1394	0.512907		-0.29	5.25	7.44	7.44	7.44	0.45	0.50	0.45	0.50	0.45	Chen B and Arakawa Y.	2005	Geochimica et Cosmochimica Acta	69(5)	1307-1320
1233	KM-52	CAOB	Northwestern Junggar Basin	84.8167	45.6167	Granitoid		300	300	4.72	19.29	0.1481	0.512938		-0.25	5.85	7.71	7.71	7.71	0.43	0.49	0.43	0.49	0.43	Chen B and Arakawa Y.	2005	Geochimica et Cosmochimica Acta	69(5)	1307-1320
1234	KM-50	CAOB	Northwestern Junggar Basin	84.8167	45.6167	Granitoid		300	300	6.56	25.85	0.1535	0.512925		-0.22	5.60	7.25	7.25	7.25	0.47	0.57	0.47	0.57	0.47	Chen B and Arakawa Y.	2005	Geochimica et Cosmochimica Acta	69(5)	1307-1320
1235	KM-45	CAOB	Northwestern Junggar Basin	84.8167	45.6167	Granitoid		300	300	2.61	10.69	0.1477	0.512907		-0.25	5.25	7.12	7.12	7.12	0.48	0.56	0.48	0.56	0.48	Chen B and Arakawa Y.	2005	Geochimica et Cosmochimica Acta	69(5)	1307-1320
1236	KM-39	CAOB	Northwestern Junggar Basin	84.8167	45.6167	Granitoid		300	300	3.54	15.56	0.1377	0.512911		-0.30	5.33	7.58	7.58	7.58	0.44	0.48	0.44	0.48	0.44	Chen B and Arakawa Y.	2005	Geochimica et Cosmochimica Acta	69(5)	1307-1320
1237	KM-36	CAOB	Northwestern Junggar Basin	84.8167	45.6167	Granitoid		300	300	4.58	20.15	0.1375	0.512899		-0.30	5.09	7.36	7.36	7.36	0.46	0.50	0.46	0.50	0.46	Chen B and Arakawa Y.	2005	Geochimica et Cosmochimica Acta	69(5)	1307-1320
1238	KM-29	CAOB	Northwestern Junggar Basin	84.8167	45.6167	Granitoid		300	300	4.46	18.37	0.1470	0.512905		-0.25	5.21	7.11	7.11	7.11	0.48	0.56	0.48	0.56	0.48	Chen B and Arakawa Y.	2005	Geochimica et Cosmochimica Acta	69(5)	1307-1320
1239	KM-28	CAOB	Northwestern Junggar Basin	84.8167	45.6167	Granitoid		300	300	4.31	19.13	0.1362	0.512892		-0.31	4.95	7.27	7.27	7.27	0.47	0.51	0.47	0.51	0.47	Chen B and Arakawa Y.	2005	Geochimica et Cosmochimica Acta	69(5)	1307-1320
1240	KM-20	CAOB	Northwestern Junggar Basin	84.8167	45.6167	Granitoid		300	300	4.05	17.50	0.1399	0.512876		-0.29	4.64	6.82	6.82	6.82	0.51	0.57	0.51	0.57	0.51	Chen B and Arakawa Y.	2005	Geochimica et Cosmochimica Acta	69(5)	1307-1320
1241	KM-17	CAOB	Northwestern Junggar Basin	84.8167	45.6167	Granitoid		300	300	2.70	12.91	0.1266	0.512904		-0.36	5.19	7.87	7.87	7.87	0.42	0.43	0.42	0.43	0.42	Chen B and Arakawa Y.	2005	Geochimica et Cosmochimica Acta	69(5)	1307-1320
1242	KM-15	CAOB	Northwestern Junggar Basin	84.8167	45.6167	Granitoid		300	300	5.05	22.38	0.1363	0.512872		-0.31	4.56	6.88	6.88	6.88	0.50	0.55	0.50	0.55	0.50	Chen B and Arakawa Y.	2005	Geochimica et Cosmochimica Acta	69(5)	1307-1320
1243	KM-14	CAOB	Northwestern Junggar Basin	84.8167	45.6167	Granitoid		300	300	3.24	13.84	0.1415	0.512923		-0.28	5.56	7.67	7.67	7.67	0.44	0.48	0.44	0.48	0.44	Chen B and Arakawa Y.	2005	Geochimica et Cosmochimica Acta	69(5)	1307-1320
1244	AT-6	CAOB	Northwestern Junggar Basin	84.2500	45.5000	Granitoid		300	300	10.09	42.96	0.1420	0.512873		-0.28	4.58	6.68	6.68	6.68	0.52	0.59	0.52	0.59	0.52	Chen B and Arakawa Y.	2005	Geochimica et Cosmochimica Acta	69(5)	1307-1320
1245	AT-22	CAOB	Northwestern Junggar Basin	88.3178	49.5678	Granitoid		300	300	3.58	13.26	0.1632	0.512919		-0.17	5.48	6.76	6.76	6.76	0.51	0.70	0.51	0.70	0.51	Chen B and Arakawa Y.	2005	Geochimica et Cosmochimica Acta	69(5)	1307-1320
1246	AT-20	CAOB	Northwestern Junggar Basin	87.3008	48.5508	Granitoid		300	300	6.19	21.06	0.1776	0.512987		-0.10	6.81	7.54	7.54	7.54	0.45	0.69	0.45	0.69	0.45	Chen B and Arakawa Y.	2005	Geochimica et Cosmochimica Acta	69(5)	1307-1320
1247	AT-19	CAOB	Northwestern Junggar Basin	86.2839	47.5339	Granitoid		300	300	7.24	26.60	0.1646	0.512964		-0.16	6.36	7.59	7.59	7.59	0.44	0.58	0.44	0.58	0.44	Chen B and Arakawa Y.	2005	Geochimica et Cosmochimica Acta	69(5)	1307-1320
1248	AT-14	CAOB	Northwestern Junggar Basin	85.2669	46.5169	Granitoid		300	300	6.01	24.07	0.1510	0.512950		-0.23	6.09	7.84	7.84	7.84	0.42	0.49	0.42	0.49	0.42	Chen B and Arakawa Y.	2005	Geochimica et Cosmochimica Acta	69(5)	1307-1320
1249	K32	CAOB	Junggar	88.8015	43.9064	Dacite		405	405	4.31	22.00	0.1313	0.512902		-0.33	5.15	5.10	8.53	8.53	0.45	0.46	0.45	0.46	0.45	Zheng JP et al.	2012	Journal of Asian Earth Sciences	47	35-40
1250	K3	CAOB	Junggar	88.5471	67.0796	Andesite		405	405	4.50	18.30	0.1590	0.512860		-0.19	4.33	4.30	6.28	6.28	0.64	0.81	0.64	0.81	0.64	Zheng JP et al.	2012	Journal of Asian Earth Sciences	47	35-40
1251	K23	CAOB	Junggar	87.3968	45.2293	Rhyolite		395	395	13.00	59.10	0.1469	0.512871		-0.25	4.55	4.50	7.05	7.05	0.56	0.64	0.56	0.64	0.57	Zheng JP et al.	2012	Journal of Asian Earth Sciences	47	35-40
1252	K16	CAOB	Junggar	86.9142	45.0909	Rhyolite		395	395	5.30	24.90	0.1395	0.512806		-0.29	3.28	3.30	6.16	6.16	0.64	0.71	0.64	0.71	0.64	Zheng JP et al.	2012	Journal of Asian Earth Sciences	47	35-40
1253	K146	CAOB	Junggar	85.0604	45.0848	Rhyolite		345	345	5.67	22.00	0.1664	0.512993		-0.15	6.92	6.90	8.26	8.26	0.42	0.51	0.42	0.51	0.43	Zheng JP et al.	2012	Journal of Asian Earth Sciences	47	35-40
1254	K1	CAOB	Junggar	88.4912	45.8411	Rhyolite		405	405	10.70	58.00	0.1190	0.512853		-0.39	4.19	4.20	8.21	8.21	0.48	0.48	0.48	0.48	0.48	Zheng JP et al.	2012	Journal of Asian Earth Sciences	47	35-40
1255	K4	CAOB	Junggar basin	86.0000	44.0000	Basaltic trachyandsite	Wulungu	405	405	2.93	12.1	0.1600	0.512903		-0.19	5.17	5.20	7.07	7.78	0.57	0.70	0.57	0.70	0.44	Zhang XH et al.	2007	Journal of Asian Earth Sciences	29(5-6)	778-794
1256	K7	CAOB	Junggar basin	86.0000	44.0000	Basaltic trachyandsite	Wulungu	405	405	6.97	30	0.1460	0.512953		-0.26	6.14	6.10	8.76	9.06	0.43	0.44	0.43	0.44	0.43	Zhang J P et al.	2007	Journal of Asian Earth Sciences	29(5-6)	778-794
1257	K62	CAOB	Junggar basin	86.0000	44.0000	Basaltic andesite	Wucaiwan	405	405	6.16	28.4	0.1287	0.512826		-0.35	3.67	3.70	7.18	7.07	0.56	0.58	0.56	0.58	0.56	Zhang J P et al.	2007	Journal of Asian Earth Sciences	29(5-6)	778-794
1258	K97	CAOB	Junggar basin	86.0000	44.0000	Trachyte	Kexia	345	345	5.81	23.8	0.1567	0.512931		-0.20	5.72	5.70	7.48	7.89	0.49	0.59	0.49	0.59	0.49	Zhang J P et al.	2007	Journal of Asian Earth Sciences	29(5-6)	778-794
1259	K148	CAOB	Junggar basin	86.0000	44.0000	Basaltic trachyandsite	Kexia	345	345	4.36	15.3	0.1774	0.513017		-0.10	7.39	7.40	8.24	8.48	0.43	0.56	0.43	0.56	0.43	Zhang J P et al.	2007	Journal of Asian Earth Sciences	29(5-6)	778-794
1260	07HT112	CAOB	Baogutu, West Junggar	84.5833	45.4500	Granodiorite porphyry	No. V rock mass	311	311			0.1449	0.512955	0	-0.26	6.18	8.24	8.24	8.24	0.40	0.43	0.40	0.43	0.40	Wei SN and Zhu YF.	2010	Acta Geologica Sinica	84(7)	1017-1029
1261	07HT97	CAOB	Baogutu, West Junggar	84.5833	45.4500	Granodiorite porphyry	No. V rock mass	311	311			0.1300	0.512875	0	-0.34	4.62	7.27	7.27	7.27	0.48	0.50	0.48	0.50	0.48	Wei SN and Zhu YF.	2010	Acta Geologica Sinica	84(7)	1017-1029
1262	07HT96	CAOB	Baogutu, West Junggar	84.5833	45.4500	Chloritized granodiorite p	No. V rock mass	311	311			0.1193	0.512818	0	-0.39	3.51	6.59	6.59	6.59	0.53	0.54	0.53	0.54	0.53	Wei SN and Zhu YF.	2010	Acta Geologica Sinica	84(7)	1017-1029
1263	07HT95	CAOB	Baogutu, West Junggar	84.5833	45.4500	Chloritized granodiorite p	No. V rock mass	311	311			0.1258	0.512869	0	-0.36	4.51	7.32	7.32	7.32	0.47	0.49	0.47	0.49	0.47	Wei SN and Zhu YF.	2010	Acta Geologica Sinica	84(7)	1017-1029
1264	SLS05	CAOB	SHB	109.6214	42.5231	Granite	Mandula	314	314			0.1000	0.512420	1	-0.49	-4.25	-0.38	-0.38	-0.38	1.10	0.98	1.10	0.98	1.11	Jian P et al.	2010	Lithos	118(1)	169-190
1265	SLS02	CAOB	SHB	109.6142	42.5183	Granite	Mandula	313	313			0.1000	0.512410	1	-0.49	-4.45	-0.58	-0.59	-0.59	1.12	0.99	1.12	0.99	1.12	Jian P et al.	2010	Lithos	118(1)	169-190
1266	GO 1/5	CAOB	Western Transbaikalia	106.3000	51.2000	Essexite	ambin volcanotectonic comp	117	150	13.29	80.21	0.0997	0.51248		-0.49	-3.08	-1.60	-1.23	-1.64	1.04	0.90	1.04			Andryushchenko S V et al.	2010	Russian Geology and Geophysics	51(7)	734-749
1267	MG 3/8	CAOB	Western Transbaikalia	106.3000	51.2000	Essexite	ambin volcanotectonic comp	117	150	11.94	85.88	0.0837	0.512557		-0.57	-1.58	0.10	0.58	0.11	0.89	0.70	0.89			Andryushchenko S V et al.	2010	Russian Geology and Geophysics	51(7)	734-749
1268	GO 1/1	CAOB	Western Transbaikalia	106.3000	51.2000	Essexite	ambin volcanotectonic comp	117	150	13.71	84.7	0.0974	0.512449		-0.50	-3.69	-2.20	-1.79	-2.21	1.08	0.92	1.08			Andryushchenko S V et al.	2010	Russian Geology and Geophysics	51(7)	734-749
1269	KHAM 3/16	CAOB	Western Transbaikalia	106.3000	51.3000	Phonotephrite	ambin volcanotectonic comp	124	150	12.25	82	0.0899	0.512494		-0.54	-2.81	-1.10	-0.77	-1.13	1.00	0.81	1.00			Andryushchenko S V et al.	2010	Russian Geology and Geophysics	51(7)	734-749
1270	GO-5/3	CAOB	Western Transbaikalia	106.3000	51.2000	Basaltic trachyandesite	ambin volcanotectonic comp	155	155	7.23	48.71	0.0893	0.512564		-0.55	-1.44	0.70	0.68	0.68	0.89	0.72	0.89			Andryushchenko S V et al.	2010	Russian Geology and Geophysics	51(7)	734-749
1271	GO-5/4	CAOB	Western Transbaikalia	106.3000	51.2000	Basaltic trachyandesite	ambin volcanotectonic comp	155	155	8.32	57.26	0.0875	0.512528		-0.56	-2.15	0.00	0.01	0.02	0.94	0.75	0.94			Andryushchenko S V et al.	2010	Russian Geology and Geophysics	51(7)	734-749
1272	KHAM 1/6	CAOB	Western Transbaikalia	106.3000	51.2000	Trachybasalt	ambin volcanotectonic comp	155	155	8.28	53.86	0.0925	0.512523		-0.53	-2.24	-0.20	-0.18	-0.19	0.96	0.79	0.96			Andryushchenko S V et al.	2010	Russian Geology and Geophysics	51(7)	734-749
1273	BX-8	NCC	NNCC	120.7500	41.7800	Dacite		160	160	4.28	23.90	0.1083	0.512472		-0.45	-3.24		-1.43	-1.43	1.06	0.98	1.06			Wang Y et al.	2012	Journal of Asian Earth Sciences	47	35-40
1274	BX-7	NCC	NNCC	120.7500	41.7800	Dacite		160	160	4.66	27.30	0.1032	0.512469		-0.48	-3.30		-1.39	-1.39	1.06	0.94	1.06			Wang Y et al.	2012	Journal of Asian Earth Sciences	47	35-40
1275	BX-2	NCC	NNCC	120.7500	41.7800	Dacite		160	160	3.44	19.90	0.1045	0.512574		-0.47	-1.25		0.63	0.63	0.90	0.80	0.90			Wang Y et al.	2012	Journal of Asian Earth Sciences	47	35-40
1276	BX-1	NCC	NNCC	120.7500	41.7800	Adakite		160	160	3.68	21.50	0.1035	0.512456		-0.47	-3.55		-1.65	-1.65	1.08	0.96	1.08			Wang Y et al.	2012	Journal of Asian Earth Sciences	47	35-40

1277	B-008	NCC	NNCC	120.7500	41.7800	Andesite		160	160	5.23	28.00	0.1129	0.512570		-0.43	-1.33		0.38	0.38	0.92	0.88	0.92			Wang Y et al.	2012	Journal of Asian Earth Sciences	47	35-40
1278	B-005	NCC	NNCC	120.7500	41.7800	Adakite		160	160	3.91	22.70	0.1041	0.512449		-0.47	-3.69		-1.80	-1.80	1.09	0.98	1.09			Wang Y et al.	2012	Journal of Asian Earth Sciences	47	35-40
1279	B-001	NCC	NNCC	120.7500	41.7800	Adakite		160	160	4.04	22.70	0.1076	0.512459		-0.45	-3.49		-1.67	-1.67	1.08	0.99	1.08			Wang Y et al.	2012	Journal of Asian Earth Sciences	47	35-40
1280	420-1	NCC	NNCC	120.7500	41.7800	Andesite		160	160	5.13	26.60	0.1166	0.512617		-0.41	-0.41		1.23	1.23	0.85	0.84	0.85			Wang Y et al.	2012	Journal of Asian Earth Sciences	47	35-40
1281	407-1	NCC	NNCC	120.7500	41.7800	Adakite		160	160	3.98	21.70	0.1109	0.512427		-0.44	-4.12		-2.36	-2.36	1.14	1.07	1.14			Wang Y et al.	2012	Journal of Asian Earth Sciences	47	35-40
1282	1122CL	CAOB	Beishan	97.2184	41.6117	Qtz diorite	baiqitabeishan	436	436	3.12	15.1	0.1251	0.512535	5	-0.36	-2.01	2.00	1.97	1.97	1.01	1.06	1.01	1.01	1.06	Li XF.	2013	Dissertation, Northwest University, China		1-57
1283	1132WG	CAOB	Beishan	97.2279	41.5594	Diorite	mazongshan	364	364	3.28	15.5	0.1279	0.512489	9	-0.35	-2.91	0.30	0.29	0.29	1.09	1.17	1.09	1.09	1.18	Li XF.	2013	Dissertation, Northwest University, China		1-57
1284	XJ11-137	CAOB	Beishan	94.8044	41.1276	Granite	Mingshujing	436	436	3.876	22.9	0.1023	0.51236	4	-0.48	-5.42	-0.20	-0.17	-0.17	1.19	1.08	1.19	1.09	1.19	Li XF et al.	2015	Acta Petrologica Sinica	31(9)	2521-2538
1285	XJ11-138	CAOB	Beishan	94.8201	41.1196	Diorite	Mingshujing	437	437	4.299	22.72	0.1144	0.512329	4	-0.42	-6.03	-1.50	-1.44	-1.44	1.29	1.26	1.29	1.27	1.30	Li XF et al.	2015	Acta Petrologica Sinica	31(9)	2521-2538
1286	XJ11-142	CAOB	Beishan	94.8039	41.1129	Diorite	Mingshujing	437	437	6.201	32.15	0.1166	0.512406	3	-0.41	-4.53	-0.10	-0.06	-0.06	1.18	1.17	1.18	1.18	1.18	Li XF et al.	2015	Acta Petrologica Sinica	31(9)	2521-2538
1287	P93-1	CAOB	Northern Tianshan	81.7667	45.2167	Rhyolite	Alataw	306	306	7.44	33.06	0.1361	0.512572	±6	-0.31	-1.29	1.20	1.08	1.08	0.98	1.13	0.98	1.13	0.98	Chen JF et al.	2000	Tectonophysics	328(1-2)	53-67
1288	P203	CAOB	Northern Tianshan	81.7667	45.2167	Rhyolite	Alataw	306	306	10.56	40.46	0.1578	0.512839	±6	-0.20	3.92	5.50	5.44	5.44	0.62	0.85	0.62	0.85	0.62	Chen JF et al.	2000	Tectonophysics	328(1-2)	53-67
1289	P152	CAOB	Northern Tianshan	81.7667	45.2167	Andesite	Alataw	306	306	5.40	22.14	0.1475	0.512656	±11	-0.25	0.35	2.40	2.27	2.27	0.88	1.14	0.88	1.14	0.88	Chen JF et al.	2000	Tectonophysics	328(1-2)	53-67
1290	P701-2	CAOB	NW China	81.1222	45.0930	Granite		290	290	1.28	3.09	0.2509	0.512841		0.28	3.96	2.00	1.95	1.95	0.90	-1.28	0.90			Chen JF et al.	2000	Tectonophysics	328(1-2)	53-67
1291	P66	CAOB	NW China	81.0675	45.1229	Granite		299	299	1.95	6.90	0.1707	0.512651		-0.13	0.25	1.20	1.24	1.24	0.96	1.76	0.96			Chen JF et al.	2000	Tectonophysics	328(1-2)	53-67
1292	P44	CAOB	NW China	81.0973	45.0748	Granite		299	299	3.07	12.62	0.1470	0.512592		-0.25	-0.90	0.90	1.00	1.00	0.98	1.27	0.98			Chen JF et al.	2000	Tectonophysics	328(1-2)	53-67
1293	P262	CAOB	NW China	81.7191	45.2174	Granodiorite		299	299	3.72	16.58	0.1355	0.512519		-0.31	-2.32	0.10	0.01	0.01	1.06	1.23	1.06			Chen JF et al.	2000	Tectonophysics	328(1-2)	53-67
1294	P196	CAOB	NW China	82.3806	45.1046	Granite		299	299	7.82	33.60	0.1406	0.512617		-0.29	-0.41	1.80	1.73	1.73	0.92	1.11	0.92			Chen JF et al.	2000	Tectonophysics	328(1-2)	53-67
1295	P175	CAOB	NW China	82.2944	45.0167	Rhyolite		329	329	8.86	36.24	0.1479	0.512658		-0.25	0.39	2.30	2.44	2.44	0.89	1.14	0.89			Chen JF et al.	2000	Tectonophysics	328(1-2)	53-67
1296	P136	CAOB	NW China	82.3358	45.1892	Granite		299	299	5.66	22.64	0.1511	0.512660		-0.23	0.43	2.20	2.17	2.17	0.88	1.19	0.88			Chen JF et al.	2000	Tectonophysics	328(1-2)	53-67
1297	P122	CAOB	NW China	81.6513	45.2002	Granite		299	299	7.43	31.71	0.1418	0.512561		-0.28	-1.50	0.70	0.59	0.59	1.01	1.25	1.01			Chen JF et al.	2000	Tectonophysics	328(1-2)	53-67
1298	P117	CAOB	NW China	82.5630	44.9372	Granite		299	299	10.83	43.57	0.1503	0.512789		-0.24	2.95	4.80	4.71	4.71	0.68	0.87	0.68			Chen JF et al.	2000	Tectonophysics	328(1-2)	53-67
1299	P115	CAOB	NW China	82.4138	44.9554	Granite		299	299	9.43	40.96	0.1392	0.512876		-0.29	4.64	6.90	6.83	6.83	0.50	0.56	0.50			Chen JF et al.	2000	Tectonophysics	328(1-2)	53-67
1300	P236	CAOB	North Tianshan	81.0000	44.8000	Granitoids	Alataw	299	299	3.16	12.56	0.1529	0.512771		-0.22	2.59		4.26	4.26	0.71	0.95	0.71			Chen JF et al.	2000	Tectonophysics	328(1-2)	53-67
1301	881073-2	CAOB	East Tianshan	95.2833	41.8333	arse grained gneissic granite		919	600			0.1065	0.512098		-0.46	-10.53	0.07	-3.63	-3.63	1.60	1.49	1.60	1.60	1.57	Hu AQ et al.	2010	Geochimica	39(3)	197-212
1302	81073-1	CAOB	Central Tianshan	81.9250	42.7750	Gneissic granite		904	600			0.1243	0.512104		-0.37	-10.42	-2.05	-4.87	-4.87	1.70	1.78	1.70	1.78	1.73	Hu AQ et al.	2010	Geochimica	39(3)	197-212
1303	02XW-25b	CAOB	East Tianshan	95.2833	41.8333	arse grained gneissic granite		919	600			0.1336	0.512174		-0.32	-9.05	-1.63	-4.22	-4.22	1.65	1.85	1.65	1.90	1.71	Hu AQ et al.	2010	Geochimica	39(3)	197-212
1304	02XH-21a	CAOB	East Tianshan	95.2833	41.8333	Giant eye gneissic granite		942	600			0.1535	0.512175		-0.22	-9.03	-3.83	-5.72	-5.72	1.77	2.46	1.77	2.46	1.91	Hu AQ et al.	2010	Geochimica	39(3)	197-212
1305	020XH-19b	CAOB	East Tianshan	95.2833	41.8333	Gneissic granite		942	600			0.1289	0.512219		-0.34	-8.17	0.00	-2.98	-2.98	1.55	1.67	1.55	1.67	1.60	Hu AQ et al.	2010	Geochimica	39(3)	197-212
1306	CL-02	CAOB	SMXAB	123.9958	51.1869	Monzogranite	Chalukou	172	172			0.0984	0.512461		-0.50	-3.45	-1.30	-1.30	-1.30	1.06	0.91	1.06	0.91	1.07	Li ZZ et al.	2014	Lithos	202-203	138-156
1307	903-1273.3	CAOB	SMXAB	123.9958	51.1869	Feldspar porphyry	Chalukou	128	150			0.1000	0.512581		-0.49	-1.11	0.46	0.74	0.74	0.88	0.76	0.88	0.77	0.89	Li ZZ et al.	2014	Lithos	202-203	138-156
1308	902-1196	CAOB	SMXAB	123.9958	51.1869	Aplite porphyry	Chalukou	148	150			0.1213	0.512580		-0.38	-1.13	0.30	0.31	0.31	0.91	0.94	0.91	0.94	0.92	Li ZZ et al.	2014	Lithos	202-203	138-156
1309	804-228	CAOB	SMXAB	123.9958	51.1869	Granite porphyry	Chalukou	147	150			0.1175	0.512553		-0.40	-1.66	-0.17	-0.14	-0.14	0.95	0.95	0.95	0.95	0.95	Li ZZ et al.	2014	Lithos	202-203	138-156
1310	1902-674.1	CAOB	SMXAB	123.9958	51.1869	Aplite porphyry	Chalukou	148	150			0.1147	0.512577		-0.42	-1.19	0.36	0.38	0.38	0.91	0.88	0.91	0.89	0.91	Li ZZ et al.	2014	Lithos	202-203	138-156
1311	1708-361.7	CAOB	SMXAB	123.9958	51.1869	Feldspar porphyry	Chalukou	141	150			0.0977	0.512551		-0.50	-1.70	0.09	0.20	0.20	0.92	0.79	0.92	0.79	0.93	Li ZZ et al.	2014	Lithos	202-203	138-156
1312	1703-383	CAOB	SMXAB	123.9958	51.1869	Granite porphyry	Chalukou	147	150			0.2380	0.512665		0.21	0.53	-0.25	-0.26	-0.26	0.98	-3.08	0.98		0.96	Li ZZ et al.	2014	Lithos	202-203	138-156
1313	1702-180	CAOB	SMXAB	123.9958	51.1869	Granite porphyry	Chalukou	147	150			0.1391	0.512616		-0.29	-0.43	0.65	0.67	0.67	0.88	1.09	0.88	1.09	0.89	Li ZZ et al.	2014	Lithos	202-203	138-156
1314	1111-1116	CAOB	SMXAB	123.9958	51.1869	Aplite porphyry	Chalukou	148	150			0.1284	0.512591		-0.35	-0.92	0.38	0.39	0.39	0.91	1.00	0.91	1.00	0.91	Li ZZ et al.	2014	Lithos	202-203	138-156
1315	1104-205.7	CAOB	SMXAB	123.9958	51.1869	Granite porphyry	Chalukou	147	150			0.1295	0.512549		-0.34	-1.74	-0.48	-0.45	-0.45	0.97	1.09	0.97	1.09	0.98	Li ZZ et al.	2014	Lithos	202-203	138-156
1316	1103-886	CAOB	SMXAB	123.9958	51.1869	Granite porphyry	Chalukou	147	150			0.1471	0.512561		-0.25	-1.50	-0.56	-0.55	-0.55	0.98	1.35	0.98	1.35	0.99	Li ZZ et al.	2014	Lithos	202-203	138-156
1317	1103-1165.5	CAOB	SMXAB	123.9958	51.1869	Aplite porphyry	Chalukou	148	150			0.1020	0.512551		-0.48	-1.70	0.09	0.12	0.12	0.93	0.82	0.93	0.82	0.93	Li ZZ et al.	2014	Lithos	202-203	138-156
1318	1102-521	CAOB	SMXAB	123.9958	51.1869	Granite porphyry	Chalukou	147	150			0.1058	0.512598		-0.46	-0.78	0.92	0.96	0.96	0.86	0.78	0.86	0.78	0.86	Li ZZ et al.	2014	Lithos	202-203	138-156
1319	yQ-27	CAOB	Altai, unit 6	90.3486	46.2283	Granite	Utubrak	360	360			0.1163	0.512800		-0.41	3.16	6.85	6.85	6.85	0.55	0.55	0.55	0.55	0.55	Zhou G et al.	2009	Acta Petrologica Sinica	25(6)	1390-1402
1320	yQ-24	CAOB	Altai, unit 6	90.3486	46.2283	F-grained granite	Utubrak	360	360			0.1249	0.512710		-0.37	1.40	4.70	4.70	4.70	0.73	0.76	0.73	0.76	0.73	Zhou G et al.	2009	Acta Petrologica Sinica	25(6)	1390-1402
1321	04YJ-9	CAOB	NE China	129.4200	42.7700	Andesite		57	150	6.57	44.40	0.9090	0.512795		3.62	3.06	3.81	-10.6	-10.6	1.81	-0.08	1.81	0.44		Guo F et al.	2007	Journal of Petrology	48（4）	661-692
1322	04YJ-7	CAOB	NE China	129.4200	42.7700	Andesite		57	150	6.54	44.28	0.9080	0.512795		3.62	3.06	3.81	-10.6	-10.6	1.81	-0.08	1.81	0.44		Guo F et al.	2007	Journal of Petrology	48（4）	661-692
1323	04YJ-6	CAOB	NE China	129.4200	42.7700	Andesite		57	150	6.50	43.31	0.9230	0.512823		3.69	3.61	4.34	-10.3	-10.3	1.79	-0.07	1.79	0.41		Guo F et al.	2007	Journal of Petrology	48（4）	661-692
1324	04YJ-5	CAOB	NE China	129.4200	42.7700	Andesite		57	150	6.78	44.53	0.9360	0.512898		3.76	5.07	5.80	-9.08	-9.08	1.69	-0.05	1.69	0.32		Guo F et al.	2007	Journal of Petrology	48（4）	661-692
1325	04YJ-4	CAOB	NE China	129.4200	42.7700	Andesite		57	150	6.61	43.38	0.9360	0.512877		3.76	4.66	5.38	-9.49	-9.49	1.72	-0.06	1.72	0.35		Guo F et al.	2007	Journal of Petrology	48（4）	661-692
1326	04YJ-2	CAOB	NE China	129.4200	42.7700	Andesite		57	150	6.49	44.25	0.9020	0.512920		3.59	5.50	6.26	-8.00	-8.00	1.60	-0.05	1.60	0.28		Guo F et al.	2007	Journal of Petrology	48（4）	661-692
1327	04YJ-1	CAOB	NE China	129.4200	42.7700	Andesite		57	150	6.23	42.12	0.9100	0.512882		3.63	4.76	5.51	-8.89	-8.89	1.67	-0.06	1.67	0.33		Guo F et al.	2007	Journal of Petrology	48（4）	661-692
1328	Rh(01)Nd3	CAOB	Beishan	96.2500	40.5833	um coarse grained monzog	Queershan	286	286	2.37	11.23	0.1277	0.512809	9	-0.35	3.34	5.86	5.86	5.86	0.57	0.61	0.57	0.61	0.54	Liu MQ.	2005	Geological Bulletin of China	24(9)	831-836
1329	Rh(01)Nd1	CAOB	Beishan	96.2667	40.6000	orphyritic Hb Bi monzogran	Maanshanbei	238	238	2.56	14.64	0.1059	0.512706		-0.46	1.33	4.08	4.08	4.08	0.68	0.63	0.68	0.63	0.71	Liu MQ.	2005	Geological Bulletin of China	24(9)	831-836
1330	ZK603-146	CAOB	Western Junggar	84.6193	45.1819	Diorite		320	320	3.11	14.90	0.1261	0.512826	0	-0.36	3.67	6.60	6.55	6.55	0.54	0.56	0.54	0.57	0.55	Cao M et al.	2016	Lithos	246-247	13-30
1331	ZK603-116	CAOB	Western Junggar	84.6193	45.1819	Fine-grained diorite		320	320	1.88	7.87	0.1445	0.512921	0	-0.27	5.52	7.70	7.65	7.65	0.45	0.51	0.45	0.51	0.46	Cao M et al.	2016	Lithos	246-247	13-30
1332	ZK403-360	CAOB	Western Junggar	84.6193	45.1819	Diorite		320	320	3.22	14.30	0.1365	0.512890	0	-0.31	4.92	7.40	7.37	7.37	0.48	0.51	0.48	0.52	0.48	Cao M et al.	2016	Lithos	246-247	13-30
1333	ZK403-175	CAOB	Western Junggar	84.6193	45.1819	Fine-grained diorite		320	320	3.29	14.80	0.1345	0.512842	0	-0.32	3.98	6.50	6.52	6.52	0.55	0.59	0.55	0.60	0.55	Cao M et al.	2016	Lithos	246-247	13-30
1334	ZK211-196	CAOB	Western Junggar	84.6193	45.1819	Hb diorite porphyry		306	306	2.11	9.51	0.1345	0.512913	0	-0.32	5.36	7.80	7.79	7.79	0.43	0.46	0.43	0.46	0.43	Cao M et al.	2016	Lithos	246-247	13-30
1335	ZK205-82	CAOB	Western Junggar	84.6193	45.1819	Diorite		320	320	3.77	16.70	0.1365	0.512855	0	-0.31	4.23	6.70	6.69	6.69	0.53	0.58	0.53	0.59	0.54	Cao M et al.	2016	Lithos	246-247	13-30
1336	ZK205-236	CAOB	Western Junggar	84.6193	45.1819	Hb diorite porphyry		306	306	2.55	12.40	0.1245	0.512868	0	-0.37	4.49	7.30	7.31	7.31	0.47	0.48	0.47	0.49	0.47	Cao M et al.	2016	Lithos	246-247	13-30
1337	ZK204-41	CAOB	Western Junggar	84.6193	45.1819	Granodiorite porphyry		310	310	3.63	17.50	0.1258	0.512815	0	-0.36	3.45	6.30	6.26	6.26	0.56	0.58	0.56	0.58	0.56	Cao M et al.	2016	Lithos	246-247	13-30
1338	ZK202-98	CAOB	Western Junggar	84.6193	45.1819	Granodiorite porphyry		310	310	3.61	17.70	0.1238	0.512823	0	-0.37	3.61	6.50	6.49	6.49	0.54	0.56	0.54	0.56	0.54	Cao M et al.	2016	Lithos	246-247	13-30
1339	ZK003-305	CAOB	Western Junggar	84.6193	45.1819	Granodiorite porphyry		310	310	1.46	7.12	0.1237	0.512821	0	-0.37	3.57	6.50	6.46	6.46	0.54	0.56	0.54	0.56	0.55	Cao M et al.	2016	Lithos	246-247	13-30
1340	Taerbaha-01	CAOB	Western Junggar	83.5056	47.1750	Granite	Baishan	328	328	7.51	29.90	0.1519	0.512816	5	-0.23	3.47	5.08	5.35	5.35	0.65	0.82	0.65	0.92	0.67	Cao M et al.	2016	Lithos	246-247	13-30
1341	BHS	CAOB	Western Junggar	84.6193	45.1819	Diorite		320	320	2.93	13.10	0.1352	0.512860	0	-0.31	4.33	6.90	6.84	6.84	0.52	0.56	0.52	0.57	0.52	Cao M et al.	2016	Lithos	246-247	13-30
1342	BE2-0	CAOB	Western Junggar	84.6193	45.1819	Diorite		320	320	3.04	14.70	0.1253	0.512810	0	-0.36	3.36	6.30	6.27	6.27	0.57	0.59	0.57	0.59	0.57	Cao M et al.	2016	Lithos	246-247	13-30
1343	BD2-15	CAOB	Western Junggar	84.6193	45.1819	Diorite porphyry		312	312	2.33	10.80	0.1307	0.512877	0	-0.34	4.66	7.40	7.29	7.29	0.48	0.50	0.48	0.50	0.48	Cao M et al.	2016	Lithos	246-247	13-30
1344	BD13-5	CAOB	Western Junggar	84.6193	45.1819	Diorite porphyry		312	312	3.54	17.10	0.1253	0.512832	0	-0.36	3.78	6.70	6.63	6.63	0.53	0.55	0.53	0.55	0.53	Cao M et al.	2016	Lithos	246-247	13-30
1345	Ld-03	CAOB	East Tianshan	90.8028	42.1556	Granite	Jueluotage	276	276			0.1353	0.512560		-0.31	-1.52	0.64	0.64	0.64	0.99	1.15	0.99	1.15	0.99	Zhang DY et al.	2014	Lithos	198–199	141-152
1346	Ld-02	CAOB	East Tianshan	90.8028	42.1556	Granite	Jueluotage	276	276			0.1361	0.512519		-0.31	-2.32	-0.18	-0.19	-0.19	1.06	1.24	1.06	1.24	1.06	Zhang DY et al.	2014	Lithos	198–199	141-152
1347	Hsyt-03	CAOB	East Tianshan	94.6492	42.1650	Granite	Jueluotage	288	288			0.1167	0.512586		-0.41	-1.01	1.93	1.93	1.93	0.89	0.89	0.89	0.89	0.90	Zhang DY et al.	2014	Lithos	198–199	141-152
1348	Hsyt-02	CAOB	East Tianshan	94.6517	42.1864	Granite	Jueluotage	288	288			0.1850	0.512587		-0.06	-0.99	-0.56	-0.56	-0.56	1.09	2.97	1.09	2.98	1.10	Zhang DY et al.	2014	Lithos	198–199	141-152
1349	Hsyt-01	CAOB	East Tianshan	94.5875	42.2075	Granite	Jueluotage	288	288			0.1703	0.512524		-0.13	-2.22	-1.25	-1.25	-1.25	1.15	2.19	1.15	2.19	1.16	Zhang DY et al.	2014	Lithos	198–199	141-152
1350	Hs-01*	CAOB	East Tianshan	90.8028	42.1556	Granite	Jueluotage	283	283			0.1559	0.512664		-0.21	0.51	1.98	1.98	1.98	0.88	1.28	0.88	1.28	0.89	Zhang DY et al.	2014	Lithos	198–199	141-152
1351	Gd-05*	CAOB	East Tianshan	90.5578	42.1500	Granite	Guandao	284	284			0.1476	0.512633		-0.25	-0.10	1.68	1.68	1.68	0.91	1.19	0.91	1.19	0.92	Zhang DY et al.	2014	Lithos	198–199	141-152
1352	Gd-01*	CAOB	East Tianshan	90.5486	42.1556	Granite	Guandao	284	284			0.1662	0.512670		-0.16	0.62	1.73	1.73	1.73	0.91	1.54	0.91	1.54	0.91	Zhang DY et al.	2014	Lithos	198–199	141-152
1353	Dk-03*	CAOB	East Tianshan	89.9489	42.3864	Granite	Dikan	288	288			0.1416	0.512928		-0.28	5.66	7.69	7.68	7.68	0.43	0.47	0.43	0.47	0.43	Zhang DY et al.	2014	Lithos	198–199	141-152
1354	Dk-01*	CAOB	East Tianshan	89.9508	42.3858	Granite	Dikan	288	288			0.1426	0.512926		-0.28	5.62	7.61	7.61	7.61	0.43	0.48	0.43	0.48	0.43	Zhang DY et al.	2014	Lithos	198–199	141-152
1355	Bsd-02*	CAOB	East Tianshan	95.9494	42.4958	Granite	Jueluotage	285	285			0.1586	0.512842		-0.19	3.98	5.37	5.36	5.36	0.61	0.85	0.61	0.85	0.61	Zhang DY et al.	2014	Lithos	198–199	141-152
1356	Bsd-01*	CAOB	East Tianshan	95.9436	42.4981	Granite	Jueluotage	285	285			0.1425	0.512830		-0.28	3.75	5.72	5.71	5.71	0.58	0.69	0.58	0.69	0.59	Zhang DY et al.	2014	Lithos	198–199	141-152
1357	TCG-41 (duplicate)	CAOB	Northwestern Junggar Basin	85.8403	46.6689	A-type Granite	Tiechanggou	294	294	4.34	21.28	0.1232	0.512778		-0.37	2.73	5.49	5.49	5.49	0.61	0.63	0.61	0.63	0.61	Chen B and Jhan BM.	2004	Journal of Asian Earth Sciences	23(5)	691-703
1358	TCG-41	CAOB	Northwestern Junggar Basin	85.8403	46.6689	A-type Granite	Tiechanggou	294	294	4.41	21.57	0.1236	0.512852		-0.37	4.17	6.92	6.92	6.92	0.49	0.50	0.49	0.50	0.49	Chen B and Jhan BM.	2004	Journal of Asian Earth Sciences	23(5)	691-703
1359	MG-1	CAOB	Northwestern Junggar Basin	84.5069	46.3356	I-type Granite	Miaoergou	294	294	7.55	33.01	0.1388	0.512810		-0.29	3.36	5.53	5.53	5.53	0.61	0.69	0.61	0.69	0.61	Chen B and Jhan BM.	2004	Journal of Asian Earth Sciences	23(5)	691-703
1360	KM-6	CAOB	Northwestern Junggar Basin	84.8167	45.6167	Granitoid	Karamay	300	300	3.90	16.04	0.1470	0.512924		-0.25	5.58	7.48	7.48	7.48	0.45	0.52	0.45	0.52	0.45	Chen B and Jhan BM.	2004	Journal of Asian Earth Sciences	23(5)	691-703
1361	C69	NCC	NCC	118.4917	42.4342	Syenogranite porphyry	Chehugou	266	266	19.66	46.27	0.2696	0.512172		0.37	-9.09	-11.6	-11.6	-11.6	2.00	-2.70	2.00		1.98	Zeng QD et al.	2012	Geological Magazine	149(05)	753-767
1362	C349	NCC	NCC	118.4958	42.4378	Granite porphyry	Chehugou	246	246	16.40	36.53	0.2848	0.512123		0.45	-10.05	-12.8	-12.8	-12.8	2.09	-2.22	2.09		2.06	Zeng QD et al.	2012	Geological Magazine	149(05)	753-767
1363	C272	NCC	NCC	118.4911	42.4381	Granite porphyry	Chehugou	246	246	15.27	32.92	0.2943	0.512275		0.50	-7.08	-10.2	-10.1	-10.1	1.86	-1.67	1.86		1.84	Zeng QD et al.	2012	Geological Magazine	149(05)	753-767
1364	C270	NCC	NCC	118.4908	42.4211	Syenogranite porphyry	Chehugou	266	266	20.24	42.89	0.2994	0.512208		0.52	-8.39	-11.9	-11.9	-11.9	2.02	-1.69	2.02		2.00	Zeng QD et al.	2012	Geological Magazine	149(05)	753-767
1365	C268	NCC	NCC	118.4908	42.4364	Granite porphyry	Chehugou	246	246	16.74	35.00	0.3035	0.512224		0.54	-8.08	-11.4	-11.4	-11.4	1.97	-1.59	1.97		1.95	Zeng QD et al.	2012	Geological Magazine	149(05)	753-767
1366	C206	NCC	NCC	118.4908	42.4364	Granite porphyry	Chehugou	246	246	17.55	35.32	0.3153	0.512155		0.60	-9.42	-13.2	-13.1	-13.1	2.11	-1.51	2.11		2.09	Zeng QD et al.	2012	Geological Magazine	149(05)	753-767
1367	C202	NCC	NCC	118.5181	42.4392	Syenogranite porphyry	Chehugou	266	266	19.75	43.24	0.2898	0.512152		0.47	-9.48	-12.7	-12.6	-12.6	2.09	-2.02	2.09		2.06	Zeng QD et al.	2012	Geological Magazine	149(05)	753-767
1368	B363	NCC	NCC	118.5161	42.4406	Syenogranite porphyry	Chehugou	266	266	6.17	40.00	0.0978	0.511943		-0.50	-13.56	-10.2	-10.2	-10.2	1.86	1.58	1.86	1.59	1.86	Zeng QD et al.	2012	Geological Magazine	149(05)	753-767
1369	B231	NCC	NCC	118.4850	42.4353	Granite	Chehugou	385	385	11.52	96.33	0.0759	0.511797		-0.61	-16.41	-10.5	-10.5	-10.5	1.98	1.49	1.98	1.50	1.98	Zeng QD et al.	2012	Geological Magazine	149(05)	753-767
1370	XK10-28	CAOB	SMXAB	125.7558	49.5922	Mylonization granite	Zuohushan	202	202	2.90	8.79	0.1997	0.512839	13	0.02	3.92	3.85	3.84	3.84	0.66	3.36	0.66	3.39	0.67	Zeng QD et al.	2012	Geological Magazine	149(05)	753-767
1371	HY34	CAOB	Altai, unit 7	90.3611	45.1778	Alkaline granite	Sabei	306	306	10.52	42.21	0.1509	0.512817	11	-0.23	3.49	5.30	5.28	5.28	0.64	0.81	0.64		0.65	Tang HF et al.	2007	Acta Petrologica Sinica	23(8)	1989-1997
1372	HY19	CAOB	Altai, unit 7	90.3611	45.1778	Alkaline granite	Sabei	306	306	13.09	47.25	0.1677	0.512830	10	-0.15	3.75	4.90	4.88	4.88	0.67	1.06	0.67		0.69	Tang HF et al.	2007	Acta Petrologica Sinica	23(8)	1989-1997
1373	K0238	CAOB	Mongolia	98.4512	45.3421	Granodiorite		540	540	5.60	24.80	0.1365	0.512529		-0.31	-2.13	2.00	2.02	2.02	1.09	1.23	1.09			David Buriánek et al.	2017	Gondwana Research	47	200-227
1374	H0486	CAOB	Mongolia	98.4943	45.3753	Orthogneiss		540	540	4.50	35.30	0.0771	0.512345		-0.61	-5.72	2.50	2.53	2.53	1.05	0.90	1.05			David Buriánek et al.	2017	Gondwana Research	47	200-227

1375	XT17	CAOB	Central Tianshan Granite belt	84.7000	42.5667	Granite		437	437	6.68	37.29	0.1083	0.512052		-0.45	-11.43	-6.50	-6.50	-6.50	1.70	1.58	1.70	1.58	1.70	Long LL et al.	2011	Lithos	126(3-4)	321-340
1376	QK5	CAOB	Central Tianshan Granite belt	82.2167	42.9000	Granite		322	322	2.10	8.33	0.1523	0.512500		-0.23	-2.69	-0.87	-0.87	-0.87	1.15	1.61	1.15	1.61	1.15	Long LL et al.	2011	Lithos	126(3-4)	321-340
1377	QK1	CAOB	Central Tianshan Granite belt	82.2167	42.9000	Granite		325	325	3.20	12.58	0.1538	0.512504		-0.22	-2.61	-0.83	-0.83	-0.83	1.15	1.64	1.15	1.64	1.15	Long LL et al.	2011	Lithos	126(3-4)	321-340
1378	KKS5	CAOB	Central Tianshan	81.9000	42.7917	Monzodiorite		398	398	10.68	42.12	0.1532	0.512602		-0.22	-0.70	1.51	1.51	1.51	1.02	1.38	1.02	1.38	1.02	Long LL et al.	2011	Lithos	126(3-4)	321-340
1379	KKS3	CAOB	Central Tianshan	81.9000	42.7917	Hb granite		433	433	4.47	19.37	0.1396	0.512539		-0.29	-1.93	1.23	1.22	1.22	1.07	1.26	1.07	1.26	1.08	Long LL et al.	2011	Lithos	126(3-4)	321-340
1380	KKS12	CAOB	Central Tianshan	81.8917	42.8833	Bi Hb granite		349	349	4.14	24.59	0.1018	0.512526		-0.48	-2.18	2.05	2.04	2.04	0.94	0.85	0.94	0.85	0.94	Long LL et al.	2011	Lithos	126(3-4)	321-340
1381	KKS10	CAOB	Central Tianshan	81.9000	42.8333	Bi granite		352	352	6.97	35.99	0.1170	0.512558		-0.41	-1.56	2.02	2.02	2.02	0.94	0.93	0.94	0.93	0.94	Long LL et al.	2011	Lithos	126(3-4)	321-340
1382	KKS1	CAOB	Central Tianshan	81.9250	42.7750	Qtz syenite		275	275	5.92	28.24	0.1266	0.512216		-0.36	-8.23	-5.77	-5.77	-5.77	1.51	1.63	1.51	1.63	1.55	Long LL et al.	2011	Lithos	126(3-4)	321-340
1383	HYS73	CAOB	South Tianshan	82.4667	42.5000	Bi granite		285	285	12.15	61.63	0.1192	0.512270		-0.39	-7.18	-4.36	-4.36	-4.36	1.40	1.42	1.40	1.42	1.41	Long LL et al.	2011	Lithos	126(3-4)	321-340
1384	BK7	CAOB	Central Tianshan Granite belt	81.3333	42.5500	Granite	Gaoersayi	479	479	13.45	66.80	0.1218	0.512147		-0.38	-9.58	-5.00	-5.00	-5.00	1.61	1.66	1.61	1.66	1.61	Long LL et al.	2011	Lithos	126(3-4)	321-340
1385	AK8	CAOB	Central Tianshan Granite belt	81.1167	41.6000	Granite		409	409	9.24	34.34	0.1626	0.512506		-0.17	-2.57	-0.80	-0.80	-0.80	1.21	1.91	1.21	1.91	1.21	Long LL et al.	2011	Lithos	126(3-4)	321-340
1386	WQ2	CAOB	North Yili Block Granite belt	80.3333	44.8333	Granite		371	371	9.59	41.04	0.1413	0.512370		-0.28	-5.23	-2.61	-2.61	-2.61	1.33	1.64	1.33	1.64	1.35	Long LL et al.	2011	Lithos	126（3-4）	321-340
1387	MG6	CAOB	Junggar	83.8382	45.5562	Andesite		341	341	5.63	28.00	0.1215	0.512685		-0.38	0.92	4.20	4.19	4.19	0.75	0.77	0.75	0.77		Geng HY et al.	2011	Journal of Asian Earth Sciences	42（5）	854-866
1388	K-6	CAOB	Junggar	84.6000	45.7200	Diorite		329	329	2.89	11.54	0.1512	0.512921		-0.23	5.52	7.00	**7.43**	7.43	0.48	0.56	0.48			Zhan Y et al.	2015	Journal of Asian Earth Sciences	113	126-136
1389	K-10	CAOB	Junggar	84.6000	45.7200	Diorite		329	329	3.45	13.62	0.1532	0.512829		-0.22	3.73	5.10	**5.55**	5.55	0.63	0.81	0.63			Zhan Y et al.	2015	Journal of Asian Earth Sciences	113	126-136
1390	BLS	CAOB	East Tianshan	90.5000	41.9667	Felsic		318	318	6.79	28.70	0.1491	0.512822	13	-0.24	3.59	5.50	5.52	5.52	0.63	0.77	0.63	0.78	0.63	Zhang DY et al.	2013	Journal of Asian Earth Sciences	73	317-333
1391	KT-2	CAOB	Altai, unit 3	89.9272	47.2094	Granite	Keketuhai	407	407	5.62	29.98	0.1133	0.512398	5	-0.42	-4.68	-0.35	-0.35	-0.35	1.18	1.14	1.18	0.72	1.18	Chen B and Jahn BM.	2002	Geological Magazine	139(1)	1-13
1392	KT-1	CAOB	Altai, unit 3	89.9272	47.2094	Granite	Keketuhai	407	407	6.21	28.06	0.1338	0.512346	3	-0.32	-5.70	-2.43	-2.43	-2.43	1.35	1.53	1.35	0.72	1.35	Chen B and Jahn BM.	2002	Geological Magazine	139(1)	1-13
1393	AT-1	CAOB	Altai, unit 4	88.1736	47.8056	A-type Granite	Jiangjunshan	151	151	4.31	10.11	0.2576	0.512922	5	0.31	5.54	4.37	4.37	4.37	0.59	-0.80	0.59	0.72	0.59	Chen B and Jahn BM.	2002	Geological Magazine	139(1)	1-13
1394	AT-1	CAOB	Altai, unit 4	88.1736	47.8056	A-type Granite	Jiangjunshan	151	151	3.99	8.91	0.2711	0.512930	4	0.38	5.70	4.26	4.26	4.26	0.60	-0.59	0.60	0.72	0.60	Chen B and Jahn BM.	2002	Geological Magazine	139(1)	1-13
1395	RH155	CAOB	NE China	125.0000	45.0000	Rhyolite		152	152	8.35	47.66	0.1159	0.512713		-0.41	1.46	3.10	3.03	3.03	0.69	0.68	0.69	0.50		Wang PJ et al.	2006	Geochemical Journal	40(2)	147-159
1396	RH126	CAOB	NE China	125.0000	45.0000	Rhyolite		125	150	8.65	43.56	0.1141	0.512394		-0.42	-4.76	-3.40	-3.18	-3.18	1.20	1.16	1.20	1.00		Wang PJ et al.	2006	Geochemical Journal	40(2)	147-159
1397	RH113	CAOB	NE China	125.0000	45.0000	Rhyolite		125	150	7.91	33.71	0.1396	0.512709		-0.29	1.38	2.40	2.48	2.48	0.74	0.91	0.74	1.00		Wang PJ et al.	2006	Geochemical Journal	40(2)	147-159
1398	PE131	CAOB	NE China	125.0000	45.0000	Rhyolite		125	150	3.57	20.22	0.1103	0.512643		-0.44	0.10	1.60	1.75	1.75	0.80	0.75	0.80	0.60		Wang PJ et al.	2006	Geochemical Journal	40(2)	147-159
1399	DA130	CAOB	NE China	125.0000	45.0000	Dacite		125	150	14.90	66.97	0.1335	0.512723		-0.32	1.66	2.70	2.87	2.87	0.70	0.81	0.70	0.60		Wang PJ et al.	2006	Geochemical Journal	40(2)	147-159
1400	AN249	CAOB	NE China	125.0000	45.0000	Andesite		106	150	8.19	38.46	0.1370	0.512700		-0.30	1.21	3.40	2.35	2.35	0.75	0.89	0.75	0.80		Wang PJ et al.	2006	Geochemical Journal	40(2)	147-159
1401	AN157	CAOB	NE China	125.0000	45.0000	Andesite		152	152	2.35	10.60	0.1369	0.512848		-0.30	4.10	5.30	5.25	5.25	0.51	0.60	0.51	0.40		Wang PJ et al.	2006	Geochemical Journal	40(2)	147-159
1402	AN156	CAOB	NE China	125.0000	45.0000	Andesite		152	152	3.83	19.20	0.1238	0.512614		-0.37	-0.47	1.00	0.94	0.94	0.86	0.91	0.86	0.70		Wang PJ et al.	2006	Geochemical Journal	40(2)	147-159
1403	AN153	CAOB	NE China	125.0000	45.0000	Andesite		152	152	3.62	22.56	0.1124	0.512638		-0.43	0.00	1.80	1.63	1.63	0.81	0.77	0.81	0.60		Wang PJ et al.	2006	Geochemical Journal	40(2)	147-159
1404	AN134	CAOB	NE China	125.0000	45.0000	Andesite		125	150	15.60	63.41	0.1218	0.512685		-0.38	0.92	2.20	2.35	2.35	0.75	0.77	0.75	0.60		Wang PJ et al.	2006	Geochemical Journal	40(2)	147-159
1405	AN122	CAOB	NE China	125.0000	45.0000	Andesite		125	150	5.57	21.33	0.1430	0.512756		-0.27	2.30	3.10	3.33	3.33	0.67	0.85	0.67	0.60		Wang PJ et al.	2006	Geochemical Journal	40(2)	147-159
1406	TR146	CAOB	NE China	125.0000	45.7000	Trachyte	Songliao basin	152	152	3.44	19.86	0.1211	0.512666		-0.38	0.55	2.00	2.01	2.33	0.78	0.80	0.78	0.60		Wang PJ et al.	2006	Geochemical Journal	40(2)	149-159
1407	Bt131	CAOB	NE China	125.0000	45.7000	Basaltic trachyandesite	Songliao basin	125	150	6.37	28.7	0.1330	0.512751		-0.32	2.20	3.20	3.42	3.20	0.66	0.75	0.66	0.60		Wang PJ et al.	2006	Geochemical Journal	40(2)	149-159
1408	TA147	CAOB	NE China	125.0000	45.7000	Trachyandesite	Songliao basin	152	152	3.35	17.06	0.1133	0.512612		-0.42	-0.51	1.00	1.11	1.00	0.85	0.82	0.85	0.70		Wang PJ et al.	2006	Geochemical Journal	40(2)	149-159
1409	Bt155	CAOB	NE China	125.0000	45.7000	Basaltic trachyandesite	Songliao basin	152	152	4.97	23.4	0.1220	0.512584		-0.38	-1.05	0.40	0.39	0.27	0.91	0.94	0.91	0.80		Wang PJ et al.	2006	Geochemical Journal	40(2)	149-159
1410	11X07-5	CAOB	NE China	117.6700	44.5300	Andesite		276	276	8.60	47.20	0.1113	0.512489		-0.43	-2.91	-0.20	0.10	0.10	1.03	0.98	1.03		0.85	Gao XF et al.	2016	Lithos	252-253	173-184
1411	11X07-2	CAOB	NE China	117.6700	44.5300	Andesite		276	276	8.50	47.30	0.1108	0.512567		-0.44	-1.38	1.40	1.63	1.63	0.91	0.86	0.91		0.73	Gao XF et al.	2016	Lithos	252-253	173-184
1412	11X07-1	CAOB	NE China	117.6700	44.5300	Andesite		276	276	8.50	47.30	0.1101	0.512544		-0.44	-1.83	0.90	1.21	1.21	0.94	0.89	0.94		0.76	Gao XF et al.	2016	Lithos	252-253	173-184
1413	11X04-8	CAOB	NE China	117.6700	44.5300	Andesite		276	276	12.30	74.30	0.1015	0.512021		-0.48	-12.04	-9.00	-8.69	-8.69	1.75	1.53	1.75		1.57	Gao XF et al.	2016	Lithos	252-253	173-184
1414	11X04-6	CAOB	NE China	117.6700	44.5300	Andesite		276	276	12.00	72.70	0.1012	0.512021		-0.49	-12.04	-9.00	-8.68	-8.68	1.75	1.53	1.75		1.57	Gao XF et al.	2016	Lithos	252-253	173-184
1415	11X04-5	CAOB	NE China	117.6700	44.5300	Andesite		276	276	12.10	73.20	0.1020	0.512019		-0.48	-12.07	-9.00	-8.75	-8.75	1.75	1.54	1.75		1.57	Gao XF et al.	2016	Lithos	252-253	173-184
1416	11X04-3	CAOB	NE China	117.6700	44.5300	Andesite		276	276	12.20	73.40	0.1022	0.512022		-0.48	-12.02	-9.00	-8.69	-8.69	1.75	1.54	1.75		1.57	Gao XF et al.	2016	Lithos	252-253	173-184
1417	11X04-2	CAOB	NE China	117.6700	44.5300	Andesite		276	276	11.90	72.70	0.1011	0.512017		-0.49	-12.11	-9.00	-8.75	-8.75	1.75	1.53	1.75		1.57	Gao XF et al.	2016	Lithos	252-253	173-184
1418	11X04-13	CAOB	NE China	117.6700	44.5300	Andesite		276	276	12.80	77.20	0.1015	0.512025		-0.48	-11.96	-8.90	-8.61	-8.61	1.74	1.53	1.74		1.56	Gao XF et al.	2016	Lithos	252-253	173-184
1419	11X04-12	CAOB	NE China	117.6700	44.5300	Andesite		276	276	12.70	77.20	0.1009	0.512053		-0.49	-11.41	-8.30	-8.04	-8.04	1.70	1.48	1.70		1.51	Gao XF et al.	2016	Lithos	252-253	173-184
1420	11X04-11	CAOB	NE China	117.6700	44.5300	Andesite		276	276	13.00	78.70	0.1015	0.512045		-0.48	-11.57	-8.50	-8.22	-8.22	1.71	1.50	1.71		1.53	Gao XF et al.	2016	Lithos	252-253	173-184
1421	11X04-10	CAOB	NE China	117.6700	44.5300	Andesite		276	276	12.20	73.70	0.1021	0.512024		-0.48	-11.98	-8.90	-8.65	-8.65	1.74	1.54	1.74		1.56	Gao XF et al.	2016	Lithos	252-253	173-184
1422	11X04-1	CAOB	NE China	117.6700	44.5300	Andesite		276	276	12.20	73.50	0.1018	0.512030		-0.48	-11.86	-8.80	-8.52	-8.52	1.73	1.52	1.73		1.55	Gao XF et al.	2016	Lithos	252-253	173-184
1423	11X03-4	CAOB	NE China	117.6700	44.5300	Andesite		276	276	11.90	72.80	0.1004	0.512031		-0.49	-11.84	-8.70	-8.46	-8.46	1.73	1.50	1.73		1.55	Gao XF et al.	2016	Lithos	252-253	173-184
1424	11X02-2	CAOB	NE China	117.6700	44.5300	Andesite		276	276	9.50	52.00	0.1124	0.512493		-0.43	-2.83	-0.10	0.14	0.14	1.03	0.99	1.03		0.85	Gao XF et al.	2016	Lithos	252-253	173-184
1425		CAOB	Transbaikalia and Mongolia	117.8333	51.3000	Monzonite		160	160	4.41	24.65	0.1081	0.512489		-0.45	-2.91	-1.10	-1.10	-1.10	1.04	0.95	1.04	0.95	1.04	Berzina AP et al.	2014	Journal of Asian Earth Sciences	79	655-665
1426		CAOB	Transbaikalia and Mongolia	117.8333	51.3000	Granite		159	159	5.59	38.10	0.0887	0.512386		-0.55	-4.92	-2.73	-2.73	-2.73	1.17	0.93	1.17	0.93	1.17	Berzina AP et al.	2014	Journal of Asian Earth Sciences	79	655-665
1427		CAOB	Transbaikalia and Mongolia	117.8333	51.3000	Monzonite porphyry		159	159	5.97	35.49	0.1017	0.512643		-0.48	0.10	2.03	2.03	2.03	0.78	0.69	0.78	0.69	0.78	Berzina AP et al.	2014	Journal of Asian Earth Sciences	79	655-665
1428		CAOB	Transbaikalia and Mongolia	117.8333	51.3000	Granite porphyry		155	155	5.23	32.38	0.0977	0.512645		-0.50	0.14	2.10	2.10	2.10	0.77	0.66	0.77	0.66	0.77	Berzina AP et al.	2014	Journal of Asian Earth Sciences	79	655-665
1429	BB9	NCC	NCC	116.3303	40.3442	Alkali-feldspar granite	Badaling	130	150			0.0828	0.511876		-0.58	-14.86	-13.0	-12.7	-12.7	1.97	1.48	1.97	1.48	1.98	Qian Q et al.	2002	Acta Petrologica Sinica	18(3)	275-292
1430	BB6	NCC	NCC	116.3303	40.3442	Qtz monzonite	Badaling	130	150			0.0933	0.512066		-0.53	-11.16	-9.44	-9.18	-9.18	1.69	1.37	1.69	1.37	1.69	Qian Q et al.	2002	Acta Petrologica Sinica	18(3)	275-292
1431	BB5	NCC	NCC	116.3400	40.3322	Qtz diorite	Badaling	130	150			0.0891	0.511967		-0.55	-13.09	-11.3	-11.0	-11.0	1.84	1.44	1.84	1.45	1.85	Qian Q et al.	2002	Acta Petrologica Sinica	18(3)	275-292
1432	BB15	NCC	NCC	116.2064	40.3825	Monzonitic granite	Badaling	130	150			0.0820	0.511748		-0.58	-17.36	-15.5	-15.2	-15.2	2.17	1.62	2.17	1.62	2.18	Qian Q et al.	2002	Acta Petrologica Sinica	18(3)	275-292
1433	BB14	NCC	NCC	116.2064	40.3825	Monzonitic granite	Badaling	130	150			0.0934	0.511808		-0.53	-16.19	-14.5	-14.2	-14.2	2.09	1.70	2.09	1.70	2.10	Qian Q et al.	2002	Acta Petrologica Sinica	18(3)	275-292
1434	BB11	NCC	NCC	116.2064	40.3825	Monzonitic granite	Badaling	130	150			0.0915	0.511512		-0.53	-21.96	-20.2	-20.0	-20.0	2.56	2.04	2.56	2.04	2.57	Qian Q et al.	2002	Acta Petrologica Sinica	18(3)	275-292
1435	BB10	NCC	NCC	116.2064	40.3825	Monzonitic granite	Badaling	130	150			0.0912	0.511679		-0.54	-18.71	-17.0	-16.7	-16.7	2.30	1.83	2.30	1.83	2.30	Qian Q et al.	2002	Acta Petrologica Sinica	18(3)	275-292
1436	M-10	NCC	NCC	116.0264	40.2453	Granite	Heishantuo	201	201	4.03	26.74	0.0913	0.511581	19	-0.54	-20.62	-17.9	-17.9	-17.9	2.44	1.95	2.44	1.95	2.44	Wang Y and Jiao Y.	2015	Geotectonica et Metallogenia	39(1)	139-152
1437	M-07	NCC	NCC	116.0264	40.2453	Granite	Heishantuo	201	201	5.71	37.92	0.0912	0.511711	17	-0.54	-18.08	-15.4	-15.4	-15.4	2.23	1.79	2.23	1.79	2.23	Wang Y and Jiao Y.	2015	Geotectonica et Metallogenia	39(1)	139-152
1438	M-03	NCC	NCC	116.0389	40.2744	Syenite	Dashipo	201	201	11.38	66.40	0.1037	0.511901	19	-0.47	-14.38	-12.0	-12.0	-12.0	1.95	1.73	1.95	1.73	1.96	Wang Y and Jiao Y.	2015	Geotectonica et Metallogenia	39(1)	139-152
1439	M-01	NCC	NCC	116.0389	40.2744	Syenite	Dashipo	201	201	11.69	70.30	0.1006	0.511892	19	-0.49	-14.55	-12.1	-12.1	-12.1	1.96	1.69	1.96	1.69	1.97	Wang Y and Jiao Y.	2015	Geotectonica et Metallogenia	39(1)	139-152
1440	SC1-4	CAOB	Junggar	90.4000	44.8250	Andesite		329	329	4.28	18.76	0.0376	0.512873		-0.81	4.58	7.0	11.3	11.3	0.33	0.24	0.17	0.56		Wu XQ et al.	2008	Acta Petrologica Sinica	206（1-4）	42-57
1441	BSZK1-01	CAOB	Beishan	97.5173	40.9092	Porphyritic monzogranite	Jiujin	270	270	5.56	33.98	0.1002	0.512147	10	-0.49	-9.58	-6.25	-6.25	-6.25	1.55	1.35	1.55	1.35	1.60	Fan HH et al	2006	Uranium geology	(2)	90-98
1442	BS41	CAOB	Beishan	97.5173	40.9092	Porphyritic monzogranite	Jiujin	270	270	2.91	16.24	0.1091	0.512168	13	-0.45	-9.17	-6.15	-6.15	-6.15	1.54	1.43	1.54	1.43	1.56	Fan HH et al	2006	Uranium geology	(2)	90-98
1443	BS34	CAOB	Beishan	97.5173	40.9092	Tonalite	Jiujin	430	430	4.30	22.73	0.1184	0.512089	8	-0.40	-10.71	-6.41	-6.41	-6.41	1.69	1.69	1.69	1.69	1.69	Fan HH et al	2006	Uranium geology	(2)	90-98
1444	BS32	CAOB	Beishan	97.5173	40.9092	Tonalite	Jiujin	430	430	4.26	24.04	0.1063	0.512075	12	-0.46	-10.98	-6.02	-6.02	-6.02	1.66	1.52	1.66	1.52	1.71	Fan HH et al	2006	Uranium geology	(2)	90-98
1445	BS25	CAOB	Beishan	97.5173	40.9092	Tonalite	Jiujin	430	430	5.60	29.17	0.1219	0.512098	9	-0.38	-10.53	-6.43	-6.43	-6.43	1.69	1.74	1.69	1.74	1.67	Fan HH et al	2006	Uranium geology	(2)	90-98
1446	BS21	CAOB	Beishan	97.5173	40.9092	Porphyritic monzogranite	Jiujin	270	270	3.91	23.45	0.1026	0.512156	14	-0.48	-9.40	-6.16	-6.16	-6.16	1.54	1.36	1.54	1.36	1.58	Fan HH et al	2006	Uranium geology	(2)	90-98
1447	S-9	CAOB	SMXAB	116.2375	45.7589	Adamellite	Bayinsuhetu	296	296			0.1201	0.512551		-0.39	-1.70	1.19	1.20	1.20	0.96	0.98	0.96	0.98	0.96	Shen XL et al.	2012	ournal of Jilin University(Earth Science Editior	42	281-295
1448	S-8	CAOB	SMXAB	116.2375	45.7589	Granite	Bayinsuhetu	200	200			0.1058	0.512506		-0.46	-2.57	-0.24	-0.26	-0.26	1.00	0.91	1.00	0.91	1.00	Shen XL et al.	2012	ournal of Jilin University(Earth Science Editior	42	281-295
1449	S-6	CAOB	SMXAB	116.2375	45.7589	Adamellite	Bayinsuhetu	296	296			0.1205	0.512569		-0.39	-1.35	1.55	1.53	1.53	0.93	0.95	0.93	0.95	0.94	Shen XL et al.	2012	ournal of Jilin University(Earth Science Editior	42	281-295
1450	S-2	CAOB	SMXAB	116.2375	45.7589	Adamellite	Bayinsuhetu	296	296			0.1200	0.512537		-0.39	-1.97	0.92	0.93	0.93	0.98	1.00	0.98	1.00	0.99	Shen XL et al.	2012	ournal of Jilin University(Earth Science Editior	42	281-295
1451	S-14	CAOB	SMXAB	116.2375	45.7589	Granite	Bayinsuhetu	200	200			0.1232	0.512558		-0.37	-1.56	0.32	0.32	0.32	0.95	1.00	0.95	1.00	0.96	Shen XL et al.	2012	ournal of Jilin University(Earth Science Editior	42	281-295
1452	S-12	CAOB	SMXAB	116.2375	45.7589	Adamellite	Bayinsuhetu	296	296			0.1136	0.512549		-0.42	-1.74	1.41	1.40	1.40	0.94	0.92	0.94	0.92	0.95	Shen XL et al.	2012	ournal of Jilin University(Earth Science Editior	42	281-295
1453	S-11	CAOB	SMXAB	116.2375	45.7589	Adamellite	Bayinsuhetu	296	296			0.1317	0.512578		-0.33	-1.17	1.29	1.28	1.28	0.95	1.06	0.95	1.07	0.96	Shen XL et al.	2012	ournal of Jilin University(Earth Science Editior	42	281-295
1454	S-10	CAOB	SMXAB	116.2375	45.7589	Adamellite	Bayinsuhetu	296	296			0.1456	0.512609		-0.26	-0.57	1.37	1.36	1.36	0.95	1.21	0.95	1.21	0.95	Shen XL et al.	2012	ournal of Jilin University(Earth Science Editior	42	281-295
1455	GPG-9	CAOB	Beishan	97.1694	41.7028	Granodiorite porphyry	Gongpoquan	340	340	3.20	18.10	0.1308	0.512651	4	-0.34	0.25	3.11	3.11	3.11	0.84	0.92	0.84	0.92	0.79	Jiang SH et al.	2002	Geology Geochemistry	30(2)	25-33
1456	GPG-1	CAOB	Beishan	97.1694	41.7028	Dacite porphyry	Gongpoquan	435	435	3.22	14.82	0.1314	0.512307	4	-0.33	-6.46	-2.83	-2.83	-2.83	1.40	1.56	1.40	1.56	1.34	Jiang SH et al.	2002	Geology Geochemistry	30(2)	25-33
1457	ZO47	CAOB	Tianshan	102.7808	43.5577	**Metadacite**		420	420	5.27	20.20	0.1576	0.512891		-0.20	4.94	7.20	7.03	7.03	0.59	0.70	0.59			Helo C et al.	2006	Chemical Geology	227(3-4)	236-257
1458	TS95	CAOB	Tianshan	97.1500	45.0500	Andesite		400	400	8.66	48.30	0.1083	0.512668		-0.45	0.59	5.10	5.10	5.10	0.73	0.70	0.73		0.56	Helo C et al.	2006	Chemical Geology	227(3-4)	236-257
1459	TS92	CAOB	Tianshan	97.1156	45.1195	Andesite		360	360	3.52	16.60	0.1278	0.512750		-0.35	2.18	5.40	5.35	5.35	0.67	0.71	0.67		0.54	Helo C et al.	2006	Chemical Geology	227(3-4)	236-257
1460	TS91	CAOB	Tianshan	97.0816	45.0698	Gneiss		361	361	5.23	35.40	0.0892	0.512410		-0.55	-4.45	0.50	0.50	0.50	1.07	0.91	1.07		0.78	Helo C et al.	2006	Chemical Geology	227(3-4)	236-257
1461	TS90	CAOB	Tianshan	97.1556	45.0329	Dacite		360	360	5.41	26.20	0.1246	0.512162		-0.37	-9.29	-6.00	-5.97	-5.97	1.59	1.69	1.59		1.40	Helo C et al.	2006	Chemical Geology	227(3-4)	236-257
1462	TS90 repeat	CAOB	Tianshan	97.1556	45.0329	Dacite		360	360	5.12	24.80	0.1248	0.512152		-0.37	-9.48	-6.20	-6.18	-6.18	1.61	1.71	1.61		1.50	Helo C et al.	2006	Chemical Geology	227(3-4)	236-257
1463	TS89	CAOB	Tianshan	97.1617	45.0366	Granodiorite		289	289	8.87	55.30	0.0969	0.512639		-0.51	0.02	4.10	3.70	3.70	0.75	0.67	0.75		0.54	Helo C et al.	2006	Chemical Geology	227(3-4)	236-257
1464	TS113	CAOB	Tianshan	97.9776	45.4475	Gneiss		397	397	3.54	24.90	0.0860	0.512683		-0.56	0.88	5.70	6.49	6.49	0.61	0.56	0.61		0.46	Helo C et al.	2006	Chemical Geology	227(3-4)	236-257
1465	TS111	CAOB	Tianshan	97.9776	45.4475	Gneiss		396	396	5.61	28.70	0.1181	0.512638		-0.40	0.00	4.00	3.97	3.97	0.82	0.82	0.82		0.66	Helo C et al.	2006	Chemical Geology	227(3-4)	236-257
1466	NG66	CAOB	Tianshan	101.5680	43.6045	Metaplagiogranite		420	420	3.27	10.10	0.1954	0.513113		-0.01	9.27	9.30	9.34	9.34	0.40	0.31	0.40			Helo C et al.	2006	Chemical Geology	227(3-4)	236-257
1467	GS9	CAOB	Tianshan	103.0766	43.7948	MetaGranodiorite		420	420	2.02	12.30	0.0992	0.512848		-0.50	4.10	9.30	9.32	9.32	0.42	0.40	0.40		0.28	Helo C et al.	2006	Chemical Geology	227(3-4)	236-257
1468	GS7	CAOB	Tianshan	103.1888	43.7705	Metarhyolite		420	420	1.58	9.25	0.1034	0.512752		-0.47	2.22	7.20	7.22	7.22	0.57	0.55	0.57		0.42	Helo C et al.	2006	Chemical Geology	227(3-4)	236-257
1469	GS6	CAOB	Tianshan	103.1240	43.7823	Metadacite		420	420	1.90	9.36	0.1229	0.512815		-0.38	3.45	7.40	7.41	7.41	0.56	0.56	0.56		0.41	Helo C et al.	2006	Chemical Geology	227(3-4)	236-257
1470	GS5	CAOB	Tianshan	103.1240	43.7823	Metadiorit		420	420	1.38	5.92	0.1410	0.512870		-0.28	4.53	7.47	7.51	7.51	0.55	0.59	0.55			Helo C et al.	2006	Chemical Geology	227(3-4)	236-257
1471	BH81	CAOB	Tianshan	100.7632	44.4436	Metagranite		467	467	5.59	29.00	0.1165	0.512509		-0.41	-2.52	1.80	2.26	2.26	1.01	1.01	1.01		0.84	Helo C et al.	2006	Chemical Geology	227(3-4)	236-257
1472	BH80	CAOB	Tianshan	100.7785	44.4511	Metagranite		426	426	7.19	34.70	0.1253	0.512496		-0.36	-2.77	1.10	1.11	1.11	1.07	1.13	1.07		0.94	Helo C et al.	2006	Chemical Geology	227(3-4)	236-257

1473	BH79	CAOB	Tianshan	100.7814	44.4537	Metarhyolite		420	420	1.87	10.60	0.1067	0.512664		-0.46	0.51	5.30	5.33	5.33	0.73	0.69	0.73		0.55	Helo C et al.	2006	Chemical Geology	227(3-4)	236-257
1474	BH78	CAOB	Tianshan	100.7841	44.4568	Granodiorite		420	420	3.38	19.00	0.1076	0.512542		-0.45	-1.87	2.90	2.90	2.90	0.92	0.87	0.92		0.73	Helo C et al.	2006	Chemical Geology	227(3-4)	236-257
1475	BGT-w	CAOB	Junggar	84.5120	45.7860	Tuff		320	320	4.42	20.10	0.1328	0.512784	14	-0.32	2.85	2.74	5.46	5.46	0.63	0.69	0.63	0.69	0.63	Shen P et al.	2009	Gondwana Research	16(2)	227-242
1476	BGT8-700	CAOB	Western Junggar	84.4736	45.4833	Diorite		320	320	2.07	8.12	0.1544	0.512963		-0.22	6.34	8.07	8.07	8.07	0.42	0.48	0.42	0.48	0.42	Shen P et al.	2009	Gondwana Research	16(2)	227-242
1477	BGT5-4	CAOB	Western Junggar	84.4736	45.4833	Diorite		320	320	3.67	13.57	0.1636	0.512947		-0.17	6.03	7.38	7.38	7.38	0.48	0.62	0.48	0.62	0.48	Shen P et al.	2009	Gondwana Research	16(2)	227-242
1478	BGT5-3	CAOB	Western Junggar	84.4736	45.4833	Diorite		320	320	4.11	18.70	0.1327	0.512865		-0.33	4.43	7.04	7.04	7.04	0.50	0.54	0.50	0.54	0.50	Shen P et al.	2009	Gondwana Research	16(2)	227-242
1479	BGT5-2	CAOB	Western Junggar	84.4736	45.4833	Qtz diorite		320	320	3.21	14.14	0.1372	0.512887		-0.30	4.86	7.29	7.29	7.29	0.48	0.52	0.48	0.52	0.48	Shen P et al.	2009	Gondwana Research	16(2)	227-242
1480	BGT5-1	CAOB	Western Junggar	84.4736	45.4833	Diorite		320	320	3.68	17.32	0.1286	0.512859		-0.35	4.31	7.09	7.09	7.09	0.50	0.52	0.50	0.52	0.50	Shen P et al.	2009	Gondwana Research	16(2)	227-242
1481	BGT4-3	CAOB	Western Junggar	84.4736	45.4833	Diorite		320	320	3.73	17.10	0.1320	0.512887		-0.33	4.86	7.50	7.50	7.50	0.47	0.49	0.47	0.49	0.47	Shen P et al.	2009	Gondwana Research	16(2)	227-242
1482	BGT-4-2	CAOB	Western Junggar	84.4736	45.4833	Diorite		320	320	2.63	11.66	0.1363	0.512890		-0.31	4.92	7.38	7.38	7.38	0.48	0.51	0.48	0.51	0.48	Shen P et al.	2009	Gondwana Research	16(2)	227-242
1483	BGT4-2	CAOB	Western Junggar	84.4736	45.4833	Diorite		320	320	3.05	13.47	0.1372	0.512902		-0.30	5.15	7.58	7.58	7.58	0.46	0.49	0.46	0.49	0.46	Shen P et al.	2009	Gondwana Research	16(2)	227-242
1484	BGT-4-1	CAOB	Western Junggar	84.4736	45.4833	Diorite		320	320	2.73	11.92	0.1388	0.512906		-0.29	5.23	7.59	7.59	7.59	0.46	0.50	0.46	0.50	0.46	Shen P et al.	2009	Gondwana Research	16(2)	227-242
1485	BGT4-1	CAOB	Western Junggar	84.4736	45.4833	Diorite		320	320	2.76	11.95	0.1400	0.512913		-0.29	5.36	7.68	7.68	7.68	0.45	0.49	0.45	0.49	0.45	Shen P et al.	2009	Gondwana Research	16(2)	227-242
1486	BGT-3-3	CAOB	Western Junggar	84.4736	45.4833	Diorite		320	320	2.45	10.41	0.1425	0.512862		-0.28	4.37	6.58	6.58	6.58	0.54	0.62	0.54	0.62	0.54	Shen P et al.	2009	Gondwana Research	16(2)	227-242
1487	BGT-3-2	CAOB	Western Junggar	84.4736	45.4833	Diorite		320	320	2.28	10.00	0.1381	0.512853		-0.30	4.19	6.59	6.59	6.59	0.54	0.60	0.54	0.60	0.54	Shen P et al.	2009	Gondwana Research	16(2)	227-242
1488	BGT-3-1	CAOB	Western Junggar	84.4736	45.4833	Diorite		320	320	1.93	8.22	0.1422	0.512886		-0.28	4.84	7.06	7.06	7.06	0.50	0.56	0.50	0.56	0.50	Shen P et al.	2009	Gondwana Research	16(2)	227-242
1489	BGT-2	CAOB	Western Junggar	84.4736	45.4833	Qtz diorite		320	320	3.85	18.87	0.1232	0.512776		-0.37	2.69	5.69	5.69	5.69	0.61	0.63	0.61	0.63	0.61	Shen P et al.	2009	Gondwana Research	16(2)	227-242
1490	BGT-1	CAOB	Western Junggar	84.4736	45.4833	Diorite		320	320	2.35	10.08	0.1409	0.512899		-0.28	5.09	7.37	7.37	7.37	0.48	0.53	0.48	0.53	0.48	Shen P et al.	2009	Gondwana Research	16(2)	227-242
1491	MG9811-1	CAOB	West Junggar	84.0292	45.5461	Perilla granite	Miaoergou	296	296	7.93	32.10	0.1500	0.512809	7	-0.24	3.34	5.10	5.10	5.10	0.64	0.82	0.64	0.82	0.64	Geng HY et al.	2009	Chemical Geology	266(3-4)	364-389
1492	MG9808-4	CAOB	West Junggar	83.9056	45.5083	Perilla granite	Miaoergou	305	305	7.54	32.80	0.1400	0.512801	6	-0.29	3.18	5.39	5.39	5.39	0.63	0.72	0.63	0.72	0.63	Geng HY et al.	2009	Chemical Geology	266(3-4)	364-389
1493	MG9803-2	CAOB	West Junggar	83.9528	45.5222	Perilla granite	Miaoergou	305	305	7.28	30.70	0.1400	0.512855	8	-0.29	4.23	6.44	6.44	6.44	0.54	0.61	0.54	0.61	0.54	Geng HY et al.	2009	Chemical Geology	266(3-4)	364-389
1494	MG5-1	CAOB	West Junggar	83.8500	45.5194	Diorite	Miaoergou	305	305	4.41	19.20	0.1400	0.512894	10	-0.29	4.99	7.20	7.20	7.20	0.48	0.53	0.48	0.53	0.48	Geng HY et al.	2009	Chemical Geology	266(3-4)	364-389
1495	MG26*	CAOB	West Junggar	83.8333	45.6333	Alk-feld granite		305	305	5.83	28.10	0.1300	0.512911		-0.34	5.33	7.92	7.92	7.92	0.42	0.44	0.42	0.44	0.42	Geng HY et al.	2009	Chemical Geology	266(3-4)	364-389
1496	MG24*	CAOB	West Junggar	83.8333	45.6333	Alk-feld granite		305	305	6.63	30.50	0.1300	0.512890		-0.34	4.92	7.51	7.51	7.51	0.45	0.47	0.45	0.47	0.45	Geng HY et al.	2009	Chemical Geology	266(3-4)	364-389
1497	MG19*	CAOB	West Junggar	83.8333	45.6333	Alk-feld granite		305	305	8.42	36.60	0.1400	0.512913		-0.29	5.36	7.57	7.57	7.57	0.45	0.49	0.45	0.49	0.45	Geng HY et al.	2009	Chemical Geology	266(3-4)	364-389
1498	MG164-2	CAOB	West Junggar	83.9489	45.5167	Perilla granite	Miaoergou	305	305	10.12	42.90	0.1400	0.512817	7	-0.29	3.49	5.70	5.70	5.70	0.60	0.69	0.60	0.69	0.60	Geng HY et al.	2009	Chemical Geology	266(3-4)	364-389
1499	MG136-1	CAOB	West Junggar	83.9403	45.4917	Alkali feldspar granite	Miaoergou	308	308	9.96	39.30	0.1500	0.512954	7	-0.24	6.16	8.00	8.00	8.00	0.42	0.47	0.42	0.47	0.42	Geng HY et al.	2009	Chemical Geology	266(3-4)	364-389
1500	MG129-4	CAOB	West Junggar	83.9472	45.5222	Diorite	Miaoergou	305	305	9.96	39.30	0.1500	0.512954	7	-0.24	6.16	7.98	7.98	7.98	0.41	0.47	0.41	0.47	0.41	Geng HY et al.	2009	Chemical Geology	266(3-4)	364-389
1501	MG123-2	CAOB	West Junggar	83.8722	45.5139	Perilla granite	Miaoergou	305	305	8.17	33.40	0.1500	0.512952	9	-0.24	6.13	7.94	7.94	7.94	0.42	0.47	0.42	0.47	0.42	Geng HY et al.	2009	Chemical Geology	266(3-4)	364-389
1502	KM9918-3	CAOB	West Junggar	83.7514	45.6508	Alkali feldspar granite	Karamay	305	305	5.43	25.60	0.1300	0.512957	9	-0.34	6.22	8.82	8.82	8.82	0.35	0.35	0.35	0.35	0.35	Geng HY et al.	2009	Chemical Geology	266(3-4)	364-389
1503	KM9802-6	CAOB	West Junggar	84.8236	45.7022	Alkali feldspar granite	Karamay	305	305	6.51	29.30	0.1300	0.512773	8	-0.34	2.63	5.23	5.23	5.23	0.64	0.69	0.64	0.69	0.64	Geng HY et al.	2009	Chemical Geology	266(3-4)	364-389
1504	KM9802-11	CAOB	West Junggar	84.8230	45.7056	Dioritic MME	Karamay	306	306	5.49	26.70	0.1200	0.512830	8	-0.39	3.75	6.74	6.74	6.74	0.52	0.52	0.52	0.52	0.52	Geng HY et al.	2009	Chemical Geology	266(3-4)	364-389
1505	KM-11	CAOB	West Junggar	85.0439	45.7083	Diorite	Karamay	305	305	10.11	35.90	0.1700	0.512939	7	-0.14	5.87	6.91	6.91	6.91	0.50	0.74	0.50	0.74	0.50	Geng HY et al.	2009	Chemical Geology	266(3-4)	364-389
1506	HONG1	CAOB	West Junggar	85.0569	45.9489	Alkali feldspar granite	Hongshan	305	305	7.53	45.70	0.1000	0.512838	9	-0.49	3.90	7.66	7.66	7.66	0.44	0.42	0.44	0.42	0.44	Geng HY et al.	2009	Chemical Geology	266(3-4)	364-389
1507	Hatu	CAOB	West Junggar	83.9056	45.4100	Alkali feldspar granite	Hatu	305	305	10.29	45.30	0.1400	0.512935	7	-0.29	5.79	8.00	8.00	8.00	0.41	0.45	0.41	0.45	0.41	Geng HY et al.	2009	Chemical Geology	266(3-4)	364-389
1508	AK6	CAOB	West Junggar	84.3583	45.7597	Diorite	Akbastau	304	304	3.18	13.80	0.1400	0.512752	7	-0.29	2.22	4.42	4.42	4.42	0.70	0.82	0.70	0.82	0.70	Geng HY et al.	2009	Chemical Geology	266(3-4)	364-389
1509	AK154-2	CAOB	West Junggar	84.7589	45.4100	Alkali feldspar granite	Akbastau	305	305	3.77	19.90	0.1100	0.512911	9	-0.44	5.33	8.70	8.70	8.70	0.36	0.35	0.36	0.35	0.36	Geng HY et al.	2009	Chemical Geology	266(3-4)	364-389
1510	HPB07	CAOB	Tianshan	92.0000	42.5000	Diorite		440	440	4.01	18.40	0.1318	0.512822		-0.33	3.59	7.30	7.24	7.24	0.59	0.61	0.59	0.61		Zheng JH et al.	2018	Lithos	302-303	455-466
1511	HPB05	CAOB	Tianshan	92.0000	42.5000	Diorite		440	440	3.80	17.20	0.1336	0.512827		-0.32	3.69	7.30	7.23	7.23	0.59	0.62	0.59	0.62		Zheng JH et al.	2018	Lithos	302-303	455-466
1512	HPB04	CAOB	Tianshan	92.0000	42.5000	Diorite		440	440	3.44	15.30	0.1359	0.512804		-0.31	3.24	6.70	6.65	6.65	0.63	0.68	0.63	0.68		Zheng JH et al.	2018	Lithos	302-303	455-466
1513	HPB02	CAOB	Tianshan	92.0000	42.5000	Diorite		440	440	3.35	14.50	0.1397	0.512801		-0.29	3.18	6.40	6.38	6.38	0.66	0.72	0.66	0.72		Zheng JH et al.	2018	Lithos	302-303	455-466
1514	HPB01	CAOB	Tianshan	92.0000	42.5000	Diorite		440	440	3.38	14.50	0.1409	0.512803		-0.28	3.22	6.40	6.35	6.35	0.66	0.73	0.66	0.73		Zheng JH et al.	2018	Lithos	302-303	455-466
1515	HPA07	CAOB	Eastern Tianshan	91.9333	42.6500	Granite	Huangtupo	435	435	2.85	13.50	0.1276	0.512800	3	-0.35	3.16	7.00	6.99	6.99	0.60	0.62	0.60		0.62	Zheng JH et al.	2018	Lithos	302	455-466
1516	HPA06	CAOB	Eastern Tianshan	91.9333	42.6500	Granite	Huangtupo	435	435	2.06	8.50	0.1465	0.512838	4	-0.26	3.90	6.70	6.69	6.69	0.63	0.71	0.63		0.71	Zheng JH et al.	2018	Lithos	302	455-466
1517	HPA05	CAOB	Eastern Tianshan	91.9333	42.6500	Granite	Huangtupo	435	435	3.44	20.00	0.1040	0.512898	3	-0.47	5.07	10.2	10.2	10.2	0.44	0.35	0.34			Zheng JH et al.	2018	Lithos	302	455-466
1518	HPA04	CAOB	Eastern Tianshan	91.9333	42.6500	Granite	Huangtupo	435	435	0.92	4.90	0.1135	0.512893	3	-0.42	4.97	9.60	9.59	9.59	0.44	0.39	0.39			Zheng JH et al.	2018	Lithos	302	455-466
1519	HPA01	CAOB	Eastern Tianshan	91.9333	42.6500	Granite	Huangtupo	435	435	3.22	12.50	0.1557	0.512893	4	-0.21	4.97	7.30	7.25	7.25	0.58	0.68	0.58		0.68	Zheng JH et al.	2018	Lithos	302	455-466
1520	QS-23	CAOB	West Tianshan	82.4500	43.6833	Granite porphyry		300	300	2.34	16.20	0.0873	0.512561		-0.56	-1.50	2.69	2.69	2.69	0.84	0.71	0.84			Yan YH et al.	2013	Acta Petrologica et Mineralogica	32(2)	139-153
1521	QS-22	CAOB	West Tianshan	82.4500	43.6833	Granite porphyry		300	300	2.06	12.80	0.0973	0.512581		-0.51	-1.11	2.70	2.69	2.69	0.84	0.75	0.84			Yan YH et al.	2013	Acta Petrologica et Mineralogica	32(2)	139-153
1522	QS-18	CAOB	West Tianshan	82.4500	43.6833	Granite porphyry		300	300	2.18	13.80	0.0955	0.512551		-0.51	-1.70	2.18	2.18	2.18	0.88	0.77	0.88			Yan YH et al.	2013	Acta Petrologica et Mineralogica	32(2)	139-153
1523	QS-17	CAOB	West Tianshan	82.4500	43.6833	Granite porphyry		300	300	2.09	13.60	0.0929	0.512559		-0.53	-1.54	2.44	2.43	2.43	0.86	0.75	0.86			Yan YH et al.	2013	Acta Petrologica et Mineralogica	32(2)	139-153
1524	QS-16	CAOB	West Tianshan	82.4500	43.6833	Granite porphyry		300	300	2.62	14.00	0.1131	0.512582		-0.43	-1.09	2.11	2.11	2.11	0.89	0.86	0.89			Yan YH et al.	2013	Acta Petrologica et Mineralogica	32(2)	139-153
1525	HSS6-4	CAOB	Beishan	92.0000	40.9000	Rhyolite		309	309	10.00	37.10	0.1633	0.512730		-0.17	1.79	3.00	3.11	3.11	0.81	1.27	0.81			Su BX et al.	2011	Journal of Asian Earth Sciences	41（1）	31-43
1526	HSS6-3	CAOB	Beishan	92.0000	40.9000	Rhyolite		325	325	11.30	41.40	0.1657	0.512763		-0.16	2.44	3.50	3.72	3.72	0.78	1.23	0.78			Su BX et al.	2011	Journal of Asian Earth Sciences	41（1）	31-43
1527	HSS6-2	CAOB	Beishan	92.0000	40.9000	Rhyolite		328	328	11.60	43.00	0.1627	0.512793		-0.17	3.02	4.30	4.45	4.45	0.72	1.07	0.72			Su BX et al.	2011	Journal of Asian Earth Sciences	41（1）	31-43
1528	HSS6-1	CAOB	Beishan	92.0000	40.9000	Rhyolite		303	303	11.10	41.50	0.1616	0.512707		-0.18	1.35	2.60	2.70	2.70	0.84	1.29	0.84			Su BX et al.	2011	Journal of Asian Earth Sciences	41（1）	31-43
1529	HSS12	CAOB	Beishan	92.0000	40.9000	Diorite		283	283	3.03	14.10	0.1305	0.512424		-0.34	-4.17	-1.80	-1.78	-1.78	1.19	1.33	1.19			Su BX et al.	2011	Journal of Asian Earth Sciences	41（1）	31-43
1530	09HSS3-3	CAOB	Beishan	92.0000	40.9000	Dacite		390	390	9.09	58.50	0.0939	0.511764		-0.52	-17.05		-11.9	-11.9	2.10	1.76	2.10			Su BX et al.	2011	Journal of Asian Earth Sciences	41（1）	31-43
1531	09HSS3-1	CAOB	Beishan	92.0000	40.9000	Dacite		265	265	12.30	83.50	0.0893	0.511856		-0.55	-15.25		-11.6	-11.6	1.98	1.58	1.98			Su BX et al.	2011	Journal of Asian Earth Sciences	41（1）	31-43
1532	BK15	CAOB	Nalati,Tianshan	81.3333	42.6333	Granodiorite	Gaoersayi	419	479	0.83	5.06	0.0996	0.512242	10	-0.49	-7.72	-1.79	-1.79	-1.79	1.35	1.21	1.35		1.35	Long LL et al.	2007	Acta Petrologica Sinica	23(4)	719-732
1533	BK14	CAOB	Nalati,Tianshan	81.3333	42.6333	Granite	Gaoersayi	413	479	5.37	29.83	0.1090	0.512259	12	-0.45	-7.39	-2.03	-2.03	-2.03	1.37	1.30	1.37		1.37	Long LL et al.	2007	Acta Petrologica Sinica	23(4)	719-732
1534	BK13	CAOB	Nalati,Tianshan	81.3333	42.6333	Granite	Gaoersayi	401	479	7.02	39.06	0.1088	0.512180	12	-0.45	-8.93	-3.56	-3.56	-3.56	1.50	1.41	1.50		1.50	Long LL et al.	2007	Acta Petrologica Sinica	23(4)	719-732
1535	SHJ-32-3	CAOB	Beishan	90.3750	44.7500	Andesite	Batamayineishan Formation	341	341	5.12	23.80	0.1297	0.512710		-0.34	1.40	4.37	4.32	4.32	0.74	0.80	0.74			Zhang YY et al.	2015	Gondwana Research	28	1466-1481
1536	HSS-18-3	CAOB	Beishan	90.3750	44.7500	Rhyolite	Batamayineishan Formation	341	341	5.86	25.90	0.1365	0.512740		-0.31	1.99	4.76	4.61	4.61	0.72	0.81	0.72			Zhang YY et al.	2015	Gondwana Research	28	1466-1481
1537	ZMD-30	CAOB	Northern Inner Mongolia	111.2968	43.5376	Qtz monzonite		300	300	4.65	26.20	0.1073	0.512445	19	-0.45	-3.76	-0.30	-0.34	-0.34	1.09	1.01	1.09	1.01	1.09	Hu CS et al.	2017	Journal of Asian Earth Sciences	144	141-154
1538	ZMD-17	CAOB	Northern Inner Mongolia	111.2978	43.5386	Qtz monzonite		300	300	4.84	22.40	0.1306	0.512724	12	-0.34	1.68	4.20	4.21	4.21	0.72	0.78	0.72	0.78	0.72	Hu CS et al.	2017	Journal of Asian Earth Sciences	144	141-154
1539	ZMD-14	CAOB	Northern Inner Mongolia	111.3387	43.5604	Porphyritic granite		306	306	5.20	25.80	0.1218	0.512333	21	-0.38	-5.95	-3.00	-3.02	-3.02	1.31	1.35	1.31	1.35	1.31	Hu CS et al.	2017	Journal of Asian Earth Sciences	144	141-154
1540	ZMD-10	CAOB	Northern Inner Mongolia	111.3387	43.5452	Porphyritic granite		306	306	6.13	26.60	0.1393	0.512377	20	-0.29	-5.09	-2.80	-2.85	-2.85	1.30	1.58	1.30	1.58	1.30	Hu CS et al.	2017	Journal of Asian Earth Sciences	144	141-154
1541	MG-3	CAOB	Northern Inner Mongolia	111.3287	43.5352	Porphyritic granite		306	306	5.34	25.10	0.1286	0.512364	19	-0.35	-5.34	-2.70	-2.69	-2.69	1.28	1.41	1.28	1.41	1.28	Hu CS et al.	2017	Journal of Asian Earth Sciences	144	141-154
1542	WZ09	NCC	NCC	108.8381	41.6597	Qtz diorite	Kebu	291	291	3.22	15.87	0.1228	0.511726	14	-0.38	-17.79	-15.1	-15.0	-15.0	2.27	2.38	2.27	2.38	2.28	Luo HL et al.	2007	Acta petrologica sinica	23(4)	755-766
1543	Z5	NCC	NCC	110.8750	38.1250	Trachyte porphyry	Zijinshan	125	150	6.80	39.87	0.1032	0.512021		-0.48	-12.04	-10.5	-10.2	-10.2	1.77	1.55	1.77	1.56	1.78	Yang KX et al.	2009	Russian Geology and Geophysics	50(9)	751-762
1544	Z4	NCC	NCC	110.8783	38.1206	Porphyry	Zijinshan	132	150	7.59	43.80	0.1049	0.512033		-0.47	-11.80	-10.3	-10.0	-10.0	1.76	1.56	1.76	1.57	1.76	Yang KX et al.	2009	Russian Geology and Geophysics	50(9)	751-762
1545	Z1	NCC	NCC	110.8803	38.1203	Diopside syenite	Zijinshan	133	150	7.65	36.51	0.1268	0.512270		-0.36	-7.18	-5.99	-5.84	-5.84	1.41	1.54	1.41	1.54	1.41	Yang KX et al.	2009	Russian Geology and Geophysics	50(9)	751-762
1546	YMS-4	CAOB	East Tianshan	93.8333	41.8333	Acidic volcanic		751	600	2.36	10.30	0.1387	0.512780		-0.29	2.77	5.30	7.21	7.21	0.13	0.75	0.72		0.75	Rui C et al.	2018	International Geology Review	60(1)	43-56
1547	YMS-3	CAOB	East Tianshan	93.8333	41.8333	Acidic volcanic		706	600	2.87	12.90	0.1349	0.512790		-0.31	2.97	5.50	7.70	7.70	0.75	0.70	0.68		0.71	Rui C et al.	2018	International Geology Review	60(1)	43-56
1548	YMS-2	CAOB	East Tianshan	93.8333	41.8333	Acidic volcanic		756	600	5.61	25.30	0.1342	0.512760		-0.32	2.38	5.00	7.17	7.17	0.71	0.75	0.72		0.76	Rui C et al.	2018	International Geology Review	60(1)	43-56
1549	YMS-1	CAOB	East Tianshan	93.8333	41.8333	Acidic volcanic		764	600	4.54	19.90	0.1381	0.512770		-0.30	2.57	5.10	7.06	7.06	0.76	0.77	0.73		0.76	Rui C et al.	2018	International Geology Review	60(1)	43-56
1550	EBY04-4	CAOB	NE China	119.9600	49.8464	B-T andesite		123	150	7.09	49.57	0.0866	0.51265		-0.56	0.23	2.00	2.34	2.34	0.75	0.60	0.75	0.41		Zhang LC et al.	2008	Chemical Geology	256(1-2)	12-23
1551	EBY04-2	CAOB	NE China	119.9590	49.8422	B-T andesite		123	150	8.1	54.73	0.0896	0.51265		-0.54	0.23	1.90	2.28	2.28	0.75	0.61	0.75	0.62		Zhang LC et al.	2008	Chemical Geology	256(1-2)	12-23
1552	ESH04-2	CAOB	NE China	120.2010	50.4006	B-T andesite		123	150	6.41	40.47	0.0960	0.51263		-0.51	-0.16	1.50	1.77	1.77	0.79	0.67	0.79	0.67		Zhang LC et al.	2008	Chemical Geology	256(1-2)	12-23
1553	EBY04-1	CAOB	NE China	119.9590	49.8422	B-T andesite		123	150	9.78	55.05	0.1075	0.51266		-0.45	0.43	1.90	2.14	2.14	0.76	0.70	0.76	0.71		Zhang LC et al.	2008	Chemical Geology	256(1-2)	12-23
1554	YK04-1	CAOB	NE China	120.9500	49.2061	B-T andesite		123	150	13.04	64.11	0.1231	0.5127		-0.37	1.21	2.40	2.62	2.62	0.73	0.76	0.73	0.76		Zhang LC et al.	2008	Chemical Geology	256(1-2)	12-23
1555	YK04-3	CAOB	NE China	120.9500	49.2061	B-T andesite		123	150	12.56	63.49	0.1198	0.51268		-0.39	0.82	2.00	2.29	2.29	0.75	0.76	0.75	0.77		Zhang LC et al.	2008	Chemical Geology	256(1-2)	12-23
1556	YK04-4	CAOB	NE China	120.6160	49.2061	B-T andesite		123	150	12.5	63.32	0.1195	0.51267		-0.39	0.62	1.90	2.10	2.10	0.77	0.78	0.77	0.78		Zhang LC et al.	2008	Chemical Geology	256(1-2)	12-23
1557	YKS04-3	CAOB	NE China	121.5830	48.8464	B-T andesite		123	150	14.56	70.16	0.1256	0.51268		-0.36	0.82	2.00	2.18	2.18	0.76	0.81	0.76	0.82		Zhang LC et al.	2008	Chemical Geology	256(1-2)	12-23
1558	ZLT04-9	CAOB	NE China	122.8060	48.0050	B-T andesite		123	150	4.82	22.47	0.1298	0.5127		-0.34	1.21	2.30	2.49	2.49	0.74	0.82	0.74	0.82		Zhang LC et al.	2008	Chemical Geology	256(1-2)	12-23
1559	ER04-7	CAOB	NE China	119.9590	49.8422	B-T andesite		123	150	6.39	30.08	0.1285	0.51268		-0.35	0.82	1.90	2.12	2.12	0.77	0.84	0.77	0.84		Zhang LC et al.	2008	Chemical Geology	256(1-2)	12-23
1560	ZHJ04-1	CAOB	NE China	121.1310	44.7533	Basaltic andesite		123	150	4.76	23.06	0.1250	0.51263		-0.36	-0.16	1.00	1.22	1.22	0.84	0.89	0.84	0.89		Zhang LC et al.	2008	Chemical Geology	256(1-2)	12-23
1561	XW04-1	CAOB	NE China	117.6520	44.3150	B-T andesite		123	150	9.56	49.78	0.1162	0.51251		-0.41	-2.50	-1.20	-0.96	-0.96	1.02	1.00	1.02	1.00		Zhang LC et al.	2008	Chemical Geology	256(1-2)	12-23
1562	MAN-1	CAOB	NE China	120.8010	44.4489	Basaltic andesite		123	150	4.98	23.71	0.1271	0.51254		-0.35	-1.91	-0.80	-0.58	-0.58	0.99	1.07	0.99	1.07		Zhang LC et al.	2008	Chemical Geology	256(1-2)	12-23
1563	MAN-2	CAOB	NE China	120.8000	44.4489	Basaltic andesite		123	150	4.9	23.3	0.1272	0.51253		-0.35	-2.11	-1.00	-0.78	-0.78	1.00	1.09	1.00	1.09		Zhang LC et al.	2008	Chemical Geology	256(1-2)	12-23
1564	MAN-3	CAOB	NE China	120.7970	44.4506	Basaltic andesite		123	150	4.83	22.9	0.1274	0.51253		-0.35	-2.11	-1.00	-0.78	-0.78	1.00	1.09	1.00	1.09		Zhang LC et al.	2008	Chemical Geology	256(1-2)	12-23
1565	05TH19	CAOB	NE China	124.5590	52.5717	B-T andesite		123	150	5.56	30.85	0.1091	0.51238		-0.45	-5.03	-3.60	-3.36	-3.36	1.21	1.12	1.21	1.12		Zhang LC et al.	2008	Chemical Geology	256(1-2)	12-23
1566	LD-06	CAOB	NE China	119.4460	43.9292	Basaltic andesite		123	150	3.17	11.79	0.1654	0.51264		-0.16	0.04	0.50	0.64	0.64	0.88	1.61	0.88	0.88		Zhang LC et al.	2008	Chemical Geology	256(1-2)	12-23
1567	LD-05	CAOB	NE China	119.4460	43.9292	Basaltic andesite		123	150	2.99	10.91	0.1687	0.51261		-0.14	-0.55	-0.10	-0.01	-0.01	0.94	1.82	0.94	1.00		Zhang LC et al.	2008	Chemical Geology	256(1-2)	12-23
1568	LD-10	CAOB	NE China	119.4360	43.9294	Basaltic andesite		123	150	3.22	11.65	0.1698	0.51262		-0.14	-0.35	0.10	0.16	0.16	0.92	1.83	0.92	0.91		Zhang LC et al.	2008	Chemical Geology	256(1-2)	12-23
1569	05TH26	CAOB	NE China	124.3610	52.6606	B-T andesite		123	150	7.81	26.45	0.1788	0.51241		-0.09	-4.45	-4.20	-4.10	-4.10	1.26	3.21	1.26	1.26		Zhang LC et al.	2008	Chemical Geology	256(1-2)	12-23
1570	M9	CAOB	West Junggar	84.9336	46.0503	Alkali feldspar granite	Miaoergou	305	305	8.58	43.77	0.1186	0.512823	8	-0.40	3.61	6.65	6.65	6.65	0.52	0.52	0.52	0.52	0.52	Su YP et al.	2006	Geochimica	35(1)	55-67

1571	M1	CAOB	West Junggar	83.9167	45.0333	Alkali feldspar granite	Miaoergou	305	305	6.52	29.14	0.1352	0.512846	5	-0.31	4.06	6.45	6.45	6.45	0.54	0.59	0.54	0.59	0.54	Su YP et al.	2006	Geochimica	35(1)	55-67
1572	K9	CAOB	West Junggar	84.6667	45.4167	Alkali feldspar granite	Karamay	296	296	6.21	34.93	0.1075	0.512845	7	-0.45	4.04	7.41	7.41	7.41	0.45	0.44	0.45	0.44	0.45	Su YP et al.	2006	Geochimica	35(1)	55-67
1573	K10	CAOB	West Junggar	84.6667	45.4167	Alkali feldspar granite	Karamay	296	296	9.89	46.59	0.1284	0.512870	7	-0.35	4.53	7.11	7.11	7.11	0.48	0.50	0.48	0.50	0.48	Su YP et al.	2006	Geochimica	35(1)	55-67
1574	H7	CAOB	West Junggar	85.1333	46.0000	Alkali feldspar granite	Hongshan	301	301	6.16	30.41	0.1224	0.512851	3	-0.38	4.15	7.01	7.01	7.01	0.49	0.50	0.49	0.50	0.49	Su YP et al.	2006	Geochimica	35(1)	55-67
1575	H3	CAOB	West Junggar	85.1333	46.0000	Alkali feldspar granite	Hongshan	301	301	5.37	24.06	0.1350	0.512879	5	-0.31	4.70	7.07	7.07	7.07	0.49	0.53	0.49	0.53	0.49	Su YP et al.	2006	Geochimica	35(1)	55-67
1576	AS3	CAOB	West Junggar	84.2500	45.5000	Alkali feldspar granite	Akbastau	303	303	10.43	44.82	0.1406	0.512879	6	-0.29	4.70	6.87	6.87	6.87	0.50	0.57	0.50	0.57	0.50	Su YP et al.	2006	Geochimica	35(1)	55-67
1577	AS1	CAOB	West Junggar	84.2500	45.5000	Alkali feldspar granite	Akbastau	303	303	12.84	71.54	0.1085	0.512798	6	-0.45	3.12	6.53	6.53	6.53	0.53	0.51	0.53	0.51	0.53	Su YP et al.	2006	Geochimica	35(1)	55-67
1578	V747-8	CAOB	South Urals	58.0000	51.0000	Trachyte		395	395	4.86	19.43	0.1504	0.512597		-0.24	-0.80	3.50	1.53	1.53	1.01	1.33	1.01			Fedorov PI et al.	2017	Geochemistry International	55（4）	341-354
1579	V585-8	CAOB	South Urals	58.0000	51.0000	Syenite		395	395	3.45	17.70	0.1174	0.512544		-0.40	-1.83	4.50	2.16	2.16	0.96	0.96	0.96			Fedorov PI et al.	2017	Geochemistry International	55（4）	341-354
1580	98LP-27	NCC	NNCC	117.2000	41.2000	Andesite	Tiaojishan Fm	157	157	7.64	39.76	0.1149	0.511797		-0.42	-16.41	-14.9	-14.8	-14.8	2.14	2.08	2.14			Guo F et al.	2005	Geological Society, London, Special Publication	280（1）	101-129
1581	20XSH-88	NCC	NNCC	115.5000	39.7500	Andesite	Tiaojishan Fm	157	157	5.54	31.18	0.1065	0.511856		-0.46	-15.25	-13.5	-13.4	-13.4	2.04	1.83	2.04			Guo F et al.	2005	Geological Society, London, Special Publication	280（1）	101-129
1582	20XSH-85	NCC	NNCC	115.5000	39.7500	Andesite	Tiaojishan Fm	157	157	6.59	37.22	0.1074	0.511830		-0.45	-15.76	-14.1	-14.0	-14.0	2.08	1.89	2.08			Guo F et al.	2005	Geological Society, London, Special Publication	280（1）	101-129
1583	20XSH-77	NCC	NNCC	115.5000	39.7500	Andesite	Tiaojishan Fm	157	157	6.91	38.96	0.1070	0.511806		-0.46	-16.23	-14.5	-14.4	-14.4	2.12	1.91	2.12			Guo F et al.	2005	Geological Society, London, Special Publication	280（1）	101-129
1584	XL04-11	CAOB	NE China	116.1398	44.3018	Felsic volcanic rocks		280	280	6.60	26.71	0.1495	0.512656		-0.24	0.35	2.03	2.04	2.04	0.88	1.17	0.88	1.17	0.88	Zhang XH et al.	2008	Chemical Geology	249（3-4）	262-281
1585	XL04-17	CAOB	NE China	116.1398	44.3018	Felsic volcanic rocks		280	280	5.71	21.98	0.1572	0.512661		-0.20	0.45	1.85	1.86	1.86	0.89	1.32	0.89	1.32	0.88	Zhang XH et al.	2008	Chemical Geology	249（3-4）	262-281
1586	XL04-19	CAOB	NE China	116.1398	44.3018	Felsic volcanic rocks		280	280	7.89	31.79	0.1502	0.512652		-0.24	0.27	1.93	1.93	1.93	0.89	1.19	0.89	1.19	0.89	Zhang XH et al.	2008	Chemical Geology	249（3-4）	262-281
1587	XW04-2	CAOB	NE China	116.5684	44.1550	Rhyolite		280	280	4.33	15.57	0.1685	0.512737		-0.14	1.93	2.93	2.94	2.94	0.80	1.39	0.80	1.39	0.80	Zhang XH et al.	2008	Chemical Geology	249（3-4）	262-281
1588	XW04-4	CAOB	NE China	117.5741	44.5481	Rhyolite		280	280	6.65	26.97	0.1492	0.512697		-0.24	1.15	2.84	2.85	2.85	0.81	1.07	0.81	1.07	0.81	Zhang XH et al.	2008	Chemical Geology	249（3-4）	262-281
1589	XW04-28	CAOB	NE China	117.6756	44.4085	Rhyolite		280	280	12.36	48.64	0.1538	0.512718		-0.22	1.56	3.09	3.09	3.09	0.79	1.10	0.79	1.10	0.79	Zhang XH et al.	2008	Chemical Geology	249（3-4）	262-281
1590	XW04-34	CAOB	NE China	117.6756	44.4085	Rhyolite		280	280	10.99	44.90	0.1482	0.512721		-0.25	1.62	3.35	3.35	3.35	0.77	1.00	0.77	1.00	0.77	Zhang XH et al.	2008	Chemical Geology	249（3-4）	262-281
1591	XW04-36	CAOB	NE China	117.6756	44.4085	Rhyolite		280	280	10.39	43.07	0.1461	0.512730		-0.26	1.79	3.60	3.60	3.60	0.75	0.95	0.75	0.95	0.75	Zhang XH et al.	2008	Chemical Geology	249（3-4）	262-281
1592	XW04-39	CAOB	NE China	117.6756	44.4085	Rhyolite		280	280	9.27	36.16	0.1551	0.512729		-0.21	1.78	3.26	3.26	3.26	0.78	1.09	0.78	1.10	0.78	Zhang XH et al.	2008	Chemical Geology	249（3-4）	262-281
1593	XL04-16	CAOB	NE China	116.1400	44.3018	Felsic volcanic rocks	Dashizhai	280	280	10.25	42.47	0.1460	0.51265		-0.26	0.23	2.04	2.05	2.06	0.88	1.13	0.88	1.13	0.88	Zhang XH et al.	2008	Chemical Geology	249(3-4)	262-281
1594	XL04-27	CAOB	NE China	116.5680	44.1550	Basaltic andesite	Dashizhai	280	280	3.584	12.29	0.1765	0.512959		-0.10	6.26	6.98	6.98	7.00	0.48	0.78	0.48	0.78	0.47	Zhang XH et al.	2008	Chemical Geology	249(3-4)	262-281
1595	XL04-28	CAOB	NE China	116.5680	44.1550	Basaltic andesite	Dashizhai	280	280	3.93	15.28	0.1557	0.512937		-0.21	5.83	7.29	7.30	7.31	0.45	0.56	0.45	0.56	0.45	Zhang XH et al.	2008	Chemical Geology	249(3-4)	262-281
1596	XL04-30	CAOB	NE China	116.5680	44.1550	Basaltic andesite	Dashizhai	280	280	4.087	13.29	0.1862	0.512971		-0.05	6.50	6.87	6.87	6.89	0.48	0.99	0.48	0.99	0.48	Zhang XH et al.	2008	Chemical Geology	249(3-4)	262-281
1597	XL04-32	CAOB	NE China	116.5680	44.1550	Basaltic andesite	Dashizhai	280	280	3.412	10.22	0.2021	0.513053		0.03	8.10	7.90	7.90	7.92	0.40	1.27	0.40	1.27	0.40	Zhang XH et al.	2008	Chemical Geology	249(3-4)	262-281
1598	XL04-34	CAOB	NE China	116.5680	44.1550	Basaltic andesite	Dashizhai	280	280	7.409	26.38	0.1700	0.512908		-0.14	5.27	6.22	6.22	6.24	0.54	0.84	0.54	0.84	0.54	Zhang XH et al.	2008	Chemical Geology	249(3-4)	262-281
1599	XJ22-12	CAOB	Northern Junggar	90.0000	46.0000	Basalt andesite	Laoshankou section	385	385	1.514	5.072	0.1804	0.512943		-0.08	5.95	6.75	6.75	6.76	0.58	0.95	0.58			Zhang ZC et al.	2008	Lithos	105(3-4)	379-395
1600	XJ22-10	CAOB	Northern Junggar	90.0000	46.0000	Basalt andesite	Laoshankou section	385	385	1.439	4.594	0.1894	0.512995		-0.04	6.96	7.32	7.32	7.34	0.53	0.97	0.53			Zhang ZC et al.	2008	Lithos	105(3-4)	379-395
1601	T7Cb	CAOB	Tianshan Alatag	95.1667	42.0833	Tianhe bearing granite	Baishitou	209	209	10.05	16.31	0.3727	0.512629	24	0.89	-0.18	-4.87	-4.87	-4.87	1.39	-0.50	1.39		1.39	Gu LX et al.	2007	Geological Journal of China Universities	(02)	207-223
1602	T2Ca	CAOB	Tianshan Alatag	95.1667	42.0833	Leucogranite	Baishitou	209	209	8.68	12.16	0.4512	0.512764	22	1.29	2.46	-4.33	-4.33	-4.33	1.35	-0.25	1.35		1.35	Gu LX et al.	2007	Geological Journal of China Universities	(02)	207-223
1603	YX-261	CAOB	NE China	122.0500	42.5000	High-Mg andesites	Zhangwu	126	150	6.3	35.7	0.1082	0.512094		-0.45	-10.61	-9.15	-8.92	-9.16	1.66	1.52	1.66			Huang H et al.	2007	Science In China Series D-Earth Sciences	50(12)	1773-1788
1604	YX-262	CAOB	NE China	122.0500	42.5000	High-Mg andesites	Zhangwu	126	150	8.9	50.6	0.1045	0.512234		-0.47	-7.88	-6.36	-6.12	-6.42	1.44	1.28	1.44			Huang H et al.	2007	Science In China Series D-Earth Sciences	50(12)	1773-1786
1605	YX-263	CAOB	NE China	122.0500	42.5000	High-Mg andesites	Zhangwu	126	150	8.6	48.6	0.1069	0.512234		-0.46	-7.88	-6.40	-6.16	-6.44	1.44	1.31	1.44			Huang H et al.	2007	Science In China Series D-Earth Sciences	50(12)	1773-1787
1606	YX-264	CAOB	NE China	122.0500	42.5000	High-Mg andesites	Zhangwu	126	150	5.9	32.5	0.1107	0.511938		-0.44	-13.65	-12.2	-12.0	-12.3	1.91	1.79	1.91			Huang H et al.	2007	Science In China Series D-Earth Sciences	50(12)	1773-1789
1607	SQZ0905	CAOB	EasternTianshan	94.7768	41.9167	Rhyolitic dacite		320	320	3.85	17.20	0.1352	0.512590	0	-0.31	-0.94	1.57	1.57	1.57	0.95	1.09	0.95	1.09	0.95	Huang XW et al.	2012	Acta Petrologica et Mineralogica	31(06	799-817
1608	SQZ0904	CAOB	EasternTianshan	94.7768	41.9167	Rhyolitic dacite		320	320	4.83	23.40	0.1248	0.512481	0	-0.37	-3.06	-0.14	-0.13	-0.13	1.09	1.15	1.09	1.15	1.09	Huang XW et al.	2012	Acta Petrologica et Mineralogica	31(06	799-817
1609	07NZG-5（2）	NCC	NMNCC	118.6500	42.4250	Granite	Nianzigou	167	167			0.0309	0.512233	12	-0.84	-7.90	-4.37	-4.37	-4.37	1.31	0.77	1.31	0.77	1.31	Chen ZG et al.	2008	Acta Petrologica Sinica	24(4)	879-889
1610	07NZG-5（1）	NCC	NMNCC	118.6500	42.4250	Granite	Nianzigou	167	167			0.0338	0.512264	9	-0.83	-7.30	-3.83	-3.82	-3.82	1.27	0.75	1.27	0.75	1.27	Chen ZG et al.	2008	Acta Petrologica Sinica	24(4)	879-889
1611	07NZG-4	NCC	NMNCC	118.6500	42.4250	Granite	Nianzigou	167	167			0.0306	0.512105	10	-0.84	-10.40	-6.85	-6.86	-6.86	1.51	0.87	1.51	0.87	1.51	Chen ZG et al.	2008	Acta Petrologica Sinica	24(4)	879-889
1612	07NZG-3	NCC	NMNCC	118.6500	42.4250	Granite	Nianzigou	167	167			0.0309	0.512079	11	-0.84	-10.90	-7.38	-7.37	-7.37	1.56	0.89	1.56	0.90	1.55	Chen ZG et al.	2008	Acta Petrologica Sinica	24(4)	879-889
1613	07NZG-1（1）	NCC	NMNCC	118.6500	42.4250	Granite	Nianzigou	167	167			0.0303	0.512100	11	-0.85	-10.49	-6.95	-6.95	-6.95	1.52	0.87	1.52	0.87	1.52	Chen ZG et al.	2008	Acta Petrologica Sinica	24(4)	879-889
1614	NM12-212	NCC	NNCC	113.8528	42.0206	Andesite	Elitu	276	276	6.63	37.20	0.1131	0.511934		-0.43	-13.73	-13.70	-10.79	-10.79	1.92	1.84	1.92	1.84	1.91	Ji ZJ et al.	2006	International Journal of Earth Sciences	151（1）	1-19
1615	NM12-211	NCC	NNCC	113.8528	42.0206	Andesite	Elitu	276	276	4.93	27.10	0.1154	0.512004		-0.41	-12.37	-12.40	-9.51	-9.51	1.81	1.77	1.81	1.77	1.81	Ji ZJ et al.	2006	International Journal of Earth Sciences	151（1）	1-19
1616	NM12-210	NCC	NNCC	113.8714	42.0408	Andesite	Elitu	276	276	4.00	19.10	0.1329	0.512324		-0.32	-6.13	-6.10	-3.88	-3.88	1.36	1.56	1.36	1.56	1.37	Ji ZJ et al.	2006	International Journal of Earth Sciences	151（1）	1-19
1617	NM12-209	NCC	NNCC	113.8714	42.0408	Andesite	Elitu	276	276	6.54	36.30	0.1143	0.511718		-0.42	-17.95	-17.90	-15.05	-15.05	2.26	2.19	2.26	2.19	2.26	Ji ZJ et al.	2006	International Journal of Earth Sciences	151（1）	1-19
1618	NM12-015	NCC	NNCC	113.0186	42.1047	Rhyolite	Elitu	276	276	8.77	44.60	0.1248	0.512257		-0.37	-7.43	-7.40	-4.90	-4.90	1.44	1.53	1.44	1.53	1.45	Ji ZJ et al.	2006	International Journal of Earth Sciences	151（1）	1-19
1619	NM12-011	NCC	NNCC	113.0219	42.1053	Rhyolite	Elitu	276	276	4.48	21.20	0.1341	0.512085		-0.32	-10.79	-10.80	-8.59	-8.59	1.74	2.03	1.74	2.03	1.75	Ji ZJ et al.	2006	International Journal of Earth Sciences	151（1）	1-19
1620	NM12-008	NCC	NNCC	113.0211	42.1064	Rhyolite	Elitu	276	276	6.71	34.30	0.1218	0.512257		-0.38	-7.43	-7.40	-4.80	-4.80	1.43	1.48	1.43	1.48	1.44	Ji ZJ et al.	2006	International Journal of Earth Sciences	151（1）	1-19
1621	NM12-005	NCC	NNCC	113.0222	42.1053	Dacite	Elitu	276	276	5.86	35.00	0.1064	0.511986		-0.46	-12.72	-12.70	-9.54	-9.54	1.82	1.65	1.82	1.65	1.81	Ji ZJ et al.	2006	International Journal of Earth Sciences	151（1）	1-19
1622	NM12-003	NCC	NNCC	113.0222	42.1053	Dacite	Elitu	276	276	5.93	35.80	0.1052	0.512010		-0.47	-12.25	-12.20	-9.03	-9.03	1.78	1.60	1.78	1.60	1.78	Ji ZJ et al.	2006	International Journal of Earth Sciences	151（1）	1-19
1623	TW-43	CAOB	NE China	125.0000	51.0000	Rhyolite		123	150	2.59	15.20	0.1028	0.512524		-0.48	-2.22	-0.80	-0.43	-0.43	0.97	0.86	0.97	0.98		Hu XL et al.	2015	Acta Geologica Sinica-English Edition	89（1）	203-214
1624	TW-41-2	CAOB	NE China	125.0000	51.0000	Rhyolite		123	150	2.84	17.30	0.0994	0.512500		-0.49	-2.69	-1.20	-0.83	-0.83	1.01	0.87	1.01	1.02		Hu XL et al.	2015	Acta Geologica Sinica-English Edition	89（1）	203-214
1625	TW-41-1	CAOB	NE China	125.0000	51.0000	Rhyolite		123	150	2.91	17.70	0.0996	0.512502		-0.49	-2.65	-1.20	-0.79	-0.79	1.00	0.87	1.00	1.01		Hu XL et al.	2015	Acta Geologica Sinica-English Edition	89（1）	203-214
1626	TW-39-2	CAOB	NE China	125.0000	51.0000	Rhyolite		123	150	3.31	19.90	0.1005	0.512495		-0.49	-2.79	-1.40	-0.95	-0.95	1.02	0.88	1.02	1.02		Hu XL et al.	2015	Acta Geologica Sinica-English Edition	89（1）	203-214
1627	TW-39-1	CAOB	NE China	125.0000	51.0000	Rhyolite		123	150	3.06	19.40	0.0954	0.512506		-0.51	-2.57	-1.10	-0.64	-0.64	0.99	0.83	0.99	1.00		Hu XL et al.	2015	Acta Geologica Sinica-English Edition	89（1）	203-214
1628	DB-03-2	CAOB	NE China	125.0000	51.0000	Rhyolite		123	150	5.56	33.40	0.1030	0.512532		-0.48	-2.07	-0.50	-0.27	-0.27	0.96	0.85	0.96	0.97		Hu XL et al.	2015	Acta Geologica Sinica-English Edition	89（1）	203-214
1629	DB-03-1	CAOB	NE China	125.0000	51.0000	Rhyolite		123	150	5.58	33.30	0.1038	0.512552		-0.47	-1.68	-0.10	0.10	0.10	0.93	0.83	0.93	0.94		Hu XL et al.	2015	Acta Geologica Sinica-English Edition	89（1）	203-214
1630	ML-37	CAOB	CMEB	121.8474	53.3729	Qtz-monzonite porphyry	Luogu River	130	150	7.04	43.18	0.0987	0.512419	5	-0.50	-4.27	-2.65	-2.40	-2.40	1.13	0.97	1.13	0.97	1.14	Wu G et al., 2010	2009	Acta Petrologica Sinica	25(2)	233-247
1631	ML-34	CAOB	CMEB	121.8474	53.3729	Qtz-monzonite porphyry	Luogu River	130	150	6.65	39.65	0.1014	0.512422	10	-0.48	-4.21	-2.63	-2.39	-2.39	1.13	0.99	1.13	0.99	1.14	Wu G et al., 2010	2009	Acta Petrologica Sinica	25(2)	233-247
1632	ML-3	CAOB	CMEB	121.8474	53.3729	Granite-porphyry	Luogu River	130	150	6.34	37.52	0.1022	0.512400	7	-0.48	-4.64	-3.08	-2.83	-2.83	1.17	1.03	1.17	1.03	1.18	Wu G et al., 2010	2009	Acta Petrologica Sinica	25(2)	233-247
1633	ML-16	CAOB	CMEB	121.8474	53.3729	Granite-porphyry	Luogu River	130	150	6.42	41.89	0.0927	0.512373	6	-0.53	-5.17	-3.45	-3.18	-3.18	1.20	0.98	1.20	0.98	1.21	Wu G et al., 2010	2009	Acta Petrologica Sinica	25(2)	233-247
1634	ML-1	CAOB	CMEB	121.8474	53.3729	Granite-porphyry	Luogu River	130	150	5.25	28.21	0.1127	0.512400	10	-0.43	-4.64	-3.25	-3.03	-3.03	1.19	1.13	1.19	1.13	1.19	Wu G et al., 2010	2009	Acta Petrologica Sinica	25(2)	233-247
1635	XM3-136	CAOB	Western Junggar	85.6083	46.6583	Alkali feldspar granite		422	422	7.39	32.80	0.1364	0.512400		-0.31	-4.64	4.98	-1.40	-1.40	1.27	1.48	1.27	0.84	0.71	Wang M et al.	2018	Acta Petrologica Sinica	34(3)	618-636
1636	XM3-135	CAOB	Western Junggar	85.6083	46.6583	Granodiorite		411	411	2.74	14.40	0.1153	0.512300		-0.41	-6.59	3.87	-2.32	-2.32	1.34	1.32	1.34	0.83	0.77	Wang M et al.	2018	Acta Petrologica Sinica	34(3)	618-636
1637	XM3-134	CAOB	Western Junggar	85.6083	46.6583	Alkali feldspar granite		422	422	5.75	24.20	0.1437	0.512400		-0.27	-4.64	6.58	-1.79	-1.79	1.31	1.63	1.31	0.70	0.60	Wang M et al.	2018	Acta Petrologica Sinica	34(3)	618-636
1638	XM3-133	CAOB	Western Junggar	85.6083	46.6583	Granodiorite		411	411	3.04	15.70	0.1171	0.512300		-0.40	-6.59	3.86	-2.42	-2.42	1.35	1.34	1.35	0.83	0.77	Wang M et al.	2018	Acta Petrologica Sinica	34(3)	618-636
1639	XM3-131	CAOB	Western Junggar	85.6083	46.6583	Alkali feldspar granite		422	422	5.50	21.60	0.1543	0.512300		-0.22	-6.59	4.49	-4.31	-4.31	1.51	2.17	1.51	1.02	0.74	Wang M et al.	2018	Acta Petrologica Sinica	34(3)	618-636
1640	XM2-49	CAOB	Western Junggar	85.6083	46.6583	Granodiorite		411	411	2.72	13.90	0.1181	0.512200		-0.40	-8.54	1.66	-4.42	-4.42	1.51	1.51	1.51	1.02	0.93	Wang M et al.	2018	Acta Petrologica Sinica	34(3)	618-636
1641	XM2-45	CAOB	Western Junggar	85.6083	46.6583	Granodiorite		411	411	3.17	16.30	0.1179	0.512300		-0.40	-6.59	2.76	-2.46	-2.46	1.35	1.35	1.35	0.93	0.85	Wang M et al.	2018	Acta Petrologica Sinica	34(3)	618-636
1642	14ALT-37	CAOB	Western Junggar	86.6083	47.6583	Granite		403	403	5.30	27.70	0.1157	0.512761	0	-0.41	2.40	6.60	6.56	6.56	0.61	0.61	0.61		0.61	Wang M et al.	2018	Acta Petrologica Sinica	34(3)	618-636
1643	WTX04	CAOB	Northwest Junggar	85.5000	47.0083	Monzogranite	Sawur	338	338	3.00	15.80	0.1150	0.512784	12	-0.42	2.85	6.37	6.37	6.37	0.57	0.57	0.57	0.57	0.57	Chen JF et al.	2010	Acta Petrologica Sinica	26(8)	2317-2335
1644	WTX01	CAOB	Northwest Junggar	85.5000	47.0083	Monzogranite	Sawur	338	338	3.10	16.50	0.1131	0.512785	11	-0.43	2.87	6.47	6.47	6.47	0.56	0.55	0.56	0.55	0.56	Chen JF et al.	2010	Acta Petrologica Sinica	26(8)	2317-2335
1645	WLST05	CAOB	Northwest Junggar	83.0167	47.1167	Granodiorite	Zhuqingshan	325	325	4.70	24.40	0.1167	0.512780	10	-0.41	2.77	6.09	6.09	6.09	0.59	0.58	0.59	0.58	0.59	Chen JF et al.	2010	Acta Petrologica Sinica	26(8)	2317-2335
1646	WLST04	CAOB	Northwest Junggar	83.0167	47.1167	Granodiorite	Zhuqingshan	325	325	4.70	24.40	0.1169	0.512799	11	-0.41	3.14	6.45	6.45	6.45	0.56	0.55	0.56	0.55	0.56	Chen JF et al.	2010	Acta Petrologica Sinica	26(8)	2317-2335
1647	TLG03	CAOB	Northwest Junggar	85.2250	46.7500	Granite	Tologie	281	281	8.40	39.40	0.1287	0.512829	13	-0.35	3.73	6.16	6.16	6.16	0.54	0.58	0.54	0.58	0.54	Chen JF et al.	2010	Acta Petrologica Sinica	26(8)	2317-2335
1648	TLG02	CAOB	Northwest Junggar	85.2250	46.7500	Granite	Tologie	281	281	8.10	38.50	0.1271	0.512780	12	-0.35	2.77	5.27	5.27	5.27	0.62	0.65	0.62	0.65	0.62	Chen JF et al.	2010	Acta Petrologica Sinica	26(8)	2317-2335
1649	NET04	CAOB	Northwest Junggar	83.1083	47.1083	Granodiorite	Buergan	332	332	4.20	20.00	0.1274	0.512827	12	-0.35	3.69	6.62	6.62	6.62	0.55	0.57	0.55	0.57	0.55	Chen JF et al.	2010	Acta Petrologica Sinica	26(8)	2317-2335
1650	NET02	CAOB	Northwest Junggar	83.1083	47.1083	Granodiorite	Buergan	332	332	4.30	21.50	0.1222	0.512820	12	-0.38	3.55	6.71	6.71	6.71	0.54	0.55	0.54	0.55	0.54	Chen JF et al.	2010	Acta Petrologica Sinica	26(8)	2317-2335
1651	LST02	CAOB	Northwest Junggar	85.6750	47.3167	Potash feldspar granite	Raster	321	321	4.50	23.10	0.1181	0.512804	11	-0.40	3.24	6.46	6.46	6.46	0.55	0.55	0.55	0.55	0.55	Chen JF et al.	2010	Acta Petrologica Sinica	26(8)	2317-2335
1652	LST01	CAOB	Northwest Junggar	85.6750	47.3167	Potash feldspar granite	Raster	321	321	3.70	19.20	0.1171	0.512805	10	-0.40	3.26	6.52	6.52	6.52	0.55	0.55	0.55	0.55	0.55	Chen JF et al.	2010	Acta Petrologica Sinica	26(8)	2317-2335
1653	LMZ03	CAOB	Northwest Junggar	84.0833	47.3167	Potash feldspar granite	Kurumusu	302	302	9.40	38.50	0.1473	0.512900	10	-0.25	5.11	7.01	7.01	7.01	0.49	0.57	0.49	0.57	0.49	Chen JF et al.	2010	Acta Petrologica Sinica	26(8)	2317-2335
1654	LMZ02	CAOB	Northwest Junggar	84.0833	47.3167	Potash feldspar granite	Kurumusu	302	302	9.00	38.60	0.1408	0.512888	10	-0.28	4.88	7.03	7.03	7.03	0.49	0.55	0.49	0.55	0.49	Chen JF et al.	2010	Acta Petrologica Sinica	26(8)	2317-2335
1655	KSS04	CAOB	Northwest Junggar	84.5833	46.4167	Potash feldspar granite	Selleck	304	304	7.60	35.10	0.1312	0.512880	14	-0.33	4.72	7.26	7.26	7.26	0.47	0.50	0.47	0.50	0.47	Chen JF et al.	2010	Acta Petrologica Sinica	26(8)	2317-2335
1656	KSS01	CAOB	Northwest Junggar	84.5833	46.4167	Potash feldspar granite	Selleck	304	304	6.80	30.40	0.1357	0.512856	12	-0.31	4.25	6.62	6.62	6.62	0.53	0.58	0.53	0.58	0.53	Chen JF et al.	2010	Acta Petrologica Sinica	26(8)	2317-2335
1657	DYS05	CAOB	Northwest Junggar	84.3333	46.8333	Augite diorite	Dansu	333	333	3.90	16.20	0.1432	0.512890	12	-0.27	4.92	7.19	7.19	7.19	0.50	0.56	0.50	0.56	0.50	Chen JF et al.	2010	Acta Petrologica Sinica	26(8)	2317-2335
1658	DYS03	CAOB	Northwest Junggar	84.3333	46.8333	Augite diorite	Dansu	333	333	3.60	15.50	0.1413	0.512889	11	-0.28	4.90	7.25	7.25	7.25	0.50	0.55	0.50	0.55	0.50	Chen JF et al.	2010	Acta Petrologica Sinica	26(8)	2317-2335
1659	BYMZ03	CAOB	Northwest Junggar	84.5000	46.8333	Monzogranite	Huojill	325	325	6.60	31.10	0.1276	0.512845	11	-0.35	4.04	6.90	6.90	6.90	0.52	0.54	0.52	0.54	0.52	Chen JF et al.	2010	Acta Petrologica Sinica	26(8)	2317-2335
1660	BYMZ02	CAOB	Northwest Junggar	84.5000	46.8333	Monzogranite	Huojill	325	325	6.60	30.70	0.1290	0.512838	11	-0.34	3.90	6.71	6.71	6.71	0.53	0.56	0.53	0.56	0.53	Chen JF et al.	2010	Acta Petrologica Sinica	26(8)	2317-2335
1661	ABDL03	CAOB	Northwest Junggar	83.2833	46.9333	Monzogranite	Abdullah	332	332	2.90	16.40	0.1062	0.512780	11	-0.46	2.77	6.60	6.60	6.60	0.55	0.53	0.55	0.53	0.55	Chen JF et al.	2010	Acta Petrologica Sinica	26(8)	2317-2335
1662	ABDL02	CAOB	Northwest Junggar	83.2833	46.9333	Monzogranite	Abdullah	332	332	3.60	20.40	0.1056	0.512762	12	-0.46	2.42	6.28	6.28	6.28	0.58	0.55	0.58	0.55	0.58	Chen JF et al.	2010	Acta Petrologica Sinica	26(8)	2317-2335
1663	05MZL01	CAOB	North Great Xing'an Range	117.4300	49.6000	Felsic volcanic rocks	Manzhouli	138	150	11	67.8	0.0961	0.512601		-0.51	-0.72	1.30	1.20	1.02	0.84	0.71	0.84			Ying JF et al.	2010	International Journal of Earth Sciences	99(2)	357-378
1664	05MZL02	CAOB	North Great Xing'an Range	117.4300	49.6000	Felsic volcanic rocks	Manzhouli	138	150	10.7	67	0.0953	0.512588		-0.52	-0.98	1.10	0.97	0.79	0.86	0.72	0.86			Ying JF et al.	2010	International Journal of Earth Sciences	99(2)	357-378
1665	05MZL06	CAOB	North Great Xing'an Range	117.4300	49.6000	Felsic volcanic rocks	Manzhouli	138	150	6.13	36.1	0.1015	0.512565		-0.48	-1.42	0.50	0.40	0.24	0.91	0.80	0.91			Ying JF et al.	2010	International Journal of Earth Sciences	99(2)	357-378
1666	05MZL20	CAOB	North Great Xing'an Range	117.4300	49.6000	Felsic volcanic rocks	Manzhouli	138	150	9.81	54.3	0.1021	0.512556		-0.48	-1.60	0.20	0.21	-0.06	0.92	0.81	0.92			Ying JF et al.	2010	International Journal of Earth Sciences	99(2)	357-378
1667	05TH03	CAOB	North Great Xing'an Range	124.7300	52.3300	Felsic volcanic rocks	Tahe	138	150	6.76	37.8	0.1050	0.512505		-0.47	-2.59	-1.10	-0.84	-1.03	1.01	0.90	1.01			Ying JF et al.	2010	International Journal of Earth Sciences	99(2)	357-378
1668	05TH05	CAOB	North Great Xing'an Range	124.7300	52.3300	Felsic volcanic rocks	Tahe	138	150	5.19	26.2	0.1184	0.512503		-0.40	-2.63	-1.30	-1.13	-1.27	1.03	1.03	1.03			Ying JF et al.	2010	International Journal of Earth Sciences	99(2)	357-378

1669	05TH12	CAOB	North Great Xing'an Range	124.7300	52.3300	Felsic volcanic rocks	Tahe	138	150	7.81	42.2	0.1074	0.512567		-0.45	-1.38	0.10	0.32	0.11	0.91	0.84	0.91			Ying JF et al.	2010	International Journal of Earth Sciences	99(2)	357-378
1670	05TH14	CAOB	North Great Xing'an Range	124.7300	52.3300	Felsic volcanic rocks	Tahe	138	150	7.75	43	0.1081	0.512494		-0.45	-2.81	-1.30	-1.11	-1.26	1.03	0.95	1.03			Ying JF et al.	2010	International Journal of Earth Sciences	99(2)	357-378
1671	05TH16	CAOB	North Great Xing'an Range	124.7300	52.3300	Felsic volcanic rocks	Tahe	138	150	6.81	34.9	0.1099	0.51252		-0.44	-2.30	-0.90	-0.64	-0.91	0.99	0.93	0.99			Ying JF et al.	2010	International Journal of Earth Sciences	99(2)	357-378
1672	05TH17	CAOB	North Great Xing'an Range	124.7300	52.3300	Felsic volcanic rocks	Tahe	138	150	6.35	33.1	0.1107	0.512493		-0.44	-2.83	-1.40	-1.18	-1.40	1.04	0.97	1.04			Ying JF et al.	2010	International Journal of Earth Sciences	99(2)	357-378
1673	05TH21	CAOB	North Great Xing'an Range	124.7300	52.3300	Felsic volcanic rocks	Tahe	138	150	5.9	30.6	0.0673	0.512549		-0.66	-1.74	0.40	0.74	-0.32	0.88	0.63	0.88			Ying JF et al.	2010	International Journal of Earth Sciences	99(2)	357-378
1674	05TH23	CAOB	North Great Xing'an Range	124.7300	52.3300	Felsic volcanic rocks	Tahe	138	150	4.47	20.9	0.1241	0.512526		-0.37	-2.18	-1.00	-0.80	-0.99	1.00	1.06	1.00			Ying JF et al.	2010	International Journal of Earth Sciences	99(2)	357-378
1675	05TH25	CAOB	North Great Xing'an Range	124.7300	52.3300	Felsic volcanic rocks	Tahe	138	150	8.12	42.3	0.1102	0.512537		-0.44	-1.97	-0.50	-0.31	-0.55	0.96	0.90	0.96			Ying JF et al.	2010	International Journal of Earth Sciences	99(2)	357-378
1676	ERBY04-1	CAOB	North Great Xing'an Range	121.5200	50.7800	Felsic volcanic rocks	Genhe	138	150	9.1	56.4	0.1075	0.512656		-0.45	0.35	2.00	2.06	2.10	0.77	0.71	0.77			Ying JF et al.	2010	International Journal of Earth Sciences	99(2)	357-378
1677	ERBY04-2	CAOB	North Great Xing'an Range	121.5200	50.7800	Felsic volcanic rocks	Genhe	138	150	8.91	54.7	0.0896	0.512647		-0.54	0.18	2.10	2.23	1.91	0.76	0.62	0.76			Ying JF et al.	2010	International Journal of Earth Sciences	99(2)	357-378
1678	ERBY04-4	CAOB	North Great Xing'an Range	121.5200	50.7800	Felsic volcanic rocks	Genhe	138	150	8.92	53.9	0.0866	0.512647		-0.56	0.18	2.10	2.28	1.88	0.75	0.60	0.75			Ying JF et al.	2010	International Journal of Earth Sciences	99(2)	357-378
1679	GH04-1	CAOB	North Great Xing'an Range	121.5200	50.7800	Felsic volcanic rocks	Genhe	138	150	7.98	41.2	0.1203	0.512762		-0.39	2.42	3.60	3.88	3.82	0.62	0.63	0.62			Ying JF et al.	2010	International Journal of Earth Sciences	99(2)	357-378
1680	GH04-2	CAOB	North Great Xing'an Range	121.5200	50.7800	Felsic volcanic rocks	Genhe	138	150	7.94	50	0.0999	0.512638		-0.49	0.00	1.50	1.85	1.78	0.79	0.69	0.79			Ying JF et al.	2010	International Journal of Earth Sciences	99(2)	357-378
1681	GH04-3	CAOB	North Great Xing'an Range	121.5200	50.7800	Felsic volcanic rocks	Genhe	138	150	5.1	28.4	0.1579	0.512663		-0.20	0.49	1.10	1.23	2.04	0.84	1.33	0.84			Ying JF et al.	2010	International Journal of Earth Sciences	99(2)	357-378
1682	GH04-4	CAOB	North Great Xing'an Range	121.5200	50.7800	Felsic volcanic rocks	Genhe	138	150	5.23	28.5	0.1191	0.512677		-0.39	0.76	2.00	2.25	2.27	0.76	0.76	0.76			Ying JF et al.	2010	International Journal of Earth Sciences	99(2)	357-378
1683	05MZL22	CAOB	NE China	117.4300	49.6000	Andesite		138	150	10.80	63.80	0.1030	0.512541		-0.48	-1.89	-0.10	-0.10	-0.10	0.95	0.84	0.95			Ying JF et al.	2010	International Journal of Earth Sciences	99（2）	357-378
1684	05MZL19	CAOB	NE China	117.4300	49.6000	Andesite		138	150	10.70	60.90	0.1043	0.512559		-0.47	-1.54	0.20	0.23	0.23	0.92	0.82	0.92			Ying JF et al.	2010	International Journal of Earth Sciences	99（2）	357-378
1685	05MZL14	CAOB	NE China	117.4300	49.6000	Rhyolite		138	150	4.10	22.00	0.1161	0.512515		-0.41	-2.40	-0.80	-0.86	-0.86	1.01	0.99	1.01			Ying JF et al.	2010	International Journal of Earth Sciences	99（2）	357-378
1686	05MZL13	CAOB	NE China	117.4300	49.6000	Rhyolite		138	150	5.12	26.60	0.1192	0.512511		-0.39	-2.48	-1.00	-0.99	-0.99	1.02	1.03	1.02			Ying JF et al.	2010	International Journal of Earth Sciences	99（2）	357-378
1687	05MZL11	CAOB	NE China	117.4300	49.6000	Andesite		138	150	10.20	54.10	0.1126	0.512544		-0.43	-1.83	-0.10	-0.22	-0.22	0.96	0.91	0.96			Ying JF et al.	2010	International Journal of Earth Sciences	99（2）	357-378
1688	05MZL09	CAOB	NE China	117.4300	49.6000	Andesite		138	150	9.83	53.50	0.1131	0.512523		-0.43	-2.24	-0.50	-0.64	-0.64	0.99	0.95	0.99			Ying JF et al.	2010	International Journal of Earth Sciences	99（2）	357-378
1689	05MZL08	CAOB	NE China	117.4300	49.6000	Andesite		138	150	9.86	52.50	0.1122	0.512535		-0.43	-2.01	-0.30	-0.39	-0.39	0.97	0.92	0.97			Ying JF et al.	2010	International Journal of Earth Sciences	99（2）	357-378
1690	05GH14	CAOB	NE China	121.5200	50.7800	Rhyolite		138	150	4.04	23.50	0.1044	0.512615		-0.47	-0.45	1.00	1.32	1.32	0.83	0.75	0.83			Ying JF et al.	2010	International Journal of Earth Sciences	99（2）	357-378
1691	05GH13	CAOB	NE China	121.5200	50.7800	Rhyolite		138	150	3.58	20.70	0.1045	0.512634		-0.47	-0.08	1.40	1.69	1.69	0.80	0.72	0.80			Ying JF et al.	2010	International Journal of Earth Sciences	99（2）	357-378
1692	05GH11	CAOB	NE China	121.5200	50.7800	Rhyolite		138	150	6.22	33.40	0.1074	0.512636		-0.45	-0.04	1.40	1.67	1.67	0.80	0.74	0.80			Ying JF et al.	2010	International Journal of Earth Sciences	99（2）	357-378
1693	BH5-4	CAOB	Altai, unit 4	86.5927	48.2326	Doleritic	Habahe	376	376	2.55	8.42	0.1843	0.512885	12	-0.06	4.82	5.40	5.41	5.41	0.68	1.37	0.68	0.72	0.68	Cai KD et al.	2010	Gondwana Research	18(4)	638–652
1694	BH5-3	CAOB	Altai, unit 4	86.5927	48.2326	Doleritic	Habahe	376	376	2.72	9.33	0.1774	0.512804	12	-0.10	3.24	4.20	4.16	4.16	0.78	1.45	0.78	0.72	0.78	Cai KD et al.	2010	Gondwana Research	18(4)	638–652
1695	BH24	CAOB	Altai, unit 4	86.5927	48.2326	Doleritic	Habahe	376	376	0.74	2.84	0.1586	0.512716	11	-0.19	1.52	3.40	3.35	3.35	0.85	1.20	0.85	0.72	0.85	Cai KD et al.	2010	Gondwana Research	18(4)	638–652
1696	BYH-10-15		Junggar	84.9352	46.5584	kali-feldspar granite porphy	Baiyanghe	313	313	2.29	11.10		0.512720		-1.00		4.06		4.06	0.85			0.85	0.85	Zhang X and Zhang H.	2014	Gondwana Research	25(4)	1554-1569
1697	BYH-10-14		Junggar	84.9352	46.5584	kali-feldspar granite porphy	Baiyanghe	313	313	1.90	9.84		0.512750		-1.00		4.91		4.91	0.72			0.85	0.72	Zhang X and Zhang H.	2014	Gondwana Research	25(4)	1554-1569
1698	BYH-10-13		Junggar	84.9352	46.5584	kali-feldspar granite porphy	Baiyanghe	313	313	1.53	8.45		0.512740		-1.00		5.08		5.08	0.67			0.85	0.67	Zhang X and Zhang H.	2014	Gondwana Research	25(4)	1554-1569
1699	BYH-10-12		Junggar	84.9352	46.5584	kali-feldspar granite porphy	Baiyanghe	313	313	1.96	10.10		0.512740		-1.00		5.29		5.29	0.63			0.85	0.63	Zhang X and Zhang H.	2014	Gondwana Research	25(4)	1554-1569
1700	XT-9	CAOB	SMXAB	125.7500	50.2333	Granite	Duobaoshan	300	300	2.50	13.18	0.1144	0.512660	1	-0.42	0.43	3.58	3.58	3.58	0.77	0.75	0.77	0.76	0.77	Liu C.	1994	Master's thesis of Peking University		
1701	XT-7	CAOB	SMXAB	125.7500	50.2333	Granite	Zhanzigou	240	240	3.54	24.38	0.0878	0.512810	2	-0.55	3.36	6.69	6.69	6.69	0.47	0.41	0.47	0.41	0.47	Liu C.	1994	Master's thesis of Peking University		
1702	XT-21	CAOB	SMXAB	125.7500	50.2333	Granite	Duobaoshan	300	300	0.90	5.27	0.1028	0.512590	2	-0.48	-0.94	2.66	2.66	2.66	0.85	0.77	0.85	0.77	0.85	Liu C.	1994	Master's thesis of Peking University		
1703	XF-5	CAOB	SMXAB	125.8333	50.1833	Granite	Tongshan	300	300	2.50	12.68	0.1193	0.512850	6	-0.39	4.14	7.11	7.10	7.10	0.48	0.49	0.48	0.49	0.49	Liu C.	1994	Master's thesis of Peking University		
1704	T W -16	CAOB	SMXAB	125.8333	50.1833	Granite	Tongshan	300	300	2.52	13.12	0.1164	0.512680	1	-0.41	0.82	3.89	3.89	3.89	0.74	0.74	0.74	0.74	0.75	Liu C.	1994	Master's thesis of Peking University		
1705	T W -14	CAOB	SMXAB	125.8333	50.1833	Granite	Tongshan	300	300	2.10	11.05	0.1151	0.512660	1	-0.41	0.43	3.56	3.55	3.55	0.77	0.76	0.77	0.76	0.77	Liu C.	1994	Master's thesis of Peking University		
1706	TBD-14	CAOB	SHB	119.0458	43.5417	Monzogranitic porphyry	Taibudai	137	150			0.0942	0.512454	7	-0.52	-3.59	-1.79	-1.63	-1.63	1.07	0.89	1.07	0.89	1.08	Zhou ZH et al.	2015	Journal of Asian Earth Sciences	103	212-228
1707	TBD-13	CAOB	SHB	119.0458	43.5417	Bi granite	Taibudai	138	150			0.0965	0.512466	8	-0.51	-3.36	-1.59	-1.44	-1.44	1.06	0.89	1.06	0.89	1.06	Zhou ZH et al.	2015	Journal of Asian Earth Sciences	103	212-228
1708	TBD-12	CAOB	SHB	119.0458	43.5417	Bi granite	Taibudai	138	150			0.0912	0.512450	6	-0.54	-3.67	-1.80	-1.65	-1.65	1.07	0.87	1.07	0.87	1.08	Zhou ZH et al.	2015	Journal of Asian Earth Sciences	103	212-228
1709	TBD-11	CAOB	SHB	119.0458	43.5417	Monzogranitic porphyry	Taibudai	137	150			0.0969	0.512457	8	-0.51	-3.53	-1.79	-1.62	-1.62	1.07	0.90	1.07	0.91	1.08	Zhou ZH et al.	2015	Journal of Asian Earth Sciences	103	212-228
1710	TBD-10	CAOB	SHB	119.0458	43.5417	Monzogranitic porphyry	Taibudai	137	150			0.0951	0.512441	8	-0.52	-3.84	-2.06	-1.90	-1.90	1.09	0.91	1.09	0.91	1.10	Zhou ZH et al.	2015	Journal of Asian Earth Sciences	103	212-228
1711	TBD-09	CAOB	SHB	119.0458	43.5417	Bi granite	Taibudai	138	150			0.0952	0.512459	7	-0.52	-3.49	-1.71	-1.55	-1.55	1.07	0.89	1.07	0.89	1.07	Zhou ZH et al.	2015	Journal of Asian Earth Sciences	103	212-228
1712	TBD-08	CAOB	SHB	119.0458	43.5417	Monzogranitic porphyry	Taibudai	137	150			0.1037	0.512447	10	-0.47	-3.73	-2.10	-1.95	-1.95	1.10	0.97	1.10	0.98	1.10	Zhou ZH et al.	2015	Journal of Asian Earth Sciences	103	212-228
1713	TBD-07	CAOB	SHB	119.0458	43.5417	Monzogranitic porphyry	Taibudai	137	150			0.0897	0.512459	6	-0.54	-3.49	-1.61	-1.44	-1.44	1.06	0.85	1.06	0.85	1.06	Zhou ZH et al.	2015	Journal of Asian Earth Sciences	103	212-228
1714	TBD-06	CAOB	SHB	119.0458	43.5417	Monzogranitic porphyry	Taibudai	137	150			0.0904	0.512430	7	-0.54	-4.06	-2.20	-2.02	-2.02	1.10	0.89	1.10	0.89	1.11	Zhou ZH et al.	2015	Journal of Asian Earth Sciences	103	212-228
1715	TBD-05	CAOB	SHB	119.0458	43.5417	Monzogranitic porphyry	Taibudai	137	150			0.0958	0.512450	10	-0.51	-3.67	-1.91	-1.74	-1.74	1.08	0.91	1.08	0.91	1.09	Zhou ZH et al.	2015	Journal of Asian Earth Sciences	103	212-228
1716	BS07-16	CAOB	Beishan	96.6853	40.7006	Hb granite	Qaiowan	304	304	2.86	17.10	0.1016	0.512429	13	-0.48	-4.08	-0.40	-0.39	-0.39	1.10	0.98	1.10			Feng JC et al.	2012	Acta Scientiarum Naturalium Universitatis Peki	6(2)	288-293
1717	BS07-17	CAOB	Beishan	96.6853	40.7006	Hb granite	Qaiowan	304	304	2.48	13.7	0.1097	0.512453	13	-0.44	-3.61	-0.24	-0.23	-0.23	1.08	1.02	1.08			Feng JC et al.	2012	Acta Scientiarum Naturalium Universitatis Peki	6(2)	288-293
1718	BS07-18	CAOB	Beishan	96.6853	40.7006	Hb granite	Qaiowan	304	304	3.82	20.4	0.1133	0.512469	13	-0.42	-3.30	-0.06	-0.06	-0.06	1.07	1.03	1.07			Feng JC et al.	2012	Acta Scientiarum Naturalium Universitatis Peki	6(2)	288-293
1719	TS-007	CAOB	Junggar	90.1151	46.4609	Andesite		385	385	3.05	11.70	0.1576	0.512822		-0.20	3.59	5.50	5.51	5.51	0.68	0.89	0.68	0.90	0.69	Liang P et al.	2016	Lithos	266-267	115-132
1720	TS-002	CAOB	Junggar	90.1151	46.4609	Syenite		376	376	1.54	6.70	0.1389	0.512821		-0.29	3.57	6.30	6.34	6.34	0.61	0.67	0.61	0.68	0.61	Liang P et al.	2016	Lithos	266-267	115-132
1721	LS-032	CAOB	Junggar	90.1151	46.4609	Syenite		376	376	1.83	8.00	0.1383	0.512779		-0.30	2.75	5.60	5.55	5.55	0.67	0.75	0.67	0.75	0.67	Liang P et al.	2016	Lithos	266-267	115-132
1722	BL-065	CAOB	East Tianshan	90.3417	41.8667	Granodiorite		318	317.7	1.54	7.00	0.1330	0.512705	5	-0.32	1.31	3.90	3.89	3.89	0.76	0.84	0.76		0.84	Zhang WF et al.	2016	Gondwana Research	38	40-59
1723	BL-058	CAOB	East Tianshan	91.3500	41.8306	Monzonitic granite		314	313.7	2.61	10.60	0.1488	0.512836	5	-0.24	3.86	5.78	5.78	5.78	0.60	0.74	0.60		0.74	Zhang WF et al.	2016	Gondwana Research	38	40-59
1724	BL-050	CAOB	East Tianshan	91.3500	41.8306	Granite		307	307.2	2.83	11.80	0.1450	0.512838	5	-0.26	3.90	5.94	5.93	5.93	0.58	0.69	0.58		0.69	Zhang WF et al.	2016	Gondwana Research	38	40-59
1725	BL-009	CAOB	East Tianshan	91.3250	41.8250	Dacitic tufflava		324	324.1	2.11	8.60	0.1483	0.512761	6	-0.25	2.40	4.41	4.40	4.40	0.72	0.91	0.72		0.91	Zhang WF et al.	2016	Gondwana Research	38	40-59
1726	BL-008	CAOB	East Tianshan	91.3167	41.8500	Dacitic tufflava		324	324.1	3.31	12.70	0.1576	0.512659	6	-0.20	0.41	2.03	2.03	2.03	0.91	1.33	0.91		1.33	Zhang WF et al.	2016	Gondwana Research	38	40-59
1727	BL-004	CAOB	East Tianshan	91.3000	41.8333	Dacitic tufflava		324	324.1	2.58	11.10	0.1405	0.512628	5	-0.29	-0.20	2.14	2.13	2.13	0.91	1.09	0.91		1.09	Zhang WF et al.	2016	Gondwana Research	38	40-59
1728	14SZ45	CAOB	Xing-Meng orogenic belt	115.5756	45.2993	Alkaline granites	Jinggesitai	301	301	6.94	34.10	0.1287	0.512775	3	-0.35	2.67	5.30	5.28	5.28	0.63	0.67	0.63	0.68	0.63	Wang SQ et al.	2017	Acta Geologica Sinica	91(7)	1467-1482
1729	14SZ44	CAOB	Xing-Meng orogenic belt	115.5756	45.2993	Alkaline granites	Jinggesitai	301	301	4.20	22.00	0.1207	0.512748	4	-0.39	2.15	5.10	5.06	5.06	0.65	0.66	0.65	0.66	0.65	Wang SQ et al.	2017	Acta Geologica Sinica	91(7)	1467-1482
1730	14SZ43	CAOB	Xing-Meng orogenic belt	115.5756	45.2993	Alkaline granites	Jinggesitai	301	301	5.85	30.70	0.1205	0.512748	3	-0.39	2.15	5.10	5.07	5.07	0.65	0.66	0.65	0.66	0.65	Wang SQ et al.	2017	Acta Geologica Sinica	91(7)	1467-1482
1731	14SZ42	CAOB	Xing-Meng orogenic belt	115.5756	45.2993	Alkaline granites	Jinggesitai	301	301	2.13	9.64	0.1397	0.512829	4	-0.29	3.73	5.90	5.91	5.91	0.58	0.66	0.58	0.66	0.58	Wang SQ et al.	2017	Acta Geologica Sinica	91(7)	1467-1482
1732	DWQ-2	CAOB	NE China	116.0000	46.0000	Andesite		311	311	5.85	24.83	0.1426	0.512421		-0.28	-4.23	-2.10	-2.09	-2.09	1.24	1.56	1.24	1.565		Fu D et al.	2016	Gondwana Research	36	545-560
1733	DWQ-1	CAOB	NE China	116.0000	46.0000	Andesite		311	311	9.01	42.37	0.1307	0.512386		-0.34	-4.92	-2.40	-2.30	-2.30	1.26	1.40	1.26	1.41		Fu D et al.	2016	Gondwana Research	36	545-560
1734	06XJ23-2	CAOB	Northern margin of Yili block	80.9786	44.6761	Granodiorite porphyry		366	366			0.1098	0.512239		-0.44	-7.78	-3.72	-3.72	-3.72	1.42	1.33	1.42		1.43	Tang G J et al.	2010	Lithos	119(3-4)	393-411
1735	06XJ22	CAOB	Northern margin of Yili block	80.9786	44.6761	Granodiorite porphyry		366	366			0.1060	0.512379		-0.46	-5.04	-0.80	-0.81	-0.81	1.18	1.09	1.18		1.19	Tang G J et al.	2010	Lithos	119(3-4)	393-411
1736	06XJ20	CAOB	Northern margin of Yili block	80.9786	44.6761	Granodiorite porphyry		366	366			0.1248	0.512177		-0.37	-8.99	-5.63	-5.63	-5.63	1.57	1.66	1.57		1.58	Tang G J et al.	2010	Lithos	119(3-4)	393-411
1737	06XJ19-2	CAOB	Northern margin of Yili block	80.9786	44.6761	Granodiorite porphyry		366	366			0.1101	0.512502		-0.44	-2.66	1.39	1.39	1.39	1.00	0.95	1.00		1.01	Tang G J et al.	2010	Lithos	119(3-4)	393-411
1738	06XJ15	CAOB	Northern margin of Yili block	81.4236	44.7417	Granite porphyry		290	290			0.1780	0.512514		-0.10	-2.42	-1.72	-1.72	-1.72	1.19	2.70	1.19		1.20	Tang G J et al.	2010	Lithos	119(3-4)	393-411
1739	06XJ13	CAOB	Northern margin of Yili block	81.4236	44.7417	Granite porphyry		290	290			0.1686	0.512534		-0.14	-2.02	-0.99	-0.99	-0.99	1.13	2.07	1.13		1.14	Tang G J et al.	2010	Lithos	119(3-4)	393-411
1740	06XJ06	CAOB	Northern margin of Yili block	81.4236	44.7417	Dacite		316	316			0.1374	0.512510		-0.30	-2.50	-0.11	-0.11	-0.11	1.08	1.28	1.08		1.09	Tang G J et al.	2010	Lithos	119(3-4)	393-411
1741	06XJ04	CAOB	Northern margin of Yili block	81.4236	44.7417	Dacite		316	316			0.1305	0.512672		-0.34	0.67	3.34	3.33	3.33	0.80	0.88	0.80		0.81	Tang G J et al.	2010	Lithos	119(3-4)	393-411
1742	06XJ018	CAOB	Northern margin of Yili block	80.9786	44.6761	Granodiorite porphyry		366	366			0.1389	0.512674		-0.29	0.69	3.40	3.39	3.39	0.84	0.97	0.84		0.84	Tang G J et al.	2010	Lithos	119(3-4)	393-411
1743	06XJ017	CAOB	Northern margin of Yili block	80.9786	44.6761	Granodiorite porphyry		366	366			0.1195	0.512266		-0.39	-7.25	-3.65	-3.65	-3.65	1.41	1.43	1.41		1.42	Tang G J et al.	2010	Lithos	119(3-4)	393-411
1744	06XJ010	CAOB	Northern margin of Yili block	81.4236	44.7417	Granite porphyry		290	290			0.1950	0.512635		-0.01	-0.06	0.00	0.00	0.00	1.04	4.16	1.04		1.06	Tang G J et al.	2010	Lithos	119(3-4)	393-411
1745	NM12-113	CAOB	NE China	114.0000	44.0000	Rhyolite	Dashizhai	276	276	8.79	41.70	0.1337	0.512678		-0.32	0.78	3.00	3.00	3.00	0.80	0.90	0.80	0.90	0.81	Zhang ZC et al.	2017	Journal of Asian Earth Sciences	135	370-389
1746	NM12-90	CAOB	NE China	114.0000	44.0000	Rhyolite	Dashizhai	276	276	12.00	53.20	0.1431	0.512935		-0.27	5.79	7.70	7.68	7.68	0.42	0.46	0.42	0.47	0.44	Zhang ZC et al.	2017	Journal of Asian Earth Sciences	135	370-389
1747	NM12-87	CAOB	NE China	114.0000	44.0000	Rhyolite	Dashizhai	276	276	6.06	28.40	0.1354	0.512729		-0.31	1.78	4.00	3.93	3.93	0.72	0.82	0.72	0.82	0.73	Zhang ZC et al.	2017	Journal of Asian Earth Sciences	135	370-389
1748	NM10-39	CAOB	NE China	114.0000	44.0000	Rhyolite	Dashizhai	276	276	16.60	70.70	0.1419	0.512691		-0.28	1.03	3.30	2.96	2.96	0.80	0.97	0.80	0.97	0.82	Zhang ZC et al.	2017	Journal of Asian Earth Sciences	135	370-389
1749	NM10-37	CAOB	NE China	114.0000	44.0000	Rhyolite	Dashizhai	276	276	6.45	27.70	0.1408	0.512669		-0.28	0.60	2.90	2.57	2.57	0.83	1.01	0.83	1.01	0.85	Zhang ZC et al.	2017	Journal of Asian Earth Sciences	135	370-389
1750	NM10-34	CAOB	NE China	114.0000	44.0000	Dacite	Dashizhai	276	276	6.27	28.30	0.1339	0.512657		-0.32	0.37	2.60	2.58	2.58	0.83	0.94	0.83	0.94	0.84	Zhang ZC et al.	2017	Journal of Asian Earth Sciences	135	370-389
1751	NM10-30	CAOB	NE China	114.0000	44.0000	Rhyolite	Dashizhai	276	276	10.20	41.20	0.1497	0.512704		-0.24	1.29	3.00	2.94	2.94	0.80	1.06	0.80	1.06	0.83	Zhang ZC et al.	2017	Journal of Asian Earth Sciences	135	370-389
1752	SDG2	NCC	NCC	109.6083	40.8039	Biotitic syenite-granite	Shadegai	222	222			0.0924	0.511754		-0.53	-17.24	-14.3	-14.3	-14.3	2.16	1.75	2.16	1.75	2.16	Hou WR et al.	2011	ournal of Jilin University (Earth Science Edition	41(6)	1914-1927
1753	SDG1	NCC	NCC	109.6083	40.8039	Biotitic syenite-granite	Shadegai	222	222			0.0790	0.511797		-0.60	-16.41	-13.1	-13.1	-13.1	2.06	1.53	2.06	1.53	2.06	Hou WR et al.	2011	ournal of Jilin University (Earth Science Edition	41(6)	1914-1927
1754	JN75	NCC	NCC	115.4000	40.8453	Granite	Shangshuiquan	140	150	6.87	28.30	0.1542	0.511792	10	-0.22	-16.50	-15.7	-15.7	-15.7	2.20	3.45	2.20	3.46	2.21	Miao LC et al.	2008	Journal of Asian Earth Sciences	32(5-6)	348-370
1755	JN74	NCC	NCC	115.4000	40.8453	Granite	Shangshuiquan	140	150	7.78	29.20	0.1638	0.511810	13	-0.17	-16.15	-15.6	-15.5	-15.5	2.18	4.05	2.18	4.06	2.20	Miao LC et al.	2008	Journal of Asian Earth Sciences	32(5-6)	348-370
1756	JN73	NCC	NCC	115.4000	40.8453	Granite	Shangshuiquan	140	150	6.44	28.00	0.1385	0.511748	6	-0.30	-17.36	-16.3	-16.2	-16.2	2.25	2.82	2.25	2.83	2.26	Miao LC et al.	2008	Journal of Asian Earth Sciences	32(5-6)	348-370
1757	JN72	NCC	NCC	115.4000	40.8453	Granite	Shangshuiquan	140	150	7.61	28.90	0.1581	0.511760	11	-0.20	-17.13	-16.5	-16.4	-16.4	2.26	3.78	2.26	3.79	2.27	Miao LC et al.	2008	Journal of Asian Earth Sciences	32(5-6)	348-370
1758	JN0749	NCC	NCC	115.4000	40.8453	Granite	Shangshuiquan	140	150	5.27	28.90	0.1042	0.511751	9	-0.47	-17.30	-15.7	-15.5	-15.5	2.20	1.94	2.20	1.94	2.20	Miao LC et al.	2008	Journal of Asian Earth Sciences	32(5-6)	348-370
1759	JN0747	NCC	NCC	115.4000	40.8453	Granite	Shangshuiquan	140	150	3.09	15.60	0.1252	0.511738	11	-0.36	-17.56	-16.3	-16.2	-16.2	2.25	2.42	2.25	2.43	2.25	Miao LC et al.	2008	Journal of Asian Earth Sciences	32(5-6)	348-370
1760	JN0746	NCC	NCC	115.4000	40.8453	Granite	Shangshuiquan	140	150	5.79	22.40	0.1658	0.511790	14	-0.16	-16.54	-16.0	-16.0	-16.0	2.22	4.28	2.22	4.29	2.23	Miao LC et al.	2008	Journal of Asian Earth Sciences	32(5-6)	348-370
1761	JN0743	NCC	NCC	115.4000	40.8453	Granite	Shangshuiquan	140	150	6.82	26.40	0.1568	0.511769	10	-0.20	-16.95	-16.3	-16.2	-16.2	2.24	3.67	2.24	3.68	2.25	Miao LC et al.	2008	Journal of Asian Earth Sciences	32(5-6)	348-370
1762	21DW-9	CAOB	SMXAB	116.4425	44.8614	Granodiorite	Hengengshan	244	244	1.57	7.64	0.1241	0.512872	6	-0.37	4.56	6.83	6.83	6.83	0.46	0.47	0.46	0.48	0.46	Miao LC et al.	2008	Journal of Asian Earth Sciences	32(5-6)	348-370
1763	21DW-8	CAOB	SMXAB	116.4425	44.8614	Granodiorite	Hengengshan	244	244	1.44	6.98	0.1249	0.512849	6	-0.37	4.12	6.37	6.35	6.35	0.50	0.52	0.50	0.52	0.50	Miao LC et al.	2008	Journal of Asian Earth Sciences	32(5-6)	348-370
1764	21DW-25	CAOB	SMXAB	116.4425	44.8614	Granodiorite	Hengengshan	244	244	1.50	7.18	0.1265	0.512867	6	-0.36	4.47	6.66	6.65	6.65	0.47	0.50	0.47	0.50	0.48	Miao LC et al.	2008	Journal of Asian Earth Sciences	32(5-6)	348-370
1765	21DW-11	CAOB	SMXAB	116.4425	44.8614	Granodiorite	Hengengshan	244	244	1.46	7.10	0.1246	0.512850	5	-0.37	4.14	6.39	6.38	6.38	0.50	0.51	0.50	0.52	0.50	Miao LC et al.	2008	Journal of Asian Earth Sciences	32(5-6)	348-370
1766	21DW-10	CAOB	SMXAB	116.4425	44.8614	Granodiorite	Hengengshan	244	244	1.46	7.23	0.1223	0.512843	8	-0.38	4.00	6.33	6.32	6.32	0.50	0.51	0.50	0.52	0.50	Miao LC et al.	2008	Journal of Asian Earth Sciences	32(5-6)	348-370

1767	rr-9	CAOB	EasternTianshan	91.3333	41.7000	Diorite		348	348	3.97	18.40	0.1305	0.512596		-0.34	-0.82	2.20	2.12	2.12	0.93	1.01	0.93	1.02	0.93	Li DF et al.	2016	Journal of Asian Earth Sciences	115	442-454
1768	rr-8	CAOB	EasternTianshan	91.3333	41.7000	Diorite		352	352	3.78	17.30	0.1322	0.512471		-0.33	-3.26	-0.40	-0.36	-0.36	1.13	1.27	1.13	1.27	1.13	Li DF et al.	2016	Journal of Asian Earth Sciences	115	442-454
1769	rr-7	CAOB	EasternTianshan	91.3333	41.7000	Diorite		352	352	4.01	18.30	0.1326	0.512224		-0.33	-8.08	-5.20	-5.20	-5.20	1.52	1.74	1.52	1.74	1.53	Li DF et al.	2016	Journal of Asian Earth Sciences	115	442-454
1770	rr-2	CAOB	EasternTianshan	91.3333	41.7000	Diorite		353	353	3.95	18.50	0.1292	0.512358		-0.34	-5.46	-2.40	-2.42	-2.42	1.30	1.43	1.30	1.43	1.30	Li DF et al.	2016	Journal of Asian Earth Sciences	115	442-454
1771	rr-11	CAOB	EasternTianshan	91.3333	41.7000	Diorite		348	348	3.67	17.50	0.1269	0.512397		-0.35	-4.70	-1.60	-1.60	-1.60	1.23	1.32	1.23	1.32	1.23	Li DF et al.	2016	Journal of Asian Earth Sciences	115	442-454
1772	D9-1	CAOB	Northern margin of Yili block	82.9000	44.1000	Monzonitic granite porphyry		350	350	4.27	22.37	0.1155	0.512335		-0.41	-5.91	-2.28	-2.28	-2.28	1.29	1.26	1.29	1.27	1.30	Zhang DY et al.	2010	Journal of Geology	118(5)	543-563
1773	D8-1	CAOB	Northern margin of Yili block	82.9000	44.1000	Monzonitic granite porphyry		350	350	4.26	20.43	0.1262	0.512328		-0.36	-6.05	-2.90	-2.90	-2.90	1.34	1.43	1.34	1.43	1.35	Zhang DY et al.	2010	Journal of Geology	118(5)	543-563
1774	D7-2	CAOB	Northern margin of Yili block	82.9000	44.1000	Granodiorite porphyry		350	350	4.20	20.34	0.1248	0.512355		-0.37	-5.52	-2.31	-2.31	-2.31	1.29	1.36	1.29	1.36	1.30	Zhang DY et al.	2010	Journal of Geology	118(5)	543-563
1775	D3-1	CAOB	Northern margin of Yili block	82.9000	44.1000	Granodiorite porphyry		350	350	3.33	15.85	0.1269	0.512228		-0.35	-8.00	-4.88	-4.88	-4.88	1.50	1.62	1.50	1.62	1.51	Zhang DY et al.	2010	Journal of Geology	118(5)	543-563
1776	D21-1	CAOB	Northern margin of Yili block	82.9000	44.1000	Granodiorite porphyry		390	390	3.15	15.32	0.1247	0.512459		-0.37	-3.49	0.10	0.09	0.09	1.13	1.18	1.13	1.18	1.13	Zhang DY et al.	2010	Journal of Geology	118(5)	543-563
1777	D21-01	CAOB	Tianshan	81.5000	44.5167	Plagioclase granite		390	390	3.15	15.32	0.1247	0.512459		-0.37	-3.49	0.09	0.09	0.09	1.13	1.18	1.13	1.18	1.13	Zhang DY et al.	2010	Journal of Geology	118(5)	543-563
1778	D20-2	CAOB	Northern margin of Yili block	82.9000	44.1000	Granodiorite porphyry		390	390	3.21	15.63	0.1251	0.512464		-0.36	-3.39	0.17	0.17	0.17	1.12	1.18	1.12	1.18	1.13	Zhang DY et al.	2010	Journal of Geology	118(5)	543-563
1779	D20-1	CAOB	Northern margin of Yili block	82.9000	44.1000	Granodiorite porphyry		390	390	3.35	15.87	0.1238	0.512456		-0.37	-3.55	0.08	0.08	0.08	1.13	1.18	1.13	1.18	1.13	Zhang DY et al.	2010	Journal of Geology	118(5)	543-563
1780	D20-02	CAOB	Tianshan	81.5000	44.5167	Plagioclase granite		390	390	3.21	15.63	0.1251	0.512464		-0.36	-3.39	0.17	0.17	0.17	1.12	1.18	1.12	1.18	1.12	Zhang DY et al.	2010	Journal of Geology	118(5)	543-563
1781	D20-01	CAOB	Tianshan	81.5000	44.5167	Granodiorite		390	390	3.35	15.87	0.1238	0.512456		-0.37	-3.55	0.08	0.08	0.08	1.13	1.18	1.13	1.18	1.13	Zhang DY et al.	2010	Journal of Geology	118(5)	543-563
1782	D19-2	CAOB	Northern margin of Yili block	82.9000	44.1000	Granodiorite porphyry		390	390	3.70	18.16	0.1271	0.512468		-0.35	-3.32	0.15	0.15	0.15	1.12	1.20	1.12	1.20	1.13	Zhang DY et al.	2010	Journal of Geology	118(5)	543-563
1783	D19-1	CAOB	Northern margin of Yili block	82.9000	44.1000	Granodiorite porphyry		390	390	3.80	17.95	0.1268	0.512443		-0.36	-3.80	-0.32	-0.33	-0.33	1.16	1.24	1.16	1.24	1.17	Zhang DY et al.	2010	Journal of Geology	118(5)	543-563
1784	D19-02	CAOB	Tianshan	81.5000	44.5167	Granodiorite		390	390	3.70	18.16	0.1271	0.512468		-0.35	-3.32	0.15	0.15	0.15	1.12	1.20	1.12	1.20	1.12	Zhang DY et al.	2010	Journal of Geology	118(5)	543-563
1785	D19-01	CAOB	Tianshan	81.5000	44.5167	Granodiorite		390	390	3.80	17.95	0.1268	0.512443		-0.36	-3.80	-0.33	-0.33	-0.33	1.16	1.24	1.16	1.24	1.16	Zhang DY et al.	2010	Journal of Geology	118(5)	543-563
1786	D18-1	CAOB	Northern margin of Yili block	82.9000	44.1000	Granodiorite porphyry		390	390	3.26	15.98	0.1256	0.512456		-0.36	-3.55	-0.01	-0.01	-0.01	1.14	1.20	1.14	1.20	1.14	Zhang DY et al.	2010	Journal of Geology	118(5)	543-563
1787	D18-01	CAOB	Tianshan	81.5000	44.5167	Granodiorite		390	390	3.26	15.98	0.1256	0.512456		-0.36	-3.55	-0.01	-0.01	-0.01	1.14	1.20	1.14	1.20	1.14	Zhang DY et al.	2010	Journal of Geology	118(5)	543-563
1788	D14-1	CAOB	Northern margin of Yili block	82.9000	44.1000	Monzonitic granite porphyry		350	350	3.36	17.06	0.1190	0.512382		-0.40	-4.99	-1.52	-1.52	-1.52	1.23	1.24	1.23	1.24	1.23	Zhang DY et al.	2010	Journal of Geology	118(5)	543-563
1789	D12-1	CAOB	Northern margin of Yili block	82.9000	44.1000	Monzonitic granite porphyry		350	350	5.70	25.25	0.1364	0.512469		-0.31	-3.30	-0.60	-0.60	-0.60	1.15	1.34	1.15	1.34	1.16	Zhang DY et al.	2010	Journal of Geology	118(5)	543-563
1790	D10-1	CAOB	Northern margin of Yili block	82.9000	44.1000	Monzonitic granite porphyry		350	350	6.38	31.83	0.1212	0.512353		-0.38	-5.56	-2.19	-2.19	-2.19	1.28	1.31	1.28	1.31	1.29	Zhang DY et al.	2010	Journal of Geology	118(5)	543-563
1791	TPC07-8	CAOB	CMEB	120.4292	51.4667	Porphyry	Taipingchuan	202	202	4.63	27.00	0.1039	0.512331	11	-0.47	-5.99	-3.59	-3.60	-3.60	1.27	1.14	1.27	1.14	1.28	Chen ZG et al.	2010	Acta Petrologica Sinica	26(5)	1437-1449
1792	TPC07-7	CAOB	CMEB	120.4292	51.4667	Porphyry	Taipingchuan	202	202	3.46	18.41	0.1137	0.512328	13	-0.42	-6.05	-3.90	-3.91	-3.91	1.30	1.25	1.30	1.26	1.30	Chen ZG et al.	2010	Acta Petrologica Sinica	26(5)	1437-1449
1793	TPC07-6	CAOB	CMEB	120.4292	51.4667	Porphyry	Taipingchuan	202	202	4.40	25.21	0.1057	0.512334	12	-0.46	-5.93	-3.59	-3.58	-3.58	1.27	1.15	1.27	1.15	1.28	Chen ZG et al.	2010	Acta Petrologica Sinica	26(5)	1437-1449
1794	TPC07-5	CAOB	CMEB	120.4292	51.4667	Porphyry	Taipingchuan	202	202	4.29	23.51	0.1104	0.512351	12	-0.44	-5.60	-3.38	-3.37	-3.37	1.26	1.18	1.26	1.18	1.26	Chen ZG et al.	2010	Acta Petrologica Sinica	26(5)	1437-1449
1795	TPC07-4	CAOB	CMEB	120.4292	51.4667	Porphyry	Taipingchuan	202	202	4.20	24.50	0.1038	0.512318	14	-0.47	-6.24	-3.84	-3.85	-3.85	1.29	1.15	1.29	1.16	1.30	Chen ZG et al.	2010	Acta Petrologica Sinica	26(5)	1437-1449
1796	TPC07-3	CAOB	CMEB	120.4292	51.4667	Porphyry	Taipingchuan	202	202	4.07	20.04	0.1229	0.512361	14	-0.38	-5.40	-3.49	-3.50	-3.50	1.27	1.32	1.27	1.33	1.27	Chen ZG et al.	2010	Acta Petrologica Sinica	26(5)	1437-1449
1797	TPC07-2	CAOB	CMEB	120.4292	51.4667	Porphyry	Taipingchuan	202	202	4.11	23.09	0.1078	0.512334	12	-0.45	-5.93	-3.65	-3.64	-3.64	1.28	1.17	1.28	1.18	1.28	Chen ZG et al.	2010	Acta Petrologica Sinica	26(5)	1437-1449
1798	TPC07-1	CAOB	CMEB	120.4292	51.4667	Porphyry	Taipingchuan	202	202	3.36	17.14	0.1186	0.512361	13	-0.40	-5.40	-3.40	-3.39	-3.39	1.26	1.26	1.26	1.27	1.26	Chen ZG et al.	2010	Acta Petrologica Sinica	26(5)	1437-1449
1799	09BDG-3*	CAOB	NE China	119.1333	49.9833	Granite		230	230	4.01	25.62	0.0993	0.512487		-0.50	-2.95	-0.09	-0.09	-0.09	1.01	0.88	1.01	0.88		Chen Z G.	2013	Acta Petrologica Sinica	26	1437-1449
1800	09BDG-2*	CAOB	NE China	119.1333	49.9833	Granite		230	230	3.26	16.72	0.1237	0.512539		-0.37	-1.93	0.21	0.21	0.21	0.99	1.03	0.99	1.03		Chen Z G.	2013	Acta Petrologica Sinica	26	1437-1450
1801	09BDG-1*	CAOB	NE China	119.1333	49.9833	Granite		230	230	4.17	23.69	0.1115	0.512534		-0.43	-2.03	0.47	0.47	0.47	0.97	0.92	0.97	0.92		Chen Z G.	2013	Acta Petrologica Sinica	26	1437-1451
1802	MO-158	CAOB	Transbaikalia and Mongolia	106.3000	44.9667	Rhyolite		208	208	27.90	138.70	0.1219	0.512602		-0.38	-0.70	1.28	1.28	1.28	0.88	0.91	0.88	0.91	0.88	Zhu MS et al.	2016	Journal of Asian Earth Sciences	122	41-57
1803	MO-156	CAOB	Transbaikalia and Mongolia	106.3000	44.9667	Rhyolite		208	208	28.20	143.60	0.1190	0.512603		-0.40	-0.68	1.38	1.38	1.38	0.87	0.88	0.87	0.88	0.87	Zhu MS et al.	2016	Journal of Asian Earth Sciences	122	41-57
1804	MO-145	CAOB	Transbaikalia and Mongolia	106.2833	44.9667	Granite		208	208	17.20	90.10	0.1157	0.512567		-0.41	-1.38	0.76	0.76	0.76	0.92	0.91	0.92	0.91	0.92	Zhu MS et al.	2016	Journal of Asian Earth Sciences	122	41-57
1805	MO-144	CAOB	Transbaikalia and Mongolia	106.2833	44.9667	Granite		208	208	19.90	105.90	0.1140	0.512567		-0.42	-1.38	0.81	0.81	0.81	0.92	0.89	0.92	0.89	0.92	Zhu MS et al.	2016	Journal of Asian Earth Sciences	122	41-57
1806	MO-142	CAOB	Transbaikalia and Mongolia	106.3000	44.9667	Rhyolite		208	208	35.40	182.80	0.1173	0.512562		-0.40	-1.48	0.62	0.62	0.62	0.94	0.93	0.94	0.93	0.94	Zhu MS et al.	2016	Journal of Asian Earth Sciences	122	41-57
1807	MO-132	CAOB	Transbaikalia and Mongolia	106.3000	44.9667	Rhyolite		208	208	28.10	148.00	0.1151	0.512552		-0.41	-1.68	0.49	0.49	0.49	0.95	0.92	0.95	0.92	0.95	Zhu MS et al.	2016	Journal of Asian Earth Sciences	122	41-57
1808	WS-9	CAOB	CMEB	117.3000	49.4167	Porphyry	Wunugetushan	179	179	2.37	13.65	0.1050	0.512586	12	-0.47	-1.01	1.08	1.08	1.08	0.87	0.79	0.87	0.79	0.88	Chen Z et al.	2011	Ore Geology Reviews	43(1)	92-105
1809	WS-5	CAOB	CMEB	117.3000	49.4167	Porphyry	Wunugetushan	179	179	2.20	14.50	0.0917	0.512531	12	-0.53	-2.09	0.32	0.31	0.31	0.94	0.77	0.94	0.78	0.94	Chen Z et al.	2011	Ore Geology Reviews	43(1)	92-105
1810	WS-4	CAOB	CMEB	117.3000	49.4167	Porphyry	Wunugetushan	179	179	1.85	12.71	0.0878	0.512561	10	-0.55	-1.50	0.98	0.98	0.98	0.88	0.71	0.88	0.72	0.89	Chen Z et al.	2011	Ore Geology Reviews	43(1)	92-105
1811	WS-3	CAOB	CMEB	117.3000	49.4167	Porphyry	Wunugetushan	179	179	2.90	16.99	0.1032	0.512577	13	-0.48	-1.19	0.94	0.95	0.95	0.89	0.79	0.89	0.79	0.89	Chen Z et al.	2011	Ore Geology Reviews	43(1)	92-105
1812	WS-2	CAOB	CMEB	117.3000	49.4167	Porphyry	Wunugetushan	179	179	1.67	11.22	0.0900	0.512552	14	-0.54	-1.68	0.77	0.76	0.76	0.90	0.74	0.90	0.74	0.90	Chen Z et al.	2011	Ore Geology Reviews	43(1)	92-105
1813	WS-1	CAOB	CMEB	117.3000	49.4167	Porphyry	Wunugetushan	179	179	1.81	12.33	0.0885	0.512559	12	-0.55	-1.54	0.93	0.93	0.93	0.89	0.72	0.89	0.72	0.89	Chen Z et al.	2011	Ore Geology Reviews	43(1)	92-105
1814	W08-170	CAOB	Alashan	107.1208	42.0172	Granite	Zhuxiaobuguhe	286	286	5.75	30.80	0.1128	0.512426	7	-0.43	-4.14	-0.93	-1.07	-1.07	1.14	1.09	1.14	1.09	1.13	Zheng RG et al.	2013	Acta Petrologica Sinica	29(8)	2664-2675
1815	W08-168	CAOB	Alashan	106.1208	42.0172	Granite	Zhuxiaobuguhe	286	286	6.12	35.50	0.1041	0.512360	3	-0.47	-5.42	-1.90	-2.05	-2.05	1.22	1.10	1.22	1.10	1.21	Zheng RG et al.	2013	Acta Petrologica Sinica	29(8)	2664-2675
1816	AB10-30	CAOB	Alashan	102.7761	41.8075	Bi monzogranite	Wudenghan	383	383	4.87	23.50	0.1253	0.512454	3	-0.36	-3.59	0.03	-0.10	-0.10	1.14	1.20	1.14	1.20	1.13	Zheng RG et al.	2013	Acta Petrologica Sinica	29(8)	2664-2675
1817	AB10-28	CAOB	Alashan	102.7761	41.8075	Bi monzogranite	Wudenghan	382	382	4.74	22.50	0.1274	0.512454	2	-0.35	-3.59	-0.08	-0.21	-0.21	1.15	1.23	1.15	1.23	1.14	Zheng RG et al.	2013	Acta Petrologica Sinica	29(8)	2664-2675
1818	HG-4-1	CAOB	SHB	117.4908	43.6689	Granitoids	Huanggang	137	150	9.39	28.70	0.1980	0.512634	6	0.01	-0.08	-0.09	-0.10	-0.10	0.93	4.94	0.93	4.99	0.94	Zhou ZH et al.	2012	Journal of Asian Earth Sciences	49	272-286
1819	HG-3-5	CAOB	SHB	117.4908	43.6689	Granitoids	Huanggang	137	150	5.63	25.30	0.1374	0.512600	6	-0.30	-0.74	0.30	0.39	0.39	0.91	1.10	0.91	1.10	0.91	Zhou ZH et al.	2012	Journal of Asian Earth Sciences	49	272-286
1820	HG-3-20	CAOB	SHB	117.4908	43.6689	Granitoids	Huanggang	137	150	5.05	25.40	0.1201	0.512617	10	-0.39	-0.41	0.92	1.06	1.06	0.85	0.87	0.85	0.87	0.86	Zhou ZH et al.	2012	Journal of Asian Earth Sciences	49	272-286
1821	HG-1-7	CAOB	SHB	117.4908	43.6689	Granitoids	Huanggang	137	150	7.36	40.90	0.1089	0.512520	9	-0.45	-2.30	-0.77	-0.62	-0.62	0.99	0.92	0.99	0.92	0.99	Zhou ZH et al.	2012	Journal of Asian Earth Sciences	49	272-286
1822	HG-1-21	CAOB	SHB	117.4908	43.6689	Granitoids	Huanggang	137	150	15.20	109.00	0.0842	0.512538	4	-0.57	-1.95	0.03	0.20	0.20	0.92	0.72	0.92	0.72	0.93	Zhou ZH et al.	2012	Journal of Asian Earth Sciences	49	272-286
1823	HG-1-10	CAOB	SHB	117.4908	43.6689	Granitoids	Huanggang	137	150	10.40	54.80	0.1149	0.512560	4	-0.42	-1.52	-0.09	0.04	0.04	0.94	0.91	0.94	0.91	0.94	Zhou ZH et al.	2012	Journal of Asian Earth Sciences	49	272-286
1824	G0217-2	CAOB	SMXAB	121.3769	46.5097	Granite	Chagan	230	230	10.03	45.52	0.1332	0.512846	8	-0.32	4.06	5.92	5.92	5.92	0.52	0.58	0.52	0.58	0.52	Zhou ZH et al.	2012	Journal of Asian Earth Sciences	49	272-286
1825	G0217-1	CAOB	SMXAB	121.3769	46.5097	Granite	Chagan	230	230	10.59	47.25	0.1355	0.512828	8	-0.31	3.71	5.51	5.50	5.50	0.56	0.63	0.56	0.63	0.56	Zhou ZH et al.	2012	Journal of Asian Earth Sciences	49	272-286
1826	G0215-5	CAOB	SMXAB	121.2781	46.6022	Granite-porphyry	Suolunzhen	125	150	3.51	20.39	0.1042	0.512736	8	-0.47	1.91	3.39	3.68	3.68	0.64	0.58	0.64	0.58	0.64	Zhou ZH et al.	2012	Journal of Asian Earth Sciences	49	272-286
1827	G0215-4	CAOB	SMXAB	121.2781	46.6022	Granite-porphyry	Suolunzhen	125	150	6.51	35.14	0.1120	0.512743	8	-0.43	2.05	3.39	3.67	3.67	0.64	0.61	0.64	0.61	0.64	Zhou ZH et al.	2012	Journal of Asian Earth Sciences	49	272-286
1828	G0215-1	CAOB	SMXAB	121.2781	46.6022	Granite-porphyry	Suolunzhen	125	150	5.23	29.35	0.1078	0.512725	8	-0.45	1.70	3.12	3.40	3.40	0.66	0.61	0.66	0.61	0.67	Zhou ZH et al.	2012	Journal of Asian Earth Sciences	49	272-286
1829	G0213-5	CAOB	SMXAB	121.2808	46.5947	Granite-porphyry	Jingyang	175	175	2.50	12.34	0.1223	0.512786	10	-0.38	2.89	4.55	4.55	4.55	0.59	0.61	0.59	0.61	0.59	Zhou ZH et al.	2012	Journal of Asian Earth Sciences	49	272-286
1830	G0213-4	CAOB	SMXAB	121.2808	46.5947	Granite-porphyry	Jingyang	175	175	2.07	10.21	0.1233	0.512787	9	-0.37	2.91	4.55	4.55	4.55	0.59	0.61	0.59	0.62	0.59	Zhou ZH et al.	2012	Journal of Asian Earth Sciences	49	272-286
1831	G0213-2	CAOB	SMXAB	121.2808	46.5947	Granite	Jingyang	175	175	2.28	11.43	0.1208	0.512826	10	-0.39	3.67	5.37	5.36	5.36	0.52	0.53	0.52	0.54	0.52	Zhou ZH et al.	2012	Journal of Asian Earth Sciences	49	272-286
1832	G0211-3	CAOB	SMXAB	121.3908	46.2519	Granite	Dashizhai	175	175	12.14	35.11	0.2092	0.512944	8	0.06	5.97	5.68	5.69	5.69	0.48	6.84	0.48	7.03	0.50	Zhou ZH et al.	2012	Journal of Asian Earth Sciences	49	272-286
1833	G0211-2	CAOB	SMXAB	121.3908	46.2519	Granite	Dashizhai	175	175	10.35	30.70	0.2040	0.512955	8	0.04	6.18	6.02	6.02	6.02	0.46	3.04	0.46	3.09	0.47	Zhou ZH et al.	2012	Journal of Asian Earth Sciences	49	272-286
1834	G0208-3	CAOB	SMXAB	122.1158	46.4850	Granodiorite	Qingshan	135	150	2.72	15.42	0.1067	0.512706	12	-0.46	1.33	2.88	3.05	3.05	0.69	0.63	0.69	0.64	0.69	Zhou ZH et al.	2012	Journal of Asian Earth Sciences	49	272-286
1835	G0208-1	CAOB	SMXAB	122.1158	46.4850	Granodiorite	Qingshan	135	150	2.85	14.92	0.1155	0.512706	11	-0.41	1.33	2.73	2.88	2.88	0.70	0.69	0.70	0.69	0.71	Zhou ZH et al.	2012	Journal of Asian Earth Sciences	49	272-286
1836	G0206-2	CAOB	SMXAB	122.6839	46.6042	Granite	Yonghe	128	150	5.28	28.83	0.1106	0.512674	14	-0.44	0.70	2.10	2.35	2.35	0.75	0.70	0.75	0.71	0.75	Zhou ZH et al.	2012	Journal of Asian Earth Sciences	49	272-286
1837	G0206-1	CAOB	SMXAB	122.6839	46.6042	Granite	Yonghe	128	150	4.65	24.41	0.1151	0.512718	13	-0.41	1.56	2.90	3.12	3.12	0.68	0.67	0.68	0.67	0.69	Zhou ZH et al.	2012	Journal of Asian Earth Sciences	49	272-286
1838	E-30	CAOB	Nouthern Mongolia	104.1333	49.1167	Quartzdiorite		240	240	3.95	26.90	0.0887	0.512715		-0.55	1.50	4.81	4.81	4.81	0.62	0.53	0.62	0.53		Jiang S et al.	2010	Acta Geoscientica Sinica	82(03)	424-436
1839	E-29	CAOB	Nouthern Mongolia	104.1333	49.1167	Quartzdiorite		240	240	3.59	24.30	0.0893	0.512727		-0.55	1.74	5.03	5.03	5.03	0.60	0.52	0.60	0.52		Jiang S et al.	2010	Acta Geoscientica Sinica	82(03)	424-436
1840	E-21	CAOB	Nouthern Mongolia	104.1333	49.0167	Quartzdiorite		240	240	3.01	20.90	0.0872	0.512769		-0.56	2.56	5.91	5.91	5.91	0.53	0.46	0.53	0.46		Jiang S et al.	2010	Acta Geoscientica Sinica	82(03)	424-436
1841	E-20	CAOB	Nouthern Mongolia	104.1333	49.0167	Quartzdiorite		240	240	2.74	18.90	0.0878	0.512623		-0.55	-0.29	3.05	3.04	3.04	0.76	0.64	0.76	0.64		Jiang S et al.	2010	Acta Geoscientica Sinica	82(03)	424-436
1842	E-16	CAOB	Nouthern Mongolia	104.1333	49.0167	Granodiorite		240	240	2.38	15.60	0.0921	0.512685		-0.53	0.92	4.12	4.12	4.12	0.68	0.58	0.68	0.59		Jiang S et al.	2010	Acta Geoscientica Sinica	82(03)	424-436
1843	E-15	CAOB	Nouthern Mongolia	104.1333	49.0167	Granodiorite		240	240	2.87	20.60	0.0842	0.512648		-0.57	0.20	3.64	3.64	3.64	0.72	0.59	0.72	0.59		Jiang S et al.	2010	Acta Geoscientica Sinica	82(03)	424-436
1844	E-14	CAOB	Nouthern Mongolia	104.1333	49.0167	Granodiorite		240	240	1.51	11.80	0.0772	0.512704		-0.61	1.29	4.95	4.95	4.95	0.61	0.50	0.61	0.50		Jiang S et al.	2010	Acta Geoscientica Sinica	82(03)	424-436
1845	E-13	CAOB	Nouthern Mongolia	104.1333	49.0167	Granodiorite		240	240	1.28	9.67	0.0803	0.512620		-0.59	-0.35	3.22	3.21	3.21	0.75	0.61	0.75	0.61		Jiang S et al.	2010	Acta Geoscientica Sinica	82(03)	424-436
1846	E-12	CAOB	Nouthern Mongolia	104.1333	49.0167	Granodiorite		240	240	1.57	12.60	0.0758	0.512609		-0.61	-0.57	3.14	3.14	3.14	0.76	0.60	0.76	0.60		Jiang S et al.	2010	Acta Geoscientica Sinica	82(03)	424-436
1847	JD16-007	NCC	NCC	118.7970	40.3042	Syenogranite		186	186	1.66	10.00	0.1004	0.511877		-0.49	-14.85	-12.5	-12.6	-12.6	1.99	1.71	1.99	1.71	1.99	Jiang SH et al.	2018	Lithos	312-313	21-37
1848	JD16-008	NCC	NCC	118.8139	40.2423	Granodiorite		166	166	2.71	15.70	0.1044	0.511966		-0.47	-13.10	-11.1	-11.1	-11.1	1.86	1.65	1.86	1.65	1.86	Jiang SH et al.	2018	Lithos	312-313	21-37
1849	JD16-061	NCC	NCC	118.2045	40.2800	Monzonite		199	199	9.09	57.90	0.0949	0.511893		-0.52	-14.54	-12.0	-12.0	-12.0	1.95	1.61	1.95	1.61	1.95	Jiang SH et al.	2018	Lithos	312-313	21-37
1850	JD16-072	NCC	NCC	117.8899	40.2931	Monzonite		126	150	3.57	21.60	0.0999	0.511691		-0.49	-18.48	-16.9	-16.6	-16.6	2.29	1.95	2.29	1.95	2.29	Jiang SH et al.	2018	Lithos	312-313	21-37
1851	JD16-077	NCC	NCC	118.8139	40.2423	Monzogranite		178	178	4.31	16.50	0.1579	0.511942		-0.20	-13.57	-12.7	-12.7	-12.7	1.98	3.27	1.98	3.28	2.00	Jiang SH et al.	2018	Lithos	312-313	21-37
1852	JD16-083	NCC	NCC	117.6417	40.3081	Monzogranite		170	170	3.01	18.10	0.1005	0.511908		-0.49	-14.23	-12.2	-12.1	-12.1	1.94	1.67	1.94	1.67	1.94	Jiang SH et al.	2018	Lithos	312-313	21-37
1853	JD16-086	NCC	NCC	117.6365	40.3157	Monzogranite		160	160	3.84	23.30	0.0996	0.511935		-0.49	-13.71	-11.6	-11.7	-11.7	1.90	1.62	1.90	1.62	1.90	Jiang SH et al.	2018	Lithos	312-313	21-37
1854	JD16-088	NCC	NCC	117.7569	40.3055	Monzogranite		164	164	3.92	25.80	0.0919	0.511687		-0.53	-18.56	-16.4	-16.4	-16.4	2.28	1.83	2.28	1.83	2.28	Jiang SH et al.	2018	Lithos	312-313	21-37
1855	JD16-095	NCC	NCC	118.8449	40.4356	Monzogranite		218	218	2.74	19.00	0.0872	0.511804		-0.56	-16.28	-13.2	-13.2	-13.2	2.07	1.62	2.07	1.62	2.07	Jiang SH et al.	2018	Lithos	312-313	21-37
1856	JD16-100	NCC	NCC	118.9334	40.4766	Monzogranite		162	162	2.16	11.50	0.1136	0.511786		-0.42	-16.62	-14.9	-14.9	-14.9	2.16	2.07	2.16	2.07	2.16	Jiang SH et al.	2018	Lithos	312-313	21-37
1857	JD16-102	NCC	NCC	118.9080	40.4628	Monzogranite		170	170	2.93	15.20	0.1165	0.511769		-0.41	-16.95	-15.2	-15.2	-15.2	2.19	2.16	2.19	2.16	2.19	Jiang SH et al.	2018	Lithos	312-313	21-37
1858	JD16-104	NCC	NCC	118.9023	40.3678	Granodiorite		150	150	4.33	30.70	0.0853	0.511602		-0.57	-20.20	-18.1	-18.1	-18.1	2.41	1.83	2.41	1.83	2.41	Jiang SH et al.	2018	Lithos	312-313	21-37
1859	JD16-107	NCC	NCC	118.9795	40.3284	Syenogranite		189	189	3.32	19.70	0.1019	0.511901		-0.48	-14.37	-12.1	-12.1	-12.1	1.95	1.70	1.95	1.70	1.95	Jiang SH et al.	2018	Lithos	312-313	21-37
1860	SKG-081	CAOB	SMXAB	125.7119	50.6411	Granodiorite	Sankuanggou	176	176	3.60	19.70	0.1108	0.512663		-0.44	0.49	2.41	2.42	2.42	0.76	0.72	0.76	0.72	0.77	Hu XL.	2016	Geological Journal	51(1)	1-20
1861	DHS-0151	CAOB	SMXAB	126.5350	50.3206	Granodiorite	Daheishan	147	150	4.00	24.30	0.0989	0.512560		-0.50	-1.52	0.32	0.35	0.35	0.91	0.78	0.91	0.79	0.91	Hu XL.	2016	Geological Journal	51(1)	1-20
1862	CLK-011	CAOB	SMXAB	123.9478	51.2050	Bi monzogranite	Chalukou	163	163	3.10	20.30	0.0908	0.512350		-0.54	-5.62	-3.42	-3.42	-3.42	1.23	0.99	1.23	0.99	1.23	Hu XL.	2016	Geological Journal	51(1)	1-20
1863	AGM-1		Junggar	84.8060	45.8367	Dike		316	316	3.38	15.60	0.1310	0.512863	14	-0.33	4.39	6.89	7.04	7.04	0.50	0.53	0.50	0.53	0.50	Li D et al.	2015	Geoscience Frontiers	6(2)	153-173
1864	AG-2		Junggar	84.7860	45.8167	Host granite		300	300	3.00	14.70	0.1234	0.512911	13	-0.37	5.33	8.14	8.13	8.13	0.40	0.40	0.40	0.41	0.40	Li D et al.	2015	Geoscience Frontiers	6(2)	153-173

1865	AG-10		Junggar	84.7860	45.8167	Host granite		300	300	4.20	18.70	0.1358	0.512891	11	-0.31	4.94	7.27	7.27	7.27	0.47	0.51	0.47	0.51	0.47	Li D et al.	2015	Geoscience Frontiers	6(2)	153-173
1866	AG-1		Junggar	84.7860	45.8167	Host granite		300	300	3.11	14.50	0.1297	0.512880	14	-0.34	4.72	7.29	7.29	7.29	0.47	0.49	0.47	0.49	0.47	Li D et al.	2015	Geoscience Frontiers	6(2)	153-173
1867	ABG-2		Junggar	84.7960	45.8267	MME		301	301	4.15	17.50	0.1434	0.512884	14	-0.27	4.80	6.85	6.85	6.85	0.50	0.58	0.50	0.58	0.50	Li D et al.	2015	Geoscience Frontiers	6(2)	153-173
1868	ABG-1		Junggar	84.7960	45.8267	MME		301	301	4.18	17.20	0.1469	0.512965	11	-0.25	6.38	8.29	8.29	8.29	0.39	0.42	0.39	0.43	0.39	Li D et al.	2015	Geoscience Frontiers	6(2)	153-173
1869	BYN1	CAOB	SHB	118.8373	44.4887	Bi-syenogranite	Baiyinnuoer	135	150			0.1190	0.512622	10	-0.40	-0.31	1.03	1.18	1.18	0.84	0.85	0.84	0.85	0.85	Jiang SH et al.	2011	Mineral Deposits	30(5)	787-798
1870	BY09-5	CAOB	SHB	118.8864	44.4485	Granodiorite	Baiyinnuoer	245	245			0.1139	0.512601	8	-0.42	-0.72	1.86	1.87	1.87	0.86	0.84	0.86	0.84	0.87	Jiang SH et al.	2011	Mineral Deposits	30(5)	787-798
1871	BY09-3	CAOB	SHB	118.8860	44.4489	Granodiorite	Baiyinnuoer	245	245			0.1248	0.512557	5	-0.37	-1.58	0.67	0.67	0.67	0.96	1.02	0.96	1.02	0.96	Jiang SH et al.	2011	Mineral Deposits	30(5)	787-798
1872	BY09-2	CAOB	SHB	118.8364	44.4852	Bi-syenogranite	Baiyinnuoer	135	150			0.1004	0.512611	7	-0.49	-0.53	1.13	1.32	1.32	0.83	0.73	0.83	0.73	0.84	Jiang SH et al.	2011	Mineral Deposits	30(5)	787-798
1873	BY09-19	CAOB	SHB	118.8860	44.4489	Granodiorite	Baiyinnuoer	245	245			0.1124	0.512588	7	-0.43	-0.98	1.67	1.66	1.66	0.88	0.85	0.88	0.85	0.88	Jiang SH et al.	2011	Mineral Deposits	30(5)	787-798
1874	BY09-1	CAOB	SHB	118.8376	44.4884	Bi-syenogranite	Baiyinnuoer	135	150			0.1120	0.512550	9	-0.43	-1.72	-0.26	-0.10	-0.10	0.95	0.90	0.95	0.90	0.95	Jiang SH et al.	2011	Mineral Deposits	30(5)	787-798
1875	MY-8	CAOB	Altai, unit 3	89.0011	47.5189	Gneissic plagioclase granit	Monku	400	400	4.67	22.57	0.1252	0.512504	13	-0.36	-2.61	1.04	1.04	1.04	1.06	1.11	1.06	1.11	1.05	Yang FQ et al.	2008	Acta Geologica Sinica	82(4)	485-499
1876	MY-7	CAOB	Altai, unit 3	89.0011	47.5189	Gneissic plagioclase granit	Monku	400	400	7.06	33.82	0.1263	0.512563	13	-0.36	-1.46	2.14	2.13	2.13	0.97	1.02	0.97	1.02	0.97	Yang FQ et al.	2008	Acta Geologica Sinica	82(4)	485-499
1877	MY-6	CAOB	Altai, unit 3	89.0011	47.5189	Gneissic plagioclase granit	Monku	400	400	6.65	30.67	0.1310	0.512566	13	-0.33	-1.40	1.95	1.95	1.95	0.98	1.08	0.98	1.08	0.98	Yang FQ et al.	2008	Acta Geologica Sinica	82(4)	485-499
1878	MY-5	CAOB	Altai, unit 3	89.0011	47.5189	Gneissic plagioclase granit	Monku	400	400	9.15	45.02	0.1229	0.512528	12	-0.38	-2.15	1.63	1.62	1.62	1.01	1.04	1.01	1.04	1.02	Yang FQ et al.	2008	Acta Geologica Sinica	82(4)	485-499
1879	MY-4	CAOB	Altai, unit 3	89.0011	47.5189	Gneissic plagioclase granit	Monku	400	400	8.05	39.62	0.1229	0.512541	9	-0.38	-1.89	1.88	1.87	1.87	0.99	1.02	0.99	1.02	1.00	Yang FQ et al.	2008	Acta Geologica Sinica	82(4)	485-499
1880	MY-3	CAOB	Altai, unit 3	89.0011	47.5189	Gneissic plagioclase granit	Monku	400	400	8.53	38.89	0.1325	0.512574	13	-0.33	-1.25	2.03	2.03	2.03	0.98	1.08	0.98	1.08	0.98	Yang FQ et al.	2008	Acta Geologica Sinica	82(4)	485-499
1881	MY-2	CAOB	Altai, unit 3	89.0011	47.5189	Gneissic plagioclase granit	Monku	400	400	7.66	35.05	0.1321	0.512580	15	-0.33	-1.13	2.17	2.17	2.17	0.97	1.06	0.97	1.06	0.97	Yang FQ et al.	2008	Acta Geologica Sinica	82(4)	485-499
1882	MY-1	CAOB	Altai, unit 3	89.0011	47.5189	Gneissic plagioclase granit	Monku	400	400	9.13	42.80	0.1289	0.512542	13	-0.34	-1.87	1.59	1.59	1.59	1.01	1.09	1.01	1.09	1.01	Yang FQ et al.	2008	Acta Geologica Sinica	82(4)	485-499
1883	CWL-72	CAOB	South Tianshan	76.6667	40.7667	Monzonite		286	286	5.91	34.98	0.1017	0.512342		-0.48	-5.77	-2.31	-2.31	-2.31	1.24	1.10	1.24	1.10	1.24	Huang H et al.	2014	Lithos	190-191	37-51
1884	CWL-71	CAOB	South Tianshan	76.6667	40.7667	Granite		286	286	3.21	19.76	0.0984	0.512336		-0.50	-5.89	-2.30	-2.30	-2.30	1.24	1.08	1.24	1.08	1.24	Huang H et al.	2014	Lithos	190-191	37-51
1885	CWL-40	CAOB	South Tianshan	76.6667	40.7667	Syenite		286	286	4.25	26.37	0.0981	0.512357		-0.50	-5.48	-1.88	-1.88	-1.88	1.20	1.05	1.20	1.05	1.20	Huang H et al.	2014	Lithos	190-191	37-51
1886	CWL-38	CAOB	South Tianshan	76.6667	40.7667	Bi Qtz monzonite		286	286	4.68	28.70	0.0974	0.512362		-0.50	-5.38	-1.76	-1.76	-1.76	1.19	1.03	1.19	1.03	1.19	Huang H et al.	2014	Lithos	190-191	37-51
1887	CWL-23	CAOB	South Tianshan	76.6667	40.7667	Bi Qtz monzonite		286	286	4.57	28.66	0.0956	0.512354		-0.51	-5.54	-1.85	-1.85	-1.85	1.20	1.03	1.20	1.03	1.20	Huang H et al.	2014	Lithos	190-191	37-51
1888	LY-6-27	CAOB	Beishan	95.0217	41.1775	Potash feldspar granite	Huitongshan	436	436	10.68	54.94	0.1176	0.512409	9	-0.40	-4.47	-0.06	-0.06	-0.06	1.18	1.17	1.18	1.17	1.18	Zhao ZH.	2007	Acta Petrologica Sinica	23(8)	1847-1860
1889	LY-6-18	CAOB	Beishan	95.6667	41.2833	Monzogranite	Liuyuan	396	396	6.67	33.60	0.1200	0.512346	11	-0.39	-5.70	-1.82	-1.82	-1.82	1.29	1.31	1.29	1.31	1.28	Zhao ZH.	2007	Acta Petrologica Sinica	23(8)	1847-1860
1890	LY-3-4	CAOB	Beishan	95.5833	41.2333	Granodiorite	Liuyuan	450	450	4.30	23.67	0.1100	0.512266	10	-0.44	-7.26	-2.28	-2.28	-2.28	1.37	1.30	1.37	1.30	1.41	Zhao ZH.	2007	Acta Petrologica Sinica	23(8)	1847-1860
1891	LY-3-24	CAOB	Beishan	95.6333	41.2500	Potash feldspar granite	Huanniushan	400	400	6.55	46.88	0.0845	0.512428	10	-0.57	-4.10	1.63	1.63	1.63	1.01	0.85	1.01	0.85	1.15	Zhao ZH.	2007	Acta Petrologica Sinica	23(8)	1847-1860
1892	LY-2-61	CAOB	Beishan	95.0217	41.1775	Potash feldspar granite	Huitongshan	436	436	3.64	20.72	0.1061	0.512250	11	-0.46	-7.57	-2.53	-2.53	-2.53	1.38	1.27	1.38	1.27	1.43	Zhao ZH.	2007	Acta Petrologica Sinica	23(8)	1847-1860
1893	LY-2-55	CAOB	Beishan	95.6500	41.2667	Monzogranite	Liuyuan	396	396	7.04	31.75	0.1340	0.512283	10	-0.32	-6.92	-3.76	-3.76	-3.76	1.44	1.65	1.44	1.65	1.38	Zhao ZH.	2007	Acta Petrologica Sinica	23(8)	1847-1860
1894	LY-12-17	CAOB	Beishan	95.5667	41.2167	Granodiorite	Liuyuan	430	430	4.53	34.78	0.0788	0.512275	9	-0.60	-7.08	-0.61	-0.61	-0.61	1.22	0.99	1.22	0.99	1.39	Zhao ZH.	2007	Acta Petrologica Sinica	23(8)	1847-1860
1895	H18	CAOB	NE China	120.4736	45.4633	Rhyolite		123	150	5.18	30.98	0.1012	0.512662		-0.49	0.47	1.98	2.30	2.30	0.75	0.66	0.75		0.76	Yang WB et al.	2015	Journal of Asian Earth Sciences	113	454-470
1896	H16	CAOB	NE China	120.4736	45.4633	Rhyolite		123	150	5.83	32.92	0.1071	0.512605		-0.46	-0.64	0.76	1.07	1.07	0.85	0.78	0.85		0.85	Yang WB et al.	2015	Journal of Asian Earth Sciences	113	454-470
1897	H14	CAOB	NE China	120.4736	45.4633	Rhyolite		123	150	5.50	31.67	0.1049	0.512670		-0.47	0.62	2.08	2.38	2.38	0.74	0.67	0.74		0.75	Yang WB et al.	2015	Journal of Asian Earth Sciences	113	454-470
1898	H13	CAOB	NE China	120.4736	45.4633	Rhyolite		123	150	5.38	30.54	0.1065	0.512581		-0.46	-1.11	0.30	0.61	0.61	0.89	0.81	0.89		0.89	Yang WB et al.	2015	Journal of Asian Earth Sciences	113	454-470
1899	12WLT-5.2	NCC	NCC	106.6604	41.3028	Granodiorite	Alunzhusilang	260	260	1.83	8.20	0.1349	0.512449	10	-0.31	-3.69	-1.64	-1.64	-1.64	1.16	1.35	1.16	1.36	1.16	Wang ZZ et al.	2015	Journal of Asian Earth Sciences	97(B)	337-351
1900	12WLT-14.2	NCC	NCC	106.3280	41.1528	Two-mica granite	Shandamiao	418	418	3.41	21.30	0.0968	0.511972	7	-0.51	-12.99	-7.67	-7.66	-7.66	1.78	1.53	1.78	1.54	1.78	Wang ZZ et al.	2015	Journal of Asian Earth Sciences	97(B)	337-351
1901	12WLT-13.2	NCC	NCC	106.8560	41.2529	Granodiorite	Shaerchulu	273	273	4.92	18.40	0.1617	0.512133	6	-0.18	-9.85	-8.64	-8.63	-8.63	1.73	2.96	1.73	2.97	1.74	Wang ZZ et al.	2015	Journal of Asian Earth Sciences	97(B)	337-351
1902	11HB20	NCC	NCC	109.3719	40.7075	Biotitic monzonite-granite	Dahuabei	328	328			0.0919	0.511974	3	-0.53	-12.95	-8.56	-8.57	-8.57	1.78	1.47	1.78	1.47	1.78	Wang ZZ et al.	2015	Journal of Asian Earth Sciences	97(B)	337-351
1903	11HB18	NCC	NCC	109.3719	40.7075	Biotitic monzonite-granite	Dahuabei	328	328			0.0909	0.511960	2	-0.54	-13.23	-8.80	-8.80	-8.80	1.80	1.47	1.80	1.48	1.80	Wang ZZ et al.	2015	Journal of Asian Earth Sciences	97(B)	337-351
1904	11HB16	NCC	NCC	109.3719	40.7075	Biotitic monzonite-granite	Dahuabei	328	328			0.0909	0.511990	2	-0.54	-12.64	-8.21	-8.21	-8.21	1.75	1.44	1.75	1.44	1.76	Wang ZZ et al.	2015	Journal of Asian Earth Sciences	97(B)	337-351
1905	11HB13	NCC	NCC	109.3719	40.7075	Biotitic monzonite-granite	Dahuabei	328	328			0.1130	0.512005	7	-0.43	-12.35	-8.86	-8.84	-8.84	1.80	1.73	1.80	1.73	1.81	Wang ZZ et al.	2015	Journal of Asian Earth Sciences	97(B)	337-351
1906	12WLT-8.2	NCC	Langshan	107.4840	41.3134	Gabbro-diorite	Langshan	273	273	7.46	34.10	0.1323	0.512301	0	-0.33	-6.57	-4.19	-4.33	-4.33	1.39	1.59	1.39	1.59	1.38	Wang ZZ et al.	2015	Journal of Asian Earth Sciences	97(B)	337-351
1907	12WLT-5.1	NCC	Langshan	106.6604	41.3028	Granodiorite	Langshan	260	260	2.28	8.30	0.1661	0.512449	0	-0.16	-3.69	-2.54	-2.67	-2.67	1.24	2.24	1.24	2.24	1.23	Wang ZZ et al.	2015	Journal of Asian Earth Sciences	97(B)	337-351
1908	12WLT-17.2	NCC	Langshan	106.9304	41.4815	Leuconorite	Langshan	255	255	0.84	3.30	0.1539	0.512598	0	-0.22	-0.78	0.75	0.61	0.61	0.97	1.40	0.97	1.40	0.97	Wang ZZ et al.	2015	Journal of Asian Earth Sciences	97(B)	337-351
1909	12WLT-17.1	NCC	Langshan	106.9304	41.4815	Leuconorite	Langshan	255	255	0.97	3.00	0.1955	0.512600	0	-0.01	-0.74	-0.57	-0.70	-0.70	1.07	4.55	1.07	4.55	1.04	Wang ZZ et al.	2015	Journal of Asian Earth Sciences	97(B)	337-351
1910	12WLT-14.1	NCC	Langshan	106.3280	41.1528	Granite	Langshan	418	418	3.66	26.50	0.0835	0.511976	0	-0.58	-12.91	-6.74	-6.88	-6.88	1.72	1.37	1.72	1.37	1.71	Wang ZZ et al.	2015	Journal of Asian Earth Sciences	97(B)	337-351
1911	12WLT-13.1	NCC	WNCC	106.8560	41.2529	Granodiorite	Langshan	273	273	5.55	31.30	0.1072	0.512096	0	-0.46	-10.57	-7.31	-7.45	-7.45	1.65	1.51	1.65	1.51	1.64	Wang ZZ et al.	2015	Journal of Asian Earth Sciences	97(B)	337-351
1912	09SF17	CAOB	East Tianshan	91.8333	42.6667	Rhyolite		280	280	8.01	29.10	0.1666	0.513158		-0.15	10.14	11.2	11.2	11.2	0.38	-0.03	0.13		0.13	Mao QG et al.	2014	Lithos	190-191	37-51
1913	09SF10	CAOB	East Tianshan	91.8333	42.6667	Granite		280	280	12.52	61.90	0.1223	0.512861		-0.38	4.35	7.02	7.01	7.01	0.47	0.48	0.47	0.48	0.48	Mao QG et al.	2014	Lithos	190-191	37-51
1914	HJS-11	CAOB	South Tianshan	88.1333	42.1333	Plagioclase granite	Huangjianshi	423	423	2.94	21.40	0.0734	0.512106	10	-0.63	-10.38	-3.72	-3.72	-3.72	1.47	1.13	1.47		1.47	Zhang CL et al.	2007	Acta Petrologica Sinica	23(8)	1821-1829
1915	HJS-07	CAOB	Tianshan	88.1333	42.1333	Plagioclase granite	Huangjianshi	423	423	3.56	14.50	0.1672	0.512181		-0.15	-8.91	-7.32	-7.32	-7.32	1.75	3.15	1.75	3.15	1.75	Zhang CL et al.	2007	Acta Petrologica Sinica	23(8)	1821-1829
1916	HJS-05	CAOB	South Tianshan	88.0833	42.1167	Plagioclase granite	Huangjianshi	423	423			0.1746	0.512335	14	-0.11	-5.91	-4.72	-4.72	-4.72	1.54	3.15	1.54		1.54	Zhang CL et al.	2007	Acta Petrologica Sinica	23(8)	1821-1829
1917	HJS-04	CAOB	rthern margin of South Tiansh	88.0833	42.1167	Plagioclase granite	Huangjianshishan	423	423			0.2508	0.512490		0.28	-2.89	-5.82	-5.81	-5.81	1.66	-2.74	1.66		1.64	Zhang CL et al.	2007	Acta Petrologica Sinica	23(8)	1821-1829
1918	HJS-03	CAOB	South Tianshan	88.0833	42.1167	Plagioclase granite	Huangjianshi	423	423	5.16	22.30	0.1519	0.512211	8	-0.23	-8.33	-5.91	-5.91	-5.91	1.64	2.31	1.64		1.64	Zhang CL et al.	2007	Acta Petrologica Sinica	23(8)	1821-1829
1919	HSG48	CAOB	SHB	118.9686	44.0567	Granite	Haisugou	138	150			0.1187	0.512607	3	-0.40	-0.60	0.77	0.89	0.89	0.87	0.87	0.87	0.87	0.87	Shu QH et al.	2014	Journal of Asian Earth Sciences	79(pt.B)	777–791
1920	HSG47	CAOB	SHB	118.9686	44.0567	Granite	Haisugou	138	150			0.1309	0.512593	11	-0.33	-0.88	0.28	0.38	0.38	0.91	1.03	0.91	1.03	0.91	Shu QH et al.	2014	Journal of Asian Earth Sciences	79(pt.B)	777–791
1921	HSG46	CAOB	SHB	118.9686	44.0567	Granite	Haisugou	138	150			0.1268	0.512595	2	-0.36	-0.84	0.40	0.50	0.50	0.90	0.97	0.90	0.98	0.90	Shu QH et al.	2014	Journal of Asian Earth Sciences	79(pt.B)	777–791
1922	HSG45	CAOB	SHB	118.9686	44.0567	Granite	Haisugou	138	150			0.1287	0.512619	2	-0.35	-0.37	0.83	0.93	0.93	0.86	0.95	0.86	0.96	0.86	Shu QH et al.	2014	Journal of Asian Earth Sciences	79(pt.B)	777–791
1923	HSG44	CAOB	SHB	118.9686	44.0567	Granite	Haisugou	138	150			0.1354	0.512655	5	-0.31	0.33	1.42	1.50	1.50	0.82	0.96	0.82	0.97	0.82	Shu QH et al.	2014	Journal of Asian Earth Sciences	79(pt.B)	777–791
1924	HSG43	CAOB	SHB	118.9686	44.0567	Granite	Haisugou	138	150			0.1237	0.512583	2	-0.37	-1.07	0.21	0.32	0.32	0.91	0.96	0.91	0.96	0.91	Shu QH et al.	2014	Journal of Asian Earth Sciences	79(pt.B)	777–791
1925	HSG42	CAOB	SHB	118.9686	44.0567	Granite	Haisugou	138	150			0.1264	0.512625	3	-0.36	-0.25	0.99	1.09	1.09	0.85	0.92	0.85	0.92	0.85	Shu QH et al.	2014	Journal of Asian Earth Sciences	79(pt.B)	777–791
1926	HSG41	CAOB	SHB	118.9686	44.0567	Granite	Haisugou	138	150			0.1320	0.512609	2	-0.33	-0.57	0.58	0.67	0.67	0.88	1.01	0.88	1.01	0.89	Shu QH et al.	2014	Journal of Asian Earth Sciences	79(pt.B)	777–791
1927	HSG09	CAOB	SHB	118.9686	44.0567	Granite	Haisugou	138	150			0.1362	0.512623	2	-0.31	-0.29	0.77	0.87	0.87	0.87	1.04	0.87	1.04	0.87	Shu QH et al.	2014	Journal of Asian Earth Sciences	79(pt.B)	777–791
1928	HSG03	CAOB	SHB	118.9686	44.0567	Granite	Haisugou	138	150			0.1199	0.512609	2	-0.39	-0.57	0.79	0.90	0.90	0.87	0.88	0.87	0.88	0.87	Shu QH et al.	2014	Journal of Asian Earth Sciences	79(pt.B)	777–791
1929	HSG02	CAOB	SHB	118.9686	44.0567	Granite	Haisugou	138	150			0.1281	0.512633	2	-0.35	-0.10	1.10	1.22	1.22	0.84	0.92	0.84	0.92	0.84	Shu QH et al.	2014	Journal of Asian Earth Sciences	79(pt.B)	777–791
1930	HSG01	CAOB	SHB	118.9686	44.0567	Granite	Haisugou	138	150			0.1272	0.512657	5	-0.35	0.37	1.59	1.70	1.70	0.80	0.87	0.80	0.87	0.80	Shu QH et al.	2014	Journal of Asian Earth Sciences	79(pt.B)	777–791
1931	SK77	CAOB	NE China	125.5000	50.5000	Granodiorite		188	188	3.27	18.20	0.1086	0.512739		-0.45	1.97	4.00	4.08	4.08	0.64	0.60	0.64	0.597	0.64	Deng K et al.	2018	Lithos	296-299	113-128
1932	SK76	CAOB	NE China	125.5000	50.5000	Granodiorite		188	188	4.06	20.70	0.1185	0.512731		-0.40	1.81	3.60	3.69	3.69	0.67	0.67	0.67	0.67	0.67	Deng K et al.	2018	Lithos	296-299	113-128
1933	SK75	CAOB	NE China	125.5000	50.5000	Granodiorite		188	188	3.89	21.20	0.1109	0.512731		-0.44	1.81	3.80	3.87	3.87	0.65	0.62	0.65	0.62	0.65	Deng K et al.	2018	Lithos	296-299	113-128
1934	SK74	CAOB	NE China	125.5000	50.5000	Granodiorite		188	188	4.13	20.80	0.1200	0.512741		-0.39	2.01	3.80	3.84	3.84	0.66	0.67	0.66	0.67	0.66	Deng K et al.	2018	Lithos	296-299	113-128
1935	SK73	CAOB	NE China	125.5000	50.5000	Granodiorite		188	188	3.64	19.80	0.1111	0.512736		-0.44	1.91	3.90	3.96	3.96	0.65	0.62	0.65	0.62	0.65	Deng K et al.	2018	Lithos	296-299	113-128
1936	SK48	CAOB	NE China	125.5000	50.5000	Granodiorite		188	188	4.34	22.30	0.1176	0.512739		-0.40	1.97	3.80	3.86	3.86	0.65	0.65	0.65	0.65	0.66	Deng K et al.	2018	Lithos	296-299	113-128
1937	SK46	CAOB	NE China	125.5000	50.5000	Granodiorite		188	188	4.35	23.00	0.1143	0.512750		-0.42	2.18	4.00	4.16	4.16	0.63	0.61	0.63	0.62	0.63	Deng K et al.	2018	Lithos	296-299	113-128
1938	SK45	CAOB	NE China	125.5000	50.5000	Granodiorite		188	188	3.49	19.70	0.1071	0.512736		-0.46	1.91	3.90	4.06	4.06	0.64	0.59	0.64	0.59	0.64	Deng K et al.	2018	Lithos	296-299	113-128
1939	14SNT-18	CAOB	Xing-Meng orogenic belt	112.0603	43.9751	Syenogranite		317	317	12.08	55.40	0.1318	0.512728	4	-0.33	1.76	4.40	4.38	4.38	0.72	0.79	0.72	0.80	0.72	Kong LJ et al.	2017	Acta Petrologica et Mineralogica	36(4)	433-457
1940	14SNT-17	CAOB	Xing-Meng orogenic belt	112.0603	43.9751	Syenogranite		317	317	3.39	14.50	0.1413	0.512724	3	-0.28	1.68	3.90	3.92	3.92	0.76	0.90	0.76	0.90	0.76	Kong LJ et al.	2017	Acta Petrologica et Mineralogica	36(4)	433-457
1941	14SNT-16	CAOB	Xing-Meng orogenic belt	112.0603	43.9751	Syenogranite		317	317	11.50	55.00	0.1264	0.512738	7	-0.36	1.95	4.80	4.79	4.79	0.68	0.72	0.68	0.70	0.68	Kong LJ et al.	2017	Acta Petrologica et Mineralogica	36(4)	433-457
1942	14SNT-15	CAOB	Xing-Meng orogenic belt	112.0603	43.9751	Syenogranite		317	317	7.35	33.80	0.1315	0.512730	6	-0.33	1.79	4.40	4.43	4.43	0.71	0.78	0.71	0.80	0.71	Kong LJ et al.	2017	Acta Petrologica et Mineralogica	36(4)	433-457
1943	NM10-132	NCC	NNCC	110.4500	41.7750	Andesite	Suji	276	276	3.68	19.40	0.1147	0.511890		-0.42	-14.59	-11.8	-11.7	-11.7	1.99	1.93	1.99	1.93		Chen C et al.	2015	Journal of Asian Earth Sciences	97	307-319
1944	NM10-131	NCC	NNCC	110.4500	41.7750	Andesite	Suji	276	276	4.60	23.60	0.1178	0.511988		-0.40	-12.68	-10.00	-9.91	-9.91	1.85	1.84	1.85	1.84		Chen C et al.	2015	Journal of Asian Earth Sciences	97	307-319
1945	NM10-130	NCC	NNCC	110.4500	41.7750	Dacite	Suji	276	276	5.20	28.30	0.1111	0.511986		-0.44	-12.72	-9.80	-9.71	-9.71	1.83	1.72	1.83	1.73		Chen C et al.	2015	Journal of Asian Earth Sciences	97	307-319
1946	NM08-058	NCC	NNCC	110.4500	41.7750	Dacite	Suji	276	276	6.29	47.50	0.0801	0.511947		-0.59	-13.48	-9.50	-9.38	-9.38	1.81	1.37	1.81	1.37		Chen C et al.	2015	Journal of Asian Earth Sciences	97	307-319
1947	NM08-046	NCC	NNCC	110.4500	41.7750	Rhyolite	Suji	276	276	6.98	48.20	0.0876	0.511927		-0.55	-13.87	-10.1	-10.0	-10.0	1.86	1.48	1.86	1.48		Chen C et al.	2015	Journal of Asian Earth Sciences	97	307-319
1948	NM08-042	NCC	NNCC	110.4500	41.7750	Rhyolite	Suji	276	276	7.07	48.10	0.0887	0.511930		-0.55	-13.81	-10.1	-10.0	-10.0	1.86	1.49	1.86	1.49		Chen C et al.	2015	Journal of Asian Earth Sciences	97	307-319
1949	NM08-038	NCC	NNCC	110.4500	41.7750	Rhyolite	Suji	276	276	6.85	47.30	0.0875	0.511920		-0.56	-14.01	-10.3	-10.2	-10.2	1.87	1.48	1.87	0.60		Chen C et al.	2015	Journal of Asian Earth Sciences	97	307-319
1950	HDB2-4	CAOB	SMXAB	125.7869	50.2467	Granodiorite	Duobaoshan	479	479	2.57	13.50	0.1152	0.512662	7	-0.41	0.47	5.47	5.45	5.45	0.76	0.76	0.76	0.76	0.77	Wu G et al.	2015	Journal of Asian Earth Sciences	97	229-250
1951	HDB2-12	CAOB	SMXAB	125.7869	50.2467	Granodiorite	Duobaoshan	479	479	2.66	14.00	0.1146	0.512657	7	-0.42	0.37	5.39	5.39	5.39	0.77	0.76	0.77	0.76	0.77	Wu G et al.	2015	Journal of Asian Earth Sciences	97	229-250
1952	HDB2-10	CAOB	SMXAB	125.7869	50.2467	Granodiorite	Duobaoshan	479	479	2.52	13.30	0.1142	0.512654	7	-0.42	0.31	5.37	5.35	5.35	0.77	0.76	0.77	0.76	0.77	Wu G et al.	2015	Journal of Asian Earth Sciences	97	229-250
1953	HDB2-1	CAOB	SMXAB	125.7869	50.2467	Granodiorite	Duobaoshan	479	479	2.64	14.00	0.1142	0.512649	14	-0.42	0.21	5.28	5.26	5.26	0.78	0.77	0.78	0.77	0.78	Wu G et al.	2015	Journal of Asian Earth Sciences	97	229-250
1954	B70823-4	CAOB	Beishan	95.0346	41.1384	Pyroxenite	Shijinpo	400	400	0.08	0.18	0.2728	0.513179	14	0.39	10.55	6.67	6.67	6.67	0.60	0.08	0.60	0.08	0.46	Li S et al.	2012	Lithos	134-135	123-145
1955	B70823-2	CAOB	Beishan	95.0182	41.1784	(phenocrysts are mostly p	Shijinpo	400	400	6.15	28.49	0.1306	0.512352	10	-0.34	-5.58	-2.21	-2.21	-2.21	1.32	1.46	1.32	1.46	1.27	Li S et al.	2012	Lithos	134-135	123-145
1956	B70820-2.1	CAOB	Beishan	97.5339	40.8758	Gray black gneissic granite	Jinmiaogou	270	270	4.11	21.79	0.1141	0.512649	10	-0.42	0.21	3.06	3.06	3.06	0.79	0.77	0.79	0.77	0.80	Li S et al.	2012	Lithos	134-135	123-145
1957	B70820-12.1	CAOB	Beishan	97.0695	40.6564	Coarse grained Bi granite	Yinmachang	400	400	0.76	5.18	0.0885	0.511924	10	-0.55	-13.93	-8.41	-8.41	-8.41	1.83	1.49	1.83	1.49	1.95	Li S et al.	2012	Lithos	134-135	123-145
1958	B70820-11	CAOB	Beishan	97.3204	40.7936	Gneissic granite	Bantan	1400	600	12.65	58.24	0.1314	0.512070	7	-0.33	-11.08	0.59	-6.08	-6.08	1.80	1.99	1.80	1.99	1.71	Li S et al.	2012	Lithos	134-135	123-145
1959	B70818-9.1	CAOB	Beishan	96.8815	41.1356	arse grained K-feldspar gra	Yinouxia	280	280	3.75	19.84	0.1144	0.512464	5	-0.42	-3.39	-0.45	-0.45	-0.45	1.08	1.05	1.08	1.05	1.09	Li S et al.	2012	Lithos	134-135	123-145
1960	B70818-5.2	CAOB	Beishan	96.9387	41.2651	coarse-grained potash feld	Tongchangkou	280	280	5.80	29.07	0.1206	0.512336	8	-0.39	-5.89	-3.17	-3.17	-3.17	1.30	1.33	1.30	1.33	1.30	Li S et al.	2012	Lithos	134-135	123-145
1961	B70817-8.2	CAOB	Beishan	96.9848	41.5324	Granodiorite	Tongchangkou	280	280	2.65	13.48	0.1191	0.512448	5	-0.39	-3.71	-0.93	-0.93	-0.93	1.12	1.13	1.12	1.13	1.12	Li S et al.	2012	Lithos	134-135	123-145
1962	B70815-9.2	CAOB	Beishan	97.5967	43.0987	Monzogranite	Chaganchunzi	280	280	5.02	23.31	0.1303	0.512336	10	-0.34	-5.89	-3.52	-3.52	-3.52	1.33	1.49	1.33	1.49	1.29	Li S et al.	2012	Lithos	134-135	123-145

1963	B70815-9.1	CAOB	Beishan	96.5801	42.0821	Gneissic granite	Chaganchunzi	430	430	3.54	18.03	0.1186	0.512290	7	-0.40	-6.79	-2.50	-2.50	-2.50	1.37	1.38	1.37	1.38	1.37	Li S et al.	2012	Lithos	134-135	123-145
1964	B70815-4.8	CAOB	Beishan	96.3459	42.0106	Fine pyroxenite	quanshuijingxi	300	300	3.70	13.95	0.1607	0.512644	5	-0.18	0.12	1.50	1.50	1.50	0.94	1.45	0.94	1.45	0.80	Li S et al.	2012	Lithos	134-135	123-145
1965	B70815-11.1	CAOB	Beishan	96.7682	41.8717	Plagioclase granite	Tiaohunan	280	280	5.47	27.75	0.1193	0.512160	8	-0.39	-9.32	-6.56	-6.56	-6.56	1.58	1.60	1.58	1.60	1.57	Li S et al.	2012	Lithos	134-135	123-145
1966	B70814-4.2	CAOB	Beishan	97.0441	42.1722	Diorite porphyrite	Gongpoquan	270	270	5.86	26.76	0.1325	0.512624	5	-0.33	-0.27	1.94	1.94	1.94	0.88	0.99	0.88	0.99	0.84	Li S et al.	2012	Lithos	134-135	123-145
1967	B70814-15	CAOB	Beishan	97.0556	42.1706	Granite gneiss	Shuangjingzi	430	430	3.48	19.46	0.1082	0.512249	5	-0.45	-7.59	-2.73	-2.73	-2.73	1.39	1.30	1.39	1.30	1.43	Li S et al.	2012	Lithos	134-135	123-145
1968	B70814-1	CAOB	Beishan	97.1167	42.1041	Granodiorite	Shuangjingzixi	270	270	5.68	24.94	0.1378	0.512206	15	-0.30	-8.43	-6.40	-6.40	-6.40	1.55	1.89	1.55	1.89	1.50	Li S et al.	2012	Lithos	134-135	123-145
1969		CAOB	Beishan	89.9412	47.3387	Granite		210	210	8.56	49.44	0.1055	0.512387		-0.46	-4.90	-4.90	-2.45	-2.45	1.19	1.07	1.19	1.07	1.19	Wang T et al.	2014	American Journal of Science	314 (1)	1-42
1970	3037	CAOB	Beishan	89.9412	47.3387	Granite		210	210	6.08	27.61	0.1331	0.512368		-0.32	-5.27	-5.30	-3.56	-3.56	1.28	1.48	1.28	1.48	1.28	Wang T et al.	2014	American Journal of Science	314 (1)	1-42
1971	3013	CAOB	Beishan	89.6464	47.5488	Granite		210	210	10.30	60.60	0.1025	0.512464		-0.48	-3.39	-3.40	-0.87	-0.87	1.06	0.94	1.06	0.94	1.06	Wang T et al.	2014	American Journal of Science	314 (1)	1-42
1972	MSD09821-6.1	CAOB	Beishan	94.8696	41.1456	Granite		224	224	4.11	21.58	0.1152	0.512475		-0.41	-3.18	–0.41	-0.85	-0.85	1.07	1.04	1.07	1.04	1.07	Li S et al.	2012	Lithos	134-135	123-145
1973	MSD09821-4.1	CAOB	Beishan	94.8215	41.1273	Granite		224	224	10.65	59.85	0.1076	0.512365		-0.45	-5.33	–0.45	-2.78	-2.78	1.23	1.13	1.23	1.13	1.23	Li S et al.	2012	Lithos	134-135	123-145
1974	HNS09823-2.2	CAOB	Beishan	95.5280	41.1976	Granite		217	217	10.43	68.85	0.0916	0.512413		-0.53	-4.39	–0.53	-1.48	-1.48	1.11	0.92	1.11	0.92	1.11	Li S et al.	2012	Lithos	134-135	123-145
1975	DQ09819-9.2	CAOB	Beishan	95.3610	41.2492	Granite		225	225	14.04	102.12	0.0832	0.512391		-0.58	-4.82	–0.58	-1.56	-1.56	1.13	0.89	1.13	0.89	1.13	Li S et al.	2012	Lithos	134-135	123-145
1976	DQ09819-8	CAOB	Beishan	95.3499	41.2778	Granite		225	225	7.28	46.80	0.0941	0.512411		-0.52	-4.43	–0.52	-1.48	-1.48	1.12	0.94	1.12	0.94	1.12	Li S et al.	2012	Lithos	134-135	123-145
1977	DQ09819-4.2	CAOB	Beishan	95.3718	41.3031	Granite		221	221	12.14	66.11	0.1110	0.512437		-0.44	-3.92	–0.44	-1.50	-1.50	1.12	1.06	1.12	1.06	1.12	Li S et al.	2012	Lithos	134-135	123-145
1978	B80628-3	CAOB	Beishan	95.7793	41.2511	Granite		221	221	16.32	95.39	0.1034	0.512394		-0.47	-4.76	–0.47	-2.13	-2.13	1.17	1.04	1.17	1.04	1.17	Li S et al.	2012	Lithos	134-135	123-145
1979	B80625-6	CAOB	Beishan	95.3577	41.3103	Granite		221	221	20.10	119.38	0.1018	0.512433		-0.48	-4.00	–0.48	-1.32	-1.32	1.10	0.98	1.10	0.98	1.11	Li S et al.	2012	Lithos	134-135	123-145
1980	B80624-5.1	CAOB	Beishan	95.6472	41.2196	Granite		221	221	5.34	37.33	0.0866	0.512389		-0.56	-4.86	–0.56	-1.75	-1.75	1.14	0.91	1.14	0.91	1.14	Li S et al.	2012	Lithos	134-135	123-145
1981	B70822-4	CAOB	Beishan	95.5283	41.2075	Granite		221	221	6.62	45.98	0.0871	0.512405		-0.56	-4.55	–0.56	-1.45	-1.45	1.12	0.90	1.12	0.90	1.12	Li S et al.	2012	Lithos	134-135	123-145
1982	B70817-2.1	CAOB	Beishan	96.7182	41.4539	Granodiorite	Dahulujing	238	375	6.78	39.09	0.1049	0.512440	5	-0.47	-3.86	–0.47	0.53	0.53	1.08	0.99	1.08	0.99	1.10	Li S et al.	2012	Lithos	134-135	123-145
1983	EK-1	CAOB	East Kounrad mineral deposit	75.1356	47.0000	Leucogranite	East Kounrad mineral deposi	298	298	1.88	13.75	0.0828	0.512559	13	-0.58	-1.54	2.80	2.79	2.79	0.83	0.69	0.83		0.84	Li et al.	2016	Lithos	252-253	16-31
1984	Zh-5	CAOB	Zhanet mineral deposit	74.3154	47.5213	Granite porphyry	Zhanet mineral deposit	309	309	3.87	24.87	0.0941	0.512476	11	-0.52	-3.16	0.90	0.88	0.88	1.00	0.86	1.00		1.00	Li et al.	2016	Lithos	252-253	16-31
1985	Zh-2-10	CAOB	Zhanet mineral deposit	74.3154	47.5213	Granite porphyry	Zhanet mineral deposit	309	309	2.53	15.93	0.0960	0.512469	1	-0.51	-3.30	0.70	0.67	0.67	1.01	0.88	1.01		1.02	Li et al.	2016	Lithos	252-253	16-31
1986	Zh-1-1	CAOB	Zhanet mineral deposit	74.3154	47.5213	Aplite	Zhanet mineral deposit	285	285	1.64	14.64	0.0678	0.512480	17	-0.66	-3.08	1.60	1.61	1.61	0.92	0.70	0.92		0.92	Li et al.	2016	Lithos	252-253	16-31
1987	EK-4-2	CAOB	East Kounrad mineral deposit	75.1356	47.0000	Aplite	East Kounrad mineral deposi	297	297	0.43	3.98	0.0662	0.512453	12	-0.66	-3.61	1.30	1.33	1.33	0.95	0.72	0.95		0.95	Li et al.	2016	Lithos	252-253	16-31
1988	EK-4-1	CAOB	East Kounrad mineral deposit	75.1356	47.0000	Aplite	East Kounrad mineral deposi	297	297	2.72	17.32	0.0951	0.512535	11	-0.52	-2.01	1.80	1.84	1.84	0.91	0.79	0.91		0.91	Li et al.	2016	Lithos	252-253	16-31
1989	Ash-5	CAOB	Akshatau mineral deposit	74.0429	47.9949	Aplite	Akshatau mineral deposit	294	294	1.89	15.57	0.0735	0.512427	15	-0.63	-4.12	0.50	0.51	0.51	1.02	0.79	1.02		1.02	Li et al.	2016	Lithos	252-253	16-31
1990	Ash-2	CAOB	Akshatau mineral deposit	74.0429	47.9949	Aplite	Akshatau mineral deposit	294	294	1.68	12.10	0.0838	0.512462	13	-0.57	-3.43	0.80	0.80	0.80	0.99	0.81	0.99		0.99	Li et al.	2016	Lithos	252-253	16-31
1991	Ash-1	CAOB	Akshatau mineral deposit	74.0429	47.9949	Granite	Akshatau mineral deposit	320	320	3.04	17.69	0.1039	0.512417	14	-0.47	-4.31	-0.50	-0.53	-0.53	1.12	1.02	1.12		1.12	Li et al.	2016	Lithos	252-253	16-31
1992	HM2-6	CAOB	Altai, unit 2	87.5769	48.6905	-grained two-mica monzon	Youyifeng	393	393	13.00	56.51	0.1396	0.512440	13	-0.29	-3.86	-1.00	-1.00	-1.00	1.22	1.46	1.22	0.72	1.33	Cai KD et al.	2011	Lithos	127(1-2)	261-281
1993	HM2-1	CAOB	Altai, unit 2	87.5769	48.6905	-grained two-mica monzon	Youyifeng	393	393	9.17	39.67	0.1407	0.512445	12	-0.28	-3.76	-1.00	-0.96	-0.96	1.21	1.47	1.21	0.72	1.33	Cai KD et al.	2011	Lithos	127(1-2)	261-281
1994	HM1-3	CAOB	Altai, unit 2	87.5769	48.6905	-grained two-mica monzon	Youyifeng	393	393	10.00	47.11	0.1297	0.512422	13	-0.34	-4.21	-0.86	-0.85	-0.85	1.21	1.32	1.21	0.72	1.32	Cai KD et al.	2011	Lithos	127(1-2)	261-281
1995	HM12	CAOB	Altai, unit 2	87.5989	48.5986	Leucocratic granite	Hemu	412	412	8.02	35.96	0.1357	0.512390	8	-0.31	-4.84	-1.80	-1.63	-1.63	1.28	1.48	1.28	0.72	1.40	Cai KD et al.	2011	Lithos	127(1-2)	261-281
1996	HM02	CAOB	Altai, unit 2	87.5989	48.5986	Leucocratic granite	Hemu	412	412	5.84	24.79	0.1434	0.512333	9	-0.27	-5.95	-3.30	-3.15	-3.15	1.41	1.77	1.41	0.72	1.51	Cai KD et al.	2011	Lithos	127(1-2)	261-281
1997	YQ-45	CAOB	Altai, unit 5	90.3586	46.4944	Monzogranite	Areletuobie	300	300	7.77	35.84	0.1310	0.512642	9	-0.33	0.08	2.59	2.59	2.59	0.85	0.94	0.85	0.94	0.85	Zhou G.	2007	dissertation of Chinese Academy of Geological Sciences		
1998	YQ-44	CAOB	Altai, unit 5	90.3586	46.4944	Monzogranite	Areletuobie	300	300	10.60	52.31	0.1225	0.512654	12	-0.38	0.31	3.15	3.15	3.15	0.80	0.83	0.80	0.83	0.80	Zhou G.	2007	dissertation of Chinese Academy of Geological Sciences		
1999	BS-1505-9	CAOB	Tianshan	95.9333	42.4750	Monzogranite	Baishan	228	228	1.65	8.38	0.1190	0.512632		-0.40	-0.12	2.10	2.14	2.14	0.83	0.83	0.83	0.84	0.83	Wang YH et al.	2017	Mineralium Deposita	268-271	32-47
2000	BS-1505-31	CAOB	Tianshan	95.9333	42.4750	Monzogranite	Baishan	228	228	2.62	16.50	0.0960	0.512570		-0.51	-1.33	1.60	1.60	1.60	0.87	0.75	0.87	0.76	0.88	Wang YH et al.	2017	Mineralium Deposita	268-271	32-47
2001	BS-3102-3	CAOB	Tianshan	95.9333	42.4750	Monzogranite	Baishan	228	228	2.62	14.40	0.1100	0.512628		-0.44	-0.20	2.30	2.33	2.33	0.81	0.77	0.81	0.77	0.82	Wang YH et al.	2017	Mineralium Deposita	268-271	32-47
2002	BS-3102-17	CAOB	Tianshan	95.9333	42.4750	Monzogranite	Baishan	227	227	2.65	16.40	0.0977	0.512673		-0.50	0.68	3.60	3.55	3.55	0.71	0.63	0.71	0.63	0.72	Wang YH et al.	2017	Mineralium Deposita	268-271	32-47
2003	BS-1505-15	CAOB	Tianshan	95.9333	42.4750	Granite	Baishan	227	227	2.98	19.20	0.0938	0.512597		-0.52	-0.80	2.20	2.18	2.18	0.82	0.70	0.82	0.71	0.83	Wang YH et al.	2017	Mineralium Deposita	268-271	32-47
2004	BS-1505-30	CAOB	Tianshan	95.9333	42.4750	Granite	Baishan	227	227	2.26	14.40	0.0949	0.512585		-0.52	-1.03	1.90	1.91	1.91	0.85	0.73	0.85	0.73	0.85	Wang YH et al.	2017	Mineralium Deposita	268-271	32-47
2005	BS-1505-32	CAOB	Tianshan	95.9333	42.4750	Granite	Baishan	227	227	2.55	15.90	0.0970	0.512635		-0.51	-0.06	2.80	2.83	2.83	0.77	0.67	0.77	0.68	0.78	Wang YH et al.	2017	Mineralium Deposita	268-271	32-47
2006	J-137	CAOB	Baogutu, West Junggar	84.2500	45.5083	Qtz monzodiorite porphyrite	Dike	283	283	2.23	16.96	0.1312	0.513002	33	-0.33	7.10	9.47	9.47	9.47	0.23	0.27	0.28	0.27	0.28	Qi JY.	1993	Acta Petrologica Sinica	9(3)	288-299
2007	9-200	CAOB	Baogutu, West Junggar	84.2500	45.6750	e crystalline diorite porphy	Dike	283	283	2.07	13.74	0.1508	0.512948	48	-0.23	6.05	7.70	7.70	7.70	0.42	0.49	0.42	0.49	0.42	Qi JY.	1993	Acta Petrologica Sinica	9(3)	288-299
2008	KP49	CAOB	NW China	86.0000	47.0000	Andesite		340	340	4.79	25.90	0.1120	0.512815		-0.43	3.45	3.50	7.13	7.13	0.51	0.50	0.51			Shen P et al.	2016	Journal of Asian Earth Sciences	39	1-21
2009	KP14	CAOB	NW China	86.0000	47.0000	Andesite		340	340	2.70	13.40	0.1232	0.512849		-0.37	4.12	4.10	7.30	7.30	0.50	0.51	0.50			Shen P et al.	2016	Journal of Asian Earth Sciences	39	1-21
2010	KLA12	CAOB	NW China	86.0000	47.0000	Andesite		340	340	4.70	22.90	0.1240	0.512825		-0.37	3.65	3.60	6.80	6.80	0.54	0.55	0.54			Shen P et al.	2016	Journal of Asian Earth Sciences	39	1-21
2011	KE12	CAOB	Altay	86.0000	47.0000	Basaltic andesite	Sawur	340	340	3.7	19.5	0.1158	0.512845		-0.41	4.04	4.00	7.55	7.60	0.48	0.48	0.48			Shen P et al.	2008	Journal of Asian Earth Sciences	32(2-4)	259-279
2012	ZK125-380	CAOB	Altay	86.0000	47.0000	Basaltic andesite	Sawur	340	340	5.2	24.3	0.1314	0.512882		-0.33	4.76	4.70	7.59	7.69	0.48	0.50	0.48			Shen P et al.	2008	Journal of Asian Earth Sciences	32(2-4)	259-279
2013	ZK125-450	CAOB	Altay	86.0000	47.0000	Basaltic andesite	Sawur	340	340	5.2	24.8	0.1269	0.512865		-0.35	4.43	4.40	7.46	7.47	0.49	0.50	0.49			Shen P et al.	2008	Journal of Asian Earth Sciences	32(2-4)	259-279
2014	KP326	CAOB	Altay	86.0000	47.0000	Basaltic andesite	Sawur	340	340	4.6	23	0.1230	0.512856		-0.37	4.25	4.20	7.45	7.55	0.49	0.49	0.49			Shen P et al.	2008	Journal of Asian Earth Sciences	32(2-4)	259-279
2015	KP9	CAOB	Altay	86.0000	47.0000	Basaltic andesite	Sawur	340	340	3.3	16	0.1266	0.512857		-0.36	4.27	4.20	7.31	7.41	0.50	0.51	0.50			Shen P et al.	2008	Journal of Asian Earth Sciences	32(2-4)	259-279
2016	KP6	CAOB	Altay	86.0000	47.0000	Basaltic andesite	Sawur	340	340	3.1	13.9	0.1364	0.512886		-0.31	4.84	4.80	7.45	7.53	0.49	0.52	0.49			Shen P et al.	2008	Journal of Asian Earth Sciences	32(2-4)	259-279
2017	KP232	CAOB	Altay	86.0000	47.0000	Basaltic andesite	Sawur	340	340	3.6	16.9	0.1314	0.512848		-0.33	4.10	4.10	6.93	7.05	0.53	0.56	0.53			Shen P et al.	2008	Journal of Asian Earth Sciences	32(2-4)	259-279
2018	KP325-1	CAOB	Altay	86.0000	47.0000	Basaltic andesite	Sawur	340	340	5.1	23.7	0.1306	0.512849		-0.34	4.12	4.10	6.98	7.02	0.52	0.55	0.52			Shen P et al.	2008	Journal of Asian Earth Sciences	32(2-4)	259-279
2019	KL78	CAOB	Altay	86.0000	47.0000	Basaltic andesite	Sawur	340	340	3.348	16.2	0.1251	0.512816		-0.36	3.47	3.50	6.58	6.59	0.56	0.58	0.56			Shen P et al.	2008	Journal of Asian Earth Sciences	32(2-4)	259-279
2020	KP231	CAOB	Altay	86.0000	47.0000	Basaltic andesite	Sawur	340	340	4.1	18.7	0.1325	0.512838		-0.33	3.90	3.90	6.69	6.69	0.55	0.59	0.55			Shen P et al.	2008	Journal of Asian Earth Sciences	32(2-4)	259-279
2021	LLK-7	CAOB	Northern margin of Yili block	82.9000	44.1000	Granodiorite porphyry		354	354			0.1266	0.512441		-0.36	-3.84	-0.67	-0.68	-0.68	1.16	1.24	1.16	1.24	1.17	Xue CJ et al.	2011	ers (China Uni versit y of Geosci ences(Beijin	18(1)	149-165
2022	LLK-13	CAOB	Northern margin of Yili block	82.9000	44.1000	Granodiorite porphyry		354	354			0.1185	0.512425		-0.40	-4.15	-0.62	-0.62	-0.62	1.16	1.16	1.16	1.16	1.16	Xue CJ et al.	2011	ers (China University of Geosciences(Beijing	18(1)	149-165
2023	LLK-1	CAOB	Northern margin of Yili block	82.9000	44.1000	Granodiorite porphyry		354	354			0.1152	0.512300		-0.41	-6.59	-2.91	-2.91	-2.91	1.34	1.31	1.34	1.32	1.35	Xue CJ et al.	2011	ers (China University of Geosciences(Beijing	18(1)	149-165
2024	D9-02	CAOB	Northern margin of Yili block	82.9000	44.1000	Granodiorite porphyry		346	346			0.1155	0.512335		-0.41	-5.91	-2.32	-2.33	-2.33	1.29	1.26	1.29	1.27	1.30	Xue CJ et al.	2011	ers (China University of Geosciences(Beijing	18(1)	149-165
2025	D8-01	CAOB	Northern margin of Yili block	82.9000	44.1000	Granodiorite porphyry		346	346			0.1262	0.512330		-0.36	-6.01	-2.90	-2.90	-2.90	1.33	1.43	1.33	1.43	1.34	Xue CJ et al.	2011	ers (China University of Geosciences(Beijing	18(1)	149-165
2026	D7-02	CAOB	Northern margin of Yili block	82.9000	44.1000	Granodiorite porphyry		346	346			0.1248	0.512360		-0.37	-5.42	-2.25	-2.25	-2.25	1.28	1.35	1.28	1.35	1.29	Xue CJ et al.	2011	ers (China University of Geosciences(Beijing	18(1)	149-165
2027	D3-01	CAOB	Northern margin of Yili block	82.9000	44.1000	Granodiorite porphyry		346	346			0.1269	0.512290		-0.35	-6.79	-3.71	-3.71	-3.71	1.40	1.51	1.40	1.51	1.41	Xue CJ et al.	2011	ers (China University of Geosciences(Beijing	18(1)	149-165
2028	D14-01	CAOB	Northern margin of Yili block	82.9000	44.1000	Granodiorite porphyry		346	346			0.1190	0.512382		-0.40	-4.99	-1.56	-1.56	-1.56	1.23	1.24	1.23	1.24	1.23	Xue CJ et al.	2011	ers (China University of Geosciences(Beijing	18(1)	149-165
2029	D12-01	CAOB	Northern margin of Yili block	82.9000	44.1000	Granodiorite porphyry		346	346			0.1364	0.512470		-0.31	-3.28	-0.61	-0.61	-0.61	1.15	1.34	1.15	1.34	1.15	Xue CJ et al.	2011	ers (China University of Geosciences(Beijing	18(1)	149-165
2030	KE-18	CAOB	West Junggar	86.3000	47.1833	Granite porphyry	Kaerjia	302	302	8.60	42.84	0.1215	0.512794	0	-0.38	3.04	5.94	5.94	5.94	0.58	0.59	0.58	0.59	0.58	Zhou ZH et al.	2012	Lithos	106(3-4)	191-206
2031	GTS09-04	NCC	NCC	118.4514	41.2875	Peralkaline granite	Guangtoushan	208	208			0.0847	0.512063	12	-0.57	-11.22	-8.24	-8.24	-8.24	1.66	1.28	1.66	1.29	1.66	Zhang Z et al.	2012	Lithos	149	174-187
2032	GTS09-03	NCC	NCC	118.4514	41.2875	Porphyritic granite	Guangtoushan	250	250			0.0883	0.511863	13	-0.55	-15.12	-11.7	-11.7	-11.7	1.97	1.56	1.97	1.56	1.97	Zhang Z et al.	2012	Lithos	149	174-187
2033	GTS09-01	NCC	NCC	118.4514	41.2875	Porphyritic granite	Guangtoushan	250	250			0.0857	0.511835	11	-0.56	-15.66	-12.1	-12.1	-12.1	2.01	1.56	2.01	1.57	2.01	Zhang Z et al.	2012	Lithos	149	174-187
2034	CHY-4	CAOB	NE China	129.0000	48.5000	Biotite syenogranite	Cuihongshan	502	502	9.16	52.30	0.1059	0.512354		-0.46	-5.54	0.28	0.28	0.28	1.20	1.12	1.20	1.13	1.21	Fei XH et al.	2018	Lithos	302-303	158-117
2035	CHY-7	CAOB	NE China	129.0000	48.5000	Biotite syenogranite	Cuihongshan	502	502	9.67	51.80	0.1128	0.512092		-0.43	-10.65	-5.29	-5.28	-5.28	1.65	1.60	1.65	1.60	1.66	Fei XH et al.	2018	Lithos	302-303	158-117
2036	CHY-10	CAOB	NE China	129.0000	48.5000	Biotite syenogranite	Cuihongshan	502	502	7.40	39.90	0.1121	0.512065		-0.43	-11.18	-5.76	-5.76	-5.76	1.69	1.62	1.69	1.63	1.70	Fei XH et al.	2018	Lithos	302-303	158-117
2037	CHY-18	CAOB	NE China	129.0000	48.5000	Biotite syenogranite	Cuihongshan	502	502	11.90	65.40	0.1100	0.512036		-0.44	-11.74	-6.19	-6.19	-6.19	1.73	1.63	1.73	1.64	1.73	Fei XH et al.	2018	Lithos	302-303	158-117
2038	CHY-2	CAOB	NE China	129.0000	48.5000	Porphyritic quartz monzonit	Cuihongshan	197	197	8.27	46.10	0.1084	0.512369		-0.45	-5.25	-3.04	-3.03	-3.03	1.22	1.13	1.22	1.13	1.23	Fei XH et al.	2018	Lithos	302-303	158-117
2039	CHY-14	CAOB	NE China	129.0000	48.5000	Porphyritic quartz monzonit	Cuihongshan	197	197	7.92	43.70	0.1096	0.512366		-0.44	-5.31	-3.13	-3.12	-3.12	1.23	1.15	1.23	1.15	1.23	Fei XH et al.	2018	Lithos	302-303	158-117
2040	CHY-19	CAOB	NE China	129.0000	48.5000	Porphyritic quartz monzonit	Cuihongshan	197	197	7.89	43.60	0.1094	0.512415		-0.44	-4.35	-2.16	-2.16	-2.16	1.15	1.07	1.15	1.08	1.15	Fei XH et al.	2018	Lithos	302-303	158-117
2041	CHY-20	CAOB	NE China	129.0000	48.5000	Porphyritic quartz monzonit	Cuihongshan	197	197	7.75	41.90	0.1118	0.512342		-0.43	-5.77	-3.66	-3.65	-3.65	1.27	1.21	1.27	1.21	1.28	Fei XH et al.	2018	Lithos	302-303	158-117
2042	CHY-21	CAOB	NE China	129.0000	48.5000	Porphyritic quartz monzonit	Cuihongshan	197	197	8.33	46.40	0.1085	0.512331		-0.45	-5.99	-3.78	-3.78	-3.78	1.28	1.19	1.28	1.19	1.29	Fei XH et al.	2018	Lithos	302-303	158-117
2043	CHYX-1	CAOB	NE China	129.0000	48.5000	Monzogranite	Cuihongshan	207	207	5.48	31.00	0.1069	0.512489		-0.46	-2.91	-0.54	-0.54	-0.54	1.03	0.94	1.03	0.94	1.03	Fei XH et al.	2018	Lithos	302-303	158-117
2044	CHYX-2	CAOB	NE China	129.0000	48.5000	Monzogranite	Cuihongshan	207	207	7.29	46.10	0.0956	0.512069		-0.51	-11.10	-8.43	-8.43	-8.43	1.67	1.39	1.67	1.39	1.67	Fei XH et al.	2018	Lithos	302-303	158-117
2045	CHYX-3	CAOB	NE China	129.0000	48.5000	Monzogranite	Cuihongshan	207	207	5.90	33.70	0.1058	0.512394		-0.46	-4.76	-2.36	-2.36	-2.36	1.18	1.07	1.18	1.07	1.18	Fei XH et al.	2018	Lithos	302-303	158-117
2046	CHYX-4	CAOB	NE China	129.0000	48.5000	Monzogranite	Cuihongshan	207	207	6.78	38.00	0.1079	0.512433		-0.45	-4.00	-1.66	-1.66	-1.66	1.12	1.03	1.12	1.03	1.12	Fei XH et al.	2018	Lithos	302-303	158-117
2047	CHYX-7	CAOB	NE China	129.0000	48.5000	Monzogranite	Cuihongshan	207	207	5.58	35.20	0.0958	0.512418		-0.51	-4.29	-1.63	-1.63	-1.63	1.12	0.95	1.12	0.95	1.12	Fei XH et al.	2018	Lithos	302-303	158-117
2048	W-7	CAOB	SMXAB	125.6500	52.5600	Granites	Woduhe	130	150	4.51	10.37	0.2627	0.512692	5	0.34	1.05	-0.03	-0.21	-0.21	0.96	-1.44	0.96		0.93	Jahn BM et al.	2001	Lithos	59(4)	171-198
2049	W-6	CAOB	SMXAB	125.6500	52.5600	Granites	Woduhe	130	150	4.19	9.35	0.2708	0.512706	5	0.38	1.33	0.10	-0.09	-0.09	0.95	-1.19	0.95		0.92	Jahn BM et al.	2001	Lithos	59(4)	171-198
2050	W-5	CAOB	SMXAB	125.6500	52.5600	Granites	Woduhe	130	150	3.71	8.11	0.2762	0.512702	6	0.40	1.25	-0.07	-0.27	-0.27	0.97	-1.10	0.97		0.93	Jahn BM et al.	2001	Lithos	59(4)	171-198
2051	W-4	CAOB	SMXAB	125.6500	52.5600	Granites	Woduhe	130	150	3.06	6.46	0.2860	0.512694	6	0.45	1.09	-0.38	-0.62	-0.62	1.00	-0.97	1.00		0.96	Jahn BM et al.	2001	Lithos	59(4)	171-198
2052	W-3	CAOB	SMXAB	125.6500	52.5600	Granites	Woduhe	130	150	2.90	6.47	0.2710	0.512703	5	0.38	1.27	0.03	-0.15	-0.15	0.96	-1.20	0.96		0.92	Jahn BM et al.	2001	Lithos	59(4)	171-198
2053	W-2	CAOB	SMXAB	125.6500	52.5600	Granites	Woduhe	130	150	3.57	7.46	0.2893	0.512719	6	0.47	1.58	0.04	-0.19	-0.19	0.96	-0.87	0.96		0.92	Jahn BM et al.	2001	Lithos	59(4)	171-198
2054	W-1	CAOB	SMXAB	125.6500	52.5600	Granites	Woduhe	130	150	3.02	6.68	0.2737	0.512706	6	0.39	1.33	0.04	-0.15	-0.15	0.96	-1.14	0.96		0.92	Jahn BM et al.	2001	Lithos	59(4)	171-198
2055	Ba-8	CAOB	SMXAB	120.4850	45.4500	Granites	Baerzhe	122	150	172.40	510.20	0.2043	0.512761	18	0.04	2.40	2.28	2.25	2.25	0.74	6.20	0.74	6.28	0.73	Jahn BM et al.	2001	Lithos	59(4)	171-198
2056	Ba-6	CAOB	SMXAB	120.4850	45.4500	Granites	Baerzhe	122	150	169.30	531.60	0.1925	0.512760	11	-0.02	2.38	2.44	2.46	2.46	0.73	2.79	0.73	2.81	0.72	Jahn BM et al.	2001	Lithos	59(4)	171-198
2057	Ba-30	CAOB	SMXAB	120.4850	45.4500	Granites	Baerzhe	122	150	18.70	71.30	0.1586	0.512706	13	-0.19	1.33	1.91	2.06	2.06	0.77	1.23	0.77	1.23	0.76	Jahn BM et al.	2001	Lithos	59(4)	171-198
2058	Ba-22	CAOB	SMXAB	120.4850	45.4500	Granites	Baerzhe	122	150	32.60	120.10	0.1641	0.512724	24	-0.17	1.68	2.19	2.30	2.30	0.75	1.31	0.75	1.31	0.74	Jahn BM et al.	2001	Lithos	59(4)	171-198
2059	Ba-16	CAOB	SMXAB	120.4850	45.4500	Granites	Baerzhe	122	150	64.30	214.00	0.1817	0.512754	10	-0.08	2.26	2.50	2.55	2.55	0.73	1.88	0.73	1.89	0.72	Jahn BM et al.	2001	Lithos	59(4)	171-198
2060	Ba-14	CAOB	SMXAB	120.4850	45.4500	Granites	Baerzhe	122	150	89.00	269.80	0.1994	0.512737	19	0.01	1.93	1.89	1.88	1.88	0.77	4.35	0.77	4.39	0.76	Jahn BM et al.	2001	Lithos	59(4)	171-198

2061	YL-8	CAOB	SMXAB	122.0814	48.5939	Moyite	Yalu	140	150	4.05	88.46	0.0999	0.512608	6	-0.49	-0.59	1.14	1.27	1.27	0.84	0.73	0.84	0.73	0.84	Wu FY et al.	2003	Lithos	67(3-4)	191-204
2062	YL-7	CAOB	SMXAB	122.0814	48.5939	Moyite	Yalu	140	150	4.60	94.18	0.1136	0.512625	6	-0.42	-0.25	1.23	1.34	1.34	0.83	0.80	0.83	0.80	0.83	Wu FY et al.	2003	Lithos	67(3-4)	191-204
2063	S-7	CAOB	SMXAB	123.4725	50.6547	Moyite	Alihe	120	150	2.56	26.65	0.1146	0.512610	36	-0.42	-0.55	0.71	1.03	1.03	0.86	0.83	0.86	0.83	0.86	Wu FY et al.	2003	Lithos	67(3-4)	191-204
2064	9825-14	CAOB	SMXAB	124.9311	52.4861	Qtz diorote	Tafeng	430	430	4.20	22.72	0.1117	0.512227	12	-0.43	-8.02	-3.36	-3.35	-3.35	1.44	1.38	1.44	1.38	1.44	Wu FY et al.	2003	Lithos	67(3-4)	191-204
2065	9825-13	CAOB	SMXAB	124.9311	52.4861	Qtz diorote	Tafeng	430	430	5.46	28.73	0.1149	0.512227	12	-0.42	-8.02	-3.54	-3.53	-3.53	1.45	1.42	1.45	1.43	1.46	Wu FY et al.	2003	Lithos	67(3-4)	191-204
2066	9831-1	CAOB	SMXAB	127.0833	50.8947	Tonalit	Baishilazi	130	150	7.62	44.55	0.1034	0.512547	8	-0.47	-1.78	-0.23	0.01	0.01	0.94	0.83	0.94	0.84	0.94	Wu FY et al.	2003	Lithos	67(3-4)	191-204
2067	9828-1	CAOB	SMXAB	126.2128	50.0064	Adamellite	Xinghua	110	150	5.63	545.90	0.1228	0.512719	18	-0.38	1.58	2.63	2.99	2.99	0.69	0.72	0.69	0.73	0.70	Wu FY et al.	2003	Lithos	67(3-4)	191-204
2068	9825-12	CAOB	SMXAB	124.9311	52.4861	Diorote	Tafeng	430	430	5.21	38.36	0.0821	0.512233	8	-0.58	-7.90	-1.60	-1.61	-1.61	1.30	1.06	1.30	1.06	1.30	Wu FY et al.	2003	Lithos	67(3-4)	191-204
2069	9825-7	CAOB	SMXAB	124.9311	52.4861	Diorote	Tafeng	430	430	4.71	25.13	0.1133	0.512215	7	-0.42	-8.25	-3.67	-3.68	-3.68	1.47	1.42	1.47	1.42	1.47	Wu FY et al.	2003	Lithos	67(3-4)	191-204
2070	9825-6	CAOB	SMXAB	124.9311	52.4861	Diorote	Tafeng	430	430	6.39	29.89	0.1292	0.512308	8	-0.34	-6.44	-2.74	-2.73	-2.73	1.39	1.52	1.39	1.52	1.39	Wu FY et al.	2003	Lithos	67(3-4)	191-204
2071	9825-4	CAOB	SMXAB	124.9311	52.4861	Diorote	Tafeng	430	430	3.53	16.26	0.1311	0.512240	9	-0.33	-7.76	-4.16	-4.16	-4.16	1.50	1.68	1.50	1.68	1.51	Wu FY et al.	2003	Lithos	67(3-4)	191-204
2072	9808-1	CAOB	SMXAB	125.7306	50.3806	Granodiorite	Huaduoshan	170	170	3.63	492.60	0.1088	0.512721	6	-0.45	1.62	3.53	3.53	3.53	0.67	0.62	0.67	0.63	0.67	Wu FY et al.	2003	Lithos	67(3-4)	191-204
2073	9485-3	CAOB	SMXAB	123.4725	50.6547	Moyite	Alihe	120	150	3.94	45.23	0.1249	0.512712	10	-0.37	1.44	2.55	2.82	2.82	0.71	0.75	0.71	0.76	0.71	Wu FY et al.	2003	Lithos	67(3-4)	191-204
2074	9436-3	CAOB	SMXAB	121.7947	48.7875	Tonalite	Boketou	156	156	5.07	710.60	0.1226	0.512587	8	-0.38	-0.99	0.48	0.48	0.48	0.90	0.94	0.90	0.95	0.91	Wu FY et al.	2003	Lithos	67(3-4)	191-204
2075	YE-9	CAOB	NE China	120.5000	47.6667	Granitoid		143	150	2.62	15.07	0.1050	0.512663	8	-0.47	0.49	2.20	2.24	2.24	0.76	0.68	0.76		0.76	Wu F Y et al.	2003	Lithos	67(3-4)	191-204
2076	YE-8	CAOB	NE China	120.5000	47.6667	Granitoid		143	150	2.30	15.34	0.0906	0.512640	7	-0.54	0.04	2.00	2.07	2.07	0.77	0.63	0.77		0.77	Wu F Y et al.	2003	Lithos	67(3-4)	191-204
2077	YE-7	CAOB	NE China	120.5000	47.6667	Granitoid		143	150	0.25	2.06	0.0746	0.512619	7	-0.62	-0.37	1.90	1.97	1.97	0.78	0.58	0.78		0.78	Wu F Y et al.	2003	Lithos	67(3-4)	191-204
2078	YE-6	CAOB	NE China	120.5000	47.6667	Granitoid		143	150	0.24	2.30	0.0636	0.512618	7	-0.68	-0.39	2.00	2.16	2.16	0.76	0.54	0.76		0.77	Wu F Y et al.	2003	Lithos	67(3-4)	191-204
2079	YE-5	CAOB	NE China	120.5000	47.6667	Granitoid		143	150	4.16	23.81	0.1056	0.512677	7	-0.46	0.76	2.40	2.50	2.50	0.73	0.67	0.73		0.74	Wu F Y et al.	2003	Lithos	67(3-4)	191-204
2080	YE-4	CAOB	NE China	120.5000	47.6667	Granitoid		143	150	3.31	20.71	0.0967	0.512660	6	-0.51	0.43	2.30	2.34	2.34	0.75	0.64	0.75		0.75	Wu F Y et al.	2003	Lithos	67(3-4)	191-204
2081	YE-3	CAOB	NE China	120.5000	47.6667	Granitoid		143	150	3.57	22.41	0.0963	0.512670	7	-0.51	0.62	2.50	2.55	2.55	0.73	0.62	0.73		0.73	Wu F Y et al.	2003	Lithos	67(3-4)	191-204
2082	YE-2	CAOB	NE China	120.5000	47.6667	Granitoid		143	150	1.94	13.41	0.0875	0.512649	7	-0.56	0.21	2.20	2.30	2.30	0.75	0.61	0.75		0.75	Wu F Y et al.	2003	Lithos	67(3-4)	191-204
2083	YE-1	CAOB	NE China	122.9167	48.5000	Granitoid		143	150	3.31	19.94	0.1004	0.512642	7	-0.49	0.08	1.80	1.92	1.92	0.78	0.68	0.78		0.78	Wu F Y et al.	2003	Lithos	67(3-4)	191-204
2084	X-7	CAOB	NE China	127.3333	46.6667	Granitoid		173	173	3.17	14.53	0.1317	0.512709	6	-0.33	1.38	2.80	2.82	2.82	0.73	0.82	0.73		0.73	Wu FY et al.	2003	Lithos	67(3-4)	191-204
2085	X-6	CAOB	NE China	127.3333	46.6667	Granitoid		173	173	2.82	13.16	0.1296	0.512700	8	-0.34	1.21	2.70	2.69	2.69	0.74	0.82	0.74		0.74	Wu FY et al.	2003	Lithos	67(3-4)	191-204
2086	X-5	CAOB	NE China	127.3333	46.6667	Granitoid		173	173	4.60	19.13	0.1452	0.512688	8	-0.26	0.98	2.10	2.11	2.11	0.78	1.03	0.78		0.79	Wu FY et al.	2003	Lithos	67(3-4)	191-204
2087	X-4	CAOB	NE China	127.3333	46.6667	Granitoid		173	173	6.42	41.67	0.0932	0.512670	7	-0.53	0.62	2.90	2.91	2.91	0.72	0.61	0.72		0.72	Wu FY et al.	2003	Lithos	67(3-4)	191-204
2088	X-3	CAOB	NE China	127.3333	46.6667	Granitoid		173	173	2.47	13.39	0.1116	0.512686	6	-0.43	0.94	2.80	2.81	2.81	0.73	0.69	0.73		0.73	Wu FY et al.	2003	Lithos	67(3-4)	191-204
2089	X-2	CAOB	NE China	127.3333	46.6667	Granitoid		173	173	3.62	22.29	0.0982	0.512662	9	-0.50	0.47	2.60	2.64	2.64	0.74	0.64	0.74		0.74	Wu FY et al.	2003	Lithos	67(3-4)	191-204
2090	X-1	CAOB	NE China	127.3333	46.6667	Granitoid		173	173	1.35	7.10	0.1152	0.512674	7	-0.41	0.70	2.50	2.50	2.50	0.75	0.74	0.75		0.75	Wu FY et al.	2003	Lithos	67(3-4)	191-204
2091	L-9	CAOB	NE China	122.9167	48.5000	Granitoid		154	154	0.89	6.92	0.0782	0.512583	7	-0.60	-1.07	1.30	1.26	1.26	0.84	0.64	0.84		0.84	Wu FY et al.	2003	Lithos	67(3-4)	191-204
2092	L-8	CAOB	NE China	122.9167	48.5000	Granitoid		154	154	1.06	7.77	0.0828	0.512597	7	-0.58	-0.80	1.40	1.44	1.44	0.83	0.64	0.83		0.83	Wu FY et al.	2003	Lithos	67(3-4)	191-204
2093	L-7	CAOB	NE China	122.9167	48.5000	Granitoid		154	154	1.74	12.50	0.0840	0.512606	7	-0.57	-0.62	1.60	1.59	1.59	0.81	0.64	0.81		0.81	Wu FY et al.	2003	Lithos	67(3-4)	191-204
2094	L-6	CAOB	NE China	122.9167	48.5000	Granitoid		154	154	1.96	14.24	0.0831	0.512591	7	-0.58	-0.92	1.30	1.32	1.32	0.84	0.65	0.84		0.84	Wu FY et al.	2003	Lithos	67(3-4)	191-204
2095	L-5	CAOB	NE China	122.9167	48.5000	Granitoid		154	154	1.65	11.72	0.0851	0.512601	7	-0.57	-0.72	1.50	1.47	1.47	0.82	0.65	0.82		0.82	Wu FY et al.	2003	Lithos	67(3-4)	191-204
2096	L-4	CAOB	NE China	122.9167	48.5000	Granitoid		154	154	1.93	13.37	0.0871	0.512607	7	-0.56	-0.60	1.60	1.55	1.55	0.82	0.65	0.82		0.82	Wu FY et al.	2003	Lithos	67(3-4)	191-204
2097	L-3	CAOB	NE China	122.9167	48.5000	Granitoid		154	154	3.64	21.50	0.1024	0.512615	7	-0.48	-0.45	1.40	1.40	1.40	0.83	0.73	0.83		0.83	Wu FY et al.	2003	Lithos	67(3-4)	191-204
2098	L-2	CAOB	NE China	122.9167	48.5000	Granitoid		154	154	3.47	20.76	0.1012	0.512616	8	-0.49	-0.43	1.40	1.45	1.45	0.82	0.72	0.82		0.82	Wu FY et al.	2003	Lithos	67(3-4)	191-204
2099	L-1	CAOB	NE China	127.3333	46.6667	Granitoid	Lamashan	154	154	3.82	23.05	0.1001	0.512625	6	-0.49	-0.25	1.60	1.64	1.64	0.81	0.71	0.81		0.81	Wu FY et al.	2003	Lithos	67(3-4)	191-204
2100	ZK5-6	CAOB	Eastern Tianshan	94.7701	41.9140	Diorite		299	299	3.71	15.10	0.1496	0.512769	0	-0.24	2.56	4.35	4.35	4.35	0.71	0.91	0.71	0.91	0.71	Jiang HJ et al.	2017	Geoscience Frontiers	8(006)	1447-1467
2101	ZK5-1	CAOB	Eastern Tianshan	94.7701	41.9140	Diorite		299	299	4.21	16.20	0.1583	0.512783	0	-0.20	2.83	4.29	4.29	4.29	0.71	1.01	0.71	1.01	0.72	Jiang HJ et al.	2017	Geoscience Frontiers	8(006)	1447-1467
2102	ZK2-12	CAOB	Eastern Tianshan	94.7701	41.9140	Diorite		299	299	2.39	8.50	0.1713	0.512802	0	-0.13	3.20	4.17	4.17	4.17	0.72	1.25	0.72	1.26	0.73	Jiang HJ et al.	2017	Geoscience Frontiers	8(006)	1447-1467
2103	SQZ0914	CAOB	Eastern Tianshan	94.7716	41.9081	Andesite		315	315	8.79	39.80	0.1047	0.512557	0	-0.47	-1.58	2.12	2.12	2.12	0.90	0.83	0.90	0.83	0.91	Jiang HJ et al.	2017	Geoscience Frontiers	8(006)	1447-1467
2104	SQZ0913	CAOB	Eastern Tianshan	94.7716	41.9081	Andesite		315	315	3.76	14.00	0.1393	0.512668	0	-0.29	0.59	2.90	2.89	2.89	0.84	0.99	0.84	0.99	0.84	Jiang HJ et al.	2017	Geoscience Frontiers	8(006)	1447-1467
2105	SQZ0912	CAOB	Eastern Tianshan	94.7716	41.9081	Andesite		315	315	4.09	14.30	0.1255	0.512730	0	-0.36	1.79	4.66	4.66	4.66	0.69	0.73	0.69	0.73	0.70	Jiang HJ et al.	2017	Geoscience Frontiers	8(006)	1447-1467
2106	993	CAOB	SMXAB	114.3858	45.0772	Granite	Taolegai	125	150	7.02	24.85	0.1729	0.512117	6	-0.12	-10.16	-9.78	-9.71	-9.71	1.71	3.82	1.71	3.84	1.71	Chen JQ et al.	2013	Geological Journal of China Universities	19(3)	415
2107	992	CAOB	SMXAB	114.3858	45.0772	Granite	Taolegai	125	150	6.50	24.39	0.1601	0.512102	9	-0.19	-10.46	-9.88	-9.76	-9.76	1.72	2.96	1.72	2.97	1.72	Chen JQ et al.	2013	Geological Journal of China Universities	19(3)	415
2108	991	CAOB	SMXAB	114.3858	45.0772	Granite	Taolegai	125	150	6.92	25.06	0.1706	0.512116	8	-0.13	-10.18	-9.78	-9.68	-9.68	1.71	3.62	1.71	3.64	1.71	Chen JQ et al.	2013	Geological Journal of China Universities	19(3)	415
2109	990	CAOB	SMXAB	114.3858	45.0772	Granite	Taolegai	125	150	6.98	25.72	0.1633	0.512105	7	-0.17	-10.40	-9.88	-9.76	-9.76	1.72	3.14	1.72	3.15	1.72	Chen JQ et al.	2013	Geological Journal of China Universities	19(3)	415
2110	988	CAOB	SMXAB	114.3858	45.0772	Granite	Taolegai	125	150	7.39	26.84	0.1647	0.512107	9	-0.16	-10.36	-9.86	-9.75	-9.75	1.72	3.22	1.72	3.23	1.71	Chen JQ et al.	2013	Geological Journal of China Universities	19(3)	415
2111	987	CAOB	SMXAB	114.3858	45.0772	Granite	Taolegai	125	150	6.19	22.59	0.1628	0.512104	12	-0.17	-10.42	-9.88	-9.77	-9.77	1.72	3.11	1.72	3.12	1.72	Chen JQ et al.	2013	Geological Journal of China Universities	19(3)	415
2112	986	CAOB	SMXAB	114.3858	45.0772	Granite	Taolegai	125	150	7.81	26.03	0.1852	0.512123	7	-0.06	-10.05	-9.86	-9.83	-9.83	1.71	5.41	1.71	5.44	1.70	Chen JQ et al.	2013	Geological Journal of China Universities	19(3)	415
2113	985	CAOB	SMXAB	114.3858	45.0772	Granite	Taolegai	125	150	7.56	25.17	0.1849	0.512117	10	-0.06	-10.16	-9.97	-9.94	-9.94	1.72	5.39	1.72	5.41	1.71	Chen JQ et al.	2013	Geological Journal of China Universities	19(3)	415
2114	984	CAOB	SMXAB	114.3858	45.0772	Granite	Taolegai	125	150	13.95	41.23	0.2043	0.512128	8	0.04	-9.95	-10.1	-10.1	-10.1	1.67	15.78	1.67	15.96	1.67	Chen JQ et al.	2013	Geological Journal of China Universities	19(3)	415
2115	983	CAOB	SMXAB	114.3858	45.0772	Granite	Taolegai	125	150	8.41	28.36	0.1825	0.512114	9	-0.07	-10.22	-9.99	-9.95	-9.95	1.73	4.99	1.73	5.02	1.72	Chen JQ et al.	2013	Geological Journal of China Universities	19(3)	415
2116	B36a	CAOB	EasternTianshan	92.5200	43.0759	Rhyolite		340	340	9.50	35.14	0.1633	0.512912	9	-0.17	5.34	6.80	6.79	6.79	0.54	0.72	0.54	0.73	0.54	Chen XJ et al.	2013	Lithos	168-169	48-66
2117	B34a	CAOB	EasternTianshan	92.5200	43.0759	Rhyolite		340	340	4.20	19.02	0.1334	0.512802	12	-0.32	3.20	5.95	5.95	5.95	0.61	0.66	0.61	0.66	0.61	Chen XJ et al.	2013	Lithos	168-169	48-66
2118	B33a	CAOB	EasternTianshan	92.5200	43.0759	Rhyolite		340	340	11.04	45.16	0.1477	0.512848	10	-0.25	4.10	6.23	6.22	6.22	0.59	0.70	0.59	0.70	0.59	Chen XJ et al.	2013	Lithos	168-169	48-66
2119	B32a	CAOB	EasternTianshan	92.5200	43.0759	Rhyolite		340	340	10.32	33.78	0.1846	0.512910	11	-0.06	5.31	5.84	5.83	5.83	0.62	1.26	0.62	1.27	0.62	Chen XJ et al.	2013	Lithos	168-169	48-66
2120	B31a	CAOB	EasternTianshan	92.5200	43.0759	Rhyolite		340	340	12.91	44.36	0.1758	0.512925	10	-0.11	5.60	6.51	6.50	6.50	0.56	0.91	0.56	0.91	0.57	Chen XJ et al.	2013	Lithos	168-169	48-66
2121	B27a	CAOB	EasternTianshan	92.5200	43.0759	Rhyolite		340	340	7.32	30.80	0.1436	0.512761	5	-0.27	2.40	4.71	4.71	4.71	0.71	0.85	0.71	0.85	0.71	Chen XJ et al.	2013	Lithos	168-169	48-66
2122	B15a	CAOB	EasternTianshan	92.5200	43.0759	Rhyolite		340	340	10.87	32.63	0.2013	0.512987	10	0.02	6.81	6.61	6.61	6.61	0.55	1.99	0.55	2.02	0.56	Chen XJ et al.	2013	Lithos	168-169	48-66
2123	B144a	CAOB	EasternTianshan	92.5200	43.0759	Rhyolite		340	340	8.91	36.38	0.1480	0.512859	9	-0.25	4.31	6.43	6.43	6.43	0.57	0.68	0.57	0.68	0.57	Chen XJ et al.	2013	Lithos	168-169	48-66
2124	B126a	CAOB	EasternTianshan	92.5200	43.0759	Rhyolite		340	340	15.70	46.85	0.2025	0.512982	15	0.03	6.71	6.46	6.46	6.46	0.57	2.27	0.57	2.31	0.57	Chen XJ et al.	2013	Lithos	168-169	48-66
2125	B10a	CAOB	EasternTianshan	92.5200	43.0759	Rhyolite		340	340	9.45	35.09	0.1627	0.512933	10	-0.17	5.75	7.24	7.23	7.23	0.50	0.65	0.50	0.65	0.51	Chen XJ et al.	2013	Lithos	168-169	48-66
2126	748-5	CAOB	EasternTianshan	92.5200	43.0759	Rhyolite		345	345	11.57	48.29	0.1447	0.512860	25	-0.26	4.33	6.63	6.62	6.62	0.56	0.64	0.56	0.65	0.56	Chen XJ et al.	2013	Lithos	168-169	48-66
2127	748-4	CAOB	EasternTianshan	92.5200	43.0759	Rhyolite		345	345	10.44	47.16	0.1338	0.512826	3	-0.32	3.67	6.45	6.44	6.44	0.57	0.62	0.57	0.62	0.58	Chen XJ et al.	2013	Lithos	168-169	48-66
2128	748-2	CAOB	EasternTianshan	92.5200	43.0759	Rhyolite		345	345	13.83	60.24	0.1387	0.512944	2	-0.29	5.97	8.53	8.52	8.52	0.40	0.42	0.40	0.42	0.41	Chen XJ et al.	2013	Lithos	168-169	48-66
2129	648-5	CAOB	EasternTianshan	92.5200	43.0759	Rhyolite		345	345	12.92	55.43	0.1408	0.512837	4	-0.28	3.88	6.35	6.34	6.34	0.58	0.66	0.58	0.66	0.58	Chen XJ et al.	2013	Lithos	168-169	48-66
2130	648-3	CAOB	EasternTianshan	92.5200	43.0759	Rhyolite		345	345	12.09	50.17	0.1456	0.512944	3	-0.26	5.97	8.23	8.22	8.22	0.43	0.46	0.43	0.47	0.43	Chen XJ et al.	2013	Lithos	168-169	48-66
2131	45/208.7	CAOB	Russia	88.5833	54.9167	Carbonatite		509	509	2.97	11.90	0.1504	0.512740	7	-0.24	1.99	5.01	5.00	5.00	0.83	0.99	0.83			Vrublevskii VV et al	2014	Russian Geology and Geophysics	55(11)	1265-1277
2132	45/208.7	CAOB	Russia	88.5833	54.9167	Basic foidolite		509	509	5.18	27.50	0.1138	0.512618	5	-0.42	-0.39	5.00	4.99	4.99	0.83	0.81	0.83			Vrublevskii VV et al	2014	Russian Geology and Geophysics	55(11)	1265-1277
2133	45/208.7	CAOB	Russia	88.5833	54.9167	Carbonatite		509	509	21.50	106	0.1224	0.512648	4	-0.38	0.20	5.04	5.02	5.02	0.82	0.84	0.82			Vrublevskii VV et al	2014	Russian Geology and Geophysics	55(11)	1265-1277
2134	15/94.4	CAOB	Russia	88.5833	54.9167	Carbonatite		502	502	147	756	0.1176	0.512630	4	-0.40	-0.16	4.98	4.91	4.91	0.83	0.83	0.83			Vrublevskii VV et al	2014	Russian Geology and Geophysics	55(11)	1265-1277
2135	C-1041	CAOB	Umlekano–Ogodzhinskii Belt	125.0000	53.0000	Trachyandesibasalt	Gal'kinskii volcanic complex	119	150	7.72	41.54	0.1123	0.512529		-0.43	-2.13	-0.80	-0.51	-0.84	0.98	0.93	0.98	0.89		Sorokin AA et al.	2010	Doklady Earth Sciences	435(2)	1650-1655
2136	R-28	CAOB	Umlekano–Ogodzhinskii Belt	130.0000	52.0000	Granite	Amur	190	190	2.06	15.38	0.0809	0.51233		-0.59	-6.01	-3.20	-3.20	-3.20	1.23	0.94	1.23	0.94	1.26	Sorokin AA et al.	2010	Doklady Earth Sciences	435(2)	1650-1655
2137	C-1041-1	CAOB	Umlekano–Ogodzhinskii Belt	125.0000	53.0000	Trachyandesibasalt	Gal'kinskii volcanic complex	119	150	7.72	41.35	0.1129	0.51252		-0.43	-2.30	-1.00	-0.70	-1.03	1.00	0.95	1.00	0.95		Sorokin AA et al.	2010	Doklady Earth Sciences	435(2)	1650-1655
2138	K-48/3	CAOB	Umlekano–Ogodzhinskii Belt	125.0000	53.0000	Granitoids	amurskii and Burindinskii co	135	150	3.89	22.91	0.1026	0.512445		-0.48	-3.76	-2.10	-1.96	-2.14	1.10	0.97	1.10	0.97	1.12	Sorokin AA et al.	2010	Doklady Earth Sciences	435(2)	1650-1655
2139	C-1040	CAOB	Umlekano–Ogodzhinskii Belt	125.0000	53.0000	Rhyolite	Gal'kinskii volcanic complex	115	150	5.39	31.23	0.1042	0.512446		-0.47	-3.75	-2.40	-1.97	-2.39	1.10	0.98	1.10	0.98	1.13	Sorokin AA et al.	2010	Doklady Earth Sciences	435(2)	1650-1655
2140	K-291/4	CAOB	Umlekano–Ogodzhinskii Belt	125.0000	53.0000	associating with the bimod	Amur	115	150	4.99	32.36	0.0932	0.51237		-0.53	-5.23	-3.70	-3.25	-3.71	1.20	0.99	1.20	0.99	1.24	Sorokin AA et al.	2010	Doklady Earth Sciences	435(2)	1650-1655
2141	C-1040-1	CAOB	Umlekano–Ogodzhinskii Belt	125.0000	53.0000	Rhyolite	Gal'kinskii volcanic complex	115	150	5.17	29.94	0.1043	0.512442		-0.47	-3.82	-2.50	-2.05	-2.47	1.11	0.99	1.11	0.99	1.13	Sorokin AA et al.	2010	Doklady Earth Sciences	435(2)	1650-1655
2142	K-66A	CAOB	Umlekano–Ogodzhinskii Belt	125.0000	53.0000	Rhyolite	Gal'kinskii volcanic complex	115	150	4.75	27.71	0.1035	0.512436		-0.47	-3.94	-2.60	-2.16	-2.57	1.11	0.99	1.11	0.99	1.14	Sorokin AA et al.	2010	Doklady Earth Sciences	435(2)	1650-1655
2143	C-1032-1	CAOB	Umlekano–Ogodzhinskii Belt	125.0000	53.0000	Granitoids	amurskii and Burindinskii co	140	150	4.22	26.04	0.9080	0.512397		3.62	-4.70	-2.90	-18.32	-2.93	2.45	-0.17	2.45	0.99	1.99	Sorokin AA et al.	2010	Doklady Earth Sciences	435(2)	1650-1655
2144	C-1035	CAOB	Umlekano–Ogodzhinskii Belt	125.0000	53.0000	associating with the bimod	Amur	119	150	4.89	30.47	0.0970	0.512371		-0.51	-5.21	-3.70	-3.30	-3.69	1.21	1.02	1.21	1.02	1.24	Sorokin AA et al.	2010	Doklady Earth Sciences	435(2)	1650-1655
2145	C-1037-1	CAOB	Umlekano–Ogodzhinskii Belt	125.0000	53.0000	Andesite	Taldanskii volcanic complex	127	150	4.51	26.36	0.1033	0.512389		-0.47	-4.86	-3.30	-3.07	-3.34	1.19	1.05	1.19	1.05		Sorokin AA et al.	2010	Doklady Earth Sciences	435(2)	1650-1655
2146	C-1033-1	CAOB	Umlekano–Ogodzhinskii Belt	125.0000	53.0000	associating with the bimod	Amur	119	150	1.75	10.77	0.0982	0.512352		-0.50	-5.58	-4.10	-3.69	-4.08	1.24	1.05	1.24	1.05	1.27	Sorokin AA et al.	2010	Doklady Earth Sciences	435(2)	1650-1655
2147	C-1031-1	CAOB	Umlekano–Ogodzhinskii Belt	125.0000	53.0000	Granitoids	amurskii and Burindinskii co	140	150	3.7	21.61	0.1033	0.512375		-0.47	-5.13	-3.50	-3.34	-3.46	1.21	1.07	1.21	1.07	1.24	Sorokin AA et al.	2010	Doklady Earth Sciences	435(2)	1650-1655
2148	C-1031	CAOB	Umlekano–Ogodzhinskii Belt	125.0000	53.0000	Granitoids	amurskii and Burindinskii co	140	150	3.33	19.9	0.1012	0.512355		-0.49	-5.52	-3.80	-3.69	-3.81	1.24	1.08	1.24	1.08	1.27	Sorokin AA et al.	2010	Doklady Earth Sciences	435(2)	1650-1655
2149	C-1034	CAOB	Umlekano–Ogodzhinskii Belt	125.0000	53.0000	Granitoids	amurskii and Burindinskii co	140	150	4.71	27.35	0.1040	0.512375		-0.47	-5.13	-3.50	-3.36	-3.47	1.21	1.08	1.21	1.08	1.24	Sorokin AA et al.	2010	Doklady Earth Sciences	435(2)	1650-1655
2150	K-290/1	CAOB	Umlekano–Ogodzhinskii Belt	125.0000	53.0000	Andesite	Taldanskii volcanic complex	123	150	4.09	24.04	0.1028	0.512366		-0.48	-5.31	-3.80	-3.51	-3.83	1.22	1.08	1.22	1.08		Sorokin AA et al.	2010	Doklady Earth Sciences	435(2)	1650-1655
2151	K-294	CAOB	Umlekano–Ogodzhinskii Belt	125.0000	53.0000	Granitoids	amurskii and Burindinskii co	127	150	3.71	21.23	0.1056	0.512376		-0.46	-5.11	-3.60	-3.37	-3.63	1.21	1.09	1.21	1.09	1.24	Sorokin AA et al.	2010	Doklady Earth Sciences	435(2)	1650-1655
2152	C-1037	CAOB	Umlekano–Ogodzhinskii Belt	125.0000	53.0000	Andesite	Taldanskii volcanic complex	127	150	4.55	25.12	0.1095	0.512403		-0.44	-4.58	-3.20	-2.92	-3.17	1.18	1.09	1.18	1.09		Sorokin AA et al.	2010	Doklady Earth Sciences	435(2)	1650-1655
2153	C-1038	CAOB	Umlekano–Ogodzhinskii Belt	125.0000	53.0000	Andesite	Taldanskii volcanic complex	127	150	3.84	21.48	0.1079	0.51239		-0.45	-4.84	-3.40	-3.14	-3.40	1.19	1.09	1.19	1.10		Sorokin AA et al.	2010	Doklady Earth Sciences	435(2)	1650-1655
2154	C-1037-2	CAOB	Umlekano–Ogodzhinskii Belt	125.0000	53.0000	Andesite	Taldanskii volcanic complex	127	150	4.39	25.05	0.1059	0.512372		-0.46	-5.19	-3.70	-3.45	-3.72	1.22	1.10	1.22	1.10		Sorokin AA et al.	2010	Doklady Earth Sciences	435(2)	1650-1655
2155	K-291/3	CAOB	Umlekano–Ogodzhinskii Belt	125.0000	53.0000	Granitoids	amurskii and Burindinskii co	142	150	4.7	26.42	0.1074	0.512382		-0.45	-4.99	-3.40	-3.28	-3.37	1.21	1.10	1.21	1.10	1.23	Sorokin AA et al.	2010	Doklady Earth Sciences	435(2)	1650-1655
2156	C-1032	CAOB	Umlekano–Ogodzhinskii Belt	125.0000	53.0000	Granitoids	amurskii and Burindinskii co	140	150	3.22	18.85	1.1031	0.512346		4.61	-5.70	-4.00	-23.05	-4.02	2.83	-0.14	2.83	1.11	1.28	Sorokin AA et al.	2010	Doklady Earth Sciences	435(2)	1650-1655
2157	C-990	CAOB	Umlekano–Ogodzhinskii Belt	130.0000	52.0000	Granite	Amur	200	200	4.82	28.37	0.1028	0.512341		-0.48	-5.79	-3.40	-3.40	-3.39	1.26	1.11	1.26	1.11	1.28	Sorokin AA et al.	2010	Doklady Earth Sciences	435(2)	1650-1655
2158	K-68	CAOB	Umlekano–Ogodzhinskii Belt	125.0000	53.0000	Andesite	Taldanskii volcanic complex	125	150	3.7	20.68	0.1080	0.512373		-0.45	-5.17	-3.80	-3.47	-3.75	1.22	1.12	1.22	1.12		Sorokin AA et al.	2010	Doklady Earth Sciences	435(2)	1650-1655

2159	C-1039	CAOB	Umlekano–Ogodzhinskii Belt	125.0000	53.0000	Andesite	Taldanskii volcanic complex	127	150	4.15	22.46	0.1117	0.512374		-0.43	-5.15	-3.80	-3.52	-3.77	1.23	1.16	1.23	1.16		Sorokin AA et al.	2010	Doklady Earth Sciences	435(2)	1650-1655
2160	C-923	CAOB	Umlekano–Ogodzhinskii Belt	130.0000	52.0000	Granite	Amur	190	190	4.21	21.63	0.1178	0.512398		-0.40	-4.68	-2.80	-2.77	-2.76	1.20	1.19	1.20	1.20	1.22	Sorokin AA et al.	2010	Doklady Earth Sciences	435(2)	1650-1655
2161	K-311	CAOB	Umlekano–Ogodzhinskii Belt	125.0000	53.0000	Granitoids	amurskii and Burindinskii co	130	150	3.84	21.28	0.1092	0.512329		-0.44	-6.03	-4.60	-4.35	-4.57	1.29	1.20	1.29	1.20	1.32	Sorokin AA et al.	2010	Doklady Earth Sciences	435(2)	1650-1655
2162	C-801	CAOB	Umlekano–Ogodzhinskii Belt	130.0000	52.0000	Granite	Amur	208	208	3.88	22.94	0.1023	0.512252		-0.48	-7.53	-5.00	-5.02	-5.02	1.40	1.23	1.40	1.23	1.42	Sorokin AA et al.	2010	Doklady Earth Sciences	435(2)	1650-1655
2163	C-909	CAOB	Umlekano–Ogodzhinskii Belt	130.0000	52.0000	Granite	Amur	200	200	7.96	34.91	0.1377	0.51236		-0.30	-5.42	-2.80	-3.92	-3.91	1.30	1.58	1.30	1.20	1.22	Sorokin AA et al.	2010	Doklady Earth Sciences	435(2)	1650-1655
2164	TST-15	CAOB	Baogutu, West Junggar	86.0750	47.1417	Monzogranite	Taste	314	314	5.06	23.13	0.1323	0.512872	19	-0.33	4.56	7.14	7.14	7.14	0.49	0.52	0.49	0.52	0.49	Yuan F et al.	2006	Acta Petrologica Sinica	(05)	1238-1248
2165	TST-10	CAOB	Baogutu, West Junggar	86.0750	47.1417	Monzogranite	Taste	314	314	2.28	11.43	0.1204	0.512815	13	-0.39	3.45	6.51	6.51	6.51	0.54	0.55	0.54	0.55	0.54	Yuan F et al.	2006	Acta Petrologica Sinica	(05)	1238-1248
2166	STS-1	CAOB	Baogutu, West Junggar	86.0167	47.0333	Monzogranite	Sentas	328	328	3.98	20.94	0.1149	0.512846	8	-0.42	4.06	7.48	7.48	7.48	0.47	0.47	0.47	0.47	0.47	Yuan F et al.	2006	Acta Petrologica Sinica	(05)	1238-1248
2167	QQH-14	CAOB	Baogutu, West Junggar	86.1750	47.5000	Alkali feldspar granite	Chaqihai	291	291	8.79	47.82	0.1112	0.512865	12	-0.43	4.43	7.60	7.60	7.60	0.43	0.42	0.43	0.42	0.43	Yuan F et al.	2006	Acta Petrologica Sinica	(05)	1238-1248
2168	OK-17	CAOB	Baogutu, West Junggar	86.2583	47.0167	Monzogranite	Woken Sara	324	324	3.83	20.69	0.1119	0.512804	16	-0.43	3.24	6.74	6.74	6.74	0.53	0.52	0.53	0.52	0.53	Yuan F et al.	2006	Acta Petrologica Sinica	(05)	1238-1248
2169	OK-16	CAOB	Baogutu, West Junggar	86.2583	47.0167	Monzogranite	Woken Sara	324	324	3.62	17.46	0.1255	0.512765	17	-0.36	2.48	5.42	5.42	5.42	0.64	0.67	0.64	0.67	0.64	Yuan F et al.	2006	Acta Petrologica Sinica	(05)	1238-1248
2170	0076-9	CAOB	CMEB	124.1564	51.6278	Granodiorite	Xinlinzhen	130	150	2.86	16.00	0.1100	0.512553	14	-0.44	-1.66	-0.23	0.00	0.00	0.94	0.88	0.94	0.88	0.94	Zhang YL et al.	2008	ournal of Jilin University (Earth Science Edition	38(2)	177-186
2171	0076-2	CAOB	CMEB	124.1564	51.6278	Granodiorite	Xinlinzhen	130	150	2.58	16.80	0.0900	0.512541	10	-0.54	-1.89	-0.13	0.15	0.15	0.93	0.75	0.93	0.75	0.94	Zhang YL et al.	2008	ournal of Jilin University (Earth Science Edition	38(2)	177-186
2172	0075-4	CAOB	CMEB	124.3239	51.6063	Granodiorite	Xinlinzhen	130	150	1.58	10.40	0.0900	0.512549	13	-0.54	-1.74	0.03	0.31	0.31	0.91	0.74	0.91	0.74	0.92	Zhang YL et al.	2008	ournal of Jilin University (Earth Science Edition	38(2)	177-186
2173	0075-3	CAOB	CMEB	124.3239	51.6063	Granodiorite	Xinlinzhen	130	150	2.86	17.40	0.1000	0.512598	12	-0.49	-0.78	0.83	1.07	1.07	0.85	0.74	0.85	0.74	0.86	Zhang YL et al.	2008	ournal of Jilin University (Earth Science Edition	38(2)	177-186
2174	T81	CAOB	Nalati,Tianshan	80.7500	42.5833	Monzogranite	Xiatedongnan	344	344	5.41	30.96	0.1057	0.512000	9	-0.46	-12.45	-8.45	-8.45	-8.45	1.78	1.62	1.78	1.62	1.78	Zhou TX et al.	2000	Acta Petrologica Sinica	16(2)	153-160
2175	T8	CAOB	Central Tianshan	81.9250	42.7750	Alkali feldspar granite		287	287	4.43	17.30	0.1549	0.512363		-0.21	-5.36	-3.84	-3.83	-3.83	1.36	2.03	1.36	2.04	1.37	Zhou TX et al.	2000	Acta Petrologica Sinica	16(2)	153-160
2176	T75	CAOB	Central Tianshan	81.9250	42.7750	Diorite		344	344			0.1414	0.511992		-0.28	-12.60	-10.18	-10.17	-10.17	1.92	2.43	1.92	2.43	1.89	Zhou TX et al.	2000	Acta Petrologica Sinica	16(2)	153-160
2177	T60	CAOB	Nalati,Tianshan	80.7833	42.5667	Felsic granulite	Alasan	302	302			0.1209	0.511995	8	-0.39	-12.54	-9.72	-9.62	-9.62	1.84	1.89	1.84		1.85	Zhou TX et al.	2000	Acta Petrologica Sinica	16(2)	153-160
2178	T53	CAOB	Nalati,Tianshan	80.7833	42.5667	Diorite	Alasandongnan	382	382			0.1317	0.512308	6	-0.33	-6.44	-3.42	-3.27	-3.27	1.39	1.56	1.39		1.41	Zhou TX et al.	2000	Acta Petrologica Sinica	16(2)	153-160
2179	T3	CAOB	Nalati,Tianshan	80.3250	41.7775	Monzogranite	Tuomuerdongnan	290	290			0.1180	0.512187	10	-0.40	-8.80	-6.02	-5.89	-5.89	1.53	1.53	1.53		1.54	Zhou TX et al.	2000	Acta Petrologica Sinica	16(2)	153-160
2180	T23	CAOB	Nalati,Tianshan	80.7500	42.3333	Rhyolite Porphyry	Tulasudabandongnan	260	260			0.1394	0.512077	9	-0.29	-10.94	-9.18	-9.04	-9.04	1.76	2.19	1.76		1.77	Zhou TX et al.	2000	Acta Petrologica Sinica	16(2)	153-160
2181	T17	CAOB	Central Tianshan	81.9250	42.7750	Dolomite Qtz schist		300	300			0.1070	0.511591		-0.46	-20.42	-17.0	-17.0	-17.0	2.44	2.22	2.44	2.22		Zhou TX et al.	2000	Acta Petrologica Sinica	16(2)	153-160
2182	T13	CAOB	Nalati,Tianshan	80.8333	41.9167	Alkali feldspar granite	Yingmanlai	260	260			0.1458	0.512072	11	-0.26	-11.04	-9.39	-9.35	-9.35	1.78	2.41	1.78	2.41	1.79	Zhou TX et al.	2000	Acta Petrologica Sinica	16(2)	153-160
2183	T104	CAOB	Nalati,Tianshan	80.5833	42.6167	Tonalite	Xiatezhengnan	218	218			0.1059	0.511960	7	-0.46	-13.23	-10.7	-10.7	-10.7	1.86	1.68	1.86	1.67	1.85	Zhou TX et al.	2000	Acta Petrologica Sinica	16(2)	153-160
2184	87-54	CAOB	SMXAB	122.9150	47.6281	Adamellite	Nianzishan	125	150	8.50	40.70	0.1265	0.512701		-0.36	1.23	2.4	2.6	2.6	0.73	0.79	0.73	0.79	0.73	Li PZ and Yu JS.	1994	Zhejiang University Press		269-286
2185	87-43	CAOB	SMXAB	122.9150	47.6281	Alkali granite	Nianzishan	125	150	8.60	41.00	0.1260	0.512714		-0.36	1.48	2.61	2.84	2.84	0.71	0.76	0.71	0.76	0.71	Li PZ and Yu JS.	1994	Zhejiang University Press		269-286
2186	87-143	CAOB	SMXAB	122.9150	47.6281	Alkali granite	Nianzishan	125	150	13.30	70.70	0.1137	0.512710		-0.42	1.40	2.73	2.99	2.99	0.69	0.67	0.69	0.67	0.70	Li PZ and Yu JS.	1994	Zhejiang University Press		269-286
2187	84-311	CAOB	SMXAB	122.9150	47.6281	Alkali granite	Nianzishan	125	150	18.50	82.80	0.1349	0.512735		-0.31	1.89	2.89	3.08	3.08	0.69	0.80	0.69	0.81	0.69	Li PZ and Yu JS.	1994	Zhejiang University Press		269-286
2188	PM5LT124a	CAOB	SMXAB	122.8847	49.4108	Orthoclase granite	Liufangjian	172	172	2.24	14.53	0.0931	0.512506	7	-0.53	-2.57	-0.30	-0.30	-0.30	0.98	0.81	0.98	0.82	0.98	Li H.	2012	Dissertation China University of Geosciences, Wuhan		
2189	PM14LT151	CAOB	SMXAB	123.0525	49.4039	Adamellite	Xieniqi	161	161	6.90	34.37	0.1214	0.512626	8	-0.38	-0.23	1.31	1.31	1.31	0.84	0.87	0.84	0.87	0.84	Li H.	2012	Dissertation China University of Geosciences, Wuhan		
2190	PM14LT111	CAOB	SMXAB	123.0525	49.4039	Adamellite	Xieniqi	161	161	3.21	21.14	0.0917	0.512627	6	-0.53	-0.21	1.94	1.94	1.94	0.79	0.65	0.79	0.66	0.79	Li H.	2012	Dissertation China University of Geosciences, Wuhan		
2191	D2032	CAOB	SMXAB	122.8847	49.4108	Adamellite	Liufangjian	172	172	1.62	9.07	0.1083	0.512579	9	-0.45	-1.15	0.79	0.79	0.79	0.89	0.83	0.89	0.83	0.90	Li H.	2012	Dissertation China University of Geosciences, Wuhan		
2192	EL10-9-1	CAOB	NE China	112.1572	43.8728	Monzogranite	Gancihuduge	270	270	3.24	14.60	0.1341	0.512770		-0.32	2.57	4.70	4.73	4.73	0.65	0.73	0.65	0.73	0.65	Yuan LL et al.	2016	Lithos	264	524-539
2193	EL10-9-2	CAOB	NE China	112.1572	43.8728	Monzogranite	Gancihuduge	270	270	3.78	17.20	0.1333	0.512740		-0.32	1.99	4.30	4.17	4.17	0.70	0.78	0.70	0.78	0.70	Yuan LL et al.	2016	Lithos	264	524-539
2194	EL10-9-5	CAOB	NE China	112.1572	43.8728	Monzogranite	Gancihuduge	270	270	3.44	15.60	0.1338	0.512660		-0.32	0.43	2.70	2.60	2.60	0.83	0.93	0.83	0.94	0.83	Yuan LL et al.	2016	Lithos	264	524-539
2195	EL10-9-8	CAOB	NE China	112.1572	43.8728	Monzogranite	Gancihuduge	270	270	3.29	14.80	0.1345	0.512753		-0.32	2.24	4.40	4.39	4.39	0.68	0.76	0.68	0.77	0.68	Yuan LL et al.	2016	Lithos	264	524-539
2196	EL10-6-1	CAOB	NE China	112.1575	43.8050	Porphyritic monzogranite	Cailiwusu	276	276	5.95	24.20	0.1487	0.512647		-0.24	0.18	1.90	1.87	1.87	0.89	1.18	0.89	1.18	0.89	Yuan LL et al.	2016	Lithos	264	524-539
2197	EL10-6-2	CAOB	NE China	112.1575	43.8050	Porphyritic monzogranite	Cailiwusu	276	276	6.79	29.30	0.1403	0.512659		-0.29	0.41	2.40	2.40	2.40	0.85	1.02	0.85	1.02	0.85	Yuan LL et al.	2016	Lithos	264	524-539
2198	EL10-14-1	CAOB	NE China	112.2447	43.6314	Syenogranite	Kunduleng	270	270	8.59	40.20	0.1293	0.512661		-0.34	0.45	2.90	2.77	2.77	0.81	0.88	0.81	0.89	0.82	Yuan LL et al.	2016	Lithos	264	524-539
2199	EL10-15-1	CAOB	NE China	112.2447	43.6314	Syenogranite	Kunduleng	270	270	7.59	34.10	0.1349	0.512707		-0.31	1.35	3.50	3.48	3.48	0.75	0.86	0.75	0.86	0.76	Yuan LL et al.	2016	Lithos	264	524-539
2200	EL10-16-1	CAOB	NE China	112.2706	43.6308	Syenogranite	Kunduleng	270	270	7.74	30.10	0.1558	0.512697		-0.21	1.15	2.60	2.56	2.56	0.83	1.19	0.83	1.20	0.84	Yuan LL et al.	2016	Lithos	264	524-539
2201	EL10-20-1	CAOB	NE China	112.3344	43.6717	Syenogranite	Kunduleng	280	280	5.93	27.30	0.1315	0.512679		-0.33	0.80	3.10	3.13	3.13	0.79	0.87	0.79	0.88	0.79	Yuan LL et al.	2016	Lithos	264	524-539
2202	EL10-20-2	CAOB	NE China	112.3344	43.6717	Syenogranite	Kunduleng	280	280	5.37	25.40	0.1280	0.512688		-0.35	0.98	3.40	3.43	3.43	0.77	0.82	0.77	0.83	0.77	Yuan LL et al.	2016	Lithos	264	524-539
2203	EL10-20-5	CAOB	NE China	112.3344	43.6717	Syenogranite	Kunduleng	280	280	5.28	24.20	0.1323	0.512643		-0.33	0.10	2.40	2.40	2.40	0.85	0.95	0.85	0.95	0.85	Yuan LL et al.	2016	Lithos	264	524-539
2204	EL10-21-1	CAOB	NE China	112.3344	43.6717	Syenogranite	Kunduleng	280	280	7.23	32.00	0.1367	0.512710		-0.31	1.40	3.50	3.55	3.55	0.76	0.87	0.76	0.87	0.76	Yuan LL et al.	2016	Lithos	264	524-539
2205	EL10-21-3	CAOB	NE China	112.2722	43.6267	Syenogranite	Kunduleng	280	280	7.51	33.10	0.1374	0.512683		-0.30	0.88	3.00	3.00	3.00	0.80	0.93	0.80	0.94	0.80	Yuan LL et al.	2016	Lithos	264	524-539
2206	Д828	CAOB	Russia	95.5100	51.6667	Granitoid		562	562	######	5357.00	0.2345	0.513107	11	0.19	9.15	6.50	6.44	6.44	0.75	-0.32	0.75			Rudnev SN et al.	2006	Литосфера	2	3-33
2207	Д675/1	CAOB	Russia	95.1667	51.7500	Granitoid		535	535	######	8598	0.2097	0.513012	13	0.07	7.30	6.40	6.41	6.41	0.72	5.23	0.72			Rudnev SN et al.	2006	Литосфера	2	3-33
2208	Д624	CAOB	Russia	95.3300	51.7500	Granitoid		474	474	######	13863	0.1072	0.512561	7	-0.45	-1.50	3.90	3.91	3.91	0.89	0.84	0.89		0.90	Rudnev SN et al.	2006	Литосфера	2	3-33
2209	Д515	CAOB	Russia	95.5000	51.4667	Granitoid		385	385	######	26597	0.1108	0.512540	17	-0.44	-1.91	2.30	2.31	2.31	0.94	0.90	0.94		0.96	Rudnev SN et al.	2006	Литосфера	2	3-33
2210	Д35	CAOB	Russia	95.4700	51.6333	Granitoid		512	512	######	30447	0.1058	0.512468	9	-0.46	-3.32	2.60	2.62	2.62	1.02	0.96	1.02		1.04	Rudnev SN et al.	2006	Литосфера	2	3-33
2211	Д1019e	CAOB	Russia	96.0833	51.4167	Granitoid		450	450	######	50863	0.1066	0.512459	11	-0.46	-3.49	1.70	1.68	1.68	1.05	0.98	1.05		1.06	Rudnev SN et al.	2006	Литосфера	2	3-33
2212	5563	CAOB	Russia	96.0500	51.3833	Granitoid		450	450	######	23250	0.1235	0.512596	8	-0.37	-0.82	3.40	3.38	3.38	0.91	0.94	0.91		0.92	Rudnev SN et al.	2006	Литосфера	2	3-33
2213	P2	CAOB	Northeast Tarim Craton	90.6000	41.2667	Granite		816	600			0.0857	0.511723		-0.56	-17.85	-6.27	-9.35	-9.35	2.07	1.70	2.07	1.70	2.01	Cao XF et al.	2011	Precambrian Research	186(1-4)	205-219
2214	P1	CAOB	Northeast Tarim Craton	90.6000	41.2667	Granite		816	600			0.1067	0.511755		-0.46	-17.22	-7.84	-10.3	-10.3	2.14	1.98	2.14	1.98	2.14	Cao XF et al.	2011	Precambrian Research	186(1-4)	205-219
2215	M1	CAOB	Northeast Tarim Craton	90.6000	41.2667	Granite		816	600			0.0990	0.511370		-0.50	-24.73	-14.6	-17.3	-17.3	2.70	2.35	2.70	2.36	2.69	Cao XF et al.	2011	Precambrian Research	186(1-4)	205-219
2216	K1	CAOB	Northeast Tarim Craton	90.6000	41.2667	Granite		816	600			0.1029	0.511802		-0.48	-16.31	-6.53	-9.13	-9.13	2.05	1.85	2.05	1.85	2.03	Cao XF et al.	2011	Precambrian Research	186(1-4)	205-219
2217		CAOB	Northeast Tarim Craton	90.6000	41.2667	Bi monzogranite		816	600			0.1033	0.511749		-0.47	-17.34	-7.61	-10.2	-10.2	2.13	1.93	2.13	1.93	2.12	Cao XF et al.	2011	Precambrian Research	186(1-4)	205-219
2218	YBL-5	CAOB	CAOB	58.6833	48.9167	Tonalite porphyry		380	380	1.61	6.53	0.1489	0.512371		-0.24	-5.21	-2.90	-2.89	-2.89	1.36	1.83	1.36	1.83	1.37	Shen P et al.	2018	Gondwana Research	58	161-194
2219	YBL-3	CAOB	CAOB	58.6833	48.9167	Tonalite porphyry		380	380	1.96	7.88	0.1500	0.512387		-0.24	-4.90	-2.65	-2.63	-2.63	1.34	1.82	1.34	1.83	1.34	Shen P et al.	2018	Gondwana Research	58	161-194
2220	YBL-2	CAOB	CAOB	58.6833	48.9167	Tonalite porphyry		380	380	2.21	9.48	0.1407	0.512364		-0.28	-5.34	-2.63	-2.63	-2.63	1.34	1.64	1.34	1.64	1.34	Shen P et al.	2018	Gondwana Research	58	161-194
2221	9HS76-4	CAOB	CAOB	78.4667	44.4833	Granodiorite porphyry		440	440	1.46	8.77	0.1006	0.512459		-0.49	-3.49	0.90	1.91	1.91	1.02	0.93	1.02	0.93	1.02	Shen P et al.	2018	Gondwana Research	58	161-194
2222	9HS76-3	CAOB	CAOB	78.4667	44.4833	Granodiorite porphyry		440	440	3.59	20.40	0.1063	0.512381		-0.46	-5.01	0.04	0.06	0.06	1.17	1.09	1.17	1.09	1.17	Shen P et al.	2018	Gondwana Research	58	161-194
2223	12HSB03-7	CAOB	CAOB	74.3005	51.8533	Tonalite porphyry		490	490	2.33	11.82	0.1186	0.512618		-0.40	-0.39	4.48	4.49	4.49	0.85	0.85	0.85	0.86	0.86	Shen P et al.	2018	Gondwana Research	58	161-194
2224	12HSB03-14	CAOB	CAOB	74.3005	51.8533	Tonalite porphyry		490	490	2.48	12.64	0.1195	0.512658		-0.39	0.39	5.28	5.22	5.22	0.79	0.80	0.79	0.79	0.79	Shen P et al.	2018	Gondwana Research	58	161-194
2225	12HS03-1	CAOB	CAOB	72.9978	50.1743	Granodiorite porphyry		440	440	2.70	13.87	0.1179	0.512639		-0.40	0.02	4.47	4.45	4.45	0.81	0.81	0.81	0.82	0.82	Shen P et al.	2018	Gondwana Research	58	161-194
2226	YZK6307-610	CAOB	CAOB	92.6031	42.1146	Tonalite porphyry		332	332	1.62	8.96	0.1091	0.512703	6	-0.45	1.27	4.91	4.98	4.98	0.68	0.65	0.68	0.65	0.69	Shen P et al.	2018	Gondwana Research	58	161-194
2227	YZK6307-270	CAOB	CAOB	92.6031	42.1146	Tonalite porphyry		332	332	1.64	10.20	0.0973	0.512662	9	-0.51	0.47	4.60	4.68	4.68	0.71	0.64	0.71	0.64	0.71	Shen P et al.	2018	Gondwana Research	58	161-194
2228	TZK002-379	CAOB	CAOB	92.6031	42.1146	Tonalite porphyry		332	332	1.80	10.50	0.1040	0.512650	7	-0.47	0.23	4.09	4.16	4.16	0.75	0.70	0.75	0.70	0.75	Shen P et al.	2018	Gondwana Research	58	161-194
2229	TZK002-316	CAOB	CAOB	92.6031	42.1146	Tonalite porphyry		332	332	1.59	8.77	0.1100	0.512624	6	-0.44	-0.27	3.33	3.40	3.40	0.81	0.77	0.81	0.78	0.81	Shen P et al.	2018	Gondwana Research	58	161-194
2230	9HS49-7	CAOB	CAOB	79.9846	46.9685	Diorite porphyry		331	331	2.84	16.43	0.1047	0.512697	13	-0.47	1.15	5.02	5.04	5.04	0.68	0.63	0.68	0.64	0.68	Shen P et al.	2018	Gondwana Research	58	161-194
2231	9HS49-5	CAOB	CAOB	79.9846	46.9685	Tonalite porphyry		331	331	3.53	20.86	0.1026	0.512685	14	-0.48	0.92	4.88	4.89	4.89	0.69	0.64	0.69	0.64	0.69	Shen P et al.	2018	Gondwana Research	58	161-194
2232	T33-5	NCC	NCC	122.0000	41.0000	Rhyolite		123	150	5.26	45.00	0.0710	0.511788		-0.64	-16.58	-14.7	-14.2	-14.2	2.09	1.45	2.09		1.46	Sun J et al.	2017	Lithos	292-293	69-80
2233	T33-4	NCC	NCC	122.0000	41.0000	Rhyolite		123	150	5.98	49.00	0.0731	0.511789		-0.63	-16.56	-14.7	-14.2	-14.2	2.09	1.47	2.09		1.48	Sun J et al.	2017	Lithos	292-293	69-80
2234	T33-3	NCC	NCC	122.0000	41.0000	Rhyolite		123	150	6.21	51.00	0.0732	0.511786		-0.63	-16.62	-14.7	-14.3	-14.3	2.10	1.48	2.10		1.48	Sun J et al.	2017	Lithos	292-293	69-80
2235	T33-2	NCC	NCC	122.0000	41.0000	Rhyolite		123	150	5.33	45.00	0.0720	0.511741		-0.63	-17.50	-15.6	-15.1	-15.1	2.17	1.51	2.17		1.52	Sun J et al.	2017	Lithos	292-293	69-80
2236	T33-1	NCC	NCC	122.0000	41.0000	Rhyolite		123	150	5.08	44.00	0.0720	0.511791		-0.63	-16.52	-14.6	-14.1	-14.1	2.09	1.46	2.09		1.44	Sun J et al.	2017	Lithos	292-293	69-80
2237	T32-2	NCC	NCC	122.0000	41.0000	Rhyolite		123	150	5.80	48.00	0.0736	0.511774		-0.63	-16.85	-15.0	-14.5	-14.5	2.12	1.49	2.12		1.50	Sun J et al.	2017	Lithos	292-293	69-80
2238	T32-1	NCC	NCC	122.0000	41.0000	Rhyolite		123	150	6.32	52.00	0.0734	0.511766		-0.63	-17.01	-15.1	-14.6	-14.6	2.13	1.50	2.13		1.50	Sun J et al.	2017	Lithos	292-293	69-80
2239	QG2-4	NCC	NCC	122.0000	41.0000	Dacite		123	150	3.32	21.00	0.0955	0.511596		-0.51	-20.33	-18.3	-18.4	-18.4	2.43	2.00	2.43		2.01	Sun J et al.	2017	Lithos	292-293	69-80
2240	DG102-2	NCC	NCC	122.0000	41.0000	Diorite		123	150	4.26	26.60	0.1009	0.511606		-0.49	-20.13	-18.1	-18.3	-18.3	2.42	2.08	2.42		2.16	Sun J et al.	2017	Lithos	292-293	69-80
2241	DG102-4	NCC	NCC	122.0000	41.0000	Diorite		123	150	4.20	25.20	0.0969	0.511551		-0.51	-21.20	-19.3	-19.3	-19.3	2.51	2.08	2.51		2.00	Sun J et al.	2017	Lithos	292-293	69-80
2242	2014GA-265	CAOB	Southwest Mongolia	97.8375	44.5972	Granite		314	314	8.93	49.19	0.1097	0.512777	±10	-0.44	2.71	6.20	6.20	6.20	0.57	0.55	0.57	0.55	0.59	Zhu M et al.	2017	Journal of Asian Earth Sciences	144	54-68
2243	2014GA-264	CAOB	Southwest Mongolia	97.8544	44.6125	Granite		314	314	6.06	26.71	0.1371	0.512808	±9	-0.30	3.32	5.70	5.71	5.71	0.61	0.68	0.61	0.68	0.57	Zhu M et al.	2017	Journal of Asian Earth Sciences	144	54-68
2244	2014GA-250-1	CAOB	Southwest Mongolia	97.8375	44.5972	Rhyolite		314	314	11.78	62.20	0.1145	0.512784	±8	-0.42	2.85	6.10	6.14	6.14	0.57	0.56	0.57	0.56	0.58	Zhu M et al.	2017	Journal of Asian Earth Sciences	144	54-68
2245	2014GA-245	CAOB	Southwest Mongolia	97.8544	44.6125	Rhyolite		314	314	15.17	78.67	0.1166	0.512798	±7	-0.41	3.12	6.30	6.33	6.33	0.56	0.55	0.56	0.55	0.56	Zhu M et al.	2017	Journal of Asian Earth Sciences	144	54-68
2246	2014GA-244	CAOB	Southwest Mongolia	97.8375	44.5972	Rhyolite		314	314	16.52	84.15	0.1187	0.512787	±8	-0.40	2.91	6.00	6.03	6.03	0.58	0.58	0.58	0.58	0.58	Zhu M et al.	2017	Journal of Asian Earth Sciences	144	54-68
2247	2014GA-243	CAOB	Southwest Mongolia	97.8544	44.6125	Rhyolite		314	314	16.19	93.18	0.1051	0.512780	±9	-0.47	2.77	6.40	6.44	6.44	0.55	0.52	0.55	0.52	0.58	Zhu M et al.	2017	Journal of Asian Earth Sciences	144	54-68
2248	GA-265	CAOB	Southwest Mongolia	98.0000	44.5000	Granite		329	329	8.93	49.19	0.1097	0.512777		-0.44	2.71	6.20	6.36	6.36	0.57	0.55	0.57	0.55	0.59	Zhu M S et al.	2017	Journal of Asian Earth Sciences	144	54-68
2249	GA-264	CAOB	Southwest Mongolia	98.0000	44.5000	Granite		329	329	6.06	26.71	0.1371	0.512808		-0.30	3.32	5.70	5.82	5.82	0.61	0.68	0.61	0.68	0.57	Zhu M S et al.	2017	Journal of Asian Earth Sciences	144	54-68
2250	GA-250-1	CAOB	Southwest Mongolia	98.0000	44.5000	Rhyolite		329	329	11.78	62.20	0.1145	0.512784		-0.42	2.85	6.10	6.30	6.30	0.57	0.56	0.57	0.56	0.58	Zhu M S et al.	2017	Journal of Asian Earth Sciences	144	54-68
2251	GA-245	CAOB	Southwest Mongolia	98.0000	44.5000	Rhyolite		329	329	15.17	78.67	0.1166	0.512798		-0.41	3.12	6.30	6.48	6.48	0.56	0.55	0.56	0.55	0.56	Zhu M S et al.	2017	Journal of Asian Earth Sciences	144	54-68
2252	GA-244	CAOB	Southwest Mongolia	98.0000	44.5000	Rhyolite		329	329	16.52	84.15	0.1187	0.512787		-0.40	2.91	6.00	6.18	6.18	0.58	0.58	0.58	0.58	0.58	Zhu M S et al.	2017	Journal of Asian Earth Sciences	144	54-68
2253	GA-243	CAOB	Southwest Mongolia	98.0000	44.5000	Rhyolite		329	329	16.19	93.18	0.1051	0.512780		-0.47	2.77	6.40	6.62	6.62	0.55	0.52	0.55	0.52	0.58	Zhu M S et al.	2017	Journal of Asian Earth Sciences	144	54-68
2254	06XJ182*	CAOB	Western Junggar	84.8236	45.7022	Granitoid		310	310	3.43	16.30	0.1285	0.512922		-0.35	5.54	8.24	8.24	8.24	0.40	0.41	0.40	0.41	0.40	Tang GJ et al.	2012	Lithos	140-141	86-102
2255	06XJ181	CAOB	Western Junggar	84.8236	45.7022	Granitoid		310	310	2.96	12.00	0.1496	0.512965		-0.24	6.38	8.24	8.24	8.24	0.40	0.44	0.40	0.44	0.40	Tang GJ et al.	2012	Lithos	140-141	86-102
2256	06XJ179*	CAOB	Western Junggar	84.8236	45.7022	Granitoid		310	310	3.08	16.00	0.1168	0.512889		-0.41	4.90	8.06	8.06	8.06	0.41	0.41	0.41	0.41	0.41	Tang GJ et al.	2012	Lithos	140-141	86-102

2257	06XJ178	CAOB	Western Junggar	84.8236	45.7022	Granitoid		310	310	2.10	10.60	0.1201	0.512863		-0.39	4.39	7.42	7.42	7.42	0.46	0.47	0.46	0.47	0.46	Tang GJ et al.	2012	Lithos	140-141	86-102
2258	06XJ176	CAOB	Western Junggar	84.8236	45.7022	Granitoid		310	310	2.82	13.50	0.1271	0.512881		-0.35	4.74	7.49	7.49	7.49	0.46	0.47	0.46	0.47	0.46	Tang GJ et al.	2012	Lithos	140-141	86-102
2259	06XJ174*	CAOB	Western Junggar	84.8236	45.7022	Granitoid		310	310	2.64	13.20	0.1218	0.512852		-0.38	4.17	7.14	7.14	7.14	0.49	0.49	0.49	0.49	0.49	Tang GJ et al.	2012	Lithos	140-141	86-102
2260	06XJ173	CAOB	Western Junggar	84.8236	45.7022	Granitoid		310	310	4.51	17.70	0.1551	0.512987		-0.21	6.81	8.45	8.45	8.45	0.38	0.42	0.38	0.42	0.38	Tang GJ et al.	2012	Lithos	140-141	86-102
2261	06XJ170*	CAOB	Western Junggar	84.8236	45.7022	Granitoid		310	310	3.55	16.00	0.1350	0.512917		-0.31	5.44	7.88	7.88	7.88	0.43	0.45	0.43	0.45	0.43	Tang GJ et al.	2012	Lithos	140-141	86-102
2262	06XJ167	CAOB	Western Junggar	84.8236	45.7022	Granitoid		310	310	3.18	13.80	0.1400	0.512969		-0.29	6.46	8.70	8.70	8.70	0.36	0.38	0.36	0.38	0.36	Tang GJ et al.	2012	Lithos	140-141	86-102
2263	06XJ136*	CAOB	Western Junggar	84.8236	45.7022	Granitoid		310	310	4.43	19.80	0.1362	0.512900		-0.31	5.11	7.50	7.50	7.50	0.46	0.49	0.46	0.49	0.46	Tang GJ et al.	2012	Lithos	140-141	86-102
2264	06XJ134	CAOB	Western Junggar	84.8236	45.7022	Diorite dike		309	309	2.91	11.90	0.1485	0.512939		-0.25	5.87	7.77	7.77	7.77	0.43	0.49	0.43	0.49	0.44	Tang GJ et al.	2012	Lithos	140-141	86-102
2265	06XJ131*	CAOB	Western Junggar	84.8236	45.7022	Bi granite		312	312	1.97	12.50	0.0964	0.512819		-0.51	3.53	7.52	7.52	7.52	0.46	0.43	0.46	0.43	0.46	Tang GJ et al.	2012	Lithos	140-141	86-102
2266	06XJ129	CAOB	Western Junggar	84.8236	45.7022	Granitoid		310	310	2.36	10.00	0.1439	0.512906		-0.27	5.23	7.32	7.32	7.32	0.47	0.53	0.47	0.53	0.47	Tang GJ et al.	2012	Lithos	140-141	86-102
2267	06XJ127*	CAOB	Western Junggar	84.8236	45.7022	Granitoid		310	310	3.11	15.00	0.1266	0.512898		-0.36	5.07	7.84	7.84	7.84	0.43	0.44	0.43	0.44	0.43	Tang GJ et al.	2012	Lithos	140-141	86-102
2268	06XJ125	CAOB	Western Junggar	84.8236	45.7022	Granitoid		310	310	3.07	14.10	0.1327	0.512909		-0.33	5.29	7.82	7.82	7.82	0.43	0.45	0.43	0.45	0.43	Tang GJ et al.	2012	Lithos	140-141	86-102
2269	06XJ123	CAOB	Western Junggar	84.8236	45.7022	Granite porphyry dike		304	304	12.60	37.80	0.2024	0.513014		0.03	7.33	7.11	7.11	7.11	0.48	1.83	0.48	1.83	0.48	Tang GJ et al.	2012	Lithos	140-141	86-102
2270	06XJ122	CAOB	Western Junggar	84.8236	45.7022	granitoid		310	310	2.65	10.90	0.1485	0.512921		-0.24	5.52	7.43	7.43	7.43	0.46	0.54	0.46	0.54	0.46	Tang GJ et al.	2012	Lithos	140-141	86-102
2271	06XJ121-1-2	CAOB	Western Junggar	84.8236	45.7022	Monzonite MME		309	309	12.40	44.30	0.1711	0.513015		-0.13	7.35	8.36	8.36	8.36	0.39	0.48	0.39	0.48	0.39	Tang GJ et al.	2012	Lithos	140-141	86-102
2272	06XJ121-1-1*	CAOB	Western Junggar	84.8236	45.7022	Granitoid		310	310	6.29	32.80	0.1167	0.512884		-0.41	4.80	7.96	7.96	7.96	0.42	0.42	0.42	0.42	0.42	Tang GJ et al.	2012	Lithos	140-141	86-102
2273	06XJ120*	CAOB	Western Junggar	84.8236	45.7022	Granitoid		310	310	7.37	35.60	0.1258	0.512929		-0.36	5.68	8.48	8.48	8.48	0.38	0.38	0.38	0.38	0.38	Tang GJ et al.	2012	Lithos	140-141	86-102
2274	06XJ118	CAOB	Western Junggar	84.8236	45.7022	Granitoid		310	310	7.17	33.70	0.1295	0.512949		-0.34	6.07	8.73	8.73	8.73	0.36	0.36	0.36	0.36	0.36	Tang GJ et al.	2012	Lithos	140-141	86-102
2275	S-21**	CAOB	Western Junggar	82.4750	45.7875	Granitoid		295	295	2.28	12.00	0.1149	0.512797		-0.42	3.10	6.20	6.18	6.18	0.55	0.55	0.55		0.55	Yang M et al.	2015	Acta Petrologica Sinica	31(02)	523-533
2276	S-19**	CAOB	Western Junggar	82.4750	45.7875	Granitoid		295	295	3.22	16.20	0.1202	0.512743		-0.39	2.05	4.90	4.93	4.93	0.66	0.66	0.66		0.66	Yang M et al.	2015	Acta Petrologica Sinica	31(02)	523-533
2277	S-16**	CAOB	Western Junggar	82.4750	45.7875	Granitoid		295	295	2.55	14.30	0.1078	0.512645		-0.45	0.14	3.50	3.48	3.48	0.77	0.73	0.77		0.77	Yang M et al.	2015	Acta Petrologica Sinica	31(02)	523-533
2278	S-12**	CAOB	Western Junggar	82.4750	45.7875	Granitoid		295	295	1.44	6.31	0.1380	0.512813		-0.30	3.41	5.60	5.62	5.62	0.60	0.68	0.60		0.60	Yang M et al.	2015	Acta Petrologica Sinica	31(02)	523-533
2279	14NM99	CAOB	King'an Mongolia Orogenic Bel	112.9620	43.1923	Dacite		310	310	5.67	19.80	0.1727	0.513004	6	-0.12	7.14	8.06	8.09	8.09	0.41	0.54	0.41	0.54	0.41	Pang CJ et al.	2016	Lithos	261	55-71
2280	14NM98	CAOB	King'an Mongolia Orogenic Bel	112.9620	43.1923	Andesite		310	310	5.04	15.00	0.2028	0.513027	5	0.03	7.59	7.36	7.35	7.35	0.47	1.72	0.47	1.72	0.47	Pang CJ et al.	2016	Lithos	261	55-71
2281	MZ13-9	CAOB	South Tianshan	82.5333	40.2667	Garnet-bearing rhyolite		300	300	7.06	41.70	0.1693	0.511985		-0.14	-12.74	-11.7	-11.7	-11.7	2.00	3.96	2.00		2.01	Cheng ZG et al.	2017	Lithos	282-283	373-387
2282	MZ13-8	CAOB	South Tianshan	82.5333	40.2667	Garnet-bearing rhyolite		300	300	6.49	38.50	0.1686	0.511968		-0.14	-13.07	-12.0	-12.0	-12.0	2.02	3.95	2.02		2.03	Cheng ZG et al.	2017	Lithos	282-283	373-387
2283	MZ13-7	CAOB	South Tianshan	82.5333	40.2667	Garnet-bearing rhyolite		300	300	6.57	37.50	0.1752	0.511946		-0.11	-13.50	-12.7	-12.7	-12.7	2.07	4.71	2.07		2.09	Cheng ZG et al.	2017	Lithos	282-283	373-387
2284	MZ13-4	CAOB	South Tianshan	82.5333	40.2667	Garnet-bearing rhyolite		280	280	6.76	38.00	0.1779	0.511933		-0.10	-13.75	-13.1	-13.1	-13.1	2.08	5.11	2.08		2.11	Cheng ZG et al.	2017	Lithos	282-283	373-387
2285	MZ13-3	CAOB	South Tianshan	82.5333	40.2667	Garnet-bearing rhyolite		280	280	6.58	38.90	0.1692	0.511950		-0.14	-13.42	-12.4	-12.4	-12.4	2.04	4.06	2.04		2.05	Cheng ZG et al.	2017	Lithos	282-283	373-387
2286	MZ13-20	CAOB	South Tianshan	82.5333	40.2667	Garnet-bearing rhyolite		280	280	7.63	43.80	0.1742	0.511950		-0.11	-13.42	-12.6	-12.6	-12.6	2.05	4.58	2.05		2.07	Cheng ZG et al.	2017	Lithos	282-283	373-387
2287	MZ13-14	CAOB	South Tianshan	82.5333	40.2667	Garnet-bearing rhyolite		300	300	6.22	35.40	0.1757	0.511960		-0.11	-13.23	-12.4	-12.4	-12.4	2.05	4.72	2.05		2.07	Cheng ZG et al.	2017	Lithos	282-283	373-387
2288	MZ13-12	CAOB	South Tianshan	82.5333	40.2667	Garnet-bearing rhyolite		300	300	6.23	37.60	0.1657	0.511970		-0.16	-13.03	-11.9	-11.8	-11.8	2.01	3.71	2.01		2.02	Cheng ZG et al.	2017	Lithos	282-283	373-387
2289	LHS-5B	CAOB	NE China	130.9241	43.6861	Basaltic andesite		14	150	2.79	8.55	0.1975	0.512710		0.00	1.40	1.40	1.39	1.39	0.81	4.10	0.81			Yu S Y et al.	2018	Journal of Asian Earth Sciences	164	260-273
2290	LHS-2	CAOB	NE China	130.9241	43.6861	Basaltic andesite		14	150	2.90	9.01	0.1946	0.512686		-0.01	0.94	0.94	0.98	0.98	0.85	3.67	0.85			Yu S Y et al.	2018	Journal of Asian Earth Sciences	164	260-273
2291	08WLG-17	CAOB	Altai, unit 7	89.1861	46.2750	Monzogranite	Saertielieke	308	308	9.64	54.37	0.1072	0.512746	7	-0.46	2.11	5.62	5.62	5.62	0.61	0.58	0.61	0.72	0.58	Shen XM et al.	2011	Journal of Asian Earth Sciences	42(5)	986-1007
2292	08WLG-16	CAOB	Altai, unit 7	89.1667	46.3167	Diorite	Saertielieke	308	308	7.36	41.60	0.1070	0.512749	7	-0.46	2.17	5.69	5.69	5.69	0.60	0.57	0.60	0.72	0.57	Shen XM et al.	2011	Journal of Asian Earth Sciences	42(5)	986-1007
2293	08WLG-13	CAOB	Altai, unit 7	89.1333	46.3194	Alkaline granite	Saertielieke	308	308	8.07	38.72	0.1260	0.512769	8	-0.36	2.56	5.33	5.33	5.33	0.63	0.66	0.63	0.72	0.66	Shen XM et al.	2011	Journal of Asian Earth Sciences	42(5)	986-1007
2294	08WLG-07	CAOB	Altai, unit 7	89.3833	46.2500	Monzogranite	Jierdekala	291	291	12.90	62.29	0.1252	0.512809	6	-0.36	3.34	5.99	5.99	5.99	0.57	0.59	0.57	0.72	0.59	Shen XM et al.	2011	Journal of Asian Earth Sciences	42(5)	986-1007
2295	08QH-29	CAOB	Altai, unit 6	90.8833	46.2000	Qtz monzonite	North-kouan	367	367	6.83	33.45	0.1235	0.512797	7	-0.37	3.10	6.53	6.53	6.53	0.58	0.60	0.58	0.72	0.58	Shen XM et al.	2011	Journal of Asian Earth Sciences	42(5)	986-1007
2296	08QH-27	CAOB	Altai, unit 6	90.9269	46.1347	Alkali feldspar granite	East-kouan	367	367	4.75	24.44	0.1174	0.512809	6	-0.40	3.34	7.05	7.05	7.05	0.54	0.54	0.54	0.72	0.54	Shen XM et al.	2011	Journal of Asian Earth Sciences	42(5)	986-1007
2297	08QH-26	CAOB	Altai, unit 6	90.7333	46.1833	Monzogranite	East-kouan	382	382	6.88	35.24	0.1180	0.512795	8	-0.40	3.06	6.90	6.90	6.90	0.57	0.57	0.57	0.72	0.57	Shen XM et al.	2011	Journal of Asian Earth Sciences	42(5)	986-1007
2298	08QH-25	CAOB	Altai, unit 6	90.7333	46.1833	Granodiorite	East-kouan	382	382	7.46	30.79	0.1466	0.512880	8	-0.25	4.72	7.16	7.16	7.16	0.54	0.61	0.54	0.72	0.54	Shen XM et al.	2011	Journal of Asian Earth Sciences	42(5)	986-1007
2299	08QH-24	CAOB	Altai, unit 6	90.7333	46.1833	Alkali feldspar granite	East-kouan	382	382	7.78	31.72	0.1483	0.512911	7	-0.25	5.33	7.68	7.68	7.68	0.50	0.56	0.50	0.72	0.50	Shen XM et al.	2011	Journal of Asian Earth Sciences	42(5)	986-1007
2300	DL10-8 /4	CAOB	SMXAB	112.8417	44.7100	Moyite	Dalaimiao	160	160			0.1158	0.512663	11	-0.41	0.49	2.15	2.14	2.14	0.77	0.76	0.77	0.76	0.78	Xue FH et al.	2015	Acta Petrologica Sinica	31(6)	1774-1788
2301	DL10-8 /3	CAOB	SMXAB	112.8417	44.7100	Moyite	Dalaimiao	160	160			0.1254	0.512682	11	-0.36	0.86	2.32	2.31	2.31	0.76	0.81	0.76	0.81	0.76	Xue FH et al.	2015	Acta Petrologica Sinica	31(6)	1774-1788
2302	EGN-20	CAOB	NE China	121.5000	50.0000	altic andesites/trachyandes	Murui Farm	126	150	9.66	54.06	0.1081	0.51258		-0.45	-1.13	0.45	0.56	0.30	0.89	0.82	0.89			Fan WM et al.	2003	ournal of Volcanology and Geothermal Researc	121(1-2)	115-135
2303	EGN-34	CAOB	NE China	121.5000	50.0000	altic andesites/trachyandes	Murui Farm	139	150	8.25	43.61	0.1140	0.512609		-0.42	-0.57	0.32	1.02	0.90	0.86	0.83	0.86			Fan WM et al.	2003	ournal of Volcanology and Geothermal Researc	121(1-2)	115-135
2304	EGN-21	CAOB	NE China	121.5000	50.0000	altic andesites/trachyandes	Murui Farm	126	150	9.81	55.47	0.1070	0.512559		-0.46	-1.54	0.06	0.18	-0.09	0.92	0.84	0.92			Fan WM et al.	2003	ournal of Volcanology and Geothermal Researc	121(1-2)	115-135
2305	EGN-32	CAOB	NE China	121.5000	50.0000	altic andesites/trachyandes	Murui Farm	139	150	9.6	50.86	0.1141	0.512579		-0.42	-1.15	0.32	0.43	0.31	0.90	0.87	0.90			Fan WM et al.	2003	ournal of Volcanology and Geothermal Researc	121(1-2)	115-135
2306	HLR-19	CAOB	NE China	120.5000	49.5000	altic andesites/trachyandes	Qiyi Prairie	130	150	6.18	30.92	0.1209	0.512602		-0.39	-0.70	0.55	0.75	0.56	0.88	0.90	0.88			Fan WM et al.	2003	ournal of Volcanology and Geothermal Researc	121(1-2)	115-135
2307	HLR-11	CAOB	NE China	120.5000	49.5000	altic andesites/trachyandes	Qiyi Prairie	130	150	6.55	31.76	0.1252	0.512621		-0.36	-0.33	0.85	1.04	0.86	0.85	0.91	0.85			Fan WM et al.	2003	ournal of Volcanology and Geothermal Researc	121(1-2)	115-135
2308	EGN-30	CAOB	NE China	121.5000	50.0000	altic andesites/trachyandes	Murui Farm	126	150	9.46	49.15	0.1163	0.512571		-0.41	-1.31	0.13	0.23	-0.01	0.92	0.91	0.92			Fan WM et al.	2003	ournal of Volcanology and Geothermal Researc	121(1-2)	115-135
2309	EGN-26	CAOB	NE China	121.5000	50.0000	altic andesites/trachyandes	Murui Farm	126	150	11.65	61.33	0.1148	0.512523		-0.42	-2.24	-0.78	-0.68	-0.92	0.99	0.97	0.99			Fan WM et al.	2003	ournal of Volcanology and Geothermal Researc	121(1-2)	115-135
2310	GH-10	CAOB	NE China	121.5200	50.7800	rnblende andesites/trachyt	Genhe	127	150	4.93	23.18	0.1287	0.512645		-0.35	0.14	1.24	1.44	1.24	0.82	0.91	0.82			Fan WM et al.	2003	ournal of Volcanology and Geothermal Researc	121(1-2)	115-135
2311	GH-16	CAOB	NE China	121.5200	50.7800	rnblende andesites/trachyt	Genhe	127	150	4.74	24.13	0.1187	0.512637		-0.40	-0.02	1.24	1.47	1.25	0.82	0.82	0.82			Fan WM et al.	2003	ournal of Volcanology and Geothermal Researc	121(1-2)	115-135
2312	GH-19	CAOB	NE China	121.5200	50.7800	rnblende andesites/trachyt	Genhe	127	150	4.76	23.41	0.1230	0.512621		-0.37	-0.33	0.86	1.08	0.87	0.85	0.89	0.85			Fan WM et al.	2003	ournal of Volcanology and Geothermal Researc	121(1-2)	115-135
2313	GH-28	CAOB	NE China	121.5200	50.7800	rnblende andesites/trachyt	Genhe	127	150	4.91	23.73	0.1250	0.512643		-0.36	0.10	1.26	1.47	1.26	0.82	0.87	0.82			Fan WM et al.	2003	ournal of Volcanology and Geothermal Researc	121(1-2)	115-135
2314	EGN-14	CAOB	central Kazakhstan	120.5000	48.5000	Rhyolite	Halar	116	150	7.63	40.38	0.1142	0.512583		-0.42	-1.07	0.15	0.51	0.51	0.90	0.87	0.90			Fan WM et al.	2003	ournal of Volcanology and Geothermal Researc	121(1-2)	115-135
2315	EGN-12	CAOB	central Kazakhstan	120.5000	48.5000	Rhyolite	Halar	116	150	6.44	33.43	0.1164	0.512590		-0.41	-0.94	0.25	0.60	0.60	0.89	0.88	0.89			Fan WM et al.	2003	ournal of Volcanology and Geothermal Researc	121(1-2)	115-135
2316	YTH-8	CAOB	central Kazakhstan	121.7200	50.4800	Rhyolite	Tulihe	116	150	4.39	23.30	0.1140	0.512566		-0.42	-1.40	-0.18	0.18	0.18	0.92	0.89	0.92			Fan WM et al.	2003	ournal of Volcanology and Geothermal Researc	121(1-2)	115-135
2317	YTH-1	CAOB	central Kazakhstan	121.7200	50.4800	Rhyolite	Tulihe	116	150	4.64	23.53	0.1192	0.512558		-0.39	-1.56	-0.41	-0.08	-0.08	0.94	0.95	0.94			Fan WM et al.	2003	ournal of Volcanology and Geothermal Researc	121(1-2)	115-135
2318	LYB19-2	CAOB	Beishan	95.5000	41.1000	Adakite		420	420	3.62	21.20	0.1034	0.512337		-0.47	-5.87	-0.90	-0.87	-0.87	1.23	1.12	1.23		0.42	Mao Q G et al.	2012	Gondwana Research	22（2）	534-553
2319	08LY03-06	CAOB	Beishan	95.5000	41.1000	Adakite		374	374	2.63	17.20	0.0924	0.512422		-0.53	-4.21	0.80	0.76	0.76	1.06	0.91	1.06		0.37	Mao Q G et al.	2012	Gondwana Research	22（2）	534-553
2320	08LY03-02	CAOB	Beishan	95.5000	41.1000	Adakite		374	374	2.71	17.50	0.0938	0.512415		-0.52	-4.35	0.60	0.56	0.56	1.08	0.93	1.08		0.37	Mao Q G et al.	2012	Gondwana Research	22（2）	534-553
2321	08LY01-01	CAOB	Beishan	95.5000	41.1000	Dacite		440	440	5.02	29.30	0.1169	0.512482		-0.41	-3.04	1.20	1.44	1.44	1.06	1.05	1.06		0.45	Mao Q G et al.	2012	Gondwana Research	22（2）	534-553
2322	08L14	CAOB	Beishan	95.5000	41.1000	Adakite		374	374	2.39	15.80	0.0916	0.512499		-0.53	-2.71	2.30	2.30	2.30	0.93	0.81	0.93		0.37	Mao Q G et al.	2012	Gondwana Research	22（2）	534-553
2323	08L06	CAOB	Beishan	95.5000	41.1000	Adakite		374	374	2.44	15.80	0.0932	0.512578		-0.53	-1.17	3.80	3.77	3.77	0.82	0.72	0.82		0.37	Mao Q G et al.	2012	Gondwana Research	22（2）	534-553
2324	08L01	CAOB	Beishan	95.5000	41.1000	Adakite		374	374	2.38	15.10	0.0952	0.512535		-0.52	-2.01	2.80	2.83	2.83	0.89	0.79	0.89		0.37	Mao Q G et al.	2012	Gondwana Research	22（2）	534-553
2325	A03-2	CAOB	Eastern Junggar	90.0000	46.0000	Adakite	Suoerkuduke	375	375	3.665	17.37	0.1276	0.512676		-0.35	0.74	4.06	4.05	**4.05**	0.79	0.84	0.79			Zhang HX et al.	2004	Geological Journal of China Universities	10(1)	106-113
2326	A06-3	CAOB	Eastern Junggar	90.0000	46.0000	Adakite	Suoerkuduke	375	375	4.277	20.2	0.1280	0.512624		-0.35	-0.27	3.02	3.01	**3.01**	0.88	0.94	0.88			Zhang HX et al.	2004	Geological Journal of China Universities	10(1)	106-113
2327	A10-2	CAOB	Eastern Junggar	90.0000	46.0000	Adakite	Suoerkuduke	375	375	3.507	1.023	0.1265	0.512644		-0.36	0.12	3.48	3.48	3.48	0.84	0.88	0.84			Zhang HX et al.	2004	Geological Journal of China Universities	10(1)	106-113
2328	YXAn-8	CAOB	Western Tianshan	84.4983	43.4660	Basaltic andesite	Yuximolegai	311	311	3.52	14.20	0.1496	0.512728	0	-0.24	1.76	3.70	3.62	3.62	0.77	1.00	0.77	1.00	0.78	Yang WB et al.	2012	Gondwana Research	22(1)	325-340
2329	YX8-7	CAOB	Western Tianshan	84.4983	43.4660	Qtz diorite	Yuximolegai	311	311	3.64	15.00	0.1469	0.512652	0	-0.25	0.27	2.30	2.25	2.25	0.89	1.14	0.89	1.14	0.89	Yang WB et al.	2012	Gondwana Research	22(1)	325-340
2330	YX8-5	CAOB	Western Tianshan	84.4983	43.4660	Qtz diorite	Yuximolegai	311	311	3.88	16.70	0.1404	0.512697	0	-0.29	1.15	3.40	3.38	3.38	0.79	0.94	0.79	0.94	0.80	Yang WB et al.	2012	Gondwana Research	22(1)	325-340
2331	YX8-4	CAOB	Western Tianshan	84.4983	43.4660	Qtz diorite	Yuximolegai	311	311	3.56	16.00	0.1344	0.512689	0	-0.32	0.99	3.50	3.47	3.47	0.79	0.89	0.79	0.89	0.79	Yang WB et al.	2012	Gondwana Research	22(1)	325-340
2332	YX8-3	CAOB	Western Tianshan	84.4983	43.4660	Qtz diorite	Yuximolegai	311	311	4.04	17.70	0.1381	0.512662	0	-0.30	0.47	2.80	2.79	2.79	0.84	0.98	0.84	0.98	0.84	Yang WB et al.	2012	Gondwana Research	22(1)	325-340
2333	YX8-1	CAOB	Western Tianshan	84.4983	43.4660	Qtz diorite	Yuximolegai	311	311	3.91	17.30	0.1368	0.512650	0	-0.30	0.23	2.60	2.61	2.61	0.86	0.99	0.86	0.99	0.86	Yang WB et al.	2012	Gondwana Research	22(1)	325-340
2334	YX3-3	CAOB	Western Tianshan	84.4983	43.4660	Basaltic andesite	Yuximolegai	311	311	3.27	14.50	0.1362	0.512794	0	-0.31	3.04	5.50	5.44	5.44	0.63	0.70	0.63	0.70	0.63	Yang WB et al.	2012	Gondwana Research	22(1)	325-340
2335	YX2-7	CAOB	Western Tianshan	84.4983	43.4660	Basaltic andesite	Yuximolegai	311	311	3.76	15.70	0.1447	0.512782	0	-0.26	2.81	4.90	4.87	4.87	0.67	0.81	0.67	0.81	0.68	Yang WB et al.	2012	Gondwana Research	22(1)	325-340
2336	YX2-6	CAOB	Western Tianshan	84.4983	43.4660	K-rich trachyandesite	Yuximolegai	311	311	4.22	18.80	0.1361	0.512791	0	-0.31	2.98	5.40	5.39	5.39	0.63	0.71	0.63	0.71	0.63	Yang WB et al.	2012	Gondwana Research	22(1)	325-340
2337	YX2-5	CAOB	Western Tianshan	84.4983	43.4660	K-rich trachyandesite	Yuximolegai	311	311	4.82	20.60	0.1411	0.512786	0	-0.28	2.89	5.10	5.09	5.09	0.66	0.76	0.66	0.77	0.66	Yang WB et al.	2012	Gondwana Research	22(1)	325-340
2338	YX2-2	CAOB	Western Tianshan	84.4983	43.4660	Basaltic andesite	Yuximolegai	311	311	3.99	16.90	0.1427	0.512804	0	-0.27	3.24	5.40	5.38	5.38	0.63	0.74	0.63	0.74	0.63	Yang WB et al.	2012	Gondwana Research	22(1)	325-340
2339	YX2-1	CAOB	Western Tianshan	84.4983	43.4660	K-rich trachyandesite	Yuximolegai	311	311	3.86	17.40	0.1342	0.512763	0	-0.32	2.44	5.00	4.92	4.92	0.67	0.74	0.67	0.74	0.67	Yang WB et al.	2012	Gondwana Research	22(1)	325-340
2340	YX08G6	CAOB	Western Tianshan	84.4983	43.4660	Qtz diorite	Yuximolegai	311	311	4.46	19.90	0.1355	0.512665	0	-0.31	0.53	3.00	2.95	2.95	0.83	0.95	0.83	0.95	0.83	Yang WB et al.	2012	Gondwana Research	22(1)	325-340
2341	YX08G5	CAOB	Western Tianshan	84.4983	43.4660	Qtz diorite	Yuximolegai	311	311	4.46	19.00	0.1416	0.512682	0	-0.28	0.86	3.10	3.04	3.04	0.82	0.99	0.82	0.99	0.82	Yang WB et al.	2012	Gondwana Research	22(1)	325-340
2342	YXQ-7	CAOB	Tianshan	84.5000	43.4500	Quartz syenite porphyry		284	284	9.45	46.50	0.1230	0.512700		-0.37	1.21	4.00	3.88	3.88	0.73	0.76	0.73	0.76	0.73	Yang W B et al.	2012	Gondwana Research	22(1)	325-340
2343	YXQ-6	CAOB	Tianshan	84.5000	43.4500	Quartz syenite porphyry		284	284	9.64	46.50	0.1252	0.512681		-0.36	0.84	3.50	3.43	3.43	0.77	0.81	0.77	0.81	0.77	Yang W B et al.	2012	Gondwana Research	22(1)	325-340
2344	YXQ-4	CAOB	Tianshan	84.5000	43.4500	Quartz syenite porphyry		284	284	8.60	42.80	0.1215	0.512700		-0.38	1.21	4.00	3.93	3.93	0.73	0.74	0.73	0.75	0.73	Yang W B et al.	2012	Gondwana Research	22(1)	325-340
2345	YXQ-3	CAOB	Tianshan	84.5000	43.4500	Quartz syenite porphyry		284	284	10.40	54.80	0.1151	0.512675		-0.41	0.72	3.80	3.68	3.68	0.75	0.73	0.75	0.74	0.75	Yang W B et al.	2012	Gondwana Research	22(1)	325-340
2346	YXQ-2	CAOB	Tianshan	84.5000	43.4500	Quartz syenite porphyry		284	284	8.90	44.20	0.1217	0.512694		-0.38	1.09	3.90	3.81	3.81	0.74	0.76	0.74	0.76	0.74	Yang W B et al.	2012	Gondwana Research	22(1)	325-340
2347	YXQ-1	CAOB	Tianshan	84.5000	43.4500	Quartz syenite porphyry		284	284	8.56	41.60	0.1244	0.512690		-0.37	1.01	3.70	3.63	3.63	0.75	0.79	0.75	0.79	0.75	Yang W B et al.	2012	Gondwana Research	22(1)	325-340
2348	Xh080919-5(5)	CAOB	Balkhash metallogenic belt	79.8333	47.0050	Granite		331	331	3.08	17.10	0.1089	0.512738		-0.45	1.95	5.44	5.66	5.44	0.64	0.60	0.63		0.64	Liu G et al.	2012	Acta Petrologica Sinica	6（2）	288-293
2349	Xh080919-5(4)	CAOB	Balkhash metallogenic belt	79.9833	46.9667	Granite		331	331	2.92	17.00	0.1038	0.512731		-0.47	1.81	5.53	5.74	5.53	0.64	0.58	0.62		0.64	Liu G et al.	2012	Acta Petrologica Sinica	6（2）	288-293
2350	Xh080919-4(1)	CAOB	Balkhash metallogenic belt	79.9000	46.9500	Granite		331	331	4.14	17.50	0.1430	0.512815		-0.27	3.45	5.51	5.72	5.51	0.64	0.72	0.62		0.64	Liu G et al.	2012	Acta Petrologica Sinica	6（2）	288-293
2351	Xh080918-2(1)	CAOB	Balkhash metallogenic belt	77.1983	47.0300	Granite		310	310	1.57	9.81	0.0968	0.512753		-0.51	2.24	5.80	6.20	5.80	0.58	0.52	0.56		0.58	Liu G et al.	2012	Acta Petrologica Sinica	6（2）	288-293
2352	Xh080917-9(2)	CAOB	Balkhash metallogenic belt	77.2833	46.9833	Granite		310	310	3.93	18.20	0.1305	0.512815		-0.34	3.45	5.76	6.07	5.76	0.59	0.61	0.57		0.59	Liu G et al.	2012	Acta Petrologica Sinica	6（2）	288-293
2353	Xh080917-6(4)	CAOB	Balkhash metallogenic belt	77.4000	46.9833	Granite		310	310	2.85	13.60	0.1267	0.512810		-0.36	3.36	5.91	6.12	5.91	0.59	0.60	0.57		0.59	Liu G et al.	2012	Acta Petrologica Sinica	6（2）	288-293
2354	Xh080917-6(2)	CAOB	Balkhash metallogenic belt	77.4000	46.9833	Granite		310	310	3.28	16.70	0.1187	0.512782		-0.40	2.81	5.94	5.89	5.94	0.61	0.59	0.59		0.61	Liu G et al.	2012	Acta Petrologica Sinica	6（2）	288-293

2355	Xh080917-12(1)	CAOB	Balkhash metallogenic belt	77.2833	46.9667	Diorite		310	310	2.48	12.70	0.1181	0.512736		-0.40	1.91	4.82	5.02	4.82	0.68	0.66	0.66		0.68	Liu G et al.	2012	Acta Petrologica Sinica	6（2）	288-293
2356	Xh080917-11(1)	CAOB	Balkhash metallogenic belt	77.1983	47.0300	Granite		310	310	2.55	12.40	0.1243	0.512756		-0.37	2.30	4.95	5.16	4.95	0.67	0.67	0.65		0.67	Liu G et al.	2012	Acta Petrologica Sinica	6（2）	288-293
2357	Xh080915-5(5)	CAOB	Balkhash metallogenic belt	74.2150	47.5583	Granite		295	295	1.01	8.35	0.0731	0.512529		-0.63	-2.13	2.56	2.53	2.56	0.86	0.67	0.85		0.86	Liu G et al.	2012	Acta Petrologica Sinica	6（2）	288-293
2358	Xh080915-5(3)	CAOB	Balkhash metallogenic belt	74.2150	47.5583	Granite		295	295	4.23	26.60	0.0961	0.512526		-0.51	-2.18	1.57	1.60	1.57	0.94	0.81	0.93		0.94	Liu G et al.	2012	Acta Petrologica Sinica	6（2）	288-293
2359	Xh080915-4(1)	CAOB	Balkhash metallogenic belt	74.2050	47.5667	Granite		295	295	1.87	10.10	0.1119	0.512522		-0.43	-2.26	0.71	0.93	0.71	1.66	0.94	0.98		1.66	Liu G et al.	2012	Acta Petrologica Sinica	6（2）	288-293
2360	Xh080915-1(1)	CAOB	Balkhash metallogenic belt	74.0017	47.7133	Granite		295	295	2.18	12.60	0.1046	0.512088		-0.47	-10.73	-5.87	-7.26	-5.87	1.14	1.48	1.65		1.14	Liu G et al.	2012	Acta Petrologica Sinica	6（2）	288-293
2361	Xh080914-9(1)	CAOB	Balkhash metallogenic belt	74.0000	48.0000	Granite		289	289	3.22	17.30	0.1125	0.512424		-0.43	-4.17	-1.06	-1.07	-1.06	1.16	1.09	1.14		1.16	Liu G et al.	2012	Acta Petrologica Sinica	6（2）	288-293
2362	Xh080914-2(1)	CAOB	Balkhash metallogenic belt	74.0300	47.7267	Granite		289	289	5.49	28.60	0.1161	0.512449		-0.41	-3.69	-0.72	-0.71	-0.72	1.13	1.09	1.11		1.13	Liu G et al.	2012	Acta Petrologica Sinica	6（2）	288-293
2363	Xh080914-10(2)	CAOB	Balkhash metallogenic belt	74.0000	48.0000	Diorite		289	289	3.23	18.80	0.1039	0.512419		-0.47	-4.27	-0.81	-0.85	-0.81	1.14	1.01	1.12		1.14	Liu G et al.	2012	Acta Petrologica Sinica	6（2）	288-293
2364	Xh080914-10(1)	CAOB	Balkhash metallogenic belt	74.0000	48.0000	Granite		289	289	1.23	9.14	0.0814	0.512472		-0.59	-3.24	1.11	1.02	1.11	1.98	0.78	0.97		1.98	Liu G et al.	2012	Acta Petrologica Sinica	6（2）	288-293
2365	Xh080912-9(3)	CAOB	Balkhash metallogenic belt	74.7114	47.1958	Granite		316	316	3.55	17.70	0.1213	0.512502		-0.38	-2.65	0.09	0.39	0.09	1.06	1.07	1.04		1.06	Liu G et al.	2012	Acta Petrologica Sinica	6（2）	288-293
2366	Xh080912-9(2)	CAOB	Balkhash metallogenic belt	74.7114	47.1958	Granite		316	316	3.81	21.80	0.1057	0.512435		-0.46	-3.96	-0.46	-0.29	-0.46	1.12	1.01	1.10		1.12	Liu G et al.	2012	Acta Petrologica Sinica	6（2）	288-293
2367	Xh080912-2(1)	CAOB	Balkhash metallogenic belt	74.1817	47.1483	Granodiorite		300	300	5.84	30.30	0.1165	0.512428		-0.41	-4.10	-1.12	-1.03	-1.12	1.16	1.13	1.14		1.16	Liu G et al.	2012	Acta Petrologica Sinica	6（2）	288-293
2368	Xh080911-3(1)	CAOB	Balkhash metallogenic belt	74.8950	47.6017	Granodiorite		327	327	3.50	19.90	0.1063	0.512594		-0.46	-0.86	2.58	2.92	2.58	0.87	0.79	0.85		0.87	Liu G et al.	2012	Acta Petrologica Sinica	6（2）	288-293
2369	Xh080911-26(1)	CAOB	Balkhash metallogenic belt	75.1200	47.8400	Granodiorite		300	300	8.08	48.30	0.1011	0.512599		-0.49	-0.76	2.85	2.90	2.85	0.84	0.75	0.83		0.84	Liu G et al.	2012	Acta Petrologica Sinica	6（2）	288-293
2370	Xh080911-24(1)	CAOB	Balkhash metallogenic belt	75.1083	47.8767	Granodiorite		300	300	0.82	7.62	0.0651	0.512529		-0.67	-2.13	2.88	2.91	2.88	0.83	0.64	0.82		0.83	Liu G et al.	2012	Acta Petrologica Sinica	6（2）	288-293
2371	Xh080911-1(1)	CAOB	Balkhash metallogenic belt	74.7733	47.6383	Granodiorite		327	327	4.86	21.60	0.1360	0.512630		-0.31	-0.16	1.89	2.38	1.89	0.90	1.02	0.89		0.90	Liu G et al.	2012	Acta Petrologica Sinica	6（2）	288-293
2372	Xh080910-7(1)	CAOB	Balkhash metallogenic belt	74.9833	46.9972	Granodiorite		327	327	3.40	17.70	0.1161	0.512512		-0.41	-2.46	0.53	0.91	0.53	1.03	1.00	1.01		1.03	Liu G et al.	2012	Acta Petrologica Sinica	6（2）	288-293
2373	Xh080910-2(1)	CAOB	Balkhash metallogenic belt	74.9833	46.9833	Granite		327	327	3.38	17.60	0.1161	0.512472		-0.41	-3.24	-0.07	0.13	-0.07	1.09	1.06	1.07		1.09	Liu G et al.	2012	Acta Petrologica Sinica	6（2）	288-293
2374	Xh080910-10(1)	CAOB	Balkhash metallogenic belt	74.9667	46.9833	Granodiorite		327	327	1.98	15.40	0.0777	0.512509		-0.60	-2.52	1.83	2.45	1.83	0.91	0.72	0.88		0.91	Liu G et al.	2012	Acta Petrologica Sinica	6（2）	288-293
2375	Zg64-04a	CAOB	Transbaikalia and Mongolia	111.1667	55.3333	Gneiss-granite		325	325	3.75	22.03	0.1029	0.511781		-0.48	-16.72	-12.8	-12.8	-12.8	2.12	1.88	2.12	1.88	2.12	Tsygankov AA.	2014	Russian Geology & Geophysics	55（2）	153-176
2376	Zg64/3-04	CAOB	Transbaikalia and Mongolia	113.7000	54.4167	Gneiss-granite		325	325	0.86	4.30	0.1204	0.512148		-0.39	-9.56	-6.39	-6.39	-6.39	1.60	1.63	1.60	1.63	1.60	Tsygankov AA.	2014	Russian Geology & Geophysics	55（2）	153-176
2377	Zg51/2-03	CAOB	Transbaikalia and Mongolia	111.0000	54.5000	Gneiss-granite		325	325	1.44	7.00	0.1247	0.512093		-0.37	-10.63	-7.64	-7.64	-7.64	1.70	1.80	1.70	1.80	1.70	Tsygankov AA.	2014	Russian Geology & Geophysics	55（2）	153-176
2378	Xc-59-02a	CAOB	Transbaikalia and Mongolia	106.5000	51.3333	Monzonite		283	283	7.78	46.21	0.1018	0.512114		-0.48	-10.22	-6.79	-6.79	-6.79	1.60	1.41	1.60	1.41	1.60	Tsygankov AA.	2014	Russian Geology & Geophysics	55（2）	153-176
2379	Te-44-02	CAOB	Transbaikalia and Mongolia	100.1667	54.3333	Porph. granite		318	318	1.43	7.71	0.1117	0.512090		-0.43	-10.69	-7.24	-7.24	-7.24	1.66	1.58	1.66	1.58	1.66	Tsygankov AA.	2014	Russian Geology & Geophysics	55（2）	153-176
2380	Sh-07-25	CAOB	Transbaikalia and Mongolia	114.0000	54.0000	Qtz syenite		300	300	4.37	21.30	0.1241	0.512201		-0.37	-8.52	-5.75	-5.75	-5.75	1.53	1.61	1.53	1.61	1.53	Tsygankov AA.	2014	Russian Geology & Geophysics	55（2）	153-176
2381	PR-050-04a	CAOB	Transbaikalia and Mongolia	113.8333	56.3000	Qtz monzonite		278	278	3.98	27.26	0.0883	0.512273		-0.55	-7.12	-3.28	-3.28	-3.28	1.31	1.07	1.31	1.07	1.31	Tsygankov AA.	2014	Russian Geology & Geophysics	55（2）	153-176
2382	Kh-62a-02a	CAOB	Transbaikalia and Mongolia	109.5000	55.7000	Qtz syenite		302	302	4.86	31.69	0.0926	0.512220		-0.53	-8.15	-4.14	-4.14	-4.14	1.40	1.17	1.40	1.17	1.40	Tsygankov AA.	2014	Russian Geology & Geophysics	55（2）	153-176
2383	Kh-62-02	CAOB	Transbaikalia and Mongolia	109.5000	55.7000	Qtz monzonite		302	302	4.22	24.00	0.1063	0.512223		-0.46	-8.10	-4.61	-4.61	-4.61	1.44	1.31	1.44	1.31	1.44	Tsygankov AA.	2014	Russian Geology & Geophysics	55（2）	153-176
2384	Kh-6-03	CAOB	Transbaikalia and Mongolia	113.8333	56.3000	Qtz monzonite		302	302	6.27	35.54	0.1066	0.512093		-0.46	-10.63	-7.16	-7.16	-7.16	1.64	1.50	1.64	1.50	1.64	Tsygankov AA.	2014	Russian Geology & Geophysics	55（2）	153-176
2385	Hk-12-06a	CAOB	Transbaikalia and Mongolia	100.1667	54.0000	Porph. granite		318	318	3.17	20.82	0.0922	0.512154		-0.53	-9.44	-5.20	-5.20	-5.20	1.50	1.25	1.50	1.25	1.50	Tsygankov AA.	2014	Russian Geology & Geophysics	55（2）	153-176
2386	Gl-8/3-03	CAOB	Transbaikalia and Mongolia	111.0000	55.0000	Granite		313	313	2.41	13.17	0.1105	0.512152		-0.44	-9.48	-6.04	-6.04	-6.04	1.56	1.47	1.56	1.47	1.56	Tsygankov AA.	2014	Russian Geology & Geophysics	55（2）	153-176
2387	Gl-5/3-03	CAOB	Transbaikalia and Mongolia	100.3333	54.1667	Granite		313	313	3.75	22.79	0.0996	0.512149		-0.49	-9.54	-5.66	-5.66	-5.66	1.53	1.34	1.53	1.34	1.53	Tsygankov AA.	2014	Russian Geology & Geophysics	55（2）	153-176
2388	Bu-114-06a	CAOB	Transbaikalia and Mongolia	113.8333	56.3000	Qtz syenite		287	287	5.12	31.41	0.0986	0.512129		-0.50	-9.93	-6.34	-6.34	-6.34	1.57	1.35	1.57	1.35	1.57	Tsygankov AA.	2014	Russian Geology & Geophysics	55（2）	153-176
2389	B341	CAOB	Transbaikalia and Mongolia	106.5000	51.3333	Syenite		280	280	11.00	68.00	0.0978	0.512246		-0.50	-7.65	-4.11	-4.11	-4.11	1.38	1.19	1.38	1.19	1.38	Tsygankov AA.	2014	Russian Geology & Geophysics	55（2）	153-176
2390	543/4-04a	CAOB	Transbaikalia and Mongolia	114.3333	54.4000	Granite		293	293	9.03	30.17	0.1810	0.512056		-0.08	-11.35	-10.8	-10.8	-10.8	1.91	5.03	1.91	5.03	1.91	Tsygankov AA.	2014	Russian Geology & Geophysics	55（2）	153-176
2391	177-05a	CAOB	Transbaikalia and Mongolia	110.0000	52.1667	Qtz syenite		300	300	5.87	32.69	0.1086	0.512539		-0.45	-1.93	1.44	1.44	1.44	0.94	0.89	0.94	0.89	0.94	Tsygankov AA.	2014	Russian Geology & Geophysics	55（2）	153-176
2392	174-05a	CAOB	Transbaikalia and Mongolia	110.0000	52.1667	Granite		300	300	4.60	27.21	0.1023	0.512490		-0.48	-2.89	0.73	0.73	0.73	1.00	0.90	1.00	0.90	1.00	Tsygankov AA.	2014	Russian Geology & Geophysics	55（2）	153-176
2393	166-05b	CAOB	Transbaikalia and Mongolia	104.1667	50.5000	Granite		286	286	6.24	37.50	0.1006	0.512141		-0.49	-9.69	-6.19	-6.19	-6.19	1.55	1.36	1.55	1.36	1.55	Tsygankov AA.	2014	Russian Geology & Geophysics	55（2）	153-176
2394	162-05b	CAOB	Transbaikalia and Mongolia	110.0000	52.1667	Granite		286	286	16.60	96.00	0.1045	0.512105		-0.47	-10.40	-7.03	-7.03	-7.03	1.62	1.46	1.62	1.46	1.62	Tsygankov AA.	2014	Russian Geology & Geophysics	55（2）	153-176
2395	162/6-05b	CAOB	Transbaikalia and Mongolia	114.0000	54.4000	Granite		286	286	6.31	32.90	0.1160	0.512093		-0.41	-10.63	-7.68	-7.68	-7.68	1.67	1.64	1.67	1.64	1.67	Tsygankov AA.	2014	Russian Geology & Geophysics	55（2）	153-176
2396	162/4-05b	CAOB	Transbaikalia and Mongolia	104.1667	50.5000	Granite		286	286	5.53	31.60	0.1058	0.512114		-0.46	-10.22	-6.90	-6.90	-6.90	1.61	1.46	1.61	1.46	1.61	Tsygankov AA.	2014	Russian Geology & Geophysics	55（2）	153-176
2397	044/1-04	CAOB	Transbaikalia and Mongolia	104.1667	50.5000	Granite		303	303	2.10	12.01	0.1056	0.512178		-0.46	-8.97	-5.45	-5.45	-5.45	1.51	1.37	1.51	1.37	1.51	Tsygankov AA.	2014	Russian Geology & Geophysics	55（2）	153-176
2398	002/1-04	CAOB	Transbaikalia and Mongolia	114.0000	54.4000	Granite		303	303	1.96	11.78	0.1004	0.512163		-0.49	-9.27	-5.54	-5.54	-5.54	1.51	1.33	1.51	1.33	1.51	Tsygankov AA.	2014	Russian Geology & Geophysics	55（2）	153-176
2399	SL14-017	CAOB	Tianshan	91.4857	41.9677	Granodiorite		307	307	3.38	14.00	0.1459	0.512806		-0.26	3.28	5.28	5.27	5.27	0.64	0.77	0.64		0.77	Zhao LD et al.	2018	Gondwana Research	93(18)	231-234
2400	SL14-016	CAOB	Tianshan	91.1027	41.4340	Diorite		329	329	5.84	23.10	0.1528	0.512884		-0.22	4.80	6.65	6.64	6.64	0.54	0.67	0.54		0.67	Zhao LD et al.	2018	Gondwana Research	93(18)	231-234
2401	SL14-015	CAOB	Tianshan	91.0610	41.4592	Granodiorite		307	307	3.05	12.90	0.1429	0.512879		-0.27	4.70	6.81	6.80	6.80	0.51	0.58	0.51		0.58	Zhao LD et al.	2018	Gondwana Research	93(18)	231-234
2402	SL14-004-2	CAOB	Tianshan	91.0610	41.4592	Monzogranite		313	313	4.05	15.90	0.1540	0.512869		-0.22	4.52	6.23	6.22	6.22	0.56	0.72	0.56		0.72	Zhao LD et al.	2018	Gondwana Research	93(18)	231-234
2403	SL14-004-1	CAOB	Tianshan	91.0610	41.4592	Monzogranite		313	313	4.52	20.30	0.1346	0.512842		-0.32	3.98	6.46	6.46	6.46	0.55	0.59	0.55		0.59	Zhao LD et al.	2018	Gondwana Research	93(18)	231-234
2404	SL14-003	CAOB	Tianshan	91.1027	41.4340	Diorite		323	323	3.76	14.90	0.1525	0.512898		-0.22	5.07	6.90	6.89	6.89	0.52	0.63	0.52		0.63	Zhao LD et al.	2018	Gondwana Research	93(18)	231-234
2405	SL14-001-2	CAOB	Tianshan	91.1063	41.4488	Diorite		323	323	3.11	12.30	0.1528	0.512877		-0.22	4.67	6.49	6.48	6.48	0.55	0.68	0.55		0.68	Zhao LD et al.	2018	Gondwana Research	93(18)	231-234
2406	XJ22-8	CAOB	NE China	91.0000	46.5000	Andesite	Beitashan	388	388	2.98	13.16	0.1367	0.512839		-0.31	3.92	6.89	6.89	6.89	0.57	0.62	0.57			Zhao Z C et al.	2009	Gondwana Research	264	201-215
2407	A1022	CAOB	NE China	91.0000	46.5000	Basalt Andesite	Tuoranggekeuduke	406	406	3.51	16.76	0.1265	0.512644		-0.36	0.12	3.70	3.75	3.75	0.84	0.88	0.84			Zhao Z C et al.	2009	Gondwana Research	264	201-215
2408	D0925	CAOB	MOS	104.9958	46.1461	Granodiorite	296 Intrusion	350	350	4.27	26.91	0.0959	0.512319	4	-0.51	-6.22	-1.72	-1.72	-1.72	1.24	1.07	1.24		1.26	Ariuntsetseg G et al.	2021	Lithos	404-405	106455
2409	D1709	CAOB	MOS	104.7706	47.0790	Rhyolite	Batkhaan	274	274	4.46	19.19	0.1403	0.512673	3	-0.29	0.68	2.65	2.65	2.65	0.82	0.99	0.82		0.84	Ariuntsetseg G et al.	2021	Lithos	404-405	106455
2410	D1710	CAOB	MOS	104.1339	47.0684	Syenogranite	Batkhaan	282	282	4.71	21.14	0.1271	0.512719	2	-0.35	1.57	4.08	4.08	4.08	0.71	0.76	0.71		0.73	Ariuntsetseg G et al.	2021	Lithos	404-405	106455
2411	D1726	CAOB	MOS	104.3372	46.9385	Granodiorite	Zambalkhudag	220	220	4.93	19.62	0.1518	0.512537	2	-0.23	-1.97	-0.71	-0.71	-0.71	1.05	1.51	1.05		1.07	Ariuntsetseg G et al.	2021	Lithos	404-405	106455
2412	D1742	CAOB	MOS	104.6233	46.6705	Monzogranite	Zambalkhudag	240	240	2.65	20.11	0.0797	0.512539	4	-0.60	-1.93	1.65	1.65	1.65	0.88	0.70	0.88		0.89	Ariuntsetseg G et al.	2021	Lithos	404-405	106455
2413	FX06-74	NCC	NCC	121.6617	42.3367	Granite	Ahalai	257	257	2.20	14.86	0.0893	0.511642	15	-0.55	-19.43	-15.9	-15.9	-15.9	2.32	1.84	2.32	1.85	2.32	Zhang XH et al.	2012	Lithos	155	201-217
2414	FX06-65	NCC	NCC	121.6531	42.3331	Appinite	Ahalai	257	257	5.42	21.86	0.1500	0.512096	14	-0.24	-10.57	-9.04	-9.04	-9.04	1.76	2.51	1.76	2.52	1.76	Zhang XH et al.	2012	Lithos	155	201-217
2415	FX06-62	NCC	NCC	121.6531	42.3331	Appinite	Ahalai	257	257	5.18	20.51	0.1528	0.512086	12	-0.22	-10.77	-9.33	-9.33	-9.33	1.78	2.65	1.78	2.66	1.79	Zhang XH et al.	2012	Lithos	155	201-217
2416	FX06-59	NCC	NCC	121.6531	42.3331	Appinite	Ahalai	257	257	8.25	36.09	0.1381	0.511946	12	-0.30	-13.50	-11.6	-11.6	-11.6	1.96	2.42	1.96	2.42	1.97	Zhang XH et al.	2012	Lithos	155	201-217
2417	FX06-57	NCC	NCC	121.6531	42.3331	Appinite	Ahalai	257	257	7.70	32.91	0.1414	0.511920	11	-0.28	-14.01	-12.2	-12.2	-12.2	2.01	2.58	2.01	2.59	2.02	Zhang XH et al.	2012	Lithos	155	201-217
2418	FX06-54	NCC	NCC	121.6439	42.2933	Appinite	Tiejianggeleng	252	252	3.73	14.61	0.1543	0.512140	13	-0.22	-9.71	-8.4	-8.4	-8.4	1.70	2.58	1.70	2.59	1.70	Zhang XH et al.	2012	Lithos	155	201-217
2419	FX06-52	NCC	NCC	121.6439	42.2933	Appinite	Tiejianggeleng	252	252	8.84	47.05	0.1136	0.512001	13	-0.42	-12.43	-9.75	-9.75	-9.75	1.81	1.75	1.81	1.75	1.82	Zhang XH et al.	2012	Lithos	155	201-217
2420	FX06-23	NCC	NCC	121.7922	42.2617	Appinite	Shichang	256	256	2.41	15.48	0.0939	0.511634	12	-0.52	-19.58	-16.2	-16.2	-16.2	2.34	1.92	2.34	1.93	2.34	Zhang XH et al.	2012	Lithos	155	201-217
2421	FX06-22	NCC	NCC	121.7922	42.2617	Appinite	Shichang	256	256	2.91	21.27	0.0827	0.511628	11	-0.58	-19.70	-16.0	-16.0	-16.0	2.32	1.77	2.32	1.77	2.32	Zhang XH et al.	2012	Lithos	155	201-217
2422	FX06-21	NCC	NCC	121.7922	42.2617	Appinite	Shichang	256	256	1.89	10.46	0.1092	0.511693	13	-0.44	-18.43	-15.6	-15.6	-15.6	2.29	2.12	2.29	2.12	2.29	Zhang XH et al.	2012	Lithos	155	201-217
2423	FX06-20	NCC	NCC	121.7922	42.2617	Appinite	Shichang	254	254	6.85	35.75	0.1158	0.511624	13	-0.41	-19.78	-17.2	-17.2	-17.2	2.41	2.36	2.41	2.37	2.42	Zhang XH et al.	2012	Lithos	155	201-217
2424	FX06-19	NCC	NCC	121.7922	42.2617	Appinite	Shichang	254	254	7.26	38.23	0.1148	0.511863	11	-0.42	-15.12	-12.5	-12.5	-12.5	2.03	1.98	2.03	1.98	2.04	Zhang XH et al.	2012	Lithos	155	201-217
2425	FX06-18	NCC	NCC	121.7922	42.2617	Appinite	Shichang	254	254	8.41	42.54	0.1195	0.511827	14	-0.39	-15.82	-13.3	-13.3	-13.3	2.10	2.13	2.10	2.14	2.11	Zhang XH et al.	2012	Lithos	155	201-217
2426	FX06-15	NCC	NCC	121.7922	42.2617	Appinite	Shichang	254	254	5.67	24.11	0.1421	0.511907	13	-0.28	-14.26	-12.5	-12.5	-12.5	2.03	2.63	2.03	2.64	2.04	Zhang XH et al.	2012	Lithos	155	201-217
2427	KY12-33-4	NCC	NCC	124.2567	42.5825	Andesite		256	256	4.50	24.50	0.1110	0.512477		-0.44	-3.14	-0.40	-0.34	-0.34	1.05	1.00	1.05	1.00	1.06	Yuan LL et al.	2016	Lithos	258	58-76
2428	KY12-33-3	NCC	NCC	124.2567	42.5825	Andesite		256	256	4.30	23.30	0.1114	0.512502		-0.43	-2.65	1.00	0.13	0.13	1.01	0.97	1.01	0.97	1.02	Yuan LL et al.	2016	Lithos	258	58-76
2429	KY12-32-3	NCC	NCC	124.2425	42.5842	Andesite		256	256	4.70	23.00	0.1227	0.512627		-0.38	-0.21	2.20	2.20	2.20	0.85	0.88	0.85	0.88	0.85	Yuan LL et al.	2016	Lithos	258	58-76
2430	FK06-45	NCC	NMNCC	123.4453	42.4208	Monzonite	Faku	260	260	5.25	32.17	0.0987	0.512452	13	-0.50	-3.63	-0.38	-0.38	-0.38	1.06	0.93	1.06	0.93	1.06	Zhang XH et al.	2010	Lithos	117(1-4)	283-306
2431	FK06-44	NCC	NMNCC	123.4453	42.4208	Granite	Faku	250	250	21.09	118.22	0.1078	0.512548	13	-0.45	-1.76	1.09	1.08	1.08	0.93	0.87	0.93	0.87	0.94	Zhang XH et al.	2010	Lithos	117(1-4)	283-306
2432	FK06-40	NCC	NMNCC	123.4528	42.3642	Granite	Faku	250	250	13.52	59.70	0.1369	0.512575	13	-0.30	-1.23	0.68	0.68	0.68	0.96	1.14	0.96	1.14	0.97	Zhang XH et al.	2010	Lithos	117(1-4)	283-306
2433	FK06-39	NCC	NMNCC	123.4528	42.3642	Granite	Faku	250	250	8.71	49.72	0.1059	0.512579	12	-0.46	-1.15	1.75	1.75	1.75	0.88	0.81	0.88	0.81	0.88	Zhang XH et al.	2010	Lithos	117(1-4)	283-306
2434	FK06-35	NCC	NMNCC	123.4850	42.3706	Monzodiorite	Faku	260	260	6.21	31.53	0.1190	0.512429	14	-0.40	-4.08	-1.51	-1.50	-1.50	1.15	1.16	1.15	1.16	1.15	Zhang XH et al.	2010	Lithos	117(1-4)	283-306
2435	FK06-32	NCC	NMNCC	123.4850	42.3706	Monzodiorite	Faku	260	260	6.24	31.51	0.1196	0.512413	15	-0.39	-4.39	-1.84	-1.83	-1.83	1.18	1.19	1.18	1.20	1.18	Zhang XH et al.	2010	Lithos	117(1-4)	283-306
2436	FK06-31	NCC	NMNCC	123.4850	42.3706	Monzodiorite	Faku	260	260	6.03	30.80	0.1185	0.512415	9	-0.40	-4.35	-1.76	-1.76	-1.76	1.17	1.18	1.17	1.18	1.17	Zhang XH et al.	2010	Lithos	117(1-4)	283-306
2437	FK06-24	NCC	NMNCC	123.4933	42.3894	Granite	Faku	260	260	6.45	34.31	0.1136	0.512468	13	-0.42	-3.32	-0.55	-0.56	-0.56	1.07	1.04	1.07	1.04	1.08	Zhang XH et al.	2010	Lithos	117(1-4)	283-306
2438	FK06-18	NCC	NMNCC	123.4861	42.4042	Granite	Faku	250	250	11.65	55.23	0.1275	0.512497	14	-0.35	-2.75	-0.55	-0.54	-0.54	1.06	1.15	1.06	1.16	1.07	Zhang XH et al.	2010	Lithos	117(1-4)	283-306
2439	FK04-53	NCC	NMNCC	123.4233	42.4708	Granite	Faku	250	250	3.34	15.70	0.1287	0.512574	12	-0.35	-1.25	0.91	0.92	0.92	0.95	1.03	0.95	1.04	0.95	Zhang XH et al.	2010	Lithos	117(1-4)	283-306
2440	FK04-52	NCC	NMNCC	123.4233	42.4708	Granite	Faku	250	250	4.63	30.64	0.0915	0.512495	14	-0.53	-2.79	0.56	0.57	0.57	0.97	0.82	0.97	0.82	0.98	Zhang XH et al.	2010	Lithos	117(1-4)	283-306
2441	FK04-37	NCC	NMNCC	123.3878	42.4267	Granite	Faku	250	250	5.17	24.58	0.1275	0.512587	12	-0.35	-0.99	1.21	1.21	1.21	0.92	1.00	0.92	1.00	0.92	Zhang XH et al.	2010	Lithos	117(1-4)	283-306
2442	FK04-3	NCC	NMNCC	123.5017	42.4542	Granite	Faku	250	250	2.42	7.15	0.2049	0.512721	13	0.04	1.62	1.36	1.36	1.36	0.89	7.28	0.89	7.38	0.91	Zhang XH et al.	2010	Lithos	117(1-4)	283-306
2443	FK04-24	NCC	NMNCC	123.4617	42.3897	Granite	Faku	250	250	3.13	19.71	0.0961	0.512483	12	-0.51	-3.02	0.19	0.19	0.19	1.01	0.86	1.01	0.87	1.01	Zhang XH et al.	2010	Lithos	117(1-4)	283-306
2444	FK04-23	NCC	NMNCC	123.4617	42.3897	Granite	Faku	250	250	3.19	18.48	0.1043	0.512518	18	-0.47	-2.34	0.60	0.61	0.61	0.97	0.88	0.97	0.88	0.97	Zhang XH et al.	2010	Lithos	117(1-4)	283-306
2445	FK04-19	NCC	NMNCC	123.4867	42.3825	Monzodiorite	Faku	260	260	4.51	20.27	0.1346	0.512662	14	-0.32	0.47	2.53	2.53	2.53	0.82	0.94	0.82	0.94	0.83	Zhang XH et al.	2010	Lithos	117(1-4)	283-306
2446	FK04-18	NCC	NMNCC	123.4867	42.3825	Granite	Faku	260	260	3.49	17.52	0.1207	0.512648	11	-0.39	0.20	2.73	2.72	2.72	0.81	0.82	0.81	0.83	0.81	Zhang XH et al.	2010	Lithos	117(1-4)	283-306
2447	FK04-16	NCC	NMNCC	123.4867	42.3825	Monzonite	Faku	260	260	6.32	34.21	0.1119	0.512528	11	-0.43	-2.15	0.68	0.67	0.67	0.97	0.93	0.97	0.93	0.98	Zhang XH et al.	2010	Lithos	117(1-4)	283-306
2448	FK04-12	NCC	NMNCC	123.4867	42.3825	Monzonite	Faku	260	260	6.73	37.09	0.1098	0.512503	16	-0.44	-2.63	0.25	0.25	0.25	1.01	0.95	1.01	0.95	1.01	Zhang XH et al.	2010	Lithos	117(1-4)	283-306
2449	FK04-11	NCC	NMNCC	123.4847	42.4019	Granite	Faku	250	250	6.19	31.94	0.1172	0.512625	13	-0.40	-0.25	2.28	2.28	2.28	0.83	0.83	0.83	0.83	0.84	Zhang XH et al.	2010	Lithos	117(1-4)	283-306
2450	WJ1136-3	CAOB	Western Junggar	85.8885	46.7619	Syenitic granite		407	407	4.13	22.37	0.1116	0.512747	0	-0.43	2.13	6.56	6.55	6.55	0.62	0.60	0.62		0.63	Yin J et al.	2017	Gondwana Research	43	55-73
2451	WJ1136-1	CAOB	Western Junggar	85.8885	46.7619	Syenitic granite		407	407	3.69	17.89	0.1247	0.512771	0	-0.37	2.59	6.34	6.33	6.33	0.63	0.65	0.63		0.67	Yin J et al.	2017	Gondwana Research	43	55-73
2452	WJ1132-4	CAOB	Western Junggar	87.6167	48.6590	Granodiorite		420	420	3.20	16.92	0.1144	0.512677	0	-0.42	0.76	5.18	5.17	5.17	0.74	0.73	0.74		0.76	Yin J et al.	2017	Gondwana Research	43	55-73

2453	WJ1132-3	CAOB	Western Junggar	86.6167	47.6590	Granodiorite		420	420	2.87	15.83	0.1095	0.512656	0	-0.44	0.35	5.03	5.02	5.02	0.75	0.72	0.75		0.77	Yin J et al.	2017	Gondwana Research	43	55-73
2454	WJ1132-1	CAOB	Western Junggar	85.6167	46.6590	Granodiorite		420	420	3.38	16.83	0.1213	0.512695	0	-0.38	1.11	5.16	5.15	5.15	0.74	0.75	0.74		0.76	Yin J et al.	2017	Gondwana Research	43	55-73
2455	WJ1128-4	CAOB	Western Junggar	85.4750	46.5790	Rhyolite		419	419	11.54	51.94	0.1342	0.512745	0	-0.32	2.09	5.44	5.43	5.43	0.72	0.78	0.72		0.72	Yin J et al.	2017	Gondwana Research	43	55-73
2456	WJ1128-1	CAOB	Western Junggar	85.4750	46.5790	Rhyolite		419	419	9.19	42.76	0.1299	0.512762	0	-0.34	2.42	6.00	5.99	5.99	0.67	0.71	0.67		0.67	Yin J et al.	2017	Gondwana Research	43	55-73
2457	WJ1126-3	CAOB	Western Junggar	85.4596	46.7080	Syenitic granite		418	418	4.01	21.87	0.1107	0.512604	0	-0.44	-0.66	3.93	3.92	3.92	0.84	0.81	0.84		0.84	Yin J et al.	2017	Gondwana Research	43	55-73
2458	WJ1126-1	CAOB	Western Junggar	85.4596	46.7080	Syenitic granite		418	418	3.76	20.00	0.1136	0.512636	0	-0.42	-0.04	4.40	4.39	4.39	0.80	0.78	0.80		0.80	Yin J et al.	2017	Gondwana Research	43	55-73
2459	WJ1125-4	CAOB	Western Junggar	85.5056	46.7106	K-feldspar granite		420	420	10.97	55.93	0.1185	0.512672	0	-0.40	0.66	4.86	4.85	4.85	0.76	0.77	0.76		0.77	Yin J et al.	2017	Gondwana Research	43	55-73
2460	13TL06	NCC	NCC	120.3783	40.0789	Adakitic granite	Taili	220	220			0.0980	0.511813	2	-0.50	-16.10	-13.4	-13.3	-13.3	2.08	1.76	2.08	1.76	2.08	Wang C et al.	2015	Lithos	212	353-367
2461	13TL05	NCC	NCC	120.3783	40.0789	Adakitic granite	Taili	220	220			0.0960	0.511827	4	-0.51	-15.83	-13.0	-13.0	-13.0	2.05	1.71	2.05	1.71	2.05	Wang C et al.	2015	Lithos	212	353-367
2462	13TL04	NCC	NCC	120.3783	40.0789	Adakitic granite	Taili	220	220			0.0960	0.511836	5	-0.51	-15.64	-12.8	-12.8	-12.8	2.04	1.70	2.04	1.70	2.04	Wang C et al.	2015	Lithos	212	353-367
2463	13TL03	NCC	NCC	120.3783	40.0789	Adakitic granite	Taili	220	220			0.0910	0.511816	3	-0.54	-16.03	-13.1	-13.1	-13.1	2.06	1.65	2.06	1.66	2.06	Wang C et al.	2015	Lithos	212	353-367
2464	13TL02	NCC	NCC	120.3783	40.0789	Adakitic granite	Taili	220	220			0.0980	0.511833	2	-0.50	-15.70	-12.9	-12.9	-12.9	2.05	1.73	2.05	1.73	2.05	Wang C et al.	2015	Lithos	212	353-367
2465	TSKH 1/8	CAOB	Mongolo-Transbaikalian	111.1700	51.5800	comendite	Tsagan-Hurtei Range	209	209	12.1	66.7	0.1100	0.512625		-0.44	-0.25		2.06	2.07	0.82	0.77	0.82			Litvinovsky BA et al.	2001	Russian Geology and Geophysics	42(3)	445-455
2466	TSKH 1/7	CAOB	Mongolo-Transbaikalian	111.1700	51.5800	Trachybasalt	Tsagan-Hurtei Range	209	209	7.22	37.4	0.1168	0.512646		-0.41	0.16		2.29	2.29	0.80	0.79	0.80			Litvinovsky BA et al.	2001	Russian Geology and Geophysics	42(3)	445-455
2467	TSKH 1/6	CAOB	Mongolo-Transbaikalian	111.1700	51.5800	Trachybasalt	Tsagan-Hurtei Range	209	209	6.09	30.9	0.1192	0.512632		-0.39	-0.12		1.95	1.96	0.83	0.84	0.83			Litvinovsky BA et al.	2001	Russian Geology and Geophysics	42(3)	445-455
2468	TSKH 1/1	CAOB	Mongolo-Transbaikalian	111.1700	51.5800	Trachybasalt	Tsagan-Hurtei Range	209	209	7.79	39.3	0.1198	0.512573		-0.39	-1.27		0.78	0.79	0.92	0.94	0.92			Litvinovsky BA et al.	2001	Russian Geology and Geophysics	42(3)	445-455
2469	11XL-5.4	CAOB	SMXAB	117.5233	43.9392	Granodiorite	Beidashan	277	277	6.97	30.90	0.1364	0.512689	6	-0.31	0.99	3.14	3.13	3.13	0.79	0.91	0.79	0.91	0.79	Li S et al.	2014	Gondwana Research	29(1)	168-180
2470	11XL-5.2	CAOB	SMXAB	117.5233	43.9392	Granodiorite	Beidashan	277	277	7.06	30.30	0.1409	0.512695	10	-0.28	1.11	3.10	3.08	3.08	0.79	0.95	0.79	0.96	0.79	Li S et al.	2014	Gondwana Research	29(1)	168-180
2471	11SH-3	CAOB	SMXAB	116.8944	43.7314	Granodiorite	Shahutong	275	275	5.89	26.90	0.1322	0.512561	8	-0.33	-1.50	0.76	0.76	0.76	0.98	1.10	0.98	1.10	0.98	Li S et al.	2014	Gondwana Research	29(1)	168-180
2472	11SH-2.1	CAOB	SMXAB	116.8944	43.7314	Granodiorite	Shahutong	275	275	4.72	21.50	0.1323	0.512504	8	-0.33	-2.61	-0.35	-0.35	-0.35	1.07	1.21	1.07	1.21	1.07	Li S et al.	2014	Gondwana Research	29(1)	168-180
2473	TL1071	NCC	NCC	114.8436	39.3917	Dioritic porphyrite	Mujicun	144	150			0.0943	0.511739	9	-0.52	-17.54	-15.7	-15.6	-15.6	2.20	1.80	2.20	1.80	2.21	Qu K et al.	2014	Geoscience	28(3)	449
2474	MJ1161	NCC	NCC	114.8436	39.3917	Dioritic porphyrite	Mujicun	144	150			0.0887	0.511775	7	-0.55	-16.83	-14.9	-14.8	-14.8	2.14	1.67	2.14	1.68	2.14	Qu K et al.	2014	Geoscience	28(3)	449
2475	MJ1010	NCC	NCC	114.8436	39.3917	Dioritic porphyrite	Mujicun	144	150			0.0985	0.511775	9	-0.50	-16.83	-15.0	-15.0	-15.0	2.15	1.81	2.15	1.82	2.16	Qu K et al.	2014	Geoscience	28(3)	449
2476	MJ1005	NCC	NCC	114.8436	39.3917	Dioritic porphyrite	Mujicun	144	150			0.0866	0.511677	6	-0.56	-18.75	-16.7	-16.6	-16.6	2.29	1.76	2.29	1.76	2.30	Qu K et al.	2014	Geoscience	28(3)	449
2477	GZ1029	NCC	NCC	114.8436	39.3917	Dioritic porphyrite	Mujicun	144	150			0.0922	0.511760	7	-0.53	-17.13	-15.2	-15.1	-15.1	2.17	1.74	2.17	1.74	2.17	Qu K et al.	2014	Geoscience	28(3)	449
2478	GJ10169	NCC	NCC	114.8436	39.3917	Dioritic porphyrite	Mujicun	144	150			0.0946	0.511747	8	-0.52	-17.38	-15.5	-15.4	-15.4	2.19	1.79	2.19	1.79	2.20	Qu K et al.	2014	Geoscience	28(3)	449
2479	12XTM10	NCC	NCC	120.0000	41.0000	Rhyolite		125	150	3.06	20.92	0.0930	0.511816		-0.53	-16.03	-14.4	-14.0	-14.0	2.08	1.68	2.08	1.68	2.09	Wang C et al.	2017	Lithos	292-293	437-451
2480	12XTM08	NCC	NCC	120.0000	41.0000	Rhyolite		125	150	3.09	21.08	0.0930	0.511720		-0.53	-17.91	-16.3	-15.9	-15.9	2.23	1.80	2.23	1.80	2.24	Wang C et al.	2017	Lithos	292-293	437-451
2481	12XTM05	NCC	NCC	120.0000	41.0000	Rhyolite		125	150	3.09	21.22	0.0920	0.511703		-0.53	-18.24	-16.6	-16.2	-16.2	2.26	1.81	2.26	1.81	2.26	Wang C et al.	2017	Lithos	292-293	437-451
2482	12XTM04	NCC	NCC	120.0000	41.0000	Rhyolite		125	150	3.20	21.98	0.0920	0.511720		-0.53	-17.91	-16.2	-15.9	-15.9	2.23	1.79	2.23	1.79	2.24	Wang C et al.	2017	Lithos	292-293	437-451
2483	12XTM03	NCC	NCC	120.0000	41.0000	Rhyolite		125	150	3.14	21.08	0.0950	0.511723		-0.52	-17.85	-16.2	-15.9	-15.9	2.23	1.83	2.23	1.82	2.24	Wang C et al.	2017	Lithos	292-293	437-451
2484	11XC12	NCC	NCC	120.0000	41.0000	Rhyolite		125	150	3.34	22.76	0.0930	0.511720		-0.53	-17.91	-16.2	-15.9	-15.9	2.23	1.80	2.23	1.80	2.24	Wang C et al.	2017	Lithos	292-293	437-451
2485	11XC11	NCC	NCC	120.0000	41.0000	Rhyolite		125	150	3.28	22.24	0.0940	0.511724		-0.52	-17.83	-16.2	-15.9	-15.9	2.23	1.81	2.23	1.81	2.23	Wang C et al.	2017	Lithos	292-293	437-451
2486	11XC10	NCC	NCC	120.0000	41.0000	Rhyolite		125	150	3.26	22.10	0.0940	0.511721		-0.52	-17.89	-16.3	-15.9	-15.9	2.23	1.81	2.23	1.81	2.24	Wang C et al.	2017	Lithos	292-293	437-451
2487	NM97	NCC	WNCC	110.3358	41.1191	Diorite	Guyang batholith	279	279	4.72	24.33	0.1171	0.511787	0	-0.40	-16.60	-13.6	-13.8	-13.8	2.16	2.14	2.16	2.14	2.15	Zhang XH et al.	2012	Lithos	125(1-2)	569-591
2488	NM96	NCC	WNCC	110.3349	41.1171	Diorite	Guyang batholith	279	279	4.02	19.56	0.1244	0.511798	0	-0.37	-16.39	-13.7	-13.8	-13.8	2.16	2.30	2.16	2.30	2.15	Zhang XH et al.	2012	Lithos	125(1-2)	569-591
2489	NM74	NCC	WNCC	110.6176	41.0859	Gabbroic diorite	Guyang batholith	280	280	6.77	35.68	0.1148	0.511981	0	-0.42	-12.82	-9.75	-9.89	-9.89	1.85	1.80	1.85	1.80	1.84	Zhang XH et al.	2012	Lithos	125(1-2)	569-591
2490	NM71	NCC	WNCC	110.6176	41.0692	Leuconorite	Guyang batholith	280	280	0.80	4.14	0.1168	0.512096	0	-0.41	-10.57	-7.58	-7.72	-7.72	1.67	1.65	1.67	1.65	1.67	Zhang XH et al.	2012	Lithos	125(1-2)	569-591
2491	NM68	NCC	WNCC	110.6176	41.0692	Leuconorite	Guyang batholith	280	280	0.76	3.85	0.1185	0.512094	0	-0.40	-10.61	-7.68	-7.82	-7.82	1.68	1.69	1.68	1.69	1.67	Zhang XH et al.	2012	Lithos	125(1-2)	569-591
2492	NM67	NCC	WNCC	110.6176	41.0692	Leuconorite	Guyang batholith	280	280	0.46	2.35	0.1179	0.512097	0	-0.40	-10.55	-7.60	-7.74	-7.74	1.67	1.67	1.67	1.67	1.67	Zhang XH et al.	2012	Lithos	125(1-2)	569-591
2493	NM62	NCC	WNCC	110.6193	41.0692	Gabbroic diorite	Guyang batholith	286	286	5.41	28.33	0.1154	0.512043	0	-0.41	-11.61	-8.49	-8.64	-8.64	1.75	1.71	1.75	1.71	1.75	Zhang XH et al.	2012	Lithos	125(1-2)	569-591
2494	NM59	NCC	WNCC	110.6193	41.0692	Gabbroic diorite	Guyang batholith	286	286	4.65	24.02	0.1170	0.512078	0	-0.41	-10.92	-7.87	-8.01	-8.01	1.70	1.69	1.70	1.69	1.69	Zhang XH et al.	2012	Lithos	125(1-2)	569-591
2495	NM58	NCC	WNCC	110.6193	41.0692	Gabbroic diorite	Guyang batholith	286	286	5.11	24.96	0.1237	0.512054	0	-0.37	-11.39	-8.58	-8.72	-8.72	1.76	1.85	1.76	1.85	1.75	Zhang XH et al.	2012	Lithos	125(1-2)	569-591
2496	NM56	NCC	WNCC	110.6193	41.0692	Gabbroic diorite	Guyang batholith	286	286	5.74	29.58	0.1173	0.512069	0	-0.40	-11.10	-8.06	-8.20	-8.20	1.72	1.71	1.72	1.71	1.71	Zhang XH et al.	2012	Lithos	125(1-2)	569-591
2497	NM181	NCC	WNCC	110.8341	41.1833	Diorite	Guyang batholith	279	279	5.40	30.33	0.1077	0.512047	0	-0.45	-11.53	-8.22	-8.36	-8.36	1.72	1.58	1.72	1.58	1.72	Zhang XH et al.	2012	Lithos	125(1-2)	569-591
2498	NM168	NCC	WNCC	110.8354	41.1352	Granite	Guyang batholith	282	282	1.72	10.10	0.1035	0.511888	0	-0.47	-14.63	-11.1	-11.3	-11.3	1.96	1.74	1.96	1.74	1.96	Zhang XH et al.	2012	Lithos	125(1-2)	569-591
2499	NM167	NCC	WNCC	110.8857	41.1357	Granodiorite	Guyang batholith	280	280	4.00	24.15	0.1002	0.511944	0	-0.49	-13.54	-9.94	-10.1	-10.1	1.87	1.62	1.87	1.62	1.86	Zhang XH et al.	2012	Lithos	125(1-2)	569-591
2500	NM162	NCC	WNCC	110.8675	41.1343	Granite	Guyang batholith	282	282	1.48	9.88	0.0904	0.511886	0	-0.54	-14.67	-10.7	-10.9	-10.9	1.93	1.56	1.93	1.56	1.92	Zhang XH et al.	2012	Lithos	125(1-2)	569-591
2501	NM156	NCC	WNCC	110.8840	41.1341	Granite	Guyang batholith	282	282	1.73	12.40	0.0843	0.511893	0	-0.57	-14.53	-10.4	-10.5	-10.5	1.90	1.48	1.90	1.48	1.90	Zhang XH et al.	2012	Lithos	125(1-2)	569-591
2502	NM152	NCC	WNCC	110.4525	41.1846	Granodiorite	Guyang batholith	280	280	3.09	19.42	0.0962	0.511822	0	-0.51	-15.92	-12.2	-12.3	-12.3	2.05	1.72	2.05	1.72	2.04	Zhang XH et al.	2012	Lithos	125(1-2)	569-591
2503	NM146	NCC	WNCC	110.4180	41.1690	Diorite	Guyang batholith	279	279	3.93	20.17	0.1178	0.511963	0	-0.40	-13.17	-10.2	-10.4	-10.4	1.88	1.88	1.88	1.88	1.88	Zhang XH et al.	2012	Lithos	125(1-2)	569-591
2504	NM144	NCC	WNCC	110.3834	41.1686	Granodiorite	Guyang batholith	280	280	4.22	21.13	0.1207	0.511913	0	-0.39	-14.14	-11.3	-11.4	-11.4	1.97	2.02	1.97	2.02	1.96	Zhang XH et al.	2012	Lithos	125(1-2)	569-591
2505	NM143	NCC	WNCC	110.3168	41.1686	Diorite	Guyang batholith	279	279	4.77	25.63	0.1126	0.511773	0	-0.43	-16.87	-13.7	-13.9	-13.9	2.17	2.07	2.17	2.07	2.16	Zhang XH et al.	2012	Lithos	125(1-2)	569-591
2506	NM138	NCC	WNCC	110.5355	41.0687	Dyke	Guyang batholith	276	276	5.13	25.88	0.1197	0.512258	0	-0.39	-7.41	-4.56	-4.70	-4.70	1.42	1.44	1.42	1.44	1.42	Zhang XH et al.	2012	Lithos	125(1-2)	569-591
2507	NM108	NCC	WNCC	110.4690	41.0838	Granodiorite	Guyang batholith	280	280	3.25	20.89	0.0942	0.511758	0	-0.52	-17.17	-13.36	-13.50	-13.50	2.14	1.77	2.14	1.77	2.14	Zhang XH et al.	2012	Lithos	125(1-2)	569-591
2508	NM100	NCC	WNCC	110.3358	41.1191	Diorite	Guyang batholith	279	279	3.60	17.03	0.1278	0.511802	0	-0.35	-16.31	-13.72	-13.85	-13.85	2.17	2.38	2.17	2.38	2.16	Zhang XH et al.	2012	Lithos	125(1-2)	569-591
2509	MG11-89-9	CAOB	Transbaikalia and Mongolia	100.0000	46.4167	Host granite		546	546	3.47	30.60	0.0687	0.512074		-0.65	-11.00	-2.08	-2.08	-2.08	1.43	1.13	1.43	1.13	1.43	Zhang YY et al.	2015	Journal of Asian Earth Sciences	113	353-368
2510	MG11-89-8	CAOB	Transbaikalia and Mongolia	100.0000	46.4167	MME		547	547	8.22	51.10	0.0972	0.512174		-0.51	-9.05	-2.11	-2.11	-2.11	1.43	1.28	1.43	1.28	1.43	Zhang YY et al.	2015	Journal of Asian Earth Sciences	113	353-368
2511	MG11-89-5	CAOB	Transbaikalia and Mongolia	100.0000	46.4167	MME		547	547	13.00	67.40	0.1168	0.512264		-0.41	-7.30	-1.72	-1.72	-1.72	1.40	1.39	1.40	1.39	1.40	Zhang YY et al.	2015	Journal of Asian Earth Sciences	113	353-368
2512	MG11-89-3	CAOB	Transbaikalia and Mongolia	100.0000	46.4167	MME		547	547	3.87	35.10	0.0667	0.512070		-0.66	-11.08	-2.01	-2.01	-2.01	1.43	1.12	1.43	1.12	1.43	Zhang YY et al.	2015	Journal of Asian Earth Sciences	113	353-368
2513	MG11-89-2	CAOB	Transbaikalia and Mongolia	100.0000	46.4167	MME		547	547	11.90	63.80	0.1124	0.512256		-0.43	-7.45	-1.57	-1.57	-1.57	1.39	1.34	1.39	1.34	1.39	Zhang YY et al.	2015	Journal of Asian Earth Sciences	113	353-368
2514	MG11-89-18	CAOB	Transbaikalia and Mongolia	100.0000	46.4167	Host granite		546	546	7.81	44.40	0.1062	0.512216		-0.46	-8.23	-1.93	-1.93	-1.93	1.42	1.32	1.42	1.32	1.42	Zhang YY et al.	2015	Journal of Asian Earth Sciences	113	353-368
2515	MG11-89-15	CAOB	Transbaikalia and Mongolia	100.0000	46.4167	Host granite		546	546	5.37	31.30	0.1038	0.512212		-0.47	-8.31	-1.84	-1.84	-1.84	1.41	1.30	1.41	1.30	1.41	Zhang YY et al.	2015	Journal of Asian Earth Sciences	113	353-368
2516	MG11-89-14	CAOB	Transbaikalia and Mongolia	100.0000	46.4167	Host granite		546	546	8.70	73.20	0.0718	0.512097		-0.63	-10.55	-1.85	-1.85	-1.85	1.41	1.13	1.41	1.13	1.41	Zhang YY et al.	2015	Journal of Asian Earth Sciences	113	353-368
2517	H-63	CAOB	SHB	120.3142	44.4036	Porphyry	Aolunhua	132	150	3.11	17.94	0.1099	0.512613	1	-0.44	-0.49	0.97	1.17	1.17	0.84	0.79	0.84	0.79	0.85	Ma X et al.	2013	Gondwana Research	24(3-4)	1152-1171
2518	H-56	CAOB	SHB	120.2994	44.3972	Porphyry	Aolunhua	132	150	1.22	5.88	0.1312	0.512605	1	-0.33	-0.64	0.47	0.61	0.61	0.89	1.01	0.89	1.01	0.89	Ma X et al.	2013	Gondwana Research	24(3-4)	1152-1171
2519	H-54	CAOB	SHB	120.2994	44.3972	Porphyry	Aolunhua	132	150	3.22	18.68	0.1094	0.512628	5	-0.44	-0.20	1.29	1.48	1.48	0.82	0.76	0.82	0.77	0.82	Ma X et al.	2013	Gondwana Research	24(3-4)	1152-1171
2520	H-32	CAOB	SHB	120.3058	44.4036	Porphyry	Aolunhua	132	150	3.02	17.15	0.1118	0.512612	7	-0.43	-0.51	0.91	1.12	1.12	0.85	0.81	0.85	0.81	0.85	Ma X et al.	2013	Gondwana Research	24(3-4)	1152-1171
2521	H-31	CAOB	SHB	120.3058	44.4036	porphyry	Aolunhua	132	150	3.21	19.67	0.1036	0.512631	5	-0.47	-0.14	1.44	1.65	1.65	0.80	0.72	0.80	0.72	0.81	Ma X et al.	2013	Gondwana Research	24(3-4)	1152-1171
2522	BNM08	NCC	NMNCC	112.7500	42.2208	Metadiorite	Baiyinduxi	438	438	3.80	18.00	0.1300	0.512539	8	-0.34	-1.93	1.80	1.80	1.80	1.03	1.11	1.03	1.12	1.03	Zhang W et al.	2013	Journal of Asian Earth Sciences	72	63-74
2523	BNM05	NCC	NMNCC	112.6072	42.1978	Diorite	Baiyinduxi	419	419	5.40	22.00	0.1500	0.512279	10	-0.24	-7.00	-4.52	-4.51	-4.51	1.52	2.08	1.52	2.08	1.53	Zhang W et al.	2013	Journal of Asian Earth Sciences	72	63-74
2524	Sh-9	CAOB	CMEB	107.6825	44.3431	Granodiorite	Shuteen	322	322	10.50	1.70	0.0990	0.512780		-0.50	2.77	6.79	6.78	6.78	0.53	0.49	0.53	0.49	0.53	Batkhishig B et al.	2010	Journal of Asian Earth Sciences	37(5-6)	399-411
2525	Sh-7	CAOB	CMEB	107.6825	44.3431	Granodiorite	Shuteen	322	322	18.20	3.90	0.1290	0.512853		-0.34	4.19	6.98	6.98	6.98	0.51	0.54	0.51	0.54	0.51	Batkhishig B et al.	2010	Journal of Asian Earth Sciences	37(5-6)	399-411
2526	Sh-6	CAOB	CMEB	107.6825	44.3431	Granodiorite	Shuteen	322	322	15.70	3.30	0.1290	0.512824		-0.34	3.63	6.42	6.41	6.41	0.56	0.59	0.56	0.59	0.56	Batkhishig B et al.	2010	Journal of Asian Earth Sciences	37(5-6)	399-411
2527	Sh-18	CAOB	CMEB	107.6825	44.3431	Granodiorite	Shuteen	322	322	13.60	2.50	0.1120	0.512733		-0.43	1.85	5.34	5.33	5.33	0.64	0.63	0.64	0.63	0.65	Batkhishig B et al.	2010	Journal of Asian Earth Sciences	37(5-6)	399-411
2528	Sh-17	CAOB	CMEB	107.6825	44.3431	Granodiorite	Shuteen	322	322	1.60	0.30	0.1180	0.512815		-0.40	3.45	6.69	6.69	6.69	0.53	0.53	0.53	0.54	0.54	Batkhishig B et al.	2010	Journal of Asian Earth Sciences	37(5-6)	399-411
2529	Sh-11	CAOB	CMEB	107.6825	44.3431	Granodiorite	Shuteen	322	322	6.60	1.40	0.1290	0.512806		-0.34	3.28	6.06	6.06	6.06	0.59	0.62	0.59	0.62	0.59	Batkhishig B et al.	2010	Journal of Asian Earth Sciences	37(5-6)	399-411
2530	Sh-0124	CAOB	CMEB	107.6825	44.3431	Granodiorite	Shuteen	322	322	14.60	3.00	0.1240	0.512792		-0.37	3.00	6.01	5.99	5.99	0.59	0.61	0.59	0.61	0.59	Batkhishig B et al.	2010	Journal of Asian Earth Sciences	37(5-6)	399-411
2531	Sh-0104	CAOB	CMEB	107.6825	44.3431	Granodiorite	Shuteen	322	322	13.80	3.00	0.1310	0.512820		-0.33	3.55	6.26	6.25	6.25	0.57	0.61	0.57	0.61	0.57	Batkhishig B et al.	2010	Journal of Asian Earth Sciences	37(5-6)	399-411
2532	Sh-0101	CAOB	CMEB	107.6825	44.3431	Granodiorite	Shuteen	322	322	13.90	2.40	0.1040	0.512748		-0.47	2.15	5.97	5.95	5.95	0.59	0.56	0.59	0.56	0.60	Batkhishig B et al.	2010	Journal of Asian Earth Sciences	37(5-6)	399-411
2533	WXT750	CAOB	Altai	88.0000	44.0000	Andesite		388	388	3.24	14.00	0.1411	0.512567		-0.28	-1.38	0.90	1.37	1.37	1.02	1.22	1.02		1.22	Zhao ZH et al.	2009	International Journal of Earth Sciences	98	1325-1340
2534	BB10-3	CAOB	NW China	87.0000	48.0000	Andesite		388	388	0.95	3.40	0.1681	0.512905		-0.15	5.21	6.60	6.62	6.62	0.59	0.82	0.59		0.53	Zhao ZH et al.	2009	International Journal of Earth Sciences	98	1325-1340
2535	AZ504-6	CAOB	NW China	87.0000	48.0000	Andesite		388	388	1.13	3.71	0.1753	0.512643		-0.11	0.10	0.28	1.16	1.16	1.04	2.01	1.04		0.93	Zhao ZH et al.	2009	International Journal of Earth Sciences	98	1325-1340
2536	AZ504-4	CAOB	NW China	87.0000	48.0000	Dacite		388	388	1.53	5.69	0.1720	0.512644		-0.13	0.12	0.44	1.34	1.34	1.02	1.84	1.02		0.92	Zhao ZH et al.	2009	International Journal of Earth Sciences	98	1325-1340
2537	2079	CAOB	NW China	88.0000	44.0000	Andesite		388	388	7.42	60.10	0.0746	0.512564		-0.62	-1.44	3.78	4.60	4.60	0.76	0.64	0.76		0.71	Zhao ZH et al.	2009	International Journal of Earth Sciences	98	1325-1340
2538	7580—3	CAOB	Transbaikalia and Mongolia	101.5333	45.7333	Tatsaingol complex		603	600	4.70	20.10	0.1415	0.512381	2	-0.28	-5.01	-0.77	-0.79	-0.79	1.37	1.62	1.37	1.62	1.37	Kozakov I K. et al.	2015	Petrology	23（4）	309-330
2539	7580—2	CAOB	Transbaikalia and Mongolia	101.5333	45.7333	Tatsaingol complex		603	600	6.75	31.60	0.1292	0.512345	5	-0.34	-5.72	-0.52	-0.55	-0.55	1.35	1.45	1.35	1.45	1.35	Kozakov I K. et al.	2015	Petrology	23（4）	309-330
2540	7291—1	CAOB	Transbaikalia and Mongolia	101.5333	45.7333	Tatsaingol complex		603	600	4.40	22.00	0.1206	0.512229	2	-0.39	-7.98	-2.12	-2.15	-2.15	1.48	1.51	1.48	1.51	1.48	Kozakov I K. et al.	2015	Petrology	23（4）	309-330
2541	6201—2	CAOB	Transbaikalia and Mongolia	101.3950	45.7667	conjugate with late metamorphism M2		547	547	7.93	39.70	0.1207	0.512182	2	-0.39	-8.90	-3.59	-3.59	-3.59	1.55	1.58	1.55	1.58	1.55	Kozakov I K. et al.	2015	Petrology	23（4）	309-330
2542	6201—1	CAOB	Transbaikalia and Mongolia	101.3950	45.7667	conjugate with late metamorphism M2		547	547	8.09	60.00	0.0814	0.512000	2	-0.59	-12.45	-4.40	-4.40	-4.40	1.62	1.32	1.62	1.32	1.62	Kozakov I K. et al.	2015	Petrology	23（4）	309-330
2543	6180—1	CAOB	Transbaikalia and Mongolia	101.3950	45.7667	conjugate with late metamorphism M1		565	565	1.76	11.13	0.0954	0.512131	7	-0.51	-9.89	-2.59	-2.59	-2.59	1.49	1.31	1.49	1.31	1.49	Kozakov I K. et al.	2015	Petrology	23（4）	309-330
2544	6036—1	CAOB	Transbaikalia and Mongolia	101.3950	45.7667	conjugate with late metamorphism M2		547	547	8.18	38.00	0.1300	0.512321	2	-0.34	-6.18	-1.53	-1.53	-1.53	1.39	1.51	1.39	1.51	1.39	Kozakov I K. et al.	2015	Petrology	23（4）	309-330
2545	2074639	CAOB	Transbaikalia and Mongolia	101.3833	45.8000	association of the Tatsaingol complex		603	600	4.70	20.10	0.1415	0.512381		-0.28	-5.01	-0.77	-0.79	-0.79	1.37	1.62	1.37	1.62	1.37	Kozakov I K. et al.	2015	Petrology	23（4）	309-330
2546	2074610	CAOB	Transbaikalia and Mongolia	101.3833	45.8000	association of the Tatsaingol complex		603	600	6.75	31.60	0.1292	0.512345		-0.34	-5.72	-0.52	-0.55	-0.55	1.35	1.45	1.35	1.45	1.35	Kozakov I K. et al.	2015	Petrology	23（4）	309-330
2547	2068005	CAOB	Transbaikalia and Mongolia	101.5333	45.7333	phic rocks of the Tatsaingol complex		603	600	8.13	42.50	0.1158	0.511910		-0.41	-14.20	-7.98	-8.01	-8.01	1.96	1.92	1.96	1.92	1.96	Kozakov I K. et al.	2015	Petrology	23（4）	309-330
2548	1969025	CAOB	Transbaikalia and Mongolia	101.3833	45.8000	association of the Tatsaingol complex		603	600	4.40	22.00	0.1206	0.512229		-0.39	-7.98	-2.12	-2.15	-2.15	1.48	1.51	1.48	1.51	1.48	Kozakov I K. et al.	2015	Petrology	23（4）	309-330
2549	1570941	CAOB	Transbaikalia and Mongolia	101.4167	45.7167	conjugate with late metamorphism M7		547	547	7.93	39.70	0.1207	0.512182		-0.39	-8.90	-3.59	-3.59	-3.59	1.55	1.58	1.55	1.58	1.55	Kozakov I K. et al.	2015	Petrology	23（4）	309-330
2550	1570910	CAOB	Transbaikalia and Mongolia	101.4167	45.7167	conjugate with late metamorphism M3		547	547	8.09	60.00	0.0814	0.512000		-0.59	-12.45	-4.40	-4.40	-4.40	1.62	1.32	1.62	1.32	1.62	Kozakov I K. et al.	2015	Petrology	23（4）	309-330

2551	1563240	CAOB	Transbaikalia and Mongolia	101.3833	45.6833	conjugate with early metamorphism Mi		565	565	1.76	11.13	0.0954	0.512131		-0.51	-9.89	-2.59	-2.59	-2.59	1.49	1.31	1.49	1.31	1.49	Kozakov I K. et al.	2015	Petrology	23（4）	309-330
2552	1510645	CAOB	Transbaikalia and Mongolia	101.4167	45.7167	conjugate with late metamorphism M5		547	547	8.18	38.00	0.1300	0.512321		-0.34	-6.18	-1.53	-1.53	-1.53	1.39	1.51	1.39	1.51	1.39	Kozakov I K. et al.	2015	Petrology	23（4）	309-330
2553	7580	CAOB	Transbaikalia and Mongolia	101.5167	45.7313	association of the Tatsaingol complex		603	600	6.32	29.90	0.1276	0.512330		-0.35	-6.01	-0.69	-0.72	-0.72	1.36	1.45	1.36	1.45	1.36	Kozakov I K. et al.	2015	Petrology	23（4）	309-330
2554	7562	CAOB	Transbaikalia and Mongolia	100.8833	45.9051	conjugate with early metamorphism Mi		570	570	3.64	19.96	0.1103	0.512085		-0.44	-10.79	-4.50	-4.50	-4.50	1.65	1.57	1.65	1.57	1.65	Kozakov I K. et al.	2015	Petrology	23（4）	309-330
2555	7559	CAOB	Transbaikalia and Mongolia	101.5333	45.7333	phic rocks of the Tatsaingol complex		603	600	5.21	26.90	0.1169	0.511681		-0.41	-18.67	-12.5	-12.6	-12.6	2.32	2.30	2.32	2.30	2.32	Kozakov I K. et al.	2015	Petrology	23（4）	309-330
2556	7291	CAOB	Transbaikalia and Mongolia	101.3833	45.8000	association of the Tatsaingol complex		603	600	3.41	13.84	0.1490	0.512436		-0.24	-3.94	-0.27	-0.29	-0.29	1.33	1.68	1.33	1.68	1.33	Kozakov I K. et al.	2015	Petrology	23（4）	309-330
2557	6427	CAOB	Transbaikalia and Mongolia	101.4167	45.7167	Postmetamorphic granitoids		545	545	8.65	47.30	0.1106	0.511807		-0.44	-16.21	-10.22	-10.22	-10.22	2.09	1.98	2.09	1.98	2.09	Kozakov I K. et al.	2015	Petrology	23（4）	309-330
2558	6425	CAOB	Transbaikalia and Mongolia	101.3833	45.6833	conjugate with early metamorphism Mi		561	561	3.58	20.60	0.1052	0.511826		-0.47	-15.84	-9.29	-9.29	-9.29	2.03	1.85	2.03	1.85	2.03	Kozakov I K. et al.	2015	Petrology	23（4）	309-330
2559	6422	CAOB	Transbaikalia and Mongolia	101.4167	45.7167	Postmetamorphic granitoids		545	545	0.93	6.76	0.0828	0.512144		-0.58	-9.64	-1.72	-1.72	-1.72	1.40	1.17	1.40	1.17	1.40	Kozakov I K. et al.	2015	Petrology	23（4）	309-330
2560	6201	CAOB	Transbaikalia and Mongolia	101.4167	45.7167	conjugate with late metamorphism M6		547	547	6.66	39.20	0.1026	0.512247		-0.48	-7.63	-1.06	-1.06	-1.06	1.35	1.24	1.35	1.24	1.35	Kozakov I K. et al.	2015	Petrology	23（4）	309-330
2561	6197	CAOB	Transbaikalia and Mongolia	101.4167	45.7167	Postmetamorphic granitoids		545	545	3.01	18.19	0.1000	0.511801		-0.49	-16.33	-9.60	-9.60	-9.60	2.04	1.80	2.04	1.80	2.04	Kozakov I K. et al.	2015	Petrology	23（4）	309-330
2562	6194	CAOB	Transbaikalia and Mongolia	101.4014	45.7485	conjugate with early metamorphism Mi		552	552	5.52	31.50	0.1059	0.512050		-0.46	-11.47	-5.07	-5.07	-5.07	1.68	1.55	1.68	1.55	1.68	Kozakov I K. et al.	2015	Petrology	23（4）	309-330
2563	6184	CAOB	Transbaikalia and Mongolia	101.3833	45.8000	association of the Tatsaingol complex		603	600	6.47	37.00	0.1056	0.512271		-0.46	-7.16	-0.15	-0.18	-0.18	1.32	1.24	1.32	1.24	1.32	Kozakov I K. et al.	2015	Petrology	23（4）	309-330
2564	6180	CAOB	Transbaikalia and Mongolia	101.3833	45.8000	association of the Tatsaingol complex		603	600	10.46	57.00	0.1109	0.512238		-0.44	-7.80	-1.20	-1.23	-1.23	1.41	1.35	1.41	1.35	1.41	Kozakov I K. et al.	2015	Petrology	23（4）	309-330
2565	6176	CAOB	Transbaikalia and Mongolia	101.6667	45.5333	conjugate with early metamorphism Mi		565	565	2.84	9.68	0.1773	0.512022		-0.10	-12.02	-10.6	-10.6	-10.6	2.12	4.67	2.12	4.67	2.13	Kozakov I K. et al.	2015	Petrology	23（4）	309-330
2566	6130	CAOB	Transbaikalia and Mongolia	101.5333	45.7333	phic rocks of the Tatsaingol complex		603	600	12.73	56.80	0.1354	0.511957		-0.31	-13.28	-8.57	-8.59	-8.59	2.00	2.31	2.00	2.31	2.00	Kozakov I K. et al.	2015	Petrology	23（4）	309-330
2567	6124	CAOB	Transbaikalia and Mongolia	101.6667	45.5333	conjugate with early metamorphism Mi		565	565	2.70	16.96	0.0962	0.511870		-0.51	-14.98	-7.74	-7.74	-7.74	1.91	1.66	1.91	1.66	1.91	Kozakov I K. et al.	2015	Petrology	23（4）	309-330
2568	6040	CAOB	Transbaikalia and Mongolia	101.5333	45.7333	phic rocks of the Tatsaingol complex		603	600	5.47	27.30	0.1212	0.511748		-0.38	-17.36	-11.6	-11.6	-11.6	2.24	2.30	2.24	2.30	2.24	Kozakov I K. et al.	2015	Petrology	23（4）	309-330
2569	6036	CAOB	Transbaikalia and Mongolia	101.4167	45.7167	conjugate with late metamorphism M4		547	547	5.21	30.10	0.1047	0.512164		-0.47	-9.25	-2.82	-2.82	-2.82	1.49	1.38	1.49	1.38	1.49	Kozakov I K. et al.	2015	Petrology	23（4）	309-330
2570	6035	CAOB	Transbaikalia and Mongolia	101.3667	45.7167	conjugate with early metamorphism Mi		565	565	5.23	31.30	0.1008	0.512209		-0.49	-8.37	-1.45	-1.45	-1.45	1.40	1.27	1.40	1.27	1.40	Kozakov I K. et al.	2015	Petrology	23（4）	309-330
2571	6012	CAOB	Transbaikalia and Mongolia	101.5333	45.7333	phic rocks of the Tatsaingol complex		603	600	5.88	25.40	0.1401	0.512299		-0.29	-6.61	-2.26	-2.28	-2.28	1.49	1.76	1.49	1.76	1.49	Kozakov I K. et al.	2015	Petrology	23（4）	309-330
2572	6006	CAOB	Transbaikalia and Mongolia	101.4167	45.7167	conjugate with late metamorphism M8		546	546	4.49	26.00	0.1042	0.511998		-0.47	-12.48	-6.04	-6.04	-6.04	1.75	1.60	1.75	1.60	1.75	Kozakov I K. et al.	2015	Petrology	23（4）	309-330
2573	6003	CAOB	Transbaikalia and Mongolia	101.5333	45.7333	phic rocks of the Tatsaingol complex		603	600	8.99	48.30	0.1125	0.511787		-0.43	-16.60	-10.12	-10.15	-10.15	2.13	2.05	2.13	2.05	2.13	Kozakov I K. et al.	2015	Petrology	23（4）	309-330
2574	5975	CAOB	Transbaikalia and Mongolia	101.4167	45.7167	conjugate with late metamorphism M2		547	547	3.73	35.70	0.0632	0.512068		-0.68	-11.12	-1.80	-1.80	-1.80	1.41	1.10	1.41	1.10	1.41	Kozakov I K. et al.	2015	Petrology	23（4）	309-330
2575	5967	CAOB	Transbaikalia and Mongolia	101.3833	45.7833	conjugate with early metamorphism Mi		562	562	0.93	5.69	0.0988	0.511962		-0.50	-13.19	-6.17	-6.17	-6.17	1.78	1.57	1.78	1.57	1.78	Kozakov I K. et al.	2015	Petrology	23（4）	309-330
2576	5965	CAOB	Transbaikalia and Mongolia	101.4167	45.7167	Postmetamorphic granitoids		545	545	10.27	52.70	0.1178	0.511819		-0.40	-15.98	-10.49	-10.49	-10.49	2.11	2.11	2.11	2.11	2.11	Kozakov I K. et al.	2015	Petrology	23（4）	309-330
2577	V045	CAOB	Mongolian Altai	96.1417	45.3765	Augen gneiss		375	375	5.34	34.10	0.0947	0.512593	0	-0.52	-0.88	4.02	4.00	4.00	0.80	0.71	0.80	0.72	0.80	Hanzl P et al.	2016	Journal of Geosciences	61(1)	25-50
2578	V044	CAOB	Mongolian Altai	96.1243	45.4766	Metatuff		375	375	4.68	22.50	0.1258	0.512620	0	-0.36	-0.35	3.05	3.04	3.04	0.87	0.92	0.87	0.72	0.88	Hanzl P et al.	2016	Journal of Geosciences	61(1)	25-50
2579	V039b	CAOB	Mongolian Altai	96.2503	45.4488	Orthogneiss		375	375	2.12	11.80	0.1086	0.512572	0	-0.45	-1.29	2.93	2.93	2.93	0.88	0.84	0.88	0.72	0.89	Hanzl P et al.	2016	Journal of Geosciences	61(1)	25-50
2580	V039a	CAOB	Mongolian Altai	96.2503	45.4488	Orthogneiss		375	375	3.99	19.10	0.1263	0.512291	0	-0.36	-6.77	-3.40	-3.40	-3.40	1.40	1.50	1.40	0.72	1.40	Hanzl P et al.	2016	Journal of Geosciences	61(1)	25-50
2581	V033	CAOB	Mongolian Altai	96.3055	45.3762	Orthogneiss		375	375	5.06	28.60	0.1070	0.512701	0	-0.46	1.23	5.53	5.52	5.52	0.67	0.64	0.67	0.72	0.68	Hanzl P et al.	2016	Journal of Geosciences	61(1)	25-50
2582	P110	CAOB	Mongolian Altai	96.1421	45.3755	Orthogneiss		375	375	6.06	34.50	0.1062	0.512542	0	-0.46	-1.87	2.46	2.46	2.46	0.92	0.86	0.92	0.72	0.93	Hanzl P et al.	2016	Journal of Geosciences	61(1)	25-50
2583	D246	CAOB	Mongolian Altai	96.5585	45.3058	Orthogneiss		375	375	4.37	19.30	0.1369	0.512700	0	-0.30	1.21	4.08	4.07	4.07	0.79	0.89	0.79	0.72	0.79	Hanzl P et al.	2016	Journal of Geosciences	61(1)	25-50
2584	D214	CAOB	Mongolian Altai	96.5236	45.2690	Orthogneiss		375	375	1.93	15.90	0.0734	0.512554	0	-0.63	-1.64	4.28	4.26	4.26	0.78	0.65	0.78	0.72	0.78	Hanzl P et al.	2016	Journal of Geosciences	61(1)	25-50
2585	D044	CAOB	Mongolian Altai	96.3642	45.3338	Meta-rhyolite		375	375	7.84	39.50	0.1200	0.512628	0	-0.39	-0.20	3.48	3.48	3.48	0.84	0.85	0.84	0.72	0.84	Hanzl P et al.	2016	Journal of Geosciences	61(1)	25-50
2586	X3SS-9	CAOB	East Tianshan	90.5000	41.9667	Diorite MME		335	335	3.09	12.60	0.1482	0.512530	6	-0.25	-2.11	-0.02	-0.03	-0.03	1.09	1.44	1.09	1.45	1.11	Du L et al.	2018	Lithos	304-307	230-244
2587	X3SS-6	CAOB	East Tianshan	90.5000	41.9667	Granodiorite		336	336	2.21	10.70	0.1250	0.512478	5	-0.36	-3.12	-0.05	-0.05	-0.05	1.09	1.15	1.09	1.16	1.11	Du L et al.	2018	Lithos	304-307	230-244
2588	X3SS-4	CAOB	East Tianshan	90.5000	41.9667	Granodiorite		336	336	1.91	9.92	0.1167	0.512453	7	-0.41	-3.61	-0.17	-0.18	-0.18	1.11	1.09	1.11	1.10	1.12	Du L et al.	2018	Lithos	304-307	230-244
2589	X3SS30	CAOB	East Tianshan	90.5000	41.9667	K-feldspar granite		292	292	8.26	23.80	0.2095	0.512830	5	0.07	3.75	3.27	3.27	3.27	0.76	11.23	0.76	11.63	0.80	Du L et al.	2018	Lithos	304-307	230-244
2590	X3SS29	CAOB	East Tianshan	90.5000	41.9667	K-feldspar granite		292	292	9.92	30.10	0.1990	0.512784	5	0.01	2.85	2.77	2.76	2.76	0.82	3.76	0.82	3.78	0.84	Du L et al.	2018	Lithos	304-307	230-244
2591	X3SS26	CAOB	East Tianshan	90.5000	41.9667	Monzonitic granite		281	281	5.89	26.90	0.1324	0.512640	4	-0.33	0.04	2.34	2.34	2.34	0.85	0.96	0.85	0.96	0.87	Du L et al.	2018	Lithos	304-307	230-244
2592	X3SS24	CAOB	East Tianshan	90.5000	41.9667	Monzonitic granite		281	281	5.23	24.50	0.1290	0.512627	9	-0.34	-0.21	2.22	2.21	2.21	0.87	0.94	0.87	0.94	0.88	Du L et al.	2018	Lithos	304-307	230-244
2593	X3SS-2	CAOB	East Tianshan	90.5000	41.9667	Granodiorite		336	336	1.98	9.93	0.1206	0.512416	19	-0.39	-4.33	-1.06	-1.07	-1.07	1.18	1.20	1.18	1.20	1.20	Du L et al.	2018	Lithos	304-307	230-244
2594	X3SS-19	CAOB	East Tianshan	90.5000	41.9667	Albitophyre		333	333	3.95	15.30	0.1556	0.512529	5	-0.21	-2.13	-0.37	-0.38	-0.38	1.12	1.63	1.12	1.63	1.14	Du L et al.	2018	Lithos	304-307	230-244
2595	X3SS-17	CAOB	East Tianshan	90.5000	41.9667	Albitophyre		333	333	3.26	12.90	0.1533	0.512508	10	-0.22	-2.54	-0.69	-0.69	-0.69	1.14	1.62	1.14	1.62	1.16	Du L et al.	2018	Lithos	304-307	230-244
2596	X3SS-15	CAOB	East Tianshan	90.5000	41.9667	Albitophyre		333	333	2.96	11.40	0.1575	0.512523	6	-0.20	-2.24	-0.57	-0.58	-0.58	1.13	1.70	1.13	1.70	1.15	Du L et al.	2018	Lithos	304-307	230-244
2597	X3SS-11	CAOB	East Tianshan	90.5000	41.9667	Diorite MME		335	335	2.71	11.90	0.1383	0.512512	8	-0.30	-2.46	0.03	0.04	0.04	1.09	1.29	1.09	1.29	1.10	Du L et al.	2018	Lithos	304-307	230-244
2598	Dy09-9	NCC	NCC	118.5000	41.4167	Qtz diorite	Ningcheng	226	226	6.00	35.10	0.1030	0.512354	13	-0.48	-5.54	-2.83	-2.84	-2.84	1.23	1.10	1.23	1.10	1.23	Zhang Z et al.	2014	Journal of Asian Earth Sciences	79(pt.A)	112-129
2599	Dy09-10-2	NCC	NCC	118.5000	41.4167	Qtz diorite	Ningcheng	226	226	3.00	18.00	0.1000	0.512334	14	-0.49	-5.93	-3.14	-3.14	-3.14	1.26	1.09	1.26	1.10	1.26	Zhang Z et al.	2014	Journal of Asian Earth Sciences	79(pt.A)	112-129
2600	X-945	CAOB	Kazakhstan	82.5000	50.0000	Monzonite		290	290	0.70	3.60	0.1184	0.512847		-0.40	4.08	6.98	6.97	6.97	0.48	0.49	0.48			Khromykh SV et al.	2018	Petrology	26(4)	368-388
2601	X-1079	CAOB	Kazakhstan	82.5000	50.0000	Granite		290	290	6.30	31.30	0.1226	0.512874		-0.38	4.60	7.35	7.35	7.35	0.45	0.46	0.45			Khromykh SV et al.	2018	Petrology	26(4)	368-388
2602	T5-140	CAOB	Tianshan	72.4367	42.5032	Rhyolite		1131	600	5.92	44.46	0.0811	0.511456		-0.59	-23.06	-6.33	-14.2	-14.2	2.46	1.94	2.46			Kröner A et al.	2013	Gondwana Research	23(1)	272-295
2603	KG-16	CAOB	Tianshan	75.6814	42.0581	Gneiss		441	441	4.65	27.07	0.1046	0.512199		-0.47	-8.56	-3.38	-3.38	-3.38	1.45	1.33	1.45			Kröner A et al.	2013	Gondwana Research	23(1)	272-295
2604	KG-15	CAOB	Tianshan	75.8758	42.2969	Rhyolite		451	451	6.64	32.14	0.1258	0.512224		-0.36	-8.08	-3.99	-3.99	-3.99	1.51	1.60	1.51			Kröner A et al.	2013	Gondwana Research	23(1)	272-295
2605	KG-14	CAOB	Tianshan	75.8847	42.3056	Granite		1153	600	10.93	70.58	0.0943	0.511435		-0.52	-23.47	-8.37	-15.6	-15.6	2.6	2.18	2.57			Kröner A et al.	2013	Gondwana Research	23(1)	272-295
2606	SS980605	CAOB	Xing-Meng orogenic belt	120.5000	49.5000	Basaltic andesite		275	275	3.67	28.51	0.1285	0.512700		-0.35	1.21	3.64	3.60	3.60	0.75	0.81	0.75		0.63	Zhang YB et al.	2006	Acta Petrologica Sinica	22(9)	2289-2304
2607	SS091810	CAOB	Xing-Meng orogenic belt	118.8681	43.5069	Sodium rhyolite		150	150				0.512420				-0.45		-0.45	0.45				0.45	Zhang YB et al.	2006	Acta Petrologica Sinica	22(9)	2289-2304
2608	SS091809	CAOB	Xing-Meng orogenic belt	118.8681	43.5069	Adakite		150	150	8.06	51.74	0.1558	0.512898		-0.21	5.07	5.99	5.85	5.85	0.46	0.66	0.46		0.42	Zhang YB et al.	2006	Acta Petrologica Sinica	22(9)	2289-2304
2609	SS091807	CAOB	Xing-Meng orogenic belt	118.9874	43.5344	Sodium rhyolite		150	150	7.58	53.75	0.1411	0.512642		-0.28	0.08	1.31	1.14	1.14	0.84	1.07	0.84		0.85	Zhang YB et al.	2006	Acta Petrologica Sinica	22(9)	2289-2304
2610	SS091804	CAOB	Xing-Meng orogenic belt	119.0046	43.4981	Adakite		150	150	4.02	19.84		0.512545				1.99		1.99	0.37				0.37	Zhang YB et al.	2006	Acta Petrologica Sinica	22(9)	2289-2304
2611	SS091720	CAOB	Xing-Meng orogenic belt	118.8508	43.9029	Adakite		150	150	5.76	40.04	0.1438	0.512738		-0.27	1.95	3.46	2.96	2.96	0.70	0.90	0.70		0.68	Zhang YB et al.	2006	Acta Petrologica Sinica	22(9)	2289-2304
2612	0846-8	CAOB	West Tianshan	84.5000	42.8000	Mineralized basalt		430	430	4.72	16.60	0.1724	0.512845		-0.12	4.04	5.38	5.37	5.37	0.73	1.13	0.73			Zhang X et al.	2012	Acta Petrologica Sinica	28(7)	2057-2073
2613	0846-1	CAOB	West Tianshan	84.5000	42.8000	Disseminated ore		430	430	7.50	25.70	0.1763	0.512867		-0.10	4.47	5.60	5.59	5.59	0.71	1.15	0.71			Zhang X et al.	2012	Acta Petrologica Sinica	28(7)	2057-2073
2614	0842-2	CAOB	West Tianshan	84.5000	42.8000	Basaltic andesite		430	430	6.27	23.50	0.1619	0.512787		-0.18	2.91	4.82	4.82	4.82	0.77	1.07	0.77			Zhang X et al.	2012	Acta Petrologica Sinica	28(7)	2057-2073
2615	0840-2	CAOB	West Tianshan	84.5000	42.8000	Basaltic andesite		430	430	5.70	21.00	0.1646	0.512828		-0.16	3.71	5.47	5.47	5.47	0.72	1.00	0.72			Zhang X et al.	2012	Acta Petrologica Sinica	28(7)	2057-2073
2616	0840-1	CAOB	West Tianshan	84.5000	42.8000	Basaltic andesite		430	430	6.11	21.00	0.1762	0.512815		-0.10	3.45	4.58	4.58	4.58	0.79	1.36	0.79			Zhang X et al.	2012	Acta Petrologica Sinica	28(7)	2057-2073
2617	0839-1	CAOB	West Tianshan	84.5000	42.8000	Basaltic andesite		430	430	5.70	20.70	0.1667	0.512826		-0.15	3.67	5.32	5.31	5.31	0.73	1.05	0.73			Zhang X et al.	2012	Acta Petrologica Sinica	28(7)	2057-2073
2618	0838-1	CAOB	West Tianshan	84.5000	42.8000	Basaltic andesite		430	430	5.55	21.00	0.1599	0.512802		-0.19	3.20	5.23	5.22	5.22	0.74	0.99	0.74			Zhang X et al.	2012	Acta Petrologica Sinica	28(7)	2057-2073
2619	XXG99-1	CAOB	Beishan	99.5000	40.6833	Granodiorite	Jiusidun	300	300	2.67	15.47	0.1045	0.512790	6	-0.47	2.97	6.49	6.49	6.49	0.53	0.50	0.53	0.50	0.57	Nie FJ et al.	2002	The Geological Publishing Hous		1-408
2620	NJS01-B1	CAOB	Beishan	96.0833	42.0167	Monzogranite	Mingshui	218	218	4.95	32.02	0.0934	0.512804	3	-0.53	3.24	6.12	6.12	6.12	0.50	0.44	0.50	0.44	0.55	Nie FJ et al.	2002	The Geological Publishing Hous		1-408
2621	MZS9-B2	CAOB	Beishan	95.7500	42.0833	Qtz porphyry	Mazhungshan	309	309	3.62	22.55	0.0970	0.512326	2	-0.51	-6.09	-2.15	-2.15	-2.15	1.24	1.08	1.24	1.08	1.31	Nie FJ et al.	2002	The Geological Publishing Hous		1-408
2622	LSSP1-S1	CAOB	Beishan	98.3833	42.2542	Monzogranite	Liushanshan	260	260	3.34	15.98	0.1262	0.513076	5	-0.36	8.54	10.9	10.9	10.9	0.26	0.13	0.14	0.13	0.12	Nie FJ et al.	2002	The Geological Publishing Hous		1-408
2623	LSS8-3	CAOB	Beishan	98.3833	42.2542	Granodiorite porphyry	Liushanshan	260	260	3.55	15.25	0.1407	0.512813	3	-0.28	3.41	5.27	5.27	5.27	0.60	0.70	0.60	0.70	0.54	Nie FJ et al.	2002	The Geological Publishing Hous		1-408
2624	LSS8-2	CAOB	Beishan	98.3833	42.2542	Granodiorite porphyry	Liushanshan	260	260	3.72	19.80	0.1137	0.512987	3	-0.42	6.81	9.56	9.56	9.56	0.26	0.25	0.25	0.25	0.26	Nie FJ et al.	2002	The Geological Publishing Hous		1-408
2625	HTS99-1	CAOB	Beishan	95.0217	41.1775	Potash feldspar granite	Huitongshan	436	436	2.01	23.69	0.0514	0.512579	3	-0.74	-1.15	6.93	6.93	6.93	0.61	0.54	0.61	0.54	0.91	Nie FJ et al.	2002	The Geological Publishing Hous		1-408
2626	HNS99-5	CAOB	Beishan	95.5500	41.2000	Potash feldspar granite	Huaniushan	194	194	4.98	34.34	0.0877	0.512701	4	-0.55	1.23	3.93	3.93	3.93	0.65	0.54	0.65	0.54	0.72	Nie FJ et al.	2002	The Geological Publishing Hous		1-408
2627	B5	CAOB	Beishan	98.3833	42.2542	Monzogranite	Liushanshan	260	260	3.47	15.24	0.1378	0.512940	4	-0.30	5.89	7.85	7.85	7.85	0.39	0.42	0.39	0.42	0.34	Nie FJ et al.	2002	The Geological Publishing Hous		1-408
2628	B1	CAOB	Beishan	98.3833	42.2542	Granodiorite	Liushanshan	260	260	4.42	21.73	0.1229	0.512653	4	-0.38	0.29	2.74	2.74	2.74	0.81	0.83	0.81	0.83	0.79	Nie FJ et al.	2002	The Geological Publishing Hous		1-408
2629	XT4-21	CAOB	Western Tianshan	81.5986	44.1994	Microdiorite MME		356	356	4.14	18.20	0.1375	0.512321	0	-0.30	-6.18	-3.50	-3.49	-3.49	1.39	1.65	1.39	1.65	1.39	Zhao X et al.	2014	Journal of Asian Earth Sciences	85	210-223
2630	XT4-20	CAOB	Western Tianshan	81.5986	44.1994	Microdiorite MME		356	356	3.83	16.00	0.1447	0.512273	0	-0.26	-7.12	-4.80	-4.76	-4.76	1.49	1.93	1.49	1.93	1.49	Zhao X et al.	2014	Journal of Asian Earth Sciences	85	210-223
2631	XT4-19	CAOB	Western Tianshan	81.5986	44.1994	Microdiorite MME		356	356	4.53	21.40	0.1280	0.512203	0	-0.35	-8.49	-5.40	-5.36	-5.36	1.54	1.68	1.54	1.68	1.54	Zhao X et al.	2014	Journal of Asian Earth Sciences	85	210-223
2632	XT4-18	CAOB	Western Tianshan	81.5986	44.1994	Microdiorite MME		356	356	3.85	17.70	0.1315	0.512341	0	-0.33	-5.79	-2.80	-2.83	-2.83	1.34	1.50	1.34	1.50	1.34	Zhao X et al.	2014	Journal of Asian Earth Sciences	85	210-223
2633	KG1402-20	CAOB	Tianshan	71.1419	41.5358	Andesite		464	464	2.69	14.39	0.1138	0.512440		-0.42	-3.86	1.06	1.05	1.05	1.11	1.08	1.11			Alexeiev DV et al.	2016	Gondwana Research	39	261-291
2634	KG1402-11	CAOB	Tianshan	70.9083	41.4586	Diorite		464	464	4.83	22.67	0.1298	0.512454		-0.34	-3.59	0.37	0.37	0.37	1.16	1.26	1.16			Alexeiev DV et al.	2016	Gondwana Research	39	261-291
2635	881053-6	CAOB	Tianshan	80.6167	44.9083	Granodiorite		455	455	3.83	17.20	0.1356	0.512506		-0.31	-2.57	0.98	0.97	0.97	1.11	1.26	1.11			Wang B et al.	2012	Journal of Asian Earth Sciences	49	40-53
2636	07-8d	CAOB	Tianshan	80.6167	44.9083	Granodiorite		447	447	13.20	71.50	0.1124	0.512349		-0.43	-5.64	-0.83	-0.83	-0.83	1.25	1.20	1.25			Wang B et al.	2012	Journal of Asian Earth Sciences	49	40-53
2637	07-1a	CAOB	Tianshan	80.6333	44.9500	Monzogranite		450	450	2.20	32.20	0.0416	0.512194		-0.79	-8.66	0.26	0.24	0.24	1.16	0.85	1.16			Wang B et al.	2012	Journal of Asian Earth Sciences	49	40-53
2638	02XW04b	CAOB	Tianshan	80.6167	44.9083	Granodiorite		455	455	3.55	18.60	0.1162	0.512416		-0.41	-4.33	0.35	0.34	0.34	1.16	1.15	1.16			Wang B et al.	2012	Journal of Asian Earth Sciences	49	40-53
2639	ZK0408-22	CAOB	East Tianshan	93.3333	41.9167	Porphyritic granite		236	236	17.10	4.10	0.1450	0.512589	0	-0.26	-0.96	0.60	0.60	0.60	0.96	1.24	0.96	1.24	0.96	Wu YS et al.	2017	Ore Geology Reviews	81(2)	641-671
2640	KZK0003-2	CAOB	East Tianshan	93.3333	41.9167	porphyritic granite		236	236	24.20	4.73	0.1182	0.512600	0	-0.40	-0.74	1.62	1.62	1.62	0.88	0.88	0.88	0.88	0.88	Wu YS et al.	2017	Ore Geology Reviews	81(2)	641-671
2641	KZK0003-1	CAOB	East Tianshan	93.3333	41.9167	porphyritic granite		236	236	23.00	4.55	0.1196	0.512602	0	-0.39	-0.70	1.62	1.62	1.62	0.88	0.89	0.88	0.89	0.88	Wu YS et al.	2017	Ore Geology Reviews	81(2)	641-671
2642	LS063	CAOB	Alashan, NH zone	105.3544	40.1841	MME	yamatu	270	270	7.28	40.44	0.1089	0.512021	9	-0.45	-12.04	-8.87	-9.01	-9.01	1.77	1.64	1.77	1.64	1.76	Zhang JJ et al.	2016	Journal of Petrology	157(1)	183-197
2643	LS062	CAOB	Alashan, NH zone	105.3544	40.1841	MME	yamatu	270	270	7.43	38.08	0.1180	0.512071	11	-0.40	-11.06	-8.21	-8.35	-8.35	1.71	1.71	1.71	1.71	1.71	Zhang JJ et al.	2016	Journal of Petrology	157(1)	183-197
2644	LS061	CAOB	Alashan, NH zone	105.3544	40.1841	MME	yamatu	270	270	7.33	40.76	0.1088	0.512012	10	-0.45	-12.21	-9.04	-9.18	-9.18	1.78	1.65	1.78	1.65	1.78	Zhang JJ et al.	2016	Journal of Petrology	157(1)	183-197
2645	LS058	CAOB	Alashan, NH zone	105.3544	40.1841	MME	yamatu	270	270	7.21	36.48	0.1194	0.512045	11	-0.39	-11.57	-8.76	-8.91	-8.91	1.76	1.78	1.76	1.78	1.75	Zhang JJ et al.	2016	Journal of Petrology	157(1)	183-197
2646	LS057	CAOB	Alashan, NH zone	105.3544	40.1841	MME	yamatu	270	270	7.88	39.07	0.1219	0.511992	10	-0.38	-12.60	-9.88	-10.0	-10.0	1.85	1.92	1.85	1.92	1.84	Zhang JJ et al.	2016	Journal of Petrology	157(1)	183-197
2647	LS056	CAOB	Alashan, NH zone	105.3544	40.1841	MME	yamatu	270	270	7.20	37.36	0.1165	0.512047	11	-0.41	-11.53	-8.62	-8.77	-8.77	1.75	1.73	1.75	1.73	1.74	Zhang JJ et al.	2016	Journal of Petrology	157(1)	183-197
2648	LS055	CAOB	Alashan, NH zone	105.3544	40.1841	MME	yamatu	270	270	7.20	37.24	0.1168	0.512032	10	-0.41	-11.82	-8.93	-9.07	-9.07	1.77	1.75	1.77	1.75	1.77	Zhang JJ et al.	2016	Journal of Petrology	157(1)	183-197

2649	LS049	CAOB	Alashan, NH zone	105.3544	40.1841	Granodiorite	yamatu	271	271	2.70	13.16	0.1239	0.511977	11	-0.37	-12.89	-10.2	-10.4	-10.4	1.88	1.98	1.88	1.98	1.87	Zhang JJ et al.	2016	Journal of Petrology	157(1)	183-197
2650	LS048	CAOB	Alashan, NH zone	105.3544	40.1841	Granodiorite	yamatu	271	271	6.69	31.83	0.1271	0.511906	10	-0.35	-14.28	-11.7	-11.9	-11.9	2.00	2.18	2.00	2.18	1.99	Zhang JJ et al.	2016	Journal of Petrology	157(1)	183-197
2651	LS047	CAOB	Alashan, NH zone	105.3544	40.1841	Monzogranite	yamatu	271	271	1.31	7.61	0.1043	0.511832	11	-0.47	-15.72	-12.4	-12.5	-12.5	2.05	1.83	2.05	1.83	2.05	Zhang JJ et al.	2016	Journal of Petrology	157(1)	183-197
2652	LS046	CAOB	Alashan, NH zone	105.3544	40.1841	Monzogranite	yamatu	271	271	2.01	12.46	0.0974	0.511854	13	-0.50	-15.29	-11.7	-11.9	-11.9	2.00	1.69	2.00	1.69	2.00	Zhang JJ et al.	2016	Journal of Petrology	157(1)	183-197
2653	09Q-5C	Tarim	Tarim	88.0300	41.0797	Granite		735	600	2.38	14.90	0.0962	0.511573		-0.51	-20.77	-11.5	-13.1	-13.1	2.37	2.04	2.37			Zhang CL et al.	2012	Precambrian Research	222-223	488-502
2654	09Q2-2B	Tarim	Tarim	88.0300	41.0797	Granite		737	600	0.74	4.32	0.1029	0.511485		-0.48	-22.49	-13.8	-15.3	-15.3	2.55	2.28	2.55			Zhang CL et al.	2012	Precambrian Research	222-223	488-503
2655	09Q2-2A	Tarim	Tarim	88.0300	41.0797	granite		737	600	1.77	9.62	0.1110	0.511593		-0.48	-20.38	-12.4	-13.2	-13.2	2.38	2.13	2.38			Zhang CL et al.	2013	Precambrian Research	222-223	488-504
2656	TL-38	CAOB	Tianshan	75.6700	42.6569	Granite		428	428	4.24	21.00	0.1230	0.512047		-0.37	-11.53	-7.51	-7.50	-7.50	1.77	1.85	1.77			Zhao XB et al.	2017	Ore Geology Reviews	82	217-231
2657	TL-37	CAOB	Tianshan	75.6700	42.6569	Granite		428	428	6.19	34.70	0.1087	0.511994		-0.45	-12.56	-7.76	-7.75	-7.75	1.80	1.67	1.80			Zhao XB et al.	2017	Ore Geology Reviews	82	217-231
2658	TL-35	CAOB	Tianshan	75.6700	42.6569	Granite		428	428	5.07	27.10	0.1139	0.512030		-0.42	-11.86	-7.34	-7.34	-7.34	1.76	1.71	1.76			Zhao XB et al.	2017	Ore Geology Reviews	82	217-231
2659	BSN88003	NCC	NMNCC	112.4994	42.2192	Granodiorite porphyry	Bainaimiao	440	440	4.11	21.46	0.1157	0.512250	5	-0.41	-7.57	-3.01	-3.02	-3.02	1.42	1.40	1.42	1.40	1.42	Nie et al.	1995	Acta Geoscientia Sinica	1	36-44
2660	BOS08	NCC	NMNCC	112.4994	42.2192	Granodiorite porphyry	Bainaimiao	440	440	2.94	11.78	0.1511	0.512351	8	-0.23	-5.60	-3.03	-3.04	-3.04	1.42	1.94	1.42	1.95	1.43	Nie et al.	1995	Acta Geoscientia Sinica	1	36-44
2661	BOS07	NCC	NMNCC	112.4994	42.2192	Granodiorite porphyry	Bainaimiao	440	440	3.11	12.99	0.1451	0.512325	6	-0.26	-6.11	-3.21	-3.21	-3.21	1.43	1.83	1.43	1.83	1.44	Nie et al.	1995	Acta Geoscientia Sinica	1	36-44
2662	BOS06	NCC	NMNCC	112.4994	42.2192	Granodiorite porphyry	Bainaimiao	440	440	2.29	10.85	0.1275	0.512285	6	-0.35	-6.89	-2.99	-3.00	-3.00	1.42	1.53	1.42	1.53	1.42	Nie et al.	1995	Acta Geoscientia Sinica	1	36-44
2663	BOS05	NCC	NMNCC	112.4994	42.2192	Granodiorite porphyry	Bainaimiao	440	440	2.91	14.52	0.1211	0.512251	6	-0.38	-7.55	-3.30	-3.30	-3.30	1.44	1.48	1.44	1.48	1.45	Nie et al.	1995	Acta Geoscientia Sinica	1	36-44
2664	BK8806	NCC	NMNCC	112.4994	42.2192	Granodiorite porphyry	Bainaimiao	440	440	2.10	9.13	0.1394	0.512303	5	-0.29	-6.53	-3.32	-3.32	-3.32	1.44	1.73	1.44	1.74	1.45	Nie et al.	1995	Acta Geoscientia Sinica	1	36-44
2665	BK8802	NCC	NMNCC	112.4994	42.2192	Granodiorite porphyry	Bainaimiao	440	440	2.22	9.87	0.1361	0.512295	8	-0.31	-6.69	-3.28	-3.29	-3.29	1.44	1.68	1.44	1.68	1.45	Nie et al.	1995	Acta Geoscientia Sinica	1	36-44
2666	CH-069	CAOB	East Kazakhstan	78.5000	49.0000	Bi-granite		400	400	2.79	15.36	0.1096	0.512631	2	-0.44	-0.14	4.30	4.31	4.31	0.79	0.76	0.79			Degtyarev KE et al.	2015	Doklady Earth Sciences	462(pt.1)	433-436
2667	CH-068	CAOB	East Kazakhstan	78.5000	49.0000	Bi-granite		400	400	2.10	11.64	0.1093	0.512647	2	-0.44	0.18	4.70	4.64	4.64	0.77	0.73	0.77			Degtyarev KE et al.	2015	Doklady Earth Sciences	462(pt.1)	433-436
2668	CH-066	CAOB	East Kazakhstan	78.5000	49.0000	Granodiorite		470	470	4.46	24.50	0.1099	0.512579	7	-0.44	-1.15	4.10	4.05	4.05	0.87	0.84	0.87			Degtyarev KE et al.	2015	Doklady Earth Sciences	462(pt.1)	433-436
2669	CH-063	CAOB	East Kazakhstan	78.5000	49.0000	Granite		400	400	0.73	2.31	0.1896	0.512900	13	-0.04	5.11	5.50	5.47	5.47	0.70	1.58	0.70			Degtyarev KE et al.	2015	Doklady Earth Sciences	462(pt.1)	433-436
2670	CH-062	CAOB	East Kazakhstan	78.5000	49.0000	Granite		400	400	3.05	17.50	0.1055	0.512636	3	-0.46	-0.04	4.60	4.62	4.62	0.77	0.72	0.77			Degtyarev KE et al.	2015	Doklady Earth Sciences	462(pt.1)	433-436
2671	CH-059-2	CAOB	East Kazakhstan	78.5000	49.0000	Diorite		419	419	3.24	16.17	0.1209	0.512704	4	-0.39	1.29	5.40	5.34	5.34	0.72	0.73	0.72			Degtyarev KE et al.	2015	Doklady Earth Sciences	462(pt.1)	433-436
2672	CH-059-1	CAOB	East Kazakhstan	78.5000	49.0000	Q-diorite		419	419	3.14	15.93	0.1192	0.512698	3	-0.39	1.17	5.30	5.31	5.31	0.73	0.73	0.73			Degtyarev KE et al.	2015	Doklady Earth Sciences	462(pt.1)	433-436
2673	CH-058	CAOB	East Kazakhstan	78.5000	49.0000	Granite		419	419	1.95	11.90	0.0993	0.512637	4	-0.50	-0.02	5.20	5.19	5.19	0.74	0.68	0.74			Degtyarev KE et al.	2015	Doklady Earth Sciences	462(pt.1)	433-436
2674	CH-056	CAOB	East Kazakhstan	78.5000	49.0000	Hb-tonalite		470	470	2.43	12.40	0.1180	0.512603	7	-0.40	-0.68	4.10	4.04	4.04	0.87	0.87	0.87			Degtyarev KE et al.	2015	Doklady Earth Sciences	462(pt.1)	433-436
2675	CH-055	CAOB	East Kazakhstan	78.5000	49.0000	Monzonite		470	470	2.95	15.80	0.1131	0.512592	7	-0.43	-0.90	4.10	4.12	4.12	0.87	0.85	0.87			Degtyarev KE et al.	2015	Doklady Earth Sciences	462(pt.1)	433-436
2676	CH-049	CAOB	East Kazakhstan	79.0000	49.0000	Granodiorite		520	520	7.64	29.80	0.1549	0.512798	7	-0.21	3.12	5.90	5.89	5.89	0.76	0.91	0.76			Degtyarev KE et al.	2015	Doklady Earth Sciences	462(pt.1)	433-436
2677	CH-047	CAOB	East Kazakhstan	78.7500	49.0000	Granite		419	419	2.54	16.82	0.0913	0.512535	3	-0.54	-2.01	3.60	3.63	3.63	0.86	0.77	0.86			Degtyarev KE et al.	2015	Doklady Earth Sciences	462(pt.1)	433-436
2678	CH-046	CAOB	East Kazakhstan	79.0000	49.0000	Diorite		419	419	5.47	26.10	0.1269	0.512663	3	-0.35	0.49	4.20	4.22	4.22	0.81	0.86	0.81			Degtyarev KE et al.	2015	Doklady Earth Sciences	462(pt.1)	433-436
2679	CH-043	CAOB	East Kazakhstan	78.7500	49.0000	Bi-leucogranite		419	419	2.90	16.60	0.1057	0.512618	3	-0.46	-0.39	4.50	4.48	4.48	0.79	0.75	0.79			Degtyarev KE et al.	2015	Doklady Earth Sciences	462(pt.1)	433-436
2680	CH-040	CAOB	East Kazakhstan	79.0000	49.0000	Bi-granite		520	520	4.83	19.60	0.1493	0.512786	7	-0.24	2.89	6.00	6.03	6.03	0.75	0.86	0.75			Degtyarev KE et al.	2015	Doklady Earth Sciences	462(pt.1)	433-436
2681	CH-035	CAOB	East Kazakhstan	79.0000	49.0000	Leucogranite		280	280	3.06	20.50	0.0901	0.512677	3	-0.54	0.76	4.60	4.57	4.57	0.67	0.58	0.67			Degtyarev KE et al.	2015	Doklady Earth Sciences	462(pt.1)	433-436
2682	CH-034	CAOB	East Kazakhstan	79.0000	49.0000	Leucogranite		280	280	2.49	16.40	0.0918	0.512664	3	-0.53	0.51	4.30	4.26	4.26	0.70	0.61	0.70			Degtyarev KE et al.	2015	Doklady Earth Sciences	462(pt.1)	433-436
2683	CH-026	CAOB	East Kazakhstan	79.0000	49.0000	Granite		280	280	2.30	14.30	0.0972	0.512669	7	-0.51	0.60	4.20	4.16	4.16	0.71	0.63	0.71			Degtyarev KE et al.	2015	Doklady Earth Sciences	462(pt.1)	433-436
2684	CH-022	CAOB	East Kazakhstan	79.0000	49.0000	Granite		280	280	3.54	23.08	0.0928	0.512647	3	-0.53	0.18	3.90	3.89	3.89	0.73	0.63	0.73			Degtyarev KE et al.	2015	Doklady Earth Sciences	462(pt.1)	433-436
2685	NJS01-25	CAOB	Beishan	96.1667	42.0167	Monzogranite	Nanjinshan	218	218	2.98	23.71	0.0759	0.512023	7	-0.61	-12.00	-8.63	-8.63	-8.63	1.70	1.25	1.70	1.25	1.79	Jiang SH et al.	2006	Acta Geologica Sinica	80(6)	826-842
2686	NJS01-15	CAOB	Beishan	96.0167	42.0000	Diorite	Nanjinshan	244	244	5.62	27.39	0.1241	0.512205	5	-0.37	-8.45	-6.18	-6.18	-6.18	1.52	1.60	1.52	1.60	1.50	Jiang SH et al.	2006	Acta Geologica Sinica	80(6)	826-842
2687	NJS01-14	CAOB	Beishan	96.0167	42.0000	Diorite	Nanjinshan	244	244	5.97	28.15	0.1283	0.512214	6	-0.35	-8.27	-6.14	-6.14	-6.14	1.51	1.67	1.51	1.67	1.49	Jiang SH et al.	2006	Acta Geologica Sinica	80(6)	826-842
2688	NJS01-13	CAOB	Beishan	96.1667	42.0167	Monzogranite	Nanjinshan	218	218	3.72	19.93	0.1129	0.511808	4	-0.43	-16.19	-13.9	-13.9	-13.9	2.12	2.02	2.12	2.02	2.13	Jiang SH et al.	2006	Acta Geologica Sinica	80(6)	826-842
2689	NJS01-11	CAOB	Beishan	96.1667	42.0167	Monzogranite	Nanjinshan	218	218	7.47	46.62	0.0963	0.511655	5	-0.51	-19.18	-16.4	-16.4	-16.4	2.32	1.93	2.32	1.93	2.37	Jiang SH et al.	2006	Acta Geologica Sinica	80(6)	826-842
2690	NJS01-10	CAOB	Beishan	96.0833	42.0167	Granodiorite	Nanjinshan	272	272	6.96	45.51	0.0925	0.512725	6	-0.53	1.70	5.31	5.31	5.31	0.61	0.54	0.61	0.54	0.68	Jiang SH et al.	2006	Acta Geologica Sinica	80(6)	826-842
2691	HSS01-9	CAOB	Beishan	96.7447	42.3800	Monzogranite	Langwashan	272	272	4.48	17.78	0.1524	0.512585	4	-0.23	-1.03	0.50	0.50	0.50	1.00	1.40	1.00	1.40	0.90	Jiang SH et al.	2006	Acta Geologica Sinica	80(6)	826-842
2692	HSS01-8	CAOB	Beishan	96.7447	42.3800	Tonalite	Hongshishannandong	272	272	1.75	7.91	0.1339	0.512500	4	-0.32	-2.69	-0.51	-0.51	-0.51	1.08	1.24	1.08	1.24	1.03	Jiang SH et al.	2006	Acta Geologica Sinica	80(6)	826-842
2693	HSS01-7	CAOB	Beishan	96.7447	42.3800	Tonalite	Hongshishannandong	272	272	2.11	11.16	0.1145	0.512629	5	-0.42	-0.18	2.68	2.68	2.68	0.82	0.80	0.82	0.80	0.83	Jiang SH et al.	2006	Acta Geologica Sinica	80(6)	826-842
2694	HSS01-6	CAOB	Beishan	96.7447	42.3800	Potash feldspar granite	Hongshishannandong	272	272	1.69	10.01	0.1019	0.512564	4	-0.48	-1.44	1.85	1.85	1.85	0.89	0.80	0.89	0.80	0.93	Jiang SH et al.	2006	Acta Geologica Sinica	80(6)	826-842
2695	HSS01-5	CAOB	Beishan	96.7447	42.3800	Potash feldspar granite	Hongshishannandong	272	272	1.73	10.21	0.1022	0.512526	8	-0.48	-2.18	1.10	1.10	1.10	0.95	0.85	0.95	0.85	0.99	Jiang SH et al.	2006	Acta Geologica Sinica	80(6)	826-842
2696	HSS01-4	CAOB	Beishan	97.8339	42.3008	Tonalite	Heiyingshannan	272	272	3.89	24.11	0.0976	0.512463	4	-0.50	-3.41	0.03	0.03	0.03	1.04	0.90	1.04	0.90	1.09	Jiang SH et al.	2006	Acta Geologica Sinica	80(6)	826-842
2697	HSS01-3	CAOB	Beishan	97.8339	42.3008	Tonalite	Heiyingshannan	272	272	3.64	13.39	0.1643	0.511936	8	-0.16	-13.69	-12.6	-12.6	-12.6	2.05	3.71	2.05	3.71	1.91	Jiang SH et al.	2006	Acta Geologica Sinica	80(6)	826-842
2698	HSS01-2	CAOB	Beishan	97.8672	42.3008	Monzogranite	Heiyingshannan	272	272	4.36	17.51	0.1504	0.512324	4	-0.24	-6.13	-4.52	-4.52	-4.52	1.40	1.98	1.40	1.98	1.31	Jiang SH et al.	2006	Acta Geologica Sinica	80(6)	826-842
2699	HSS01-17	CAOB	Beishan	96.7264	42.4492	Granodiorite	Queershan	286	286	3.68	15.84	0.1404	0.512774	5	-0.29	2.65	4.71	4.71	4.71	0.67	0.78	0.67	0.78	0.60	Jiang SH et al.	2006	Acta Geologica Sinica	80(6)	826-842
2700	HSS01-16	CAOB	Beishan	96.7261	42.4489	ım coarse grained monzog	Queershan	286	286	2.71	11.53	0.1422	0.512922	6	-0.28	5.54	7.53	7.53	7.53	0.44	0.49	0.44	0.49	0.36	Jiang SH et al.	2006	Acta Geologica Sinica	80(6)	826-842
2701	HSS01-15	CAOB	Beishan	96.7281	42.4467	Porphyritic monzogranite	Queershan	272	272	3.77	13.70	0.1663	0.512323	8	-0.15	-6.14	-5.09	-5.09	-5.09	1.45	2.64	1.45	2.64	1.31	Jiang SH et al.	2006	Acta Geologica Sinica	80(6)	826-842
2702	HSS01-14	CAOB	Beishan	96.7283	42.4469	Porphyritic monzogranite	Queershan	272	272	1.72	6.14	0.1690	0.512347	7	-0.14	-5.68	-4.72	-4.72	-4.72	1.42	2.72	1.42	2.72	1.27	Jiang SH et al.	2006	Acta Geologica Sinica	80(6)	826-842
2703	HSS01-13	CAOB	Beishan	96.7286	42.4472	Porphyritic monzogranite	Queershan	272	272	3.25	13.38	0.1471	0.512854	7	-0.25	4.21	5.93	5.93	5.93	0.55	0.68	0.55	0.68	0.47	Jiang SH et al.	2006	Acta Geologica Sinica	80(6)	826-842
2704	HSS01-12	CAOB	Beishan	96.8131	42.4086	Tonalite	Langwashan	294	294	2.37	10.25	0.1396	0.511853	8	-0.29	-15.31	-13.17	-13.17	-13.17	2.12	2.65	2.12	2.65	2.05	Jiang SH et al.	2006	Acta Geologica Sinica	80(6)	826-842
2705	HSS01-11	CAOB	Beishan	96.8131	42.4086	Tonalite	Langwashan	294	294	2.36	10.07	0.1419	0.512512	4	-0.28	-2.46	-0.40	-0.40	-0.40	1.09	1.35	1.09	1.35	1.01	Jiang SH et al.	2006	Acta Geologica Sinica	80(6)	826-842
2706	HSS01-10	CAOB	Beishan	96.8131	42.4086	Tonalite	Langwashan	294	294	1.46	6.66	0.1323	0.512396	6	-0.33	-4.72	-2.30	-2.30	-2.30	1.24	1.41	1.24	1.41	1.20	Jiang SH et al.	2006	Acta Geologica Sinica	80(6)	826-842
2707	HSS01-1	CAOB	Beishan	97.8672	42.3008	Monzogranite	Heiyingshannan	272	272	3.34	11.09	0.1821	0.512489	7	-0.07	-2.91	-2.40	-2.40	-2.40	1.23	3.17	1.23	3.17	1.04	Jiang SH et al.	2006	Acta Geologica Sinica	80(6)	826-842
2708	HLZD01-4	CAOB	Beishan	99.0533	42.3775	Tonalite	Horezadegai	272	272	8.39	33.72	0.1503	0.512414	4	-0.24	-4.37	-2.76	-2.76	-2.76	1.26	1.76	1.26	1.76	1.17	Jiang SH et al.	2006	Acta Geologica Sinica	80(6)	826-842
2709	BZG-07	CAOB	Beishan	97.1694	41.7028	Granodiorite	Gongpoquan	337	337	3.56	27.54	0.0782	0.512166	6	-0.60	-9.21	-4.11	-4.11	-4.11	1.43	1.11	1.43	1.11	1.57	Jiang SH et al.	2006	Acta Geologica Sinica	80(6)	826-842
2710	BZG-06	CAOB	Beishan	98.3783	41.8958	Granodiorite	Shibanjin	337	337	5.31	39.58	0.0811	0.512367	5	-0.59	-5.29	-0.32	-0.32	-0.32	1.12	0.90	1.12	0.90	1.25	Jiang SH et al.	2006	Acta Geologica Sinica	80(6)	826-842
2711	BZG-05	CAOB	Beishan	98.3783	41.8958	Monzogranite	Shibanjin	352	352	5.83	41.14	0.0857	0.512039	6	-0.56	-11.68	-6.69	-6.69	-6.69	1.65	1.32	1.65	1.32	1.77	Jiang SH et al.	2006	Acta Geologica Sinica	80(6)	826-842
2712	BZG-04	CAOB	Beishan	98.3783	41.8958	Potash feldspar granite	Shibanjin	352	352	2.55	10.02	0.1539	0.511899	6	-0.22	-14.42	-12.5	-12.5	-12.5	2.11	3.17	2.11	3.17	1.98	Jiang SH et al.	2006	Acta Geologica Sinica	80(6)	826-842
2713	BZG-03	CAOB	Beishan	98.3783	41.8958	Tonalite	Shibanjin	352	352	4.04	43.40	0.0563	0.511591	5	-0.71	-20.42	-14.1	-14.1	-14.1	2.25	1.51	2.25	1.51	2.48	Jiang SH et al.	2006	Acta Geologica Sinica	80(6)	826-842
2714	BZG-02	CAOB	Beishan	98.3783	41.8958	Qtz diorite	Shibanjin	352	352	7.50	43.09	0.1052	0.511938	5	-0.47	-13.65	-9.54	-9.54	-9.54	1.88	1.70	1.88	1.70	1.93	Jiang SH et al.	2006	Acta Geologica Sinica	80(6)	826-842
2715	BZG-01	CAOB	Beishan	98.3783	41.8958	Monzogranite	Shibanjin	352	352	5.84	37.38	0.0944	0.511893	4	-0.52	-14.53	-9.93	-9.93	-9.93	1.91	1.60	1.91	1.60	2.00	Jiang SH et al.	2006	Acta Geologica Sinica	80(6)	826-842
2716	46001-4	CAOB	Beishan	96.7000	42.3000	Monzogranite	Dashishan	272	272	0.64	3.32	0.1158	0.512430	8	-0.41	-4.06	-1.25	-1.25	-1.25	1.14	1.12	1.14	1.12	1.15	Jiang SH et al.	2006	Acta Geologica Sinica	80(6)	826-842
2717	46001-1	CAOB	Beishan	96.7000	42.3000	Tonalite	Dashishan	272	272	0.63	30.21	0.1187	0.512197	10	-0.40	-8.60	-5.90	-5.90	-5.90	1.52	1.53	1.52	1.53	1.52	Jiang SH et al.	2006	Acta Geologica Sinica	80(6)	826-842
2718	GZ10-52	CAOB	SMXAB	119.6722	44.5731	Bi-monzogranite	Wulandaba	137	150	6.75	31.13	0.1311	0.512676	5	-0.33	0.74	1.88	2.00	2.00	0.78	0.87	0.78	0.88	0.78	Yang QD et al.	2017	Lithos	272–273	164-184
2719	GZ10-49	CAOB	SMXAB	119.7750	44.5175	Bi-monzogranite	Xiaojingzi	138	150	6.35	32.40	0.1185	0.512625	5	-0.40	-0.25	1.12	1.24	1.24	0.84	0.84	0.84	0.84	0.84	Yang QD et al.	2017	Lithos	272–273	164-184
2720	GZ10-28	CAOB	SMXAB	120.4192	44.8278	Granite porphyry	Bailimuhada	125	150	4.39	24.15	0.1101	0.512600	9	-0.44	-0.74	0.64	0.92	0.92	0.86	0.81	0.86	0.81	0.87	Yang QD et al.	2017	Lithos	272–273	164-184
2721	GZ10-16	CAOB	SMXAB	119.6575	44.8364	Bi-monzogranite	Wunegetu	228	228	3.42	17.23	0.1202	0.512800	13	-0.39	3.16	5.40	5.39	5.39	0.56	0.57	0.56	0.57	0.57	Yang QD et al.	2017	Lithos	272–273	164-184
2722	E10925-5	CAOB	SMXAB	112.1958	44.1086	Syengranite	Baruntuolanhuduge	274	274	2.77	14.33	0.1170	0.512749	13	-0.41	2.17	4.95	4.95	4.95	0.64	0.63	0.64	0.64	0.64	Yang QD et al.	2017	Lithos	272–273	164-184
2723	E10101-2	CAOB	SMXAB	112.8264	44.3192	Alkali granite	Baiyinwula	285	285	6.25	25.51	0.1482	0.512828	8	-0.25	3.71	5.48	5.47	5.47	0.60	0.75	0.60	0.75	0.61	Yang QD et al.	2017	Lithos	272–273	164-184
2724	GZ10-65	CAOB	SHB	119.3008	44.3053	Bi-Moyite	Tumufuzhou	154	154	5.85	25.11	0.1408	0.512887	13	-0.28	4.86	5.96	5.96	5.96	0.46	0.55	0.46	0.55	0.46	Yang QD et al.	2017	Lithos	272–273	164-184
2725	GZ10-57	CAOB	SHB	119.5267	44.1564	Bi-monzogranite	Huajialaga	139	150	4.23	24.30	0.1054	0.512629	12	-0.46	-0.18	1.44	1.57	1.57	0.81	0.73	0.81	0.74	0.81	Yang QD et al.	2017	Lithos	272–273	164-184
2726	Ⅲ-10	CAOB	SMXAB	119.0533	44.6117	Granite	Haobugao	158	158	8.26	38.06	0.1313	0.512650	10	-0.33	0.23	1.55	1.55	1.55	0.82	0.93	0.82	0.93	0.82	Cai J et al.	2004	Acta Petrologica Sinica	20(5)	1225-1242
2727	991304	CAOB	SMXAB	118.7567	44.3689	Granodiorite porphyry	Baiyinnuo	171	171	5.11	24.26	0.1275	0.512670	8	-0.35	0.62	2.13	2.13	2.13	0.78	0.85	0.78	0.85	0.78	Cai J et al.	2004	Acta Petrologica Sinica	20(5)	1225-1242
2728	88103	CAOB	SMXAB	117.6800	44.2017	Granodiorite	Hadatu	150	150	8.82	47.20	0.1130	0.512750	11	-0.43	2.18	3.79	3.79	3.79	0.63	0.61	0.63	0.61	0.63	Cai J et al.	2004	Acta Petrologica Sinica	20(5)	1225-1242
2729	Tawati-04	CAOB	Altai	90.4886	46.8425	S-type granites		334	334	6.15	32.00	0.1400	0.512218	3	-0.29	-8.19	-5.80	-5.77	-5.77	1.56	1.92	1.56	1.92	1.38	Zhang C et al.	2019	Lithos	332	261-273
2730	Tawati-03	CAOB	Altai	90.4886	46.8425	S-type granites		334	334	6.50	30.10	0.1387	0.512212	4	-0.29	-8.31	-5.80	-5.84	-5.84	1.56	1.90	1.56	1.90	1.38	Zhang C et al.	2019	Lithos	332	261-273
2731	Tawati-02	CAOB	Altai	90.4881	46.8422	S-type granites		334	334	6.63	33.40	0.1458	0.512213	8	-0.26	-8.29	-6.10	-6.12	-6.12	1.58	2.10	1.58	2.10	1.40	Zhang C et al.	2019	Lithos	332	261-273
2732	Tawati-01	CAOB	Altai	90.4881	46.8422	S-type granites		334	334	6.52	29.20	0.1330	0.512218	3	-0.32	-8.19	-5.50	-5.48	-5.48	1.53	1.76	1.53	1.76	1.36	Zhang C et al.	2019	Lithos	332	261-273
2733	Qinghe-North-04	CAOB	Altai	90.2614	46.8606	S-type granites		345	345	5.76	25.20	0.1254	0.512352	9	-0.36	-5.58	-2.40	-2.44	-2.44	1.30	1.38	1.30	1.38	1.15	Zhang C et al.	2019	Lithos	332	261-273
2734	Qinghe-North-03	CAOB	Altai	90.2614	46.8606	S-type granites		345	345	5.20	24.80	0.1376	0.512358	4	-0.30	-5.46	-2.90	-2.86	-2.86	1.33	1.58	1.33	1.58	1.18	Zhang C et al.	2019	Lithos	332	261-273
2735	Qinghe-North-02	CAOB	Altai	90.2608	46.8600	S-type granites		345	345	5.79	24.10	0.1277	0.512355	9	-0.35	-5.52	-2.50	-2.48	-2.48	1.30	1.41	1.30	1.41	1.16	Zhang C et al.	2019	Lithos	332	261-273
2736	Qinghe-North-01	CAOB	Altai	90.2608	46.8600	S-type granites		345	345	5.15	26.30	0.1215	0.512357	5	-0.38	-5.48	-2.20	-2.17	-2.17	1.27	1.31	1.27	1.31	1.14	Zhang C et al.	2019	Lithos	332	261-273
2737	Kumaier-04	CAOB	Altai	90.0108	46.9200	S-type granites		331	331	7.83	36.00	0.1578	0.512385	7	-0.20	-4.94	-3.30	-3.29	-3.29	1.35	2.08	1.35	2.08	1.20	Zhang C et al.	2019	Lithos	332	261-273
2738	Kumaier-03	CAOB	Altai	90.0108	46.9200	S-type granites		331	331	7.38	37.10	0.1332	0.512387	4	-0.32	-4.90	-2.20	-2.22	-2.22	1.27	1.44	1.27	1.44	1.13	Zhang C et al.	2019	Lithos	332	261-273
2739	Kumaier-02	CAOB	Altai	90.0106	46.9258	S-type granites		331	331	8.25	40.70	0.1522	0.512381	8	-0.23	-5.01	-3.10	-3.13	-3.13	1.34	1.90	1.34	1.90	1.19	Zhang C et al.	2019	Lithos	332	261-273
2740	Kumaier-01	CAOB	Altai	90.0106	46.9258	S-type granites		331	331	7.55	38.30	0.1626	0.512388	9	-0.17	-4.88	-3.40	-3.44	-3.44	1.36	2.26	1.36	2.26	1.21	Zhang C et al.	2019	Lithos	332	261-273
2741	Halaqiao-04	CAOB	Altai	90.4294	46.4553	Monzonite		390	390	8.21	43.70	0.1144	0.512256		-0.42	-7.45	-3.36	-3.36	-3.36	1.41	1.37	1.41			Zhang C et al.	2019	Lithos	332	261-273
2742	Halaqiao-03	CAOB	Altai	90.4294	46.4553	Gneiss		390	390	7.07	40.50	0.1063	0.512253		-0.46	-7.51	-3.01	-3.01	-3.01	1.38	1.27	1.38			Zhang C et al.	2019	Lithos	332	261-273
2743	Halaqiao-02	CAOB	Altai	90.4294	46.4553	Gneiss		390	390	6.30	37.50	0.1023	0.512258		-0.48	-7.41	-2.71	-2.72	-2.72	1.36	1.22	1.36			Zhang C et al.	2019	Lithos	332	261-273
2744	Halaqiao-01	CAOB	Altai	90.4294	46.4553	Granite		390	390	6.75	35.40	0.1161	0.512251		-0.41	-7.55	-3.54	-3.54	-3.54	1.42	1.40	1.42			Zhang C et al.	2019	Lithos	332	261-273
2745	68(Wang)	CAOB	Altai	90.6795	46.2187	Kf granite		350	350	6.46	30.37	0.1287	0.512736		-0.35	1.91	4.90	4.95	4.95	0.70	0.74	0.70	0.74	0.70	Wang T et al.	2009	Lithos	110（1-4）	359-372
2746	67(Wang)	CAOB	Altai	90.6795	46.2187	Alkaline granite		350	350	11.74	56.07	0.1267	0.512555		-0.36	-1.62	1.50	1.51	1.51	0.98	1.04	0.98	1.04	0.98	Wang T et al.	2009	Lithos	110（1-4）	359-372

2747	66(Wang)	CAOB	Altai	90.6795	46.2187	Granodiorite		350	350	3.93	19.54	0.1216	0.512672		-0.38	0.66	4.00	4.02	4.02	0.77	0.79	0.77	0.79	0.77	Wang T et al.	2009	Lithos	110（1-4）	359-372
2748	39(Wang)	CAOB	Altai	88.2412	47.8139	Granite		150	150	9.98	44.23	0.1363	0.512632		-0.31	-0.12	1.00	1.04	1.04	0.85	1.02	0.85	1.02	0.85	Wang T et al.	2009	Lithos	110（1-4）	359-372
2749	38(Wang)	CAOB	Altai	88.2412	47.8139	Granite		150	150	9.67	42.88	0.1362	0.512648		-0.31	0.20	1.40	1.35	1.35	0.83	0.99	0.83	0.99	0.83	Wang T et al.	2009	Lithos	110（1-4）	359-372
2750	35(Wang)	CAOB	Altai	88.3991	47.7896	Granite		276	276	6.46	31.94	0.1200	0.512586		-0.39	-1.01	1.60	1.69	1.69	0.90	0.92	0.90	0.92	0.90	Wang T et al.	2009	Lithos	110（1-4）	359-372
2751	33(Wang)	CAOB	Altai	88.2009	47.9391	Monzogranite		220	220	4.31	22.00	0.1183	0.512312		-0.40	-6.36	-4.20	-4.16	-4.16	1.33	1.34	1.33	1.34	1.33	Wang T et al.	2009	Lithos	110（1-4）	359-372
2752	31(Wang)	CAOB	Altai	89.6814	47.4351	Monzogranite		209	209	8.56	49.44	0.1046	0.512387		-0.47	-4.90	-2.40	-2.44	-2.44	1.19	1.07	1.19	1.07	1.19	Wang T et al.	2009	Lithos	110（1-4）	359-372
2753	24(Wang)	CAOB	Altai	88.6414	47.7061	Monozgranite		375	375	9.27	40.30	0.1391	0.512458		-0.29	-3.51	-0.80	-0.75	-0.75	1.18	1.41	1.18	1.41	1.18	Wang T et al.	2009	Lithos	110（1-4）	359-372
2754	189/1	CAOB	Altai	89.4839	46.9945	Granite		275	275	1.21	6.20	0.1176	0.512808		-0.40	3.32	6.10	6.09	6.09	0.54	0.54	0.54	0.54	0.54	Wang T et al.	2009	Lithos	110（1-4）	359-372
2755	18(Wang)	CAOB	Altai	90.1559	47.1761	Granodiorite		407	407	4.63	22.00	0.1271	0.512481		-0.35	-3.06	1.00	0.55	0.55	1.10	1.18	1.10	1.18	1.10	Wang T et al.	2009	Lithos	110（1-4）	359-372
2756	17(Wang)	CAOB	Altai	89.9015	47.2623	Granite		407	407	12.73	64.51	0.1192	0.512497		-0.39	-2.75	1.30	1.27	1.27	1.04	1.05	1.04	1.05	1.04	Wang T et al.	2009	Lithos	110（1-4）	359-372
2757	169/1	CAOB	Altai	88.0354	47.8354	Granitoid gneiss		460	460	5.56	20.99	0.1600	0.512467		-0.19	-3.34	-1.20	-1.18	-1.18	1.29	1.93	1.29	1.93	1.29	Wang T et al.	2009	Lithos	110（1-4）	359-372
2758	120/5	CAOB	Altai	89.9015	47.2623	Diorite		407	407	2.10	6.74	0.1888	0.512629		-0.04	-0.18	0.20	0.23	0.23	1.12	3.17	1.12	3.17	1.12	Wang T et al.	2009	Lithos	110（1-4）	359-372
2759	120/4	CAOB	Altai	89.9015	47.2623	Granodiorite		407	407	0.64	2.78	0.1390	0.512591		-0.29	-0.92	2.10	2.08	2.08	0.98	1.14	0.98	1.14	0.98	Wang T et al.	2009	Lithos	110（1-4）	359-372
2760	3170	CAOB	Altai	86.4296	48.3134	C-grained		398	398	4.12	15.72	0.1585	0.512785		-0.19	2.87	4.80	4.81	4.81	0.75	1.01	0.75	1.01	0.75	Wang T et al.	2009	Lithos	110（1-4）	359-372
2761	AT-2	CAOB	Altai, unit 4	88.2431	47.7572	Granite	Altai	330	330	3.42	14.00	0.1476	0.512428	4	-0.25	-4.10	-2.00	-2.03	-2.03	1.25	1.66	1.25	1.66	1.25	Wang T et al.	2009	Lithos	110(1-4)	359-372
2762	175-1	CAOB	Altai, unit 4	86.9750	48.0572	M-grained granite	Yelaman	400	400	3.44	11.84	0.1755	0.512466	8	-0.11	-3.36	-2.27	-2.27	-2.27	1.32	2.71	1.32	2.71	1.32	Wang T et al.	2009	Lithos	110(1-4)	359-372
2763	169/1	CAOB	Altai, unit 4	87.7881	47.7614	F-grained granitois gneiss	Qiemuerqieke	460	460	5.56	20.99	0.1600	0.512467	16	-0.19	-3.34	-1.18	-1.18	-1.18	1.29	1.93	1.29	1.93	1.29	Wang T et al.	2009	Lithos	110(1-4)	359-372
2764	161/1	CAOB	Altai, unit 4	87.5853	47.8858	Granitoid gneiss	Qiemuerqieke	460	460	5.79	25.20	0.1389	0.512466	15	-0.29	-3.36	0.04	0.04	0.04	1.19	1.39	1.19	1.39	1.19	Wang T et al.	2009	Lithos	110(1-4)	359-372
2765	158/1	CAOB	Altai, unit 3	88.0381	48.1161	Qtz granite	Wuliqitawu	375	375	4.38	18.36	0.1443	0.512480	15	-0.27	-3.08	-0.58	-0.58	-0.58	1.17	1.47	1.17	1.47	1.17	Wang T et al.	2009	Lithos	110(1-4)	359-372
2766	157/3	CAOB	Altai, unit 3	88.0200	48.0308	Bi alkaline granitic vein	Jiashikelagai	375	375	8.96	28.25	0.1919	0.512637	18	-0.02	-0.02	0.21	0.21	0.21	1.10	3.55	1.10	3.55	1.10	Wang T et al.	2009	Lithos	110(1-4)	359-372
2767	157/2	CAOB	Altai, unit 3	88.0200	48.0308	Alkaline granitic vein	Jiashikelagai	375	375	7.49	22.03	0.2058	0.512650	12	0.05	0.23	-0.20	-0.20	-0.20	1.11	9.34	1.11	9.34	1.11	Wang T et al.	2009	Lithos	110(1-4)	359-372
2768	149/3	CAOB	Altai, unit 3	88.7117	47.8003	C-grained granodiorie	Wuliqi daqiao	375	375	8.56	48.65	0.1063	0.512446	12	-0.46	-3.75	0.58	0.58	0.58	1.08	1.00	1.08	1.00	1.08	Wang T et al.	2009	Lithos	110(1-4)	359-372
2769	149/2	CAOB	Altai, unit 3	88.7117	47.8003	Granitic gneiss	Wuliqi daqiao	375	375	1.32	7.17	0.1113	0.512573	10	-0.43	-1.27	2.82	2.82	2.82	0.89	0.86	0.89	0.86	0.89	Wang T et al.	2009	Lithos	110(1-4)	359-372
2770	139-1	CAOB	Altai, unit 4	87.9856	47.8261	Bi-granodiorite	Aweitan	400	400	8.58	45.60	0.1137	0.512595	5	-0.42	-0.84	3.40	3.40	3.40	0.87	0.85	0.87	0.85	0.87	Wang T et al.	2009	Lithos	110(1-4)	359-372
2771	138/2	CAOB	Altai, unit 4	87.9856	47.8261	F-grained granodiorite	Aweitan	400	400	9.65	50.35	0.1159	0.512620	15	-0.41	-0.35	3.77	3.77	3.77	0.84	0.83	0.84	0.83	0.84	Wang T et al.	2009	Lithos	110(1-4)	359-372
2772	138/1	CAOB	Altai, unit 4	87.9856	47.8261	C-grained granitodiorite	Aweitan	400	400	3.55	18.37	0.1168	0.512407	14	-0.41	-4.51	-0.43	-0.43	-0.43	1.18	1.17	1.18	1.17	1.18	Wang T et al.	2009	Lithos	110(1-4)	359-372
2773	120/3	CAOB	Altai, unit 3	89.9667	47.2667	M-c grained Bi granodiorite	Keketuohai	407	407	5.00	21.50	0.1408	0.512458	15	-0.28	-3.51	-0.61	-0.61	-0.61	1.20	1.44	1.20	1.44	1.20	Wang T et al.	2009	Lithos	110(1-4)	359-372
2774	112/2	CAOB	Altai, unit 3	89.6556	47.1681	ne-grained tao-mica granit	Shuidianzhan	407	407	3.64	16.43	0.1340	0.512447	12	-0.32	-3.73	-0.47	-0.47	-0.47	1.19	1.34	1.19	1.34	1.19	Wang T et al.	2009	Lithos	110(1-4)	359-372
2775	107/1	CAOB	Altai, unit 4	89.9150	46.8503	C-M-grained Bi granodiorite	S. Tuerhong	400	400	4.48	19.02	0.1425	0.512649	13	-0.28	0.21	2.98	2.98	2.98	0.90	1.07	0.90	1.07	0.90	Wang T et al.	2009	Lithos	110(1-4)	359-372
2776	106/1	CAOB	Altai, unit 4	89.9150	46.8503	Bi monzogranite	S. Tuerhong	400	400	3.67	19.31	0.1151	0.512640	10	-0.41	0.04	4.21	4.21	4.21	0.80	0.79	0.80	0.79	0.80	Wang T et al.	2009	Lithos	110(1-4)	359-372
2777	4123	CAOB	Altai, unit 2	87.0369	48.7828	M-c grained Bi granite	Hanasi	398	398	6.54	26.85	0.1474	0.512440	13	-0.25	-3.85	-1.35	-1.35	-1.35	1.25	1.63	1.25	1.63	1.25	Wang T et al.	2009	Lithos	110(1-4)	359-372
2778	3214	CAOB	Altai, unit 3	88.2764	47.9806	M-grained Bi granodiorite	Shangkelan	220	220	5.08	26.09	0.1177	0.512500	6	-0.40	-2.69	-0.47	-0.47	-0.47	1.03	1.03	1.03	1.03	1.03	Wang T et al.	2009	Lithos	110(1-4)	359-372
2779	3187	CAOB	Altai, unit 2	87.4361	48.8639	M-c grained Bi granodiorite	Tielike	408	408	8.60	40.78	0.1277	0.512428	12	-0.35	-4.10	-0.51	-0.51	-0.51	1.19	1.28	1.19	1.28	1.19	Wang T et al.	2009	Lithos	110(1-4)	359-372
2780	3110	CAOB	Altai, unit 3	88.1267	48.0667	C-grained Bi monozgranite	Wuliqi (west)	375	375	11.84	47.83	0.1496	0.512517	4	-0.24	-2.36	-0.11	-0.11	-0.11	1.13	1.50	1.13	1.50	1.13	Wang T et al.	2009	Lithos	110(1-4)	359-372
2781	3084	CAOB	Altai, unit 4	88.4797	47.6511	Granitoid	Halasu	260	260	9.31	42.68	0.1321	0.512666	13	-0.33	0.55	2.69	2.69	2.69	0.81	0.90	0.81	0.90	0.81	Wang T et al.	2009	Lithos	110(1-4)	359-372
2782	3049	CAOB	Altai, unit 3	89.3694	47.3578	M-c grained Bi granodiorite	Kuerti	416	416	14.15	56.03	0.1529	0.512660	12	-0.22	0.43	2.75	2.75	2.75	0.93	1.23	0.93	1.23	0.93	Wang T et al.	2009	Lithos	110(1-4)	359-372
2783	3045	CAOB	Altai, unit 3	89.3694	47.3578	M-grained M granite	Kuerti	416	416	3.03	10.38	0.1770	0.512610	14	-0.10	-0.55	0.49	0.49	0.49	1.11	2.23	1.11	2.23	1.11	Wang T et al.	2009	Lithos	110(1-4)	359-372
2784	3028	CAOB	Altai, unit 3	89.6833	47.4917	M-c-grained Bi monzogranit	Alaer	209	209	5.75	29.10	0.1194	0.512331	6	-0.39	-5.99	-3.93	-3.93	-3.93	1.31	1.32	1.31	1.32	1.31	Wang T et al.	2009	Lithos	110(1-4)	359-372
2785	177	CAOB	Altai, unit 4	86.9750	48.0572	C-grain Bi granite	Yelaman	400	400	7.29	33.20	0.1378	0.512417	4	-0.30	-4.31	-1.30	-1.30	-1.30	1.25	1.47	1.25	1.47	1.25	Wang T et al.	2009	Lithos	110(1-4)	359-372
2786	168	CAOB	Altai, unit 4	87.7881	47.7614	Granitoid gneiss	Qiemuerqieke	460	460	7.98	35.15	0.1373	0.512410	18	-0.30	-4.45	-0.96	-0.96	-0.96	1.27	1.47	1.27	1.47	1.27	Wang T et al.	2009	Lithos	110(1-4)	359-372
2787	165	CAOB	Altai, unit 4	87.6714	47.8292	Granitoid gneiss	Qiemuerqieke	460	460	5.17	21.75	0.1437	0.512468	13	-0.27	-3.32	-0.21	-0.21	-0.21	1.21	1.48	1.21	1.48	1.21	Wang T et al.	2009	Lithos	110(1-4)	359-372
2788	164	CAOB	Altai, unit 4	87.6714	47.8292	Granitoid gneiss	Qiemuerqieke	460	460	3.74	17.64	0.1280	0.512458	12	-0.35	-3.51	0.52	0.52	0.52	1.15	1.23	1.15	1.23	1.15	Wang T et al.	2009	Lithos	110(1-4)	359-372
2789	153	CAOB	Altai, unit 3	88.6292	47.6419	Light two-mica granite	Wuliqi daqiao	375	375	5.69	16.62	0.2069	0.512527	10	0.05	-2.17	-2.65	-2.65	-2.65	1.29	13.47	1.29	0.01	1.29	Wang T et al.	2009	Lithos	110(1-4)	359-372
2790	132	CAOB	Altai, unit 3	89.8325	47.0272	M-f-grained Bi granodiorite	Tuerhong	407	407	5.34	23.25	0.1389	0.512481	15	-0.29	-3.06	-0.06	-0.06	-0.06	1.15	1.36	1.15	1.36	1.15	Wang T et al.	2009	Lithos	110(1-4)	359-372
2791		CAOB	Altai, unit 3	90.0350	47.3292	Porphyritic Bi monzogranite	Alaer	209	209	8.56	49.44	0.1046	0.512387		-0.47	-4.90	-2.44	-2.44	-2.44	1.19	1.07	1.19	1.07	1.19	Wang T et al.	2009	Lithos	110(1-4)	359-372
2792	97NZS53	CAOB	SMXAB	122.9150	47.6281	Alkali granite	Nianzishan	125	150	17.00	87.50	0.1174	0.512748		-0.40	2.15	3.41	3.66	3.66	0.64	0.64	0.64	0.64	0.64	Wei CS et al.	2001	Acta Petrologica Sinica	17(1)	95-111
2793	97NZS45	CAOB	SMXAB	122.9150	47.6281	Alkali granite	Nianzishan	125	150	19.70	97.50	0.1220	0.512676		-0.38	0.74	1.93	2.17	2.17	0.76	0.79	0.76	0.79	0.76	Wei CS et al.	2001	Acta Petrologica Sinica	17(1)	95-111
2794	97NZS39	CAOB	SMXAB	122.9150	47.6281	Alkali granite	Nianzishan	125	150	17.20	93.80	0.1110	0.512740		-0.44	1.99	3.35	3.63	3.63	0.64	0.61	0.64	0.61	0.65	Wei CS et al.	2001	Acta Petrologica Sinica	17(1)	95-111
2795	97NZS32	CAOB	SMXAB	122.9150	47.6281	Alkali granite	Nianzishan	125	150	16.00	91.10	0.1062	0.512784		-0.46	2.85	4.29	4.58	4.58	0.57	0.52	0.57	0.52	0.57	Wei CS et al.	2001	Acta Petrologica Sinica	17(1)	95-111
2796	97NZS20	CAOB	SMXAB	122.9150	47.6281	Alkali granite	Nianzishan	125	150	15.70	87.00	0.1088	0.512783		-0.45	2.83	4.23	4.51	4.51	0.57	0.53	0.57	0.54	0.58	Wei CS et al.	2001	Acta Petrologica Sinica	17(1)	95-111
2797	97NZS19	CAOB	SMXAB	122.9150	47.6281	Alkali granite	Nianzishan	125	150	7.00	35.50	0.1089	0.512722		-0.45	1.64	3.04	3.32	3.32	0.67	0.62	0.67	0.63	0.67	Wei CS et al.	2001	Acta Petrologica Sinica	17(1)	95-111
2798	97NZS13	CAOB	SMXAB	122.9150	47.6281	Alkali granite	Nianzishan	125	150	13.40	74.50	0.1089	0.512686		-0.45	0.94	2.34	2.62	2.62	0.73	0.68	0.73	0.68	0.73	Wei CS et al.	2001	Acta Petrologica Sinica	17(1)	95-111
2799	97NZS11	CAOB	SMXAB	122.9150	47.6281	Alkali granite	Nianzishan	125	150	16.10	94.20	0.1031	0.512707		-0.48	1.35	2.85	3.14	3.14	0.68	0.61	0.68	0.61	0.69	Wei CS et al.	2001	Acta Petrologica Sinica	17(1)	95-111
2800	97NZS07	CAOB	SMXAB	122.9150	47.6281	Alkali granite	Nianzishan	125	150	20.80	103.30	0.1217	0.512720		-0.38	1.60	2.79	3.04	3.04	0.69	0.71	0.69	0.72	0.69	Wei CS et al.	2001	Acta Petrologica Sinica	17(1)	95-111
2801	97NZS05	CAOB	SMXAB	122.9150	47.6281	Alkali granite	Nianzishan	125	150	19.40	119.30	0.0984	0.512603		-0.50	-0.68	0.90	1.20	1.20	0.84	0.72	0.84	0.73	0.85	Wei CS et al.	2001	Acta Petrologica Sinica	17(1)	95-111
2802	NM07-23	NCC	WNCC	110.9179	41.1189	Charnockites	Guyang-Wuchuan	2512	600	0.79	5.70	0.0843	0.510834	12	-0.57	-35.19	0.90	-26.6	-26.6	3.46	2.71	3.46	2.71	2.78	Zhang XH et al.	2014	Precambrian Research	255	563-582
2803	NM07-21	NCC	WNCC	110.9179	41.1189	Charnockites	Guyang-Wuchuan	2512	600	3.86	16.50	0.1417	0.511827	10	-0.28	-15.82	1.80	-11.6	-11.6	2.50	2.78	2.24	2.78	2.72	Zhang XH et al.	2014	Precambrian Research	255	563-582
2804	NM07-13	NCC	WNCC	110.9179	41.1189	Charnockites	Guyang-Wuchuan	2512	600	0.90	5.80	0.0933	0.511010	12	-0.53	-31.76	1.44	-23.8	-23.8	3.24	2.69	3.24	2.69	2.74	Zhang XH et al.	2014	Precambrian Research	255	563-582
2805	YH-62	CAOB	Tianshan Alatag	95.0833	41.8000	Gneissic Bi granodiorite	Xuankuangzhanghoushan	724	600	6.13	31.53	0.1175	0.511991	15	-0.40	-12.62	-5.31	-6.56	-6.56	1.84	1.83	1.84		1.84	Wang YX et al.	1991	Acta Petrologica Sinica	7(3)	19-26
2806	YH-60	CAOB	Tianshan Alatag	95.0833	41.8000	Gneissic Bi granodiorite	Xuankuangzhanghoushan	724	600	2.24	11.20	0.1210	0.512044	18	-0.38	-11.59	-11.59	-5.79	-5.79	1.78	1.81	1.78		1.76	Wang YX et al.	1991	Acta Petrologica Sinica	7(3)	19-26
2807	YH-4-2	CAOB	Tianshan Alatag	95.1667	42.0833	Tianhe granite	Baishitou	209	209	16.95	26.63	0.3852	0.512902	19	0.96	5.15	0.12	0.12	0.12	0.98	-0.22	0.98		0.98	Wang YX et al.	1991	Acta Petrologica Sinica	7(3)	19-26
2808	YH-33	CAOB	Tianshan Alatag	95.3333	41.8333	Gneissic granite	Pingdingshan	913	600	7.00	32.15	0.1347	0.512042	30	-0.32	-11.63	-4.40	-6.88	-6.88	1.86	2.13	1.86		1.92	Wang YX et al.	1991	Acta Petrologica Sinica	7(3)	19-26
2809	YH-3-2	CAOB	Tianshan Alatag	95.1667	42.0833	Tianhe granite	Baishitou	209	209	12.97	20.69	0.3794	0.512871	16	0.93	4.55	-0.33	-0.33	-0.33	1.02	-0.26	1.02		1.02	Wang YX et al.	1991	Acta Petrologica Sinica	7(3)	19-26
2810	YH-32	CAOB	Tianshan Alatag	95.3333	41.8333	Gneissic granite	Pingdingshan	913	600	5.29	23.90	0.1340	0.512074	21	-0.32	-11.00	-3.69	-6.20	-6.20	1.81	2.05	1.81		1.86	Wang YX et al.	1991	Acta Petrologica Sinica	7(3)	19-26
2811	YH-26	CAOB	Tianshan Alatag	94.6667	41.6333	Gneissic Bi granodiorite	Tianhudong	696	600	4.25	24.12	0.1067	0.511845	28	-0.46	-15.47	-7.47	-8.58	-8.58	2.00	1.85	2.00		1.99	Wang YX et al.	1991	Acta Petrologica Sinica	7(3)	19-26
2812	YH-24	CAOB	Tianshan Alatag	94.6667	41.6333	Gneissic Bi granodiorite	Tianhudong	696	600	3.90	23.16	0.1016	0.511806	19	-0.48	-16.23	-7.78	-8.95	-8.95	2.03	1.82	2.03		2.02	Wang YX et al.	1991	Acta Petrologica Sinica	7(3)	19-26
2813	YH-23-2	CAOB	Tianshan Alatag	93.3333	41.5833	Bi monzogranite	Shalongdongce	470	470	2.93	17.70	0.1000	0.512462	20	-0.49	-3.43	2.37	2.37	2.37	1.01	0.92	1.01		1.01	Wang YX et al.	1991	Acta Petrologica Sinica	7(3)	19-26
2814	YH-22-2	CAOB	Tianshan Alatag	93.3333	41.5833	Bi monzogranite	Shalongdongce	470	470	5.20	23.36	0.1348	0.512589	16	-0.31	-0.96	2.75	2.75	2.75	0.98	1.08	0.98		0.98	Wang YX et al.	1991	Acta Petrologica Sinica	7(3)	19-26
2815	YH-13	CAOB	Tianshan Alatag	94.4167	41.6333	Fine grained monzogranite	Weiya	379	379	2.63	14.24	0.1117	0.512381	25	-0.43	-5.01	-2.40	-0.90	-0.90	1.20	1.15	1.20		1.21	Wang YX et al.	1991	Acta Petrologica Sinica	7(3)	19-26
2816	YH-1-2	CAOB	Tianshan Alatag	95.1667	42.0833	Tianhe granite	Baishitou	209	209	18.89	28.68	0.3985	0.512947	15	1.03	6.03	0.65	0.65	0.65	0.94	-0.17	0.94		0.94	Wang YX et al.	1991	Acta Petrologica Sinica	7(3)	19-26
2817	YH-11	CAOB	Tianshan Alatag	94.4167	41.6333	F-grained monzogranite	Weiya	379	379	2.77	15.31	0.1092	0.512367	20	-0.44	-5.29	-2.60	-1.06	-1.06	1.21	1.14	1.21		1.23	Wang YX et al.	1991	Acta Petrologica Sinica	7(3)	19-26
2818	2033-135	CAOB	Tianshan Alatag	94.3333	41.7167	Bi monzogranite	Weiya	380	380	2.88	16.14	0.1078	0.512349	30	-0.45	-5.64	-2.72	-1.33	-1.33	1.23	1.15	1.23		1.25	Wang YX et al.	1991	Acta Petrologica Sinica	7(3)	19-26
2819	KB13	CAOB	Yili Block	82.3933	43.1821	Granite		947	600	6.92	33.90	0.1234	0.511967		-0.37	-13.09	-4.23	-7.48	-7.48	1.91	1.99	1.91		1.93	Xiong FH et al.	2019	Precambrian Research	328	235-249
2820	KB12	CAOB	Yili Block	82.3620	43.1863	Granite		947	600	5.40	27.10	0.1205	0.511973		-0.39	-12.97	-3.75	-7.13	-7.13	1.88	1.92	1.88		1.89	Xiong FH et al.	2019	Precambrian Research	328	235-249
2821	KB11	CAOB	Yili Block	82.2620	43.1480	Granite		947	600	7.13	35.40	0.1218	0.511969		-0.38	-13.05	-3.99	-7.31	-7.31	1.90	1.95	1.90		1.91	Xiong FH et al.	2019	Precambrian Research	328	235-249
2822	KB10	CAOB	Yili Block	82.2611	43.1583	Granite		947	600	5.93	28.80	0.1245	0.511962		-0.37	-13.19	-4.45	-7.66	-7.66	1.93	2.02	1.93		1.95	Xiong FH et al.	2019	Precambrian Research	328	235-249
2823	KB09	CAOB	Yili Block	82.2611	43.1690	Granite		892	600	6.73	32.40	0.1256	0.512006		-0.36	-12.33	-4.22	-6.88	-6.88	1.86	1.97	1.86		1.89	Xiong FH et al.	2019	Precambrian Research	328	235-249
2824	KB05	CAOB	Yili Block	82.2358	43.1919	Granite		892	600	8.00	41.70	0.1160	0.511954		-0.41	-13.34	-4.14	-7.16	-7.16	1.89	1.86	1.89		1.88	Xiong FH et al.	2019	Precambrian Research	328	235-249
2825	WG-7	CAOB	Yili Block	82.2265	43.1653	Granite		890	600	7.66	36.30	0.1276	0.511995		-0.35	-12.54	-4.66	-7.25	-7.25	1.89	2.04	1.89		1.92	Xiong FH et al.	2019	Precambrian Research	328	235-249
2826	WG-5	CAOB	Yili Block	82.1937	43.1831	Granite		890	600	5.66	16.70	0.2049	0.512413		0.04	-4.39	-5.33	-5.02	-5.02	1.66	12.27	1.66		1.93	Xiong FH et al.	2019	Precambrian Research	328	235-249
2827	WG-2	CAOB	Yili Block	82.0885	43.1517	Granite		890	600	5.46	16.60	0.1988	0.512372		0.01	-5.19	-5.44	-5.35	-5.35	1.71	7.80	1.71		1.96	Xiong FH et al.	2019	Precambrian Research	328	235-249
2828	09AL66	CAOB	Alashan, NH zone	104.4064	40.2089	Two-mica granite	Dabusushan	930	600			0.1343	0.511747	7	-0.32	-17.38	-9.88	-12.60	-12.60	2.32	2.68	2.32	2.68	2.37	Dan W et al.	2014	American Journal of Science	314(1)	110-153
2829	09AL64	CAOB	Alashan, NH zone	104.4064	40.2087	Two-mica granite	Dabusushan	909	600	9.90	40.00	0.1495	0.511993	9	-0.24	-12.58	-7.01	-8.97	-8.97	2.03	2.73	2.03	2.73	2.12	Dan W et al.	2014	American Journal of Science	314(1)	110-153
2830	09AL63	CAOB	Alashan, NH zone	104.4064	40.2087	Two-mica granite	Dabusushan	909	600	7.00	32.00	0.1322	0.511883	9	-0.33	-14.73	-7.15	-9.79	-9.79	2.10	2.36	2.10	2.36	2.14	Dan W et al.	2014	American Journal of Science	314(1)	110-153
2831	09AL62	CAOB	Alashan, NH zone	104.4064	40.2087	Two-mica granite	Dabusushan	909	600	8.60	43.00	0.1208	0.511747	10	-0.39	-17.38	-8.47	-11.6	-11.6	2.24	2.29	2.24	2.29	2.25	Dan W et al.	2014	American Journal of Science	314(1)	110-153
2832	09AL55	CAOB	Alashan, NH zone	104.4262	40.2124	Two-mica granite	Dabusushan	914	600	6.70	24.00	0.1687	0.512181	13	-0.14	-8.91	-5.56	-6.77	-6.77	1.85	3.26	1.85	3.26	2.00	Dan W et al.	2014	American Journal of Science	314(1)	110-153
2833	09AL54	CAOB	Alashan, NH zone	104.4262	40.2124	Two-mica granite	Dabusushan	914	600	7.10	26.00	0.1650	0.512217	12	-0.16	-8.21	-4.43	-5.79	-5.79	1.77	2.90	1.77	2.90	1.91	Dan W et al.	2014	American Journal of Science	314(1)	110-153
2834	09AL53	CAOB	Alashan, NH zone	104.4262	40.2124	Two-mica granite	Dabusushan	914	600	7.70	28.00	0.1662	0.512177	7	-0.16	-8.99	-5.35	-6.66	-6.66	1.84	3.10	1.84	3.10	1.98	Dan W et al.	2014	American Journal of Science	314(1)	110-153
2835	09AL105	CAOB	Alashan, NH zone	104.2392	40.2980	Two-mica granite	Naimumaodao	929	600			0.1359	0.511971	12	-0.31	-13.01	-5.71	-8.36	-8.36	1.98	2.30	1.98	2.30	2.04	Dan W et al.	2014	American Journal of Science	314(1)	110-153
2836	09AL101	CAOB	Alashan, NH zone	104.2392	40.2980	Two-mica granite	Naimumaodao	929	600			0.1265	0.511938	12	-0.36	-13.65	-5.23	-8.28	-8.28	1.98	2.11	1.98	2.11	2.00	Dan W et al.	2014	American Journal of Science	314(1)	110-153
2837	ER7-2	CAOB	NE China	120.8661	51.5992	Syenogranite		851	600			0.1265	0.512202	5	-0.36	-8.51	-0.86	-3.13	-3.13	1.56	1.65	1.56		1.65	Zhao S.	2017	Doctoral Dissertation of Jilin University		
2838	ER2M	CAOB	NE China	120.5000	51.5000	Syenogranite		851	600			0.1151	0.512119	2	-0.41	-10.12	-1.24	-3.88	-3.88	1.62	1.59	1.62		1.59	Zhao S.	2017	Doctoral Dissertation of Jilin University		
2839	ER23-5	CAOB	NE China	120.5000	51.5000	Syenogranite		737	600			0.1275	0.512236	4	-0.35	-7.84	-1.33	-2.54	-2.54	1.51	1.61	1.51		1.61	Zhao S.	2017	Doctoral Dissertation of Jilin University		
2840	ER13-1	CAOB	NE China	120.5000	51.5000	Syenogranite		737	600			0.1249	0.512198	3	-0.37	-8.58	-1.82	-3.09	-3.09	1.56	1.63	1.56		1.63	Zhao S.	2017	Doctoral Dissertation of Jilin University		
2841	12ER36-3	CAOB	NE China	120.5000	51.5000	Monzogranite		455	455			0.1310	0.512332	3	-0.33	-5.97	-2.15	-2.15	-2.15	1.36	1.50	1.36		1.51	Zhao S.	2017	Doctoral Dissertation of Jilin University		
2842	12ER36-2	CAOB	NE China	120.5000	51.5000	Monzogranite		455	455			0.1248	0.512314	8	-0.37	-6.32	-2.14	-2.15	-2.15	1.36	1.43	1.36		1.43	Zhao S.	2017	Doctoral Dissertation of Jilin University		
2843	12ER14-3	CAOB	NE China	120.5000	51.5000	Monzogranite		762	600			0.0896	0.512039	2	-0.54	-11.68	-0.27	-3.48	-3.48	1.59	1.36	1.59		1.30	Zhao S.	2017	Doctoral Dissertation of Jilin University		
2844	12ER14-2	CAOB	NE China	120.5000	51.5000	Monzogranite		762	600			0.0933	0.512102	2	-0.53	-10.46	-3.80	-2.54	-2.54	1.51	1.33	1.51		1.33	Zhao S.	2017	Doctoral Dissertation of Jilin University		

2845	11ER23-4	CAOB	NE China	120.5000	51.5000	Syenogranite		792	600			0.1200	0.512155	3	-0.39	-9.42	-1.66	-3.55	-3.55	1.59	1.62	1.59		1.62	Zhao S.	2017	Doctoral Dissertation of Jilin University		
2846	11ER23-3	CAOB	NE China	120.5000	51.5000	Syenogranite		792	600			0.1237	0.512166	7	-0.37	-9.21	-1.81	-3.62	-3.62	1.60	1.66	1.60		1.66	Zhao S.	2017	Doctoral Dissertation of Jilin University		
2847	XA − 6	CAOB	Northwestern Junggar Basin	85.7122	46.6128	Granite porphyry		420	420	3.19	17.99	0.1072	0.512731		-0.46	1.81	6.61	6.61	6.61	0.62	0.60	0.62	0.60	0.62	Shen P et al.	2012	Lithos	140-141	103-118
2848	XA − 5	CAOB	Northwestern Junggar Basin	85.7389	46.6161	Rhyolite		420	420	2.97	13.72	0.1309	0.512733		-0.33	1.85	5.38	5.38	5.38	0.72	0.77	0.72	0.77	0.72	Shen P et al.	2012	Lithos	140-141	103-118
2849	XA − 3	CAOB	Northwestern Junggar Basin	85.7367	46.6208	Felsite		420	420	2.58	14.47	0.1078	0.512671		-0.45	0.64	5.41	5.41	5.41	0.72	0.69	0.72	0.69	0.72	Shen P et al.	2012	Lithos	140-141	103-118
2850	XA − 2	CAOB	Northwestern Junggar Basin	85.7367	46.6208	Rhyolite		420	420	1.98	10.93	0.1097	0.512654		-0.44	0.31	4.97	4.97	4.97	0.75	0.73	0.75	0.73	0.75	Shen P et al.	2012	Lithos	140-141	103-118
2851	XA − 1	CAOB	Northwestern Junggar Basin	85.7278	46.6083	Rhyolite		420	420	2.90	14.36	0.1223	0.512718		-0.38	1.56	5.55	5.55	5.55	0.71	0.72	0.71	0.72	0.71	Shen P et al.	2012	Lithos	140-141	103-118
2852	S24 − 5	CAOB	Northwestern Junggar Basin	85.7333	46.6111	Andesite		420	420	3.42	16.93	0.1224	0.512686		-0.38	0.94	4.92	4.92	4.92	0.76	0.77	0.76	0.77	0.76	Shen P et al.	2012	Lithos	140-141	103-118
2853	S24 − 1	CAOB	Northwestern Junggar Basin	85.7333	46.6111	Rhyolite		420	420	1.62	8.26	0.1184	0.512648		-0.40	0.20	4.39	4.39	4.39	0.80	0.80	0.80	0.80	0.80	Shen P et al.	2012	Lithos	140-141	103-118
2854	S12 + 51	CAOB	Northwestern Junggar Basin	85.7125	46.6042	Rhyolite		420	420	4.83	23.01	0.1272	0.512704		-0.35	1.29	5.01	5.01	5.01	0.75	0.79	0.75	0.79	0.75	Shen P et al.	2012	Lithos	140-141	103-118
2855	GE1 + 360	CAOB	Northwestern Junggar Basin	85.7122	46.6128	Granite porphyry		420	420	3.57	18.69	0.1158	0.512715		-0.41	1.50	5.84	5.84	5.84	0.68	0.68	0.68	0.68	0.68	Shen P et al.	2012	Lithos	140-141	103-118
2856	GE1 + 280	CAOB	Northwestern Junggar Basin	85.7122	46.6128	Granite porphyry		420	420	3.68	18.99	0.1173	0.512735		-0.40	1.89	6.15	6.15	6.15	0.66	0.66	0.66	0.66	0.66	Shen P et al.	2012	Lithos	140-141	103-118
2857	JSH-2	CAOB	Jilin	128.4169	43.1061	Granodiorite		184	184	3.30	20.07	0.1001	0.512632		-0.49	-0.12	2.15	2.15	2.15	0.79	0.70	0.79			Zhang Y et al.	2016	International Geology Review	58(9)	1-17
2858	JSH-1	CAOB	Jilin	128.4169	43.1061	Granodiorite		184	184	3.50	20.84	0.1023	0.512631		-0.48	-0.14	2.08	2.08	2.08	0.80	0.71	0.80			Zhang Y et al.	2016	International Geology Review	58(9)	1-17
2859	JDT-2	CAOB	Jilin	127.1133	44.2803	Granodiorite		170	170	4.06	24.00	0.1030	0.512654		-0.48	0.31	2.35	2.34	2.34	0.76	0.68	0.76			Zhang Y et al.	2016	International Geology Review	58(9)	1-17
2860	JDT-1	CAOB	Jilin	127.1133	44.2803	Granodiorite		170	170	4.61	24.89	0.1128	0.512645		-0.43	0.14	1.96	1.96	1.96	0.80	0.76	0.80			Zhang Y et al.	2016	International Geology Review	58(9)	1-17
2861	FAP-2	CAOB	Jilin	127.2689	44.4000	Monzogranite		167	167	4.89	26.61	0.1119	0.512606		-0.43	-0.62	1.18	1.18	1.18	0.86	0.82	0.86			Zhang Y et al.	2016	International Geology Review	58(9)	1-17
2862	FAP-1	CAOB	Jilin	127.2689	44.4000	Monzogranite		167	167	3.83	19.90	0.1172	0.512609		-0.40	-0.57	1.13	1.13	1.13	0.86	0.86	0.86			Zhang Y et al.	2016	International Geology Review	58(9)	1-17
2863	DSH-2	CAOB	Jilin	127.8458	43.5917	Granodiorite		187	187	2.63	16.06	0.0997	0.512528		-0.49	-2.15	0.17	0.17	0.17	0.96	0.83	0.96			Zhang Y et al.	2016	International Geology Review	58(9)	1-17
2864	DSH-1	CAOB	Jilin	127.8458	43.5917	Granodiorite		187	187	2.75	16.58	0.1010	0.512551		-0.49	-1.70	0.59	0.59	0.59	0.92	0.81	0.92			Zhang Y et al.	2016	International Geology Review	58(9)	1-17
2865	DHS-4	CAOB	Jilin	126.3203	43.4889	Granodiorite		170	170	4.03	23.72	0.1035	0.512603		-0.47	-0.68	1.34	1.34	1.34	0.85	0.76	0.85			Zhang Y et al.	2016	International Geology Review	58(9)	1-17
2866	DHS-3	CAOB	Jilin	126.3203	43.4889	Granodiorite		170	170	4.09	23.82	0.1046	0.512600		-0.47	-0.74	1.26	1.26	1.26	0.85	0.77	0.85			Zhang Y et al.	2016	International Geology Review	58(9)	1-17
2867	DHS-2	CAOB	Jilin	126.3203	43.4889	Granodiorite		170	170	3.24	19.50	0.1012	0.512611		-0.49	-0.53	1.55	1.54	1.54	0.83	0.73	0.83			Zhang Y et al.	2016	International Geology Review	58(9)	1-17
2868	DHS-1	CAOB	Jilin	126.3203	43.4889	Granodiorite		170	170	3.66	21.81	0.1022	0.512611		-0.48	-0.53	1.52	1.52	1.52	0.83	0.74	0.83			Zhang Y et al.	2016	International Geology Review	58(9)	1-17
2869	SD-47	CAOB	East Tianshan	93.4500	41.5389	Granite	Shadong	239	239	4.38	25.20	0.1090	0.512401		-0.45	-4.62	-1.96	-1.95	-1.95	1.17	1.09	1.17	0.20	1.17	Chen C et al.	2018	Journal of Earth Science	29	114-129
2870	SD-42	CAOB	East Tianshan	93.4500	41.5389	Granite	Shadong	239	239	4.78	24.00	0.1250	0.512397		-0.36	-4.70	-2.53	-2.51	-2.51	1.22	1.29	1.22	1.30	1.22	Chen C et al.	2018	Journal of Earth Science	29	114-129
2871	SD-41	CAOB	East Tianshan	93.4500	41.5389	Granite	Shadong	239	239	3.50	24.60	0.0890	0.512363		-0.55	-5.36	-2.10	-2.08	-2.08	1.18	0.96	1.18	0.97	1.19	Chen C et al.	2018	Journal of Earth Science	29	114-129
2872	SD-33	CAOB	East Tianshan	93.4500	41.5389	Granite	Shadong	239	239	4.24	23.20	0.1150	0.512420		-0.42	-4.25	-1.77	-1.76	-1.76	1.15	1.13	1.15	1.13	1.16	Chen C et al.	2018	Journal of Earth Science	29	114-129
2873	XT91	CAOB	usun-Awulale mountain, Tiansl	82.4167	43.7500	Sodic dacite		260	260	2.44	16.24	0.0909	0.512560	11	-0.54	-1.52	1.99	1.99	1.99	0.87	0.73	0.87		0.87	Xiong XL et al.	2001	Acta Petrologica Sinica	17(4)	514-522
2874	XT90	CAOB	usun-Awulale mountain, Tiansl	82.4167	43.7500	Sodic dacite		260	260	2.46	15.82	0.0940	0.512570	13	-0.52	-1.33	2.08	2.08	2.08	0.86	0.74	0.86		0.86	Xiong XL et al.	2001	Acta Petrologica Sinica	17(4)	514-522
2875	XT196	CAOB	usun-Awulale mountain, Tiansl	82.4167	43.7500	Qtz albite porphyry	Tetuedaban	260	260	1.73	10.38	0.1009	0.512642	10	-0.49	0.08	3.26	3.26	3.26	0.76	0.69	0.76		0.76	Xiong XL et al.	2001	Acta Petrologica Sinica	17(4)	514-522
2876	XT195	CAOB	usun-Awulale mountain, Tiansl	82.4167	43.7500	Qtz albite porphyry	Tetuedaban	260	260	1.90	11.14	0.1032	0.512589	11	-0.48	-0.96	2.15	2.15	2.15	0.85	0.77	0.85		0.85	Xiong XL et al.	2001	Acta Petrologica Sinica	17(4)	514-522
2877	XT162	CAOB	usun-Awulale mountain, Tiansl	82.4167	43.7500	Albite porphyry		260	260	3.23	20.25	0.0953	0.512567	13	-0.52	-1.38	1.98	1.98	1.98	0.87	0.75	0.87		0.87	Xiong XL et al.	2001	Acta Petrologica Sinica	17(4)	514-522
2878	XT161	CAOB	usun-Awulale mountain, Tiansl	82.4167	43.7500	Albite porphyry		260	260	3.23	20.36	0.0961	0.512578	10	-0.51	-1.17	2.17	2.17	2.17	0.85	0.74	0.85		0.85	Xiong XL et al.	2001	Acta Petrologica Sinica	17(4)	514-522
2879	XT104	CAOB	usun-Awulale mountain, Tiansl	82.4167	43.7500	Qtz albite porphyry	Mosizaote	248	248	2.25	14.19	0.0959	0.512547	8	-0.51	-1.78	1.41	1.41	1.41	0.90	0.78	0.90		0.90	Xiong XL et al.	2001	Acta Petrologica Sinica	17(4)	514-522
2880	QZZ-5	NCC	NCC	114.8436	39.3917	Porphyries	Taihang orogen	143	150			0.1040	0.511826	9	-0.47	-15.84	-14.2	-14.1	-14.1	2.08	1.83	2.08	1.84	2.09	Gao YF et al.	2013	Journal of Asian Earth Sciences	78	143-159
2881	MSG-5	NCC	NCC	114.8436	39.3917	Porphyries	Taihang orogen	143	150			0.1169	0.511739	7	-0.41	-17.54	-16.1	-16.0	-16.0	2.24	2.21	2.24	2.22	2.24	Gao YF et al.	2013	Journal of Asian Earth Sciences	78	143-159
2882	MSG-2	NCC	NCC	114.8436	39.3917	Porphyries	Taihang orogen	143	150			0.0872	0.511724	5	-0.56	-17.83	-15.8	-15.7	-15.7	2.22	1.71	2.22	1.72	2.22	Gao YF et al.	2013	Journal of Asian Earth Sciences	78	143-159
2883	MJC-8	NCC	NCC	114.8436	39.3917	Porphyries	Taihang orogen	143	150			0.0878	0.511694	7	-0.55	-18.41	-16.4	-16.3	-16.3	2.27	1.76	2.27	1.76	2.27	Gao YF et al.	2013	Journal of Asian Earth Sciences	78	143-159
2884	MJC-6	NCC	NCC	114.8436	39.3917	Porphyries	Taihang orogen	143	150			0.0847	0.511694	7	-0.57	-18.41	-16.4	-16.3	-16.3	2.26	1.72	2.26	1.72	2.27	Gao YF et al.	2013	Journal of Asian Earth Sciences	78	143-159
2885	MJC-14	NCC	NCC	114.8436	39.3917	Porphyries	Taihang orogen	143	150			0.0881	0.511707	8	-0.55	-18.16	-16.2	-16.1	-16.1	2.25	1.75	2.25	1.75	2.25	Gao YF et al.	2013	Journal of Asian Earth Sciences	78	143-159
2886	MJC-12	NCC	NCC	114.8436	39.3917	Porphyries	Taihang orogen	143	150			0.0877	0.511708	8	-0.55	-18.14	-16.2	-16.1	-16.1	2.24	1.74	2.24	1.74	2.25	Gao YF et al.	2013	Journal of Asian Earth Sciences	78	143-159
2887	HMT-38	NCC	NCC	114.8436	39.3917	Porphyries	Taihang orogen	143	150			0.0926	0.511650	9	-0.53	-19.27	-17.4	-17.3	-17.3	2.34	1.88	2.34	1.89	2.35	Gao YF et al.	2013	Journal of Asian Earth Sciences	78	143-159
2888	HMT-37	NCC	NCC	114.8436	39.3917	Diorite porphyry	Taihang orogen	143	150			0.0882	0.511674	9	-0.55	-18.80	-16.8	-16.7	-16.7	2.30	1.79	2.30	1.79	2.30	Gao YF et al.	2013	Journal of Asian Earth Sciences	78	143-159
2889	HMT-35	NCC	NCC	114.8436	39.3917	Porphyries	Taihang orogen	143	150			0.0855	0.511591	9	-0.57	-20.42	-18.4	-18.3	-18.3	2.43	1.85	2.43	1.85	2.43	Gao YF et al.	2013	Journal of Asian Earth Sciences	78	143-159
2890	LD-26	CAOB	NE China	120.5000	49.5000	Rhyolite		106	150	4.39	18.65	0.1434	0.512513		-0.27	-2.44	-1.72	-1.42	-1.42	1.05	1.38	1.05			Gao XF et al.	2005	Acta Petrologica Sinica	21(3)	737-748
2891	LD-25	CAOB	NE China	120.5000	49.5000	Rhyolite		106	150	5.19	23.16	0.1365	0.512538		-0.31	-1.95	-1.14	-0.80	-0.80	1.00	1.21	1.00			Gao XF et al.	2005	Acta Petrologica Sinica	21(3)	737-748
2892	LD-20	CAOB	NE China	120.5000	49.5000	Dacite		106	150	4.87	18.41	0.1611	0.512565		-0.18	-1.42	-0.95	-0.74	-0.74	1.00	1.69	1.00			Gao XF et al.	2005	Acta Petrologica Sinica	21(3)	737-748
2893	LD-19	CAOB	NE China	120.5000	49.5000	Andesite		106	150	6.21	23.94	0.1580	0.512571		-0.20	-1.31	-0.79	-0.57	-0.57	0.98	1.58	0.98			Gao XF et al.	2005	Acta Petrologica Sinica	21(3)	737-748
2894	LD-17	CAOB	NE China	120.5000	49.5000	Dacite		106	150	6.21	23.95	0.1579	0.512583		-0.20	-1.07	-0.55	-0.33	-0.33	0.96	1.55	0.96			Gao XF et al.	2005	Acta Petrologica Sinica	21(3)	737-748
2895	LD-15	CAOB	NE China	120.5000	49.5000	Dacite		106	150	6.22	24.00	0.1579	0.512584		-0.20	-1.05	-0.53	-0.31	-0.31	0.96	1.54	0.96			Gao XF et al.	2005	Acta Petrologica Sinica	21(3)	737-748
2896	21LD-4	CAOB	NE China	120.5000	49.5000	Adakite		106	150	2.14	12.66	0.1030	0.512659		-0.48	0.41	1.67	2.20	2.20	0.76	0.68	0.76			Gao XF et al.	2005	Acta Petrologica Sinica	21(3)	737-748
2897	21LD-3	CAOB	NE China	120.5000	49.5000	Adakite		106	150	2.19	12.69	0.1051	0.512603		-0.47	-0.68	0.56	1.08	1.08	0.85	0.77	0.85			Gao XF et al.	2005	Acta Petrologica Sinica	21(3)	737-748
2898	21LD-2	CAOB	NE China	120.5000	49.5000	Adakite		106	150	2.37	13.80	0.1046	0.512622		-0.47	-0.31	0.93	1.45	1.45	0.82	0.74	0.82			Gao XF et al.	2005	Acta Petrologica Sinica	21(3)	737-748
2899	21LD-1	CAOB	NE China	120.5000	49.5000	Adakite		106	150	2.29	12.64	0.1103	0.512656		-0.44	0.34	1.50	1.99	1.99	0.78	0.73	0.78			Gao XF et al.	2005	Acta Petrologica Sinica	21(3)	737-748
2900	ZJG-3	NCC	NCC	115.3090	39.8565	Diorite		128	150	6.83	36.80	0.1130	0.511953		-0.43	-13.36	-12.0	-11.8	-11.8	1.89	1.81	1.89			Chen B et al.	2009	Lithos	110(1-4)	110(1-4)
2901	ZJG-1	NCC	NCC	115.3090	39.8565	Diorite		128	150	6.42	33.90	0.1153	0.511852		-0.41	-15.33	-14.0	-13.8	-13.8	2.06	2.00	2.06			Chen B et al.	2009	Lithos	110(1-4)	110(1-4)
2902	HL-7	NCC	NCC	116.0340	40.2378	Diorite		128	150	9.90	58.00	0.1040	0.511780		-0.47	-16.74	-15.2	-15.0	-15.0	2.15	1.90	2.15			Chen B et al.	2009	Lithos	110(1-4)	110(1-4)
2903	HL-5	NCC	NCC	116.0453	40.2998	Diorite		128	150	7.86	44.00	0.1088	0.511785		-0.45	-16.64	-15.2	-15.0	-15.0	2.15	1.98	2.15			Chen B et al.	2009	Lithos	110(1-4)	110(1-4)
2904	HL-1	NCC	NCC	116.0453	40.2998	Diorite		128	150	8.31	47.50	0.1066	0.511794		-0.46	-16.46	-15.0	-14.7	-14.7	2.14	1.92	2.14			Chen B et al.	2009	Lithos	110(1-4)	110(1-4)
2905	DHN-3	NCC	NCC	115.2132	39.7833	Diorite		128	150	4.95	32.10	0.0939	0.511740		-0.52	-17.52	-15.8	-15.5	-15.5	2.20	1.79	2.20			Chen B et al.	2009	Lithos	110(1-4)	110(1-4)
2906	14BDG-80	CAOB	NE China	119.1389	49.9792	Granodioritic porphyry		230	230	4.60	24.30	0.1145	0.512574	7	-0.42	-1.25	1.17	1.17	1.17	0.91	0.88	0.91			Mi K et al.	2017	Ore Geology Reviews	81	154-172
2907	14BDG-36	CAOB	NE China	119.1333	49.9806	Granodioritic porphyry		230	230	4.60	22.20	0.1267	0.512541	7	-0.36	-1.89	0.16	0.16	0.16	0.99	1.07	0.99			Mi K et al.	2017	Ore Geology Reviews	81	154-172
2908	14BDG-11	CAOB	NE China	119.1278	49.9778	Granodioritic porphyry		230	230	3.80	19.50	0.1177	0.512540	7	-0.40	-1.91	0.41	0.41	0.41	0.97	0.97	0.97			Mi K et al.	2017	Ore Geology Reviews	81	154-172
2909	2064	CAOB	Khentey uplift	106.9763	47.8816	Granite	Bogd Uul	206	206	10.36	44.66	0.1402	0.512442		-0.29	-3.82	1.30	-2.34	-2.34	1.17	1.47	1.17			Sodnom K et al.	2012	Bull. Nagoya Univ. Museum	28	45-59
2910	2057	CAOB	Khentey uplift	106.5881	47.5180	Granite	Bogd Uul	206	206	8.97	45.31	0.1197	0.512445		-0.39	-3.76	1.40	-1.74	-1.74	1.13	1.14	1.13			Sodnom K et al.	2012	Bull. Nagoya Univ. Museum	28	45-59
2911	2053	CAOB	Khentey uplift	107.0024	47.7908	Granite	Bogd Uul	208	208	5.24	31.22	0.1015	0.512404		-0.48	-4.56	0.70	-2.04	-2.04	1.15	1.01	1.15			Sodnom K et al.	2012	Bull. Nagoya Univ. Museum	28	45-59
2912	2034	CAOB	Khentey uplift	107.0282	47.8003	Granite	Bogd Uul	208	208	4.13	22.55	0.1107	0.512433		-0.44	-4.00	1.20	-1.72	-1.72	1.13	1.06	1.13			Sodnom K et al.	2012	Bull. Nagoya Univ. Museum	28	45-59
2913	PQ1205	CAOB	Northern margin of Tarim	77.2500	40.3333	Diorite dyke		275	275			0.1296	0.512423		-0.34	-4.19	-1.84	-1.84	-1.84	1.19	1.32	1.19	1.32	1.20	Cao J et al.	2013	Acta Petrologica Sinica	29(10)	3336-3352
2914	PQ1125	CAOB	Northern margin of Tarim	77.2500	40.3333	Granitic dyke		275	275			0.1341	0.512486		-0.32	-2.97	-0.77	-0.77	-0.77	1.10	1.27	1.10	1.27	1.11	Cao J et al.	2013	Acta Petrologica Sinica	29(10)	3336-3352
2915	YD-7	CAOB	East Tianshan	92.5333	42.1222	Tonalite		335	335	1.86	9.47	0.1187	0.512772	0	-0.40	2.62	5.96	5.96	5.96	0.60	0.61	0.60	0.61	0.61	Wang YH et al.	2017	Mineralium Deposita	53(5)	629-662
2916	YD-6	CAOB	East Tianshan	92.5333	42.1222	Tonalite		335	335	2.27	11.60	0.1183	0.512728	0	-0.40	1.75	5.11	5.10	5.10	0.67	0.68	0.67	0.68	0.68	Wang YH et al.	2017	Mineralium Deposita	53(5)	629-660
2917	YD-3104-45-6	CAOB	East Tianshan	92.5333	42.1222	Tonalite		335	335	1.07	5.11	0.1266	0.512819	0	-0.36	3.52	6.53	6.52	6.52	0.56	0.58	0.56	0.58	0.56	Wang YH et al.	2017	Mineralium Deposita	53(5)	629-658
2918	YD2-6307-31	CAOB	East Tianshan	92.5333	42.1222	Tonalite		335	335	2.32	15.70	0.0893	0.512710	0	-0.55	1.41	6.01	6.01	6.01	0.60	0.54	0.60	0.54	0.60	Wang YH et al.	2017	Mineralium Deposita	53(5)	629-656
2919	YD2-6307-29	CAOB	East Tianshan	92.5333	42.1222	Tonalite		335	335	2.36	13.30	0.1073	0.512796	0	-0.45	3.08	6.92	6.91	6.91	0.53	0.51	0.53	0.51	0.53	Wang YH et al.	2017	Mineralium Deposita	53(5)	629-654
2920	YD2-6307-25	CAOB	East Tianshan	92.5333	42.1222	Tonalite		335	335	2.94	16.90	0.1052	0.512793	0	-0.47	3.03	6.96	6.95	6.95	0.52	0.50	0.52	0.50	0.53	Wang YH et al.	2017	Mineralium Deposita	53(5)	629-652
2921	YD2-6307-24	CAOB	East Tianshan	92.5333	42.1222	Tonalite		335	335	2.68	14.80	0.1095	0.512819	0	-0.44	3.53	7.27	7.26	7.26	0.50	0.49	0.50	0.49	0.50	Wang YH et al.	2017	Mineralium Deposita	53(5)	629-650
2922	YD2-6307-23	CAOB	East Tianshan	92.5333	42.1222	Tonalite		335	335	2.13	12.20	0.1056	0.512799	0	-0.46	3.14	7.05	7.04	7.04	0.52	0.50	0.52	0.50	0.52	Wang YH et al.	2017	Mineralium Deposita	53(5)	629-648
2923	YD-005-969-22	CAOB	East Tianshan	92.5333	42.1222	Tonalite		335	335	5.55	38.50	0.0872	0.512785	0	-0.56	2.88	7.57	7.56	7.56	0.47	0.44	0.47	0.44	0.48	Wang YH et al.	2017	Mineralium Deposita	53	629–647
2924	YD-005-968-23	CAOB	East Tianshan	92.5333	42.1222	Tonalite		335	335	1.48	8.60	0.1040	0.512813	0	-0.47	3.41	7.38	7.37	7.37	0.49	0.47	0.49	0.47	0.49	Wang YH et al.	2017	Mineralium Deposita	53	629–647
2925	YX2	CAOB	Northern margin of Tarim	83.5000	41.5000	Rhyolite		287	287			0.1065	0.512100		-0.46	-10.49	-7.20	-7.19	-7.19	1.64	1.49	1.64	1.49	1.65	Liu HQ et al.	2014	Lithos	204	59-72
2926	YX1	CAOB	Northern margin of Tarim	83.5000	41.5000	Rhyolite		287	287			0.1068	0.512073		-0.46	-11.02	-7.73	-7.73	-7.73	1.68	1.53	1.68	1.54	1.69	Liu HQ et al.	2014	Lithos	204	59-72
2927	YM16-2	CAOB	Northern margin of Tarim	82.1667	41.2833	Rhyolite		287	287			0.1057	0.512295		-0.46	-6.69	-3.36	-3.36	-3.36	1.32	1.21	1.32	1.21	1.33	Liu HQ et al.	2014	Lithos	204	59-72
2928	WQ09-2	CAOB	Northern margin of Tarim	82.4167	42.2667	Rhyolite		287	287			0.1098	0.512169		-0.44	-9.15	-5.97	-5.96	-5.96	1.54	1.44	1.54	1.44	1.54	Liu HQ et al.	2014	Lithos	204	59-72
2929	WQ09-1	CAOB	Northern margin of Tarim	82.4167	42.2667	Rhyolite		287	287			0.1137	0.512197		-0.42	-8.60	-5.56	-5.56	-5.56	1.50	1.45	1.50	1.45	1.51	Liu HQ et al.	2014	Lithos	204	59-72
2930	SX1	CAOB	Northern margin of Tarim	83.5000	41.5000	Rhyolite		287	287			0.1026	0.512389		-0.48	-4.86	-1.41	-1.41	-1.41	1.17	1.04	1.17	1.05	1.17	Liu HQ et al.	2014	Lithos	204	59-72
2931	NK1-3	CAOB	Northern margin of Tarim	83.5000	41.5000	Rhyolite		287	287			0.1067	0.512179		-0.46	-8.95	-5.66	-5.66	-5.66	1.51	1.38	1.51	1.38	1.52	Liu HQ et al.	2014	Lithos	204	59-72
2932	MN1(Liu)	CAOB	Tianshan	82.0000	40.0000	granite		290	290	18.30	105.90	0.1053	0.512532		-0.46	-2.07	1.32	1.32	1.32	0.95	0.87	0.95			Liu HQ et al.	2014	Lithos	204	59-72
2933	HA2-2	CAOB	Northern margin of Tarim	83.5000	41.5000	Rhyolite		287	287	13.70	75.60	0.1099	0.512149		-0.44	-9.54	-6.36	-6.36	-6.36	1.57	1.47	1.57	1.47	1.58	Liu HQ et al.	2014	Lithos	204	59-72
2934	HA1	CAOB	Northern margin of Tarim	83.5000	41.5000	Rhyolite		287	287	13.50	82.30	0.0994	0.512137		-0.49	-9.77	-6.21	-6.21	-6.21	1.56	1.35	1.56	1.35	1.56	Liu HQ et al.	2014	Lithos	204	59-72
2935	71	CAOB	tau-borokonu mountain, Tians	81.1667	45.2000	Alkali feldspar granite		290	290	3.30	14.91	0.1341	0.512643		-0.32	0.10	2.48	2.41	2.41	0.86	0.97	0.86		0.86	Zhou TX et al.	1996	Scientia Geologica Sinica	（1）	71-79
2936	701	CAOB	tau-borokonu mountain, Tians	81.1667	45.2000	Alkali feldspar granite		290	290	3.21	13.01	0.1483	0.512656		-0.25	0.35	2.19	2.14	2.14	0.88	1.15	0.88		0.88	Zhou TX et al.	1996	Scientia Geologica Sinica	（1）	71-79
2937	107	CAOB	tau-borokonu mountain, Tians	81.1667	45.2000	Monzogranite		290	290	6.51	24.54	0.1605	0.512732		-0.18	1.83	3.21	3.17	3.17	0.79	1.20	0.79		0.80	Zhou TX et al.	1996	Scientia Geologica Sinica	（1）	71-79
2938	132	CAOB	tau-borokonu mountain, Tians	81.1667	45.2000	Granodiorite		290	290	3.65	14.27	0.1550	0.512701		-0.21	1.23	2.79	2.77	2.77	0.83	1.17	0.83		0.83	Zhou TX et al.	1996	Scientia Geologica Sinica	（1）	71-79
2939	09AL41	CAOB	Alashan, NH zone	104.7692	40.1583	MME	Bayannuoergong	278	278			0.1237	0.511776	7	-0.37	-16.81	-14.31	-14.2	-14.2	2.20	2.32	2.20			Dan W et al.	2015	Lithos	230	133-145
2940	09AL40	CAOB	Alashan, NH zone	104.7692	40.1583	MME	Bayannuoergong	278	278			0.1228	0.511752	8	-0.38	-17.28	-14.74	-14.7	-14.7	2.23	2.33	2.23			Dan W et al.	2015	Lithos	230	133-146
2941	09AL29	CAOB	Alashan, NH zone	104.7692	40.1583	Monzogranite	Bayannuoergong	278	278			0.1036	0.511791	3	-0.47	-16.52	-13.33	-13.2	-13.2	2.12	1.88	2.12			Dan W et al.	2015	Lithos	230	133-147
2942	09AL28-1	CAOB	Alashan, NH zone	104.8881	40.0953	MME	Bayannuoergong	278	278			0.1317	0.511847	11	-0.33	-15.43	-13.19	-13.1	-13.1	2.11	2.41	2.11			Dan W et al.	2015	Lithos	230	133-148

2943	09AL19-1	CAOB	Alashan, NH zone	104.7692	40.1583	Monzogranite	Bayannuoergong	278	278			0.0772	0.511932	7	-0.61	-13.77	-9.65	-9.5	-9.5	1.82	1.36	1.82			Dan W et al.	2015	Lithos	230	133-149
2944	09AL163	CAOB	Alashan, NH zone	105.4922	40.2386	MME	Yamaitu	270	270			0.1356	0.511944	6	-0.31	-13.54	-11.45	-11.4	-11.4	1.96	2.34	1.96			Dan W et al.	2015	Lithos	230	133-150
2945	09AL155	CAOB	Alashan, NH zone	105.4922	40.2386	Granodiorite	Yamaitu	270	270			0.1013	0.511879	16	-0.49	-14.81	-11.52	-11.5	-11.5	1.97	1.72	1.97			Dan W et al.	2015	Lithos	230	133-151
2946	09AL154	CAOB	Alashan, NH zone	105.3544	40.1841	Granodiorite	Yamaitu	270	270			0.1580	0.511916	13	-0.20	-14.08	-12.76	-12.8	-12.8	2.06	3.35	2.06			Dan W et al.	2015	Lithos	230	133-152
2947	09AL152	CAOB	Alashan, NH zone	105.3544	40.1841	Granodiorite	Yamaitu	270	270			0.1513	0.511937	8	-0.23	-13.67	-12.11	-12.1	-12.1	2.01	2.94	2.01			Dan W et al.	2015	Lithos	230	133-153
2948	09AL12-3	CAOB	Alashan, NH zone	104.9717	39.9286	Monzogranite		279	279	7.93	40.70	0.1178	0.512061	7	-0.40	-11.26	-8.31	-8.45	-8.45	1.73	1.73	1.73	1.73	1.72	Dan W et al.	2015	Lithos	230	133-154
2949	12HSB03-4	CAOB	CAOB	74.3000	51.8500	Tonalite porphyry		490	490	3.78	19.44	0.1178	0.512677		-0.40	0.76	5.71	5.70	5.70	0.75	0.75	0.75	0.76	0.76	Shen P et al.	2015	Economic Geology	110	1861-1878
2950	12HSB03-3	CAOB	CAOB	74.3000	51.8500	Tonalite porphyry		490	490	1.54	7.33	0.1269	0.512665		-0.35	0.53	4.89	4.89	4.89	0.82	0.85	0.82	0.855	0.82	Shen P et al.	2015	Economic Geology	110	1861-1878
2951	ZH 9	CAOB	Kazakhstan	71.0300	51.4300	Granitoid		447	447	12.98	2.74	0.1276	0.512540	2	-0.35	-1.91	2.00	2.03	2.03	1.02	1.08	1.02			Kröner A et al.	2008	Journal of Asian Earth Sciences	32(2–4)	118-130
2952	ZH 4	CAOB	Kazakhstan	71.0300	51.4300	Granitoid		453	453	13.20	2.78	0.1276	0.512561	3	-0.35	-1.50	2.50	2.49	2.49	0.98	1.04	0.98			Kröner A et al.	2008	Journal of Asian Earth Sciences	32(2–4)	118-130
2953	VE 9	CAOB	Kazakhstan	79.9800	48.0000	Granitoid		250	250	106.30	28.79	0.1637	0.512854	5	-0.17	4.21	5.30	5.27	5.27	0.59	0.90	0.59			Kröner A et al.	2008	Journal of Asian Earth Sciences	32(2–4)	118-130
2954	AK 21	CAOB	Kazakhstan	71.5200	52.5000	Granitoid		457	457	9.91	2.35	0.1435	0.512696	4	-0.27	1.13	4.20	4.23	4.23	0.84	0.99	0.84			Kröner A et al.	2008	Journal of Asian Earth Sciences	32(2–4)	118-130
2955	09AL262	NCC	WNCC	105.9347	38.6094	Two-mica granite	Helanshan	1956	600	4.67	23.00	0.1225	0.511629	7	-0.38	-19.68	-1.12	-14.00	-14.00	2.44	2.53	2.44	2.53	2.50	Dan W et al.	2014	Precambrian Research	254	59-72
2956	09AL260	NCC	WNCC	105.9347	38.6094	Two-mica granite	Helanshan	1956	600	11.00	51.80	0.1284	0.511728	5	-0.35	-17.75	-0.66	-12.5	-12.5	2.32	2.53	2.32	2.53	2.46	Dan W et al.	2014	Precambrian Research	254	59-72
2957	09AL259	NCC	WNCC	105.9347	38.6094	Two-mica granite	Helanshan	1956	600	2.44	13.50	0.1088	0.511557	8	-0.45	-21.09	0.91	-14.4	-14.4	2.47	2.30	2.47	2.30	2.34	Dan W et al.	2014	Precambrian Research	254	59-72
2958	09AL258	NCC	WNCC	105.9347	38.6094	Two-mica granite	Helanshan	1956	600	5.32	29.80	0.1078	0.511532	6	-0.45	-21.57	0.67	-14.8	-14.8	2.50	2.32	2.50	2.32	2.36	Dan W et al.	2014	Precambrian Research	254	59-72
2959	09AL233	NCC	WNCC	106.2925	39.3047	orphyritic K-feldspar granit	Helanshan	1947	600	11.70	76.80	0.0919	0.511287	8	-0.53	-26.35	-0.24	-18.3	-18.3	2.79	2.32	2.79	2.32	2.42	Dan W et al.	2014	Precambrian Research	254	59-72
2960	09AL232	NCC	WNCC	106.2925	39.3047	orphyritic K-feldspar granit	Helanshan	1947	600	10.90	90.80	0.0725	0.511039	6	-0.63	-31.19	-0.24	-21.7	-21.7	3.06	2.27	3.06	2.27	2.42	Dan W et al.	2014	Precambrian Research	254	59-72
2961	09AL227	NCC	WNCC	106.2842	39.2894	orphyritic K-feldspar granit	Helanshan	1947	600	17.60	117.00	0.0909	0.511249	6	-0.54	-27.10	-0.73	-19.0	-19.0	2.85	2.35	2.85	2.35	2.46	Dan W et al.	2014	Precambrian Research	254	59-72
2962	09AL225	NCC	WNCC	106.2842	39.2894	orphyritic K-feldspar granit	Helanshan	1947	600	17.00	87.10	0.1181	0.511640	7	-0.40	-19.47	0.11	-13.5	-13.5	2.39	2.40	2.39	2.40	2.39	Dan W et al.	2014	Precambrian Research	254	59-72
2963	VE4	CAOB	Kazakhstan	80.5000	48.0000	Riebeckite granite		250	250	10.80	52.00	0.0865	0.512731		-0.56	1.81	1.81	5.33	5.33	0.59	0.50	0.59		0.67	Heinhorst J et al.	2000	Tectonophysics	328(1)	69-87
2964	VE3	CAOB	Kazakhstan	80.5000	48.0000	Riebeckite granite		250	250	10.02	39.60	0.1012	0.512850		-0.49	4.14	4.14	7.18	7.18	0.43	0.41	0.43		0.48	Heinhorst J et al.	2000	Tectonophysics	328(1)	69-87
2965	VE2	CAOB	Kazakhstan	80.5000	48.0000	Riebeckite granite		250	250	23.62	75.30	0.0643	0.512824		-0.67	3.63	3.63	7.85	7.85	0.38	0.33	0.38		0.52	Heinhorst J et al.	2000	Tectonophysics	328(1)	69-87
2966	VE1	CAOB	Kazakhstan	80.5000	48.0000	Leucogranite		290	290	6.80	34.60	0.1305	0.512558		-0.34	-1.56	-1.56	0.89	0.89	0.98	1.08	0.98		0.94	Heinhorst J et al.	2000	Tectonophysics	328(1)	69-87
2967	ST26	CAOB	Kazakhstan	69.9000	51.6000	Granite		450	450	1.51	0.80	0.1045	0.512511		-0.47	-2.48	-2.48	2.82	2.82	0.95	0.89	0.95		1.02	Heinhorst J et al.	2000	Tectonophysics	328(1)	69-87
2968	NurK4	CAOB	Kazakhstan	73.2000	48.9000	Leucogranite		330	330	3.61	17.40	0.0979	0.512529		-0.50	-2.13	-2.13	2.03	2.03	0.92	0.82	0.92		0.99	Heinhorst J et al.	2000	Tectonophysics	328(1)	69-87
2969	NurK3	CAOB	Kazakhstan	73.2000	48.9000	Hb granodiorite		325	325	3.51	17.80	0.1127	0.512496		-0.43	-2.77	-2.77	0.71	0.71	1.02	0.99	1.02		1.04	Heinhorst J et al.	2000	Tectonophysics	328(1)	69-87
2970	KoG25	CAOB	Kazakhstan	75.3000	46.7000	Granophyre		300	300	2.05	11.80	0.0949	0.512743		-0.52	2.05	2.05	5.94	5.94	0.58	0.52	0.58		0.65	Heinhorst J et al.	2000	Tectonophysics	328(1)	69-87
2971	Do90	CAOB	Kazakhstan	76.5000	45.4000	Qtz diorite		300	300	5.20	22.20	0.1409	0.512782		-0.28	2.81	2.81	4.94	4.94	0.66	0.77	0.66		0.59	Heinhorst J et al.	2000	Tectonophysics	328(1)	69-87
2972	Do89	CAOB	Kazakhstan	76.5000	45.4000	Monzodiorite		300	300	13.40	55.90	0.1441	0.512818		-0.27	3.51	3.51	5.53	5.53	0.61	0.73	0.61		0.53	Heinhorst J et al.	2000	Tectonophysics	328(1)	69-87
2973	Do88	CAOB	Kazakhstan	76.5000	45.4000	Granodiorite		300	300	6.06	26.80	0.1341	0.512747		-0.32	2.13	2.13	4.52	4.52	0.69	0.77	0.69		0.64	Heinhorst J et al.	2000	Tectonophysics	328(1)	69-87
2974	Do87	CAOB	Kazakhstan	76.5000	45.4000	Qtz monzodiorite		450	450	4.55	21.10	0.1311	0.512406		-0.33	-4.53	-4.53	-0.76	-0.76	1.24	1.37	1.24		1.18	Heinhorst J et al.	2000	Tectonophysics	328(1)	69-87
2975	BatG7	CAOB	Kazakhstan	74.0000	48.2000	Leucogranite		310	310	3.71	22.70	0.0929	0.512397		-0.53	-4.70	-4.70	-0.59	-0.59	1.12	0.95	1.12		1.20	Heinhorst J et al.	2000	Tectonophysics	328(1)	69-87
2976	BATG5	CAOB	Kazakhstan	74.0000	48.2000	Leucogranite		285	285	0.91	7.50	0.1012	0.512296		-0.49	-6.67	-6.67	-3.20	-3.20	1.31	1.16	1.31		1.36	Heinhorst J et al.	2000	Tectonophysics	328(1)	69-87
2977	AqsK9	CAOB	Kazakhstan	74.3000	47.5000	Leucogranite		330	330	3.70	18.10	0.1074	0.512491		-0.45	-2.87	-2.87	0.89	0.89	1.01	0.95	1.01		1.05	Heinhorst J et al.	2000	Tectonophysics	328(1)	69-87
2978	AQSG3	CAOB	Kazakhstan	74.3000	47.5000	Leucogranite		285	285	1.86	14.40	0.0696	0.512427		-0.65	-4.12	-4.12	0.51	0.51	1.01	0.77	1.01		1.15	Heinhorst J et al.	2000	Tectonophysics	328(1)	69-87
2979	AQ7	CAOB	Kazakhstan	79.8000	46.5000	Granite		300	300	3.60	15.90	0.1371	0.512798		-0.30	3.12	3.12	5.40	5.40	0.62	0.70	0.62		0.56	Heinhorst J et al.	2000	Tectonophysics	328(1)	69-87
2980	AQ4	CAOB	Kazakhstan	79.8000	46.5000	Granodiorite		300	300	4.39	19.30	0.1378	0.512750		-0.30	2.18	2.18	4.44	4.44	0.70	0.80	0.70		0.64	Heinhorst J et al.	2000	Tectonophysics	328(1)	69-87
2981	AQ3	CAOB	Kazakhstan	79.8000	46.5000	Granodiorite		300	300	4.04	17.50	0.1398	0.512812		-0.29	3.39	3.39	5.57	5.57	0.61	0.70	0.61		0.54	Heinhorst J et al.	2000	Tectonophysics	328(1)	69-87
2982	AQ13	CAOB	Kazakhstan	79.8000	46.5000	Porphyry stock		300	300	1.92	10.70	0.1194	0.512633		-0.39	-0.10	-0.10	2.86	2.86	0.83	0.84	0.83		0.82	Heinhorst J et al.	2000	Tectonophysics	328(1)	69-87
2983	AQ1	CAOB	Kazakhstan	79.8000	46.5000	Qtz diorite		300	300	7.89	33.10	0.1448	0.512809		-0.26	3.34	3.34	5.32	5.32	0.63	0.75	0.63		0.54	Heinhorst J et al.	2000	Tectonophysics	328(1)	69-87
2984	AK39	CAOB	Kazakhstan	70.2000	51.5000	Granodiorite		450	450	2.42	13.20	0.1113	0.512546		-0.43	-1.79	-1.79	3.11	3.11	0.93	0.90	0.93		0.96	Heinhorst J et al.	2000	Tectonophysics	328(1)	69-87
2985	Ak249	CAOB	Kazakhstan	70.2000	51.5000	Qtz monzodiorite		450	450	1.19	8.30	0.1254	0.512609		-0.36	-0.57	-0.57	3.53	3.53	0.90	0.93	0.90		0.86	Heinhorst J et al.	2000	Tectonophysics	328(1)	69-87
2986	Ak248	CAOB	Kazakhstan	70.2000	51.5000	Granodiorite		450	450	3.26	17.80	0.1111	0.512546		-0.44	-1.79	-1.79	3.12	3.12	0.93	0.90	0.93		0.96	Heinhorst J et al.	2000	Tectonophysics	328(1)	69-87
2987	Ak245	CAOB	Kazakhstan	70.2000	51.5000	Qtz monzodiorite		450	450	4.44	19.90	0.1335	0.512631		-0.32	-0.14	-0.14	3.49	3.49	0.90	0.99	0.90		0.83	Heinhorst J et al.	2000	Tectonophysics	328(1)	69-87
2988	ZK9901	CAOB	Altai	89.7000	46.7400	Diorite		320	320	4.89	24.50	0.1216	0.512800		-0.38	3.16	6.24	6.23	6.23	0.57	0.58	0.57			Qian ZZ et al.	2018	Journal of Asian Earth Sciences	159	294-307
2989	YH15-99	CAOB	East Tianshan	94.6667	42.3333	Pyroxene diorite		291	291	5.99	21.10	0.1729	0.512900		-0.12	5.11	6.42	5.99	5.99	0.56	0.93	0.56	0.86	0.53	Wang YF et al.	2018	Journal of Asian Earth Sciences	153	282-306
2990	YH15-96	CAOB	East Tianshan	94.6667	42.3333	Pyroxene diorite		291	291	6.30	23.50	0.1633	0.512900		-0.17	5.11	5.77	6.35	6.35	0.54	0.76	0.54	0.85	0.58	Wang YF et al.	2018	Journal of Asian Earth Sciences	153	282-306
2991	YH15-180	CAOB	East Tianshan	94.6667	42.3333	Qtz diorite		444	444	3.41	17.10	0.1215	0.512700		-0.38	1.21	6.34	5.47	5.47	0.73	0.74	0.73	0.67	0.64	Wang YF et al.	2018	Journal of Asian Earth Sciences	153	282-306
2992	YH15-176	CAOB	East Tianshan	94.6667	42.3333	Pyroxene diorite		291	291	6.14	21.30	0.1756	0.512900		-0.11	5.11	6.23	5.89	5.89	0.57	1.00	0.57	0.94	0.55	Wang YF et al.	2018	Journal of Asian Earth Sciences	153	282-306
2993	YH15-124	CAOB	East Tianshan	94.6667	42.3333	Qtz diorite		444	444	3.63	18.20	0.1215	0.512700		-0.38	1.21	6.31	5.47	5.47	0.73	0.74	0.73	0.68	0.61	Wang YF et al.	2018	Journal of Asian Earth Sciences	153	282-306
2994	YH15-004	CAOB	East Tianshan	94.6667	42.3333	Granite		325	325	3.49	17.60	0.1208	0.512800		-0.39	3.16	5.40	6.31	6.31	0.57	0.57	0.57	0.65	0.66	Wang YF et al.	2018	Journal of Asian Earth Sciences	153	282-306
2995	YH15-002	CAOB	East Tianshan	94.6667	42.3333	Granite		325	325	3.34	16.90	0.1204	0.512800		-0.39	3.16	5.76	6.33	6.33	0.57	0.57	0.57	0.62	0.67	Wang YF et al.	2018	Journal of Asian Earth Sciences	153	282-306
2996	YH15-001	CAOB	East Tianshan	94.6667	42.3333	Granite		325	325	2.14	10.90	0.1199	0.512700		-0.39	1.21	5.17	4.40	4.40	0.72	0.73	0.72	0.67	0.62	Wang YF et al.	2018	Journal of Asian Earth Sciences	153	282-306
2997	S102-08	CAOB	East Tianshan	94.6667	42.3333	Qtz diorite		444	444	3.08	15.80	0.1187	0.512800		-0.40	3.16	6.84	7.57	7.57	0.56	0.56	0.56	0.63	0.66	Wang YF et al.	2018	Journal of Asian Earth Sciences	153	282-306
2998	M-511	CAOB	Transbaikalia and Mongolia	111.4500	52.3000	AFS rhyolite	Khorinsk	285	285	8.95	45.96	0.1177	0.512357		-0.40	-5.48	-2.61	-2.61	-2.61	1.26	1.26	1.26	1.26	1.26	Jahn BM et al.	2009	Lithos	113（3-4）	521-539
2999	M-501	CAOB	Transbaikalia and Mongolia	111.4500	52.3000	Syenite	Khorinsk	280	280	5.43	34.99	0.0938	0.512311		-0.52	-6.38	-2.70	-2.70	-2.70	1.27	1.07	1.27	1.07	1.27	Jahn BM et al.	2009	Lithos	113（3-4）	521-539
3000	M-492	CAOB	Transbaikalia and Mongolia	111.4500	52.3000	AFS rhyolite	Khorinsk	285	285	6.74	31.91	0.1276	0.512327		-0.35	-6.07	-3.55	-3.55	-3.55	1.34	1.45	1.34	1.45	1.34	Jahn BM et al.	2009	Lithos	113（3-4）	521-539
3001	M-350	CAOB	Transbaikalia and Mongolia	111.4500	52.3000	PA granite	Khorinsk	280	280	7.20	38.91	0.1119	0.512379		-0.43	-5.05	-2.02	-2.02	-2.02	1.21	1.15	1.21	1.15	1.21	Jahn BM et al.	2009	Lithos	113（3-4）	521-539
3002	M-347	CAOB	Transbaikalia and Mongolia	111.4500	52.3000	AFS syenite	Khorinsk	280	280	10.18	62.51	0.0985	0.512295		-0.50	-6.69	-3.18	-3.18	-3.18	1.30	1.13	1.30	1.13	1.30	Jahn BM et al.	2009	Lithos	113（3-4）	521-539
3003	M-340	CAOB	Transbaikalia and Mongolia	111.4500	52.3000	PA granite	Khorinsk	280	280	5.06	30.63	0.1000	0.512352		-0.49	-5.58	-2.12	-2.12	-2.12	1.22	1.07	1.22	1.07	1.22	Jahn BM et al.	2009	Lithos	113（3-4）	521-539
3004	L728	CAOB	Transbaikalia and Mongolia	112.0333	52.1000	PA syenite	Atha	210	210	11.00	65.00	0.1023	0.512634		-0.48	-0.08	2.45	2.45	2.45	0.79	0.71	0.79	0.71	0.79	Jahn BM et al.	2009	Lithos	113（3-4）	521-539
3005	L727	CAOB	Transbaikalia and Mongolia	112.0333	52.1000	PA syenite	Atha	210	210	12.00	65.00	0.1116	0.512652		-0.43	0.27	2.55	2.55	2.55	0.78	0.74	0.78	0.74	0.78	Jahn BM et al.	2009	Lithos	113（3-4）	521-539
3006	L726	CAOB	Transbaikalia and Mongolia	112.0333	52.1000	PA syenite	Atha	210	210	10.00	58.00	0.1042	0.512620		-0.47	-0.35	2.13	2.13	2.13	0.81	0.74	0.81	0.74	0.81	Jahn BM et al.	2009	Lithos	113（3-4）	521-539
3007	L725	CAOB	Transbaikalia and Mongolia	112.0333	52.1000	PA granite	Atha	210	210	11.00	64.00	0.1039	0.512616		-0.47	-0.43	2.06	2.06	2.06	0.82	0.74	0.82	0.74	0.82	Jahn BM et al.	2009	Lithos	113（3-4）	521-539
3008	L722	CAOB	Transbaikalia and Mongolia	112.0333	52.1000	PA granite	Atha	210	210	12.00	70.00	0.1036	0.512619		-0.47	-0.37	2.12	2.12	2.12	0.81	0.74	0.81	0.74	0.81	Jahn BM et al.	2009	Lithos	113（3-4）	521-539
3009	L716	CAOB	Transbaikalia and Mongolia	112.0333	52.1000	PA granite	Atha	210	210	11.00	65.00	0.1023	0.512622		-0.48	-0.31	2.22	2.22	2.22	0.81	0.72	0.81	0.72	0.81	Jahn BM et al.	2009	Lithos	113（3-4）	521-539
3010	KH14	CAOB	Transbaikalia and Mongolia	111.0833	51.1667	Afs syenite	Kharitonovo	230	230	12.00	67.00	0.1083	0.512468		-0.45	-3.32	-0.72	-0.72	-0.72	1.06	0.99	1.06	0.99	1.06	Jahn BM et al.	2009	Lithos	113（3-4）	521-539
3011	B69-2	CAOB	Transbaikalia and Mongolia	112.0333	52.1000	Afs syenite	Atha	230	230	5.00	33.00	0.0916	0.512514		-0.53	-2.42	0.67	0.67	0.67	0.95	0.79	0.95	0.79	0.95	Jahn BM et al.	2009	Lithos	113（3-4）	521-539
3012	B69-1	CAOB	Transbaikalia and Mongolia	112.0333	52.1000	Afs syenite	Atha	230	230	10.00	67.00	0.0902	0.512515		-0.54	-2.40	0.73	0.73	0.73	0.95	0.78	0.95	0.78	0.95	Jahn BM et al.	2009	Lithos	113（3-4）	521-539
3013	B590	CAOB	Transbaikalia and Mongolia	111.0833	51.2167	PA granite	Oshurkovo	150	150	6.92	38.50	0.1087	0.512438		-0.45	-3.90	-2.22	-2.22	-2.22	1.12	1.03	1.12	1.03	1.12	Jahn BM et al.	2009	Lithos	113（3-4）	521-539
3014	B361-20	CAOB	Transbaikalia and Mongolia	111.4500	52.3000	Syenite	Khorinsk	280	280	3.30	17.49	0.1141	0.512238		-0.42	-7.80	-4.85	-4.85	-4.85	1.44	1.39	1.44	1.39	1.44	Jahn BM et al.	2009	Lithos	113（3-4）	521-539
3015	B144	CAOB	Transbaikalia and Mongolia	111.4500	52.3000	Syenite	Khorinsk	270	270	6.62	35.95	0.1113	0.512297		-0.43	-6.65	-3.71	-3.71	-3.71	1.34	1.27	1.34	1.27	1.34	Jahn BM et al.	2009	Lithos	113（3-4）	521-539
3016	B111	CAOB	Transbaikalia and Mongolia	111.4500	52.3000	Monzonite	Khorinsk	280	280	6.50	36.31	0.1082	0.512263		-0.45	-7.32	-4.15	-4.15	-4.15	1.38	1.28	1.38	1.28	1.38	Jahn BM et al.	2009	Lithos	113（3-4）	521-539
3017	A66	CAOB	Transbaikalia and Mongolia	112.0333	52.1000	PA granite	Atha	210	210	6.00	32.00	0.1134	0.512585		-0.42	-1.03	1.20	1.20	1.20	0.89	0.86	0.89	0.86	0.89	Jahn BM et al.	2009	Lithos	113（3-4）	521-539
3018	A65	CAOB	Transbaikalia and Mongolia	112.0333	52.1000	PA granite	Atha	210	210	6.00	30.00	0.1209	0.512556		-0.39	-1.60	0.43	0.43	0.43	0.95	0.98	0.95	0.98	0.95	Jahn BM et al.	2009	Lithos	113（3-4）	521-539
3019	A519	CAOB	Transbaikalia and Mongolia	112.0333	52.1000	PA syenite	Atha	220	220	5.33	28.23	0.1141	0.512689		-0.42	0.99	3.31	3.31	3.31	0.73	0.71	0.73	0.71	0.73	Jahn BM et al.	2009	Lithos	113（3-4）	521-539
3020	A516a	CAOB	Transbaikalia and Mongolia	112.0333	52.1000	PA syenite	Atha	220	220	7.00	42.00	0.1008	0.512718		-0.49	1.56	4.25	4.25	4.25	0.65	0.58	0.65	0.58	0.65	Jahn BM et al.	2009	Lithos	113（3-4）	521-539
3021	A13-1	CAOB	Transbaikalia and Mongolia	112.0333	52.1000	PA granite	Atha	210	210	17.00	82.00	0.1253	0.512664		-0.36	0.51	2.42	2.42	2.42	0.79	0.84	0.79	0.84	0.79	Jahn BM et al.	2009	Lithos	113（3-4）	521-539
3022	A13	CAOB	Transbaikalia and Mongolia	112.0333	52.1000	PA granite	Atha	210	210	3.80	17.00	0.1351	0.512657		-0.31	0.37	2.02	2.02	2.02	0.82	0.96	0.82	0.96	0.82	Jahn BM et al.	2009	Lithos	113（3-4）	521-539
3023	A102	CAOB	Transbaikalia and Mongolia	111.0833	51.2167	PA granite	Oshurkovo	150	150	5.45	33.40	0.0986	0.512427		-0.50	-4.12	-2.24	-2.24	-2.24	1.12	0.96	1.12	0.96	1.12	Jahn BM et al.	2009	Lithos	113（3-4）	521-539
3024	65-1	CAOB	Transbaikalia and Mongolia	111.0833	51.2167	AFS syenite	Oshurkovo	130	150	6.12	48.10	0.0769	0.512387		-0.61	-4.90	-2.91	-2.60	-2.60	1.15	0.85	1.15	0.85	1.16	Jahn BM et al.	2009	Lithos	113（3-4）	521-539
3025	23743	CAOB	Transbaikalia and Mongolia	108.0000	51.0000	Syenite		130	150	6.12	48.10	0.0775	0.512387		-0.61	-4.90	-2.92	-2.61	-2.61	1.15	0.85	1.15			Jahn BM et al.	2009	Lithos	113（3-4）	521-539
3026	37-1	CAOB	Transbaikalia and Mongolia	111.0833	51.2167	Monzodiorite	Oshurkovo	130	150	25.11	153.10	0.0992	0.512437		-0.50	-3.92	-2.30	-2.05	-2.05	1.11	0.95	1.11	0.95	1.11	Jahn BM et al.	2009	Lithos	113（3-4）	521-539
3027	34-1	CAOB	Transbaikalia and Mongolia	111.0833	51.2167	Monzonite	Oshurkovo	130	150	14.46	93.20	0.0938	0.512451		-0.52	-3.65	-1.94	-1.68	-1.68	1.08	0.89	1.08	0.89	1.08	Jahn BM et al.	2009	Lithos	113（3-4）	521-539
3028	3070	CAOB	Transbaikalia and Mongolia	111.4500	52.3000	AFS granite	Khorinsk	280	280	7.98	44.69	0.1080	0.512311		-0.45	-6.38	-3.21	-3.21	-3.21	1.31	1.21	1.31	1.21	1.31	Jahn BM et al.	2009	Lithos	113（3-4）	521-539
3029	3008	CAOB	Transbaikalia and Mongolia	111.0833	51.1667	AFS granite	Kharitonovo	230	230	4.80	31.00	0.0936	0.512336		-0.52	-5.89	-2.87	-2.87	-2.87	1.24	1.03	1.24	1.03	1.24	Jahn BM et al.	2009	Lithos	113（3-4）	521-539
3030	870	CAOB	Transbaikalia and Mongolia	111.0833	51.1667	AFS syenite	Kharitonovo	230	230	14.00	77.00	0.1099	0.512513		-0.44	-2.44	0.11	0.11	0.11	1.00	0.94	1.00	0.94	1.00	Jahn BM et al.	2009	Lithos	113（3-4）	521-539
3031	866	CAOB	Transbaikalia and Mongolia	111.0833	51.1667	PA syenite	Kharitonovo	230	230	29.00	155.00	0.1131	0.512496		-0.43	-2.77	-0.32	-0.32	-0.32	1.03	0.99	1.03	0.99	1.03	Jahn BM et al.	2009	Lithos	113（3-4）	521-539
3032	536	CAOB	Transbaikalia and Mongolia	111.4500	52.3000	AFS granite	Khorinsk	280	280	12.00	59.00	0.1230	0.512279		-0.37	-7.00	-4.37	-4.37	-4.37	1.40	1.46	1.40	1.46	1.40	Jahn BM et al.	2009	Lithos	113（3-4）	521-539
3033	530	CAOB	Transbaikalia and Mongolia	111.4500	52.3000	AFS syenite	Khorinsk	280	280	4.00	21.00	0.1152	0.512415		-0.41	-4.35	-1.44	-1.44	-1.44	1.16	1.14	1.16	1.14	1.16	Jahn BM et al.	2009	Lithos	113（3-4）	521-539
3034	479	CAOB	Transbaikalia and Mongolia	111.4500	52.3000	PA granite	Khorinsk	280	280	6.07	35.98	0.1020	0.512253		-0.48	-7.51	-4.13	-4.13	-4.13	1.38	1.22	1.38	1.22	1.38	Jahn BM et al.	2009	Lithos	113（3-4）	521-539
3035	14	CAOB	Transbaikalia and Mongolia	111.0833	51.2167	AFS syenite	Oshurkovo	130	150	7.14	55.90	0.0772	0.512358		-0.61	-5.46	-3.48	-3.17	-3.17	1.20	0.88	1.20	0.88	1.21	Jahn BM et al.	2009	Lithos	113（3-4）	521-539
3036	X-532	CAOB	East Tianshan	95.8667	42.6000	Peraluminous granite	Tulaergengou	275	275			0.1286	0.512906		-0.35	5.23	7.63	7.62	7.62	0.42	0.44	0.42	0.44	0.42	Tang JH et al.	2008	Acta Petrologica Sinica	24(5)	921-946
3037	X-495	CAOB	East Tianshan	95.8667	42.6000	Peraluminous granite	Jingerquan	254	254			0.1195	0.512841		-0.39	3.96	6.47	6.46	6.46	0.50	0.50	0.50	0.50	0.50	Tang JH et al.	2008	Acta Petrologica Sinica	24(5)	921-946
3038	X-272	CAOB	East Tianshan	95.8667	42.6000	Peraluminous granite	Huangshannan	254	254			0.1362	0.512906		-0.31	5.23	7.20	7.19	7.19	0.44	0.48	0.44	0.48	0.44	Tang JH et al.	2008	Acta Petrologica Sinica	24(5)	921-946
3039	E930-1.2	CAOB	SMXAB	112.3131	43.6361	Alkaline granite	Saiyinwusu	279	279	6.41	25.82	0.1500	0.512664	1	-0.24	0.51	2.17	2.17	2.17	0.87	1.16	0.87	1.17	0.87	Tong Y et al.	2015	Journal of Asian Earth Sciences	97	320-336
3040	E930-1.1	CAOB	SMXAB	112.3131	43.6361	Alkaline granite	Saiyinwusu	279	279	5.52	23.89	0.1397	0.512716	6	-0.29	1.52	3.56	3.55	3.55	0.75	0.89	0.75	0.90	0.76	Tong Y et al.	2015	Journal of Asian Earth Sciences	97	320-336

3041	E930-1	CAOB	SMXAB	112.3131	43.6361	Alkaline granite	Saiyinwusu	279	279	6.92	26.78	0.1562	0.512881	2	-0.21	4.74	6.19	6.18	6.18	0.54	0.71	0.54	0.72	0.54	Tong Y et al.	2015	Journal of Asian Earth Sciences	97	320-336
3042	E925-7.1	CAOB	SMXAB	112.0825	44.8208	Alkaline granite	Hongol	276	276	5.44	23.82	0.1381	0.512839	1	-0.30	3.92	5.98	5.98	5.98	0.55	0.63	0.55	0.63	0.56	Tong Y et al.	2015	Journal of Asian Earth Sciences	97	320-336
3043	E925-7	CAOB	SMXAB	112.0825	44.8208	Alkaline granite	Hongol	276	276	4.83	23.43	0.1248	0.512695	9	-0.37	1.11	3.66	3.64	3.64	0.74	0.78	0.74	0.78	0.75	Tong Y et al.	2015	Journal of Asian Earth Sciences	97	320-336
3044	E101-1.3	CAOB	SMXAB	112.9725	43.6719	Alkaline granite	Baolag	285	285	7.96	31.99	0.1506	0.512824	3	-0.23	3.63	5.31	5.30	5.30	0.62	0.79	0.62	0.79	0.62	Tong Y et al.	2015	Journal of Asian Earth Sciences	97	320-336
3045	E101-1.2	CAOB	SMXAB	112.9725	43.6719	Alkaline granite	Baolag	285	285	5.24	24.90	0.1272	0.512835	10	-0.35	3.84	6.38	6.37	6.37	0.53	0.56	0.53	0.56	0.53	Tong Y et al.	2015	Journal of Asian Earth Sciences	97	320-336
3046	E101-1.1	CAOB	SMXAB	112.9725	43.6719	Alkaline granite	Baolag	285	285	7.35	30.00	0.1483	0.512862	11	-0.25	4.37	6.13	6.13	6.13	0.55	0.67	0.55	0.68	0.55	Tong Y et al.	2015	Journal of Asian Earth Sciences	97	320-336
3047	13PY-8	CAOB	Beishan	91.5600	40.6000	Granite		276	276	18.70	88.10	0.1293	0.512396		-0.34	-4.72	-2.35	-2.35	-2.35	1.23	1.36	1.23			Xue SC et al.	2018	Lithos	14	617-629
3048	13PY-7	CAOB	Beishan	91.5600	40.6000	Granite		276	276	9.02	43.70	0.1257	0.512443		-0.36	-3.80	-1.31	-1.31	-1.31	1.15	1.22	1.15			Xue SC et al.	2018	Lithos	14	617-629
3049	13PY-10	CAOB	Beishan	91.5600	40.6000	Granite		276	276	7.21	37.00	0.1187	0.512415		-0.40	-4.35	-1.61	-1.61	-1.61	1.17	1.18	1.17			Xue SC et al.	2018	Lithos	14	617-629
3050	823_4	CAOB	Kangur-juerotage,Tianshan	93.0167	42.5833	Diorite	Chatcaltag	277	277	3.88	20.87	0.1123	0.512830	34	-0.43	3.75	6.73	6.73	6.73	0.49	0.48	0.49		0.49	Li JY et al.	2006	Acta Geoscientica Sinica	(05)	424-446
3051	823_11	CAOB	Kangur-juerotage,Tianshan	93.0167	42.5833	Anorthosite	Chatcaltag	277	277	0.25	1.02	0.1400	0.512934	26	-0.29	5.77	7.78	7.78	7.78	0.41	0.45	0.41		0.41	Li JY et al.	2006	Acta Geoscientica Sinica	(05)	424-446
3052	821_9	CAOB	Kangur-juerotage,Tianshan	93.0167	42.5833	Diorite	Chatcaltag	269	269	8.34	37.05	0.1362	0.512761	57	-0.31	2.40	4.48	4.48	4.48	0.67	0.77	0.67		0.67	Li JY et al.	2006	Acta Geoscientica Sinica	(05)	424-446
3053	821_7	CAOB	Kangur-juerotage,Tianshan	93.0167	42.5833	Diorite	Chatcaltag	269	269	1.76	5.21	0.2044	0.513090	10	0.04	8.82	8.55	8.55	8.55	0.34	0.98	0.34		0.34	Li JY et al.	2006	Acta Geoscientica Sinica	(05)	424-446
3054	ZYF50	CAOB	NE China	118.0000	43.5200	Basaltic trachy-andesite		270	270	5.46	23.67	0.1390	0.512691		-0.29	1.03	-0.94	3.02	3.01	0.79	0.94	0.79			Zhu YF et al.	2001	Geological Magazine	138(2)	101-115
3055	ZYF60	CAOB	NE China	118.0000	43.5200	Trachy-andesite		270	270	6.21	25.01	0.1500	0.512683		-0.24	0.88	-0.73	2.49	2.49	0.83	1.12	0.83			Zhu YF et al.	2001	Geological Magazine	138(2)	101-115
3056	ZYF2	CAOB	NE China	118.0000	43.5200	Trachy-andesite		270	270	3.68	15.29	0.1460	0.512591		-0.26	-0.92	-2.68	0.83	0.86	0.97	1.26	0.97			Zhu YF et al.	2001	Geological Magazine	138(2)	101-115
3057	ZYF73	CAOB	NE China	118.0000	43.5200	Trachy-andesite		270	270	6	25.1	0.1450	0.512547		-0.26	-1.78	-3.57	0.01	0.03	1.04	1.34	1.04			Zhu YF et al.	2001	Geological Magazine	138(2)	101-115
3058	ZYF62	CAOB	NE China	118.0000	43.5200	Trachy-andesite		270	270	2.23	7.57	0.1780	0.512614		-0.10	-0.47	-1.11	0.18	0.18	1.02	2.28	1.02			Zhu YF et al.	2001	Geological Magazine	138(2)	101-115
3059	ZYF25	CAOB	NE China	118.0000	43.5200	Trachy-andesite		270	270	3.51	11.19	0.1900	0.512664		-0.03	0.51	0.26	0.74	0.76	0.97	3.10	0.97			Zhu YF et al.	2001	Geological Magazine	138(2)	101-115
3060	ZYF8	CAOB	NE China	118.0000	43.5200	Basaltic andesite		270	270	3.71	11.33	0.1980	0.512708		0.01	1.37	1.41	1.32	1.33	0.92	4.25	0.92			Zhu YF et al.	2001	Geological Magazine	138(2)	101-115
3061	09ZK09	CAOB	Northern margin of Tarim	77.1333	40.4167	Granite		272	272			0.1012	0.512449		-0.49	-3.69	-0.37	-0.37	-0.37	1.07	0.95	1.07	0.95	1.07	Zhang CL and Zou HB.	2013	Lithos	172-173	47-60
3062	09ZK04	CAOB	Northern margin of Tarim	77.1333	40.4167	Granite		272	272			0.1073	0.512345		-0.45	-5.72	-2.61	-2.61	-2.61	1.25	1.15	1.25	1.15	1.26	Zhang CL and Zou HB.	2013	Lithos	172-173	47-60
3063	09ZK01	CAOB	Northern margin of Tarim	77.1333	40.4167	Granite		272	272			0.1021	0.512397		-0.48	-4.70	-1.41	-1.41	-1.41	1.15	1.03	1.15	1.03	1.16	Zhang CL and Zou HB.	2013	Lithos	172-173	47-60
3064	09HLJ V-9	CAOB	Northern margin of Tarim	77.1667	40.3500	Granite	Halajun	271	271			0.1147	0.512481		-0.42	-3.06	-0.23	-0.23	-0.23	1.06	1.03	1.06	1.03	1.06	Zhang CL and Zou HB.	2013	Lithos	172-173	47-60
3065	09HLJ V-6	CAOB	Northern margin of Tarim	77.1667	40.3500	Granite	Halajun	271	271			0.1191	0.512501		-0.39	-2.67	0.01	0.01	0.01	1.04	1.05	1.04	1.05	1.04	Zhang CL and Zou HB.	2013	Lithos	172-173	47-60
3066	09HLJ V-2	CAOB	Northern margin of Tarim	77.1667	40.3500	Granite	Halajun	271	271			0.1109	0.512520		-0.44	-2.30	0.67	0.67	0.67	0.98	0.93	0.98	0.94	0.99	Zhang CL and Zou HB.	2013	Lithos	172-173	47-60
3067	09HLJ V-1	CAOB	Northern margin of Tarim	77.1667	40.3500	Granite	Halajun	271	271			0.1167	0.512481		-0.41	-3.06	-0.29	-0.30	-0.30	1.06	1.05	1.06	1.05	1.07	Zhang CL and Zou HB.	2013	Lithos	172-173	47-60
3068	09GL10	CAOB	Northern margin of Tarim	77.1167	40.5167	Granite		277	277			0.1117	0.512479		-0.43	-3.10	-0.10	-0.10	-0.10	1.05	1.00	1.05	1.00	1.06	Zhang CL and Zou HB.	2013	Lithos	172-173	47-60
3069	09GL05	CAOB	Northern margin of Tarim	77.1167	40.5167	Granite		277	277			0.0918	0.512551		-0.53	-1.70	2.01	2.01	2.01	0.88	0.75	0.88	0.75	0.88	Zhang CL and Zou HB.	2013	Lithos	172-173	47-60
3070	09GL03	CAOB	Northern margin of Tarim	77.1167	40.5167	Granite		277	277			0.0936	0.512346		-0.52	-5.70	-2.05	-2.06	-2.06	1.21	1.02	1.21	1.02	1.22	Zhang CL and Zou HB.	2013	Lithos	172-173	47-60
3071	09GL01	CAOB	Northern margin of Tarim	77.1167	40.5167	Granite		277	277			0.1155	0.512477		-0.41	-3.14	-0.27	-0.27	-0.27	1.06	1.04	1.06	1.05	1.07	Zhang CL and Zou HB.	2013	Lithos	172-173	47-60
3072	436	CAOB	Transbaikalia and Mongolia	113.7490	55.0348	Syenite	Verkhnii Uligli massif	245	245	8.99	60.29	0.0901	0.512379		-0.54	-5.05	-1.74	-1.72	-1.72	1.16	0.95	1.16			Doroshkevich AG et al.	2018	Russian Geology and Geophysics	59（9）	1061–1077
3073	435	CAOB	Transbaikalia and Mongolia	113.7490	55.0348	Syenite	Verkhnii Uligli massif	245	245	11.93	82.38	0.0876	0.512363		-0.55	-5.36	-1.97	-1.95	-1.95	1.18	0.95	1.18			Doroshkevich AG et al.	2018	Russian Geology and Geophysics	59（9）	1061–1077
3074	PU-38/16	CAOB	Transbaikalia and Mongolia	113.5786	55.1344	Syenite	Pravyi Uligli massif	249	249	4.12	25.45	0.0979	0.512739		-0.50	1.97	5.09	5.11	5.11	0.60	0.54	0.60			Doroshkevich AG et al.	2018	Russian Geology and Geophysics	59（9）	1061–1077
3075	506	CAOB	Transbaikalia and Mongolia	112.0968	53.8310	Syenite	Amalat massif	262	262	2.66	14.68	0.1095	0.512573		-0.44	-1.27	1.62	1.65	1.65	0.90	0.84	0.90			Doroshkevich AG et al.	2018	Russian Geology and Geophysics	59（9）	1061–1077
3076	419/3	CAOB	Transbaikalia and Mongolia	113.5786	55.1344	Syenite	Pravyi Uligli massif	249	249	12.20	62.09	0.1188	0.512784		-0.40	2.85	5.30	5.32	5.32	0.59	0.59	0.59			Doroshkevich AG et al.	2018	Russian Geology and Geophysics	59（9）	1061–1077
3077	529	CAOB	Transbaikalia and Mongolia	112.1044	53.8445	Syenite	Sirikta massif	257	257	2.90	12.63	0.1390	0.512833		-0.29	3.80	5.67	5.70	5.70	0.56	0.65	0.56			Doroshkevich AG et al.	2018	Russian Geology and Geophysics	59（9）	1061–1077
3078	389	CAOB	Transbaikalia and Mongolia	113.5113	55.1256	Syenite	Tsipa massif	242	242	3.68	22.07	0.1009	0.512781		-0.49	2.79	5.73	5.75	5.75	0.55	0.50	0.55			Doroshkevich AG et al.	2018	Russian Geology and Geophysics	59（9）	1061–1077
3079	DQ08-70-3	NCC	NCC	111.8831	40.9922	Granodiorite	Daqingshan	114	150	2.03	10.60	0.1154	0.511937	7	-0.41	-13.67	-12.5	-12.1	-12.1	1.92	1.88	1.92	1.88	1.93	Guo L et al.	2012	International Geology Review	54(16)	1885-1905
3080	DQ08-70-1	NCC	NCC	111.8831	40.9922	Granodiorite	Daqingshan	114	150	1.77	8.30	0.1292	0.511925	10	-0.34	-13.91	-12.9	-12.6	-12.6	1.96	2.20	1.96	2.21	1.96	Guo L et al.	2012	International Geology Review	54(16)	1885-1905
3081	DQ08-68-7	NCC	NCC	111.8903	40.9733	Granodiorite	Daqingshan	132	150	4.22	19.60	0.1303	0.511839	7	-0.34	-15.59	-14.5	-14.3	-14.3	2.10	2.38	2.10	2.39	2.10	Guo L et al.	2012	International Geology Review	54(16)	1885-1905
3082	DQ08-68-2	NCC	NCC	111.8903	40.9733	Granodiorite	Daqingshan	132	150	3.04	15.90	0.1156	0.511777	5	-0.41	-16.80	-15.4	-15.2	-15.2	2.18	2.13	2.18	2.13	2.18	Guo L et al.	2012	International Geology Review	54(16)	1885-1905
3083	DQ08-38-1	NCC	NCC	111.8461	41.0042	Granodiorite	Daqingshan	142	150	3.10	15.59	0.1201	0.511948	11	-0.39	-13.46	-12.1	-12.0	-12.0	1.91	1.95	1.91	1.96	1.92	Guo L et al.	2012	International Geology Review	54(16)	1885-1905
3084	DQ08-110	NCC	NCC	112.0161	41.0556	Granodiorite	Daqingshan	148	150	2.96	21.42	0.0835	0.511820	10	-0.58	-15.96	-13.8	-13.8	-13.8	2.06	1.55	2.06	1.56	2.06	Guo L et al.	2012	International Geology Review	54(16)	1885-1905
3085	DQ08-109	NCC	NCC	112.0161	41.0556	Granodiorite	Daqingshan	148	150	2.40	15.96	0.0907	0.511877	11	-0.54	-14.84	-12.9	-12.8	-12.8	1.98	1.57	1.98	1.58	1.98	Guo L et al.	2012	International Geology Review	54(16)	1885-1905
3086	DQ08-105	NCC	NCC	112.0419	41.0272	Granodiorite	Daqingshan	140	150	536.00	40.70	0.0797	0.511939	6	-0.59	-13.64	-11.6	-11.4	-11.4	1.87	1.38	1.87	1.38	1.87	Guo L et al.	2012	International Geology Review	54(16)	1885-1905
3087	DQ08-100	NCC	NCC	112.0419	41.0272	Granodiorite	Daqingshan	140	150	7.07	51.60	0.0829	0.511953	5	-0.58	-13.36	-11.3	-11.2	-11.2	1.85	1.39	1.85	1.40	1.85	Guo L et al.	2012	International Geology Review	54(16)	1885-1905
3088	X-620	CAOB	East Tianshan	95.3972	43.0261	Granite	Xiaopudong	298	298	3.50	20.16	0.1049	0.512688	12	-0.47	0.98	4.47	4.47	4.47	0.70	0.65	0.70		0.65	Wang CS et al.	2009	Acta Petrologica Sinica	25(06)	1499-1511
3089	X-604	CAOB	East Tianshan	94.1097	42.9750	Granite	Badashi	298	298	5.63	24.68	0.1378	0.512680	12	-0.30	0.82	3.06	3.06	3.06	0.81	0.94	0.81		0.94	Wang CS et al.	2009	Acta Petrologica Sinica	25(06)	1499-1511
3090	PM4TC-07	CAOB	NE China	124.9347	51.8339	Gneissic granite	Bowuleshan	915	600				0.512657		-1.00	0.37	3.52		3.52	1.28				1.28	Yang H B et al.	2017	Geological bulletin of China	36(Z1)	342-356
3091	14GW173	CAOB	NE China	133.3024	48.0824	Rhyolite		188	188	3.02	15.90	0.1157	0.512400		-0.41	-4.64	-2.70	-2.70	-2.70	1.19	1.17	1.19			Wang ZH et al.	2015	Journal of Asian Earth Sciences	113(PT.1)	173-193
3092	14GW171	CAOB	NE China	133.3024	48.0824	Rhyolite		188	188	3.76	17.90	0.1279	0.512400		-0.35	-4.64	-3.00	-3.00	-3.00	1.21	1.33	1.21			Wang ZH et al.	2015	Journal of Asian Earth Sciences	113(PT.1)	173-193
3093	14GW161	CAOB	NE China	133.1575	46.5501	Rhyolite		188	188	4.84	24.80	0.1189	0.512400		-0.40	-4.64	-2.78	-2.78	-2.78	1.20	1.20	1.20			Wang ZH et al.	2015	Journal of Asian Earth Sciences	113(PT.1)	173-193
3094	14GW144	CAOB	NE China	133.2317	46.3950	Rhyolite		188	188	6.92	35.60	0.1184	0.512400		-0.40	-4.64	-2.77	-2.77	-2.77	1.19	1.20	1.19			Wang ZH et al.	2015	Journal of Asian Earth Sciences	113(PT.1)	173-193
3095	14GW137	CAOB	NE China	133.1578	46.2628	Dacite		188	188	4.64	24.50	0.1154	0.512400		-0.41	-4.64	-2.70	-2.70	-2.70	1.19	1.16	1.19			Wang ZH et al.	2015	Journal of Asian Earth Sciences	113(PT.1)	173-193
3096	13GW177	CAOB	NE China	133.3024	48.0824	Andesite		188	188	5.06	23.40	0.1317	0.512500		-0.33	-2.69	-1.14	-1.14	-1.14	1.06	1.21	1.06			Wang ZH et al.	2015	Journal of Asian Earth Sciences	113(PT.1)	173-193
3097	09DDS45-1	CAOB	Beishan	96.0000	41.0000	Rhyolite	Dundunshan	371	371	9.74	46.30	0.1274	0.512329		-0.35	-6.03	-2.80	-2.75	-2.75	1.34	1.45	1.34	1.45		Guo QQ et al.	2018	Lithos	278-281	84-96
3098	09DDS41-1	CAOB	Beishan	96.0000	41.0000	Rhyolite	Dundunshan	371	371	9.21	43.60	0.1278	0.512285		-0.35	-6.89	-3.60	-3.62	-3.62	1.41	1.53	1.41	1.53		Guo QQ et al.	2018	Lithos	278-281	84-96
3099	09DDS03-2	CAOB	Beishan	96.0000	41.0000	Andesite	Dundunshan	371	371	5.36	23.70	0.1370	0.512331		-0.30	-5.99	-3.20	-3.16	-3.16	1.38	1.62	1.38	1.62		Guo QQ et al.	2018	Lithos	278-281	84-96
3100	09DDS49-3	CAOB	Beishan	96.0000	41.0000	Rhyolite	Dundunshan	371	371	8.97	42.50	0.1278	0.512344		-0.35	-5.74	-2.50	-2.47	-2.47	1.32	1.43	1.32	1.43		Guo QQ et al.	2017	Lithos	278-281	84-96
3101	09DDS03	CAOB	Beishan	96.0000	41.0000	Rhyolite	Dundunshan	371	371	7.49	34.20	0.1325	0.512325		-0.33	-6.11	-3.10	-3.07	-3.07	1.37	1.55	1.37	1.55		Guo QQ et al.	2017	Lithos	278-281	84-96
3102	ASL12-35	CAOB	Altai	87.0000	48.0000	Dacite		389	389	1.41	5.01	0.1714	0.512770		-0.13	2.57	3.84	3.83	3.83	0.82	1.37	0.82			Wu YF et al.	2015	Journal of Asian Earth Sciences	112(15)	60-73
3103	ASL12-34	CAOB	Altai	87.0000	48.0000	Dacite		389	389	1.22	4.24	0.1753	0.512791		-0.11	2.98	4.05	4.05	4.05	0.80	1.42	0.80			Wu YF et al.	2015	Journal of Asian Earth Sciences	112(15)	60-73
3104	ASL12-33	CAOB	Altai	87.0000	48.0000	Dacite		389	389	1.33	4.53	0.1788	0.512870		-0.09	4.53	5.42	5.41	5.41	0.69	1.22	0.69			Wu YF et al.	2015	Journal of Asian Earth Sciences	112(15)	60-73
3105	ASL12-23	CAOB	Altai	87.0000	48.0000	Dacite		389	389	1.35	4.54	0.1811	0.512780		-0.08	2.77	3.55	3.54	3.54	0.84	1.73	0.84			Wu YF et al.	2015	Journal of Asian Earth Sciences	112(15)	60-73
3106	ASL12-22	CAOB	Altai	87.0000	48.0000	Dacite		389	389	1.35	4.66	0.1764	0.512968		-0.10	6.44	7.45	7.44	7.44	0.53	0.75	0.53			Wu YF et al.	2015	Journal of Asian Earth Sciences	112(15)	60-73
3107	06XJ-79-1	CAOB	Western Tianshan	82.5833	43.6167	Granitic intrusive	Zhongyangchang	317	317	2.70	10.10	0.1470	0.512681	0	-0.25	0.84	2.80	2.85	2.85	0.84	1.07	0.84	1.07	0.84	Tang GJ et al.	2014	Lithos	202-203	85-99
3108	FW04-117	NCC	NCC	117.5500	40.6750	Syenite		124	150	6.04	34.30	0.1073	0.512221		-0.45	-8.13	-6.73	-6.42	-6.42	1.46	1.33	1.46			Yang JH et al.	2008	Journal of Petrolog	49(2)	315-351
3109	FW04-116	NCC	NCC	117.5500	40.6750	Syenite		124	150	6.44	35.30	0.1111	0.512250		-0.44	-7.57	-6.22	-5.93	-5.93	1.42	1.34	1.42			Yang JH et al.	2008	Journal of Petrolog	49(2)	315-351
3110	FW04-115	NCC	NCC	117.5500	40.6750	Syenite		124	150	7.76	38.10	0.1241	0.512240		-0.37	-7.76	-6.62	-6.37	-6.37	1.46	1.54	1.46			Yang JH et al.	2008	Journal of Petrolog	49(2)	315-351
3111	FW04-114	NCC	NCC	117.5500	40.6750	Syenite		124	150	8.21	39.40	0.1269	0.512233		-0.35	-7.90	-6.80	-6.56	-6.56	1.47	1.61	1.47			Yang JH et al.	2008	Journal of Petrolog	49(2)	315-351
3112	FW04-113	NCC	NCC	117.5500	40.6750	Syenite		124	150	7.36	44.60	0.1005	0.512216		-0.49	-8.23	-6.72	-6.39	-6.39	1.46	1.26	1.46			Yang JH et al.	2008	Journal of Petrolog	49(2)	315-351
3113	FW04-109	NCC	NCC	117.5500	40.6750	Syenite		124	150	9.39	51.30	0.1115	0.512240		-0.43	-7.76	-6.42	-6.13	-6.13	1.44	1.36	1.44			Yang JH et al.	2008	Journal of Petrolog	49(2)	315-351
3114	F04-117	NCC	NCC	117.5236	40.5950	Syenite	Wulingshan	142	150	5.98	34.30	0.1053	0.512221	13	-0.46	-8.13	-6.48	-6.38	-6.38	1.46	1.30	1.46	1.31	1.46	Yang JH et al.	2008	Journal of Petrology	49(2)	315-351
3115	F04-116	NCC	NCC	117.5236	40.5950	Syenite	Wulingshan	142	150	6.32	35.30	0.1083	0.512250	12	-0.45	-7.57	-5.98	-5.88	-5.88	1.42	1.30	1.42	1.30	1.42	Yang JH et al.	2008	Journal of Petrology	49(2)	315-351
3116	F04-115	NCC	NCC	117.5236	40.5950	Porphyritic syenite	Wulingshan	129	150	7.70	38.10	0.1222	0.512240	12	-0.38	-7.76	-6.54	-6.34	-6.34	1.45	1.51	1.45	1.52	1.46	Yang JH et al.	2008	Journal of Petrology	49(2)	315-351
3117	F04-114	NCC	NCC	117.5236	40.5950	Porphyritic syenite	Wulingshan	130	150	8.09	39.40	0.1243	0.512233	13	-0.37	-7.90	-6.71	-6.51	-6.51	1.47	1.56	1.47	1.56	1.47	Yang JH et al.	2008	Journal of Petrology	49(2)	315-351
3118	F04-113	NCC	NCC	117.5236	40.5950	Syenite	Wulingshan	142	150	7.51	44.60	0.1018	0.512216	12	-0.48	-8.23	-6.52	-6.42	-6.42	1.46	1.27	1.46	1.27	1.46	Yang JH et al.	2008	Journal of Petrology	49(2)	315-351
3119	F04-112	NCC	NCC	117.5236	40.5950	Porphyritic syenite	Wulingshan	129	150	6.43	38.20	0.1018	0.512221	15	-0.48	-8.13	-6.58	-6.32	-6.32	1.45	1.26	1.45	1.27	1.46	Yang JH et al.	2008	Journal of Petrology	49(2)	315-351
3120	F04-111	NCC	NCC	117.5236	40.5950	Porphyritic syenite	Wulingshan	129	150	6.39	38.00	0.1017	0.512203	8	-0.48	-8.49	-6.93	-6.67	-6.67	1.48	1.29	1.48	1.29	1.49	Yang JH et al.	2008	Journal of Petrology	49(2)	315-351
3121	F04-110	NCC	NCC	117.5236	40.5950	Porphyritic syenite	Wulingshan	129	150	15.30	82.10	0.1123	0.512182	11	-0.43	-8.90	-7.51	-7.28	-7.28	1.53	1.45	1.53	1.46	1.53	Yang JH et al.	2008	Journal of Petrology	49(2)	315-351
3122	F04-109	NCC	NCC	117.5292	40.7747	Syenite	Wulingshan	142	150	9.47	51.30	0.1115	0.512240	13	-0.43	-7.76	-6.23	-6.13	-6.13	1.44	1.36	1.44	1.36	1.44	Yang JH et al.	2008	Journal of Petrology	49(2)	315-351
3123	F04-108	NCC	NCC	117.5292	40.7747	Alkali Hb granite	Qiancengbei	129	150	15.40	97.20	0.0959	0.512093	12	-0.51	-10.63	-8.98	-8.70	-8.70	1.65	1.37	1.65	1.37	1.65	Yang JH et al.	2008	Journal of Petrology	49(2)	315-351
3124	F04-107	NCC	NCC	117.5292	40.7747	Alkali Hb granite	Qiancengbei	129	150	12.00	81.80	0.0889	0.512069	13	-0.55	-11.10	-9.33	-9.04	-9.04	1.67	1.32	1.67	1.32	1.68	Yang JH et al.	2008	Journal of Petrology	49(2)	315-351
3125	F04-106	NCC	NCC	117.5292	40.7747	Alkali Hb granite	Qiancengbei	129	150	13.80	90.20	0.0923	0.512082	12	-0.53	-10.85	-9.13	-8.85	-8.85	1.66	1.34	1.66	1.34	1.67	Yang JH et al.	2008	Journal of Petrology	49(2)	315-351
3126	F04-078	NCC	NCC	119.7044	40.0800	Alkali Hb granite	Houshihushan	120	150	14.50	69.30	0.1267	0.511980	13	-0.36	-12.84	-11.8	-11.5	-11.5	1.87	2.04	1.87	2.05	1.87	Yang JH et al.	2008	Journal of Petrology	49(2)	315-351
3127	F04-077	NCC	NCC	119.7044	40.0800	Alkali Hb granite	Houshihushan	120	150	16.00	75.30	0.1287	0.511970	12	-0.35	-13.03	-12.0	-11.7	-11.7	1.89	2.11	1.89	2.11	1.89	Yang JH et al.	2008	Journal of Petrology	49(2)	315-351
3128	F04-076	NCC	NCC	119.7044	40.0800	Alkali Hb granite	Houshihushan	120	150	13.50	62.80	0.1296	0.511982	12	-0.34	-12.80	-11.8	-11.5	-11.5	1.87	2.11	1.87	2.11	1.87	Yang JH et al.	2008	Journal of Petrology	49(2)	315-351
3129	F04-075	NCC	NCC	119.7044	40.0800	Alkali Hb granite	Houshihushan	120	150	11.30	57.50	0.1188	0.511962	13	-0.40	-13.19	-12.0	-11.7	-11.7	1.89	1.90	1.89	1.91	1.89	Yang JH et al.	2008	Journal of Petrology	49(2)	315-351
3130	F04-074	NCC	NCC	119.7044	40.0800	Alkali Hb granite	Houshihushan	120	150	12.50	64.80	0.1162	0.511954	13	-0.41	-13.34	-12.1	-11.8	-11.8	1.90	1.86	1.90	1.87	1.90	Yang JH et al.	2008	Journal of Petrology	49(2)	315-351
3131	F04-073	NCC	NCC	119.7044	40.0800	Alkali Hb granite	Houshihushan	120	150	12.70	65.00	0.1179	0.511962	13	-0.40	-13.19	-12.0	-11.7	-11.7	1.89	1.88	1.89	1.89	1.89	Yang JH et al.	2008	Journal of Petrology	49(2)	315-351
3132	F04-072	NCC	NCC	119.7044	40.0800	Syenite	Houshihushan	120	150	7.11	37.20	0.1155	0.511881	12	-0.41	-14.77	-13.5	-13.2	-13.2	2.01	1.96	2.01	1.97	2.02	Yang JH et al.	2008	Journal of Petrology	49(2)	315-351
3133	F04-071	NCC	NCC	119.7044	40.0800	Syenite	Houshihushan	120	150	6.62	36.10	0.1108	0.511846	13	-0.44	-15.45	-14.1	-13.8	-13.8	2.06	1.93	2.06	1.93	2.06	Yang JH et al.	2008	Journal of Petrology	49(2)	315-351
3134	F04-070	NCC	NCC	119.7044	40.0800	Syenite	Houshihushan	120	150	6.74	36.80	0.1108	0.511857	14	-0.44	-15.23	-13.9	-13.6	-13.6	2.04	1.91	2.04	1.91	2.05	Yang JH et al.	2008	Journal of Petrology	49(2)	315-351
3135	F04-069	NCC	NCC	119.7044	40.0800	Syenite	Houshihushan	120	150	7.04	38.20	0.1113	0.511839	11	-0.43	-15.59	-14.3	-14.0	-14.0	2.07	1.95	2.07	1.95	2.08	Yang JH et al.	2008	Journal of Petrology	49(2)	315-351
3136	F04-068	NCC	NCC	119.7044	40.0800	Syenite	Houshihushan	120	150	6.49	35.50	0.1107	0.511847	12	-0.44	-15.43	-14.1	-13.8	-13.8	2.06	1.92	2.06	1.93	2.06	Yang JH et al.	2008	Journal of Petrology	49(2)	315-351
3137	F04-067	NCC	NCC	119.7044	40.0800	Syenite	Houshihushan	120	150	6.51	35.60	0.1105	0.511841	13	-0.44	-15.55	-14.2	-13.9	-13.9	2.07	1.93	2.07	1.93	2.07	Yang JH et al.	2008	Journal of Petrology	49(2)	315-351
3138	F04-066	NCC	NCC	119.7044	40.0800	Alkali rhyolite	Houshihushan	120	150	7.12	40.50	0.1062	0.511900	13	-0.46	-14.40	-13.0	-12.7	-12.7	1.97	1.77	1.97	1.77	1.97	Yang JH et al.	2008	Journal of Petrology	49(2)	315-351

3139	F04-065	NCC	NCC	119.7044	40.0800	Alkali rhyolite	Houshihushan	120	150	9.11	54.50	0.1010	0.511828	13	-0.49	-15.80	-14.3	-14.0	-14.0	2.07	1.78	2.07	1.79	2.08	Yang JH et al.	2008	Journal of Petrology	49(2)	315-351
3140	F04-064	NCC	NCC	119.7044	40.0800	Alkali rhyolite	Houshihushan	120	150	6.85	39.20	0.1056	0.511828	12	-0.46	-15.80	-14.4	-14.1	-14.1	2.08	1.86	2.08	1.86	2.09	Yang JH et al.	2008	Journal of Petrology	49(2)	315-351
3141	F04-063	NCC	NCC	119.7044	40.0800	Alkali rhyolite	Houshihushan	120	150	10.90	63.40	0.1043	0.511868	13	-0.47	-15.02	-13.6	-13.3	-13.3	2.02	1.78	2.02	1.78	2.02	Yang JH et al.	2008	Journal of Petrology	49(2)	315-351
3142	F04-062	NCC	NCC	119.7044	40.0800	Alkali rhyolite	Houshihushan	120	150	9.28	55.70	0.1007	0.511878	11	-0.49	-14.83	-13.4	-13.0	-13.0	1.99	1.71	1.99	1.71	2.00	Yang JH et al.	2008	Journal of Petrology	49(2)	315-351
3143	F04-061	NCC	NCC	119.7044	40.0800	Alkali Hb granite	Houshihushan	120	150	11.30	59.70	0.1145	0.512144	14	-0.42	-9.64	-8.38	-8.06	-8.06	1.59	1.54	1.59	1.55	1.60	Yang JH et al.	2008	Journal of Petrology	49(2)	315-351
3144	F04-060	NCC	NCC	119.7044	40.0800	Alkali Hb granite	Houshihushan	120	150	15.30	84.60	0.1092	0.511933	13	-0.44	-13.75	-12.4	-12.1	-12.1	1.92	1.77	1.92	1.77	1.93	Yang JH et al.	2008	Journal of Petrology	49(2)	315-351
3145	F04-059	NCC	NCC	119.7044	40.0800	Alkali Hb granite	Houshihushan	120	150	6.94	36.30	0.1155	0.511933	11	-0.41	-13.75	-12.5	-12.2	-12.2	1.93	1.88	1.93	1.89	1.93	Yang JH et al.	2008	Journal of Petrology	49(2)	315-351
3146	F04-057	NCC	NCC	119.7044	40.0800	Alkali rhyolite	Houshihushan	120	150	12.00	65.00	0.1117	0.512111	14	-0.43	-10.28	-8.99	-8.65	-8.65	1.64	1.55	1.64	1.55	1.65	Yang JH et al.	2008	Journal of Petrology	49(2)	315-351
3147	F04-039	NCC	NCC	119.4394	40.0964	Alkali Hb granite	Xiangshan	117	150	13.40	57.90	0.1400	0.512035	11	-0.29	-11.76	-10.9	-10.7	-10.7	1.80	2.30	1.80	2.30	1.80	Yang JH et al.	2008	Journal of Petrology	49(2)	315-351
3148	F04-038	NCC	NCC	119.4394	40.0964	Alkali Hb granite	Xiangshan	117	150	9.30	39.00	0.1440	0.512019	13	-0.27	-12.07	-11.3	-11.1	-11.1	1.83	2.46	1.83	2.47	1.83	Yang JH et al.	2008	Journal of Petrology	49(2)	315-351
3149	F04-037	NCC	NCC	119.4394	40.0964	Alkali Hb granite	Xiangshan	117	150	6.26	33.50	0.1128	0.512000	15	-0.43	-12.45	-11.2	-10.8	-10.8	1.82	1.73	1.82	1.74	1.82	Yang JH et al.	2008	Journal of Petrology	49(2)	315-351
3150	F04-036	NCC	NCC	119.4394	40.0964	Alkali Hb granite	Xiangshan	117	150	15.70	59.10	0.1610	0.512048	13	-0.18	-11.51	-11.0	-10.8	-10.8	1.81	3.16	1.81	3.17	1.81	Yang JH et al.	2008	Journal of Petrology	49(2)	315-351
3151	F04-035	NCC	NCC	119.4394	40.0964	Alkali-feldspar granite	Xiangshan	117	150	6.30	34.10	0.1115	0.511949	13	-0.43	-13.44	-12.2	-11.8	-11.8	1.90	1.79	1.90	1.79	1.90	Yang JH et al.	2008	Journal of Petrology	49(2)	315-351
3152	F04-034	NCC	NCC	119.4394	40.0964	Alkali-feldspar granite	Xiangshan	117	150	6.25	34.60	0.1092	0.511923	14	-0.44	-13.95	-12.7	-12.3	-12.3	1.94	1.78	1.94	1.79	1.94	Yang JH et al.	2008	Journal of Petrology	49(2)	315-351
3153	F04-033	NCC	NCC	119.4394	40.0964	Alkali-feldspar granite	Xiangshan	117	150	6.68	35.80	0.1129	0.511942	12	-0.43	-13.58	-12.3	-12.0	-12.0	1.91	1.82	1.91	1.83	1.92	Yang JH et al.	2008	Journal of Petrology	49(2)	315-351
3154	F04-032	NCC	NCC	119.4394	40.0964	Alkali-feldspar granite	Xiangshan	117	150	7.04	36.30	0.1173	0.511953	12	-0.40	-13.36	-12.2	-11.8	-11.8	1.90	1.89	1.90	1.89	1.90	Yang JH et al.	2008	Journal of Petrology	49(2)	315-351
3155	XT-30	CAOB	Northern Tianshan	81.7667	45.2167	Adakite	Alataw	320	320	1.80	8.27	0.1320	0.512751	±10	-0.33	2.20	4.80	4.85	4.85	0.68	0.74	0.68	0.74	0.68	Wang Q et al.	2007	Chemical Geology	236(1-2)	42-64
3156	XT-29	CAOB	Northern Tianshan	81.7667	45.2167	Adakite	Alataw	320	320	1.38	7.96	0.1053	0.512753	±13	-0.46	2.24	6.00	5.98	5.98	0.59	0.56	0.59	0.56	0.59	Wang Q et al.	2007	Chemical Geology	236(1-2)	42-64
3157	XT-28	CAOB	Northern Tianshan	81.7667	45.2167	Adakite	Alataw	320	320	2.72	12.43	0.1325	0.512678	±6	-0.33	0.78	3.40	3.40	3.40	0.80	0.89	0.80	0.89	0.80	Wang Q et al.	2007	Chemical Geology	236(1-2)	42-64
3158	XT-27	CAOB	Northern Tianshan	81.7667	45.2167	Adakite	Alataw	320	320	1.71	8.08	0.1287	0.512832	±14	-0.35	3.78	6.60	6.56	6.56	0.54	0.57	0.54	0.57	0.54	Wang Q et al.	2007	Chemical Geology	236(1-2)	42-64
3159	WXT742	CAOB	Northern Tianshan	81.7667	45.2167	Adakite	Alataw	320	320	2.45	11.15	0.1331	0.512774	±14	-0.32	2.65	5.30	5.25	5.25	0.65	0.71	0.65	0.71	0.65	Wang Q et al.	2007	Chemical Geology	236(1-2)	42-64
3160	WXT737	CAOB	Northern Tianshan	81.7667	45.2167	Rhyolite	Alataw	306	306	1.20	3.22	0.2260	0.513027	±20	0.15	7.59	6.40	6.44	6.44	0.55	-1.54	0.55		0.55	Wang Q et al.	2007	Chemical Geology	236(1-2)	42-64
3161	WXT734	CAOB	Northern Tianshan	81.7500	45.2333	Dacite	Alataw	306	306	6.11	25.88	0.1428	0.512639	±10	-0.27	0.02	2.20	2.12	2.12	0.89	1.10	0.89	1.10	0.89	Wang Q et al.	2007	Chemical Geology	236(1-2)	42-64
3162	841	CAOB	West Junggar	83.9167	45.0333	Perilla granite	Miaoergou	320	320	7.54	32.78	0.1390	0.512810	6	-0.29	3.36	5.71	5.71	5.71	0.61	0.69	0.61	0.69	0.61	Zhang LF et al.	2004	XinJiang Geology	(01)	36-42
3163	207	CAOB	West Junggar	83.9167	45.0333	Perilla granite	Miaoergou	320	320	7.93	32.08	0.1495	0.512809	7	-0.24	3.34	5.26	5.26	5.26	0.65	0.81	0.65	0.81	0.65	Zhang LF et al.	2004	XinJiang Geology	(01)	36-42
3164	134	CAOB	West Junggar	83.9167	45.0333	Perilla granite	Miaoergou	320	320	3.18	13.84	0.1391	0.512752	7	-0.29	2.22	4.58	4.58	4.58	0.70	0.81	0.70	0.81	0.70	Zhang LF et al.	2004	XinJiang Geology	(01)	36-42
3165	114	CAOB	West Junggar	83.9167	45.0333	Perilla granite	Miaoergou	320	320	10.12	42.88	0.1428	0.512817	7	-0.27	3.49	5.69	5.69	5.69	0.61	0.72	0.61	0.72	0.61	Zhang LF et al.	2004	XinJiang Geology	(01)	36-42
3166	S-08-10/15a	CAOB	Transbaikalia and Mongolia	108.0000	52.0000	Syenite		290	290	7.63	46.09	0.1000	0.512223		-0.49	-8.10	-3.79	-4.52	-4.52	1.42	1.24	1.42			Burmakina GN et al.	2018	Russian Geology and Geophysics	59（1）	19-40
3167	06XJ-40	CAOB	Northern margin of Yili block	81.6333	44.2167	Granite		349	349	2.74	17.30	0.0960	0.512447		-0.51	-3.73	0.76	0.76	0.76	1.04	0.91	1.04	0.91	1.04	Tang GJ et al.	2013	Journal of Asian Earth Sciences	74(S1)	113-128
3168	06XJ-35	CAOB	Northern margin of Yili block	81.6333	44.2167	Granite		349	349	3.63	19.20	0.1150	0.512437		-0.42	-3.92	-0.28	-0.28	-0.28	1.12	1.10	1.12	1.10	1.13	Tang GJ et al.	2013	Journal of Asian Earth Sciences	74(S1)	113-128
3169	06XJ-34	CAOB	Northern margin of Yili block	81.6333	44.2167	Granite		349	349	3.55	19.90	0.1090	0.512588		-0.45	-0.98	2.94	2.93	2.93	0.86	0.82	0.86	0.82	0.87	Tang GJ et al.	2013	Journal of Asian Earth Sciences	74(S1)	113-128
3170	06XJ-32	CAOB	Northern margin of Yili block	81.6333	44.2167	Andesite		349	349			0.1330	0.512699		-0.32	1.19	4.03	4.03	4.03	0.77	0.85	0.77	0.85	0.78	Tang GJ et al.	2013	Journal of Asian Earth Sciences	74(S1)	113-128
3171	06XJ-31	CAOB	Northern margin of Yili block	81.6333	44.2167	Andesite		349	349			0.1290	0.512576		-0.34	-1.21	1.81	1.81	1.81	0.95	1.03	0.95	1.03	0.96	Tang GJ et al.	2013	Journal of Asian Earth Sciences	74(S1)	113-128
3172	06XJ-30	CAOB	Northern margin of Yili block	81.6333	44.2167	Andesite		349	349			0.1160	0.512477		-0.41	-3.14	0.46	0.45	0.45	1.06	1.05	1.06	1.05	1.07	Tang GJ et al.	2013	Journal of Asian Earth Sciences	74(S1)	113-128
3173	KL-2	CAOB	West Junggar	84.8167	45.6167	Granite	Karamay	315	315	2.76	13.65	0.1225	0.512850	4	-0.38	4.14	7.12	7.12	7.12	0.49	0.50	0.49	0.50	0.49	He JB and Chen B.	2011	Earth Science Frontiers	18(2)	191-211
3174	KL-19	CAOB	West Junggar	84.8167	45.6167	Diorite	Karamay	315	315	3.40	14.54	0.1416	0.512880	5	-0.28	4.72	6.94	6.94	6.94	0.51	0.57	0.51	0.57	0.51	He JB and Chen B.	2011	Earth Science Frontiers	18(2)	191-211
3175	KL-18	CAOB	West Junggar	84.8167	45.6167	Granite	Karamay	315	315	2.50	11.62	0.1304	0.512872	3	-0.34	4.56	7.23	7.23	7.23	0.48	0.51	0.48	0.51	0.48	He JB and Chen B.	2011	Earth Science Frontiers	18(2)	191-211
3176	KL-17	CAOB	West Junggar	84.8167	45.6167	Diorite	Karamay	315	315	3.10	14.43	0.1298	0.512868	4	-0.34	4.49	7.18	7.18	7.18	0.49	0.51	0.49	0.51	0.49	He JB and Chen B.	2011	Earth Science Frontiers	18(2)	191-211
3177	KL-13	CAOB	West Junggar	84.8167	45.6167	Gabbro diorite	Karamay	315	315	4.08	18.99	0.1301	0.512858	4	-0.34	4.29	6.97	6.97	6.97	0.51	0.53	0.51	0.53	0.51	He JB and Chen B.	2011	Earth Science Frontiers	18(2)	191-211
3178	KL-12	CAOB	West Junggar	84.8167	45.6167	Granite	Karamay	315	315	3.18	14.72	0.1306	0.512881	5	-0.34	4.74	7.40	7.40	7.40	0.47	0.49	0.47	0.49	0.47	He JB and Chen B.	2011	Earth Science Frontiers	18(2)	191-211
3179	KL-11	CAOB	West Junggar	84.8167	45.6167	Granodiorite	Karamay	315	315	3.44	15.99	0.1304	0.512860	4	-0.34	4.33	7.00	7.00	7.00	0.50	0.53	0.50	0.53	0.50	He JB and Chen B.	2011	Earth Science Frontiers	18(2)	191-211
3180	DH055	CAOB	Jillin	126.7800	43.3400	Andesite		329	329	4.88	24.60	0.1208	0.512580		-0.39	-1.13	2.06	2.05	2.05	0.92	0.94	0.92			Yu Q et al.	2014	Lithos	192	116-131
3181	DH023	CAOB	Jillin	126.7800	43.3400	Andesite		329	329	3.89	18.00	0.1316	0.512599		-0.33	-0.76	1.98	1.97	1.97	0.92	1.02	0.92			Yu Q et al.	2014	Lithos	192	116-131
3182	DH013	CAOB	Jillin	126.7800	43.3400	Dacite		329	329	2.99	15.80	0.1153	0.512629		-0.41	-0.18	3.25	3.24	3.24	0.82	0.81	0.82			Yu Q et al.	2014	Lithos	192	116-131
3183	DH006	CAOB	Jillin	126.7254	43.3723	Dacite		329	329	5.00	23.20	0.1313	0.512569		-0.33	-1.35	1.40	1.40	1.40	0.97	1.07	0.97			Yu Q et al.	2014	Lithos	192	116-131
3184	K17-35	CAOB	Junggar	88.5000	47.0000	Dacite		316	316	6.52	32.40	0.1226	0.512841		-0.38	3.96	6.96	6.95	6.95	0.51	0.52	0.51			Li D et al.	2014	Lithos	184–187	167–193
3185	K17-34	CAOB	Junggar	88.5000	47.0000	Dacite		316	316	5.27	25.70	0.1249	0.512852		-0.37	4.17	7.08	7.07	7.07	0.50	0.51	0.50			Li D et al.	2014	Lithos	184–187	167–193
3186	YSH01-5	NCC	NMNCC	119.1094	42.5764	Granodiorite	Yongshenghao	229	229	3.45	17.87	0.1169	0.512466	5	-0.41	-3.36	-1.02	-1.02	-1.02	1.09	1.08	1.09	1.08	1.09	Liu JF et al.	2012	Journal of Asian Earth Sciences	60	147-159
3187	YSH01-3	NCC	NMNCC	119.1094	42.5764	Granodiorite	Yongshenghao	229	229	4.86	23.24	0.1264	0.512473	6	-0.36	-3.22	-1.16	-1.16	-1.16	1.10	1.18	1.10	1.18	1.10	Liu JF et al.	2012	Journal of Asian Earth Sciences	60	147-159
3188	YSH01-1	NCC	NMNCC	119.1094	42.5764	Granodiorite	Yongshenghao	229	229	4.63	23.33	0.1200	0.512486	10	-0.39	-2.97	-0.73	-0.72	-0.72	1.06	1.08	1.06	1.08	1.07	Liu JF et al.	2012	Journal of Asian Earth Sciences	60	147-159
3189	06JH64	NCC	NCC	117.1719	41.6306	Qtz syenite	Liangjia	246	246			0.1010	0.511924	2	-0.49	-13.93	-10.9	-10.9	-10.9	1.91	1.65	1.91	1.66	1.91	Yang JH et al.	2012	Chemical Geology	328	149-167
3190	06JH63	NCC	NCC	117.1719	41.6306	Qtz syenite	Liangjia	246	246			0.1010	0.511912	2	-0.49	-14.16	-11.2	-11.2	-11.2	1.92	1.67	1.92	1.67	1.93	Yang JH et al.	2012	Chemical Geology	328	149-167
3191	06JH62	NCC	NCC	117.1719	41.6306	Qtz syenite	Liangjia	246	246			0.0971	0.511910	3	-0.51	-14.20	-11.1	-11.1	-11.1	1.92	1.62	1.92	1.62	1.92	Yang JH et al.	2012	Chemical Geology	328	149-167
3192	06JH128	NCC	NCC	118.1744	41.3042	Qtz syenite	Jianfang	254	254			0.0886	0.511915	4	-0.55	-14.10	-10.6	-10.6	-10.6	1.89	1.50	1.89	1.51	1.89	Yang JH et al.	2012	Chemical Geology	328	149-167
3193	06JH127	NCC	NCC	118.1744	41.3042	Qtz syenite	Jianfang	254	254			0.0856	0.511922	5	-0.56	-13.97	-10.4	-10.4	-10.4	1.87	1.46	1.87	1.46	1.87	Yang JH et al.	2012	Chemical Geology	328	149-167
3194	06JH126	NCC	NCC	118.1744	41.3042	Qtz syenite	Jianfang	254	254			0.0866	0.511915	4	-0.56	-14.10	-10.5	-10.5	-10.5	1.88	1.48	1.88	1.48	1.88	Yang JH et al.	2012	Chemical Geology	328	149-167
3195	06JH125	NCC	NCC	118.1744	41.3042	Qtz syenite	Jianfang	254	254			0.1445	0.512093	3	-0.27	-10.63	-8.94	-8.94	-8.94	1.75	2.32	1.75	2.32	1.75	Yang JH et al.	2012	Chemical Geology	328	149-167
3196	06JH124	NCC	NCC	118.1744	41.3042	Qtz syenite	Jianfang	254	254			0.1446	0.512167	3	-0.26	-9.19	-7.50	-7.50	-7.50	1.63	2.16	1.63	2.17	1.64	Yang JH et al.	2012	Chemical Geology	328	149-167
3197	05FW59	NCC	NCC	119.2400	40.8919	Syenite	Hekanzi	225	225			0.1015	0.512202	11	-0.48	-8.51	-5.76	-5.77	-5.77	1.47	1.29	1.47	1.29	1.47	Yang JH et al.	2012	Chemical Geology	328	149-167
3198	05FW58	NCC	NCC	119.2400	40.8919	Syenite	Hekanzi	225	225			0.0977	0.512223	10	-0.50	-8.10	-5.26	-5.25	-5.25	1.43	1.22	1.43	1.22	1.43	Yang JH et al.	2012	Chemical Geology	328	149-167
3199	05FW57	NCC	NCC	119.2400	40.8919	Syenite	Hekanzi	225	225			0.1095	0.512384	14	-0.44	-4.95	-2.45	-2.45	-2.45	1.20	1.12	1.20	1.12	1.20	Yang JH et al.	2012	Chemical Geology	328	149-167
3200	05FW56	NCC	NCC	119.2400	40.8919	Syenite	Hekanzi	225	225			0.0975	0.512160	14	-0.50	-9.32	-6.49	-6.48	-6.48	1.53	1.30	1.53	1.30	1.53	Yang JH et al.	2012	Chemical Geology	328	149-167
3201	05FW55	NCC	NCC	119.2400	40.8919	Syenite	Hekanzi	225	225			0.0987	0.512194	13	-0.50	-8.66	-5.84	-5.85	-5.85	1.48	1.27	1.48	1.27	1.48	Yang JH et al.	2012	Chemical Geology	328	149-167
3202	05FW51	NCC	NCC	119.2400	40.8919	Syenite	Hekanzi	225	225			0.0966	0.512175	29	-0.51	-9.03	-6.15	-6.16	-6.16	1.50	1.27	1.50	1.27	1.50	Yang JH et al.	2012	Chemical Geology	328	149-167
3203	05FW50	NCC	NCC	119.2400	40.8919	Syenite	Hekanzi	225	225			0.0941	0.512191	11	-0.52	-8.72	-5.78	-5.77	-5.77	1.47	1.22	1.47	1.22	1.47	Yang JH et al.	2012	Chemical Geology	328	149-167
3204	05FW48	NCC	NCC	119.2400	40.8919	Syenite	Hekanzi	225	225			0.0951	0.512198	10	-0.52	-8.58	-5.67	-5.67	-5.67	1.46	1.22	1.46	1.23	1.46	Yang JH et al.	2012	Chemical Geology	328	149-167
3205	05FW46	NCC	NCC	119.2400	40.8919	Syenite	Hekanzi	225	225			0.1058	0.512264	10	-0.46	-7.30	-4.69	-4.69	-4.69	1.38	1.25	1.38	1.25	1.38	Yang JH et al.	2012	Chemical Geology	328	149-167
3206	05FW45	NCC	NCC	119.2400	40.8919	Syenite	Hekanzi	224	224			0.1060	0.512205	11	-0.46	-8.45	-5.85	-5.85	-5.85	1.48	1.34	1.48	1.34	1.48	Yang JH et al.	2012	Chemical Geology	328	149-167
3207	05FW44	NCC	NCC	119.2400	40.8919	Syenite	Hekanzi	224	224			0.1038	0.512209	10	-0.47	-8.37	-5.71	-5.71	-5.71	1.46	1.30	1.46	1.31	1.47	Yang JH et al.	2012	Chemical Geology	328	149-167
3208	B626-1	CAOB	N. of MOS	108.4500	50.6100	AFS syenite	Bryansky	280	280.00	9.61	62.43	0.0931	0.512301		-0.53	-6.57	-2.87	-2.87	-2.87	1.28	1.07	1.28			Litvinovsky BA et al.	2002	Chemical Geology	189(1)	105-133
3209	B626	CAOB	N. of MOS	108.3600	50.5800	AFS syenite	Bryansky	280	280.00	16.37	97.7	0.1013	0.512293		-0.49	-6.73	-3.32	-3.32	-3.32	1.32	1.16	1.32			Litvinovsky BA et al.	2002	Chemical Geology	189(1)	105-133
3210	B627	CAOB	Transbaikalia and Mongolia	112.2000	52.3500	PA syenite	Bryansky	280	280	18.74	113.46	0.0999	0.512317		-0.49	-6.26	-2.80		-2.80	1.27	1.12	1.27	1.12	1.27	Litvinovsky BA et al.	2002	Chemical Geology	189（1-2）	105 – 133
3211	B444	CAOB	Transbaikalia and Mongolia	111.3333	52.2000	Comendite	Tsagan-Khurtei	210	210	18.74	100.50	0.1127	0.512676		-0.43	0.74	2.99	2.99	2.99	0.74	0.72	0.74	0.72	0.74	Litvinovsky BA et al.	2002	Chemical Geology	189（1-2）	105 – 133
3212	B440	CAOB	Transbaikalia and Mongolia	111.3333	52.2000	Comendite	Tsagan-Khurtei	210	210	11.43	61.28	0.1128	0.512670		-0.43	0.62	2.87	2.87	2.87	0.75	0.73	0.75	0.73	0.75	Litvinovsky BA et al.	2002	Chemical Geology	189（1-2）	105 – 133
3213	B425	CAOB	Transbaikalia and Mongolia	112.2000	52.3500	AFS granite	Bryansky	280	280	2.09	14.73	0.0858	0.512320		-0.56	-6.20	-2.24	-2.24	-2.24	1.23	0.99	1.23	0.99	1.23	Litvinovsky BA et al.	2002	Chemical Geology	189（1-2）	105 – 133
3214	B388	CAOB	Transbaikalia and Mongolia	112.2000	52.3500	PA granite	Bryansky	280	280	10.92	56.44	0.1170	0.512364		-0.41	-5.34	-2.50	-2.50	-2.50	1.25	1.24	1.25	1.24	1.25	Litvinovsky BA et al.	2002	Chemical Geology	189（1-2）	105 – 133
3215	B382-2	CAOB	Transbaikalia and Mongolia	112.2000	52.3500	comendite	Bryansky	285	285	15.35	76.07	0.1220	0.512342		-0.38	-5.77	-3.06	-3.06	-3.06	1.30	1.34	1.30	1.34	1.30	Litvinovsky BA et al.	2002	Chemical Geology	189（1-2）	105 – 133
3216	B 626-1	CAOB	Transbaikalia and Mongolia	112.2000	52.3500	AFS syenite	Bryansky	280	280	9.61	62.43	0.0931	0.512301		-0.53	-6.57	-2.87	-2.87	-2.87	1.28	1.07	1.28	1.07	1.28	Litvinovsky BA et al.	2002	Chemical Geology	189（1-2）	105 – 133
3217	B 626	CAOB	Transbaikalia and Mongolia	112.2000	52.3500	AFS syenite	Bryansky	280	280	16.37	97.70	0.1013	0.512293		-0.49	-6.73	-3.32	-3.32	-3.32	1.32	1.16	1.32	1.16	1.32	Litvinovsky BA et al.	2002	Chemical Geology	189（1-2）	105 – 133
3218	A447-4	CAOB	Transbaikalia and Mongolia	112.2000	52.3500	Comendite	Bryansky	285	285	21.81	116.14	0.1135	0.512356		-0.42	-5.50	-2.48	-2.48	-2.48	1.25	1.21	1.25	1.21	1.25	Litvinovsky BA et al.	2002	Chemical Geology	189（1-2）	105 – 133
3219	1/8	CAOB	Transbaikalia and Mongolia	112.0333	52.1000	Comendite	Tsagan-Khurtei	210	210	12.10	66.70	0.1097	0.512625		-0.44	-0.25	2.08	2.08	2.08	0.82	0.77	0.82	0.77	0.82	Litvinovsky BA et al.	2002	Chemical Geology	189（1-2）	105 – 133
3220	BSZK15-5-405	CAOB	Eastern Tianshan	96.0000	42.5000	Granite	Baishan	293	293	2.30	10.44	0.1333	0.512888	0	-0.32	4.88	7.20	7.25	7.25	0.46	0.50	0.46	0.50	0.47	Cao MJ et al.	2012	Lithos	146	65-79
3221	BS-17	CAOB	Eastern Tianshan	95.9000	42.4333	Granite	Baishan	293	293	5.72	30.63	0.1130	0.512739	0	-0.43	1.97	5.10	5.10	5.10	0.64	0.62	0.64	0.62	0.64	Cao MJ et al.	2012	Lithos	146	65-79
3222	BS-16	CAOB	Eastern Tianshan	95.9500	42.4333	Granite	Baishan	293	293	2.97	12.74	0.1412	0.512787	0	-0.28	2.91	5.00	4.98	4.98	0.65	0.76	0.65	0.77	0.65	Cao MJ et al.	2012	Lithos	146	65-79
3223	10BSD-9	CAOB	Eastern Tianshan	96.0167	42.5333	Granite	Baishan	293	293	2.58	12.41	0.1257	0.512791	0	-0.36	2.98	5.60	5.64	5.64	0.60	0.62	0.60	0.63	0.60	Cao MJ et al.	2012	Lithos	146	65-79
3224	10BSD-12	CAOB	Eastern Tianshan	95.9500	42.4500	Granite	Baishan	293	293	3.69	19.13	0.1326	0.512789		-0.33	2.95	5.30	5.34	5.34	0.62	0.68	0.62	0.68	0.62	Cao MJ et al.	2012	Lithos	146	65-79
3225	10BSD-11	CAOB	Eastern Tianshan	95.9667	42.4667	Granite	Baishan	293	293	1.76	9.71	0.1100	0.512669	0	-0.44	0.60	3.80	3.85	3.85	0.74	0.71	0.74	0.71	0.75	Cao MJ et al.	2012	Lithos	146	65-79
3226	BSD-02*	CAOB	Eastern Tianshan	96.0000	42.5000	Granite	Baishan	293	293	2.78	10.60	0.1586	0.512842	0	-0.19	3.98	5.40	5.40	5.40	0.61	0.85	0.61	0.86	0.62	Cao MJ et al.	2012	Lithos	146	65-79
3227	BSD-01*	CAOB	Eastern Tianshan	96.0000	42.5000	Granite	Baishan	293	293	2.83	12.00	0.1425	0.512830	0	-0.28	3.75	5.80	5.77	5.77	0.59	0.69	0.59	0.69	0.59	Cao MJ et al.	2012	Lithos	146	65-79
3228	BSSG-3	CAOB	Tarim	76.5333	40.0833	Alkali granite		275	275	11.23	67.02	0.1015	0.512459		-0.48	-3.49	-2.10	-0.15	-0.15	1.05	0.94	1.05	1.08	1.21	Ma Y et al.	2012	Lithos	146	65-79
3229	BSSG-2	CAOB	Tarim	76.5333	40.0833	Alkali granite		275	275	13.85	68.87	0.1209	0.512503		-0.39	-2.63	-1.92	0.03	0.03	1.04	1.06	1.04	1.23	1.20	Ma Y et al.	2012	Lithos	146	65-79
3230	BSSG-1	CAOB	Tarim	76.5333	40.0833	Alkali granite		275	275	13.92	79.94	0.1056	0.512471		-0.46	-3.26	-2.01	-0.06	-0.06	1.05	0.96	1.05	1.10	1.20	Ma Y et al.	2012	Lithos	146	65-79
3231	DZZ-6	NCC	NCC	116.0847	41.1378	A-type granite	Dengzhazi	140	150	12.59	73.20	0.1091	0.511785	7	-0.45	-16.64	-15.1	-15.0	-15.0	2.15	1.98	2.15	1.99	2.16	Niu XL et al.	2011	Journal of Asian Earth Sciences	41(2）	133-146
3232	DZZ-5	NCC	NCC	116.2456	41.0769	A-type granite	Dengzhazi	140	150	12.19	69.92	0.1107	0.511766	15	-0.44	-17.01	-15.5	-15.4	-15.4	2.19	2.04	2.19	2.04	2.19	Niu XL et al.	2011	Journal of Asian Earth Sciences	41(2）	133-146
3233	DZZ-4	NCC	NCC	116.2678	41.1058	A-type granite	Dengzhazi	140	150	12.77	73.49	0.1102	0.511766	8	-0.44	-17.01	-15.5	-15.4	-15.4	2.19	2.03	2.19	2.04	2.19	Niu XL et al.	2011	Journal of Asian Earth Sciences	41(2）	133-146
3234	DZZ-3	NCC	NCC	116.2947	41.1378	A-type granite	Dengzhazi	140	150	12.55	71.80	0.1109	0.511776	7	-0.44	-16.81	-15.3	-15.2	-15.2	2.17	2.03	2.17	2.03	2.17	Niu XL et al.	2011	Journal of Asian Earth Sciences	41(2）	133-146
3235	DZZ-2	NCC	NCC	116.2903	41.0289	A-type granite	Dengzhazi	140	150	11.21	54.01	0.1317	0.511786	6	-0.33	-16.62	-15.5	-15.4	-15.4	2.18	2.52	2.18	2.53	2.19	Niu XL et al.	2011	Journal of Asian Earth Sciences	41(2）	133-146
3236	DZZ-1	NCC	NCC	116.3303	41.0864	A-type granite	Dengzhazi	140	150	11.96	69.12	0.1098	0.511765	7	-0.44	-17.03	-15.5	-15.4	-15.4	2.19	2.02	2.19	2.03	2.19	Niu XL et al.	2011	Journal of Asian Earth Sciences	41(2）	133-146

3237	BC-6	NCC	NCC	116.0581	41.1828	A-type granite	Dengzhazi	140	150	13.09	76.25	0.1089	0.511779	8	-0.45	-16.76	-15.2	-15.1	-15.1	2.16	1.99	2.16	1.99	2.17	Niu XL et al.	2011	Journal of Asian Earth Sciences	41(2）	133-146
3238	BC-5	NCC	NCC	116.0936	41.1697	A-type granite	Dengzhazi	140	150	12.73	74.32	0.1087	0.511772	10	-0.45	-16.89	-15.3	-15.2	-15.2	2.17	1.99	2.17	2.00	2.18	Niu XL et al.	2011	Journal of Asian Earth Sciences	41(2）	133-146
3239	BC-4	NCC	NCC	116.1072	41.1408	A-type granite	Dengzhazi	140	150	13.68	79.71	0.1089	0.511786	6	-0.45	-16.62	-15.1	-14.9	-14.9	2.15	1.98	2.15	1.98	2.16	Niu XL et al.	2011	Journal of Asian Earth Sciences	41(2）	133-146
3240	BC-3	NCC	NCC	116.0714	41.1122	A-type granite	Dengzhazi	140	150	14.40	83.16	0.1098	0.511724	7	-0.44	-17.83	-16.3	-16.2	-16.2	2.25	2.09	2.25	2.09	2.25	Niu XL et al.	2011	Journal of Asian Earth Sciences	41(2）	133-146
3241	BC-2	NCC	NCC	116.1294	41.0931	A-type granite	Dengzhazi	140	150	12.64	73.19	0.1096	0.511757	10	-0.44	-17.19	-15.6	-15.5	-15.5	2.20	2.03	2.20	2.04	2.20	Niu XL et al.	2011	Journal of Asian Earth Sciences	41(2）	133-146
3242	BC-1	NCC	NCC	116.1472	41.1281	A-type granite	Dengzhazi	140	150	4.46	24.30	0.1164	0.511760	7	-0.41	-17.13	-15.7	-15.6	-15.6	2.20	2.17	2.20	2.17	2.21	Niu XL et al.	2011	Journal of Asian Earth Sciences	41(2）	133-146
3243	DHD11- 4	NCC	NCC	116.1977	41.2320	K-feldspar granite porphyry	Donghouding	129	150	10.50	67.30	0.0947	0.511818		-0.52	-16.00	-14.3	-14.0	-14.0	2.08	1.70	2.08	1.71	2.09	Liang QL et al.	2013	Geological Review	59(6)	1119-1130
3244	DHD11- 3	NCC	NCC	116.2166	41.2367	K-feldspar granite porphyry	Donghouding	129	150	10.20	67.40	0.0913	0.511863		-0.54	-15.12	-13.4	-13.1	-13.1	2.00	1.60	2.00	1.60	2.01	Liang QL et al.	2013	Geological Review	59(6)	1119-1130
3245	DHD11- 2	NCC	NCC	116.3151	41.2528	K-feldspar granite porphyry	Donghouding	129	150	10.50	63.40	0.1000	0.511693		-0.49	-18.43	-16.8	-16.6	-16.6	2.29	1.95	2.29	1.95	2.29	Liang QL et al.	2013	Geological Review	59(6)	1119-1130
3246	DHD11- 1	NCC	NCC	116.3132	41.2546	K-feldspar granite porphyry	Donghouding	129	150	14.50	103.00	0.0852	0.511865		-0.57	-15.08	-13.3	-12.9	-12.9	1.99	1.52	1.99	1.52	2.00	Liang QL et al.	2013	Geological Review	59(6)	1119-1130
3247	JS11-4	NCC	NCC	118.2949	40.8173	Syenite	Jiashan	112	150	10.60	61.00	0.1053	0.512317		-0.46	-6.26	-4.95	-4.51	-4.51	1.31	1.17	1.31	1.17	1.31	Liu Y et al.	2015	Acta Petrologica et Mineralogica	34	14-34
3248	JS11-3	NCC	NCC	118.3048	40.8189	Syenite	Jiashan	111	150	8.97	48.40	0.1120	0.512407		-0.43	-4.51	-3.30	-2.88	-2.88	1.17	1.11	1.17	1.12	1.18	Liu Y et al.	2015	Acta Petrologica et Mineralogica	34	14-34
3249	JS11-2	NCC	NCC	118.3005	40.8186	Syenite	Jiashan	109	150	10.20	53.00	0.1164	0.512534		-0.41	-2.03	-0.91	-0.49	-0.49	0.98	0.96	0.98	0.97	0.98	Liu Y et al.	2015	Acta Petrologica et Mineralogica	34	14-34
3250	JS11-1	NCC	NCC	118.3005	40.8186	Syenite	Jiashan	109	150	11.50	66.30	0.1046	0.512369		-0.47	-5.25	-3.98	-3.48	-3.48	1.22	1.09	1.22	1.09	1.23	Liu Y et al.	2015	Acta Petrologica et Mineralogica	34	14-34
3251	KKS-34	CAOB	Western Tianshan	82.0000	44.6000	Monzogranite		306	306	2.31	11.43	0.1207	0.512531		-0.39	-2.09	-2.09	0.88	0.88	0.99	1.01	0.99	0.88	1.01	Zhang D et al.	2012	Lithos	146	65-79
3252	KKS-25	CAOB	Western Tianshan	82.0000	44.6000	Granodiorite		289	289	3.40	18.04	0.1165	0.512539		-0.41	-1.93	-1.93	1.02	1.02	0.97	0.96	0.97	1.02	0.96	Zhang D et al.	2012	Lithos	146	65-79
3253	KKS-23	CAOB	Western Tianshan	82.0000	44.6000	Granodiorite		289	289	3.25	16.31	0.1183	0.512542		-0.40	-1.87	-1.87	1.02	1.02	0.97	0.97	0.97	1.02	0.97	Zhang D et al.	2012	Lithos	146	65-79
3254	KKS-18	CAOB	Western Tianshan	82.0000	44.6000	Monzogranite		306	306	2.02	12.59	0.0958	0.512479		-0.51	-3.10	-3.10	0.83	0.83	1.00	0.87	1.00	0.83	0.87	Zhang D et al.	2012	Lithos	146	65-79
3255	KKS-15	CAOB	Western Tianshan	82.0000	44.6000	Monzogranite		306	306	1.76	10.98	0.0974	0.512486		-0.50	-2.97	-2.97	0.91	0.91	0.99	0.87	0.99	0.91	0.87	Zhang D et al.	2012	Lithos	146	65-79
3256	JB6037-1	NCC	NCC	117.1117	41.3128	Granite	Fengshan	307	307	2.99	16.63	0.1087	0.511899	4	-0.45	-14.42	-11.0	-11.0	-11.0	1.96	1.81	1.96	1.82	1.96	Feng YG et al.	2009	a Scientiarum Naturalium Universitatis Pekiner	45（1）	59+70
3257	JB6035	NCC	NCC	117.1117	41.3128	Granite	Fengshan	307	307	2.49	18.23	0.0827	0.511643	5	-0.58	-19.41	-15.0	-14.9	-14.9	2.28	1.75	2.28	1.75	2.29	Feng YG et al.	2009	a Scientiarum Naturalium Universitatis Pekiner	45（1）	59+70
3258	14SZ18	CAOB	Xing-Meng orogenic belt	112.6811	44.4838	edium grained monzogranite		301	301	6.81	24.70	0.1744	0.512598		-0.11	-0.78	0.08	0.08	0.08	1.05	2.13	1.05	2.13	1.05	Yang ZL et al.	2019	Earth Science	44(1)	268-283
3259	14SZ17	CAOB	Xing-Meng orogenic belt	112.6911	44.4938	edium grained monzogranite		301	301	10.90	45.80	0.1505	0.512681		-0.23	0.84	2.62	2.61	2.61	0.85	1.13	0.85	1.13	0.85	Yang ZL et al.	2019	Earth Science	44(1)	268-283
3260	14SZ15	CAOB	Xing-Meng orogenic belt	112.7091	44.5038	ium fine grained monzogranite		301	301	3.44	14.20	0.1532	0.512612		-0.22	-0.51	1.17	1.16	1.16	0.97	1.35	0.97	1.35	0.97	Yang ZL et al.	2019	Earth Science	44(1)	268-283
3261	14SZ14	CAOB	Xing-Meng orogenic belt	112.7061	44.5028	ium fine grained monzogranite		301	301	4.60	23.00	0.1265	0.512641		-0.36	0.06	2.77	2.75	2.75	0.84	0.89	0.84	0.89	0.84	Yang ZL et al.	2019	Earth Science	44(1)	268-283
3262	WL852656	CAOB	SHB	118.0642	43.7567	Bi granite	Maanzi	148	150	3.81	16.86	0.1367	0.512797	8	-0.30	3.10	4.24	4.25	4.25	0.59	0.70	0.59	0.70	0.59	Liu W et al.	2005	Chemical Geology	219(1-4)	5-35
3263	WL852655	CAOB	SHB	118.0642	43.7567	Bi granite	Maanzi	148	150	7.33	36.68	0.1208	0.512793	9	-0.39	3.02	4.46	4.48	4.48	0.57	0.59	0.57	0.59	0.58	Liu W et al.	2005	Chemical Geology	219(1-4)	5-35
3264	WL852654	CAOB	SHB	118.0642	43.7567	Bi granite	Maanzi	148	150	4.88	24.82	0.1206	0.512843	2	-0.39	4.00	5.43	5.46	5.46	0.49	0.50	0.49	0.51	0.50	Liu W et al.	2005	Chemical Geology	219(1-4)	5-35
3265	WL852653	CAOB	SHB	118.0642	43.7567	Bi granite	Maanzi	148	150	7.05	30.41	0.1318	0.512796	11	-0.33	3.08	4.30	4.33	4.33	0.59	0.66	0.59	0.66	0.59	Liu W et al.	2005	Chemical Geology	219(1-4)	5-35
3266	WL852652	CAOB	SHB	118.0642	43.7567	Bi granite	Maanzi	148	150	9.35	44.90	0.1259	0.512777	11	-0.36	2.71	4.05	4.07	4.07	0.61	0.65	0.61	0.65	0.61	Liu W et al.	2005	Chemical Geology	219(1-4)	5-35
3267	WL852651	CAOB	SHB	118.0642	43.7567	Bi granite	Maanzi	148	150	8.67	29.12	0.1801	0.512880	12	-0.08	4.72	5.04	5.04	5.04	0.53	1.23	0.53	1.23	0.53	Liu W et al.	2005	Chemical Geology	219(1-4)	5-35
3268	WL60425	CAOB	SHB	118.0642	43.7567	Adamellite	Yelaigai	147	150	6.64	33.71	0.1192	0.512664	13	-0.39	0.51	1.96	1.99	1.99	0.78	0.78	0.78	0.79	0.78	Liu W et al.	2005	Chemical Geology	219(1-4)	5-35
3269	WL60424	CAOB	SHB	118.0642	43.7567	Adamellite	Yelaigai	147	150	6.45	32.25	0.1209	0.512620	7	-0.39	-0.35	1.08	1.10	1.10	0.85	0.87	0.85	0.87	0.85	Liu W et al.	2005	Chemical Geology	219(1-4)	5-35
3270	WL60422	CAOB	SHB	118.0642	43.7567	Adamellite	Yelaigai	147	150	12.70	53.39	0.1191	0.512666	9	-0.39	0.55	1.99	2.03	2.03	0.77	0.78	0.77	0.78	0.78	Liu W et al.	2005	Chemical Geology	219(1-4)	5-35
3271	WL60420	CAOB	SHB	118.1856	43.7703	Granodiorite	Longtoushan1	226	226	3.33	17.07	0.1179	0.512753	12	-0.40	2.24	4.53	4.52	4.52	0.63	0.63	0.63	0.64	0.64	Liu W et al.	2005	Chemical Geology	219(1-4)	5-35
3272	WL53069	CAOB	SHB	117.9736	43.4792	Granodiorite	Xiaochengzi	110	150	4.31	26.75	0.0981	0.512428	15	-0.50	-4.10	-2.72	-2.21	-2.21	1.12	0.95	1.12	0.95	1.13	Liu W et al.	2005	Chemical Geology	219(1-4)	5-35
3273	WL53068	CAOB	SHB	117.9736	43.4792	Granodiorite	Xiaochengzi	110	150	4.20	23.65	0.1075	0.512436	8	-0.45	-3.94	-2.68	-2.23	-2.23	1.12	1.02	1.12	1.03	1.13	Liu W et al.	2005	Chemical Geology	219(1-4)	5-35
3274	WL53066	CAOB	SHB	117.9736	43.4792	Granodiorite	Xiaochengzi	110	150	3.78	21.92	0.1042	0.512473	9	-0.47	-3.22	-1.92	-1.45	-1.45	1.06	0.94	1.06	0.95	1.07	Liu W et al.	2005	Chemical Geology	219(1-4)	5-35
3275	WL53065	CAOB	SHB	117.9736	43.4792	Granodiorite	Xiaochengzi	110	150	4.19	25.31	0.1001	0.512472	10	-0.49	-3.24	-1.88	-1.39	-1.39	1.05	0.91	1.05	0.91	1.06	Liu W et al.	2005	Chemical Geology	219(1-4)	5-35
3276	WL53064	CAOB	SHB	117.9736	43.4792	Granodiorite	Xiaochengzi	110	150	4.22	22.33	0.1142	0.512456	9	-0.42	-3.55	-2.39	-1.97	-1.97	1.10	1.06	1.10	1.07	1.10	Liu W et al.	2005	Chemical Geology	219(1-4)	5-35
3277	WL53063	CAOB	SHB	117.9736	43.4792	Granodiorite	Xiaochengzi	110	150	4.34	11.79	0.1153	0.512431	12	-0.41	-4.04	-2.90	-2.48	-2.48	1.14	1.11	1.14	1.12	1.14	Liu W et al.	2005	Chemical Geology	219(1-4)	5-35
3278	V Ⅱ 07B	CAOB	SHB	118.1856	43.7703	Adamellite	Longtoushan1	226	226	4.73	25.51	0.1122	0.512667	3	-0.43	0.57	3.00	3.00	3.00	0.76	0.73	0.76	0.73	0.76	Liu W et al.	2005	Chemical Geology	219(1-4)	5-35
3279	Lw97080	CAOB	SHB	118.0642	43.7567	Adamellite	Yelaigai	147	150	11.96	45.33	0.2690	0.512690	8	0.37	1.01	-0.35	-0.37	-0.37	0.98	-1.28	0.98		0.97	Liu W et al.	2005	Chemical Geology	219(1-4)	5-35
3280	Lw97079	CAOB	SHB	118.0642	43.7567	Adamellite	Yelaigai	147	150	10.58	41.01	0.1434	0.512665	11	-0.27	0.53	1.53	1.55	1.55	0.81	1.05	0.81	1.06	0.81	Liu W et al.	2005	Chemical Geology	219(1-4)	5-35
3281	Lw97078	CAOB	SHB	118.1856	43.7703	Granodiorite	Longtoushan1	226	226	3.23	17.39	0.1125	0.512712	8	-0.43	1.44	3.88	3.87	3.87	0.69	0.66	0.69	0.66	0.69	Liu W et al.	2005	Chemical Geology	219(1-4)	5-35
3282	Lw97077	CAOB	SHB	118.1856	43.7703	Granodiorite	Longtoushan1	226	226	3.55	18.90	0.1137	0.512747	12	-0.42	2.13	4.53	4.52	4.52	0.63	0.62	0.63	0.62	0.64	Liu W et al.	2005	Chemical Geology	219(1-4)	5-35
3283	Lw97076	CAOB	SHB	118.1856	43.7703	Granodiorite	Longtoushan1	226	226	3.06	16.68	0.1110	0.512798	7	-0.44	3.12	5.60	5.59	5.59	0.55	0.52	0.55	0.53	0.55	Liu W et al.	2005	Chemical Geology	219(1-4)	5-35
3284	Lw97066	CAOB	SHB	118.0642	43.7567	Diorite-granite	Maanzi	148	150	5.09	25.76	0.1194	0.512787	9	-0.39	2.91	4.36	4.39	4.39	0.58	0.59	0.58	0.59	0.58	Liu W et al.	2005	Chemical Geology	219(1-4)	5-35
3285	Lw97065	CAOB	SHB	118.0642	43.7567	Diorite-granite	Maanzi	148	150	5.45	28.49	0.1157	0.512769	11	-0.41	2.56	4.09	4.11	4.11	0.60	0.59	0.60	0.60	0.61	Liu W et al.	2005	Chemical Geology	219(1-4)	5-35
3286	Lw97064-1	CAOB	SHB	118.0642	43.7567	Diorite-granite	Maanzi	148	150	5.68	27.65	0.1242	0.512779	12	-0.37	2.75	4.13	4.14	4.14	0.60	0.63	0.60	0.64	0.60	Liu W et al.	2005	Chemical Geology	219(1-4)	5-35
3287	Lw97064	CAOB	SHB	118.0642	43.7567	Diorite-granite	Maanzi	148	150	7.91	40.87	0.1197	0.512812	13	-0.39	3.39	4.85	4.87	4.87	0.54	0.55	0.54	0.55	0.54	Liu W et al.	2005	Chemical Geology	219(1-4)	5-35
3288	Lw97020	CAOB	SHB	118.1856	43.7703	Granodiorite	Longtoushan2	117	150	7.50	40.28	0.1348	0.512626	6	-0.31	-0.23	0.69	0.95	0.95	0.86	1.01	0.86	1.02	0.86	Liu W et al.	2005	Chemical Geology	219(1-4)	5-35
3289	Lw97019	CAOB	SHB	118.1856	43.7703	Granodiorite	Lingtoushan2	117	150	7.42	39.86	0.1126	0.512606	11	-0.43	-0.62	0.64	0.99	0.99	0.86	0.82	0.86	0.82	0.86	Liu W et al.	2005	Chemical Geology	219(1-4)	5-35
3290	Lw97016	CAOB	SHB	117.9736	43.4792	Adamellite	Xiaochengzi	110	150	3.39	18.60	0.1103	0.512428	9	-0.44	-4.10	-2.88	-2.44	-2.44	1.14	1.06	1.14	1.07	1.14	Liu W et al.	2005	Chemical Geology	219(1-4)	5-35
3291	D058-1	NCC	NCC	117.7725	41.2053	Diorites	Gushan	390	390	3.83	19.90	0.1162	0.511944	11	-0.41	-13.54	-9.54	-9.53	-9.53	1.91	1.88	1.91	1.88	1.91	Zhang SH et al.	2007	Geological Magazine	144(03)	116-24
3292	D055-1	NCC	NCC	117.8011	41.2025	Diorites	Gushan	390	390	6.69	33.27	0.1216	0.512064	12	-0.38	-11.20	-7.47	-7.46	-7.46	1.74	1.79	1.74	1.80	1.74	Zhang SH et al.	2007	Geological Magazine	144(03)	116-24
3293	KoG25	CAOB	Kazakhstan	74.9667	46.9833	Granite		322	322	3.51	17.80	0.1201	0.512260		-0.39	-7.37	-4.23	-4.23	-4.23	1.42	1.45	1.42			Shen P et al.	2017	Lithos	286	53-74
3294	KoeK16	CAOB	Kazakhstan	75.1667	46.9833	Granite		295	295	0.51	5.70	0.0545	0.512341		-0.72	-5.79	-0.44	-0.44	-0.44	1.09	0.78	1.09			Shen P et al.	2017	Lithos	286	53-74
3295	9HS16-2	CAOB	Kazakhstan	75.1667	46.9833	Granite		295	295	5.65	34.00	0.1012	0.512530		-0.49	-2.11	1.49	1.49	1.49	0.94	0.84	0.94			Shen P et al.	2017	Lithos	286	53-74
3296	9HS16-1	CAOB	Kazakhstan	75.1667	46.9833	Granite		295	295	2.16	20.00	0.0658	0.512430		-0.67	-4.06	0.88	0.87	0.87	0.99	0.74	0.99			Shen P et al.	2017	Lithos	286	53-74
3297	9HS15-6-2	CAOB	Kazakhstan	74.9667	46.9833	Granite		322	322	2.02	11.00	0.1118	0.512456		-0.43	-3.55	-0.06	-0.06	-0.06	1.08	1.04	1.08			Shen P et al.	2017	Lithos	286	53-74
3298	9HS15-6-1	CAOB	Kazakhstan	74.9667	46.9833	Granodiorite		322	322	2.64	15.00	0.1072	0.512491		-0.46	-2.87	0.81	0.81	0.81	1.01	0.94	1.01			Shen P et al.	2017	Lithos	286	53-74
3299	9HS15-3-2	CAOB	Kazakhstan	74.9667	46.9833	Granodiorite		322	322	3.00	18.00	0.1015	0.512488		-0.48	-2.93	0.99	0.99	0.99	1.00	0.90	1.00			Shen P et al.	2017	Lithos	286	53-74
3300	9HS13-3	CAOB	Kazakhstan	74.7167	47.1667	Granite		311	311	3.36	17.00	0.1204	0.512458		-0.39	-3.51	-0.48	-0.48	-0.48	1.11	1.13	1.11			Shen P et al.	2017	Lithos	286	53-74
3301	9HS13-12	CAOB	Kazakhstan	74.7167	47.1667	Granite		311	311	3.93	22.00	0.1088	0.512394		-0.45	-4.76	-1.27	-1.27	-1.27	1.17	1.10	1.17			Shen P et al.	2017	Lithos	286	53-74
3302	9499-3	CAOB	SMXAB	125.6672	50.5414	Granite	Woduhe	127	150	3. 607	8. 312	0.2625	0.512564	17	0.33	-1.44	-2.51	-2.70	-2.70	1.17	-1.85	1.17		1.13	Wu FY et al.	1999	Acta Petrologica Sinica	15(2)	181-189
3303	ST03	NCC	NCC	115.4319	40.1981	Syenite	fanshan	218	218			0.0930	0.512010	4	-0.53	-12.25	-9.38	-9.37	-9.37	1.76	1.44	1.76	1.44	1.76	Hou T et al.	2015	Journal of Petrology	2015(5)	251-256
3304	ST02	NCC	NCC	115.4319	40.1981	Syenite	fanshan	218	218			0.0929	0.512010	7	-0.53	-12.25	-9.38	-9.36	-9.36	1.76	1.44	1.76	1.44	1.76	Hou T et al.	2015	Journal of Petrology	2015(5)	251-256
3305	ST01	NCC	NCC	115.4319	40.1981	Syenite	fanshan	218	218			0.0935	0.512000	3	-0.52	-12.45	-9.57	-9.57	-9.57	1.77	1.46	1.77	1.46	1.78	Hou T et al.	2015	Journal of Petrology	2015(5)	251-256
3306	KSh-26/1	CAOB	Russia	88.5500	55.0667	Carbonatite		490	490	25.10	121.00	0.1253	0.512704	16	-0.36	1.29	5.72	5.75	5.75	0.75	0.77	0.75			Vrublevskii VV.	2020	Journal of Asian Earth Sciences	188	104163
3307	KL	CAOB	Russia	96.7000	51.1000	Carbonatite		490	490	30.30	176.00	0.1044	0.512521	5	-0.47	-2.28	3.50	3.49	3.49	0.93	0.88	0.93			Vrublevskii VV.	2020	Journal of Asian Earth Sciences	188	104163
3308	KF-2	CAOB	Russia	96.7000	51.1000	Foyaite*		490	490	4.18	18.20	0.1384	0.512607	10	-0.30	-0.60	3.05	3.04	3.04	0.97	1.10	0.97			Vrublevskii VV.	2020	Journal of Asian Earth Sciences	188	104163
3309	KF-1	CAOB	Russia	96.7000	51.1000	Ijolite		490	490	2.36	10.70	0.1331	0.512587	10	-0.32	-0.99	2.86	2.98	2.98	0.97	1.06	0.97			Vrublevskii VV.	2020	Journal of Asian Earth Sciences	188	104163
3310	KC	CAOB	Russia	96.7000	51.1000	Foyaite		490	490	2.22	9.73	0.1377	0.512603	17	-0.30	-0.68	3.01	3.01	3.01	0.97	1.10	0.97			Vrublevskii VV.	2020	Journal of Asian Earth Sciences	188	104163
3311	DI-2	CAOB	Russia	96.7000	51.1000	Ijolite		490	490	6.44	33.80	0.1151	0.512626	8	-0.41	-0.23	4.88	4.87	4.87	0.82	0.81	0.82			Vrublevskii VV.	2020	Journal of Asian Earth Sciences	188	104163
3312	DI-1	CAOB	Russia	96.7000	51.1000	Jacupirangite		490	490	6.68	34.60	0.1166	0.512591	14	-0.41	-0.92	4.10	4.09	4.09	0.88	0.88	0.88			Vrublevskii VV.	2020	Journal of Asian Earth Sciences	188	104163
3313	DF	CAOB	Russia	96.7000	51.1000	Ijolite*		490	490	3.15	14.20	0.1344	0.512759	10	-0.32	2.36	6.27	6.26	6.26	0.71	0.75	0.71			Vrublevskii VV.	2020	Journal of Asian Earth Sciences	188	104163
3314	DC	CAOB	Russia	96.7000	51.1000	Foyaite		490	490	5.88	25.20	0.1414	0.512777	15	-0.28	2.71	6.17	6.17	6.17	0.71	0.79	0.71			Vrublevskii VV.	2020	Journal of Asian Earth Sciences	188	104163
3315	CL-2	CAOB	Russia	96.7000	51.1000	Ijolite*		490	490	23.20	64.70	0.2164	0.512965	10	0.10	6.38	5.16	5.15	5.15	0.82	-10.98	0.82			Vrublevskii VV.	2020	Journal of Asian Earth Sciences	188	104163
3316	CL-1	CAOB	Russia	96.7000	51.1000	Ijolite		490	490	1.55	6.45	0.1448	0.512709	18	-0.26	1.38	4.63	4.63	4.63	0.84	0.98	0.84			Vrublevskii VV.	2020	Journal of Asian Earth Sciences	188	104163
3317	CL	CAOB	Russia	96.7000	51.1000	Carbonatite		490	490	15.40	93.80	0.0993	0.512480	10	-0.50	-3.08	3.01	3.01	3.01	0.97	0.89	0.97			Vrublevskii VV.	2020	Journal of Asian Earth Sciences	188	104163
3318	BJ	CAOB	Russia	96.7000	51.1000	Foyaite		490	490	2.42	11.40	0.1286	0.512581	7	-0.35	-1.11	3.14	3.14	3.14	0.96	1.02	0.96			Vrublevskii VV.	2020	Journal of Asian Earth Sciences	188	104163
3319	BF	CAOB	Russia	96.7000	51.1000	Carbonatite		490	490	2.87	12.40	0.1402	0.512789	14	-0.29	2.95	6.48	6.48	6.48	0.69	0.75	0.69			Vrublevskii VV.	2020	Journal of Asian Earth Sciences	188	104163
3320	BC	CAOB	Russia	96.7000	51.1000	Juvite		490	490	1.15	5.60	0.1237	0.512583	8	-0.37	-1.07	3.50	3.49	3.49	0.93	0.96	0.93			Vrublevskii VV.	2020	Journal of Asian Earth Sciences	188	104163
3321	BB-117	CAOB	Tianshan	88.0800	44.2000	Metagranodiorite		341	341	6.05	26.80	0.1535	0.512849		-0.22	4.12	5.89	6.00	6.00	0.61	0.76	0.61			Xia L Q et al.	2004	Geological Society of America Bulletin	116(3-4)	419-433
3322	X-606	CAOB	ogda-halrik mountain, Tiansha	93.9667	42.1333	Dioritic MME	Badashi	301	301	6.19	27.83	0.1345	0.512728	11	-0.32	1.76	4.15	4.15	4.15	0.72	0.81	0.72		0.72	Wang CS et al.	2009	Acta Petrologica et Mineralogica	28(04)	299-315
3323	98-30	NCC	NCC	114.9492	39.4567	Qtz syenite	Wang'anzhen	126	150	7.03	50.98	0.0834	0.511712	15	-0.58	-18.06	-16.3	-15.9	-15.9	2.23	1.68	2.23	1.68	2.24	Cai J et al.	2003	Acta Petrologica Sinica	19（1）	81-92
3324	97-52	NCC	NCC	114.9492	39.4567	Granodiorite	Wang'anzhen	146	150	5.58	41.46	0.0960	0.511711	8	-0.51	-18.08	-16.2	-16.2	-16.2	2.25	1.86	2.25	1.86	2.25	Cai J et al.	2003	Acta Petrologica Sinica	19（1）	81-92
3325	20-33	NCC	NCC	114.9492	39.4567	Granite	Wang'anzhen	138	150	3.34	24.33	0.0831	0.511804	8	-0.58	-16.27	-14.3	-14.1	-14.1	2.09	1.57	2.09	1.57	2.09	Cai J et al.	2003	Acta Petrologica Sinica	19（1）	81-92
3326	20-21	NCC	NCC	114.9492	39.4567	Diorite	Wang'anzhen	187	187	6.19	35.95	0.1041	0.511668	9	-0.47	-18.92	-16.7	-16.7	-16.7	2.33	2.05	2.33	2.06	2.33	Cai J et al.	2003	Acta Petrologica Sinica	19（1）	81-92
3327	20-3	NCC	NCC	114.9492	39.4567	Hornblendite	Wang'anzhen	223	223	2.73	12.95	0.1273	0.511977	9	-0.35	-12.89	-10.9	-10.9	-10.9	1.88	2.06	1.88	2.07	1.89	Cai J et al.	2003	Acta Petrologica Sinica	19（1）	81-92
3328	FS-9	NCC	NCC	115.4319	40.1981	Syenites	Fanshan	220	220	23.80	145.80	0.0986	0.512201	2	-0.50	-8.52	-5.77	-5.77	-5.77	1.47	1.26	1.47	1.26	1.47	Niu XL et al.	2012	Lithos	149	146-158
3329	FS-6	NCC	NCC	115.4319	40.1981	arnet-clinopyroxene syenite	Fanshan	220	220	72.40	275.40	0.1592	0.512313	3	-0.19	-6.34	-5.29	-5.29	-5.29	1.42	2.33	1.42	2.34	1.43	Niu XL et al.	2012	Lithos	149	146-158
3330	FS-5	NCC	NCC	115.4319	40.1981	Syenites	Fanshan	220	220	20.00	117.40	0.1030	0.512223	3	-0.48	-8.10	-5.46	-5.46	-5.46	1.44	1.28	1.44	1.28	1.44	Niu XL et al.	2012	Lithos	149	146-158
3331	FS-4	NCC	NCC	115.4319	40.1981	Syenites	Fanshan	220	220	2.30	17.20	0.0815	0.512178	3	-0.59	-8.97	-5.73	-5.74	-5.74	1.46	1.12	1.46	1.12	1.47	Niu XL et al.	2012	Lithos	149	146-158
3332	FS-3	NCC	NCC	115.4319	40.1981	arnet-clinopyroxene syenite	Fanshan	220	220	22.70	114.60	0.1198	0.512230	6	-0.39	-7.96	-5.79	-5.80	-5.80	1.47	1.49	1.47	1.49	1.47	Niu XL et al.	2012	Lithos	149	146-158
3333	FS-13	NCC	NCC	115.4319	40.1981	arnet-clinopyroxene syenite	Fanshan	220	220	18.60	106.10	0.1063	0.512217	5	-0.46	-8.21	-5.68	-5.67	-5.67	1.46	1.32	1.46	1.33	1.46	Niu XL et al.	2012	Lithos	149	146-158
3334	FS-12	NCC	NCC	115.4319	40.1981	arnet-clinopyroxene syenite	Fanshan	220	220	23.80	140.90	0.1022	0.512216	3	-0.48	-8.23	-5.58	-5.58	-5.58	1.45	1.28	1.45	1.28	1.45	Niu XL et al.	2012	Lithos	149	146-158

3335	FS-10	NCC	NCC	115.4319	40.1981	arnet-clinopyroxene syenite	Fanshan	220	220	18.90	113.90	0.1003	0.512227	5	-0.49	-8.02	-5.30	-5.31	-5.31	1.43	1.24	1.43	1.24	1.43	Niu XL et al.	2012	Lithos	149	146-158
3336	NJ11-5	CAOB	South Tianshan	82.5000	42.0833	Rhyolite		295	295			0.0996	0.512130		-0.49	-9.91	-6.26	-6.25	-6.25	1.57	1.36	1.57	1.36	1.58	Liu DD et al.	2014	International Journal of Earth Sciences	103(3)	737-756
3337	NJ11-27	CAOB	South Tianshan	82.5000	42.0833	Qtz porphyry		295	295			0.1241	0.512220		-0.37	-8.15	-5.42	-5.42	-5.42	1.50	1.58	1.50	1.58	1.51	Liu DD et al.	2014	International Journal of Earth Sciences	103(3)	737-756
3338	NJ11-2	CAOB	South Tianshan	82.5000	42.0833	Qtz porphyry		295	295			0.1087	0.512143		-0.45	-9.66	-6.35	-6.34	-6.34	1.57	1.46	1.57	1.46	1.58	Liu DD et al.	2014	International Journal of Earth Sciences	103(3)	737-756
3339	T19	NCC	NCC	115.5308	40.9622	Leucosyenite	Shuiquangou	388	388	4.60	22.40	0.1240	0.511981	5	-0.37	-12.82	-9.22	-9.22	-9.22	1.88	1.98	1.88	1.98	1.88	Jiang N et al.	2005	Journal of the Geological Society	162(1)	203-215
3340	T18	NCC	NCC	115.5308	40.9622	Leucosyenite	Shuiquangou	388	388	5.10	23.90	0.1296	0.511990	13	-0.34	-12.64	-9.32	-9.32	-9.32	1.89	2.10	1.89	2.10	1.89	Jiang N et al.	2005	Journal of the Geological Society	162(1)	203-215
3341	N69	NCC	NCC	115.5308	40.9622	Leucosyenite	Shuiquangou	388	388	8.20	46.50	0.1071	0.511945	10	-0.46	-13.52	-9.08	-9.08	-9.08	1.87	1.72	1.87	1.72	1.87	Jiang N et al.	2005	Journal of the Geological Society	162(1)	203-215
3342	N46	NCC	NCC	115.5308	40.9622	Leucosyenite	Shuiquangou	388	388	1.10	4.30	0.1493	0.511912	5	-0.24	-14.16	-11.8	-11.8	-11.8	2.09	2.91	2.09	2.92	2.09	Jiang N et al.	2005	Journal of the Geological Society	162(1)	203-215
3343	N45	NCC	NCC	115.5308	40.9622	Leucosyenite	Shuiquangou	388	388	3.70	19.50	0.1151	0.511941	8	-0.41	-13.60	-9.57	-9.56	-9.56	1.91	1.86	1.91	1.87	1.91	Jiang N et al.	2005	Journal of the Geological Society	162(1)	203-215
3344	N44	NCC	NCC	115.5308	40.9622	Leucosyenite	Shuiquangou	388	388	3.00	12.60	0.1449	0.511959	3	-0.26	-13.25	-10.7	-10.7	-10.7	2.00	2.62	2.00	2.63	2.00	Jiang N et al.	2005	Journal of the Geological Society	162(1)	203-215
3345	N43	NCC	NCC	115.5308	40.9622	Leucosyenite	Shuiquangou	388	388	2.40	9.30	0.1560	0.512008	11	-0.21	-12.29	-10.3	-10.3	-10.3	1.96	3.00	1.96	3.01	1.97	Jiang N et al.	2005	Journal of the Geological Society	162(1)	203-215
3346	N27	NCC	NCC	115.5308	40.9622	Leucosyenite	Shuiquangou	388	388	3.20	12.70	0.1504	0.512057	11	-0.24	-11.33	-9.05	-9.04	-9.04	1.86	2.62	1.86	2.63	1.87	Jiang N et al.	2005	Journal of the Geological Society	162(1)	203-215
3347	80-2	NCC	NCC	115.5308	40.9622	Aplite	Shuiquangou	388	388	0.60	2.70	0.1335	0.511802	19	-0.32	-16.31	-13.2	-13.2	-13.2	2.20	2.55	2.20	2.55	2.21	Jiang N et al.	2005	Journal of the Geological Society	162(1)	203-215
3348	80-1	NCC	NCC	115.5308	40.9622	Leucosyenite	Shuiquangou	388	388	1.50	6.70	0.1319	0.511931	10	-0.33	-13.79	-10.6	-10.6	-10.6	1.99	2.26	1.99	2.27	2.00	Jiang N et al.	2005	Journal of the Geological Society	162(1)	203-215
3349	114	NCC	NCC	115.5308	40.9622	Melasyenite	Shuiquangou	388	388	16.10	79.20	0.1233	0.512077	2	-0.37	-10.94	-7.31	-7.31	-7.31	1.73	1.80	1.73	1.81	1.73	Jiang N et al.	2005	Journal of the Geological Society	162(1)	203-215
3350	106	NCC	NCC	115.5308	40.9622	Melasyenite	Shuiquangou	388	388	5.20	35.00	0.0894	0.511495	7	-0.55	-22.30	-16.99	-16.98	-16.98	2.51	2.02	2.51	2.03	2.51	Jiang N et al.	2005	Journal of the Geological Society	162(1)	203-215
3351	84	NCC	NCC	115.5308	40.9622	Leucosyenite	Shuiquangou	388	388	0.70	4.40	0.0898	0.511805	9	-0.54	-16.25	-10.96	-10.96	-10.96	2.02	1.65	2.02	1.65	2.03	Jiang N et al.	2005	Journal of the Geological Society	162(1)	203-215
3352	82	NCC	NCC	115.5308	40.9622	Aplite	Shuiquangou	388	388	0.40	2.40	0.1080	0.511740	7	-0.45	-17.52	-13.15	-13.13	-13.13	2.20	2.03	2.20	2.03	2.20	Jiang N et al.	2005	Journal of the Geological Society	162(1)	203-215
3353	81	NCC	NCC	115.5308	40.9622	Aplite	Shuiquangou	388	388	0.40	2.50	0.1050	0.511774	7	-0.47	-16.85	-12.33	-12.32	-12.32	2.13	1.92	2.13	1.93	2.14	Jiang N et al.	2005	Journal of the Geological Society	162(1)	203-215
3354	YC08-2	NCC	NCC	112.4311	39.9186	Monzonite	Dishuiyan	241	241	5.64	30.80	0.1110	0.512079	6	-0.44	-10.90	-8.27	-8.27	-8.27	1.69	1.59	1.69	1.59	1.69	Yin JF et al.	2007	Lithos	98(1-4)	45-66
3355	YC08-1	NCC	NCC	112.4311	39.9186	Monzonite	Dishuiyan	241	241	4.69	24.90	0.1140	0.512103	5	-0.42	-10.44	-7.90	-7.89	-7.89	1.65	1.60	1.65	1.60	1.66	Yin JF et al.	2007	Lithos	98(1-4)	45-66
3356	XL27	CAOB	Altai	120.6500	41.7800	Adakite		155	155	4.17	22.82	0.1113	0.512517		-0.43	-2.36	-0.68	-0.68	-0.68	1.00	0.94	1.00			Kruk NN et al.	2008	Petrology	16(5)	512-530
3357	XL22	CAOB	Altai	120.6500	41.7800	Adakite		155	155	4.11	22.81	0.1096	0.512442		-0.44	-3.82	-2.11	-2.11	-2.11	1.11	1.04	1.11			Kruk NN et al.	2008	Petrology	16(5)	512-530
3358	XL21	CAOB	Altai	120.6500	41.7800	Dacite		155	155	4.02	22.23	0.1100	0.512413		-0.44	-4.39	-2.68	-2.68	-2.68	1.16	1.08	1.16			Kruk NN et al.	2008	Petrology	16(5)	512-530
3359	XL20	CAOB	Altai	120.6500	41.7800	Dacite		155	155	5.14	25.98	0.1205	0.512567		-0.39	-1.38	0.12	0.12	0.12	0.93	0.95	0.93			Kruk NN et al.	2008	Petrology	16(5)	512-530
3360	XL19	CAOB	Altai	120.6500	41.7800	Dacite		155	155	3.90	21.14	0.1122	0.512525		-0.43	-2.20	-0.54	-0.54	-0.54	0.99	0.94	0.99			Kruk NN et al.	2008	Petrology	16(5)	512-530
3361	XL18	CAOB	Altai	120.6500	41.7800	Dacite		155	155	3.88	20.67	0.1142	0.512556		-0.42	-1.60	0.03	0.03	0.03	0.94	0.91	0.94			Kruk NN et al.	2008	Petrology	16(5)	512-530
3362	XL17	CAOB	Altai	120.6500	41.7800	Dacite		155	155	4.31	23.92	0.1097	0.512575		-0.44	-1.23	0.49	0.49	0.49	0.90	0.84	0.90			Kruk NN et al.	2008	Petrology	16(5)	512-530
3363	XL16	CAOB	Altai	120.6500	41.7800	Andesite		155	155	6.14	30.68	0.1220	0.512668		-0.38	0.59	2.06	2.06	2.06	0.77	0.80	0.77			Kruk NN et al.	2008	Petrology	16(5)	512-530
3364	XL15	CAOB	Altai	120.6500	41.7800	Adakite		155	155	6.24	30.69	0.1238	0.512667		-0.37	0.57	2.00	2.00	2.00	0.78	0.82	0.78			Kruk NN et al.	2008	Petrology	16(5)	512-530
3365	XL12	CAOB	Altai	120.6500	41.7800	Adakite		155	155	5.10	26.62	0.1167	0.512609		-0.41	-0.57	1.01	1.01	1.01	0.86	0.85	0.86			Kruk NN et al.	2008	Petrology	16(5)	512-530
3366	XL05	CAOB	Altai	120.6500	41.7800	Adakite		155	155	3.96	21.08	0.1143	0.512511		-0.42	-2.48	-0.85	-0.85	-0.85	1.01	0.98	1.01			Kruk NN et al.	2008	Petrology	16(5)	512-530
3367	XL04	CAOB	Altai	120.6500	41.7800	Adakite		155	155	4.03	21.48	0.1142	0.512448		-0.42	-3.71	-2.08	-2.08	-2.08	1.11	1.08	1.11			Kruk NN et al.	2008	Petrology	16(5)	512-530
3368	XL03	CAOB	Altai	120.6500	41.7800	Adakite		155	155	4.05	21.62	0.1141	0.512511		-0.42	-2.48	-0.85	-0.85	-0.85	1.01	0.98	1.01			Kruk NN et al.	2008	Petrology	16(5)	512-530
3369	XL02	CAOB	Altai	120.6500	41.7800	Adakite		155	155	3.56	20.27	0.1068	0.512471		-0.46	-3.26	-1.49	-1.49	-1.49	1.06	0.97	1.06			Kruk NN et al.	2008	Petrology	16(5)	512-530
3370	XL01	CAOB	Altai	120.6500	41.7800	Dacite		155	155	4.00	22.00	0.1107	0.512489		-0.44	-2.91	-1.21	-1.21	-1.21	1.04	0.98	1.04			Kruk NN et al.	2008	Petrology	16(5)	512-530
3371	TS191	CAOB	Tianshan	85.0000	43.0000	Rhyolite		329	329	9.12	37.40	0.1485	0.512646		-0.24	0.16	2.18	2.18	2.18	0.91	1.18	0.91			Zhu YF et al.	2009	Journal of the Geological Society	166(Pt.6)	1085-1099
3372	TS022	CAOB	Tianshan	85.0000	44.0000	Andesite		329	329	8.55	36.27	0.1436	0.512716		-0.27	1.52	3.76	3.75	3.75	0.78	0.94	0.78			Zhu YF et al.	2009	Journal of the Geological Society	166(Pt.6)	1085-1099
3373	TS018	CAOB	Tianshan	85.0000	44.0000	Rhyolite		329	329	2.90	12.88	0.1371	0.512730		-0.30	1.79	4.30	4.30	4.30	0.73	0.84	0.73			Zhu YF et al.	2009	Journal of the Geological Society	166(Pt.6)	1085-1099
3374	TS017	CAOB	Tianshan	85.0000	44.0000	Rhyolite		329	329	3.05	15.15	0.1226	0.512649		-0.38	0.21	3.33	3.32	3.32	0.81	0.84	0.81			Zhu YF et al.	2009	Journal of the Geological Society	166(Pt.6)	1085-1099
3375	TS010	CAOB	Tianshan	85.0000	44.0000	Andesite		329	329	6.30	27.46	0.1397	0.512663		-0.29	0.49	2.88	2.88	2.88	0.85	1.00	0.85			Zhu YF et al.	2009	Journal of the Geological Society	166(Pt.6)	1085-1099
3376	TS007	CAOB	Tianshan	85.0000	44.0000	Dacite		329	329	4.79	19.81	0.1473	0.512835		-0.25	3.84	5.92	5.92	5.92	0.60	0.72	0.60			Zhu YF et al.	2009	Journal of the Geological Society	166(Pt.6)	1085-1099
3377	09AL202	CAOB	Alashan	106.1333	41.4561	Monzogranite	South Diebusige	337	337	4.26	26.40	0.1037	0.511504	8	-0.47	-22.12	-18.0	-18.1	-18.1	2.56	2.27	2.56	2.27	2.55	Dan W et al.	2016	Gondwana Research	32	105-121
3378	09AL200	CAOB	Alashan, NH zone	106.1208	40.4561	Monzogranite	South Diebusige	337	337	6.23	37.40	0.1005	0.511503	6	-0.49	-22.14	-17.9	-18.0	-18.0	2.55	2.21	2.55	2.21	2.54	Dan W et al.	2016	Gondwana Research	32	105-121
3379	09AL198	CAOB	Alashan, NH zone	106.1333	40.4508	Monzogranite	Diebusige	344	344	3.23	22.00	0.0888	0.511424	7	-0.55	-23.68	-18.8	-18.9	-18.9	2.63	2.10	2.63	2.10	2.63	Dan W et al.	2016	Gondwana Research	32	105-121
3380	09AL191	CAOB	Alashan, NH zone	106.1333	40.4508	Monzogranite	Diebusige	346	346	3.01	20.80	0.0874	0.511410	12	-0.56	-23.95	-19.0	-19.1	-19.1	2.65	2.09	2.65	2.09	2.65	Dan W et al.	2016	Gondwana Research	32	105-121
3381	09AL148	CAOB	Alashan	105.5342	40.1061	Qtz diorite	Bayanbulage	423	423	6.47	36.00	0.1087	0.512007	7	-0.45	-12.31	-7.42	-7.56	-7.56	1.78	1.66	1.78	1.66	1.77	Dan W et al.	2016	Gondwana Research	32	105-121
3382	09AL147	CAOB	Alashan, NH zone	105.5342	40.1061	Qtz diorite	Bayanbulage	423	423	8.55	37.90	0.1362	0.512000	7	-0.31	-12.45	-9.05	-9.18	-9.18	1.90	2.25	1.90	2.25	1.90	Dan W et al.	2016	Gondwana Research	32	105-121
3383	09AL131	CAOB	Alashan, NH zone	104.9694	39.3917	Monzogranite	Hetun	366	366	1.28	7.61	0.1017	0.511731	7	-0.48	-17.69	-13.1	-13.3	-13.3	2.19	1.93	2.19	1.93	2.19	Dan W et al.	2016	Gondwana Research	32	105-121
3384	09AL130	CAOB	Alashan	104.9769	39.4328	Monzogranite	Hetun	345	345	2.04	11.60	0.1060	0.511738	9	-0.46	-17.56	-13.4	-13.6	-13.6	2.20	1.99	2.20	1.99	2.19	Dan W et al.	2016	Gondwana Research	32	105-121
3385	09AL128	CAOB	Alashan	104.9769	39.4328	Monzogranite	Hetun	345	345	2.68	15.60	0.1036	0.511731	7	-0.47	-17.69	-13.5	-13.6	-13.6	2.20	1.96	2.20	1.96	2.20	Dan W et al.	2016	Gondwana Research	32	105-121
3386	09AL127	CAOB	Alashan, NH zone	104.9769	39.4328	Monzogranite	Hetun	345	345	2.05	6.73	0.1838	0.511761	6	-0.07	-17.11	-16.4	-16.5	-16.5	2.40	6.94	2.40	6.94	2.36	Dan W et al.	2016	Gondwana Research	32	105-121
3387	A4	CAOB	Altai, unit 4	87.9856	47.8261	Porphyritic granite	Aweitan	271	271	2.87	17.49	0.0992	0.512744	9	-0.50	2.07	5.50	5.44	5.44	0.59	0.54	0.59	0.54	0.60	Tong Y et al.	2014	American Journal of Science	314(1)	80-109
3388	A3	CAOB	Altai, unit 6	88.9083	47.0456	Bi monzogranite	Adenbluk	271	271	12.59	66.21	0.1059	0.512771	16	-0.46	2.59	5.73	5.73	5.73	0.57	0.54	0.57	0.54	0.57	Tong Y et al.	2014	American Journal of Science	314(1)	80-109
3389	A14	CAOB	Altai, unit 5	89.4258	47.0033	Granodiorite	Fuyunnan	275	275	11.61	53.51	0.1312	0.512808	12	-0.33	3.32	5.60	5.61	5.61	0.58	0.63	0.58	0.63	0.58	Tong Y et al.	2014	American Journal of Science	314(1)	80-109
3390	A13	CAOB	Altai, unit 5	89.4258	47.0033	Mylonitized diorite	Fuyunnan	282	282	1.24	6.47	0.1154	0.512852	12	-0.41	4.17	7.10	7.10	7.10	0.47	0.46	0.47	0.46	0.47	Tong Y et al.	2014	American Journal of Science	314(1)	80-109
3391	97-19	CAOB	Altai, unit 6	88.9083	47.0456	Bi monzogranite	Adenbluk	271	271	7.74	35.26	0.1327	0.512850	5	-0.33	4.14	6.34	6.35	6.35	0.52	0.57	0.52	0.57	0.52	Tong Y et al.	2014	American Journal of Science	314(1)	80-109
3392	97-18	CAOB	Altai, unit 6	88.9083	47.0456	Bi monzogranite	Adenbluk	271	271	6.59	30.34	0.1315	0.512870	7	-0.33	4.53	6.74	6.78	6.78	0.48	0.52	0.48	0.52	0.48	Tong Y et al.	2014	American Journal of Science	314(1)	80-109
3393	190-1	CAOB	Altai, unit 5	89.4258	47.0033	Mylonitized diorite	Fuyunnan	282	282	1.02	7.24	0.0855	0.512660	11	-0.57	0.43	4.40	4.43	4.43	0.69	0.58	0.69	0.58	0.69	Tong Y et al.	2014	American Journal of Science	314(1)	80-109
3394	189-1	CAOB	Altai, unit 5	89.4258	47.0033	Mylonitized diorite	Fuyunnan	282	282	1.21	6.20	0.1176	0.512808	14	-0.40	3.32	6.20	6.16	6.16	0.54	0.54	0.54	0.54	0.54	Tong Y et al.	2014	American Journal of Science	314(1)	80-109
3395	97-2	CAOB	Altai, unit 6	86.8181	47.6044	Alkaline granite	Buerjin	268	268	4.54	23.05	0.1192	0.512630	10	-0.39	-0.16	2.42	2.49	2.49	0.83	0.84	0.83	0.84	0.83	Tong Y et al.	2014	American Journal of Science	314(1)	80-109
3396	97-1	CAOB	Altai, unit 6	86.8181	47.6044	Alkaline granite	Buerjin	268	268	5.60	24.91	0.1361	0.512856	11	-0.31	4.25	6.39	6.32	6.32	0.52	0.58	0.52	0.58	0.52	Tong Y et al.	2014	American Journal of Science	314(1)	80-109
3397	3098	CAOB	Altai, unit 6	86.8181	47.6044	Alkali feldspar granite	Buerjin	268	268	4.78	24.38	0.1256	0.512733	12	-0.36	1.85	4.32	4.28	4.28	0.69	0.72	0.69	0.72	0.68	Tong Y et al.	2014	American Journal of Science	314(1)	80-109
3398	3055	CAOB	Altai, unit 5	88.3146	47.3229	Monzogranite	Xibodu	267	267	4.16	26.82	0.0936	0.512675	6	-0.52	0.72	4.20	4.23	4.23	0.69	0.60	0.69	0.60	0.69	Tong Y et al.	2014	American Journal of Science	314(1)	80-109
3399	152	CAOB	Altai, unit 3	88.9167	47.7500	Granite	South Bridge	267	267	17.75	82.67	0.1298	0.512586	12	-0.34	-1.01	1.30	1.26	1.26	0.93	1.02	0.93	1.02	0.95	Tong Y et al.	2014	American Journal of Science	314(1)	80-109
3400	151	CAOB	Altai, unit 3	88.9167	47.7500	Granite	South Bridge	267	267	20.08	102.09	0.1189	0.512614	15	-0.40	-0.47	2.20	2.18	2.18	0.86	0.86	0.86	0.86	0.87	Tong Y et al.	2014	American Journal of Science	314(1)	80-109
3401	04XJ-282	CAOB	West Tianshan	84.3083	43.3333	Trachybasalt		312	312	5.37	23.20	0.1400	0.512727		-0.29	1.74	3.99	3.99	3.99	0.75	0.88	0.75	0.88	0.75	Sun LH et al.	2008	Gondwana Research	14(3)	383-394
3402	04XJ-274	CAOB	West Tianshan	84.3083	43.3333	Trachyandesite		312	312	9.70	39.00	0.1502	0.512743		-0.24	2.05	3.90	3.90	3.90	0.75	0.98	0.75	0.98	0.75	Sun LH et al.	2008	Gondwana Research	14(3)	383-394
3403	04XJ-268	CAOB	West Tianshan	84.3083	43.3333	Syenite		312	312	6.74	31.90	0.1277	0.512747		-0.35	2.13	4.87	4.87	4.87	0.67	0.71	0.67	0.71	0.67	Sun LH et al.	2008	Gondwana Research	14(3)	383-394
3404	04XJ-265	CAOB	West Tianshan	84.3083	43.3333	Syenite		312	312	6.57	34.00	0.1166	0.512614		-0.41	-0.47	2.72	2.72	2.72	0.85	0.84	0.85	0.84	0.85	Sun LH et al.	2008	Gondwana Research	14(3)	383-394
3405	04XJ-264	CAOB	West Tianshan	84.3083	43.3333	Syenite		312	312	7.09	30.80	0.1393	0.512791		-0.29	2.98	5.27	5.27	5.27	0.64	0.74	0.64	0.74	0.64	Sun LH et al.	2008	Gondwana Research	14(3)	383-394
3406	04XJ-262	CAOB	West Tianshan	84.3083	43.3333	Syenite		312	312	6.80	33.50	0.1228	0.512703		-0.38	1.27	4.21	4.21	4.21	0.73	0.75	0.73	0.75	0.73	Sun LH et al.	2008	Gondwana Research	14(3)	383-394
3407	BS07-329	CAOB	Beishan	99.4297	41.0314	Granites	Bandaoshan	285	285	9.06	41.8	0.1319	0.512297	14	-0.33	-6.65	-4.30	-4.30	-4.30	1.40	1.59	1.40	1.57		Zhang W et al.	2012	Journal of Asian Earth Sciences	58	51-63
3408	AB09-98	CAOB	Beishan	99.4636	41.0308	Granites	Bandaoshan	285	285	11.64	52.8	0.1266	0.512299	12	-0.36	-6.61	-3.00	-4.06	-4.06	1.38	1.49	1.38	1.49		Zhang W et al.	2012	Journal of Asian Earth Sciences	58	51-63
3409	AB09-100	CAOB	Beishan	99.4633	41.0306	Granites	Bandaoshan	285	285	9.8	46.6	0.1316	0.512331	10	-0.33	-5.99	-2.70	-3.62	-3.62	1.34	1.52	1.34	1.52		Zhang W et al.	2012	Journal of Asian Earth Sciences	58	51-63
3410	BS07-75	CAOB	Beishan	96.8833	41.0514	Granites	Yin'aoxia	282	282	2.97	14.6	0.1046	0.512534	12	-0.47	-2.03	1.30	1.28	1.28	0.94	0.86	0.94	0.86		Zhang W et al.	2012	Journal of Asian Earth Sciences	58	51-63
3411	BS07-76	CAOB	Beishan	96.8858	41.0561	Granites	Yin'aoxia	282	282	4.16	24.1	0.1118	0.512524	13	-0.43	-2.22	0.80	0.83	0.83	0.98	0.94	0.98	0.94		Zhang W et al.	2012	Journal of Asian Earth Sciences	58	51-63
3412	BS07-79	CAOB	Beishan	96.8883	41.0608	Granites	Yin'aoxia	282	282	5.06	26.3	0.1228	0.512502	15	-0.38	-2.65	0.00	0.00	0.00	1.05	1.09	1.05	1.09		Zhang W et al.	2012	Journal of Asian Earth Sciences	58	51-63
3413	OYQ-20	CAOB	Altai, unit 6	90.1911	46.3697	Alkaline granite	Hadanxun	290	290	2.85	16. 31	0.1052	0.512795	14	-0.47	3.06	6.45	6.45	6.45	0.53	0.50	0.53	0.50	0.53	Zhou G et al.	2003	Acta Geoscientica Sinica	83(3)	331-346
3414	OYQ-18	CAOB	Altai, unit 6	90.1911	46.3697	Alkaline granite	Hadanxun	290	290	8.83	44.58	0.1197	0.512825	12	-0.39	3.65	6.50	6.50	6.50	0.52	0.53	0.52	0.53	0.52	Zhou G et al.	2003	Acta Geoscientica Sinica	83(3)	331-346
3415	1024	CAOB	Olkhon terrane	107.5000	53.5500	Granodiorite		1870	600	4.95	21.42	0.1397	0.511881		-0.29	-14.77	-1.10	-10.40	-10.40	2.15	2.60	2.15	2.61	2.45	Donskaya TV et al.	2017	Gondwana Research	42	243-263
3416	14106	CAOB	Olkhon terrane	106.5333	52.8667	Diorite		500	500	0.96	6.76	0.0879	0.511905		-0.55	-14.30	-7.40	-7.36	-7.36	1.82	1.51	1.82	1.51	1.85	Donskaya TV et al.	2017	Gondwana Research	42	243-263
3417	9107	CAOB	Olkhon terrane	106.3333	52.7500	Granodiorite		495	495	2.32	14.78	0.0973	0.512512		-0.51	-2.46	3.80	3.82	3.82	0.91	0.84	0.91	0.84	0.92	Donskaya TV et al.	2017	Gondwana Research	42	243-263
3418	11125	CAOB	Olkhon terrane	106.3667	52.6500	Granite		495	495	1.85	10.56	0.1057	0.512493		-0.46	-2.83	2.90	2.92	2.92	0.98	0.93	0.98	0.93	1.00	Donskaya TV et al.	2017	Gondwana Research	42	243-263
3419	11122	CAOB	Olkhon terrane	106.3667	52.6500	Granite		495	495	2.13	13.39	0.0957	0.512518		-0.51	-2.34	4.10	4.04	4.04	0.89	0.82	0.89	0.82	0.90	Donskaya TV et al.	2017	Gondwana Research	42	243-263
3420	CG 34	NCC	NCC	118.5072	42.4178	Monzogranite	Chehugou	260	260	2.81	20.77	0.0819	0.511375	14	-0.58	-24.64	-20.8	-20.8	-20.8	2.72	2.05	2.72	2.05	2.72	Wan B et al.	2009	Economic Geology	104(3)	351-363
3421	CG 33	NCC	NCC	118.5072	42.4178	Monzogranite	Chehugou	260	260	3.48	25.34	0.0831	0.511388	11	-0.58	-24.38	-20.6	-20.6	-20.6	2.70	2.05	2.70	2.05	2.70	Wan B et al.	2009	Economic Geology	104(3)	351-363
3422	CG 32	NCC	NCC	118.5069	42.4178	Monzogranite	Chehugou	260	260	4.10	27.97	0.0886	0.511399	10	-0.55	-24.17	-20.6	-20.6	-20.6	2.70	2.13	2.70	2.13	2.70	Wan B et al.	2009	Economic Geology	104(3)	351-363
3423	CG 31	NCC	NCC	118.5069	42.4178	Monzogranite	Chehugou	260	260	2.82	20.40	0.0836	0.511382	12	-0.57	-24.50	-20.8	-20.7	-20.7	2.71	2.06	2.71	2.07	2.71	Wan B et al.	2009	Economic Geology	104(3)	351-363
3424	CG 14	NCC	NCC	118.5072	42.4181	Syenogranite	Chehugou	260	260	1.47	10.07	0.0886	0.511288	9	-0.55	-26.33	-22.8	-22.7	-22.7	2.87	2.26	2.87	2.26	2.87	Wan B et al.	2009	Economic Geology	104(3)	351-363
3425	CG 13	NCC	NCC	118.5072	42.4181	Syenogranite	Chehugou	260	260	1.55	9.23	0.1014	0.511333	12	-0.48	-25.46	-22.3	-22.3	-22.3	2.84	2.45	2.84	2.46	2.84	Wan B et al.	2009	Economic Geology	104(3)	351-363
3426	CG 12	NCC	NCC	118.5069	42.4181	Syenogranite	Chehugou	260	260	1.28	6.50	0.1189	0.511354	12	-0.40	-25.05	-22.5	-22.5	-22.5	2.85	2.87	2.85	2.87	2.85	Wan B et al.	2009	Economic Geology	104(3)	351-363
3427	CG 11	NCC	NCC	118.5069	42.4181	Syenogranite	Chehugou	260	260	2.07	15.39	0.0813	0.511269	10	-0.59	-26.71	-22.9	-22.9	-22.9	2.89	2.16	2.89	2.16	2.88	Wan B et al.	2009	Economic Geology	104(3)	351-363
3428	B65	CAOB	ogda-halrik mountain, Tiansha	88.5000	43.3000	Potash feldspar granite	Shangdahe	298	298			0.1335	0.512724		-0.32	1.68	4.08	4.08	4.08	0.73	0.81	0.73		0.73	Gu LX et al.	2000	lletin of Mineralogy, petrology and Geochemis	(01)	19-21
3429	B43	CAOB	ogda-halrik mountain, Tiansha	88.5000	43.3000	Granite	Shangdahe	298	298	5.15	25.72	0.1215	0.512697		-0.38	1.15	4.01	4.02	4.02	0.73	0.75	0.73		0.73	Gu LX et al.	2000	lletin of Mineralogy, petrology and Geochemis	(01)	19-21
3430	B149	CAOB	ogda-halrik mountain, Tiansha	88.5000	43.3000	Qtz diorite	Shangdahe	298	298			0.1200	0.512688		-0.39	0.98	3.89	3.89	3.89	0.74	0.75	0.74		0.74	Gu LX et al.	2000	lletin of Mineralogy, petrology and Geochemis	(01)	19-21
3431	40083	CAOB	Altai, unit 6	90.8911	46.1467	Alkali feldspar granite	Buergen	358	358	17.46	92.09	0.1148	0.512771	12	-0.42	2.59	6.50	6.34	6.34	0.59	0.58	0.59	0.58	0.59	Tong Y et al.	2012	International Journal of Earth Sciences	101	937-950
3432	40042	CAOB	Altai, unit 6	90.8911	46.1467	Alkali feldspar granite	Buergen	358	358	10.69	38.35	0.1688	0.512897	13	-0.14	5.05	6.46	6.33	6.33	0.59	0.86	0.59	0.86	0.59	Tong Y et al.	2012	International Journal of Earth Sciences	101	937-950

3433	4054	CAOB	Altai, unit 5	90.7011	46.2572	Bi monzogranite	North buergen	343	343	3.36	17.37	0.1171	0.512789	11	-0.40	2.95	6.60	6.43	6.43	0.57	0.57	0.57	0.57	0.57	Tong Y et al.	2012	International Journal of Earth Sciences	101	937-950
3434	0951-9	CAOB	NE China	85.0089	43.2139	Granite		934	600	5.74	34.20	0.1018	0.511898	12	-0.48	-14.44	-3.50	-7.17	-7.17	1.89	1.70	1.89		1.84	Gao J et al.	2015	Journal of Asian Earth Sciences	113(PT.1)	173-193
3435	0951-8	CAOB	NE China	85.0089	43.2139	Granite		904	600	5.11	30.80	0.1003	0.511890	15	-0.49	-14.59	-3.40	-7.21	-7.21	1.89	1.69	1.89		1.84	Gao J et al.	2015	Journal of Asian Earth Sciences	113(PT.1)	173-193
3436	WQ-12	CAOB	Altai, unit 3	89.9272	47.2094	Bi granie	Keketuohai	407	407	9.27	40.30	0.1391	0.512458	7	-0.29	-3.51	-0.52	-0.52	-0.52	1.19	1.41	1.19	1.41	1.19	Zhao ZH.	1991	lletin of Mineralogy, petrology and Geochemis	3(24)	176-178
3437	HM-4	CAOB	Altai, unit 3	89.1417	47.8292	Granite	Halon	375	375	8.22	37.84	0.1313	0.512500	6	-0.33	-2.69	0.44	0.44	0.44	1.09	1.20	1.09	1.20	1.09	Zhao ZH.	1991	lletin of Mineralogy, petrology and Geochemis	3(24)	176-178
3438	HL-4	CAOB	Altai, unit 2	87.0861	48.7783	M-c grained Bi granite	Hanasi	398	398	5.37	22.97	0.1413	0.512505	10	-0.28	-2.59	0.22	0.22	0.22	1.12	1.36	1.12	1.36	1.12	Zhao ZH.	1991	lletin of Mineralogy, petrology and Geochemis	3(24)	176-178
3439	Hb-4	CAOB	Altai, unit 4	86.4478	48.2369	Granite	Habahe	389	389	2.83	30.45	0.0562	0.512434	6	-0.71	-3.98	2.99	2.99	2.99	0.89	0.69	0.89	0.69	0.89	Zhao ZH.	1991	lletin of Mineralogy, petrology and Geochemis	3(24)	176-178
3440	Ha-4	CAOB	Altai, unit 2	87.0861	48.7783	M-c grained Bi granite	Hanasi	398	398	4.19	18.17	0.1394	0.512383	7	-0.29	-4.97	-2.06	-2.06	-2.06	1.31	1.57	1.31	1.57	1.31	Zhao ZH.	1991	lletin of Mineralogy, petrology and Geochemis	3(24)	176-178
3441	DH-5	CAOB	Altai, unit 3	89.1417	47.8292	Granite	Halon	375	375	1.97	9.36	0.1272	0.512518	52	-0.35	-2.34	0.98	0.98	0.98	1.04	1.11	1.04	1.11	1.04	Zhao ZH.	1991	lletin of Mineralogy, petrology and Geochemis	3(24)	176-178
3442	A81549	CAOB	Altai, unit 3	89.9272	47.2094	Bi-Granie	Keketuohai	407	407	6.38	26.38	0.1462	0.512425	6	-0.26	-4.15	-1.53	-1.53	-1.53	1.27	1.63	1.27	1.63	1.27	Zhao ZH.	1991	lletin of Mineralogy, petrology and Geochemis	3(24)	176-178
3443		CAOB	Altai, unit 4	87.7881	47.7614	Gneissic Bi granite	Qiemuerqieke	460	460	3.38	15.87	0.1287	0.512550		-0.35	-1.72	2.27	2.27	2.27	1.01	1.08	1.01	1.08	1.01	Zhao ZH.	1991	lletin of Mineralogy, petrology and Geochemis	3(24)	176-178
3444		CAOB	Altai, unit 4	87.7881	47.7614	Gneissic Bi granite	Qiemuerqieke	460	460	11.18	65.41	0.1040	0.512361		-0.47	-5.40	0.04	0.04	0.04	1.19	1.10	1.19	1.10	1.19	Zhao ZH.	1991	lletin of Mineralogy, petrology and Geochemis	3(24)	176-178
3445		CAOB	Altai, unit 3	89.9272	47.2094	Bi-Granie	Keketuohai	407	407	12.73	64.51	0.1192	0.512497		-0.39	-2.75	1.27	1.27	1.27	1.04	1.05	1.04	1.05	1.04	Zhao ZH.	1991	lletin of Mineralogy, petrology and Geochemis	3(24)	176-178
3446		CAOB	Altai, unit 3	90.2719	46.8875	Granodiorite	Qinggeli	407	407	4.63	22.00	0.1271	0.512481		-0.35	-3.06	0.55	0.55	0.55	1.10	1.18	1.10	1.18	1.10	Zhao ZH.	1991	lletin of Mineralogy, petrology and Geochemis	3(24)	176-178
3447		CAOB	Altai, unit 3	88.0381	48.1161	Bi granite	Wuliqitawu	375	375	9.27	40.30	0.1391	0.512458		-0.29	-3.51	-0.75	-0.75	-0.75	1.18	1.41	1.18	1.41	1.18	Zhao ZH.	1991	lletin of Mineralogy, petrology and Geochemis	3(24)	176-178
3448		CAOB	Altai, unit 3	89.9189	47.0186	Two mica granite	Haoshan	220	220	4.31	22.03	0.1183	0.512312		-0.40	-6.36	-4.16	-4.16	-4.16	1.33	1.34	1.33	1.34	1.33	Zhao ZH.	1991	lletin of Mineralogy, petrology and Geochemis	3(24)	176-178
3449		CAOB	Altai, unit 4	88.1736	47.8056	Amazonite granite	Jiangjunshan	150	150	9.67	42.88	0.1362	0.512648		-0.31	0.20	1.35	1.35	1.35	0.83	0.99	0.83	0.99	0.83	Zhao ZH.	1991	lletin of Mineralogy, petrology and Geochemis	3(24)	176-178
3450		CAOB	Altai, unit 4	88.1736	47.8056	Amazonite granite	Jiangjunshan	150	150	9.98	44.23	0.1363	0.512632		-0.31	-0.12	1.04	1.04	1.04	0.85	1.02	0.85	1.02	0.85	Zhao ZH.	1991	lletin of Mineralogy, petrology and Geochemis	3(24)	176-178
3451	BGT48*	CAOB	Western Junggar	84.4736	45.4833	Adakitic		315	315	3.21	15.75	0.1175	0.512800		-0.40	3.16	6.34	6.34	6.34	0.56	0.56	0.56	0.56	0.56	Tang GJ et al.	2010	Chemical Geology	277(3-4)	281-300
3452	BGT40*	CAOB	Western Junggar	84.4736	45.4833	Adakitic		315	315	1.35	5.02	0.1452	0.512900		-0.26	5.11	7.18	7.18	7.18	0.49	0.56	0.49	0.56	0.49	Tang GJ et al.	2010	Chemical Geology	277(3-4)	281-300
3453	BGT39*	CAOB	Western Junggar	84.4736	45.4833	Adakitic		315	315	2.56	11.88	0.1230	0.512800		-0.37	3.16	6.12	6.12	6.12	0.57	0.59	0.57	0.59	0.57	Tang GJ et al.	2010	Chemical Geology	277(3-4)	281-300
3454	BGT23*	CAOB	Western Junggar	84.4736	45.4833	Adakitic		315	315	3.29	19.70	0.0988	0.512800		-0.50	3.16	7.10	7.10	7.10	0.50	0.47	0.50	0.47	0.50	Tang GJ et al.	2010	Chemical Geology	277(3-4)	281-300
3455	06XJ-158	CAOB	Western Junggar	84.4736	45.4833	Adakitic		307	307	2.21	10.59	0.0382	0.512862		-0.81	4.38	10.58	10.58	10.58	0.32	0.25	0.20	0.25	0.20	Tang GJ et al.	2010	Chemical Geology	277(3-4)	281-300
3456	06XJ-156	CAOB	Western Junggar	84.4736	45.4833	Adakitic		307	307	1.89	9.97	0.0613	0.512828		-0.69	3.70	9.00	9.00	9.00	0.33	0.32	0.33	0.32	0.33	Tang GJ et al.	2010	Chemical Geology	277(3-4)	281-300
3457	06XJ-154	CAOB	Western Junggar	84.4736	45.4833	Adakitic		314	314	2.35	10.09	0.0325	0.512848		-0.83	4.09	10.7	10.7	10.7	0.31	0.25	0.20	0.25	0.20	Tang GJ et al.	2010	Chemical Geology	277(3-4)	281-300
3458	06XJ-153	CAOB	Western Junggar	84.4736	45.4833	Adakitic		314	314	2.62	12.28	0.0202	0.512798		-0.90	3.12	10.2	10.2	10.2	0.31	0.28	0.24	0.28	0.24	Tang GJ et al.	2010	Chemical Geology	277(3-4)	281-300
3459	06XJ-148	CAOB	Western Junggar	84.4736	45.4833	Adakitic		310	310	3.42	15.13	0.1374	0.512830		-0.30	3.74	6.08	6.08	6.08	0.57	0.64	0.57	0.64	0.57	Tang GJ et al.	2010	Chemical Geology	277(3-4)	281-300
3460	06XJ-147	CAOB	Western Junggar	84.4736	45.4833	Adakitic		310	310	3.27	15.27	0.1305	0.512824		-0.34	3.63	6.25	6.25	6.25	0.56	0.60	0.56	0.60	0.56	Tang GJ et al.	2010	Chemical Geology	277(3-4)	281-300
3461	06XJ-145	CAOB	Western Junggar	84.4736	45.4833	Adakitic		315	315	3.17	14.83	0.1301	0.512810		-0.34	3.36	6.03	6.03	6.03	0.58	0.62	0.58	0.62	0.58	Tang GJ et al.	2010	Chemical Geology	277(3-4)	281-300
3462	06XJ-143	CAOB	Western Junggar	84.4736	45.4833	Adakitic		315	315	3.21	12.07	0.1619	0.512965		-0.18	6.38	7.77	7.77	7.77	0.44	0.55	0.44	0.55	0.44	Tang GJ et al.	2010	Chemical Geology	277(3-4)	281-300
3463	06XJ-141	CAOB	Western Junggar	84.4736	45.4833	Adakitic		315	315	2.09	9.38	0.1358	0.512830		-0.31	3.75	6.20	6.20	6.20	0.57	0.63	0.57	0.63	0.57	Tang GJ et al.	2010	Chemical Geology	277(3-4)	281-300
3464	06XJ-140	CAOB	Western Junggar	84.4736	45.4833	Adakitic		315	315	2.59	10.83	0.1454	0.512873		-0.26	4.58	6.64	6.64	6.64	0.53	0.62	0.53	0.62	0.53	Tang GJ et al.	2010	Chemical Geology	277(3-4)	281-300
3465	06XJ-138-2	CAOB	Western Junggar	84.4736	45.4833	Adakitic		315	315	2.66	10.98	0.1473	0.512872		-0.25	4.56	6.54	6.54	6.54	0.54	0.64	0.54	0.64	0.54	Tang GJ et al.	2010	Chemical Geology	277(3-4)	281-300
3466	06XJ-137	CAOB	Western Junggar	84.4736	45.4833	Adakitic		315	315	2.49	9.75	0.1555	0.512978		-0.21	6.63	8.29	8.29	8.29	0.40	0.45	0.40	0.45	0.40	Tang GJ et al.	2010	Chemical Geology	277(3-4)	281-300
3467	06XJ-158	CAOB	Junggar	84.5000	45.5000	Diorite		307	307	2.21	10.59	0.0382	0.512862		-0.81	4.37	7.12	10.6	10.6	0.32	0.25	0.20		0.49	Tang GJ et al.	2010	Chemical Geology	277(3-4)	281-300
3468	06XJ-156	CAOB	Junggar	84.5000	45.5000	Diorite		307	307	1.89	9.97	0.0613	0.512828		-0.69	3.71	6.89	9.01	9.01	0.33	0.32	0.33		0.51	Tang GJ et al.	2010	Chemical Geology	277(3-4)	281-300
3469	06XJ-154	CAOB	Junggar	84.5000	45.5000	Diorite		314	314	2.35	10.09	0.0325	0.512848		-0.83	4.10	6.26	10.7	10.7	0.31	0.25	0.20		0.56	Tang GJ et al.	2010	Chemical Geology	277(3-4)	281-300
3470	06XJ-153	CAOB	Junggar	84.5000	45.5000	Diorite		314	314	2.62	12.28	0.0202	0.512798		-0.90	3.12	5.80	10.2	10.2	0.31	0.28	0.24		0.61	Tang GJ et al.	2010	Chemical Geology	277(3-4)	281-300
3471	XGG5	CAOB	Balguntay, Tianshan	88.5167	42.5167	Granite	Gangou	361	361	4.18	23.39	0.1081	0.512309	8	-0.45	-6.42	-2.34	-2.34	-2.34	1.30	1.21	1.30		1.30	Shi YR et al.	2006	Chinese Science Bulletin	51(22)	2665-2672
3472	XGG3	CAOB	Balguntay, Tianshan	88.5167	42.5167	Porphyritic granite	Gangou	361	361	5.23	30.15	0.1050	0.512152	10	-0.47	-9.48	-5.26	-5.26	-5.26	1.54	1.40	1.54		1.54	Shi YR et al.	2006	Chinese Science Bulletin	51(22)	2665-2672
3473	XGG2	CAOB	Balguntay, Tianshan	88.5167	42.5167	Granite	Gangou	368	368	2.05	9.90	0.1249	0.512277	11	-0.37	-7.04	-3.67	-3.67	-3.67	1.41	1.50	1.41		1.42	Shi YR et al.	2006	Chinese Science Bulletin	51(22)	2665-2672
3474	XGG1	CAOB	Balguntay, Tianshan	88.5167	42.5167	Augen granite	Boertuowubeiipo	428	428	10.01	48.53	0.1248	0.512357	11	-0.37	-5.48	-1.55	-1.55	-1.55	1.29	1.36	1.29		1.29	Shi YR et al.	2006	Chinese Science Bulletin	51(22)	2665-2672
3475	881053-7	CAOB	Northern margin of Yili block	81.0337	44.9277	Plagioclase amphibolite		455	455			0.1425	0.512506		-0.28	-2.57	0.58	0.57	0.57	1.14	1.38	1.14	1.38	1.15	Hu AQ et al.	2008	Acta Petrologica Sinica	24(12)	2731-2740
3476	881053-6	CAOB	Northern margin of Yili block	81.0337	44.9277	Plagioclase amphibolite		455	455			0.1192	0.512322		-0.39	-6.16	-1.66	-1.66	-1.66	1.32	1.33	1.32	1.34	1.33	Hu AQ et al.	2008	Acta Petrologica Sinica	24(12)	2731-2740
3477	881053-1	CAOB	Northern margin of Yili block	81.0337	44.9277	Plagioclase amphibolite		455	455			0.1270	0.512485		-0.35	-2.98	1.07	1.06	1.06	1.10	1.17	1.10	1.17	1.11	Hu AQ et al.	2008	Acta Petrologica Sinica	24(12)	2731-2740
3478	02xw-20a	CAOB	Northern margin of Yili block	80.4980	44.9126	Plagioclase amphibolite		451	451			0.1038	0.512337		-0.47	-5.87	-0.52	-0.53	-0.53	1.23	1.13	1.23	1.13	1.23	Hu AQ et al.	2008	Acta Petrologica Sinica	24(12)	2731-2740
3479	02xw04b	CAOB	Northern margin of Yili block	81.0337	44.9277	Plagioclase amphibolite		455	455			0.1154	0.512416		-0.41	-4.33	0.40	0.39	0.39	1.16	1.14	1.16	1.14	1.16	Hu AQ et al.	2008	Acta Petrologica Sinica	24(12)	2731-2740
3480	AHGY-46	CAOB	SMXAB	117.8493	46.1073	Adamellite	Aqinchulu	300	300	2.22	10.90	0.1230	0.512709		-0.37	1.38	4.18	4.21	4.21	0.72	0.74	0.72	0.74	0.72	Wang ZH et al.	2015	ournal of Jilin University (Earth Science Edition	45(1)	166-187
3481	AHGY-44	CAOB	SMXAB	117.8493	46.1073	Adamellite	Aqinchulu	299	299	1.94	9.82	0.1191	0.512704		-0.39	1.29	4.22	4.25	4.25	0.71	0.72	0.71	0.72	0.72	Wang ZH et al.	2015	ournal of Jilin University (Earth Science Edition	45(1)	166-187
3482	AHGY-43	CAOB	SMXAB	117.8493	46.1073	Adamellite	Aqinchulu	298	298	2.17	10.41	0.1261	0.512714		-0.36	1.48	4.16	4.17	4.17	0.72	0.76	0.72	0.76	0.72	Wang ZH et al.	2015	ournal of Jilin University (Earth Science Edition	45(1)	166-187
3483	AHGY-37	CAOB	SMXAB	117.8493	46.1073	Adamellite	Aqinchulu	297	297	1.49	7.64	0.1176	0.512702		-0.40	1.25	4.24	4.25	4.25	0.71	0.71	0.71	0.71	0.72	Wang ZH et al.	2015	ournal of Jilin University (Earth Science Edition	45(1)	166-187
3484	AHGY-36	CAOB	SMXAB	117.8493	46.1073	Adamellite	Aqinchulu	296	296	1.89	9.71	0.1177	0.512702		-0.40	1.25	4.24	4.23	4.23	0.71	0.71	0.71	0.71	0.72	Wang ZH et al.	2015	ournal of Jilin University (Earth Science Edition	45(1)	166-187
3485	A-7	NCC	NCC	110.8803	38.1203	Nepheline syenites	Zijinshan	133	150			0.1417	0.512567		-0.28	-1.38	-0.45	-0.33	-0.33	0.96	1.23	0.96	1.24	0.96	Wang YY et al.	2014	Acta Petrologica et Mineralogica	33(6)	1052-1072
3486	A-3	NCC	NCC	110.8803	38.1203	Malignite	Zijinshan	133	150			0.0848	0.512327		-0.57	-6.07	-4.17	-3.92	-3.92	1.26	0.97	1.26	0.98	1.27	Wang YY et al.	2014	Acta Petrologica et Mineralogica	33(6)	1052-1072
3487	A-16	NCC	NCC	110.8803	38.1203	Monzonite	Zijinshan	134	150			0.1163	0.511939		-0.41	-13.64	-12.3	-12.1	-12.1	1.92	1.89	1.92	1.89	1.92	Wang YY et al.	2014	Acta Petrologica et Mineralogica	33(6)	1052-1072
3488	A-11	NCC	NCC	110.8803	38.1203	Diopside syenite	Zijinshan	133	150			0.0837	0.512132		-0.57	-9.87	-7.96	-7.71	-7.71	1.57	1.19	1.57	1.20	1.58	Wang YY et al.	2014	Acta Petrologica et Mineralogica	33(6)	1052-1072
3489	XH-3	CAOB	Altai, unit 6	90.0725	46.5278	Syenite	Xileketehalasu	380	380	1.30	5.73	0.1378	0.512920	15	-0.30	5.50	8.36	8.36	8.36	0.45	0.46	0.45	0.46	0.45	Zhang ZC et al.	2006	Chinese Science Bulletin	51	952–962
3490	XH-18	CAOB	Altai, unit 6	90.0725	46.5278	orphyritic Hb monzogranit	Xileketehalasu	380	380	2.95	11.80	0.1512	0.512957	10	-0.23	6.22	8.43	8.43	8.43	0.44	0.47	0.44	0.47	0.44	Zhang ZC et al.	2006	Chinese Science Bulletin	51	952–962
3491	XH-13	CAOB	Altai, unit 6	90.0725	46.5278	Syenogranite	Xileketehalasu	380	380	1.76	8.15	0.1306	0.512844	10	-0.34	4.02	7.22	7.22	7.22	0.54	0.56	0.54	0.56	0.54	Zhang ZC et al.	2006	Chinese Science Bulletin	51	952–962
3492	C-8	CAOB	SMXAB	108.2539	43.8719	Granodiorite	Tsagaan Suvarga	366	366	1.84	12.70	0.0874	0.512674		-0.56	0.70	5.83	5.81	5.81	0.64	0.58	0.64	0.58	0.65	Hou WR et al.	2010	Acta Geoscientica Sinica	31(3)	331-342
3493	C-7	CAOB	SMXAB	108.2539	43.8719	Granodiorite	Tsagaan Suvarga	366	366	1.45	9.24	0.0948	0.512650		-0.52	0.23	5.01	4.99	4.99	0.71	0.64	0.71	0.64	0.71	Hou WR et al.	2010	Acta Geoscientica Sinica	31(3)	331-342
3494	C-2	CAOB	SMXAB	108.2539	43.8719	onzonitic granite porphyry	Tsagaan Suvarga	366	366	0.64	4.91	0.0782	0.512644		-0.60	0.12	5.67	5.65	5.65	0.65	0.57	0.65	0.57	0.66	Hou WR et al.	2010	Acta Geoscientica Sinica	31(3)	331-342
3495	C-11	CAOB	SMXAB	108.2539	43.8719	Granodiorite	Tsagaan Suvarga	366	366	1.57	10.90	0.0872	0.512725		-0.56	1.70	6.82	6.81	6.81	0.56	0.51	0.56	0.51	0.56	Hou WR et al.	2010	Acta Geoscientica Sinica	31(3)	331-342
3496	C-10	CAOB	SMXAB	108.2539	43.8719	Granodiorite	Tsagaan Suvarga	366	366	1.52	10.30	0.0894	0.512812		-0.55	3.39	8.42	8.41	8.41	0.43	0.42	0.43	0.42	0.43	Hou WR et al.	2010	Acta Geoscientica Sinica	31(3)	331-342
3497	C-1	CAOB	SMXAB	108.2539	43.8719	onzonitic granite porphyry	Tsagaan Suvarga	366	366	0.78	5.46	0.0868	0.512498		-0.56	-2.73	2.41	2.40	2.40	0.92	0.78	0.92	0.79	0.92	Hou WR et al.	2010	Acta Geoscientica Sinica	31(3)	331-342
3498	HK3	NCC	NMNCC	113.6314	42.3128	Granite-porphyry	Hadamiao	271	271	3.15	18.27	0.1045	0.512327	5	-0.47	-6.07	-2.87	-2.88	-2.88	1.27	1.15	1.27	1.15	1.27	Liu J et al.	2014	Acta Petrologica Sinica	30(1)	95-108
3499	HK1	NCC	NMNCC	113.6314	42.3131	Granite-porphyry	Hadamiao	271	271	4.73	28.57	0.1002	0.512266	5	-0.49	-7.26	-3.92	-3.92	-3.92	1.36	1.19	1.36	1.19	1.36	Liu J et al.	2014	Acta Petrologica Sinica	30(1)	95-108
3500	H4-186	NCC	NMNCC	113.6003	42.2994	Diorite	Hadamiao	267	267	4.31	23.78	0.1097	0.512539	5	-0.44	-1.93	1.03	1.03	1.03	0.95	0.90	0.95	0.90	0.95	Liu J et al.	2014	Acta Petrologica Sinica	30(1)	95-108
3501	H30-7	NCC	NMNCC	113.6314	42.3006	Diorite	Hadamiao	267	267	2.52	11.02	0.1386	0.512502	6	-0.30	-2.65	-0.67	-0.67	-0.67	1.09	1.31	1.09	1.32	1.09	Liu J et al.	2014	Acta Petrologica Sinica	30(1)	95-108
3502	H30-19	NCC	NMNCC	113.6314	42.3067	Diorite	Hadamiao	267	267	3.89	20.10	0.1171	0.512313	6	-0.40	-6.34	-3.64	-3.63	-3.63	1.33	1.32	1.33	1.32	1.33	Liu J et al.	2014	Acta Petrologica Sinica	30(1)	95-108
3503	O11Bi8	CAOB	North Tianshan	84.8567	44.0200	Plagioclase granite		325	325	0.54	1.60	0.1912	0.513055		-0.03	8.13	8.36	8.36	8.36	0.40	0.64	0.40	0.64	0.40	Xu XY et al.	2006	Acta Petrologica Sinica	(01)	83-94
3504	O11B77	CAOB	North Tianshan	84.8546	44.0166	Plagioclase granite		325	325	0.50	1.70	0.1946	0.513070		-0.01	8.43	8.52	8.51	8.51	0.39	0.64	0.39	0.64	0.39	Xu XY et al.	2006	Acta Petrologica Sinica	(01)	83-94
3505	01IBH-1	CAOB	Balguntay, Tianshan	84.8333	44.0167	Bi granite	Yizhigou	320	320	7.17	31.16	0.1392	0.512249	19	-0.29	-7.59	-5.24	-5.24	-5.24	1.50	1.84	1.50		1.50	Xu XY et al.	2006	Acta Petrologica Sinica	(01)	83-94
3506	01IBb-3	CAOB	Balguntay, Tianshan	84.8333	44.0167	Bi granite		320	320	7.42	42.01	0.1068	0.512109	10	-0.46	-10.32	-6.65	-6.65	-6.65	1.62	1.48	1.62		1.62	Xu XY et al.	2006	Acta Petrologica Sinica	(01)	83-94
3507	01IBb-262	CAOB	Balguntay, Tianshan	84.8333	44.0167	lagioclase granite porphyr	Luotuogou	352	352	7.67	29.83	0.1555	0.512894		-0.21	4.99	6.84	6.84	6.84	0.55	0.67	0.55		0.55	Xu XY et al.	2006	Acta Petrologica Sinica	(01)	83-94
3508	D98-2	CAOB	SHB	118.0600	43.2767	Granite	Shuangjingzi	233	233	33.14	20.28	0.0936	0.512342	11	-0.52	-5.77	-2.71	-2.71	-2.71	1.23	1.03	1.23	1.03	1.23	Li JY et al.	2007	Acta Petrologica Sinica	23(3)	565-582
3509	D97-1	CAOB	SHB	118.0600	43.2683	Granite	Shuangjingzi	233	233	1.05	24.77	0.0989	0.512286	10	-0.50	-6.87	-3.96	-3.96	-3.96	1.33	1.15	1.33	1.15	1.33	Li JY et al.	2007	Acta Petrologica Sinica	23(3)	565-582
3510	D106-1	CAOB	SHB	118.1683	43.3417	Granite	Shuangjingzi	233	233	1.82	10.44	0.1056	0.512229	13	-0.46	-7.98	-5.27	-5.27	-5.27	1.44	1.30	1.44	1.30	1.44	Li JY et al.	2007	Acta Petrologica Sinica	23(3)	565-582
3511	D105-4	CAOB	SHB	118.1750	43.3200	Granite	Shuangjingzi	233	233	4.85	34.81	0.0843	0.512264	11	-0.57	-7.30	-3.96	-3.95	-3.95	1.33	1.04	1.33	1.05	1.33	Li JY et al.	2007	Acta Petrologica Sinica	23(3)	565-582
3512	D103-1	CAOB	SHB	118.1617	43.3117	Granite	Shuangjingzi	233	233	2.86	18.37	0.0943	0.512288	13	-0.52	-6.83	-3.79	-3.78	-3.78	1.32	1.10	1.32	1.10	1.32	Li JY et al.	2007	Acta Petrologica Sinica	23(3)	565-582
3513	D100-1	CAOB	SHB	118.0675	43.2983	Granite	Shuangjingzi	233	233	3.08	17.54	0.1060	0.512343	12	-0.46	-5.75	-3.07	-3.06	-3.06	1.26	1.14	1.26	1.14	1.26	Li JY et al.	2007	Acta Petrologica Sinica	23(3)	565-582
3514	DQS-5	CAOB	Beishan	99.5975	41.3836	Bi granite	Dongqiyishan	511	511	0.32	0.82	0.2330	0.512540	6	0.18	-1.91	-4.28	-4.28	-4.28	1.61	-4.91	1.61		1.00	Nie FJ et al.	2002	MINERAL DEPOSITS	21(1)	10-15
3515	DQS-4	CAOB	Beishan	99.5975	41.3836	Bi granite	Dongqiyishan	511	511	1.17	2.72	0.2609	0.512634	6	0.33	-0.08	-4.26	-4.26	-4.26	1.60	-1.68	1.60		0.83	Nie FJ et al.	2002	MINERAL DEPOSITS	21(1)	10-15
3516	DQS-3	CAOB	Beishan	99.5975	41.3836	Bi granite	Dongqiyishan	511	511	0.42	1.24	0.2024	0.512438	4	0.03	-3.90	-4.27	-4.27	-4.27	1.55	9.34	1.55	9.34	1.10	Nie FJ et al.	2002	MINERAL DEPOSITS	21(1)	10-15
3517	DQS-2	CAOB	Beishan	99.5975	41.3836	Bi granite	Dongqiyishan	511	511	0.77	2.29	0.2044	0.512444	4	0.04	-3.78	-4.29	-4.29	-4.29	1.54	11.19	1.54	11.19	1.08	Nie FJ et al.	2002	MINERAL DEPOSITS	21(1)	10-15
3518	DQS-1	CAOB	Beishan	99.5975	41.3836	Bi granite	Dongqiyishan	511	511	0.43	1.88	0.1299	0.512195	6	-0.34	-8.64	-4.29	-4.29	-4.29	1.58	1.73	1.58	1.73	1.52	Nie FJ et al.	2002	MINERAL DEPOSITS	21(1)	10-15
3519	SKh-13/8	CAOB	Transbaikalia and Mongolia	102.1655	48.9852	Trachyandesite		10	150	6.57	30.92	0.1284	0.512576		-0.35	-1.21	-1.12	0.10	0.10	0.93	1.03	0.93			Savatenkov VM et al.	2010	Petrology	18(3)	278-307
3520	SKh-13/4	CAOB	Transbaikalia and Mongolia	102.0652	48.8333	Trachyandesite		10	150	6.12	27.00	0.1370	0.512587		-0.30	-0.99	-0.92	0.15	0.15	0.93	1.12	0.93			Savatenkov VM et al.	2010	Petrology	18(3)	278-307
3521	SKh-13/2	CAOB	Transbaikalia and Mongolia	102.0754	48.8895	Trachyandesite		10	150	7.30	33.28	0.1326	0.512592		-0.33	-0.90	-0.82	0.33	0.33	0.91	1.05	0.91			Savatenkov VM et al.	2010	Petrology	18(3)	278-307
3522	SKh-12/7	CAOB	Transbaikalia and Mongolia	103.1616	48.8114	Trachyandesite		10	150	8.60	40.40	0.1287	0.512501		-0.35	-2.67	-2.59	-1.37	-1.37	1.05	1.16	1.05			Savatenkov VM et al.	2010	Petrology	18(3)	278-307
3523	SKh-12/34	CAOB	Transbaikalia and Mongolia	102.6234	48.7679	Trachyandesite		10	150	2.44	8.84	0.1665	0.512625		-0.15	-0.25	-0.22	0.32	0.32	0.91	1.69	0.91			Savatenkov VM et al.	2010	Petrology	18(3)	278-307
3524	M99-26	CAOB	Transbaikalia and Mongolia	105.1333	46.9667	Granite		120	150	7.24	40.04	0.1092	0.512529		-0.44	-2.13	-0.80	-0.45	-0.45	0.98	0.91	0.98	0.91	0.98	Jahn BM et al.	2004	Journal of Asian Earth Sciences	23(5)	0-653
3525	M99-25	CAOB	Transbaikalia and Mongolia	105.0833	47.0333	Granite		120	150	5.18	35.68	0.0878	0.512517		-0.55	-2.36	-0.70	-0.28	-0.28	0.96	0.77	0.96	0.77	0.97	Jahn BM et al.	2004	Journal of Asian Earth Sciences	23(5)	0-653
3526	M99-21	CAOB	Transbaikalia and Mongolia	105.1833	47.0333	Ongonite		120	150	5.53	14.02	0.2383	0.512609		0.21	-0.57	-1.20	-1.36	-1.36	1.07	-3.40	1.07		1.03	Jahn BM et al.	2004	Journal of Asian Earth Sciences	23(5)	0-653
3527	M99-20	CAOB	Transbaikalia and Mongolia	105.1833	47.0333	Ongonite		120	150	3.40	8.06	0.2549	0.512632		0.30	-0.12	-1.00	-1.23	-1.23	1.05	-1.93	1.05		1.00	Jahn BM et al.	2004	Journal of Asian Earth Sciences	23(5)	0-653
3528	M99-19	CAOB	Transbaikalia and Mongolia	105.1833	47.0333	Ongonite		120	150	5.86	14.59	0.2427	0.512639		0.23	0.02	-0.70	-0.86	-0.86	1.02	-2.72	1.02		0.98	Jahn BM et al.	2004	Journal of Asian Earth Sciences	23(5)	0-653
3529	M99-18	CAOB	Transbaikalia and Mongolia	104.5500	47.2667	Granite		120	150	5.68	30.20	0.1137	0.512450		-0.42	-3.67	-2.40	-2.08	-2.08	1.11	1.07	1.11	1.07	1.11	Jahn BM et al.	2004	Journal of Asian Earth Sciences	23(5)	0-653
3530	M99-17	CAOB	Transbaikalia and Mongolia	102.3667	45.9000	Granodiorite		230	230	4.26	25.40	0.1014	0.512134		-0.48	-9.83	-7.00	-7.03	-7.03	1.58	1.38	1.58	1.38	1.58	Jahn BM et al.	2004	Journal of Asian Earth Sciences	23(5)	0-653

3531	M99-16	CAOB	Transbaikalia and Mongolia	101.5167	45.9333	Granite		230	230	8.33	46.02	0.1094	0.512300		-0.44	-6.59	-4.00	-4.03	-4.03	1.33	1.24	1.33	1.24	1.33	Jahn BM et al.	2004	Journal of Asian Earth Sciences	23(5)	0-653
3532	M99-15	CAOB	Transbaikalia and Mongolia	101.4167	45.9500	Granite		230	230	3.85	26.22	0.0887	0.512297		-0.55	-6.65	-3.50	-3.48	-3.48	1.29	1.04	1.29	1.04	1.29	Jahn BM et al.	2004	Journal of Asian Earth Sciences	23(5)	0-653
3533	M99-13	CAOB	Transbaikalia and Mongolia	100.2833	46.6333	Microgranite		250	250	5.97	30.46	0.1185	0.512490		-0.40	-2.89	-0.40	-0.39	-0.39	1.05	1.06	1.05	1.06	1.05	Jahn BM et al.	2004	Journal of Asian Earth Sciences	23(5)	0-653
3534	M99-12	CAOB	Transbaikalia and Mongolia	99.9167	46.7000	Pelitic sediments		600	600	5.15	25.92	0.1200	0.512179		-0.39	-8.95	-3.10	-3.08	-3.08	1.56	1.58	1.56	1.58	1.56	Jahn BM et al.	2004	Journal of Asian Earth Sciences	23(5)	0-653
3535	M99-11	CAOB	Transbaikalia and Mongolia	99.9167	46.7000	Metapelite		600	600	5.13	29.23	0.1062	0.512111		-0.46	-10.28	-3.30	-3.35	-3.35	1.58	1.47	1.58	1.47	1.58	Jahn BM et al.	2004	Journal of Asian Earth Sciences	23(5)	0-653
3536	M99-10	CAOB	Transbaikalia and Mongolia	99.9667	46.8167	Granitic dike		237	237	1.65	6.20	0.1610	0.512542		-0.18	-1.87	-0.80	-0.79	-0.79	1.07	1.75	1.07	1.75	1.07	Jahn BM et al.	2004	Journal of Asian Earth Sciences	23(5)	0-653
3537	M99-09	CAOB	Transbaikalia and Mongolia	99.9667	46.8167	Granite		250	250	3.85	21.69	0.1074	0.512469		-0.45	-3.30	-0.40	-0.45	-0.45	1.06	0.98	1.06	0.98	1.06	Jahn BM et al.	2004	Journal of Asian Earth Sciences	23(5)	0-653
3538	M99-08	CAOB	Transbaikalia and Mongolia	100.0500	46.8167	Granite		237	237	3.56	22.89	0.0941	0.512400		-0.52	-4.64	-1.50	-1.54	-1.54	1.14	0.96	1.14	0.96	1.14	Jahn BM et al.	2004	Journal of Asian Earth Sciences	23(5)	0-653
3539	M99-07	CAOB	Transbaikalia and Mongolia	100.0500	46.8167	Granite		250	250	5.31	29.98	0.1072	0.512489		-0.46	-2.91	-0.10	-0.05	-0.05	1.02	0.95	1.02	0.95	1.02	Jahn BM et al.	2004	Journal of Asian Earth Sciences	23(5)	0-653
3540	M99-06	CAOB	Transbaikalia and Mongolia	100.0500	46.8167	Granite		237	237	3.46	21.38	0.0977	0.512448		-0.50	-3.71	-0.70	-0.71	-0.71	1.07	0.92	1.07	0.92	1.07	Jahn BM et al.	2004	Journal of Asian Earth Sciences	23(5)	0-653
3541	M99-05	CAOB	Transbaikalia and Mongolia	100.0167	46.4833	Granite		250	250	3.92	23.48	0.1010	0.512430		-0.49	-4.06	-1.00	-1.00	-1.00	1.10	0.97	1.10	0.97	1.10	Jahn BM et al.	2004	Journal of Asian Earth Sciences	23(5)	0-653
3542	M99-04	CAOB	Transbaikalia and Mongolia	100.0167	46.4833	Diorite		250	250	5.04	26.73	0.1139	0.512434		-0.42	-3.98	-1.30	-1.34	-1.34	1.13	1.09	1.13	1.09	1.13	Jahn BM et al.	2004	Journal of Asian Earth Sciences	23(5)	0-653
3543	M99-03	CAOB	Transbaikalia and Mongolia	99.8500	46.4333	Granodiorite		540	540	6.15	39.66	0.0937	0.512196		-0.52	-8.62	-1.50	-1.52	-1.52	1.38	1.21	1.38	1.21	1.38	Jahn BM et al.	2004	Journal of Asian Earth Sciences	23(5)	0-653
3544	M99-02	CAOB	Transbaikalia and Mongolia	99.7500	46.3667	Granite		514	514	3.37	19.42	0.1049	0.511988		-0.47	-12.68	-6.70	-6.66	-6.66	1.78	1.62	1.78	1.62	1.78	Jahn BM et al.	2004	Journal of Asian Earth Sciences	23(5)	0-653
3545	M99-01	CAOB	Transbaikalia and Mongolia	99.7333	46.3500	Granite		514	514	3.09	18.45	0.1012	0.511952		-0.49	-13.38	-7.10	-7.12	-7.12	1.81	1.62	1.81	1.62	1.81	Jahn BM et al.	2004	Journal of Asian Earth Sciences	23(5)	0-653
3546	x-99	CAOB	East Tianshan	94.3853	41.7678	Qtz syenite		246	246	4.00	16.97	0.1385	0.512624		-0.30	-0.27	1.55	1.55	1.55	0.89	1.07	0.89	1.07	0.89	Wu CZ et al.	2010	Geochemical Journal	44(4)	285-298
3547	x-61	CAOB	East Tianshan	94.4561	41.7297	Qtz syenite		246	246	7.20	33.25	0.1378	0.512613		-0.30	-0.49	1.36	1.36	1.36	0.91	1.08	0.91	1.08	0.91	Wu CZ et al.	2010	Geochemical Journal	44(4)	285-298
3548	x-60	CAOB	East Tianshan	94.3728	41.7286	Qtz syenite		246	246	4.21	26.88	0.0961	0.512561		-0.51	-1.50	1.66	1.66	1.66	0.88	0.76	0.88	0.76	0.88	Wu CZ et al.	2010	Geochemical Journal	44(4)	285-298
3549	x-58	CAOB	East Tianshan	94.3689	41.7222	Qtz syenite		246	246	5.01	25.04	0.1237	0.512598		-0.37	-0.78	1.51	1.51	1.51	0.89	0.93	0.89	0.93	0.89	Wu CZ et al.	2010	Geochemical Journal	44(4)	285-298
3550	x-358	CAOB	East Tianshan	94.3758	41.7308	Qtz syenite		246	246	4.61	23.46	0.1204	0.512591		-0.39	-0.92	1.48	1.48	1.48	0.90	0.91	0.90	0.91	0.90	Wu CZ et al.	2010	Geochemical Journal	44(4)	285-298
3551	GLS07	CAOB	SMXAB	111.1667	43.0667	Granite	Aobaotu	138	150	4.16	26.50	0.0948	0.512299	9	-0.52	-6.61	-4.83	-4.66	-4.66	1.32	1.09	1.32	1.09	1.32	Xu DQ et al.	2008	Mineral Deposits	27(5)	543-558
3552	G7	CAOB	SMXAB	111.1667	43.0667	Bi granite	Aobaotu	138	150	1.35	5.89	0.1385	0.512490		-0.30	-2.89	-1.86	-1.77	-1.77	1.08	1.34	1.08	1.34	1.08	Xu DQ et al.	2008	Mineral Deposits	27(5)	543-558
3553	G5	CAOB	SMXAB	111.1667	43.0667	Bi granite	Aobaotu	138	150	4.60	21.90	0.1270	0.512600		-0.35	-0.74	0.48	0.59	0.59	0.89	0.97	0.89	0.97	0.89	Xu DQ et al.	2008	Mineral Deposits	27(5)	543-558
3554	G4	CAOB	SMXAB	111.1667	43.0667	Moyite	Aobaotu	138	150	3.66	20.20	0.1095	0.512590		-0.44	-0.94	0.60	0.73	0.73	0.88	0.82	0.88	0.82	0.88	Xu DQ et al.	2008	Mineral Deposits	27(5)	543-558
3555	G3	CAOB	SMXAB	111.1667	43.0667	Moyite	Aobaotu	138	150	2.51	13.70	0.1107	0.512590		-0.44	-0.94	0.58	0.71	0.71	0.88	0.83	0.88	0.83	0.88	Xu DQ et al.	2008	Mineral Deposits	27(5)	543-558
3556	G2	CAOB	SMXAB	111.1667	43.0667	Moyite	Aobaotu	138	150	2.26	9.70	0.1408	0.512620		-0.28	-0.35	0.64	0.72	0.72	0.88	1.11	0.88	1.11	0.88	Xu DQ et al.	2008	Mineral Deposits	27(5)	543-558
3557	Sm31	CAOB	SMXAB	111.1667	43.0667	Rhyolite porphyry	Aobaotu	138	150	5.37	27.30	0.1189	0.512510		-0.40	-2.50	-1.12	-1.01	-1.01	1.02	1.03	1.02	1.03	1.02	Xu DQ et al.	2008	Mineral Deposits	27(5)	543-558
3558	Sm30	CAOB	SMXAB	111.1667	43.0667	Rhyolite porphyry	Aobaotu	138	150	6.50	29.40	0.1336	0.512540		-0.32	-1.91	-0.81	-0.70	-0.70	1.00	1.16	1.00	1.16	1.00	Xu DQ et al.	2008	Mineral Deposits	27(5)	543-558
3559	Jc91–4	NCC	NCC	120.2803	41.9603	Diorite porphyry	Jinchanggouliang	227	227	5.98	31.37	0.1155	0.512193	11	-0.41	-8.68	-6.34	-6.33	-6.33	1.52	1.48	1.52	1.49	1.52	Chen D et al.	2008	Geological publishing house		
3560	Jc91–11-2	NCC	NCC	120.2803	41.9603	Diorite	Jinchanggouliang	227	227	4.95	25.92	0.1157	0.512493	9	-0.41	-2.83	-0.48	-0.48	-0.48	1.04	1.02	1.04	1.02	1.04	Chen D et al.	2008	Geological publishing house		
3561	J26–711-1–3	NCC	NCC	120.2803	41.9603	Diorite porphyry	Jinchanggouliang	227	227	5.03	31.96	0.0953	0.512302	11	-0.52	-6.55	-3.63	-3.62	-3.62	1.30	1.09	1.30	1.09	1.30	Chen D et al.	2008	Geological publishing house		
3562	J26–211-1	NCC	NCC	120.2803	41.9603	Diorite	Jinchanggouliang	227	227	3.58	17.42	0.1243	0.512586	12	-0.37	-1.01	1.08	1.08	1.08	0.91	0.96	0.91	0.96	0.92	Chen D et al.	2008	Geological publishing house		
3563	J26–13-5–2	NCC	NCC	120.2803	41.9603	Diorite porphyry	Jinchanggouliang	227	227	5.65	30.26	0.1130	0.512012	11	-0.43	-12.21	-9.79	-9.79	-9.79	1.80	1.72	1.80	1.72	1.80	Chen D et al.	2008	Geological publishing house		
3564	J13–1	NCC	NCC	120.2803	41.9603	Diorite	Jinchanggouliang	227	227	4.12	19.93	0.1251	0.512557	11	-0.36	-1.58	0.49	0.49	0.49	0.96	1.02	0.96	1.02	0.96	Chen D et al.	2008	Geological publishing house		
3565	GW05133	CAOB	SMXAB	126.7500	51.0500	Hb-granodiorite	Sandaoka	190	190	8.22	44.11	0.1126	0.512664	11	-0.43	0.51	2.55	2.55	2.55	0.76	0.73	0.76	0.74	0.77	Sui ZM and Xu XC.	2010	Geology in China	37(1)	48-55
3566	GW05129	CAOB	SMXAB	126.5833	50.8333	Bi granodiorite	Heihuashan	171	171	5.55	26.66	0.1258	0.512636	14	-0.36	-0.04	1.50	1.51	1.51	0.83	0.89	0.83	0.89	0.84	Sui ZM and Xu XC.	2010	Geology in China	37(1)	48-55
3567	GW05127	CAOB	SMXAB	126.5833	50.8333	Adamellite	Heihuashan	188	188	6.29	37.78	0.1007	0.512532	11	-0.49	-2.07	0.23	0.24	0.24	0.95	0.83	0.95	0.84	0.95	Sui ZM and Xu XC.	2010	Geology in China	37(1)	48-55
3568	GW05120	CAOB	SMXAB	126.2500	51.8667	Biotitic monzonite-granite	Jiweidianzi	176	176	5.62	35.95	0.0945	0.512215	12	-0.52	-8.25	-5.96	-5.96	-5.96	1.45	1.19	1.45	1.20	1.45	Sui ZM and Xu XC.	2010	Geology in China	37(1)	48-55
3569	GW05112	CAOB	SMXAB	126.2500	51.8667	Porphyritic adamellite	Jiweidianzi	181	181	2.68	19.98	0.0811	0.512218	0	-0.59	-8.19	-5.52	-5.52	-5.52	1.41	1.07	1.41	1.07	1.42	Sui ZM and Xu XC.	2010	Geology in China	37(1)	48-55
3570	GW05110	CAOB	SMXAB	125.3333	52.0000	Adamellite	Wudingshan	188	188	4.50	25.17	0.1080	0.512031	10	-0.45	-11.84	-9.72	-9.71	-9.71	1.76	1.61	1.76	1.61	1.76	Sui ZM and Xu XC.	2010	Geology in China	37(1)	48-55
3571	GW05101	CAOB	SMXAB	125.3333	52.0333	Hb-Qtz diorite	Hanjiayuanzi	188	188	4.92	30.08	0.0988	0.512258	10	-0.50	-7.41	-5.07	-5.06	-5.06	1.38	1.18	1.38	1.19	1.38	Sui ZM and Xu XC.	2010	Geology in China	37(1)	48-55
3572	GW05099	CAOB	SMXAB	125.3333	52.0333	Hb-Qtz diorite	Hanjiayuanzi	188	188	12.49	60.12	0.1256	0.512362	8	-0.36	-5.38	-3.69	-3.68	-3.68	1.27	1.36	1.27	1.37	1.27	Sui ZM and Xu XC.	2010	Geology in China	37(1)	48-55
3573	GW05088	CAOB	SMXAB	126.1667	52.0833	Biotitic monzonite-granite	Xinghua-Fanshentun	178	178	3.75	22.94	0.0988	0.512398	11	-0.50	-4.68	-2.46	-2.46	-2.46	1.16	1.00	1.16	1.00	1.16	Sui ZM and Xu XC.	2010	Geology in China	37(1)	48-55
3574	GW05085	CAOB	SMXAB	126.1667	52.0833	Biotitic monzonite-granite	Xinghua-Fanshentun	178	178	3.68	22.07	0.1007	0.512253	14	-0.49	-7.51	-5.33	-5.33	-5.33	1.40	1.21	1.40	1.21	1.40	Sui ZM and Xu XC.	2010	Geology in China	37(1)	48-55
3575	GW05067	CAOB	SMXAB	126.1667	52.5000	Hb-granodiorite	Zhengqicun	190	190	4.18	22.59	0.1120	0.512222	13	-0.43	-8.11	-6.06	-6.06	-6.06	1.46	1.39	1.46	1.39	1.47	Sui ZM and Xu XC.	2010	Geology in China	37(1)	48-55
3576	GW05064	CAOB	SMXAB	126.1667	52.5000	Hb-granodiorite	Zhengqicun	190	190	5.28	26.14	0.1221	0.512249	13	-0.38	-7.59	-5.79	-5.78	-5.78	1.44	1.50	1.44	1.50	1.44	Sui ZM and Xu XC.	2010	Geology in China	37(1)	48-55
3577	ZG-5	NCC	NCC	114.9492	39.4567	Leucogranite	Wang'an	140	150	1.73	15.37	0.0682	0.511436	9	-0.65	-23.45	-21.2	-21.0	-21.0	2.65	1.79	2.65	1.79	2.65	Chen B et al.	2003	Journal of the Geological Society	160(6)	963-970
3578	ZG-4	NCC	NCC	114.9492	39.4567	Monzonite	Wang'an	140	150	7.23	49.36	0.0886	0.511730	8	-0.55	-17.71	-15.8	-15.6	-15.6	2.21	1.73	2.21	1.73	2.21	Chen B et al.	2003	Journal of the Geological Society	160(6)	963-970
3579	ZG-1	NCC	NCC	114.9492	39.4567	Monzonite	Wang'an	140	150	6.76	43.27	0.0945	0.511748	8	-0.52	-17.36	-15.6	-15.4	-15.4	2.19	1.79	2.19	1.79	2.19	Chen B et al.	2003	Journal of the Geological Society	160(6)	963-970
3580	YM-9	NCC	NCC	114.9492	39.4567	Monzonite	Wang'an	140	150	5.57	36.29	0.0929	0.511789	7	-0.53	-16.56	-14.7	-14.6	-14.6	2.12	1.71	2.12	1.72	2.13	Chen B et al.	2003	Journal of the Geological Society	160(6)	963-970
3581	YM-6	NCC	NCC	114.9492	39.4567	Leucogranite	Wang'an	140	150	0.47	5.66	0.0502	0.511827	10	-0.74	-15.82	-13.2	-13.0	-13.0	2.00	1.23	2.00	1.23	2.01	Chen B et al.	2003	Journal of the Geological Society	160(6)	963-970
3582	YM-5	NCC	NCC	114.9492	39.4567	Monzonite	Wang'an	140	150	5.50	34.21	0.0973	0.511787	8	-0.51	-16.60	-14.8	-14.7	-14.7	2.13	1.78	2.13	1.78	2.14	Chen B et al.	2003	Journal of the Geological Society	160(6)	963-970
3583	YM-3	NCC	NCC	114.9492	39.4567	Qtz monzonite	Wang'an	140	150	5.76	37.64	0.0925	0.511784	8	-0.53	-16.66	-14.8	-14.7	-14.7	2.13	1.71	2.13	1.72	2.13	Chen B et al.	2003	Journal of the Geological Society	160(6)	963-970
3584	YM-1	NCC	NCC	114.9492	39.4567	Qtz monzonite	Wang'an	140	150	4.13	29.05	0.0860	0.511812	8	-0.56	-16.11	-14.1	-14.0	-14.0	2.08	1.59	2.08	1.60	2.08	Chen B et al.	2003	Journal of the Geological Society	160(6)	963-970
3585	XZ-9	NCC	NCC	114.9492	39.4567	Monzonite-diorite	Wang'an	140	150	8.71	42.42	0.1242	0.511928	10	-0.37	-13.85	-12.6	-12.5	-12.5	1.95	2.07	1.95	2.08	1.95	Chen B et al.	2003	Journal of the Geological Society	160(6)	963-970
3586	XZ-6	NCC	NCC	114.9492	39.4567	Monzonite-diorite	Wang'an	140	150	4.85	28.58	0.1027	0.511952	8	-0.48	-13.38	-11.7	-11.6	-11.6	1.88	1.64	1.88	1.64	1.88	Chen B et al.	2003	Journal of the Geological Society	160(6)	963-970
3587	WA-9	NCC	NCC	114.9492	39.4567	Monzonite-diorite	Wang'an	140	150	6.02	35.57	0.1024	0.511834	9	-0.48	-15.68	-14.0	-13.9	-13.9	2.07	1.80	2.07	1.80	2.07	Chen B et al.	2003	Journal of the Geological Society	160(6)	963-970
3588	WA-7	NCC	NCC	114.9492	39.4567	Monzonite	Wang'an	140	150	8.62	31.27	0.0893	0.511681	11	-0.55	-18.67	-16.8	-16.6	-16.6	2.29	1.80	2.29	1.80	2.29	Chen B et al.	2003	Journal of the Geological Society	160(6)	963-970
3589	WA5-1	NCC	NCC	114.9492	39.4567	Monzonite-diorite	Wang'an	140	150	6.10	35.99	0.1024	0.511783	2	-0.48	-16.68	-15.0	-14.9	-14.9	2.15	1.87	2.15	1.87	2.15	Chen B et al.	2003	Journal of the Geological Society	160(6)	963-970
3590	WA-35	NCC	NCC	114.9492	39.4567	Monzonite	Wang'an	140	150	5.27	33.91	0.0940	0.511628	7	-0.52	-19.70	-17.9	-17.7	-17.7	2.38	1.93	2.38	1.94	2.38	Chen B et al.	2003	Journal of the Geological Society	160(6)	963-970
3591	WA-33	NCC	NCC	114.9492	39.4567	Monzonite	Wang'an	140	150	3.94	26.54	0.0899	0.511740	9	-0.54	-17.52	-15.6	-15.5	-15.5	2.20	1.73	2.20	1.73	2.20	Chen B et al.	2003	Journal of the Geological Society	160(6)	963-970
3592	WA-30	NCC	NCC	114.9492	39.4567	Qtz monzonite	Wang'an	140	150	4.80	32.50	0.0893	0.511768	8	-0.55	-16.97	-15.1	-14.9	-14.9	2.15	1.69	2.15	1.69	2.16	Chen B et al.	2003	Journal of the Geological Society	160(6)	963-970
3593	WA-3	NCC	NCC	114.9492	39.4567	Monzonitedior	Wang'an	140	150	4.57	25.23	0.1096	0.511802	7	-0.44	-16.31	-14.8	-14.6	-14.6	2.13	1.97	2.13	1.97	2.13	Chen B et al.	2003	Journal of the Geological Society	160(6)	963-970
3594	WA-28	NCC	NCC	114.9492	39.4567	Leucogranite	Wang'an	140	150	5.45	37.09	0.0888	0.511694	9	-0.55	-18.41	-16.5	-16.3	-16.3	2.27	1.77	2.27	1.78	2.27	Chen B et al.	2003	Journal of the Geological Society	160(6)	963-970
3595	WA-25	NCC	NCC	114.9492	39.4567	Monzonite	Wang'an	140	150	5.78	4.05	0.0873	0.511783	9	-0.56	-16.68	-14.7	-14.6	-14.6	2.13	1.64	2.13	1.65	2.13	Chen B et al.	2003	Journal of the Geological Society	160(6)	963-970
3596	WA-23	NCC	NCC	114.9492	39.4567	Qtz monzonite	Wang'an	140	150	5.92	40.78	0.0878	0.511773	9	-0.55	-16.87	-14.9	-14.8	-14.8	2.14	1.66	2.14	1.67	2.15	Chen B et al.	2003	Journal of the Geological Society	160(6)	963-970
3597	WA-2	NCC	NCC	114.9492	39.4567	Monzonite-diorite	Wang'an	140	150	5.72	32.59	0.1061	0.511819	7	-0.46	-15.98	-14.4	-14.2	-14.2	2.10	1.88	2.10	1.88	2.10	Chen B et al.	2003	Journal of the Geological Society	160(6)	963-970
3598	WA-16	NCC	NCC	114.9492	39.4567	Qtz monzonite	Wang'an	140	150	4.56	31.67	0.0871	0.511821	7	-0.56	-15.94	-14.0	-13.8	-13.8	2.06	1.60	2.06	1.60	2.07	Chen B et al.	2003	Journal of the Geological Society	160(6)	963-970
3599	WA14	NCC	NCC	114.9492	39.4567	Qtz monzonite	Wang'an	140	150	5.08	34.07	0.0902	0.511778	8	-0.54	-16.78	-14.9	-14.7	-14.7	2.14	1.69	2.14	1.69	2.14	Chen B et al.	2003	Journal of the Geological Society	160(6)	963-970
3600	DH-9	NCC	NCC	115.1461	39.7378	Qtz monzonite	Dahenan	140	150	4.89	32.70	0.0905	0.511782	15	-0.54	-16.70	-14.8	-14.7	-14.7	2.13	1.69	2.13	1.69	2.13	Chen B et al.	2003	Journal of the Geological Society	160(6)	963-970
3601	DH-6	NCC	NCC	115.1461	39.7378	Qtz monzonite	Dahenan	140	150	4.21	27.36	0.0931	0.511774	6	-0.53	-16.85	-15.0	-14.9	-14.9	2.15	1.73	2.15	1.74	2.15	Chen B et al.	2003	Journal of the Geological Society	160(6)	963-970
3602	DH-4	NCC	NCC	115.1461	39.7378	Monzonite	Dahenan	140	150	4.40	29.45	0.0904	0.511783	8	-0.54	-16.68	-14.8	-14.6	-14.6	2.13	1.69	2.13	1.69	2.13	Chen B et al.	2003	Journal of the Geological Society	160(6)	963-970
3603	DH-33	NCC	NCC	115.1461	39.7378	Monzonite	Dahenan	140	150	6.84	42.67	0.0970	0.511810	8	-0.51	-16.15	-14.4	-14.2	-14.2	2.10	1.75	2.10	1.75	2.10	Chen B et al.	2003	Journal of the Geological Society	160(6)	963-970
3604	DH-29	NCC	NCC	115.1461	39.7378	Diorite	Dahenan	140	150	5.79	33.73	0.1038	0.511801	6	-0.47	-16.33	-14.7	-14.5	-14.5	2.12	1.87	2.12	1.87	2.12	Chen B et al.	2003	Journal of the Geological Society	160(6)	963-970
3605	DH-25	NCC	NCC	115.1461	39.7378	Leucogranite	Dahenan	140	150	4.98	29.38	0.1026	0.511752	6	-0.48	-17.28	-15.6	-15.5	-15.5	2.20	1.91	2.20	1.92	2.20	Chen B et al.	2003	Journal of the Geological Society	160(6)	963-970
3606	DH-23	NCC	NCC	115.1461	39.7378	Leucogranite	Dahenan	140	150	6.30	39.82	0.0956	0.511764	9	-0.51	-17.05	-15.3	-15.1	-15.1	2.17	1.78	2.17	1.79	2.17	Chen B et al.	2003	Journal of the Geological Society	160(6)	963-970
3607	DH-22	NCC	NCC	115.1461	39.7378	Qtz monzonite	Dahenan	140	150	3.88	26.20	0.0897	0.511730	9	-0.54	-17.71	-15.8	-15.7	-15.7	2.21	1.74	2.21	1.74	2.22	Chen B et al.	2003	Journal of the Geological Society	160(6)	963-970
3608	DH-19	NCC	NCC	115.1461	39.7378	Qtz monzonite	Dahenan	140	150	6.82	45.56	0.0906	0.511806	8	-0.54	-16.23	-14.3	-14.2	-14.2	2.09	1.66	2.09	1.66	2.10	Chen B et al.	2003	Journal of the Geological Society	160(6)	963-970
3609	DH-02	NCC	NCC	115.1461	39.7378	Monzonite	Dahenan	140	150	6.54	39.18	0.1009	0.511754	9	-0.49	-17.24	-15.5	-15.4	-15.4	2.19	1.88	2.19	1.88	2.19	Chen B et al.	2003	Journal of the Geological Society	160(6)	963-970
3610	DH-01	NCC	NCC	115.1461	39.7378	Diorite	Dahenan	140	150	4.61	31.55	0.0883	0.511748	14	-0.55	-17.36	-15.4	-15.3	-15.3	2.18	1.70	2.18	1.70	2.19	Chen B et al.	2003	Journal of the Geological Society	160(6)	963-970
3611	TD514(16312-1631.	CAOB	Southern Mongolia	106.8609	43.0458	granite		374	374	2.72	13.30	0.1246	0.512637		-0.37	-0.02	3.43	3.42	3.42	0.84	0.88	0.84			Dolgopolova A et al.	2000	Lithos	328(1)	157-182
3612	D514(1405.85-1410.	CAOB	Southern Mongolia	106.8617	43.0562	granite		367	367	1.26	4.76	0.1612	0.512929		-0.18	5.68	7.35	7.34	7.34	0.52	0.64	0.52			Dolgopolova A et al.	2000	Lithos	328(1)	157-182
3613	TD514(1289.15-1293	CAOB	Southern Mongolia	106.8622	43.0454	granite		364	364	0.54	2.49	0.1321	0.512766		-0.33	2.50	5.51	5.50	5.50	0.67	0.72	0.67			Dolgopolova A et al.	2000	Lithos	328(1)	157-182
3614	TD1218(1048-1048.	CAOB	Southern Mongolia	106.8690	43.0508	granite		361	361	2.73	12.50	0.1330	0.512715		-0.32	1.50	4.44	4.44	4.44	0.75	0.82	0.75			Dolgopolova A et al.	2000	Lithos	328(1)	157-182
3615	EGRCD0073	CAOB	Southern Mongolia	106.8999	43.1434	granite		361	361	4.27	18.20	0.1429	0.512814		-0.27	3.43	5.92	5.91	5.91	0.63	0.72	0.63			Dolgopolova A et al.	2000	Lithos	328(1)	157-182
3616	OTRCD388(504)	CAOB	Southern Mongolia	106.8617	43.0324	Qtz monzodiorite		364	364	1.72	6.22	0.1672	0.512890		-0.15	4.92	6.41	6.29	6.29	0.60	0.85	0.60	0.85	0.60	Dolgopolova A et al.	2013	Lithosphere	164-167	47-64
3617	OTD786(150)	CAOB	Southern Mongolia	106.8452	43.0075	Qtz monzodiorite		376	376	3.24	14.40	0.1360	0.512791		-0.31	2.98	6.03	5.90	5.90	0.64	0.71	0.64	0.71	0.64	Dolgopolova A et al.	2013	Lithosphere	164-167	47-64
3618	OTD514(2161.35)	CAOB	Southern Mongolia	106.8592	43.0465	Qtz monzodiorite		364	364	2.18	10.20	0.1292	0.512773		-0.34	2.63	5.90	5.77	5.77	0.64	0.68	0.64	0.68	0.64	Dolgopolova A et al.	2013	Lithosphere	164-167	47-64
3619	TD514(1631.2–1631	CAOB	Southern Mongolia	106.8609	43.0458	Bi granodiorite		374	374	2.72	13.30	0.1236	0.512637		-0.37	-0.02	3.60	3.47	3.47	0.84	0.87	0.84	0.87	0.84	Dolgopolova A et al.	2013	Lithosphere	164-167	47-64
3620	D514(1405.85–1410	CAOB	Southern Mongolia	106.8617	43.0562	Qtz monzodiorite		367	367	1.26	4.76	0.1600	0.512929		-0.19	5.68	7.52	7.39	7.39	0.51	0.63	0.51	0.63	0.51	Dolgopolova A et al.	2013	Lithosphere	164-167	47-64
3621	TD514(1289.15–1293	CAOB	Southern Mongolia	106.8622	43.0454	Qtz monzodiorite		364	364	0.54	2.49	0.1311	0.512766		-0.33	2.50	5.68	5.54	5.54	0.66	0.71	0.66	0.71	0.66	Dolgopolova A et al.	2013	Lithosphere	164-167	47-64
3622	TD514(1631.2-1631	CAOB	SMXAB	106.8608	43.0458	Bi granodiorite	Oyu Tolgoi	374	374			0.1236	0.512637		-0.37	-0.02	3.47	3.47	3.47	0.84	0.87	0.84	0.87	0.84	Dolgopolova A et al.	2013	Lithosphere	164-167	47-64
3623	OTD404(399)	CAOB	Southern Mongolia	106.8580	43.0247	Dacite		362	362	1.63	6.90	0.1428	0.512804		-0.27	3.24	5.86	5.73	5.73	0.65	0.74	0.65	0.74	0.64	Dolgopolova A et al.	2013	Lithosphere	164-167	47-64
3624	OTD343(1247)	CAOB	Southern Mongolia	106.8555	43.0295	Qtz monzodiorite		366	366	3.46	14.60	0.1433	0.512827		-0.27	3.69	6.31	6.18	6.18	0.61	0.70	0.61	0.70	0.61	Dolgopolova A et al.	2013	Lithosphere	164-167	47-64
3625	OTD258(82.5)	CAOB	Southern Mongolia	106.8533	43.0170	Qtz monzodiorite		359	359	3.04	12.80	0.1436	0.512796		-0.27	3.08	5.65	5.52	5.52	0.66	0.77	0.66	0.77	0.66	Dolgopolova A et al.	2013	Lithosphere	164-167	47-64
3626	TD1218(1048–1048.	CAOB	Southern Mongolia	106.8690	43.0508	Dacite		361	361	2.73	12.50	0.1320	0.512715		-0.33	1.50	4.61	4.48	4.48	0.75	0.81	0.75	0.81	0.74	Dolgopolova A et al.	2013	Lithosphere	164-167	47-64
3627	EGRDC066(172)	CAOB	Southern Mongolia	106.9055	43.1208	Dacite		374	374	2.94	14.10	0.1261	0.512638		-0.36	0.00	3.51	3.37	3.37	0.85	0.89	0.85	0.89	0.84	Dolgopolova A et al.	2013	Lithosphere	164-167	47-64
3628	EGRCD073(120)	CAOB	Southern Mongolia	106.8999	43.1434	Qtz monzodiorite		361	361	4.27	18.20	0.1418	0.512814		-0.28	3.43	6.09	5.96	5.96	0.63	0.71	0.63	0.71	0.62	Dolgopolova A et al.	2013	Lithosphere	164-167	47-64

3629	EGD001(75)	CAOB	Southern Mongolia	106.8365	43.0515	Granodiorite		348	348	5.08	22.30	0.1377	0.512789		-0.30	2.95	5.70	5.56	5.56	0.65	0.72	0.65	0.72	0.65	Dolgopolova A et al.	2013	Lithosphere	164-167	47-64
3630	HH5503-32	CAOB	CMEB	119.9917	48.2911	Adamellite	Honghuaerji	181	181	4.30	19.30	0.1349	0.512692	2	-0.31	1.05	2.48	2.48	2.48	0.76	0.89	0.76	0.89	0.76	Guo ZJ et al.	2014	Geology in China	41(4)	1226-1241
3631	HH5503-01	CAOB	CMEB	119.9917	48.2911	Adamellite	Honghuaerji	184	184	5.34	29.30	0.1104	0.512694	4	-0.44	1.09	3.12	3.12	3.12	0.71	0.67	0.71	0.68	0.72	Guo ZJ et al.	2014	Geology in China	41(4)	1226-1241
3632	HH4318-01	CAOB	CMEB	119.9819	48.2875	Adamellite	Honghuaerji	183	183	4.07	18.20	0.1352	0.512703	2	-0.31	1.27	2.70	2.70	2.70	0.75	0.87	0.75	0.87	0.75	Guo ZJ et al.	2014	Geology in China	41(4)	1226-1241
3633	HH4309-158	CAOB	CMEB	119.9861	48.2861	Adamellite	Honghuaerji	180	180	5.32	27.60	0.1167	0.512648	3	-0.41	0.20	2.04	2.03	2.03	0.80	0.79	0.80	0.79	0.80	Guo ZJ et al.	2014	Geology in China	41(4)	1226-1241
3634	HH4309-157	CAOB	CMEB	119.9861	48.2861	Adamellite	Honghuaerji	179	179	5.83	29.20	0.1205	0.512659	2	-0.39	0.41	2.15	2.15	2.15	0.79	0.80	0.79	0.81	0.79	Guo ZJ et al.	2014	Geology in China	41(4)	1226-1241
3635	HH4309-146	CAOB	CMEB	119.9833	48.2861	Adamellite	Honghuaerji	185	185	5.17	29.40	0.1064	0.512684	3	-0.46	0.90	3.03	3.03	3.03	0.72	0.66	0.72	0.67	0.72	Guo ZJ et al.	2014	Geology in China	41(4)	1226-1241
3636	HH2704-12	CAOB	CMEB	119.9806	48.2889	Adamellite	Honghuaerji	182	182	5.10	23.90	0.1290	0.512651	5	-0.34	0.25	1.82	1.83	1.83	0.82	0.90	0.82	0.90	0.82	Guo ZJ et al.	2014	Geology in China	41(4)	1226-1241
3637	TSKH 1/19	CAOB	Western Transbaikalia	111.1700	51.5800	Trachyte	Tsagan-Khurtei graben	209	209	7.99	42.9	0.1128	0.512616	6	-0.43	-0.43	1.80	1.81	1.82	0.84	0.81	0.84			Vorontsov A A et al.	2004	Geochemistry International	42(11)	1046-1061
3638	12-BDG-8	CAOB	NE China	119.1361	49.9861	Granite		230	230	3.80	22.40	0.1076	0.512497		-0.45	-2.75	-0.14	-0.14	-0.14	1.02	0.94	1.02	0.94		Li C F.	2015	University of Geoscience		1-106
3639	12-BDG-2	CAOB	NE China	119.1361	49.9861	Granite		230	230	3.62	19.40	0.1184	0.512538		-0.40	-1.95	0.35	0.35	0.35	0.98	0.98	0.98	0.98		Li C F.	2015	University of Geoscience		1-106
3640	12-BDG-12	CAOB	NE China	119.1361	49.9861	Granite		230	230	0.55	3.20	0.1090	0.512486		-0.45	-2.97	-0.39	-0.39	-0.39	1.04	0.97	1.04	0.97		Li C F.	2015	University of Geoscience		1-106
3641	99-9	NCC	NMNCC	113.8244	42.2228	Granite	Xianghuangqi	277	277	2.42	12.34	0.1188	0.512163	7	-0.40	-9.27	-6.51	-6.51	-6.51	1.57	1.58	1.57	1.59	1.57	Xiao QH.	2009	The Geological Publishing Hou		1-528
3642	99-8	NCC	NMNCC	113.8244	42.2228	Granite	Xianghuangqi	277	277	2.78	15.70	0.1066	0.512288	9	-0.46	-6.83	-3.64	-3.64	-3.64	1.34	1.23	1.34	1.23	1.34	Xiao QH.	2009	The Geological Publishing Hou		1-528
3643	99-7	NCC	NMNCC	113.8244	42.2228	Granite	Xianghuangqi	277	277	5.89	34.23	0.1041	0.512513	7	-0.47	-2.44	0.83	0.83	0.83	0.97	0.89	0.97	0.89	0.98	Xiao QH.	2009	The Geological Publishing Hou		1-528
3644	99-6	NCC	NMNCC	113.8244	42.2228	Granite	Xianghuangqi	277	277	4.03	20.93	0.1165	0.512229	7	-0.41	-7.98	-5.14	-5.14	-5.14	1.46	1.44	1.46	1.45	1.46	Xiao QH.	2009	The Geological Publishing Hou		1-528
3645	99-5	NCC	NMNCC	113.8244	42.2228	Granite	Xianghuangqi	262	262	3.88	20.49	0.1145	0.512224	8	-0.42	-8.08	-5.32	-5.33	-5.33	1.46	1.42	1.46	1.42	1.47	Xiao QH.	2009	The Geological Publishing Hou		1-528
3646	99-4	NCC	NMNCC	113.8244	42.2228	Granite	Xianghuangqi	262	262	4.04	20.09	0.1215	0.512237	6	-0.38	-7.82	-5.30	-5.31	-5.31	1.46	1.51	1.46	1.51	1.46	Xiao QH.	2009	The Geological Publishing Hou		1-528
3647	99-3	NCC	NMNCC	113.8244	42.2228	Granite	Xianghuangqi	261	261	4.80	22.80	0.1274	0.512160	7	-0.35	-9.32	-7.03	-7.02	-7.02	1.60	1.74	1.60	1.75	1.60	Xiao QH.	2009	The Geological Publishing Hou		1-528
3648	99-1	NCC	NMNCC	113.8244	42.2228	Granite	Xianghuangqi	261	261	2.31	10.61	0.1316	0.512228	7	-0.33	-8.00	-5.84	-5.83	-5.83	1.50	1.71	1.50	1.71	1.51	Xiao QH.	2009	The Geological Publishing Hou		1-528
3649	Z-6	CAOB	SMXAB	113.9764	44.6744	Granite	Zhanawula	277	277	6.18	30.13	0.1241	0.512563		-0.37	-1.46	1.11	1.10	1.10	0.95	1.00	0.95	1.00	0.96	Xiao QH.	2009	The Geological Publishing House		1-528
3650	Z-43	CAOB	SMXAB	113.8761	44.5083	Granite	Zuhengdaleng	284	284	5.96	21.60	0.1668	0.512915		-0.15	5.40	6.49	6.49	6.49	0.52	0.76	0.52	0.77	0.52	Xiao QH.	2009	The Geological Publishing House		1-528
3651	Z-14	CAOB	SMXAB	113.8761	44.5083	Granite	Zuhengdaleng	284	284	7.32	31.01	0.1428	0.512811		-0.27	3.37	5.34	5.33	5.33	0.61	0.73	0.61	0.73	0.62	Xiao QH.	2009	The Geological Publishing House		1-528
3652	Z-13	CAOB	SMXAB	113.8761	44.5083	Granite	Zuhengdaleng	284	284	3.67	16.81	0.1320	0.512818		-0.33	3.51	5.87	5.86	5.86	0.57	0.62	0.57	0.62	0.57	Xiao QH.	2009	The Geological Publishing House		1-528
3653	N-7	CAOB	SMXAB	114.1397	44.6353	Granite	Narenbaolige	277	277	10.24	49.23	0.1258	0.512858		-0.36	4.29	6.81	6.80	6.80	0.49	0.51	0.49	0.51	0.49	Xiao QH.	2009	The Geological Publishing House		1-528
3654	N-6	CAOB	SMXAB	114.1397	44.6353	Granite	Narenbaolige	277	277	9.03	38.20	0.1429	0.512779		-0.27	2.75	4.66	4.65	4.65	0.66	0.80	0.66	0.80	0.67	Xiao QH.	2009	The Geological Publishing House		1-528
3655	N-5	CAOB	SMXAB	114.1397	44.6353	Granite	Narenbaolige	277	277	8.60	42.06	0.1236	0.512896		-0.37	5.03	7.63	7.62	7.62	0.42	0.43	0.42	0.43	0.42	Xiao QH.	2009	The Geological Publishing House		1-528
3656	N-3	CAOB	SMXAB	114.1397	44.6353	Granite	Narenbaolige	277	277	8.34	41.89	0.1204	0.512860		-0.39	4.33	7.04	7.03	7.03	0.47	0.47	0.47	0.48	0.47	Xiao QH.	2009	The Geological Publishing House		1-528
3657	L-6	CAOB	SMXAB	113.9764	44.6744	Granite	Zhanawula	277	277	3.58	15.80	0.1370	0.512773		-0.30	2.63	4.76	4.74	4.74	0.66	0.75	0.66	0.75	0.66	Xiao QH.	2009	The Geological Publishing House		1-528
3658	L-4	CAOB	SMXAB	113.9764	44.6744	Granite	Zhanawula	277	277	12.20	59.17	0.1247	0.512824		-0.37	3.63	6.18	6.17	6.17	0.54	0.56	0.54	0.56	0.54	Xiao QH.	2009	The Geological Publishing House		1-528
3659	L-17	CAOB	SMXAB	113.9764	44.6744	Granite	Zhanawula	277	277	9.97	41.36	0.1458	0.512842		-0.26	3.98	5.79	5.78	5.78	0.57	0.69	0.57	0.70	0.57	Xiao QH.	2009	The Geological Publishing House		1-528
3660	H-1	CAOB	SMXAB	116.3836	45.3811	Granite	Shaerhada	277	277	17.00	83.39	0.1233	0.512760		-0.37	2.38	4.97	4.97	4.97	0.64	0.66	0.64	0.66	0.64	Xiao QH.	2009	The Geological Publishing House		1-528
3661	B-3	CAOB	SMXAB	113.8567	44.3592	Granite	Baiyinwula	286	286	6.46	25.35	0.1541	0.512853		-0.22	4.19	5.74	5.75	5.75	0.58	0.76	0.58	0.76	0.58	Xiao QH.	2009	The Geological Publishing House		1-528
3662	B-19	CAOB	SMXAB	113.8567	44.3592	Granite	Baiyinwula	286	286	7.96	31.99	0.1506	0.512824		-0.23	3.63	5.31	5.31	5.31	0.62	0.79	0.62	0.79	0.62	Xiao QH.	2009	The Geological Publishing House		1-528
3663	99-31	CAOB	SMXAB	114.1272	43.9739	Granite	Sunitezuoqi	252	252	6.01	21.23	0.1712	0.512520	7	-0.13	-2.30	-1.47	-1.48	-1.48	1.14	2.25	1.14	2.26	1.15	Xiao QH.	2009	The Geological Publishing House		1-528
3664	99-30	CAOB	SMXAB	114.1272	43.9739	Granite	Sunitezuoqi	252	252	2.38	12.30	0.1172	0.512384	11	-0.40	-4.95	-2.39	-2.40	-2.40	1.22	1.21	1.22	1.21	1.22	Xiao QH.	2009	The Geological Publishing House		1-528
3665	99-29	CAOB	SMXAB	114.1272	43.9739	Granite	Sunitezuoqi	252	252	2.85	11.36	0.1517	0.512478	9	-0.23	-3.12	-1.67	-1.67	-1.67	1.16	1.65	1.16	1.65	1.16	Xiao QH.	2009	The Geological Publishing House		1-528
3666	99-24	CAOB	SMXAB	113.9061	43.8711	Granite	Sunitezuoqi	216	216	15.87	109.10	0.0879	0.512570	9	-0.55	-1.33	1.68	1.67	1.67	0.86	0.70	0.86	0.71	0.86	Xiao QH.	2009	The Geological Publishing House		1-528
3667	99-23A	CAOB	SMXAB	113.9061	43.8711	Granite	Sunitezuoqi	216	216	2.87	5.32	0.3257	0.512783	10	0.66	2.83	-0.72	-0.73	-0.73	1.06	-0.50	1.06		1.06	Xiao QH.	2009	The Geological Publishing House		1-528
3668	99-23	CAOB	SMXAB	113.9061	43.8711	Granite	Sunitezuoqi	216	216	6.55	23.76	0.1668	0.512690	8	-0.15	1.01	1.84	1.84	1.84	0.84	1.49	0.84	1.50	0.85	Xiao QH.	2009	The Geological Publishing House		1-528
3669	99-22	CAOB	SMXAB	113.9061	43.8711	Granite	Sunitezuoqi	216	216	4.19	19.15	0.1322	0.512485	8	-0.33	-2.98	-1.21	-1.21	-1.21	1.09	1.24	1.09	1.25	1.09	Xiao QH.	2009	The Geological Publishing House		1-528
3670	99-21	CAOB	SMXAB	113.9061	43.8711	Granite	Sunitezuoqi	216	216	3.96	20.69	0.1157	0.512376	7	-0.41	-5.11	-2.89	-2.89	-2.88	1.23	1.20	1.23	1.21	1.23	Xiao QH.	2009	The Geological Publishing House		1-528
3671	99-20	CAOB	SMXAB	113.9061	43.8711	Granite	Sunitezuoqi	216	216	10.63	56.65	0.1135	0.512513	8	-0.42	-2.44	-0.14	-0.15	-0.15	1.00	0.97	1.00	0.97	1.01	Xiao QH.	2009	The Geological Publishing House		1-528
3672	99-19	CAOB	SMXAB	113.7717	43.7869	Granite	Sunitezuoqi	152	152	11.30	43.96	0.1554	0.512701	6	-0.21	1.23	2.02	2.03	2.03	0.77	1.17	0.77	1.18	0.78	Xiao QH.	2009	The Geological Publishing House		1-528
3673	99-18	CAOB	SMXAB	113.9061	43.8711	Granite	Sunitezuoqi	216	216	1.03	3.55	0.1748	0.512657	10	-0.11	0.37	0.98	0.97	0.97	0.91	1.93	0.91	1.93	0.92	Xiao QH.	2009	The Geological Publishing House		1-528
3674	XIMZ143	CAOB	Beishan	96.7986	41.8572	Granite		395	395	5.18	28.60	0.1103	0.512245		-0.44	-7.67	-3.31	-3.31	-3.31	1.41	1.33	1.41			Wang XY et al.	2018	Journal of Asian Earth Sciences	153	206-222
3675	XIMZ136	CAOB	Beishan	96.7986	41.8572	Granite		409	409	5.79	29.90	0.1179	0.512193		-0.40	-8.68	-4.57	-4.57	-4.57	1.52	1.52	1.52			Wang XY et al.	2018	Journal of Asian Earth Sciences	153	206-222
3676	XIMZ135	CAOB	Beishan	96.7986	41.8572	Granite		409	409	6.25	31.20	0.1220	0.512171		-0.38	-9.11	-5.21	-5.21	-5.21	1.57	1.62	1.57			Wang XY et al.	2018	Journal of Asian Earth Sciences	153	206-222
3677	12WLT-8.1	NCC	WNCC	107.4576	41.5135	Granite		233	150	8.99	44.70	0.1216	0.512317	0	-0.38	-6.26	-6.11	-4.82	-4.82	1.33	1.38	1.33	1.38	1.32	Wang ZZ et al.	2016	Lithos	261	278-295
3678	12WLT-18.2	NCC	WNCC	106.2480	40.7304	cogranite (host of amphibolite)		914	600	0.83	3.70	0.1356	0.512453	0	-0.31	-3.61	3.62	1.07	1.07	1.22	1.36	1.22	1.36	1.27	Wang ZZ et al.	2016	Lithos	261	278-295
3679	12WLT-18.1	NCC	WNCC	106.2480	40.7304	cogranite (host of amphibolite)		914	600	1.13	3.60	0.1898	0.512394	0	-0.04	-4.76	-3.88	-4.23	-4.23	1.64	4.76	1.64	4.76	1.84	Wang ZZ et al.	2016	Lithos	261	278-295
3680	ZK1501-480	CAOB	Western Junggar	83.1222	45.8750	Pyroxene diorite		312	312	1.53	5.99	0.1476	0.512906	0	-0.25	5.23	7.10	7.18	7.18	0.49	0.56	0.49		0.49	Wu C et al.	2018	Journal of Asian Earth Sciences	160	48-66
3681	ZK1501-270	CAOB	Western Junggar	83.1222	45.8750	Pyroxene diorite		312	312	2.93	13.10	0.1434	0.512839	0	-0.27	3.92	6.00	6.04	6.04	0.58	0.67	0.58		0.58	Wu C et al.	2018	Journal of Asian Earth Sciences	160	48-66
3682	XM2-41	CAOB	Western Junggar	85.6083	46.6583	Alkali feldspar granite		422	422	7.09	30.20	0.1418	0.512300		-0.28	-6.59	4.68	-3.64	-3.64	1.45	1.80	1.45	0.90	0.73	Wu C et al.	2018	Journal of Asian Earth Sciences	160	48-66
3683	14SW-05	CAOB	Western Junggar	83.1222	45.8750	Pyroxene diorite		312	312	3.01	11.20	0.1608	0.512937	0	-0.18	5.83	7.30	7.26	7.26	0.48	0.61	0.48		0.48	Wu C et al.	2018	Journal of Asian Earth Sciences	160	48-66
3684	XDG-4	CAOB	Altai, unit 3	88.2345	47.9833	Granite	Xiaodonggou	409	409	9.98	45.60	0.1400	0.512451	12	-0.29	-3.65	-0.60	-0.69	-0.69	1.21	1.44	1.21	1.42	1.20	Zheng JH et al.	2016	International Geology Review	58(5)	540-555
3685	XDG-31	CAOB	Altai, unit 3	88.2345	47.9833	Porphyritic granite	Xiaodonggou	400	400	6.79	29.90	0.1400	0.512455	10	-0.29	-3.57	-0.90	-0.68	-0.68	1.20	1.44	1.20	1.52	1.21	Zheng JH et al.	2016	International Geology Review	58(5)	540-555
3686	XDG-30	CAOB	Altai, unit 3	88.2345	47.9833	Porphyritic granite	Xiaodonggou	400	400	7.22	31.90	0.1400	0.512468	9	-0.29	-3.32	-0.60	-0.42	-0.42	1.18	1.41	1.18	1.48	1.19	Zheng JH et al.	2016	International Geology Review	58(5)	540-555
3687	XDG-27	CAOB	Altai, unit 3	88.2345	47.9833	Porphyritic granite	Xiaodonggou	400	400	8.15	36.10	0.1400	0.512467	6	-0.29	-3.34	-0.60	-0.44	-0.44	1.18	1.41	1.18	1.48	1.19	Zheng JH et al.	2016	International Geology Review	58(5)	540-555
3688	XDG-25	CAOB	Altai, unit 3	88.2345	47.9833	Porphyritic granite	Xiaodonggou	400	400	6.09	26.60	0.1500	0.512432	4	-0.24	-4.02	-1.40	-1.63	-1.63	1.27	1.71	1.27	1.60	1.26	Zheng JH et al.	2016	International Geology Review	58(5)	540-555
3689	XDG-11	CAOB	Altai, unit 3	88.2345	47.9833	Granite	Xiaodonggou	409	409	7.67	35.80	0.1400	0.512475	22	-0.29	-3.18	0.00	-0.22	-0.22	1.17	1.39	1.17	1.32	1.15	Zheng JH et al.	2016	International Geology Review	58(5)	540-555
3690	XDG-10	CAOB	Altai, unit 3	88.2345	47.9833	Granite	Xiaodonggou	409	409	8.30	39.50	0.1300	0.512447	9	-0.34	-3.73	-0.40	-0.24	-0.24	1.17	1.28	1.17	1.33	1.18	Zheng JH et al.	2016	International Geology Review	58(5)	540-555
3691	622-6A	CAOB	Western Siberian margin	93.0500	60.1333	Granite		875	600	3.13	18.90	0.1002	0.511825		-0.49	-15.86	-5.10	-8.47	-8.47	1.99	1.77	1.99	1.78	1.77	Vernikovsky VA et al.	2007	Precambrian Research	154（3-4）	175-191
3692	01-21	CAOB	Western Siberian margin	92.3000	60.1000	Granite		865	600	7.62	36.20	0.1273	0.512106		-0.35	-10.38	-2.70	-5.06	-5.06	1.72	1.84	1.72	1.84	1.77	Vernikovsky VA et al.	2007	Precambrian Research	154（3-4）	175-191
3693	01-12	CAOB	Western Siberian margin	92.2500	60.0833	Granite		868	600	7.32	38.38	0.1153	0.512036		-0.41	-11.74	-2.70	-5.51	-5.51	1.75	1.72	1.75	1.73	1.77	Vernikovsky VA et al.	2007	Precambrian Research	154（3-4）	175-191
3694	01-10	CAOB	Western Siberian margin	92.1333	60.1333	Granite		865	600	16.45	90.48	0.1099	0.512003		-0.44	-12.39	-2.80	-5.74	-5.74	1.77	1.68	1.77	1.68	1.97	Vernikovsky VA et al.	2007	Precambrian Research	154（3-4）	175-191
3695	YD205-2	CAOB	East Tianshan	92.2500	42.1000	Qtz porphyry		290	290	7.71	38.40	0.1216	0.512769		-0.38	2.56	5.33	5.33	5.33	0.62	0.63	0.62	0.63	0.62	Zhang L et al.	2006	Mineralium Deposita	41	188-200
3696	YD205-1	CAOB	East Tianshan	92.2500	42.1000	Qtz porphyry		290	290	7.58	37.74	0.1216	0.512779		-0.38	2.75	5.53	5.53	5.53	0.60	0.61	0.60	0.61	0.60	Zhang L et al.	2006	Mineralium Deposita	41	188-200
3697	YD203	CAOB	East Tianshan	92.2500	42.1000	Plagiogranite		330	330	2.51	11.27	0.1347	0.512757		-0.32	2.32	4.93	4.93	4.93	0.68	0.76	0.68	0.76	0.68	Zhang L et al.	2006	Mineralium Deposita	41	188-200
3698	TW219	CAOB	East Tianshan	92.2500	42.1000	Plagiogranite		330	330	4.16	19.89	0.1266	0.512872		-0.36	4.56	7.52	7.52	7.52	0.47	0.49	0.47	0.49	0.47	Zhang L et al.	2006	Mineralium Deposita	41	188-200
3699	TW208	CAOB	East Tianshan	92.2500	42.1000	Plagiogranite		330	330	1.77	9.82	0.1092	0.512744		-0.44	2.07	5.75	5.75	5.75	0.62	0.59	0.62	0.59	0.62	Zhang L et al.	2006	Mineralium Deposita	41	188-200
3700	TW204	CAOB	East Tianshan	92.2500	42.1000	Plagiogranite		330	330	2.43	11.55	0.1275	0.512824		-0.35	3.63	6.54	6.54	6.54	0.55	0.58	0.55	0.58	0.55	Zhang L et al.	2006	Mineralium Deposita	41	188-200
3701	TW203	CAOB	East Tianshan	92.2500	42.1000	Plagiogranite		330	330	1.12	6.18	0.1093	0.512773		-0.44	2.63	6.31	6.31	6.31	0.57	0.55	0.57	0.55	0.57	Zhang L et al.	2006	Mineralium Deposita	41	188-200
3702	TW201	CAOB	East Tianshan	92.2500	42.1000	Andesite		330	330	4.21	16.60	0.1535	0.512859		-0.22	4.31	6.13	6.13	6.13	0.59	0.74	0.59	0.74	0.59	Zhang L et al.	2006	Mineralium Deposita	41	188-200
3703	R-07a	CAOB	East Tianshan	92.2500	42.1000	Plagiogranite		330	330			0.1038	0.512730		-0.47	1.79	5.71	5.71	5.71	0.62	0.58	0.62	0.58	0.62	Zhang L et al.	2006	Mineralium Deposita	41	188-200
3704	R-06a	CAOB	East Tianshan	92.2500	42.1000	Plagiogranite		330	330			0.1012	0.512767		-0.49	2.52	6.54	6.54	6.54	0.55	0.52	0.55	0.52	0.55	Zhang L et al.	2006	Mineralium Deposita	41	188-200
3705	R-05a	CAOB	East Tianshan	92.2500	42.1000	Plagiogranite		330	330			0.1368	0.512421		-0.30	-4.23	-1.71	-1.71	-1.71	1.22	1.44	1.22	1.44	1.22	Zhang L et al.	2006	Mineralium Deposita	41	188-200
3706	R-04a	CAOB	East Tianshan	92.2500	42.1000	Plagiogranite		330	330			0.1175	0.512870		-0.40	4.53	7.86	7.86	7.86	0.45	0.44	0.45	0.44	0.45	Zhang L et al.	2006	Mineralium Deposita	41	188-200
3707	R-03a	CAOB	East Tianshan	92.2500	42.1000	Plagiogranite		330	330			0.1119	0.512812		-0.43	3.39	6.97	6.97	6.97	0.52	0.51	0.52	0.51	0.52	Zhang L et al.	2006	Mineralium Deposita	41	188-200
3708	R-02a	CAOB	East Tianshan	92.2500	42.1000	Plagiogranite		330	330			0.1126	0.512806		-0.43	3.28	6.82	6.82	6.82	0.53	0.52	0.53	0.52	0.53	Zhang L et al.	2006	Mineralium Deposita	41	188-200
3709	R-01a	CAOB	East Tianshan	92.2500	42.1000	Plagiogranite		330	330			0.1235	0.512944		-0.37	5.97	9.05	9.05	9.05	0.35	0.35	0.35	0.35	0.35	Zhang L et al.	2006	Mineralium Deposita	41	188-200
3710	1770	CAOB	Kuznetsk Alatau	89.4500	51.8500	Monzonite		483	483	3.13	21.02	0.0901	0.512568		-0.54	-1.37	5.45	5.21	5.21	0.79	0.72	0.79	0.72		Vrublevskii VV et al.	2018	Russian Geology and Geophysics	59（7）	718-744
3711	1764	CAOB	Kuznetsk Alatau	89.4500	51.8500	Monzonite		483	483	6.27	36.04	0.1052	0.512586		-0.47	-1.01	4.82	4.63	4.63	0.83	0.79	0.83	0.80		Vrublevskii VV et al.	2018	Russian Geology and Geophysics	59（7）	718-744
3712	1714	CAOB	Kuznetsk Alatau	89.8200	54.2500	Monzodiorite		500	500	6.21	38.78	0.0968	0.512513		-0.51	-2.44	3.95	3.93	3.93	0.90	0.83	0.90	0.83		Vrublevskii VV et al.	2018	Russian Geology and Geophysics	59（7）	718-744
3713	1702	CAOB	Kuznetsk Alatau	89.8200	54.2500	Monzodiorite		500	500	7.88	46.93	0.1015	0.512511		-0.48	-2.48	3.61	3.60	3.60	0.93	0.87	0.93	0.87		Vrublevskii VV et al.	2018	Russian Geology and Geophysics	59（7）	718-744
3714	N0741-1	NCC	NCC	110.5000	41.0000	Hb syenite	Gaojiacun	399	399	2.49	14.10	0.1070	0.511640	11	-0.46	-19.47	-14.9	-14.9	-14.9	2.35	2.15	2.35	2.15	2.35	Wang HC et al.	2012	Acta Petrologica Sinica	28（9）	2843-2854
3715	N0741	NCC	NCC	110.5000	41.0000	Hb syenite	Gaojiacun	399	399	3.62	20.80	0.1052	0.511664	20	-0.47	-19.00	-14.4	-14.3	-14.3	2.31	2.08	2.31	2.08	2.31	Wang HC et al.	2012	Acta Petrologica Sinica	28（9）	2843-2854
3716	HG9	NCC	NCC	120.3086	41.9525	Granitoids	Duimiangou	128	150	4.34	26.83	0.0978	0.512115	2	-0.50	-10.20	-8.59	-8.31	-8.31	1.62	1.36	1.62	1.36	1.62	Fu LB et al.	2012	Lithos	134-135(none)	273-288
3717	HG5	NCC	NCC	120.2983	41.9528	Granitoids	Duimiangou	128	150	4.10	26.82	0.0925	0.512119	2	-0.53	-10.12	-8.42	-8.13	-8.13	1.60	1.30	1.60	1.30	1.61	Fu LB et al.	2012	Lithos	134-135(none)	273-288
3718	HG4	NCC	NCC	120.2964	41.9619	Granitoids	Duimiangou	128	150	4.22	26.83	0.0952	0.512117	2	-0.52	-10.16	-8.51	-8.22	-8.22	1.61	1.33	1.61	1.33	1.62	Fu LB et al.	2012	Lithos	134-135(none)	273-288
3719	HG11	NCC	NCC	120.3122	41.9453	Granitoids	Duimiangou	128	150	4.29	27.01	0.0961	0.512118	2	-0.51	-10.14	-8.49	-8.22	-8.22	1.61	1.34	1.61	1.34	1.61	Fu LB et al.	2012	Lithos	134-135(none)	273-288
3720	HG10	NCC	NCC	120.3131	41.9500	Granitoids	Duimiangou	128	150	4.43	27.38	0.0980	0.512120	2	-0.50	-10.10	-8.49	-8.22	-8.22	1.61	1.36	1.61	1.36	1.61	Fu LB et al.	2012	Lithos	134-135(none)	273-288
3721	BHG9	NCC	NCC	120.3061	41.9553	Granitoids	Duimiangou	128	150	2.95	18.24	0.0980	0.512161	3	-0.50	-9.30	-7.69	-7.42	-7.42	1.54	1.30	1.54	1.30	1.55	Fu LB et al.	2012	Lithos	134-135(none)	273-288
3722	BHG8	NCC	NCC	120.3008	41.9558	Granitoids	Duimiangou	128	150	3.00	18.50	0.0983	0.512176	2	-0.50	-9.01	-7.40	-7.13	-7.13	1.52	1.29	1.52	1.29	1.53	Fu LB et al.	2012	Lithos	134-135(none)	273-288

3723	BHG5	NCC	NCC	120.3069	41.9578	Granitoids	Duimiangou	128	150	2.83	17.18	0.0996	0.512155	3	-0.49	-9.42	-7.83	-7.56	-7.56	1.55	1.33	1.55	1.33	1.56	Fu LB et al.	2012	Lithos	134-135(none)	273-288
3724	BHG4	NCC	NCC	120.3025	41.9589	Granitoids	Duimiangou	128	150	3.02	19.04	0.0961	0.512152	3	-0.51	-9.48	-7.83	-7.55	-7.55	1.55	1.29	1.55	1.30	1.56	Fu LB et al.	2012	Lithos	134-135(none)	273-288
3725	BHG3	NCC	NCC	120.2992	41.9594	Granitoids	Duimiangou	128	150	2.93	18.11	0.0979	0.512154	3	-0.50	-9.44	-7.83	-7.55	-7.55	1.55	1.31	1.55	1.31	1.56	Fu LB et al.	2012	Lithos	134-135(none)	273-288
3726	SJ39	CAOB	East Junggar	90.4583	45.0528	Alkaline granite	Sujiquan	305	305	7.66	38.95	0.1189	0.512785	5	-0.40	2.87	5.90	5.90	5.90	0.58	0.59	0.58		0.59	Su YP et al.	2006	Acta Petrologica et Mineralogica	25(3)	175-184
3727	SJ37	CAOB	East Junggar	90.4472	45.0417	Alkaline granite	Sujiquan	305	305	10.19	34.40	0.1765	0.512905	5	-0.10	5.21	6.00	5.99	5.99	0.58	1.00	0.58		0.58	Su YP et al.	2006	Acta Petrologica et Mineralogica	25(3)	175-184
3728	SJ15	CAOB	East Junggar	90.3667	45.0000	Alkaline granite	Sujiquan	305	305	10.44	39.87	0.1583	0.512884	5	-0.20	4.80	6.30	6.29	6.29	0.55	0.73	0.55		0.55	Su YP et al.	2006	Acta Petrologica et Mineralogica	25(3)	175-184
3729	09HL12-5	CAOB	EasternTianshan	92.2186	41.5573	Neutral volcanic rock		356	356	5.50	27.30	0.1220	0.512238	0	-0.38	-7.80	-4.41	-4.41	-4.41	1.47	1.51	1.47	1.52	1.47	Mao QG et al.	2014	Acta Geologica Sinica	88(10)	1790-1799
3730	09HL11-3	CAOB	EasternTianshan	92.2186	41.5573	Neutral volcanic rock		356	356	5.28	27.40	0.1166	0.512160	0	-0.41	-9.32	-5.69	-5.69	-5.69	1.57	1.55	1.57	1.55	1.57	Mao QG et al.	2014	Acta Geologica Sinica	88(10)	1790-1799
3731	GY14-36	NCC	NCC	109.6709	41.4421	Rhyolite		123	150	13.10	85.50	0.0933	0.511956		-0.53	-13.30	-11.7	-11.3	-11.3	1.86	1.51	1.86			Guo PY et al.	2018	Journal of Asian Earth Sciences	160	348-364
3732	GY14-35	NCC	NCC	109.6709	41.4421	Rhyolite		123	150	9.80	61.40	0.0972	0.511962		-0.51	-13.19	-11.6	-11.3	-11.3	1.86	1.55	1.86			Guo PY et al.	2018	Journal of Asian Earth Sciences	160	348-364
3733	GY14-34	NCC	NCC	109.6709	41.4421	Rhyolite		123	150	12.60	80.70	0.0951	0.511960		-0.52	-13.23	-11.6	-11.3	-11.3	1.86	1.53	1.86			Guo PY et al.	2018	Journal of Asian Earth Sciences	160	348-364
3734	GY14-32	NCC	NCC	109.7330	41.4568	Rhyolite		123	150	14.70	112.00	0.0799	0.511951		-0.59	-13.40	-11.6	-11.2	-11.2	1.85	1.36	1.85			Guo PY et al.	2018	Journal of Asian Earth Sciences	160	348-364
3735	GY14-30	NCC	NCC	109.7279	41.4409	Rhyolite		123	150	9.90	54.70	0.1102	0.511982		-0.44	-12.80	-11.4	-11.1	-11.1	1.84	1.72	1.84			Guo PY et al.	2018	Journal of Asian Earth Sciences	160	348-364
3736	GY14-12	NCC	NCC	109.6844	41.2121	Rhyolite		123	150	6.95	21.80	0.1942	0.512024		-0.01	-11.98	-11.9	-11.9	-11.9	1.86	8.57	1.86			Guo PY et al.	2018	Journal of Asian Earth Sciences	160	348-364
3737	GY14-03	NCC	NCC	109.9755	41.4314	Rhyolite		123	150	4.37	29.50	0.0902	0.511751		-0.54	-17.30	-15.6	-15.3	-15.3	2.18	1.72	2.18			Guo PY et al.	2018	Journal of Asian Earth Sciences	160	348-364
3738	GY14-02	NCC	NCC	109.9755	41.4314	Rhyolite		123	150	4.22	28.50	0.0902	0.511761		-0.54	-17.11	-15.4	-15.1	-15.1	2.16	1.71	2.16			Guo PY et al.	2018	Journal of Asian Earth Sciences	160	348-364
3739	GY14-02	NCC	NCC	109.9755	41.4314	Rhyolite		123	150	4.14	28.00	0.0901	0.512011		-0.54	-12.23	-10.6	-10.2	-10.2	1.77	1.40	1.77			Guo PY et al.	2018	Journal of Asian Earth Sciences	160	348-364
3740	zk9235–447	CAOB	Western Junggar	82.4750	46.7697	Medium-grained granite		297	297	2.96	13.50	0.1325	0.512819	10	-0.33	3.53	6.00	5.96	5.96	0.57	0.62	0.57		0.62	Shen P et al.	2017	Ore Geology Reviews	81(Pt.2)	808-828
3741	ZK0003-221*	CAOB	Western Junggar	82.4750	46.7697	Monzonitic granite porphyry	Suyunhe	297	297	2.48	11.70	0.1280	0.512763	6	-0.35	2.44	5.00	5.04	5.04	0.65	0.69	0.65		0.69	Shen P et al.	2017	Ore Geology Reviews	81(Pt.2)	808-828
3742	ZK0003-215*	CAOB	Western Junggar	82.4750	46.7697	Monzonitic granite porphyry	Suyunhe	297	297	2.66	12.00	0.1345	0.512749	8	-0.32	2.17	4.50	4.52	4.52	0.69	0.77	0.69		0.77	Shen P et al.	2017	Ore Geology Reviews	81(Pt.2)	808-828
3743	SYP9-3	CAOB	Western Junggar	82.4750	46.7697	Tonalite porphyry		294	294	3.00	16.30	0.1111	0.512702	6	-0.44	1.25	4.50	4.46	4.46	0.69	0.67	0.69		0.67	Shen P et al.	2017	Ore Geology Reviews	81(Pt.2)	808-828
3744	SYP9-2	CAOB	Western Junggar	82.4750	46.7697	Tonalite porphyry		294	294	2.86	15.60	0.1111	0.512667	7	-0.44	0.57	3.80	3.78	3.78	0.75	0.72	0.75		0.72	Shen P et al.	2017	Ore Geology Reviews	81(Pt.2)	808-828
3745	14SW-02	CAOB	Western Junggar	83.1222	45.8750	Diorite		312	312	2.40	10.50	0.1291	0.512835	0	-0.34	3.84	6.50	6.53	6.53	0.54	0.57	0.54		0.54	Shen P et al.	2017	Ore Geology Reviews	81(Pt.2)	808-828
3746	zk5219–429	CAOB	Western Junggar	82.4750	46.7697	Medium-grained granite		297	297	2.87	12.82	0.1356	0.512748	15	-0.31	2.15	4.40	4.46	4.46	0.70	0.79	0.70		0.79	Shen P et al.	2017	Ore Geology Reviews	81(Pt.2)	808-828
3747	STH-6	CAOB	Junggar	94.0000	43.4000	Andesite		341	341	3.57	16.95	0.1283	0.512859		-0.35	4.31	7.30	7.29	7.29	0.50	0.52	0.50			Zhao ZH et al.	2006	Acta Petrologica Sinica	(01)	199-214
3748	STH-4	CAOB	Junggar	94.0000	43.4000	Andesite		341	341	5.34	26.21	0.1241	0.512873		-0.37	4.58	7.75	7.74	7.74	0.46	0.47	0.46			Zhao ZH et al.	2006	Acta Petrologica Sinica	(01)	199-214
3749	T-17-8	CAOB	CMEB	113.3324	46.7851	Bi granite	Tumueryanaobao	240	240	3.03	18.50	0.0988	0.512573	7	-0.50	-1.27	1.74	1.73	1.73	0.87	0.77	0.87	0.77	0.87	Jiang SH et al.	2010	Acta Geoscientica Sinica	31(3)	321-330
3750	T-17-2	CAOB	CMEB	113.3324	46.7851	Bi granite	Tumueryanaobao	240	240	2.52	20.70	0.0738	0.512501	7	-0.62	-2.67	1.09	1.09	1.09	0.92	0.71	0.92	0.71	0.93	Jiang SH et al.	2010	Acta Geoscientica Sinica	31(3)	321-330
3751	T-17-19	CAOB	CMEB	113.3324	46.7851	Bi granite	Tumueryanaobao	240	240	3.63	27.20	0.0806	0.512529	14	-0.59	-2.13	1.42	1.43	1.43	0.90	0.71	0.90	0.71	0.90	Jiang SH et al.	2010	Acta Geoscientica Sinica	31(3)	321-330
3752	T-16	CAOB	CMEB	113.3289	46.7889	Bi granite	Tumueryanaobao	240	240	2.71	21.50	0.0763	0.512494	7	-0.61	-2.81	0.88	0.88	0.88	0.94	0.73	0.94	0.73	0.94	Jiang SH et al.	2010	Acta Geoscientica Sinica	31(3)	321-330
3753	T-14	CAOB	CMEB	113.3264	46.7919	Bi granite	Tumueryanaobao	240	240	2.33	18.70	0.0754	0.512491	4	-0.62	-2.87	0.86	0.85	0.85	0.94	0.73	0.94	0.73	0.95	Jiang SH et al.	2010	Acta Geoscientica Sinica	31(3)	321-330
3754	T-13	CAOB	CMEB	113.3264	46.7919	Bi granite	Tumueryanaobao	240	240	4.08	33.90	0.0728	0.512500	6	-0.63	-2.69	1.11	1.10	1.10	0.92	0.70	0.92	0.71	0.93	Jiang SH et al.	2010	Acta Geoscientica Sinica	31(3)	321-330
3755	Ttw92	CAOB	Kangur-juerotage,Tianshan	92.7000	42.0500	plagioclase granite porphyr	Tuwu	334	334	1.45	7.94	0.1106	0.512814	11	-0.44	3.43	7.10	7.10	7.10	0.51	0.50	0.51		0.51	Li WM et al.	2002	Northwestern Geology	（4）	41-64
3756	Biw38	CAOB	Kangur-juerotage,Tianshan	92.7000	42.0500	Diorite porphyrite	Tuwu	334	334	2.10	9.90	0.1281	0.512853	9	-0.35	4.19	7.12	7.12	7.12	0.51	0.53	0.51		0.51	Li WM et al.	2002	Northwestern Geology	（4）	41-64
3757	D277	NCC	NCC	118.3047	40.8194	Syenite	Jiashan	113	150	9.91	51.39	0.1166	0.512379	9	-0.41	-5.05	-3.89	-3.52	-3.52	1.23	1.21	1.23	1.21	1.23	Yang FQ et al.	2007	Journal of Asian Earth Sciences	29(5-6)	619-636
3758	D276	NCC	NCC	118.2986	40.8189	Syenite	Jiashan	113	150	10.09	54.57	0.1118	0.512421	10	-0.43	-4.23	-3.02	-2.61	-2.61	1.15	1.09	1.15	1.09	1.16	Yang FQ et al.	2007	Journal of Asian Earth Sciences	29(5-6)	619-636
3759	D275	NCC	NCC	118.2936	40.8175	Syenite	Jiashan	113	150	9.99	54.37	0.1112	0.512340	8	-0.43	-5.81	-4.58	-4.18	-4.18	1.28	1.20	1.28	1.21	1.28	Yang FQ et al.	2007	Journal of Asian Earth Sciences	29(5-6)	619-636
3760	D273	NCC	NCC	118.2853	40.8156	Syenite	Jiashan	113	150	12.77	69.17	0.1116	0.512335	8	-0.43	-5.91	-4.69	-4.28	-4.28	1.29	1.22	1.29	1.22	1.29	Yang FQ et al.	2007	Journal of Asian Earth Sciences	29(5-6)	619-636
3761	D272	NCC	NCC	118.2797	40.8144	Syenite	Jiashan	113	150	8.60	48.19	0.1080	0.512456	7	-0.45	-3.55	-2.27	-1.85	-1.85	1.09	1.00	1.09	1.00	1.10	Yang FQ et al.	2007	Journal of Asian Earth Sciences	29(5-6)	619-636
3762	D262	NCC	NCC	118.3000	40.8189	Syenite	Jiashan	113	150	11.03	65.86	0.1013	0.512325	10	-0.49	-6.11	-4.73	-4.28	-4.28	1.29	1.12	1.29	1.12	1.30	Yang FQ et al.	2007	Journal of Asian Earth Sciences	29(5-6)	619-636
3763	D261	NCC	NCC	118.2961	40.8167	Syenite	Jiashan	113	150	10.47	59.14	0.1071	0.512286	6	-0.46	-6.87	-5.57	-5.15	-5.15	1.36	1.23	1.36	1.24	1.37	Yang FQ et al.	2007	Journal of Asian Earth Sciences	29(5-6)	619-636
3764	D260	NCC	NCC	118.2797	40.8119	Syenite	Jiashan	113	150	8.82	46.15	0.1156	0.512360	9	-0.41	-5.42	-4.25	-3.87	-3.87	1.25	1.23	1.25	1.23	1.26	Yang FQ et al.	2007	Journal of Asian Earth Sciences	29(5-6)	619-636
3765	HJ13-110	CAOB	Eastern Tianshan	91.5799	41.8450	Felsic brecciated tuff		316	316	2.60	11.10	0.1416	0.512866	0	-0.28	4.45	6.70	6.67	6.67	0.53	0.60	0.53	0.60	0.53	Zhao L et al.	2010	Journal of Asian Earth Sciences	153	170-192
3766	HJ13-103	CAOB	Eastern Tianshan	91.5799	41.8450	Basaltic andesite porphyry		316	316	3.06	6.10	0.3032	0.513174	0	0.54	10.46	6.20	6.17	6.17	0.57	0.04	0.57	0.04	0.57	Zhao L et al.	2010	Journal of Asian Earth Sciences	153	170-192
3767	HJ13-102	CAOB	Eastern Tianshan	91.5799	41.8450	Basaltic andesite porphyry		316	316	9.22	14.90	0.3741	0.513351	0	0.90	13.91	6.80	6.77	6.77	0.52	0.19	0.52	0.19	0.52	Zhao L et al.	2010	Journal of Asian Earth Sciences	153	170-192
3768	HJ13-082	CAOB	Eastern Tianshan	91.5799	41.8450	Felsic brecciated tuff		316	316	2.55	8.50	0.1814	0.512899	0	-0.08	5.09	5.70	5.71	5.71	0.61	1.18	0.61	1.18	0.61	Zhao L et al.	2010	Journal of Asian Earth Sciences	153	170-192
3769	HJ13-025	CAOB	Eastern Tianshan	91.5799	41.8450	Diorite porphyry		316	316	4.60	16.20	0.1716	0.512876	0	-0.13	4.64	5.70	5.65	5.65	0.61	0.99	0.61	0.99	0.61	Zhao L et al.	2010	Journal of Asian Earth Sciences	153	170-192
3770	HJ13-007	CAOB	Eastern Tianshan	91.5799	41.8450	Felsic brecciated tuff		316	316	2.35	10.40	0.1366	0.512804	0	-0.31	3.24	5.70	5.66	5.66	0.61	0.68	0.61	0.68	0.61	Zhao L et al.	2010	Journal of Asian Earth Sciences	153	170-192
3771	XDG-122	CAOB	SHB	117.8453	43.0406	Porphyritic granite	Xiaodonggou	139	150	2.15	10.28	0.1266	0.512433	12	-0.36	-4.00	-2.75	-2.66	-2.66	1.15	1.25	1.15	1.26	1.16	Qin F et al.	2008	Geoscience	22(2)	173-180
3772	XDG-121	CAOB	SHB	117.8453	43.0406	Porphyritic granite	Xiaodonggou	138	150	1.45	6.59	0.1330	0.512455	90	-0.32	-3.57	-2.45	-2.35	-2.35	1.13	1.31	1.13	1.32	1.13	Qin F et al.	2008	Geoscience	22(2)	173-180
3773	MON-65E	CAOB	MOS	111.8190	46.6500	Granite		273	273	3.31	21.70	0.0929	0.512458		-0.53	-3.51	0.11	0.10	0.10	1.03	0.87	1.03			Zhao P et al.	2017	Journal of Asian Earth Sciences	144	225-242
3774	MON-65C	CAOB	MOS	111.8190	46.6500	Granite		273	273	1.61	9.40	0.1043	0.512447		-0.47	-3.73	-0.51	-0.51	-0.51	1.08	0.98	1.08			Zhao P et al.	2017	Journal of Asian Earth Sciences	144	225-242
3775	MON-65A	CAOB	MOS	111.8190	46.6500	Granite		273	273	3.80	23.30	0.0993	0.512417		-0.49	-4.31	-0.92	-0.92	-0.92	1.11	0.98	1.11			Zhao P et al.	2017	Journal of Asian Earth Sciences	144	225-242
3776	MON-60C	CAOB	MOS	112.1420	46.8670	Granite		265	265	3.17	16.00	0.1207	0.512598		-0.39	-0.78	1.79	1.79	1.79	0.89	0.90	0.89			Zhao P et al.	2017	Journal of Asian Earth Sciences	144	225-242
3777	MON-60A	CAOB	MOS	112.1420	46.8670	Granite		265	265	3.61	17.90	0.1228	0.512605		-0.38	-0.64	1.86	1.85	1.85	0.88	0.91	0.88			Zhao P et al.	2017	Journal of Asian Earth Sciences	144	225-242
3778	MON-37C	CAOB	MOS	112.7260	47.0540	Granite		254	254	3.20	20.50	0.0951	0.512502		-0.52	-2.65	0.64	0.64	0.64	0.97	0.83	0.97			Zhao P et al.	2017	Journal of Asian Earth Sciences	144	225-242
3779	MON-37A	CAOB	MOS	112.7260	47.0540	Granite		254	254	3.27	20.90	0.0953	0.512513		-0.52	-2.44	0.85	0.85	0.85	0.96	0.82	0.96			Zhao P et al.	2017	Journal of Asian Earth Sciences	144	225-242
3780	Zhaoshen-3	CAOB	SHB	125.1617	45.6897	Tonalite	Song-liao Basin	165	165	4.65	29.69	0.0946	0.512610	10	-0.52	-0.55	1.61	1.60	1.60	0.82	0.69	0.82	0.69	0.82	Wu FY et al.	2001	Physics and Chemistry of the Earth	26(9-10)	793-803
3781	Fangshen-4	CAOB	SHB	125.0956	45.9875	Gneiss	Song-liao Basin	165	165	2.28	12.65	0.1092	0.512504	12	-0.44	-2.61	-0.77	-0.77	-0.77	1.01	0.94	1.01	0.95	1.02	Wu FY et al.	2001	Physics and Chemistry of the Earth	26(9-10)	793-803
3782	Ershen-1	CAOB	SHB	125.2697	45.5800	Gneiss	Song-liao Basin	165	165	2.19	12.23	0.1085	0.512626	9	-0.45	-0.23	1.63	1.62	1.62	0.82	0.76	0.82	0.76	0.82	Wu FY et al.	2001	Physics and Chemistry of the Earth	26(9-10)	793-803
3783	Chang401	CAOB	SHB	125.0978	45.9606	Gneiss	Song-liao Basin	165	165	1.93	11.39	0.1022	0.512521	9	-0.48	-2.28	-0.29	-0.29	-0.29	0.98	0.86	0.98	0.86	0.98	Wu FY et al.	2001	Physics and Chemistry of the Earth	26(9-10)	793-803
3784	110	CAOB	Mongolia	98.0000	48.0000	Juvite		426	426	2.78	15.30	0.1099	0.512391	7	-0.44	-4.82	0.10	-0.10	-0.10	1.17	1.11	1.17			Vassily VV et al.	2019	Minerals	9(3)	170
3785	59	CAOB	Russia	90.0000	50.0000	Pulaskite		400	400	2.61	14.10	0.1120	0.512340	5	-0.43	-5.81	-1.20	-1.49	-1.49	1.26	1.21	1.26			Vassily VV et al.	2019	Minerals	9(3)	170
3786		CAOB	CMEB	117.9167	51.3322	Monzonite	Shakhtama	160	160	4.41	24.65	0.1081	0.512489	16	-0.45	-2.91	-1.09	-1.10	-1.10	1.04	0.95	1.04	0.96	1.04	Berzina et al.	2013	Russian Geology & Geophysics	54(6)	587-605
3787		CAOB	CMEB	117.9167	51.3322	Granite	Shakhtama	159	159	5.59	38.10	0.0887	0.512386	14	-0.55	-4.92	-2.70	-2.73	-2.73	1.17	0.93	1.17	0.93	1.17	Berzina et al.	2013	Russian Geology & Geophysics	54(6)	587-605
3788		CAOB	CMEB	117.9167	51.3322	Monzonite porphyry	Shakhtama	159	159	5.97	35.49	0.1017	0.512643	17	-0.48	0.10	2.02	2.03	2.03	0.78	0.69	0.78	0.69	0.78	Berzina et al.	2013	Russian Geology & Geophysics	54(6)	587-605
3789		CAOB	CMEB	117.9167	51.3322	Granite porphyry	Shakhtama	155	155	5.23	32.38	0.0977	0.512645	16	-0.50	0.14	2.10	2.10	2.10	0.77	0.66	0.77	0.67	0.77	Berzina et al.	2013	Russian Geology & Geophysics	54(6)	587-605
3790	YS-05	CAOB	SMXAB	117.5511	44.1125	Diorite	Bairendaba	327	327	4.37	19.10	0.1380	0.512380	7	-0.30	-5.03	-2.57	-2.58	-2.58	1.29	1.55	1.29	1.55	1.30	Liu YF et al.	2010	Geological Bulletin of China	29(5)	688-696
3791	YS-04	CAOB	SMXAB	117.5511	44.1125	Diorite	Bairendaba	327	327	3.14	18.30	0.1041	0.512224	7	-0.47	-8.08	-4.21	-4.21	-4.21	1.43	1.29	1.43		1.43	Liu YF et al.	2010	Geological Bulletin of China	29(5)	688-696
3792	YS-03	CAOB	SMXAB	117.5511	44.1125	Diorite	Bairendaba	327	327	2.56	17.20	0.0898	0.512200	5	-0.54	-8.54	-4.08	-4.08	-4.08	1.42	1.17	1.42	1.17	1.42	Liu YF et al.	2010	Geological Bulletin of China	29(5)	688-696
3793	YS-02	CAOB	SMXAB	117.5511	44.1125	Diorite	Bairendaba	327	327	2.04	17.90	0.0688	0.512250	6	-0.65	-7.57	-2.22	-2.23	-2.23	1.27	0.95	1.27	0.95	1.27	Liu YF et al.	2010	Geological Bulletin of China	29(5)	688-696
3794	BS-13	CAOB	SMXAB	117.5511	44.1125	Diorite	Bairendaba	327	327	4.84	33.30	0.0877	0.512351	7	-0.55	-5.60	-1.05	-1.05	-1.05	1.17	0.97	1.17	0.97	1.17	Liu YF et al.	2010	Geological Bulletin of China	29(5)	688-696
3795	BK16	CAOB	SMXAB	117.5511	44.1125	Diorite	Bairendaba	327	327	5.79	34.33	0.1020	0.512264	11	-0.48	-7.30	-3.34	-3.34	-3.34	1.36	1.21	1.36	1.21	1.36	Liu YF et al.	2010	Geological Bulletin of China	29(5)	688-696
3796	BK14	CAOB	SMXAB	117.5511	44.1125	Diorite	Bairendaba	327	327	2.75	16.39	0.1015	0.512464	6	-0.48	-3.39	0.59	0.58	0.58	1.04	0.93	1.04	0.93	1.04	Liu YF et al.	2010	Geological Bulletin of China	29(5)	688-696
3797	BK13	CAOB	SMXAB	117.5511	44.1125	Diorite	Bairendaba	327	327	3.11	19.07	0.0987	0.512237	14	-0.50	-7.82	-3.73	-3.73	-3.73	1.39	1.21	1.39	1.21	1.39	Liu YF et al.	2010	Geological Bulletin of China	29(5)	688-696
3798	MN1a	CAOB	Northern margin of Tarim	81.5833	40.8667	Rhyolite		287	287			0.1042	0.512532		-0.47	-2.07	1.32	1.32	1.32	0.94	0.86	0.94	0.86	0.95	Tian W et al.	2010	Contributions to Mineralogy & Petrology	160	407-425
3799	P14-4-1	CAOB	Inner Mongolia	112.0000	44.0000	Rhyolite		329	329	18.30	82.90	0.1345	0.512641		-0.32	0.06	2.67	2.67	2.67	0.87	0.98	0.87			Wei RH et al.	2017	Gondwana Research	51	234-254
3800	P141-8-1	CAOB	Inner Mongolia	112.0000	44.0000	Rhyolite		329	329	14.80	81.70	0.1103	0.512659		-0.44	0.40	4.03	4.03	4.03	0.76	0.73	0.76			Wei RH et al.	2017	Gondwana Research	51	234-254
3801	P14-13-1	CAOB	Inner Mongolia	112.0000	44.0000	Rhyolite		329	329	18.10	81.20	0.1358	0.512704		-0.31	1.29	3.85	3.85	3.85	0.77	0.87	0.77			Wei RH et al.	2017	Gondwana Research	51	234-254
3802	P14-11-1	CAOB	Inner Mongolia	112.0000	44.0000	Rhyolite		329	329	12.50	53.60	0.1420	0.512651		-0.28	0.26	2.56	2.55	2.55	0.88	1.06	0.88			Wei RH et al.	2017	Gondwana Research	51	234-254
3803	P14-1-1	CAOB	Inner Mongolia	112.0000	44.0000	Rhyolite		329	329	13.50	73.10	0.1125	0.512553		-0.43	-1.66	1.88	1.88	1.88	0.93	0.90	0.93			Wei RH et al.	2017	Gondwana Research	51	234-254
3804	P13-32-1	CAOB	Inner Mongolia	112.0000	44.0000	Rhyolite		329	329	14.00	74.20	0.1149	0.512691		-0.42	1.03	4.47	4.46	4.46	0.72	0.71	0.72			Wei RH et al.	2017	Gondwana Research	51	234-254
3805	P13-27-1	CAOB	Inner Mongolia	112.0000	44.0000	Rhyolite		329	329	10.20	61.30	0.1013	0.512701		-0.48	1.24	5.25	5.24	5.24	0.66	0.61	0.66			Wei RH et al.	2017	Gondwana Research	51	234-254
3806	P07-28-1	CAOB	Inner Mongolia	112.0000	44.0000	Dacite		329	329	5.03	22.80	0.1344	0.512707		-0.32	1.35	3.97	3.96	3.96	0.76	0.85	0.76			Wei RH et al.	2017	Gondwana Research	51	234-254
3807	P04-22-1	CAOB	Inner Mongolia	112.0000	44.0000	Dacite		329	329	7.26	33.40	0.1324	0.512705		-0.33	1.30	4.01	4.00	4.00	0.76	0.84	0.76			Wei RH et al.	2017	Gondwana Research	51	234-254
3808	P04-18-1	CAOB	Inner Mongolia	112.0000	44.0000	Dacite		329	329	6.86	31.70	0.1318	0.512750		-0.33	2.18	4.91	4.90	4.90	0.69	0.75	0.69			Wei RH et al.	2017	Gondwana Research	51	234-254
3809	P04-12-1	CAOB	Inner Mongolia	112.0000	44.0000	Dacite		329	329	4.84	22.50	0.1310	0.512690		-0.33	1.02	3.78	3.78	3.78	0.78	0.85	0.78			Wei RH et al.	2017	Gondwana Research	51	234-254
3810	Er-1-95	CAOB	Transbaikalia and Mongolia	109.9332	51.3944	Granodiorite		226	226	20.60	145.00	0.0859	0.512490		-0.56	-2.89	0.29	0.31	0.31	0.98	0.79	0.98			Lykhin DA et al.	2010	Geology of Ore Deposits	52(4)	289-301
3811	E-783/a	CAOB	Transbaikalia and Mongolia	110.0607	51.4391	Granite		227	227	8.13	48.20	0.1020	0.512250		-0.48	-7.57	-4.85	-4.83	-4.83	1.39	1.23	1.39			Lykhin DA et al.	2010	Geology of Ore Deposits	52(4)	289-301
3812	E-483	CAOB	Transbaikalia and Mongolia	110.0607	51.4391	Granite		227	227	13.90	71.70	0.1173	0.512570		-0.40	-1.33	0.95	0.97	0.97	0.92	0.92	0.92			Lykhin DA et al.	2010	Geology of Ore Deposits	52(4)	289-301
3813	E-400	CAOB	Transbaikalia and Mongolia	109.9332	51.3944	Granite		226	226	4.31	29.70	0.0880	0.512450		-0.55	-3.67	-0.54	-0.53	-0.53	1.04	0.85	1.04			Lykhin DA et al.	2010	Geology of Ore Deposits	52(4)	289-301
3814	E-300	CAOB	Transbaikalia and Mongolia	109.9332	51.3944	Granite		226	226	44.60	353.00	0.0764	0.512490		-0.61	-2.89	0.57	0.58	0.58	0.95	0.73	0.95			Lykhin DA et al.	2010	Geology of Ore Deposits	52(4)	289-301
3815	03T-58	CAOB	tau-borokonu mountain, Tians	84.4522	43.7061	ourmaline muscovite granite		287	287	4.20	23.10	0.1099	0.512426	9	-0.44	-4.14	-0.96	-0.96	-0.96	1.13	1.06	1.13		1.06	Xu XY et al.	2006	Northwestern Geology	39(1)	50-75
3816	03T-95	CAOB	South Yili Block Granite belt	83.3249	43.6467	Granite		302	302	1.34	4.45	0.1821	0.512600		-0.07	-0.74	-0.18	-0.18	-0.18	1.07	2.64	1.07	2.64	1.07	Xu XY et al.	2006	Northwest Geology	39(1)	50-75
3817	03T-64	CAOB	South Tianshan Granite belt	84.3423	43.1126	Granite		447	447	2.12	10.70	0.1198	0.512656		-0.39	0.35	4.74	4.74	4.74	0.80	0.80	0.80	0.80	0.80	Xu XY et al.	2006	Northwest Geology	39(1)	50-75
3818	03T-137	CAOB	Central Tianshan Granite belt	88.5370	42.4529	Granite		327	327	4.89	24.00	0.1232	0.512578		-0.37	-1.17	1.90	1.90	1.90	0.93	0.96	0.93	0.96	0.93	Xu XY et al.	2006	Northwest Geology	39(1)	50-75

3819	03T-129	CAOB	Central Tianshan Granite belt	88.5299	43.7061	Granite		394	394	4.00	19.00	0.1273	0.512297		-0.35	-6.65	-3.16	-3.16	-3.16	1.39	1.50	1.39	1.50	1.39	Xu XY et al.	2006	Northwest Geology	39(1)	50-75
3820	03T-106	CAOB	North Yili Block Granite belt	81.0384	44.4154	Granite		352	352	6.37	34.00	0.1133	0.512394		-0.42	-4.76	-1.01	-1.01	-1.01	1.19	1.15	1.19	1.15	1.19	Xu XY et al.	2006	Northwest Geology	39(1)	50-75
3821	02TB-24	CAOB	Central Tianshan Granite belt	88.5264	42.5223	Granite		424	424	9.08	47.00	0.1167	0.512385		-0.41	-4.94	-0.61	-0.61	-0.61	1.21	1.20	1.21	1.20	1.21	Xu XY et al.	2006	Northwest Geology	39(1)	50-75
3822	mg-7	CAOB	West Junggar	83.9167	45.0333	Hb monzogranite	Miaoergou	300	300	8.31	34.05	0.1476	0.512894	14	-0.25	4.99	6.87	6.87	6.87	0.50	0.59	0.50	0.59	0.50	Wu JJ and Chen B.	2004	XinJiang Geology	(01)	29-35
3823	mg-4	CAOB	West Junggar	83.9167	45.0333	Potash feldspar granite	Miaoergou	300	300	8.17	34.85	0.1417	0.512893	15	-0.28	4.97	7.08	7.08	7.08	0.48	0.54	0.48	0.54	0.48	Wu JJ and Chen B.	2004	XinJiang Geology	(01)	29-35
3824	mg-19	CAOB	West Junggar	83.9167	45.0333	Potash feldspar granite	Miaoergou	300	300	8.42	36.62	0.1390	0.512913	14	-0.29	5.36	7.57	7.57	7.57	0.44	0.48	0.44	0.48	0.44	Wu JJ and Chen B.	2004	XinJiang Geology	(01)	29-35
3825	mg-18	CAOB	West Junggar	83.9167	45.0333	Granodiorite	Miaoergou	300	300	9.27	38.10	0.1472	0.512885	9	-0.25	4.82	6.71	6.71	6.71	0.51	0.61	0.51	0.61	0.51	Wu JJ and Chen B.	2004	XinJiang Geology	(01)	29-35
3826	mg-16	CAOB	West Junggar	83.9167	45.0333	Potash feldspar granite	Miaoergou	300	300	7.88	42.40	0.1124	0.512903	15	-0.43	5.17	8.40	8.40	8.40	0.38	0.37	0.38	0.37	0.38	Wu JJ and Chen B.	2004	XinJiang Geology	(01)	29-35
3827	mg-13	CAOB	West Junggar	83.9167	45.0333	Potash feldspar granite	Miaoergou	300	300	1.78	7.92	0.1362	0.512898	12	-0.31	5.07	7.39	7.39	7.39	0.46	0.50	0.46	0.50	0.46	Wu JJ and Chen B.	2004	XinJiang Geology	(01)	29-35
3828	mg-12	CAOB	West Junggar	83.9167	45.0333	Qtz monzogranite	Miaoergou	300	300	7.81	31.85	0.1483	0.512883	12	-0.25	4.78	6.63	6.63	6.63	0.52	0.62	0.52	0.62	0.52	Wu JJ and Chen B.	2004	XinJiang Geology	(01)	29-35
3829	MAC10-35	CAOB	Western Junggar	84.7521	45.6910	Diorite dyke	Karamay	300	300	1.90	7.50	0.1543	0.512983	12	-0.22	6.73	8.35	8.35	8.35	0.38	0.43	0.38	0.43	0.38	Ma C et al.	2012	Lithos	140–141(3)	152-165
3830	MAC10-34	CAOB	Western Junggar	84.7521	45.6921	Diorite dyke	Karamay	300	300	4.29	23.40	0.1110	0.512810	11	-0.44	3.36	6.64	6.64	6.64	0.52	0.51	0.52	0.51	0.52	Ma C et al.	2012	Lithos	140–141(3)	152-165
3831	MAC10-24	CAOB	Western Junggar	84.6851	45.6572	Diorite dyke	Karamay	300	300	3.44	15.10	0.1376	0.512901	13	-0.30	5.13	7.39	7.39	7.39	0.46	0.50	0.46	0.50	0.46	Ma C et al.	2012	Lithos	140–141(3)	152-165
3832	MAC10-21	CAOB	Western Junggar	84.3170	45.5147	Diorite dyke	Baogutu	300	300	3.14	13.20	0.1428	0.512919	15	-0.27	5.48	7.54	7.54	7.54	0.45	0.50	0.45	0.50	0.45	Ma C et al.	2012	Lithos	140–141(3)	152-165
3833	MAC10-19	CAOB	Western Junggar	84.3170	45.5147	Diorite dyke	Baogutu	300	300	2.37	10.70	0.1344	0.512886	15	-0.32	4.84	7.22	7.22	7.22	0.47	0.51	0.47	0.51	0.47	Ma C et al.	2012	Lithos	140–141(3)	152-165
3834	MAC10-18	CAOB	Western Junggar	84.3170	45.5147	Diorite dyke	Baogutu	300	300	1.81	8.40	0.1306	0.512873	15	-0.34	4.58	7.11	7.11	7.11	0.48	0.51	0.48	0.51	0.48	Ma C et al.	2012	Lithos	140–141(3)	152-165
3835	MAC10-17	CAOB	Western Junggar	84.3217	45.5094	Diorite dyke	Baogutu	300	300	2.20	9.70	0.1369	0.512914	13	-0.30	5.38	7.67	7.67	7.67	0.44	0.47	0.44	0.47	0.44	Ma C et al.	2012	Lithos	140–141(3)	152-165
3836	HST0610	CAOB	Inner Mongolia	118.0000	43.5200	Andesite		227	227	4.04	19.60	0.1255	0.512753		-0.36	2.24	4.30	4.30	4.30	0.65	0.69	0.65			Liu YS et al.	2012	Chemical Geology	328	89-108
3837	HST0605	CAOB	Inner Mongolia	118.0000	43.5200	Andesite		227	227	3.29	16.20	0.1237	0.512703		-0.37	1.27	3.38	3.38	3.38	0.73	0.76	0.73			Liu YS et al.	2012	Chemical Geology	328	89-108
3838	HST0603	CAOB	Inner Mongolia	118.0000	43.5200	Andesite		227	227	3.87	18.10	0.1302	0.512782		-0.34	2.81	4.73	4.73	4.73	0.62	0.67	0.62			Liu YS et al.	2012	Chemical Geology	328	89-108
3839	HST0602	CAOB	Inner Mongolia	118.0000	43.5200	Andesite		227	227	3.26	15.80	0.1257	0.512708		-0.36	1.37	3.42	3.42	3.42	0.72	0.77	0.72			Liu YS et al.	2012	Chemical Geology	328	89-108
3840	SJ024-4	CAOB	Tianshan	113.0000	45.0000	Granodiorite		300	300	4.63	22.70	0.1242	0.512461		-0.37	-3.45	-0.68	-0.68	-0.68	1.12	1.17	1.12			Zhang XR et al.	2017	Journal of Asian Earth Sciences	145(Part.B)	446-459
3841	SJ024-3	CAOB	Tianshan	113.0000	45.0000	Granodiorite		300	300	4.79	23.00	0.1268	0.512454		-0.36	-3.59	-0.91	-0.92	-0.92	1.14	1.22	1.14			Zhang XR et al.	2017	Journal of Asian Earth Sciences	145(Part.B)	446-459
3842	SJ007-4	CAOB	Tianshan	113.0000	45.0000	Diorite		300	300	5.63	26.50	0.1294	0.512636		-0.34	-0.04	2.54	2.54	2.54	0.85	0.93	0.85			Zhang XR et al.	2017	Journal of Asian Earth Sciences	145(Part.B)	446-459
3843	SJ007-3	CAOB	Tianshan	113.0000	45.0000	Diorite		300	300	5.86	28.50	0.1252	0.512602		-0.36	-0.70	2.04	2.03	2.03	0.90	0.94	0.90			Zhang XR et al.	2017	Journal of Asian Earth Sciences	145(Part.B)	446-459
3844	B19	CAOB	Tianshan	113.0000	45.0000	Granodiorite		300	300	4.88	23.30	0.1276	0.512553		-0.35	-1.66	0.99	0.99	0.99	0.98	1.06	0.98			Zhang XR et al.	2017	Journal of Asian Earth Sciences	145(Part.B)	446-459
3845	B12	CAOB	Tianshan	113.0000	45.0000	Granodiorite		300	300	4.03	19.50	0.1259	0.512483		-0.36	-3.02	-0.31	-0.31	-0.31	1.09	1.16	1.09			Zhang XR et al.	2017	Journal of Asian Earth Sciences	145(Part.B)	446-459
3846	SCJ8	NCC	NCC	120.2803	41.9603	Diorite porphyry	Jinchanggouliang	227	227	4.46	26.68	0.1011	0.512026	3	-0.49	-11.94	-9.17	-9.17	-9.17	1.75	1.52	1.75	1.52	1.75	Fu LB et al.	2012	Geological Magazine	149(1)	39-55
3847	SCJ7	NCC	NCC	120.2803	41.9603	Diorite porphyry	Jinchanggouliang	227	227	4.37	25.86	0.1022	0.512012	6	-0.48	-12.21	-9.48	-9.47	-9.47	1.77	1.55	1.77	1.56	1.77	Fu LB et al.	2012	Geological Magazine	149(1)	39-55
3848	SCJ6	NCC	NCC	120.2803	41.9603	Diorite porphyry	Jinchanggouliang	227	227	4.44	27.72	0.0969	0.512035	3	-0.51	-11.76	-8.88	-8.87	-8.87	1.72	1.45	1.72	1.46	1.73	Fu LB et al.	2012	Geological Magazine	149(1)	39-55
3849	SCJ4	NCC	NCC	120.2803	41.9603	Diorite	Jinchanggouliang	227	227	6.14	35.33	0.1052	0.512491	2	-0.47	-2.87	-0.21	-0.22	-0.22	1.02	0.93	1.02	0.93	1.02	Fu LB et al.	2012	Geological Magazine	149(1)	39-55
3850	SCJ3	NCC	NCC	120.2803	41.9603	Diorite	Jinchanggouliang	227	227	6.29	37.44	0.1017	0.512483	3	-0.48	-3.02	-0.27	-0.27	-0.27	1.02	0.91	1.02	0.91	1.03	Fu LB et al.	2012	Geological Magazine	149(1)	39-55
3851	SCJ1	NCC	NCC	120.2803	41.9603	Diorite	Jinchanggouliang	227	227	5.29	29.51	0.1084	0.511893	9	-0.45	-14.53	-11.98	-11.97	-11.97	1.97	1.81	1.97	1.82	1.98	Fu LB et al.	2012	Geological Magazine	149(1)	39-55
3852	SCB3	NCC	NCC	120.2803	41.9603	Diorite porphyry	Jinchanggouliang	227	227	3.97	21.93	0.1096	0.512331	3	-0.44	-5.99	-3.47	-3.47	-3.47	1.28	1.20	1.28	1.20	1.29	Fu LB et al.	2012	Geological Magazine	149(1)	39-55
3853	SCB2	NCC	NCC	120.2803	41.9603	Diorite porphyry	Jinchanggouliang	227	227	4.08	22.57	0.1094	0.512319	2	-0.44	-6.22	-3.70	-3.69	-3.69	1.30	1.21	1.30	1.22	1.31	Fu LB et al.	2012	Geological Magazine	149(1)	39-55
3854	SCB1	NCC	NCC	120.2803	41.9603	Diorite porphyry	Jinchanggouliang	227	227	4.03	22.58	0.1078	0.512318	6	-0.45	-6.24	-3.66	-3.67	-3.67	1.30	1.20	1.30	1.20	1.30	Fu LB et al.	2012	Geological Magazine	149(1)	39-55
3855	C14TL59	CAOB	Yili	82.5667	45.2000	Bi Granite		288	288	2.38	12.30	0.1178	0.512939	0	-0.40	5.87	8.80	8.77	8.77	0.34	0.34	0.34	0.34	0.34	Wang XS et al.	2018	Journal of Asian Earth Sciences	153	118-138
3856	C14TL56	CAOB	Yili	82.5667	45.2000	Bi Granite		288	288	2.42	12.50	0.1178	0.512883	0	-0.40	4.78	7.70	7.67	7.67	0.43	0.43	0.43	0.43	0.43	Wang XS et al.	2018	Journal of Asian Earth Sciences	153	118-138
3857	C14BL31	CAOB	Yili	81.5833	45.2000	Rhyolite		303	303	6.16	30.00	0.1250	0.512694	0	-0.36	1.09	3.80	3.86	3.86	0.75	0.78	0.75	0.79	0.75	Wang XS et al.	2018	Journal of Asian Earth Sciences	153	118-138
3858	C14BL29	CAOB	Yili	81.5833	45.2000	Rhyolite		303	303	5.88	27.90	0.1283	0.512654	0	-0.35	0.31	2.90	2.96	2.96	0.82	0.89	0.82	0.89	0.83	Wang XS et al.	2018	Journal of Asian Earth Sciences	153	118-138
3859	C14BL25	CAOB	Yili	81.7833	45.1667	Ignimbrite		300	300	10.80	55.50	0.1184	0.512840	0	-0.40	3.94	7.00	6.94	6.94	0.50	0.50	0.50	0.50	0.50	Wang XS et al.	2018	Journal of Asian Earth Sciences	153	118-138
3860	C14BL22	CAOB	Yili	81.7833	45.1667	Ignimbrite		300	300	9.06	42.30	0.1304	0.512861	0	-0.34	4.35	6.90	6.89	6.89	0.50	0.53	0.50	0.53	0.50	Wang XS et al.	2018	Journal of Asian Earth Sciences	153	118-138
3861	C14BL20	CAOB	Yili	81.7833	45.1667	Rhyolite		299	299	6.51	30.90	0.1282	0.512683	0	-0.35	0.88	3.50	3.49	3.49	0.78	0.83	0.78	0.84	0.78	Wang XS et al.	2018	Journal of Asian Earth Sciences	153	118-138
3862	C14BL17	CAOB	Yili	81.7833	45.1667	Rhyolite		299	299	6.03	28.30	0.1297	0.512701	0	-0.34	1.23	3.80	3.78	3.78	0.75	0.82	0.75	0.82	0.76	Wang XS et al.	2018	Journal of Asian Earth Sciences	153	118-138
3863	C14BL09	CAOB	Yili	81.7833	45.1667	Rhyolite		301	301	6.19	29.60	0.1273	0.512698	0	-0.35	1.17	3.80	3.83	3.83	0.75	0.80	0.75	0.80	0.75	Wang XS et al.	2018	Journal of Asian Earth Sciences	153	118-138
3864	C14BL06	CAOB	Yili	81.7833	45.1667	Rhyolite		301	301	6.20	29.10	0.1297	0.512690	0	-0.34	1.01	3.60	3.59	3.59	0.77	0.84	0.77	0.84	0.77	Wang XS et al.	2018	Journal of Asian Earth Sciences	153	118-138
3865	BP37-4	CAOB	Northern Inner Mongolia	113.7500	43.6667	Gabbroic diorite		310	310	2.48	9.63	0.1550	0.512678	5	-0.21	0.78	2.40	2.43	2.43	0.87	1.22	0.87	1.24	0.89	Chen B.	2000	Tectonophysics	328(1)	157-182
3866	BP35-3	CAOB	SMXAB	113.5583	43.6569	Tonalite	Baolidao	310	310	2.12	8.30	0.1540	0.512563	7	-0.22	-1.46	0.22	0.23	0.23	1.05	1.50	1.05	1.50	1.05	Chen B.	2000	Tectonophysics	328(1)	157-182
3867	BP31-2	CAOB	SMXAB	113.7167	43.2503	Adamellite	Halatu	230	230	3.66	17.71	0.1250	0.512550	6	-0.36	-1.72	0.39	0.39	0.39	0.97	1.03	0.97	1.03	0.98	Chen B.	2000	Tectonophysics	328(1)	157-182
3868	BP30-9	CAOB	SMXAB	113.5583	43.6569	Granodiorite	Baolidao	310	310	1.56	7.21	0.1300	0.512539	7	-0.34	-1.93	0.71	0.71	0.71	1.01	1.11	1.01	1.12	1.01	Chen B.	2000	Tectonophysics	328(1)	157-182
3869	BP30-11	CAOB	SMXAB	113.5583	43.6569	Qtz diorite	Baolidao	310	310	2.10	8.11	0.1560	0.512579	4	-0.21	-1.15	0.45	0.46	0.46	1.03	1.51	1.03	1.51	1.04	Chen B.	2000	Tectonophysics	328(1)	157-182
3870	BP27-3	CAOB	SMXAB	113.7167	43.2503	Adamellite	Halatu	230	230	4.06	26.39	0.0930	0.512529	5	-0.53	-2.13	0.92	0.92	0.92	0.93	0.78	0.93	0.79	0.93	Chen B.	2000	Tectonophysics	328(1)	157-182
3871	BP24-5	CAOB	SMXAB	113.7167	43.2503	Granodiorite	Halatu	230	230	4.33	25.89	0.1010	0.512547	5	-0.49	-1.78	1.04	1.03	1.03	0.92	0.82	0.92	0.82	0.92	Chen B.	2000	Tectonophysics	328(1)	157-182
3872	BP22-4	CAOB	SMXAB	113.7167	43.2503	Adamellite	Halatu	230	230	3.62	20.10	0.1080	0.512511	7	-0.45	-2.48	0.12	0.13	0.13	0.99	0.92	0.99	0.92	1.00	Chen B.	2000	Tectonophysics	328(1)	157-182
3873	BP21-15	CAOB	SMXAB	113.7167	43.2503	Adamellite	Halatu	230	230	1.67	8.32	0.1210	0.512413	8	-0.38	-4.39	-2.16	-2.17	-2.17	1.18	1.21	1.18	1.21	1.18	Chen B.	2000	Tectonophysics	328(1)	157-182
3874	BP20-15	CAOB	SMXAB	113.7167	43.2503	Muscovite granite	Halatu	230	230	1.37	7.25	0.1140	0.512413	6	-0.42	-4.39	-1.97	-1.96	-1.96	1.16	1.13	1.16	1.13	1.17	Chen B.	2000	Tectonophysics	328(1)	157-182
3875	BM-5	CAOB	Northern Inner Mongolia	113.7500	43.6667	Amph. Schist		310	310	3.21	9.27	0.2090	0.512887	7	0.06	4.86	4.30	4.37	4.37	0.70	8.33	0.70	9.51	1.46	Chen B.	2000	Tectonophysics	328(1)	157-182
3876	BM-2	CAOB	Northern Inner Mongolia	113.7500	43.6667	Amph. Schist		310	310	2.35	8.85	0.1600	0.512688	7	-0.19	0.98	2.40	2.43	2.43	0.87	1.31	0.87	1.33	1.62	Chen B.	2000	Tectonophysics	328(1)	157-182
3877	93YS-2	CAOB	SMXAB	113.5583	43.6569	Tonalite	Baolidao	310	310	2.07	8.56	0.1450	0.512542	7	-0.26	-1.87	0.18	0.17	0.17	1.05	1.35	1.05	1.35	1.06	Chen B.	2000	Tectonophysics	328(1)	157-182
3878	93SS-2	CAOB	SMXAB	113.5583	43.6569	Qtz diorite	Baolidao	310	310	3.16	12.94	0.1470	0.512527	7	-0.25	-2.17	-0.19	-0.20	-0.20	1.08	1.42	1.08	1.43	1.09	Chen B.	2000	Tectonophysics	328(1)	157-182
3879	93SC-2	CAOB	Northern Inner Mongolia	113.7500	43.6667	Gabbroic diorite		310	310	2.28	9.05	0.1520	0.512653	7	-0.23	0.29	2.10	2.06	2.06	0.90	1.23	0.90	1.23	0.92	Chen B.	2000	Tectonophysics	328(1)	157-182
3880	zk9235-457	CAOB	Western Junggar	82.4750	45.7875	Granitoid		295	295	3.02	14.94	0.1223	0.512758		-0.38	2.34	5.10	5.14	5.14	0.64	0.65	0.64		0.64	Zhong SH et al.	2017	Ore Geology Reviews	88	116-139
3881	zk6-19-490	CAOB	Western Junggar	82.4750	45.7875	Granitoid		295	295	2.02	10.05	0.1219	0.512790		-0.38	2.97	5.80	5.78	5.78	0.59	0.60	0.59		0.59	Zhong SH et al.	2017	Ore Geology Reviews	88	116-139
3882	zk6019-507	CAOB	Western Junggar	82.4750	45.7875	Granitoid		295	295	3.27	15.32	0.1294	0.512800		-0.34	3.16	5.70	5.69	5.69	0.59	0.63	0.59		0.59	Zhong SH et al.	2017	Ore Geology Reviews	88	116-139
3883	zk5219-474	CAOB	Western Junggar	82.4750	45.7875	Granitoid		295	295	2.70	10.96	0.1494	0.512799		-0.24	3.14	4.90	4.92	4.92	0.66	0.83	0.66		0.66	Zhong SH et al.	2017	Ore Geology Reviews	88	116-139
3884	zk0809-193	CAOB	Western Junggar	82.4750	45.7875	Granitoid		295	295	4.46	21.59	0.1251	0.512828		-0.36	3.71	6.40	6.40	6.40	0.54	0.55	0.54		0.54	Zhong SH et al.	2017	Ore Geology Reviews	88	116-139
3885	zk0809-166	CAOB	Western Junggar	82.4750	45.7875	Granitoid		295	295	1.67	8.23	0.1230	0.512817		-0.37	3.49	6.30	6.27	6.27	0.55	0.56	0.55		0.55	Zhong SH et al.	2017	Ore Geology Reviews	88	116-139
3886	s3d4-3	CAOB	Western Junggar	82.4750	45.7875	Granitoid		295	295	2.93	15.95	0.1111	0.512770		-0.44	2.57	5.80	5.80	5.80	0.58	0.57	0.58		0.58	Zhong SH et al.	2017	Ore Geology Reviews	88	116-139
3887	s3d1-1	CAOB	Western Junggar	82.4750	45.7875	Granitoid		295	295	3.26	17.84	0.1106	0.512740		-0.44	1.99	5.20	5.23	5.23	0.63	0.61	0.63		0.63	Zhong SH et al.	2017	Ore Geology Reviews	88	116-139
3888	s1p3-1	CAOB	Western Junggar	82.4750	45.7875	Granitoid		295	295	2.27	11.19	0.1228	0.512748		-0.38	2.15	4.90	4.93	4.93	0.66	0.67	0.66		0.66	Zhong SH et al.	2017	Ore Geology Reviews	88	116-139
3889	QJ1-7	CAOB	South Tianshan	82.4167	43.7000	Granite		296	296	8.67	50.30	0.1040	0.512662	0	-0.47	0.47	4.00	3.97	3.97	0.73	0.68	0.73		0.74	Li NB et al.	2015	Journal of Asian Earth Sciences	113（PT.1)	238-253
3890	QJ1-6	CAOB	South Tianshan	82.4167	43.7000	Granite		296	296	8.43	47.90	0.1060	0.512660	0	-0.46	0.43	3.90	3.86	3.86	0.74	0.69	0.74		0.75	Li NB et al.	2015	Journal of Asian Earth Sciences	113（PT.1)	238-253
3891	QJ1-5	CAOB	South Tianshan	82.4167	43.7000	Granite		296	296	8.11	43.90	0.1120	0.512670	0	-0.43	0.62	3.80	3.82	3.82	0.75	0.72	0.75		0.75	Li NB et al.	2015	Journal of Asian Earth Sciences	113（PT.1)	238-253
3892	QJ1-4	CAOB	South Tianshan	82.4167	43.7000	Granite		296	296	8.46	46.60	0.1100	0.512665	0	-0.44	0.53	3.80	3.80	3.80	0.75	0.71	0.75		0.75	Li NB et al.	2015	Journal of Asian Earth Sciences	113（PT.1)	238-253
3893	QJ1-3	CAOB	South Tianshan	82.4167	43.7000	Granite		296	296	7.81	42.10	0.1120	0.512670	0	-0.43	0.62	3.80	3.82	3.82	0.75	0.72	0.75		0.75	Li NB et al.	2015	Journal of Asian Earth Sciences	113（PT.1)	238-253
3894	QJ1-2	CAOB	South Tianshan	82.4167	43.7000	Granite		296	296	8.54	49.10	0.1050	0.512659	0	-0.47	0.41	3.90	3.87	3.87	0.74	0.69	0.74		0.74	Li NB et al.	2015	Journal of Asian Earth Sciences	113（PT.1)	238-253
3895	11QJ1-4	CAOB	South Tianshan	82.4167	43.7000	Rhyolite		306	306	4.93	25.80	0.1150	0.512740	0	-0.42	1.99	5.20	5.18	5.18	0.64	0.63	0.64		0.65	Li NB et al.	2015	Journal of Asian Earth Sciences	113（PT.1)	238-253
3896	11QJ1-3	CAOB	South Tianshan	82.4167	43.7000	Rhyolite		306	306	5.22	30.50	0.1040	0.512724	0	-0.47	1.68	5.30	5.30	5.30	0.63	0.59	0.63		0.63	Li NB et al.	2015	Journal of Asian Earth Sciences	113（PT.1)	238-253
3897	11QJ1-1	CAOB	South Tianshan	82.4167	43.7000	Rhyolite		306	306	4.83	23.70	0.1230	0.512765	0	-0.37	2.48	5.40	5.36	5.36	0.63	0.65	0.63		0.63	Li NB et al.	2015	Journal of Asian Earth Sciences	113（PT.1)	238-253
3898	10QJ-3D	CAOB	South Tianshan	82.4167	43.7000	Granite		296	296	7.12	34.20	0.1260	0.512722	0	-0.36	1.64	4.30	4.31	4.31	0.71	0.74	0.71		0.71	Li NB et al.	2015	Journal of Asian Earth Sciences	113（PT.1)	238-253
3899	NM12-128	CAOB	Xing-Meng orogenic belt	113.4611	44.3953	Granite porphyry	Baoligezu	287	287	7.30	34.40	0.1346	0.512805	13	-0.32	3.26	5.52	5.53	5.53	0.60	0.67	0.60	0.67	0.60	Li K et al.	2015	Acta Geologica Sinica	89(2)	272-288
3900	NM12-127	CAOB	Xing-Meng orogenic belt	113.4511	44.3903	Andesite	Baoligezu	311	311	4.51	21.90	0.1307	0.512723	13	-0.34	1.66	4.27	4.28	4.28	0.72	0.78	0.72	0.78	0.72	Li K et al.	2015	Acta Geologica Sinica	89(2)	272-288
3901	NM12-126	CAOB	Xing-Meng orogenic belt	113.4511	44.3903	Basaltic trachyandesite	Baoligezu	311	311	5.48	26.60	0.1307	0.512602	15	-0.34	-0.70	1.91	1.92	1.92	0.91	1.01	0.91	1.01	0.91	Li K et al.	2015	Acta Geologica Sinica	89(2)	272-288
3902	xt88	CAOB	Southern margin of Yili block	82.2500	43.7833	Dacite		261	261	2.03	12.72	0.0967	0.512556		-0.51	-1.60	1.73	1.73	1.73	0.89	0.77	0.89	0.78	0.89	Zhao ZH et al.	2008	Lithos	102（1-2）	374-391
3903	s2	CAOB	Southern margin of Yili block	82.2500	43.7833	Tonalite		278	278	2.74	12.86	0.1288	0.512806		-0.35	3.28	5.69	5.69	5.69	0.58	0.62	0.58	0.62	0.58	Zhao ZH et al.	2008	Lithos	102（1-2）	374-391
3904	NL2-3	CAOB	Southern margin of Yili block	82.2500	43.7833	Qtz albite porphyry		260	260			0.0978	0.512518		-0.50	-2.34	0.94	0.94	0.94	0.95	0.83	0.95	0.83	0.96	Zhao ZH et al.	2008	Lithos	102（1-2）	374-391
3905	NL2-13	CAOB	Southern margin of Yili block	82.2500	43.7833	Qtz albite porphyry		260	260			0.1092	0.512542		-0.44	-1.87	1.03	1.03	1.03	0.94	0.89	0.94	0.89	0.95	Zhao ZH et al.	2008	Lithos	102（1-2）	374-391
3906	AJW-06-476	CAOB	Southern Mongolia	107.2000	43.2000	Granodiorite		366	366	3.16	15.80	0.1218	0.512629		-0.38	-0.18	3.33	3.32	3.32	0.84	0.86	0.84			Wainwright AJ et al.	2011	Gondwana Research	19(3)	764-787
3907	AJW-06-476	CAOB	Southern Mongolia	107.2000	43.2000	Granodiorite		366	366	3.16	15.80	0.1218	0.512635		-0.38	-0.06	3.45	3.44	3.44	0.84	0.85	0.84			Wainwright AJ et al.	2011	Gondwana Research	19(3)	764-787
3908	AJW-06-451	CAOB	Southern Mongolia	107.2000	43.2000	Monzodiorite		372	372	3.27	14.90	0.1337	0.512799		-0.32	3.14	6.14	6.13	6.13	0.62	0.67	0.62			Wainwright AJ et al.	2011	Gondwana Research	19(3)	764-787
3909	AJW-06-415	CAOB	Southern Mongolia	107.2000	43.2000	Granodiorite		366	366	3.63	18.60	0.1189	0.512659		-0.40	0.41	4.05	4.04	4.04	0.79	0.79	0.79			Wainwright AJ et al.	2011	Gondwana Research	19(3)	764-787
3910	AJW-04-385	CAOB	Southern Mongolia	107.2000	43.2000	Monzodiorite		372	372	3.75	17.40	0.1313	0.512844		-0.33	4.02	7.13	7.12	7.12	0.54	0.57	0.54			Wainwright AJ et al.	2011	Gondwana Research	19(3)	764-787
3911	AJW-03-183	CAOB	Southern Mongolia	107.2000	43.2000	Andesite		345	345	4.40	20.50	0.1307	0.512817		-0.34	3.49	6.40	6.40	6.40	0.58	0.61	0.58			Wainwright AJ et al.	2011	Gondwana Research	19(3)	764-787
3912	AJW-03-183	CAOB	Southern Mongolia	107.2000	43.2000	Andesite		345	345	4.40	20.50	0.1307	0.512805		-0.34	3.26	6.17	6.16	6.16	0.60	0.63	0.60			Wainwright AJ et al.	2011	Gondwana Research	19(3)	764-787
3913	AJW-03-179	CAOB	Southern Mongolia	107.2000	43.2000	Granodiorite		366	366	2.40	11.20	0.1305	0.512795		-0.34	3.06	6.16	6.15	6.15	0.61	0.65	0.61			Wainwright AJ et al.	2011	Gondwana Research	19(3)	764-787
3914	AJW-03-132	CAOB	Southern Mongolia	107.2000	43.2000	Granite		324	324	1.40	9.20	0.0927	0.512729		-0.53	1.78	6.08	6.08	6.08	0.59	0.53	0.59			Wainwright AJ et al.	2011	Gondwana Research	19(3)	764-787
3915	AJW-03-120	CAOB	Southern Mongolia	107.2000	43.2000	Rhyolite		335	335	2.20	12.00	0.1117	0.512805		-0.43	3.26	6.90	6.89	6.89	0.53	0.52	0.53			Wainwright AJ et al.	2011	Gondwana Research	19(3)	764-787
3916	AJW-03-107	CAOB	Southern Mongolia	107.2000	43.2000	Rhyolite		335	335	2.10	11.70	0.1093	0.512766		-0.44	2.50	6.24	6.23	6.23	0.58	0.56	0.58			Wainwright AJ et al.	2011	Gondwana Research	19(3)	764-787

3917	AJW-03-093	CAOB	Southern Mongolia	107.2000	43.2000	Granodiorite		366	366	3.50	17.30	0.1232	0.512643		-0.37	0.10	3.54	3.53	3.53	0.83	0.85	0.83			Wainwright AJ et al.	2011	Gondwana Research	19(3)	764-787
3918	AJW-03-091	CAOB	Southern Mongolia	107.2000	43.2000	Dacite		347	347	5.00	24.50	0.1243	0.512802		-0.37	3.20	6.41	6.40	6.40	0.58	0.59	0.58			Wainwright AJ et al.	2011	Gondwana Research	19(3)	764-787
3919	AJW-03-091	CAOB	Southern Mongolia	107.2000	43.2000	Dacite		347	347	5.00	24.50	0.1243	0.512815		-0.37	3.45	6.67	6.66	6.66	0.56	0.57	0.56			Wainwright AJ et al.	2011	Gondwana Research	19(3)	764-787
3920	AJW-03-068	CAOB	Southern Mongolia	107.2000	43.2000	Monzodiorite		372	372	5.30	23.70	0.1362	0.512818		-0.31	3.51	6.39	6.38	6.38	0.60	0.65	0.60			Wainwright AJ et al.	2011	Gondwana Research	19(3)	764-787
3921	AJW-03-068	CAOB	Southern Mongolia	107.2000	43.2000	Monzodiorite		372	372	5.30	23.70	0.1362	0.512821		-0.31	3.57	6.45	6.44	6.44	0.60	0.65	0.60			Wainwright AJ et al.	2011	Gondwana Research	19(3)	764-787
3922	AJW-03-055	CAOB	Southern Mongolia	107.2000	43.2000	Andesite		346	346	5.40	25.50	0.1290	0.512662		-0.34	0.47	3.46	3.46	3.46	0.82	0.88	0.82			Wainwright AJ et al.	2011	Gondwana Research	19(3)	764-787
3923	5-446*	CAOB	Southern Gornyi Altai	89.0639	49.3986	Elvan	Eastern Kalguty	202	202	1.56	5.84	0.1621	0.512350	6	-0.18	-5.62	-4.72	-4.73	-4.73	1.36	2.35	1.36	2.36	1.37	Annikova IY et al.	2006	Petrology	14(1)	81–97
3924	1-278/1	CAOB	Southern Gornyi Altai	89.0639	49.3986	Elvan	Eastern Kalguty	202	202	0.73	2.99	0.1471	0.512313	9	-0.25	-6.34	-5.05	-5.06	-5.06	1.39	1.91	1.39	1.92	1.40	Annikova IY et al.	2006	Petrology	14(1)	81–97
3925	1-277	CAOB	Southern Gornyi Altai	88.9361	49.3861	Porphyritic Bi granite	Kalguty	216	216	6.57	31.90	0.1245	0.512442	10	-0.37	-3.82	-1.83	-1.83	-1.83	1.14	1.21	1.14	1.21	1.14	Annikova IY et al.	2006	Petrology	14(1)	81–97
3926	1-269	CAOB	Southern Gornyi Altai	89.0639	49.3986	Elvan	Eastern Kalguty	202	202	6.78	33.20	0.1234	0.512280	6	-0.37	-6.98	-5.09	-5.09	-5.09	1.39	1.47	1.39	1.47	1.40	Annikova IY et al.	2006	Petrology	14(1)	81–97
3927	1-259	CAOB	Southern Gornyi Altai	89.0639	49.3986	Elvan	Eastern Kalguty	202	202	7.06	34.60	0.1233	0.512351	6	-0.37	-5.60	-3.71	-3.71	-3.71	1.28	1.35	1.28	1.35	1.29	Annikova IY et al.	2006	Petrology	14(1)	81–97
3928	12HS05-4	CAOB	Kazakhstan	72.9978	50.1743	Diorite		440	440	4.03	18.30	0.1341	0.512756		-0.32	2.30	5.83	5.81	5.81	0.70	0.76	0.70			Shen P et al.	2016	Journal of Asian Earth Sciences	116	232-248
3929	12HS05-1	CAOB	Kazakhstan	72.9978	50.1743	Granodiorite		440	440	2.56	11.46	0.1361	0.512765		-0.31	2.48	5.89	5.88	5.88	0.70	0.76	0.70			Shen P et al.	2016	Journal of Asian Earth Sciences	116	232-248
3930	12HS04-4	CAOB	Kazakhstan	72.9978	50.1743	Tonalite		440	440	2.79	12.88	0.1319	0.512754		-0.33	2.26	5.91	5.90	5.90	0.70	0.74	0.70			Shen P et al.	2016	Journal of Asian Earth Sciences	116	232-248
3931	12HS01-2	CAOB	Kazakhstan	72.9978	50.1743	Tonalite		437	437	3.73	16.09	0.1412	0.512779		-0.28	2.75	5.86	5.85	5.85	0.70	0.78	0.70			Shen P et al.	2016	Journal of Asian Earth Sciences	116	232-248
3932	AES-6-3	CAOB	SMXAB	120.0917	47.1183	hyraceous alkali feldspar g	Nanxingan	142	150	2.38	13.10	0.1098	0.512718	20	-0.44	1.56	3.14	3.22	3.22	0.68	0.63	0.68	0.64	0.68	Ynag QD.	2014	Chinese Academy of Geological Science		1-219
3933	AES-6-1	CAOB	SMXAB	120.0500	47.1033	hyraceous alkali feldspar g	Nanxingan	142	150	1.17	9.96	0.0710	0.512638	6	-0.64	0.00	2.28	2.41	2.41	0.74	0.55	0.74	0.55	0.75	Ynag QD.	2014	Chinese Academy of Geological Science		1-219
3934	Rudnev20139	CAOB	Altai	93.2910	50.5010	Granite		522	522	3.61	14.27	0.1541	0.512839		-0.22	3.92	6.77	6.76	6.76	0.69	0.80	0.69			Rudnev SN et al.	2013	Russian Geology and Geophysics	54(10)	1272-1287
3935	Rudnev20138	CAOB	Altai	93.2910	50.5010	Tonalite		518	518	3.48	8.21	0.2582	0.513163		0.31	10.24	6.18	6.18	6.18	0.74	0.04	0.74			Rudnev SN et al.	2013	Russian Geology and Geophysics	54(10)	1272-1287
3936	Rudnev20137	CAOB	Altai	95.0830	51.6910	Tonalite		536	536	2.98	8.60	0.2111	0.513012		0.07	7.30	6.32	6.31	6.31	0.73	7.77	0.73			Rudnev SN et al.	2013	Russian Geology and Geophysics	54(10)	1272-1287
3937	Rudnev20135	CAOB	Altai	95.0420	51.8420	Diorite		562	562	2.08	5.36	0.2364	0.513107		0.20	9.15	6.31	6.30	6.30	0.76	-0.29	0.76			Rudnev SN et al.	2013	Russian Geology and Geophysics	54(10)	1272-1287
3938	Rudnev20134	CAOB	Altai	91.6420	52.7930	Diorite		524	524	1.46	6.04	0.1472	0.512720		-0.25	1.60	4.92	4.91	4.91	0.84	0.99	0.84			Rudnev SN et al.	2013	Russian Geology and Geophysics	54(10)	1272-1287
3939	Rudnev20133	CAOB	Altai	89.7120	52.0340	Diorite		545	545	7.72	35.30	0.1332	0.512760		-0.32	2.38	6.81	6.80	6.80	0.71	0.74	0.71			Rudnev SN et al.	2013	Russian Geology and Geophysics	54(10)	1272-1287
3940	Rudnev201321	CAOB	Altai	95.4390	46.3820	Granite		530	530	2.76	13.93	0.1207	0.512753		-0.39	2.24	7.40	7.38	7.38	0.65	0.65	0.65			Rudnev SN et al.	2013	Russian Geology and Geophysics	54(10)	1272-1287
3941	Rudnev201320	CAOB	Altai	93.6460	48.4210	Granite		514	514	6.78	23.61	0.1749	0.512966		-0.11	6.40	7.84	7.83	7.83	0.60	0.72	0.60			Rudnev SN et al.	2013	Russian Geology and Geophysics	54(10)	1272-1287
3942	Rudnev20132	CAOB	Altai	88.4400	51.8690	Diorite		545	545	0.71	4.13	0.1047	0.512692		-0.47	1.05	7.47	7.45	7.45	0.65	0.64	0.65			Rudnev SN et al.	2013	Russian Geology and Geophysics	54(10)	1272-1287
3943	Rudnev201319	CAOB	Altai	93.5100	48.3530	Granite		514	514	2.05	9.51	0.1313	0.512854		-0.33	4.21	8.52	8.50	8.50	0.54	0.55	0.54			Rudnev SN et al.	2013	Russian Geology and Geophysics	54(10)	1272-1287
3944	Rudnev201318	CAOB	Altai	93.4550	48.4620	Granite		514	514	2.22	10.14	0.1333	0.512884		-0.32	4.80	8.97	8.95	8.95	0.51	0.51	0.51			Rudnev SN et al.	2013	Russian Geology and Geophysics	54(10)	1272-1287
3945	Rudnev201317	CAOB	Altai	93.4550	48.4620	Granite		514	514	2.20	10.34	0.1296	0.512865		-0.34	4.43	8.85	8.83	8.83	0.52	0.52	0.52			Rudnev SN et al.	2013	Russian Geology and Geophysics	54(10)	1272-1287
3946	Rudnev201316	CAOB	Altai	93.8240	48.8040	Granite		519	519	2.76	13.02	0.1291	0.512742		-0.34	2.03	6.52	6.50	6.50	0.71	0.74	0.71			Rudnev SN et al.	2013	Russian Geology and Geophysics	54(10)	1272-1287
3947	Rudnev201315	CAOB	Altai	93.9470	47.4770	Granite		524	524	1.99	9.68	0.1252	0.512745		-0.36	2.09	6.88	6.87	6.87	0.69	0.70	0.69			Rudnev SN et al.	2013	Russian Geology and Geophysics	54(10)	1272-1287
3948	Rudnev201314	CAOB	Altai	94.3030	47.6690	Granite		535	535	0.43	2.00	0.1310	0.512859		-0.33	4.31	8.82	8.80	8.80	0.54	0.54	0.54			Rudnev SN et al.	2013	Russian Geology and Geophysics	54(10)	1272-1287
3949	Rudnev201313	CAOB	Altai	94.1390	47.5600	Granite		551	551	2.89	11.06	0.1592	0.512887		-0.19	4.86	7.51	7.50	7.50	0.66	0.74	0.66			Rudnev SN et al.	2013	Russian Geology and Geophysics	54(10)	1272-1287
3950	Rudnev201312	CAOB	Altai	93.2360	48.1610	Granite		531	531	0.70	3.40	0.1254	0.512768		-0.36	2.54	7.38	7.37	7.37	0.65	0.66	0.65			Rudnev SN et al.	2013	Russian Geology and Geophysics	54(10)	1272-1287
3951	Rudnev201311	CAOB	Altai	93.5920	48.1750	Granite		529	529	1.66	6.23	0.1623	0.512921		-0.17	5.52	7.86	7.84	7.84	0.61	0.68	0.61			Rudnev SN et al.	2013	Russian Geology and Geophysics	54(10)	1272-1287
3952	Rudnev201310	CAOB	Altai	94.1660	50.6000	Granite		518	518	6.23	22.10	0.1717	0.512900		-0.13	5.11	6.77	6.76	6.76	0.69	0.91	0.69			Rudnev SN et al.	2013	Russian Geology and Geophysics	54(10)	1272-1287
3953	Rudnev20131	CAOB	Altai	87.8650	55.4610	Tonalite		532	532	1.83	5.71	0.1952	0.512869		-0.01	4.51	4.61	4.61	4.61	0.87	2.31	0.87			Rudnev SN et al.	2013	Russian Geology and Geophysics	54(10)	1272-1287
3954	GLS11	CAOB	SMXAB	111.2597	43.2678	Granite	Guilesitai	138	150	4.74	28.72	0.0998	0.512347	7	-0.49	-5.68	-3.97	-3.82	-3.82	1.25	1.07	1.25	1.08	1.26	Xu DQ et al.	2008	Acta Petrologica et Mineralogica	27(2)	89-100
3955	GLS09	CAOB	SMXAB	111.2597	43.2678	Granite	Guilesitai	138	150	4.57	28.31	0.0977	0.512340	4	-0.50	-5.81	-4.07	-3.92	-3.92	1.26	1.06	1.26	1.07	1.26	Xu DQ et al.	2008	Acta Petrologica et Mineralogica	27(2)	89-100
3956	GLS08	CAOB	SMXAB	111.2597	43.2678	Granite	Guilesitai	138	150	4.32	26.87	0.0972	0.512383	16	-0.51	-4.97	-3.23	-3.07	-3.07	1.19	1.00	1.19	1.01	1.19	Xu DQ et al.	2008	Acta Petrologica et Mineralogica	27(2)	89-100
3957	GLS06	CAOB	SMXAB	111.1667	43.0667	Granite	Aobaotu	138	150	5.27	41.91	0.0760	0.512293	5	-0.61	-6.73	-4.61	-4.42	-4.42	1.30	0.95	1.30	0.95	1.31	Xu DQ et al.	2008	Acta Petrologica et Mineralogica	27(2)	89-100
3958	GLS05	CAOB	SMXAB	111.1667	43.0667	Granite	Aobaotu	138	150	4.51	33.85	0.0806	0.512295	5	-0.59	-6.69	-4.65	-4.47	-4.47	1.30	0.98	1.30	0.98	1.31	Xu DQ et al.	2008	Acta Petrologica et Mineralogica	27(2)	89-100
3959	ABi07	CAOB	SMXAB	111.2597	43.2678	Porphyric granite	Guilesitai	138	150	2.40	17.16	0.0845	0.512576	16	-0.57	-1.21	0.77	0.94	0.94	0.86	0.68	0.86	0.68	0.87	Xu DQ et al.	2008	Acta Petrologica et Mineralogica	27(2)	89-100
3960	ABi03	CAOB	SMXAB	111.1667	43.0667	Granite-porphyry	Aobaotu	138	150	5.97	42.40	0.0851	0.512449	3	-0.57	-3.69	-1.72	-1.55	-1.55	1.07	0.83	1.07	0.83	1.07	Xu DQ et al.	2008	Acta Petrologica et Mineralogica	27(2)	89-100
3961	2012SM-325	CAOB	Transbaikalia and Mongolia	102.4333	42.7500	Rhyolite		326	326	3.70	18.93	0.1185	0.512758		-0.40	2.34	5.59	5.59	5.59	0.63	0.63	0.63	0.63	0.63	Ynag SH et al.	2016	Journal of Asian Earth Sciences	120	29-42
3962	2012SM-319	CAOB	Transbaikalia and Mongolia	102.7100	42.7333	Rhyolite		326	326	4.60	22.96	0.1212	0.512716		-0.38	1.52	4.66	4.66	4.66	0.70	0.72	0.70	0.72	0.70	Ynag SH et al.	2016	Journal of Asian Earth Sciences	120	29-42
3963	7340	CAOB	Transbaikalia and Mongolia	99.7333	48.6500	Ider Complex		2540	600	3.29	16.19	0.1229	0.511313		-0.38	-25.85	-1.93	-20.2	-20.2	2.94	3.06	2.94	3.06	3.04	Kröner A et al.	2015	Gondwana Research	27(4)	1392-1406
3964	7011	CAOB	Transbaikalia and Mongolia	99.4667	48.6833	Ider Complex		1855	600	0.22	2.37	0.0561	0.510532		-0.71	-41.08	-7.80	-30.3	-30.3	3.76	2.52	3.76	2.52	2.95	Kröner A et al.	2015	Gondwana Research	27(4)	1392-1406
3965	6658	CAOB	Transbaikalia and Mongolia	99.5667	48.1667	Ider Complex		2540	600	4.74	27.93	0.1025	0.511042		-0.48	-31.13	-0.60	-23.9	-23.9	3.24	2.87	3.24	2.87	2.93	Kröner A et al.	2015	Gondwana Research	27(4)	1392-1406
3966	6657	CAOB	Transbaikalia and Mongolia	99.6000	48.1333	Ider Complex		2540	600	4.97	25.01	0.1202	0.511369		-0.39	-24.75	0.04	-18.9	-18.9	2.83	2.89	2.83	2.89	2.88	Kröner A et al.	2015	Gondwana Research	27(4)	1392-1406
3967	6153	CAOB	Transbaikalia and Mongolia	99.4167	48.7067	Ider Complex		2219	600	1.44	10.92	0.0797	0.510698		-0.59	-37.84	-4.71	-28.9	-28.9	3.64	2.77	3.64	2.77	2.99	Kröner A et al.	2015	Gondwana Research	27(4)	1392-1406
3968	6152	CAOB	Transbaikalia and Mongolia	99.4500	48.6967	Ider Complex		2540	600	2.75	22.64	0.0733	0.510647		-0.63	-38.84	1.16	-29.4	-29.4	3.69	2.70	3.69	2.70	2.78	Kröner A et al.	2015	Gondwana Research	27(4)	1392-1406
3969	6151	CAOB	Transbaikalia and Mongolia	99.4600	48.7000	Ider Complex		2540	600	3.76	20.07	0.0685	0.510570		-0.65	-40.34	1.21	-30.5	-30.5	3.78	2.69	3.78	2.69	2.77	Kröner A et al.	2015	Gondwana Research	27(4)	1392-1406
3970	A1455	CAOB	Tianshan	79.0744	42.2062	Granite		2333	600	11.24	70.40	0.0972	0.511041		-0.51	-31.15	-1.33	-23.5	-23.5	3.21	2.74	3.21			Kröner A et al.	2017	Journal of Asian Earth Sciences	135	122-135
3971	A1453	CAOB	Tianshan	79.0719	42.2067	Dacite		1830	600	30.60	276.00	0.0675	0.510884		-0.66	-34.22	-3.90	-24.3	-24.3	3.28	2.35	3.28			Kröner A et al.	2017	Journal of Asian Earth Sciences	135	122-135
3972	KG-5	CAOB	Tianshan	76.1347	42.8819	Granite		834	600	4.79	28.77	0.1014	0.511872		-0.48	-14.94	-4.79	-7.65	-7.65	1.93	1.73	1.93			Kröner A et al.	2012	Gondwana Research	21(4)	901-927
3973	KG-38	CAOB	Tianshan	76.0258	42.8034	Rhyolite		472	472	2.81	12.43	0.1379	0.512357		-0.30	-5.48	-1.94	-1.94	-1.94	1.36	1.59	1.36			Kröner A et al.	2012	Gondwana Research	21(4)	901-927
3974	KG-37	CAOB	Tianshan	76.0258	42.8034	Granite		472	472	2.23	10.63	0.1278	0.512297		-0.35	-6.65	-2.50	-2.50	-2.50	1.40	1.51	1.40			Kröner A et al.	2012	Gondwana Research	21(4)	901-927
3975	KG-3	CAOB	Tianshan	76.1347	42.8819	Diorite		562	562	10.25	50.77	0.1230	0.512115		-0.37	-10.20	-4.91	-4.91	-4.91	1.67	1.73	1.67			Kröner A et al.	2012	Gondwana Research	21(4)	901-927
3976	KG-2	CAOB	Tianshan	75.8837	42.6925	Diorite		810	600	4.83	29.87	0.0985	0.511668		-0.50	-18.92	-8.76	-11.4	-11.4	2.23	1.95	2.23			Kröner A et al.	2012	Gondwana Research	21(4)	901-927
3977	KG-11	CAOB	Tianshan	75.8898	42.6851	Andesite		810	600	5.10	29.64	0.1048	0.511514		-0.47	-21.93	-12.4	-14.9	-14.9	2.51	2.28	2.51			Kröner A et al.	2012	Gondwana Research	21(4)	901-927
3978	KG-1	CAOB	Tianshan	75.8819	42.6928	Diorite		844	600	5.88	34.76	0.1029	0.511543		-0.48	-21.36	-11.3	-14.2	-14.2	2.46	2.20	2.46			Kröner A et al.	2012	Gondwana Research	21(4)	901-927
3979	X-544	CAOB	Altai, unit 7	94.7961	43.2544	Bi granite	Yiwu	284	284	2.67	11.84	0.1365	0.512764	12	-0.31	2.46	4.64	4.64	4.64	0.67	0.76	0.67		0.76	Wang CS et al.	2010	Acta Petrologica Sinica	26(4)	1045-1058
3980	X-538	CAOB	Altai, unit 7	94.8258	43.3031	Bi granite	Yiwu	285	285	4.80	23.91	0.1213	0.512730	12	-0.38	1.79	4.53	4.54	4.54	0.68	0.69	0.68		0.69	Wang CS et al.	2010	Acta Petrologica Sinica	26(4)	1045-1058
3981	CG7	CAOB	SMXAB	118.3108	46.0078	Qtz diorite	Chaganaobao	237	237			0.0671	0.512605		-0.66	-0.64	3.28	3.28	3.28	0.74	0.57	0.74	0.57	0.75	Zhang, WY et al.	2008	Acta Petrologica et Mineralogica	27(3)	177-184
3982	CG6	CAOB	SMXAB	118.3108	46.0078	Qtz diorite	Chaganaobao	237	237			0.0674	0.512631		-0.66	-0.14	3.77	3.77	3.77	0.70	0.54	0.70	0.54	0.71	Zhang, WY et al.	2009	Acta Petrologica et Mineralogica	27(3)	177-185
3983	CG2	CAOB	SMXAB	118.3108	46.0078	Qtz diorite	Chaganaobao	237	237			0.0679	0.512620		-0.65	-0.35	3.55	3.54	3.54	0.72	0.55	0.72	0.56	0.72	Zhang, WY et al.	2010	Acta Petrologica et Mineralogica	27(3)	177-186
3984	NM10-51	CAOB	North of Sonlonker suture	113.2608	44.3700	Rhyolite	Bayanwula	310	310	3.86	19.20	0.1215	0.512685	8	-0.38	0.92	3.85	3.89	3.89	0.75	0.77	0.75	0.77		Li K et al.	2014	Acta Petrologica Sinica	79(2)	256-261
3985	NM10-48	CAOB	North of Sonlonker suture	113.1047	44.4549	Rhyolite	Bayanwula	309	309	3.28	17.80	0.1114	0.512751	9	-0.43	2.20	5.54	5.57	5.57	0.62	0.60	0.62	0.60		Li K et al.	2014	Acta Petrologica Sinica	79(2)	256-261
3986	NM10-26	CAOB	North of Sonlonker suture	113.1047	44.4549	Rhyolite	Bayanwula	309	309	4.13	22.40	0.1115	0.512645	8	-0.43	0.14	3.46	3.50	3.50	0.78	0.75	0.78	0.75		Li K et al.	2014	Acta Petrologica Sinica	79(2)	256-261
3987	NM10-25	CAOB	North of Sonlonker suture	113.1047	44.4549	Rhyolite	Bayanwula	309	309	4.22	23.50	0.1086	0.512643	9	-0.45	0.10	3.54	3.57	3.57	0.78	0.74	0.78	0.74		Li K et al.	2014	Acta Petrologica Sinica	79(2)	256-261
3988	DW-87	CAOB	SMXAB	117.6882	46.0612	Adamellite	Xidunhuanaote	297	297	2.67	15.14	0.1064	0.512629		-0.46	-0.18	3.25	3.25	3.25	0.79	0.74	0.79	0.74	0.80	Wang ZH et al.	2013	Journal Mineral Petral	33	72-84
3989	DW-86	CAOB	SMXAB	117.6882	46.0612	Adamellite	Xidunhuanaote	297	297	5.71	28.44	0.1213	0.512647		-0.38	0.18	3.03	3.03	3.03	0.81	0.83	0.81	0.83	0.81	Wang ZH et al.	2013	Journal Mineral Petral	33	72-84
3990	DW-84	CAOB	SMXAB	117.6882	46.0612	Adamellite	Xidunhuanaote	297	297	0.56	1.67	0.2042	0.512673		0.04	0.68	0.40	0.40	0.40	1.00	7.49	1.00	7.58	1.03	Wang ZH et al.	2013	Journal Mineral Petral	33	72-84
3991	DW-83	CAOB	SMXAB	117.6882	46.0612	Adamellite	Xidunhuanaote	297	297	6.92	34.24	0.1221	0.512648		-0.38	0.20	3.03	3.02	3.02	0.81	0.84	0.81	0.84	0.82	Wang ZH et al.	2013	Journal Mineral Petral	33	72-84
3992	DW-81	CAOB	SMXAB	117.6882	46.0612	Adamellite	Xidunhuanaote	297	297	5.54	27.98	0.1197	0.512645		-0.39	0.14	3.05	3.05	3.05	0.81	0.82	0.81	0.82	0.81	Wang ZH et al.	2013	Journal Mineral Petral	33	72-84
3993	DW-133	CAOB	SMXAB	117.6882	46.0612	Adamellite	Xidunhuanaote	297	297	6.25	23.98	0.1575	0.512772		-0.20	2.61	4.11	4.10	4.10	0.72	1.02	0.72	1.03	0.73	Wang ZH et al.	2013	Journal Mineral Petral	33	72-84
3994	DW-132	CAOB	SMXAB	117.6882	46.0612	Adamellite	Xidunhuanaote	297	297	8.70	43.85	0.1200	0.512720		-0.39	1.60	4.52	4.51	4.51	0.69	0.70	0.69	0.70	0.69	Wang ZH et al.	2013	Journal Mineral Petral	33	72-84
3995	DW-131	CAOB	SMXAB	117.6882	46.0612	Adamellite	Xidunhuanaote	297	297	7.75	39.71	0.1181	0.512717		-0.40	1.54	4.52	4.52	4.52	0.69	0.69	0.69	0.69	0.69	Wang ZH et al.	2013	Journal Mineral Petral	33	72-84
3996	DW-114	CAOB	SMXAB	117.6882	46.0612	Adamellite	Xidunhuanaote	297	297	8.43	322.08	0.1582	0.512770		-0.20	2.57	4.03	4.03	4.03	0.73	1.04	0.73	1.05	0.73	Wang ZH et al.	2013	Journal Mineral Petral	33	72-84
3997	DW-113	CAOB	SMXAB	117.6882	46.0612	Adamellite	Xidunhuanaote	297	297	2.77	8.56	0.1957	0.512821		-0.01	3.57	3.62	3.61	3.61	0.76	2.77	0.76	2.79	0.77	Wang ZH et al.	2013	Journal Mineral Petral	33	72-84
3998	966056-2	CAOB	tau-borokonu mountain, Tians	81.5000	44.5000	Granite gneiss	Tianchuan	798	600	6.85	29.38	0.1409	0.512021	30	-0.28	-12.04	-6.35	-7.76	-7.76	1.93	2.35	1.93		1.98	Chen YB et al.	1999	Geochimica	28(6)	515-520
3999	92II-8	CAOB	Northern margin of Yili block	81.6422	44.3753	Granite gneiss	Tianchuang	798	600			0.1265	0.512040		-0.36	-11.67	-4.51	-6.29	-6.29	1.82	1.93	1.82	1.94	1.85	Chen YB et al.	1999	Geochimica	28(6)	515-520
4000	92II-7	CAOB	Northern margin of Yili block	81.6422	44.3753	Granite gneiss	Tianchuang	798	600			0.1273	0.512048		-0.35	-11.51	-4.43	-6.20	-6.20	1.81	1.94	1.81	1.94	1.84	Chen YB et al.	1999	Geochimica	28(6)	515-520
4001	92 ‖ -8	CAOB	tau-borokonu mountain, Tians	81.5000	44.5000	Granite gneiss	Tianchuan	798	600	6.70	32.01	0.1265	0.512040	7	-0.36	-11.67	-4.52	-6.29	-6.29	1.82	1.93	1.82		1.83	Chen YB et al.	1999	Geochimica	28(6)	515-520
4002	92 ‖ -7	CAOB	tau-borokonu mountain, Tians	81.5000	44.5000	Granite gneiss	Tianchuan	798	600	8.36	39.72	0.1273	0.512048	7	-0.35	-11.51	-4.44	-6.20	-6.20	1.81	1.94	1.81		1.83	Chen YB et al.	1999	Geochimica	28(6)	515-520
4003	HY9	CAOB	East Junggar	90.3333	45.1083	Granodiorite	Huangyangshan	305	305	8.42	36.46	0.1396	0.512853	6	-0.29	4.19	6.40	6.42	6.42	0.54	0.61	0.54		0.55	Su YP et al.	2008	Acta Mineralogica Sinica	28(2)	117-126
4004	HY42	CAOB	East Junggar	90.3722	45.0778	Granodiorite porphyry	Huangyangshan	305	305	6.62	26.51	0.1510	0.512849	6	-0.23	4.12	5.90	5.89	5.89	0.58	0.73	0.58		0.59	Su YP et al.	2008	Acta Mineralogica Sinica	28(2)	117-126
4005	HY13	CAOB	East Junggar	90.2833	45.1194	orphyritic K-feldspar granit	Huangyangshan	305	305	9.13	41.64	0.1326	0.512843	4	-0.33	4.00	6.50	6.49	6.49	0.54	0.58	0.54		0.54	Su YP et al.	2008	Acta Mineralogica Sinica	28(2)	117-126
4006	B70823-9	CAOB	Beishan	95.2444	41.0691	Qtz diorite	Huitongshan	420	420	4.40	24.02	0.1109	0.512360	11	-0.44	-5.42	-0.82	-0.82	-0.82	1.23	1.17	1.23	1.17	1.26	Li S et al.	2011	Acta Petrologica Sinica	122(4)	718-744
4007	B80206-5.1	CAOB	Beishan	95.1596	41.1318	K-Feldspar Granites	Huitongshan	397	397	9.41	40.99	0.1387	0.512425	12	-0.29	-4.15	-1.20	-1.22	-1.22	1.24	1.47	1.24	1.47	1.24	Li S et al.	2011	Acta Petrologica Sinica	122(4)	718-744
4008	B70823-6	CAOB	Beishan	95.1828	41.1147	K-Feldspar Granites	Huitongshan	397	397	10.27	59.38	0.1045	0.512463	12	-0.47	-3.41	1.20	1.26	1.26	1.04	0.96	1.04	0.96	1.04	Li S et al.	2011	Acta Petrologica Sinica	122(4)	718-744
4009	B70823-8	CAOB	Beishan	95.2317	41.1054	K-Feldspar Granites	Huitongshan	397	397	7.97	33.88	0.1423	0.512453	14	-0.28	-3.61	-1.30	-0.85	-0.85	1.21	1.49	1.21	1.53	1.24	Li S et al.	2011	Acta Petrologica Sinica	122(4)	718-744
4010	3-12	NCC	NCC	115.9503	39.7297	Granite-diorite	Fangshan	131	150	6.49	38.28	0.1025	0.511819	11	-0.48	-15.98	-14.4	-14.2	-14.2	2.09	1.82	2.09	1.82	2.10	Cai J et al.	2005	Acta Petrologica Sinica	21(3)	776-788
4011	3-2	NCC	NCC	115.9503	39.7297	Qtz diorite	Fangshan	131	150	6.66	37.01	0.1088	0.511858	11	-0.45	-15.22	-13.8	-13.5	-13.5	2.04	1.87	2.04	1.88	2.04	Cai J et al.	2005	Acta Petrologica Sinica	21(3)	776-788
4012	DJS03	CAOB	SMXAB	125.5833	50.2417	Granodiorite	Jinshuihe	179	179	7.72	37.88	0.1147	0.512615	8	-0.42	-0.45	1.43	1.42	1.42	0.85	0.82	0.85	0.83	0.85	Li YC et al.	2013	Geological Bulletin of China	32(5)	717-729
4013	DJGO1	CAOB	SMXAB	125.5833	50.2417	Adamellite	Jiguanshan	169	169	3.47	20.70	0.1015	0.512601	8	-0.48	-0.72	1.34	1.33	1.33	0.85	0.75	0.85	0.75	0.85	Li YC et al.	2013	Geological Bulletin of China	32(5)	717-729
4014	DJG05	CAOB	SMXAB	125.5833	50.2417	Granodiorite	Jiguanshan	169	169	4.44	24.32	0.1104	0.512664	18	-0.44	0.51	2.37	2.37	2.37	0.76	0.72	0.76	0.72	0.76	Li YC et al.	2013	Geological Bulletin of China	32(5)	717-729

4015	DJG03	CAOB	SMXAB	125.5833	50.2417	Adamellite	Jiguanshan	169	169	6.61	31.01	0.1290	0.512713	8	-0.34	1.46	2.92	2.92	2.92	0.72	0.79	0.72	0.79	0.72	Li YC et al.	2013	Geological Bulletin of China	32(5)	717-729
4016	A14830-1	CAOB	Altai, unit 3	90.7125	46.8233	Porphyry granodiorite	Butiou	385	385	7.49	34.50	0.1313	0.512446		-0.33	-3.75	-0.53	-0.53	-0.53	1.17	1.30	1.17	1.30	1.18	Song P et al.	2017	Acta Geologica Sinica	91(1)	55-79
4017	A14829-2	CAOB	Altai, unit 3	90.1666	47.0875	Granodiorite	Kungeyt	382	382	5.46	24.20	0.1364	0.512363		-0.31	-5.36	-2.42	-2.43	-2.43	1.32	1.55	1.32	1.55	1.33	Song P et al.	2017	Acta Geologica Sinica	91(1)	55-79
4018	08XM28	NCC	WNCC	110.2160	40.9840	anukitoids and related rock	Haolaigou	2466	600	0.87	9.19	0.0574	0.510397	11	-0.71	-43.72	0.21	-33.05	-33.05	3.98	2.67	3.98	2.67	2.80	Ma XD et al.	2013	Precambrian Research	230	61-78
4019	08XM20	NCC	WNCC	110.3096	40.9574	anukitoids and related rock	Dajitu	2523	600	3.11	27.82	0.0677	0.510585	10	-0.66	-40.05	1.56	-30.17	-30.17	3.75	2.66	3.75	2.66	2.74	Ma XD et al.	2013	Precambrian Research	230	61-78
4020	08XM16	NCC	WNCC	110.2160	40.9840	anukitoids and related rock	Dajitu	2523	600	7.85	41.11	0.1156	0.511382	9	-0.41	-24.50	1.63	-18.29	-18.29	2.79	2.73	2.79	2.73	2.74	Ma XD et al.	2013	Precambrian Research	230	61-78
4021	08XM12	NCC	WNCC	110.0369	40.1504	anukitoids and related rock	Haolaigou	2480	600	2.58	17.44	0.0897	0.510819	9	-0.54	-35.48	-1.57	-27.29	-27.29	3.51	2.85	3.51	2.85	2.96	Ma XD et al.	2013	Precambrian Research	230	61-78
4022	08XM11	NCC	WNCC	110.0369	40.1504	anukitoids and related rock	Haolaigou	2480	600	3.55	20.23	0.1063	0.511061	12	-0.46	-30.76	-2.12	-23.84	-23.84	3.23	2.95	3.23	2.95	3.00	Ma XD et al.	2013	Precambrian Research	230	61-78
4023	08XM06	NCC	WNCC	110.0369	40.1504	anukitoids and related rock	Haolaigou	2480	600	4.35	22.74	0.1157	0.511252	11	-0.41	-27.04	-1.38	-20.83	-20.83	2.99	2.93	2.99	2.93	2.94	Ma XD et al.	2013	Precambrian Research	230	61-78
4024	08XM04	NCC	WNCC	110.0369	40.1504	anukitoids and related rock	Haolaigou	2480	600	4.43	22.35	0.1200	0.511307	13	-0.39	-25.96	-1.67	-20.09	-20.09	2.93	2.98	2.93	2.98	2.97	Ma XD et al.	2013	Precambrian Research	230	61-78
4025	07GY23	NCC	WNCC	110.3096	40.9574	anukitoids and related rock	Dajitu	2523	600	1.82	12.87	0.0855	0.510873	11	-0.57	-34.43	1.43	-25.92	-25.92	3.40	2.69	3.40	2.69	2.75	Ma XD et al.	2013	Precambrian Research	230	61-78
4026	07GY22	NCC	WNCC	110.3096	40.9574	anukitoids and related rock	Dajitu	2523	600	3.72	22.45	0.1004	0.511119	9	-0.49	-29.63	1.41	-22.26	-22.26	3.11	2.72	3.11	2.72	2.75	Ma XD et al.	2013	Precambrian Research	230	61-78
4027	07GY21	NCC	WNCC	110.3096	40.9574	anukitoids and related rock	Haolaigou	2466	600	1.53	11.51	0.0802	0.510778	12	-0.59	-36.28	0.44	-27.36	-27.36	3.52	2.69	3.52	2.69	2.78	Ma XD et al.	2013	Precambrian Research	230	61-78
4028	07GY15	NCC	WNCC	110.2160	40.9840	anukitoids and related rock	Haolaigou	2466	600	2.77	18.91	0.0885	0.510918	10	-0.55	-33.55	0.55	-25.27	-25.27	3.35	2.70	3.35	2.70	2.77	Ma XD et al.	2013	Precambrian Research	230	61-78
4029	ZK0408-23	CAOB	Eastern Tianshan	93.3500	41.9167	Porphyritic granite		236	236	22.30	4.68	0.1269	0.512596		-0.35	-0.82	1.23	1.28	1.28	0.90	0.97	0.90	0.97	0.90	Wu YS et al.	2017	Ore Geology Reviews	81(Pt.2)	794-807
4030	WJL070204	Tarim	Tarim	78.9133	46.5528	Granite		290	290	5.91	40.01	0.0893	0.512634		-0.55	-0.08	3.88	3.90	3.90	0.74	0.63	0.74			Zou SY et al.	2015	Lithos	212-215	428-442
4031	WJL050712	Tarim	Tarim	78.9133	46.5528	Granite		290	290	8.56	47.70	0.1085	0.512520		-0.45	-2.30	0.94	0.96	0.96	0.98	0.91	0.98			Zou SY et al.	2015	Lithos	212-215	428-442
4032	WJL050711	Tarim	Tarim	78.9133	46.5528	Granite		290	290	6.51	37.44	0.1051	0.512478		-0.47	-3.12	0.24	0.27	0.27	1.03	0.94	1.03			Zou SY et al.	2015	Lithos	212-215	428-442
4033	WJL050710	Tarim	Tarim	78.9133	46.5528	Granite		290	290	8.33	46.26	0.1089	0.512517		-0.45	-2.36	0.86	0.89	0.89	0.98	0.92	0.98			Zou SY et al.	2015	Lithos	212-215	428-442
4034	WJL050605-4	Tarim	Tarim	78.9133	46.5528	Granite		290	290	10.91	71.88	0.0917	0.512597		-0.53	-0.80	3.06	3.08	3.08	0.80	0.69	0.80			Zou SY et al.	2015	Lithos	212-215	428-442
4035	WJL050605-3	Tarim	Tarim	78.9133	46.5528	Granite		290	290	12.80	76.91	0.1006	0.512648		-0.49	0.20	3.73	3.75	3.75	0.75	0.68	0.75			Zou SY et al.	2015	Lithos	212-215	428-442
4036	WJL050605-2	Tarim	Tarim	78.9133	46.5528	Granite		290	290	10.88	73.33	0.0897	0.512631		-0.54	-0.14	3.80	3.82	3.82	0.74	0.64	0.74			Zou SY et al.	2015	Lithos	212-215	428-442
4037	WJL050605-1	Tarim	Tarim	78.9133	46.5528	Granite		290	290	10.42	66.95	0.9407	0.512615		3.78	-0.45	3.33	-27.98	-27.98	3.35	-0.11	3.35			Zou SY et al.	2015	Lithos	212-215	428-442
4038	SKG-9	CAOB	SMXAB	125.7119	50.6411	Granodiorite	Sankuanggou	176	176	4.43	23.90	0.1122	0.512701	9	-0.43	1.23	3.13	3.13	3.13	0.71	0.67	0.71	0.68	0.71	Chu SX et al.	2012	Acta Petrologica Sinica	28(2)	433-450
4039	SKG-4	CAOB	SMXAB	125.7119	50.6411	Granodiorite	Sankuanggou	176	176	4.05	22.80	0.1072	0.512699	8	-0.46	1.19	3.21	3.20	3.20	0.70	0.65	0.70	0.65	0.70	Chu SX et al.	2012	Acta Petrologica Sinica	28(2)	433-450
4040	SKG-16	CAOB	SMXAB	125.7119	50.6411	Granodiorite	Sankuanggou	176	176	3.99	21.70	0.1115	0.512693	9	-0.43	1.07	3.00	2.99	2.99	0.72	0.68	0.72	0.68	0.72	Chu SX et al.	2012	Acta Petrologica Sinica	28(2)	433-450
4041	SKG-15	CAOB	SMXAB	125.7119	50.6411	Granodiorite	Sankuanggou	176	176	4.41	24.10	0.1103	0.512737	8	-0.44	1.93	3.87	3.87	3.87	0.64	0.61	0.64	0.61	0.65	Chu SX et al.	2012	Acta Petrologica Sinica	28(2)	433-450
4042	SKG-10	CAOB	SMXAB	125.7119	50.6411	Granodiorite	Sankuanggou	176	176	3.61	20.60	0.1062	0.512678	7	-0.46	0.78	2.82	2.81	2.81	0.73	0.67	0.73	0.67	0.73	Chu SX et al.	2012	Acta Petrologica Sinica	28(2)	433-450
4043	7HZGNZK11-1-890	CAOB	Baogutu, West Junggar	86.5833	47.0833	Granodiorite	Hanzhenaineng	340	340			0.1258	0.512835		-0.36	3.84	6.92	6.92	6.92	0.53	0.55	0.53	0.55	0.53	Guo ZL et al.	2010	Acta Petrologica Sinica	26(12)	3563-3578
4044	7HZGNZK0-2-820	CAOB	Baogutu, West Junggar	86.5833	47.0833	Qtz monzogranite	Hanzhenaineng	340	340			0.1100	0.512827		-0.44	3.69	7.45	7.45	7.45	0.49	0.48	0.49	0.48	0.49	Guo ZL et al.	2010	Acta Petrologica Sinica	26(12)	3563-3578
4045	7HZGN-3	CAOB	Baogutu, West Junggar	86.5833	47.0833	Monzogranite	Hanzhenaineng	340	340			0.0973	0.512758		-0.51	2.34	6.65	6.65	6.65	0.55	0.51	0.55	0.51	0.55	Guo ZL et al.	2010	Acta Petrologica Sinica	26(12)	3563-3578
4046	7HZGN-2	CAOB	Baogutu, West Junggar	86.5833	47.0833	Granodiorite	Hanzhenaineng	340	340			0.1358	0.512867		-0.31	4.47	7.11	7.11	7.11	0.51	0.55	0.51	0.55	0.51	Guo ZL et al.	2010	Acta Petrologica Sinica	26(12)	3563-3578
4047	7HZCN-5	CAOB	Baogutu, West Junggar	86.5833	47.0833	Fine grained diorite	Hanzhenaineng	340	340			0.1423	0.512900		-0.28	5.11	7.47	7.47	7.47	0.49	0.53	0.49	0.53	0.49	Guo ZL et al.	2010	Acta Petrologica Sinica	26(12)	3563-3578
4048	YTS9	CAOB	Tianshan	82.4833	43.7333	Granite		269	269	1.86	12.27	0.0917	0.512625		-0.53	-0.25	3.36	3.35	3.35	0.76	0.66	0.76			Li NB et al.	2013	Acta Petrologica Sinica	29(10)	3402-3412
4049	YTS7	CAOB	Tianshan	82.4833	43.7333	Granite		269	269	1.81	12.24	0.0892	0.512623		-0.55	-0.29	3.42	3.40	3.40	0.76	0.65	0.76			Li NB et al.	2013	Acta Petrologica Sinica	29(10)	3402-3412
4050	YTS6	CAOB	Tianshan	82.4833	43.7333	Granite		269	269	1.68	10.96	0.0924	0.512614		-0.53	-0.47	3.14	3.11	3.11	0.78	0.67	0.78			Li NB et al.	2013	Acta Petrologica Sinica	29(10)	3402-3412
4051	YTS12	CAOB	Tianshan	82.4833	43.7333	Granite		269	269	2.04	14.63	0.0844	0.512610		-0.57	-0.55	3.34	3.31	3.31	0.77	0.64	0.77			Li NB et al.	2013	Acta Petrologica Sinica	29(10)	3402-3412
4052	YTS11	CAOB	Tianshan	82.4833	43.7333	Granite		269	269	1.86	12.11	0.0926	0.512630		-0.53	-0.16	3.43	3.42	3.42	0.76	0.66	0.76			Li NB et al.	2013	Acta Petrologica Sinica	29(10)	3402-3412
4053	TST087-3	CAOB	Western Tianshan	83.0000	44.0000	Dacite		354	354	3.79	18.88	0.1223	0.512581		-0.38	-1.11	2.26	2.25	2.25	0.92	0.95	0.92			Yu XQ et al.	2016	International Journal of Earth Sciences	105(5)	1637-1661
4054	TST087-2	CAOB	Western Tianshan	83.0000	44.0000	Dacite		354	354	3.68	18.09	0.1239	0.512497		-0.37	-2.75	0.54	0.54	0.54	1.06	1.11	1.06			Yu XQ et al.	2016	International Journal of Earth Sciences	105(5)	1637-1661
4055	TST087-1	CAOB	Western Tianshan	83.0000	44.0000	Andesite		354	354	4.56	22.64	0.1227	0.512516		-0.38	-2.38	0.97	0.96	0.96	1.03	1.06	1.03			Yu XQ et al.	2016	International Journal of Earth Sciences	105(5)	1637-1661
4056	TST086-1	CAOB	Western Tianshan	83.0000	44.0000	Dacite		354	354	6.28	30.30	0.1262	0.512606		-0.36	-0.62	2.56	2.56	2.56	0.90	0.95	0.90			Yu XQ et al.	2016	International Journal of Earth Sciences	105(5)	1637-1661
4057	TST086-0	CAOB	Western Tianshan	83.0000	44.0000	Dacite		354	354	5.82	26.53	0.1336	0.512475		-0.32	-3.18	-0.33	-0.33	-0.33	1.13	1.28	1.13			Yu XQ et al.	2016	International Journal of Earth Sciences	105(5)	1637-1661
4058	TST085-1	CAOB	Western Tianshan	83.0000	44.0000	Andesite		367	367	7.13	37.97	0.1144	0.512477		-0.42	-3.14	0.72	0.71	0.71	1.06	1.03	1.06			Yu XQ et al.	2016	International Journal of Earth Sciences	105(5)	1637-1661
4059	TST083-1	CAOB	Western Tianshan	83.0000	44.0000	Andesite		367	367	4.86	29.87	0.0991	0.512455		-0.50	-3.57	1.01	1.00	1.00	1.03	0.92	1.03			Yu XQ et al.	2016	International Journal of Earth Sciences	105(5)	1637-1661
4060	TST076-1	CAOB	Western Tianshan	88.0000	44.0000	Andesite		375	375	6.85	31.54	0.1323	0.512415		-0.33	-4.35	-1.27	-1.27	-1.27	1.23	1.37	1.23			Yu XQ et al.	2016	International Journal of Earth Sciences	105(5)	1637-1661
4061	TST075-1	CAOB	Western Tianshan	88.0000	44.0000	Andesite		375	375	7.16	38.16	0.1143	0.512403		-0.42	-4.58	-0.64	-0.64	-0.64	1.17	1.14	1.17			Yu XQ et al.	2016	International Journal of Earth Sciences	105(5)	1637-1661
4062	TST072-3	CAOB	Western Tianshan	88.0000	44.0000	Andesite		375	375	5.28	25.40	0.1266	0.512451		-0.36	-3.65	-0.29	-0.29	-0.29	1.15	1.22	1.15			Yu XQ et al.	2016	International Journal of Earth Sciences	105(5)	1637-1661
4063	TST072-2	CAOB	Western Tianshan	88.0000	44.0000	Andesite		375	375	4.77	22.95	0.1266	0.512449		-0.36	-3.69	-0.33	-0.33	-0.33	1.15	1.23	1.15			Yu XQ et al.	2016	International Journal of Earth Sciences	105(5)	1637-1661
4064	SB17	CAOB	SHB	118.9222	44.0772	Monzogranite	Shabutai	138	150			0.1442	0.512597		-0.27	-0.80	0.13	0.21	0.21	0.92	1.21	0.92	1.22	0.92	Shu QH et al.	2015	Lithos	238	64-75
4065	SB16	CAOB	SHB	118.9222	44.0772	Monzogranite	Shabutai	138	150			0.1420	0.512597		-0.28	-0.80	0.17	0.25	0.25	0.92	1.17	0.92	1.18	0.92	Shu QH et al.	2015	Lithos	238	64-75
4066	SB15	CAOB	SHB	118.9222	44.0772	Monzogranite	Shabutai	138	150			0.1442	0.512611		-0.27	-0.53	0.40	0.48	0.48	0.90	1.18	0.90	1.19	0.90	Shu QH et al.	2015	Lithos	238	64-75
4067	SB14	CAOB	SHB	118.9222	44.0772	Monzogranite	Shabutai	138	150			0.1231	0.512580		-0.37	-1.13	0.17	0.28	0.28	0.92	0.96	0.92	0.96	0.92	Shu QH et al.	2015	Lithos	238	64-75
4068	SB12	CAOB	SHB	118.9222	44.0772	Monzogranite	Shabutai	138	150			0.1330	0.512592		-0.32	-0.90	0.23	0.32	0.32	0.91	1.05	0.91	1.06	0.91	Shu QH et al.	2015	Lithos	238	64-75
4069	SB11	CAOB	SHB	118.9222	44.0772	Monzogranite	Shabutai	138	150			0.1455	0.512596		-0.26	-0.82	0.09	0.16	0.16	0.92	1.24	0.92	1.24	0.92	Shu QH et al.	2015	Lithos	238	64-75
4070	SB04	CAOB	SHB	118.9222	44.0772	Monzogranite	Shabutai	138	150			0.1525	0.512609		-0.22	-0.57	0.21	0.28	0.28	0.91	1.35	0.91	1.35	0.91	Shu QH et al.	2015	Lithos	238	64-75
4071	SB03	CAOB	SHB	118.9222	44.0772	Monzogranite	Shabutai	138	150			0.1545	0.512610		-0.21	-0.55	0.19	0.26	0.26	0.92	1.39	0.92	1.39	0.91	Shu QH et al.	2015	Lithos	238	64-75
4072	YKH06	CAOB	Junggar	90.0198	46.5598	Qtz porphyry		348	348	2.03	9.50	0.1289	0.512837	14	-0.34	3.88	6.90	6.89	6.89	0.54	0.56	0.54	0.56	0.51	Yang FQ et al.	2014	Lithos	190–191(3)	85-103
4073	YKH05	CAOB	Junggar	90.0198	46.5598	Qtz porphyry		348	348	2.30	11.60	0.1196	0.512819	15	-0.39	3.53	7.00	6.95	6.95	0.53	0.54	0.53	0.54	0.53	Yang FQ et al.	2014	Lithos	190–191(3)	85-103
4074	YKH04	CAOB	Junggar	90.0198	46.5598	Qtz porphyry		348	348	2.19	10.60	0.1250	0.512821	15	-0.36	3.57	6.80	6.75	6.75	0.55	0.57	0.55	0.57	0.53	Yang FQ et al.	2014	Lithos	190–191(3)	85-103
4075	YKH03	CAOB	Junggar	90.0198	46.5598	Qtz porphyry		348	348	2.12	10.40	0.1230	0.512809	18	-0.37	3.34	6.60	6.61	6.61	0.56	0.57	0.56	0.57	0.55	Yang FQ et al.	2014	Lithos	190–191(3)	85-103
4076	YKH02	CAOB	Junggar	90.0198	46.5598	Qtz porphyry		348	348	1.95	9.20	0.1282	0.512827	13	-0.35	3.69	6.70	6.73	6.73	0.55	0.58	0.55	0.58	0.53	Yang FQ et al.	2014	Lithos	190–191(3)	85-103
4077	YHK01	CAOB	Junggar	90.0198	46.5598	Qtz porphyry		348	348	2.13	10.60	0.1218	0.512828	15	-0.38	3.71	7.00	7.03	7.03	0.53	0.53	0.53	0.54	0.52	Yang FQ et al.	2014	Lithos	190–191(3)	85-103
4078	259/1	CAOB	Altai, unit 4	88.4333	47.7667	covite bearing Bi monzogra	Lamazhao	276	276	6.21	30.01	0.1252	0.512655	18	-0.36	0.33	2.85	2.85	2.85	0.81	0.85	0.81	0.85	0.81	Wang T et al.	2005	Acta Petrologica Sinica	21(3)	640-650
4079	256/2	CAOB	Altai, unit 4	88.4333	47.7667	edium grained mica monz	Lamazhao	276	276	8.34	28.05	0.1798	0.512753	15	-0.09	2.24	2.84	2.84	2.84	0.81	1.78	0.81	1.78	0.81	Wang T et al.	2005	Acta Petrologica Sinica	21(3)	640-650
4080	258	CAOB	Altai, unit 4	88.4333	47.7667	Hb bearing Bi monzogranite	Lamazhao	276	276	7.09	28.17	0.1523	0.512630	12	-0.23	-0.16	1.41	1.41	1.41	0.93	1.29	0.93	1.29	0.93	Wang T et al.	2005	Acta Petrologica Sinica	21(3)	640-650
4081	JLG-6-1	CAOB	Beishan	127.0000	44.0000	Granodiorite	Daheishan	169	169	4.38	22.61	0.1172	0.512648		-0.40	0.20	1.90	1.91	1.91	0.80	0.79	0.80		0.80	Guo YP et al.	2018	Lithos	165	59-78
4082	JLG-6	CAOB	Beishan	127.0000	44.0000	Granodiorite	Daheishan	169	169	3.90	21.35	0.1107	0.512650		-0.44	0.23	2.10	2.09	2.09	0.78	0.74	0.78		0.79	Guo YP et al.	2018	Lithos	165	59-78
4083	JLG-5-1	CAOB	Beishan	127.0000	44.0000	Diorite	Daheishan	169	169	4.73	24.85	0.1151	0.512668		-0.41	0.59	2.40	2.34	2.34	0.76	0.75	0.76		0.77	Guo YP et al.	2018	Lithos	165	59-78
4084	JLG-5	CAOB	Beishan	127.0000	44.0000	Diorite	Daheishan	169	169	5.05	26.00	0.1175	0.512689		-0.40	0.99	2.70	2.70	2.70	0.73	0.73	0.73		0.74	Guo YP et al.	2018	Lithos	165	59-78
4085	JLG-4-1	CAOB	Beishan	127.0000	44.0000	Granodiorite	Daheishan	169	169	3.83	21.36	0.1087	0.512657		-0.45	0.37	2.30	2.27	2.27	0.77	0.72	0.77		0.77	Guo YP et al.	2018	Lithos	165	59-78
4086	JLG-4	CAOB	Beishan	127.0000	44.0000	Granodiorite	Daheishan	169	169	3.68	21.53	0.1036	0.512670		-0.47	0.62	2.60	2.63	2.63	0.74	0.67	0.74		0.74	Guo YP et al.	2018	Lithos	165	59-78
4087	JLG-3-1	CAOB	Beishan	127.0000	44.0000	Monzogranite	Daheishan	188	188	4.07	22.76	0.1083	0.512656		-0.45	0.35	2.30	2.47	2.47	0.77	0.71	0.77		0.77	Guo YP et al.	2018	Lithos	165	59-78
4088	JLG-3	CAOB	Beishan	127.0000	44.0000	Monzogranite	Daheishan	188	188	16.49	92.98	0.1074	0.512645		-0.45	0.14	2.10	2.27	2.27	0.78	0.72	0.78		0.79	Guo YP et al.	2018	Lithos	165	59-78
4089	JLG-2-1	CAOB	Beishan	127.0000	44.0000	Monzogranite	Daheishan	188	188	3.74	20.65	0.1097	0.512656		-0.44	0.35	2.30	2.43	2.43	0.77	0.72	0.77		0.77	Guo YP et al.	2018	Lithos	165	59-78
4090	JLG-2	CAOB	Beishan	127.0000	44.0000	Monzogranite	Daheishan	188	188	4.44	24.42	0.1100	0.512656		-0.44	0.35	2.30	2.43	2.43	0.77	0.73	0.77		0.78	Guo YP et al.	2018	Lithos	165	59-78
4091	JLG-1-1	CAOB	Beishan	127.0000	44.0000	Granite	Daheishan	188	188	3.84	22.09	0.1052	0.512628		-0.47	-0.20	1.90	1.99	1.99	0.81	0.73	0.81		0.81	Guo YP et al.	2018	Lithos	165	59-78
4092	JLG-1	CAOB	Beishan	127.0000	44.0000	Granite	Daheishan	188	188	3.06	18.11	0.1023	0.512614		-0.48	-0.47	1.70	1.79	1.79	0.82	0.73	0.82		0.83	Guo YP et al.	2018	Lithos	165	59-78
4093	SU382	CAOB	Southern Urals	58.1925	51.9335	Diorite		400	400	1.69	4.91	0.2081	0.513000		0.06	7.06		6.48	6.48	0.61	4.03	0.61			Spadea P et al.	2000	Special Papers-Geological Society of America		461-472
4094	LK29-2	CAOB	Southern Urals	58.1474	51.9961	Boninites		400	400	0.74	3.77	0.1187	0.512950		-0.40	6.09		10.07	10.07	0.40	0.32	0.32			Spadea P et al.	2000	Special Papers-Geological Society of America		461-472
4095	AKT48	CAOB	Southern Urals	58.1925	51.9335	Andesite		400	400	1.07	3.47	0.1864	0.512950		-0.05	6.09		6.61	6.61	0.60	1.12	0.60			Spadea P et al.	2000	Special Papers-Geological Society of America		461-472
4096	xushenglin01	CAOB	Western Junggar	84.9352	46.5661	Granite		300	300	8.84	39.40	0.1356	0.512765	14	-0.31	2.48	4.90	4.81	4.81	0.67	0.75	0.67		0.67	Xu SL et al.	2022	Earth Science Frontiers (ICWEB)	29((2)	1-20
4097	xushenglin02	CAOB	Western Junggar	84.4065	46.0421	Granodiorite porphyrite		317	317	2.62	12.30	0.1288	0.512748	5	-0.35	2.15	4.91	4.89	4.89	0.68	0.72	0.68		0.68	Xu SL et al.	2022	Earth Science Frontiers (ICWEB)	29((2)	1-21
4098	xushenglin03	CAOB	Western Junggar	84.4122	46.0291	Granodiorite		317	317	2.84	13.60	0.1262	0.512748	5	-0.36	2.15	5.00	5.00	5.00	0.67	0.70	0.67		0.67	Xu SL et al.	2022	Earth Science Frontiers (ICWEB)	29((2)	1-22
4099	xushenglin04	CAOB	Western Junggar	84.4065	46.0421	Diorite		316	316	2.72	13.10	0.1255	0.512760	5	-0.36	2.38	5.26	5.25	5.25	0.65	0.67	0.65		0.65	Xu SL et al.	2022	Earth Science Frontiers (ICWEB)	29((2)	1-23
4100	xushenglin05	CAOB	Western Junggar	85.3290	46.7035	Intermediate acid dike		300	300	5.83	26.80	0.1315	0.512783	5	-0.33	2.83	5.33	5.32	5.32	0.63	0.68	0.63		0.63	Xu SL et al.	2022	Earth Science Frontiers (ICWEB)	29((2)	1-24
4101	xushenglin06	CAOB	Western Junggar	84.6555	46.4015	Biotite granite		300	300	5.97	30.40	0.1187	0.512771	10	-0.40	2.59	5.59	5.58	5.58	0.61	0.61	0.61		0.61	Xu SL et al.	2022	Earth Science Frontiers (ICWEB)	29((2)	1-25
4102	xushenglin07	CAOB	Western Junggar	83.8553	45.4271	Granodiorite		305	305	3.84	18.90	0.1228	0.512781	8	-0.38	2.79	5.68	5.66	5.66	0.60	0.62	0.60		0.60	Xu SL et al.	2022	Earth Science Frontiers (ICWEB)	29((2)	1-26
4103	xushenglin08	CAOB	Western Junggar	82.4624	45.7315	Granodiorite porphyry		300	300	2.30	10.50	0.1324	0.512803	8	-0.33	3.22	5.69	5.68	5.68	0.60	0.65	0.60		0.60	Xu SL et al.	2022	Earth Science Frontiers (ICWEB)	29((2)	1-27
4104	xushenglin09	CAOB	Western Junggar	85.9827	46.7355	Alkali syenite		300	300	6.49	33.20	0.1182	0.512780	14	-0.40	2.77	5.78	5.78	5.78	0.59	0.59	0.59		0.59	Xu SL et al.	2022	Earth Science Frontiers (ICWEB)	29((2)	1-28
4105	xushenglin10	CAOB	Western Junggar	84.4550	45.5009	Granodiorite		300	300	4.44	18.10	0.1483	0.512845	7	-0.25	4.04	5.90	5.89	5.89	0.58	0.71	0.58		0.58	Xu SL et al.	2022	Earth Science Frontiers (ICWEB)	29((2)	1-29
4106	xushenglin11	CAOB	Western Junggar	82.4825	45.7418	Diorite porphyrite		300	300	2.32	12.00	0.1169	0.512784	8	-0.41	2.85	5.91	5.90	5.90	0.58	0.58	0.58		0.58	Xu SL et al.	2022	Earth Science Frontiers (ICWEB)	29((2)	1-30
4107	xushenglin12	CAOB	Western Junggar	83.8301	45.7419	Granodiorite		300	300	6.17	26.50	0.1408	0.512830	6	-0.28	3.75	5.96	5.89	5.89	0.58	0.67	0.58		0.58	Xu SL et al.	2022	Earth Science Frontiers (ICWEB)	29((2)	1-31
4108	xushenglin13	CAOB	Western Junggar	84.1159	45.9608	Fine-grained granite		300	300	8.99	22.60	0.2405	0.513036	12	0.22	7.76	6.09	6.09	6.09	0.57	-0.65	0.57		0.57	Xu SL et al.	2022	Earth Science Frontiers (ICWEB)	29((2)	1-32
4109	xushenglin14	CAOB	Western Junggar	82.4821	45.7420	Granodiorite		300	300	2.91	14.90	0.1181	0.512796	9	-0.40	3.08	6.10	6.09	6.09	0.56	0.56	0.56		0.57	Xu SL et al.	2022	Earth Science Frontiers (ICWEB)	29((2)	1-33
4110	xushenglin15	CAOB	Western Junggar	82.4746	45.7381	Granodiorite		300	300	2.45	13.90	0.1066	0.512783	6	-0.46	2.83	6.29	6.28	6.28	0.55	0.52	0.55		0.55	Xu SL et al.	2022	Earth Science Frontiers (ICWEB)	29((2)	1-34
4111	xushenglin16	CAOB	Western Junggar	82.4747	45.7366	Granodiorite		300	300	2.60	15.20	0.1034	0.512780	6	-0.47	2.77	6.35	6.34	6.34	0.54	0.51	0.54		0.55	Xu SL et al.	2022	Earth Science Frontiers (ICWEB)	29((2)	1-35
4112	xushenglin17	CAOB	Western Junggar	84.4192	46.0968	Granite		300	300	6.15	29.10	0.1278	0.512832	12	-0.35	3.78	6.47	6.42	6.42	0.54	0.56	0.54		0.54	Xu SL et al.	2022	Earth Science Frontiers (ICWEB)	29((2)	1-36

4113	xushenglin18	CAOB	Western Junggar	84.1263	45.9485	Alkali feldspar granite		300	300	7.40	30.00	0.1491	0.512877	10	-0.24	4.66	6.49	6.48	6.48	0.53	0.65	0.53		0.53	Xu SL et al.	2022	Earth Science Frontiers (ICWEB)	29((2)	1-37
4114	xushenglin19	CAOB	Western Junggar	84.1676	45.9208	Alkali feldspar granite		294	294	9.23	36.10	0.1546	0.512892	6	-0.21	4.95	6.54	6.54	6.54	0.52	0.67	0.52		0.52	Xu SL et al.	2022	Earth Science Frontiers (ICWEB)	29((2)	1-38
4115	xushenglin20	CAOB	Western Junggar	84.4486	46.1459	Alkali feldspar granite		300	300	4.55	12.60	0.2183	0.513025	7	0.11	7.55	6.71	6.72	6.72	0.52	-4.20	0.52		0.53	Xu SL et al.	2022	Earth Science Frontiers (ICWEB)	29((2)	1-39
4116	xushenglin21	CAOB	Western Junggar	84.4686	45.4976	Granodiorite		300	300	2.86	15.50	0.1116	0.512818	10	-0.43	3.51	6.78	6.77	6.77	0.51	0.50	0.51		0.51	Xu SL et al.	2022	Earth Science Frontiers (ICWEB)	29((2)	1-40
4117	xushenglin22	CAOB	Western Junggar	83.5510	45.7666	Alkali feldspar granite		300	300	9.06	40.10	0.1366	0.512866	5	-0.31	4.45	6.81	6.75	6.75	0.51	0.56	0.51		0.51	Xu SL et al.	2022	Earth Science Frontiers (ICWEB)	29((2)	1-41
4118	xushenglin23	CAOB	Western Junggar	83.8412	45.6156	Alkali feldspar granite		302	302	6.52	29.00	0.1359	0.512871	11	-0.31	4.55	6.90	6.89	6.89	0.50	0.55	0.50		0.50	Xu SL et al.	2022	Earth Science Frontiers (ICWEB)	29((2)	1-42
4119	xushenglin24	CAOB	Western Junggar	84.4192	46.0968	Alkali feldspar granite		304	304	3.54	17.60	0.1216	0.512843	8	-0.38	4.00	6.92	6.91	6.91	0.50	0.51	0.50		0.50	Xu SL et al.	2022	Earth Science Frontiers (ICWEB)	29((2)	1-43
4120	xushenglin25	CAOB	Western Junggar	83.8548	45.5226	Alkali feldspar granite		300	300	6.46	30.70	0.1272	0.512854	6	-0.35	4.21	6.96	6.87	6.87	0.50	0.52	0.50		0.50	Xu SL et al.	2022	Earth Science Frontiers (ICWEB)	29((2)	1-44
4121	xushenglin26	CAOB	Western Junggar	84.6528	46.4008	Alkali feldspar granite		298	298	7.51	35.10	0.1294	0.512864	6	-0.34	4.41	6.98	6.97	6.97	0.49	0.52	0.49		0.49	Xu SL et al.	2022	Earth Science Frontiers (ICWEB)	29((2)	1-45
4122	xushenglin27	CAOB	Western Junggar	84.2810	45.8306	Granite		309	309	7.30	34.00	0.1298	0.512862	10	-0.34	4.37	7.01	7.01	7.01	0.50	0.52	0.50		0.50	Xu SL et al.	2022	Earth Science Frontiers (ICWEB)	29((2)	1-46
4123	xushenglin28	CAOB	Western Junggar	84.5397	45.4732	Diorite		300	300	3.19	13.90	0.1387	0.512878	12	-0.29	4.68	7.02	6.90	6.90	0.50	0.55	0.50		0.50	Xu SL et al.	2022	Earth Science Frontiers (ICWEB)	29((2)	1-47
4124	xushenglin29	CAOB	Western Junggar	84.1000	45.9640	Diorite		317	317	19.90	57.60	0.2089	0.513024	5	0.06	7.53	7.04	7.04	7.04	0.50	3.94	0.50		0.50	Xu SL et al.	2022	Earth Science Frontiers (ICWEB)	29((2)	1-48
4125	xushenglin30	CAOB	Western Junggar	84.6555	46.4015	Biotite granite		303	303	8.78	40.40	0.1314	0.512869	8	-0.33	4.51	7.05	7.03	7.03	0.49	0.52	0.49		0.49	Xu SL et al.	2022	Earth Science Frontiers (ICWEB)	29((2)	1-49
4126	xushenglin31	CAOB	Western Junggar	83.2396	45.9916	Hornblende granite		316	316	8.81	39.60	0.1345	0.512872	6	-0.32	4.56	7.08	7.07	7.07	0.50	0.54	0.50		0.50	Xu SL et al.	2022	Earth Science Frontiers (ICWEB)	29((2)	1-50
4127	xushenglin32	CAOB	Western Junggar	84.1000	45.9640	Alkali feldspar granite		299	299	5.73	24.70	0.1402	0.512892	12	-0.29	4.95	7.12	7.11	7.11	0.48	0.54	0.48		0.48	Xu SL et al.	2022	Earth Science Frontiers (ICWEB)	29((2)	1-51
4128	xushenglin33	CAOB	Western Junggar	84.6465	45.9003	Alkali feldspar granite		305	305	3.44	10.30	0.2019	0.513016	5	0.03	7.37	7.18	7.17	7.17	0.48	1.73	0.48		0.48	Xu SL et al.	2022	Earth Science Frontiers (ICWEB)	29((2)	1-52
4129	xushenglin34	CAOB	Western Junggar	84.1565	46.3148	Granodiorite		294	294	8.17	38.30	0.1290	0.512875	10	-0.34	4.62	7.18	7.16	7.16	0.47	0.50	0.47		0.47	Xu SL et al.	2022	Earth Science Frontiers (ICWEB)	29((2)	1-53
4130	xushenglin35	CAOB	Western Junggar	83.4770	45.7003	Alkali feldspar granite		304	304	8.57	39.40	0.1315	0.512876	5	-0.33	4.64	7.18	7.17	7.17	0.48	0.51	0.48		0.48	Xu SL et al.	2022	Earth Science Frontiers (ICWEB)	29((2)	1-54
4131	xushenglin36	CAOB	Western Junggar	83.6614	45.7730	Granite		300	300	6.33	31.50	0.1215	0.512857	10	-0.38	4.27	7.18	7.15	7.15	0.48	0.49	0.48		0.48	Xu SL et al.	2022	Earth Science Frontiers (ICWEB)	29((2)	1-55
4132	xushenglin37	CAOB	Western Junggar	85.0614	45.9946	Alkali feldspar granite		316	316	6.85	32.80	0.1263	0.512862	8	-0.36	4.37	7.22	7.21	7.21	0.49	0.50	0.49		0.49	Xu SL et al.	2022	Earth Science Frontiers (ICWEB)	29((2)	1-56
4133	xushenglin38	CAOB	Western Junggar	84.7470	45.6598	Granodiorite		300	300	3.78	17.80	0.1284	0.512873	8	-0.35	4.58	7.29	7.20	7.20	0.47	0.50	0.47		0.48	Xu SL et al.	2022	Earth Science Frontiers (ICWEB)	29((2)	1-57
4134	xushenglin39	CAOB	Western Junggar	84.6629	45.6713	Granodiorite		300	300	3.75	15.90	0.1426	0.512905	8	-0.28	5.21	7.36	7.28	7.28	0.47	0.53	0.47		0.47	Xu SL et al.	2022	Earth Science Frontiers (ICWEB)	29((2)	1-58
4135	xushenglin40	CAOB	Western Junggar	83.5654	45.7024	Alkali feldspar granite		307	307	8.30	45.50	0.1103	0.512842	5	-0.44	3.98	7.38	7.36	7.36	0.47	0.45	0.47		0.47	Xu SL et al.	2022	Earth Science Frontiers (ICWEB)	29((2)	1-59
4136	xushenglin41	CAOB	Western Junggar	84.5397	45.4732	Diorite		300	300	4.34	23.00	0.1141	0.512847	10	-0.42	4.08	7.41	7.24	7.24	0.47	0.46	0.47		0.47	Xu SL et al.	2022	Earth Science Frontiers (ICWEB)	29((2)	1-60
4137	xushenglin42	CAOB	Western Junggar	83.7024	46.2633	Granodiorite		307	307	7.70	35.60	0.1308	0.512888	5	-0.34	4.88	7.47	7.46	7.46	0.46	0.48	0.46		0.46	Xu SL et al.	2022	Earth Science Frontiers (ICWEB)	29((2)	1-61
4138	xushenglin43	CAOB	Western Junggar	84.1643	46.3038	Alkali feldspar granite		300	300	8.06	32.30	0.1509	0.512932	8	-0.23	5.74	7.50	7.49	7.49	0.45	0.53	0.45		0.45	Xu SL et al.	2022	Earth Science Frontiers (ICWEB)	29((2)	1-62
4139	xushenglin44	CAOB	Western Junggar	84.1565	46.3148	Alkali feldspar granite		300	300	7.50	35.20	0.1288	0.512895	12	-0.35	5.01	7.57	7.61	7.61	0.44	0.46	0.44		0.44	Xu SL et al.	2022	Earth Science Frontiers (ICWEB)	29((2)	1-63
4140	xushenglin45	CAOB	Western Junggar	84.5627	45.8882	Biotite granite		298	298	8.84	43.70	0.1223	0.512886	6	-0.38	4.84	7.68	7.67	7.67	0.43	0.44	0.43		0.44	Xu SL et al.	2022	Earth Science Frontiers (ICWEB)	29((2)	1-64
4141	xushenglin46	CAOB	Western Junggar	84.3633	45.7632	Alkali feldspar granite		300	300	9.97	50.90	0.1184	0.512879	9	-0.40	4.70	7.71	7.70	7.70	0.43	0.43	0.43		0.43	Xu SL et al.	2022	Earth Science Frontiers (ICWEB)	29((2)	1-65
4142	xushenglin47	CAOB	Western Junggar	84.8491	45.7223	Biotite granite		303	303	4.54	25.90	0.1060	0.512853	6	-0.46	4.19	7.72	7.70	7.70	0.44	0.42	0.44		0.44	Xu SL et al.	2022	Earth Science Frontiers (ICWEB)	29((2)	1-66
4143	xushenglin48	CAOB	Western Junggar	84.7143	45.6760	Granite		313	313	3.15	15.20	0.1253	0.512888	8	-0.36	4.88	7.74	7.73	7.73	0.44	0.45	0.44		0.44	Xu SL et al.	2022	Earth Science Frontiers (ICWEB)	29((2)	1-67
4144	xushenglin49	CAOB	Western Junggar	84.5570	45.7015	Granite		300	300	2.42	13.80	0.1060	0.512853	14	-0.46	4.19	7.79	7.67	7.67	0.44	0.42	0.44		0.44	Xu SL et al.	2022	Earth Science Frontiers (ICWEB)	29((2)	1-68
4145	xushenglin50	CAOB	Western Junggar	84.5387	45.6884	Biotite granite		312	312	3.44	20.60	0.1010	0.512885	12	-0.49	4.82	8.64	8.63	8.63	0.37	0.36	0.37		0.37	Xu SL et al.	2022	Earth Science Frontiers (ICWEB)	29((2)	1-69
4146	xushenglin51	CAOB	Western Junggar	84.9352	46.5661	Granite		300	300	8.84	39.40	0.1356	0.512765	14	-0.31	2.48	4.90	4.81	4.81	0.67	0.75	0.67		0.67	Xu SL et al.	2022	Earth Science Frontiers (ICWEB)	29((2)	1-70
4147	xushenglin52	CAOB	Western Junggar	84.4065	46.0421	Granodiorite porphyrite		317	317	2.62	12.30	0.1288	0.512748	5	-0.35	2.15	4.91	4.89	4.89	0.68	0.72	0.68		0.68	Xu SL et al.	2022	Earth Science Frontiers (ICWEB)	29((2)	1-71
4148	xushenglin53	CAOB	Western Junggar	84.4122	46.0291	Granodiorite		317	317	2.84	13.60	0.1262	0.512748	5	-0.36	2.15	5.00	5.00	5.00	0.67	0.70	0.67		0.67	Xu SL et al.	2022	Earth Science Frontiers (ICWEB)	29((2)	1-72
4149	xushenglin54	CAOB	Western Junggar	84.4065	46.0421	Diorite		316	316	2.72	13.10	0.1255	0.512760	5	-0.36	2.38	5.26	5.25	5.25	0.65	0.67	0.65		0.65	Xu SL et al.	2022	Earth Science Frontiers (ICWEB)	29((2)	1-73
4150	xushenglin55	CAOB	Western Junggar	85.3290	46.7035	Intermediate acid dike		300	300	5.83	26.80	0.1315	0.512783	5	-0.33	2.83	5.33	5.32	5.32	0.63	0.68	0.63		0.63	Xu SL et al.	2022	Earth Science Frontiers (ICWEB)	29((2)	1-74
4151	xushenglin56	CAOB	Western Junggar	84.6555	46.4015	Biotite granite		300	300	5.97	30.40	0.1187	0.512771	10	-0.40	2.59	5.59	5.58	5.58	0.61	0.61	0.61		0.61	Xu SL et al.	2022	Earth Science Frontiers (ICWEB)	29((2)	1-75
4152	xushenglin57	CAOB	Western Junggar	83.8553	45.4271	Granodiorite		305	305	3.84	18.90	0.1228	0.512781	8	-0.38	2.79	5.68	5.66	5.66	0.60	0.62	0.60		0.60	Xu SL et al.	2022	Earth Science Frontiers (ICWEB)	29((2)	1-76
4153	xushenglin58	CAOB	Western Junggar	82.4624	45.7315	Granodiorite porphyry		300	300	2.30	10.50	0.1324	0.512803	8	-0.33	3.22	5.69	5.68	5.68	0.60	0.65	0.60		0.60	Xu SL et al.	2022	Earth Science Frontiers (ICWEB)	29((2)	1-77
4154	xushenglin59	CAOB	Western Junggar	85.9827	46.7355	Alkali syenite		300	300	6.49	33.20	0.1182	0.512780	14	-0.40	2.77	5.78	5.78	5.78	0.59	0.59	0.59		0.59	Xu SL et al.	2022	Earth Science Frontiers (ICWEB)	29((2)	1-78
4155	xushenglin60	CAOB	Western Junggar	84.4550	45.5009	Granodiorite		300	300	4.44	18.10	0.1483	0.512845	7	-0.25	4.04	5.90	5.89	5.89	0.58	0.71	0.58		0.58	Xu SL et al.	2022	Earth Science Frontiers (ICWEB)	29((2)	1-79
4156	xushenglin61	CAOB	Western Junggar	82.4825	45.7418	Diorite porphyrite		300	300	2.32	12.00	0.1169	0.512784	8	-0.41	2.85	5.91	5.90	5.90	0.58	0.58	0.58		0.58	Xu SL et al.	2022	Earth Science Frontiers (ICWEB)	29((2)	1-80
4157	xushenglin62	CAOB	Western Junggar	83.8301	45.7419	Granodiorite		300	300	6.17	26.50	0.1408	0.512830	6	-0.28	3.75	5.96	5.89	5.89	0.58	0.67	0.58		0.58	Xu SL et al.	2022	Earth Science Frontiers (ICWEB)	29((2)	1-81
4158	xushenglin63	CAOB	Western Junggar	84.1159	45.9608	Fine-grained granite		300	300	8.99	22.60	0.2405	0.513036	12	0.22	7.76	6.09	6.09	6.09	0.57	-0.65	0.57		0.57	Xu SL et al.	2022	Earth Science Frontiers (ICWEB)	29((2)	1-82
4159	xushenglin64	CAOB	Western Junggar	82.4821	45.7420	Granodiorite		300	300	2.91	14.90	0.1181	0.512796	9	-0.40	3.08	6.10	6.09	6.09	0.56	0.56	0.56		0.57	Xu SL et al.	2022	Earth Science Frontiers (ICWEB)	29((2)	1-83
4160	xushenglin65	CAOB	Western Junggar	82.4746	45.7381	Granodiorite		300	300	2.45	13.90	0.1066	0.512783	6	-0.46	2.83	6.29	6.28	6.28	0.55	0.52	0.55		0.55	Xu SL et al.	2022	Earth Science Frontiers (ICWEB)	29((2)	1-84
4161	xushenglin66	CAOB	Western Junggar	82.4747	45.7366	Granodiorite		300	300	2.60	15.20	0.1034	0.512780	6	-0.47	2.77	6.35	6.34	6.34	0.54	0.51	0.54		0.55	Xu SL et al.	2022	Earth Science Frontiers (ICWEB)	29((2)	1-85
4162	xushenglin67	CAOB	Western Junggar	84.4192	46.0968	Granite		300	300	6.15	29.10	0.1278	0.512832	12	-0.35	3.78	6.44	6.42	6.42	0.54	0.56	0.54		0.54	Xu SL et al.	2022	Earth Science Frontiers (ICWEB)	29((2)	1-86
4163	xushenglin68	CAOB	Western Junggar	84.1263	45.9485	Alkali feldspar granite		300	300	7.40	30.00	0.1491	0.512877	10	-0.24	4.66	6.49	6.48	6.48	0.53	0.65	0.53		0.53	Xu SL et al.	2022	Earth Science Frontiers (ICWEB)	29((2)	1-87
4164	xushenglin69	CAOB	Western Junggar	84.1676	45.9208	Alkali feldspar granite		294	294	9.23	36.10	0.1546	0.512892	6	-0.21	4.95	6.54	6.54	6.54	0.52	0.67	0.52		0.52	Xu SL et al.	2022	Earth Science Frontiers (ICWEB)	29((2)	1-88
4165	xushenglin70	CAOB	Western Junggar	84.4486	46.1459	Alkali feldspar granite		300	300	4.55	12.60	0.2183	0.513025	7	0.11	7.55	6.71	6.72	6.72	0.52	-4.20	0.52		0.53	Xu SL et al.	2022	Earth Science Frontiers (ICWEB)	29((2)	1-89
4166	xushenglin71	CAOB	Western Junggar	84.4686	45.4976	Granodiorite		300	300	2.86	15.50	0.1116	0.512818	10	-0.43	3.51	6.78	6.77	6.77	0.51	0.50	0.51		0.51	Xu SL et al.	2022	Earth Science Frontiers (ICWEB)	29((2)	1-90
4167	xushenglin72	CAOB	Western Junggar	83.5510	45.7666	Alkali feldspar granite		300	300	9.06	40.10	0.1366	0.512866	5	-0.31	4.45	6.81	6.75	6.75	0.51	0.56	0.51		0.51	Xu SL et al.	2022	Earth Science Frontiers (ICWEB)	29((2)	1-91
4168	xushenglin73	CAOB	Western Junggar	83.8412	45.6156	Alkali feldspar granite		302	302	6.52	29.00	0.1359	0.512871	11	-0.31	4.55	6.90	6.89	6.89	0.50	0.55	0.50		0.50	Xu SL et al.	2022	Earth Science Frontiers (ICWEB)	29((2)	1-92
4169	xushenglin74	CAOB	Western Junggar	84.4192	46.0968	Alkali feldspar granite		304	304	3.54	17.60	0.1216	0.512843	8	-0.38	4.00	6.92	6.91	6.91	0.50	0.51	0.50		0.50	Xu SL et al.	2022	Earth Science Frontiers (ICWEB)	29((2)	1-93
4170	xushenglin75	CAOB	Western Junggar	83.8548	45.5226	Alkali feldspar granite		300	300	6.46	30.70	0.1272	0.512854	6	-0.35	4.21	6.96	6.87	6.87	0.50	0.52	0.50		0.50	Xu SL et al.	2022	Earth Science Frontiers (ICWEB)	29((2)	1-94
4171	xushenglin76	CAOB	Western Junggar	84.6528	46.4008	Alkali feldspar granite		298	298	7.51	35.10	0.1294	0.512864	6	-0.34	4.41	6.98	6.97	6.97	0.49	0.52	0.49		0.49	Xu SL et al.	2022	Earth Science Frontiers (ICWEB)	29((2)	1-95
4172	xushenglin77	CAOB	Western Junggar	84.2810	45.8306	Granite		309	309	7.30	34.00	0.1298	0.512862	10	-0.34	4.37	7.01	7.01	7.01	0.50	0.52	0.50		0.50	Xu SL et al.	2022	Earth Science Frontiers (ICWEB)	29((2)	1-96
4173	xushenglin78	CAOB	Western Junggar	84.5397	45.4732	Diorite		300	300	3.19	13.90	0.1387	0.512878	12	-0.29	4.68	7.02	6.90	6.90	0.50	0.55	0.50		0.50	Xu SL et al.	2022	Earth Science Frontiers (ICWEB)	29((2)	1-97
4174	xushenglin79	CAOB	Western Junggar	84.1000	45.9640	Diorite		317	317	19.90	57.60	0.2089	0.513024	5	0.06	7.53	7.04	7.04	7.04	0.50	3.94	0.50		0.50	Xu SL et al.	2022	Earth Science Frontiers (ICWEB)	29((2)	1-98
4175	xushenglin80	CAOB	Western Junggar	84.6555	46.4015	Biotite granite		303	303	8.78	40.40	0.1314	0.512869	8	-0.33	4.51	7.05	7.03	7.03	0.49	0.52	0.49		0.49	Xu SL et al.	2022	Earth Science Frontiers (ICWEB)	29((2)	1-99
4176	xushenglin81	CAOB	Western Junggar	83.2396	45.9916	Hornblende granite		316	316	8.81	39.60	0.1345	0.512872	6	-0.32	4.56	7.08	7.07	7.07	0.50	0.54	0.50		0.50	Xu SL et al.	2022	Earth Science Frontiers (ICWEB)	29((2)	1-100
4177	xushenglin82	CAOB	Western Junggar	84.1000	45.9640	Alkali feldspar granite		299	299	5.73	24.70	0.1402	0.512892	12	-0.29	4.95	7.12	7.11	7.11	0.48	0.54	0.48		0.48	Xu SL et al.	2022	Earth Science Frontiers (ICWEB)	29((2)	1-101
4178	xushenglin83	CAOB	Western Junggar	84.6465	45.9003	Alkali feldspar granite		305	305	3.44	10.30	0.2019	0.513016	5	0.03	7.37	7.18	7.17	7.17	0.48	1.73	0.48		0.48	Xu SL et al.	2022	Earth Science Frontiers (ICWEB)	29((2)	1-102
4179	xushenglin84	CAOB	Western Junggar	84.1565	46.3148	Granodiorite		294	294	8.17	38.30	0.1290	0.512875	10	-0.34	4.62	7.18	7.16	7.16	0.47	0.50	0.47		0.47	Xu SL et al.	2022	Earth Science Frontiers (ICWEB)	29((2)	1-103
4180	xushenglin85	CAOB	Western Junggar	83.4770	45.7003	Alkali feldspar granite		304	304	8.57	39.40	0.1315	0.512876	5	-0.33	4.64	7.18	7.17	7.17	0.48	0.51	0.48		0.48	Xu SL et al.	2022	Earth Science Frontiers (ICWEB)	29((2)	1-104
4181	xushenglin86	CAOB	Western Junggar	83.6614	45.7730	Granite		300	300	6.33	31.50	0.1215	0.512857	10	-0.38	4.27	7.18	7.15	7.15	0.48	0.49	0.48		0.48	Xu SL et al.	2022	Earth Science Frontiers (ICWEB)	29((2)	1-105
4182	xushenglin87	CAOB	Western Junggar	85.0614	45.9946	Alkali feldspar granite		316	316	6.85	32.80	0.1263	0.512862	8	-0.36	4.37	7.22	7.21	7.21	0.49	0.50	0.49		0.49	Xu SL et al.	2022	Earth Science Frontiers (ICWEB)	29((2)	1-106
4183	xushenglin88	CAOB	Western Junggar	84.7470	45.6598	Granodiorite		300	300	3.78	17.80	0.1284	0.512873	8	-0.35	4.58	7.29	7.20	7.20	0.47	0.50	0.47		0.48	Xu SL et al.	2022	Earth Science Frontiers (ICWEB)	29((2)	1-107
4184	xushenglin89	CAOB	Western Junggar	84.6629	45.6713	Granodiorite		300	300	3.75	15.90	0.1426	0.512905	8	-0.28	5.21	7.36	7.28	7.28	0.47	0.53	0.47		0.47	Xu SL et al.	2022	Earth Science Frontiers (ICWEB)	29((2)	1-108
4185	xushenglin90	CAOB	Western Junggar	83.5654	45.7024	Alkali feldspar granite		307	307	8.30	45.50	0.1103	0.512842	5	-0.44	3.98	7.38	7.36	7.36	0.47	0.45	0.47		0.47	Xu SL et al.	2022	Earth Science Frontiers (ICWEB)	29((2)	1-109
4186	xushenglin91	CAOB	Western Junggar	84.5397	45.4732	Diorite		300	300	4.34	23.00	0.1141	0.512847	10	-0.42	4.08	7.41	7.24	7.24	0.47	0.46	0.47		0.47	Xu SL et al.	2022	Earth Science Frontiers (ICWEB)	29((2)	1-110
4187	xushenglin92	CAOB	Western Junggar	83.7024	46.2633	Granodiorite		307	307	7.70	35.60	0.1308	0.512888	5	-0.34	4.88	7.47	7.46	7.46	0.46	0.48	0.46		0.46	Xu SL et al.	2022	Earth Science Frontiers (ICWEB)	29((2)	1-111
4188	xushenglin93	CAOB	Western Junggar	84.1643	46.3038	Alkali feldspar granite		300	300	8.06	32.30	0.1509	0.512932	8	-0.23	5.74	7.50	7.49	7.49	0.45	0.53	0.45		0.45	Xu SL et al.	2022	Earth Science Frontiers (ICWEB)	29((2)	1-112
4189	xushenglin94	CAOB	Western Junggar	84.1565	46.3148	Alkali feldspar granite		300	300	7.50	35.20	0.1288	0.512895	12	-0.35	5.01	7.57	7.61	7.61	0.44	0.46	0.44		0.44	Xu SL et al.	2022	Earth Science Frontiers (ICWEB)	29((2)	1-113
4190	xushenglin95	CAOB	Western Junggar	84.5627	45.8882	Biotite granite		298	298	8.84	43.70	0.1223	0.512886	6	-0.38	4.84	7.68	7.67	7.67	0.43	0.44	0.43		0.44	Xu SL et al.	2022	Earth Science Frontiers (ICWEB)	29((2)	1-114
4191	xushenglin96	CAOB	Western Junggar	84.3633	45.7632	Alkali feldspar granite		300	300	9.97	50.90	0.1184	0.512879	9	-0.40	4.70	7.71	7.70	7.70	0.43	0.43	0.43		0.43	Xu SL et al.	2022	Earth Science Frontiers (ICWEB)	29((2)	1-115
4192	xushenglin97	CAOB	Western Junggar	84.8491	45.7223	Biotite granite		303	303	4.54	25.90	0.1060	0.512853	6	-0.46	4.19	7.72	7.70	7.70	0.44	0.42	0.44		0.44	Xu SL et al.	2022	Earth Science Frontiers (ICWEB)	29((2)	1-116
4193	xushenglin98	CAOB	Western Junggar	84.7143	45.6760	Granite		313	313	3.15	15.20	0.1253	0.512888	8	-0.36	4.88	7.74	7.73	7.73	0.44	0.45	0.44		0.44	Xu SL et al.	2022	Earth Science Frontiers (ICWEB)	29((2)	1-117
4194	xushenglin99	CAOB	Western Junggar	84.5570	45.7015	Granite		300	300	2.42	13.80	0.1060	0.512853	14	-0.46	4.19	7.79	7.67	7.67	0.44	0.42	0.44		0.44	Xu SL et al.	2022	Earth Science Frontiers (ICWEB)	29((2)	1-118
4195	xushenglin100	CAOB	Western Junggar	84.5387	45.6884	Biotite granite		312	312	3.44	20.60	0.1010	0.512885	12	-0.49	4.82	8.64	8.63	8.63	0.37	0.36	0.37		0.37	Xu SL et al.	2022	Earth Science Frontiers (ICWEB)	29((2)	1-119
4196	LS93	CAOB	Alashan	104.9199	40.9524	Granite		279	279	4.16	32.04	0.0829	0.512831	9	-0.58	3.76	7.95	7.81	7.81	0.41	0.37	0.41	0.37	0.41	This study				
4197	LS27	CAOB	Alashan	105.3316	40.1249	Granodiorite		275	275	4.38	15.14	0.1748	0.512160	7	-0.11	-9.32	-8.42	-8.55	-8.55	1.72	3.84	1.72	3.84	1.70	This study				
4198	LS094	CAOB	Alashan	104.9199	40.9524	Granite		279	279	4.86	38.42	0.0765	0.511865	7	-0.61	-15.08	-10.7	-10.8	-10.8	1.92	1.43	1.92	1.43	1.92	This study				
4199	LS016	CAOB	Alashan	105.5534	40.1428	Granite	Aolun Prague	321	321	0.46	3.11	0.0891	0.511747	12	-0.55	-17.39	-17.2	-13.0	-13.0	2.13	1.71	2.13	1.71	2.22	This study				
4200	LS015	CAOB	Alashan	105.5534	40.1428	Granite	Aolun Prague	321	321	0.44	2.60	0.1012	0.511778	10	-0.49	-16.78	-16.6	-12.9	-12.9	2.12	1.85	2.12	1.85	2.17	This study				
4201	LS013	CAOB	Alashan	105.5843	40.1002	Granite	Aolun Prague	321	321	0.60	2.87	0.1273	0.511430	11	-0.35	-23.57	-20.6	-20.7	-20.7	2.75	3.02	2.75	3.02	2.74	This study				
4202	LS012	CAOB	Alashan	105.5843	40.1002	Granite	Aolun Prague	321	321	2.79	17.26	0.0978	0.511517	18	-0.50	-21.87	-17.7	-17.8	-17.8	2.52	2.14	2.52	2.14	2.52	This study				
4203	13AX166	CAOB	Alashan	102.5107	42.0697	Diorite		272	272	4.93	23.71	0.1257	0.512366	8	-0.36	-5.31	-5.15	-2.84	-2.84	1.27	1.36	1.27	1.36	1.24	This study				
4204	13AX165	CAOB	Alashan	102.5107	42.0697	Diorite		272	272	4.32	19.71	0.1325	0.512358	8	-0.33	-5.46	-5.31	-3.23	-3.23	1.30	1.48	1.30	1.48	1.25	This study				
4205	13AX159	CAOB	Alashan	102.5037	42.0880	Monzogranite		272	272	2.43	13.04	0.1126	0.512310	9	-0.43	-6.40	-6.24	-3.48	-3.48	1.32	1.27	1.32	1.27	1.33	This study				
4206	13AX158	CAOB	Alashan	102.5037	42.0880	Monzogranite		272	272	2.62	15.42	0.1026	0.512296	9	-0.48	-6.67	-6.52	-3.40	-3.40	1.32	1.17	1.32	1.17	1.36	This study				
4207	13AX154	CAOB	Alashan	102.3292	41.5236	K-feldspar granite		272	272	6.77	27.34	0.1496	0.512607	7	-0.24	-0.60	-0.45	1.03	1.03	0.95	1.29	0.95	1.29	0.86	This study				
4208	13AX153	CAOB	Alashan	102.3292	41.5236	K-feldspar granite		272	272	6.05	26.58	0.1376	0.512627	8	-0.30	-0.21	-0.06	1.84	1.84	0.89	1.05	0.89	1.05	0.83	This study				
4209	13AX146	CAOB	Alashan	104.3355	41.6024	K-feldspar granite		272	272	10.49	48.21	0.1316	0.512547	7	-0.33	-1.78	-1.62	0.49	0.49	1.00	1.12	1.00	1.12	0.95	This study				
4210	13AX145	CAOB	Alashan	103.3355	41.6024	K-feldspar granite		272	272	11.20	52.56	0.1288	0.512543	6	-0.35	-1.85	-1.70	0.51	0.51	1.00	1.09	1.00	1.09	0.96	This study				

4211	13AX135rep.	CAOB	Alashan	103.0175	40.6088	Mylonite		272	272	4.99	31.34	0.0962	0.511665	11	-0.51	-18.98	-18.8	-15.5	-15.5	2.30	1.92	2.30	1.92	2.35	This study				
4212	13AX135	CAOB	Alashan	103.0175	40.6088	Mylonite		272	272	4.78	29.98	0.0964	0.511664	7	-0.51	-19.00	-18.8	-15.5	-15.5	2.30	1.92	2.30	1.92	2.35	This study				
4213	13AX133rep.	CAOB	Alashan	103.0175	40.6088	Mylonite		272	272	2.69	11.01	0.1475	0.511998	8	-0.25	-12.48	-12.3	-10.8	-10.8	1.91	2.64	1.91	2.64	1.81	This study				
4214	13AX133	CAOB	Alashan	103.0175	40.6088	Mylonite		272	272	2.67	10.88	0.1481	0.511995	8	-0.25	-12.54	-12.4	-10.9	-10.9	1.91	2.67	1.91	2.67	1.81	This study				
4215	12LS169	CAOB	Alashan	103.5171	40.7636	K-feldspar granite		272	272	0.40	2.60	0.0919	0.512380	9	-0.53	-5.03	-1.25	-1.39	-1.39	1.15	0.96	1.15	0.96	1.15	This study				
4216	12LS168	CAOB	Alashan	103.5171	40.7636	K-feldspar granite		272	272	5.56	32.26	0.1041	0.512333	8	-0.47	-5.95	-2.59	-2.73	-2.73	1.26	1.14	1.26	1.14	1.26	This study				
4217	12LS164	CAOB	Alashan	103.4903	40.7784	Diorite		249	249	6.16	32.49	0.1146	0.512474	7	-0.42	-3.20	-0.45	-0.59	-0.59	1.07	1.04	1.07	1.04	1.06	This study				
4218	12LS163	CAOB	Alashan	103.4903	40.7784	Diorite		249	249	6.51	34.66	0.1136	0.512487	7	-0.42	-2.95	-0.17	-0.31	-0.31	1.04	1.01	1.04	1.01	1.04	This study				
4219	12LS158	CAOB	Alashan	103.4903	40.7754	Granodiorite		247	247	4.48	24.37	0.1112	0.512466	7	-0.43	-3.36	-0.52	-0.67	-0.67	1.07	1.02	1.07	1.02	1.07	This study				
4220	11LS98	CAOB	Alashan	104.3669	40.9291	Granodiorite		266	266	2.78	12.15	0.1384	0.512217	10	-0.30	-8.21	-6.09	-6.23	-6.23	1.54	1.88	1.54	1.88	1.53	This study				
4221	11LS91	CAOB	Alashan	104.3976	40.9013	Granite		254	254	2.16	10.77	0.1210	0.512500	9	-0.38	-2.69	-0.10	-0.24	-0.24	1.04	1.07	1.04	1.07	1.04	This study				
4222	11LS90	CAOB	Alashan	104.3976	40.9013	Granite		251	251	2.39	12.90	0.1120	0.512481	8	-0.43	-3.06	-2.91	-0.35	-0.35	1.05	1.00	1.05	1.00	1.06	This study				
4223	11LS83	CAOB	Alashan	104.3671	40.9819	Granidiorite		251	251	1.73	9.26	0.1132	0.512500	11	-0.42	-2.69	-2.54	-0.02	-0.02	1.02	0.99	1.02	0.99	1.03	This study				
4224	11LS82	CAOB	Alashan	104.3671	40.9819	Granodiorite		251	251	1.97	11.98	0.0993	0.512199	7	-0.50	-8.56	-8.41	-5.44	-5.44	1.46	1.27	1.46	1.27	1.51	This study				
4225	11LS52	CAOB	Alashan	105.1326	40.3484	Bi-granite		251	251	5.39	29.71	0.1098	0.511901	7	-0.44	-14.38	-14.2	-11.6	-11.6	1.96	1.83	1.96	1.83	1.98	This study				
4226	11LS51	CAOB	Alashan	105.1326	40.3484	Biotie-granite		251	251	4.26	17.02	0.1512	0.511932	7	-0.23	-13.77	-13.6	-12.3	-12.3	2.01	2.95	2.01	2.95	1.91	This study				
4227	11LS176	CAOB	Alashan	104.9558	40.9322	Granite		251	251	2.88	16.29	0.1068	0.512466	9	-0.46	-3.36	-0.33	-0.48	-0.48	1.06	0.98	1.06	0.98	1.06	This study				
4228	11LS122	CAOB	Alashan	104.4624	40.8451	Granodiorite		252	252	3.88	21.14	0.1110	0.512532	7	-0.44	-2.07	0.83	0.69	0.69	0.97	0.92	0.97	0.92	0.96	This study				
4229	M90717-1b	CAOB	Altai	92.0667	45.9833	phyritic C-grained granodiorite		312	312	7.79	44.30	0.1063	0.512689	0	-0.46	0.99	4.59	4.59	4.59	0.70	0.65	0.70	0.65	0.74	This study				
4230	M90717-1a	CAOB	Altai	92.0667	45.9833	M-grained granodiorite		312	312	4.42	24.90	0.1073	0.512693	0	-0.45	1.07	4.63	4.63	4.63	0.69	0.66	0.69	0.66	0.73	This study				
4231	M90716-6	CAOB	Altai	92.5000	46.0833	Garnodiorite		268	268	3.97	19.20	0.1250	0.512138	0	-0.36	-9.75	-7.30	-7.30	-7.30	1.63	1.73	1.63	1.73	1.61	This study				
4232	M90715-8B	CAOB	Altai	92.5203	46.1128	Plagioclase amphibolite		394	394	3.72	13.50	0.1666	0.512954	1	-0.15	6.16		7.68	7.68	0.51	0.63	0.51			This study				
4233	M90715-3d	CAOB	Altai	91.2775	46.5478	C-grained Bi granite		391	391	2.52	13.99	0.1089	0.512758	2	-0.45	2.34	6.72	6.72	6.72	0.59	0.57	0.59	0.57	0.63	This study				
4234	M90715-3B	CAOB	Altai	92.2837	46.5328	M-C-grained diorite		391	391	6.98	29.10	0.1450	0.512647	1	-0.26	0.18		2.76	2.76	0.91	1.12	0.91			This study				
4235	M90715-3a	CAOB	Altai	91.2775	46.5478	M-C-grained granodiorite		391	391	6.98	29.10	0.1450	0.512647	0	-0.26	0.18	2.76	2.76	2.76	0.91	1.12	0.91	1.12	0.80	This study				
4236	M90715-2b	CAOB	Altai	91.2574	46.6123	Bi monzogranite		411	411	0.60	4.30	0.0844	0.512517	0	-0.57	-2.36	3.53	3.53	3.53	0.86	0.75	0.86	0.75	1.01	This study				
4237	M90714-3b	CAOB	Altai	91.4878	46.2525	F-grained granite		422	422	1.33	4.90	0.1641	0.512535	0	-0.17	-2.01	-0.25	-0.25	-0.25	1.18	1.88	1.18	1.88	0.98	This study				
4238	M90714-3a	CAOB	Altai	91.4878	46.2525	Bi. mozogranite		422	422	2.49	14.38	0.1046	0.512093	3	-0.47	-10.63	-5.67	-5.67	-5.67	1.62	1.47	1.62	1.47	1.68	This study				
4239	M90713-6a	CAOB	Altai	91.0406	47.2471	Porphyritic Bi monzogranite		414	414	6.67	29.20	0.1381	0.512391	0	-0.30	-4.82	-1.72	-1.72	-1.72	1.29	1.53	1.29	1.53	1.21	This study				
4240	M90713-5a	CAOB	Altai	91.0632	47.2133	Porphyritic Bi monzogranite		421	421	8.31	35.30	0.1423	0.512403	0	-0.28	-4.58	-1.66	-1.66	-1.66	1.29	1.59	1.29	1.59	1.19	This study				
4241	M90713-3a	CAOB	Altai	91.0914	47.1370	M-grained Bi monzogranite		402	402	8.75	37.70	0.1403	0.512485	0	-0.29	-2.98	-0.09	-0.09	-0.09	1.15	1.38	1.15	1.38	1.06	This study				
4242	M90711-1a	CAOB	Altai	91.4511	47.5287	Bi monzogranite		388	388	2.77	12.50	0.1340	0.512482	0	-0.32	-3.04	0.06	0.06	0.06	1.13	1.28	1.13	1.28	1.06	This study				
4243	M90710-2c	CAOB	Altai	91.5275	47.9944	Granodiorite		387	387	2.74	13.70	0.1209	0.512608	0	-0.39	-0.59	3.16	3.16	3.16	0.88	0.89	0.88	0.89	0.86	This study				
4244	M90710-2a	CAOB	Altai	91.5275	47.9944	Diorite		387	387	3.24	12.22	0.1600	0.512940	1	-0.19	5.89	7.70	7.70	7.70	0.50	0.60	0.50	0.60	0.33	This study				
4245	A15703-4	CAOB	Altai, unit 6	90.3058	46.4633	Alkaline granite	Ageletobe	278	278	8.45	40.06	0.1275	0.512603	5	-0.35	-0.68	1.78	1.78	1.78	0.90	0.97	0.90	0.97	0.90	This study (By Song P.).				
4246	A14905-5	CAOB	Altai, unit 4	90.8124	46.4699	Qtz diorite	Chagandong	384	384	2.03	11.80	0.1040	0.512356		-0.47	-5.50	-0.95	-0.96	-0.96	1.21	1.10	1.21	1.11	1.21	This study (By Song P.).				
4247	A14830-2	CAOB	Altai, unit 3	90.6744	46.8142	Granodiorite	Sonkirk	285	285	5.71	27.10	0.1274	0.512461		-0.35	-3.45	-0.94	-0.93	-0.93	1.12	1.22	1.12	1.22	1.13	This study (By Song P.).				
4248	A14829-1	CAOB	Altai, unit 3	90.2696	46.9479	Granodiorite	Kungeyt	260	260	3.40	17.20	0.1195	0.512664	10	-0.39	0.51	3.08	3.07	3.07	0.78	0.79	0.78	0.79	0.78	This study (By Song P.).				
4249	380205	CAOB	Altai, unit 4	90.2717	47.0881	Porphyry granodiorite	kalateyubie	363	363	2.85	14.60	0.1180	0.512436		-0.40	-3.94	-0.28	-0.29	-0.29	1.14	1.14	1.14	1.14	1.14	This study (By Song P.).				
4250	3005	CAOB	Altai, unit 3	90.2067	46.9450	Qtz diorite	Kugilt	381	381	5.67	27.70	0.1237	0.512315		-0.37	-6.30	-2.76	-2.75	-2.75	1.35	1.41	1.35	1.42	1.35	This study (By Song P.).				
4251	3002	CAOB	Altai, unit 3	90.3426	46.8826	Qtz diorite	Jusling	403	403	5.19	27.70	0.1133	0.512400		-0.42	-4.64	-0.35	-0.35	-0.35	1.17	1.14	1.17	1.14	1.18	This study (By Song P.).				
4252	T15709-2	CAOB	Altai, unit 7	94.0560	43.5316	Alkaline granite	Uzumbrak	301	301	3.58	15.71	0.1378	0.512772	5	-0.30	2.61	4.87	4.88	4.88	0.66	0.76	0.66	0.76	0.76	This study (By Song P.).				
4253	M90722-3E	CAOB	Mongolia	96.2534	46.2848	Plagioclase granite		420	420	0.35	1.10	0.1924	0.512869	1	-0.02	4.51		4.74	4.74	0.77	2.00	0.77			This study (by Tong Y.).				
4254	M90722-3A	CAOB	Mongolia	96.2534	46.2848	Plagioclase granite		420	420	0.30	1.00	0.1814	0.512913	1	-0.08	5.36		6.18	6.18	0.66	1.12	0.66			This study (by Tong Y.).				
4255	M90722-1F	CAOB	Mongolia	94.9088	45.7120	Hb.two-mica granite		490	490	2.09	11.60	0.1089	0.512439	8	-0.45	-3.88		1.61	1.61	1.09	1.03	1.09			This study (by Tong Y.).				
4256	M90722-1B	CAOB	Mongolia	94.9088	45.7120	Two-mica granite		490	490	2.40	13.80	0.1051	0.512431	1	-0.47	-4.04		1.69	1.69	1.08	1.01	1.08			This study (by Tong Y.).				
4257	M90720-3A	CAOB	Mongolia	94.3360	45.5021	Granodiorite		394	394	8.62	42.00	0.1241	0.512553	1	-0.37	-1.66		1.99	1.99	0.98	1.02	0.98			This study (by Tong Y.).				
4258	M90719-13B	CAOB	Mongolia	93.9770	45.4018	K-feldspar granite		285	285	4.93	26.30	0.1133	0.512749	9	-0.42	2.17		5.20	5.20	0.63	0.61	0.63			This study (by Tong Y.).				
4259	M90719-13A	CAOB	Mongolia	93.9770	45.4018	K-feldspar granite		285	285	4.66	25.40	0.1109	0.512761	1	-0.44	2.40		5.52	5.52	0.60	0.58	0.60			This study (by Tong Y.).				
4260	M90717-1C	CAOB	Mongolia	92.7579	45.8458	Porphyritic granodiorite		312	312	7.79	44.30	0.1063	0.512689	1	-0.46	0.99		4.59	4.59	0.70	0.65	0.70			This study (by Tong Y.).				
4261	M1499-20.1	CAOB	Mongolia	109.9341	43.1352	Gneissic monzogranit		960	600	9.80	46.50	0.1277	0.512003	11	-0.35	-12.39		-7.11	-7.11	1.88	2.03	1.88			This study (by Tong Y.).				
4262	M1499-19.5	CAOB	Mongolia	109.9322	43.1232	Gneissic granodiorite		916	600	4.14	22.29	0.1122	0.512455	0	-0.43	-3.57		2.90	2.90	1.07	1.04	1.07			This study (by Tong Y.).				
4263	M1499-10.2	CAOB	Mongolia	109.6132	43.3321	Gneissic monzogranit		916	600	7.84	35.24	0.1344	0.512104	0	-0.32	-10.41		-5.65	-5.65	1.76	2.00	1.76			This study (by Tong Y.).				
4264	M1494-14.2	CAOB	Mongolia	109.3832	45.2312	Granitic gneiss		905	600	7.80	36.91	0.1285	0.511908	0	-0.35	-14.24		-9.02	-9.02	2.04	2.21	2.04			This study (by Tong Y.).				
4265	M1494-14.1	CAOB	Mongolia	109.3832	45.2312	Granitic gneiss		905	600	5.63	28.70	0.1186	0.511928	0	-0.40	-13.84		-7.86	-7.86	1.94	1.95	1.94			This study (by Tong Y.).				
4266	M14915-4.2	CAOB	Mongolia	106.9827	43.0306	Alkaline granite		259	259	34.59	147.70	0.1416	0.512487	14	-0.28	-2.95		-1.12	-1.12	1.12	1.40	1.12			This study (by Tong Y.).				
4267	M14915-4.1	CAOB	Mongolia	106.9827	43.0306	Alkaline granite		259	259	20.14	93.90	0.1297	0.512962	13	-0.34	6.32		8.53	8.53	0.33	0.34	0.33			This study (by Tong Y.).				
4268	M14914-6.2	CAOB	Mongolia	108.4000	43.1445	Ryolite		301	301	9.21	45.70	0.1218	0.512845	16	-0.38	4.04		6.91	6.91	0.50	0.51	0.50			This study (by Tong Y.).				
4269	M14914-6.1	CAOB	Mongolia	108.4000	43.1445	Ryolite		301	301	9.55	49.60	0.1164	0.512812	8	-0.41	3.39		6.48	6.48	0.53	0.53	0.53			This study (by Tong Y.).				
4270	M14914-35.5	CAOB	Mongolia	109.6743	42.8725	Granite		483	483	5.80	33.60	0.1045	0.512238	9	-0.47	-7.81		-2.12	-2.12	1.38	1.27	1.38			This study (by Tong Y.).				
4271	M14914-35.2	CAOB	Mongolia	109.6743	42.8725	Granite		483	483	5.22	29.27	0.1078	0.512258	11	-0.45	-7.42		-1.94	-1.94	1.37	1.28	1.37			This study (by Tong Y.).				
4272	M14914-12.1	CAOB	Mongolia	107.1949	43.1787	Alkaline granite		310	310	29.93	162.60	0.1113	0.512843	14	-0.43	4.00		7.38	7.38	0.47	0.46	0.47			This study (by Tong Y.).				
4273	M13723-8.2	CAOB	Mongolia	110.7034	45.8053	Gneissic granodiorite		130	150	1.02	6.37	0.0970	0.512646	0	-0.51	0.16		2.06	2.06	0.77	0.66	0.77			This study (by Tong Y.).				
4274	M13723-8.1	CAOB	Mongolia	110.7034	45.8053	Gneissic granodiorite		130	150	0.93	5.63	0.0996	0.512651	0	-0.49	0.26		2.11	2.11	0.77	0.67	0.77			This study (by Tong Y.).				
4275	M13723-33.2	CAOB	Mongolia	110.6649	45.8123	M-grained granite		141	150	3.38	18.95	0.1079	0.512722	0	-0.45	1.64		3.34	3.34	0.67	0.62	0.67			This study (by Tong Y.).				
4276	M13723-33.1	CAOB	Mongolia	110.6649	45.8123	M-grained granite		141	150	3.42	19.38	0.1066	0.512729		-0.46	1.77		3.50	3.50	0.65	0.60	0.65			This study (by Tong Y.).				
4277	M13722-10.2	CAOB	Mongolia	110.6846	45.8279	M-grained Bi granite		125	150	3.67	19.42	0.1142	0.512720	0	-0.42	1.60		3.18	3.18	0.68	0.66	0.68			This study (by Tong Y.).				
4278	M13722-10.1	CAOB	Mongolia	110.6846	45.8279	M-grained Bi granite		125	150	3.76	20.59	0.1105	0.512724	0	-0.44	1.68		3.33	3.33	0.67	0.63	0.67			This study (by Tong Y.).				
4279	M13718-3.2	CAOB	Mongolia	115.0816	45.7968	-c-grained K-feldspar granite		179	179	3.75	17.32	0.1310	0.512581	0	-0.33	-1.12		0.38	0.38	0.93	1.05	0.93			This study (by Tong Y.).				
4280	M13718-3.1	CAOB	Mongolia	115.0816	45.7968	-c-grained K-feldspar granite		179	179	4.06	18.93	0.1298	0.512573	0	-0.34	-1.27		0.26	0.26	0.94	1.05	0.94			This study (by Tong Y.).				
4281	M13717-5.1	CAOB	Mongolia	115.6743	45.8209	C-grained Hb granodiorite		301	301	7.56	31.00	0.1473	0.512566	0	-0.25	-1.40		0.49	0.49	1.02	1.34	1.02			This study (by Tong Y.).				
4282	M13717-11.1	CAOB	Mongolia	115.6031	45.6515	M-c-grained Bi monzogranite		308	308	9.03	39.38	0.1386	0.512624	0	-0.30	-0.27		2.01	2.01	0.90	1.07	0.90			This study (by Tong Y.).				
4283	M13713-9.2	CAOB	Mongolia	114.0285	47.1848	-f-grained K-feldspar granite		238	238	1.35	7.77	0.1049	0.512546	0	-0.47	-1.79		1.00	1.00	0.93	0.85	0.93			This study (by Tong Y.).				
4284	M13713-9.1	CAOB	Mongolia	114.0285	47.1848	-f-grained K-feldspar granite		238	238	1.20	7.23	0.1005	0.512533		-0.49	-2.05		0.87	0.87	0.94	0.83	0.94			This study (by Tong Y.).				
4285	M13713-10.1	CAOB	Mongolia	113.6868	47.1290	Monzogranite		243	243	3.72	30.89	0.0729	0.512491	0	-0.63	-2.87		0.97	0.97	0.94	0.71	0.94			This study (by Tong Y.).				
4286	M13712-6.1	CAOB	Mongolia	114.3325	47.7901	Andesite		130	150	5.93	35.98	0.0996	0.512599	0	-0.49	-0.76		1.10	1.10	0.85	0.74	0.85			This study (by Tong Y.).				
4287	M1288-2.1	CAOB	Mongolia	111.4038	43.7327	ylonitized felsic volcanic rock		305	305	3.28	16.40	0.1209	0.512558	27	-0.39	-1.56		1.39	1.39	0.95	0.97	0.95			This study (by Tong Y.).				
4288	M1288-1.7	CAOB	Mongolia	111.3725	43.7044	M-grained monzogranite		320	320	3.50	13.00	0.1628	0.512627	11	-0.17	-0.21		1.17	1.17	0.98	1.56	0.98			This study (by Tong Y.).				
4289	M1288-1.4	CAOB	Mongolia	111.3725	43.7044	M-grained monzogranite		275	275	2.66	8.00	0.2010	0.512748	1	0.02	2.15		1.99	1.99	0.87	4.76	0.87			This study (by Tong Y.).				
4290	M1288-1.1	CAOB	Mongolia	111.3725	43.7044	M-grained monzogranite		275	275	3.18	9.40	0.2045	0.512729	1	0.04	1.78		1.50	1.50	0.90	6.84	0.90			This study (by Tong Y.).				
4291	M1287-5.1	CAOB	Mongolia	110.3191	43.8272	M-grained syenogranite		306	306	2.29	13.60	0.1018	0.512717	9	-0.48	1.54		5.25	5.25	0.64	0.59	0.64			This study (by Tong Y.).				
4292	M1287-3.2	CAOB	Mongolia	109.9176	43.7356	d fleshy red potash feldspar granite		270	270	3.04	16.20	0.1134	0.512756	1	-0.42	2.30		5.17	5.17	0.62	0.60	0.62			This study (by Tong Y.).				
4293	M1287-3.1	CAOB	Mongolia	109.9176	43.7356	d fleshy red potash feldspar granite		270	270	2.67	13.80	0.1170	0.512721	11	-0.41	1.62		4.37	4.37	0.68	0.68	0.68			This study (by Tong Y.).				
4294	M1287-2.3	CAOB	Mongolia	109.8889	43.7221	M-c-grained Bi monzogranite		274	274	4.20	22.70	0.1119	0.512729	8	-0.43	1.78		4.74	4.74	0.65	0.63	0.65			This study (by Tong Y.).				
4295	M1287-2.1	CAOB	Mongolia	109.8889	43.7221	M-c-grained Bi monzogranite		274	274	3.33	19.40	0.1038	0.512719	9	-0.47	1.58		4.83	4.83	0.65	0.60	0.65			This study (by Tong Y.).				
4296	M1286-6.1	CAOB	Mongolia	110.0750	43.1305	rained gneissic muscovite granite		942	600	0.91	3.11	0.1768	0.512119	0	-0.10	-10.13		-8.60	-8.60	1.99	4.21	1.99			This study (by Tong Y.).				
4297	M1286-1.2	CAOB	Mongolia	110.1094	43.1320	Gneissic granite		900	600	3.69	16.21	0.1379	0.512092	0	-0.30	-10.65		-6.15	-6.15	1.80	2.12	1.80			This study (by Tong Y.).				
4298	M1286-1.1	CAOB	Mongolia	110.1094	43.1320	Gneissic granite		900	600	5.94	27.16	0.1321	0.512094	0	-0.33	-10.61		-5.67	-5.67	1.76	1.97	1.76			This study (by Tong Y.).				
4299	M1285-6.1	CAOB	Mongolia	109.8464	42.9615	Monzonite syenite		237	237	6.79	44.20	0.0929	0.512338	1	-0.53	-5.85		-2.71	-2.71	1.23	1.02	1.23			This study (by Tong Y.).				
4300	M1285-3.1	CAOB	Mongolia	109.9076	42.9006	grained gray white granodiorite		469	469	2.49	15.10	0.0997	0.512160	11	-0.49	-9.32		-3.52	-3.52	1.49	1.32	1.49			This study (by Tong Y.).				
4301	M1285-10.2	CAOB	Mongolia	109.7892	43.0452	Mylonitized granite		275	275	4.23	18.60	0.1375	0.512641	14	-0.30	0.06		2.14	2.14	0.87	1.02	0.87			This study (by Tong Y.).				
4302	M1285-10.1	CAOB	Mongolia	109.7892	43.0452	Mylonitized granite		275	275	5.32	25.40	0.1266	0.512632	1	-0.36	-0.12		2.34	2.34	0.85	0.91	0.85			This study (by Tong Y.).				
4303	M1285-1.1	CAOB	Mongolia	110.0054	42.9781	ssium feldspar granite porphyry		244	244	6.29	43.30	0.0878	0.512372	9	-0.55	-5.19		-1.80	-1.80	1.16	0.94	1.16			This study (by Tong Y.).				
4304	M1284-9.1	CAOB	Mongolia	110.0576	43.2603	lium grained K-feldspar granite		220	220	5.37	33.50	0.0969	0.512321	9	-0.51	-6.18		-3.38	-3.38	1.27	1.08	1.27			This study (by Tong Y.).				
4305	M1284-8.1	CAOB	Mongolia	110.0370	43.2815	K-feldspar (syenogranite)		222	222	5.89	35.50	0.1003	0.512351	9	-0.49	-5.60		-2.87	-2.87	1.23	1.07	1.23			This study (by Tong Y.).				
4306	M1284-7.1	CAOB	Mongolia	110.0368	43.2816	Gneissic granite		326	326	2.31	11.70	0.1194	0.512620	11	-0.39	-0.35		2.86	2.86	0.85	0.86	0.85			This study (by Tong Y.).				
4307	M1284-5.1	CAOB	Mongolia	110.0451	43.3117	Syenogranite		220	220	4.96	29.40	0.1020	0.512330	8	-0.48	-6.01		-3.35	-3.35	1.27	1.12	1.27			This study (by Tong Y.).				
4308	M1284-20.1	CAOB	Mongolia	110.0479	43.1654	Diorite		375	375	1.05	3.80	0.1671	0.512366	14	-0.15	-5.31		-3.89	-3.89	1.43	2.55	1.43			This study (by Tong Y.).				

4309	M1284-19	CAOB	Mongolia	110.0215	43.1697	felsic gneiss		392	392	1.94	9.20	0.1275	0.512477	1	-0.35	-3.14		0.32	0.32	1.11	1.19	1.11			This study (by Tong Y.).				
4310	M1284-17.1	CAOB	Mongolia	110.0242	43.1980	M-c-grained Bi granite		230	230	3.62	19.90	0.1100	0.512442	19	-0.44	-3.82		-1.28	-1.28	1.11	1.04	1.11			This study (by Tong Y.).				
4311	M1284-10.1	CAOB	Mongolia	110.0805	43.2296	Medium coarse Bi granite		217	217	4.99	29.70	0.1016	0.512440	1	-0.48	-3.86		-1.23	-1.23	1.09	0.97	1.09			This study (by Tong Y.).				
4312	M1283-3.1	CAOB	Mongolia	109.9752	43.0691	Dacite		380	380	4.87	27.30	0.1078	0.512477	1	-0.45	-3.14		1.17	1.17	1.03	0.97	1.03			This study (by Tong Y.).				
4313	M12731-7.3	CAOB	Mongolia	109.3299	42.6989	Granitic mylonite		286	286	7.51	32.20	0.1410	0.512362	12	-0.28	-5.38		-3.35	-3.35	1.32	1.65	1.32			This study (by Tong Y.).				
4314	M12731-7.1	CAOB	Mongolia	109.3299	42.6989	Granitic mylonite		286	286	5.92	25.70	0.1393	0.512402	14	-0.29	-4.60		-2.51	-2.51	1.25	1.53	1.25			This study (by Tong Y.).				
4315	M12731-6.3	CAOB	Mongolia	109.3204	42.6982	K-feldspar granite		288	288	4.21	17.90	0.1422	0.512323	14	-0.28	-6.14		-4.14	-4.14	1.39	1.76	1.39			This study (by Tong Y.).				
4316	M12731-6.1	CAOB	Mongolia	109.3204	42.6982	K-feldspar granite		288	288	6.67	30.70	0.1313	0.512397	13	-0.33	-4.70		-2.30	-2.30	1.24	1.39	1.24			This study (by Tong Y.).				
4317	M12731-17.3	CAOB	Mongolia	109.1334	42.6419	Bi granite		256	256	0.99	6.20	0.0965	0.512578	8	-0.51	-1.17		2.10	2.10	0.85	0.74	0.85			This study (by Tong Y.).				
4318	M12731-17.1	CAOB	Mongolia	109.1334	42.6419	Bi granite		256	256	1.02	6.40	0.0964	0.512592	15	-0.51	-0.90		2.38	2.38	0.83	0.73	0.83			This study (by Tong Y.).				
4319	M12731-15.2	CAOB	Mongolia	109.1084	42.6157	Granite		279	279	7.35	38.60	0.1151	0.512283	15	-0.41	-6.92		-4.02	-4.02	1.37	1.34	1.37			This study (by Tong Y.).				
4320	M12731-15.1	CAOB	Mongolia	109.1084	42.6157	Granite		279	279	13.60	62.30	0.1320	0.512290	12	-0.33	-6.79		-4.48	-4.48	1.41	1.60	1.41			This study (by Tong Y.).				
4321	M12730-11.2	CAOB	Mongolia	109.4735	43.0145	Monzogranite		283	283	6.54	36.80	0.1074	0.512406	11	-0.45	-4.53		-1.30	-1.30	1.15	1.07	1.15			This study (by Tong Y.).				
4322	M12730-11.1	CAOB	Mongolia	109.4735	43.0145	Monzogranite		283	283	6.44	32.90	0.1183	0.512403	13	-0.40	-4.58		-1.75	-1.75	1.19	1.19	1.19			This study (by Tong Y.).				
4323	M12730-10.1	CAOB	Mongolia	109.5168	43.0029	Mylonitized granite		285	285	5.03	22.90	0.1328	0.512328	8	-0.32	-6.05		-3.72	-3.72	1.35	1.55	1.35			This study (by Tong Y.).				
4324	M12729-8.1	CAOB	Mongolia	109.5262	43.0770	Gneissic granodiorite		386	386	2.58	15.40	0.1013	0.512360	4	-0.49	-5.42		-0.72	-0.72	1.19	1.07	1.19			This study (by Tong Y.).				
4325	M12729-5.3	CAOB	Mongolia	109.3802	43.0202	Strongly deformed granite		285	285	6.43	33.50	0.1160	0.512384	7	-0.41	-4.95		-2.02	-2.02	1.21	1.19	1.21			This study (by Tong Y.).				
4326	M12729-5.1	CAOB	Mongolia	109.3802	43.0202	Strongly deformed granite		285	285	6.09	30.60	0.1203	0.512386	12	-0.39	-4.92		-2.14	-2.14	1.22	1.25	1.22			This study (by Tong Y.).				
4327	M12729-13.1	CAOB	Mongolia	109.5807	43.0592	Felsic volcanic rock		281	281	5.94	33.10	0.1085	0.512272	8	-0.45	-7.14		-3.98	-3.98	1.37	1.27	1.37			This study (by Tong Y.).				
4328	M12729-11.3	CAOB	Mongolia	109.5582	43.0733	Potash feldspar granite		382	382	2.32	12.20	0.1150	0.512393	9	-0.42	-4.78		-0.80	-0.80	1.19	1.17	1.19			This study (by Tong Y.).				
4329	M12729-11.1	CAOB	Mongolia	109.5582	43.0733	Mylonitized granite		381	381	3.76	14.60	0.1557	0.512366	9	-0.21	-5.31		-3.31	-3.31	1.39	2.05	1.39			This study (by Tong Y.).				
4330	M12729-1.3	CAOB	Mongolia	109.3598	43.0161	Gneissic granodiorite		285	285	5.66	30.50	0.1122	0.512275	8	-0.43	-7.08		-4.01	-4.01	1.38	1.31	1.38			This study (by Tong Y.).				
4331	M12729-1.1	CAOB	Mongolia	109.3598	43.0161	Gneissic granodiorite		285	285	5.33	28.70	0.1123	0.512324	24	-0.43	-6.13		-3.06	-3.06	1.30	1.24	1.30			This study (by Tong Y.).				
4332	M12728-5.4	CAOB	Mongolia	109.3614	42.9583	Rhyolite		280	280	1.34	8.30	0.0976	0.512521	8	-0.50	-2.28		1.26	1.26	0.94	0.83	0.94			This study (by Tong Y.).				
4333	M12728-5.1	CAOB	Mongolia	109.3614	42.9583	Rhyolite		280	280	1.16	5.90	0.1189	0.512515	1	-0.40	-2.40		0.38	0.38	1.01	1.02	1.01			This study (by Tong Y.).				
4334	M12727-8.2	CAOB	Mongolia	109.2057	43.1612	K-feldspar granite		253	253	2.15	19.90	0.0653	0.512414	8	-0.67	-4.37		-0.13	-0.13	1.03	0.76	1.03			This study (by Tong Y.).				
4335	M12727-8.1	CAOB	Mongolia	109.2057	43.1612	Potash feldspar granite		253	253	2.34	13.60	0.1040	0.512373	9	-0.47	-5.17		-2.18	-2.18	1.20	1.08	1.20			This study (by Tong Y.).				
4336	M12727-7.1	CAOB	Mongolia	109.1911	43.1630	M-grained Bi granite		432	432	8.66	29.20	0.1793	0.512342	9	-0.09	-5.77		-4.81	-4.81	1.55	3.55	1.55			This study (by Tong Y.).				
4337	M12727-6.2	CAOB	Mongolia	109.1727	43.1601	Gneissic granite		440	440	12.42	74.30	0.1011	0.512337	1	-0.49	-5.87		-0.50	-0.50	1.22	1.10	1.22			This study (by Tong Y.).				
4338	M12727-5	CAOB	Mongolia	109.2664	43.0044	Granite		250	250	3.97	28.20	0.0851	0.512299	9	-0.57	-6.61		-3.05	-3.05	1.27	1.01	1.27			This study (by Tong Y.).				
4339	M12727-20.1	CAOB	Mongolia	109.3179	43.0823	-c-grained K-feldspar granite		406	406	4.61	21.00	0.1327	0.512403	8	-0.33	-4.58		-1.27	-1.27	1.25	1.40	1.25			This study (by Tong Y.).				
4340	M12727-19.1	CAOB	Mongolia	109.3138	43.0933	M-grained gneissic granite		311	311	3.02	16.40	0.1113	0.512328	8	-0.43	-6.05		-2.66	-2.66	1.29	1.22	1.29			This study (by Tong Y.).				
4341	M12727-17.1	CAOB	Mongolia	109.3368	43.1022	Granite		315	315	1.92	9.10	0.1276	0.512384	9	-0.35	-4.95		-2.18	-2.18	1.25	1.35	1.25			This study (by Tong Y.).				
4342	M12727-14.5	CAOB	Mongolia	109.2926	43.1443	Gneissic granite		316	316	6.21	33.90	0.1107	0.512253	9	-0.44	-7.51		-4.04	-4.04	1.40	1.33	1.40			This study (by Tong Y.).				
4343	M12727-10.1	CAOB	Mongolia	109.2165	43.1517	-grained granite granodiorite		429	429	1.47	6.70	0.1326	0.511859	1	-0.33	-15.20		-11.7	-11.7	2.11	2.41	2.11			This study (by Tong Y.).				
4344	M12726-7.3	CAOB	Mongolia	109.2664	43.0044	Diorite		292	292	5.56	29.60	0.1136	0.512365	17	-0.42	-5.33		-2.23	-2.23	1.24	1.19	1.24			This study (by Tong Y.).				
4345	M12726-7.2	CAOB	Mongolia	109.2664	43.0044	M-grained Bi granite		292	292	7.94	43.70	0.1098	0.512342	7	-0.44	-5.77		-2.54	-2.54	1.26	1.18	1.26			This study (by Tong Y.).				
4346	HLH-6	CAOB	NE China	129.50	45.50	Dacite	Huolinhe	135	150	5.39	27.62	0.119900	0.512852	11	-0.390442	4.2	5.55	5.64	5.64	0.49	0.49	0.48	-	-	Feng Guo et al.	2008	International Journal of Earth Sciences	98	1395-1411
4347	HLH-13	CAOB	NE China	129.50	45.50	Dacite	Huolinhe	135	150	5.33	26.96	0.121500	0.512845	10	-0.382308	4.0	5.37	5.48	5.48	0.50	0.50	0.49	-	-	Feng Guo et al.	2008	International Journal of Earth Sciences	98	1395-1411
4348	HLH-14	CAOB	NE China	129.50	45.50	Dacite	Huolinhe	135	150	5.51	26.97	0.125500	0.512779	9	-0.361973	2.8	4.02	4.11	4.11	0.64	0.64	0.60	-	-	Feng Guo et al.	2008	International Journal of Earth Sciences	98	1395-1411
4349	HLH-15	CAOB	NE China	129.50	45.50	Dacite	Huolinhe	135	150	5.63	26.69	0.129700	0.512878	10	-0.340620	4.7	5.88	5.96	5.96	0.49	0.49	0.45	-	-	Feng Guo et al.	2008	International Journal of Earth Sciences	98	1395-1411
4350	HLH-21	CAOB	NE China	129.50	45.50	Rhyolite	Huolinhe	135	150	3.88	22.04	0.108300	0.512773	8	-0.449415	2.6	4.22	4.33	4.33	0.55	0.55	0.59	-	-	Feng Guo et al.	2008	International Journal of Earth Sciences	98	1395-1411
4351	HLH-22	CAOB	NE China	129.50	45.50	Rhyolite	Huolinhe	135	150	3.70	20.81	0.109200	0.512862	11	-0.444840	4.4	5.94	6.04	6.04	0.42	0.42	0.45	-	-	Feng Guo et al.	2008	International Journal of Earth Sciences	98	1395-1411
4352	HLH-23	CAOB	NE China	129.50	45.50	Rhyolite	Huolinhe	135	150	2.61	15.63	0.102800	0.512801	9	-0.48	3.2	4.87	4.98	4.98	0.48	0.48	0.53	-	-	Feng Guo et al.	2008	International Journal of Earth Sciences	98	1395-1411
4353	HLH-24	CAOB	NE China	129.50	45.50	Rhyolite	Huolinhe	135	150	2.92	17.16	0.104600	0.512825	9	-0.47	3.6	5.30	5.41	5.41	0.45	0.45	0.50	-	-	Feng Guo et al.	2008	International Journal of Earth Sciences	98	1395-1411
4354	QB2-38	CAOB	NW China	82.00	42.83	granitoids	Jingbulake	431	431	0.86	2.22	0.233200	0.512204	11	0.19	-8.5	2.40	-10.47	-10.47	2.08	-7.60	2.08	-	-	Sheng-Hong Yang et al.	2009	Lithos	113	259-273
4355	QB2-45	CAOB	NW China	82.00	42.83	granitoids	Jingbulake	431	431	0.74	1.98	0.225700	0.512856	12	0.15	4.3	2.70	2.66	2.66	0.97	-3.79	0.97	-	-	Sheng-Hong Yang et al.	2009	Lithos	113	259-273
4356	QB2-60	CAOB	NW China	82.00	42.83	granitoids	Jingbulake	431	431	0.31	0.82	0.226300	0.51281	12	0.15	3.4	1.70	1.73	1.73	1.04	-4.18	1.04	-	-	Sheng-Hong Yang et al.	2009	Lithos	113	259-273
4357	QB2-140	CAOB	NW China	82.00	42.83	granitoids	Jingbulake	431	431	10.7	34.60	0.187700	0.512729	12	-0.05	1.8	2.30	2.27	2.27	0.98	2.46	0.98	-	-	Sheng-Hong Yang et al.	2009	Lithos	113	259-273
4358	QB2-18	CAOB	NW China	82.00	42.83	granitoids	Jingbulake	431	431	1.04	2.80	0.224700	0.5128	14	0.14	3.2	1.60	1.62	1.62	1.06	-4.94	1.06	-	-	Sheng-Hong Yang et al.	2009	Lithos	113	259-273
4359	QB2-82	CAOB	NW China	82.00	42.83	granitoids	Jingbulake	431	431	0.46	1.36	0.204000	0.512755	11	0.04	2.3	1.90	1.88	1.88	1.00	6.10	1.00	-	-	Sheng-Hong Yang et al.	2009	Lithos	113	259-273
4360	QB2-111	CAOB	NW China	82.00	42.83	granitoids	Jingbulake	431	431	8.58	29.10	0.178000	0.512702	13	-0.10	1.2	2.30	2.28	2.28	0.98	1.91	0.98	-	-	Sheng-Hong Yang et al.	2009	Lithos	113	259-273
4361	QB2-127	CAOB	NW China	82.00	42.83	granitoids	Jingbulake	431	431	0.52	1.68	0.187200	0.512749	21	-0.05	2.2	2.70	2.69	2.69	0.95	2.30	0.95	-	-	Sheng-Hong Yang et al.	2009	Lithos	113	259-273
4362	QB2-155	CAOB	NW China	82.00	42.83	granitoids	Jingbulake	431	431	1.11	3.35	0.200800	0.512855	12	0.02	4.2	4.00	4.01	4.01	0.84	3.46	0.84	-	-	Sheng-Hong Yang et al.	2009	Lithos	113	259-273
4363	Heng-1	CAOB	NE China	129.10	44.86	Adamellite	Hengdaohezi	170	170	1.808	10.10	0.108300	0.51266	9	-0.45	0.4	2.35	2.35	2.35	0.71	0.71	0.76	0.71	0.76	Fu-yuan Wu et al.	2000	Tectonophysics	328	89-113
4364	9501-1	CAOB	NE China	128.85	42.64	Granodiorite	Shimeng	245	245	3.971	19.37	0.123900	0.512544	10	-0.37	-1.8	0.44	0.44	0.44	1.03	1.03	0.98	1.03	0.98	Fu-yuan Wu et al.	2000	Tectonophysics	328	89-113
4365	Tian-1	CAOB	NE China	126.83	44.01	Adamellite	Tiangang	151	151	4.674	25.78	0.109600	0.512584	8	-0.44	-1.1	0.63	0.62	0.62	0.83	0.83	0.89	0.83	0.89	Fu-yuan Wu et al.	2000	Tectonophysics	328	89-113
4366	9701-1	CAOB	NE China	126.83	44.01	Monzogranite	Tiangang	151	151	5.57	31.72	0.106200	0.512581	6	-0.46	-1.1	0.63	0.63	0.63	0.81	0.81	0.89	0.81	0.89	Fu-yuan Wu et al.	2000	Tectonophysics	328	89-113
4367	9704-2	CAOB	NE China	126.59	44.04	Diorite	Jiangmifeng	164	164	4.745	26.10	0.109900	0.512583	7	-0.44	-1.1	0.74	0.74	0.74	0.83	0.83	0.89	0.83	0.89	Fu-yuan Wu et al.	2000	Tectonophysics	328	89-113
4368	9715-1	CAOB	NE China	127.05	43.85	Af granite	Tianqiaogang	191	191	6.662	35.13	0.114600	0.512656	5	-0.42	0.4	2.35	2.35	2.35	0.76	0.76	0.78	0.76	0.78	Fu-yuan Wu et al.	2000	Tectonophysics	328	89-113
4369	9718-1	CAOB	NE China	127.06	43.86	Syenogranite	Tianqiaogang	196	196	6.645	32.92	0.122000	0.512614	6	-0.38	-0.5	1.35	1.40	1.40	0.89	0.89	0.86	0.89	0.86	Fu-yuan Wu et al.	2000	Tectonophysics	328	89-113
4370	Bai-1	CAOB	NE China	126.98	43.43	Granodiorite	Baishishan	196	196	3.494	19.48	0.108400	0.512609	7	-0.45	-0.6	1.64	1.64	1.64	0.78	0.78	0.84	0.78	0.84	Fu-yuan Wu et al.	2000	Tectonophysics	328	89-113
4371	9724-1	CAOB	NE China	126.98	43.45	Tonalite	Baishishan	196	196	3.306	19.28	0.103700	0.512603	5	-0.47	-0.7	1.65	1.64	1.64	0.76	0.76	0.84	0.76	0.84	Fu-yuan Wu et al.	2000	Tectonophysics	328	89-113
4372	9725-1	CAOB	NE China	126.97	43.44	Granodiorite	Baishishan	185	185	3.115	17.07	0.110300	0.512611	6	-0.44	-0.5	1.64	1.51	1.51	0.79	0.79	0.84	0.80	0.84	Fu-yuan Wu et al.	2000	Tectonophysics	328	89-113
4373	9747-1	CAOB	NE China	127.86	45.21	Monzogranite	Wujimi	183	183	2.108	14.52	0.087800	0.512613	6	-0.55	-0.5	2.09	2.06	2.06	0.65	0.65	0.80	0.65	0.80	Fu-yuan Wu et al.	2000	Tectonophysics	328	89-113
4374	9749-1	CAOB	NE China	127.73	45.26	Monzogranite	Dawangzhezi	183	183	3.994	23.24	0.103900	0.51273	5	-0.47	1.8	3.96	3.96	3.96	0.58	0.58	0.64	0.58	0.64	Fu-yuan Wu et al.	2000	Tectonophysics	328	89-113
4375	9749-4	CAOB	NE China	127.74	45.21	Monzogranite	Dawangzhezi	195	195	4.006	22.68	0.106800	0.512695	7	-0.46	1.1	3.21	3.35	3.35	0.65	0.65	0.70	0.65	0.70	Fu-yuan Wu et al.	2000	Tectonophysics	328	89-113
4376	9756-1	CAOB	NE China	128.33	45.44	Granodiorite	Yanshou	195	195	4.641	24.45	0.114700	0.512623	7	-0.42	-0.3	1.75	1.75	1.75	0.81	0.81	0.83	0.81	0.83	Fu-yuan Wu et al.	2000	Tectonophysics	328	89-113
4377	9756-3	CAOB	NE China	128.25	45.47	Syenogranite	Yanshou	210	210	4.456	26.12	0.103100	0.512477	6	-0.48	-3.1	-0.81	-0.63	-0.63	0.93	0.93	1.04	0.93	1.04	Fu-yuan Wu et al.	2000	Tectonophysics	328	89-113
4378	9757-3	CAOB	NE China	127.22	46.92	Alkaline Granite	Maojiatun	210	210	3.047	13.09	0.140800	0.512573	7	-0.28	-1.3	0.23	0.23	0.23	0.97	1.21	0.97	1.21	0.97	Fu-yuan Wu et al.	2000	Tectonophysics	328	89-113
4379	9757-4	CAOB	NE China	127.23	46.92	Alkaline Granite	Maojiatun	206	206	2.235	9.813	0.137700	0.512484	10	-0.30	-3.0	-1.42	-1.45	-1.45	1.33	1.33	1.10	1.33	1.10	Fu-yuan Wu et al.	2000	Tectonophysics	328	89-113
4380	9765-1	CAOB	NE China	128.89	46.94	Granodiorite	Langxiang	206	206	6.473	31.75	0.123200	0.512646	15	-0.37	0.2	2.09	2.09	2.09	0.85	0.85	0.81	0.85	0.81	Fu-yuan Wu et al.	2000	Tectonophysics	328	89-113
4381	9766-2	CAOB	NE China	128.88	46.95	Granodiorite	Langxiang	200	200	5.59	31.48	0.107400	0.512341	7	-0.45	-5.8	-3.45	-3.51	-3.51	1.16	1.16	1.27	1.16	1.27	Fu-yuan Wu et al.	2000	Tectonophysics	328	89-113
4382	9767-1	CAOB	NE China	128.58	47.96	Alkaline Granite	Milin	200	200	11.78	41.59	0.171300	0.512597	7	-0.13	-0.8	-0.15	-0.15	-0.15	0.99	1.98	0.99	1.98	0.99	Fu-yuan Wu et al.	2000	Tectonophysics	328	89-113
4383	9771-1	CAOB	NE China	128.79	46.75	Granodiorite	Tuanjie	200	200	3.837	22.86	0.101500	0.512494	9	-0.48	-2.8	-0.38	-0.38	-0.38	0.89	0.89	1.01	0.89	1.01	Fu-yuan Wu et al.	2000	Tectonophysics	328	89-113
4384	9773-1	CAOB	NE China	128.59	46.85	Monzogranite	Xiaobai	200	200	3.164	17.15	0.111500	0.512465	11	-0.43	-3.4	-1.20	-1.20	-1.20	1.02	1.02	1.08	1.02	1.08	Fu-yuan Wu et al.	2000	Tectonophysics	328	89-113
4385	9773-2	CAOB	NE China	128.62	46.8	Monzogranite	Xiaobai	130	150	2.838	15.42	0.111300	0.512522	16	-0.43	-2.3	-0.08	-0.63	-0.63	0.93	0.93	0.99	0.94	0.99	Fu-yuan Wu et al.	2000	Tectonophysics	328	89-113
4386	9776-6	CAOB	NE China	127.36	45.66	Granodiorite	Shichang	130	150	5.02	29.62	0.102500	0.512449	5	-0.48	-3.7	-2.12	-1.88	-1.88	0.96	0.96	1.09	0.96	1.10	Fu-yuan Wu et al.	2000	Tectonophysics	328	89-113
4387	9777-2	CAOB	NE China	127.38	45.56	Granodiorite	Shichang	170	170	4.788	28.27	0.102400	0.512411	6	-0.48	-4.4	-2.86	-2.38	-2.38	1.01	1.01	1.15	1.01	1.16	Fu-yuan Wu et al.	2000	Tectonophysics	328	89-113
4388	9780-1	CAOB	NE China	127.87	47.44	Alkaline Granite	Qingshui	170	170	6.689	37.81	0.106900	0.512424	9	-0.46	-4.2	-2.23	-2.23	-2.23	1.04	1.04	1.14	1.04	1.14	Fu-yuan Wu et al.	2000	Tectonophysics	328	89-113
4389	9781-1	CAOB	NE China	127.75	47.47	Alkaline Granite	Qingshui	170	170	17.1	65.7	0.157400	0.512570	7	-0.20	-1.3	-0.47	-0.47	-0.47	0.99	1.57	0.99	1.57	0.99	Fu-yuan Wu et al.	2000	Tectonophysics	328	89-113
4390	9781-5	CAOB	NE China	127.7	47.63	Alkaline Granite	Qingshui	170	170	9.171	34.89	0.158900	0.512548	32	-0.19	-1.8	-0.94	-0.94	-0.94	1.03	1.67	1.03	1.67	1.03	Fu-yuan Wu et al.	2000	Tectonophysics	328	89-113
4391	9782-1	CAOB	NE China	127.8	47.55	Af granite	Qingshui	170	170	6.853	30.65	0.135200	0.512515	6	-0.31	-2.4	-1.06	-1.07	-1.07	1.23	1.23	1.04	1.23	1.04	Fu-yuan Wu et al.	2000	Tectonophysics	328	89-113
4392	97100-2	CAOB	NE China	127.06	47.26	Granodiorite	Dongqing	207	207	1.558	9.652	0.097600	0.512480	5	-0.50	-3.1	-0.46	-0.46	-0.46	0.88	0.88	1.02	0.88	1.02	Fu-yuan Wu et al.	2000	Tectonophysics	328	89-113
4393	97100-5	CAOB	NE China	127.1	47.3	Monzogranite	Dongqing	207	207	2.248	14.63	0.092900	0.512458	16	-0.53	-3.5	-0.77	-0.77	-0.77	0.87	0.87	1.05	0.87	1.05	Fu-yuan Wu et al.	2000	Tectonophysics	328	89-113
4394	9503-4	CAOB	NE China	127.12	47.31	Granodiorite	Dongqing	207	207	2.103	12.36	0.102900	0.512489	7	-0.48	-2.9	-0.43	-0.43	-0.43	0.91	0.91	1.02	0.91	1.02	Fu-yuan Wu et al.	2000	Tectonophysics	328	89-113
4395	9503-5	CAOB	NE China	127.08	47.33	Kf-granite	Dongqing	207	207	1.033	3.251	0.192100	0.512534	8	-0.02	-2.0	-1.91	-1.91	-1.91	1.13	4.30	1.13	4.30	1.13	Fu-yuan Wu et al.	2000	Tectonophysics	328	89-113
4396	9798-2	CAOB	NE China	129.98	45.63	Granodiorite	Chushan	253	253	6.095	33.72	0.109300	0.512116	10	-0.44	-10.2	-7.36	-7.36	-7.36	1.51	1.51	1.62	1.51	1.62	Fu-yuan Wu et al.	2000	Tectonophysics	328	89-113
4397	98119	CAOB	NE China	129.76	45.51	Granodiorite	Chushan	254	254	8.73	37.76	0.139800	0.512184	6	-0.29	-8.9	-7.02	-7.01	-7.01	1.99	1.99	1.59	1.99	1.59	Fu-yuan Wu et al.	2000	Tectonophysics	328	89-113
4398	9799-1	CAOB	NE China	129.68	44.88	Monzogranite	Chaihe	254	254	3.572	22.5	0.096000	0.512120	6	-0.51	-10.1	-6.84	-6.84	-6.84	1.33	1.33	1.58	1.33	1.58	Fu-yuan Wu et al.	2000	Tectonophysics	328	89-113
4399	KM2102-5	CAOB	Tianshan	89.97	41.77	Quartz gneiss	Gangou	1100	600	7.49	38.73	0.116900	0.512313	8	-0.41	-6.3	4.91	-0.23	-0.23	1.32	1.32	1.32			QIUGEN LI et al.	2007	Geological Magazine	144	305-317
4400	KM2102-6	CAOB	Tianshan	89.97	41.78	Quartz gneiss	Gangou	1100	600	9.43	36.94	0.100200	0.51224	13	-0.49	-7.8	5.84	-0.38	-0.38	1.22	1.22	1.34	-	-	QIUGEN LI et al.	2007	Geological Magazine	144	305-317
4401	KM2102-7	CAOB	Tianshan	89.98	41.76	Quartz gneiss	Gangou	1100	600	6.48	39.27	0.099800	0.511817	10	-0.49	-16.0	−2.37	-8.60	-8.60	1.78	1.78	2.00	-	-	QIUGEN LI et al.	2007	Geological Magazine	144	305-317
4402	KM2111-1	CAOB	Tianshan	89.98	41.77	Granitic gneiss	Gangou	1100	600	5.34	26.37	0.122400	0.512054	15	-0.38	-11.4	−0.93	-5.70	-5.70	1.82	1.82	1.77	-	-	QIUGEN LI et al.	2007	Geological Magazine	144	305-317
4403	KM2109-1	CAOB	Tianshan	90.03	41.78	Granitic gneiss	Gangou	1100	600	692	30.29	0.138100	0.511993	12	-0.30	-12.6	−4.34	-8.10	-8.10	2.32	2.32	1.96	-	-	QIUGEN LI et al.	2007	Geological Magazine	144	305-317
4404	DK2105-1	CAOB	Tianshan	90.03	41.76	Granitic gneiss	Gangou	1100	600	1.9	8.76	0.131200	0.512087	12	-0.33	-10.7	−1.53	-5.73	-5.73	1.96	1.96	1.77	-	-	QIUGEN LI et al.	2007	Geological Magazine	144	305-317
4405	SS02-2	CAOB	Tianshan	90.02	41.77	Granitic gneiss	Gangou	1100	600	4.23	24.07	0.107700	0.511776	7	-0.45	-16.8	−4.29	-10.00	-10.00	1.97	1.97	2.12	-	-	QIUGEN LI et al.	2007	Geological Magazine	144	305-317
4406	SS02-3	CAOB	Tianshan	90.02	41.78	Granitic gneiss	Gangou	1100	600	7.38	48.00	0.093200	0.511658	6	-0.53	-19.1	−4.55	-11.19	-11.19	1.88	1.88	2.21	-	-	QIUGEN LI et al.	2007	Geological Magazine	144	305-317

4407	SS02-4	CAOB	Tianshan	90.02	41.76	Granitic gneiss	Gangou	1100	600	8.81	52.97	0.100600	0.511689	7	-0.49	-18.5	−4.99	-11.15	-11.15	1.96	1.96	2.21	-	-	QIUGEN LI et al.	2007	Geological Magazine	144	305-317
4408	SS02-5	CAOB	Tianshan	90.03	41.77	Granitic gneiss	Gangou	1100	600	10.72	50.47	0.128500	0.51192	6	-0.35	-14.0	−4.41	-8.78	-8.78	2.19	2.19	2.02	-	-	QIUGEN LI et al.	2007	Geological Magazine	144	305-317
4409	SS02-8	CAOB	Tianshan	90.03	41.78	Granitic gneiss	Gangou	1100	600	6.4	30.60	0.126500	0.511886	9	-0.36	-14.7	−4.80	-9.29	-9.29	2.20	2.20	2.06	-	-	QIUGEN LI et al.	2007	Geological Magazine	144	305-317
4410	TS60	CAOB	Tianshan	90.03	41.76	Granitic gneiss	Gangou	1100	600	6.53	31.86	0.123800	0.51198	26	-0.37	-12.8	−2.58	-7.25	-7.25	1.98	1.98	1.89	-	-	QIUGEN LI et al.	2007	Geological Magazine	144	305-317
4411	TS61	CAOB	Tianshan	90.03	41.77	Granitic gneiss	Gangou	1100	600	12.72	70.15	0.109600	0.51185	30	-0.44	-15.4	−3.11	-8.70	-8.70	1.90	1.90	2.01	-	-	QIUGEN LI et al.	2007	Geological Magazine	144	305-317
4412	TS62	CAOB	Tianshan	90.03	41.78	Granitic gneiss	Gangou	1100	600	3.66	23.80	0.093000	0.511631	6	-0.53	-19.6	−5.05	-11.70	-11.70	1.91	1.91	2.26	-	-	QIUGEN LI et al.	2007	Geological Magazine	144	305-317
4413	TS63	CAOB	Tianshan	90.03	41.76	Granitic gneiss	Gangou	1100	600	3.65	22.11	0.099700	0.51169	34	-0.49	-18.5	−4.84	-11.07	-11.07	1.95	1.95	2.20	-	-	QIUGEN LI et al.	2007	Geological Magazine	144	305-317
4414	18SZ-15	CAOB	Tianshan	91.66	42.10	granitoids	Qiziltag	289	289	7.93	31.26	0.150000	0.512930		-0.24	5.7	7.30	7.42	7.42	0.45	0.53	0.45	-	0.46	M.N. Muhtar et al.	2019	GSA Bulletin	132	0-0
4415	18SZ-16	CAOB	Tianshan	91.66	42.10	granitoids	Qiziltag	289	289	8.20	32.92	0.150000	0.512934		-0.24	5.8	7.48	7.50	7.50	0.44	0.52	0.44	-	0.45	M.N. Muhtar et al.	2019	GSA Bulletin	132	0-0
4416	18SZ-18	CAOB	Tianshan	91.66	42.10	granitoids	Qiziltag	289	289	5.89	21.12	0.170000	0.512938		-0.14	5.9	6.90	6.84	6.84	0.49	0.74	0.49	-	0.49	M.N. Muhtar et al.	2019	GSA Bulletin	132	0-0
4417	18SZ-19	CAOB	Tianshan	91.66	42.10	granitoids	Qiziltag	289	289	5.45	18.96	0.170000	0.512952		-0.14	6.1	6.98	7.11	7.11	0.47	0.69	0.47	-	0.49	M.N. Muhtar et al.	2019	GSA Bulletin	132	0-0
4418	18SZ-20	CAOB	Tianshan	91.66	42.10	granitoids	Qiziltag	289	289	5.79	24.72	0.140000	0.512909		-0.29	5.3	7.32	7.38	7.38	0.50	0.50	0.45	-	0.46	M.N. Muhtar et al.	2019	GSA Bulletin	132	0-0
4419	18SZ-23	CAOB	Tianshan	91.66	42.08	granitoids	Qiziltag	289	289	1.33	5.31	0.150000	0.512904		-0.24	5.2	6.89	6.91	6.91	0.49	0.59	0.49	-	0.50	M.N. Muhtar et al.	2019	GSA Bulletin	132	0-0
4420	18SZ-25	CAOB	Tianshan	91.66	42.08	granitoids	Qiziltag	289	289	1.48	7.62	0.120000	0.512839		-0.39	3.9	6.89	6.75	6.75	0.51	0.51	0.50	-	0.50	M.N. Muhtar et al.	2019	GSA Bulletin	132	0-0
4421	18SZ-26	CAOB	Tianshan	91.66	42.08	granitoids	Qiziltag	289	289	1.83	7.47	0.150000	0.512868		-0.24	4.5	6.29	6.21	6.21	0.55	0.68	0.55	-	0.54	M.N. Muhtar et al.	2019	GSA Bulletin	132	0-0
4422	18SZ-03	CAOB	Tianshan	91.66	42.07	granitoids	Qiziltag	289	289	11.44	24.30	0.280000	0.513138		0.42	9.8	6.54	6.68	6.68	0.51	-0.03	0.51	-	0.52	M.N. Muhtar et al.	2019	GSA Bulletin	132	0-0
4423	18SZ-04	CAOB	Tianshan	91.66	42.07	granitoids	Qiziltag	289	289	10.05	20.38	0.300000	0.513180		0.53	10.6	6.85	6.76	6.76	0.50	0.05	0.50	-	0.50	M.N. Muhtar et al.	2019	GSA Bulletin	132	0-0
4424	18SZ-05	CAOB	Tianshan	91.66	42.07	granitoids	Qiziltag	289	289	12.03	21.40	0.340000	0.513287		0.73	12.7	7.41	7.38	7.38	0.45	0.17	0.45	-	0.45	M.N. Muhtar et al.	2019	GSA Bulletin	132	0-0
4425	18SZ-29	CAOB	Tianshan	91.66	42.11	granitoids	Qiziltag	289	289	7.15	30.07	0.140000	0.512870		-0.29	4.5	6.41	6.62	6.62	0.58	0.58	0.51	-	0.52	M.N. Muhtar et al.	2019	GSA Bulletin	132	0-0
4426	18SZ-30	CAOB	Tianshan	91.66	42.11	granitoids	Qiziltag	289	289	6.82	31.08	0.130000	0.512855		-0.34	4.2	6.51	6.69	6.69	0.54	0.54	0.51	-	0.52	M.N. Muhtar et al.	2019	GSA Bulletin	132	0-0
4427	18SZ-31	CAOB	Tianshan	91.66	42.11	granitoids	Qiziltag	289	289	8.42	40.99	0.120000	0.512847		-0.39	4.1	6.66	6.91	6.91	0.49	0.49	0.49	-	0.50	M.N. Muhtar et al.	2019	GSA Bulletin	132	0-0
4428	18SZ-32	CAOB	Tianshan	91.66	42.11	granitoids	Qiziltag	289	289	7.31	35.93	0.120000	0.512848		-0.39	4.1	6.72	6.92	6.92	0.49	0.49	0.49	-	0.50	M.N. Muhtar et al.	2019	GSA Bulletin	132	0-0
4429	18SZ-33	CAOB	Tianshan	91.66	42.11	granitoids	Qiziltag	289	289	7.44	35.12	0.130000	0.512847		-0.34	4.1	6.53	6.54	6.54	0.55	0.55	0.52	-	0.51	M.N. Muhtar et al.	2019	GSA Bulletin	132	0-0
4430	18SZ-07	CAOB	Tianshan	91.66	42.08	granitoids	Qiziltag	289	289	6.06	15.55	0.240000	0.513177		0.22	10.5	9.13	8.92	8.92	0.33	0.16	0.33	-	0.30	M.N. Muhtar et al.	2019	GSA Bulletin	132	0-0
4431	18SZ-08	CAOB	Tianshan	91.66	42.08	granitoids	Qiziltag	289	289	4.14	10.76	0.230000	0.513200		0.17	11.0	9.69	9.73	9.73	0.29	0.47	0.26	-	0.26	M.N. Muhtar et al.	2019	GSA Bulletin	132	0-0
4432	18SZ-09	CAOB	Tianshan	91.66	42.08	granitoids	Qiziltag	289	289	4.55	11.84	0.230000	0.513172		0.17	10.4	9.16	9.19	9.19	0.30	0.21	0.30	-	0.30	M.N. Muhtar et al.	2019	GSA Bulletin	132	0-0
4433	18SZ-10	CAOB	Tianshan	91.66	42.08	granitoids	Qiziltag	289	289	4.82	12.82	0.230000	0.513180		0.17	10.6	9.49	9.34	9.34	0.29	0.28	0.29	-	0.27	M.N. Muhtar et al.	2019	GSA Bulletin	132	0-0
4434	18SZ-11	CAOB	Tianshan	91.66	42.08	granitoids	Qiziltag	289	289	6.46	17.09	0.230000	0.513182		0.17	10.6	9.48	9.38	9.38	0.29	0.30	0.29	-	0.27	M.N. Muhtar et al.	2019	GSA Bulletin	132	0-0
4435	V015	CAOB	Lake Zone	97.44	45.90	tonalites	marginal	500	500	2.49	6.7	0.224700	0.512907	7	0.14	5.2	3.50	3.46	3.46	0.95	-3.42	0.95	0.95	0.99	Vojtěch Janoušek et al.	2018	Gondwana Research	54	122-149
4436	V012a	CAOB	Lake Zone	97.44	45.89	tonalites	marginal	500	500	2.48	6.3	0.238000	0.512908	10	0.21	5.3	2.60	2.63	2.63	1.02	-1.53	1.02	1.07	1.07	Vojtěch Janoušek et al.	2018	Gondwana Research	54	122-149
4437	V007	CAOB	Lake Zone	97.50	45.85	tonalites	marginal	500	500	1.75	6.6	0.160800	0.512753	5	-0.18	2.2	4.50	4.53	4.53	0.86	1.14	0.86	1.04	0.89	Vojtěch Janoušek et al.	2018	Gondwana Research	54	122-149
4438	V003	CAOB	Lake Zone	97.51	45.79	tonalites	marginal	500	500	4.16	18.5	0.135900	0.512679	5	-0.31	0.8	4.70	4.68	4.68	0.92	0.92	0.84	0.87	0.88	Vojtěch Janoušek et al.	2018	Gondwana Research	54	122-149
4439	V008c	CAOB	Lake Zone	97.51	45.85	tonalites	marginal	500	500	2.66	11.6	0.138600	0.512197	10	-0.30	-8.6	4.90	-4.90	-4.90	1.93	1.93	1.62	1.80	1.79	Vojtěch Janoušek et al.	2018	Gondwana Research	54	122-149
4440	V005	CAOB	Lake Zone	97.51	45.81	tonalites	marginal	500	500	4.06	17.7	0.138700	0.512419	4	-0.29	-4.3	-0.60	-0.57	-0.57	1.48	1.48	1.27	1.39	1.38	Vojtěch Janoušek et al.	2018	Gondwana Research	54	122-149
4441	V004	CAOB	Lake Zone	97.51	45.80	tonalites	marginal	500	500	4.54	23.7	0.115800	0.512669	5	-0.41	0.6	5.80	5.77	5.77	0.75	0.75	0.76	0.71	0.77	Vojtěch Janoušek et al.	2018	Gondwana Research	54	122-149
4442	06XJ160-1	CAOB	Junggar	84.32	45.91	andesites	Hatu	315	315	2.43	7.66	0.193200	0.512927	9	-0.02	5.6	5.80	5.78	5.78	0.60	1.65	0.60	-	-	Gong-Jian Tang et al.	2012	Earth and Planetary Science Letters	0	84-96
4443	06XJ161-2	CAOB	Junggar	84.31	45.91	andesites	Hatu	315	315	2.31	6.95	0.202300	0.512943	10	0.03	5.9	5.80	5.72	5.72	0.60	2.75	0.60	-	-	Gong-Jian Tang et al.	2012	Earth and Planetary Science Letters	0	84-96
4444	06XJ162	CAOB	Junggar	84.31	45.91	andesites	Hatu	315	315	2.14	6.49	0.201000	0.512951	9	0.02	6.1	6.00	5.93	5.93	0.59	2.38	0.59	-	-	Gong-Jian Tang et al.	2012	Earth and Planetary Science Letters	0	84-96
4445	06XJ163-2	CAOB	Junggar	84.31	45.91	andesites	Hatu	315	315	2.50	7.65	0.198800	0.512911	12	0.01	5.3	5.30	5.24	5.24	0.64	2.43	0.64	-	-	Gong-Jian Tang et al.	2012	Earth and Planetary Science Letters	0	84-96
4446	06XJ164-2	CAOB	Junggar	84.31	45.90	andesites	Hatu	315	315	2.14	6.41	0.202900	0.512935	10	0.03	5.8	5.90	5.54	5.54	0.62	3.01	0.62	-	-	Gong-Jian Tang et al.	2012	Earth and Planetary Science Letters	0	84-96
4447	06XJ165	CAOB	Junggar	84.31	45.90	andesites	Hatu	315	315	2.43	7.43	0.199400	0.512935	8	0.01	5.8	5.70	5.69	5.69	0.61	2.28	0.61	-	-	Gong-Jian Tang et al.	2012	Earth and Planetary Science Letters	0	84-96
4448	P2104-2	CAOB	Inner Mongolia	84.31	46.00	Aplitic granite	Diyanqinamu	156	156	0.96	5.97	0.097000	0.51267	10	-0.51	0.6	2.60	2.61	2.61	0.63	0.63	0.73	-	-	Hairui Sun et al.	2015	Ore Geology Reviews	65	70-83
4449	P2104-3	CAOB	Inner Mongolia	84.31	46.00	granites	Diyanqinamu	156	156	1.10	7.37	0.089900	0.51265	6	-0.54	0.2	2.40	2.36	2.36	0.62	0.62	0.75	-	-	Hairui Sun et al.	2015	Ore Geology Reviews	65	70-83
4450	P2104-4	CAOB	Inner Mongolia	84.31	46.00	granites	Diyanqinamu	156	156	1.50	9.56	0.094600	0.512699	10	-0.52	1.2	3.20	3.22	3.22	0.58	0.58	0.68	-	-	Hairui Sun et al.	2015	Ore Geology Reviews	65	70-83
4451	P2104-5	CAOB	Inner Mongolia	84.31	46.00	granites	Diyanqinamu	156	156	1.20	7.66	0.094200	0.512698	10	-0.52	1.2	3.20	3.21	3.21	0.58	0.58	0.68	-	-	Hairui Sun et al.	2015	Ore Geology Reviews	65	70-83
4452	9701-16	CAOB	Inner Mongolia	118.22	46.00	Porphyritic	Diyanqinamu	156	156	3.78	22.6	0.100600	0.512711	12	-0.49	1.4	3.30	3.34	3.34	0.59	0.59	0.67	-	-	Hairui Sun et al.	2015	Ore Geology Reviews	65	70-83
4453	9701-17	CAOB	Inner Mongolia	118.22	46.00	granite	Diyanqinamu	156	156	3.67	21.6	0.102400	0.512725	4	-0.48	1.7	3.60	3.57	3.57	0.58	0.58	0.65	-	-	Hairui Sun et al.	2015	Ore Geology Reviews	65	70-83
4454	9701-18	CAOB	Inner Mongolia	118.22	46.00	granites	Diyanqinamu	156	156	3.08	19.0	0.097800	0.512724	6	-0.50	1.7	3.70	3.65	3.65	0.56	0.56	0.65	-	-	Hairui Sun et al.	2015	Ore Geology Reviews	65	70-83
4455	9701-20	CAOB	Inner Mongolia	118.22	46.00	granites	Diyanqinamu	156	156	3.95	23.4	0.101500	0.512734	8	-0.48	1.9	3.80	3.77	3.77	0.57	0.57	0.64	-	-	Hairui Sun et al.	2015	Ore Geology Reviews	65	70-83
4456	9701-21	CAOB	Inner Mongolia	118.22	46.00	granites	Diyanqinamu	156	156	4.74	26.8	0.106500	0.512733	6	-0.46	1.9	3.70	3.65	3.65	0.59	0.59	0.65	-	-	Hairui Sun et al.	2015	Ore Geology Reviews	65	70-83
4457	ZK3314-4	CAOB	Inner Mongolia	118.22	46.00	Purple fluorite	Diyanqinamu	156	156	19.4	53.9	0.216600	0.512844	8	0.10	4.0	3.6	3.62	3.62	0.70	-17.05	0.70	-	-	Hairui Sun et al.	2015	Ore Geology Reviews	65	70-83
4458	ZK3314-8	CAOB	Inner Mongolia	118.22	46.00	granites	Diyanqinamu	156	156	7.19	19.7	0.219600	0.512785	8	0.12	2.9	2.4	2.41	2.41	0.78	-9.76	0.78	-	-	Hairui Sun et al.	2015	Ore Geology Reviews	65	70-83
4459	ZK3314-19	CAOB	Inner Mongolia	118.22	46.00	granites	Diyanqinamu	156	156	5.56	18.2	0.183800	0.512817	6	-0.07	3.5	3.8	3.75	3.75	0.64	1.69	0.64	-	-	Hairui Sun et al.	2015	Ore Geology Reviews	65	70-83
4460	ZK3314-21	CAOB	Inner Mongolia	118.22	46.00	granites	Diyanqinamu	156	156	5.56	18.2	0.183800	0.512785	4	-0.07	2.9	3.1	3.12	3.12	0.69	1.86	0.69	-	-	Hairui Sun et al.	2015	Ore Geology Reviews	65	70-83
4461	ZK2505-8	CAOB	Inner Mongolia	118.22	46.00	granites	Diyanqinamu	156	156	7.19	19.7	0.219600	0.512838	8	0.12	3.9	3.40	3.45	3.45	0.69	-8.31	0.69	-	-	Hairui Sun et al.	2015	Ore Geology Reviews	65	70-83
4462	ZK2505-10	CAOB	Inner Mongolia	118.22	46.00	granites	Diyanqinamu	156	156	11.3	26.4	0.257600	0.512886	4	0.31	4.8	3.60	3.63	3.63	0.65	-0.92	0.65	-	-	Hairui Sun et al.	2015	Ore Geology Reviews	65	70-83
4463	BDG-1	CAOB	Ergun block	118.97	49.86	porphyry	Badaguan	229	229	4.64	25.4	0.106400	0.512534	11	-0.46	-2.0	0.60	0.61	0.61	0.88	0.88	0.95	-	-	Bingyu Gao et al.	2015	International Journal of Earth Sciences	105	507-519
4464	BDG-2	CAOB	Ergun block	118.97	49.86	porphyry	Badaguan	229	229	3.62	18.2	0.118000	0.512539	12	-0.40	-1.9	0.40	0.37	0.37	0.97	0.97	0.97	-	-	Bingyu Gao et al.	2015	International Journal of Earth Sciences	105	507-519
4465	BDG-3	CAOB	Ergun block	118.97	49.86	porphyry	Badaguan	229	229	3.78	19.1	0.104400	0.512487	15	-0.47	-2.9	0.10	-0.25	-0.25	0.92	0.92	1.02	-	-	Bingyu Gao et al.	2015	International Journal of Earth Sciences	105	507-519
4466	TPC07-1	CAOB	Ergun block	118.97	49.86	porphyry	Badaguan	229	229	3.358	17.14	0.118600	0.512361	13	-0.40	-5.4	-3.40	-3.12	-3.12	1.26	1.26	1.26	-	-	Bingyu Gao et al.	2015	International Journal of Earth Sciences	105	507-519
4467	TPC07-2	CAOB	Ergun block	118.97	49.86	porphyry	Badaguan	229	229	3.65	21.5	0.107800	0.512334	12	-0.45	-5.9	-3.60	-3.33	-3.33	1.17	1.17	1.27	-	-	Bingyu Gao et al.	2015	International Journal of Earth Sciences	105	507-519
4468	TPC07-3	CAOB	Ergun block	118.97	49.86	porphyry	Badaguan	229	229	4.07	20.04	0.122900	0.512361	14	-0.38	-5.4	-3.50	-3.25	-3.25	1.32	1.32	1.27	-	-	Bingyu Gao et al.	2015	International Journal of Earth Sciences	105	507-519
4469	ZK209-284	CAOB	Junggar	84.47	45.45	diorite	Baogutu	320	320	/	/	0.154930	0.512913	25	-0.21	5.4	7.10	7.07	7.07	0.50	0.62	0.50	-	0.53	MingJian Cao et al.	2015	Ore Geology Reviews	65	940-954
4470	BCK2-1	CAOB	Junggar	84.47	45.45	granodiorite porphyry	Baogutu	310	310	/	/	0.279100	0.513161	43	0.42	10.2	6.90	6.94	6.94	0.50	0.03	0.50	-	0.44	MingJian Cao et al.	2015	Ore Geology Reviews	65	940-954
4471	ZK213-218	CAOB	Junggar	84.47	45.45	ornblende diorite porphyrit	Baogutu	310	310	/	/	0.181180	0.512998	43	-0.08	7.0	8.00	7.64	7.64	0.45	0.71	0.45	-	0.34	MingJian Cao et al.	2015	Ore Geology Reviews	65	940-954
4472	MS02-7	CAOB	Inner Mongolia	113.70	43.35	Tonalite	Sonidzuoqi	479	479	2.67	11.85	0.144000	0.512553	/	-0.27	-1.7	1.50	1.56	1.56	1.08	1.30	1.08	-	-	Yuruo Shi et al.	2016	Journal of Asian Earth Sciences	117	153-169
4473	MB1-3	CAOB	Inner Mongolia	113.70	43.35	Granodiorite	Sonidzuoqi	472	472	2.19	15.8	0.084000	0.512402	/	-0.57	-4.6	2.20	2.18	2.18	0.88	0.88	1.02	-	-	Yuruo Shi et al.	2016	Journal of Asian Earth Sciences	117	153-169
4474	MS3-5	CAOB	Inner Mongolia	113.70	43.35	Tonalite	Sonidzuoqi	464	464	2.32	9.03	0.157000	0.512590	/	-0.20	-0.9	1.40	1.41	1.41	1.08	1.50	1.08	-	-	Yuruo Shi et al.	2016	Journal of Asian Earth Sciences	117	153-169
4475	MB1-6	CAOB	Inner Mongolia	113.70	43.35	Tonalite	Sonidzuoqi	329	329	3.63	16.2	0.137000	0.512768	/	-0.30	2.5	5.10	5.04	5.04	0.76	0.76	0.67	-	-	Yuruo Shi et al.	2016	Journal of Asian Earth Sciences	117	153-169
4476	MB1-5	CAOB	Inner Mongolia	113.70	43.35	Quartz-diorite	Sonidzuoqi	320	320	2.61	11.6	0.149000	0.512644	/	-0.24	0.1	2.10	2.06	2.06	0.91	1.19	0.91	-	-	Yuruo Shi et al.	2016	Journal of Asian Earth Sciences	117	153-169
4477	MB1-1	CAOB	Inner Mongolia	113.70	43.35	Granodiorite	Sonidzuoqi	320	320	3.09	16.2	0.115000	0.512517	/	-0.42	-2.4	1.00	0.98	0.98	0.98	0.98	1.00	-	-	Yuruo Shi et al.	2016	Journal of Asian Earth Sciences	117	153-169
4478	MB1-2	CAOB	Inner Mongolia	113.70	43.35	Granite	Sonidzuoqi	297	297	1.33	7.12	0.104000	0.512484	/	-0.47	-3.0	0.50	0.51	0.51	0.93	0.93	1.02	-	-	Yuruo Shi et al.	2016	Journal of Asian Earth Sciences	117	153-169
4479	HS-01	CAOB	Junggar	85.16	45.12	Granites	Karamay	309	309	4.68	20.3	0.139400	0.512866	3	-0.29	4.4	6.70	6.71	6.71	0.58	0.58	0.52	0.54	0.63	Jia Lu et al.	2020	Minerals	10	397-0
4480	HS-02	CAOB	Junggar	85.16	45.12	Granites	Karamay	307	307	6.81	30.9	0.133200	0.512863	2	-0.32	4.4	6.88	6.88	6.88	0.54	0.54	0.51	0.46	0.55	Jia Lu et al.	2020	Minerals	10	397-0
4481	HS-03	CAOB	Junggar	85.16	45.12	Granites	Karamay	307	307	7.38	33.9	0.131600	0.512865	4	-0.33	4.4	6.98	6.98	6.98	0.53	0.53	0.50	0.51	0.60	Jia Lu et al.	2020	Minerals	10	397-0
4482	HS-04	CAOB	Junggar	85.16	45.12	Granites	Karamay	309	309	8.36	40.6	0.124500	0.512862	2	-0.37	4.4	7.21	7.22	7.22	0.49	0.49	0.48	0.49	0.60	Jia Lu et al.	2020	Minerals	10	397-0
4483	NK-01	CAOB	Junggar	84.81	45.04	Granites	Karamay	309	309	3.35	15.4	0.131500	0.512751	2	-0.33	2.2	4.89	4.78	4.78	0.74	0.74	0.68	0.65	0.84	Jia Lu et al.	2020	Minerals	10	397-0
4484	NK-02	CAOB	Junggar	84.81	45.04	Granites	Karamay	309	309	3.27	15	0.131800	0.512748	2	-0.33	2.1	4.82	4.70	4.70	0.75	0.75	0.69	0.53	0.65	Jia Lu et al.	2020	Minerals	10	397-0
4485	NK-03	CAOB	Junggar	84.81	45.04	Granites	Karamay	309	309	3.58	16.7	0.129600	0.51275	1	-0.34	2.2	4.95	4.83	4.83	0.73	0.73	0.68	0.63	0.81	Jia Lu et al.	2020	Minerals	10	397-0
4486	NK-04	CAOB	Junggar	84.81	45.04	Granites	Karamay	309	309	3.43	15.9	0.130400	0.512461	4	-0.34	-3.5	4.86	-0.84	-0.84	1.26	1.26	1.14	0.51	0.61	Jia Lu et al.	2020	Minerals	10	397-0
4487	SMZG-01	CAOB	NE China	127.89	43.05	Granodiorite	Shimenzi	263	263	6.11	29	0.127400	0.512549	11	-0.35	-1.7	0.60	0.59	0.59	1.06	1.06	0.98	1.06	0.98	Shen Liu et al.	2010	Lithos	114	423-436
4488	SMZG-03	CAOB	NE China	127.89	43.05	Granodiorite	Shimenzi	263	263	6.09	28.2	0.130500	0.512545	11	-0.34	-1.8	0.40	0.41	0.41	1.11	1.11	1.00	1.11	1.00	Shen Liu et al.	2010	Lithos	114	423-436
4489	SMZG-07	CAOB	NE China	127.89	43.05	Granodiorite	Shimenzi	263	263	5.43	25.2	0.130300	0.512551	10	-0.34	-1.7	0.50	0.53	0.53	1.09	1.09	0.99	1.09	0.99	Shen Liu et al.	2010	Lithos	114	423-436
4490	SMZG-08	CAOB	NE China	127.89	43.05	Granodiorite	Shimenzi	263	263	6.21	29	0.129400	0.512539	16	-0.34	-1.9	0.30	0.33	0.33	1.10	1.10	1.00	1.10	1.00	Shen Liu et al.	2010	Lithos	114	423-436
4491	SMZG-12	CAOB	NE China	127.89	43.05	Granodiorite	Shimenzi	263	263	5.9	27.1	0.131600	0.512558	12	-0.33	-1.6	0.60	0.62	0.62	1.10	1.10	0.98	1.10	0.98	Shen Liu et al.	2010	Lithos	114	423-436
4492	SMZG-15	CAOB	NE China	127.89	43.05	Granodiorite	Shimenzi	263	263	5.68	26.5	0.129600	0.512558	13	-0.34	-1.6	0.70	0.69	0.69	1.07	1.07	0.97	1.07	0.97	Shen Liu et al.	2010	Lithos	114	423-436
4493	SMZG-17	CAOB	NE China	127.89	43.05	Granodiorite	Shimenzi	263	263	6.38	29.2	0.132100	0.512567	12	-0.33	-1.4	0.80	0.78	0.78	1.09	1.09	0.97	1.09	0.97	Shen Liu et al.	2010	Lithos	114	423-436
4494	SMZG-21	CAOB	NE China	127.89	43.05	Granodiorite	Shimenzi	263	263	6.6	31	0.128700	0.512564	10	-0.35	-1.4	0.80	0.84	0.84	1.05	1.05	0.96	1.05	0.96	Shen Liu et al.	2010	Lithos	114	423-436
4495	11LJ-2.2	CAOB	North China	117.88	43.75	Diorite	etaceous intracontinental gra	141	150	5.2	24.80	0.125700	0.512554	9	-0.36	-1.6	–0.40	-0.28	-0.28	1.03	1.03	0.96	-	-	Shan Li et al.	2017	GSA Bulletin	130	0-0
4496	11LJ-2.3	CAOB	North China	117.88	43.75	Diorite	etaceous intracontinental gra	141	150	5.5	26.80	0.122700	0.512546	8	-0.38	-1.8	–0.50	-0.38	-0.38	1.01	1.01	0.97	-	-	Shan Li et al.	2017	GSA Bulletin	130	0-0
4497	14XF-4	CAOB	North China	118.93	43.78	Granodiorite	etaceous intracontinental gra	141	150	6.1	32.40	0.114500	0.512476	8	-0.42	-3.2	–1.70	-1.59	-1.59	1.04	1.04	1.07	-	-	Shan Li et al.	2017	GSA Bulletin	130	0-0
4498	14XF-5	CAOB	North China	118.93	43.78	Granodiorite	etaceous intracontinental gra	141	150	6	31.00	0.117700	0.512500	6	-0.40	-2.7	–1.30	-1.18	-1.18	1.03	1.03	1.03	-	-	Shan Li et al.	2017	GSA Bulletin	130	0-0
4499	14XF-8	CAOB	North China	118.93	43.78	Granodiorite	etaceous intracontinental gra	141	150	5.7	29.20	0.117400	0.512637	7	-0.40	0.0	1.40	1.50	1.50	0.81	0.81	0.82	-	-	Shan Li et al.	2017	GSA Bulletin	130	0-0
4500	14RS-2	CAOB	North China	117.59	43.52	Monzogranite	etaceous intracontinental gra	138	150	10.7	51.70	0.125300	0.512492	8	-0.36	-2.8	–1.60	-1.48	-1.48	1.13	1.13	1.06	-	-	Shan Li et al.	2017	GSA Bulletin	130	0-0
4501	14RS-3	CAOB	North China	117.59	43.52	Monzogranite	etaceous intracontinental gra	138	150	11.7	54.80	0.128700	0.512468	6	-0.35	-3.3	–2.10	-2.01	-2.01	1.22	1.22	1.10	-	-	Shan Li et al.	2017	GSA Bulletin	130	0-0
4502	14RS-4	CAOB	North China	117.65	43.46	Monzogranite	etaceous intracontinental gra	138	150	3.6	19.40	0.112100	0.512303	7	-0.43	-6.5	–5.00	-4.92	-4.92	1.27	1.27	1.34	-	-	Shan Li et al.	2017	GSA Bulletin	130	0-0
4503	14RS-7	CAOB	North China	117.65	43.46	Monzogranite	etaceous intracontinental gra	138	150	7.4	42.50	0.104600	0.512350	6	-0.47	-5.6	–4.00	-3.86	-3.86	1.12	1.12	1.25	-	-	Shan Li et al.	2017	GSA Bulletin	130	0-0
4504	14RS-8	CAOB	North China	117.72	43.44	Monzogranite	etaceous intracontinental gra	138	150	5.1	21.50	0.142900	0.512399	9	-0.27	-4.7	–3.70	-3.63	-3.63	1.23	1.61	1.23	-	-	Shan Li et al.	2017	GSA Bulletin	130	0-0

4505	14RS-10	CAOB	North China	117.72	43.44	Syenogranite	retaceous intracontinental gra	138	150	4.4	24.30	0.108300	0.512390	5	-0.45	-4.8	–3.30	-3.15	-3.15	1.10	1.10	1.20	-	-	Shan Li et al.	2017	GSA Bulletin	130	0-0
4506	14XC-2	CAOB	North China	118.12	43.33	Syenogranite	retaceous intracontinental gra	129	150	4.3	22.10	0.118400	0.512254	7	-0.40	-7.5	–6.20	-5.99	-5.99	1.43	1.43	1.43	-	-	Shan Li et al.	2017	GSA Bulletin	130	0-0
4507	14HS-3	CAOB	North China	118.20	43.91	Granite porphyry	retaceous intracontinental gra	133	150	6	32.70	0.111000	0.512549	7	-0.44	-1.7	–0.30	-0.10	-0.10	0.89	0.89	0.95	-	-	Shan Li et al.	2017	GSA Bulletin	130	0-0
4508	14HS-5	CAOB	North China	118.20	43.91	Granite porphyry	retaceous intracontinental gra	133	150	5	27.20	0.111500	0.512484	5	-0.43	-3.0	-1.60	-1.37	-1.37	0.99	0.99	1.05	-	-	Shan Li et al.	2017	GSA Bulletin	130	0-0
4509	14WL-4	CAOB	North China	118.69	43.72	Granite porphyry	retaceous intracontinental gra	129	150	4.5	25.50	0.107200	0.512422	6	-0.46	-4.2	–2.70	-2.50	-2.50	1.04	1.04	1.14	-	-	Shan Li et al.	2017	GSA Bulletin	130	0-0
4510	MDLH-1	CAOB	Altai	88.51	48.28	granites	Mandalehai	327	327	-	-	0.128200	0.512383	8	-0.35	-5.0	− 1.40	-2.12	-2.12	1.37	1.37	1.25	1.37	1.14	QUN LUO et al.	2019	Geological Magazine	156	585-604
4511	MDLH-2	CAOB	Altai	88.51	48.28	granites	Mandalehai	327	327	-	-	0.130500	0.512376	5	-0.34	-5.1	− 1.70	-2.35	-2.35	1.42	1.42	1.27	1.42	1.15	QUN LUO et al.	2019	Geological Magazine	156	585-604
4512	MDLH-3	CAOB	Altai	88.51	48.28	granites	Mandalehai	327	327	-	-	0.129400	0.512384	4	-0.34	-5.0	− 1.40	-2.15	-2.15	1.38	1.38	1.26	1.38	1.14	QUN LUO et al.	2019	Geological Magazine	156	585-604
4513	MDLH-4	CAOB	Altai	88.51	48.28	granites	Mandalehai	327	327	-	-	0.131800	0.512388	4	-0.33	-4.9	− 1.50	-2.17	-2.17	1.42	1.42	1.26	1.42	1.14	QUN LUO et al.	2019	Geological Magazine	156	585-604
4514	HT-1	CAOB	Altai	88.69	48.15	granites	Huiteng	327	327	-	-	0.140500	0.512389	7	-0.29	-4.9	− 2.50	-2.51	-2.51	1.29	1.58	1.29	1.58	1.14	QUN LUO et al.	2019	Geological Magazine	156	585-604
4515	HT-2	CAOB	Altai	88.69	48.15	granites	Huiteng	327	327	-	-	0.140000	0.512394	8	-0.29	-4.8	− 2.40	-2.39	-2.39	1.56	1.56	1.28	1.56	1.14	QUN LUO et al.	2019	Geological Magazine	156	585-604
4516	HT-3	CAOB	Altai	88.69	48.15	granites	Huiteng	327	327	-	-	0.141800	0.512381	9	-0.28	-5.0	− 2.70	-2.72	-2.72	1.30	1.63	1.30	1.63	1.14	QUN LUO et al.	2019	Geological Magazine	156	585-604
4517	HT-4	CAOB	Altai	88.69	48.15	granites	Huiteng	327	327	-	-	0.141300	0.51239	5	-0.28	-4.8	− 2.50	-2.53	-2.53	1.29	1.60	1.29	1.60	1.15	QUN LUO et al.	2019	Geological Magazine	156	585-604
4518	TEG-1	CAOB	Altai	87.72	48.60	granites	Tuergen	327	327	-	-	0.134700	0.512409	7	-0.32	-4.5	− 1.30	-1.88	-1.88	1.43	1.43	1.24	1.43	1.12	QUN LUO et al.	2019	Geological Magazine	156	585-604
4519	TEG-2	CAOB	Altai	87.72	48.60	granites	Tuergen	327	327	-	-	0.132200	0.512414	8	-0.33	-4.4	− 1.10	-1.68	-1.68	1.37	1.37	1.22	1.37	1.11	QUN LUO et al.	2019	Geological Magazine	156	585-604
4520	TEG-3	CAOB	Altai	87.72	48.60	granites	Tuergen	327	327	-	-	0.136000	0.512403	7	-0.31	-4.6	− 1.50	-2.05	-2.05	1.46	1.46	1.25	1.46	1.16	QUN LUO et al.	2019	Geological Magazine	156	585-604
4521	TEG-4	CAOB	Altai	87.72	48.60	granites	Tuergen	327	327	-	-	0.133500	0.51241	4	-0.32	-4.4	− 1.20	-1.81	-1.81	1.40	1.40	1.23	1.40	1.12	QUN LUO et al.	2019	Geological Magazine	156	585-604
4522	ZGST-1	CAOB	Altai	87.74	48.63	granites	Zhagesitai	327	327	-	-	0.142700	0.512322	8	-0.27	-6.2	− 3.20	-3.91	-3.91	1.40	1.77	1.40	1.77	1.28	QUN LUO et al.	2019	Geological Magazine	156	585-604
4523	ZGST-2	CAOB	Altai	87.74	48.63	granites	Zhagesitai	327	327	-	-	0.143200	0.512317	8	-0.27	-6.3	− 3.30	-4.03	-4.03	1.41	1.80	1.41	1.80	1.29	QUN LUO et al.	2019	Geological Magazine	156	585-604
4524	ZGST-3	CAOB	Altai	87.74	48.63	granites	Zhagesitai	327	327	-	-	0.143900	0.512323	5	-0.27	-6.1	− 3.20	-3.94	-3.94	1.40	1.80	1.40	1.80	1.28	QUN LUO et al.	2019	Geological Magazine	156	585-604
4525	ZGST-4	CAOB	Altai	87.74	48.63	granites	Zhagesitai	327	327	-	-	0.144500	0.512325	7	-0.27	-6.1	− 3.20	-3.93	-3.93	1.40	1.81	1.40	1.81	1.28	QUN LUO et al.	2019	Geological Magazine	156	585-604
4526	ZPJLK-1	CAOB	Altai	87.56	48.43	granites	Zhepujilieke	327	327	-	-	0.122100	0.51237	4	-0.38	-5.2	− 0.90	-2.12	-2.12	1.30	1.30	1.26	1.30	1.14	QUN LUO et al.	2019	Geological Magazine	156	585-604
4527	ZPJLK-2	CAOB	Altai	87.56	48.43	granites	Zhepujilieke	327	327	-	-	0.134200	0.512376	7	-0.32	-5.1	− 1.50	-2.50	-2.50	1.48	1.48	1.29	1.48	1.18	QUN LUO et al.	2019	Geological Magazine	156	585-604
4528	ZPJLK-3	CAOB	Altai	87.56	48.43	granites	Zhepujilieke	327	327	-	-	0.123300	0.512364	8	-0.37	-5.3	− 1.10	-2.28	-2.28	1.32	1.32	1.27	1.32	1.15	QUN LUO et al.	2019	Geological Magazine	156	585-604
4529	ZPJLK-4	CAOB	Altai	87.56	48.43	granites	Zhepujilieke	327	327	-	-	0.135400	0.512371	9	-0.31	-5.2	− 1.70	-2.65	-2.65	1.51	1.51	1.30	1.51	1.19	QUN LUO et al.	2019	Geological Magazine	156	585-604
4530	J11-5-2	CAOB	Junggar Basin	87.96	45.29	rhyolite	northern Junggar Basin	325	325	4.26	19.6	0.131403	0.512750	6	-0.33	2.2	4.90	4.89	4.89	0.74	0.74	0.68	-	-	Di Li et al.	2015	Gondwana Research	27	1110-1130
4531	J11-5-2R	CAOB	Junggar Basin	87.96	45.29	Dacite	northern Junggar Basin	325	325	4.26	19.6	0.131402	0.512742	6	-0.33	2.0	4.80	4.74	4.74	0.70	0.76	0.70	-	-	Di Li et al.	2015	Gondwana Research	27	1110-1130
4532	ALH-07	CAOB	NE China	121.88	44.53	Adakitic	Aolunhua	160	160	2.51	13.7	0.110758	0.51247	2	-0.44	-3.3	−1.88	-1.52	-1.52	1.07	1.01	1.07	-	1.07	Xiaohu He et al.	2020	Minerals	10	332-0
4533	ALH-12	CAOB	NE China	121.88	44.53	Adakitic	Aolunhua	160	160	2.62	15.3	0.103522	0.512492	2	-0.47	-2.8	−1.33	-0.95	-0.95	1.02	0.91	1.02	-	1.02	Xiaohu He et al.	2020	Minerals	10	332-0
4534	ALH-17	CAOB	NE China	121.88	44.53	Adakitic	Aolunhua	160	160	2.87	16.9	0.102664	0.512491	3	-0.48	-2.9	−1.33	-0.95	-0.95	1.02	0.90	1.02	-	1.02	Xiaohu He et al.	2020	Minerals	10	332-0
4535	ALH-20	CAOB	NE China	121.88	44.53	Adakitic	Aolunhua	160	160	1.95	11.1	0.106201	0.51244	2	-0.46	-3.9	−2.39	-2.01	-2.01	1.11	1.01	1.11	-	1.11	Xiaohu He et al.	2020	Minerals	10	332-0
4536	ALH-21	CAOB	NE China	121.88	44.53	Adakitic	Aolunhua	160	160	2.81	14.7	0.115559	0.512418	5	-0.41	-4.3	−2.98	-2.63	-2.63	1.16	1.14	1.16	-	1.16	Xiaohu He et al.	2020	Minerals	10	332-0
4537	ALH-22	CAOB	NE China	121.88	44.53	Adakitic	Aolunhua	160	160	2.91	17.2	0.102278	0.51244	3	-0.48	-3.9	−2.32	-1.93	-1.93	1.10	0.97	1.10	-	1.10	Xiaohu He et al.	2020	Minerals	10	332-0
4538	ALW-07	CAOB	NE China	121.88	44.53	Adakitic	Aolunhua	160	160	3.69	20.5	0.108820	0.512612	2	-0.45	-0.5	0.92	1.29	1.29	0.84	0.78	0.84	-	0.84	Xiaohu He et al.	2020	Minerals	10	332-0
4539	ALW-14	CAOB	NE China	121.88	44.53	Adakitic	Aolunhua	160	160	2.86	15.3	0.113004	0.512442	2	-0.43	-3.8	−2.46	-2.11	-2.11	1.12	1.07	1.12	-	1.12	Xiaohu He et al.	2020	Minerals	10	332-0
4540	KN01	CAOB	the Chinese Altai	87.13	48.75	granitic	Hanasi	398	398	-	-	0.133300	0.512445	5	-0.32	-3.8	-0.60	-0.54	-0.54	/	1.33	1.19	-	-	Yang Yu et al.	2017	Gondwana Research	47	131-141
4541	KN02	CAOB	the Chinese Altai	87.13	48.75	granitic	Hanasi	398	398	-	-	0.132800	0.512454	5	-0.32	-3.6	-0.40	-0.34	-0.34	/	1.31	1.17	-	-	Yang Yu et al.	2017	Gondwana Research	47	131-141
4542	KN04	CAOB	the Chinese Altai	87.13	48.75	granitic	Hanasi	398	398	-	-	0.132100	0.512437	5	-0.33	-3.9	-0.70	-0.64	-0.64	/	1.33	1.19	-	-	Yang Yu et al.	2017	Gondwana Research	47	131-141
4543	ALT66	CAOB	the Chinese Altai	87.90	47.97	granitic	Kurmutu	421	421	-	-	0.134400	0.512518	9	-0.32	-2.3	0.80	1.01	1.01	/	1.21	1.08	-	-	Yang Yu et al.	2017	Gondwana Research	47	131-141
4544	ALT67	CAOB	the Chinese Altai	87.90	47.97	granitic	Kurmutu	421	421	-	-	0.149100	0.512538	13	-0.24	-2.0	0.40	0.61	0.61	/	1.44	1.11	-	-	Yang Yu et al.	2017	Gondwana Research	47	131-141
4545	ALT03	CAOB	the Chinese Altai	88.10	47.72	granitic	Altay	368	368	-	-	0.139800	0.512505	8	-0.29	-2.6	-0.30	0.08	0.08	/	1.33	1.11	-	-	Yang Yu et al.	2017	Gondwana Research	47	131-141
4546	ALT04	CAOB	the Chinese Altai	88.10	47.72	granitic	Altay	368	368	-	-	0.159300	0.512533	10	-0.19	-2.0	-0.20	-0.29	-0.29	/	1.72	1.14	-	-	Yang Yu et al.	2017	Gondwana Research	47	131-141
4547	ALT15	CAOB	the Chinese Altai	88.35	47.55	granitic	Kergar	411	411	-	-	0.173600	0.512526	9	-0.12	-2.2	-1.00	-0.97	-0.97	/	2.36	1.23	-	-	Yang Yu et al.	2017	Gondwana Research	47	131-141
4548	ALT16	CAOB	the Chinese Altai	88.35	47.55	granitic	Kergar	411	411	-	-	0.158100	0.512509	9	-0.20	-2.5	-0.60	-0.49	-0.49	/	1.75	1.19	-	-	Yang Yu et al.	2017	Gondwana Research	47	131-141
4549	ALT17	CAOB	the Chinese Altai	88.35	47.55	granitic	Kergar	411	411	-	-	0.178200	0.512481	5	-0.09	-3.1	-2.20	-2.09	-2.09	/	2.85	1.32	-	-	Yang Yu et al.	2017	Gondwana Research	47	131-141
4550	HM02	CAOB	the Chinese Altai	87.59	48.56	granitic	Hemu	412	412	-	-	0.142400	0.512344	10	-0.28	-5.7	-3.00	-2.88	-2.88	/	1.72	1.39	-	-	Yang Yu et al.	2017	Gondwana Research	47	131-141
4551	HM03	CAOB	the Chinese Altai	87.59	48.56	granitic	Hemu	412	412	-	-	0.138200	0.512342	9	-0.30	-5.8	-2.90	-2.70	-2.70	/	1.63	1.37	-	-	Yang Yu et al.	2017	Gondwana Research	47	131-141
4552	HM04	CAOB	the Chinese Altai	87.59	48.56	granitic	Hemu	412	412	-	-	0.139300	0.512341	9	-0.29	-5.8	-2.90	-2.78	-2.78	/	1.65	1.38	-	-	Yang Yu et al.	2017	Gondwana Research	47	131-141
4553	HM12	CAOB	the Chinese Altai	87.59	48.56	granitic	Hemu	412	412	-	-	0.134700	0.512373	10	-0.32	-5.2	-2.10	-1.91	-1.91	/	1.50	1.31	-	-	Yang Yu et al.	2017	Gondwana Research	47	131-141
4554	KW14	CAOB	the Chinese Altai	89.36	47.24	granitic	Kuwei	419	419	-	-	0.118500	0.512356	8	-0.40	-5.5	-1.60	-1.32	-1.32	/	1.27	1.27	-	-	Yang Yu et al.	2017	Gondwana Research	47	131-141
4555	KW15	CAOB	the Chinese Altai	89.36	47.24	granitic	Kuwei	419	419	-	-	0.112000	0.512509	7	-0.43	-2.5	1.70	2.01	2.01	/	0.96	0.99	-	-	Yang Yu et al.	2017	Gondwana Research	47	131-141
4556	KW16	CAOB	the Chinese Altai	89.36	47.24	granitic	Kuwei	419	419	-	-	0.120500	0.51235	7	-0.39	-5.6	-1.80	-1.54	-1.54	/	1.31	1.28	-	-	Yang Yu et al.	2017	Gondwana Research	47	131-141
4557	JDY13	CAOB	the Chinese Altai	87.20	48.51	granitic	Jiadengyu	445	445	-	-	0.118100	0.512403	8	-0.40	-4.6	-0.70	-0.12	-0.12	/	1.19	1.19	-	-	Yang Yu et al.	2017	Gondwana Research	47	131-141
4558	JDY13	CAOB	the Chinese Altai	87.20	48.51	granitic	Jiadengyu	445	445	-	-	0.132400	0.51239	8	-0.33	-4.8	-1.60	-1.19	-1.19	/	1.42	1.28	-	-	Yang Yu et al.	2017	Gondwana Research	47	131-141
4559	JDY13	CAOB	the Chinese Altai	87.20	48.51	granitic	Jiadengyu	445	445	-	-	0.128200	0.512405	8	-0.35	-4.5	-1.10	-0.66	-0.66	/	1.33	1.23	-	-	Yang Yu et al.	2017	Gondwana Research	47	131-141
4560	H19HX8	CAOB	China	118.6361111	44.74944444	Pyroxene diorite		281	281	7.01	26.2	0.1618	0.512762	6	-0.18	2.4	3.7	3.67	3.67	1.14	0.75	0.75	1.14	0.68	Zhang XF et al.	2021	Journal of Asian Earth Sciences	5	100059
4561	H19HX9	CAOB	China	118.6361111	44.74944444	Pyroxene diorite		281	281	7.48	28	0.1615	0.512774	4	-0.18	2.7	3.9	3.91	3.91	1.10	0.73	0.73	1.11	0.66	Zhang XF et al.	2021	Journal of Asian Earth Sciences	5	100059
4562	H19HX11	CAOB	China	118.6361111	44.74944444	Pyroxene diorite		281	281	5.85	20.9	0.1692	0.512776	5	-0.14	2.7	3.7	3.68	3.68	1.28	0.74	0.74	1.28	0.68	Zhang XF et al.	2021	Journal of Asian Earth Sciences	5	100059
4563	H19HX12	CAOB	China	118.6361111	44.74944444	Alkali feldspar granite		281	281	1.47	6.93	0.1282	0.512708	6	-0.35	1.4	3.8	3.82	3.82	0.79	0.73	0.79	0.79	0.67	Zhang XF et al.	2021	Journal of Asian Earth Sciences	5	100059
4564	H19HX13	CAOB	China	118.6361111	44.74944444	Alkali feldspar granite		281	281	1.65	6.54	0.1525	0.512739	11	-0.22	2.0	3.6	3.55	3.55	1.02	0.76	0.76	1.02	0.69	Zhang XF et al.	2021	Journal of Asian Earth Sciences	5	100059
4565	H19HX15	CAOB	China	118.6361111	44.74944444	Alkali feldspar granite		281	281	13	60.7	0.1295	0.512695	4	-0.34	1.1	3.5	3.52	3.52	0.82	0.76	0.82	0.82	0.69	Zhang XF et al.	2021	Journal of Asian Earth Sciences	5	100059
4566	SMZG-01	CAOB	NE China	127.9166667	43.08333333	Granodiorite		262.8	262.8	6.11	29	0.1274	0.512549	8	-0.35	-1.7	0.6	0.59	0.59	1.06	0.98	1.06	1.06	0.98	Liu S et al.	2010	Lithos	114(3-4)	423-436
4567	SMZG-03	CAOB	NE China	127.9166667	43.08333333	Granodiorite		262.8	262.8	6.09	28.2	0.1305	0.512545	12	-0.34	-1.8	0.4	0.41	0.41	1.11	1.00	1.11	1.11	1	Liu S et al.	2010	Lithos	114(3-4)	423-436
4568	SMZG-07	CAOB	NE China	127.9166667	43.08333333	Granodiorite		262.8	262.8	5.43	25.2	0.1303	0.512551	6	-0.34	-1.7	0.5	0.53	0.53	1.09	0.99	1.09	1.09	0.99	Liu S et al.	2010	Lithos	114(3-4)	423-436
4569	SMZG-08	CAOB	NE China	127.9166667	43.08333333	Granodiorite		262.8	262.8	6.21	29	0.1294	0.512539	10	-0.34	-1.9	0.3	0.33	0.33	1.10	1.00	1.10	1.1	1	Liu S et al.	2010	Lithos	114(3-4)	423-436
4570	SMZG-12	CAOB	NE China	127.9166667	43.08333333	Granodiorite		262.8	262.8	5.9	27.1	0.1316	0.512558	9	-0.33	-1.6	0.6	0.62	0.62	1.10	0.98	1.10	1.1	0.98	Liu S et al.	2010	Lithos	114(3-4)	423-436
4571	SMZG-15	CAOB	NE China	127.9166667	43.08333333	Granodiorite		262.8	262.8	5.68	26.5	0.1296	0.512558	10	-0.34	-1.6	0.7	0.69	0.69	1.07	0.97	1.07	1.07	0.97	Liu S et al.	2010	Lithos	114(3-4)	423-436
4572	SMZG-17	CAOB	NE China	127.9166667	43.08333333	Granodiorite		262.8	262.8	6.38	29.2	0.1321	0.512567	12	-0.33	-1.4	0.8	0.78	0.78	1.09	0.97	1.09	1.09	0.97	Liu S et al.	2010	Lithos	114(3-4)	423-436
4573	SMZG-21	CAOB	NE China	127.9166667	43.08333333	Granodiorite		262.8	262.8	6.6	31	0.1287	0.512564	9	-0.35	-1.4	0.8	0.84	0.84	1.05	0.96	1.05	1.05	0.96	Liu S et al.	2010	Lithos	114(3-4)	423-436
4574	G-28/1	CAOB	Russian	85.16666667	59.2	quartz syenite	Aya	250	250	3.9	23.1	0.1014	0.512588	12	-0.48	-1.0	2.1	2.06	2.06	0.76	0.85	0.76	765	867	Wang YJ et al.	2016	Lithos	266–267	264-286
4575	2-756a*	CAOB	Russian	85.16666667	59.2	syenite	Aya	250	250	31.1	162.5	0.1155	0.512617	7	-0.41	-0.4	2.2	2.18	2.18	0.83	0.84	0.83	830	858	Wang YJ et al.	2016	Lithos	266–267	264-286
4576	2-755a*	CAOB	Russian	85.16666667	59.2	syenogranite	Aya	250	250	3.9	25.8	0.09047	0.512618	8	-0.54	-0.4	3	3.00	3.00	0.66	0.78	0.66	660	790	Wang YJ et al.	2016	Lithos	266–267	264-286
4577	2-742a	CAOB	Russian	85.16666667	59.2	granite	Aya	250	250	2.9	19.5	0.09	0.512596	8	-0.54	-0.8	2.6	2.58	2.58	0.68	0.81	0.68	685	824	Wang YJ et al.	2016	Lithos	266–267	264-286
4578	2-748a*	CAOB	Russian	85.16666667	59.2	leucogranite	Aya	250	250	6.2	40.8	0.0922	0.512619	7	-0.53	-0.4	3	2.96	2.96	0.67	0.78	0.67	669	793	Wang YJ et al.	2016	Lithos	266–267	264-286
4579	2-745a*	CAOB	Russian	85.16666667	59.2	leucogranite	Aya	250	250	1.9	14.4	0.0792	0.5126	8	-0.60	-0.7	3	3.01	3.01	0.62	0.78	0.62	626	789	Wang YJ et al.	2016	Lithos	266–267	264-286
4580	8-07-19	CAOB	Russian	88.58333333	47.6	monzogranodiorite	Tarkhata	254	254	13.5	86.9	0.0936	0.512342	5	-0.52	-5.8	-2.4	-2.43	-2.43	1.03	1.22	1.03	1027	1245	Wang YJ et al.	2016	Lithos	266–267	264-286
4581	8-07-22	CAOB	Russian	88.58333333	47.6	syenite	Tarkhata	254	254	11.3	72.6	0.0938	0.512361	9	-0.52	-5.4	-2.1	-2.07	-2.07	1.00	1.19	1.00	1005	1214	Wang YJ et al.	2016	Lithos	266–267	264-286
4582	5-451	CAOB	Russian	88.58333333	47.6	syenogranite	Tarkhata	254	254	7.2	47	0.0924	0.512287	8	-0.53	-6.8	-3.5	-3.47	-3.47	1.08	1.31	1.08	1086	1331	Wang YJ et al.	2016	Lithos	266–267	264-286
4583	8002	CAOB	Russian	88.58333333	47.6	syenogranite	Tarkhata	254	254	9.7	63.1	0.0926	0.512332	2	-0.53	-6.0	-2.6	-2.60	-2.60	1.03	1.24	1.03	1031	1258	Wang YJ et al.	2016	Lithos	266–267	264-286
4584	A-041**	CAOB	Russian	87.96666667	53.21666667	monzodiorite	Terandzhik	248	248	8	54.2	0.0887	0.512606	3	-0.55	-0.6	2.8	2.79	2.79	0.66	0.79	0.66	666	805	Wang YJ et al.	2016	Lithos	266–267	264-286
4585	09-103/2**	CAOB	Russian	87.96666667	53.21666667	syenogranite	Terandzhik	248	248	6.4	43.6	0.0889	0.512631	4	-0.55	-0.1	3.3	3.27	3.27	0.63	0.75	0.63	636	765	Wang YJ et al.	2016	Lithos	266–267	264-286
4586	13-51/4	CAOB	Russian	82.31666667	57.4	monzodiorite	Savvushka	244	244	19.4	100.7	0.1162	0.512696	18	-0.41	1.1	3.6	3.64	3.64	0.71	0.72	0.71	712	731	Wang YJ et al.	2016	Lithos	266–267	264-286
4587	13-54/2	CAOB	Russian	82.31666667	57.4	quartz diorite	Savvushka	244	244	18.1	91.2	0.1203	0.512675	7	-0.39	0.7	3.1	3.10	3.10	0.78	0.76	0.78	778	776	Wang YJ et al.	2016	Lithos	266–267	264-286
4588	13-55/2	CAOB	Russian	82.31666667	57.4	granodiorite	Savvushka	244	244	18.0	92.5	0.1174	0.512662	14	-0.40	0.5	2.9	2.94	2.94	0.77	0.78	0.77	775	790	Wang YJ et al.	2016	Lithos	266–267	264-286
4589	8-376	CAOB	Russian	82.31666667	57.4	granite	Savvushka	244	244	3.4	20.7	0.0992	0.512591	7	-0.50	-0.9	2.1	2.12	2.12	0.74	0.84	0.74	747	858	Wang YJ et al.	2016	Lithos	266–267	264-286
4590	13-52/2	CAOB	Russian	82.31666667	57.4	leucogranite	Savvushka	244	244	0.8	7	0.0712	0.512576	13	-0.64	-1.2	2.7	2.70	2.70	0.61	0.80	0.80	616	810	Wang YJ et al.	2016	Lithos	266–267	264-286
4591	L30-1	CAOB	southern China	106.15	40.56666667	Syeno granite		279.5	279.5	11.3	68.6	0.0995	0.511918		-0.49	-14.0	-10.8	-10.58	-10.58	1.64	1.90	1.64	1.644	1.91	Zheng RG et al.	2019	Lithos	348–349	105174
4592	Rg50-2	CAOB	southern China	106.3666667	40.65	monzogranodiorite		260.2	260.2	12.4	68.6	0.1092	0.511816		-0.44	-16.0	-13.1	-13.13	-13.13	1.94	2.09	1.94	1.943	2.096	Zheng RG et al.	2019	Lithos	348–349	105174
4593	LZ04-4	CAOB	southern China	106.2	40.58333333	monzogranodiorite		281.4	281.4	7.36	38.2	0.1164	0.511899		-0.41	-14.4	-11.5	-11.53	-11.53	1.95	1.98	1.95	1.957	1.984	Zheng RG et al.	2019	Lithos	348–349	105174
4594	LZ06-1	CAOB	southern China	106.25	40.61666667	Syeno granite		269.8	269.8	6.94	42	0.0998	0.511907		-0.49	-14.3	-10.9	-10.92	-10.92	1.66	1.92	1.66	1.663	1.926	Zheng RG et al.	2019	Lithos	348–349	105174
4595	LZ07-1	CAOB	southern China	106.25	40.6	Quartz diorite		269.8	269.8	7.64	36.6	0.1261	0.511879		-0.36	-14.8	-12.4	-12.38	-12.38	2.20	2.04	2.20	2.208	2.044	Zheng RG et al.	2019	Lithos	348–349	105174
4596	LZ08-2	CAOB	southern China	106.2833333	40.61666667	Quartz monzodiorite		270.5	270.5	10.3	56.9	0.1094	0.511885		-0.44	-14.7	11.7	-11.68	-11.68	1.84	1.99	1.84	1.846	1.987	Zheng RG et al.	2019	Lithos	348–349	105174
4597	H2-14	CAOB	central Kazakhstan	76.28991667	46.84331667	diorite		470	470	0.51	1.53	0.2	0.51304		0.02	7.8	7.7	7.64	7.64	1.22	0.58	0.58			Safonova I et al.	2020	Gondwana Research	79	49-69
4598	JD-6/1	CAOB	central Kazakhstan	76.05075	46.95036667	diorite		500	500	17.36	17.36	0.1309	0.51263		-0.33	-0.2	4	4.04	4.04	0.96	0.90	0.96			Safonova I et al.	2020	Gondwana Research	79	49-69
4599	18KY011	CAOB	southeastern CAOB	124.26	48.02033058	Granite		242.1	242.1	1.63	10.5	0.093453	0.512218		-0.52	-8.2	-5	-5.00	-5.00	1.18	1.42	1.18	-1.181	-1.422	Jing Y et al.	2022	Gondwana Research	103	146-171
4600	18KY015	CAOB	southeastern CAOB	124.2083333	48.29316667	Granite		243.1	243.1	2.43	14.4	0.102076	0.512263		-0.48	-7.3	-4.38	-4.38	-4.38	1.21	1.37	1.21	-1.21	-1.372	Jing Y et al.	2022	Gondwana Research	103	146-171
4601	18KY029	CAOB	southeastern CAOB	124.3716667	46.22833333	Granite		242.6	242.6	1.82	12.1	0.091356	0.512186		-0.54	-8.8	-5.56	-5.56	-5.56	1.20	1.47	1.20	-1.2	-1.467	Jing Y et al.	2022	Gondwana Research	103	146-171
4602	18KY030	CAOB	southeastern CAOB	124.3716667	46.22833333	Granite		242.6	242.6	1.77	11.3	0.094856	0.512185		-0.52	-8.8	-5.69	-5.68	-5.68	1.24	1.48	1.24	-1.237	-1.477	Jing Y et al.	2022	Gondwana Research	103	146-171

4603	18KY031	CAOB	southeastern CAOB	124.3716667	46.22833333	Granite		242.6	242.6	1.69	11.1	0.092168	0.512181		-0.53	-8.9	-5.68	-5.68	-5.68	1.21	1.48	1.21	-1.214	-1.477	Jing Y et al.	2022	Gondwana Research	103	146-171
4604	18KY032	CAOB	southeastern CAOB	124.3716667	46.22833333	Granite		242.6	242.6	1.69	11.3	0.090194	0.512185		-0.54	-8.8	-5.54	-5.54	-5.54	1.19	1.47	1.19	-1.19	-1.466	Jing Y et al.	2022	Gondwana Research	103	146-171
4605	18KY008	CAOB	southeastern CAOB	124.1716667	47.54016667	Granite		243.1	243.1	2.33	15.1	0.093023	0.512242		-0.53	-7.7	-4.51	-4.51	-4.51	1.15	1.38	1.15	-1.146	-1.382	Jing Y et al.	2022	Gondwana Research	103	146-171
4606	D301-YQ1	CAOB	NE China	123.66	42.57616667	high-Mg andesite		369	369	6.4	29.52	0.171857	0.512711		-0.13	1.4	2.6	2.59	2.59	1.60	0.90	0.90	1600	907	Shi Y et al.	2022	Gondwana Research	103	64-83
4607	D301-YQ2	CAOB	NE China	123.66	42.57616667	high-Mg andesite		369	369	6.18	28.85	0.118374	0.512734		-0.40	1.9	5.57	5.56	5.56	0.67	0.66	0.67	667	666	Shi Y et al.	2022	Gondwana Research	103	64-83
4608	D301-YQ3	CAOB	NE China	123.66	42.57616667	high-Mg andesite		369	369	6.03	30.25	0.120532	0.512713		-0.39	1.5	5.06	5.05	5.05	0.72	0.71	0.72	718	709	Shi Y et al.	2022	Gondwana Research	103	64-83
4609	BJGCQY1	CAOB	NE China	123.63	42.555	granite		248	248	2.88	18.15	0.096063	0.512503		-0.51	-2.6	0.57	0.55	0.55	0.84	0.97	0.84	840	976	Shi Y et al.	2022	Gondwana Research	103	64-83
4610	BJGCQY2	CAOB	NE China	123.63	42.555	granite		248	248	3.73	23.27	0.096906	0.512497		-0.51	-2.8	0.42	0.41	0.41	0.85	0.99	0.85	855	989	Shi Y et al.	2022	Gondwana Research	103	64-83
4611	BJGCQY3	CAOB	NE China	123.63	42.555	granite		248	248	3.99	25.03	0.096324	0.512529		-0.51	-2.1	1.06	1.05	1.05	0.81	0.93	0.81	808	935	Shi Y et al.	2022	Gondwana Research	103	64-83
4612	BJGCQY5	CAOB	NE China	123.63	42.555	granite		248	248	3.87	25.52	0.09159	0.512553		-0.53	-1.7	1.66	1.67	1.67	0.75	0.88	0.75	748	886	Shi Y et al.	2022	Gondwana Research	103	64-83
4613	G-28/1	CAOB	Russian	84.83333333	50.85	quartz syenite		250	250	3.9	23.1	0.1014	0.512588		-0.48	-1.0	2.1	2.06	2.06	0.76	0.85	0.76	0.765	0.867	Gavryushkina OA et al.	2019	Lithos	326–327	71-89
4614	2-756a	CAOB	Russian	84.83333333	50.85	syenite		250	250	31.1	162.5	0.1155	0.512617		-0.41	-0.4	2.2	2.18	2.18	0.83	0.84	0.83	0.83	0.858	Gavryushkina OA et al.	2019	Lithos	326–327	71-89
4615	2-755a	CAOB	Russian	84.83333333	50.85	syenogranite		250	250	3.9	25.8	0.09047	0.512618		-0.54	-0.4	3	3.00	3.00	0.66	0.78	0.66	0.66	0.79	Gavryushkina OA et al.	2019	Lithos	326–327	71-89
4616	2-742a	CAOB	Russian	84.83333333	50.85	granite		250	250	2.9	19.5	0.09	0.512596		-0.54	-0.8	2.6	2.58	2.58	0.68	0.81	0.68	0.685	0.824	Gavryushkina OA et al.	2019	Lithos	326–327	71-89
4617	2-748a	CAOB	Russian	84.83333333	50.85	leucogranite		250	250	6.2	40.8	0.0922	0.512619		-0.53	-0.4	3	2.96	2.96	0.67	0.78	0.67	0.669	0.793	Gavryushkina OA et al.	2019	Lithos	326–327	71-89
4618	2-745a	CAOB	Russian	84.83333333	50.85	leucogranite		250	250	1.9	14.4	0.0792	0.5126		-0.60	-0.7	3	3.01	3.01	0.62	0.78	0.62	0.626	0.789	Gavryushkina OA et al.	2019	Lithos	326–327	71-89
4619	8–07-19	CAOB	Russian	84.83333333	50.85	monzodiorite		246	246	13.5	86.9	0.0936	0.512342		-0.52	-5.8	-2.4	-2.54	-2.54	1.03	1.22	1.03	1.027	1.245	Gavryushkina OA et al.	2019	Lithos	326–327	71-89
4620	8–07-22	CAOB	Russian	84.83333333	50.85	syenite		246	246	11.3	72.6	0.0938	0.512361		-0.52	-5.4	-2.1	-2.17	-2.17	1.00	1.19	1.00	1.005	1.214	Gavryushkina OA et al.	2019	Lithos	326–327	71-89
4621	5–451	CAOB	Russian	84.83333333	50.85	syenogranite		246	246	7.2	47	0.0924	0.512287		-0.53	-6.8	-3.5	-3.57	-3.57	1.08	1.31	1.08	1.086	1.331	Gavryushkina OA et al.	2019	Lithos	326–327	71-89
4622	8002	CAOB	Russian	84.83333333	50.85	syenogranite		246	246	9.7	63.1	0.0926	0.512332		-0.53	-6.0	-2.6	-2.70	-2.70	1.03	1.24	1.03	1.031	1.258	Gavryushkina OA et al.	2019	Lithos	326–327	71-89
4623	A-041	CAOB	Russian	84.83333333	50.85	monzodiorite		248	248	8	54.2	0.0887	0.512606		-0.55	-0.6	2.8	2.79	2.79	0.66	0.79	0.66	0.666	0.805	Gavryushkina OA et al.	2019	Lithos	326–327	71-89
4624	09–103/2	CAOB	Russian	84.83333333	50.85	syenogranite		248	248	6.4	43.6	0.0889	0.512631		-0.55	-0.1	3.3	3.27	3.27	0.63	0.75	0.63	0.636	0.765	Gavryushkina OA et al.	2019	Lithos	326–327	71-89
4625	13–51/4	CAOB	Russian	84.83333333	50.85	monzodiorite		244	244	19.4	100.7	0.1162	0.512696		-0.41	1.1	3.6	3.64	3.64	0.71	0.72	0.71	0.712	0.731	Gavryushkina OA et al.	2019	Lithos	326–327	71-89
4626	13–54/2	CAOB	Russian	84.83333333	50.85	quartz diorite		244	244	18.1	91.2	0.1203	0.512675		-0.39	0.7	3.1	3.10	3.10	0.78	0.76	0.78	0.778	0.776	Gavryushkina OA et al.	2019	Lithos	326–327	71-89
4627	13–55/2	CAOB	Russian	84.83333333	50.85	granodiorite		244	244	18	92.5	0.1174	0.512662		-0.40	0.5	2.9	2.94	2.94	0.77	0.78	0.77	0.775	0.79	Gavryushkina OA et al.	2019	Lithos	326–327	71-89
4628	8–376	CAOB	Russian	84.83333333	50.85	granite		244	244	3.4	20.7	0.0992	0.512591		-0.50	-0.9	2.1	2.12	2.12	0.74	0.84	0.74	0.747	0.858	Gavryushkina OA et al.	2019	Lithos	326–327	71-89
4629	13–52/2	CAOB	Russian	84.83333333	50.85	leucogranite		244	244	0.8	7	0.0712	0.512576		-0.64	-1.2	2.7	2.70	2.70	0.61	0.80	0.80	0.616	0.81	Gavryushkina OA et al.	2019	Lithos	326–327	71-89
4630	1711	CAOB	Mongolia	90.00533333	54.00266667	Gabbro	Kogtakh massif	500	500	7.84	36.9	0.128441	0.512627	14	-0.35	-0.2	4.14	4.14	4.14	0.94	0.89	0.94			Vrublevskii VV et al	2018	Russian Geology and Geophysics	59(7)	718-744
4631	1702	CAOB	Mongolia	90.00533333	54.00266667	monzodiorite	Kogtakh massif	500	500	7.88	46.93	0.101466	0.512511	10	-0.48	-2.5	3.61	3.60	3.60	0.87	0.93	0.87			Vrublevskii VV et al	2018	Russian Geology and Geophysics	59(7)	718-744
4632	1714	CAOB	Mongolia	90.00533333	54.00266667	monzodiorite	Kogtakh massif	501	501	6.21	38.78	0.096826	0.512513	10	-0.51	-2.4	3.95	3.95	3.95	0.83	0.90	0.83			Vrublevskii VV et al	2018	Russian Geology and Geophysics	59(7)	718-744
4633	1764	CAOB	Mongolia	90.0045	54.0035	monzonite	Kashpar massif	489	489	6.27	36.04	0.1052	0.512586	10	-0.47	-1.0	4.82	4.70	4.70	0.79	0.83	0.79			Vrublevskii VV et al	2018	Russian Geology and Geophysics	59(7)	718-744
4634	1770	CAOB	Mongolia	90.0045	54.0035	monzonite	Kashpar massif	483	489	3.13	21.02	0.090075	0.512568	9	-0.54	-1.4	5.45	5.29	5.29	0.72	0.79	0.72			Vrublevskii VV et al	2018	Russian Geology and Geophysics	59(7)	718-744
4635	1	CAOB	Mongolia	90.0045	54.0035	monzodiorite	Chas-Taiga massif	489	489	10.79	63.43	0.102841	0.512538	5	-0.48	-2.0	4.04	3.91	3.91	0.84	0.90	0.84			Vrublevskii VV et al	2018	Russian Geology and Geophysics	59(7)	718-744
4636	4215	CAOB	Mongolia	90.0045	54.01533333	monzonite	Kiskach massif	483	483	6.08	40.36	0.090983	0.512494	5	-0.54	-2.8	3.95	3.71	3.71	0.82	0.91	0.82			Vrublevskii VV et al	2018	Russian Geology and Geophysics	59(7)	718-744
4637	14LQ11D	CAOB	NE China	104.9034833	39.98658333	Qtz diorite	Alxa Terrane	268	268	8.5	36.2	0.1419	0.512016	8	-0.28	-12.1	-10.3	-10.26	-10.26	2.40	1.86	1.86			Liu Q et al.	2017	Lithos	274–275	19-30
4638	14LQ11E	CAOB	NE China	104.9034833	39.98658333	Qtz diorite	Alxa Terrane	268	268	6.56	33.5	0.1183	0.512018	11	-0.40	-12.1	-9.4	-9.41	-9.41	1.80	1.80	1.80			Yuan LL et al.	2019	Lithos	324–325	859-876
4639	EL14-18-2	CAOB	NE China	111.2083333	43.13333333	Dlabase	Erenhot	141	150	9.583	52.61	0.1103	0.51269	12	-0.44	1.0	2.6	2.67	2.67	0.68	0.72	0.68			Yuan LL et al.	2019	Lithos	324–325	859-876
4640	EL12-5-1	CAOB	NE China	111.2083333	43.16666667	Monzodiorite	Erenhot	141	150	11.93	68.67	0.1052	0.512676	9	-0.47	0.7	2.4	2.49	2.49	0.67	0.74	0.67			Yuan LL et al.	2019	Lithos	324–325	859-876
4641	EL12-5-3	CAOB	NE China	111.2083333	43.16666667	Monzodiorite	Erenhot	141	150	12.32	71.18	0.1047	0.512662	14	-0.47	0.5	2.1	2.23	2.23	0.68	0.76	0.68			Yuan LL et al.	2019	Lithos	324–325	859-876
4642	EL12-3-1	CAOB	NE China	110.2166667	43.1	Syenogranite	Erenhot	141	150	3.009	19.11	0.0954	0.51256	13	-0.51	-1.5	0.3	0.42	0.42	0.76	0.91	0.76			Yuan LL et al.	2019	Lithos	324–325	859-876
4643	EL12-3-2	CAOB	NE China	110.2166667	43.1	Syenogranite	Erenhot	141	150	1.999	11.82	0.1023	0.512547	10	-0.48	-1.8	-0.1	0.03	0.03	0.83	0.94	0.83			Yuan LL et al.	2019	Lithos	324–325	859-876
4644	EL12-3-4	CAOB	NE China	110.2166667	43.1	Syenogranite	Erenhot	141	150	2.982	19.44	0.0929	0.51254	13	-0.53	-1.9	0	0.08	0.08	0.77	0.93	0.77			Yuan LL et al.	2019	Lithos	324–325	859-876
4645	C-1189	CAOB	Russia	131.2166667	51.03333333	biotite gneiss	ulovchikha Complex	950	600	4.27	18.33	0.1409	0.512143	3	-0.28	-9.7	-2.9	-5.38	-5.38	2.10	1.74	1.74			Sorokin AA et al.	2019	Lithos	332–333	340-354
4646	R-58-1	CAOB	Russia	131.5833333	50.58333333	biotite gneiss	ulovchikha Complex	950	600	4.79	21.3	0.1361	0.512146	2	-0.31	-9.6	-2.2	-4.96	-4.96	1.97	1.71	1.97			Sorokin AA et al.	2019	Lithos	332–333	340-354
4647	R-30	CAOB	Russia	131.5833333	50.58333333	biotite gneiss	ulovchikha Complex	950	600	6.78	32.1	0.1277	0.512126	3	-0.35	-10.0	-1.6	-4.71	-4.71	1.81	1.69	1.81			Sorokin AA et al.	2019	Lithos	332–333	340-354
4648	R-31	CAOB	Russia	131.5833333	50.58333333	biotite gneiss	ulovchikha Complex	950	600	5.67	26.0	0.1318	0.512180	2	-0.33	-8.9	-1.0	-3.97	-3.97	1.80	1.63	1.80			Sorokin AA et al.	2019	Lithos	332–333	340-354
4649	R-32	CAOB	Russia	131.5833333	50.58333333	biotite gneiss	ulovchikha Complex	950	600	4.13	17.76	0.1406	0.512074	1	-0.29	-11.0	-4.2	-6.71	-6.71	2.23	1.85	1.85			Sorokin AA et al.	2019	Lithos	332–333	340-354
4650	R-33	CAOB	Russia	131.5833333	50.58333333	biotite gneiss	ulovchikha Complex	950	600	3.64	17.87	0.1230	0.512072	3	-0.37	-11.0	-2.1	-5.40	-5.40	1.81	1.74	1.81			Sorokin AA et al.	2019	Lithos	332–333	340-354
4651	R-36	CAOB	Russia	131.5833333	50.58333333	biotite gneiss	ulovchikha Complex	950	600	3.21	15.12	0.1283	0.512077	3	-0.35	-10.9	-2.6	-5.71	-5.71	1.91	1.77	1.91			Sorokin AA et al.	2019	Lithos	332–333	340-354
4652	R-92	CAOB	Russia	131.5833333	50.58333333	biotite gneiss	ulovchikha Complex	950	600	7.58	39.5	0.1161	0.512028	2	-0.41	-11.9	-2.1	-5.73	-5.73	1.75	1.77	1.75			Sorokin AA et al.	2019	Lithos	332–333	340-354
4653	R-95	CAOB	Russia	131.5833333	50.58333333	biotite gneiss	ulovchikha Complex	950	600	4.85	20.9	0.1401	0.512229	2	-0.29	-8.0	-1.1	-3.64	-3.64	1.90	1.60	1.60			Sorokin AA et al.	2019	Lithos	332–333	340-354
4654	R-99	CAOB	Russia	131.5833333	50.58333333	biotite gneiss	ulovchikha Complex	950	600	11.65	64.3	0.1095	0.512125	2	-0.44	-10.0	0.6	-3.34	-3.34	1.50	1.58	1.50			Sorokin AA et al.	2019	Lithos	332–333	340-354
4655	C-1261	CAOB	Russia	131.5833333	50.58333333	biotite gneiss	ulovchikha Complex	950	600	4.50	19.39	0.1402	0.511985	4	-0.29	-12.7	-5.9	-8.41	-8.41	2.40	1.99	1.99			Sorokin AA et al.	2019	Lithos	332–333	340-354
4656	C-1152	CAOB	Russia	131.2166667	51.03333333	Granite	ulovchikha Complex	940	600	5.98	26.2	0.1378	0.512242	2	-0.30	-7.7	-0.6	-3.22	-3.22	1.82	1.57	1.82			Sorokin AA et al.	2019	Lithos	332–333	340-354
4657	C-1152-1	CAOB	Russia	131.2166667	51.03333333	Granite	ulovchikha Complex	940	600	2.69	11.38	0.1427	0.512188	4	-0.27	-8.8	-2.3	-4.65	-4.65	2.06	1.68	1.68			Sorokin AA et al.	2019	Lithos	332–333	340-354
4658	C-1154	CAOB	Russia	131.2166667	51.03333333	Granite	ulovchikha Complex	940	600	6.10	30.3	0.1215	0.512088	2	-0.38	-10.7	-1.7	-4.97	-4.97	1.75	1.71	1.75			Sorokin AA et al.	2019	Lithos	332–333	340-354
4659	C-1154-1	CAOB	Russia	131.2166667	51.03333333	Granite	ulovchikha Complex	940	600	5.14	22.0	0.1413	0.512117	3	-0.28	-10.2	-3.5	-5.92	-5.92	2.17	1.78	1.78			Sorokin AA et al.	2019	Lithos	332–333	340-354
4660	C-1191	CAOB	Russia	131.2166667	51.03333333	Granite	ulovchikha Complex	933	600	8.19	38.1	0.1299	0.512140	2	-0.34	-9.7	-1.7	-4.60	-4.60	1.83	1.68	1.83			Sorokin AA et al.	2019	Lithos	332–333	340-354
4661	C-1214-6	CAOB	Russia	131.2166667	51.03333333	Granite	ulovchikha Complex	933	600	3.75	19.91	0.1138	0.512006	4	-0.42	-12.3	-2.4	-5.98	-5.98	1.74	1.79	1.74			Sorokin AA et al.	2019	Lithos	332–333	340-354
4662	C-1211	CAOB	Russia	131.2166667	51.03333333	Granite	ulovchikha Complex	933	600	2.10	7.05	0.1803	0.512518	4	-0.08	-2.3	-0.4	-1.09	-1.09	2.87	1.39	1.39			Sorokin AA et al.	2019	Lithos	332–333	340-354
4663	C-1187	CAOB	Russia	131.2166667	51.03333333	Granite	ulovchikha Complex	933	600	3.77	13.92	0.1638	0.512491	2	-0.17	-2.9	1.1	-0.35	-0.35	2.01	1.33	1.33			Sorokin AA et al.	2019	Lithos	332–333	340-354
4664	C-1192	CAOB	Russia	131.2166667	51.03333333	Granite	ulovchikha Complex	933	600	2.75	11.62	0.1433	0.512480	3	-0.27	-3.1	3.3	1.02	1.02	1.45	1.22	1.22			Sorokin AA et al.	2019	Lithos	332–333	340-354
4665	C-1192-1	CAOB	Russia	131.2166667	51.03333333	Granite	ulovchikha Complex	933	600	3.23	14.07	0.1389	0.512472	3	-0.29	-3.2	3.7	1.18	1.18	1.38	1.21	1.38			Sorokin AA et al.	2019	Lithos	332–333	340-354
4666	C-1213	CAOB	Russia	131.2166667	51.03333333	Granite	ulovchikha Complex	933	600	2.05	7.56	0.1642	0.512443	3	-0.16	-3.8	0.1	-1.32	-1.32	2.17	1.41	1.41			Sorokin AA et al.	2019	Lithos	332–333	340-354
4667	D0925	CAOB	China	104.99583	46.14611	Granodiorite		350	350	4.2701	26.90919	0.095915	0.51231918		-0.51	-6.2	-1.7	-1.72	-1.72	1.07	1.24	1.07			Ganbat A et al.	2021	Lithos	404–405	106455
4668	D1709	CAOB	China	104.77056	47.07903	Rhyolite		274	274	4.4565	19.18895	0.140295	0.51267275		-0.29	0.7	2.7	2.65	2.65	0.99	0.82	0.82			Ganbat A et al.	2021	Lithos	404–405	106455
4669	D1710	CAOB	China	104.13389	47.06842	Syenogranite		282	282	4.71	21.13731	0.127136	0.51271864		-0.35	1.6	4.1	4.08	4.08	0.76	0.71	0.76			Ganbat A et al.	2021	Lithos	404–405	106455
4670	D1726	CAOB	China	104.33722	46.93847	Granodiorite		220	220	4.9265	19.6204	0.15177	0.51253692		-0.23	-2.0	-0.7	-0.71	-0.71	1.51	1.05	1.05			Ganbat A et al.	2021	Lithos	404–405	106455
4671	D1742	CAOB	China	104.62325	46.67053	Monzogranite		240	240	2.6504	20.11185	0.079656	0.51253893		-0.60	-1.9	1.7	1.65	1.65	0.70	0.88	0.70			Ganbat A et al.	2021	Lithos	404–405	106455
4672	NM-190①	CAOB	China	116.2488333	43.84333333	Felsic rocks		272	272	1.9	9.2	0.122	0.512677		-0.38	0.8	3.4	3.35	3.35	0.79	0.77	0.79			Zhu JB	2022	Lithos	424–425	106765
4673	NM-191①	CAOB	China	116.2488333	43.84333333	Felsic rocks		272	272	1.1	12.1	0.0563	0.512661		-0.71	0.4	5.3	5.32	5.32	0.47	0.60	0.60			Zhu JB	2022	Lithos	424–425	106765
4674	NM-193①	CAOB	China	116.2488333	43.84333333	Felsic rocks		322	322	3	17.9	0.1013	0.51255		-0.49	-1.7	2.2	2.20	2.20	0.81	0.90	0.81			Zhu JB	2022	Lithos	424–425	106765
4675	NM-195①	CAOB	China	116.2366667	43.87666667	Felsic rocks		322	322	1.5	8.2	0.1106	0.512695		-0.44	1.1	4.7	4.65	4.65	0.67	0.70	0.67			Zhu JB	2022	Lithos	424–425	106765
4676	NM-195②	CAOB	China	116.2366667	43.87666667	Felsic rocks		322	322	2.1	10.6	0.1185	0.512722		-0.40	1.6	4.9	4.85	4.85	0.69	0.68	0.69			Zhu JB	2022	Lithos	424–425	106765
4677	SDHS-6	CAOB	northwestern China	96.00166667	42.63166667	Granodiorite	EasternTianshanTerrane	427	427	1.7	7.2	0.1439	0.5128		-0.27	3.2	5.9	6.04	6.04	0.76	0.67	0.67			He Xh et al.	2021	Ore Geology Reviews	135	104228
4678	SDHS-7	CAOB	northwestern China	96.00166667	42.63166667	Granodiorite	EasternTianshanTerrane	427	427	1.7	9.4	0.11	0.5127		-0.44	1.2	6.6	5.93	5.93	0.66	0.68	0.66			He Xh et al.	2021	Ore Geology Reviews	135	104228
4679	SDHS-11	CAOB	northwestern China	96.00166667	42.63166667	Granodiorite	EasternTianshanTerrane	427	427	2.9	13.2	0.1307	0.5128		-0.34	3.2	6.4	6.76	6.76	0.64	0.61	0.64			He Xh et al.	2021	Ore Geology Reviews	135	104228
4680	SDHS-14	CAOB	northwestern China	96.00166667	42.63166667	Granodiorite	EasternTianshanTerrane	427	427	2.7	12.4	0.1289	0.5128		-0.34	3.2	6.4	6.85	6.85	0.63	0.61	0.63			He Xh et al.	2021	Ore Geology Reviews	135	104228
4681	ZK002-3	CAOB	northwestern China	95.99666667	42.63666667	Granodiorite	EasternTianshanTerrane	427	427	2.7	13.4	0.1242	0.5128		-0.37	3.2	6.6	7.11	7.11	0.60	0.59	0.60			He Xh et al.	2021	Ore Geology Reviews	135	104228
4682	ZK002-10	CAOB	northwestern China	95.99666667	42.63666667	Granodiorite	EasternTianshanTerrane	427	427	3	13.8	0.1319	0.5128		-0.33	3.2	6.4	6.69	6.69	0.65	0.62	0.65			He Xh et al.	2021	Ore Geology Reviews	135	104228
4683	SDHS-2	CAOB	northwestern China	95.995	42.64283333	Monzogranite	EasternTianshanTerrane	382	382	2.2	10.1	0.1327	0.5128		-0.33	3.2	6.9	6.28	6.28	0.66	0.62	0.66			He Xh et al.	2021	Ore Geology Reviews	135	104228
4684	SDHS-3	CAOB	northwestern China	96.00016667	42.62333333	Monzogranite	EasternTianshanTerrane	382	382	2.2	14.7	0.0898	0.5127		-0.54	1.2	6.6	6.42	6.42	0.55	0.61	0.55			He Xh et al.	2021	Ore Geology Reviews	135	104228
4685	ZK8202-5	CAOB	NW China	96.05	42.51666667	Monzogranite	Hongling deposit	238	238	2.94	16.6	0.1067	0.512663		-0.46	0.5	3.23	3.22	3.22	0.69	0.75	0.69			He Xh et al.	2022	Ore Geology Reviews	146	104947
4686	ZK8601-1	CAOB	NW China	96.05	42.51666667	Monzogranite	Hongling deposit	238	238	2.41	14	0.104	0.512663		-0.47	0.5	3.31	3.30	3.30	0.68	0.74	0.68			He Xh et al.	2022	Ore Geology Reviews	146	104947
4687	HL-6	CAOB	NW China	96.05	42.51666667	Monzogranite	Hongling deposit	234	234	2.28	12.1	0.1134	0.512702		-0.42	1.2	3.73	3.74	3.74	0.68	0.70	0.68			He Xh et al.	2022	Ore Geology Reviews	146	104947
4688	HL-10	CAOB	NW China	96.05	42.51666667	Monzogranite	Hongling deposit	234	234	1.76	8.84	0.1204	0.512691		-0.39	1.0	3.3	3.31	3.31	0.75	0.74	0.75			He Xh et al.	2022	Ore Geology Reviews	146	104947
4689	ZK8601-5	CAOB	NW China	96.05	42.51666667	Monzogranite	Hongling deposit	234	234	2.87	16	0.1082	0.512653		-0.45	0.3	2.92	2.94	2.94	0.72	0.77	0.72			He Xh et al.	2022	Ore Geology Reviews	146	104947
4690	ZK8601-7	CAOB	NW China	96.05	42.51666667	Monzogranite	Hongling deposit	234	234	2.84	14.7	0.1165	0.512665		-0.41	0.5	2.91	2.92	2.92	0.76	0.77	0.76			He Xh et al.	2022	Ore Geology Reviews	146	104947
4691	ZK8202-6	CAOB	NW China	96.05	42.51666667	Monzogranite porphyry	Hongling deposit	232	232	2.74	13.8	0.1204	0.51267		-0.39	0.6	2.89	2.88	2.88	0.78	0.77	0.78			He Xh et al.	2022	Ore Geology Reviews	146	104947
4692	HL-1	CAOB	NW China	96.055	42.50983333	Porphyritic granite	Hongling deposit	232	232	2.32	9.09	0.154	0.512706		-0.22	1.3	2.59	2.59	2.59	1.13	0.79	0.79			He Xh et al.	2022	Ore Geology Reviews	146	104947
4693	HL-2	CAOB	NW China	96.055	42.50983333	Porphyritic granite	Hongling deposit	232	232	2.47	10.5	0.1417	0.512687		-0.28	1.0	2.59	2.58	2.58	0.98	0.79	0.79			He Xh et al.	2022	Ore Geology Reviews	146	104947
4694	HL-5	CAOB	NW China	96.055	42.50983333	Porphyritic granite	Hongling deposit	232	232	3.02	13	0.1404	0.512704		-0.29	1.3	2.96	2.95	2.95	0.93	0.76	0.76			He Xh et al.	2022	Ore Geology Reviews	146	104947
4695	HL-9	CAOB	NW China	96.055	42.50983333	Porphyritic granite	Hongling deposit	232	232	2.77	13.5	0.1237	0.512689		-0.37	1.0	3.16	3.16	3.16	0.78	0.75	0.78			He Xh et al.	2022	Ore Geology Reviews	146	104947
4696	XWQ6-QY17	CAOB	North China	117.0216667	44.04166667	Andesite	Xilinhot	262	262	10.739	64.3419	0.100892	0.51203231	14	-0.49	-11.8	-8.6	-8.61	-8.61	1.51	1.73	1.51	1.51	1.76	Li JL et al.	2021	Lithos	384–385	105969
4697	XWQ6-QY18	CAOB	North China	117.0216667	44.04166667	Andesite	Xilinhot	262	262	12.477	74.66127	0.101021	0.51203183	8	-0.49	-11.8	-8.6	-8.63	-8.63	1.51	1.73	1.51	1.51	1.76	Li JL et al.	2021	Lithos	384–385	105969
4698	XWQ6-QY20	CAOB	North China	117.0216667	44.04166667	Andesite	Xilinhot	262	262	12.711	75.77986	0.101398	0.51204952	12	-0.48	-11.5	-8.3	-8.29	-8.29	1.49	1.70	1.49	1.49	1.73	Li JL et al.	2021	Lithos	384–385	105969
4699	XWQ6-QY21	CAOB	North China	117.0216667	44.04166667	Andesite	Xilinhot	262	262	12.373	75.0979	0.099592	0.51202666	5	-0.49	-11.9	-8.7	-8.68	-8.68	1.50	1.74	1.50	1.50	1.77	Li JL et al.	2021	Lithos	384–385	105969
4700	MD1-1	CAOB	North China	116.9833333	44.01666667	Andesite	Xilinhot	305	305	8.6012	33.94104	0.153216	0.51287925	7	-0.22	4.7	6.4	6.40	6.40	0.68	0.54	0.54	0.68	0.55	Li JL et al.	2021	Lithos	384–385	105969

4701	MD1-2	CAOB	North China	116.9833333	44.01666667	Andesite	Xilinhot	305	305	8.4132	32.11136	0.158408	0.51289343	8	-0.19	5.0	6.5	6.47	6.47	0.71	0.54	0.54	0.71	0.54	Li JL et al.	2021	Lithos	384–385	105969
4702	MD1-3	CAOB	North China	116.9833333	44.01666667	Andesite	Xilinhot	305	305	9.3133	35.65482	0.157929	0.51290778	9	-0.20	5.3	6.8	6.77	6.77	0.66	0.51	0.51	0.66	0.52	Li JL et al.	2021	Lithos	384–385	105969
4703	MD1-4	CAOB	North China	116.9833333	44.01666667	Andesite	Xilinhot	305	305	9.8929	37.60311	0.159064	0.51288901	8	-0.19	4.9	6.4	6.36	6.36	0.73	0.55	0.55	0.73	0.55	Li JL et al.	2021	Lithos	384–385	105969
4704	MD1-5	CAOB	North China	115.0216667	45.06666667	Andesite	Xilinhot	305	305	7.1237	27.73789	0.155276	0.51284275	12	-0.21	4.0	5.6	5.61	5.61	0.80	0.61	0.61	0.80	0.62	Li JL et al.	2021	Lithos	384–385	105969
4705	DWQ2-QY6	CAOB	North China	115.0216667	45.06666667	Rhyolite	Xilinhot	316	316	5.0748	26.07111	0.117681	0.51257408	10	-0.40	-1.2	1.9	1.94	1.94	0.91	0.92	0.91	0.91	0.93	Li JL et al.	2021	Lithos	384–385	105969
4706	DWQ2-QY7	CAOB	North China	115.0216667	45.06666667	Rhyolite	Xilinhot	316	316	4.4276	23.17362	0.115509	0.51256943	10	-0.41	-1.3	1.9	1.94	1.94	0.90	0.92	0.90	0.90	0.93	Li JL et al.	2021	Lithos	384–385	105969
4707	DWQ2-QY9	CAOB	North China	115.0216667	45.06666667	Rhyolite	Xilinhot	316	316	5.0415	26.04948	0.117003	0.51256026	5	-0.41	-1.5	1.7	1.70	1.70	0.93	0.94	0.93	0.93	0.95	Li JL et al.	2021	Lithos	384–385	105969
4708	DWQ2-QY10	CAOB	North China	115.0216667	45.06666667	Rhyolite	Xilinhot	316	316	5.2513	26.90396	0.118004	0.51257056	6	-0.40	-1.3	1.9	1.86	1.86	0.92	0.92	0.92	0.92	0.94	Li JL et al.	2021	Lithos	384–385	105969
4709	DWQ5-QY16	CAOB	North China	115.0058333	45.05833333	Alkali-feldspar granite	Xilinhot	300	300	10.41	46.1754	0.136297	0.5127525	9	-0.31	2.2	4.5	4.55	4.55	0.78	0.69	0.78	0.78	0.70	Li JL et al.	2021	Lithos	384–385	105969
4710	DWQ5-QY17	CAOB	North China	115.0058333	45.05833333	Alkali-feldspar granite	Xilinhot	300	300	5.9242	25.27791	0.141693	0.51275666	7	-0.28	2.3	4.4	4.42	4.42	0.83	0.70	0.70	0.83	0.71	Li JL et al.	2021	Lithos	384–385	105969
4711	DWQ5-QY18	CAOB	North China	115.0058333	45.05833333	Alkali-feldspar granite	Xilinhot	300	300	6.9475	30.07546	0.13966	0.51271008	5	-0.29	1.4	3.6	3.59	3.59	0.91	0.77	0.91	0.91	0.78	Li JL et al.	2021	Lithos	384–385	105969
4712	NM12-146	CAOB	Inner Mongolia	112.65	43.85	Rhyolite	stern Erenhot ophiolitic comp	360	360	3.02	10.25	0.1863	0.512962		-0.05	6.3	6.8	6.80	6.80	1.05	0.56	0.56	1.044	0.548	Yang JF et al.	2017	Journal of Asian Earth Sciences	144	126-140
4713	NM12-147	CAOB	Inner Mongolia	112.65	43.85	Rhyolite	stern Erenhot ophiolitic comp	360	360	2.26	7.86	0.1818	0.51296		-0.08	6.3	7	6.97	6.97	0.91	0.54	0.54	0.907	0.535	Yang JF et al.	2017	Journal of Asian Earth Sciences	144	126-140
4714	NM12-149	CAOB	Inner Mongolia	112.65	43.85	Plagiogranite	stern Erenhot ophiolitic comp	356	356	2.12	8.66	0.1548	0.513073		-0.21	8.5	10.4	10.39	10.39	0.36	0.36	0.36	0.201	0.258	Yang JF et al.	2017	Journal of Asian Earth Sciences	144	126-140
4715	NM12-151	CAOB	Inner Mongolia	112.65	43.85	Plagiogranite	stern Erenhot ophiolitic comp	356	356	1.23	5.23	0.1487	0.513028		-0.24	7.6	9.8	9.79	9.79	0.36	0.36	0.36	0.287	0.307	Yang JF et al.	2017	Journal of Asian Earth Sciences	144	126-140
4716	NM12-152	CAOB	Inner Mongolia	112.65	43.85	Plagiogranite	stern Erenhot ophiolitic comp	348	348	0.97	4.21	0.1457	0.513021		-0.26	7.5	9.8	9.74	9.74	0.36	0.35	0.35	0.289	0.307	Yang JF et al.	2017	Journal of Asian Earth Sciences	144	126-140
4717	NM12-153	CAOB	Inner Mongolia	112.65	43.85	Plagiogranite	stern Erenhot ophiolitic comp	348	348	1.18	5.64	0.1323	0.513037		-0.33	7.8	10.7	10.64	10.64	0.35	0.35	0.35	0.211	0.232	Yang JF et al.	2017	Journal of Asian Earth Sciences	144	126-140

Note:

Table S1b. Whole-rock Sm-Nd data of the mafic rocks of the Altaids.

No.	Sample	GeoTectonic Unit	Tectonic unit/Locationh	Longitude	Latitude	Lithology	Pluton	Sample Age (Ma)	Revised Age (Ma)	Sm	Nd	147Sm/144Nd	143Nd/144Nd	2σ	$f_{Sm/Nd}$	Calcul.ε$_{Nd}$(0)	Orogi. ε$_{Nd}$(t)	Recal. ε$_{Nd}$(t)	Mapped ε$_{Nd}$(t)	Mapped T_{DM} (Ga)	Recal. T_{DM1} (Ga)	Recal. T_{DM2} (Ga)	Origi. T_{DM1} (Ga)	Origi. T_{DM2} (Ga)	Res. Age (Ma)	Ref. Author	Ref. year	Ref_Journal	Volume and	Page
1	TS5	CAOB	Tianshan Terrane	80.83	45.83	Amphibolite		1700	600	12.10	48.04	0.15230	0.512459	42	-0.23	-3.49	6.14	-0.09	-0.09	1.71	1.71	1.31			1111	Hu. A et al.	2000	Tectonophysics	328(1–2)	15-51
2	TS6	CAOB	Tianshan Terrane	80.83	45.83	Amphibolite		1700	600	10.64	41.05	0.15670	0.512548	7	-0.20	-1.76	6.92	1.31	1.31	1.61	1.61	1.20			1006	Hu. A et al.	2000	Tectonophysics	328(1–2)	15-51
3	TS7	CAOB	Tianshan Terrane	80.83	45.83	Amphibolite		1700	600	9.20	35.88	0.15490	0.512512	9	-0.21	-2.46	6.61	0.74	0.74	1.65	1.65	1.24			1050	Hu. A et al.	2000	Tectonophysics	328(1–2)	15-51
4	TS8	CAOB	Tianshan Terrane	80.83	45.83	Amphibolite		1700	600	8.94	36.09	0.14970	0.512401	5	-0.24	-4.62	5.57	-1.02	-1.02	1.78	1.78	1.39			1179	Hu. A et al.	2000	Tectonophysics	328(1–2)	15-51
5	TS9	CAOB	Tianshan Terrane	80.83	45.83	Amphibolite		1700	600	8.57	34.11	0.15190	0.512453	8	-0.23	-3.61	6.11	-0.18	-0.18	1.71	1.71	1.32			1115	Hu. A et al.	2000	Tectonophysics	328(1–2)	15-51
6	TS10	CAOB	Tianshan Terrane	80.83	45.83	Amphibolite		1700	600	5.59	21.53	0.15690	0.512484	6	-0.20	-3.00	5.63	0.04	0.04	1.78	1.78	1.30			1182	Hu. A et al.	2000	Tectonophysics	328(1–2)	15-51
7	TS11	CAOB	Tianshan Terrane	80.83	45.83	Amphibolite		1700	600	14.26	58.87	0.14640	0.512401	8	-0.26	-4.62	6.29	-0.77	-0.77	1.69	1.69	1.37			1092	Hu. A et al.	2000	Tectonophysics	328(1–2)	15-51
8	TS12	CAOB	Tianshan Terrane	80.83	45.83	Amphibolite		1700	600	5.18	19.77	0.15830	0.512449	9	-0.20	-3.69	4.64	-0.75	-0.75	1.92	1.92	1.37			1323	Hu. A et al.	2000	Tectonophysics	328(1–2)	15-51
9	TS13	CAOB	Tianshan Terrane	80.83	45.83	Amphibolite		1700	600	5.41	21.12	0.15480	0.512445	7	-0.21	-3.76	5.32	-0.56	-0.56	1.82	1.82	1.35			1219	Hu. A et al.	2000	Tectonophysics	328(1–2)	15-51
10	TS14	CAOB	Tianshan Terrane	80.83	45.83	Amphibolite		1700	600	6.99	28.18	0.14990	0.512515	7	-0.24	-2.40	7.75	1.18	1.18	1.51	1.51	1.21			914	Hu. A et al.	2000	Tectonophysics	328(1–2)	15-51
11	TS15	CAOB	Tianshan Terrane	80.83	45.83	Amphibolite		1400	600	4.05	19.92	0.12290	0.512322	38	-0.38	-6.16	7.02	-0.51	-0.51	1.39	1.39	1.35			788	Hu. A et al.	2000	Tectonophysics	328(1–2)	15-51
12	TS16	CAOB	Tianshan Terrane	80.83	45.83	Amphibolite		1400	600	3.61	15.32	0.14260	0.512506	28	-0.28	-2.57	7.09	1.57	1.57	1.38	1.38	1.18			779	Hu. A et al.	2000	Tectonophysics	328(1–2)	15-51
13	TS29	CAOB	Tianshan Terrane	83.53	44.38	Amphibolite		1000	600	11.43	50.85	0.13590	0.512480	10	-0.31	-3.08	4.68	1.57	1.57	1.31	1.31	1.18			711	Hu. A et al.	2000	Tectonophysics	328(1–2)	15-51
14	TS30	CAOB	Tianshan Terrane	83.53	44.38	Amphibolite		1000	600	12.20	55.85	0.13210	0.512484	9	-0.33	-3.00	5.24	1.94	1.94	1.24	1.24	1.15			643	Hu. A et al.	2000	Tectonophysics	328(1–2)	15-51
15	TS31	CAOB	Tianshan Terrane	83.53	44.38	Amphibolite		1000	600	9.68	44.41	0.13180	0.512497	8	-0.33	-2.75	5.53	2.22	2.22	1.21	1.21	1.13			614	Hu. A et al.	2000	Tectonophysics	328(1–2)	15-51
16	TS32	CAOB	Tianshan Terrane	83.53	44.38	Meta-gabbro		700	600	2.50	10.43	0.14500	0.512190	12	-0.26	-8.74	-4.12	-4.78	-4.78	2.12	2.12	1.69		2	1522	Hu. A et al.	2000	Tectonophysics	328(1–2)	15-51
17	TS33	CAOB	Tianshan Terrane	83.53	44.38	Meta-Gabbro		1000	600	5.31	22.33	0.14380	0.512472	9	-0.27	-3.24	3.51	0.81	0.81	1.48	1.48	1.24		1	876	Hu. A et al.	2000	Tectonophysics	328(1–2)	15-51
18	TS34	CAOB	Tianshan Terrane	83.53	44.38	Amphibolite		1000	600	7.24	37.72	0.11600	0.511707	22	-0.41	-18.16	-7.86	-11.98	-11.98	2.24	2.24	2.28			1642	Hu. A et al.	2000	Tectonophysics	328(1–2)	15-51
19	TS66	CAOB	Tianshan Terrane	94.83	42.00	Amphibolite		1800	600	4.25	15.29	0.16810	0.512521	30	-0.15	-2.28	4.29	-0.09	-0.09	2.09	2.09	1.31			1495	Hu. A et al.	2000	Tectonophysics	328(1–2)	15-51
20	TS36	CAOB	Tianshan Terrane	83.53	44.39	Amphibolite		1000	600	3.27	15.9	0.12420	0.512169	30	-0.37	-9.15	0.10	-3.60	-3.60	1.67	1.67	1.60			1067	Hu. A et al.	2000	Tectonophysics	328(1–2)	15-51
21	TS37	CAOB	Tianshan Terrane	83.54	44.39	Amphibolite		1000	600	3.34	15	0.13450	0.512425	40	-0.32	-4.15	3.78	0.61	0.61	1.39	1.39	1.26			793	Hu. A et al.	2000	Tectonophysics	328(1–2)	15-51
22	TS38	CAOB	Tianshan Terrane	83.54	44.39	Amphibolite		1000	600	4.39	18.22	0.14570	0.512332	26	-0.26	-5.97	0.54	-2.06	-2.06	1.83	1.83	1.47			1228	Hu. A et al.	2000	Tectonophysics	328(1–2)	15-51
23	TS69	CAOB	Tianshan Terrane	94.83	42.00	Amphibolite		800	600	4.36	17.37	0.15160	0.512517	44	-0.23	-2.36	2.24	1.09	1.09	1.55	1.55	1.22			951	Hu. A et al.	2000	Tectonophysics	328(1–2)	15-51
24	TS70	CAOB	Tianshan Terrane	94.83	42.00	Amphibolite		800	600	3.30	14	0.14250	0.512518	8	-0.28	-2.34	3.19	1.81	1.81	1.35	1.35	1.16			751	Hu. A et al.	2000	Tectonophysics	328(1–2)	15-51
25	ALT18	CAOB	Altai Terrane	87.00	49.00	Amphibolite		700	600	11.20	47.04	0.14390	0.512720	7	-0.27	1.60	6.32	5.64	5.64	0.94	0.94	0.85			339	Hu. A et al.	2000	Tectonophysics	328(1–2)	15-51
26	ALT19	CAOB	Altai Terrane	87.00	49.00	Amphibolite		700	600	9.51	39.54	0.14540	0.512737	9	-0.26	1.93	6.51	5.86	5.86	0.92	0.92	0.83			322	Hu. A et al.	2000	Tectonophysics	328(1–2)	15-51
27	ALT20	CAOB	Altai Terrane	87.00	49.00	Amphibolite		700	600	8.92	36.59	0.14740	0.512752	6	-0.25	2.22	6.63	6.00	6.00	0.92	0.92	0.82			315	Hu. A et al.	2000	Tectonophysics	328(1–2)	15-51
28	ALT21	CAOB	Altai Terrane	87.00	49.00	Amphibolite		700	600	10.07	42.6	0.14290	0.512712	10	-0.27	1.44	6.25	5.56	5.56	0.94	0.94	0.85			343	Hu. A et al.	2000	Tectonophysics	328(1–2)	15-51
29	ALT22	CAOB	Altai Terrane	87.00	49.00	Amphibolite		700	600	10.84	46.03	0.14240	0.512711	8	-0.28	1.42	6.27	5.58	5.58	0.94	0.94	0.85			339	Hu. A et al.	2000	Tectonophysics	328(1–2)	15-51
30	ALT16	CAOB	Altai Terrane	89.92	47.50	Amphibolite		700	600	2.10	6.58	0.19330	0.512951	18	-0.02	6.11	6.41	6.37	6.37	1.48	1.48	0.79			884	Hu. A et al.	2000	Tectonophysics	328(1–2)	15-51
31	ALT23	CAOB	Altai Terrane	87.00	49.00	Amphibolite		700	600	10.87	46.44	0.14150	0.512701	8	-0.28	1.23	6.16	5.46	5.46	0.95	0.95	0.86			348	Hu. A et al.	2000	Tectonophysics	328(1–2)	15-51
32	ER25-6	CAOB	Ergun	120.50	51.50	Gabbro		465	465			0.12600	0.512340	2	-0.36	-5.81	-1.62	-1.62	-0.40	1.41	1.41	1.33		1407	941	Zhao S.	2017	l Dissertation of Jilin University		
33	ER25-S	CAOB	Ergun	120.50	51.50	Gabbro		465	465			0.12520	0.512345	3	-0.36	-5.72	-1.47	-1.47	-0.24	1.38	1.38	1.32		1384	920	Zhao S.	2017	l Dissertation of Jilin University		
34	ER27-2	CAOB	Ergun	120.78	51.57	Gabbro		792	600			0.12070	0.512225	3	-0.39	-8.06	-2.24	-2.24	-2.24	1.51	1.51	1.49		1513	913	Zhao S.	2017	l Dissertation of Jilin University		
35	ER27-3	CAOB	Ergun	120.50	51.50	Gabbro		792	600			0.11620	0.512213	4	-0.41	-8.29	-2.13	-2.13	-2.13	1.46	1.46	1.48		1462	862	Zhao S.	2017	l Dissertation of Jilin University		
36	07-1b	CAOB	Kazakhstan continent	80.83	44.87	gabbro enclave		462	600	5.82	-3.2	0.12950	0.512474	6	-0.34	-3.20	0.80	1.95	1.95	1.22	1.22	1.15				Wang et al.	2012	nal of Asian Earth Scie	49	40-53
37	07-8c	CAOB	Kazakhstan continent	80.58	44.93	fine-grained amphibolite		950	600	6.01	1.67	0.14980	0.512283	12	-0.24	-6.92	-1.20	-3.33	-3.33	2.06	2.06	1.58				Wang et al.	2012	nal of Asian Earth Scie	49	40-53
38	NT43	CAOB	Tarim Terrane	86.00	40.53	Amphibolite		2500	600	4.54	20.63	0.13300	0.511825	32	-0.32	-15.86	4.50	-10.98	-10.98	2.49	2.49	2.19				Hu. A et al.	2000	Tectonophysics	328(1-2)	15-51
39	NT44	CAOB	Tarim Terrane	86.00	40.53	Amphibolite		2500	600	2.88	12.69	0.13730	0.511767	28	-0.30	-16.99	2.00	-12.44	-12.44	2.74	2.74	2.31				Hu. A et al.	2000	Tectonophysics	328(1-2)	15-51
40	NT45	CAOB	Tarim Terrane	86.01	40.53	Amphibolite		2500	600	3.50	16.91	0.12530	0.511563	28	-0.36	-20.97	2.00	-15.50	-15.50	2.72	2.72	2.56				Hu. A et al.	2000	Tectonophysics	328(1-2)	15-51
41	WWM11A	CAOB	Western Tianshan Oroger	86.79	43.04	Meta-gabbro		773	600	4.56	18.2	0.15140	0.512201	14	-0.23	-8.52	-4.30	-5.06	-5.06	2.31	2.31	1.71		1766	1712	Gao J et al.	2015	nal of Asian Earth Scie	113	173-193
42	WWM10-2	CAOB	Western Tianshan Oroger	86.27	42.62	Granite dyke		728	600	3.43	21.8	0.09550	0.511582	11	-0.51	-20.60	-11.20	-12.85	-12.85	2.02	2.02	2.35		2319	1415	Gao J et al.	2015	nal of Asian Earth Scie	113	173-193
43	NT1	CAOB	Tarim Terrane	88.58	42.50	Amphibolite		3300	600	4.01	13.24	0.18320	0.512521	41	-0.07	-2.28	3.40	-1.25	-1.25	1.40	3.12	1.40	3.13		802	Hu. A et al.	2000	Tectonophysics	328(1–2)	15-51
44	NT2	CAOB	Tarim Terrane	88.58	42.50	Amphibolite		3300	600	5.93	22.34	0.16040	0.512061	50	-0.18	-11.26	4.03	-8.48	-8.48	1.99	3.09	1.99	3.09		1387	Hu. A et al.	2000	Tectonophysics	328(1–2)	15-51
45	NT3	CAOB	Tarim Terrane	88.58	42.50	Amphibolite		3300	600	2.42	7.86	0.18620	0.512569	37	-0.05	-1.35	3.08	-0.54	-0.54	1.34	3.20	1.34	3.19		744	Hu. A et al.	2000	Tectonophysics	328(1–2)	15-51
46	NT4	CAOB	Tarim Terrane	88.58	42.50	Amphibolite		3300	600	2.61	8.54	0.18450	0.512538	37	-0.06	-1.95	3.19	-1.02	-1.02	1.38	3.17	1.38	3.17		783	Hu. A et al.	2000	Tectonophysics	328(1–2)	15-51
47	NT5	CAOB	Tarim Terrane	88.58	42.50	Amphibolite		3300	600	2.23	7.39	0.18250	0.512533	28	-0.07	-2.05	3.93	-0.96	-0.96	1.38	2.99	1.38	2.99		779	Hu. A et al.	2000	Tectonophysics	328(1–2)	15-51
48	NT6	CAOB	Tarim Terrane	88.58	42.50	Amphibolite		3300	600	3.29	10.26	0.19370	0.512710	31	-0.02	1.40	2.67	1.63	1.63	1.17	3.33	1.17	3.32		568	Hu. A et al.	2000	Tectonophysics	328(1–2)	15-51
49	NT7	CAOB	Tarim Terrane	88.58	42.50	Amphibolite		3300	600	4.42	13.97	0.19120	0.512688	26	-0.03	0.98	3.29	1.40	1.40	1.19	3.11	1.19	3.11		588	Hu. A et al.	2000	Tectonophysics	328(1–2)	15-51
50	NT8	CAOB	Tarim Terrane	88.58	42.50	Amphibolite		3300	600	3.78	18.08	0.12650	0.511308	39	-0.36	-25.94	3.62	-20.57	-20.57	3.20	3.20	2.97	3.20		2596	Hu. A et al.	2000	Tectonophysics	328(1–2)	15-51
51	NT9	CAOB	Tarim Terrane	88.58	42.50	Amphibolite		3300	600	3.16	14.93	0.12800	0.511282	28	-0.35	-26.45	2.48	-21.19	-21.19	3.30	3.30	3.02	3.30		2697	Hu. A et al.	2000	Tectonophysics	328(1–2)	15-51
52	NT10	CAOB	Tarim Terrane	88.58	42.50	Amphibolite		3300	600	2.72	7.97	0.20640	0.512921	49	0.05	5.52	1.44	4.78	4.78	0.91	4.72	0.91	4.71		311	Hu. A et al.	2000	Tectonophysics	328(1–2)	15-51
53	R0427	CAOB	SW Mongolia	98.02	45.49	metabasalt		940	600	3.10	9	0.20830	0.513204		0.06	11.04	9.70	10.15	10.15	0.48	-1.54	0.48			-118	Buriánek D et al.	2017	Gondwana Research	47	200–227
54	A2119	CAOB	SW Mongolia	98.04	45.47	metabasalt		940	600	4.70	20.9	0.13600	0.512802		-0.31	3.20	10.50	7.85	7.85	0.68	0.68	0.67		0.68	83	Buriánek D et al.	2017	Gondwana Research	47	200–227
55	A2157	CAOB	SW Mongolia	98.24	45.45	metatuf		940	600	1.60	5.4	0.17910	0.512863		-0.09	4.39	6.50	5.74	5.74	1.26	1.26	0.84			663	Buriánek D et al.	2017	Gondwana Research	47	200–227
56	R0374	CAOB	SW Mongolia	98.23	45.36	peridotite		940	600	2.80	7.7	0.21990	0.513163		0.12	10.24	7.50	8.46	8.46	0.62	0.32	0.62			18	Buriánek D et al.	2017	Gondwana Research	47	200–227
57	D0665	CAOB	SW Mongolia	98.11	45.42	basalt		940	600	2.20	7.4	0.17970	0.512898		-0.09	5.07	7.10	6.37	6.37	1.13	1.13	0.79		1.13	529	Buriánek D et al.	2017	Gondwana Research	47	200–227
58	D0759	CAOB	SW Mongolia	98.18	45.40	peridotite		940	600	0.10	0.4	0.15110	0.512500		-0.23	-2.69	3.70	0.80	0.80	1.58	1.58	1.24			979	Buriánek D et al.	2017	Gondwana Research	47	200–227
59	Ch-25-08	CAOB	Vasilkovo	81.39	49.20	Aphyric andesibasalt		366	366	0.50	2.6	0.19231	0.513043	5	-0.02	7.90	7.30	8.11	7.30	0.76	0.76	0.45				Safonova I et al.	2018	Gondwana Research	59	59-179
60	C-6b-08	CAOB	Ozyorny	81.78	49.26	Andesibasalt/gabbro-diorite		378	378	2.00	7	0.28571	0.512774	6	0.45	2.65	3.60	-1.64	3.60	1.27	-0.80	1.27				Safonova I et al.	2018	Gondwana Research	59	59-179
61	Ch-06-12	CAOB	Ozyorny	81.78	49.25	Microgabbro/basalt		378	378	4.10	18	0.22778	0.512755	5	0.16	2.28	4.60	0.78	4.60	1.08	-4.35	1.08				Safonova I et al.	2018	Gondwana Research	59	59-179
62	IT-62-17	CAOB	central Kazakhstan	76.36	46.81	porphyric basalt		470	470	6.05	23.67	0.15460	0.512134	10	-0.21	-9.83		-7.31	-7.31	2.61	2.61	1.79			2136	Safonova I et al.	2020	Gondwana Research	79	49-69
63	IT-63-17	CAOB	central Kazakhstan	76.36	46.80	porphyric basalt		470	470	10.34	41.85	0.14940	0.512163	14	-0.24	-9.27		-6.43	-6.43	2.33	2.33	1.72			1859	Safonova I et al.	2020	Gondwana Research	79	49-69
64	IT-64-17	CAOB	central Kazakhstan	76.36	46.80	aphyric basalt		470	470	10.60	43.27	0.14800	0.512178	7	-0.25	-8.97		-6.05	-6.05	2.25	2.25	1.69			1776	Safonova I et al.	2020	Gondwana Research	79	49-69
65	IT-22-17	CAOB	central Kazakhstan	76.35	46.82	aphyric basalt		470	470	9.21	43.11	0.12910	0.512211	10	-0.34	-8.33		-4.28	-4.45	1.69	1.69	1.55			1218	Safonova I et al.	2020	Gondwana Research	79	49-69
66	IT-35-17	CAOB	central Kazakhstan	76.75	46.72	aphyric basalt		470	470	13.26	59.15	0.13550	0.512316	6	-0.31	-6.28		-2.61	-2.53	1.62	1.62	1.41			1152	Safonova I et al.	2020	Gondwana Research	79	49-69
67	IT-76-17	CAOB	central Kazakhstan	76.28	46.88	dolerite		450	450	3.94	12.62	0.18860	0.512921	8	-0.04	5.52		5.99	6.02	1.39	1.39	0.70			939	Safonova I et al.	2020	Gondwana Research	79	49-69
68	IT-79-17	CAOB	central Kazakhstan	76.29	46.87	porphyric basalt		480	480	5.14	15.53	0.19980	0.513022	8	0.02	7.49		7.30	7.30	1.40	1.40	0.61			922	Safonova I et al.	2020	Gondwana Research	79	49-69
69	IT-80-17	CAOB	central Kazakhstan	76.29	46.88	aphyric basalt		480	480	5.42	16.76	0.19550	0.513026	7	-0.01	7.57		7.64	7.64	1.04	1.04	0.59			558	Safonova I et al.	2020	Gondwana Research	79	49-69
70	IT-73-17	CAOB	central Kazakhstan	76.31	46.83	gabbro-dolerite		480	480	4.15	12.37	0.20260	0.513027	18	0.03	7.59		7.23	7.23	1.69	1.69	0.62			1205	Safonova I et al.	2020	Gondwana Research	79	49-69
71	17081902	CAOB	central Kazakhstan	76.31	46.83	microgabbro		480	480	3.69	13.63	0.16360	0.512773	5	-0.17	2.63		4.66	4.62	1.15	1.15	0.83			666	Safonova I et al.	2020	Gondwana Research	79	49-69
72	H2-14	CAOB	central Kazakhstan	76.29	46.85	aphyric basalt		470	470	3.75	10.93	0.20720	0.513069	8	0.05	8.41		7.78	7.78	1.89	1.89	0.57			1424	Safonova I et al.	2020	Gondwana Research	79	49-69
73	H2-16/1	CAOB	central Kazakhstan	76.30	46.85	gabbro		470	470	0.51	1.53	0.20000	0.513040	10	0.02	7.84		7.64	7.64	1.22	1.22	0.58			753	Safonova I et al.	2020	Gondwana Research	79	49-69
74	ИХ-14	CAOB	central Kazakhstan	76.37	46.81	microgabbro		470	470	0.45	0.97	0.27680	0.513355	14	0.41	13.99		9.18	8.88	0.50	0.50	0.45			26	Safonova I et al.	2020	Gondwana Research	79	49-69
75	JD-6/1	CAOB	central Kazakhstan	76.05	46.95	microgabbro		470	470	0.34	1.24	0.16510	0.512789	19	-0.16	2.95		4.84	4.51	1.13	1.13	0.81			662	Safonova I et al.	2020	Gondwana Research	79	49-69
76	Ch-07-08	CAOB	central Kazakhstan	81.18	49.60	diorite		500	500	3.36	17.36	0.13090	0.512630	7	-0.33	-0.16		4.04	3.10	0.96	0.96	0.90			457	Safonova I et al.	2020	Gondwana Research	79	49-69
77	Ch-08-08	CAOB	Russia	81.19	49.60	Basalt		388	388	1.00	2.5	0.40000	0.512938	5	1.03	5.85	3.80		3.80	1.48	-0.17	1.48			1096	Safonova I et al.	2018	Gondwana Research	59	59-179
78	Ch-02-12	CAOB	Russia	81.15	49.53	Dolerite		388	388	6.20	25.7	0.24125	0.512922	6	0.23	5.54	7.60		7.60	0.87	-1.27	0.87			480	Safonova I et al.	2018	Gondwana Research	59	59-179
79	Ch-10-13	CAOB	Russia	81.15	49.53	Pl-porphyric andesite		388	388	4.60	18.9	0.24339	0.512900	4	0.24	5.11	7.80		7.80	0.91	-1.29	0.91			524	Safonova I et al.	2018	Gondwana Research	59	59-179
80	C-5d-08	CAOB	Russia	81.78	49.26	Gabbro-diorite		418	418	1.50	7.7	0.19481	0.512904	4	-0.01	5.19	3.50		3.50	1.98	1.98	0.72			1560	Safonova I et al.	2018	Gondwana Research	59	59-179
81	C-7v-08	CAOB	Russia	81.77	49.26	Microgabbro/basalt		404	404	2.10	6.8	0.30882	0.512768	3	0.57	2.54	3.40		3.40	1.42	-0.62	1.42			1015	Safonova I et al.	2018	Gondwana Research	59	59-179
82	C-21v-08	CAOB	Russia	82.02	49.12	Andesibasalt/gabbro-diorite		378	378	2.80	10.3	0.27184	0.512778	5	0.38	2.73	7.30		7.30	1.21	-0.98	1.21			828	Safonova I et al.	2018	Gondwana Research	59	59-179
83	Ch-44-09	CAOB	Russia	81.97	49.05	Microgabbro/basalt		341	341	3.40	15.8	0.21519	0.512934	3	0.09	5.77	6.60		6.60	0.74	-23.95	0.74			404	Safonova I et al.	2018	Gondwana Research	59	59-179
84	XII04-12	CAOB	Eastern Tianshan	85.25	42.333	Lherzolite		274	274	0.622	2.79	0.13478004	0.512952		-0.31	6.13	8.29	8.29	8.29	0.38	0.38	0.36			109	Qian ZZ et al.	2012	ology (In Chinese with	45(4)	145-154
85	XII04-13	CAOB	Eastern Tianshan	85.25	42.333	Lherzolite		274	274	0.891	3.33	0.16176065	0.512961		-0.18	6.30	7.53	7.52	7.52	0.56	0.56	0.43			281	Qian ZZ et al.	2012	ology (In Chinese with	45(4)	145-154
86	XII04-14	CAOB	Eastern Tianshan	85.25	42.333	Lherzolite		274	274	0.759	3.23	0.14206224	0.512965		-0.28	6.38	8.29	8.29	8.29	0.39	0.39	0.36			120	Qian ZZ et al.	2012	ology (In Chinese with	45(4)	145-154
87	IID12/1	CAOB	Eastern Tianshan	85.25	42.333	Wehrlite		274	274	2.04	6.88	0.17925907	0.512919		-0.09	5.48	7.80	6.09	6.09	1.02	1.02	0.54			748	Qian ZZ et al.	2012	ology (In Chinese with	45(4)	145-154
88	IID10/1	CAOB	Eastern Tianshan	85.25	42.333	Gabbronorite		274	274	2.23	9.08	0.14847674	0.512889		-0.25	4.90	6.60	6.58	6.58	0.61	0.61	0.50			337	Qian ZZ et al.	2012	ology (In Chinese with	45(4)	145-154
89	IID11/1	CAOB	Eastern Tianshan	85.25	42.333	Gabbronorite		274	274	3.03	12.5	0.14654534	0.512889		-0.25	4.90	6.70	6.65	6.65	0.59	0.59	0.50			319	Qian ZZ et al.	2012	ology (In Chinese with	45(4)	145-154
90	XII04-1	CAOB	Eastern Tianshan	85.25	42.333	gabbro		274	274	1.37	4.7	0.17622281	0.512982		-0.10	6.71	7.46	7.43	7.43	0.68	0.68	0.43			410	Qian ZZ et al.	2012	ology (In Chinese with	45(4)	145-154

91	XII04-3	CAOB	Eastern Tianshan	85.25	42.333	gabbro		274	274	1.38	4.6	0.181368	0.512987		-0.08	6.81	7.32	7.34	7.34	0.77	0.77	0.44			495	Qian ZZ et al.	2012	ology (In Chinese with	45(4)	145-154
92	XII04-6	CAOB	Eastern Tianshan	85.25	42.333	gabbro		274	274	1.04	3.1	0.20282013	0.51299		0.03	6.87	7.66	6.65	6.65	2.23	2.23	0.49			1958	Qian ZZ et al.	2012	ology (In Chinese with	45(4)	145-154
93	XII04-8	CAOB	Eastern Tianshan	85.25	42.333	gabbro		274	274	1.34	4.22	0.19196929	0.513003		-0.02	7.12	7.28	7.29	7.29	1.03	1.03	0.45			757	Qian ZZ et al.	2012	ology (In Chinese with	45(4)	145-154
94	2012SM-315	CAOB	Transbaikalia and mongoli	102.717	42.783	Basalt		326	326	3.683	16.18	0.1378	0.512761		-0.30	2.40	4.85	4.85	2.40	0.78	0.78	0.69	781.66	687	456	Our unpublished data				
95	2012SM-316	CAOB	Transbaikalia and mongoli	102.717	42.783	Basalt		326	326	3.763	16.29	0.1398	0.512774		-0.29	2.65	5.02	5.02	2.65	0.78	0.78	0.67	776.00	674	450	Our unpublished data				
96	2012SM-317	CAOB	Transbaikalia and mongoli	102.717	42.783	Basalt		326	326	3.793	16.59	0.1384	0.512747		-0.30	2.13	4.55	4.55	2.13	0.82	0.82	0.71	816.16	712	490	Our unpublished data				
97	2012SM-331	CAOB	Transbaikalia and mongoli	102.267	42.767	Basalt		326	326	4.815	23.12	0.1261	0.512691		-0.36	1.03	3.97	3.97	1.03	0.80	0.80	0.76	799.09	759	473	Our unpublished data				
98	2012SM-332	CAOB	Transbaikalia and mongoli	102.267	42.767	Basalt		326	326	4.323	18.57	0.1409	0.512701		-0.28	1.23	3.55	3.55	1.23	0.94	0.94	0.79	940.16	793	614	Our unpublished data				
99	MO-122	CAOB	Transbaikalia and mongoli	106.3	44.967	Basalt		208	208	8.6	43.4	0.12	0.512514		-0.39	-2.42	-0.38	-0.38	-2.42	1.03	1.03	1.02	1034.36	1017	826	Our unpublished data				
100	MO-130	CAOB	Transbaikalia and mongoli	106.3	44.967	Basalt		208	208	7.9	40.6	0.1175	0.512492		-0.40	-2.85	-0.75	-0.75	-2.85	1.04	1.04	1.05	1042.30	1047	834	Our unpublished data				
101	MO-137	CAOB	Transbaikalia and mongoli	106.3	44.967	Basalt		208	208	7.8	39.4	0.1206	0.512496		-0.39	-2.77	-0.75	-0.75	-2.77	1.07	1.07	1.05	1070.36	1047	862	Our unpublished data				
102	MO-164	CAOB	Transbaikalia and mongoli	106.3	44.967	Basalt		208	208	9.7	32.6	0.1793	0.512486		-0.09	-2.97	-2.50	-2.50	-2.97	2.92	2.92	1.18	2923.30	1184	2715	Our unpublished data				
103	MO-165	CAOB	Transbaikalia and mongoli	106.3	44.967	Basalt		208	208	5.6	28.8	0.1175	0.512472		-0.40	-3.24	-1.14	-1.14	-3.24	1.07	1.07	1.08	1073.87	1079	866	Our unpublished data				
104	MO-166	CAOB	Transbaikalia and mongoli	106.3	44.967	Basalt		208	208	5.6	28.5	0.1195	0.512473		-0.39	-3.22	-1.17	-1.17	-3.22	1.09	1.09	1.08	1094.97	1081	887	Our unpublished data				
105	492	CAOB	Transbaikalia and mongoli	90.0333	69.167	Basalts		251	251			0.13107757	0.51252		-0.33	-2.30	-0.20	-0.20	-2.30	1.16	1.16	1.04	1161.49	1037	910	Our unpublished data				
106	530/12	CAOB	Transbaikalia and mongoli	90.05	69.167	Picritic basalts		251	251			0.13370499	0.51211933		-0.32	-10.12	-8.10	-8.10	-10.12	1.96	1.96	1.68	1957.47	1678	1706	Our unpublished data				
107	530/15	CAOB	Transbaikalia and mongoli	90.05	69.167	Picritic basalts		251	251			0.14953122	0.51212481		-0.24	-10.01	-8.50	-8.50	-10.01	2.42	2.42	1.71	2423.57	1708	2173	Our unpublished data				
108	CY-32	CAOB	Transbaikalia and mongoli	90.05	69.167	Picritic basalts		251	251			0.17374097	0.51253366		-0.12	-2.04	-1.30	-1.30	-2.04	2.34	2.34	1.12	2340.45	1124	2089	Our unpublished data				
109	CY-33	CAOB	Transbaikalia and mongoli	90.05	69.167	Picritic basalts		251	251			0.17003117	0.51262497		-0.14	-0.25	0.60	0.60	-0.25	1.83	1.83	0.97	1827.41	970	1576	Our unpublished data				
110	CY-38	CAOB	Transbaikalia and mongoli	90.05	69.167	Picritic basalts		251	251			0.17963133	0.51263048		-0.09	-0.15	0.40	0.40	-0.15	2.31	2.31	0.98	2314.08	986	2063	Our unpublished data				
111	CY-50	CAOB	Transbaikalia and mongoli	90.05	69.167	Picrites		251	251			0.16071114	0.51291725		-0.18	5.45	6.60	6.60	5.45	0.67	0.67	0.48	670.16	483	419	Our unpublished data				
112	xc 51.131.2	CAOB	Transbaikalia and mongoli	90.0667	69.183	Picrites		251	251			0.16395135	0.51286618		-0.17	4.45	5.50	5.50	4.45	0.87	0.87	0.57	869.86	573	619	Our unpublished data				
113	xc 51/129	CAOB	Transbaikalia and mongoli	90.0667	69.183	Picrites		251	251			0.16614453	0.51282877		-0.16	3.72	4.70	4.70	3.72	1.03	1.03	0.64	1029.38	638	778	Our unpublished data				
114	xc 51.108.3	CAOB	Transbaikalia and mongoli	90.0667	69.183	Picrites		251	251			0.16159395	0.51279054		-0.18	2.98	4.10	4.10	2.98	1.05	1.05	0.69	1051.21	687	800	Our unpublished data				
115	XC59/1144	CAOB	Transbaikalia and mongoli	90.0667	69.183	Picritic basalts		251	251			0.15865093	0.51262679		-0.19	-0.22	1.00	1.00	-0.22	1.45	1.45	0.94	1446.41	939	1195	Our unpublished data				
116	4032	CAOB	Transbaikalia and mongoli	90.0667	69.183	Basalts		251	251			0.16521837	0.51275548		-0.16	2.29	3.30	3.30	2.29	1.24	1.24	0.75	1239.23	752	988	Our unpublished data				
117	4002/14	CAOB	Transbaikalia and mongoli	90.0667	69.183	Basalts		251	251			0.16781026	0.51268284		-0.15	0.87	1.80	1.80	0.87	1.55	1.55	0.87	1548.71	873	1298	Our unpublished data				
118	4002/19	CAOB	Transbaikalia and mongoli	90.0667	69.183	Basalts		251	251			0.17868698	0.5127981		-0.09	3.12	3.70	3.70	3.12	1.53	1.53	0.72	1529.11	719	1278	Our unpublished data				
119	530/11	CAOB	Transbaikalia and mongoli	90.0667	69.2	Picritic basalts		251	251	1.45	5.01	0.1751	0.512181		-0.11	-8.91	-8.22	-8.22	-8.91	1.68	3.79	1.68	3791.09	1679	1426	Our unpublished data				
120	530/13	CAOB	Transbaikalia and mongoli	90.0667	69.2	Picritic basalts		251	251	1.83	7.75	0.1426	0.512112		-0.28	-10.26	-8.53	-8.53	-10.26	2.22	2.22	1.71	2216.15	1711	1965	Our unpublished data				
121	530/12	CAOB	Transbaikalia and mongoli	90.0667	69.2	Picritic basalts		251	251	2.22	10.13	0.1324	0.512089		-0.33	-10.71	-8.65	-8.65	-10.71	1.98	1.98	1.72	1982.57	1722	1732	Our unpublished data				
122	530/5a	CAOB	Transbaikalia and mongoli	90.0667	69.2	Picritic basalts		251	251	3.06	13.9	0.1331	0.512125		-0.32	-10.01	-7.97	-7.97	-10.01	1.93	1.93	1.67	1932.25	1667	1681	Our unpublished data				
123	xc51-130	CAOB	Transbaikalia and mongoli	90.0833	69.2	Picrites		251	251	1.82	6.57	0.1672	0.5128		-0.15	3.16	4.10	4.10	3.16	1.15	1.15	0.69	1146.59	686	896	Our unpublished data				
124	cy-50	CAOB	Transbaikalia and mongoli	90.0833	69.2	Picrites		251	251	3.52	12.56	0.1697	0.512865		-0.14	4.43	5.29	5.29	4.43	0.99	0.99	0.59	987.21	590	736	Our unpublished data				
125	4283	CAOB	Transbaikalia and mongoli	90.0833	69.2	Picrites		251	251	2.8	10.25	0.165	0.512783		-0.16	2.83	3.84	3.84	2.83	1.15	1.15	0.71	1147.96	708	897	Our unpublished data				
126	4270/13	CAOB	Transbaikalia and mongoli	90.0833	69.2	Picrites		251	251	4.53	16.21	0.1691	0.512889		-0.14	4.90	5.78	5.78	4.90	0.89	0.89	0.55	892.20	550	641	Our unpublished data				
127	CY-31	CAOB	Transbaikalia and mongoli	90.0833	69.2	Picritic basalts		251	251	0.79	2.74	0.1731	0.51254		-0.12	-1.91	-1.16	-1.16	-1.91	2.28	2.28	1.11	2280.26	1112	2029	Our unpublished data				
128	CY-36	CAOB	Transbaikalia and mongoli	90.0833	69.2	Picritic basalts		251	251	0.83	2.77	0.1803	0.51254		-0.08	-1.91	-1.39	-1.39	-1.91	2.77	2.77	1.13	2767.39	1129	2516	Our unpublished data				
129	HB01	CAOB		86	46.758	Basalt		340	340	3.1	17.8	0.104805	0.512811		-0.47	3.37	7.36	7.36	3.37	0.48	0.48	0.49			135	Geng YS et al.	2011	nal of Asian Earth Scie	42 (5)	854-866
130	HB06	CAOB		86	46.758	Basalt		340	340	4.47	24.1	0.112423	0.512849		-0.43	4.12	7.77	7.77	4.12	0.45	0.45	0.46			114	Geng YS et al.	2011	nal of Asian Earth Scie	42 (5)	854-866
131	BT-2	CAOB	Tianshan	86.5	42.8	basic lava		345	345	4.54	17.19	0.15966855	0.512866		-0.19	4.45	6.08	6.08	4.45	0.80	0.80	0.60			457	Xia LQ et al.	2008	nal of Asian Earth Scie	31 (4-6)	357-378
132	Bb-142-3	CAOB	Tianshan	86.5	42.8	basic lava		345	345	5.75	19.96	0.17415932	0.512959		-0.11	6.26	7.25	7.25	6.26	0.74	0.74	0.51			392	Xia LQ et al.	2008	nal of Asian Earth Scie	31 (4-6)	357-378
133	Bb-142-9	CAOB	Tianshan	86.5	42.8	basic lava		345	345	2.95	9.04	0.19728451	0.512961		0.00	6.30	6.28	6.28	6.30	1.75	1.75	0.58			1405	Xia LQ et al.	2008	nal of Asian Earth Scie	31 (4-6)	357-378
134	Bb-255-4	CAOB	Tianshan	86.5	42.8	basic lava		345	345	6.53	25.85	0.15271864	0.512741		-0.22	2.01	3.95	3.95	2.01	1.02	1.02	0.78			677	Xia LQ et al.	2008	nal of Asian Earth Scie	31 (4-6)	357-378
135	Bb-255-5	CAOB	Tianshan	86.5	42.8	basic lava		345	345	4.44	15.52	0.17295402	0.512891		-0.12	4.94	5.98	5.98	4.94	0.97	0.97	0.61			624	Xia LQ et al.	2008	nal of Asian Earth Scie	31 (4-6)	357-378
136	Bb-263-4	CAOB	Tianshan	86.5	42.8	basic lava		345	345	6.09	23.91	0.15398454	0.512766		-0.22	2.50	4.38	4.38	2.50	0.98	0.98	0.74			635	Xia LQ et al.	2008	nal of Asian Earth Scie	31 (4-6)	357-378
137	Bb-263-5	CAOB	Tianshan	86.5	42.8	basic lava		345	345	5.39	21.11	0.15436184	0.512754		-0.22	2.26	4.13	4.13	2.26	1.02	1.02	0.76			672	Xia LQ et al.	2008	nal of Asian Earth Scie	31 (4-6)	357-378
138	05T-7	CAOB	Tianshan	82.1	43.2	basic lava		345	345	4.539	15.63	0.17556608	0.513021		-0.11	7.47	8.40	8.40	7.47	0.52	0.52	0.41			171	Xia LQ et al.	2008	nal of Asian Earth Scie	31 (4-6)	357-378
139	05T-8	CAOB	Tianshan	82.1	43.2	basic lava		345	345	9.04	32.92	0.16601526	0.512912		-0.16	5.34	6.70	6.70	5.34	0.76	0.76	0.55			416	Xia LQ et al.	2008	nal of Asian Earth Scie	31 (4-6)	357-378
140	04T-37	CAOB	Tianshan	82.1	43.2	basic lava		345	345	6.09	25.1	0.14668408	0.512847		-0.25	4.08	6.28	6.28	4.08	0.69	0.69	0.59			345	Xia LQ et al.	2008	nal of Asian Earth Scie	31 (4-6)	357-378
141	04T-54	CAOB	Tianshan	82.1	43.2	basic lava		345	345	3.367	15.07	0.13507323	0.512818		-0.31	3.51	6.22	6.22	3.51	0.64	0.64	0.59			299	Xia LQ et al.	2008	nal of Asian Earth Scie	31 (4-6)	357-378
142	05T-1	CAOB	Tianshan	82.1	43.2	basic lava		345	345	5.773	25.56	0.13654636	0.512716		-0.31	1.52	4.17	4.17	1.52	0.86	0.86	0.76			513	Xia LQ et al.	2008	nal of Asian Earth Scie	31 (4-6)	357-378
143	05T-2	CAOB	Tianshan	82.1	43.2	basic lava		345	345	4.517	19.58	0.13946872	0.512723		-0.29	1.66	4.18	4.18	1.66	0.88	0.88	0.76			532	Xia LQ et al.	2008	nal of Asian Earth Scie	31 (4-6)	357-378
144	05T-3	CAOB	Tianshan	82.1	43.2	basic lava		345	345	5.213	21.99	0.14331838	0.512792		-0.27	3.00	5.35	5.35	3.00	0.78	0.78	0.66			431	Xia LQ et al.	2008	nal of Asian Earth Scie	31 (4-6)	357-378
145	05T-4	CAOB	Tianshan	82.1	43.2	basic lava		345	345	2.598	9.281	0.16923251	0.512841		-0.14	3.96	5.17	5.17	3.96	1.06	1.06	0.68			714	Xia LQ et al.	2008	nal of Asian Earth Scie	31 (4-6)	357-378
146	Bb-111	CAOB	Tianshan	93.1	42.2	basic lava		310	310	4.71	18.5	0.15391771	0.512837		-0.22	3.88	5.57	5.57	3.88	0.80	0.80	0.62			488	Xia LQ et al.	2008	nal of Asian Earth Scie	31 (4-6)	357-378
147	Bb-128	CAOB	Tianshan	93.1	42.2	basic lava		310	310	4.07	16.21	0.15179267	0.512831		-0.23	3.76	5.54	5.54	3.76	0.79	0.79	0.62			476	Xia LQ et al.	2008	nal of Asian Earth Scie	31 (4-6)	357-378
148	TB-33	CAOB	Tianshan	93.1	42.2	basic lava		345	345	4.13	18.37	0.13591904	0.51295		-0.31	6.09	8.76	8.76	6.09	0.39	0.39	0.38			48	Xia LQ et al.	2008	nal of Asian Earth Scie	31 (4-6)	357-378
149	Bb-158	CAOB	Tianshan	93.1	42.2	basic lava		325	325	5.63	24.45	0.13920952	0.512828		-0.29	3.71	6.09	6.09	3.71	0.66	0.66	0.59			335	Xia LQ et al.	2008	nal of Asian Earth Scie	31 (4-6)	357-378
150	Bb-165	CAOB	Tianshan	93.1	42.2	basic lava		325	325	6.16	28.01	0.13295572	0.512791		-0.32	2.98	5.63	5.63	2.98	0.68	0.68	0.62			353	Xia LQ et al.	2008	nal of Asian Earth Scie	31 (4-6)	357-378
151	XSX01	CAOB	Tianshan	94.5	42.317	gabbro		283	283	1.8	8.73	0.125	0.512862		-0.36	4.37	7.00	6.96	4.37	0.50	0.50	0.48			213	Li DD et al.	2012	view (In Chinese with E	58(6)	1145-1160
152	XSX02	CAOB	Tianshan	94.5	42.317	Pyroxene peridotite		283	283	0.24	1.15	0.128	0.5127		-0.35	1.21	3.70	3.69	1.21	0.80	0.80	0.75			518	Li DD et al.	2012	view (In Chinese with E	58(6)	1145-1160
153	XSX03	CAOB	Tianshan	94.5	42.317	Pyroxene peridotite		283	283	0.22	0.98	0.138	0.512601		-0.30	-0.72	1.40	1.40	-0.72	1.10	1.10	0.93			822	Li DD et al.	2012	view (In Chinese with E	58(6)	1145-1160
154	XSX04	CAOB	Tianshan	94.5	42.317	Pyroxene peridotite		283	283	2.06	8.33	0.149	0.512943		-0.24	5.95	7.70	7.67	5.95	0.49	0.49	0.42			205	Li DD et al.	2012	view (In Chinese with E	58(6)	1145-1160
155	XSX05	CAOB	Tianshan	94.5	42.317	gabbro		283	283	3.13	13.4	0.142	0.512897		-0.28	5.05	7.00	7.03	5.05	0.54	0.54	0.47			256	Li DD et al.	2012	view (In Chinese with E	58(6)	1145-1160
156	XSX06	CAOB	Tianshan	94.5	42.317	gabbro		283	283	1.02	4.13	0.149	0.51289		-0.24	4.92	6.60	6.64	4.92	0.61	0.61	0.51			330	Li DD et al.	2012	view (In Chinese with E	58(6)	1145-1160
157	NMQ0I	CAOB	Tianshan	94.5	42.667	gabbro		289	289	1.2	4.2	0.174	0.512877		-0.12	4.66	5.50	5.50	4.66	1.05	1.05	0.60			759	Li DD et al.	2012	view (In Chinese with E	58(6)	1145-1160
158	NMQ02	CAOB	Tianshan	94.5	42.667	gabbro		289	289	3.42	17.5	0.118	0.512783		-0.40	2.83	5.70	5.73	2.83	0.59	0.59	0.59			296	Li DD et al.	2012	view (In Chinese with E	58(6)	1145-1160
159	NMQ03	CAOB	Tianshan	94.5	42.667	gabbro		289	289	1.12	3.4	0.199	0.512918		0.01	5.46	5.40	5.38	5.46	2.39	2.39	0.61			2105	Li DD et al.	2012	view (In Chinese with E	58(6)	1145-1160
160	NMQ04	CAOB	Tianshan	94.5	42.667	gabbro		289	289	0.35	1.7	0.124	0.512754		-0.37	2.26	5.00	4.94	2.26	0.67	0.67	0.65			385	Li DD et al.	2012	view (In Chinese with E	58(6)	1145-1160
161	EHW01	CAOB	Tianshan	94.2	42.133	troctolite		274	274	0.9	4.08	0.133	0.512777		-0.32	2.71	5.00	4.94	2.71	0.71	0.71	0.64			431	Li DD et al.	2012	view (In Chinese with E	58(6)	1145-1160
162	EHW02	CAOB	Tianshan	94.2	42.133	diorite		274	274	5.34	22.4	0.144	0.512794		-0.27	3.04	4.90	4.89	3.04	0.78	0.78	0.64			505	Li DD et al.	2012	view (In Chinese with E	58(6)	1145-1160
163	EHW03	CAOB	Tianshan	94.2	42.133	gabbro		274	274	1.33	3.52	0.227	0.513152		0.15	10.03	8.90	8.97	10.03	0.31	0.02	0.31			35	Li DD et al.	2012	view (In Chinese with E	58(6)	1145-1160
164	EHW04	CAOB	Tianshan	94.2	42.133	gabbro		274	274	1.71	5.43	0.191	0.5129		-0.03	5.11	5.30	5.31	5.11	1.67	1.67	0.61			1401	Li DD et al.	2012	view (In Chinese with E	58(6)	1145-1160
165	HLZ01	CAOB	Tianshan	95.6	42.2	gabbro		309.7	309.7	2.62	13.6	0.116	0.512471		-0.41	-3.26	-0.10	-0.07	-3.26	1.06	1.06	1.07			749	Li DD et al.	2012	view (In Chinese with E	58(6)	1145-1160
166	HLZ02	CAOB	Tianshan	95.6	42.2	Pyroxene peridotite		309.7	309.7	1.79	9.7	0.112	0.512453		-0.43	-3.61	-0.20	-0.26	-3.61	1.04	1.04	1.09			735	Li DD et al.	2012	view (In Chinese with E	58(6)	1145-1160
167	HLZ03	CAOB	Tianshan	95.6	42.2	gabbro		309.7	309.7	3.61	18	0.121	0.512582		-0.38	-1.09	1.90	1.90	-1.09	0.93	0.93	0.91			624	Li DD et al.	2012	view (In Chinese with E	58(6)	1145-1160
168	HLZ04	CAOB	Tianshan	95.6	42.2	gabbro		309.7	309.7	4.13	22.5	0.111	0.512707		-0.44	1.35	4.80	4.73	1.35	0.66	0.66	0.68			348	Li DD et al.	2012	view (In Chinese with E	58(6)	1145-1160
169	HLZ05	CAOB	Tianshan	95.6	42.2	gabbro		309.7	309.7	2.01	10.1	0.121	0.512521		-0.38	-2.28	0.70	0.71	-2.28	1.03	1.03	1.01			724	Li DD et al.	2012	view (In Chinese with E	58(6)	1145-1160
170	04XJ-282	CAOB	Tianshan	84.3083	43.333	trachybasalt		312	312	5.37	23.2	0.14	0.512727		-0.29	1.74	3.99	3.99	1.74	0.88	0.88	0.75			563	Sun L et al.	2008	Gondwana Research	14 (3)	383-394
171	D976B(266–2	CAOB	Southern Mongolia	106.868	43.049	Basalt		362	362	6.46	31.6	0.12359	0.512538		-0.37	-1.95	1.56	1.43	-1.95	1.03	1.03	1.00			673	Dolgopolova A et al.	2013	Lithos	164–167	164–167
172	DTD976B(552	CAOB	Southern Mongolia	106.867	43.049	Basalt		362	362	3.46	14.5	0.14427	0.512814		-0.27	3.43	5.99	5.86	3.43	0.74	0.74	0.63			376	Dolgopolova A et al.	2013	Lithos	164–167	164–167
173	DTB976B(772	CAOB	Southern Mongolia	106.867	43.049	Basalt dyke		362	362	2.8	11.3	0.14981	0.512776		-0.24	2.69	4.99	4.86	2.69	0.89	0.89	0.72			530	Dolgopolova A et al.	2013	Lithos	164–167	164–167
174	76B(1382.5–1	CAOB	Southern Mongolia	106.865	43.049	Basalt		362	362	3.43	12.6	0.16459	0.51293		-0.16	5.70	7.31	7.18	5.70	0.68	0.68	0.53			321	Dolgopolova A et al.	2013	Lithos	164–167	164–167
175	IDO1A(590–5	CAOB	Southern Mongolia	106.597	42.954	Basalt		362	362	5.57	23.8	0.14149	0.512784		-0.28	2.85	5.53	5.40	2.85	0.77	0.77	0.67			411	Dolgopolova A et al.	2013	Lithos	164–167	164–167
176	DTD1220(522	CAOB	Southern Mongolia	106.849	43.04	Basalt		345	345	2.65	10.2	0.15707	0.512801		-0.20	3.18	5.06	4.92	3.18	0.94	0.94	0.70			594	Dolgopolova A et al.	2013	Lithos	164–167	164–167
177	OTD318(223	CAOB	Southern Mongolia	106.835	43.002	Basalt		362	362	3.2	11.2	0.17274	0.51283		-0.12	3.75	4.98	4.85	3.75	1.19	1.19	0.72			828	Dolgopolova A et al.	2013	Lithos	164–167	164–167
178	SUM-19	CAOB	WNCC	108.09	41.529	gabbro	eiqigetao plut	269	269	3.67	14.24	0.15651	0.511815	9	-0.20	-16.05	-14.54	-14.67	-16.05	2.22	3.53	2.22			1947	Zhao L et al.	2011	Acta Petrologica Sinica	27(10)	3071-3082
179	SUM-22	CAOB	WNCC	108.09	41.529	gabbro	eiqigetao plut	269	269	3.57	15.36	0.140614	0.511822	12	-0.29	-15.92	-13.85	-13.99	-15.92	2.75	2.75	2.17			2484	Zhao L et al.	2011	Acta Petrologica Sinica	27(10)	3071-3082
180	SUM-36	CAOB	WNCC	108.09	41.529	gabbro	eiqigetao plut	269	269	2.44	10.94	0.135113	0.511827	12	-0.31	-15.82	-13.57	-13.71	-15.82	2.55	2.55	2.14			2284	Zhao L et al.	2011	Acta Petrologica Sinica	27(10)	3071-3082
181	SUM-48	CAOB	WNCC	108.09	41.529	gabbro	eiqigetao plut	269	269	3.94	17.05	0.139746	0.511834	13	-0.29	-15.68	-13.59	-13.73	-15.68	2.70	2.70	2.15			2428	Zhao L et al.	2011	Acta Petrologica Sinica	27(10)	3071-3082
182	SUM-29	CAOB	WNCC	108.09	41.529	gabbro	eiqigetao plut	269	269	1.18	6.21	0.114903	0.511831	11	-0.42	-15.74	-12.79	-12.93	-15.74	2.03	2.03	2.09			1759	Zhao L et al.	2011	Acta Petrologica Sinica	27(10)	3071-3082
183	12WLT-19.1	CAOB	Langshan	107.009	41.634	gabbro	Langshan	327.9	327.9	2.74	7.8	0.2124	0.512571	0	0.08	-1.31	-1.84	-1.96	-1.31	1.03	56.33	1.03			703	Wang ZZ et al.	2015	nal of Asian Earth Scie	97	337-351
184	12WLT-19.2	CAOB	Langshan	107.009	41.634	gabbro	Langshan	327.9	327.9	2.59	9.9	0.1582	0.512536	0	-0.20	-1.99	-0.25	-0.38	-1.99	1.68	1.68	1.11			1354	Wang ZZ et al.	2015	nal of Asian Earth Scie	97	337-351

185	NM07-3	CAOB	WNCC	110.934	41.119	Metagabbros	ıyang-Wuchu	2511	600	5.35	25	0.1293	0.511517	11	-0.34	-21.87	-0.26	-16.71	-21.87	2.93	2.93	2.66			2330	Zhang XH et al.	2014	Precambrian Research	255(2)	563-582
186	NM07-4	CAOB	WNCC	110.934	41.119	Metagabbros	ıyang-Wuchu	2511	600	5.22	22.3	0.1415	0.511758	10	-0.28	-17.17	0.51	-12.94	-17.17	2.92	2.92	2.35			2320	Zhang XH et al.	2014	Precambrian Research	255(2)	563-582
187	NM07-10	CAOB	WNCC	110.934	41.119	Metagabbros	ıyang-Wuchu	2511	600	3.32	14	0.1432	0.511804	13	-0.27	-16.27	0.87	-12.17	-16.27	2.89	2.89	2.29			2292	Zhang XH et al.	2014	Precambrian Research	255(2)	563-582
188	NM07-17	CAOB	WNCC	110.918	41.119	Metagabbros	ıyang-Wuchu	2511	600	7.74	39.2	0.1194	0.511422	11	-0.39	-23.72	1.07	-17.80	-23.72	2.78	2.78	2.75			2177	Zhang XH et al.	2014	Precambrian Research	255(2)	563-582
189	NM07-19	CAOB	WNCC	110.918	41.119	Metagabbros	ıyang-Wuchu	2511	600	15.7	69.3	0.1369	0.51173	13	-0.30	-17.71	1.45	-13.13	-17.71	2.80	2.80	2.37			2201	Zhang XH et al.	2014	Precambrian Research	255(2)	563-582
190	LS058	CAOB	Altai	105.512	40.228	gabbroic enclave	yamatu	270	270	7.2060651	36.480227	0.11942127	0.512045	11	-0.39	-11.57	-8.76	-8.91	-11.57	1.78	1.78	1.76			1512	Zhang JJ et al.	2016	Journal of Petrology	57(5)	933–980
191	LS055	CAOB	Altai	105.512	40.228	gabbroic enclave	yamatu	270	270	7.1964049	37.238519	0.11683265	0.512032	10	-0.41	-11.82	-8.93	-9.07	-11.82	1.75	1.75	1.77			1485	Zhang JJ et al.	2016	Journal of Petrology	57(5)	933–980
192	LS056	CAOB	Altai	105.512	40.228	gabbroic enclave	yamatu	270	270	7.1996701	37.361603	0.11650059	0.512047	11	-0.41	-11.53	-8.62	-8.77	-11.53	1.73	1.73	1.75			1455	Zhang JJ et al.	2016	Journal of Petrology	57(5)	933–980
193	LS062	CAOB	Altai	105.512	40.228	gabbroic enclave	yamatu	270	270	7.432	38.08	0.118	0.512071	11	-0.40	-11.06	-8.21	-8.35	-11.06	1.71	1.71	1.71			1444	Zhang JJ et al.	2016	Journal of Petrology	57(5)	933–980
194	LS063	CAOB	Altai	105.512	40.228	gabbroic enclave	yamatu	270	270	7.2823776	40.443972	0.10885802	0.512021	9	-0.45	-12.04	-8.87	-9.01	-12.04	1.64	1.64	1.77			1368	Zhang JJ et al.	2016	Journal of Petrology	57(5)	933–980
195	11LS16	CAOB	Altai	105.354	40.184	Gabbro		276	276	1.993	9.08	0.1327	0.512134	7	-0.33	-9.83	-7.44	-7.58	-9.83	1.91	1.91	1.66			1630	Our unpublished data				
196	11LS17	CAOB	Altai	105.354	40.184	Gabbro		276	276	1.49	6.38	0.1411	0.51217	9	-0.28	-9.13	-7.03	-7.17	-9.13	2.05	2.05	1.62			1774	Our unpublished data				
197	11LS106	CAOB	Altai	104.232	40.942	Gabbro		264	264	1.03	3.45	0.1807	0.512832	10	-0.08	3.78	4.46	4.32	3.78	1.47	1.47	0.68			1202	Our unpublished data				
198	11LS107	CAOB	Altai	104.232	40.942	Gabbro		264	264	0.951	3.35	0.1718	0.512829	7	-0.13	3.73	4.70	4.56	3.73	1.17	1.17	0.66			903	Our unpublished data				
199	09AL82	CAOB	Altai			Gabbro		278	278	2.69	8.92	0.1822	0.512103	9	-0.07	-10.44	-9.79	-9.92	-9.33	1.83	5.00	1.83			1551	Dan W et al.	2014	Lithos	204	144-158
200	09AL83	CAOB	Altai			Gabbro		278	278	2.87	10.4	0.1674	0.512096	10	-0.15	-10.57	-9.40	-9.53	-8.33	1.81	3.44	1.81			1529	Dan W et al.	2014	Lithos	204	144-158
201	KR16	CAOB	Gorny Altai	87.8	50.23	Basalt	SW.Kurai	600	600	6.131	23.55	0.15712653	0.512826		-0.20	3.67		6.70	6.70	0.87	0.87	0.76			273	Utsunomiya A et al.	2009	Lithos	113	437–453
202	KR87	CAOB	Gorny Altai	87.8	50.23	Basalt	W.Kurai	600	600	5.832	17.77	0.19809016	0.513057		0.01	8.17		8.07	8.07	0.91	0.91	0.65			308	Utsunomiya A et al.	2009	Lithos	113	437–453
203	99CU4	CAOB	Gorny Altai	88.25	50	Basalt	Chagan-Uzur	600	600	3.5	13.09	0.16137536	0.512823		-0.18	3.61		6.31	6.31	0.95	0.95	0.79			353	Utsunomiya A et al.	2009	Lithos	113	437–453
204	AT12	CAOB	Gorny Altai	86	51.5	Basalt	Teletskoy	600	600	4.26	16.08	0.1598937	0.512813		-0.19	3.41		6.23	6.23	0.95	0.95	0.80			355	Utsunomiya A et al.	2009	Lithos	113	437–453
205	98KR400	CAOB	Gorny Altai	87.8	50.23	Basalt	W.Kurai	600	600	5.38	16.11	0.20156786	0.513074		0.02	8.51		8.13	8.13	0.95	0.95	0.64			355	Utsunomiya A et al.	2009	Lithos	113	437–453
206	AT49	CAOB	Gorny Altai	86	51.5	Basalt	Gorno-Altaisl	600	600	7.13	29.42	0.14626455	0.512661		-0.26	0.45		4.31	4.31	1.10	1.10	0.96			505	Utsunomiya A et al.	2009	Lithos	113	437–453
207	KR82	CAOB	Gorny Altai	87.8	50.23	Basalt	W.Kurai	600	600	5.896	17.53	0.20300643	0.513071		0.03	8.45		7.96	7.96	1.13	1.13	0.66			525	Utsunomiya A et al.	2009	Lithos	113	437–453
208	KR90	CAOB	Gorny Altai	87.8	50.23	Basalt	W.Kurai	600	600	4.237	12.43	0.20574217	0.513088		0.05	8.78		8.09	8.09	1.19	1.19	0.65			587	Utsunomiya A et al.	2009	Lithos	113	437–453
209	KR81	CAOB	Gorny Altai	87.8	50.23	Basalt	W.Kurai	600	600	4.194	12.22	0.20715443	0.513098		0.05	8.97		8.17	8.17	1.21	1.21	0.64			610	Utsunomiya A et al.	2009	Lithos	113	437–453
210	U51	CAOB	Gorny Altai	86	51.5	Basalt	Ust-Syoma	600	600	4.73	17.45	0.16359361	0.512743		-0.17	2.05		4.58	4.58	1.24	1.24	0.93			637	Utsunomiya A et al.	2009	Lithos	113	437–453
211	KR83	CAOB	Gorny Altai	87.8	50.23	Dolerite	W.Kurai	600	600	4.875	14.64	0.20098563	0.513044		0.02	7.92		7.59	7.59	1.27	1.27	0.69			669	Utsunomiya A et al.	2009	Lithos	113	437–453
212	01CU12	CAOB	Gorny Altai	88.25	50	Basalt	Chagan-Uzur	600	600	5.87	21.32	0.1661695	0.512741		-0.16	2.01		4.35	4.35	1.31	1.31	0.95			710	Utsunomiya A et al.	2009	Lithos	113	437–453
213	U48	CAOB	Gorny Altai	86	51.5	Basalt	Ust-Syoma	600	600	4.03	14.45	0.16832133	0.512756		-0.14	2.30		4.47	4.47	1.32	1.32	0.94			722	Utsunomiya A et al.	2009	Lithos	113	437–453
214	KR80	CAOB	Gorny Altai	87.8	50.23	Basalt	W.Kurai	600	600	5.08	14.93	0.20537069	0.513074		0.04	8.51		7.84	7.84	1.39	1.39	0.67			789	Utsunomiya A et al.	2009	Lithos	113	437–453
215	99KR17	CAOB	Gorny Altai	87.8	50.23	Basalt	W.Kurai	600	600	4.29	14.08	0.18389448	0.512874		-0.07	4.60		5.58	5.58	1.41	1.41	0.85			809	Utsunomiya A et al.	2009	Lithos	113	437–453
216	99KR218	CAOB	Gorny Altai	87.8	50.23	Dolerite	W.Kurai	600	600	4.29	14.32	0.18080916	0.512797		-0.08	3.10		4.32	4.32	1.63	1.63	0.95			1032	Utsunomiya A et al.	2009	Lithos	113	437–453
217	98KR48	CAOB	Gorny Altai	87.8	50.23	Basalt	SW.Kurai	600	600	4.57	15.14	0.1821784	0.512801		-0.07	3.18		4.29	4.29	1.68	1.68	0.96			1084	Utsunomiya A et al.	2009	Lithos	113	437–453
218	AT74	CAOB	Gorny Altai	86	51.5	Dolerite	Gorno-Altaisl	600	600	2.49	7.47	0.20118854	0.512976		0.02	6.59		6.25	6.40	2.11	2.11	0.80			1512	Utsunomiya A et al.	2009	Lithos	113	437–453
219	99CU23	CAOB	Gorny Altai	88.25	50	Dolerite	Chagan-Uzur	600	600	5.29	15.44	0.20679447	0.513035		0.05	7.74		6.97	7.32	2.53	2.53	0.74			1925	Utsunomiya A et al.	2009	Lithos	113	437–453
220	B009	CAOB	东天山	91.6	43.47	Basalt	Bogeda	329	329	5.016	20.32	0.14898697	0.512878		-0.24	4.68		6.68	4.77	0.64	0.64	0.54			312	Gu LX et al.	2000	Acta Petrologica Sinica	16（3）	305-316
221	B014	CAOB	东天山	91.6	43.47	Basalt	Bogeda	329	329	6.829	27.94	0.14751711	0.512851		-0.25	4.15		6.22	4.16	0.69	0.69	0.58			360	Gu LX et al.	2000	Acta Petrologica Sinica	16（3）	305-316
222	JB-25	CAOB	NE China	128.94	43.88	alkali basalts	Jingpohu	14.15	150	5.26	22.12	0.14352311	0.512944		-0.27	5.97		6.99	5.97	0.45	0.45	0.37			298	Yan J and Zhao JX	2008	nal of Asian Earth Scie	1-2（33）	106-121
223	JB-7	CAOB	NE China	128.94	43.88	alkali basalts	Jingpohu	0.0049	150	8.37	44.02	0.11475904	0.512849		-0.42	4.12		5.68	4.12	0.46	0.46	0.48			314	Yan J and Zhao JX	2008	nal of Asian Earth Scie	1-2（33）	106-121
224	JB-8	CAOB	NE China	128.94	43.88	alkali basalts	Jingpohu	0.0049	150	9.15	48.81	0.11314182	0.512843		-0.42	4.00		5.60	4.00	0.47	0.47	0.48			316	Yan J and Zhao JX	2008	nal of Asian Earth Scie	1-2（33）	106-121
225	JB-9	CAOB	NE China	128.94	43.88	alkali basalts	Jingpohu	0.0049	150	9.09	48.34	0.11349226	0.512825		-0.42	3.65		5.24	3.80	0.50	0.50	0.51			345	Yan J and Zhao JX	2008	nal of Asian Earth Scie	1-2（33）	106-121
226	JB-5	CAOB	NE China	128.94	43.88	alkali basalts	Jingpohu	0.0049	150	8.01	41.69	0.11596058	0.512832		-0.41	3.78		5.33	3.93	0.50	0.50	0.50			347	Yan J and Zhao JX	2008	nal of Asian Earth Scie	1-2（33）	106-121
227	JB-26	CAOB	NE China	128.94	43.88	alkali basalts	Jingpohu	14.15	150	5.4	22.44	0.14524135	0.512926		-0.26	5.62		6.60	5.63	0.50	0.50	0.40			349	Yan J and Zhao JX	2008	nal of Asian Earth Scie	1-2（33）	106-121
228	JB-27	CAOB	NE China	128.94	43.88	alkali basalts	Jingpohu	14.15	150	5.35	22.12	0.14597825	0.512927		-0.26	5.64		6.61	5.64	0.50	0.50	0.40			353	Yan J and Zhao JX	2008	nal of Asian Earth Scie	1-2（33）	106-121
229	JB-1	CAOB	NE China	128.94	43.88	alkali basalts	Jingpohu	1.305	150	5.51	25.66	0.12959905	0.512804		-0.34	3.24		4.52	3.24	0.63	0.63	0.57			478	Yan J and Zhao JX	2008	nal of Asian Earth Scie	1-2（33）	106-121
230	JB-17	CAOB	NE China	128.94	43.88	alkali basalts	Jingpohu	0.0049	150	6.52	33.34	0.11802714	0.512736		-0.40	1.91		3.42	1.91	0.66	0.66	0.66			510	Yan J and Zhao JX	2008	nal of Asian Earth Scie	1-2（33）	106-121
231	JB-16A	CAOB	NE China	128.94	43.88	alkali basalts	Jingpohu	0.0049	150	6.66	33.89	0.11860491	0.512737		-0.40	1.93		3.43	2.07	0.66	0.66	0.66			513	Yan J and Zhao JX	2008	nal of Asian Earth Scie	1-2（33）	106-121
232	JB-15	CAOB	NE China	128.94	43.88	alkali basalts	Jingpohu	0.0049	150	6.71	34.04	0.11896871	0.512735		-0.40	1.89		3.38	1.89	0.67	0.67	0.66			518	Yan J and Zhao JX	2008	nal of Asian Earth Scie	1-2（33）	106-121
233	JB-24	CAOB	NE China	128.94	43.88	alkali basalts	Jingpohu	14.15	150	6.59	32.59	0.12203991	0.512745		-0.38	2.09		3.52	2.09	0.67	0.67	0.65			524	Yan J and Zhao JX	2008	nal of Asian Earth Scie	1-2（33）	106-121
234	JB-14	CAOB	NE China	128.94	43.88	alkali basalts	Jingpohu	0.0049	150	6.62	33.6	0.11890992	0.512731		-0.40	1.81		3.30	1.81	0.67	0.67	0.67			524	Yan J and Zhao JX	2008	nal of Asian Earth Scie	1-2（33）	106-121
235	JB-20	CAOB	NE China	128.94	43.88	alkali basalts	Jingpohu	0.0049	150	6.63	33.43	0.11969517	0.512732		-0.39	1.83		3.31	1.83	0.68	0.68	0.67			528	Yan J and Zhao JX	2008	nal of Asian Earth Scie	1-2（33）	106-121
236	JB-12	CAOB	NE China	128.94	43.88	alkali basalts	Jingpohu	0.0049	150	6.27	30.96	0.12222676	0.512734		-0.38	1.87		3.30	1.87	0.69	0.69	0.67			544	Yan J and Zhao JX	2008	nal of Asian Earth Scie	1-2（33）	106-121
237	JB-18	CAOB	NE China	128.94	43.88	alkali basalts	Jingpohu	0.0049	150	6.13	30.27	0.12222151	0.512733		-0.38	1.85		3.28	1.85	0.70	0.70	0.67			545	Yan J and Zhao JX	2008	nal of Asian Earth Scie	1-2（33）	106-121
238	JB-23	CAOB	NE China	128.94	43.88	alkali basalts	Jingpohu	0.0049	150	6.38	31.45	0.12243329	0.512732		-0.38	1.83		3.26	1.83	0.70	0.70	0.67			549	Yan J and Zhao JX	2008	nal of Asian Earth Scie	1-2（33）	106-121
239	JB-16B	CAOB	NE China	128.94	43.88	alkali basalts	Jingpohu	0.0049	150	6.7	33.77	0.11974059	0.512714		-0.39	1.48		2.96	1.48	0.71	0.71	0.70			558	Yan J and Zhao JX	2008	nal of Asian Earth Scie	1-2（33）	106-121
240	JB-19	CAOB	NE China	128.94	43.88	alkali basalts	Jingpohu	0.0049	150	6.21	30.29	0.12373461	0.512726		-0.37	1.72		3.11	1.72	0.72	0.72	0.68			569	Yan J and Zhao JX	2008	nal of Asian Earth Scie	1-2（33）	106-121
241	JB-22	CAOB	NE China	128.94	43.88	alkali basalts	Jingpohu	0.0049	150	6.27	30.76	0.12302112	0.512722		-0.37	1.64		3.05	1.64	0.72	0.72	0.69			570	Yan J and Zhao JX	2008	nal of Asian Earth Scie	1-2（33）	106-121
242	JB-11	CAOB	NE China	128.94	43.88	alkali basalts	Jingpohu	0.0049	150	6.16	30.16	0.12326708	0.512715		-0.37	1.50		2.91	1.51	0.73	0.73	0.70			584	Yan J and Zhao JX	2008	nal of Asian Earth Scie	1-2（33）	106-121
243	JB-21	CAOB	NE China	128.94	43.88	alkali basalts	Jingpohu	0.0049	150	6.02	29.08	0.1249392	0.512704		-0.36	1.29		2.66	4.68	0.77	0.77	0.72			616	Yan J and Zhao JX	2008	nal of Asian Earth Scie	1-2（33）	106-121
244	JB-2	CAOB	NE China	128.94	43.88	alkali basalts	Jingpohu	1.305	150	4.82	20.25	0.14365657	0.512771		-0.27	2.59		3.61	5.10	0.83	0.83	0.64			675	Yan J and Zhao JX	2008	nal of Asian Earth Scie	1-2（33）	106-121
245	OYU-5	CAOB	Gorny Altai	89.1	49.7	Basaltic trachyandesite	ıksai Comple	370	370	6.82	37.7	0.10917413	0.51251		-0.44	-2.50		1.64	-2.48	0.93	0.93	0.99			563	Vrublevsky VV et al	2007	Doklady Earth Science	416(1)	1090-1095
246	ZHU-4	CAOB	Gorny Altai	89.1	49.7	rhyolite	ıksai Comple	370	370	5.24	24.4	0.12960219	0.512453		-0.34	-3.61		-0.44	-3.60	1.26	1.26	1.15			892	Vrublevsky VV et al	2007	Doklady Earth Science	416(1)	1090-1095
247	HS2-2	CAOB	NE China	126.17	48.75	potassic basalts	Huoshao	1.305	150	13.9	80	0.10485643	0.512447		-0.47	-3.73		-1.97	-3.71	0.98	0.98	1.10			834	Zhao HB et al.	2003	Chemical Geology	200	189–201
248	LE6-4	CAOB	NE China	126.17	48.75	potassic basalts	Laohei	1.305	150	11	62.1	0.10689808	0.512433		-0.46	-4.00		-2.28	-3.98	1.02	1.02	1.12			873	Zhao HB et al.	2003	Chemical Geology	200	189–201
249	LW5-2	CAOB	NE China	126.17	48.75	potassic basalts	Laohei	1.305	150	12	69.7	0.10389988	0.512411		-0.47	-4.43		-2.65	-4.41	1.03	1.03	1.16			876	Zhao HB et al.	2003	Chemical Geology	200	189–201
250	HN6-1	CAOB	NE China	126.17	48.75	potassic basalts	Huoshao	1.305	150	10.7	59.4	0.10870932	0.512439		-0.45	-3.88		-2.20	1.06	1.03	1.03	1.12			882	Zhao HB et al.	2003	Chemical Geology	200	189–201
251	LS3-1	CAOB	NE China	126.17	48.75	potassic basalts	Laohei	1.305	150	11.8	68.4	0.10410925	0.51238		-0.47	-5.03		-3.26	0.17	1.07	1.07	1.20			921	Zhao HB et al.	2003	Chemical Geology	200	189–201
252	21WLHT-9	CAOB	NE China	121.394	46.335	basalts	Dashizhai	440	440	8.14	42.14	0.11658283	0.512783		-0.41	2.83		7.33	7.33	0.58	0.58	0.58			137	Guo F et al.	2009	SCI. CHINA SER. B	52(7)	940-951
253	21WLHT-12	CAOB	NE China	121.394	46.335	basalts	Dashizhai	440	440	8.08	42.16	0.11566778	0.512753		-0.41	2.24		6.79	6.79	0.62	0.62	0.62			178	Guo F et al.	2009	SCI. CHINA SER. B	52(7)	940-951
254	21WLHT-7	CAOB	NE China	121.394	46.335	basalts	Dashizhai	440	440	8.13	41.38	0.11857745	0.512757		-0.40	2.32		6.71	6.71	0.63	0.63	0.63			190	Guo F et al.	2009	SCI. CHINA SER. B	52(7)	940-951
255	21WLHT-10	CAOB	NE China	121.394	46.335	basalts	Dashizhai	440	440	7.42	39.81	0.11248911	0.512725		-0.43	1.70		6.43	6.43	0.64	0.64	0.65			201	Guo F et al.	2009	SCI. CHINA SER. B	52(7)	940-951
256	21WLHT-15	CAOB	NE China	121.394	46.335	basalts	Dashizhai	440	440	7.95	40.47	0.11855892	0.51274		-0.40	1.99		6.38	4.88	0.66	0.66	0.66			218	Guo F et al.	2009	SCI. CHINA SER. B	52(7)	940-951
257	21WLHT-11	CAOB	NE China	121.394	46.335	basalts	Dashizhai	440	440	8.59	44.41	0.11673696	0.512698		-0.41	1.17		5.66	4.13	0.71	0.71	0.71			271	Guo F et al.	2009	SCI. CHINA SER. B	52(7)	940-951
258	SN07-2	CAOB	NE China	113	44	basalt	Sonidquozi	290	290	6.72	33.3	0.12179701	0.51285		-0.38	4.14		6.91	4.27	0.50	0.50	0.49			208	Zhang XH et al.	2011	ıal of the Geological S	168	525-543
259	BY04-14	CAOB	NE China	113	44	basalt	Sonidquozi	290	290	8.93	42.1	0.12801639	0.5127		-0.35	1.21		3.75	1.24	0.80	0.80	0.75			511	Zhang XH et al.	2011	ıal of the Geological S	168	525-543
260	IM 51A	CAOB	NE China	114	43.33	basalts	Abaga	14.15	150	10.27	52.7	0.11761722	0.512845		-0.40	4.04		5.55	4.08	0.48	0.48	0.49			335	Ho KS et al.	2008	Geochemical Journal	42(4)	339-357
261	IM 44A	CAOB	NE China	115.5	43.33	basalts	Abaga	3.95	150	13	51.9	0.15117897	0.512887		-0.23	4.86		5.73	4.94	0.64	0.64	0.47			492	Ho KS et al.	2008	Geochemical Journal	42(4)	339-357
262	IM 45A	CAOB	NE China	115.5	43.33	basalts	Abaga	3.95	150	11.54	44.6	0.15616528	0.512869		-0.21	4.51		5.28	4.58	0.74	0.74	0.51			595	Ho KS et al.	2008	Geochemical Journal	42(4)	339-357
263	IM 48C	CAOB	NE China	114	43.33	basalts	Abaga	14.15	150	10.97	41.6	0.15915781	0.51288		-0.19	4.72		5.44	4.79	0.76	0.76	0.49			605	Ho KS et al.	2008	Geochemical Journal	42(4)	339-357
264	IM 56A	CAOB	NE China	114	43.33	basalts	Abaga	14.15	150	9.98	37	0.16279575	0.512876		-0.17	4.64		5.29	4.70	0.82	0.82	0.51			671	Ho KS et al.	2008	Geochemical Journal	42(4)	339-357
265	IM 50	CAOB	NE China	114	43.33	basalts	Abaga	14.15	150	7.91	29.7	0.16074314	0.512858		-0.18	4.29		4.98	4.36	0.84	0.84	0.53			691	Ho KS et al.	2008	Geochemical Journal	42(4)	339-357
266	IM 54A	CAOB	NE China	114	43.33	basalts	Abaga	14.15	150	11.61	41.6	0.16844358	0.512889		-0.14	4.90		5.44	4.95	0.88	0.88	0.49			729	Ho KS et al.	2008	Geochemical Journal	42(4)	339-357
267	IM 55A	CAOB	NE China	114	43.33	basalts	Abaga	14.15	150	7.19	24.1	0.18006431	0.512891		-0.08	4.94		5.25	5.77	1.17	1.17	0.51			1023	Ho KS et al.	2008	Geochemical Journal	42(4)	339-357
268	IM 58A	CAOB	NE China	115.5	43.33	basalts	Abaga	14.15	150	6.44	22	0.17667503	0.512854		-0.10	4.21		4.60	5.25	1.22	1.22	0.56			1068	Ho KS et al.	2008	Geochemical Journal	42(4)	339-357
269	K10	CAOB	Junggar basin	86	44	Trachybasalt	Luliang	395	395	6.39	28.5	0.13532385	0.512916		-0.31	5.42		8.52	8.59	0.46	0.46	0.44			61	Zheng JP et al.	2007	nal of Asian Earth Scie	29(5-6)	778–794
270	K46	CAOB	Junggar basin	86	44	basalt	Wucaiwan	405	405	3.53	15.5	0.13745464	0.512897		-0.30	5.05		8.11	8.11	0.51	0.51	0.49			102	Zheng JP et al.	2007	nal of Asian Earth Scie	29(5-6)	778–794
271	K4	CAOB	Junggar basin	86	44	Basaltic trachyandsite	Wulungu	405	405	2.93	12.1	0.14615015	0.512903		-0.26	5.17		7.78	7.39	0.56	0.56	0.51			153	Zheng JP et al.	2007	nal of Asian Earth Scie	29(5-6)	778–794
272	K62	CAOB	Junggar basin	86	44	Basaltic andesite	Wucaiwan	405	405	6.16	28.4	0.1309096	0.512826		-0.33	3.67		7.07	6.56	0.60	0.60	0.57			192	Zheng JP et al.	2007	nal of Asian Earth Scie	29(5-6)	778–794
273	K173	CAOB	Junggar basin	86	44	basalt	Kexia	345	345	1.63	5.79	0.16991535	0.512967		-0.14	6.42		7.60	6.42	0.64	0.64	0.48			293	Zheng JP et al.	2007	nal of Asian Earth Scie	29(5-6)	778–794
274	K178	CAOB	Junggar basin	86	44	basalt	Kexia	345	345	1.93	6.45	0.18060211	0.512983		-0.08	6.73		7.44	6.73	0.77	0.77	0.49			425	Zheng JP et al.	2007	nal of Asian Earth Scie	29(5-6)	778–794
275	DZ-4	CAOB	NE China	126	48	olivine leucitite	chi, Erkesha	0	150	10.4	64.3	0.09761078	0.512497		-0.50	-2.75		-0.85	-2.75	0.86	0.86	1.01			708	Zhang M et al.	1991	Geochemical Journal	25(5)	387-398
276	H-38	CAOB	NE China	126	48	leucite basanite;	chi, Erkesha	0	150	13.3	82.3	0.09752647	0.512451		-0.50	-3.65		-1.75	-3.65	0.92	0.92	1.08			767	Zhang M et al.	1991	Geochemical Journal	25(5)	387-398
277	RB53	CAOB	NE China	126	48	leucite basanite;	chi, Erkesha	0	150	10.7	73.7	0.08761392	0.512316		-0.55	-6.28		-4.19	-6.10	1.01	1.01	1.28			858	Zhang M et al.	1991	Geochemical Journal	25(5)	387-398
278	L-13	CAOB	NE China	126	48	trachybasalt.	chi, Erkesha	0	150	11.2	65.3	0.10350672	0.512383		-0.47	-4.97		-3.19	-1.06	1.06	1.06	1.20			911	Zhang M et al.	1991	Geochemical Journal	25(5)	387-398
279	73-16B	CAOB	southern Baikal region	103	51	Basalt	Hamar-Dabai	12.8	150	6.21	30	0.12493231	0.51278		-0.36	2.77		4.14	5.78	0.64	0.64	0.60			486	Ionov DA et al.	1995	Contrib Mineral Petrol	122(1)	174-190
280	BI-07	CAOB	中天山	86.5	43	trachybasalt.	骆驼沟	329	329	4.23	15.4	0.16577625	0.512763		-0.16	2.44		3.74	3.74	1.23	1.23	0.78			901	Chen DL et al.	2001	Acta Petrologica Sinica	17(3)	378-384
281	BI-24	CAOB	中天山	86.5	43	Basalt	骆驼沟	329	329	6.7	23.9	0.16919194	0.512764		-0.14	2.46		3.61	4.21	1.32	1.32	0.79			991	Chen DL et al.	2001	Acta Petrologica Sinica	17(3)	378-384
282	BI-05	CAOB	中天山	86.5	43	Basalt	骆驼沟	329	329	4.27	13	0.1982378	0.512756		0.01	2.30		2.24	2.20	0.89	3.85	0.89			566	Chen DL et al.	2001	Acta Petrologica Sinica	17(3)	378-384
283	E-2921	CAOB	NW Russian Altai	82	51.5	basalt	Zasur'ya	500	500	4.21	16.7	0.15215464	0.512928		-0.23	5.66		8.50	8.50	0.55	0.55	0.53			51	Safonova I et al.	2011	nal of Asian Earth Scie	42(3)	191-207
284	ZAS-21-07	CAOB	NW Russian Altai	82	51.5	basalt	Zasur'ya	500	500	4.17	13.6	0.18506628	0.513031		-0.06	7.67		8.41	8.41	0.63	0.63	0.54			134	Safonova I et al.	2011	nal of Asian Earth Scie	42(3)	191-207
285	ZAS30-07	CAOB	NW Russian Altai	82	51.5	basalt	Zasur'ya	500	500	6.76	32.4	0.12592303	0.512771		-0.36	2.59		7.11	7.11	0.66	0.66	0.65			159	Safonova I et al.	2011	nal of Asian Earth Scie	42(3)	191-207
286	ZAS23-07	CAOB	NW Russian Altai	82	51.5	basalt	Zasur'ya	500	500	6.45	28.2	0.13804405	0.512806		-0.30	3.28		7.02	4.19	0.69	0.69	0.65			194	Safonova I et al.	2011	nal of Asian Earth Scie	42(3)	191-207
287	G-2921	CAOB	NW Russian Altai	82	51.5	basalt	Zasur'ya	500	500	3.23	10.5	0.18566627	0.51293		-0.06	5.70		6.40	5.87	1.20	1.20	0.70			695	Safonova I et al.	2011	nal of Asian Earth Scie	42(3)	191-207
288	GH04-1	CAOB	NE China	120.447	50.359	Trachybasalt	at Xinggan R	122.55	150	7.83	39.42	0.11988021	0.51276		-0.39	2.38		3.85	3.58	0.63	0.63	0.62			484	Zhang LC et al.	2008	Geochemical Journal	256(1-2)	12–23
289	05TH11	CAOB	NE China	124.642	52.462	Basalt	at Xinggan R	122.55	150	4.9	43.77	0.06756135	0.51253		-0.66	-2.11		0.37	-0.09	0.65	0.65	0.91			497	Zhang LC et al.	2008	Geochemical Journal	256(1-2)	12–23
290	GH04-4	CAOB	NE China	121.321	50.988	Trachybasalt	at Xinggan R	122.55	150	5.62	28.58	0.11867746	0.51268		-0.40	0.82		2.31	2.04	0.75	0.75	0.75			604	Zhang LC et al.	2008	Geochemical Journal	256(1-2)	12–23
291	JGD04-4	CAOB	NE China	124.38	49.948	Basalt	at Xinggan R	122.55	150	7.99	36.9	0.13068226	0.5127		-0.34	1.21		2.47	2.24	0.83	0.83	0.74			677	Zhang LC et al.	2008	Geochemical Journal	256(1-2)	12–23
292	XFZH-04	CAOB	NE China	118.894	43.876	Basalt	at Xinggan R	122.55	150	7.2	31.9	0.13621975	0.51272		-0.31	1.60		2.76	2.55	0.85	0.85	0.71			696	Zhang LC et al.	2008	Geochemical Journal	256(1-2)	12–23
293	ZHJ04-1	CAOB	NE China	121.131	44.753	Basaltic andesite	at Xinggan R	122.55	150	4.76	23.06	0.12457666	0.51263		-0.37	-0.16		1.22	2.60	0.89	0.89	0.84			740	Zhang LC et al.	2008	Geochemical Journal	256(1-2)	12–23

294	05TH10	CAOB	NE China	124.688	52.379	Basalt	at Xinggan R	122.55	150	6.34	33.36	0.11468784	0.51229		-0.42	-6.79		-5.22	-3.65	1.32	1.32	1.36			1172	Zhang LC et al.	2008	Geochemical Journal	256(1-2)	12–23
295	121/10A	CAOB	central Kazakhstan	76	50	Ignimbrites	as and Aksor	300	300	3.18	17.8	0.10781809	0.512586		-0.45	-1.01		2.39	2.39	0.81	0.81	0.87			512	Kurchavov AM et al	2008	Prtrology	16(6)	613-628
296	301/1A	CAOB	central Kazakhstan	76	50	Ignimbrites	as and Aksor	300	300	5.29	27.23	0.11724502	0.512604		-0.40	-0.66		2.38	2.38	0.86	0.86	0.87			563	Kurchavov AM et al	2008	Prtrology	16(6)	613-628
297	333	CAOB	central Kazakhstan	76	50	Ignimbrites	as and Aksor	300	300	5.92	30.48	0.11721746	0.512596		-0.40	-0.82		2.22	2.22	0.88	0.88	0.88			575	Kurchavov AM et al	2008	Prtrology	16(6)	613-628
298	283/1	CAOB	central Kazakhstan	76	50	Ignimbrites	as and Aksor	300	300	5.86	30.48	0.11602919	0.512587		-0.41	-0.99		2.09	2.09	0.88	0.88	0.89			579	Kurchavov AM et al	2008	Prtrology	16(6)	613-628
299	299	CAOB	central Kazakhstan	76	50	Ignimbrites	as and Aksor	300	300	4.98	24.46	0.12287417	0.512617		-0.38	-0.41		2.42	2.42	0.89	0.89	0.86			595	Kurchavov AM et al	2008	Prtrology	16(6)	613-628
300	123/2	CAOB	central Kazakhstan	76	50	Ignimbrites	as and Aksor	300	300	4.91	24.59	0.12050613	0.512602		-0.39	-0.70		2.21	2.21	0.90	0.90	0.88			596	Kurchavov AM et al	2008	Prtrology	16(6)	613-628
301	12A	CAOB	central Kazakhstan	76	50	Ignimbrites	as and Aksor	300	300	5.3	25.9	0.12349907	0.512616		-0.37	-0.43		2.37	2.37	0.90	0.90	0.87			603	Kurchavov AM et al	2008	Prtrology	16(6)	613-628
302	121/10	CAOB	central Kazakhstan	76	50	Ignimbrites	as and Aksor	300	300	4.72	23.21	0.12273018	0.512586		-0.38	-1.01		1.82	1.82	0.95	0.95	0.91			645	Kurchavov AM et al	2008	Prtrology	16(6)	613-628
303	17	CAOB	central Kazakhstan	76	50	Ignimbrites	as and Aksor	300	300	3.58	16.7	0.12937585	0.512607		-0.34	-0.60		1.97	1.97	0.98	0.98	0.90			681	Kurchavov AM et al	2008	Prtrology	16(6)	613-628
304	301/4	CAOB	central Kazakhstan	76	50	Ignimbrites	as and Aksor	300	300	5.06	25.23	0.12103553	0.512538		-0.38	-1.95		0.95	1.58	1.01	1.01	0.98			707	Kurchavov AM et al	2008	Prtrology	16(6)	613-628
305	1027/2A	CAOB	central Kazakhstan	76	50	Ignimbrites	as and Aksor	300	300	4.1	18.82	0.13147728	0.512605		-0.33	-0.64		1.85	2.19	1.01	1.01	0.91			710	Kurchavov AM et al	2008	Prtrology	16(6)	613-628
306	06XJ-018	CAOB	northwestern Tianshan	80.97	44.67	Granodiorite porphyry	amasu–Daba	366	366	3.575	15.66	0.13777735	0.512674		-0.30	0.70		3.45	3.26	0.96	0.96	0.83			590	Tang GJ et al.	2010	Lithos	3-4(119)	393-411
307	ZK125-390	CAOB	Altay	86	47	basalt	Sawur	340	340	5.3	25.4	0.12593756	0.512865		-0.36	4.43		7.50	7.50	0.50	0.50	0.48			156	Shen P et al.	2008	nal of Asian Earth Scie	2-4(32)	259-279
308	S20	CAOB	Altay	86	47	basalt	Sawur	340	340	3.636	17.11	0.12825856	0.512859		-0.35	4.31		7.28	5.33	0.52	0.52	0.50			180	Shen P et al.	2008	nal of Asian Earth Scie	2-4(32)	259-279
309	KLA12	CAOB	Altay	86	47	andesite	Sawur	340	340	4.7	22.9	0.1238715	0.512825		-0.37	3.65		6.81	4.74	0.55	0.55	0.54			212	Shen P et al.	2008	nal of Asian Earth Scie	2-4(32)	259-279
310	AC-80	CAOB	mlekano–Ogodzhinskii Be	127	52	Andesibasalt	ro–Zeiskaya	117	150	4	19.82	0.12180113	0.512688		-0.38	0.98		2.41	2.10	0.77	0.77	0.74			617	Sorokin AA et al.	2010	Doklady Earth Science	435(2)	1650-1655
311	AC-75	CAOB	mlekano–Ogodzhinskii Be	127	52	Andesibasalt	ro–Zeiskaya	118	150	3.86	19.06	0.12222481	0.512688		-0.38	0.98		2.40	2.11	0.77	0.77	0.74			620	Sorokin AA et al.	2010	Doklady Earth Science	435(2)	1650-1655
312	AC-100	CAOB	mlekano–Ogodzhinskii Be	127	52	Andesibasalt	ro–Zeiskaya	118	150	3.62	17.14	0.1274663	0.512714		-0.35	1.48		2.81	2.53	0.77	0.77	0.71			621	Sorokin AA et al.	2010	Doklady Earth Science	435(2)	1650-1655
313	K-65/4	CAOB	mlekano–Ogodzhinskii Be	125	53	Trachyandesibasalt	skii volcanic c	119	150	9.43	52.81	0.10776433	0.512537		-0.45	-1.97		-0.27	-0.62	0.88	0.88	0.96			732	Sorokin AA et al.	2010	Doklady Earth Science	435(2)	1650-1655
314	C-1041	CAOB	mlekano–Ogodzhinskii Be	125	53	Trachyandesibasal	skii volcanic c	119	150	7.72	41.54	0.1121578	0.512529		-0.43	-2.13		-0.51	1.89	0.93	0.93	0.98			782	Sorokin AA et al.	2010	Doklady Earth Science	435(2)	1650-1655
315	C-1041-1	CAOB	mlekano–Ogodzhinskii Be	125	53	Trachyandesibasal	skii volcanic c	119	150	7.72	41.35	0.11267292	0.51252		-0.43	-2.30		-0.69	1.69	0.95	0.95	1.00			801	Sorokin AA et al.	2010	Doklady Earth Science	435(2)	1650-1655
316	0906BB06-1	CAOB	Altay	89.2687	46.915	basalts	Suoerkuduke	372	372	5.407	18.8	0.17358986	0.512989		-0.12	6.85		7.94	7.94	0.61	0.61	0.47			241	Niu HC et al.	2006	nal of Asian Earth Scie	4-6(28)	439-456
317	0906BB06-7	CAOB	Altay	89.2687	46.915	basalts	Suoerkuduke	372	372	3.331	13.36	0.15048165	0.512887		-0.23	4.86		7.05	7.05	0.63	0.63	0.55			263	Niu HC et al.	2006	nal of Asian Earth Scie	4-6(28)	439-456
318	A05-1	CAOB	Altay	89.5758	46.531	basalts	Suoerkuduke	372	372	14.04	68.1	0.12442833	0.51273		-0.37	1.79		5.23	5.23	0.72	0.72	0.69			346	Niu HC et al.	2006	nal of Asian Earth Scie	4-6(28)	439-456
319	A04-2	CAOB	Altay	89.577	46.532	basalts	Suoerkuduke	372	372	14.44	69.07	0.12617557	0.512713		-0.36	1.46		4.81	4.81	0.76	0.76	0.73			390	Niu HC et al.	2006	nal of Asian Earth Scie	4-6(28)	439-456
320	A01-2	CAOB	Altay	89.5773	46.526	basalts	Suoerkuduke	372	372	3.987	19.11	0.12591574	0.512683		-0.36	0.88		4.24	4.24	0.81	0.81	0.77			439	Niu HC et al.	2006	nal of Asian Earth Scie	4-6(28)	439-456
321	0909BB10-3	CAOB	Altay	89.2672	46.92	basalts	Suoerkuduke	372	372	0.945	3.399	0.16780228	0.512905		-0.15	5.21		6.58	6.58	0.81	0.81	0.58			442	Niu HC et al.	2006	nal of Asian Earth Scie	4-6(28)	439-456
322	A03-2	CAOB	Altay	89.5768	46.528	basalts	Suoerkuduke	372	372	3.665	17.37	0.1273409	0.512676		-0.35	0.74		4.03	4.03	0.84	0.84	0.79			465	Niu HC et al.	2006	nal of Asian Earth Scie	4-6(28)	439-456
323	A010-2	CAOB	Altay	89.6912	46.5	basalts	Suoerkuduke	372	372	3.507	16.76	0.12628513	0.512644		-0.36	0.12		3.46	3.49	0.88	0.88	0.84			511	Niu HC et al.	2006	nal of Asian Earth Scie	4-6(28)	439-456
324	A06-3	CAOB	Altay	89.5758	46.543	basalts	Suoerkuduke	372	372	4.277	20.2	0.12778395	0.512624		-0.35	-0.27		3.00	3.02	0.93	0.93	0.88			561	Niu HC et al.	2006	nal of Asian Earth Scie	4-6(28)	439-456
325	A10-2	CAOB	Kazakhstan-Junggar	89.58	46.47	basalt	oranggekudu	375	375	3.51	16.8	0.12609222	0.512644		-0.36	0.12		3.50	3.59	0.88	0.88	0.84			506	Zhang HX et al.	2005	The Island Arc	1(14)	55-68
326	XJ21-20	CAOB	Eastern Junggar	90	46	Picrite	Beitash	385	385	1.648	5.233	0.19007762	0.512976		-0.03	6.59		6.92	6.92	1.12	1.12	0.57			737	Zhang ZC et al.	2008	Gondwana Research	16	201-218
327	XJ20-6	CAOB	Eastern Junggar	90	46	ankaramite	Beitash	385	385	1.498	5.511	0.16405904	0.51292		-0.17	5.50		7.10	7.10	0.71	0.71	0.55			322	Zhang ZC et al.	2008	Gondwana Research	16	201-219
328	XJ23-17-1	CAOB	Eastern Junggar	90	46	ankaramite	Beitash	385	385	1.733	6.646	0.15738151	0.512888		-0.20	4.88		6.81	6.81	0.71	0.71	0.58			325	Zhang ZC et al.	2008	Gondwana Research	16	201-220
329	XJ23-14	CAOB	Eastern Junggar	90	46	ankaramite	Beitash	385	385	1.811	6.257	0.17469212	0.512942		-0.11	5.93		7.01	6.80	0.81	0.81	0.56			428	Zhang ZC et al.	2008	Gondwana Research	16	201-221
330	XJ21-22	CAOB	Eastern Junggar	90	46	Picrite	Beitash	385	385	1.847	6.454	0.172726	0.51293		-0.12	5.70		6.87	6.64	0.82	0.82	0.57			434	Zhang ZC et al.	2008	Gondwana Research	16	201-222
331	ZHB-24	CAOB	Eastern Junggar	90	46	Basalt and basaltic andesite	Batamayine	310	310	2.37	7.96	0.17970408	0.512958		-0.09	6.24		6.91	6.91	0.86	0.86	0.51			551	Zhang HX et al.	2006	Acta petrologica Sinica	22(1)	31-40
332	ZHB-20	CAOB	Eastern Junggar	90	46	Basalt and basaltic andesite	Batamayine	310	310	2.32	7.81	0.17929129	0.512954		-0.09	6.16		6.85	6.85	0.87	0.87	0.51			559	Zhang HX et al.	2006	Acta petrologica Sinica	22(1)	31-40
333	ZHB-12	CAOB	Eastern Junggar	90	46	Basalt and basaltic andesite	Batamayine	310	310	2.03	6.87	0.17834672	0.512989		-0.09	6.85		7.57	7.57	0.69	0.69	0.45			385	Zhang HX et al.	2006	Acta petrologica Sinica	22(1)	31-40
334	ZHB-17	CAOB	Eastern Junggar	90	46	Basalt and basaltic andesite	Batamayine	310	310	2.5	8.95	0.16859196	0.512931		-0.14	5.72		6.83	5.72	0.74	0.74	0.51			431	Zhang HX et al.	2006	Acta petrologica Sinica	22(1)	31-40
335	ZHB-22	CAOB	Eastern Junggar	90	46	Basalt and basaltic andesite	Batamayine	310	310	2.56	8.63	0.17903992	0.512939		-0.09	5.87		6.57	5.87	0.93	0.93	0.53			618	Zhang HX et al.	2006	Acta petrologica Sinica	22(1)	31-40
336	82H-6	CAOB	NE China	126.17	48.75	basalts	Wudalianchi	0.274	150	11.54	69.24	0.10058131	0.51243		-0.49	-4.06		-2.22	-4.04	0.97	0.97	1.12			820	Basu AR et al.	1991	and Planetary Science	105	149-169
337	82H-4	CAOB	NE China	126.17	48.75	basalts	Wudalianchi	1.305	150	10.31	61.32	0.10146681	0.51242		-0.48	-4.25		-2.43	-4.24	0.99	0.99	1.14			841	Basu AR et al.	1991	and Planetary Science	105	149-169
338	82H-2	CAOB	NE China	126.17	48.75	basalts	Wudalianchi	1.305	150	16.6	78.1	0.12826863	0.51238		-0.35	-5.03		-3.72	-5.02	1.37	1.37	1.24			1222	Basu AR et al.	1991	and Planetary Science	105	149-169
339	MJ-35	CAOB	NE China	129.543	44.467	basaltic rocks		1.305	150	4.71	20.1	0.14142667	0.5128		-0.28	3.16		4.22	3.17	0.74	0.74	0.59			589	Peng ZC et al.	1986	Geochemical Journal	59(1)	3-33
340	MJ-5-1	CAOB	NE China	128.403	43.573	basaltic rocks		1.305	150	3.48	14.9	0.14096012	0.512769		-0.28	2.56		3.62	4.48	0.80	0.80	0.64			649	Peng ZC et al.	1986	Geochemical Journal	59(1)	3-33
341	HW-8	CAOB	NE China	126.333	48.633	basaltic rocks		1.305	150	11.4	72.5	0.09489111	0.512334		-0.52	-5.93		-3.98	-2.42	1.05	1.05	1.26			897	Peng ZC et al.	1986	Geochemical Journal	59(1)	3-33
342	ZYF29	CAOB	NE China	118	43.52	Basalt		270	270	2.18	8.2	0.16044972	0.512701		-0.18	1.23		2.48	2.48	1.28	1.28	0.83			1014	Zhu YF et al.	2001	Geological Magazine	138(02)	101-115
343	ZYF24	CAOB	NE China	118	43.52	Basalt		270	270	2.38	8.27	0.17368853	0.512734		-0.12	1.87		2.67	2.67	1.58	1.58	0.82			1312	Zhu YF et al.	2001	Geological Magazine	138(02)	101-115
344	ZYF31	CAOB	NE China	118	43.52	Basalt		270	270	2	6.68	0.18070098	0.512805		-0.08	3.26		3.81	3.81	1.59	1.59	0.72			1320	Zhu YF et al.	2001	Geological Magazine	138(02)	101-115
345	ZYF14	CAOB	NE China	118	43.52	Basalt		270	270	2.49	8.56	0.17555851	0.512701		-0.11	1.23		1.96	1.96	1.79	1.79	0.87			1519	Zhu YF et al.	2001	Geological Magazine	138(02)	101-115
346	ZYF26	CAOB	NE China	118	43.52	Basalt		270	270	2.18	6.92	0.19013097	0.512761		-0.03	2.40		2.63	2.63	2.50	2.50	0.82			2233	Zhu YF et al.	2001	Geological Magazine	138(02)	101-115
347	ZYF27	CAOB	NE China	118	43.52	Basalt		270	270	2.15	6.98	0.18589937	0.512687		-0.05	0.96		1.33	1.33	2.53	2.53	0.92			2256	Zhu YF et al.	2001	Geological Magazine	138(02)	101-115
348	ZYF7	CAOB	NE China	118	43.52	Basalt		270	270	3.2	10.39	0.18587698	0.512651		-0.06	0.25		0.63	0.25	2.72	2.72	0.98			2448	Zhu YF et al.	2001	Geological Magazine	138(02)	101-115
349	ZYF44	CAOB	NE China	118	43.52	Basalt		270	270	3.58	11.03	0.19588679	0.512714		0.00	1.48		1.51	1.48	0.91	3.70	0.91			635	Zhu YF et al.	2001	Geological Magazine	138(02)	101-115
350	DX4-28	CAOB	NE China	128.94	43.88	basanite	Jingpohu	0.0049	150	8.48	39.22	0.13049445	0.512774		-0.34	2.65		3.92	2.65	0.69	0.69	0.62			539	Zhang ZC et al.	2002	Geochemical Journal	36(2)	133-153
351	DX1-26	CAOB	NE China	128.94	43.88	basanite	Jingpohu	0.0049	150	7.19	31.42	0.13811083	0.512786		-0.30	2.89		4.01	2.89	0.73	0.73	0.61			585	Zhang ZC et al.	2002	Geochemical Journal	36(2)	133-153
352	HM-3	CAOB	NE China	128.94	43.88	Tephrite	Jingpohu	0.0049	150	8.8	41.89	0.12678471	0.512684		-0.36	0.90		2.24	0.90	0.82	0.82	0.76			668	Zhang ZC et al.	2002	Geochemical Journal	36(2)	133-153
353	HM-5	CAOB	NE China	128.94	43.88	Tephrite	Jingpohu	0.0049	150	9.2	42.68	0.13009378	0.51267		-0.34	0.62		1.90	0.62	0.88	0.88	0.78			725	Zhang ZC et al.	2002	Geochemical Journal	36(2)	133-153
354	JP-3	CAOB	NE China	128.94	43.88	basanite	Jingpohu	0.0049	150	6.01	26.8	0.13534213	0.512672		-0.31	0.66		1.84	0.66	0.93	0.93	0.79			780	Zhang ZC et al.	2002	Geochemical Journal	36(2)	133-153
355	DX1-4	CAOB	NE China	128.94	43.88	basanite	Jingpohu	0.0049	150	6.59	29.69	0.13395746	0.512656		-0.32	0.35		1.55	0.35	0.94	0.94	0.81			794	Zhang ZC et al.	2002	Geochemical Journal	36(2)	133-153
356	JP-2	CAOB	NE China	128.94	43.88	basanite	Jingpohu	0.0049	150	4.84	21.68	0.13473424	0.512658		-0.32	0.39		1.58	2.92	0.95	0.95	0.81			800	Zhang ZC et al.	2002	Geochemical Journal	36(2)	133-153
357	RY-1	CAOB	NE China	128.94	43.88	basanite	Jingpohu	0.0049	150	6.27	26.7	0.14172586	0.512667		-0.28	0.57		1.62	2.81	1.02	1.02	0.81			873	Zhang ZC et al.	2002	Geochemical Journal	36(2)	133-153
358	WXT721	CAOB	northern Tianshan	82.6981	45.121	basalt	Alataw	320	320	5.808	20.66	0.16967695	0.512996		-0.14	6.98		8.09	8.09	0.53	0.53	0.42			214	Wang Q et al.	2007	Chemical Geology	1(236)	42-64
359	WXT742	CAOB	northern Tianshan	81.8056	45.477	basalt	Alataw	320	320	2.451	11.15	0.13266985	0.512774		-0.33	2.65		5.27	2.68	0.71	0.71	0.65			388	Wang Q et al.	2007	Chemical Geology	1(236)	42-64
360	XT017	CAOB	northern Tianshan	81.5	45	basalt	Alataw	320	320	8.057	30.5	0.159437	0.512889		-0.19	4.90		6.42	4.92	0.73	0.73	0.55			414	Wang Q et al.	2007	Chemical Geology	1(236)	42-64
361	MN-15.1	CAOB	Mongolia	100.438	48.17	tephrite basanite	Tariat-Chuloc	3	150	8.08	39.87	0.12231023	0.512714		-0.38	1.48		2.91	1.51	0.73	0.73	0.70			578	Barry TL et al.	2003	Journal of Petrology	1	55-91
362	MN-11.2.2	CAOB	Mongolia	100.44	48.229	basaltic trachyandesite	Tariat-Morun	6	150	6.71	30.88	0.13114195	0.512702		-0.33	1.25		2.50	1.27	0.83	0.83	0.73			677	Barry TL et al.	2003	Journal of Petrology	1	55-91
363	MN-5.2.2	CAOB	Mongolia	100.433	48.217	Basalt	Tariat-Chuloc	3	150	5.77	26.36	0.13210739	0.512705		-0.33	1.31		2.54	1.33	0.83	0.83	0.73			682	Barry TL et al.	2003	Journal of Petrology	1	55-91
364	MN-16.2.1	CAOB	Mongolia	100.44	48.229	tephrite basanite	Tariat-Chuloc	3	150	6.6	34.06	0.11694635	0.512614		-0.41	-0.47		1.06	-0.44	0.84	0.84	0.85			695	Barry TL et al.	2003	Journal of Petrology	1	55-91
365	MN-25.1	CAOB	Mongolia	101.745	48.828	tephrite basanite	Hanui	3	150	6.13	25.27	0.14640543	0.512756		-0.26	2.30		3.26	2.51	0.89	0.89	0.67			743	Barry TL et al.	2003	Journal of Petrology	1	55-91
366	MN-9.1.3	CAOB	Mongolia	100.44	48.229	tephrite basanite	Tariat-Chuloc	3	150	6.37	29.95	0.12836105	0.512646		-0.35	0.16		1.46	0.44	0.90	0.90	0.82			750	Barry TL et al.	2003	Journal of Petrology	1	55-91
367	IM8810	CAOB	NE China	116	44	alkali basalt	linhot and Ab	32.75	150	11.3	54.9	0.12422842	0.512882		-0.37	4.76		6.15	5.06	0.46	0.46	0.44			307	g FL and Macdougal	1992	Nature	360(6402)	333-336
368	IM8801	CAOB	NE China	114	43.33	alkali basalt	linhot and Ab	32.75	150	8.97	41.2	0.13140461	0.512887		-0.33	4.86		6.11	5.13	0.49	0.49	0.44			338	g FL and Macdougal	1992	Nature	360(6402)	333-336
369	IM8814	CAOB	NE China	114	43.33	alkali basalt	linhot and Ab	32.75	150	8.65	41.5	0.12580017	0.512866		-0.36	4.45		5.80	4.74	0.49	0.49	0.47			343	g FL and Macdougal	1992	Nature	360(6402)	333-336
370	IM8801	CAOB	NE China	114	43.33	alkali basalt	linhot and Ab	32.75	150	8.52	39.2	0.13118026	0.512883		-0.33	4.78		6.03	5.05	0.49	0.49	0.45			344	g FL and Macdougal	1992	Nature	360(6402)	333-336
371	IM8808	CAOB	NE China	116	44	alkali basalt	linhot and Ab	32.75	150	6.87	29.7	0.13960976	0.512888		-0.29	4.88		5.97	5.12	0.54	0.54	0.45			390	g FL and Macdougal	1992	Nature	360(6402)	333-336
372	IM8814	CAOB	NE China	114	43.33	alkali basalt	linhot and Ab	32.75	150	9.81	47.1	0.12570671	0.512838		-0.36	3.90		5.26	4.20	0.54	0.54	0.51			391	g FL and Macdougal	1992	Nature	360(6402)	333-336
373	IM8808	CAOB	NE China	116	44	alkali basalt	linhot and Ab	32.75	150	5.93	25	0.14316286	0.51289		-0.27	4.92		5.94	5.14	0.56	0.56	0.45			413	g FL and Macdougal	1992	Nature	360(6402)	333-336
374	IM8815	CAOB	NE China	114	43.33	alkali basalt	linhot and Ab	32.75	150	6.3	28.2	0.13483477	0.512839		-0.31	3.92		5.11	6.62	0.60	0.60	0.52			452	g FL and Macdougal	1992	Nature	360(6402)	333-336
375	IM8815	CAOB	NE China	114	43.33	alkali basalt	linhot and Ab	32.75	150	6.18	27.6	0.13514182	0.512838		-0.31	3.90		5.08	6.59	0.61	0.61	0.52			456	g FL and Macdougal	1992	Nature	360(6402)	333-336
376	T-18	CAOB	Tianshan	89	44.5	basic lava	Conglomerate	342.5	342.5	6.58	29.9	0.13282284	0.512913		-0.32	5.36		8.16	8.16	0.45	0.45	0.43			105	Xia LQ et al.	2008	nal of Asian Earth Scie	4-6(31)	357-378
377	T-19	CAOB	Tianshan	89	44.5	basic lava	Conglomerate	342.5	342.5	6.58	29.9	0.13282237	0.512898		-0.32	5.07		7.86	7.86	0.48	0.48	0.46			133	Xia LQ et al.	2008	nal of Asian Earth Scie	4-6(31)	357-378
378	T-17	CAOB	Tianshan	89	44.5	basic lava	Conglomerate	342.5	342.5	7.02	28.6	0.14814583	0.512916		-0.25	5.42		7.54	7.54	0.54	0.54	0.48			202	Xia LQ et al.	2008	nal of Asian Earth Scie	4-6(31)	357-378
379	05T-18	CAOB	Tianshan	83.15	43.45	basic lava	ajunshan For	342.5	342.5	3.846	14.29	0.16243926	0.512873		-0.17	4.58		6.08	6.08	0.82	0.82	0.60			482	Xia LQ et al.	2008	nal of Asian Earth Scie	4-6(31)	357-378
380	04T-18	CAOB	Tianshan	81.78	43.19	basic lava	ajunshan For	342.5	342.5	5.933	27.08	0.13222628	0.51266		-0.33	0.43		3.25	3.25	0.92	0.92	0.83			574	Xia LQ et al.	2008	nal of Asian Earth Scie	4-6(31)	357-378
381	04T-13	CAOB	Tianshan	81.08	43.12	basic lava	ajunshan For	342.5	342.5	3.502	12.69	0.16655741	0.512829		-0.15	3.73		5.04	5.04	1.04	1.04	0.68			695	Xia LQ et al.	2008	nal of Asian Earth Scie	4-6(31)	357-378
382	05T-13	CAOB	Tianshan	81.78	43.19	basic lava	ajunshan For	342.5	342.5	3.871	15.68	0.14898743	0.512469		-0.24	-3.30		-1.21	-1.21	1.60	1.60	1.19			1258	Xia LQ et al.	2008	nal of Asian Earth Scie	4-6(31)	357-378
383	03T-98	CAOB	Tianshan	81.08	43.12	basic lava	ajunshan For	342.5	342.5	3.04	12.1	0.1516218	0.512478		-0.23	-3.12		-1.15	-2.55	1.65	1.65	1.19			1304	Xia LQ et al.	2008	nal of Asian Earth Scie	4-6(31)	357-378
384	NTS-05	CAOB	margin of the North Chin	119.29	42.04	basalts	Niutoushan	100	150	5.78	34.86	0.10007107	0.512816		-0.49	3.47		5.32	4.71	0.45	0.45	0.50			299	Zhang HF et al.	2003	iimica et Cosmochimic	67(22)	4373-4387
385	NTS-04	CAOB	margin of the North Chin	119.29	42.04	basalts	Niutoushan	100	150	6.16	34.62	0.10738857	0.51278		-0.45	2.77		4.48	3.91	0.53	0.53	0.57			381	Zhang HF et al.	2003	iimica et Cosmochimic	67(22)	4373-4387
386	NTS-03	CAOB	margin of the North Chin	119.29	42.04	basalts	Niutoushan	100	150	6.38	34.63	0.11119052	0.512733		-0.43	1.85		3.49	2.94	0.62	0.62	0.65			471	Zhang HF et al.	2003	iimica et Cosmochimic	67(22)	4373-4387
387	NTS-01	CAOB	margin of the North Chin	119.29	42.04	basalts	Niutoushan	100	150	5.83	34.02	0.10342546	0.512671		-0.47	0.64		2.43	1.83	0.66	0.66	0.74			513	Zhang HF et al.	2003	iimica et Cosmochimic	67(22)	4373-4387
388	NTS-07	CAOB	margin of the North Chin	119.29	42.04	basalts	Niutoushan	100	150	5.82	34.57	0.10160515	0.51266		-0.48	0.43		2.25	1.64	0.67	0.67	0.76			517	Zhang HF et al.	2003	iimica et Cosmochimic	67(22)	4373-4387
389	NTS-06	CAOB	margin of the North Chin	119.29	42.04	basalts	Niutoushan	100	150	6.1	35.13	0.10479588	0.512664		-0.47	0.51		2.27	1.68	0.68	0.68	0.75			531	Zhang HF et al.	2003	iimica et Cosmochimic	67(22)	4373-4387
390	NTS-08	CAOB	margin of the North Chin	119.29	42.04	basalts	Niutoushan	100	150	6.16	34.24	0.10857769	0.512675		-0.45	0.72		2.41	1.85	0.69	0.69	0.74			539	Zhang HF et al.	2003	iimica et Cosmochimic	67(22)	4373-4387
391	NTS-02	CAOB	margin of the North Chin	119.29	42.04	basalts	Niutoushan	100	150	6.53	36.46	0.10809095	0.512667		-0.45	0.57		2.26	0.94	0.70	0.70	0.75			548	Zhang HF et al.	2003	iimica et Cosmochimic	67(22)	4373-4387
392	NTS-09	CAOB	margin of the North Chin	119.29	42.04	basalts	Niutoushan	100	150	6.38	36.31	0.10604403	0.512657		-0.46	0.37		2.11	2.79	0.70	0.70	0.77			549	Zhang HF et al.	2003	iimica et Cosmochimic	67(22)	4373-4387
393	MHP 79/4 BA	CAOB	central Mongolia	99	48	PERIDOTITE XENOLITHS	ariat Depressi	32.75	150	8.57	47.31	0.10932249	0.512558		-0.44	-1.56		0.11	0.77	0.86	0.86	0.93			715	Stosch HG et al.	1986	iimica et Cosmochimic	50(12)	2601-2614
394	TSKH 1/8	CAOB	Western Transbaikalia	111.17	51.58	felsic rocks	an-Khurtei g	209	209	12.1	66.7	0.10948334	0.512625		-0.44	-0.25		2.07	2.07	0.77	0.77	0.82			559	Vorontsov AA et al.	2004	Geokhimiya	42(11)	1046-1061
395	TSKH 1/7	CAOB	Western Transbaikalia	111.17	51.58	felsic rocks	an-Khurtei g	209	209	7.22	37.4	0.11650814	0.512646		-0.41	0.16		2.29	2.29	0.79	0.79	0.80			582	Vorontsov AA et al.	2004	Geokhimiya	42(11)	1046-1061
396	TSKH 1/19	CAOB	Western Transbaikalia	111.17	51.58	felsic rocks	an-Khurtei g	209	209	7.99	42.9	0.11240279	0.512616		-0.43	-0.43		1.82	1.82	0.80	0.80	0.84			595	Vorontsov AA et al.	2004	Geokhimiya	42(11)	1046-1061
397	TSKH 1/6	CAOB	Western Transbaikalia	111.17	51.58	felsic rocks	an-Khurtei g	209	209	6.09	30.9	0.11894551	0.512632		-0.40	-0.12		1.96	3.28	0.83	0.83	0.83			625	Vorontsov AA et al.	2004	Geokhimiya	42(11)	1046-1061
398	TSKH 1/1	CAOB	Western Transbaikalia	111.17	51.58	felsic rocks	an-Khurtei g	209	209	7.79	39.3	0.11962669	0.512573		-0.39	-1.27		0.79	2.10	0.93	0.93	0.92			726	Vorontsov AA et al.	2004	Geokhimiya	42(11)	1046-1061
399	TW206-1	CAOB	eastern Tianshan	93	42	basalt	uwu–Yandor	342.5	342.5	4.294	20.1	0.12893715	0.512855		-0.34	4.23		7.19	7.19	0.53	0.53	0.51			189	Zhang LC et al.	2006	Mineralium Deposita	41(2)	188–200
400	TW202-1	CAOB	eastern Tianshan	93	42	basalt	uwu–Yandor	342.5	342.5	4.484	21.08	0.128382	0.512826		-0.35	3.67		6.65	7.15	0.58	0.58	0.55			237	Zhang LC et al.	2006	Mineralium Deposita	41(2)	188–200
401	TW206-2	CAOB	eastern Tianshan	93	42	basalt	uwu–Yandor	342.5	342.5	4.895	20.95	0.14102022	0.512861		-0.28	4.35		6.78	7.19	0.61	0.61	0.54			264	Zhang LC et al.	2006	Mineralium Deposita	41(2)	188–200
402	KHOL-1/3	CAOB	Tuvinian	99.5	52.8	basite	ropotkin Ridg	400	400	7.82	38.17	0.12364163	0.512543		-0.37	-1.85		1.88	1.88	1.03	1.03	0.99			627	Vorontsov AA et al.	2010	an Geology and Geoph	51(8)	833-845
403	MNG-1/2	CAOB	Tuvinian	98	53	basite	ropotkin Ridg	400	400	8.63	40.75	0.12781	0.512558		-0.35	-1.56		1.96	1.96	1.05	1.05	0.98			650	Vorontsov AA et al.	2010	an Geology and Geoph	51(8)	833-845
404	MNG-1/6	CAOB	Tuvinian	98	53	basite	ropotkin Ridg	400	400	10.79	44.81	0.14532363	0.512637		-0.26	-0.02		2.60	2.60	1.14	1.14	0.93			743	Vorontsov AA et al.	2010	an Geology and Geoph	51(8)	833-845
405	KHOL-1/8	CAOB	Tuvinian	99.5	52.8	basite	ropotkin Ridg	400	400	8.13	38.08	0.12884482	0.512477		-0.34	-3.14		0.32	0.32	1.21	1.21	1.12			808	Vorontsov AA et al.	2010	an Geology and Geoph	51(8)	833-845

406	MNG-1/1	CAOB	Tuvinian	98	53	basite	ropotkin Ridg	400	400	8	35.42	0.13630784	0.512536		-0.31	-1.99		1.09	1.09	1.21	1.21	1.05			808	Vorontsov AA et al.	2010	an Geology and Geoph	51(8)	833-845
407	KHOL-1/5	CAOB	Tuvinian	99.5	52.8	basite	ropotkin Ridg	400	400	6.51	29.87	0.1315283	0.512478		-0.33	-3.12		0.21	0.21	1.25	1.25	1.13			845	Vorontsov AA et al.	2010	an Geology and Geoph	51(8)	833-845
408	ARY-1/8	CAOB	Tuvinian	99.7	52.9	basite	ropotkin Ridg	400	400	8.76	40.81	0.12953896	0.512375		-0.34	-5.13		-1.70	-5.02	1.40	1.40	1.28			1002	Vorontsov AA et al.	2010	an Geology and Geoph	51(8)	833-845
409	MNG-1/4	CAOB	Tuvinian	98	53	basite	ropotkin Ridg	400	400	9.98	48.05	0.12533745	0.512188		-0.36	-8.78		-5.14	-8.66	1.66	1.66	1.56			1256	Vorontsov AA et al.	2010	an Geology and Geoph	51(8)	833-845
410	3250	CAOB	ene Alkaline Basaltic Volc	100	50	hawaiite	Heven Platea	12.8	150	7.15	30.3	0.14242138	0.512852		-0.28	4.17		5.21	4.26	0.64	0.64	0.51			488	Perepelov AB et al.	2010	Doklady Earth Science	2(434)	1230–1234
411	3252	CAOB	ene Alkaline Basaltic Volc	100	50	hawaiite	Heven Platea	12.8	150	7.54	35.4	0.12854952	0.51276		-0.35	2.38		3.68	2.49	0.70	0.70	0.64			549	Perepelov AB et al.	2010	Doklady Earth Science	2(434)	1230–1234
412	3433	CAOB	ene Alkaline Basaltic Volc	100	50	hawaiite	Heven Platea	12.8	150	7.17	31.2	0.13869656	0.512748		-0.29	2.15		3.26	2.24	0.82	0.82	0.67			667	Perepelov AB et al.	2010	Doklady Earth Science	2(434)	1230–1234
413	3254	CAOB	ene Alkaline Basaltic Volc	100	50	hawaiite	Heven Platea	12.8	150	7.92	33.5	0.14268598	0.512746		-0.27	2.11		3.14	2.20	0.87	0.87	0.68			717	Perepelov AB et al.	2010	Doklady Earth Science	2(434)	1230–1234
414	3257	CAOB	ene Alkaline Basaltic Volc	100	50	hawaiite	Heven Platea	12.8	150	6.7	31.6	0.12796138	0.512651		-0.35	0.25		1.57	0.38	0.89	0.89	0.81			737	Perepelov AB et al.	2010	Doklady Earth Science	2(434)	1230–1234
415	980/1	CAOB	Baikal	103	51	tle xenoliths and enclosing ba	Khamar-Daba	14.15	150	6.95	41	0.10230751	0.512801		-0.48	3.18		4.99	3.18	0.48	0.48	0.53			328	Grachev AF	1998	iya Physics of the Soli	34(3)	175-200
416	302-36	CAOB	Baikal	113	54	peridotite xenoliths	Vitim	14.15	150	6	27.1	0.13362522	0.5128		-0.32	3.16		4.37	3.16	0.67	0.67	0.58			517	Ionov DA et al.	2005	Chemical Geology	217(1-2)	41-75
417	J99823-1	CAOB	NE China	128.5	44	alkali basalt	Jingbohu	0.0029	150	9.12	46.7	0.11786502	0.512805		-0.40	3.26		4.77	3.26	0.55	0.55	0.55			399	Zou HB et al.	2008	Chemical Geology	1-2(255)	134-142
418	J99821-5	CAOB	NE China	128.5	44	alkali basalt	Jingbohu	0.0029	150	8.46	43.3	0.11792042	0.512801		-0.40	3.18		4.69	3.29	0.56	0.56	0.56			406	Zou HB et al.	2008	Chemical Geology	1-2(255)	134-142
419	J99822-1	CAOB	NE China	128.5	44	alkali basalt	Jingbohu	0.0029	150	9.61	50.4	0.11507749	0.512713		-0.41	1.46		3.03	1.58	0.68	0.68	0.69			526	Zou HB et al.	2008	Chemical Geology	1-2(255)	134-142
420	93VBS 4	CAOB	Baikal Rift Zone	112	53	melanephelinite	tuff pit	11	150	10.97	56.89	0.11638221	0.512892		-0.41	4.95		6.49	5.07	0.40	0.40	0.41			255	Johnson JS et al.	2005	Journal of Petrology	7	1309-1344
421	93VBS 25	CAOB	Baikal Rift Zone	112.88	53.98	nephelinite	Hoygot	11	150	8.633	44.237	0.11778493	0.51287		-0.40	4.53		6.04	4.64	0.45	0.45	0.45			296	Johnson JS et al.	2005	Journal of Petrology	7	1309-1344
422	93VBS 356	CAOB	Baikal Rift Zone	112.405	53.758	basanite	Ekzar	11	150	11.3705	58.768	0.11677535	0.512859		-0.41	4.31		5.84	4.42	0.46	0.46	0.46			308	Johnson JS et al.	2005	Journal of Petrology	7	1309-1344
423	93VBS 162	CAOB	Baikal Rift Zone	112.405	53.758	basanite	Ekzar	11	150	8.103	39.395	0.12414222	0.512881		-0.37	4.74		6.13	4.84	0.46	0.46	0.44			309	Johnson JS et al.	2005	Journal of Petrology	7	1309-1344
424	93VBS 205	CAOB	Baikal Rift Zone	112.98	53.77	nephelinite	Antasey	11	150	9.808	49.317	0.12003194	0.512864		-0.39	4.41		5.88	4.52	0.47	0.47	0.46			316	Johnson JS et al.	2005	Journal of Petrology	7	1309-1344
425	93VBS 268	CAOB	Baikal Rift Zone	112.455	53.605	melanephelinite	Bortovoy	11	150	8.667	44.096	0.11862644	0.512853		-0.40	4.19		5.69	4.30	0.48	0.48	0.47			327	Johnson JS et al.	2005	Journal of Petrology	7	1309-1344
426	93VBS 355	CAOB	Baikal Rift Zone	112.405	53.758	basanite	Ekzar	11	150	6.6	31.244	0.12749441	0.512872		-0.35	4.56		5.89	4.66	0.49	0.49	0.46			342	Johnson JS et al.	2005	Journal of Petrology	7	1309-1344
427	93VBS 112	CAOB	Baikal Rift Zone	112.9	53.65	melanephelinite	Kolichikan	11	150	7.262	34.495	0.12706137	0.512868		-0.35	4.49		5.82	4.58	0.50	0.50	0.46			347	Johnson JS et al.	2005	Journal of Petrology	7	1309-1344
428	93VBS 365	CAOB	Baikal Rift Zone	112.405	53.758	basanite	Ekzar	11	150	7.384	35.669	0.12494321	0.512853		-0.36	4.19		5.57	4.29	0.51	0.51	0.48			361	Johnson JS et al.	2005	Journal of Petrology	7	1309-1344
429	93VBS 43	CAOB	Baikal Rift Zone	113.31	53.65	alkali basalt	Muliha	11	150	7.011	31.599	0.13391214	0.512867		-0.32	4.47		5.67	4.56	0.54	0.54	0.48			391	Johnson JS et al.	2005	Journal of Petrology	7	1309-1344
430	93VBS 215	CAOB	Baikal Rift Zone	112.98	53.77	melanephelinite	Antasey	11	150	6.6235	32.301	0.12376	0.512822		-0.37	3.59		4.99	3.69	0.56	0.56	0.53			407	Johnson JS et al.	2005	Journal of Petrology	7	1309-1344
431	93VBS 55	CAOB	Baikal Rift Zone	112.23	53.7	alkali basalt	Burul'zay	11	150	5.4685	24.404	0.13524392	0.51284		-0.31	3.94		5.12	4.03	0.60	0.60	0.52			453	Johnson JS et al.	2005	Journal of Petrology	7	1309-1344
432	93VBS 370	CAOB	Baikal Rift Zone	112.405	53.758	tholeiitic basalt	Ekzar	11	150	5.088	22.408	0.13704244	0.512845		-0.30	4.04		5.18	4.12	0.61	0.61	0.52			457	Johnson JS et al.	2005	Journal of Petrology	7	1309-1344
433	93VBS 369	CAOB	Baikal Rift Zone	112.405	53.758	tholeiitic basalt	Ekzar	11	150	5.2815	23.324	0.13666746	0.512843		-0.31	4.00		5.15	4.08	0.61	0.61	0.52			458	Johnson JS et al.	2005	Journal of Petrology	7	1309-1344
434	93VBS 63	CAOB	Baikal Rift Zone	112.88	53.98	alkali basalt	Burul'zay	11	150	5.5845	25.681	0.1312444	0.512819		-0.33	3.53		4.78	3.62	0.61	0.61	0.55			463	Johnson JS et al.	2005	Journal of Petrology	7	1309-1344
435	93VBS 315	CAOB	Baikal Rift Zone	112.9	53.65	tholeiitic basalt	Kolichikan	11	150	5.8525	23.795	0.14844629	0.51287		-0.25	4.53		5.45	4.59	0.65	0.65	0.49			505	Johnson JS et al.	2005	Journal of Petrology	7	1309-1344
436	93VBS 283	CAOB	Baikal Rift Zone	112.98	53.77	alkali basalt	Ekzar	11	150	6.7165	29.699	0.13649266	0.512816		-0.31	3.47		4.62	3.47	0.66	0.66	0.56			510	Johnson JS et al.	2005	Journal of Petrology	7	1309-1344
437	93VBS 92	CAOB	Baikal Rift Zone	112.88	53.98	alkali basalt	Hoygot	11	150	5.386	22.52	0.14434371	0.512736		-0.27	1.91		2.91	1.91	0.91	0.91	0.70			760	Johnson JS et al.	2005	Journal of Petrology	7	1309-1344
438	J1122-1	CAOB		85.5389	46.693	Gabbro		419.8	419.8	4.09	16	0.15452321	0.512873		-0.21	4.58		6.84	6.37	0.71	0.71	0.60			294	Yin JY et al.	2017	Gondwana Research	43	55-73
439	J1122-4	CAOB		85.5389	46.693	Gabbro		419.8	419.8	4.14	15.8	0.15870223	0.512873		-0.19	4.58		6.62	4.58	0.77	0.77	0.62			348	Yin JY et al.	2017	Gondwana Research	43	55-73
440	ZK403-421	CAOB		84.5021	45.414	Gabbro		310	310	3.06	14.1	0.14149671	0.51292142		-0.28	5.53		7.71	5.53	0.48	0.48	0.44			173	Cao M et al.	2016	Lithos	246-247	13-30
441	ZK603-347	CAOB		84.502	45.418	Gabbro		310	310	2.96	14.1	0.13588853	0.51290885		-0.31	5.28		7.69	5.28	0.47	0.47	0.44			163	Cao M et al.	2016	Lithos	246-247	13-30
442	QXH111	CAOB		90.1145	46.454	Pyroxene basalt		380.5	380.5	2.57	9.45	0.04411	0.51289		-0.78	4.92		12.32	4.92	0.38	0.23	0.12			-1	Chai F et al.	2012	Acta Petrologica Sinica	28	2183-2198
443	QXH113	CAOB		90.1145	46.454	Pyroxene basalt		380.5	380.5	3.44	13.9	0.14716	0.51288		-0.25	4.72		7.13	4.72	0.62	0.62	0.55			239	Chai F et al.	2012	Acta Petrologica Sinica	28	2183-2198
444	HG-2	CAOB		85.5317	46.674	hornblede gabbro		422	422	3.46	13	0.16090597	0.512799		-0.18	3.14		5.07	3.14	1.01	1.01	0.75			591	Chen JF et al.	2015	nal of Asian Earth Scie	113	90-109
445	K24-7	CAOB		83.2275	46.286	Basalt		315.3	315.3	6.28	30.93	0.122693	0.512802		-0.38	3.20		6.18	3.20	0.58	0.58	0.57			268	Li D et al.	2015	Gondwana Research	27	342-362
446	K24-8	CAOB		83.2275	46.286	Basalt		315.3	315.3	6.44	31.8	0.122408	0.512785		-0.38	2.87		5.86	2.87	0.61	0.61	0.60			295	Li D et al.	2015	Gondwana Research	27	342-362
447	K24-9	CAOB		83.2275	46.286	Basalt		315.3	315.3	5.93	28.63	0.125301	0.512841		-0.36	3.96		6.83	3.96	0.53	0.53	0.52			218	Li D et al.	2015	Gondwana Research	27	342-362
448	K24-11	CAOB		83.2275	46.286	Basalt		315.3	315.3	6.22	30.25	0.124267	0.512843		-0.37	4.00		6.91	4.00	0.52	0.52	0.51			209	Li D et al.	2015	Gondwana Research	27	342-362
449	K24-12	CAOB		83.2275	46.286	Basalt		315.3	315.3	6.7	32.5	0.12472	0.51284		-0.37	3.94		6.84	3.94	0.53	0.53	0.52			216	Li D et al.	2015	Gondwana Research	27	342-362
450	K24-13	CAOB		83.2275	46.286	Basalt		315.3	315.3	5.18	25.27	0.12388	0.512829		-0.37	3.73		6.66	3.73	0.55	0.55	0.53			230	Li D et al.	2015	Gondwana Research	27	342-362
451	HTP47-3	CAOB		84.2867	45.874	basalt		324	324	5.26	19.8	0.1653	0.512761		-0.16	2.40		3.70	2.40	1.22	1.22	0.78			900	Shen P et al.		International Geology Rev	55	1991-2007
452	HTP43-2	CAOB		84.2867	45.874	basalt		324	324	5.05	18.7	0.1645	0.512751		-0.16	2.20		3.54	2.20	1.24	1.24	0.79			911	Shen P et al.		International Geology Rev	55	1991-2007
453	HTP33	CAOB		84.2867	45.874	basalt		324	324	2.22	6.98	0.1995	0.512844		0.01	4.02		3.90	4.02	0.76	3.26	0.76			433	Shen P et al.		International Geology Rev	55	1991-2007
454	HTP36-1	CAOB		84.2867	45.874	basalt		324	324	2.49	7.88	0.1934	0.5128		-0.02	3.16		3.30	3.16	2.61	2.61	0.81			2290	Shen P et al.		International Geology Rev	55	1991-2007
455	HG11-9	CAOB		86.565	46.818	basalt		450	450	2.65	9.07	0.177	0.512866		-0.10	4.45		5.58	4.45	1.18	1.18	0.73			729	Shen P et al.		International Geology Rev	56	206-223
456	HG16-1	CAOB		86.565	46.818	basalt		450	450	2.87	10.4	0.1638	0.512827		-0.17	3.69		5.58	3.69	0.99	0.99	0.73			537	Shen P et al.		International Geology Rev	56	206-223
457	HG19-1	CAOB		86.565	46.818	basalt		450	450	2.95	9.66	0.1781	0.512863		-0.09	4.39		5.46	4.39	1.23	1.23	0.74			778	Shen P et al.		International Geology Rev	56	206-223
458	HG21-1	CAOB		86.565	46.818	basalt		450	450	3.13	10.5	0.183	0.51287		-0.07	4.53		5.31	4.53	1.39	1.39	0.75			938	Shen P et al.		International Geology Rev	56	206-223
459	HG6-1	CAOB		86.565	46.818	basalt		450	450	1.84	6.95	0.1449	0.512731		-0.26	1.81		4.79	1.81	0.93	0.93	0.79			478	Shen P et al.		International Geology Rev	56	206-223
460	TK-02-1*	CAOB		84.4	46.034	Mafic dike		292	292	2.64	11.3	0.1411	0.512813		-0.28	3.41		5.49	3.41	0.71	0.71	0.61			416	Yin J et al.	2015	Tectonophysics	662	385-397
461	TK-02-4*	CAOB		84.4	46.034	Mafic dike		292	292	2.5	11	0.1369	0.51281		-0.30	3.36		5.58	3.36	0.68	0.68	0.60			383	Yin J et al.	2015	Tectonophysics	662	385-397
462	TK-02-5*	CAOB		84.4	46.034	Mafic dike		292	292	2.6	11.1	0.1411	0.512816		-0.28	3.47		5.54	3.47	0.70	0.70	0.60			410	Yin J et al.	2015	Tectonophysics	662	385-397
463	TC-22	CAOB		83.5	47.05	Basalt		478	478	5.37	14.8	0.2082	0.513083		0.06	8.68		7.98	8.68	1.85	1.85	0.56			1373	Chen YC et al.	2017	nal of Asian Earth Scie	145	424-445
464	TC-23	CAOB		83.5	47.05	Basalt		478	478	3.75	11.3	0.2039	0.513066		0.04	8.35		7.91	8.35	1.31	1.31	0.56			827	Chen YC et al.	2017	nal of Asian Earth Scie	145	424-445
465	TC-25	CAOB		83.5	47.05	Basalt		478	478	4.82	13.8	0.1991	0.51306		0.01	8.23		8.09	8.23	0.94	0.94	0.55			462	Chen YC et al.	2017	nal of Asian Earth Scie	145	424-445
466	TC-26	CAOB		83.5	47.05	Basalt		478	478	4.61	14	0.2002	0.513062		0.02	8.27		8.06	8.27	0.99	0.99	0.55			515	Chen YC et al.	2017	nal of Asian Earth Scie	145	424-445
467	TC-28	CAOB		83.5	47.05	Basalt		478	478	3.41	9.82	0.2019	0.513072		0.03	8.47		8.15	8.47	1.01	1.01	0.54			529	Chen YC et al.	2017	nal of Asian Earth Scie	145	424-445
468	TC-29	CAOB		83.5	47.05	Basalt		478	478	3.63	10.7	0.2017	0.513062		0.03	8.27		7.97	8.27	1.12	1.12	0.56			639	Chen YC et al.	2017	nal of Asian Earth Scie	145	424-445
469	TC-141	CAOB		83.5	47	Basalt		478	478	3.51	9.72	0.2172	0.513082		0.10	8.66		7.41	7.09	0.61	-3.00	0.61			131	Chen YC et al.	2017	nal of Asian Earth Scie	145	424-445
470	TC-143	CAOB		83.5	47	Basalt		478	478	3.68	10.4	0.2169	0.513085		0.10	8.72		7.49	7.17	0.60	-3.14	0.60			125	Chen YC et al.	2017	nal of Asian Earth Scie	145	424-445
471	BY04-4	CAOB	NE China	113	44	rhyolite	Sonidquozi	290	290	2.82	14.4	0.11819392	0.51282		-0.40	3.55		6.46	3.69	0.53	0.53	0.53			237	Zhang XH et al.	2011	nal of the Geological S	168	525-543
472	XJ22-8	CAOB	Eastern Junggar	90	46	andesite	Beitash	385	385	2.976	13.16	0.13648581	0.512839		-0.31	3.92		6.88	6.88	0.61	0.61	0.57			230	Zhang ZC et al.	2008	Gondwana Research	16	201-215
473	XJ22-12	CAOB	Eastern Junggar	90	46	basaltic andesite	Beitash	385	385	1.514	5.072	0.18016389	0.512943		-0.08	5.95		6.76	6.76	0.94	0.94	0.58			556	Zhang ZC et al.	2008	Gondwana Research	16	201-216
474	XJ22-10	CAOB	Eastern Junggar	90	46	basaltic andesite	Beitash	385	385	1.439	4.594	0.18905852	0.512995		-0.04	6.96		7.34	7.34	0.96	0.96	0.53			574	Zhang ZC et al.	2008	Gondwana Research	16	201-217
475	BT-9/1	CAOB		99	52			21.6	150	7.2	36.8	0.11808486	0.51283		-0.40	3.75		5.25	3.80	0.51	0.51	0.51			361	Yarmolyuk VV et al.	2003	Petrology	11(1)	1-30
476	EL-12/5	CAOB		99	52			14	150	6.1	31.8	0.11577296	0.51278		-0.41	2.77		4.32	3.06	0.58	0.58	0.59			427	Yarmolyuk VV et al.	2003	Petrology	11(1)	1-30
477	KHUB-4/28	CAOB		103.5	51.5			5.75	150	7.7	38.3	0.12133787	0.51278		-0.38	2.77		4.21	2.82	0.61	0.61	0.60			461	Yarmolyuk VV et al.	2003	Petrology	11(1)	1-30
478	UYU-1/3	CAOB		99	52			28	150	6.7	33.2	0.12179827	0.51278		-0.38	2.77		4.20	2.77	0.61	0.61	0.60			464	Yarmolyuk VV et al.	2003	Petrology	11(1)	1-30
479	EL-7/1	CAOB		103.5	51.5			4.8	150	6.4	30.9	0.12500487	0.51279		-0.36	2.97		4.34	3.13	0.62	0.62	0.59			469	Yarmolyuk VV et al.	2003	Petrology	11(1)	1-30
480	SHT-2/2	CAOB		99	52			0.3	150	7.4	35.3	0.12652095	0.51279		-0.36	2.97		4.31	3.22	0.63	0.63	0.59			480	Yarmolyuk VV et al.	2003	Petrology	11(1)	1-30
481	KHUB-9/2	CAOB		99	52			17.5	150	5.4	24.7	0.13194845	0.51281		-0.33	3.36		4.59	3.50	0.63	0.63	0.56			485	Yarmolyuk VV et al.	2003	Petrology	11(1)	1-30
482	KHUB-9/14	CAOB		99	52			29	150	5	22.8	0.1323557	0.51281		-0.33	3.36		4.59	3.48	0.64	0.64	0.56			488	Yarmolyuk VV et al.	2003	Petrology	11(1)	1-30
483	ZS-1/4	CAOB		99	52			18	150	6.2	30	0.12473083	0.51277		-0.37	2.57		3.95	2.72	0.65	0.65	0.62			502	Yarmolyuk VV et al.	2003	Petrology	11(1)	1-30
484	MO-1/10	CAOB		99	52			14.7	150	6.94	30.7	0.1364362	0.51282		-0.31	3.55		4.70	3.74	0.65	0.65	0.56			502	Yarmolyuk VV et al.	2003	Petrology	11(1)	1-30
485	ZS-1/12	CAOB		99	52			15.7	150	7.3	33.5	0.13151717	0.51278		-0.33	2.77		4.02	2.90	0.69	0.69	0.61			537	Yarmolyuk VV et al.	2003	Petrology	11(1)	1-30
486	KHUB-8/1	CAOB		99	52			25	150	10.7	51.3	0.12588319	0.51275		-0.36	2.18		3.54	2.31	0.69	0.69	0.65			545	Yarmolyuk VV et al.	2003	Petrology	11(1)	1-30
487	OK-5/1	CAOB		99	52			15.2	150	7.9	39.1	0.12194096	0.51273		-0.38	1.79		3.23	1.94	0.70	0.70	0.68			548	Yarmolyuk VV et al.	2003	Petrology	11(1)	1-30
488	OK-10/1	CAOB		99	52			14	150	7	33.9	0.12462313	0.51274		-0.37	1.99		3.37	2.19	0.70	0.70	0.66			552	Yarmolyuk VV et al.	2003	Petrology	11(1)	1-30
489	EL-13/3	CAOB		99	52			15	150	4.8	21.3	0.13600882	0.51279		-0.31	2.97		4.13	3.08	0.71	0.71	0.60			557	Yarmolyuk VV et al.	2003	Petrology	11(1)	1-30
490	DZH-11/1	CAOB		99	52			22	150	5.6	24.8	0.13628303	0.51279		-0.31	2.97		4.12	2.97	0.71	0.71	0.60			559	Yarmolyuk VV et al.	2003	Petrology	11(1)	1-30
491	OK-6/3	CAOB		99	52			15.2	150	6.9	33.1	0.12581181	0.51274		-0.36	1.99		3.35	2.00	0.71	0.71	0.67			562	Yarmolyuk VV et al.	2003	Petrology	11(1)	1-30
492	DE-1/9	CAOB		99	52			0.75	150	5.4	23.8	0.1369368	0.51277		-0.30	2.57		3.72	2.58	0.76	0.76	0.64			605	Yarmolyuk VV et al.	2003	Petrology	11(1)	1-30
493	EL-6/2 1.4	CAOB		103.5	51.5			1.4	150	7.4	33.6	0.13292105	0.51275		-0.32	2.18		3.41	2.18	0.76	0.76	0.66			605	Yarmolyuk VV et al.	2003	Petrology	11(1)	1-30
494	PL-1/5	CAOB		99	52			0.2	150	5.7	26.3	0.13080321	0.51273		-0.34	1.79		3.06	1.81	0.77	0.77	0.69			623	Yarmolyuk VV et al.	2003	Petrology	11(1)	1-30
495	OK-1/27	CAOB		99	52			0.01	150	5.9	26.8	0.13286712	0.51274		-0.32	1.99		3.21	2.01	0.77	0.77	0.68			624	Yarmolyuk VV et al.	2003	Petrology	11(1)	1-30
496	EL-13/3	CAOB		103.5	51.5			2	150	5.1	21.5	0.1431651	0.51279		-0.27	2.97		3.99	3.16	0.78	0.78	0.61			628	Yarmolyuk VV et al.	2003	Petrology	11(1)	1-30
497	DZH-8/1	CAOB		103.5	51.5			2.5	150	7.6	31.1	0.14748936	0.51281		-0.25	3.36		4.30	3.42	0.78	0.78	0.59			633	Yarmolyuk VV et al.	2003	Petrology	11(1)	1-30
498	NO-1/5	CAOB		103.5	51.5			28.7	150	7.5	33.3	0.13593095	0.51275		-0.31	2.18		3.35	2.30	0.78	0.78	0.67			634	Yarmolyuk VV et al.	2003	Petrology	11(1)	1-30
499	ZM-1/10	CAOB		99	52			9.8	150	6.6	31.7	0.12565502	0.51269		-0.36	1.01		2.37	1.02	0.80	0.80	0.75			647	Yarmolyuk VV et al.	2003	Petrology	11(1)	1-30
500	KHUB-22/5	CAOB		99	52			15.2	150	7.1	34.1	0.12566061	0.51269		-0.36	1.01		2.37	1.02	0.80	0.80	0.75			647	Yarmolyuk VV et al.	2003	Petrology	11(1)	1-30
501	SCH-1/4	CAOB		99	52			0.1	150	6.1	27.5	0.13387374	0.51272		-0.32	1.60		2.80	1.60	0.82	0.82	0.71			671	Yarmolyuk VV et al.	2003	Petrology	11(1)	1-30
502	EL-6/2 1	CAOB		103.5	51.5			1	150	5.6	24.2	0.13966063	0.51275		-0.29	2.18		3.28	2.19	0.82	0.82	0.67			674	Yarmolyuk VV et al.	2003	Petrology	11(1)	1-30
503	BE-1/12	CAOB		99	52			0.6	150	6.56	29.9	0.13241277	0.51271		-0.33	1.40		2.64	1.56	0.83	0.83	0.72			675	Yarmolyuk VV et al.	2003	Petrology	11(1)	1-30
504	UA-1/2	CAOB		99	52			0.5	150	6.6	29.8	0.13366722	0.51271		-0.32	1.40		2.61	1.49	0.84	0.84	0.73			688	Yarmolyuk VV et al.	2003	Petrology	11(1)	1-30
505	KHUB-18/1	CAOB		99	52			19	150	6.7	28.6	0.1413872	0.51275		-0.28	2.18		3.24	2.26	0.84	0.84	0.67			693	Yarmolyuk VV et al.	2003	Petrology	11(1)	1-30
506	KM-4/2	CAOB		99	52			11	150	5.1	20.8	0.1479828	0.51278		-0.25	2.77		3.70	2.78	0.86	0.86	0.64			708	Yarmolyuk VV et al.	2003	Petrology	11(1)	1-30
507	VS-1/5	CAOB		99	52			10	150	4.3	21.6	0.12014417	0.51262		-0.39	-0.35		1.11	-0.25	0.86	0.86	0.85			714	Yarmolyuk VV et al.	2003	Petrology	11(1)	1-30
508	KHTS-2/4	CAOB		103.5	51.5			2.4	150	8	30.4	0.15882759	0.51283		-0.19	3.75		4.47	3.79	0.89	0.89	0.57			739	Yarmolyuk VV et al.	2003	Petrology	11(1)	1-30
509	OK-7/1	CAOB		99	52			10.8	150	6.1	26.9	0.13685913	0.5127		-0.30	1.21		2.35	1.21	0.89	0.89	0.75			743	Yarmolyuk VV et al.	2003	Petrology	11(1)	1-30
510	KHUB-4/11	CAOB		99	52			10	150	6.2	26	0.14391917	0.51274		-0.27	1.99		3.00	1.99	0.90	0.90	0.69			746	Yarmolyuk VV et al.	2003	Petrology	11(1)	1-30
511	OK-1/1	CAOB		99	52			0.01	150	5.8	25.2	0.13890718	0.51271		-0.29	1.40		2.51	1.56	0.90	0.90	0.73			747	Yarmolyuk VV et al.	2003	Petrology	11(1)	1-30
512	KD-1/3	CAOB		99	52			0.6	150	5.9	24.4	0.14593636	0.51275		-0.26	2.18		3.16	2.22	0.90	0.90	0.68			750	Yarmolyuk VV et al.	2003	Petrology	11(1)	1-30
513	KHUB-4/20	CAOB		99	52			21.6	150	9.6	41.6	0.13927563	0.51271		-0.29	1.40		2.50	1.41	0.90	0.90	0.73			751	Yarmolyuk VV et al.	2003	Petrology	11(1)	1-30
514	OK-10/6	CAOB		99	52			4.75	150	5.5	24.2	0.13716475	0.51269		-0.30	1.01		2.15	1.02	0.92	0.92	0.76			766	Yarmolyuk VV et al.	2003	Petrology	11(1)	1-30
515	SHT-1/8	CAOB		99	52			0.1	150	5.3	23.6	0.13553705	0.51268		-0.31	0.82		1.99	0.85	0.92	0.92	0.78			767	Yarmolyuk VV et al.	2003	Petrology	11(1)	1-30
516	BE-3/4	CAOB		99	52			0.5	150	4.77	21.2	0.13579214	0.51266		-0.31	0.43		1.59	0.47	0.96	0.96	0.81			809	Yarmolyuk VV et al.	2003	Petrology	11(1)	1-30
517	EL-1/3	CAOB		103.5	51.5			4.3	150	5.9	25.8	0.1380144	0.51266		-0.30	0.43		1.55	0.44	0.99	0.99	0.81			837	Yarmolyuk VV et al.	2003	Petrology	11(1)	1-30

518	OK-7/5	CAOB		99	52			4.75	150	6.5	27.5	0.14265134	0.51269		-0.27	1.01		2.05	1.03	0.99	0.99	0.77			837	Yarmolyuk VV et al.	2003	Petrology	11(1)	1-30
519	BE-1/19	CAOB		99	52			2.1	150	5.83	23.9	0.14722036	0.51271		-0.25	1.40		2.35	1.41	1.01	1.01	0.75			859	Yarmolyuk VV et al.	2003	Petrology	11(1)	1-30
520	BE-1/4	CAOB		99	52			2.1	150	5.2	21.3	0.14733976	0.5127		-0.25	1.21		2.15	1.28	1.03	1.03	0.76			883	Yarmolyuk VV et al.	2003	Petrology	11(1)	1-30
521	BE-3/3	CAOB		99	52			0.5	150	4.8	19.5	0.14855892	0.51266		-0.24	0.43		1.35	0.48	1.15	1.15	0.83			996	Yarmolyuk VV et al.	2003	Petrology	11(1)	1-30
522	KHUB-12/4	CAOB		99	52			11.6	150	6.6	27	0.14752447	0.51258		-0.25	-1.13		-0.19	-0.97	1.31	1.31	0.95			1161	Yarmolyuk VV et al.	2003	Petrology	11(1)	1-30
523	KHUB-13/7	CAOB		99	52			8.9	150	6.8	28.4	0.14450118	0.51255		-0.27	-1.72		-0.72	-1.71	1.32	1.32	1.00			1170	Yarmolyuk VV et al.	2003	Petrology	11(1)	1-30
524	KHUB-2/4	CAOB		99	52			25	150	7.4	34.1	0.13096155	0.51241		-0.33	-4.45		-3.19	-4.43	1.36	1.36	1.20			1211	Yarmolyuk VV et al.	2003	Petrology	11(1)	1-30
525	P1-2/5	CAOB		99	52			1.7	150	5.3	19.3	0.1657352	0.5127		-0.16	1.21		1.80	1.30	1.43	1.43	0.79			1278	Yarmolyuk VV et al.	2003	Petrology	11(1)	1-30
526	BE-1/18	CAOB		99	52			1.7	150	4.85	17.6	0.16631265	0.5127		-0.15	1.21		1.79	1.24	1.45	1.45	0.79			1295	Yarmolyuk VV et al.	2003	Petrology	11(1)	1-30
527	KHUB-3/2	CAOB		99	52			22	150	6.7	29.7	0.13613746	0.51234		-0.31	-5.81		-4.65	-5.81	1.59	1.59	1.32			1439	Yarmolyuk VV et al.	2003	Petrology	11(1)	1-30
528	BOL-5/13	CAOB		103.5	51.5			6.8	150	10.3	51.1	0.12163317	0.51211		-0.38	-10.30		-8.86	-10.29	1.72	1.72	1.66			1568	Yarmolyuk VV et al.	2003	Petrology	11(1)	1-30
529	XT-29	CAOB	northern Tianshan	81.5	45	Adakite	Alataw	320	320	1.377	7.956	0.10445778	0.512753		-0.47	2.24		6.01	6.01	0.55	0.55	0.59			235	Wang Q et al.	2007	Chemical Geology	1(236)	42-64
530	XT-27	CAOB	northern Tianshan	81.5	45	Adakite	Alataw	320	320	1.709	8.077	0.12770318	0.512832		-0.35	3.78		6.60	6.60	0.56	0.56	0.54			244	Wang Q et al.	2007	Chemical Geology	1(236)	42-64
531	XT-30	CAOB	northern Tianshan	81.5	45	Adakite	Alataw	320	320	1.803	8.27	0.13158054	0.512751		-0.33	2.20		4.86	4.76	0.74	0.74	0.68			421	Wang Q et al.	2007	Chemical Geology	1(236)	42-64
532	XT-28	CAOB	northern Tianshan	81.5	45	Adakite	Alataw	320	320	2.722	12.43	0.13216332	0.512678		-0.33	0.78		3.42	0.80	0.88	0.88	0.80			563	Wang Q et al.	2007	Chemical Geology	1(236)	42-64
533	WXT-734	CAOB	northern Tianshan	81.8792	45.313	Dacite	Alataw	308	308	6.105	25.88	0.14236766	0.512639		-0.28	0.02		2.15	0.04	1.09	1.09	0.89			783	Wang Q et al.	2007	Chemical Geology	1(236)	42-64
534	AQS21	CAOB		90.8513	41.804	basalt		318	318	3.22	13.4	0.14625	0.512703	4	-0.26	3.32	0.81	5.37	1.01	1.01	1.01	0.81				Wang JW et al.	2022	e (In Chinese with Eng	47(9)	3229-3243
535	XJ11-150	CAOB		94.0058	41.65	gabbro		449	449	7.352	37.43	0.118737	0.512403	5	-0.40	-0.20	1.20	4.27	1.20	1.20	1.20	1.19				Li XF et al	2015	Acta petrologica Sinica	31(9)	2521-2538
536	WH2	CAOB		107.005	41.643	gabbro		320	320			0.193077	0.512821		-0.02	3.72	0.77	3.87	2.42	0.78	2.42	0.78				Zhang Y et al.	2020	(In Chinese with Engl	34(3)	450-465
537	WH6	CAOB		106.992	41.675	gabbro		325	325			0.18944	0.512663		-0.04	0.78	1.01	1.08	3.04	1.01	3.04	1.01				Zhang Y et al.	2020	(In Chinese with Engl	34(3)	450-465
538	KL1	CAOB		88.7533	46.083	gabbro		278	278	7.86	34.4	0.1331	0.512815		-0.32	5.73	0.63	7.99	0.63	0.63	0.63	0.64				Tang HJ et al.	2022	e (In Chinese with Eng	47(9)	3192-3209
539	HD	CAOB		94.675	42.175	mafic rocks		274	274	2.04	6.88	0.17925907	0.512919		-0.09	7.80	0.54	8.41	1.02	1.02	1.02	0.64				Qian ZZ et al.	2013	ology (In Chinese with	45(4)	145-154
540	Ak246	CAOB	Central Kazakhstan	71.5000	######	Hornblende gabbro		450	450	5.52	26.50	0.1253	0.512573		-0.36	-1.27	2.8	2.83	2.8	0.99	0.99	0.95				Heinhorst J et al.	2000	Tectonophysics	328(1)	69-87
541	ST2	CAOB	Central Kazakhstan	70.7800	######	Hornblende gabbro		450	450	4.49	18.80	0.1438	0.512485		-0.27	-2.98	0.1	0.05	0.1	1.45	1.45	1.18				Heinhorst J et al.	2000	Tectonophysics	328(1)	69-87
542	Do86	CAOB	Central Kazakhstan	77.8300	######	Gabbro		300	300	3.73	13.80	0.1578	0.512846		-0.20	4.06	5.5	5.55	5.5	0.83	0.83	0.61				Heinhorst J et al.	2000	Tectonophysics	328(1)	69-87
543	H19HX1	CAOB	China	118.636	44.749	gabbro		281	281	1.6	6.11	0.1583	0.51278	9	-0.20	2.8	4.2	4.15	4.15	1.02	1.02	0.71	1.01	0.64		Zhang XF et al.	2021	nal of Asian Earth Scie	5	100059
544	H19HX2	CAOB	China	118.636	44.749	gabbro		281	281	0.97	3.71	0.1581	0.512781	9	-0.20	2.8	4.2	4.17	4.17	1.01	1.01	0.71	1.02	0.64		Zhang XF et al.	2021	nal of Asian Earth Scie	5	100059
545	H19HX4	CAOB	China	118.636	44.749	gabbro		281	281	1.36	5.09	0.1615	0.512812	6	-0.18	3.4	4.7	4.66	4.66	0.99	0.99	0.67	0.99	0.61		Zhang XF et al.	2021	nal of Asian Earth Scie	5	100059
546	SMZM-02	CAOB	NE China	127.917	43.083	gabbro		262.1	262.1	7.02	30.9	0.1373	0.512251	8	-0.30	-7.5	-5.6	-5.56	-5.56	1.79	1.79	1.48	1.79	1.48		Liu S et al.	2010	Lithos	114(3-4)	423-436
547	SMZM-05	CAOB	NE China	127.917	43.083	gabbro		262.1	262.1	7.31	33.5	0.1319	0.512225	7	-0.33	-8.1	-5.9	-5.89	-5.89	1.72	1.72	1.51	1.72	1.51		Liu S et al.	2010	Lithos	114(3-4)	423-436
548	SMZM-08	CAOB	NE China	127.917	43.083	gabbro		262.1	262.1	6.57	31.9	0.1245	0.512234	10	-0.37	-7.9	-5.5	-5.47	-5.47	1.56	1.56	1.47	1.56	1.47		Liu S et al.	2010	Lithos	114(3-4)	423-436
549	SMZM-12	CAOB	NE China	127.917	43.083	gabbro		262.1	262.1	7.81	33.6	0.1405	0.512269	10	-0.29	-7.2	-5.3	-5.32	-5.32	1.83	1.83	1.46	1.83	1.46		Liu S et al.	2010	Lithos	114(3-4)	423-436
550	SMZM-13	CAOB	NE China	127.917	43.083	gabbro		262.1	262.1	7.6	33.2	0.1384	0.512245	8	-0.30	-7.7	-5.7	-5.72	-5.72	1.83	1.83	1.49	1.83	1.49		Liu S et al.	2010	Lithos	114(3-4)	423-436
551	SMZM-20	CAOB	NE China	127.917	43.083	gabbro		262.1	262.1	5.83	27.5	0.1282	0.512251	12	-0.35	-7.5	-5.3	-5.26	-5.26	1.60	1.60	1.46	1.60	1.46		Liu S et al.	2010	Lithos	114(3-4)	423-436
552	09-102/2**	CAOB	Russian	87.9667	53.217	monzogabbro	Terandzhik	248	248	9.3	56.8	0.0989	0.512637	2	-0.50	0.0	3.1	3.08	3.08	0.68	0.68	0.77	684.00	782		Wang YJ et al.	2016	Lithos	266-267	264-286
553	LTS-1	CAOB	NW China	95.2833	41.1	troctolite		282.6	282.6	1.38	4.69	0.179085	0.512835	8.0	-0.09	3.8	4.48	4.48	4.48	1.39	1.39	0.68				Ma BC et al.	2022	al of Geochemical Expl	233	106920
554	LTS-3	CAOB	NW China	95.2833	41.1	olivine clinopyroxenite		282.6	282.6	0.71	2.13	0.202774	0.512881	####	0.03	4.7	4.53	4.53	4.53	0.67	3.72	0.67				Ma BC et al.	2022	al of Geochemical Expl	233	106920
555	LTS-9	CAOB	NW China	95.2833	41.1	wehrlite		282.6	282.6	0.41	1.21	0.206230	0.512968	####	0.05	6.4	6.10	6.09	6.09	0.54	3.68	0.54				Ma BC et al.	2022	al of Geochemical Expl	233	106920
556	LTS-2	CAOB	NW China	95.2833	41.1	olivine gabbro		282.6	282.6	1.23	4.15	0.179971	0.512708	6.0	-0.09	1.4	1.97	1.97	1.97	1.99	1.99	0.88				Ma BC et al.	2022	al of Geochemical Expl	233	106920
557	LTS-7	CAOB	NW China	95.2833	41.1	gabbro		282.6	282.6	2.52	9.07	0.169101	0.512608	4.0	-0.14	-0.6	0.42	0.42	0.42	1.85	1.85	1.01				Ma BC et al.	2022	al of Geochemical Expl	233	106920
558	Rg61-1	CAOB	southern China	106.383	40.65	gabbro		282	282	5.22	22.4	0.1408	0.511573		-0.28	-20.8	-18.8	-18.76	-18.76	3.27	3.27	2.56	3.28	2.57		Zheng RG et al.	2019	Lithos	348-349	105174
559	Rg61-2	CAOB	southern China	106.383	40.65	gabbro		282	282	4.96	22	0.1362	0.511577		-0.31	-20.7	-18.5	-18.52	-18.52	3.07	3.07	2.54	3.08	2.55		Zheng RG et al.	2019	Lithos	348-349	105174
560	Rg61-3	CAOB	southern China	106.383	40.65	gabbro		282	282	4.65	21	0.1338	0.511568		-0.32	-20.9	-18.6	-18.61	-18.61	3.00	3.00	2.55	3.00	2.557		Zheng RG et al.	2019	Lithos	348-349	105174
561	Rg61-4	CAOB	southern China	106.383	40.65	gabbro		282	282	3.87	17.4	0.1344	0.51157		-0.32	-20.8	-18.6	-18.59	-18.59	3.02	3.02	2.55	3.02	2.555		Zheng RG et al.	2019	Lithos	348-349	105174
562	XJF-01	CAOB	Paleo-Asia Ocean	117.227	44.675	gabbro	Chaokeshan	354	354	2.09	6.59	0.193	0.513082		-0.02	8.7	8.8	8.83	8.83	0.50	0.50	0.39				Liu XJ et al.	2021	and Planetary Science	567	117006
563	XJF-02	CAOB	Paleo-Asia Ocean	117.227	44.675	gabbro	Chaokeshan	354	354	2.23	6.26	0.2165	0.512992		0.10	6.9	6.2	6.01	6.01	0.63	-8.88	0.63				Liu XJ et al.	2021	and Planetary Science	567	117006
564	XJF-03	CAOB	Paleo-Asia Ocean	117.227	44.675	gabbro	Chaokeshan	354	354	2.55	8.44	0.1841	0.513182		-0.06	10.6	11.1	11.18	11.18	0.19	-0.17	0.19				Liu XJ et al.	2021	and Planetary Science	567	117006
565	XJF-07	CAOB	Paleo-Asia Ocean	117.227	44.675	diabase	Chaokeshan	354	354	1.42	4.15	0.2085	0.513113		0.06	9.3	8.8	8.73	8.73	1.08	1.08	0.39				Liu XJ et al.	2021	and Planetary Science	567	117006
566	XJF-16	CAOB	Paleo-Asia Ocean	117.227	44.675	gabbro	Chaokeshan	354	354	3.55	9.65	0.2239	0.513187		0.14	10.7	9.7	9.48	9.48	0.55	0.55	0.33				Liu XJ et al.	2021	and Planetary Science	567	117006
567	XJF-20	CAOB	Paleo-Asia Ocean	117.227	44.675	gabbro	Chaokeshan	354	354	2.34	7.01	0.2029	0.513091		0.03	8.8	8.6	8.56	8.56	0.83	0.83	0.41				Liu XJ et al.	2021	and Planetary Science	567	117006
568	XJF-21	CAOB	Paleo-Asia Ocean	117.227	44.675	gabbro	Chaokeshan	354	354	2.7	8.01	0.2051	0.513148		0.04	9.9	9.7	9.57	9.57	0.33	0.04	0.33				Liu XJ et al.	2021	and Planetary Science	567	117006
569	XJF-22	CAOB	Paleo-Asia Ocean	117.227	44.675	gabbro	Chaokeshan	354	354	2.25	6.81	0.2012	0.513074		0.02	8.5	8.4	8.30	8.30	0.93	0.93	0.43				Liu XJ et al.	2021	and Planetary Science	567	117006
570	XJF-25	CAOB	Paleo-Asia Ocean	117.227	44.675	basalt	Chaokeshan	354	354	3.25	9.42	0.2099	0.513125		0.07	9.5	9	8.90	8.90	1.00	1.00	0.38				Liu XJ et al.	2021	and Planetary Science	567	117006
571	XJF-29	CAOB	Paleo-Asia Ocean	117.227	44.675	basalt	Chaokeshan	354	354	2.13	6.48	0.1997	0.513104		0.02	9.1	9	8.95	8.95	0.50	0.50	0.38				Liu XJ et al.	2021	and Planetary Science	567	117006
572	XJF-34	CAOB	Paleo-Asia Ocean	117.993	44.991	basalt	Hegenshan	354	354	3.63	14.59	0.1513	0.512882		-0.23	4.8	6.8	6.81	6.81	0.66	0.66	0.55				Liu XJ et al.	2021	and Planetary Science	567	117006
573	XJF-35	CAOB	Paleo-Asia Ocean	117.993	44.991	basalt	Hegenshan	354	354	7.17	28.18	0.1549	0.512873		-0.21	4.6	6.5	6.47	6.47	0.72	0.72	0.58				Liu XJ et al.	2021	and Planetary Science	567	117006
574	XJF-36	CAOB	Paleo-Asia Ocean	117.993	44.991	basalt	Hegenshan	354	354	6.24	25.3	0.15	0.512837		-0.24	3.9	6	5.99	5.99	0.75	0.75	0.62				Liu XJ et al.	2021	and Planetary Science	567	117006
575	XJF-39	CAOB	Paleo-Asia Ocean	117.993	44.991	gabbro	Hegenshan	354	354	2.35	6.9	0.2071	0.512973		0.05	6.5	6.1	6.07	6.07	0.61	4.05	0.61				Liu XJ et al.	2021	and Planetary Science	567	117006
576	XJF-40	CAOB	Paleo-Asia Ocean	117.993	44.991	gabbro	Hegenshan	354	354	4.13	11.47	0.2189	0.512978		0.11	6.6	5.6	5.63	5.63	0.66	-5.14	0.66				Liu XJ et al.	2021	and Planetary Science	567	117006
577	AE-33	CAOB	Paleo-Asia Ocean	88.3393	46.843	gabbro	Armantai	503	503	1.69	6.06	0.1701	0.51292		-0.14	5.5	7.2	7.21	7.21	0.80	0.80	0.64				Liu XJ et al.	2021	and Planetary Science	567	117006
578	AE-35	CAOB	Paleo-Asia Ocean	88.3393	46.843	gabbro	Armantai	503	503	1.94	7.07	0.1674	0.51289		-0.15	4.9	6.8	6.80	6.80	0.86	0.86	0.67				Liu XJ et al.	2021	and Planetary Science	567	117006
579	AE-53	CAOB	Paleo-Asia Ocean	88.3393	46.843	basalt	Armantai	503	503	2.95	11.4	0.1572	0.512902		-0.20	5.1	7.7	7.69	7.69	0.67	0.67	0.60				Liu XJ et al.	2021	and Planetary Science	567	117006
580	AE-55	CAOB	Paleo-Asia Ocean	88.3393	46.843	gabbro	Armantai	503	503	2.39	9.71	0.1501	0.512897		-0.24	5.1	8.1	8.04	8.04	0.61	0.61	0.57				Liu XJ et al.	2021	and Planetary Science	567	117006
581	AE-59	CAOB	Paleo-Asia Ocean	88.3393	46.843	basalt	Armantai	503	503	2.97	12.15	0.1487	0.512804		-0.24	3.2	6.3	6.32	6.32	0.81	0.81	0.71				Liu XJ et al.	2021	and Planetary Science	567	117006
582	AE-63	CAOB	Paleo-Asia Ocean	88.3393	46.843	basalt	Armantai	503	503	7.46	35.56	0.1276	0.512853		-0.35	4.2	8.6	8.63	8.63	0.53	0.53	0.52				Liu XJ et al.	2021	and Planetary Science	567	117006
583	XJF-44	CAOB	Paleo-Asia Ocean	113.51	42.677	basalt	Ondor Sam	624	600	1.38	4.05	0.2077	0.512967		0.06	6.4	5.5	5.58	5.58	0.85	4.59	0.85				Liu XJ et al.	2021	and Planetary Science	567	117006
584	XJF-46	CAOB	Paleo-Asia Ocean	113.51	42.677	basalt	Ondor Sam	624	600	1.55	4.29	0.2204	0.513067		0.12	8.4	6.5	6.55	6.55	0.78	-1.91	0.78				Liu XJ et al.	2021	and Planetary Science	567	117006
585	XJF-48	CAOB	Paleo-Asia Ocean	113.51	42.677	gabbro	Ondor Sam	624	600	0.33	0.87	0.2321	0.513047		0.18	8.0	5.1	5.27	5.27	0.88	-0.86	0.88				Liu XJ et al.	2021	and Planetary Science	567	117006
586	XJF-49	CAOB	Paleo-Asia Ocean	113.51	42.677	diabase	Ondor Sam	624	600	2.77	6.51	0.2589	0.513186		0.32	10.7	5.7	5.93	5.93	0.83	0.12	0.83				Liu XJ et al.	2021	and Planetary Science	567	117006
587	XJF-52	CAOB	Paleo-Asia Ocean	113.51	42.677	basalt	Ondor Sam	624	600	3	9.45	0.1932	0.512915		-0.02	5.4	5.7	5.67	5.67	1.74	1.74	0.84				Liu XJ et al.	2021	and Planetary Science	567	117006
588	XJF-54	CAOB	Paleo-Asia Ocean	113.043	42.51	basalt	Ulan Valley	425	425	3.89	13.73	0.1725	0.512849		-0.12	4.1	5.7	5.43	5.43	1.11	1.11	0.72				Liu XJ et al.	2021	and Planetary Science	567	117006
589	XJF-55	CAOB	Paleo-Asia Ocean	113.043	42.51	basalt	Ulan Valley	425	425	4.86	17.63	0.1676	0.512968		-0.15	6.4	8.3	8.02	8.02	0.60	0.60	0.51				Liu XJ et al.	2021	and Planetary Science	567	117006
590	XJF-57	CAOB	Paleo-Asia Ocean	113.043	42.51	basalt	Ulan Valley	425	425	2.85	10.02	0.173	0.512877		-0.12	4.7	6.2	5.95	5.95	1.02	1.02	0.68				Liu XJ et al.	2021	and Planetary Science	567	117006
591	XJF-59	CAOB	Paleo-Asia Ocean	113.043	42.51	basalt	Ulan Valley	425	425	4.09	14.49	0.1718	0.512874		-0.13	4.6	6.2	5.95	5.95	1.00	1.00	0.68				Liu XJ et al.	2021	and Planetary Science	567	117006
592	XJF-63	CAOB	Paleo-Asia Ocean	113.043	42.51	basalt	Ulan Valley	425	425	4.24	15.29	0.1687	0.512906		-0.14	5.2	7	6.75	6.75	0.83	0.83	0.61				Liu XJ et al.	2021	and Planetary Science	567	117006
593	XJF-64	CAOB	Paleo-Asia Ocean	113.043	42.51	basalt	Ulan Valley	425	425	4.13	14.92	0.1685	0.51293		-0.14	5.7	7.5	7.23	7.23	0.74	0.74	0.57				Liu XJ et al.	2021	and Planetary Science	567	117006
594	XJF-65	CAOB	Paleo-Asia Ocean	113.043	42.51	gabbro	Ulan Valley	425	425	2.17	6.55	0.2013	0.513111		0.02	9.2	8.9	8.98	8.98	0.48	0.48	0.43				Liu XJ et al.	2021	and Planetary Science	567	117006
595	BD-88	CAOB	Paleo-Asia Ocean	85.51	42.755	gabbro	Bindaban	425	425	4.5	15.44	0.1774	0.512866		-0.10	4.4	5.5	5.49	5.49	1.19	1.19	0.72				Liu XJ et al.	2021	and Planetary Science	567	117006
596	BD-89	CAOB	Paleo-Asia Ocean	85.51	42.755	gabbro	Bindaban	425	425	4.06	15.36	0.1609	0.51284		-0.18	3.9	5.9	5.88	5.88	0.90	0.90	0.68				Liu XJ et al.	2021	and Planetary Science	567	117006
597	BD-95	CAOB	Paleo-Asia Ocean	85.51	42.755	gabbro	Bindaban	425	425	4.58	16.09	0.1731	0.512947		-0.12	6.0	7.3	7.31	7.31	0.76	0.76	0.57				Liu XJ et al.	2021	and Planetary Science	567	117006
598	BD-96	CAOB	Paleo-Asia Ocean	85.51	42.755	gabbro	Bindaban	425	425	2.65	8.37	0.1925	0.512964		-0.02	6.4	6.6	6.59	6.59	1.34	1.34	0.63				Liu XJ et al.	2021	and Planetary Science	567	117006
599	BD-100	CAOB	Paleo-Asia Ocean	85.51	42.755	gabbro	Bindaban	425	425	2.53	8.61	0.1785	0.512925		-0.09	5.6	6.6	6.59	6.59	0.97	0.97	0.63				Liu XJ et al.	2021	and Planetary Science	567	117006
600	BD-88P	CAOB	Paleo-Asia Ocean	85.51	42.755	pyroxene	Bindaban	425	425	3.67	13.37	0.1668	0.512881		-0.15	4.7	6.4	6.36	6.36	0.87	0.87	0.65				Liu XJ et al.	2021	and Planetary Science	567	117006
601	BD-88F	CAOB	Paleo-Asia Ocean	85.51	42.755	plagioclase	Bindaban	425	425	1.59	6.21	0.1556	0.512904		-0.21	5.2	7.4	7.42	7.42	0.65	0.65	0.56				Liu XJ et al.	2021	and Planetary Science	567	117006
602	BD-89P	CAOB	Paleo-Asia Ocean	85.51	42.755	pyroxene	Bindaban	425	425	2.48	6.76	0.2229	0.513113		0.13	9.3	7.8	7.84	7.84	0.53	-0.62	0.53				Liu XJ et al.	2021	and Planetary Science	567	117006
603	BD-91P	CAOB	Paleo-Asia Ocean	85.51	42.755	pyroxene	Bindaban	425	425	2.14	7.39	0.1764	0.512921		-0.10	5.5	6.6	6.62	6.62	0.94	0.94	0.62				Liu XJ et al.	2021	and Planetary Science	567	117006
604	BD-95F	CAOB	Paleo-Asia Ocean	85.51	42.755	plagioclase	Bindaban	425	425	2.31	8.07	0.1739	0.512906		-0.12	5.2	6.5	6.46	6.46	0.93	0.93	0.64				Liu XJ et al.	2021	and Planetary Science	567	117006
605	BD-95P	CAOB	Paleo-Asia Ocean	85.51	42.755	pyroxene	Bindaban	425	425	2.36	7.12	0.2018	0.512983		0.03	6.7	6.5	6.45	6.45	2.13	2.13	0.64				Liu XJ et al.	2021	and Planetary Science	567	117006
606	BD-100F	CAOB	Paleo-Asia Ocean	85.51	42.755	plagioclase	Bindaban	425	425	2.37	8.22	0.175	0.512937		-0.11	5.8	7	7.01	7.01	0.84	0.84	0.59				Liu XJ et al.	2021	and Planetary Science	567	117006
607	IT-61-17	CAOB	central Kazakhstan	76.3554	46.803	porphyric basalt		470	470	6.05	23.67	0.1546	0.512134		-0.21	-9.8	-7.3	-7.31	-7.31	2.61	2.61	1.79				Safonova I et al.	2020	Gondwana Research	79	49-69
608	IT-62-17	CAOB	central Kazakhstan	76.3564	46.803	porphyric basalt		470	470	10.34	41.85	0.1494	0.512163		-0.24	-9.3	-6.4	-6.43	-6.43	2.33	2.33	1.72				Safonova I et al.	2020	Gondwana Research	79	49-69
609	IT-63-17	CAOB	central Kazakhstan	76.3566	46.802	aphyric basalt		470	470	10.6	43.27	0.148	0.512178		-0.25	-9.0	-6.1	-6.05	-6.05	2.25	2.25	1.69				Safonova I et al.	2020	Gondwana Research	79	49-69
610	IT-64-17	CAOB	central Kazakhstan	76.3567	46.802	aphyric basalt		470	470	9.21	43.11	0.1291	0.512211		-0.34	-8.3	-4.3	-4.28	-4.28	1.69	1.69	1.55				Safonova I et al.	2020	Gondwana Research	79	49-69
611	IT-22-17	CAOB	central Kazakhstan	76.3416	46.81	aphyric basalt		470	470	13.26	59.15	0.1355	0.512316		-0.31	-6.3	-2.6	-2.61	-2.61	1.62	1.62	1.41				Safonova I et al.	2020	Gondwana Research	79	49-69
612	IT-35-17	CAOB	central Kazakhstan	76.7412	46.718	basalt		450	450	3.94	12.62	0.1886	0.512921		-0.04	5.5	6	5.99	5.99	1.39	1.39	0.70				Safonova I et al.	2020	Gondwana Research	79	49-69
613	IT-76-17	CAOB	central Kazakhstan	76.2759	46.874	porphyric basalt		480	480	5.14	15.53	0.1998	0.513022		0.02	7.5	7.3	7.30	7.30	1.40	1.40	0.61				Safonova I et al.	2020	Gondwana Research	79	49-69
614	IT-79-17	CAOB	central Kazakhstan	76.2848	46.87	aphyric basalt		480	480	5.42	16.76	0.1955	0.513026		-0.01	7.6	7.7	7.64	7.64	1.04	1.04	0.59				Safonova I et al.	2020	Gondwana Research	79	49-69

615	IT-80-17	CAOB	central Kazakhstan	76.2896	46.875	gabbro-dolerite		480	480	4.15	12.37	0.2026	0.513027		0.03	7.6	7.2	7.23	7.23	1.69	1.69	0.62				Safonova I et al.	2020	Gondwana Research	79	49-69
616	IT-73-17	CAOB	central Kazakhstan	76.3048	46.826	gabbro-dolerite		480	480	3.69	13.63	0.1636	0.512773		-0.17	2.6	4.7	4.66	4.66	1.15	1.15	0.83				Safonova I et al.	2020	Gondwana Research	79	49-69
617	17081902	CAOB	central Kazakhstan	76.3046	46.825	aphyric basalt		470	470	3.75	10.93	0.2072	0.513069		0.05	8.4	7.8	7.78	7.78	1.89	1.89	0.57				Safonova I et al.	2020	Gondwana Research	79	49-69
618	NX-14	CAOB	central Kazakhstan	76.3702	46.803	aphyric basalt		470	470	0.34	1.24	0.1651	0.512789		-0.16	2.9	4.9	4.84	4.84	1.13	1.13	0.81				Safonova I et al.	2020	Gondwana Research	79	49-69
619	BET19-29-02	CAOB	NW China	140.309	46.838	Gabbro		483	483	3.77	10.8	0.21103622	0.513187	5	0.07	10.7	9.8	9.83	9.83	0.41	-2.14	0.41		412		Yang YQ et al.	2022	Gondwana Research	111	121-141
620	BET20-06-1	CAOB	NW China	86.573	46.805	Pillow lava		472	472	1.99	5.53	0.21755414	0.513211	8	0.11	11.2	9.9	9.92	9.92	2.40	2.40	0.39		396		Yang YQ et al.	2022	Gondwana Research	111	121-141
621	BET20-01	CAOB	NW China	86.573	46.805	Gabbro		467	467	0.58	1.34	0.26167522	0.513189	6	0.33	10.7	6.9	6.88	6.88	0.64	0.12	0.64		641		Yang YQ et al.	2022	Gondwana Research	111	121-141
622	BET20-03	CAOB	NW China	86.5758	46.806	Gabbro		472	472	0.92	2.4	0.231748	0.513162	6	0.18	10.2	8.1	8.11	8.11	0.54	0.10	0.54		548		Yang YQ et al.	2022	Gondwana Research	111	121-141
623	BET20-04	CAOB	NW China	86.5852	46.804	Gabbro		472	472	0.35	0.79	0.26784304	0.513216	5	0.36	11.3	6.9	6.99	6.99	0.63	0.19	0.63		641		Yang YQ et al.	2022	Gondwana Research	111	121-141
624	BET19-11-02	CAOB	NW China	86.5852	46.804	Basalt		483	483	3.03	8.9	0.20582211	0.513119	5	0.05	9.4	8.8	8.82	8.82	0.60	0.60	0.49		495		Yang YQ et al.	2022	Gondwana Research	111	121-141
625	BET19-31-03	CAOB	NW China	86.5852	46.804	Andesite		456	456	3.4	15.85	0.12968479	0.512767	5	-0.34	2.5	6.4	6.42	6.42	0.70	0.70	0.67		669		Yang YQ et al.	2022	Gondwana Research	111	121-141
626	BET19-04-02	CAOB	NW China	86.5842	46.804	Andesite		456	456	4.25	18.72	0.13725321	0.512786	5	-0.30	2.9	6.3	6.35	6.35	0.73	0.73	0.67		675		Yang YQ et al.	2022	Gondwana Research	111	121-141
627	BET18-06-01	CAOB	NW China	86.575	46.808	Basaltic andesite		452	452	3.23	12.62	0.15473287	0.512861	6	-0.21	4.4	6.8	6.77	6.77	0.75	0.75	0.63		635		Yang YQ et al.	2022	Gondwana Research	111	121-141
628	EDG-3-YQ1	CAOB	NE China	123.66	42.576	Gabbro		350	350	6.25	24.96	0.151384	0.512759		-0.23	2.4	4.39	4.38	4.38	0.96	0.96	0.74	961.00	748		Shi Y et al.	2022	Gondwana Research	103	64-83
629	EDG-3-YQ2	CAOB	NE China	123.56	42.659	Gabbro		350	350	6.7	27.38	0.147876	0.512789		-0.25	2.9	5.15	5.13	5.13	0.84	0.84	0.68	839.00	686		Shi Y et al.	2022	Gondwana Research	103	64-83
630	EDG-3-YQ3	CAOB	NE China	123.56	42.659	Gabbro		350	350	5.31	21.31	0.150737	0.512732		-0.23	1.8	3.9	3.89	3.89	1.01	1.01	0.79	1015.00	787		Shi Y et al.	2022	Gondwana Research	103	64-83
631	EDG-3-YQ5	CAOB	NE China	123.56	42.659	Gabbro		350	350	3.35	13.44	0.150734	0.51275		-0.23	2.2	4.25	4.24	4.24	0.97	0.97	0.76	972.00	759		Shi Y et al.	2022	Gondwana Research	103	64-83
632	PM201-4-YQ3	CAOB	NE China	123.63	42.555	Gabbro		256	256	4.74	21.22	0.134986	0.512834		-0.31	3.8	5.83	5.84	5.84	0.61	0.61	0.55	615.00	551		Shi Y et al.	2022	Gondwana Research	103	64-83
633	PM201-4-YQ4	CAOB	NE China	123.63	42.555	Gabbro		256	256	4.46	20.62	0.130646	0.512839		-0.34	3.9	6.08	6.08	6.08	0.57	0.57	0.53	574.00	532		Shi Y et al.	2022	Gondwana Research	103	64-83
634	PM201-4-YQ5	CAOB	NE China	123.63	42.555	Gabbro		256	256	4.41	19.58	0.136165	0.512836		-0.31	3.9	5.85	5.84	5.84	0.62	0.62	0.55	620.00	551		Shi Y et al.	2022	Gondwana Research	103	64-83
635	PM201-3-YQ1	CAOB	NE China	123.63	42.555	Basalt		249	249	6.77	31.12	0.13147	0.512725		-0.33	1.7	3.78	3.77	3.77	0.79	0.79	0.71	791.00	715		Shi Y et al.	2022	Gondwana Research	103	64-83
636	PM201-3-YQ2	CAOB	NE China	123.63	42.555	Basalt		249	249	5.08	24.38	0.12602	0.512705		-0.36	1.3	3.54	3.55	3.55	0.77	0.77	0.73	777.00	733		Shi Y et al.	2022	Gondwana Research	103	64-83
637	PM201-3-YQ4	CAOB	NE China	123.63	42.555	Basalt		249	249	5.88	26.18	0.135829	0.51273		-0.31	1.8	3.74	3.73	3.73	0.82	0.82	0.72	826.00	718		Shi Y et al.	2022	Gondwana Research	103	64-83
638	09–102/2	CAOB	Russian	84.8333	50.85	monzogabbro		248	248	9.3	56.8	0.0989	0.512637		-0.50	0.0	3.1	3.08	3.08	0.68	0.68	0.77	0.68	0.782		Olga A et al.	2019	Lithos	326-327	71-89
639	1761	CAOB	Mongolia	90.0045	54.004	Gabbro	ashpar mass	487	487	4.91	26.67	0.111291	0.512538	13	-0.43	-2.0	3.52	3.36	3.36	0.91	0.91	0.94				Vrublevskii VV et al	2018	ian Geology and Geopl	59(7)	718-744
640	Jan-65	CAOB	Mongolia	90.0045	54.015	Gabbro	iskach mass	483	483	9.15	41.78	0.132441	0.512661	15	-0.33	0.4	4.55	4.41	4.41	0.92	0.92	0.85				Vrublevskii VV et al	2018	ian Geology and Geopl	59(7)	718-744
641	13WL-6	CAOB	NW China	85.0023	43.004	Hornblende gabbro	ulale Mounta	313	313	1.2	3.6	0.205	0.512845	6	0.04	4.0	3.7	3.71	3.71	0.76	5.27	0.76		0.772		Yan S et al.	2015	nal of Asian Earth Scie	113	218-237
642	13WL-7	CAOB	NW China	85.0023	43.004	Hornblende gabbro	ulale Mounta	313	313	1.7	6.1	0.171	0.512758	4	-0.13	2.3	3.4	3.37	3.37	1.40	1.40	0.80		0.8		Yan S et al.	2015	nal of Asian Earth Scie	113	218-237
643	13WL-8	CAOB	NW China	85.0023	43.004	Hornblende gabbro	ulale Mounta	313	313	1.3	4.5	0.173	0.512749	5	-0.12	2.2	3.1	3.11	3.11	1.50	1.50	0.82		0.82		Yan S et al.	2015	nal of Asian Earth Scie	113	218-237
644	13WL-9	CAOB	NW China	85.0023	43.004	Hornblende gabbro	ulale Mounta	313	313	1.1	4.4	0.15	0.512688	5	-0.24	1.0	2.8	2.84	2.84	1.10	1.10	0.84		0.841		Yan S et al.	2015	nal of Asian Earth Scie	113	218-237
645	13WL-10	CAOB	NW China	85.0023	43.004	Hornblende gabbro	ulale Mounta	313	313	1.6	5.4	0.176	0.512778	4	-0.11	2.7	3.6	3.56	3.56	1.50	1.50	0.78		0.783		Yan S et al.	2015	nal of Asian Earth Scie	113	218-237
646	13WL-11	CAOB	NW China	85.0023	43.004	Hornblende gabbro	ulale Mounta	313	313	1.9	6.4	0.179	0.5128	5	-0.09	3.2	3.9	3.87	3.87	1.53	1.53	0.76		0.759		Yan S et al.	2015	nal of Asian Earth Scie	113	218-237
647	13WL-12	CAOB	NW China	85.0023	43.004	Hornblende gabbro	ulale Mounta	313	313	2.9	10	0.172	0.512761	4	-0.13	2.4	3.4	3.39	3.39	1.42	1.42	0.79		0.796		Yan S et al.	2015	nal of Asian Earth Scie	113	218-237
648	13WL-13	CAOB	NW China	85.0023	43.004	Hornblende gabbro	ulale Mounta	313	313	2.4	8.2	0.18	0.512776	4	-0.08	2.7	3.4	3.36	3.36	1.69	1.69	0.80		0.8		Yan S et al.	2015	nal of Asian Earth Scie	113	218-237
649	XCJ04-3	CAOB	China	99.1667	41.417	Pegamtoid gabbro	Ophiolite	516	516	0.46	1.39	0.2	0.513094	10	0.02	8.9	8.5	8.68	8.68	0.62	0.62	0.53				Shi YR et al.	2018	nal of Asian Earth Scie	153	193-205
650	HLH14-1	CAOB	China	99.1667	41.417	Gabbro	Ophiolite	516	516	1.01	3.06	0.2	0.513017	9	0.02	7.4	7.2	7.18	7.18	1.48	1.48	0.65				Shi YR et al.	2018	nal of Asian Earth Scie	153	193-205
651	YYSS02-2	CAOB	China	99.1667	41.417	Gabbro	Ophiolite	516	516	2.31	5.011	0.28	0.513277	7	0.42	12.5	8.2	6.98	6.98	0.67	0.29	0.67				Shi YR et al.	2018	nal of Asian Earth Scie	153	193-205
652	XHS10	CAOB	China	99.1667	41.417	Gabbro	Ophiolite	516	516	0.11	0.37	0.17	0.512866	8	-0.14	4.4	6.1	6.21	6.21	0.99	0.99	0.73				Shi YR et al.	2018	nal of Asian Earth Scie	153	193-205
653	15JL01-B	CAOB	NE China	126.303	43.673	Hornblende gabbro	Zhangjiatun	486	486	1.23	3.96	0.18747	0.51299		-0.05	6.9	7.4	7.44	7.44	0.93	0.93	0.61	0.94			Ma HT et al.	2020	nal of Asian Earth Scie	194	104182
654	15JL01-C	CAOB	NE China	126.303	43.673	Hornblende gabbro	Zhangjiatun	486	486	1.23	4.01	0.18524	0.51299		-0.06	6.9	7.6	7.58	7.58	0.86	0.86	0.60	0.86			Ma HT et al.	2020	nal of Asian Earth Scie	194	104182
655	15JL02-A	CAOB	NE China	126.26	43.687	Quartz diorite	Zhangjiatun	476	476	1.41	5.16	0.16509	0.51287		-0.16	4.5	6.5	6.45	6.45	0.88	0.88	0.68	0.88			Ma HT et al.	2020	nal of Asian Earth Scie	194	104182
656	15JL02-C	CAOB	NE China	126.313	43.68	Tonalite	Zhangjiatun	440	440	0.45	2.49	0.10963	0.51256		-0.44	-1.5	3.3	3.37	3.37	0.86	0.86	0.90	0.87			Ma HT et al.	2020	nal of Asian Earth Scie	194	104182
657	wxt772	CAOB	NE China	86.175	42.883	gabbros	Luotuogou	312	312	4.22	13.6	0.1891724	0.512866	7	-0.04	4.4	4.7	4.75	4.75	1.76	1.76	0.68				Tang GJ et al.	2012	Lithos	155	67-80
658	wxt774	CAOB	NE China	86.2	42.883	gabbros	Luotuogou	312	312	3.63	12.5	0.1770693	0.512891	9	-0.10	4.9	5.7	5.72	5.72	1.08	1.08	0.60				Tang GJ et al.	2012	Lithos	155	67-80
659	wxt776	CAOB	NE China	86.2	42.883	gabbros	Luotuogou	312	312	5.21	19.3	0.164138	0.512899	7	-0.17	5.1	6.4	6.39	6.39	0.77	0.77	0.55				Tang GJ et al.	2012	Lithos	155	67-80
660	wxt783	CAOB	NE China	86.3	42.883	gabbros	Luotuogou	312	312	2.66	8.94	0.1809477	0.513006	6	-0.08	7.2	7.8	7.81	7.81	0.67	0.67	0.43				Tang GJ et al.	2012	Lithos	155	67-80
661	wxt784	CAOB	NE China	86.3	42.883	gabbros	Luotuogou	312	312	2.58	9.25	0.169561	0.512934	6	-0.14	5.8	6.9	6.85	6.85	0.75	0.75	0.51				Tang GJ et al.	2012	Lithos	155	67-80
662	wxt785	CAOB	NE China	86.3	42.8	gabbros	Luotuogou	312	312	1.33	4.73	0.1708232	0.512845	12	-0.13	4.0	5.1	5.07	5.07	1.08	1.08	0.66				Tang GJ et al.	2012	Lithos	155	67-80
663	wxt787	CAOB	NE China	86.3	42.8	gabbros	Luotuogou	312	312	3.69	14.4	0.1560275	0.512665	6	-0.21	0.5	2.1	2.15	2.15	1.28	1.28	0.90				Tang GJ et al.	2012	Lithos	155	67-80
664	wxt789	CAOB	NE China	86.3	42.8	gabbros	Luotuogou	312	312	1.86	7.19	0.1576582	0.51279	7	-0.20	3.0	4.5	4.52	4.52	0.98	0.98	0.70				Tang GJ et al.	2012	Lithos	155	67-80
665	14LQ07A	CAOB	NE China	104.92	39.925	Hbl gabbro	Alxa Terrane	300	300	1.32	5.01	0.1592	0.512189	9	-0.19	-8.8	-7.3	-7.32	-7.32	2.67	2.67	1.65				Liu Q et al.	2017	Lithos	274-275	19-30
666	14LQ07D	CAOB	NE China	104.92	39.925	Gabbro	Alxa Terrane	300	300	0.64	2.25	0.1711	0.512102	26	-0.13	-10.5	-9.5	-9.48	-9.48	1.82	3.72	1.82				Liu Q et al.	2017	Lithos	274-275	19-30
667	14LQ07F	CAOB	NE China	104.92	39.925	Hbl gabbro	Alxa Terrane	300	300	0.61	3.49	0.1063	0.512098	20	-0.46	-10.5	-7.1	-7.07	-7.07	1.49	1.49	1.64				Liu Q et al.	2017	Lithos	274-275	19-30
668	14LQ10B	CAOB	NE China	104.842	39.959	Hbl gabbro	Alxa Terrane	279	279	10.1	54.1	0.1128	0.512113	7	-0.43	-10.2	-7.3	-7.25	-7.25	1.56	1.56	1.63				Liu Q et al.	2017	Lithos	274-275	19-30
669	EL14-17-3	CAOB	NE China	111.208	43.133	Syenogabbro	Erenhot	141	150	9.679	53.31	0.1099	0.512686	10	-0.44	0.9	2.5	2.60	2.60	0.68	0.68	0.73				Yuan LL et al.	2019	Lithos	324-325	859-876
670	EL12-1-1	CAOB	NE China	111.233	43.133	Syenogabbro	Erenhot	141	150	3.249	18.51	0.1063	0.512591	12	-0.46	-0.9	0.7	0.81	0.81	0.79	0.79	0.87				Yuan LL et al.	2019	Lithos	324-325	859-876
671	EL12-1-3	CAOB	NE China	111.233	43.133	Syenogabbro	Erenhot	141	150	4.111	23.53	0.1058	0.51262	11	-0.46	-0.4	1.3	1.39	1.39	0.75	0.75	0.83				Yuan LL et al.	2019	Lithos	324-325	859-876
672	EL12-1-4	CAOB	NE China	111.233	43.133	Syenogabbro	Erenhot	141	150	3.339	20.78	0.0973	0.512616	11	-0.51	-0.4	1.4	1.47	1.47	0.70	0.70	0.82				Yuan LL et al.	2019	Lithos	324-325	859-876
673	EL12-2-1	CAOB	NE China	111.233	43.133	Biotite monzonite	Erenhot	141	150	22.2	119.5	0.1125	0.512651	10	-0.43	0.3	1.8	1.87	1.87	0.75	0.75	0.79				Yuan LL et al.	2019	Lithos	324-325	859-876
674	EL12-2-2	CAOB	NE China	111.233	43.133	Biotite monzonite	Erenhot	142	150	15.22	87.32	0.1055	0.512623	12	-0.46	-0.3	1.4	1.45	1.45	0.74	0.74	0.82				Yuan LL et al.	2019	Lithos	324-325	859-876
675	EL14-15-1	CAOB	NE China	111.242	43.165	Quartz monzonite	Erenhot	142	150	11	63.66	0.1046	0.512565	11	-0.47	-1.4	0.2	0.34	0.34	0.82	0.82	0.91				Yuan LL et al.	2019	Lithos	324-325	859-876
676	R-94-1	CAOB	Russia	131.8	50.667	Amphibolite	chikha Com	950	600	11.85	47.61	0.1505	0.512365	2	-0.23	-5.3	0.3	-1.80	-1.80	1.89	1.89	1.45				Sorokin AA et al.	2019	Lithos	332-333	340-354
677	C-1214	CAOB	Russia	131.217	51.033	Amphibolite	chikha Com	950	600	6.22	32.3	0.1164	0.512161	2	-0.41	-9.3	0.5	-3.15	-3.15	1.55	1.55	1.56				Sorokin AA et al.	2019	Lithos	332-333	340-354
678	C-1214-1	CAOB	Russia	131.217	51.033	Amphibolite	chikha Com	950	600	7.34	36.8	0.1207	0.512168	1	-0.39	-9.2	0.1	-3.34	-3.34	1.61	1.61	1.58				Sorokin AA et al.	2019	Lithos	332-333	340-354
679	R-93	CAOB	Russia	131.8	50.667	Amphibolite	chikha Com	950	600	5.07	22.2	0.1379	0.512187	2	-0.30	-8.8	-1.7	-4.30	-4.30	1.93	1.93	1.65				Sorokin AA et al.	2019	Lithos	332-333	340-354
680	X-1280	CAOB	Eastern Kazakhstan	131.217	51.033	gabbronorite		312	312	3.494	12.444	0.16974	0.512987	8	-0.14	6.8	7.89	7.88	7.88	0.57	0.57	0.43				Sorokin AA et al.	2019	Lithos	332-333	340-354
681	X-1147	CAOB	Eastern Kazakhstan	84.4748	48.441	gabbronorite		317	317	3.14	12.888	0.147259	0.512833	6	-0.25	3.8	5.81	5.80	5.80	0.73	0.73	0.60				Sorokin AA et al.	2019	Lithos	332-333	340-354
682	X-969	CAOB	Eastern Kazakhstan	82.8527	49.826	gabbronorite		313	313	4.737	19.415	0.147486	0.512641	16	-0.25	0.1	2.02	2.02	2.02	1.17	1.17	0.91				Sorokin AA et al.	2019	Lithos	332-333	340-354
683	X-959	CAOB	Eastern Kazakhstan	82.7704	49.855	leucogabbro		313	313	0.86	1.329	0.175361	0.512901	31	-0.11	5.1	5.98	5.98	5.98	0.99	0.99	0.58				Sorokin AA et al.	2019	Lithos	332-333	340-354
684	M-13-106	CAOB	CentralAsian	101.062	50.029	Dolerite	ayangol Rive	498.9	498.9	3.53	14.28	0.1506	0.512459		-0.23	-3.5	-0.5	-0.56	-0.56	1.67	1.67	1.27				Blyaev VA et al.	2021	Lithos	386-387	106044
685	M-13-107	CAOB	CentralAsian	101.062	50.029	Lamproph	ayangol Rive	498.9	498.9	5.06	21.95	0.1403	0.512467		-0.29	-3.3	0.3	0.25	0.25	1.42	1.42	1.20				Blyaev VA et al.	2021	Lithos	386-387	106044
686	M-13-08	CAOB	CentralAsian	101.06	50.027	Gabbro	ayangol Rive	498.9	498.9	7.42	38.1	0.1186	0.512371		-0.40	-5.2	-0.2	-0.24	-0.24	1.25	1.25	1.24				Blyaev VA et al.	2021	Lithos	386-387	106044
687	M-13-125	CAOB	CentralAsian	101.059	50.028	Lamproph	ayangol Rive	498.9	498.9	12.63	69.66	0.1104	0.512318		-0.44	-6.2	-0.7	-0.75	-0.75	1.23	1.23	1.28				Blyaev VA et al.	2021	Lithos	386-387	106044
688	M-13-129	CAOB	CentralAsian	101.058	50.028	Gabbro	ayangol Rive	498.9	498.9	3.46	13.6	0.1549	0.512628		-0.21	-0.2	2.5	2.47	2.47	1.35	1.35	1.02				Blyaev VA et al.	2021	Lithos	386-387	106044
689	M-13-129	CAOB	CentralAsian	101.058	50.028	Gabbro	ayangol Rive	498.9	498.9	3.68	14.46	0.1551	0.51262		-0.21	-0.4	2.3	2.30	2.30	1.38	1.38	1.04				Blyaev VA et al.	2021	Lithos	386-387	106044
690	NM-190③	CAOB	China	116.249	43.843	Mafic rocks		275	275	4.1	16	0.1532	0.512727		-0.22	1.7	3.3	3.26	3.26	1.07	1.07	0.77				Zhu JB	2022	Lithos	424-425	106765
691	NM-193③	CAOB	China	116.249	43.843	Mafic rocks		275	275	6.3	23.4	0.1619	0.512735		-0.18	1.9	3.1	3.11	3.11	1.22	1.22	0.79				Zhu JB	2022	Lithos	424-425	106765
692	NM-194①	CAOB	China	116.249	43.875	Mafic rocks		323	323	2.3	9.8	0.1339	0.512771		-0.32	2.6	4.9	5.18	5.18	0.72	0.72	0.66				Zhu JB	2022	Lithos	424-425	106765
693	NM-194②	CAOB	China	116.249	43.875	Mafic rocks		323	323	3.1	10.9	0.1697	0.51296		-0.14	6.3	7.4	7.39	7.39	0.66	0.66	0.48				Zhu JB	2022	Lithos	424-425	106765
694	ZK001-21	CAOB	northwestern China	95.9933	42.638	Gabbro	rnTianshanT	427	427	2	9.5	0.1293	0.5128		-0.34	3.2	6.3	6.83	6.83	0.63	0.63	0.61				He XH et al.	2021	Ore Geology Reviews	135	104228
695	SDHS-17	CAOB	northwestern China	96.0333	42.633	Gabbro	rnTianshanT	406	406	3.3	13.9	0.1424	0.5128		-0.28	3.2	5.4	5.97	5.97	0.75	0.75	0.66				He XH et al.	2021	Ore Geology Reviews	135	104228
696	SDHS-18	CAOB	northwestern China	95.995	42.643	Gabbro	rnTianshanT	406	406	1.1	5.1	0.1326	0.5127		-0.33	1.2	4.7	4.53	4.53	0.85	0.85	0.78				He XH et al.	2021	Ore Geology Reviews	135	104228
697	SDHS-19	CAOB	northwestern China	95.995	42.643	Gabbro	rnTianshanT	406	406	1.8	7.5	0.1466	0.5128		-0.25	3.2	5.5	5.76	5.76	0.80	0.80	0.68				He XH et al.	2021	Ore Geology Reviews	135	104228
698	SDHS-1	CAOB	northwestern China	95.995	42.643	Gabbro	rnTianshanT	397	397	2.2	7.3	0.1773	0.5129		-0.10	5.1	6.9	6.09	6.09	1.05	1.05	0.64				He XH et al.	2021	Ore Geology Reviews	135	104228
699	17Y01-2	CAOB	NW China	91.75	42.667	gabbro	Kalatagearea	265	265	1.706	6.611	0.156021	0.512949		-0.21	6.1	7.44	7.44	7.44	0.53	0.53	0.43				Mao QG et al.	2021	Ore Geology Reviews	136	104258
700	17Y01-6	CAOB	NW China	91.75	42.667	gabbro	Kalatagearea	265	265	1.901	7.655	0.150144	0.512956		-0.24	6.2	7.78	7.78	7.78	0.47	0.47	0.40				Mao QG et al.	2021	Ore Geology Reviews	136	104258
701	17Y04-1	CAOB	NW China	91.75	42.667	gabbro	Kalatagearea	265	265	0.872	3.727	0.141458	0.512949		-0.28	6.1	7.94	7.93	7.93	0.42	0.42	0.39				Mao QG et al.	2021	Ore Geology Reviews	136	104258
702	17Y02-4	CAOB	NW China	91.75	42.667	olivine	Kalatagearea	273	273	0.841	3.45	0.147384	0.51296		-0.25	6.3	8.01	8.00	8.00	0.44	0.44	0.39				Mao QG et al.	2021	Ore Geology Reviews	136	104258
703	17Y03-3	CAOB	NW China	91.75	42.667	leucogabbro	Kalatagearea	265	265	2.25	8.459	0.160819	0.512965		-0.18	6.4	7.63	7.59	7.59	0.53	0.53	0.41				Mao QG et al.	2021	Ore Geology Reviews	136	104258
704	17Y03-4	CAOB	NW China	91.75	42.667	leucogabbro	Kalatagearea	265	265	1.443	5.318	0.164057	0.512994		-0.17	6.9	8.06	8.05	8.05	0.48	0.48	0.38				Mao QG et al.	2021	Ore Geology Reviews	136	104258
705	BY04-2	CAOB	Inner Mongolia	113.46	44.13	Mafic volcanic rock	Sonidzuoqi	287	287	8.294	35.1	0.1432	0.5129	13	-0.27	5.1	6.79	7.07	7.07	0.54	0.54	0.47	0.55			Zhang XH et al.	2011	nal of the Geological S	168	525-543
706	BY04-14	CAOB	Inner Mongolia	113.187	44.23	Mafic volcanic rock	Sonidzuoqi	287	287	8.93	42.1	0.1283	0.5127	15	-0.35	1.2	3.79	3.71	3.71	0.80	0.80	0.75	0.80			Zhang XH et al.	2011	nal of the Geological S	168	525-543
707	SN07-2	CAOB	Inner Mongolia	113.23	42.17	Mafic volcanic rock	Sonidzuoqi	287	287	6.72	33.3	0.122	0.51285	14	-0.38	4.1	6.8	6.87	6.87	0.50	0.50	0.49	0.51			Zhang XH et al.	2011	nal of the Geological S	168	525-543
708	SN07-10	CAOB	Inner Mongolia	110.27	44.19	Mafic volcanic rock	Sonidzuoqi	287	287	5.01	26.1	0.116	0.51273	15	-0.41	1.8	4.67	4.75	4.75	0.66	0.66	0.66	0.66			Zhang XH et al.	2011	nal of the Geological S	168	525-543

709	SN07-30	CAOB	Inner Mongolia	113.22	44.27	Mafic volcanic rock	Sonidzuoqi	287	287	3.96	20.1	0.119	0.51275	12	-0.40	2.2	5.01	5.03	5.03	0.64	0.64	0.64	0.65			Zhang XH et al.	2011	al of the Geological S	168	525-543
710	GO 1/5	CAOB	TRANSBAIKALIA	106.23	51.21	Essexite	Murtoi dike	117	150	13.29	80.21	0.09973	0.51248		-0.49	-3.1	–1.6	-1.23	-1.23	0.90	0.90	1.04				Andryushchenko SV et	2010	an Geology and Geopl	51(7)	734-749
711	MG 3/8	CAOB	TRANSBAIKALIA	106.14	51.16	Essexite	Murtoi dike	117	150	11.94	85.88	0.083684	0.512557		-0.57	-1.6	0.1	0.58	0.58	0.70	0.70	0.89				Andryushchenko SV et	2010	an Geology and Geopl	51(7)	734-749
712	GO 1/1	CAOB	TRANSBAIKALIA	106.07	51.11	Essexite	Murtoi dike	117	150	13.71	84.7	0.097428	0.512449		-0.50	-3.7	–2.2	-1.79	-1.79	0.92	0.92	1.08				Andryushchenko SV et	2010	an Geology and Geopl	51(7)	734-749
713	Kham 3/14	CAOB	TRANSBAIKALIA	106.14	51.16	Trachybasalt	ta ancient vo	124	150	11.43	71.68	0.095979	0.512524		-0.51	-2.2	–0.6	-0.30	-0.30	0.81	0.81	0.96				Andryushchenko SV et	2010	an Geology and Geopl	51(7)	734-749
714	Kham 3/16	CAOB	TRANSBAIKALIA	106.16	51.17	Phonotephrite	ta ancient vo	124	150	12.25	82	0.089919	0.512494		-0.54	-2.8	–1.1	-0.77	-0.77	0.81	0.81	1.00				Andryushchenko SV et	2010	an Geology and Geopl	51(7)	734-749
715	GO 5/3	CAOB	TRANSBAIKALIA	106.18	51.18	Basaltic trachyandesite	Volcanic fiel	155	155	7.23	48.71	0.089341	0.512564		-0.55	-1.4	0.7	0.68	0.68	0.72	0.72	0.89				Andryushchenko SV et	2010	an Geology and Geopl	51(7)	734-749
716	GO 5/4	CAOB	TRANSBAIKALIA	106.19	51.19	Basaltic trachyandesite	Volcanic fiel	155	155	8.32	57.26	0.087458	0.512528		-0.56	-2.1	0	0.01	0.01	0.75	0.75	0.94				Andryushchenko SV et	2010	an Geology and Geopl	51(7)	734-749
717	Kham 1/6	CAOB	TRANSBAIKALIA	106.2	51.2	Trachybasalt	Volcanic fiel	155	155	8.28	53.86	0.092532	0.512523		-0.53	-2.2	–0.2	-0.18	-0.18	0.79	0.79	0.96				Andryushchenko SV et	2010	an Geology and Geopl	51(7)	734-749
718	KL04-3	CAOB	NW China	84.8397	45.684	mafic dyke		270	270	5	22.3	0.1359	0.512933	3	-0.31	5.8	7.86	7.85	7.85	0.43	0.43	0.40	0.43			Zhang CL et al.	2013	Lithos	174	15-27
719	KL07-1	CAOB	NW China	84.8397	45.684	mafic dyke		270	270	7.42	29.8	0.1505	0.512798	5	-0.23	3.1	4.72	4.71	4.71	0.85	0.85	0.65	0.85			Zhang CL et al.	2013	Lithos	174	15-27
720	KL08-2	CAOB	NW China	84.8397	45.684	mafic dyke		270	270	1.99	8.05	0.1495	0.512714	6	-0.24	1.5	3.11	3.11	3.11	1.03	1.03	0.78	1.04			Zhang CL et al.	2013	Lithos	174	15-27
721	KL08-4	CAOB	NW China	84.8397	45.684	mafic dyke		270	270	3.24	14.3	0.137	0.512888	3	-0.30	4.9	6.94	6.93	6.93	0.52	0.52	0.47	0.52			Zhang CL et al.	2013	Lithos	174	15-27
722	DT06-2	CAOB	NW China	91.675	41.41	mafic dyke		270	270	3.4	16.2	0.1266	0.512344	6	-0.36	-5.7	-3.32	-3.32	-3.32	1.41	1.41	1.31	1.41			Zhang CL et al.	2013	Lithos	174	15-27
723	DT06-4	CAOB	NW China	91.675	41.41	mafic dyke		270	270	5.23	26.1	0.121	0.512376	3	-0.38	-5.1	-2.5	-2.50	-2.50	1.27	1.27	1.24	1.27			Zhang CL et al.	2013	Lithos	174	15-27
724	DT06-5	CAOB	NW China	91.675	41.41	mafic dyke		270	270	5.01	21.9	0.1382	0.512405	4	-0.30	-4.5	-2.53	-2.53	-2.53	1.50	1.50	1.24	1.51			Zhang CL et al.	2013	Lithos	174	15-27
725	DT06-6	CAOB	NW China	91.675	41.41	mafic dyke		270	270	7.13	35.1	0.1228	0.512344	5	-0.38	-5.7	-3.19	-3.19	-3.19	1.35	1.35	1.30	1.35			Zhang CL et al.	2013	Lithos	174	15-27
726	DT12-6	CAOB	NW China	91.675	41.41	mafic dyke		270	270	6.93	33	0.1268	0.512478	6	-0.36	-3.1	-0.71	-0.71	-0.71	1.18	1.18	1.09	1.18			Zhang CL et al.	2013	Lithos	174	15-27
727	DT12-2	CAOB	NW China	91.675	41.41	mafic dyke		270	270	4.91	25.2	0.1176	0.512344	7	-0.40	-5.7	-3.01	-3.01	-3.01	1.28	1.28	1.28	1.28			Zhang CL et al.	2013	Lithos	174	15-27
728	WT16-2	CAOB	NW China	82.545	43.59	mafic dyke		270	270	4.3	10.8	0.241	0.513092	3	0.23	8.9	7.34	7.33	7.33	0.44	-0.33	0.44	--			Zhang CL et al.	2013	Lithos	174	15-27
729	WT16-3	CAOB	NW China	82.545	43.59	mafic dyke		270	270	4.54	9.75	0.2815	0.513156	4	0.43	10.1	7.18	7.18	7.18	0.45	0.01	0.45	--			Zhang CL et al.	2013	Lithos	174	15-27
730	18XJ19-3	CAOB	China	89.2833	47.067	meta-basaltic		380	380	5.07	17.3	0.177	0.513		-0.10	7.1	8.2	8.02	8.02	0.62	0.62	0.47				Cui X et al.	2020	Lithos	366-367	105540
731	18XJ20-3	CAOB	China	89.2833	47.067	meta-basaltic		380	380	5.58	19.6	0.172	0.513		-0.13	7.1	8.4	8.26	8.26	0.55	0.55	0.45				Cui X et al.	2020	Lithos	366-367	105540
732	18XJ21-3	CAOB	China	89.3	47.067	meta-basaltic		380	380	6.31	22.1	0.173	0.513		-0.12	7.1	8.2	8.21	8.21	0.56	0.56	0.46				Cui X et al.	2020	Lithos	366-367	105540
733	18XJ21-5	CAOB	China	89.3	47.067	meta-basaltic		380	380	6.72	23.7	0.171	0.513		-0.13	7.1	8.2	8.31	8.31	0.54	0.54	0.45				Cui X et al.	2020	Lithos	366-367	105540
734	18XJ24-1	CAOB	China	89.4	47.067	meta-basaltic		380	380	4.26	13.8	0.186	0.513		-0.05	7.1	7.5	7.58	7.58	0.83	0.83	0.51				Cui X et al.	2020	Lithos	366-367	105540
735	18XJ27-5	CAOB	China	89.4	47.067	meta-basaltic		380	380	2.29	8.05	0.172	0.5127		-0.13	1.2	3.1	2.41	2.41	1.64	1.64	0.93				Cui X et al.	2020	Lithos	366-367	105540
736	18XJ28-4	CAOB	China	89.35	47.067	meta-basaltic		380	380	3.03	8.05	0.228	0.5132		0.16	11.0	10.3	9.45	9.45	0.53	0.53	0.36				Cui X et al.	2020	Lithos	366-367	105540
737	18XJ28-5	CAOB	China	89.35	47.067	meta-basaltic		380	380	2.18	5.1	0.259	0.5131		0.32	9.0	6.6	5.99	5.99	0.64	-0.17	0.64				Cui X et al.	2020	Lithos	366-367	105540
738	18XJ30-5	CAOB	China	89.4	47.067	meta-basaltic		380	380	3.13	9.37	0.202	0.513		0.03	7.1	7.6	6.80	6.80	1.95	1.95	0.57				Cui X et al.	2020	Lithos	366-367	105540
739	14GW116	CAOB	NE China	131.461	45.044	gabbro		430	430	0.624	2.51	0.15023248	0.51242738	12	-0.24	-4.1	-1.56	-1.56	-1.56	1.73	1.73	1.29	1.73			Yang H et al.	2022	Gondwana Research	103	297-313
740	14GW117	CAOB	NE China	131.461	45.044	gabbro		430	430	1.3	5.12	0.15343663	0.51245374	10	-0.22	-3.6	-1.22	-1.22	-1.22	1.76	1.76	1.26	1.76			Yang H et al.	2022	Gondwana Research	103	297-313
741	14GW118	CAOB	NE China	131.461	45.044	gabbro		430	430	1.64	7.91	0.12528982	0.512383	11	-0.36	-5.0	-1.05	-1.06	-1.06	1.32	1.32	1.25	1.32			Yang H et al.	2022	Gondwana Research	103	297-313
742	14GW119	CAOB	NE China	131.461	45.044	gabbro		430	430	1.23	5.27	0.14104167	0.51242719	12	-0.28	-4.1	-1.06	-1.06	-1.06	1.51	1.51	1.25	1.51			Yang H et al.	2022	Gondwana Research	103	297-313
743	M-13-106	CAOB	Dzhida terrain	101.617	50.275	Dolerite	dikes	500	500	3.53	14.28	0.1506	0.512459	6	-0.23	-3.5	-0.5	-0.55	-0.55	1.67	1.67	1.27				Belyaev VA et al.	2021	Lithos	386-387	106044
744	M-13-107	CAOB	Dzhida terrain	101.633	50.275	Lamproph.	dikes	500	500	5.06	21.95	0.1403	0.512467	8	-0.29	-3.3	0.3	0.26	0.26	1.42	1.42	1.20				Belyaev VA et al.	2021	Lithos	386-387	106044
745	M-13-08	CAOB	Dzhida terrain	101.6	50.275	Gabbro	dikes	500	500	7.42	38.1	0.1186	0.512371	7	-0.40	-5.2	-0.2	-0.23	-0.23	1.25	1.25	1.24				Belyaev VA et al.	2021	Lithos	386-387	106044
746	M-13-125	CAOB	Dzhida terrain	101.617	50.275	Lamproph.	dikes	500	500	12.63	69.66	0.1104	0.512318	8	-0.44	-6.2	-0.7	-0.74	-0.74	1.23	1.23	1.28				Belyaev VA et al.	2021	Lithos	386-387	106044
747	M-13-129	CAOB	Dzhida terrain	101.633	50.275	Gabbro	dikes	500	500	3.46	13.6	0.1549	0.512628	6	-0.21	-0.2	2.5	2.47	2.47	1.35	1.35	1.02				Belyaev VA et al.	2021	Lithos	386-387	106044
748	M-13-129	CAOB	Dzhida terrain	101.6	50.275	Gabbro	dikes	500	500	3.68	14.46	0.1551	0.51262	9	-0.21	-0.4	2.3	2.30	2.30	1.38	1.38	1.04				Belyaev VA et al.	2021	Lithos	386-387	106044
749	Wy23-5	CAOB	Eastern Tianshan	94.35	41.767	Anorthosite		238	238			0.153	0.512588	10	-0.22	-1.0	0.35	0.35	0.35	1.41	1.41	0.98				Shi Y et al.	2021	Ore Geology Reviews	132	104003
750	Wy23-7	CAOB	Eastern Tianshan	94.35	41.767	Anorthosite		238	238			0.1665	0.512614	9	-0.15	-0.5	0.45	0.45	0.45	1.73	1.73	0.97				Shi Y et al.	2021	Ore Geology Reviews	132	104003
751	Wy23-11	CAOB	Eastern Tianshan	94.35	41.767	Semi-massive ore		238	238			0.1602	0.512593	4	-0.19	-0.9	0.45	0.23	0.23	1.58	1.58	0.99				Shi Y et al.	2021	Ore Geology Reviews	132	104003
752	RH02-1	CAOB	Jiamusi Block	133.019	46.185	gabbro	ongfanghon	275	275	1.37	5.16	0.1607	0.512538	4	-0.18	-2.0	-0.69	-0.69	-0.69	1.76	1.76	1.09				Sun MD et al.	2015	Tectonophysics	659	122-136
753	RH02-2	CAOB	Jiamusi Block	133.019	46.185	gabbro	ongfanghon	275	275	1.15	4.52	0.1535	0.512528	3	-0.22	-2.1	-0.64	-0.63	-0.63	1.57	1.57	1.09				Sun MD et al.	2015	Tectonophysics	659	122-136
754	RH02-8	CAOB	Jiamusi Block	133.019	46.185	gabbro	ongfanghon	275	275	1.2	4.63	0.1567	0.512527	4	-0.20	-2.2	-0.77	-0.76	-0.76	1.66	1.66	1.10				Sun MD et al.	2015	Tectonophysics	659	122-136
755	RH02-10	CAOB	Jiamusi Block	133.019	46.185	gabbro	ongfanghon	275	275	1.08	4.09	0.1592	0.512551	5	-0.19	-1.7	-0.39	-0.38	-0.38	1.67	1.67	1.07				Sun MD et al.	2015	Tectonophysics	659	122-136
756	RH02-11	CAOB	Jiamusi Block	133.019	46.185	gabbro	ongfanghon	275	275	1.06	4.12	0.155	0.512542	5	-0.21	-1.9	-0.41	-0.41	-0.41	1.58	1.58	1.07				Sun MD et al.	2015	Tectonophysics	659	122-136
757	RH02-12	CAOB	Jiamusi Block	133.019	46.185	gabbro	ongfanghon	275	275	1.01	4.03	0.1521	0.512527	4	-0.23	-2.2	-0.61	-0.60	-0.60	1.54	1.54	1.09				Sun MD et al.	2015	Tectonophysics	659	122-136
758	RH02-13	CAOB	Jiamusi Block	133.019	46.185	gabbro	ongfanghon	275	275	0.98	3.76	0.1578	0.512537	4	-0.20	-2.0	-0.61	-0.61	-0.61	1.67	1.67	1.09				Sun MD et al.	2015	Tectonophysics	659	122-136
759	KR80	CAOB	Gorny Altai	87.43	50.08	Basalt	etionary con	600	600	5.08	14.9	0.2056	0.513074	10	0.05	8.5	7.8	7.82	7.82	1.43	1.43	0.67				Utsunomiy A et al.	2009	Lithos	113(3-4)	437-453
760	KR81	CAOB	Gorny Altai	87.43	50.08	Basalt	etionary con	600	600	4.19	12.2	0.2075	0.513098	5	0.05	9.0	8.1	8.15	8.15	1.28	1.28	0.64				Utsunomiy A et al.	2009	Lithos	113(3-4)	437-453
761	KR82	CAOB	Gorny Altai	87.43	50.08	Basalt	etionary con	600	600	5.9	17.5	0.2033	0.513071	7	0.03	8.4	7.9	7.94	7.94	1.16	1.16	0.66				Utsunomiy A et al.	2009	Lithos	113(3-4)	437-453
762	KR83	CAOB	Gorny Altai	87.43	50.08	Dolerite	etionary con	600	600	4.88	14.6	0.2013	0.513044	6	0.02	7.9	7.5	7.57	7.57	1.30	1.30	0.69				Utsunomiy A et al.	2009	Lithos	113(3-4)	437-453
763	KR86	CAOB	Gorny Altai	87.43	50.08	Basalt	etionary con	600	600	2.05	5.52	0.2244	0.513134	6	0.14	9.7	7.6	7.55	7.55	0.69	-0.23	0.69				Utsunomiy A et al.	2009	Lithos	113(3-4)	437-453
764	KR87	CAOB	Gorny Altai	87.43	50.08	Basalt	etionary con	600	600	5.83	17.8	0.1984	0.513057	7	0.01	8.2	8.1	8.04	8.04	0.93	0.93	0.65				Utsunomiy A et al.	2009	Lithos	113(3-4)	437-453
765	KR90	CAOB	Gorny Altai	87.43	50.08	Basalt	etionary con	600	600	4.24	12.4	0.2061	0.513088	7	0.05	8.8	8	8.06	8.06	1.24	1.24	0.65				Utsunomiy A et al.	2009	Lithos	113(3-4)	437-453
766	98KR177	CAOB	Gorny Altai	87.44	50.14	Basalt	etionary con	600	600	3.01	8.18	0.2222	0.513078	6	0.13	8.6	6.6	6.63	6.63	0.77	-1.30	0.77				Utsunomiy A et al.	2009	Lithos	113(3-4)	437-453
767	98KR269	CAOB	Gorny Altai	87.34	50.02	Basalt	etionary con	600	600	5.72	16.18	0.2139	0.513073	6	0.09	8.5	7.2	7.17	7.17	0.83	-74.33	0.83				Utsunomiy A et al.	2009	Lithos	113(3-4)	437-453
768	98KR400	CAOB	Gorny Altai	87.44	50.14	Basalt	etionary con	600	600	5.38	16.1	0.2017	0.513074	5	0.03	8.5	8.1	8.12	8.12	0.97	0.97	0.65				Utsunomiy A et al.	2009	Lithos	113(3-4)	437-453
769	99KR17	CAOB	Gorny Altai	87.34	50.02	Basalt	etionary con	600	600	4.29	14.08	0.1841	0.512874	5	-0.06	4.6	5.6	5.57	5.57	1.42	1.42	0.85				Utsunomiy A et al.	2009	Lithos	113(3-4)	437-453
770	99KR68	CAOB	Gorny Altai	87.21	50.12	Basalt	etionary con	600	600	2.72	7.23	0.2273	0.513097	6	0.16	9.0	6.6	6.61	6.61	0.77	-0.60	0.77				Utsunomiy A et al.	2009	Lithos	113(3-4)	437-453
771	99KR218	CAOB	Gorny Altai	87.25	50.25	Dolerite	etionary con	600	600	4.29	14.32	0.1812	0.512797	7	-0.08	3.1	4.3	4.29	4.29	1.65	1.65	0.96				Utsunomiy A et al.	2009	Lithos	113(3-4)	437-453
772	KR16	CAOB	Gorny Altai	87.21	50.12	Basalt	etionary con	600	600	6.13	23.6	0.1574	0.512826	8	-0.20	3.7	6.7	6.68	6.68	0.88	0.88	0.76				Utsunomiy A et al.	2009	Lithos	113(3-4)	437-453
773	98KR48	CAOB	Gorny Altai	87.25	50.25	Basalt	etionary con	600	600	4.57	15.14	0.1823	0.512801	6	-0.07	3.2	4.3	4.28	4.28	1.69	1.69	0.96				Utsunomiy A et al.	2009	Lithos	113(3-4)	437-453
774	KR71	CAOB	Gorny Altai	87.25	50.25	Basalt	metamorphi	600	600	2.49	7.1	0.2122	0.513144	8	0.08	9.9	8.7	8.68	8.68	0.61	0.61	0.60				Utsunomiy A et al.	2009	Lithos	113(3-4)	437-453
775	99CU4	CAOB	Gorny Altai	87.25	50.25	Basalt	etionary con	600	600	3.5	12.61	0.1679	0.512823	7	-0.15	3.6	5.8	5.81	5.81	1.09	1.09	0.83				Utsunomiy A et al.	2009	Lithos	113(3-4)	437-453
776	99CU23	CAOB	Gorny Altai	87.25	50.25	Dolerite	metamorphi	600	600	5.29	15.44	0.2071	0.513035	6	0.05	7.7	7	6.95	6.95	2.64	2.64	0.74				Utsunomiy A et al.	2009	Lithos	113(3-4)	437-453
777	01CU12	CAOB	Gorny Altai	87.25	50.25	Basalt	metamorphi	600	600	5.87	21.32	0.1664	0.512741	6	-0.15	2.0	4.3	4.33	4.33	1.32	1.32	0.95				Utsunomiy A et al.	2009	Lithos	113(3-4)	437-453
778	98CU41	CAOB	Gorny Altai	87.25	50.25	Amphibolite	metamorphi	600	600	5.41	26	0.126	0.512732	6	-0.36	1.8	7.3	7.25	7.25	0.73	0.73	0.72				Utsunomiy A et al.	2009	Lithos	113(3-4)	437-453
779	UZ12	CAOB	Gorny Altai	87.25	50.25	Basalt	hiolitic comp	600	600	2.33	6.32	0.2225	0.51313	7	0.13	9.6	7.6	7.62	7.62	0.69	-0.35	0.69				Utsunomiy A et al.	2009	Lithos	113(3-4)	437-453
780	CU53	CAOB	Gorny Altai	87.25	50.25	Amphibolite	hiolitic comp	600	600	4.37	11.8	0.2235	0.513164	8	0.14	10.3	8.2	8.21	8.21	0.64	0.22	0.64				Utsunomiy A et al.	2009	Lithos	113(3-4)	437-453
781	U48	CAOB	Gorny Altai	87.25	50.25	Basalt	etionary con	600	600	4.03	14.45	0.1686	0.512756	6	-0.14	2.3	4.5	4.45	4.45	1.33	1.33	0.94				Utsunomiy A et al.	2009	Lithos	113(3-4)	437-453
782	U51	CAOB	Gorny Altai	87.25	50.25	Basalt	etionary con	600	600	4.73	17.45	0.1638	0.512743	6	-0.17	2.0	4.6	4.57	4.57	1.24	1.24	0.93				Utsunomiy A et al.	2009	Lithos	113(3-4)	437-453
783	AT12	CAOB	Gorny Altai	87.25	50.25	Basalt	hiolitic comp	600	600	4.26	16.08	0.1603	0.512813	7	-0.19	3.4	6.2	6.20	6.20	0.96	0.96	0.80				Utsunomiy A et al.	2009	Lithos	113(3-4)	437-453
784	AT49	CAOB	Gorny Altai	87.25	50.25	Basalt	etionary con	600	600	7.13	29.42	0.1464	0.512661	7	-0.26	0.4	4.3	4.30	4.30	1.11	1.11	0.96				Utsunomiy A et al.	2009	Lithos	113(3-4)	437-453
785	AT74	CAOB	Gorny Altai	87.25	50.25	Dolerite	etionary con	600	600	2.49	7.47	0.2018	0.512976	7	0.03	6.6	6.2	6.20	6.20	2.22	2.22	0.80				Utsunomiy A et al.	2009	Lithos	113(3-4)	437-453
786	XYB14-4	CAOB	Tianshan	83.3108	43.624			314	314	4.15	18.3	0.1321	0.512648		-0.33	0.2	2.8	2.78	2.78	0.94	0.94	0.85				unpublished data				
787	XYD14-6	CAOB	Tianshan	83.3261	43.573			315	315	1.92	8.5	0.143	0.512514		-0.27	-2.4	-0.3	-0.26	-0.26	1.37	1.37	1.09				unpublished data				
788	XYE14-8	CAOB	Tianshan	83.245	43.349			323	323	3.48	14.9	0.144	0.512471		-0.27	-3.3	-1.1	-1.09	-1.09	1.48	1.48	1.17				unpublished data				
789	TKSE14-15	CAOB	Tianshan	81.9903	43.245			304	304	3.09	10.6	0.1752	0.512916		-0.11	5.4	6.3	6.26	6.26	0.93	0.93	0.55				unpublished data				
790	TKSA14-6	CAOB	Tianshan	81.9075	42.794			412	412	2.72	10.6	0.158	0.512605		-0.20	-0.6	1.4	1.39	1.39	1.49	1.49	1.04				unpublished data				
791	KM9802-11	CAOB	Tianshan	84.823	45.702	Dioritic enclave		306	306	3.64	16.2	0.12	0.51283		-0.39	3.7	6.8	6.74	6.74	0.52	0.52	0.52				Geng et al.	2009	Chemical Geology	266	364-389
792	AK6	CAOB	Tianshan	84.363	45.759	Dioritic enclave		304	304	5.47	22.3	0.14	0.512752		-0.29	2.2	4.5	4.42	4.42	0.82	0.82	0.70				Geng et al.	2009	Chemical Geology	266	364-389

Note: for a few mafic rocks that show significant differences in Sm/Nd ratios from the continental crust, i.e., fSm/Nd > -0.3 or -0.5, and extremely implausible TDM1 values, we use TDM2.

Table S2a. Zircon Lu-Hf isotope data of the felsic-intermediate igneous rocks of the Altaids.

No.	Sample	Longitude	Latitude	Regions	Rock type	Age (Ma)	Error	Recalculated-Age (Ma)	$\varepsilon_{Hf}(t)$_Average	$\varepsilon_{Hf}(t)$_Median	T_{DM2}_Average	T_{DM2}_Median	Reference
1	12GW104	121.85	46.70	Hegenshan ophiolite-arc-accretion complex	Alkali feldspar granite	134	1	150	9.5	9.7	553	536	Tian DX et al., 2015 Geological Magazine
2	D9830	120.46	46.68	Uliastai active continental margin	Alkali-feldspar granite	139	2	150	8.4	8.7	616	597	Tian DX et al., 2013 GlobGlobal Geology (ICWEA)
3	08487-1	110.79	41.82	Northern margin of the North China Craton	Alkali-feldspar granite	429	0	429	-2.8	-2.5	1477	1461	Zhang SH et al., 2014 GeolGeological Society of America Bulletin
4	11RJ-2	118.35	43.42	Erdaojing accretion complex	Diorite	138	1	150	2.2	1.9	970	992	Li S et al., 2013, Journal of Asian Earth Sciences
5	D464	116.75	40.96	North China Craton	Diorite	283	0	283	-17.1	-17.2	2180	2185	Zhang SH et al., 2009 Int J Earth Sci (Geol Rundsch)
6	D1019-7	121.19	45.14	Baolidao arc-accretion complex	Granite	236	1	236	10.1	10.2	583	581	Li SC,2016,Acta Petrologica Sinica (ICWEB)
7	DW09-b6	118.55	46.21	Uliastai active continental margin	Granite	134	0	150	9.1	8.6	573	603	Xiong GQ et al., 2014 Bulletin of Mineralogy, Petrology and Geochemistry (ICWEA)
8	D002	118.93	46.51	Uliastai active continental margin	Granite	324	0	324	6.7	7.4	853	811	Ke LL et al., 2016 Acta Petrologica et Minetalogica (ICWEA)
9	DL-01	117.98	44.21	Baolidao arc-accretion complex	Granite	292	0	292	4.9	5.3	928	907	Zhou ZH et al., 2014 Acta Petrologica Sinica (ICWEA)
10	D001	119.01	46.46	Uliastai active continental margin	Granite	319	0	319	6.3	6.6	867	850	Ke LL et al., 2016 Acta Petrologica et Mineralogica (ICWEA)
11	D003	118.83	46.49	Uliastai active continental margin	Granite	332	0	332	5.8	5.9	910	904	Ke LL et al., 2016 Acta Petrologica et Mineralogica (ICWEA)
12	HLT-1	113.51	43.55	Baolidao arc-accretion complex	Granite	234	0	234	4.9	5.0	884	875	Chen Bin., 2009 Journal of Asian Earth Sciences
13	14NL2-6	113.55	42.39	Northern margin of the North China Craton	Granite	270	0	270	4.4	4.6	939	932	Liu CH et al., 2015 Lithos
14	DP-17	115.30	40.75	North China Craton	Granite	394	0	394	-19.9	-19.0	2425	2373	Cisse et al., 2016 Ore Geology Reviews
15	CF06-42	119.05	41.94	North China Craton	Granite	150	0	150	-11.9	-10.8	1780	1720	Wang YB et al., 2010 Acta Petrologica et Mineralogica (ICWEA)
16	14HS-3	118.54	43.93	Erdaojing accretion complex	Granite porphyry	133	1	150	10.9	11.0	470	462	Li S et al., 2017a, Tectonics, 2017b, GeolGeological Society of America Bulletin
17	DSW4	119.35	44.45	Erdaojing accretion complex	Granite porphyry	151	0	151	7.9	7.8	645	648	Zeng QD et al., 2015 Journal of Asian Earth Sciences
18	STTW.6	120.51	40.82	North China Craton	Granite porphyry	181	0	181	-9.1	-8.3	1647	1603	Cui FH, 2015 PhD paper (ICWEA)
19	KB6183	120.69	45.49	Hegenshan ophiolite-arc-accretion complex	Granite porphyry	122	0	150	8.0	8.0	636	637	Wu Q, 2014 Master's thesis
20	G0213-4	121.28	46.59	Hegenshan ophiolite-arc-accretion complex	Granodiorite	175	0	175	11.5	11.4	453	462	Zhou Y, 2011 Acta Petrologica et Mineralogica (ICWEA)
21	XL922-2	116.01	43.69	Baolidao arc-accretion complex	Granodiorite	255	0	255	12.0	11.6	492	513	Li S et al., 2016 Scientific Reports
22	FP2	116.98	40.99	North China Craton	Granodiorite	310	0	310	-14.4	-12.7	2044	1952	Zhang SH et al., 2009 GeolGeological Society of America Bulletin
23	10XC05	120.35	40.12	North China Craton	Granodiorite	229	0	229	-15.6	-15.4	2055	2045	Wang C et al., 2015 Lithos
24	FX09-1	121.85	42.19	North China Craton	Granodiorite	222	0	222	-3.8	-3.5	1376	1358	Zhang XH et al., 2012a Lithos
25	PM013-1-3	120.70	46.82	Hegenshan ophiolite-arc-accretion complex	Granodiorite	240	3	240	10.0	9.3	595	634	Liu XW et al., 2015 Acta Petrologica et Mineralogica (ICWEA)
26	0107-19	116.53	43.73	Erdaojing accretion complex	Granodiorite	278	0	278	7.1	7.0	789	796	Zhou XW, 2012 Phd paper(ICWEA)
27	FX09-2	121.85	42.19	North China Craton	Granodiorite	218	0	218	-5.2	-5.8	1453	1487	Zhang XH et al., 2012a Lithos
28	61910	117.62	42.91	Northern margin of the North China Craton	K-feldspar granite	270	0	270	2.7	2.5	1039	1053	Zhao P et al., 2016 Lithos
29	XN09- 203	113.87	42.12	Northern margin of the North China Craton	K-feldspar granite	263	0	263	-0.1	-0.4	1193	1208	Hao BW, 2011 Phd paper (ICWEA)
30	D055-1	117.80	41.20	North China Craton	Monzodiorite	390	0	390	-9.5	-10.3	1833	1879	Zhang SH et al., 2007 Journal of the Geological Society
31	STWH1048	120.36	40.74	North China Craton	Monzogranite	183	0	183	-9.6	-8.9	1679	1634	Cui FH, 2015 PhD paper (ICWEA)
32	STWH1056	120.46	40.66	North China Craton	Monzogranite	161	0	161	-21.8	-21.5	2355	2339	Cui FH, 2015 PhD paper (ICWEA)
33	QL38	118.81	40.31	North China Craton	Monzogranite	170	0	170	-15.7	-15.4	2013	1995	Fan WB et al., 2017 Lithos
34	SM13-4	116.74	45.96	Uliastai active continental margin	Monzogranite	146	1	150	9.2	9.0	566	581	Jiang SH et al., 2016 Lithos
35	N14624-2.1	111.49	43.37	Baolidao arc-accretion complex	Monzogranite	276	0	276	-14.1	-14.1	2003	2003	Guo L, 2015 Acta Petrologica et Mineralogica (ICWEA)
36	0102-17	116.20	43.79	Baolidao arc-accretion complex	Monzogranite	323	0	323	7.3	7.0	817	831	Zhou XW, 2012 Phd paper (ICWEA)
37	09M66	120.23	44.54	Erdaojing accretion complex	Monzogranite porphyry	132	1	150	6.4	5.9	728	757	Ma XH et al., 2013 Gondwana Research
38	LJG-1	120.03	43.81	Erdaojing accretion complex	Monzogranite porphyry	239	0	239	5.3	5.7	862	842	Duan XX et al., 2015 International Geology Review
39	09021-1	118.82	40.43	North China Craton	Monzonite	221	0	221	0.1	0.5	1147	1124	Ye H, 2014 Earth Science Frontiers(ICWEA)
40	YY03-2	112.43	39.92	North China Craton	Monzonite	241	0	241	-19.2	-12.4	2265	1882	Ying JF et al., 2011 Lithos
41	08406-1	112.72	42.29	Northern margin of the North China Craton	Muscovite granite	432	0	432	-3.9	-0.4	1539	1346	Zhang SH et al., 2014 GeolGeological Society of America Bulletin
42	DSM-1	107.06	41.12	North China Craton	Porphyritic granite	287	0	287	-4.3	-4.3	1455	1456	Hu CS et al., 2015 International Geology Review
43	07245-1	114.56	40.31	North China Craton	Pyroxene syenite	224	0	224	-9.1	-9.3	1676	1689	Zhang SH et al., 2012 Lithos
44	7245	114.56	40.31	North China Craton	Pyroxene syenite	221	0	221	-11.9	-11.8	1839	1829	Zhang SH et al., 2012 Lithos
45	070813-7	112.58	42.24	Northern margin of the North China Craton	Quartz diorite	421	0	421	-2.1	-1.9	1433	1424	Zhang SH et al., 2014 GeolGeological Society of America Bulletin
46	12P1TW01	114.81	42.07	Northern margin of the North China Craton	Quartz diorite	471	0	471	9.2	9.2	822	824	Peng SH et al., 2013 Global Geology (ICWEA)
47	D251-1	117.56	40.98	North China Craton	Quartz diorite	291	0	291	-13.2	-13.3	1965	1972	Zhang SH et al., 2009a Int J Earth Sci (Geol Rundsch)
48	STTW.2	120.64	40.69	North China Craton	Quartz diorite	156	0	156	-20.8	-20.8	2292	2294	Cui FH, 2015 PhD paper (ICWEA)
49	STTW.10	120.36	40.92	North China Craton	Quartz monzonite	230	0	230	-1.7	-1.9	1263	1274	Cui FH, 2015 PhD paper (ICWEA)
50	MB1-5	113.63	43.62	Baolidao arc-accretion complex	Quartz-diorite	320	0	320	10.1	10.1	651	652	Shi YR et al., 2016 Journal of Asian Earth Sciences
51	H09-020-01	113.63	42.31	Northern margin of the North China Craton	Quartz-diorite	273	0	273	4.8	4.0	920	965	Hao BW, 2011 Phd paper (ICWEA)
52	GEQ-5	114.49	45.02	Uliastai active continental margin	Syengranite	310	0	310	7.3	9.0	802	707	Wang JC et al., 2016 Geoscience
53	B070822-4	95.53	41.21	Western CAOB	Syenogranite	221	0	221	4.3	4.1	910	917	Li S et al., 2012 Lithos
54	STTW.5	120.54	40.74	North China Craton	Syenogranite	193	0	193	-9.8	-9.7	1698	1688	Cui FH, 2015 PhD paper (ICWEA)
55	GZ10-65	119.30	44.31	Erdaojing accretion complex	Syenogranite	154	0	154	12.4	12.6	388	375	Yang QD et al., 2014 Acta Petrologica Sinica (ICWEA)
56	D2-1	100.93	42.60	CAOB	rhyolitic tuff	311	2	311	14.3	14.3	400	401	Song et al., 2021 Acta Petrologica Sinica (ICWEA)
57	39A	84.39	43.41	CAOB	Diorite porphyrite	307	2	307	11.0	11.0	588	588	Huang et al., 2017 Journal of Earth Science and Environment (ICWEA)
58	TW3352	123.84	49.44	CAOB	A-type granite	253	1.4	253	7.5	7.5	745	746	Wu et al., 2019 Acta Geologica Sinica(ICWEA)
59	11WJ07	83.91	45.51	0	Aalkali-feldspar granite	294	2.6	294	13.0	13.1	464	459	Tang et al., 2019 Earth and Planetary Science Letters
60	11WJ16	83.86	45.54	0	Aalkali-feldspar granite	300	5.2	300	13.0	12.9	464	474	Tang et al., 2019 Earth and Planetary Science Letters
61	LYB19	95.50	41.10	Western CAOB	Adakite	424	0	424	1.8	2.0	1211	1200	Mao QG et al., 2012 Gondwana Research
62	HEBUKESAIRN	85.00	47.00	Western CAOB	Adakite	346	0	346	15.1	15.4	382	365	Zhang, C et al., 2018 Lithos
63	YX-06	120.45	40.83	Western Liaoning	Adakitic	181	0	181	-11.9	-12.8	1803	1860	Song ZW et al., 2021 Minerals
64	YX-07	120.48	40.78	Western Liaoning	Adakitic	178	0	178	-9.7	-9.5	1677	1668	Song ZW et al., 2021 Minerals
65	YY095	89.82	39.52	NW China	adakitic	445	0	445	-4.7	-4.4	1598	1584	Yu SY et al., 2018 Geological Magazine
66	YY122	89.85	39.49	NW China	adakitic	425	0	425	4.1	4.5	1078	1059	Yu SY et al., 2018 Geological Magazine
67	XJ046-1	85.84	42.17	0	Alkali feldspar granite	388	2	388	7.4	6.4	863	919	Lin W et al., 2013 Lithosphere
68	M099-1	116.12	48.52	0	Alkali feldspar granite	151	1	151	7.1	6.4	689	732	Wang, TH et al., 2014 Global Geology(ICWEA)
69	13GW308	121.72	49.48	0	Alkali feldspar granite	142	1	150	7.2	7.5	682	665	He Y et al., 2018 Canadian Journal of Earth Sciences
70	14GW505	121.83	49.42	0	Alkali feldspar granite	150	1	150	8.8	8.6	591	605	He Y et al., 2018 Canadian Journal of Earth Sciences
71	12GW106	121.84	46.70	0	Alkali feldspar granite	130	0	150	10.7	10.5	479	491	Tian DX et al., 2015 Geological Magazine
72	13GW405	119.69	47.32	0	Alkali feldspar granite	139	1	150	6.6	6.6	721	718	Dong Y et al., 2019 Acta Geologica Sinica(English Edition)
73	13GW420	119.70	47.33	0	Alkali feldspar granite	137	1	150	7.1	6.7	692	712	Dong Y et al., 2019 Acta Geologica Sinica(English Edition)
74	13GW425	119.69	47.32	0	Alkali feldspar granite	145	1	150	6.4	6.5	729	723	Dong Y et al., 2019 Acta Geologica Sinica(English Edition)
75	15GW235	130.77	46.24	Jiamusi Block	Alkali feldspar granite	276	3	276	-2.9	-2.9	1365	1368	Dong et al., 2017 Gondwana Research
76	1045-3	129.86	49.07	LXZR	Alkali feldspar granite	210	2	210	6.7	6.7	761	760	Gou J et al., 2013 Journal of Jilin University (Earth Science Edition)
77	1078-1	127.30	48.72	LXZR	Alkali feldspar granite	184	2	184	2.1	2.4	1005	986	Gou J et al., 2013 Journal of Jilin University (Earth Science Edition)
78	15XH27–1	128.42	44.09	LXZR	Alkali feldspar granite	263	2	263	4.0	3.6	961	979	Long et al., 2019 Geological Journal
79	SJ02	90.33	45.03	Eastern Junggar	alkali feldspar granite	304	1.9	304	13.5	13.5	441	440	Liu W et al., 2013 Lithos
80	G167	90.29	45.16	Eastern Junggar	alkali feldspar granite	311	2.1	311	14.7	15.3	379	344	Liu W et al., 2013 Lithos

81	11WJ85	85.50	46.71	0	Alkali granite	416	5.4	416	13.4	13.4	537	537	Tang et al., 2019 Earth and Planetary Science Letters
82	AK154-2	84.76	45.41	Western Junggar	Alkali-feldspar granite	305	4	305	13.8	14.0	426	412	Geng et al., 2009 Chemical Geology
83	MG136-1	83.94	45.49	Western Junggar	Alkali-feldspar granite	308	6	308	15.4	15.4	337	336	Geng et al., 2009 Chemical Geology
84	MG9951-1	83.88	45.53	Western Junggar	Alkali-feldspar granite	298	4	298	13.8	13.7	418	423	Geng et al., 2009 Chemical Geology
85	KM9918-3	83.75	45.65	Western Junggar	Alkali-feldspar granite	296	7	296	14.0	13.8	409	416	Geng et al., 2009 Chemical Geology
86	SN10-2	113.59	44.44	Hegenshan ophiolite-arc-accretion complex	Alkali-feldspar granite	289	0	289	13.1	13.3	450	443	Zhang et al., 2015 Gondwana Research
87	SN10-9	113.69	44.48	Hegenshan ophiolite-arc-accretion complex	Alkali-feldspar granite	290	0	290	13.0	12.8	460	471	Zhang et al., 2015 Gondwana Research
88	SN10-12	113.22	44.31	Hegenshan ophiolite-arc-accretion complex	Alkali-feldspar granite	290	0	290	12.9	12.9	464	467	Zhang et al., 2015 Gondwana Research
89	SN10-3	113.75	44.50	Hegenshan ophiolite-arc-accretion complex	Alkali-feldspar granite	288	0	288	13.3	13.7	442	418	Zhang et al., 2015 Gondwana Research
90	14HT22-1	129.42	48.25	LXZR	Alkali-feldspar granite	450	2	450	2.2	2.3	1209	1205	Wang ZW et al., 2016 Lithos
91	XH08-1	90.43	45.44	yemaquan	alkali-feldspar granite	304	0	304	11.9	11.7	536	547	Gan et al., 2010
92	08QH-25	90.74	46.17	Chinese Altai	Alkali-felspar granite	382	3	382	12.4	12.5	569	563	Shen XM et al., 2011 Journal of Asian Earth Sciences
93	08QH-27	90.84	46.15	Chinese Altai	Alkali-felspar granite	367	3	367	11.0	11.6	637	602	Shen XM et al., 2011 Journal of Asian Earth Sciences
94	08LX66	116.20	35.40	North China Craton	alkaline complex	130	0.8	150	-16.2	-16.1	2025	2022	Lan TG et al., 2020 Lithos
95	07SD74	117.60	35.10	North China Craton	alkaline complex	132	0.8	150	-14.5	-14.3	1928	1922	Lan TG et al., 2020 Lithos
96	08WLG-16	89.30	46.15	Chinese Altai	alkaline granite	308	6	308	14.7	14.5	373	387	Shen XM et al., 2011 Journal of Asian Earth Sciences
97	08WLG-07	89.38	46.23	Chinese Altai	alkaline granite	291	7	291	12.7	12.5	477	487	Shen XM et al., 2011 Journal of Asian Earth Sciences
98	WJ1138	85.71	47.23	Western Junggar	Altered diorite porphyrite	334	1.9	334	13.2	13.2	484	482	Yin et al., 2015 Gondwana Research
99	TLK-01	81.78	42.08	0	Altered felsic lava flow	285	2	285	-5.2	-3.6	1504	1410	Huang H et al., 2015 Lithos
100	M114-1	117.30	49.42	0	Altered granite	180	1	180	-7.4	-7.6	1547	1557	Wang TH et al., 2014 Global Geology(ICWEA)
101	WJ1139	85.72	47.72	Western Junggar	ered quartz diorite porphy	335	1.8	335	12.9	13.3	503	477	Yin et al., 2015 Gondwana Research
102	BL1-1	120.73	47.19	0	Andesite	236	1	236	10.0	9.9	588	598	Ji Z et al., 2018 Acta Petrologica Sinica (ICWEA)
103	14HS17-1	130.42	43.33	0	Andesite	110	1	150	9.2	9.1	571	573	Wang F et al., 2019 Lithos
104	14HS21-1	129.55	43.33	0	Andesite	114	1	150	8.9	9.1	584	574	Wang F et al., 2019 Lithos
105	14HS23-1	129.24	43.26	0	Andesite	115	1	150	8.7	9.0	596	581	Wang F et al., 2019 Lithos
106	HY1-1	129.80	46.45	0	Andesite	112	1	150	6.5	7.4	727	671	Wang F et al., 2019 Lithos
107	XDM1	125.25	43.61	0	Andesite	254	4	254	4.0	4.1	952	945	Song ZG et al., 2018, Gondwana Research
108	SD7	125.22	43.61	0	Andesite	256	3	256	3.6	3.8	978	964	Song ZG et al., 2018, Gondwana Research
109	14DX1-1	120.43	47.52	0	Andesite	228	2	228	11.4	11.2	503	515	Li Y et al., 2017 Gondwana Research
110	13HH19-1	126.07	50.11	0	Andesite	223	1	223	12.5	12.4	433	442	Li Y et al., 2017 Gondwana Research
111	NM14-19	113.13	42.34	0	Andesite	134	2	150	10.0	10.6	519	485	Tang JZ et al., 2019 International Geology Review
112	16W122-1	119.78	45.38	0	Andesite	149	2	150	10.7	9.9	483	527	Tang ZY et al., 2020 International Geology Review
113	A19-1	122.38	50.83	0	Andesite	152	1.1	152	3.8	2.9	884	935	Zhang GL et al., 2020 Geological Journal
114	04YJ-6	129.42	42.77	North China craton	Andesite	190	0	190	8.3	9.0	650	612	Guo, F et al., 2007 Journal of Petrology
115	08HLQ17	112.00	41.00	North China craton	Andesite	1909	10	600	-35.7	-35.8	3465	3472	Peng, P et al., 2011 Precambrian Research
116	HB14-1	127.50	45.63	Eastern CAOB	Andesite	292	0	292	5.3	5.5	908	897	Meng, E et al., 2011 Journal of Asian Earth Sciences
117	HB17-1	127.50	45.63	Eastern CAOB	Andesite	292	0	292	8.5	8.7	720	713	Meng, E et al., 2011 Journal of Asian Earth Sciences
118	HST0606	118.00	43.52	Eastern CAOB	Andesite	234	0	234	8.5	8.6	676	667	Liu, YS et al.,2012 Chemical Geology
119	HST0701	118.00	43.52	Eastern CAOB	Andesite	233	0	233	10.5	13.6	553	376	Liu, YS et al.,2012 Chemical Geology
120	ZF5	91.80	44.61	Western CAOB	Andesite	390	3.1	390	13.2	13.6	528	505	Xu, XW et al., 2013 Gondwana Research
121	MOSHEN-1	85.00	45.02	Western CAOB	Andesite	332	3.8	332	10.2	13.2	653	480	He, DF et al., 2013 Gondwana Research
122	06XJ-31	81.63	44.25	Western CAOB	Andesite	347	0	347	3.0	4.0	1083	1023	Tang, GJ et al., 2013 Journal of Asian Earth Sciences
123	A01-2	89.67	46.50	Western CAOB	Andesite	441	0	441	16.0	15.9	441	441	Shen, XM et al., 2014 Lithos
124	11LK19-1	124.36	42.89	North China craton	Andesite	342	0	342	10.9	11.5	622	589	Wang, ZW et al., 2015 Journal of Asian Earth Sciences
125	HTS9-13	125.70	50.20	Eastern CAOB	Andesite	447	0	447	13.3	13.5	567	557	Wu, G et al., 2015 Journal of Asian Earth Sciences
126	13ER20-1	120.19	50.25	Eastern CAOB	Andesite	180	0	180	0.1	0.0	1115	1121	Wang, W et al., 2015 Lithos
127	BPC-15	121.00	42.00	North China craton	Andesite	158	0	158	-13.3	-14.6	1866	1941	Ma, Q et al., 2009 Acta Petrologica Sinica
128	07125-1	110.54	41.77	North China craton	Andesite	278	5	278	-12.7	-11.1	1924	1836	Zhang, SH et al., 2016 Lithos
129	08550-1	109.99	41.88	North China craton	Andesite	273	3	273	-12.6	-12.0	1919	1885	Zhang, SH et al., 2016 Lithos
130	KY12-33-4	124.26	42.58	North China craton	Andesite	2500	0	600	-34.8	-40.4	3411	3727	Yuan, LL et al., 2016 Lithos
131	13GW166	132.11	46.37	Eastern CAOB	Andesite	275	0	275	4.4	4.1	945	961	Bi, JH et al., 2017 Journal of Asian Earth Sciences
132	10LF53	115.00	41.00	North China craton	Andesite	2410	0	600	-38.7	-38.9	3635	3642	Liou, P et al., 2017 Lithos
133	10LF73	115.00	41.00	North China craton	Andesite	2239	0	600	-40.1	-40.3	3710	3721	Liou, P et al., 2017 Lithos
134	TS08	84.00	43.00	Western CAOB	Andesite	361	0	361	10.0	10.1	689	685	Zhu, YF et al., 2009 Journal of the Geological Society
135	13HH19	126.07	50.11	Eastern CAOB	Andesite	223	0	223	12.5	12.4	433	442	Li, Y et al., 2017 Gondwana Research
136	14DX1	120.43	47.52	Eastern CAOB	Andesite	228	0	228	11.4	11.2	503	515	Li, Y et al., 2017 Gondwana Research
137	14DX7	122.45	48.11	Eastern CAOB	Andesite	228	0	228	14.5	14.6	321	314	Li, Y et al., 2017 Gondwana Research
138	15DX4	122.42	47.51	Eastern CAOB	Andesite	228	0	228	9.6	9.4	610	616	Li, Y et al., 2017 Gondwana Research
139	HSW11	127.08	49.94	Eastern CAOB	Andesite	321	0	321	9.7	9.2	651	681	Li, Y et al., 2017 Gondwana Research
140	Y-12	95.00	42.00	Western CAOB	Andesite	321	0	321	7.9	8.7	785	731	Jiang, HJ et al., 2017 Geoscience Frontiers
141	ZK1-112	95.00	42.00	Western CAOB	Andesite	298	0	298	8.4	8.5	736	729	Jiang, HJ et al., 2017 Geoscience Frontiers
142	06XJ-97	82.50	43.50	Western CAOB	Andesite	279	0	279	13.3	14.1	435	387	Tang, GJ et al., 2017 Geochemistry Geophysics Geosystems
143	P1-2	113.00	44.00	Eastern CAOB	Andesite	326	0	326	11.0	10.6	606	629	Chai, H et al., 2018 Lithos
144	L445B	94.49	45.39	Western CAOB	Andesite	335	0	335	11.5	12.9	590	502	Nguyen, H et al., 2018 Journal of Asian Earth Sciences
145	L462	95.65	44.50	Western CAOB	Andesite	350	0	350	8.4	8.1	771	789	Nguyen, H et al., 2018 Journal of Asian Earth Sciences
146	SL14-012	92.00	41.11	Western CAOB	Andesite	317	0	317	9.9	10.8	662	609	Zhao, LD et al., 2019 Gondwana Research
147	L5	88.08	46.06	Western CAOB	Andesite	313	0	313	13.5	13.9	449	429	Li, D., 2016 Chinese University of Geosciences (Beijing).
148	DK12-56	84.33	43.12	Western CAOB	Andesite	418	0	418	2.4	1.8	1176	1216	Wang, M et al., 2016, Journal of Asian Earth Sciences
149	K502	95.76	44.54	the Trans-Altai Zone	Andesite	326	1.4	326	12.8	13.0	500	490	Hugo Nguyen et al., 2018 Journal of Asian Earth Sciences
150	13HYC36-1	130.55	47.55	0	Andesite, Dacite	97	1	150	5.8	5.6	768	777	Wang F et al., 2019 Lithos
151	HY4-1	130.12	46.46	0	Andesite, Dacite, Rhyolite	110	2	150	6.2	6.1	743	748	Wang F et al., 2019 Lithos
152	7638	95.00	47.67	CAOB	andesites	959	0	600	7.0	7.1	1052	1049	Victor Kovach et al., 2021 Gondwana Research
153	ED4	120.76	50.79	0	Andesitic Porphyrite	160	1	160	4.7	4.8	835	833	Xu ZT et al., 2020 Minerals
154	30513	116.78	44.78	the northern North China Craton	anorthosite	1753	23	600	-30.9	-31.1	3197	3209	Zhang SH et al., 2007 Precambrian Research
155	FX06-52	121.64	42.29	North China Craton	Appinites	252	0	252	-11.8	-11.4	1858	1832	Zhang XH et al., 2012 Lithos
156	B2-8	120.48	45.45	0	A-Type Granite	124	1	150	9.4	9.2	555	569	Yang WB et al., 2017 Journal of Geophysical Research: Solid Earth
157	H4	120.48	45.46	0	A-Type Granite	122	1	150	13.6	13.5	310	318	Yang WB et al., 2017 Journal of Geophysical Research: Solid Earth
158	TKSB14-1	81.89	42.81	0	A-type granites	370	4	370	6.0	6.0	930	928	Huang H et al., 2020 Earth Science Review
159	06XJ136	84.74	45.68	Western Junggar	A-type granites	314	3	314	14.9	15.1	369	357	Tang et al., 2012 Lithos
160	10SYQ07	96.93	42.04	Beishan	augen gneiss	499	0	499	-6.1	-6.2	1720	1727	Song, D., et al., 2013 Precambrian Research
161	SBS-2	122.41	53.06	CAOB	A-type granites	481	5	481	1.8	2.8	1254	1196	Wu Q et al., 2019 Acta Geologica Sinica (ICWEA)
162	TKST-11	86.06	43.03	0	Biotite granite	362	5	362	-2.1	-1.2	1386	1336	Huang H et al., 2015 Acta Geologica Sinica (ICWEA)
163	KK3-1	81.77	42.76	Western CAOB	Biotite Granite	407	12	407	3.8	5.6	1086	983	Xu XY et al., 2010 Acta Petrologica et Mineralogica (ICWEA)
164	06XJ131	84.56	45.69	Western Junggar	biotite granite	312	3	312	13.5	14.0	449	419	Tang et al., 2012 Lithos

165	11WJ26	84.55	46.11	0	Biotite granite	298	4	298	13.1	13.1	457	460	Tang et al., 2019 Earth and Planetary Science Letters
166	HL-1-1	129.17	42.59	0	Biotite granite	249	11	249	1.4	1.0	1095	1121	Guan QB et al., 2020 Gondwana Research
167	ZK2507-479	119.28	44.63	0	Biotite granite	143	0	150	6.7	6.5	715	723	Liu LJ et al., 2021 Lithos
168	B17	119.13	50.00	0	Biotite granite	239	1	239	5.0	5.0	883	880	Kang YJ et al., 2018 Ore Geology Reviews
169	B21	119.11	49.99	0	Biotite granite	240	1	240	4.1	4.1	934	934	Kang YJ et al., 2018 Ore Geology Reviews
170	A-5	114.55	47.11	Eastern CAOB	Biotite Granite	230	0	230	6.9	6.7	765	777	Li, S et al., 2013 Earth-Science Reviews
171	A-9	114.55	47.11	Eastern CAOB	Biotite Granite	230	0	230	7.9	7.7	708	720	Li, S et al., 2013 Earth-Science Reviews
172	G-5	115.33	45.58	Eastern CAOB	Biotite Granite	226	0	226	8.2	7.8	684	708	Li, S et al., 2013 Earth-Science Reviews
173	KLW-2	89.12	47.92	Chinese Altai	biotite granite	456	5.4	456	1.5	3.0	1255	1170	Lv Zhenghang et al., 2012 Lithos
174	152	88.86	47.78	Chinese Altai	biotite granite	267	5	267	7.1	7.0	781	786	Tong Ying, 2014 American Journal of Science
175	SJZ-14	119.32	44.58	0	tite granite/K-feldspar gra	142	2	150	6.9	6.9	700	699	Liu LJ et al., 2021 Lithos
176	TPT-09	119.21	44.65	0	tite granite/K-feldspar gra	139	2	150	6.9	7.0	701	695	Liu LJ et al., 2021 Lithos
177	09KZ05	77.15	40.36	Tianshan	tite granite+minor granodio	272	1	272	1.0	1.0	1137	1140	Zhang and Zou, 2013 Lithos
178	09GL07	77.11	40.53	Tianshan	tite granite+minor granodio	277	1	277	-0.2	0.1	1212	1196	Zhang and Zou, 2013 Lithos
179	777	87.87	42.23	Tianshan	Biotite K-feldspar granite	275	2	275	-3.6	-3.5	1405	1400	Ma et al., 2015 Gondwana Research
180	777-2	87.87	42.23	Tianshan	Biotite K-feldspar granite	275	2	275	-3.4	-3.4	1392	1395	Ma et al., 2015 Gondwana Research
181	787	86.22	42.45	Tianshan	Biotite K-feldspar granite	292	2	292	-5.1	-5.4	1505	1522	Ma et al., 2015 Gondwana Research
182	794	86.90	42.91	Tianshan	Biotite K-feldspar granite	339	3	339	3.0	3.2	1073	1066	Ma XX et al.,2014 Gondwana Research
183	788	86.27	42.68	Tianshan	Biotite K-feldspar granite	300	3	300	-0.8	-0.1	1262	1223	Ma XX et al.,2015 Gondwana Research
184	B005	123.42	48.91	0	Biotite monzogranite	161	2	161	6.7	6.8	719	717	Zhang DH et al., 2015 Geological Journal
185	KK1-1	81.24	42.83	Western CAOB	Biotite Monzongranite	426	2	426	-0.9	0.3	1365	1301	Xu XY et al., 2010 Acta Petrologica et Mineralogica (ICWEA)
186	HSW2-3	127.26	48.71	0	Biotite monzonite	175	1	175	9.5	9.0	573	597	Xu MJ et al., 2013 Acta Petrologica Sinica (ICWEA)
187	AI1101-1	82.37	45.11	0	Biotite monzonitic granite	297	2	297	9.2	9.2	684	683	Yin et al., 2016, International Geology Review
188	AI1105-1	82.37	45.11	0	Biotite monzonitic granite	301	1	301	10.2	10.4	631	618	Yin et al., 2016, International Geology Review
189	10MZS04	96.52	41.49	Beishan	biotite-plagioclase gneiss	374	0	374	6.7	7.3	888	854	Song D et al., 2013 Precambrian Research
190	NLKD14-8	83.70	43.84	0	Bt granite	300	3	300	6.6	6.6	836	836	Huang H et al., 2020 Earth Science Review
191	P9-48-1	121.68	46.63	0	Bt monzogranite	126	1	150	9.5	9.5	552	552	Lu KJ et al., 2018 Global Geology (ICWEA)
192	GW05085	126.21	52.01	0	Bt monzogranite	178	1	178	-1.2	-0.3	1193	1137	Sui ZM et al., 2007 Acta Petrologica Sinica (ICWEA)
193	GW05120	126.27	51.82	0	Bt monzogranite	176	1	176	-0.1	-0.9	1128	1169	Sui ZM et al., 2007 Acta Petrologica Sinica (ICWEA)
194	BLG13-1	77.24	40.87	0	Bt monzonite	291	3	291	-0.7	-0.8	1252	1255	Huang H et al., 2015 Acta Petrologica et Mineralogica (ICWEA)
195	BLG13-16	77.24	40.87	0	Bt monzonite	283	3	283	-0.9	-0.9	1257	1258	Huang H et al., 2015 Acta Petrologica et Mineralogica (ICWEA)
196	XSS1	88.45	42.35	0	Bt-Granite	458	9	458	-10.1	-10.3	1919	1928	Shi et al., 2014 International Geology Review
197	MG123-2	83.87	45.52	Western Junggar	Charnockite	305	3	305	14.5	14.4	386	391	Geng et al., 2009 Chemical Geology
198	MG164-2	83.95	45.52	Western Junggar	Charnockite	305	3	305	14.8	14.6	369	377	Geng et al., 2009 Chemical Geology
199	MG9811-1	84.03	45.55	Western Junggar	Charnockite	296	3	296	14.1	14.3	398	390	Geng et al., 2009 Chemical Geology
200	M-13	92.33	33.42	Hf Isotopes of a K-rich Charnockite–Granite	Charnockite	68	0.29	150	4.2	4.1	856	864	Lu L et al., 2019 Journal of Petrology
201	LY-11	92.00	33.15	Hf Isotopes of a K-rich Charnockite–Granite	Charnockite	68	0.51	150	4.0	4.2	868	859	Lu L et al., 2019 Journal of Petrology
202	11WJ23	83.95	45.52	0	Charnokite	297	4	297	13.5	13.5	438	436	Tang et al., 2019 Earth and Planetary Science Letters
203	11WJ12	83.85	45.55	0	Charnokite	305	7.2	305	13.2	13.3	462	456	Tang et al., 2019 Earth and Planetary Science Letters
204	Bu-12	87.02	48.23	Chinese Altai	Coarse grained granodiorit	453	5	453	6.2	5.9	982	1001	Sun M et al., 2008 Chemical Geology
205	HB-8	86.55	48.14	Chinese Altai	Coarse-grained granodiorit	388	6	388	4.6	4.8	1020	1012	Cai KD et al., 2011 Journal of Asian Earth Sciences
206	CH10	87.33	48.13	Chinese Altai	Coarse-grained granodiorit	439	5.5	439	6.5	6.6	951	947	Cai KD et al., 2011 Journal of Asian Earth Sciences
207	CH1	87.21	48.11	Chinese Altai	Coarse-grained granodiorit	397	3	397	3.9	4.4	1071	1042	Cai KD et al., 2011 Journal of Asian Earth Sciences
208	XJ050	85.72	42.22	0	Dacite	399	2	399	0.4	0.3	1269	1279	Lin W et al., 2013 Lithosphere
209	JRH01-1	133.64	46.53	0	Dacite	116	2	150	6.2	5.7	740	773	Wang F et al., 2019 Lithos
210	HM2-1	132.05	45.74	0	Dacite	122	2	150	12.1	12.1	401	400	Wang F et al., 2019 Lithos
211	14HQ5-1	130.83	45.65	0	Dacite	113	1	150	5.6	5.5	774	782	Wang F et al., 2019 Lithos
212	SF03-1	131.11	44.40	0	Dacite	106	1	150	7.4	7.4	675	673	Wang F et al., 2019 Lithos
213	HS6-1	130.56	44.66	0	Dacite	110	10	150	8.0	8.1	635	633	Wang F et al., 2019 Lithos
214	HS1-1	130.55	44.69	0	Dacite	96	3	150	5.7	5.8	770	766	Wang F et al., 2019 Lithos
215	SF01-1	131.05	44.36	0	Dacite	93	1	150	7.1	7.2	688	684	Wang F et al., 2019 Lithos
216	15DX5-1	122.32	47.53	0	Dacite	226	2	226	10.3	10.0	567	584	Li Y et al., 2017 Gondwana Research
217	15DX6-1	122.21	47.54	0	Dacite	226	1	226	10.8	10.5	537	553	Li Y et al., 2017 Gondwana Research
218	NM14-44	111.98	43.94	0	Dacite	129	1	150	4.3	5.1	852	802	Tang JZ et al., 2019 International Geology Review
219	14ER462-1	120.27	50.66	0	Dacite	150	2	150	5.3	5.0	793	810	Gou J et al., 2019 International Geology Review
220	14P2-5	120.17	50.77	0	Dacite	150	2	150	5.4	5.5	787	784	Gou J et al., 2019 International Geology Review
221	HLH-11	120.00	45.00	Eastern CAOB	Dacite	135	0	150	9.5	9.5	549	550	Guo F et al., 2009 International Journal of Earth Sciences
222	TH05-3	91.80	44.60	Western CAOB	Dacite	453	4.4	453	12.1	12.1	642	639	Xu, XW et al., 2013 Gondwana Research
223	K28-18-1	89.30	46.50	Western CAOB	Dacite	278	0	278	12.4	12.6	484	476	Li D et al., 2014 Lithos
224	K28-5-1	89.30	46.50	Western CAOB	Dacite	279	0	279	13.2	13.3	441	433	Li, D et al., 2014 Lithos
225	HA2-5	83.50	41.00	Western CAOB	Dacite	287	0	287	-2.9	-2.7	1372	1360	Liu, HQ et al., 2014 Lithos
226	08FY-05	89.65	46.50	Western CAOB	Dacite	446	0	446	14.7	15.3	485	449	Shen, XM et al., 2014 Lithos
227	HSW5-5	127.04	49.05	Eastern CAOB	Dacite	319	0	319	6.9	6.6	837	855	Li, Y et al., 2014 Lithos
228	JK11-1	127.00	43.00	North China craton	Dacite	293	0	293	2.5	2.0	1066	1096	Cao, HH et al., 2012 Acta Petrologica Sinica
229	12BP9	74.00	52.00	Western CAOB	Dacite	501	0	501	14.1	14.2	564	560	Shen, P et al., 2015 Lithos
230	C12FY07	89.73	46.97	Western CAOB	Dacite	400	0	400	0.9	4.2	1259	1052	He, YL et al., 2015 Journal of Asian Earth Sciences
231	07D016-1	119.81	42.51	North China craton	Dacite	258	1	258	1.6	2.4	1093	1049	Zhang, SH et al., 2016 Lithos
232	07D033-1	118.95	42.56	North China craton	Dacite	261	3	261	-13.1	-13.6	1937	1967	Zhang, SH et al., 2016 Lithos
233	AS50	86.00	48.00	Western CAOB	Dacite	1958	0	600	-25.7	-26.9	2907	2977	Song, GX et al., 2010 Acta Petrologica Sinica
234	15DX5	122.32	47.53	Eastern CAOB	Dacite	226	0	226	10.3	10.0	567	584	Li, Y et al., 2017 Gondwana Research
235	15DX6	122.00	48.00	Eastern CAOB	Dacite	226	0	226	10.8	10.5	537	553	Li, Y et al., 2017 Gondwana Research
236	MO328	100.85	44.45	Eastern CAOB	Dacite	443	0	443	5.9	5.7	990	1000	Kröner, A et al.,2017 Gondwana Research
237	X10-45-1	97.04	42.18	Western CAOB	Dacite	328	0	328	9.2	10.4	717	642	Yuan, Y et al., 2018 Lithos
238	EGRDC066(172	106.91	43.12	Eastern CAOB	Dacite	353	0	353	12.5	12.6	553	555	Dolgopolova, A et al., 2013 Lithos
239	D1218(1048-104	106.87	43.05	Eastern CAOB	Dacite	344	0	344	13.2	13.6	506	487	Dolgopolova, A et al., 2013 Lithos
240	OTD404(399)	106.86	43.02	Eastern CAOB	Dacite	356	0	356	13.6	13.8	483	473	Dolgopolova, A et al., 2013 Lithos
241	L8	87.82	45.99	Western CAOB	Dacite	377	0	377	14.0	14.9	472	416	Li, D., 2016 Chinese University of Geosciences (Beijing).
242	MD4	86.45	45.59	Western CAOB	Dacite	327	0	327	6.6	6.7	856	850	Li, D., 2016 Chinese University of Geosciences (Beijing).
243	W39-1	118.68	43.88	0	Dacite porphyry	173	3	173	9.2	9.7	586	558	Guan QB et al., 2018 International Geology Review
244	LYB19	95.32	41.07	NW China	dacites	424	3.3	424	1.5	1.7	1227	1216	Mao QG et al., 2012 Gondwana Research
245	Ab7112	121.33	46.63	0	Dacitic tuff	123	1	150	9.0	8.8	581	593	Tan HY et al., 2017 Geological Bulletin of China(ICWEA)
246	13ts15B	83.97	43.10	0	Deformed monzogranite	411	4	411	-1.5	-0.6	1390	1338	Zhong LL et al. (2017)
247	AGM-1	84.75	45.73	0	Dike	316	2.7	316	10.0	9.9	652	659	Li S et al., 2015 GF
248	G2	114.53	23.58	northwestern China	dike	281	0	281	13.0	13.2	454	439	Mao YJ et al., 2018 Lithos

249	DJ160-a-10-6	114.52	23.59	northwestern China	dike	281	0	281	13.5	13.6	424	416	Mao YJ et al., 2018 Lithos
250	DJ7007	114.54	23.60	northwestern China	dike	281	0	281	12.9	12.1	460	505	Mao YJ et al., 2018 Lithos
251	HSD9	114.51	23.61	northwestern China	dike	281	0	281	12.1	12.1	505	507	Mao YJ et al., 2018 Lithos
252	06XJ182	84.75	45.68	Western Junggar	diorite	316	3	316	14.4	14.8	399	376	Tang et al., 2012 Lithos
253	06XJ138	84.45	45.50	0	Diorite	310	3.9	310	14.0	13.9	419	421	Tang et al., 2019 Earth and Planetary Science Letters
254	15XJ01	84.53	45.66	0	Diorite	316	6.6	316	13.6	14.0	446	424	Tang et al., 2019 Earth and Planetary Science Letters
255	15XJ07	85.23	46.30	0	Diorite	315	4.6	315	13.3	13.2	464	467	Tang et al., 2019 Earth and Planetary Science Letters
256	ZK003-233	84.50	45.41	Western CAOB	Diorite	321	1.6	321	13.4	13.4	461	462	Cao, M et al., 2016 Lithos
257	BHS	84.50	45.41	Western CAOB	Diorite	320	1.5	320	14.6	14.8	388	378	Cao, M et al., 2016 Lithos
258	BTC196	84.50	45.42	Western CAOB	Diorite	321	1.9	321	14.7	14.7	388	387	Cao, M et al., 2016 Lithos
259	BXS	84.48	45.39	Western CAOB	Diorite	321	1.4	321	14.3	14.4	411	401	Cao, M et al., 2016 Lithos
260	BSB	84.51	45.40	Western CAOB	Diorite	313	1.7	313	14.7	14.7	381	379	Cao, M et al., 2016 Lithos
261	13ER8-6	121.54	51.67	Eastern CAOB	Diorite	204	2	204	0.2	0.1	1131	1134	Tang J et al., 2016 Gondwana Research
262	14JEW8-1	129.77	42.62	0	Diorite	183	1	183	9.3	9.3	590	591	Wang F et al., 2017, Journal of Asian Earth Sciences
263	GW05099	125.64	52.05	0	Diorite	188	1	188	3.2	2.9	947	965	Sui ZM et al., 2007 Acta Petrologica Sinica (ICWEA)
264	12GW029	131.46	45.43	0	Diorite	296	2	296	-0.6	-0.7	1246	1253	Yang H et al., 2015 Gondwana Research
265	1665-1	121.02	52.35	0	Diorite	213	2	213	1.8	2.0	1048	1032	Sun DY et al., 2013, International Geology Review
266	H15-08	128.19	46.92	Eastern CAOB	Diorite	181	1	181	4.9	4.8	844	850	Ge MH et al., 2018 Lithos
267	H15-39-1	129.98	47.38	0	Diorite	246	1	246	-1.3	-1.8	1251	1277	Ge MH et al., 2018 Lithos
268	13GW284	123.18	48.49	0	Diorite	171	1	171	8.0	8.1	653	647	Dong Y et al., 2016 Journal of Asian Earth Sciences
269	HSS12	92.00	40.90	Western CAOB	Diorite	280	0	280	4.0	3.7	973	987	Su, B.X et al., 2011 Journal of Asian Earth Sciences
270	TH05-1	91.80	44.60	Western CAOB	Diorite	372	0	372	-37.7	-36.1	3406	3694	Xu, XW et al., 2013 Gondwana Research
271	TH7	91.80	44.60	Western CAOB	Diorite	406	5.7	406	-4.6	-4.8	1560	1577	Xu, XW et al., 2013 Gondwana Research
272	TH9	91.79	44.60	Western CAOB	Diorite	432	1.5	432	14.6	14.7	481	473	Xu, XW et al., 2013 Gondwana Research
273	MO53	93.98	45.40	Western CAOB	Diorite	290	0	290	12.3	12.3	499	501	Cai, KD et al., 2014 Lithos
274	WJL050710	78.93	46.56	Western CAOB	Diorite	275	0	275	2.4	2.5	1060	1056	Zou, SY et al., 2015 Lithos
275	TS-003	90.12	46.46	Western CAOB	Diorite	384	0	384	11.5	11.5	620	620	Liang, P et al., 2016 Lithos
276	14GW088	130.02	45.69	Eastern CAOB	Diorite	253	0	253	-5.2	-5.1	1475	1474	Yang, H et al., 2017 International Journal of Earth Sciences
277	09AL70	104.38	40.39	North China craton	Diorite	280	0	280	-10.9	-10.9	1825	1824	Dan, W et al., 2014 Lithos
278	09AL73	104.38	40.40	North China craton	Diorite	282	0	282	-8.2	-8.1	1672	1666	Dan, W et al., 2014 Lithos
279	V003	97.50	45.79	Western CAOB	Diorite	477	0	477	11.0	11.0	738	739	Janoušek, V et al., 2018 Gondwana Research
280	V004	97.52	45.80	Western CAOB	Diorite	504	0	504	11.7	11.8	710	707	Janoušek, V et al., 2018 Gondwana Research
281	CL13-009	92.00	42.00	Western CAOB	Diorite	317	0	317	13.7	13.9	442	430	Zhao, LD et al., 2018 Journal of Asian Earth Sciences
282	S102-08	92.50	42.00	Western CAOB	Diorite	440	0	440	14.8	15.0	476	461	Wang, YF et al., 2018 Journal of Asian Earth Sciences
283	YH15-99	92.50	42.00	Western CAOB	Diorite	290	0	290	10.1	10.2	629	621	Wang, YF et al., 2018 Journal of Asian Earth Sciences
284	BA11-87	96.80	41.86	Western CAOB	Diorite	435	0	435	-2.2	-1.6	1447	1415	Zheng, RG et al., 2018 Journal of Asian Earth Sciences
285	14RF46-1	133.65	45.69	Eastern CAOB	Diorite	554	0	554	0.1	-0.4	1403	1414	Xu, T et al., 2018 Lithos
286	X10-46-1	97.09	42.15	Western CAOB	Diorite	281	0	281	11.0	11.1	567	560	Yuan, Y et al., 2018 Lithos
287	X10-47-1	97.10	42.14	Western CAOB	Diorite	287	0	287	11.4	11.1	552	567	Yuan, Y et al., 2018 Lithos
288	X10-70-1	98.37	41.83	Eastern CAOB	Diorite	466	0	466	-13.4	-13.4	2116	2111	Yuan, Y et al., 2018 Lithos
289	X10-71-1	98.36	41.86	Eastern CAOB	Diorite	455	0	455	-13.3	-13.5	2099	2110	Yuan, Y et al., 2018 Lithos
290	X10-73-1	98.39	41.91	Eastern CAOB	Diorite	450	0	450	-13.5	-13.4	2107	2100	Yuan, Y et al., 2018 Lithos
291	H15-39	129.98	47.38	Eastern CAOB	Diorite	246	0	246	-1.3	-1.8	1251	1277	Ge, MH et al., 2018 Lithos
292	YD2-004-1	92.00	42.00	Western CAOB	Diorite	317	0	317	13.4	13.2	465	480	Wang, YH et al., 2018 Mineralium Deposita
293	YD2-004-3	92.00	42.00	Western CAOB	Diorite	330	0	330	13.6	13.6	454	451	Wang, YH et al., 2018 Mineralium Deposita
294	YD5501-99-2	92.00	42.00	Western CAOB	Diorite	347	0	347	11.4	11.8	592	564	Wang, YH et al., 2018 Mineralium Deposita
295	XVIII	61.70	56.93	Western CAOB	Diorite	433	0	433	12.3	12.3	613	612	Smirnov, VN et al., 2018 Geochemistry International
296	16GW110	129.57	44.67	Eastern CAOB	Diorite	320	0	320	-0.7	-0.7	1201	1193	Zhao, D et al., 2018 Lithos
297	16GW124	129.38	44.64	Eastern CAOB	Diorite	219	0	219	1.6	1.8	1061	1047	Zhao, D et al., 2018 Lithos
298	DM132	110.70	41.85	North China craton	Diorite	428	0	428	-3.8	-4.0	1537	1546	Zhou, H et al., 2018 Lithos
299	DM210	110.50	42.00	North China craton	Diorite	458	0	458	4.4	3.9	1092	1120	Zhou, H et al., 2018 Lithos
300	DM232	110.50	42.09	North China craton	Diorite	475	0	475	4.3	4.4	1106	1102	Zhou, H et al., 2018 Lithos
301	DM98	110.04	41.76	North China craton	Diorite	258	0	258	-11.9	-12.1	1866	1874	Zhou, H et al., 2018 Lithos
302	EL14-20-1	111.23	42.87	Eastern CAOB	Diorite	282	0	282	5.8	8.4	929	857	Yuan, L et al., 2018 Journal of Asian Earth Sciences
303	13ZLG43	111.00	41.00	North China craton	Diorite	2520	0	600	-38.5	-38.4	3621	3616	Wang, D et al., 2018 Journal of Petrology
304	SL14-001-1	91.11	41.45	Western CAOB	Diorite	323	0	323	13.5	13.5	458	453	Zhao, LD et al., 2019 Gondwana Research
305	SL14-016	91.10	41.43	Western CAOB	Diorite	327	0	327	14.5	14.5	403	401	Zhao, LD et al., 2019 Gondwana Research
306	09AL147	105.53	40.11	North China craton	Diorite	423	0	423	-6.8	-6.9	1703	1708	Dan, W et al., 2016 Gondwana Research
307	OTD343(1247)	106.86	43.03	Eastern CAOB	Diorite	358	0	358	13.4	13.2	496	504	Dolgopolova, A et al., 2013 Lithos
308	0514(1289.15-129	106.86	43.05	Eastern CAOB	Diorite	353	0	353	13.5	13.2	491	504	Dolgopolova, A et al., 2013 Lithos
309	514(1405.85-141	106.86	43.06	Eastern CAOB	Diorite	344	0	344	14.5	14.3	430	430	Dolgopolova, A et al., 2013 Lithos
310	OTD514(2161.35	106.86	43.05	Eastern CAOB	Diorite	353	0	353	13.9	13.8	460	472	Dolgopolova, A et al., 2013 Lithos
311	OTD786(150)	106.85	43.01	Eastern CAOB	Diorite	358	0	358	13.6	13.9	492	481	Dolgopolova, A et al., 2013 Lithos
312	12K50	86.86	41.44	Western CAOB	Diorite	1929	0	600	-34.9	-34.6	3424	3406	Ge, R et al., 2015 Geological Society of America Bulletin
313	12K09	86.72	41.70	Western CAOB	Diorite	417	0	417	-0.1	0.0	1314	1310	Ge, R et al., 2015 Geological Society of America Bulletin
314	12k33	86.31	41.76	Western CAOB	Diorite	422	0	422	-9.7	-10.5	1879	1914	Ge, R et al., 2015 Geological Society of America Bulletin
315	KG15	75.88	42.30	Western CAOB	Diorite	451	0	451	-3.8	-2.2	1554	1461	Kröner, A et al., 2013 Gondwana Research
316	K-1	74.97	46.98	Western CAOB	Diorite	353	0	353	7.5	7.4	833	831	Li, GM et al., 2016 Gondwana Research
317	E-20	104.13	49.02	Eastern CAOB	Diorite	240	0	240	10.5	10.0	562	593	Li, S et al., 2013 Earth-Science Reviews
318	E-21	104.13	49.02	Eastern CAOB	Diorite	240	0	240	9.7	9.7	610	609	Li, S et al., 2013 Earth-Science Reviews
319	E-29	104.13	49.12	Eastern CAOB	Diorite	240	0	240	11.2	11.4	525	510	Li, S et al., 2013 Earth-Science Reviews
320	E-30	104.13	49.12	Eastern CAOB	Diorite	240	0	240	10.6	10.7	559	553	Li, S et al., 2013 Earth-Science Reviews
321	9HS49-7	79.98	46.97	Western CAOB	Diorite	332	0	332	14.7	14.4	393	411	Shen, P et al., 2018 Gondwana Research
322	12HS01-2	73.00	50.12	Western CAOB	Diorite	434	0	434	14.9	15.0	465	465	Shen, P et al., 2018 Gondwana Research
323	DK12-61	83.27	42.30	Western CAOB	Diorite	422	0	422	1.2	1.4	1244	1231	Wang, M et al., 2016 Journal of Asian Earth Sciences
324	HL-012	90.06	46.55	Western CAOB	Diorite	377	0	377	11.0	11.0	651	647	Wu, C et al., 2015 Ore Geology Reviews
325	HL-028	90.05	46.58	Western CAOB	Diorite	383	0	383	11.4	11.5	620	615	Wu, C et al., 2015 Ore Geology Reviews
326	TL-27	75.67	42.66	Western CAOB	Diorite	808	0	600	-2.6	-3.4	1503	1514	Zhao, X et al., 2015 Ore Geology Reviews
327	12ts57-3	86.25	42.67	Western CAOB	Diorite	423	0	423	-8.5	-9.7	1802	1869	Zhong, LL et al., 2015 Journal of Asian Earth Sciences
328	KTG39	89.68	46.76	Chinese Altai	Diorite	308	7	308	13.8	13.8	426	426	Gao JF et al., 2013 Lithos
329	HDHLS-1	86.27	41.97	Tianshan	Diorite	420	0	420	7.1	7.3	898	888	Qin et al, 2016 International Geology Review
330	4528	116.31	43.39	Uliastai active continental margin	Diorite	320	0	320	15.5	15.2	336	354	Li M et al., 2016 China Geology(ICWEA)
331	BLD-1.01	113.55	43.62	Baolidao arc-accretion complex	Diorite	310	0	310	8.5	8.9	734	711	Chen Bin., 2009 Journal of Asian Earth Sciences
332	XH-2-01	116.21	43.95	Baolidao arc-accretion complex	Diorite	310	0	310	9.2	8.5	695	737	Chen Bin., 2009 Journal of Asian Earth Sciences

333	14XL03.1	115.66	44.01	Baolidao arc-accretion complex	Diorite	484	0	484	-8.6	-8.8	1852	1861	Wang SQ, 2016 Earth Science
334	CGDLH04	110.82	41.89	Northern margin of the North China Craton	Diorite	239	0	239	-3.5	-3.6	1368	1378	Zhang W et al., 2010 Acta Geologica Sinica (ICWEA)
335	NM07-181	110.80	41.18	North China Craton	Diorite	279	0	279	-9.6	-9.6	1749	1749	Zhang XH et al., 2011 Lithos
336	JN07-37	111.46	41.48	North China Craton	Diorite	319	0	319	-12.6	-12.6	1953	1954	Zhang XH et al., 2012 JGSL
337	JB6024.1	117.11	41.23	North China Craton	Diorite	315	0	315	-16.5	-13.3	2168	1990	Wei YG et al., 2009 Acta Scientiarum Naturalium Universitatis Pekinensis (ICWEA)
338	CF06-66	118.98	41.92	North China Craton	Diorite	128	0	150	-8.6	-8.7	1592	1600	Wang YB et al., 2010 Acta Petrologica et Mineralogica (ICWEA)
339	HDL4-1	129.49	45.68	LXZR	diorite	450	6	450	0.1	0.0	1332	1335	Wang et al., 2012 Tectonophysics
340	11LJ-2.2	117.91	43.67	Erdaojing accretion complex	Diorite	141	2	150	5.9	5.9	761	762	Li S et al., 2017a, Tectonics, 2017b, GeolGeological Society of America Bulletin
341	08432-1	112.62	42.28	Northern margin of the North China Craton	Diorite	436	0	436	-0.3	1.6	1339	1235	Zhang SH et al., 2014 GeolGeological Society of America Bulletin
342	SZ07-15	111.94	41.42	North China Craton	Diorite	320	0	320	-16.9	-16.8	2196	2194	Zhang XH et al., 2012 JGSL
343	Hq06	115.20	41.07	North China Craton	Diorite	252	0	252	-10.1	-9.3	1755	1711	Wang F et al., 2009 Acta Petrologica Sinica
344	TH04-6	91.80	44.60	Eastern Junggar	diorite （host)	419	5.5	419	-28.0	-32.8	2893	3398	Xu Xingwang et al., 2015 Gondwana Research
345	TH4-6	91.80	44.60	Eastern Junggar	diorite （host)	419	5.5	419	-37.7	-37.7	3441	3479	Xu Xingwang et al., 2015 Gondwana Research
346	SGD1-1	117.07	40.45	North China craton	Diorite Mme	157	0	157	-15.6	-15.0	2000	1964	He, XF et al., 2017 Journal of the Geological Society
347	SGD2-2	117.08	40.46	North China craton	Diorite Mme	157	0	157	-13.4	-13.3	1875	1869	He, XF et al., 2017 Journal of the Geological Society
348	SW47	88.58	47.12	Chinese Altai	diorite porphyrite	401	0	401	2.9	3.1	1130	1118	Liu, 2012
349	HG	82.82	42.32	CAOB	diorites	424	2	424	0.4	0.5	1289	1286	Zhao ZY et al., 2015 Gondwana Research
350	06XJ134	84.56	45.69	Western Junggar	dioritic dike	312	3	312	14.2	14.7	405	380	Tang et al., 2012 Lithos
351	AK6	84.36	45.76	Western Junggar	Dioritic enclave	304	2	304	14.2	14.3	403	398	Geng et al., 2009 Chemical Geology
352	KM9802-11	84.82	45.70	Western Junggar	Dioritic enclave	306	5	306	14.6	14.7	377	372	Geng et al., 2009 Chemical Geology
353	TK1	117.28	36.38	Shandong Province	dolerite	2520	6.5	600	-42.4	-42.1	3835	3820	Sun T et al., 2020 Precambrian Research
354	PD-1	91.73	40.60	the Central Asian Orogenic Belt	Dolerite	270	0	270	5.8	5.5	858	877	Xue SC et al. 2018 Contributions to Mineralogy and Petrology
355	AGB-1	84.85	45.69	0	Enclave	301	2.3	301	10.4	10.6	617	609	Li S et al., 2015 Geoscience Frontiers
356	09AL05-1	105.20	39.68	westernmost North China	felsic	2329	9	600	-36.1	-37.2	3486	3548	Dan Wei et al., 2012 Gondwana Research
357	09AL120	105.15	39.55	westernmost North China	felsic	2344	12	600	-38.3	-38.3	3609	3609	Dan Wei et al., 2012 Gondwana Research
358	09AL02	105.28	39.78	westernmost North China	felsic	1891	9	600	-31.1	-30.8	3212	3193	Dan Wei et al., 2012 Gondwana Research
359	09AL142	105.50	39.84	westernmost North China	felsic	2334	6	600	-36.2	-37.2	3495	3550	Dan Wei et al., 2012 Gondwana Research
360	09AL125	105.05	39.41	westernmost North China	felsic	1895	28	600	-37.8	-38.0	3585	3594	Dan Wei et al., 2012 Gondwana Research
361	BJ1-1	116.05	39.92	the North China	felsic	177	3	177	4.9	4.9	840	838	He XF et al., 2017 Bulletin
362	BJ2-1	116.10	39.97	the North China	felsic	177	3	177	-24.8	-15.7	2531	2017	He XF et al., 2017 Bulletin
363	BJ11	115.92	39.72	the North China	felsic	157	6	157	-24.6	-25.6	2509	2566	He XF et al., 2017 Bulletin
364	BJ2-2	116.10	39.97	the North China	felsic	164	2	164	-32.1	-32.8	2934	2972	He XF et al., 2017 Bulletin
365	BJ5-2	115.98	40.00	the North China	felsic	151	1	151	-19.5	-15.6	2212	1994	He XF et al., 2017 Bulletin
366	BJ7-1	115.73	40.03	the North China	felsic	172	1	172	-11.1	-12.6	1756	1840	He XF et al., 2017 Bulletin
367	06XJ123	84.95	45.74	Western Junggar	felsic dike	304	3	304	14.8	15.0	365	356	Tang et al., 2012 Lithos
368	X0499	88.25	47.57	Western CAOB	Felsic Gneiss	404	1.7	404	16.4	16.0	404	404	Yang, TN et al., 2011 Precambrian Research
369	11JD02-3	118.21	40.38	North China craton	Felsic Gneiss	2614	0	600	-40.0	-39.9	3705	3701	Guo, RR et al., 2013 Gondwana Research
370	11JD17-2	118.14	40.26	North China craton	Felsic Gneiss	2609	0	600	-41.4	-41.4	3780	3782	Guo, RR et al., 2013 Gondwana Research
371	12FX09-1	121.80	42.36	North China craton	Felsic Gneiss	2534	0	600	-38.2	-38.1	3603	3601	Wang, W et al., 2015 Earth-Science Reviews
372	12FX18-2	121.29	42.31	North China craton	Felsic Gneiss	2640	0	600	-38.8	-39.1	3637	3653	Wang, W et al., 2015 Earth-Science Reviews
373	FX009-1	121.80	42.36	North China craton	Felsic Gneiss	2534	0	600	-38.1	-38.1	3601	3601	Wang, W et al., 2011 Gondwana Research
374	FX013-2	121.40	42.35	North China craton	Felsic Gneiss	2589	0	600	-38.5	-38.4	3620	3615	Wang, W et al., 2011 Gondwana Research
375	YX011-2	121.25	41.73	North China craton	Felsic Gneiss	2567	0	600	-38.5	-38.3	3621	3612	Wang, W et al., 2011 Gondwana Research
376	K-19-04	110.60	66.10	Eastern CAOB	Felsic Gneiss	1909	0	600	-30.7	-30.3	3190	3168	Shatsky, VS et al., 2016 Precambrian Research
377	K-3-02	110.60	66.10	Eastern CAOB	Felsic Gneiss	1904	0	600	-30.4	-30.1	3171	3155	Shatsky, VS et al., 2016 Precambrian Research
378	K-45-02	110.60	66.10	Eastern CAOB	Felsic Gneiss	1818	0	600	-42.8	-41.6	3863	3794	Shatsky, VS et al., 2016 Precambrian Research
379	K-67-04	110.60	66.10	Eastern CAOB	Felsic Gneiss	1900	0	600	-30.8	-31.2	3191	3214	Shatsky, VS et al., 2016 Precambrian Research
380	K-68-04	110.60	66.10	Eastern CAOB	Felsic Gneiss	1881	0	600	-29.1	-29.3	3100	3111	Shatsky, VS et al., 2016 Precambrian Research
381	K-69-02	110.60	66.10	Eastern CAOB	Felsic Gneiss	2004	0	600	-35.4	-31.2	3450	3214	Shatsky, VS et al., 2016 Precambrian Research
382	K-72-03	110.60	66.10	Eastern CAOB	Felsic Gneiss	1909	0	600	-34.7	-34.9	3412	3422	Shatsky, VS et al., 2016 Precambrian Research
383	13JN21-3	127.41	42.88	North China craton	Felsic Gneiss	2579	0	600	-40.7	-40.7	3742	3746	Guo, B.R et al., 2016 Precambrian Research
384	13JN21-6	127.41	42.88	North China craton	Felsic Gneiss	2544	0	600	-39.9	-39.8	3702	3695	Guo, B.R et al., 2016 Precambrian Research
385	13JN22-5	127.31	42.90	North China craton	Felsic Gneiss	2592	0	600	-38.8	-38.8	3637	3639	Guo, B.R et al., 2016 Precambrian Research
386	14JN13-1	127.45	42.96	North China craton	Felsic Gneiss	2688	0	600	-43.0	-43.2	3874	3883	Guo, B.R et al., 2016 Precambrian Research
387	14JN17-1	127.31	42.87	North China craton	Felsic Gneiss	2536	0	600	-41.7	-41.6	3802	3795	Guo, B.R et al., 2016 Precambrian Research
388	TH26-1	125.50	41.50	North China craton	Felsic Gneiss	2495	0	600	-42.7	-39.5	3853	3679	Meng, E et al., 2017 Precambrian Research
389	TH48-1	125.50	41.50	North China craton	Felsic Gneiss	1904	0	600	-24.8	-24.9	2858	2860	Meng, E et al., 2017 Precambrian Research
390	TH48-2	125.50	41.50	North China craton	Felsic Gneiss	2203	0	600	-29.3	-28.7	3108	3078	Meng, E et al., 2017 Precambrian Research
391	TH53-3	125.50	41.50	North China craton	Felsic Gneiss	2185	0	600	-32.5	-33.4	3285	3336	Meng, E et al., 2017 Precambrian Research
392	TH59-1	125.50	41.50	North China craton	Felsic Gneiss	2173	0	600	-30.1	-30.1	3151	3152	Meng, E et al., 2017 Precambrian Research
393	M107/06	98.14	55.26	Eastern CAOB	Felsic Gneiss	350	0	350	8.0	7.9	794	800	Kröner, A et al., 2017 Gondwana Research
394	M74/06	97.81	44.87	Western CAOB	Felsic Gneiss	364	0	364	8.8	8.8	761	762	Kröner, A et al., 2017 Gondwana Research
395	15YK01-3	122.49	40.68	North China craton	Felsic Gneiss	2500	0	600	-40.4	-40.8	3725	3751	Guo, RR et al., 2017 Precambrian Research
396	14GW095	130.00	45.50	Eastern CAOB	Felsic Gneiss	752	0	600	-1.1	-0.5	1509	1484	Yang, H et al., 2018 Gondwana Research
397	14GW097	130.00	45.50	Eastern CAOB	Felsic Gneiss	752	0	600	-3.1	-2.7	1624	1604	Yang, H et al., 2018 Gondwana Research
398	14GW113	130.00	45.50	Eastern CAOB	Felsic Gneiss	752	0	600	-2.6	-2.0	1596	1562	Yang, H et al., 2018 Gondwana Research
399	15LX01?	121.32	41.19	North China craton	Felsic Gneiss	2522	0	600	-37.7	-37.7	3575	3576	Fu, JH et al., 2018 Lithos
400	15LX19?	121.09	40.93	North China craton	Felsic Gneiss	2522	0	600	-38.2	-38.0	3605	3597	Fu, JH et al., 2018 Lithos
401	15LX21?	121.06	40.88	North China craton	Felsic Gneiss	2510	0	600	-38.2	-38.5	3607	3622	Fu, JH et al., 2018 Lithos
402	15LX88?	120.82	40.51	North China craton	Felsic Gneiss	2547	0	600	-38.2	-38.1	3607	3598	Fu, JH et al., 2018 Lithos
403	05-01	69.44	53.22	Western CAOB	Felsic Gneiss	1054	0	600	-19.9	-19.9	2580	2580	Glorie, S et al., 2015 Gondwana Research
404	43958	69.44	53.22	Western CAOB	Felsic Gneiss	1054	0	600	-18.5	-20.2	2470	2600	Glorie, S et al., 2015 Gondwana Research
405	04-51	69.42	53.25	Western CAOB	Felsic Gneiss	1141	0	600	-18.4	-20.7	2479	2626	Glorie, S et al., 2015 Gondwana Research
406	X10-13-1	94.89	41.86	Western CAOB	Felsic Gneiss	1382	0	600	-14.8	-11.5	2295	2109	He, Z.Y et al., 2014 Lithos
407	X10-17-1	95.01	41.81	Western CAOB	Felsic Gneiss	927	0	600	-4.7	-3.3	1677	1606	He, Z.Y et al., 2014 Lithos
408	X10-17-2	95.01	41.81	Western CAOB	Felsic Gneiss	916	0	600	-10.2	-8.2	2010	1918	He, Z.Y et al., 2014 Lithos
409	X11-90-1	95.03	41.81	Western CAOB	Felsic Gneiss	884	0	600	-3.7	-3.9	1642	1658	He, Z.Y et al., 2014 Lithos
410	X11-75-1	94.11	41.75	Western CAOB	Felsic Gneiss	1433	0	600	-12.9	-12.6	2185	2167	He, Z.Y et al., 2015 Lithos
411	X12-33-1	94.99	41.91	Western CAOB	Felsic Gneiss	1409	0	600	-11.2	-12.2	2055	2144	He, Z.Y et al., 2015 Lithos
412	X12-38-2	92.64	41.72	Western CAOB	Felsic Gneiss	1437	0	600	-13.4	-13.2	2217	2205	He, Z.Y et al., 2015 Lithos
413	X12-39-1	92.64	41.71	Western CAOB	Felsic Gneiss	1436	0	600	-12.6	-12.8	2171	2178	He, Z.Y et al., 2015 Lithos
414	X12-39-2	92.64	41.71	Western CAOB	Felsic Gneiss	1438	0	600	-13.7	-13.6	2234	2228	He, Z.Y et al., 2015 Lithos
415	X12-40-1	92.64	41.71	Western CAOB	Felsic Gneiss	1436	0	600	-13.4	-13.3	2215	2210	He, Z.Y et al., 2015 Lithos
416	X12-41-1	92.64	41.70	Western CAOB	Felsic Gneiss	1436	0	600	-12.1	-12.4	2143	2158	He, Z.Y et al., 2015 Lithos

417	X12-42-1	92.63	41.69	Western CAOB	Felsic Gneiss	1436	0	600	-12.3	-11.5	2151	2105	He, Z.Y et al., 2015 Lithos
418	X12-43-2	92.61	41.71	Western CAOB	Felsic Gneiss	1436	0	600	-14.4	-14.4	2271	2270	He, Z.Y et al., 2015 Lithos
419	KBD1	86.84	43.10	Western CAOB	Felsic Gneiss	907	0	600	-12.4	-11.8	2159	2124	Huang, Z et al., 2015 Precambrian Research
420	KBD7	86.84	43.10	Western CAOB	Felsic Gneiss	894	0	600	-9.9	-10.3	2018	2037	Huang, Z et al., 2015 Precambrian Research
421	XBL1-2	85.45	43.14	Western CAOB	Felsic Gneiss	794	0	600	-7.9	-8.2	1906	1918	Huang, Z et al., 2015 Precambrian Research
422	XBY6-1	84.36	43.17	Western CAOB	Felsic Gneiss	925	0	600	-8.1	-7.6	1915	1885	Huang, Z et al., 2015 Precambrian Research
423	X3ET134	91.81	41.57	Western CAOB	Felsic Gneiss	919	0	600	-14.1	-9.2	2252	1974	Huang, Z et al., 2015 Precambrian Research
424	X3ET189	91.88	41.67	Western CAOB	Felsic Gneiss	895	0	600	-9.9	-9.1	2016	1972	Huang, Z et al., 2015 Precambrian Research
425	KG1	75.88	42.69	Western CAOB	Felsic Gneiss	844	0	600	-22.9	-23.0	2751	2754	Kröner, A et al., 2012 Gondwana Research
426	KG5	76.13	42.88	Western CAOB	Felsic Gneiss	834	0	600	-11.4	-10.7	2099	2062	Kröner, A et al., 2012 Gondwana Research
427	KG14	75.88	42.31	Western CAOB	Felsic Gneiss	1153	0	600	-22.1	-21.9	2703	2692	Kröner, A et al., 2012 Gondwana Research
428	T5-140	72.44	42.50	Western CAOB	Felsic Gneiss	1133	0	600	-14.1	-14.9	2246	2300	Kröner, A et al., 2012 Gondwana Research
429	KB13	82.39	43.18	Western CAOB	Felsic Gneiss	940	0	600	-10.6	-10.0	2057	2023	Xiong, FH et al., 2019 Precambrian Research
430	MO104	102.35	47.54	Eastern CAOB	Felsic Tuff	396	0	396	2.8	3.1	1129	1114	Kröner, A et al., 2017 Gondwana Research
431	16W29	122.38	46.12	0	Felsic volcanic rock	127	2	150	5.8	5.3	762	794	Tang ZY et al., 2019 GJ, 2020 International Geology Review
432	MO176	101.96	45.81	Eastern CAOB	Felsic Volcanic Rock	492	0	492	9.4	9.4	831	830	Kröner, A et al., 2017 Gondwana Research
433	MO200	99.54	44.45	Eastern CAOB	Felsic Volcanic Rock	358	0	358	2.7	3.5	1107	1062	Kröner, A et al., 2017 Gondwana Research
434	MO309	100.87	44.71	Eastern CAOB	Felsic Volcanic Rock	441	0	441	-3.3	-3.8	1517	1548	Kröner, A et al., 2017 Gondwana Research
435	CXPC7-1	118.74	44.53	0	porphyritic biotite granite	138	1	150	8.2	8.1	625	634	Wei W et al., 2021 Global Geology(ICWEA)
436	CDE4111-2B	119.22	44.41	0	Fine-grained granite	137	0	150	6.9	7.0	701	698	Zhang ZQ et al., 2017 Mineral Exploration (ICWEA)
437	HX–9	123.87	51.16	0	Fine-grained granite	148	2	150	6.4	6.7	731	716	Liu J et al., 2014 Journal of Asian Earth Sciences
438	13GF-16	128.23	42.81	0	Garnet granite	165	3	165	5.1	5.5	816	795	Guo F et al., 2019 Chemical Geology
439	13YB8-1	129.65	42.57	CAOB	gneiss	423	0	423	-0.6	-0.4	1362	1356	Wang ZW et al., 2016 Gondwana Research
440	13YB8-1	129.65	42.57	CAOB	gneiss	423	0	423	-0.6	-0.4	1362	1356	Wang ZW et al., 2016 Gondwana Research
441	YH13-1	129.65	42.57	CAOB	gneiss	514	0	514	-4.6	1.3	1649	1253	Wang ZW et al., 2016 Gondwana Research
442	J2-1	87.30	48.44	Chinese Altai	gneissic biotite granite	466	3	466	6.1	5.6	998	1029	Sun Min et al., 2009 Science in China Series D: Earth Sciences
443	Bu6-1	87.00	48.05	Chinese Altai	gneissic biotite granite	415	5	415	9.0	8.7	792	805	Sun Min et al., 2009 Science in China Series D: Earth Sciences
444	HSW6-4	127.34	49.72	0	neissic biotite syenogranit	183	2	183	8.0	7.6	662	688	Xu MJ et al., 2013 Acta Petrologica Sinica (ICWEA)
445	HSW6-12	127.34	49.72	0	neissic biotite syenogranit	185	2	185	7.9	7.7	670	680	Xu MJ et al., 2013 Acta Petrologica Sinica (ICWEA)
446	J8	87.32	48.49	Chinese Altai	Gneissic granite	479	3	479	5.5	5.4	1044	1050	Cai Keda et al., 2011 Journal of Asian Earth Sciences
447	CH15	87.65	47.91	Chinese Altai	Gneissic granite	432	7	432	5.4	5.5	1012	1007	Cai Keda et al., 2011 Journal of Asian Earth Sciences
448	bu-1	86.88	48.03	habahedong	Gneissic granitoid	427	0	427	5.1	5.0	1027	1029	Sun et al., 2008
449	LK03-1	124.44	42.88	0	Gneissic granodiorite	165	1	165	1.6	1.8	1018	1009	Zhang HH et al., 2016 Lithos
450	638	86.33	42.66	Tianshan	Gneissic granodiorites	475	2	475	-8.1	-8.3	1820	1830	Ma XX et al., 2013 International Geology Review
451	LK20-2	125.44	43.38	0	Gneissic monzogranite	175	1	175	-10.1	-10.0	1698	1693	Zhang HH et al., 2016 Lithos
452	CH15-7	87.65	47.91	taerlang	gneissic two-mica granite	355	0	355	3.5	3.8	1057	1041	Sun et al., 2009
453	08KT03-1	77.14	40.25	0	Granite	278	3	278	7.5	7.6	767	760	Zhang et al., 2010
454	2010HLJIV-3	77.21	40.37	0	Granite	269	2	269	4.0	3.9	960	970	Zhang and Zou, 2013 Lithos
455	09HLJV-2	77.22	40.36	0	Granite	271	2	271	6.0	5.8	847	859	Zhang and Zou, 2013 Lithos
456	YQ-1	85.98	42.01	0	Granite	408	5	408	5.4	6.0	993	958	Qin et al., 2016 International Geology Review
457	HLS-11	85.97	42.06	0	Granite	406	6	406	4.4	4.4	1046	1048	Qin et al., 2016 International Geology Review
458	QGX-7	86.21	42.02	0	Granite	385	6	385	-0.8	-1.4	1330	1364	Qin et al., 2016 International Geology Review
459	797-1	87.11	42.81	Tianshan	Granite	346	3	346	3.8	3.9	1036	1031	Ma XX et al.,2014 Gondwana Research
460	WLSTII-3	86.56	42.90	0	Granite	359	4	359	3.2	2.9	1077	1097	Huang H et al., 2015 Acta Geologica Sinica (ICWEA)
461	XSS2	88.45	42.35	0	Granite	404	13	404	1.0	0.7	1239	1260	Shi et al., 2014 International Geology Review
462	XSS3	88.45	42.35	0	Granite	405	14	405	0.8	1.0	1252	1242	Shi et al., 2014 International Geology Review
463	XXX01	88.52	42.33	0	Granite	393	3	393	-12.7	-12.7	2015	2017	Shi YR et al., 2014 International Geology Review
464	YSG12	88.48	42.30	0	Granite	399	2	399	0.2	0.7	1282	1253	Shi YR et al., 2014 International Geology Review
465	11WJ70	85.56	46.69	0	Granite	412	5.4	412	12.8	12.6	568	577	Tang et al., 2019 Earth and Planetary Science Letters
466	N1825	85.46	46.65	0	Granite	429	2.5	429	13.6	13.7	538	528	Yang G et al., 2015 Earth Science
467	N4814	85.30	46.70	0	Granite	428	2.3	428	13.2	13.2	560	558	Yang G et al., 2015 Earth Science
468	ER12-1	120.03	50.86	Eastern CAOB	Granite	229	4	229	3.1	3.2	981	978	Tang J et al., 2016 Gondwana Research
469	13ER6-1	121.50	51.34	Eastern CAOB	Granite	196	1	196	3.8	3.5	916	933	Tang J et al., 2016 Gondwana Research
470	ER6-1	120.16	50.64	Eastern CAOB	Granite	186	2	186	7.3	7.1	708	717	2016 Gondwana Research; Wang W et al., 2012 Geological Journal of China Universities(ICWEA)
471	ZKS1-1	120.08	50.81	Eastern CAOB	Granite	185	1	185	7.8	7.8	679	680	2016 Gondwana Research; Wang W et al., 2012 Geological Journal of China Universities(ICWEA)
472	MZ17-1	117.30	49.42	Eastern CAOB	Granite	183	2	183	4.4	4.4	872	871	2016 Gondwana Research; Wang W et al., 2012 Geological Journal of China Universities(ICWEA)
473	MZ18-2	117.32	49.42	Eastern CAOB	Granite	180	2	180	8.0	7.6	661	683	Tang J et al., 2016 Gondwana Research
474	SPM9-5-1	124.53	51.87	0	Granite	124	1	150	2.6	2.2	951	975	Xu LP, 2017 China University of Geosciences, Beijing. Master's thesis
475	17LCD-03	122.27	47.83	0	Granite	137	1	150	8.6	8.8	603	589	Qu P et al., 2019 Lithos
476	17CHZ-13	121.32	47.48	0	Granite	138	1	150	9.8	9.9	535	529	Qu P et al., 2019 Lithos
477	17JJG-10	122.68	47.75	0	Granite	137	3	150	9.8	10.2	531	513	Qu P et al., 2019 Lithos
478	BD1-1	117.54	44.01	0	Granite	140	2	150	6.9	6.6	700	722	Wu G et al., 2021 Acta Petrologica Sinica (ICWEA)
479	H15-40-1	129.98	47.38	0	Granite	241	1	241	-2.0	-1.4	1288	1251	Ge MH et al., 2018 Lithos
480	B05	119.12	49.98	0	Granite	244	1	244	5.9	5.9	835	836	Kang YJ et al., 2018 Ore Geology Reviews
481	w-9	117.28	49.43	Eastern CAOB	Granite	180	1	180	4.0	3.9	892	901	Wang YH et al., 2015 Gondwana Research
482	w-1	117.29	49.43	Eastern CAOB	Granite	204	2	204	0.5	0.7	1114	1104	Wang YH et al., 2015 Gondwana Research
483	F04-033	119.45	40.10	North China craton	Granite	117	0	150	-10.9	-11.1	1724	1734	Yang, JH et al., 2008 Journal of Petrology
484	F04-036	119.45	40.10	North China craton	Granite	117	0	150	-10.0	-10.2	1672	1684	Yang, JH et al., 2008 Journal of Petrology
485	F04-073	119.75	40.05	North China craton	Granite	118	0	150	-9.5	-9.6	1646	1648	Yang, JH et al., 2008 Journal of Petrology
486	F04-106	117.55	40.68	North China craton	Granite	129	0	150	-8.8	-8.9	1606	1613	Yang, JH et al., 2008 Journal of Petrology
487	9411-A	122.77	48.00	Eastern CAOB	Granite	130	0	150	10.6	10.3	484	505	Zhang, JH et al., 2006 Acta Geologica Sinica-English Edition
488	06XJ-13	81.42	44.74	Western CAOB	Granite	289	3	289	5.2	4.9	908	924	Tang, GJ et al., 2010 Lithos
489	HN01	87.08	48.70	Western CAOB	Granite	443	0	443	4.6	4.4	1064	1079	Wang, YJ et al., 2011 Journal of Asian Earth Sciences
490	HN16	87.18	48.46	Western CAOB	Granite	443	0	443	3.8	4.1	1113	1093	Wang, YJ et al., 2011 Journal of Asian Earth Sciences
491	JDY12	87.18	48.52	Western CAOB	Granite	445	0	445	5.1	5.6	1036	1013	Wang, YJ et al., 2011 Journal of Asian Earth Sciences
492	TH5	91.79	44.61	Western CAOB	Granite	449	1.5	449	13.1	13.3	582	568	Xu, XW et al., 2013 Gondwana Research
493	TH6	91.80	44.60	Western CAOB	Granite	443	1.7	443	13.2	13.2	568	567	Xu, XW et al., 2013 Gondwana Research
494	TH8	91.80	44.60	Western CAOB	Granite	432	1.4	432	13.0	14.9	569	461	Xu, XW et al., 2013 Gondwana Research
495	ZF10-1	91.80	44.60	Western CAOB	Granite	455	1.7	455	10.5	12.5	737	622	Xu, XW et al., 2013 Gondwana Research
496	PY	91.56	40.60	Western CAOB	Granite	251	0	251	2.4	2.4	1042	1041	Su, BX et al., 2011 Gondwana Research
497	SH-11	95.80	42.50	Western CAOB	Granite	398	0	398	14.2	14.0	476	486	Su, BX et al., 2011 Gondwana Research
498	SH-R	95.80	42.50	Western CAOB	Granite	441	0	441	14.6	14.5	487	493	Su, BX et al., 2011 Gondwana Research
499	SH-V	95.80	42.50	Western CAOB	Granite	365	0	365	12.7	12.9	535	524	Su, BX et al., 2011 Gondwana Research
500	JS4-1	126.26	44.42	Eastern CAOB	Granite	340	0	340	9.8	9.5	685	701	Wang ZW et al., 2015 Journal of Asian Earth Sciences

501	C12FY21	89.70	46.98	Western CAOB	Granite	400	0	400	-9.9	-4.6	1856	1561	He YL et al., 2015 Journal of Asian Earth Sciences
502	FX-1	92.00	42.00	Western CAOB	Granite	332	0	332	16.1	16.1	332	332	Wang YH et al., 2016 Gondwana Research
503	KG103	71.00	41.51	Western CAOB	Granite	320	0	320	2.2	2.1	1109	1114	Alexeiev DV et al., 2016 Gondwana Research
504	KG95	72.02	41.65	Western CAOB	Granite	467	0	467	-2.2	-2.9	1476	1513	Alexeiev DV et al., 2016 Gondwana Research
505	M103/06	97.95	45.46	Western CAOB	Granite	341	0	341	7.3	7.3	829	828	Kröner A et al., 2017 Gondwana Research
506	M83/06	97.79	44.99	Western CAOB	Granite	280	0	280	8.1	8.7	737	699	Kröner A et al., 2017 Gondwana Research
507	MO130	100.79	44.47	Eastern CAOB	Granite	467	0	467	6.4	6.7	980	966	Kröner A et al., 2017 Gondwana Research
508	JN1	127.50	41.50	North China craton	Granite	1845	0	600	-28.9	-27.9	3088	3030	Liu FL et al., 2017 Precambrian Research
509	JN2	127.50	41.50	North China craton	Granite	1845	0	600	-28.9	-28.7	3087	3073	Liu FL et al., 2017 Precambrian Research
510	JN3	127.50	41.50	North China craton	Granite	1844	0	600	-28.2	-27.5	3049	3006	Liu FL et al., 2017 Precambrian Research
511	JN4	127.50	41.50	North China craton	Granite	1843	0	600	-27.4	-27.0	3002	2981	Liu FL et al., 2017 Precambrian Research
512	JN5	127.50	41.50	North China craton	Granite	1844	0	600	-30.3	-28.3	3164	3053	Liu FL et al., 2017 Precambrian Research
513	JN6	125.50	41.00	North China craton	Granite	1842	0	600	-29.5	-28.5	3121	3066	Liu FL et al., 2017 Precambrian Research
514	JN7	125.50	41.00	North China craton	Granite	1845	0	600	-27.1	-27.0	2984	2980	Liu FL et al., 2017 Precambrian Research
515	JN8	125.50	41.00	North China craton	Granite	1847	0	600	-27.6	-27.6	3011	3014	Liu FL et al., 2017 Precambrian Research
516	13XX5C	95.00	42.00	Western CAOB	Granite	883	0	600	-5.5	-3.4	1537	1407	Zhang XR et al., 2017 Journal of Asian Earth Sciences
517	13YM8A	94.00	42.00	Western CAOB	Granite	245	0	245	2.2	3.3	1057	1000	Zhang XR et al., 2017 Journal of Asian Earth Sciences
518	13YM8C	94.00	42.00	Western CAOB	Granite	249	0	249	1.8	1.8	1078	1080	Zhang XR et al., 2017 Journal of Asian Earth Sciences
519	4-1CN	118.25	45.80	Eastern CAOB	Granite	152	0	152	7.5	7.5	667	668	Zhu PP et al., 2017 Geological Magazine
520	4-2CN	118.25	45.80	Eastern CAOB	Granite	158	0	158	8.1	8.1	643	643	Zhu PP et al., 2017 Geological Magazine
521	9-1CN	118.25	45.80	Eastern CAOB	Granite	152	0	152	7.8	7.8	649	661	Zhu PP et al., 2017 Geological Magazine
522	08AET048	90.00	46.00	Western CAOB	Granite	300	0	300	12.7	12.7	484	482	Tang GJ et al., 2017 Geochemistry Geophysics Geosystems
523	14HT18-1	129.81	48.29	Eastern CAOB	Granite	917	0	600	-9.9	-10.5	2019	2052	Luan JP et al., 2017 Journal of Asian Earth Sciences
524	14HT19-1	129.79	48.31	Eastern CAOB	Granite	915	0	600	-8.2	-8.4	1919	1933	Luan JP et al., 2017 Journal of Asian Earth Sciences
525	H15-63-4	127.50	45.28	Eastern CAOB	Granite	181	0	181	8.5	8.4	633	638	Ge MH et al., 2017 Tectonophysics
526	HM01	87.60	48.60	Western CAOB	Granite	563	0	563	2.6	2.4	1159	1171	Cai KD et al., 2017 Lithos
527	HM2-5	87.58	48.69	Western CAOB	Granite	2624	0	600	-7.6	5.0	2624	2624	Cai KD et al., 2017 Lithos
528	KW12	89.24	47.49	Western CAOB	Granite	419	0	419	4.1	4.2	1078	1066	Cai KD et al., 2017 Lithos
529	SGD2-1	117.07	40.45	North China craton	Granite	161	0	161	-13.6	-13.9	1885	1903	He XF et al., 2017 Journal of the Geological Society
530	SGD4-1	117.08	40.46	North China craton	Granite	162	0	162	-14.4	-14.3	1934	1927	He XF et al., 2017 Journal of the Geological Society
531	A1103	123.13	41.09	North China craton	Granite	2995	0	600	-60.9	-58.8	4858	4742	Dong CY et al., 2017 Lithos
532	A1105	123.19	41.09	North China craton	Granite	3146	0	600	-58.4	-59.3	4718	4768	Dong CY et al., 2017 Lithos
533	A1110	123.37	41.11	North China craton	Granite	2929	0	600	-57.2	-57.5	4652	4668	Dong CY et al., 2017 Lithos
534	A1113	123.49	41.11	North China craton	Granite	2948	0	600	-54.8	-53.1	4517	4425	Dong CY et al., 2017 Lithos
535	A1201	123.30	41.08	North China craton	Granite	2946	0	600	-55.9	-55.2	4582	4542	Dong CY et al., 2017 Lithos
536	A1203	123.30	41.08	North China craton	Granite	2925	0	600	-57.1	-57.6	4646	4671	Dong CY et al., 2017 Lithos
537	10BSD-13	96.00	42.50	Western CAOB	Granite	293	0	293	6.8	6.8	823	822	Cao MJ et al., 2017 Lithos
538	10BSD-9	96.00	42.50	Western CAOB	Granite	294	0	294	8.3	8.6	734	719	Cao MJ et al., 2017 Lithos
539	BS-16	96.00	42.50	Western CAOB	Granite	293	0	293	6.9	6.3	816	850	Cao MJ et al., 2017 Lithos
540	BS-17	96.00	42.50	Western CAOB	Granite	292	0	292	6.9	7.0	814	806	Cao MJ et al., 2017 Lithos
541	BSZK15-5-405	96.00	42.50	Western CAOB	Granite	294	0	294	6.3	6.6	850	834	Cao MJ et al., 2017 Lithos
542	FZ-GR	113.15	40.44	North China craton	Granite	2001	0	600	-28.8	-28.6	3079	3067	Zeng L et al., 2017 Precambrian Research
543	BT06-2	110.00	41.00	North China craton	Granite	2451	0	600	-36.6	-36.6	3513	3514	Liu JH et al., 2017 Precambrian Research
544	BT15-1	110.00	41.00	North China craton	Granite	2446	0	600	-36.2	-36.2	3491	3495	Liu JH et al., 2017 Precambrian Research
545	BT17-2	110.00	41.00	North China craton	Granite	2441	0	600	-35.7	-36.8	3464	3526	Liu JH et al., 2017 Precambrian Research
546	BT29-1	110.00	41.00	North China craton	Granite	2443	0	600	-35.9	-36.1	3476	3490	Liu JH et al., 2017 Precambrian Research
547	BT42-4	110.00	41.00	North China craton	Granite	2526	0	600	-37.4	-37.1	3560	3542	Liu JH et al., 2017 Precambrian Research
548	BT45-1	110.00	41.00	North China craton	Granite	2516	0	600	-38.0	-38.0	3595	3593	Liu JH et al., 2017 Precambrian Research
549	BT48-2	110.00	41.00	North China craton	Granite	2406	0	600	-37.4	-37.3	3563	3554	Liu JH et al., 2017 Precambrian Research
550	BT58-2	110.00	41.00	North China craton	Granite	2505	0	600	-38.1	-38.2	3603	3605	Liu JH et al., 2017 Precambrian Research
551	14HC31	111.02	41.43	North China craton	Granite	2463	0	600	-39.9	-40.0	3700	3708	Chen NHC et al., 2017 Precambrian Research
552	14HC34-1	111.34	41.43	North China craton	Granite	2465	0	600	-40.3	-40.1	3721	3713	Chen NHC et al., 2017 Precambrian Research
553	14HC39	111.00	41.00	North China craton	Granite	2533	0	600	-40.6	-40.3	3740	3722	Chen NHC et al., 2017 Precambrian Research
554	14HC53-1	111.77	41.29	North China craton	Granite	2407	0	600	-38.9	-38.9	3644	3646	Chen NHC et al., 2017 Precambrian Research
555	C14TL55	82.00	45.00	Western CAOB	Granite	288	0	288	11.4	11.5	552	544	Wang XS et al., 2018 Journal of Asian Earth Sciences
556	YH15-001	92.50	42.00	Western CAOB	Granite	330	0	330	12.8	12.6	502	516	Zheng RG et al., 2018 Journal of Asian Earth Sciences
557	BA11-96	96.80	41.86	Western CAOB	Granite	401	0	401	3.5	3.9	1095	1074	Zhang C et al., 2018 Journal of Asian Earth Sciences
558	KUJIBEIEA	84.00	47.00	Western CAOB	Granite	329	0	329	9.6	9.9	686	668	Zhang C et al., 2018 Journal of Asian Earth Sciences
559	KUJIBEIEB	84.00	47.00	Western CAOB	Granite	341	0	341	9.8	9.8	686	684	Zhang C et al., 2018 Journal of Asian Earth Sciences
560	KUJIBEIEC	84.00	47.00	Western CAOB	Granite	330	0	330	10.1	10.0	659	666	Zhang C et al., 2018 Journal of Asian Earth Sciences
561	QIBAERTIE	84.00	47.00	Western CAOB	Granite	339	0	339	10.4	11.2	648	601	Zhang C et al., 2018 Journal of Asian Earth Sciences
562	TAERBAHA	83.00	47.00	Western CAOB	Granite	328	0	328	10.0	9.8	660	676	Zhang C et al., 2018 Journal of Asian Earth Sciences
563	WUERKASHIERE	84.00	47.00	Western CAOB	Granite	380	0	380	10.9	10.9	651	650	Zhang C et al., 2018 Journal of Asian Earth Sciences
564	X10-48-1	97.10	42.05	Western CAOB	Granite	446	0	446	3.0	3.2	1158	1149	Yuan Y et al., 2018 Lithos
565	X10-49-1	97.09	42.04	Western CAOB	Granite	444	0	444	2.9	3.4	1167	1137	Yuan Y et al., 2018 Lithos
566	X10-50-1	97.08	42.04	Western CAOB	Granite	443	0	443	2.1	2.7	1208	1176	Yuan Y et al., 2018 Lithos
567	X10-51-1	97.08	42.03	Western CAOB	Granite	433	0	433	2.7	2.6	1169	1175	Yuan Y et al., 2018 Lithos
568	X10-52-1	97.08	42.02	Western CAOB	Granite	430	0	430	3.5	3.7	1122	1106	Yuan Y et al., 2018 Lithos
569	X10-54-1	97.07	42.00	Western CAOB	Granite	438	0	438	-0.2	-0.4	1339	1349	Yuan Y et al., 2018 Lithos
570	X10-69-1	98.37	41.81	Eastern CAOB	Granite	445	0	445	-4.5	-4.8	1587	1608	Yuan Y et al., 2018 Lithos
571	X10-72-1	98.38	41.89	Eastern CAOB	Granite	433	0	433	-6.9	-7.0	1717	1724	Yuan Y et al., 2018 Lithos
572	X10-77-1	98.41	41.95	Eastern CAOB	Granite	439	0	439	-6.8	-6.4	1716	1692	Yuan Y et al., 2018 Lithos
573	15XH2-1	128.28	45.50	Eastern CAOB	Granite	393	0	393	11.1	11.3	639	623	Guo P et al., 2018 Gondwana Research
574	AKETAS	89.50	47.00	Western CAOB	Granite	308	0	308	12.4	12.3	510	514	Zhang C et al., 2018 Journal of Asian Earth Sciences
575	KADELAT	89.50	47.00	Western CAOB	Granite	279	0	279	-3.9	-4.9	1424	1483	Zhang C et al., 2018 Journal of Asian Earth Sciences
576	WUKULI	90.00	46.50	Western CAOB	Granite	305	0	305	-1.9	-1.9	1332	1329	Zhang C et al., 2018 Journal of Asian Earth Sciences
577	P20-4	113.00	44.00	Eastern CAOB	Granite	283	0	283	11.9	12.0	519	514	Chai H et al., 2018 Lithos
578	P20-9	113.00	44.00	Eastern CAOB	Granite	280	0	280	12.1	11.9	503	515	Chai H et al., 2018 Lithos
579	P23-13	113.00	44.00	Eastern CAOB	Granite	285	0	285	12.3	12.1	496	505	Chai H et al., 2018 Lithos
580	P23-7	113.00	44.00	Eastern CAOB	Granite	291	0	291	12.3	12.1	502	515	Chai H et al., 2018 Lithos
581	78	61.84	57.12	Western CAOB	Granite	383	0	383	17.6	17.4	383	383	Smirnov VN et al., 2018 Geochemistry International
582	DHS-N2	124.45	50.54	Eastern CAOB	Granite	132	0	150	7.2	7.0	683	693	Zhang YT et al., 2018 Lithos
583	DHS-N4	124.48	50.53	Eastern CAOB	Granite	130	0	150	5.3	5.1	794	807	Zhang YT et al., 2018 Lithos
584	DM06	110.49	41.60	North China craton	Granite	326	0	326	-11.0	-9.0	1889	1750	Zhou H et al., 2018 Lithos

585	13PY-4	91.56	40.60	Western CAOB	Granite	270	0	270	2.0	1.8	1081	1089	Xue SC et al., 2018 Lithos
586	LA-516	125.00	56.00	Eastern CAOB	Granite	2360	0	600	-25.9	-18.3	2360	2360	Larin AM et al., 2018 Petrology
587	EL13-10-3	111.51	43.32	Eastern CAOB	Granite	2397	0	600	-31.8	-36.6	3215	3515	Yuan L et al., 2018 Journal of Asian Earth Sciences
588	EL14-11-2	111.40	43.23	Eastern CAOB	Granite	239	0	239	6.6	6.1	790	821	Yuan L et al., 2018 Journal of Asian Earth Sciences
589	13GW436	117.50	45.50	Eastern CAOB	Granite	307	0	307	11.4	11.0	569	587	Tian DX et al., 2018 Journal of Asian Earth Sciences
590	13GW443	117.50	45.50	Eastern CAOB	Granite	314	0	314	12.0	11.5	537	563	Tian DX et al., 2018 Journal of Asian Earth Sciences
591	13GW450	117.50	45.50	Eastern CAOB	Granite	306	0	306	10.9	11.2	596	574	Tian DX et al., 2018 Journal of Asian Earth Sciences
592	13GW460	117.50	45.50	Eastern CAOB	Granite	310	0	310	11.0	11.7	590	549	Tian DX et al., 2018 Journal of Asian Earth Sciences
593	MY02/1B	117.10	42.42	North China craton	Granite	1721	0	600	-30.1	-30.2	3156	3161	Wang XX et al., 2018 Journal of Asian Earth Sciences
594	MY04/1B	117.10	42.42	North China craton	Granite	1659	0	600	-29.1	-29.3	3100	3111	Wang XX et al., 2018 Journal of Asian Earth Sciences
595	MY04/2B	117.10	42.42	North China craton	Granite	1653	0	600	-29.6	-29.4	3126	3113	Wang XX et al., 2018 Journal of Asian Earth Sciences
596	MY09/1B	117.10	42.42	North China craton	Granite	1748	0	600	-31.0	-31.1	3205	3210	Wang XX et al., 2018 Journal of Asian Earth Sciences
597	MY10/1B	117.10	42.42	North China craton	Granite	1730	0	600	-28.9	-29.0	3084	3091	Wang XX et al., 2018 Journal of Asian Earth Sciences
598	13GW365	120.82	47.76	Eastern CAOB	Granite	296	0	296	8.7	8.7	714	713	Ji Z et al., 2018 Lithos
599	HL-1	77.30	40.50	Western CAOB	Granite	270	0	270	1.9	1.9	1084	1085	Su YP et al., 2019 Geological Magazine
600	HL-16	77.30	40.50	Western CAOB	Granite	2077	0	600	-1.0	0.8	2077	2077	Su YP et al., 2019 Geological Magazine
601	HL-9	77.00	40.50	Western CAOB	Granite	270	0	270	-0.1	-0.2	1199	1207	Su YP et al., 2019 Geological Magazine
602	09AL127	104.98	39.43	North China craton	Granite	345	0	345	-11.9	-12.4	1932	1961	Dan W et al., 2016 Gondwana Research
603	09AL131	104.97	39.39	North China craton	Granite	345	0	345	-10.4	-10.1	1846	1832	Dan W et al., 2016 Gondwana Research
604	09AL191	106.13	40.45	North China craton	Granite	346	0	346	-16.5	-16.7	2195	2208	Dan W et al., 2016 Gondwana Research
605	09AL198	106.13	40.45	North China craton	Granite	344	0	344	-16.3	-16.5	2182	2191	Dan W et al., 2016 Gondwana Research
606	09AL200	106.12	40.46	North China craton	Granite	337	0	337	-21.0	-21.2	2441	2451	Dan W et al., 2016 Gondwana Research
607	western Tien Sha	60.51	42.03	Western CAOB	Granite	277	0	277	1.8	1.0	1139	1157	Dolgopolova A et al., 2017 Gondwana Research
608	11K86	86.35	41.63	Western CAOB	Granite	1930	0	600	-37.5	-37.2	3567	3552	Ge R et al., 2015 Geological Society of America Bulletin
609	11K88	86.41	41.63	Western CAOB	Granite	1934	0	600	-36.7	-36.7	3525	3524	Ge R et al., 2015 Geological Society of America Bulletin
610	10T03	86.20	41.82	Western CAOB	Granite	623	0	600	-3.8	-4.1	1671	1689	Ge R et al., 2015 Geological Society of America Bulletin
611	10T08	86.20	41.82	Western CAOB	Granite	630	0	600	-3.9	-4.3	1673	1698	Ge R et al., 2015 Geological Society of America Bulletin
612	10T10	86.20	41.82	Western CAOB	Granite	664	0	600	-2.7	-3.2	1604	1638	Ge R et al., 2015 Geological Society of America Bulletin
613	10T52	86.19	41.82	Western CAOB	Granite	641	0	600	-5.2	-4.2	1750	1691	Ge R et al., 2015 Geological Society of America Bulletin
614	T4	86.19	41.82	Western CAOB	Granite	669	0	600	-5.4	-4.5	1759	1709	Ge R et al., 2015 Geological Society of America Bulletin
615	11K105	86.71	41.51	Western CAOB	Granite	828	0	600	-30.1	-28.8	3152	3083	Ge R et al., 2015 Geological Society of America Bulletin
616	11K109	86.70	41.51	Western CAOB	Granite	831	0	600	-31.7	-30.6	3243	3183	Ge R et al., 2015 Geological Society of America Bulletin
617	11K55	86.18	41.80	Western CAOB	Granite	636	0	600	-4.4	-4.5	1705	1712	Ge R et al., 2015 Geological Society of America Bulletin
618	11K64	86.25	41.81	Western CAOB	Granite	656	0	600	-1.4	-1.5	1532	1536	Ge R et al., 2015 Geological Society of America Bulletin
619	11K94	86.04	41.84	Western CAOB	Granite	830	0	600	-27.3	-25.4	2994	2892	Ge R et al., 2015 Geological Society of America Bulletin
620	11K99	86.60	41.53	Western CAOB	Granite	834	0	600	-29.0	-26.0	3089	2924	Ge R et al., 2015 Geological Society of America Bulletin
621	12K84	86.17	41.79	Western CAOB	Granite	653	0	600	-4.6	-4.1	1716	1683	Ge R et al., 2015 Geological Society of America Bulletin
622	06-43	70.58	53.13	Western CAOB	Granite	1183	0	600	-18.2	-19.7	2475	2570	Glorie S et al., 2015 Gondwana Research
623	Zhan	70.64	53.13	Western CAOB	Granite	1170	0	600	-15.6	-17.0	2334	2419	Glorie S et al., 2015 Gondwana Research
624	L-1	74.60	47.38	Western CAOB	Granite	305	0	305	9.8	8.9	657	709	Li GM et al., 2016 Gondwana Research
625	T-17	114.22	47.01	Eastern CAOB	Granite	240	0	240	8.1	8.0	705	710	Li S et al., 2013 Earth-Science Reviews
626	B80628-8.2	95.90	41.25	Western CAOB	Granite	275	0	275	6.8	6.5	808	825	Li S et al., 2013 Earth-Science Reviews
627	DDQ09824-1.1	96.10	41.17	Western CAOB	Granite	279	0	279	4.0	4.0	973	982	Li S et al., 2013 Earth-Science Reviews
628	DDQ09824-2.1	96.16	41.21	Western CAOB	Granite	279	0	279	1.9	1.9	1093	1093	Li S et al., 2013 Earth-Science Reviews
629	DDQ09824-3	96.18	41.20	Western CAOB	Granite	350	0	350	1.3	2.2	1129	1071	Li S et al., 2013 Earth-Science Reviews
630	LJ205	132.10	45.49	Eastern CAOB	Granite	193	0	193	3.5	3.8	935	915	Liu K et al., 2017 Lithos
631	T556	88.61	41.01	Western CAOB	Granite	798	0	600	-18.5	-18.4	2502	2500	Long X et al., 2017 Precambrian Research
632	T558	88.53	41.06	Western CAOB	Granite	908	0	600	-22.6	-21.5	2733	2670	Long X et al., 2017 Precambrian Research
633	T559	88.58	40.97	Western CAOB	Granite	785	0	600	-13.1	-13.0	2198	2189	Long X et al., 2017 Precambrian Research
634	T579	88.55	41.27	Western CAOB	Granite	790	0	600	-20.8	-20.9	2630	2639	Long X et al., 2017 Precambrian Research
635	12BDG-10	119.14	49.99	Eastern CAOB	Granite	233	0	233	4.5	5.2	912	874	Mi K et al., 2017 Ore Geology Reviews
636	14BDG-10	119.13	49.98	Eastern CAOB	Granite	229	0	229	4.9	5.1	878	868	Mi K et al., 2017 Ore Geology Reviews
637	9HS16-2	75.17	46.98	Western CAOB	Granite	282	0	282	7.5	7.4	778	781	Shen P et al., 2017 Lithos
638	South Muya	115.22	56.00	Eastern CAOB	Granite	768	0	600	-6.3	-6.3	1812	1811	Iizuka Y et al., 2017 Precambrian Research
639	E101-1.1	112.98	43.72	Eastern CAOB	Granite	285	0	285	11.7	11.8	531	524	Tong Y et al., 2015 Journal of Asian Earth Sciences
640	E925-7	112.04	44.81	Eastern CAOB	Granite	276	0	276	15.3	14.9	317	336	Tong Y et al., 2015 Journal of Asian Earth Sciences
641	E930-1	112.35	43.67	Eastern CAOB	Granite	279	0	279	8.3	8.6	723	703	Tong Y et al., 2015 Journal of Asian Earth Sciences
642	HL-011	90.09	46.52	Western CAOB	Granite	326	0	326	9.7	9.9	677	666	Wu C et al., 2015 Ore Geology Reviews
643	KB05	82.24	43.19	Western CAOB	Granite	920	0	600	-6.2	-6.3	1808	1811	Xiong FH et al., 2019 Precambrian Research
644	WG-1	82.08	43.15	Western CAOB	Granite	889	0	600	-7.7	-7.7	1892	1891	Xiong FH et al., 2019 Precambrian Research
645	EL10-20	112.33	43.67	Eastern CAOB	Granite	280	0	280	10.2	10.1	616	618	Yuan LL et al., 2016 Lithos
646	EL10-21	112.33	43.67	Eastern CAOB	Granite	280	0	280	10.7	10.6	586	591	Yuan LL et al., 2016 Lithos
647	EL10-6-5	112.16	43.81	Eastern CAOB	Granite	275	0	275	9.8	9.9	632	627	Yuan LL et al., 2016 Lithos
648	EL10-9-1	112.16	43.87	Eastern CAOB	Granite	270	0	270	11.3	11.5	541	528	Yuan LL et al., 2016 Lithos
649	Halaqiao	90.43	46.46	Western CAOB	Granite	390	0	390	-2.4	0.7	1424	1245	Zhang C et al., 2019 Lithos
650	Kumaier	90.01	46.92	Western CAOB	Granite	331	0	331	0.0	2.8	1238	1084	Zhang C et al., 2019 Lithos
651	Qinghe-North	90.26	46.86	Western CAOB	Granite	345	0	345	-1.5	2.8	1337	1093	Zhang C et al., 2019 Lithos
652	Tawati	90.49	46.84	Western CAOB	Granite	334	0	334	0.9	3.7	1191	1029	Zhang C et al., 2019 Lithos
653	09Q17	88.03	41.08	Western CAOB	Granite	735	0	600	-8.6	-8.6	1941	1942	Zhang CL et al., 2012 Journal of Asian Earth Sciences
654	MON37	112.73	47.05	Eastern CAOB	Granite	263	0	263	5.2	6.0	881	835	Zhao P et al., 2017 Journal of Asian Earth Sciences
655	MON-65	111.82	46.65	Eastern CAOB	Granite	267	0	267	3.9	4.5	969	939	Zhao P et al., 2017 Journal of Asian Earth Sciences
656	MON-6O	112.14	46.87	Eastern CAOB	Granite	263	0	263	7.6	7.6	754	764	Zhao P et al., 2017 Journal of Asian Earth Sciences
657	12ts30-1	86.55	42.69	Western CAOB	Granite	337	0	337	-5.4	-5.4	1606	1615	Zhong LL et al., 2015 Journal of Asian Earth Sciences
658	SCG2	94.89	42.24	Eastern Junggar	granite	276	1.1	276	-10.7	-11.1	1803	1832	Xu Xingwang et al., 2015 Gondwana Research
659	HLG-01	88.88	47.87	Chinese Altai	granite	400	2.9	400	6.6	7.0	899	886	Zhang X et al., 2016 Journal of Asian Earth Sciences
660	HLG-02	88.86	47.83	Chinese Altai	granite	397	2.4	397	4.4	4.4	1055	1042	Zhang X et al., 2016 Journal of Asian Earth Sciences
661	AG-1	84.76	45.67	Western Junggar	granite	313	2.1	313	10.7	10.8	611	604	Li Di et al., 2015 Geoscience Frontiers(ICWEA)
662	BS07-329-142	99.43	41.03	Beishan	granite	285	0	285	-0.9	-1.2	1256	1272	Zhang W et al., 2012 Journal of Asian Earth Sciences
663	5102	116.42	45.23	Uliastai active continental margin	Granite	276	0	276	11.5	11.8	535	520	Cheng et al., 2014 Journal of Asian Earth Sciences
664	G0217-1	121.38	46.51	Hegenshan ophiolite-arc-accretion complex	Granite	230	0	230	10.8	11.2	537	516	Zhou Y et al., 2011 Acta Petrologica et Mineralogica(ICWEA)
665	G0217-2	121.38	46.51	Hegenshan ophiolite-arc-accretion complex	Granite	230	0	230	11.9	12.0	475	472	Zhou Y et al., 2011 Acta Petrologica et Mineralogica(ICWEA)
666	HLT-4-01	113.75	43.54	Baolidao arc-accretion complex	Granite	234	0	234	3.3	5.4	977	853	Chen B et al., 2009 Journal of Asian Earth Sciences
667	XW21-30	117.45	44.13	Baolidao arc-accretion complex	Granite	317	0	317	2.2	2.3	1105	1097	Liu JF et al., 2009 Dissertation(ICWEA)
668	NLG-1	118.07	43.82	Erdaojing accretion complex	Granite	148	0	150	2.6	2.4	951	962	Zeng QD et al., 2015 International Geology Review

669	LTS-1	118.58	43.92	Erdaojing accretion complex	Granite	144	0	150	6.0	6.0	752	755	Zeng QD et al., 2015 International Geology Review
670	TBD12	119.20	43.72	Erdaojing accretion complex	Granite	138	0	150	2.4	2.2	961	975	Zhou ZH et al., 2015 Journal of Asian Earth Sciences
671	HSG03	119.04	44.31	Erdaojing accretion complex	Granite	138	0	150	7.5	7.5	667	665	Shu QH et al., 2014 Journal of Asian Earth Sciences
672	14L6-4	113.55	42.39	Northern margin of the North China Craton	Granite	272	0	272	2.4	2.9	1055	1030	Liu CH et al., 2015 Lithos
673	JG21-11-22	119.23	42.63	Northern margin of the North China Craton	Granite	229	0	229	3.3	3.1	974	985	Liu JF et al., 2012 Journal of Asian Earth Sciences
674	HU09-20	111.87	41.09	North China Craton	Granite	275	0	275	-7.0	-6.8	1599	1588	Mo N et al., 2014 Acta Scientiarum Naturalium Universitatis Pekinensis(ICWEA)
675	DZZ3	116.29	41.14	North China Craton	Granite	140	0	150	-14.9	-14.9	1953	1952	Niu XL et al., 2011 Journal of Asian Earth Sciences
676	09026-1	118.79	40.43	North China Craton	Granite	222	0	222	-12.2	-12.3	1856	1861	Ye H et al., 2014 Earth Science Frontiers(ICWEA)
677	H120-1	119.03	40.70	North China Craton	Granite	233	0	233	-10.5	-7.5	1765	1596	Xiong L et al., 2017 Scientific Journal of Earth Science(ICWEA)
678	CF06-065-2	119.06	41.97	North China Craton	Granite	254	0	254	-10.6	-10.3	1786	1770	Wang YB et al., 2010 Acta Petrologica et Mineralogica(ICWEA)
679	YGS-15a	112.07	41.40	North China Craton	Granite	247	0	247	-11.0	-11.0	1807	1806	Gao S., 2016 Master's thesis(ICWEA)
680	YGS-22	112.12	41.47	North China Craton	Granite	243	0	243	-20.3	-21.0	2329	2372	Gao S., 2016 Master's thesis(ICWEA)
681	JN09108	118.93	40.47	North China Craton	Granite	174	0	174	-13.2	-13.3	1876	1876	Fan WB et al., 2017 Lithos
682	15YL12-4	127.35	45.65	LXZR	Granite	184	1	184	8.6	8.6	629	628	Zhu et al., 2017 Lithosphere
683	15YL28-1	129.53	47.35	LXZR	Granite	186	1	186	5.1	5.0	831	840	Zhu et al., 2017 Lithosphere
684	DYQ19	118.21	46.03	Uliastai active continental margin	Granite	157	0	157	7.4	7.4	679	679	Wang et al., 2016 Ore Geology Reviews
685	G0215-4	121.28	46.60	Hegenshan ophiolite-arc-accretion complex	Granite	125	0	150	9.7	9.9	538	527	Zhou Y et al., 2011 Acta Petrologica et Mineralogica(ICWEA)
686	HLT-2	113.51	43.55	Baolidao arc-accretion complex	Granite	234	0	234	4.7	6.0	893	822	Chen B et al., 2009 Journal of Asian Earth Sciences
687	WL95	117.39	44.06	Baolidao arc-accretion complex	Granite	321	0	321	6.8	6.7	841	847	Wang XY et al., 2013 Geoscience(ICWEA)
688	DL-14	117.98	44.21	Baolidao arc-accretion complex	Granite	293	0	293	4.5	4.4	950	961	Zhou ZH et al., 2014 Acta Petrologica Sinica(ICWEA)
689	ELMT-04	117.94	43.68	Erdaojing accretion complex	Granite	137	0	150	3.1	3.3	921	910	Pei QM et al., 2017 Ore Geology Reviews
690	14NX3-2	112.05	42.06	Northern margin of the North China Craton	Granite	270	0	270	-4.6	-5.1	1458	1485	Wang JX et al., 2016 Lithos
691	HY09-005	114.24	42.23	Northern margin of the North China Craton	Granite	268	0	268	2.0	1.8	1077	1086	Hao BW et al., 2011 Dissertation(ICWEA)
692	YSH01-1	119.12	42.58	Northern margin of the North China Craton	Granite	229	0	229	3.0	3.3	989	973	Liu JF et al., 2012 Journal of Asian Earth Sciences
693	DQ08-68	111.89	40.97	North China Craton	Granite	132	0	150	-13.6	-15.2	1878	1967	Guo L et al., 2012 International Geology Review
694	Hg33	115.09	41.17	North China Craton	Granite	299	0	299	-10.8	-9.3	1831	1749	Wang F et al., 2009 Acta Petrologica Sinica(ICWEA)
695	D60	115.47	40.87	North China Craton	Granite	131	0	150	-21.8	-21.9	2343	2352	Li CJ., Bao ZW., 2012 Geochemistry
696	DP-13	115.30	40.75	North China Craton	Granite	143	0	150	-14.4	-14.2	1924	1910	Cisse et al., 2016 Ore Geology Reviews
697	WLC-04	112.21	41.43	North China Craton	Granite	244	0	244	-13.6	-14.1	1951	1979	Gao S et al., 2016 Master's thesis(ICWEA)
698	YGS-19	112.12	41.47	North China Craton	Granite	242	0	242	-21.0	-20.9	2368	2367	Gao S et al., 2016 Master's thesis(ICWEA)
699	HST-11	118.14	44.06	Erdaojing accretion complex	Granite	150	0	150	7.5	7.3	666	676	Zeng QD et al., 2015 International Geology Review
700	MHS	118.99	42.30	North China Craton	Granite	387	0	387	-9.8	-9.5	1843	1826	Shi et al., 2010 Gondwana Research
701	14HS-5	118.57	43.88	Erdaojing accretion complex	Granite porphyry	131	1	150	10.0	9.4	522	556	Li S et al., 2017 Tectonics
702	14WL-4	118.68	43.73	Erdaojing accretion complex	Granite porphyry	129	1	150	6.9	6.1	703	746	Li S et al., 2017 Tectonics
703	TKSG14-6	81.87	43.39	0	Granite porphyry	251	6	251	5.4	4.7	868	910	Huang H et al., 2020 Earth-Science Reviews
704	YNB14-05	82.07	44.07	0	Granite porphyry	271	3	271	5.6	5.4	875	882	Huang H et al., 2020 Earth-Science Reviews
705	06XJ-13	81.43	44.75	0	Granite porphyry	289	3	289	5.2	5.0	908	924	Tang et al., 2010 Lithos
706	NLK14-1	82.47	43.73	0	Granite porphyry	263	3	263	10.1	10.2	604	601	Huang H et al., 2020 Earth-Science Reviews
707	06XJ120	84.83	45.71	Western Junggar	granite porphyry	304	3	304	14.7	14.8	373	366	Tang et al.,2012a Lithos
708	M114-2	117.30	49.42	0	Granite porphyry	179	1	179	6.5	6.2	746	765	Wang TH et al., 2014 Global Geology(ICWEA)
709	M099-4	116.12	48.52	0	Granite porphyry	137	4	150	4.4	4.4	847	844	Wang TH et al., 2014 Global Geology(ICWEA)
710	1697-1	120.76	50.79	0	Granite porphyry	162	2	162	7.6	7.6	671	670	Gou J et al., 2019 International Geology Review
711	D5036	126.72	50.50	0	Granite porphyry	118	2	150	6.3	6.3	735	737	Zhu HL et al., 2014 Global Geology(ICWEA)
712	12GW022	131.58	45.23	0	Granite porphyry	258	2	258	3.2	3.2	1001	1001	Yang H et al., 2015 Gondwana Research
713	ZK12-50-1	119.12	44.51	0	Granite porphyry	131	1	150	6.3	6.3	736	738	Wei W et al., 2020 Geological Magazine
714	n.g.	119.35	44.45	0	Granite porphyry	151	1	151	7.9	7.8	645	648	Zeng QD et al, 2015 Journal of Asian Earth Sciences
715	GW04209	120.06	47.13	0	Granite porphyry	129	1	150	8.1	8.4	633	617	Dong Y et al., 2019 Acta Geologica Sinica(English Edition)
716	HK-61-1	133.31	46.68	0	Granite porphyry	119	3	150	6.0	6.1	752	746	HAO YJ et al., 2020 Acta Petrologica Sinica(ICWEA)
717	B23	119.14	49.99	0	Granite porphyry	232	1	232	4.7	4.3	896	915	Kang YJ et al., 2018 Ore Geology Reviews
718	D3127	120.05	44.88	0	Granite porphyry	137	1	150	8.3	8.1	618	630	Liu HN et al., 2020 Minerals
719	HD–273	123.93	51.17	0	Granite porphyry	149	5	150	4.5	5.2	842	800	Liu J et al., 2014 Journal of Asian Earth Sciences
720	HNS09823-2.2	95.53	41.20	Western CAOB	Granite Porphyry	217	0	217	1.6	1.7	1059	1054	Li S et al., 2012 Lithos
721	BYH-10	84.94	46.56	Western Junggar	granite porphyry	313	0	313	10.0	10.0	652	652	Zhang X et al., 2014 Gondwana Research
722	BB5138	120.60	45.58	Hegenshan ophiolite-arc-accretion complex	Granite porphyry	125	0	150	7.8	8.1	647	634	Wu et al., 2014 Global Geology
723	HG-3-5	117.44	43.63	Erdaojing accretion complex	Granite porphyry	137	0	150	5.6	5.6	777	775	Zhou ZH et al., 2012 Geological Science and Technology Information(ICWEA)
724	GZ10-28	120.42	44.83	Erdaojing accretion complex	Granite porphyry	125	0	150	6.1	5.8	748	765	Yang QD et al., 2014 Acta Petrologica Sinica(ICWEA)
725	J3	119.10	42.42	Northern margin of the North China Craton	Granite porphyry	156	0	156	-3.8	-3.3	1324	1291	Wu HY et al., 2014 Ore Geology Reviews
726	J4	119.10	42.42	Northern margin of the North China Craton	Granite porphyry	168	0	168	0.8	0.9	1066	1059	Wu HY et al., 2014 Ore Geology Reviews
727	B107-JBG	118.67	40.73	North China Craton	Granite porphyry	170	0	170	-10.5	-11.0	1717	1748	Li M et al., 2016 Geological bulletin
728	STWH1077	120.98	40.79	North China Craton	Granite porphyry	130	0	150	4.9	5.5	818	785	Cui FH et al., 2015 Doctoral thesis(ICWEA)
729	P24-4	129.99	47.38	LXZR	Granite porphyry	210	2	210	-2.4	-1.7	1285	1245	Wei et al., 2012 Earth Science Journal of China University of Geosciences(ICWEA)
730	LHS-01	121.95	45.69	Hegenshan ophiolite-arc-accretion complex	Granite porphyry	237	0	237	9.1	9.2	644	636	Zhou ZH et al., 2012 China Geology
731	LHS-02	121.95	45.69	Hegenshan ophiolite-arc-accretion complex	Granite porphyry	238	0	238	9.2	9.3	638	630	Zhou ZH et al., 2012 China Geology
732	EBD-1	118.09	43.95	Erdaojing accretion complex	Granite porphyry	146	0	150	7.2	7.5	684	665	Zeng QD et al., 2015 International Geology Review
733	HST-1	118.20	44.09	Erdaojing accretion complex	Granite porphyry	148	0	150	8.1	8.1	634	630	Zeng QD et al., 2015 International Geology Review
734	B09-018-01	113.52	42.34	Northern margin of the North China Craton	Granite porphyry	254	0	254	7.9	7.9	727	724	Hao BW et al., 2011 Doctoral thesis(ICWEA)
735	CS2-3	113.88	40.82	North China Craton	Granite porphyry	150	0	150	-15.9	-15.5	2008	1990	Wu HY et al., 2016 Journal of Asian Earth Sciences
736	CS8-4	113.88	40.82	North China Craton	Granite porphyry	149	0	150	-15.2	-15.0	1968	1957	Wu HY et al., 2016 Journal of Asian Earth Sciences
737	DHD11-1	116.31	41.25	North China Craton	Granite porphyry	129	0	150	-11.4	-11.3	1754	1745	Liang QL et al., 2013 Geological Review
738	DHD11-4	116.20	41.23	North China Craton	Granite porphyry	132	0	150	-15.3	-15.5	1973	1986	Liang QL et al., 2013 Geological Review
739	STWH2008	120.58	40.62	North China Craton	Granite porphyry	155	0	155	-20.5	-20.3	2274	2263	Cui FH et al., 2015 Doctoral thesis(ICWEA)
740	B115-JBG	118.67	40.73	North China Craton	Granite porphyry	170	0	170	-11.3	-11.7	1762	1784	Li M et al., 2016 Geological Bulletin of China
741	H	95.30	44.20	sangdewulan	granite porphyry	432	0	432	13.0	13.2	575	564	Zhang et al., 2010
742	FX06-21	121.79	42.26	North China Craton	Granites	256	0	256	-16.6	-16.2	2134	2109	Zhang XH et al., 2012 Lithos
743	T0832-GN	86.00	29.12	Himalayan orogenic belt	granites	472	3.8	472	-5.7	-5.5	1677	1668	Gao LE et al., 2018 Lithos
744	T0833	86.00	29.12	Himalayan orogenic belt	granites	479	7.5	479	-5.1	-5.1	1648	1651	Gao LE et al., 2018 Lithos
745	T0834-GN	86.00	29.12	Himalayan orogenic belt	granites	459	2.4	459	-5.7	-5.3	1670	1643	Gao LE et al., 2018 Lithos
746	T0834-LG	86.00	29.12	Himalayan orogenic belt	granites	466	3.9	466	-5.7	-5.8	1671	1677	Gao LE et al., 2018 Lithos
747	T0835-LG	86.00	29.12	Himalayan orogenic belt	granites	455	1.7	455	-5.8	-5.6	1667	1661	Gao LE et al., 2018 Lithos
748	T0839	86.00	29.12	Himalayan orogenic belt	granites	447	1	447	-5.8	-5.8	1665	1663	Gao LE et al., 2018 Lithos
749	T0898	87.90	28.80	Himalayan orogenic belt	granites	475	14	475	-9.4	-5.5	1889	1670	Gao LE et al., 2018 Lithos
750	T0526-LG	89.70	28.70	Himalayan orogenic belt	granites	498	7.8	498	-3.8	-3.0	1589	1546	Gao LE et al., 2018 Lithos
751	T0527-LG	89.70	28.70	Himalayan orogenic belt	granites	500	6.7	500	-2.6	-2.8	1524	1532	Gao LE et al., 2018 Lithos
752	T0812-A	85.85	28.45	Himalayan orogenic belt	granites	429	3.3	429	-8.3	-8.6	1795	1811	Gao LE et al., 2018 Lithos

753	T0812-B	85.85	28.45	Himalayan orogenic belt	granites	448	2.9	448	-8.5	-8.7	1818	1832	Gao LE et al., 2018 Lithos
754	T0814	85.85	28.45	Himalayan orogenic belt	granites	467	4.2	467	-8.8	-8.6	1851	1839	Gao LE et al., 2018 Lithos
755	ZC-10	85.85	28.45	Himalayan orogenic belt	granites	487	3.1	487	-6.3	-5.9	1725	1703	Gao LE et al., 2018 Lithos
756	T0252	94.60	29.30	Himalayan orogenic belt	granites	507	9.7	507	-4.9	-5.4	1658	1687	Gao LE et al., 2018 Lithos
757	ZBZL-16-N1	98.24	35.96	northwestern China	granites	236	0	236	-0.3	-0.4	1185	1188	Li HR et al., 2019 Canadian Journal of Earth Sciences
758	ZBZL-16-N3	98.28	35.98	northwestern China	granites	239	0	239	-1.3	-1.0	1242	1229	Li HR et al., 2019 Canadian Journal of Earth Sciences
759	TY-Hf	94.72	41.79	NW China	granites	280	2	280	4.7	4.8	932	928	Tang DM et al., 2011 Lithos
760	XGY01	115.17	40.33	the North China Craton	granites	2493	6	600	-39.5	-39.6	3674	3680	Zhang HF et al., 2011 Lithos
761	NK	84.78	45.68	CAOB	Granites	328	0	328	9.8	10.4	671	632	Jia Lu et al., 2020 Minerals
762	HS	85.15	45.95	CAOB	Granites	310	0	310	11.3	11.3	570	575	Jia Lu et al., 2020 Minerals
763	MDLH	88.52	48.28	CAOB	granites	327	0	327	-1.9	0.6	1347	1202	LUO Q et al., 2019 Geological Magazine
764	HT	88.68	48.17	CAOB	granites	327	0	327	-4.5	0.2	1494	1226	LUO Q et al., 2019 Geological Magazine
765	TEG	87.80	48.58	CAOB	granites	327	0	327	0.0	2.5	1240	1094	LUO Q et al., 2019 Geological Magazine
766	ZGST	87.55	48.57	CAOB	granites	327	0	327	2.6	2.6	1091	1087	LUO Q et al., 2019 Geological Magazine
767	ZPJLK	87.57	48.45	CAOB	granites	327	0	327	-1.8	0.5	1341	1208	LUO Q et al., 2019 Geological Magazine
768	11GW022	131.97	45.32	0	Granitic	258	2	258	1.5	2.0	1098	1071	Yang H et al., 2015 Gondwana Research
769	DL12-7-1.3	95.57	37.40	the North Qaidam UHP	granitic	444	3	444	-6.9	-5.0	1722	1616	Yu SY et al., 2015 Lithos
770	DL12-7-1.2	95.57	37.40	the North Qaidam UHP	granitic	433	2	433	-5.5	-4.4	1635	1575	Yu SY et al., 2015 Lithos
771	QES-7	99.50	32.50	eastern Tibetan Plateau	granitic	103	0	150	1.1	0.9	1035	1048	Tao W et al., 2014 Science China Earth Sciences
772	QES-11	99.50	32.50	eastern Tibetan Plateau	granitic	106	0	150	1.4	1.5	1021	1016	Tao W et al., 2014 Science China Earth Sciences
773	TH1	91.77	44.61	Eastern Junggar	Granitic diorite	372	0	372	-36.0	-36.1	3309	3656	Xu XW et al., 2013 Gondwana Research
774	10HSS01	97.11	42.39	Beishan	granitic gneiss	300	0	300	0.1	0.5	1213	1190	Song D et al., 2013 Precambrian Research
775	10SYQ03	97.09	42.04	Beishan	granitic gneiss	471	0	471	-5.4	-5.4	1658	1660	Song D et al., 2013 Precambrian Research
776	TL010-01	120.59	40.37	North China Craton	Granitic gneiss	232	0	232	-19.8	-19.5	2295	2275	Li J et al., 2009 Master's thesis(ICWEA)
777	06XJ-34	81.60	44.21	0	Granitic porphyry	349	2	349	3.6	3.5	1048	1053	Tang et al., 2013 Journal of Asian Earth Sciences
778	DHS-2-01	124.45	50.54	0	Granitoid	133	3	150	7.2	7.0	683	693	Zhang YT et al., 2018 Lithos
779	DHS-4-01	124.48	50.53	0	Granitoid	131	3	150	5.3	5.1	794	807	Zhang YT et al., 2018 Lithos
780	KeKetuohai-East	90.08	47.21	Chinese	granitoid	446	3.6	446	5.1	5.3	1035	1030	Zhang C et al., 2016 Gondwana Research
781	KeKetuohai-North	89.71	47.32	Chinese	granitoid	404	2.7	404	4.2	3.7	1058	1085	Zhang C et al., 2016 Gondwana Research
782	Kayierte	89.63	47.20	Chinese	granitoid	407	2.5	407	2.8	3.0	1136	1128	Zhang C et al., 2016 Gondwana Research
783	Daqiao-East	89.68	47.38	Chinese	granitoid	340	3.3	340	-4.0	0.6	1478	1215	Zhang C et al., 2016 Gondwana Research
784	Jaertasisayi	89.67	47.48	Chinese	granitoid	340	3.3	340	2.9	3.1	1145	1136	Zhang C et al., 2016 Gondwana Research
785	Keketale	89.83	46.93	Chinese	granitoid	425	2.5	425	2.7	3.0	1135	1137	Zhang C et al., 2016 Gondwana Research
786	Areletuobie-West	90.15	47.02	Chinese	granitoid	392	2.2	392	-0.7	4.1	1393	1128	Zhang C et al., 2016 Gondwana Research
787	Dongbute-West	90.60	46.60	Chinese	granitoid	486	4.5	486	-0.6	-1.1	1412	1446	Zhang C et al., 2016 Gondwana Research
788	Akemeiketiepu	90.60	46.50	Chinese	granitoid	507	5.5	507	-1.5	-0.7	1450	1404	Zhang C et al., 2016 Gondwana Research
789	QJ12-2B	95.92	37.38	NW China	granitoid	2360	0	600	-38.3	-38.4	3610	3616	Chuan H et al., 2018 Gondwana Research
790	QJ12-6	95.91	37.36	NW China	granitoid	2360	0	600	-37.2	-37.4	3546	3563	Chuan H et al., 2018 Gondwana Research
791	QJ12-2A	95.92	37.39	NW China	granitoid	2340	0	600	-38.0	-38.0	3596	3595	Chuan H et al., 2018 Gondwana Research
792	QJ12-3A	95.91	37.35	NW China	granitoid	2340	0	600	-38.9	-38.9	3642	3642	Chuan H et al., 2018 Gondwana Research
793	10H262	91.82	39.10	NW China	granitoid	494	5.5	494	-1.6	-2.4	1463	1509	Meng LT et al., 2017 Lithos
794	CL131	90.12	39.05	Northwest China	granitoids	499	0	499	4.4	4.5	1124	1118	Zheng K et al., 2019 Lithos
795	CL187	90.21	39.07	Northwest China	granitoids	496	0	496	0.2	0.2	1362	1362	Zheng K et al., 2019 Lithos
796	DT-18-1	78.00	38.00	northwest China	granitoids	474	0	474	-1.3	-1.5	1426	1439	Liao SY et al., 2010
797	HRE-6	98.69	35.78	Northwestern China	granitoids	448	0	448	0.2	3.0	1320	1163	Li JY et al., 2020 International Journal of Earth Sciences
798	HRE-62	98.74	35.74	Northwestern China	granitoids	444	0	444	-3.9	-3.9	1554	1556	Li JY et al., 2020 International Journal of Earth Sciences
799	A02	102.17	38.42	NW China	granitoids	426	3	426	-0.1	-0.1	1322	1323	Zhang LQ et al., 2017 Lithos
800	2015H49	102.22	38.37	NW China	granitoids	443	4	443	-6.3	-6.3	1689	1692	Zhang LQ et al., 2017 Lithos
801	D18-B-N	124.17	40.12	the North China Craton	granitoids	129	0	150	-19.1	-19.1	2190	2190	Li JY et al., 2021 Mineralogy and Petrology
802	D18-E-N	124.13	40.30	the North China Craton	granitoids	126	0	150	-19.1	-19.1	2190	2190	Li JY et al., 2021 Mineralogy and Petrology
803	D18-M-N	124.38	49.25	the North China Craton	granitoids	127	0	150	-14.1	-14.2	1908	1911	Li JY et al., 2021 Mineralogy and Petrology
804	D18-N-N	124.33	40.17	the North China Craton	granitoids	126	0	150	-14.9	-14.6	1954	1937	Li JY et al., 2021 Mineralogy and Petrology
805	D18-P-N	124.38	40.28	the North China Craton	granitoids	123	0	150	-13.0	-13.0	1845	1847	Li JY et al., 2021 Mineralogy and Petrology
806	D18-Q-N	124.35	40.27	the North China Craton	granitoids	124	0	150	-15.2	-15.3	1968	1974	Li JY et al., 2021 Mineralogy and Petrology
807	DL09	100.00	36.50	northern Tibetan Plateau	granitoids	250	0	250	2.6	3.2	1018	994	Hui H et al., 2014 Chemical Geology
808	HLHB1	118.77	44.53	the Haliheiba	granitoids	139	0.7	150	7.7	8.0	653	638	Wei W et al., 2020 Geochemistry
809	HLHB3	118.73	44.46	the Haliheiba	granitoids	138	1	150	8.2	8.1	625	634	Wei W et al., 2020 Geochemistry
810	11HNA1-1	129.42	44.39	NE China	granitoids	256	1	256	-1.4	-1.5	1264	1271	Yu JJ et al., 2013 Gondwana Research
811	11HNA4-1	128.72	44.11	NE China	granitoids	255	2	255	6.7	6.2	795	827	Yu JJ et al., 2013 Gondwana Research
812	11HNA11-1	128.55	43.82	NE China	granitoids	252	2	252	7.7	7.5	738	746	Yu JJ et al., 2013 Gondwana Research
813	11K59	86.25	41.81	0	Granodiorite	418	4	418	-4.9	-4.3	1589	1555	Ge et al., 2014 Tectonics
814	11K71	86.24	41.81	0	Granodiorite	421	4	421	-6.1	-4.6	1662	1572	Ge RF et al., 2014 Tectonics
815	11K75	86.24	41.81	0	Granodiorite	420	4	420	-11.9	-4.4	1987	1563	Ge RF et al., 2014 Tectonics
816	11K78	86.23	41.80	0	Granodiorite	420	4	420	-17.2	-11.4	2287	1963	Ge RF et al., 2014 Tectonics
817	11K81	86.23	41.80	0	Granodiorite	418	4	418	-4.6	-4.7	1574	1579	Ge RF et al., 2014 Tectonics
818	11K63	86.25	41.81	0	Granodiorite	420	4	420	-10.9	-3.8	1927	1530	Ge et al. 2014 Tectonics
819	09T11	86.24	41.81	Tianshan	Granodiorite	421	2	421	-18.0	-13.0	2333	2051	Ge RF et al., 2012 Lithos
820	10T19	86.24	41.81	Tianshan	Granodiorite	422	3	422	-3.7	-4.0	1523	1541	Ge RF et al., 2012 Lithos
821	10T25	86.24	41.81	Tianshan	Granodiorite	420	3	420	-2.4	-2.9	1446	1479	Ge RF et al., 2012 Lithos
822	784	86.31	42.74	Tianshan	Granodiorite	451	3	451	-15.4	-15.2	2212	2204	Ma XX et al.,2014 Gondwana Research
823	798	87.18	42.82	Tianshan	Granodiorite	352	3	352	3.5	3.5	1058	1057	Ma XX et al.,2014 Gondwana Research
824	BLT IV -4	86.12	42.91	0	Granodiorite	306	5	306	3.8	4.4	1005	970	Huang H et al., 2015 Acta Geologica Sinica(ICWEA)
825	BLT02	86.34	42.80	0	Granodiorite	473	6	473	5.8	6.3	1020	992	Shi et al., 2014 International Geology Review
826	TS1343-1	86.90	42.91	0	Granodiorite	354	2	354	3.9	4.0	1037	1027	Yin et al., 2016 Gondwana Research
827	TS1344-1	86.91	42.87	0	Granodiorite	354	2	354	5.3	5.4	953	948	Yin et al., 2016 Gondwana Research
828	TKSD14-1	81.90	42.90	0	Granodiorite	341	2	341	5.1	5.0	955	964	Huang H et al., 2020 Earth-Science Reviews
829	XYF14-6	83.25	43.36	0	Granodiorite	311	3	311	2.8	5.3	1063	923	Huang H et al., 2020 Earth-Science Reviews
830	W8028	80.84	42.45	Western CAOB	Granodiorite	326	3	326	7.1	7.7	831	796	Gou LL et al., 2012 Lithos
831	W8027	80.78	42.51	Western CAOB	Granodiorite	293	3	293	-2.6	-2.7	1359	1365	Gou et al., 2012 Lithos
832	NLKC14-10	83.03	43.91	0	Granodiorite	371	4	371	4.3	4.3	1027	1028	Huang H et al., 2020 Earth-Science Reviews
833	JHA14-04	82.43	44.19	0	Granodiorite	375	4	375	4.6	4.9	1010	997	Huang H et al., 2020 Earth-Science Reviews
834	ZS1-1	81.06	42.75	Western CAOB	Granodiorite	485	15	485	0.9	0.8	1311	1318	Xu XY et al., 2010 Acta Petrologica et Mineralogica(ICWEA)
835	TL2-7	83.83	43.50	0	Granodiorite	349	7	349	5.8	6.7	920	870	Xu XY et al., 2010 Acta Petrologica et Mineralogica(ICWEA)
836	XXY1-1	82.73	43.03	Western CAOB	Granodiorite	430	7	430	4.0	5.5	1089	1006	Xu XY et al., 2010 Acta Petrologica et Mineralogica(ICWEA)

837	BYH1	84.94	46.56	Western Junggar	granodiorite	313	2.3	313	9.8	10.0	665	652	Zhang X et al.,2013 Gondwana Research
838	WJ1132	85.62	46.66	0	Granodiorite	420	2	420	14.1	14.2	499	496	Yin et al., 2017 Gondwana Research
839	06XJ148	84.54	45.47	0	Granodiorite	312	3.6	312	13.9	13.7	427	434	Tang et al., 2019 Earth and Planetary Science Letters
840	08AET99	89.47	46.21	0	Granodiorite	318	5.8	318	10.0	10.1	657	651	Tang et al., 2019 Earth and Planetary Science Letters
841	10LX-63	117.55	43.85	0	Granodiorite	245	3	245	11.6	10.3	502	439	Zhao L et al., 2019 Journal of Asian Earth Sciences
842	14XF-5	118.92	43.78	Erdaojing accretion complex	Granodiorite	141	1	150	7.2	6.4	681	730	Li S et al., 2017a Tectonics, 2017b Geological Science of America Bulletin
843	13HH13-1	125.70	50.30	Eastern CAOB	Granodiorite	237	1	237	11.2	11.0	523	532	Li Y et al., 2018 Lithos
844	13HH8-1	125.79	50.25	Eastern CAOB	Granodiorite	223	1	223	14.3	14.2	332	337	Li Y et al., 2018 Lithos
845	15DX15-1	121.29	46.58	Eastern CAOB	Granodiorite	177	1	177	11.7	11.7	446	442	Li Y et al., 2018 Lithos
846	13XA33-1	123.44	48.26	Eastern CAOB	Granodiorite	175	1	175	10.2	10.1	528	536	Li Y et al., 2018 Lithos
847	16ER43-1	125.65	50.38	Eastern CAOB	Granodiorite	174	1	174	8.4	8.3	636	639	Li Y et al., 2018 Lithos
848	M543-5	116.14	48.64	0	Granodiorite	251	3	251	8.1	8.4	711	695	[illegible] al., 2013 Journal of Asian Earth Sciences; Tang J et al., 2018 Science China Earth Sciences
849	HL-5-1	129.22	42.34	0	Granodiorite	235	1	235	-11.2	-10.9	1809	1792	Guan QB et al., 2020 Gondwana Research
850	HL-3-1	129.45	42.55	0	Granodiorite	176	1	176	7.9	7.8	662	672	Guan QB et al., 2020 Gondwana Research
851	15JJW7-1	126.52	43.09	0	Granodiorite	220	3	220	7.2	7.0	741	754	Wang F et al., 2019 Journal of Geophysical Research: Solid Earth
852	13ER31-1	120.93	51.86	0	Granodiorite	246	1	246	0.9	1.5	1122	1089	Tang J et al., 2016 Gondwana Research
853	13ER46-1	119.14	49.98	0	Granodiorite	228	2	228	5.2	5.3	861	854	Tang J et al., 2016 Gondwana Research
854	13ER46-4	119.14	49.98	0	Granodiorite	228	2	228	4.6	4.4	897	908	Tang J et al., 2016 Gondwana Research
855	12ER27-1	121.76	51.58	0	Granodiorite	197	2	197	4.1	4.0	903	908	Tang J et al., 2016 Gondwana Research
856	13ER21-1	120.97	51.31	Eastern CAOB	Granodiorite	195	1	195	2.3	2.3	1003	1000	Tang J et al., 2016 Gondwana Research
857	13GF-05	128.04	42.87	0	Granodiorite	168	3	168	3.3	4.1	922	876	Guo F et al., 2019 Chemical Geology
858	13GF-50	129.84	42.34	0	Granodiorite	245	6	245	-8.8	-10.7	1680	1786	Guo F et al., 2019 Chemical Geology
859	13GF-36	128.99	43.04	0	Granodiorite	182	2	182	8.6	8.7	627	624	Guo F et al., 2019 Chemical Geology
860	13GF-124	129.51	43.13	0	Granodiorite	182	2	182	8.8	8.9	620	610	Guo F et al., 2019 Chemical Geology
861	13GF-107	129.65	43.40	0	Granodiorite	187	1	187	7.8	7.9	678	671	Guo F et al., 2019 Chemical Geology
862	13GF-109	129.94	43.60	0	Granodiorite	189	0	189	8.4	8.6	644	635	Guo F et al., 2019 Chemical Geology
863	13GF-126	129.65	43.04	0	Granodiorite	189	1	189	9.6	9.5	577	581	Guo F et al., 2019 Chemical Geology
864	13GF-113	130.22	43.80	0	Granodiorite	195	3	195	7.6	7.5	698	704	Guo F et al., 2019 Chemical Geology
865	13GF-115	130.22	43.80	0	Granodiorite	195	0	195	8.2	8.2	660	662	Guo F et al., 2019 Chemical Geology
866	11ER15-1	119.68	50.98	0	Granodiorite	241	0	241	-1.0	-0.9	1227	1224	Tang J et al., 2014 Lithos
867	11ER16-1	119.68	50.98	0	Granodiorite	246	1	246	0.9	0.9	1125	1123	Tang J et al., 2014 Lithos
868	14JEW2-1	129.15	43.18	0	Granodiorite	183	1	183	8.1	8.0	657	663	Wang F et al., 2017 Journal of Asian Earth Sciences
869	GW05067	126.14	52.53	0	Granodiorite	190	1	190	-0.4	-0.4	1155	1156	Sui ZM et al., 2007 Acta Petrologica Sinica(ICWEA)
870	GW05112	125.79	51.88	0	Granodiorite	181	1	181	1.5	-0.3	1034	1141	Sui ZM et al., 2007 ActaPetrologica Sinica(ICWEA)
871	D6001	126.75	50.50	0	Granodiorite	113	1	150	6.5	6.7	722	710	Zhu HL et al., 2013 Global Geology(ICWEA)
872	GW07005	126.90	49.05	0	Granodiorite	319	3	319	7.5	7.2	800	816	Zhang YL et al., 2010 ActaPetrologicaSinica(ICWEA)
873	H15-15-1	128.85	46.96	0	granodiorite	494	5	494	1.3	1.8	1296	1269	Ge MH et al., 2021 Lithos
874	11LK31-3	125.02	43.39	0	Granodiorite	170	1	170	5.6	5.5	793	797	Zhang HH et al., 2016 Lithos
875	LK14-1	124.80	42.33	0	Granodiorite	169	2	169	-16.3	-16.8	2045	2079	Zhang HH et al., 2016 Lithos
876	LK05-1	124.32	42.71	0	Granodiorite	163	1	163	-7.5	-7.5	1538	1538	Zhang HH et al., 2016 Lithos
877	10GW247	132.23	47.24	0	Granodiorite	54	1	150	5.6	5.5	779	783	Wang ZH et al., 2016 Acta Petrologica Sinica(ICWEA)
878	11GW048	132.25	47.23	0	Granodiorite	53	1	150	5.6	5.5	775	781	Wang ZH et al., 2016 Acta Petrologica Sinica(ICWEA)
879	FAP-N2-Q1	127.32	44.41	0	Granodiorite	173	1	173	7.8	7.9	669	664	ZHANG LS et al., 2021 Acta Geologica Sinica
880	1666-1	120.99	52.31	0	Granodiorite	210	2	210	-0.9	-0.8	1197	1193	[illegible]Y et al., 2013 International Geology Review; Gou J et al., 2017 International Geology Review
881	1605-1	122.60	52.82	0	Granodiorite	187	3	187	-2.1	-2.5	1248	1271	[illegible]Y et al., 2013 International Geology Review; Gou J et al., 2017 International Geology Review
882	14HYC8-1	129.28	46.96	0	Granodiorite	194	1	194	1.8	1.8	1029	1032	Long XY et al., 2020 Gondwana Research
883	H15-11	128.35	46.94	Eastern CAOB	Granodiorite	178	2	178	3.2	3.4	938	927	Ge MH et al., 2018 Lithos
884	H15-37-1	129.79	47.41	0	Granodiorite	251	1	251	-0.3	-0.4	1196	1201	Ge MH et al., 2018 Lithos
885	13GW276	123.18	48.60	0	Granodiorite	177	1	177	8.8	8.8	614	611	Dong Y et al., 2016 JAES
886	Lzk501-290.5	125.89	50.27	Eastern CAOB	Granodiorite	225	2	225	9.7	9.8	601	592	Zhao C et al., 2019 Lithos
887	MT3-1	125.85	50.27	Eastern CAOB	Granodiorite	240	7	240	10.0	9.9	594	602	Zhao C et al., 2019 Lithos
888	YJD03	125.71	50.27	Eastern CAOB	Granodiorite	242	2	242	9.1	9.2	648	643	Zhao C et al., 2019 Lithos
889	06XJ-017	80.98	44.71	Western CAOB	Granodiorite	366	3	366	0.8	2.0	1221	1152	Tang GJ et al., 2010 Lithos
890	WE14-1	121.00	49.00	Eastern CAOB	Granodiorite	331	0	331	13.7	13.7	454	451	Zhao Z et al., 2010 Acta Petrologica Sinica
891	HDB2-12	125.70	50.20	Eastern CAOB	Granodiorite	479	0	479	15.0	15.4	479	479	Wu G et al., 2015 Journal of Asian Earth Sciences
892	KG102	71.09	41.45	Western CAOB	Granodiorite	420	0	420	1.1	1.4	1250	1232	Alexeiev DV et al., 2016 Gondwana Research
893	KG28	75.55	41.00	Western CAOB	Granodiorite	436	0	436	-4.4	-4.6	1578	1585	Alexeiev DV et al., 2016 Gondwana Research
894	M33/06	97.15	43.27	Western CAOB	Granodiorite	300	0	300	7.9	8.0	760	758	Kröner A et al., 2017 Gondwana Research
895	14GW067	130.29	45.87	Eastern CAOB	Granodiorite	256	2	256	-5.5	-5.2	1500	1479	Yang H et al., 2017 International Journal of Earth Sciences
896	14GW083	130.00	45.70	Eastern CAOB	Granodiorite	261	0	261	-4.1	-4.4	1416	1430	Yang H et al., 2017 International Journal of Earth Sciences
897	14GW107	130.12	45.42	Eastern CAOB	Granodiorite	255	0	255	-5.6	-5.2	1507	1490	Yang H et al., 2017 International Journal of Earth Sciences
898	14GW366	130.61	45.51	Eastern CAOB	Granodiorite	266	0	266	-2.7	-2.2	1374	1333	Yang H et al., 2017 International Journal of Earth Sciences
899	09AL33	104.77	40.16	North China craton	Granodiorite	281	0	281	-11.1	-11.1	1836	1840	Dan W et al., 2014 Lithos
900	09AL37	104.71	40.17	North China craton	Granodiorite	283	0	283	-11.9	-11.3	1885	1851	Dan W et al., 2014 Lithos
901	08AET055	90.00	46.00	Western CAOB	Granodiorite	301	0	301	12.9	12.9	472	471	Tang GJ et al., 2017 Geochemistry Geophysics Geosystems
902	14HW1-1	129.91	47.25	Eastern CAOB	Granodiorite	841	0	600	-7.9	-8.4	1905	1928	Luan JP., 2017 Journal of Asian Earth Sciences
903	H15-68-1	127.84	45.32	Eastern CAOB	Granodiorite	171	0	171	7.9	7.8	661	665	Ge MH et al., 2017 Tectonophysics
904	H15-69-1	128.42	45.13	Eastern CAOB	Granodiorite	189	0	189	9.9	8.9	561	618	Ge MH et al., 2017 Tectonophysics
905	HY-N2	94.00	42.00	Western CAOB	Granodiorite	298	0	298	2.3	4.1	1087	994	Zhang WF et al., 2017 Lithos
906	14HC30	111.04	41.48	North China craton	Granodiorite	2646	0	600	-39.7	-39.5	3689	3679	Chen NHC et al., 2017 Precambrian Research
907	14SL01-1	111.93	41.61	North China craton	Granodiorite	2546	0	600	-40.2	-40.0	3718	3708	Chen NHC et al., 2017 Precambrian Research
908	SK76	125.50	50.50	Eastern CAOB	Granodiorite	177	0	177	8.0	7.8	659	669	Deng K et al., 2017 Lithos
909	CL13-010	92.00	42.00	Western CAOB	Granodiorite	307	0	307	15.8	15.8	310	311	Zhao LD et al., 2018 Journal of Asian Earth Sciences
910	BA11-118	96.80	41.70	Western CAOB	Granodiorite	434	0	434	7.2	7.2	907	912	Zheng RG et al., 2018 Journal of Asian Earth Sciences
911	14RF45-1	133.56	45.45	Eastern CAOB	Granodiorite	430	0	430	-1.7	-1.7	1437	1435	Xu T et al., 2018 Lithos
912	B12	113.00	45.00	Eastern CAOB	Granodiorite	300	0	300	9.0	9.8	698	651	Zhang XJ et al., 2018 International Journal of Earth Sciences
913	X10-44-1	97.04	42.19	Western CAOB	Granodiorite	330	0	330	7.6	7.7	802	800	Yuan Y et al., 2018 Lithos
914	H15-37	129.79	47.41	Eastern CAOB	Granodiorite	251	0	251	-0.3	-0.4	1196	1201	Ge MH et al., 2018 Lithos
915	RZ34	124.65	42.37	North China craton	Granodiorite	173	0	173	4.0	4.2	888	878	Liu J et al., 2018 Journal of Asian Earth Sciences
916	P1-8	113.00	44.00	Eastern CAOB	Granodiorite	303	0	303	12.1	11.7	519	545	Chai H et al., 2018 Lithos
917	P21-6	113.00	44.00	Eastern CAOB	Granodiorite	318	0	318	13.2	13.3	470	461	Chai H et al., 2018 Lithos
918	JD16-008	118.81	40.24	North China craton	Granodiorite	166	0	166	-11.5	-11.4	1770	1762	Jiang SH et al., 2018 Lithos
919	JD16-104	118.90	40.37	North China craton	Granodiorite	156	0	156	-23.4	-23.2	2461	2424	Jiang SH et al., 2018 Lithos
920	16GW114	129.57	44.67	Eastern CAOB	Granodiorite	263	0	263	-3.0	-2.7	1326	1310	Zhao D et al., 2018 Lithos

921	16GW126	129.38	44.64	Eastern CAOB	Granodiorite	218	0	218	1.9	2.0	1043	1040	Zhao D et al., 2018 Lithos
922	13GW340	121.12	48.36	Eastern CAOB	Granodiorite	312	0	312	9.7	10.3	675	635	Ji Z et al., 2018 Lithos
923	13GW344-1	120.61	48.07	Eastern CAOB	Granodiorite	890	0	600	8.7	9.2	890	890	Ji Z et al., 2018 Lithos
924	13GW356	120.60	48.06	Eastern CAOB	Granodiorite	890	0	600	9.0	8.7	890	890	Ji Z et al., 2018 Lithos
925	SL14-017	91.49	41.97	Western CAOB	Granodiorite	308	0	308	14.6	14.8	381	372	Zhao LD et al., 2019 Gondwana Research
926	A1	79.90	46.95	Western CAOB	Granodiorite	345	0	345	14.5	14.3	414	429	Cao MJ et al., 2016 American Journal of Science
927	A2-1	79.97	46.97	Western CAOB	Granodiorite	328	0	328	14.5	14.7	402	392	Cao MJ et al., 2016 American Journal of Science
928	A2-3-2	79.97	46.97	Western CAOB	Granodiorite	323	0	323	13.9	14.1	441	426	Cao MJ et al., 2016 American Journal of Science
929	A2-7-1	79.97	46.97	Western CAOB	Granodiorite	323	0	323	14.4	14.8	408	388	Cao MJ et al., 2016 American Journal of Science
930	BCK2	84.46	45.50	Western CAOB	Granodiorite	310	0	310	12.9	12.8	483	487	Cao MJ et al., 2016 American Journal of Science
931	EGD001(75)	106.84	43.05	Eastern CAOB	Granodiorite	358	0	358	13.7	13.4	454	480	Dolgopolova A et al., 2013 Lithos
932	D514(16312-163	106.86	43.05	Eastern CAOB	Granodiorite	371	0	371	10.7	12.3	675	565	Dolgopolova A et al., 2013 Lithos
933	12K49	86.82	41.46	Western CAOB	Granodiorite	1934	0	600	-34.9	-35.7	3420	3465	Ge R et al., 2015 Geological Society of America Bulletin
934	11K07	86.35	41.69	Western CAOB	Granodiorite	830	0	600	-32.3	-31.9	3275	3252	Ge R et al., 2015 Geological Society of America Bulletin
935	11K97	86.60	41.52	Western CAOB	Granodiorite	821	0	600	-29.9	-27.1	3141	2985	Ge R et al., 2015 Geological Society of America Bulletin
936	B-2	74.68	47.08	Western CAOB	Granodiorite	320	0	320	6.5	6.6	854	846	Li GM et al., 2016 Gondwana Research
937	K-2	74.96	46.97	Western CAOB	Granodiorite	339	0	339	6.9	7.0	848	848	Li GM et al., 2016 Gondwana Research
938	K-3	74.97	46.98	Western CAOB	Granodiorite	325	0	325	8.7	8.3	737	757	Li GM et al., 2016 Gondwana Research
939	K-8	74.96	46.98	Western CAOB	Granodiorite	328	0	328	9.0	9.2	720	709	Li GM et al., 2016 Gondwana Research
940	S1-1	77.43	47.00	Western CAOB	Granodiorite	309	0	309	14.2	14.4	409	406	Li GM et al., 2016 Gondwana Research
941	S-A	77.33	47.00	Western CAOB	Granodiorite	310	0	310	13.3	13.4	455	449	Li GM et al., 2016 Gondwana Research
942	B70817-2.3	96.71	41.45	Western CAOB	Granodiorite	240	0	240	3.8	4.0	958	942	Li S et al., 2012 Lithos
943	NE13-30	132.22	45.48	Eastern CAOB	Granodiorite	249	0	249	0.5	0.6	1149	1143	Liu K et al., 2012 Lithos
944	NE13-38	132.22	45.48	Eastern CAOB	Granodiorite	209	0	209	4.2	4.3	903	897	Liu K et al., 2012 Lithos
945	NE13-39	132.22	45.48	Eastern CAOB	Granodiorite	239	0	239	5.3	5.5	834	823	Liu K et al., 2012 Lithos
946	12HS02-6	73.00	50.15	Western CAOB	Granodiorite	415	0	415	13.5	13.3	550	553	Shen P et al., 2018 Gondwana Research
947	9HS76-4	78.47	44.48	Western CAOB	Granodiorite	289	0	289	9.1	9.3	800	797	Shen P et al., 2018 Gondwana Research
948	12HS03-1	73.00	50.17	Western CAOB	Granodiorite	432	0	432	15.1	15.2	460	456	Shen P et al., 2018 Gondwana Research
949	9HS13-14	74.72	47.17	Western CAOB	Granodiorite	313	0	313	4.2	4.0	987	992	Shen P et al., 2018 Gondwana Research
950	9HS15-3-2	74.97	46.98	Western CAOB	Granodiorite	332	0	332	7.1	6.8	833	850	Shen P et al., 2018 Gondwana Research
951	HL-017	90.07	46.55	Western CAOB	Granodiorite	373	0	373	11.1	11.2	642	628	Wu C et al., 2015 Ore Geology Reviews
952	HL-037	90.05	46.62	Western CAOB	Granodiorite	366	0	366	11.5	11.4	615	619	Wu C et al., 2015 Ore Geology Reviews
953	Qiongkushitai	82.20	42.90	Western CAOB	Granodiorite	314	0	314	3.0	3.4	1054	1032	Yin J et al., 2016 International Journal of Earth Sciences
954	12ts30-2	86.55	42.69	Western CAOB	Granodiorite	551	0	551	-13.4	-13.3	2109	2130	Zhong LL et al., 2015 International Journal of Earth Sciences
955	13Un-16	70.33	41.65	Western CAOB	Granodiorite	299	0	299	3.9	4.0	997	997	Zhong LL et al., 2015 International Journal of Earth Sciences
956	OT03	90.31	45.20	Eastern Junggar	granodiorite	310	2	310	12.8	12.9	488	481	Liu W et al., 2013 Lithos
957	KLP03	90.32	45.31	Eastern Junggar	granodiorite	315	2	315	12.8	13.0	493	482	Liu W et al., 2013 Lithos
958	HB29	86.07	48.32	Chinese Altai	granodiorite	313	5	313	9.7	10.7	668	613	Cai Keda et al., 2012 Gondwana Research
959	3214	88.17	47.93	Chinese Altai	Granodiorite	202	0	202	5.2	5.0	843	850	Wang Tao et al., 2014
960	xj40	95.29	41.90	Beishan	granodiorite	425	0	425	-7.2	-5.2	1724	1613	Lei R et al., 2011 Gondwana Research
961	G0208-1	122.12	46.49	Hegenshan ophiolite-arc-accretion complex	Granodiorite	135	0	150	8.4	8.4	613	614	Zhou Q et al., 2011 Acta Petrologica et Mineralogica.(ICWEA)
962	MB1-3	113.60	43.64	Baolidao arc-accretion complex	Granodiorite	472	0	472	9.3	9.3	820	819	Shi YR et al., 2016 Journal of Asian Earth Sciences
963	BLD-1	113.59	43.66	Baolidao arc-accretion complex	Granodiorite	316	0	316	8.7	10.3	726	637	Hu CS et al., 2015 Journal of Asian Earth Sciences
964	40	116.22	43.73	Baolidao arc-accretion complex	Granodiorite	252	0	252	7.6	9.6	739	628	Zhou XW et al., 2012 Doctoral thesis(ICWEA)
965	11XL-5.2	117.60	43.92	Baolidao arc-accretion complex	Granodiorite	277	0	277	9.0	9.0	680	681	Li S et al., 2016 Scientific Reports
966	14SJ55	119.09	44.60	Baolidao arc-accretion complex	Granodiorite	130	0	150	-6.9	-6.8	1496	1493	Liu et al., 2016
967	XL920-8	116.90	43.54	Erdaojing accretion complex	Granodiorite	252	0	252	11.6	11.7	511	506	Li S et al., 2016 Scientific Reports
968	11SH-5	117.56	43.84	Erdaojing accretion complex	Granodiorite	253	0	253	9.8	9.9	615	608	Li S et al., 2016 Scientific Reports
969	11SH-3	117.33	43.76	Erdaojing accretion complex	Granodiorite	275	0	275	9.3	9.1	661	675	Li S et al., 2016 Gondwana Research
970	AST01-1	117.48	43.84	Erdaojing accretion complex	Granodiorite	246	0	246	10.7	10.8	557	553	Liu JF et al., 2014 Acta Geologica Sinica(ICWEA)
971	JG21-29-58	118.61	43.99	Erdaojing accretion complex	Granodiorite	249	0	249	12.5	12.4	454	460	Liu JF et al., 2013 Acta Petrologica Sinica(ICWEA)
972	BD09-007-01	113.62	42.41	Northern margin of the North China Craton	Granodiorite	446	0	446	5.4	5.9	1020	996	Hao BW et al., 2011 Doctoral thesis(ICWEA)
973	ZK2805-55m	113.54	42.40	Northern margin of the North China Craton	Granodiorite	268	0	268	0.5	0.8	1161	1147	Liu CH et al., 2015 Lithos
974	NM07-144	110.31	41.16	North China Craton	Granodiorite	280	0	280	-13.2	-13.0	1956	1947	Zhang XH et al., 2011 Lithos
975	DQ08-38	111.84	41.00	North China Craton	Granodiorite	142	0	150	-12.8	-15.0	1834	1959	Guo L et al., 2012 International Geology Review
976	D079-1	117.10	41.03	North China Craton	Granodiorite	288	0	288	-21.7	-21.0	2442	2404	Zhang SH et al., 2009a International Journal of Earth Science (Geological Rundsch)
977	ALT004	90.81	46.47	chaganguole	granodiorite	394	0	394	4.1	4.1	1056	1056	Ye et al., 2015
978	BH29	86.08	48.28	Tuokesalei intrusion	granodiorite	313	0	313	9.7	10.7	668	613	Cai et al., 2012
979	KLP03	90.32	45.31	SW. portion of Kurankazigan	granodiorite	315	0	315	12.8	13.0	493	482	Liu et al., 2013
980	10XJ-51B	90.03	45.68	kulankazigan	granodiorite	442	0	442	9.7	9.4	772	790	Li., 2012
981	10XJ-214	90.27	45.63	kasikersuokekannan	granodiorite	430	0	430	13.1	12.7	566	588	Li, 2012
982	10XJ-95B	92.59	44.23	balikunmeikuangbei	granodiorite	415	0	415	11.4	11.2	651	662	Li, 2012
983	KLG08	89.30	46.70	NW China	granodiorite	331	0	331	13.5	13.5	462	462	Liu XJ et al., 2019 Lithos
984	12GW051	131.89	46.30	Jiamusi Block	granodiorite	530	5	530	-2.2	-2.2	1521	1523	Bi JH et al., 2014 Journal of Asian Earth Sciences
985	15GW073	130.51	46.44	Jiamusi Block	Granodiorite	278	2	278	-1.1	-1.1	1265	1264	Dong Y et al., 2017 Gondwana Research
986	10GW251	131.72	47.03	Jiamusi Block	Granodiorite	260	8	260	3.6	2.4	981	1046	Bi JH et al., 2014 Journal of Asian Earth Sciences
987	11HNA11	128.55	43.83	LXZR	Granodiorite	252	2	252	7.7	7.5	738	746	Yu JJ et al., 2013 Gondwana Research
988	H15-58-01	128.54	46.44	LXZR	Granodiorite	181	1	181	5.0	5.1	837	830	Ge MH et al., 2019 Lithosphere
989	1055-1	127.14	50.18	LXZR	Granodiorite	181	2	181	9.2	9.7	594	561	Gou J et al., 2013 Journal of Jilin University (Earth Science Edition)
990	STH-2	128.71	44.86	LXZR	Granodiorite	198	3	198	13.1	13.2	380	371	Qin JF et al., 2016 Lithosphere
991	15YL10-1	127.48	45.68	LXZR	Granodiorite	186	1	186	9.1	8.9	604	614	Zhu CY et al., 2017 Lithosphere
992	15YL14-1	128.50	47.60	LXZR	Granodiorite	183	1	183	2.2	2.3	997	993	Zhu CY et al., 2017 Lithosphere
993	15YL18-4	128.18	46.92	LXZR	Granodiorite	183	1	183	2.4	2.7	985	972	Zhu CY et al., 2017 Lithosphere
994	P10-2	129.82	47.41	LXZR	Granodiorite	244	2	244	-3.4	-4.0	1371	1402	Vei HY et al., 2012 Earth Science Journal of China University of Geosciences(ICWEA)
995	P10-1	129.99	47.38	LXZR	Granodiorite	234	2	234	1.3	1.7	1091	1068	Vei HY et al., 2012 Earth Science Journal of China University of Geosciences(ICWEA)
996	DW11-b6	117.49	44.98	Hegenshan ophiolite-arc-accretion complex	Granodiorite	238	0	238	14.3	14.4	342	339	Xiong GQ et al., 2015 geological review(ICWEA)
997	G0208-3	122.12	46.49	Hegenshan ophiolite-arc-accretion complex	Granodiorite	135	0	150	8.7	8.7	598	596	Zhou Q et al., 2011 Acta Petrologica et Mineralogica(ICWEA)
998	0013-102	116.15	43.82	Baolidao arc-accretion complex	Granodiorite	318	0	318	12.0	11.9	541	543	Zhou XW et al., 2012 Doctoral thesis(ICWEA)
999	XL921-14	116.64	43.76	Baolidao arc-accretion complex	Granodiorite	251	0	251	11.8	12.0	496	489	Li S et al., 2016 Scientific Reports
1000	CGDLH05	110.83	41.89	Northern margin of the North China Craton	Granodiorite	245	0	245	-3.4	-3.3	1368	1364	Zhang W et al., 2010 Acta Geologica Sinica(ICWEA)
1001	A003-5-01	120.59	40.38	North China Craton	Granodiorite	225	0	225	-18.6	-18.3	2220	2204	Li J et al., 2009 Master's thesis(ICWEA)
1002	DSL09-4	121.89	42.39	North China Craton	Granodiorite	220	0	220	0.5	1.9	1126	1045	Zhang XH et al., 2012a Lithos
1003	06XJ-017	80.98	44.67	0	Granodiorite porphyry	366	3	366	2.1	2.4	1149	1131	Tang et al., 2010 Lithos
1004	KKS-20	81.90	44.70	0	Granodiorite porphyry	289	2	289	7.8	8.3	761	732	Zhang DY et al., 2012 Lithos

1005	BCK2-1	84.51	45.40	0	Granodiorite Porphyry	310	1.6	310	12.9	12.8	483	487	Cao, M et al., 2016 Lithos
1006	SYH1	82.47	45.78	0	granodiorite porphyry	295	2	295	13.0	13.4	461	442	Shen et al., 2017 Ore Geology Reviews
1007	SYP8-4	82.50	45.79	0	granodiorite porphyry	294	2	294	12.8	12.4	476	498	Shen et al., 2017 Ore Geology Reviews
1008	15GW240	130.69	46.10	0	Granodiorite porphyry	123	1	150	9.3	9.4	562	556	Dong Y et al., 2016 Global Geology(ICWEA)
1009	B11	119.14	49.98	0	Granodiorite porphyry	231	3	231	5.1	5.0	870	873	Kang YJ et al., 2018 Ore Geology Reviews
1010	B24	119.14	49.98	0	Granodiorite porphyry	229	1	229	5.3	5.2	857	860	Kang YJ et al., 2018 Ore Geology Reviews
1011	BLS-7	120.12	44.05	Erdaojing accretion complex	Granodiorite porphyry	134	0	150	2.6	2.5	951	956	Zhang XJ et al,. 2010 Acta Geologica Sinica(ICWEA)
1012	000-209.2-01	113.55	42.39	Northern margin of the North China Craton	Granodiorite porphyry	269	0	269	4.6	4.8	931	917	Hao BW et al., 2011 Doctoral thesis(ICWEA)
1013	14L7-7	113.55	42.39	Northern margin of the North China Craton	Granodiorite porphyry	269	0	269	5.0	5.6	904	870	Liu CH et al., 2015 Lithos
1014	HMG14-6	81.04	44.42	0	Granodiorite-Granite	342	4	342	3.2	3.0	1069	1079	Huang H et al., 2020 Earth-Science Reviews
1015	ZK04-91	130.82	44.26	CAOB	granophyric granite	201	3	201	1.8	1.7	1036	1044	Zhang HF et al., 2013 Ore Geology Reviews
1016	DMP1	114.50	40.80	North China craton	Granulite	47	0	150	1.0	9.9	1039	526	Zheng JP et al., 2009 Chemical Geology
1017	DMP-508	114.50	40.80	North China craton	Granulite	118	0	150	-19.2	-19.3	2196	2203	Jiang N et al., 2010 Earth and Planetary Science Letters
1018	JN0724	114.50	40.80	North China craton	Granulite	2471	0	600	-38.8	-38.9	3641	3644	Jiang N et al., 2010 Earth and Planetary Science Letters
1019	JN0815	114.50	40.80	North China craton	Granulite	123	0	150	-20.3	-20.4	2261	2266	Jiang N et al., 2010 Earth and Planetary Science Letters
1020	11JD12-1	118.27	40.33	North China craton	Granulite	2523	0	600	-38.7	-38.7	3634	3635	Guo RR et al., 2013 Gondwana Research
1021	OFX08-5	121.58	42.39	North China craton	Granulite	2603	0	600	-38.5	-38.2	3621	3606	Wang W et al., 2015 Earth-Science Reviews
1022	L-09-02	112.80	66.33	Eastern CAOB	Granulite	1986	0	600	-34.2	-33.6	3381	3352	Shatsky, V.S. et al., 2016 Precambrian Research
1023	UD-01-44	112.32	66.43	Eastern CAOB	Granulite	1779	0	600	-46.5	-46.7	4066	4074	Shatsky, V.S. et al., 2016 Precambrian Research
1024	YUB-27-02	110.63	66.13	Eastern CAOB	Granulite	1859	0	600	-36.3	-36.3	3498	3501	Shatsky, V.S. et al., 2016 Precambrian Research
1025	01-106	112.80	66.27	Eastern CAOB	Granulite	1830	0	600	-36.5	-36.4	3512	3507	Moyen J.F. et al., 2017 Earth and Planetary Science Letters
1026	02-131	112.80	66.27	Eastern CAOB	Granulite	1840	0	600	-45.8	-45.6	4024	4014	Moyen J.F. et al., 2017 Earth and Planetary Science Letters
1027	02-177	112.80	66.27	Eastern CAOB	Granulite	2700	0	600	-44.7	-45.0	3966	3981	Moyen J.F. et al., 2017 Earth and Planetary Science Letters
1028	02-34	112.80	66.27	Eastern CAOB	Granulite	1840	0	600	-30.4	-30.5	3171	3175	Moyen J.F. et al., 2017 Earth and Planetary Science Letters
1029	02-36	112.80	66.27	Eastern CAOB	Granulite	1840	0	600	-46.6	-46.6	4069	4070	Moyen J.F. et al., 2017 Earth and Planetary Science Letters
1030	FZ-GU12	113.15	40.44	North China craton	Granulite	1919	0	600	-27.3	-27.3	2996	2996	Zeng L et al., 2017 Precambrian Research
1031	FZ-GU6	113.15	40.44	North China craton	Granulite	1999	0	600	-24.3	-24.3	2828	2828	Zeng L et al., 2017 Precambrian Research
1032	08YK03	114.42	40.28	North China Craton	granulite	1810	0	600	-32.5	-32.7	3287	3297	Min Q et al.2012
1033	ALT63	87.80	47.98	Chinese Altai	Grey biotite granite	421	6	421	7.1	7.3	902	896	Cai KD et al., 2011 Journal of Asian Earth Sciences
1034	YNA14-5	82.10	44.09	0	highly fractionated granite	305	3	305	9.1	9.0	699	702	Huang H et al., 2020 Earth-Science Reviews
1035	BJSB	84.49	45.38	0	Hornblede diorite porphyr	306	3.1	306	14.8	15.2	368	344	Cao, M et al., 2016 Lithos
1036	AG-1-1.1	84.82	45.67	Western Junggar	Host granite	313	0	313	10.6	10.5	613	610	Li Di, et al, 2013 Geoscience Frontiers
1037	HL-1-R	129.20	42.50	NE China	igneous rocks	249	1	249	1.4	1.0	1095	1121	Guan, Q.et al. 2020 Gondwana Research
1038	HL-5	129.24	42.45	NE China	igneous rocks	235	0.5	235	-11.2	-10.9	1809	1792	Guan, Q.et al. 2020 Gondwana Research
1039	HL-3	129.18	42.48	NE China	igneous rocks	176	0.7	176	7.9	7.8	662	672	Guan, Q.et al. 2020 Gondwana Research
1040	HL-2	129.19	42.52	NE China	igneous rocks	208	0.6	208	8.5	8.0	654	682	Guan, Q.et al. 2020 Gondwana Research.
1041	HL-6	129.26	42.51	NE China	igneous rocks	179	0.8	179	8.7	8.8	618	613	Guan, Q.et al. 2020 Gondwana Research.
1042	Mo46-2	92.52	46.11	SW Mongolia	Igneous-related portions	420	2	420	-0.9	-1.6	1364	1401	Yingde Jiang et al., 2012 Lithos
1043	Mo53-1	92.50	46.09	SW Mongolia	Igneous-related portions	510	0	510	-1.3	-1.3	1459	1459	Yingde Jiang et al., 2012 Lithos
1044	Mo55-1	92.52	46.07	SW Mongolia	Igneous-related portions	490	0	490	-2.3	-0.3	1496	1384	Yingde Jiang et al., 2012 Lithos
1045	C14BL21	81.85	45.17	0	Ignimbrite	300	3	300	12.3	12.3	509	507	Wang XS et al., 2018 Journal of Asian Earth Sciences
1046	XKL-ZK320-3	124.53	51.66	CAOB	Granite	124	0.6	150	2.7	3.0	942	927	Gong JD et al., 2021 Journal of Jilin University(Earth Science Edition)(ICWEA)
1047	776	87.89	42.23	Tianshan	K-feldspar granite	272	2	272	-3.5	-3.4	1398	1393	Ma et al., 2015 Gondwana Research.
1048	776-1	87.89	42.23	Tianshan	K-feldspar granite	272	2	272	-3.3	-3.4	1388	1388	Ma et al., 2015 Gondwana Research.
1049	763	87.46	42.85	Tianshan	K-feldspar granite	337	3	337	4.4	4.2	992	1004	Ma XX et al.,2014 Gondwana Research.
1050	782	86.35	42.87	Tianshan	K-feldspar granite	434	4	434	4.9	4.6	1042	1056	Ma XX et al.,2014 Gondwana Research.
1051	791	86.51	42.88	Tianshan	K-feldspar granite	366	4	366	-3.3	-0.6	1455	1305	Ma XX et al.,2014 Gondwana Research.
1052	755	88.53	42.40	Tianshan	K-feldspar granite	283	2	283	8.6	8.9	708	693	Ma XX et al.,2014 Gondwana Research.
1053	762	87.65	42.84	Tianshan	K-feldspar granite	267	4	267	11.2	11.4	547	533	Ma XX et al.,2014 Gondwana Research.
1054	792	86.56	42.88	Tianshan	K-feldspar granite	263	2	263	5.1	5.1	897	896	Ma XX et al.,2014 Gondwana Research.
1055	WLST III -6	86.64	42.90	0	K-feldspar granite	313	4	313	5.4	5.3	918	924	Huang H et al., 2015Acta Geologica Sinica(ICWEA)
1056	ZS34	81.12	43.22	0	K-feldspar granite	360	2	360	-0.3	-0.3	1284	1282	Bao ZH et al., 2018 Journal of Asian Earth Sciences
1057	WJ1125	85.51	46.71	0	K-feldspar granite	420	1	420	13.7	13.9	523	510	Yin et al., 2017 Gondwana Research.
1058	B16	119.13	49.99	0	K-feldspar granite	241	1	241	4.8	5.2	897	874	Kang YJ et al., 2018 Ore Geology Reviews
1059	DL10-8	112.84	44.71	Uliastai active continental margin	K-feldspar granite	160	0	160	8.7	8.8	604	602	Xue FH et al,. 2015 Acta Geologica Sinica(ICWEA)
1060	HG-68-1	117.48	43.64	Erdaojing accretion complex	K-feldspar granite	145	2	150	6.1	6.2	747	741	Mei W et al., 2016 Geosciences Journal
1061	Mo45-3	92.54	46.13	SW Mongolia	Leucocratic vein	385	3	385	-12.7	-14.0	2008	2083	Jiang YD et al.,2012 Lithos
1062	MO97	110.26	47.32	CAOB	leucogabbro	479	3	479	3.0	5.9	1190	1020	Alfred Kröner et al., 2017 Gonwana Research
1063	FZ-G	113.15	40.44	North China craton	Leucogranite	2164	0	600	-34.9	-34.7	3421	3412	Zeng L et al., 2017 Precambrian Research
1064	11WS02	112.50	40.50	North China craton	Leucogranite	1950	0	600	-26.1	-26.1	2931	2928	Wang LJ et al., 2017 Precambrian Research
1065	BA11-130	97.00	41.80	Western CAOB	Leucogranite	402	0	402	7.5	8.2	866	829	Zheng RG et al., 2018 Journal of Asian Earth Sciences
1066	ALT13	88.24	47.58	Chinese Altai	leucogranite	411	0	411	5.3	4.8	996	1033	Cai, 2011
1067	09M16	120.23	44.54	0	Mafic enclaves	132	1	150	6.7	6.6	710	716	Ma XH et al., 2013 Gondwana Research
1068	BJG10	102.97	39.61	NW China	magmatic rocks	400	5	400	12.8	12.8	557	557	Xiao Y.et al., 2011 Gondwana Research
1069	BJG11	102.96	39.60	NW China	magmatic rocks	435	8	435	5.7	5.4	993	1011	Xiao Y.et al., 2011 Gondwana Research
1070	BJG14	102.96	39.58	NW China	magmatic rocks	315	4	315	11.6	11.6	560	558	Xiao Y.et al., 2011 Gondwana Research
1071	BJG15	102.96	39.62	NW China	magmatic rocks	325	5	325	10.3	10.3	645	643	Xiao Y.et al., 2011 Gondwana Research
1072	ZPG-1	109.41	36.34	NW China	magmatic rocks	410	11	410	5.5	5.4	985	995	Xiao Y.et al., 2011 Gondwana Research
1073	ZPG-3	109.41	36.34	NW China	magmatic rocks	410	11	410	14.6	13.5	460	527	Xiao Y.et al., 2011 Gondwana Research
1074	CXPC3-1	118.76	44.52	0	ium-fine grained biotite gr	139	1	150	7.9	8.0	644	636	Wei W et al., 2021 Journal of Earth Science(ICWEA)
1075	CDE4111-8	119.22	44.41	0	edium-fine-grained Bt grar	137	0	150	6.0	5.9	754	758	Zhang ZQ et al., 2017 Journal of mineral exploration(ICWEA)
1076	BH7-3	86.74	48.39	Chinese Altai	edium-grained biotite gran	404	10	404	4.7	3.8	1027	1082	Cai Keda et al., 2011 Journal of Earth Science
1077	ALT01	88.08	48.80	Chinese Altai	edium-grained biotite gran	368	13	368	5.6	5.8	950	939	Cai Keda et al., 2011 Journal of Earth Science
1078	Zk5215-487	82.48	45.79	0	medium-grained granite	295	3	295	13.1	12.7	457	480	Shen et al., 2017 Ore Geology Reviews
1079	zk9235-447	82.50	45.79	0	medium-grained granite	298	2	298	13.5	13.6	434	433	Shen et al., 2017 Ore Geology Reviews
1080	Bu-3	86.97	48.05	Chinese Altai	Medium-grained granite	425	8	425	6.1	6.0	965	968	Sun M et al., 2008 Chemical Geology
1081	ARJ01	89.22	47.48	Chinese Altai	ledium-grained granodiori	404	4	404	8.2	8.1	829	833	Cai Keda et al., 2011 Journal of Earth Science
1082	WZZ06	119.12	40.37	North China craton	Meta-Felsic Volcanic Rock	2500	0	600	-38.8	-39.3	3639	3665	Lv B et al., 2012 Precambrian Research
1083	YKH09	90.03	46.60	Eastern Junggar	mineralized diorite porphyr	379	1.9	379	1.6	9.7	1178	719	Yang FQ et al., 2014 Lithos
1084	08AET052	90.00	46.00	Western CAOB	Mme	299	0	299	13.1	13.1	462	461	Tang GJ et al., 2017 Geochemistry Geophysics Geosystems
1085	TKSC14-8	81.90	42.87	0	Monzodiorite	353	2	353	6.4	6.4	892	894	Huang H et al., 2020 Earth-Science Reviews
1086	SPM13TC19	124.73	51.91	0	Monzodiorite	123	1	150	2.8	2.8	940	938	Xu LM et al., 2017Master's thesis(ICWEA)
1087	H15-61-5	127.23	45.39	Eastern CAOB	Monzodiorite	179	0	179	4.6	4.1	856	887	Ge MH et al., 2017 Tectonophysics
1088	15XH19-1	129.22	44.93	Eastern CAOB	Monzodiorite	325	0	325	8.5	8.5	751	746	Guo P et al., 2018 Gondwana Research

1089	16XH1-1	129.19	44.96	Eastern CAOB	Monzodiorite	324	0	324	8.3	8.4	761	751	Guo P et al., 2018 Gondwana Research
1090	EGRCD0073	106.90	43.14	Eastern CAOB	Monzodiorite	324	0	324	13.9	13.6	461	477	Dolgopolova, A et al.,2013 Lithos
1091	OTD258(82.5)	106.85	43.02	Eastern CAOB	Monzodiorite	353	0	353	11.6	11.7	594	588	Dolgopolova, A et al.,2013 Lithos
1092	HDL11-1	129.49	45.68	LXZR	monzodiorite	424	2	424	-1.3	-1.1	1388	1377	Wang et al., 2012 Tectonophysics
1093	SZ07-26	111.96	41.44	North China Craton	Monzodiorite	317	0	317	-15.9	-15.8	2137	2130	Zhang Xiaohui et al., 2012 Geological Society of London
1094	12K01	86.90	41.72	0	Monzogranite	408	5	408	0.0	0.4	1303	1277	Ge et al.. 2014 Tectonics
1095	12K16	86.51	41.71	0	Monzogranite	406	4	406	-21.1	-20.3	2498	2458	Ge et al.. 2014 Tectonics
1096	XJ048-3	85.68	42.19	0	Monzogranite	405	2	405	7.0	6.9	897	904	Lin W et al., 2013 Lithosphere
1097	TS1342-1	87.12	42.81	0	Monzogranite	362	2	362	4.1	4.1	1029	1031	Yin et al., 2016 Gondwana Research
1098	06XJ71	82.57	43.58	0	Monzogranite	317	3	317	10.3	10.3	638	637	Tang et al., 2014 Lithos
1099	06XJ-74	82.57	43.58	0	Monzogranite	310	4	310	11.6	11.1	556	587	Tang et al., 2014, Lithos
1100	JHB14-11	82.88	44.28	0	Monzogranite	300	3	300	5.5	5.5	903	900	Huang H et al., 2020, Earth-Science Reviews
1101	XY2_1	84.53	43.68	0	Monzogranite	316	2	316	12.0	12.3	539	520	Xu XY et al., 2010, Acta Petrologica et Mineralogica
1102	ZS21	81.11	43.23	0	Monzogranite	362	1	362	-0.6	-0.9	1300	1316	Bao ZH et al., 2018, Journal of Asian Earth sciencesences
1103	ZS26	81.11	43.23	0	Monzogranite	362	1	362	-1.1	-1.0	1328	1322	Bao ZH et al., 2018, Journal of Asian Earth sciencesences
1104	C14BL49	80.87	44.90	0	Monzogranite	372	2	372	3.9	3.3	1051	1086	Sun MJ et al., International Geology Review
1105	C14BL59	80.95	44.91	0	Monzogranite	390	2	390	2.2	2.3	1159	1158	Sun MJ et al., International Geology Review
1106	08AET72	89.53	46.19	0	Monzogranite	329	4	329	12.6	12.8	511	504	Tang et al., 2019 Earth and Planetary Science Letters
1107	10LX-41	118.02	43.97	0	Monzogranite	245	2	245	11.6	9.4	504	312	Zhao L et al., 2019, Journal of Asian Earth sciencesences
1108	XL920-2	117.64	43.42	Erdaojing accretion complex	Monzogranite	139	1	150	3.9	3.7	876	888	Li S et al., 2017a, Tectonics.
1109	14RS-4	118.92	43.78	Erdaojing accretion complex	Monzogranite	136	1	150	3.2	3.1	913	924	Li S et al., 2017a, Tectonics.
1110	13XA19-1	121.31	49.60	Eastern CAOB	Monzogranite	236	1	236	10.7	10.7	547	549	Li Y et al., 2018, Lithos
1111	16DX6-1	122.58	47.88	Eastern CAOB	Monzogranite	221	1	221	6.5	6.7	779	771	Li Y et al., 2018, Lithos
1112	16DX10-1	123.03	48.57	Eastern CAOB	Monzogranite	168	1	168	4.9	4.8	834	834	Li Y et al., 2018, Lithos
1113	13XA10-1	122.60	47.71	Eastern CAOB	Monzogranite	164	1	164	8.4	8.4	628	626	Li Y et al., 2018, Lithos
1114	M241-1	119.87	50.85	0	Monzogranite	880	10	600	0.2	0.2	1439	1442	Gou J et al., 2013, Journal of Asian Earth sciencesences.
1115	M256-1	119.98	51.32	0	Monzogranite	894	12	600	0.4	0.4	1432	1431	Gou J et al., 2013, Journal of Asian Earth sciencesences.
1116	M424-1	119.88	50.84	0	Monzogranite	306	4	306	8.2	8.2	748	750	Gou J et al., 2013, Journal of Asian Earth sciencesences.
1117	15JJW6-1	126.25	43.24	0	Monzogranite	175	1	175	6.7	6.8	735	728	Wang F et al., 2019, Journal of Geophysical Research: Solid Earth
1118	11ER26-1	120.31	51.35	0	Monzogranite	206	2	206	1.3	1.2	1069	1077	Tang J et al., 2016, Gondwana Research
1119	12ER16-3	120.37	51.32	0	Monzogranite	206	2	206	0.4	0.3	1123	1124	Tang J et al., 2016, Gondwana Research
1120	12ER17-1	120.43	51.31	0	Monzogranite	205	2	205	-0.9	-1.0	1194	1202	Tang J et al., 2016, Gondwana Research
1121	11ER13-1	119.53	50.77	0	Monzogranite	203	3	203	3.2	2.9	957	973	Tang J et al., 2016, Gondwana Research
1122	11ER10-1	119.53	50.74	0	Monzogranite	202	2	202	1.0	0.9	1081	1088	Tang J et al., 2016, Gondwana Research
1123	13GF-40	128.04	42.31	0	Monzogranite	114	2	150	4.2	4.3	860	854	Guo F et al., 2019, Chemical Geology
1124	13GF-19	128.51	42.87	0	Monzogranite	159	5	159	-1.5	-1.5	1196	1193	Guo F et al., 2019, Chemical Geology
1125	13GF08	128.04	42.87	0	Monzogranite	167	2	167	4.4	5.0	860	827	Guo F et al., 2019, Chemical Geology
1126	13GF-59	129.09	42.53	0	Monzogranite	177	2	177	-2.0	1.9	1229	1012	Guo F et al., 2019, Chemical Geology
1127	13GF-48	129.82	42.21	0	Monzogranite	245	6	245	-7.6	-7.4	1612	1600	Guo F et al., 2019, Chemical Geology
1128	13GF-03	130.83	43.14	0	Monzogranite	179	1	179	9.3	9.4	584	579	Guo F et al., 2019, Chemical Geology
1129	13GF-31	128.77	42.88	0	Monzogranite	181	2	181	7.1	7.0	713	720	Guo F et al., 2019, Chemical Geology
1130	13GF-21	128.51	42.87	0	Monzogranite	183	3	183	10.3	10.1	533	543	Guo F et al., 2019, Chemical Geology
1131	13GF-70	129.51	43.13	0	Monzogranite	184	3	184	8.8	8.8	616	618	Guo F et al., 2019, Chemical Geology
1132	13GF-38	128.11	42.98	0	Monzogranite	186	0	186	10.0	10.1	553	543	Guo F et al., 2019, Chemical Geology
1133	13GF-101	129.54	43.65	0	Monzogranite	190	2	190	9.4	9.1	589	607	Guo F et al., 2019, Chemical Geology
1134	13GF-79	130.97	43.89	0	Monzogranite	196	3	196	10.3	10.3	542	540	Guo F et al., 2019, Chemical Geology
1135	13GF-69	129.62	42.58	0	Monzogranite	196	7	196	8.7	8.7	635	634	Guo F et al., 2019, Chemical Geology
1136	13GF-89	129.50	43.83	0	Monzogranite	197	2	197	6.6	6.7	757	749	Guo F et al., 2019, Chemical Geology
1137	13GF-117	130.38	43.71	0	Monzogranite	204	2	204	9.6	9.6	589	589	Guo F et al., 2019, Chemical Geology
1138	13GF-85	130.73	43.09	0	Monzogranite	205	1	205	10.5	10.7	535	526	Guo F et al., 2019, Chemical Geology
1139	13GF-93	129.42	43.70	0	Monzogranite	215	5	215	8.9	8.8	640	642	Guo F et al., 2019, Chemical Geology
1140	11ER17-1	120.04	51.05	0	Monzogranite	242	3	242	5.1	5.0	876	885	Tang J et al., 2014, Lithos
1141	14JEW3-1	130.08	43.42	0	Monzogranite	183	1	183	8.0	8.1	663	656	Wang F et al., 2017, Journal of Asian Earth sciencesences
1142	13GW210	122.38	47.75	0	Monzogranite	212	2	212	6.9	6.7	748	760	Yang H et al., 2016, Journal of Asian Earth sciencesences
1143	13GW228	122.33	47.77	0	Monzogranite	214	3	214	8.5	8.0	660	686	Yang H et al., 2016, Journal of Asian Earth sciencesences
1144	13GW255	122.59	47.89	0	Monzogranite	206	2	206	8.5	8.4	653	660	Yang H et al., 2016, Journal of Asian Earth sciencesences
1145	PM008-4-1	120.71	46.88	Hegenshan ophiolite-arc-accretion complex	Monzogranite	243	3	243	9.4	9.5	632	627	Liu X et al., 2015, Acta Petrologica Et Mineralogica(ICWEA)
1146	M141-1	117.06	49.44	0	Monzogranite	179	1	179	1.8	1.7	1017	1027	Wang TH et al., 2014, Global Geology.(ICWEA)
1147	1702-1	119.26	50.36	0	Monzogranite	134	1	150	2.0	1.8	985	997	Gou J et al., 2019, International Geology Review
1148	13GW305	121.68	49.48	0	Monzogranite	144	2	150	7.5	7.6	664	662	He Y et al., 2018, Canadian Journal of Earth sciencesences
1149	13GW258	121.89	48.44	0	Monzogranite	137	1	150	6.3	6.3	737	735	He Y et al, 2017, Global Geology(ICWEA)
1150	13GW262	121.80	48.53	0	Monzogranite	132	2	150	5.6	5.1	775	803	He Y et al, 2017, Global Geology(ICWEA)
1151	13GW375	120.50	47.72	0	Monzogranite	141	2	150	5.5	5.6	784	774	Ji Z et al., 2016, Global Geology(ICWEA)
1152	GW05129	127.08	50.89	0	Monzogranite	171	2	171	9.3	9.0	580	595	Sui ZM et al., 2007, Acta Petrologica Sinica(ICWEA)
1153	GW07007	126.90	49.05	0	Monzogranite	316	4	316	7.3	7.4	808	805	Zhang YL et al., 2010, Acta Petrologica Sinica(ICWEA)
1154	GW07009	126.91	49.10	0	Monzogranite	315	4	315	8.8	8.8	721	722	Zhang YL et al., 2010, Acta Petrologica Sinica(ICWEA)
1155	GW07012	126.52	48.85	0	Monzogranite	169	3	169	8.0	7.7	656	671	Zhang YL et al., 2010, Acta Petrologica Sinica(ICWEA)
1156	GW07017	126.37	49.03	0	Monzogranite	187	6	187	8.9	9.3	615	590	Zhang YL et al., 2010, Acta Petrologica Sinica(ICWEA)
1157	GW07019	126.36	48.94	0	Monzogranite	171	4	171	6.9	7.1	716	706	Zhang YL et al., 2010, Acta Petrologica Sinica(ICWEA)
1158	13GW017	131.53	45.08	0	Monzogranite	211	2	211	2.8	3.9	987	922	Yang H et al., 2015, Lithos
1159	18HL-10-1	130.46	45.76	0	monzogranite	518	4	518	-6.9	-5.5	1783	1705	Ge MH et al., 2021, Lithos
1160	18HL-13-1	130.35	45.71	0	monzogranite	496	4	496	-6.9	-6.9	1762	1766	Ge MH et al., 2021, Lithos
1161	11GW028	132.41	45.57	0	Monzogranite	215	0	215	4.2	4.2	909	910	Yang H et al., 2015, Gondwana Research
1162	12GW010	131.42	45.03	0	Monzogranite	287	3	287	1.2	1.2	1136	1140	Yang H et al., 2015, Gondwana Research
1163	FAP-N1-Q1	127.31	44.39	0	Monzogranite	172	1	172	7.4	7.4	687	691	Zhang, LS et al., 2021, Acta Geology Sin. (ICWEA)
1164	D1019-2-2	121.18	44.98	Baolidao arc-accretion complex	Monzogranite	228	1	228	9.9	10.2	590	574	Li SC et al., 2016, Acta Petrologica Sinica(ICWEA)
1165	DHS-3-01	124.46	50.53	0	Monzogranite	161	3	161	6.6	6.6	726	725	Zhang YT et al., 2018, Lithos
1166	1583-1	122.33	52.89	0	Monzogranite	199	2	199	3.4	3.7	942	926	Sun DY et al., 2013, International Geology Review
1167	1588-1	121.99	52.76	0	Monzogranite	187	3	187	1.6	2.6	1036	982	Sun DY et al., 2013, International Geology Review
1168	1602-2	121.24	52.45	0	Monzogranite	193	0	193	3.5	3.5	930	929	Gou J et al., 2017, International Geology Review
1169	1608-1	124.70	52.35	0	Monzogranite	188	0	188	-0.8	-1.1	1175	1190	Gou J et al., 2017, International Geology Review
1170	1610-1	124.70	52.35	0	Monzogranite	185	2	185	-9.2	-13.5	1656	1902	Gou J et al., 2017, International Geology Review
1171	1662-1	121.04	52.49	0	Monzogranite	210	2	210	2.3	2.5	1013	1004	Sun DY et al., 2013, International Geology Review
1172	1676-1	121.30	51.03	0	Monzogranite	202	2	202	0.3	-0.2	1125	1151	Gou J et al., 2017, International Geology Review

1173	M224	120.15	50.79	0	Monzogranite	193	3	193	1.8	2.0	1028	1021	Gou J et al., 2017, International Geology Review
1174	14HYC17-1	130.01	47.20	0	Monzogranite	249	2	249	-4.1	-4.2	1412	1419	Long XY et al., 2020, Gondwana Research
1175	15XH15-5	129.88	45.70	0	Monzogranite	215	1	215	1.4	1.2	1071	1081	Long XY et al., 2020, Gondwana Research
1176	14HYC13-1	130.04	47.37	0	Monzogranite	245	2	245	-2.7	-2.9	1332	1339	Long XY et al., 2020, Gondwana Research
1177	15XH13-1	129.81	45.72	0	Monzogranite	222	4	222	2.9	3.1	989	975	Long XY et al., 2020, Gondwana Research
1178	15XH6-1	129.47	45.82	0	Monzogranite	215	2	215	5.1	5.0	855	864	Long XY et al., 2020, Gondwana Research
1179	18XH16-1	129.52	44.91	0	Monzogranite	213	1	213	6.1	6.3	799	786	Long XY et al., 2020, Gondwana Research
1180	15XH5-1	129.34	45.80	0	Monzogranite	204	3	204	4.9	4.9	858	860	Long XY et al., 2020, Gondwana Research
1181	18H25-1	129.41	47.40	0	Monzogranite	202	2	202	7.9	7.8	682	689	Long XY et al., 2020, Gondwana Research
1182	18XH11-1	128.55	44.94	0	Monzogranite	192	1	192	12.4	12.4	418	417	Long XY et al., 2020, Gondwana Research
1183	18H1-1	128.18	46.92	0	Monzogranite	190	2	190	9.4	9.6	591	575	Long XY et al., 2020, Gondwana Research
1184	18XH2-1	127.69	45.39	0	Monzogranite	181	1	181	11.5	11.1	461	484	Long XY et al., 2020, Gondwana Research
1185	H15-14-1	128.70	46.96	0	Monzogranite	191	1	191	3.7	3.4	921	934	Ge MH et al., 2018, Lithos
1186	H15-16-1	129.11	47.05	0	Monzogranite	195	2	195	2.4	2.4	998	1000	Ge MH et al., 2018, Lithos
1187	H15-35-1	129.63	47.39	0	Monzogranite	220	1	220	0.0	0.2	1156	1146	Ge MH et al., 2018, Lithos
1188	17LT01-5	122.52	48.07	0	Monzogranite	125	2	150	7.7	8.7	657	595	Huang X et al., 2021, Geological Journal
1189	SM13-3	116.88	46.04	Uliastai active continental margin	Monzogranite	153	1	153	7.8	7.2	651	688	Jiang SH et al., 2016, Lithos
1190	CHY-3	128.74	48.49	0	Monzogranite	502	2	502	-1.6	-2.3	1467	1505	Fei XH et al., 2018, Lithos
1191	CHYX-2	128.75	48.48	0	Monzogranite	197	1	197	2.2	2.3	1012	1004	Fei XH et al., 2018, Lithos
1192	ECHY-3	128.74	48.82	0	Monzogranite	196	2	196	2.9	3.0	970	963	Fei XH et al., 2018, Lithos
1193	13GW272	123.04	48.56	0	Monzogranite	151	1	151	5.1	5.6	806	778	Dong Y et al., 2016, Journal of Asian Earth sciencesences.
1194	13GW280	123.23	48.64	0	Monzogranite	180	1	180	9.2	9.1	592	596	Dong Y et al., 2016, Journal of Asian Earth sciencesences
1195	D8192	120.32	44.93	0	Monzogranite	136	1	150	7.4	7.4	670	673	Liu HN et al., 2020, Minerals
1196	HD–243	123.92	51.17	0	Monzogranite	162	2	162	3.0	2.7	937	953	Liu J et al., 2014, Journal of Asian Earth sciencesences
1197	15ER562	119.94	50.85	0	Monzogranite	253	4	253	-1.1	-0.8	1244	1229	Mao AQ et al., 2021, International Geology Review
1198	FX-3	92.00	42.00	Western CAOB	Monzogranite	329	0	329	13.3	13.6	475	454	Wang YH et al., 2016, Gondwana Research
1199	14GW074	130.13	45.86	Eastern CAOB	Monzogranite	266	0	266	-2.7	-3.7	1340	1393	Yang H et al 2017, International Journal of Earth sciences
1200	09AL27	104.89	40.10	North China craton	Monzogranite	284	0	284	-11.8	-11.7	1880	1876	Dan W et al., 2014, Lithos
1201	H15-66-1	127.69	45.39	Eastern CAOB	Monzogranite	180	0	180	9.1	9.1	599	596	Ge MH et al., 2017, Lithos
1202	H15-71-1	128.50	45.08	Eastern CAOB	Monzogranite	198	0	198	9.7	9.9	578	569	Ge MH et al., 2017, Tectonophysics.
1203	H15-72-1	128.69	44.86	Eastern CAOB	Monzogranite	193	0	193	9.0	9.5	615	584	Ge MH et al., 2017, Tectonophysics.
1204	14GW375	130.00	45.50	Eastern CAOB	Monzogranite	1056	0	600	-4.9	-4.9	1668	1680	Yang H et al., 2018, Gondwana Research
1205	W8N-1	118.00	44.00	Eastern CAOB	Monzogranite	268	0	268	10.6	11.5	583	529	Guan QB 2018, Geoscience Frontiers
1206	14RF21-1	132.07	44.35	North China craton	Monzogranite	451	0	451	4.9	4.5	1051	1079	Xu T et al., 2018, Lithos
1207	14RF21-6	132.07	44.35	North China craton	Monzogranite	451	0	451	2.8	2.7	1174	1174	Xu T et al., 2018, Lithos
1208	14RF44-1	133.56	45.45	Eastern CAOB	Monzogranite	429	0	429	-2.7	-3.1	1506	1542	Xu T et al., 2018, Lithos
1209	H15-14	128.70	46.96	Eastern CAOB	Monzogranite	191	0	191	3.7	3.4	921	934	Ge MH et al., 2018, Lithos
1210	H15-16	129.11	47.05	Eastern CAOB	Monzogranite	195	0	195	2.4	2.4	998	1000	Ge MH et al., 2018, Lithos
1211	H15-35	129.63	47.39	Eastern CAOB	Monzogranite	220	0	220	0.0	0.2	1156	1146	Ge MH et al., 2018, Lithos
1212	H15-40	129.98	47.38	Eastern CAOB	Monzogranite	241	0	241	-2.0	-1.4	1288	1251	Ge MH et al., 2018, Lithos
1213	16240-1	110.55	40.95	North China craton	Monzogranite	402	0	402	-22.3	-20.7	2563	2474	Zhang QQ et al., 2018, Lithos
1214	16243-1	110.58	40.93	North China craton	Monzogranite	403	0	403	-19.7	-18.1	2417	2325	Zhang QQ et al., 2018, Lithos
1215	16245-1	110.58	40.93	North China craton	Monzogranite	402	0	402	-18.2	-17.4	2334	2283	Zhang QQ et al., 2018, Lithos
1216	15XH12-1	129.53	45.66	Eastern CAOB	Monzogranite	313	0	313	5.6	5.6	904	907	Guo P et al., 2018, Gondwana Research.
1217	13JN38-1	125.98	41.82	North China craton	Monzogranite	2501	0	600	-38.4	-38.6	3614	3627	Guo BR et al., 2018, Gondwana Research.
1218	13JN45-5	126.38	42.03	North China craton	Monzogranite	2605	0	600	-37.7	-37.9	3576	3590	Guo BR et al., 2018, Gondwana Research.
1219	14JN01-1	127.11	42.78	North China craton	Monzogranite	2497	0	600	-37.9	-37.8	3588	3581	Guo BR et al., 2018, Gondwana Research.
1220	14JN07-1	127.27	42.80	North China craton	Monzogranite	2492	0	600	-39.5	-39.8	3678	3697	Guo BR et al., 2018, Gondwana Research.
1221	RZ27	124.93	42.44	North China craton	Monzogranite	156	0	156	3.4	3.7	911	893	Liu J et al., 2018, Journal of Asian Earth sciencesences
1222	JC14-023	118.39	40.30	North China craton	Monzogranite	186	0	186	-13.2	-13.6	1883	1907	Jiang SH et al., 2018, Lithos
1223	JC14-026	118.40	40.32	North China craton	Monzogranite	214	0	214	-9.7	-9.8	1704	1706	Jiang SH et al., 2018, Lithos
1224	JD16-077	118.81	40.24	North China craton	Monzogranite	186	0	186	-15.1	-15.2	1990	1990	Jiang SH et al., 2018, Lithos
1225	JD16-083	117.64	40.31	North China craton	Monzogranite	170	0	170	-13.6	-13.7	1893	1901	Jiang SH et al., 2018, Lithos
1226	JD16-088	117.76	40.31	North China craton	Monzogranite	169	0	169	-17.1	-18.1	2088	2145	Jiang SH et al., 2018, Lithos
1227	JD16-095	118.84	40.44	North China craton	Monzogranite	223	0	223	-12.9	-13.1	1889	1898	Jiang SH et al., 2018, Lithos
1228	JD16-100	118.93	40.48	North China craton	Monzogranite	151	0	151	-16.2	-14.4	2044	1934	Jiang SH et al., 2018, Lithos
1229	JD16-102	118.91	40.46	North China craton	Monzogranite	168	0	168	-16.4	-15.6	2060	2004	Jiang SH et al., 2018, Lithos
1230	DHS-N3	124.46	50.53	Eastern CAOB	Monzogranite	159	0	159	6.6	6.6	727	726	Jiang SH et al., 2018, Lithos
1231	16GW121	129.38	44.66	Eastern CAOB	Monzogranite	887	0	600	-0.4	-0.2	1191	1163	Zhao D et al., 2018, Lithos
1232	EL14-13-1	111.34	43.26	Eastern CAOB	Monzogranite	296	0	296	5.4	5.5	859	874	Yuan L et al., 2018, Journal of Asian Earth sciencesences
1233	13GW342	121.12	48.36	Eastern CAOB	Monzogranite	312	0	312	8.3	8.2	764	757	Ji Z et al., 2018, Lithos
1234	13GW346	121.07	48.03	Eastern CAOB	Monzogranite	321	0	321	9.6	9.6	689	680	Ji Z et al., 2018, Lithos
1235	13GW353	120.55	48.07	Eastern CAOB	Monzogranite	301	0	301	6.1	6.6	880	838	Ji Z et al., 2018, Lithos
1236	SL14-004-2	91.06	41.46	Western CAOB	Monzogranite	311	0	311	14.4	14.4	396	397	Zhao LD et al., 2019, Gondwana Research.
1237	MSD09821-6.1	94.87	41.15	Western CAOB	Monzogranite	224	0	224	4.1	3.5	970	962	Li S et al., 2012, Lithos
1238	XY2-1	84.59	43.67	Western CAOB	Monzogranite	316	0	316	12.2	12.7	536	506	Xu X et al., 2010, Acta Petrologica et Mineralogica
1239	TL-42	75.67	42.67	Western CAOB	Monzogranite	427	0	427	-4.4	-4.4	1574	1569	Zhao X et al., 2015, Ore Geology Reviews.
1240	G23	89.55	46.15	Eastern Junggar	monzogranite	322	2	322	12.4	12.4	521	520	Liu W et al., 2013 Lithos
1241	3198	86.79	47.63	Chinese Altai	monzogranite	268	5	268	7.6	7.9	752	736	Tong Y et al., 2014 American Journal of Science
1242	3055	88.41	47.33	Chinese Altai	monzogranite	267	6	267	10.7	10.4	575	591	Tong Y et al., 2014 American Journal of Science
1243	3037	89.64	47.48	Chinese Altai	Monzogranite	210	0	210	1.6	1.7	1053	1049	Wang et al., 2014
1244	3033	89.64	47.50	Chinese Altai	Monzogranite	216	0	216	0.7	0.8	1110	1108	Wang et al., 2014
1245	3038	89.64	47.47	Chinese Altai	Monzogranite	213	0	213	1.5	1.4	1060	1071	Wang et al., 2014
1246	3141A	88.30	47.78	Chinese Altai	Monzogranite	151	0	151	4.4	4.1	846	861	Wang et al., 2014
1247	GEQ-4	114.49	45.02	Uliastai active continental margin	Monzogranite	307	0	307	9.1	8.6	701	726	Wang J C et al., 2016 Geoscience(ICWEA)
1248	4001.1	116.32	43.39	Uliastai active continental margin	Monzogranite	277	0	277	9.2	8.6	671	702	Li M et al., 2015, Geology Sci. Technol. Inf..(ICWEA)
1249	N14625-10.1	111.19	43.23	Baolidao arc-accretion complex	Monzogranite	279	0	279	3.7	3.8	986	979	Gou L et al., 2015, Acta Petrologica Et Mineralogica(ICWEA)
1250	BLD-3	113.59	43.66	Baolidao arc-accretion complex	Monzogranite	322	0	322	12.3	12.3	527	523	Hu CS et al., 2015 Journal of Asian Earth sciencesences
1251	SZ-4	113.86	43.83	Baolidao arc-accretion complex	Monzogranite	224	0	224	8.5	9.5	670	608	Shi YR et al., 2016 International Geology Review
1252	WL1212	117.39	44.06	Baolidao arc-accretion complex	Monzogranite	314	0	314	4.4	4.5	974	969	Wang XY, 2013 Geoscience.(ICWEA)
1253	14SJ125	119.09	44.62	Baolidao arc-accretion complex	Monzogranite	252	0	252	13.6	13.7	395	386	Liu et al., 2016
1254	GZ10-52	119.67	44.57	Baolidao arc-accretion complex	Monzogranite	137	0	150	9.5	8.9	551	585	Yang et al., 2014
1255	TW03.1	121.16	45.32	Baolidao arc-accretion complex	Monzogranite	131	0	150	7.7	8.5	658	608	Chen LL et al., 2016, Geology of China (ICWEA)
1256	W28N-1	118.61	43.99	Erdaojing accretion complex	Monzogranite	150	0	150	8.2	7.5	628	664	Feng G et al., 2015 J. Earth sciences. Environ.(ICWEA)

1257	SB0	118.97	44.33	Erdaojing accretion complex	Monzogranite	138	0	150	6.8	6.6	706	719	Shu QH et al., 2015 Lithos
1258	LJG-3	120.03	43.81	Erdaojing accretion complex	Monzogranite	241	0	241	4.6	4.6	907	907	Duan XX et al., 2015 International Geology Review
1259	GZ10-49	119.78	44.52	Erdaojing accretion complex	Monzogranite	138	0	150	8.1	7.4	632	674	Yang QD et al., 2014 Acta Petrologica Sinica(ICWEA)
1260	GZ10-57	119.53	44.16	Erdaojing accretion complex	Monzogranite	139	0	150	5.5	5.5	780	781	Yang QD et al., 2014 Acta Petrologica Sinica(ICWEA)
1261	W18N	118.52	44.04	Erdaojing accretion complex	Monzogranite	130	0	150	6.5	6.6	727	722	Li PC et al., 2016 , Earth sciences(ICWEA)
1262	11ND-03	107.06	41.12	North China Craton	Monzogranite	259	0	259	-23.7	-16.1	2532	2106	Wu YF et al., 2013, Geology Sci. Technol. Inf.(ICWEA)
1263	DQ08-100	112.06	41.02	North China Craton	Monzogranite	140	0	150	-15.2	-15.6	1969	1993	Guo L et al., 2012 International Geology Review
1264	DQ08-70	111.89	40.99	North China Craton	Monzogranite	114	0	150	-11.4	-12.5	1753	1814	Guo L et al., 2012 International Geology Review
1265	HG-05	115.74	40.94	North China Craton	Monzogranite	245	0	245	-12.1	-11.9	1869	1856	Liu XY, 2014, Master Dissertation(ICWEA)
1266	STWH1044	120.61	40.87	North China Craton	Monzogranite	185	0	185	-8.1	-7.9	1590	1579	Cui FH., 2015 doctoral thesis(ICWEA)
1267	STWH1055	120.38	40.70	North China Craton	Monzogranite	176	0	176	-12.6	-12.8	1840	1850	Cui FH., 2015 doctoral thesis(ICWEA)
1268	STWH1069	120.69	40.92	North China Craton	Monzogranite	184	0	184	-9.7	-9.4	1682	1668	Cui FH., 2015 doctoral thesis(ICWEA)
1269	STWH1070	120.68	40.92	North China Craton	Monzogranite	169	0	169	-10.1	-10.5	1693	1718	Cui FH., 2015 doctoral thesis(ICWEA)
1270	STTW.1	120.70	40.72	North China Craton	Monzogranite	156	0	156	-22.1	-21.9	2368	2354	Cui FH., 2015 doctoral thesis(ICWEA)
1271	STWH1017	120.52	40.60	North China Craton	Monzogranite	139	0	150	-23.0	-23.2	2412	2422	Cui FH., 2015 doctoral thesis(ICWEA)
1272	A001-1	120.57	40.37	North China Craton	Monzogranite	154	0	154	-38.7	-35.9	3295	3144	Li J, 2009 Master Dissertation(ICWEA)
1273	PA09-3	121.88	42.51	North China Craton	Monzogranite	238	0	238	5.7	5.4	842	856	Zhang XH et al., 2012 Lithos
1274	HTY016	91.70	37.20	NW China	monzogranite	230	3.7	230	0.1	0.0	1156	1164	Qu HY et al., 2019 Ore Geology Reviews
1275	HTY019	91.75	37.25	NW China	monzogranite	222	1.3	222	0.7	0.7	1118	1118	Qu HY et al., 2019 Ore Geology Reviews
1276	07A001	91.69	39.17	NW China	monzogranite	514	5.6	514	-9.2	-9.0	1909	1898	Meng LT et al.2017, Lithos
1277	11GW011	131.73	45.51	NE China	monzogranite	498	3	498	-2.1	-2.7	1494	1525	Yang H et al.2014, Lithos
1278	11GW039	131.72	46.55	Jiamusi Block	monzogranite	506	3	506	0.5	0.4	1353	1358	Bi JH et al., 2014, Journal of Asian Earth sciencesences
1279	12GW032	131.89	46.30	Jiamusi Block	monzogranite	488	3	488	-0.5	-0.5	1394	1393	Bi JH et al., 2014, Journal of Asian Earth sciencesences
1280	11GW029	131.96	46.15	Jiamusi Block	monzogranite	488	3	488	0.4	0.4	1340	1344	Bi JH et al., 2014, Journal of Asian Earth sciencesences
1281	15GW075	130.65	46.40	Jiamusi Block	Monzogranite	272	2	272	-1.4	-1.0	1277	1255	Dong Y et al., 2017 Gondwana Research
1282	15GW248	130.63	46.63	Jiamusi Block	Monzogranite	267	3	267	-4.6	-4.5	1453	1450	Dong Y et al., 2017 Gondwana Research
1283	15GW261	130.65	46.53	Jiamusi Block	Monzogranite	263	3	263	-3.9	-4.0	1410	1418	Dong Y et al., 2017 Gondwana Research
1284	15GW265	130.64	46.51	Jiamusi Block	Monzogranite	266	2	266	-2.8	-3.2	1351	1374	Dong Y et al., 2017 Gondwana Research
1285	PDS5-1	129.93	48.39	Jiamusi Block	Monzogranite	273	2	273	-0.9	-0.6	1247	1230	Bao ZY et al., 2014 , Acta Geology Sin.
1286	PDS7-1	129.93	48.39	Jiamusi Block	Monzogranite	250	3	250	-2.9	-2.7	1347	1332	Bao ZY et al., 2014 , Acta Geology Sin.
1287	11GW041	131.71	47.08	Jiamusi Block	Monzogranite	261	3	261	2.4	3.3	1050	994	Bi JH et al. 2014, Journal of Asian Earth sciencesences
1288	11HNA4	128.72	44.10	LXZR	Monzogranite	255	2	255	6.7	6.2	795	827	Yu et al., 2013 Gondwana Research
1289	H15-59-01	128.68	46.56	LXZR	Monzogranite	182	1	182	3.6	3.6	915	919	Ge MH et al., 2019 Lithosphere
1290	1076-1	127.23	48.73	LXZR	Monzogranite	187	2	187	7.8	8.1	677	659	Gou J et al., 2013 Journal of Jilin University (Earth sciencesence Edition),
1291	LM-0101	128.53	47.37	LXZR	Monzogranite	181	2	181	3.4	3.3	928	936	Hu XL et al., 2014 Journal of Jilin University (Earth sciencesence Edition),
1292	XLJG-011	128.47	47.29	LXZR	Monzogranite	181	1	181	2.3	2.2	993	1000	Hu XL et al., 2014, Journal of Asian Earth sciencesences
1293	XLJG-061	128.47	47.29	LXZR	Monzogranite	180	1	180	2.1	2.1	1000	1000	Hu XL et al., 2014, Journal of Asian Earth sciencesences
1294	15XH23–1	128.54	44.03	LXZR	Monzogranite	262	2	262	2.7	2.7	1030	1034	Long X et al., 2019, Geological Journal
1295	15XH25–1	128.51	44.07	LXZR	Monzogranite	263	4	263	1.4	1.5	1108	1105	Long X et al., 2019, Geological Journal
1296	STH-1	128.67	44.87	LXZR	Monzogranite	201	2	201	12.3	12.0	430	447	Qin JF et al., 2016 , Lithosphere
1297	HDL10-4	129.49	45.68	LXZR	Monzogranite	425	3	425	1.3	1.0	1240	1259	Wang F et al., 2012 , Tectonophysics
1298	14HYC18-1	130.01	47.21	LXZR	Monzogranite	505	2	505	-5.8	-5.7	1711	1706	Wang ZW et al., 2016 Lithos
1299	14HT2-1	128.47	46.94	LXZR	Monzogranite	496	3	496	-2.0	-2.1	1486	1492	Wang ZW et al., 2016 Lithos
1300	14HT6-1	128.85	46.96	LXZR	Monzogranite	486	5	486	0.5	0.8	1333	1320	Wang ZW et al., 2016 Lithos
1301	14HT9-1	129.14	47.64	LXZR	Monzogranite	469	2	469	-1.0	-0.9	1410	1402	Wang ZW et al., 2016 Lithos
1302	14HT10-1	129.14	47.86	LXZR	Monzogranite	471	2	471	-1.4	-1.4	1432	1430	Wang ZW et al., 2016 Lithos
1303	14HYC2-1	129.51	46.83	LXZR	Monzogranite	464	4	464	2.8	2.6	1184	1197	Wang ZW et al., 2016 Lithos
1304	14HYC4-1	129.52	46.85	LXZR	Monzogranite	476	3	476	0.7	0.7	1318	1315	Wang ZW et al., 2016 Lithos
1305	14HYC5-1	129.52	46.85	LXZR	Monzogranite	470	4	470	0.8	0.8	1305	1305	Wang ZW et al., 2016 Lithos
1306	14HYC6-1	130.14	47.20	LXZR	Monzogranite	475	4	475	0.9	0.8	1304	1306	Wang ZW et al., 2016 Lithos
1307	LM1	128.68	47.33	LXZR	Monzogranite	183	2	183	2.7	2.5	973	980	Cheng GH et al., 2015, Acta Petrologica Sinica
1308	LM2	128.68	47.33	LXZR	Monzogranite	187	2	187	2.8	2.9	966	962	Cheng GH et al., 2015, Acta Petrologica Sinica
1309	1009-1	129.79	47.58	LXZR	Monzogranite	261	1	261	1.5	1.4	1100	1104	Wei HY et al., 2012 Earth sciencesence Journal of China University of Geosciences
1310	P18-9	129.82	47.41	LXZR	Monzogranite	260	1	260	1.3	0.4	1114	1162	Wei HY et al., 2012 Earth sciencesence Journal of China University of Geosciences
1311	P31-4	129.61	47.35	LXZR	Monzogranite	211	1	211	-3.6	-4.4	1357	1402	Wei HY et al., 2012 Earth sciencesence Journal of China University of Geosciences
1312	1015-1	129.30	47.49	LXZR	Monzogranite	212	2	212	0.7	0.3	1109	1131	Wei HY et al., 2012 Earth sciencesence Journal of China University of Geosciences
1313	11RJ-11	118.38	43.41	Erdaojing accretion complex	Monzogranite	126	1	150	3.0	2.9	924	934	Li S et al., 2013, Journal of Asian Earth sciencesences
1314	G0211-2	121.39	46.25	Hegenshan ophiolite-arc-accretion complex	Monzogranite	175	0	175	10.9	10.9	490	491	Zhou Y , 2011 Acta Petrologica Et Mineralogica(ICWEA)
1315	14SJ92	119.06	44.62	Baolidao arc-accretion complex	Monzogranite	254	0	254	13.1	13.2	426	420	Liu et al., 2016
1316	TGRG-4	107.54	42.18	Northern margin of the North China Craton	Monzogranite	268	0	268	2.1	1.7	1069	1093	Wang YD, 2016 doctoral thesis(ICWEA)
1317	NM07-156	110.89	41.07	North China Craton	Monzogranite	282	0	282	-10.6	-10.8	1808	1821	Zhang XH et al., 2011 Lithos
1318	HFG-2	117.27	41.17	North China Craton	Monzogranite	254	0	254	-11.0	-10.8	1808	1801	Zhang SH et al., 2009 Geological Society of America Bulletin
1319	STWH1050	120.35	40.73	North China Craton	Monzogranite	186	0	186	-10.2	-10.3	1714	1720	Cui FH., 2015 , doctoral thesis(ICWEA)
1320	STWH1068	120.68	40.95	North China Craton	Monzogranite	177	0	177	-10.2	-10.4	1705	1719	Cui FH., 2015 , doctoral thesis(ICWEA)
1321	STWH10	120.68	40.71	North China Craton	Monzogranite	157	0	157	-20.8	-22.0	2295	2363	Cui FH., 2015 , doctoral thesis(ICWEA)
1322	YC32	119.03	43.53	Erdaojing accretion complex	Monzogranite	137	0	150	5.3	5.2	792	798	Shu QH et al., 2015 Lithos
1323	11HB17	109.37	40.70	North China Craton	Monzogranite	328	0	328	-5.9	-6.0	1578	1584	Wang L, 2015 Acta Petrologica Sinica(ICWEA)
1324	09032-1	119.03	40.65	North China Craton	Monzogranite	224	0	224	-11.5	-11.6	1817	1820	Ye H, 2014 , Earth sciences. Frontiers(ICWEA)
1325	L1301	121.51	41.26	North China Craton	Monzogranite	124	0	150	-23.7	-23.7	2454	2450	Li G, 2016, Acta Petrologica Sinica(ICWEA)
1326	CM38	113.87	40.81	North China Craton	Monzogranite porphyry	145	0	150	-16.3	-16.3	2034	2029	Wu G et al., 2016 Ore Geology Reviews
1327	KKS-35	81.90	44.70	0	Monzogranitic porphyry	306	1	306	3.5	5.4	1022	910	Zhang DY et al., 2012, Lithos
1328	TBD05	119.20	43.72	Erdaojing accretion complex	Monzogranitic porphyry	137	0	150	2.9	2.6	932	951	Zhou ZH et al., 2015 Journal of Asian Earth sciencesences
1329	KK4-1	82.04	42.87	Western CAOB	Monzon Granite	352	9	352	0.5	0.3	1232	1241	Xu XY et al., 2010, Acta Petrologica et Mineralogica
1330	TL1-2	83.08	43.08	Western CAOB	Monzon Granite	366	11	366	-6.0	-4.0	1612	1496	Xu XY et al., 2010, Acta Petrologica et Mineralogica
1331	11ER21-1	119.82	51.22	0	Monzonite	155	1	155	1.0	1.0	1048	1048	Tang J et al., 2015, Tectonophysics
1332	11ER22-4	119.82	51.23	0	Monzonite	155	1	155	2.0	2.0	987	987	Tang J et al., 2015, Tectonophysics
1333	BA11-91	96.80	41.86	Western CAOB	Monzonite	421	0	421	-3.4	-2.8	1509	1472	Zheng RG et al., 2018, Journal of Asian Earth sciencesences
1334	X10-77-2	98.41	41.95	Eastern CAOB	Monzonite	428	0	428	-7.2	-6.8	1727	1707	Yuan Y et al., 2018, Lithos
1335	07053-1	113.14	41.60	North China craton	Monzonite	360	0	360	-20.0	-20.5	2417	2447	Zhang QQ et al., 2018 Lithos
1336	07169-1	110.55	40.94	North China craton	Monzonite	388	0	388	-28.2	-27.3	2892	2840	Zhang QQ et al., 2018 Lithos
1337	16200-1	113.17	41.59	North China craton	Monzonite	381	0	381	-20.6	-20.3	2455	2436	Zhang QQ et al., 2018 Lithos
1338	16206-1	112.96	41.55	North China craton	Monzonite	399	0	399	-19.6	-20.1	2410	2443	Zhang QQ et al., 2018 Lithos
1339	JD16-061	118.20	40.28	North China craton	Monzonite	203	0	203	-8.3	-8.9	1615	1645	Jiang SH et al., 2018, Lithos
1340	JD16-072	117.89	40.29	North China craton	Monzonite	137	0	150	-19.5	-19.1	2213	2190	Jiang SH et al., 2018, Lithos

1341	TS11-70	84.20	43.10	Western CAOB	Monzonite	419	0	419	5.1	5.4	1023	1001	Wang M 2016, Journal of Asian Earth sciencesences
1342	XSHT202	115.16	40.96	North China Craton	Monzonite	373	0	373	-10.7	-10.8	1889	1892	Li CM et al., 2014 Acta Petrologica Sinica(ICWEA)
1343	14HT17–1	129.74	48.14	LXZR	Monzonite	259	1	259	-1.8	-2.1	1291	1306	Long X et al., 2019 Geological Journal
1344	14HT3-1	128.51	46.95	LXZR	monzonite	491	3	491	-1.1	-1.1	1433	1432	Wang ZW et al., 2016 Lithos
1345	NLKB14-10	82.48	43.76	0	Monzonite porphyry	296	3	296	11.6	11.9	547	530	Huang H et al., 2020, Earth-Science Reviews
1346	HSD-03	119.38	44.62	0	Monzonite porphyry	140	2	150	7.0	7.2	694	683	Liu LJ et al., 2021, Lithos
1347	CWL-72	76.87	40.92	0	onite-Bt monzonite-Bt ada	286	3	286	1.7	1.9	1109	1099	Huang et al., 2012, Lithos
1348	06XJ121-2	84.84	45.72	Western Junggar	monzonitic enclave	309	3	309	14.8	15.1	367	353	Tang et al., 2012 Lithos
1349	Hq12	115.24	41.04	North China Craton	Monzonitic gneiss	300	0	300	-11.2	-9.5	1858	1761	Wang F et al., 2011 Gondwana Research
1350	MG	105.78	34.00	the Qinling	monzonitic granite	212	0	212	-2.0	-0.4	1264	1174	Qin JF et al., 2009 Lithos
1351	ME	105.87	34.33	the Qinling	monzonitic granite	212	0	212	1.2	0.6	1081	1116	Qin JF et al., 2009 Lithos
1352	G0206-2	122.68	46.60	Hegenshan ophiolite-arc-accretion complex	Monzonitic granite porphyr	130	0	150	7.8	7.6	649	663	Zhou Y 2011, Acta Petrologica Et Mineralogica(ICWEA)
1353	XH08-3	90.40	45.43	yemaquan	monzonitic granite	298	0	298	12.5	12.5	492	493	Gan et al., 2010
1354	10XJ-107B	92.71	43.84	guaishishan	monzonitic granite	444	0	444	11.9	11.7	649	656	Li, 2012
1355	11K68	86.25	41.81	0	Monzoranite	417	4	417	-8.3	-3.4	1777	1506	Ge RF et al., 2014, Tectonics
1356	11K83	86.24	41.81	0	Monzoranite	420	3	420	-2.9	-4.5	1477	1567	Ge RF et al., 2014, Tectonics
1357	H710	95.02	44.09	balegantinghaer	moyite	309	0	309	12.5	12.4	501	508	Zhao, 2014
1358	11HSW7-2	127.33	49.99	0	Muscovite granite	168	1	168	9.1	9.2	589	581	Li, Y et al., 2015, Acta Petrologica Sinica(ICWEA)
1359	10SYQ06	97.07	42.00	Beishan	mylonitic granite	448	0	448	-3.2	-1.4	1517	1413	Song D et al., 2013, Precambrian Research
1360	10SYQ08	96.99	42.01	Beishan	mylonitic granite	474	0	474	-6.2	-6.3	1710	1715	Song D et al., 2013, Precambrian Research
1361	W8034	80.92	42.45	0	Mylonitic monzonite	453	4	453	-4.2	-4.2	1579	1577	Gou LL and Zhang LF, 2016, Journal of Asian Earth sciencesences
1362	KZ	87.00	47.00	NW China	norite	278	0	278	9.6	9.9	644	630	Tang DM et al.2017, Lithos
1363	YKH10	90.03	46.60	Eastern Junggar	ore-bearing diorite porphyr	379	2.1	379	13.5	14.0	500	470	Yang FQ et al., 2014 Lithos
1364	10SYQ06-	96.93	42.04	Beishan	paragneiss	415	0	415	-11.8	-12.6	1983	2028	Song D et al., 2013, Precambrian Research
1365	BU9-1	87.00	48.06	chonghuer	pegmatite vein	281	0	281	2.9	2.3	1035	1073	Sun et al., 2009
1366	BW10	113.65	44.43	Hegenshan ophiolite-arc-accretion complex	Peralkaline granite	289	0	289	14.6	13.9	366	405	Shi et al., 2016 International Geology Review
1367	G44	89.45	46.14	Eastern Junggar	peralkaline granite	325	2	325	13.2	13.5	476	461	Liu Wei et al., 2013 Lithos
1368	G71	89.21	46.30	Eastern Junggar	peralkaline granite	322	2.2	322	14.3	14.4	412	403	Liu Wei et al., 2013 Lithos
1369	SR01	90.31	45.14	Eastern Junggar	peralkaline granite	301	2.4	301	13.3	12.9	453	474	Liu Wei et al., 2013 Lithos
1370	13GF-111	130.07	43.67	0	Plagiogranite	260	3	260	10.0	10.1	607	601	Guo F et al., 2019, Chemical Geology
1371	08Zhr-2	89.05	46.55	Eastern Junggar	lagiogranite (albite granite	495	11	495	11.7	12.0	697	678	Zeng Lingjun et al., 2015 Journal of Asian Earth sciencesences
1372	11Zhr-3-3	89.05	46.55	Eastern Junggar	Plagiogranite (albitite)	498	5.8	498	14.5	14.4	541	543	Zeng Lingjun et al., 2015 Journal of Asian Earth sciencesences
1373	ZK44	117.48	44.11	0	Porphyric K-feldspar granit	138	1	150	6.9	6.6	701	718	Wu, G. et al., 2021, Acta Petrologica Sinica(ICWEA)
1374	ZK46	117.48	44.11	0	Porphyric K-feldspar granit	137	1	150	6.0	5.8	751	764	Wu, G. et al., 2021, Acta Petrologica Sinica(ICWEA)
1375	CXWG67-1a	117.97	44.06	0	Porphyritic Bt syenogranite	146	2	150	9.9	10.0	528	520	Shang, Y. et al., 2021, Geology in China(ICWEA)
1376	ECH58-58	128.74	48.49	0	porphyritic granite	501	2	501	5.5	5.3	1061	1073	Fei XH et al., 2018, Lithos
1377	ECH58-57	128.74	48.48	0	Porphyritic granite	194	3	194	6.0	6.2	785	777	Fei XH et al., 2018, Lithos
1378	B13	119.15	49.98	0	Porphyritic granite	237	1	237	6.0	6.2	822	810	Kang YJ et al., 2018, Ore Geology Reviews
1379	B14	119.16	49.98	0	Porphyritic granite	241	1	241	3.8	4.5	955	915	Kang YJ et al., 2018, Ore Geology Reviews
1380	DYQ1	118.21	46.03	Uliastai active continental margin	Porphyritic granite	159	0	159	7.6	7.8	669	654	Wang et al., 2016 Ore Geology Reviews
1381	DYQ2	118.21	46.03	Uliastai active continental margin	Porphyritic granite	158	0	158	6.5	6.7	729	717	Wang et al., 2016 Ore Geology Reviews
1382	DSM-2	107.06	41.12	North China Craton	Porphyritic granite	284	0	284	-11.7	-12.8	1876	1938	Hu CS et al., 2015 International Geology Review
1383	YLKZK1204-9	90.04	46.61	Eastern Junggar	porphyritic monzonite	374	1	374	12.3	12.8	567	541	Yang FQ et al., 2014 Lithos
1384	CHY-2	128.74	48.49	0	orphyritic quartz monzoni	197	3	197	2.2	2.8	1009	976	Fei XH et al., 2018, Lithos
1385	DQ09819-8	95.35	41.28	Western CAOB	Porphyritic Quartz Syenite	225	0	225	1.6	1.8	1069	1057	Li S et al., 2012, Lithos
1386	MP-1	114.00	38.78	the central North China Craton	porphyry	124	1	150	-14.9	-15.0	1956	1959	Li Q et al.2015, Ore Geology Reviews
1387	ZT-1	113.80	38.61	the central North China Craton	porphyry	129	2	150	-14.8	-14.9	1950	1951	Li Q et al.2015, Ore Geology Reviews
1388	ZT-2	113.80	38.61	the central North China Craton	porphyry	129	2.8	150	-17.4	-16.5	2093	2042	Li Q et al.2015, Ore Geology Reviews
1389	ZTX-2	113.68	38.63	the central North China Craton	porphyry	129	3.2	150	-16.8	-16.4	2063	2037	Li Q et al.2015, Ore Geology Reviews
1390	ZTX-5	113.74	38.72	the central North China Craton	porphyry	123	1.6	150	-22.4	-22.2	2480	2367	Li Q et al.2015, Ore Geology Reviews
1391	TG1	88.15	43.80	CAOB	Porphyry	303	3	303	7.7	7.4	774	792	Zhang XB et al., 2020, Minerals
1392	13GF-42	129.16	42.18	0	Quartz diorite	246	3	246	-9.2	-9.3	1700	1705	Guo F et al., 2019, Chemical Geology
1393	13GF-77	130.05	42.97	0	Quartz diorite	215	2	215	11.4	11.5	490	485	Guo F et al., 2019, Chemical Geology
1394	13GW273	123.17	48.61	0	Quartz diorite	210	2	210	9.1	9.5	621	598	Yang H et al., 2016, Journal of Asian Earth sciencesences
1395	13GW010	131.24	44.99	0	Quartz diorite	204	2	204	4.2	4.2	900	901	Yang H et al., 2015, Lithos
1396	TW28	120.26	45.15	0	Quartz diorite	136	1	150	9.1	9.2	575	569	Liu HN et al., 2020, Minerals
1397	ZS2-3	81.26	42.75	Western CAOB	Quartz Diorite	402	0	402	8.7	8.7	814	813	Xu X et al., 2010, Acta Petrologica Et Mineralogica
1398	YLK28	90.02	46.60	Eastern Junggar	quartz diorite	382	0	382	13.1	13.0	527	533	Yang FQ et al., 2014 Lithos
1399	D012-2TN	106.47	41.27	Northern margin of the North China Craton	Quartz diorite	338	0	338	-1.2	-1.4	1316	1329	Liu Min et al., 2016 Journal of Asian Earth sciencesences
1400	08480-1	110.61	41.81	Northern margin of the North China Craton	Quartz diorite	436	0	436	-2.2	-2.1	1448	1445	Zhang SH et al., 2014 Geological Society of America Bulletin
1401	08557-1	109.90	42.00	Northern margin of the North China Craton	Quartz diorite	445	0	445	8.1	7.8	867	885	Zhang SH et al., 2014 Geological Society of America Bulletin
1402	HLB-G	117.34	41.07	North China Craton	Quartz diorite	302	0	302	-25.6	-21.8	2676	2461	Zhang SH et al., 2009 Geological Society of America Bulletin
1403	BLN-5-2	117.35	41.12	North China Craton	Quartz diorite	296	0	296	-19.0	-18.7	2299	2278	Ma X et al., 2012, Earth sciences(ICWEA)
1404	STWH1053	120.36	40.72	North China Craton	Quartz diorite	194	0	194	-9.2	-8.9	1663	1643	Cui F H , 2015, doctoral thesis(ICWEA)
1405	15JL02B	126.29	43.68	CAOB	quartz diorite	476	2	476	14.8	13.9	503	554	Ma HT et al., 2020 Journal of Asian Earth Sciences
1406	07130-1	110.21	41.90	Northern margin of the North China Craton	Quartz diorite	453	0	453	4.4	4.4	1084	1085	Zhang SH et al., 2014 Geological Society of America Bulletin
1407	08404-1	112.72	42.28	Northern margin of the North China Craton	Quartz diorite	439	0	439	4.7	4.8	1054	1051	Zhang SH et al., 2014 Geological Society of America Bulletin
1408	08409-1	112.61	42.21	Northern margin of the North China Craton	Quartz diorite	430	0	430	1.6	1.6	1227	1229	Zhang SH et al., 2014 Geological Society of America Bulletin
1409	DSM-6	107.05	41.11	North China Craton	Quartz diorite	275	0	275	-14.1	-14.5	2004	2025	Hu CS et al., 2015 International Geology Review
1410	SD020-3	117.78	41.33	North China Craton	Quartz diorite	311	0	311	-18.9	-16.2	2300	2149	Zhang SH et al., 2009 Geological Society of America Bulletin
1411	D018-1	117.64	41.27	North China Craton	Quartz diorite	324	0	324	-16.5	-9.6	2173	1784	Zhang SH et al., 2009 Geological Society of America Bulletin
1412	D077-1	116.97	40.99	North China Craton	Quartz diorite	288	0	288	-14.8	-14.8	2053	2055	Zhang SH et al., 2009a Int J Earth sciences (Geol Rundsch)
1413	STTW.8	120.47	40.80	North China Craton	Quartz diorite	228	0	228	-4.6	-4.5	1425	1417	Cui et al., 2015
1414	GY15	110.36	41.01	North China Craton	Quartz diorite	272	0	272	-14.6	-14.9	2032	2047	Zhang WH et al., 2012 , Geology of China(ICWEA)
1415	D169-2	117.45	41.24	North China Craton	Quartz diorite	314	0	314	-7.4	-5.4	1652	1538	Zhang SH et al., 2009 Geological Society of America Bulletin
1416	06XJ	84.45	45.50	Western Junggar	quartz dioritic porphyry	315	0	315	14.6	14.5	384	389	Tang GJ et al, 2010, Chemical Geology
1417	BY06-16	109.55	41.79	North China Craton	Quartz diotite	265	0	265	-15.3	-15.2	2063	2058	Luo H L, 2013 Acta Geology Sin. (ICWEA)
1418	17LT01-6	122.52	48.07	0	Quartz monzodiorite	125	1	150	3.1	2.8	919	937	Huang X et al., 2021, Geological Journal
1419	15ER186-6	120.07	51.01	0	Quartz Monzodiorite	253	3	253	1.6	1.5	1088	1093	Mao AQ et al., 2021, International Geology Review
1420	15XH21–1	128.63	44.01	LXZR	Quartz monzodiorite	267	2	267	3.5	3.7	988	978	Long X et al., 2019 Geological Journal
1421	14HYC16	130.14	47.20	LXZR	Quartz monzodiorite	468	3	468	-6.7	-6.7	1730	1733	Wang ZW et al., 2016 Lithos
1422	STTW.15	120.82	40.48	North China Craton	Quartz monzodiorite	121	0	150	-12.2	-11.9	1800	1784	Cui FH, 2015, doctoral thesis(ICWEA)
1423	W8026	80.78	42.51	Western CAOB	Quartz Monzonite	294	2	294	0.2	0.0	1204	1210	Gou LL et al., 2012, Lithos
1424	D1011	80.67	42.52	0	Quartz monzonite	446	2	446	-2.6	-2.3	1480	1465	Gou LL and Zhang LF, 2016, Journal of Asian Earth sciencesences

1425	D1021	80.65	42.53	0	Quartz monzonite	443	3	443	-2.3	-2.4	1458	1470	Gou LL and Zhang LF, 2016, Journal of Asian Earth sciencesences
1426	12ER35-1	121.56	53.22	0	Quartz monzonite	156	2	156	3.1	3.1	924	927	Tang J et al., 2015, Tectonophysics
1427	13ER16-1	121.50	52.16	0	Quartz monzonite	155	1	155	2.6	2.7	952	947	Tang J et al., 2015, Tectonophysics
1428	1624-1	121.50	52.23	0	Quartz monzonite	156	2	156	3.1	2.9	929	935	[illegible] et al., 2013, International Geology Review; Gou J et al., 2019, International Geology Review
1429	1558-3	124.34	51.59	0	Quartz monzonite	135	3	150	4.7	5.2	828	801	Sun RJ et al., 2016, Global Geology(ICWEA)
1430	1561-2	124.38	51.63	0	Quartz monzonite	141	2	150	4.9	5.2	815	799	Sun RJ et al., 2016, Global Geology(ICWEA)
1431	1562-2	124.39	51.64	0	Quartz monzonite	124	2	150	5.3	5.4	793	787	Sun RJ et al., 2016, Global Geology(ICWEA)
1432	18XH3-1	128.19	45.03	0	Quartz monzonite	196	2	196	12.6	13.2	405	374	Long XY et al., 2020, Gondwana Research
1433	18XH10-1	128.54	44.84	0	Quartz monzonite	187	1	187	12.9	13.3	382	360	Long XY et al., 2020, Gondwana Research
1434	TG-2	107.56	42.17	Northern margin of the North China Craton	Quartz monzonite	297	0	297	1.9	2.0	1105	1101	Wang Yingde et al., 2015 Geology Mag
1435	NM96	113.16	41.60	North China Craton	Quartz monzonite	382	0	382	-20.7	-20.9	2458	2470	Huang Dingling et al., 2017 GF
1436	ZLG08	88.95	46.80	NW China	quartz monzonite	331	0	331	12.8	13.0	505	494	Liu XJ et al., 2019 Lithos,
1437	14HT20–1	129.99	48.40	LXZR	Quartz monzonite	258	2	258	-1.5	-1.4	1269	1267	Long X et al., 2019 Geological Journal
1438	15XH22–1	128.64	43.99	LXZR	Quartz monzonite	267	2	267	2.9	2.5	1024	1046	Long X et al., 2019 Geological Journal
1439	15XH26–1	128.49	44.10	LXZR	Quartz monzonite	262	3	262	2.7	2.6	1034	1038	Long X et al., 2019 Geological Journal
1440	14HT4-1	128.63	46.92	LXZR	Quartz monzonite	491	2	491	1.1	1.0	1306	1309	Wang Z W et al., 2016 Lithos
1441	14HT5-1	128.63	46.92	LXZR	Quartz monzonite	488	2	488	0.1	0.1	1359	1361	Wang Z W et al., 2016 Lithos
1442	14HT7-1	128.95	46.99	LXZR	Quartz monzonite	488	3	488	0.4	0.4	1341	1341	Wang Z W et al., 2016 Lithos
1443	14HT13-1	129.26	48.08	LXZR	Quartz monzonite	472	2	472	-0.7	-0.7	1395	1395	Wang Z W et al., 2016 Lithos
1444	14HT14-1	129.25	48.01	LXZR	Quartz monzonite	472	2	472	-0.3	-0.1	1370	1360	Wang Z W et al., 2016 Lithos
1445	ZK809-1-1	117.49	44.08	0	Quartz porphyry	138	1	150	4.3	3.8	854	882	Yang F et al., 2019, Minerals.
1446	ZK809-2-1	117.49	44.08	0	Quartz porphyry	139	1	150	4.5	4.5	840	839	Yang F et al., 2019, Minerals.
1447	HD–47	123.92	51.18	0	Quartz porphyry	148	2	150	7.0	7.1	698	689	Liu J et al., 2014, Journal of Asian Earth sciencesences.
1448	ED10	120.76	50.79	0	Quartz Porphyry	134	1	150	6.4	6.3	733	738	Xu ZT et al., 2020, Minerals.
1449	BY-246	116.59	44.55	0	Quartz Porphyry	142	1	150	10.3	10.2	506	511	Yao, L et al., 2017, Acta Petrologica Sinica.(ICWEA)
1450	BY-20	116.59	44.55	0	Quartz Porphyry	140	1	150	9.9	9.9	525	527	Yao, L et al., 2017, Acta Petrologica Sinica.(ICWEA)
1451	YKH07	90.04	46.60	Eastern Junggar	quartz porphyry dyke	348	1.3	348	16.5	17.2	348	348	Yang Fuquan et al., 2014 Lithos
1452	2010HLJIII-1	77.18	40.35	0	Quartz syenite	269	2	269	4.4	3.8	940	977	Zhang and Zou, 2013 Lithos
1453	B80625-6	95.36	41.31	Western CAOB	Quartz Syenite	221	0	221	1.9	1.7	1045	1058	Li S et al.2012, Lithos
1454	JN07-19	112.92	41.55	North China Craton	Quartz syenite	409	0	409	-29.4	-29.6	2971	2979	Zhang XH, 2010, JGS
1455	06JH62	117.17	41.63	North China Craton	Quartz syenite	246	0	246	-10.6	-10.5	1783	1778	Yang JH et al., 2012 Chemical Geology
1456	11GW010	131.82	45.58	NE China	quartz syenite	516	7	516	0.0	1.1	1388	1327	Yang H et al., 2014 Lithos
1457	12GW031	131.48	45.52	NE China	quartz syenite	513	6	513	1.8	2.2	1282	1260	Yang H et al., 2014 Lithos
1458	06JH126	118.17	41.30	North China Craton	Quartz syenite	254	0	254	-11.1	-11.0	1818	1811	Yang JH et al., 2012 Chemical Geology
1459	STTW.16	120.82	40.48	North China Craton	Quartz syenite	125	0	150	-13.9	-14.1	1899	1908	Cui F H, 2015, doctoral thesis(ICWEA)
1460	WJ1133	86.27	46.69	0	Quartz syenite porhyry	297	2	297	12.0	12.1	526	515	Yin et al., 2016 AGS
1461	WJ1135	86.43	46.73	0	Quartz syenite porhyry	283	2	283	11.6	11.5	537	540	Yin et al., 2016 AGS
1462	DYS1	125.10	43.58	0	Quartz trachyte	254	3	254	3.2	2.7	998	1025	Song ZG et al., 2018, Gondwana Research
1463	11ER28-1	120.58	51.33	0	Quartz-diorite	242	3	242	2.5	2.5	1028	1031	Tang J et al., 2014, Lithos
1464	XW15-24-2	117.53	44.11	Baolidao arc-accretion complex	Quartz-diorite	319	0	319	1.4	1.6	1151	1138	Liu JF, 2009, doctoral thesis(ICWEA)
1465	H09-33-01	113.63	42.31	Northern margin of the North China Craton	Quartz-diorite	270	0	270	2.1	2.2	1073	1066	Hao BW et al, 2011, doctoral thesis(ICWEA)
1466	14XL02.1	115.65	44.00	Baolidao arc-accretion complex	Quartz-diorite	474	0	474	-10.8	-11.5	1969	2009	Wang SQ, 2016 Geoscience(ICWEA)
1467	WL89	117.39	44.06	Baolidao arc-accretion complex	Quartz-diorite	308	0	308	4.1	4.0	985	992	Wamg XY, 2013 Geoscience (ICWEA)
1468	WL119	117.39	44.06	Baolidao arc-accretion complex	Quartz-diorite	298	0	298	0.9	0.8	1166	1173	Wamg XY, 2013 Geoscience(ICWEA)
1469	p01-b6-1	117.55	44.13	Baolidao arc-accretion complex	Quartz-diorite	330	0	330	10.2	10.2	655	652	Zhao HT, 2014 , Master Dissertation(ICWEA)
1470	H703	95.04	44.08	qiongheba	quartz-monzonite	354	0	354	12.6	12.2	535	556	Zhao, 2014
1471	MTSL-1	86.12	42.85	0	Qz diorite	346	2	346	3.4	3.6	1056	1044	Huang H et al., 2015, Acta Geology Sin. (ICWEA)
1472	10LF63	115.00	41.00	North China craton	Rhyodacite	2502	0	600	-39.7	-39.7	3687	3690	Liou P et al., 2017, Lithos
1473	10LF64	115.00	41.00	North China craton	Rhyodacite	2442	0	600	-39.5	-39.5	3677	3676	Liou P et al., 2017, Lithos
1474	XJ045-2	85.35	42.18	0	Rhyolite	404	2	404	6.2	6.0	941	953	Lin W et al., 2013, Lithosphere
1475	not given	81.22	42.94	0	Rhyolite	340	4	340	3.3	3.2	1060	1067	Wang ZP et al., 2018, Geological Journal
1476	C14BL01	81.85	45.17	0	Rhyolite	301	5	301	7.9	7.9	766	764	Wang XS et al., 2018, Journal of Asian Earth sciencesences
1477	C14BL16	81.85	45.17	0	Rhyolite	299	2	299	6.7	6.5	834	841	Wang XS et al., 2018, Journal of Asian Earth sciencesences
1478	C14BL27	81.55	45.18	0	Rhyolite	303	1	303	7.8	8.1	772	751	Wang XS et al., 2018, Journal of Asian Earth sciencesences
1479	MZ13-16	82.54	42.30	0	Rhyolite	281	1	281	-4.4	-4.1	1454	1436	Cheng ZG et al., 2017, Lithos
1480	MZ13-17	82.54	42.30	0	Rhyolite	300	2	300	-4.6	-4.2	1483	1461	Cheng ZG et al., 2017, Lithos
1481	C15ZS07	81.30	43.02	0	Rhyolite	355	3	355	2.8	2.7	1099	1107	Su WB et al., 2018, Lithos
1482	P3-8	121.41	47.86	0	Rhyolite	143	1.3	150	7.6	7.4	661	673	Zhang C et al., 2014, Global Geology(ICWEA)
1483	HL-2-1	129.45	42.50	0	Rhyolite	208	1	208	8.5	8.0	654	682	Guan QB et al., 2020, Gondwana Research
1484	HL-6-1	129.47	42.61	0	Rhyolite	179	1	179	8.7	8.8	618	613	Guan QB et al., 2020, Gondwana Research
1485	HM1-1	131.87	45.66	0	Rhyolite	116	1	150	9.0	9.3	578	564	Wang F et al., 2019, Lithos
1486	HQ3-1	130.95	45.82	0	Rhyolite	124	3	150	8.4	8.3	613	618	Wang F et al., 2019, Lithos
1487	14HQ3-1	130.90	45.83	0	Rhyolite	111	1	150	8.9	8.8	586	589	Wang F et al., 2019, Lithos
1488	13HYC26-1	130.56	48.73	0	Rhyolite	99	1	150	7.2	7.4	685	672	Wang F et al., 2019, Lithos
1489	13HYC27-1	130.49	48.57	0	Rhyolite	100	1	150	8.6	9.1	603	572	Wang F et al., 2019, Lithos
1490	XQ1	125.25	43.58	0	Rhyolite	255	3	255	4.1	4.3	946	935	Song ZG et al., 2018, Gondwana Research
1491	14GW138	133.16	46.26	0	Rhyolite	182	4	182	8.4	8.4	641	640	Wang ZH et al., 2017, Journal of Asian Earth sciencesences.
1492	13GW184	133.30	48.08	0	Rhyolite	176	3	176	7.1	7.2	708	706	Wang ZH et al., 2017, Journal of Asian Earth sciencesences.
1493	16ZL54	121.21	47.38	0	Rhyolite	150	2	150	6.8	6.5	707	727	Tang ZY et al., 2019, Geological Journal, 2020, International Geology Review
1494	16ZL57	121.08	47.17	0	Rhyolite	142	2	150	6.9	6.7	700	716	Tang ZY et al., 2019, Geological Journal, 2020, International Geology Review
1495	16W88-1	121.07	45.81	0	Rhyolite	142	2	150	8.8	9.1	589	574	Tang ZY et al., 2019, Geological Journal, 2020, International Geology Review
1496	D1332	120.94	46.36	Eastern CAOB	Rhyolite	140	2	150	8.7	8.9	595	583	Gong MY et al., 2018, Lithos
1497	D1336	120.13	44.05	Eastern CAOB	Rhyolite	154	2	154	3.1	2.8	922	939	Gong MY et al., 2018, Lithos
1498	M215-1	120.43	50.36	0	Rhyolite	161	1	161	7.2	6.5	695	732	Gou J et al., 2019, International Geology Review
1499	W1401-2	118.64	43.86	0	Rhyolite	148	2	150	6.9	6.7	702	711	Guan QB et al., 2018, International Geology Review
1500	QP2-1	120.35	49.38	0	Rhyolite	131	2	150	8.0	8.4	637	617	Wang, Y. et al., 2017, Global Geology(ICWEA)
1501	D9843-1	120.50	46.85	Eastern CAOB	Rhyolite	162	2	162	6.3	6.0	747	764	Ji Z et al., 2018, Journal of Asian Earth sciencesences.
1502	D9845-1	120.48	46.86	Eastern CAOB	Rhyolite	157	1	157	6.3	5.9	739	763	Ji Z et al., 2018, Journal of Asian Earth sciencesences.
1503	D9849-1	120.42	46.84	Eastern CAOB	Rhyolite	161	1	161	7.1	6.7	698	721	Ji Z et al., 2018, Journal of Asian Earth sciencesences.
1504	PM012-1-1	120.72	46.93	0	Rhyolite	141	2	150	9.4	9.2	558	570	Ji Z et al., 2016, International Geology Review
1505	1579-5	123.76	52.70	0	Rhyolite	126	2	150	1.0	1.4	1044	1020	Guo HY et al., 2015, Global Geology(ICWEA)
1506	HYL3-1	127.66	46.20	0	Rhyolite	179	2	179	8.3	7.6	641	684	Tang J et al., 2011, Global Geology(ICWEA)
1507	13HYC23-1	129.83	48.78	0	Rhyolite	206	2	206	1.1	1.1	1079	1079	Guo P et al., 2016, International Geology Review
1508	13XL-14	120.60	45.03	0	Rhyolite	147	2	150	11.2	11.2	450	450	Dong JL et al., 2019, Lithos

1509	13XL-25	120.60	45.03	0	Rhyolite	148	2	150	12.3	11.8	390	420	Dong JL et al., 2019, Lithos
1510	KB2127	120.60	45.48	0	Rhyolite	141	1	150	10.8	10.6	474	486	Wang, J et al., 2013, Acta Petrologica Sinica(ICWEA)
1511	M007-1	117.09	48.28	0	Rhyolite	162	2	162	5.6	6.1	787	756	Gou J et al., 2013, International Geology Review
1512	M054-6	116.82	48.62	0	Rhyolite	141	1	150	6.2	6.2	743	742	Gou J 2010, Changchun: Jilin University (ICWEA)
1513	M122-1	117.50	49.33	0	Rhyolite	139	1	150	7.5	7.3	666	679	Gou J 2010, Changchun: Jilin University (ICWEA)
1514	M132-1	116.74	49.54	0	Rhyolite	141	1	150	5.1	4.9	804	815	Gou J 2010, Changchun: Jilin University (ICWEA)
1515	M138-1	117.19	49.50	0	Rhyolite	141	1	150	7.9	8.1	641	631	Gou J 2010, Changchun: Jilin University (ICWEA)
1516	F04-057	119.75	40.05	North China craton	Rhyolite	120	0	150	-8.7	-8.7	1600	1598	Yang JH et al., 2008, Journal of Petrology.
1517	F04-062	119.75	40.05	North China craton	Rhyolite	119	0	150	-12.5	-12.4	1816	1810	Yang JH et al., 2008, Journal of Petrology.
1518	HSS6	92.00	40.90	Western CAOB	Rhyolite	280	0	280	7.0	7.6	801	763	Su BX et al., 2011, Journal of Asian Earth sciencesences
1519	X0555-7	90.35	46.79	Western CAOB	Rhyolite	502	7.2	502	-10.6	-11.1	1980	2008	Yang TN et al., 2011, Precambrian Researchearch
1520	08HLQ04	112.00	41.00	North China craton	Rhyolite	1886	8	600	-35.8	-36.0	3471	3483	Peng P et al., 2011, Precambrian Researchearch
1521	BH39	87.19	48.45	Western CAOB	Rhyolite	411	0	411	3.5	4.2	1102	1064	Wang YJ et al., 2011, Journal of Asian Earth sciencesences
1522	641	90.50	43.50	Western CAOB	Rhyolite	293	0	293	0.5	0.2	1182	1200	Chen XJ et al., 2011, Lithos
1523	650	90.50	43.50	Western CAOB	Rhyolite	295	0	295	5.5	8.3	895	735	Chen XJ et al., 2011, Lithos
1524	652	90.50	43.50	Western CAOB	Rhyolite	294	0	294	4.0	6.5	982	837	Chen XJ et al., 2011, Lithos
1525	O9LBJE-79	89.50	45.15	Western CAOB	Rhyolite	307	0	307	14.4	14.1	389	407	Su BX et al., 2012, Gondwana Research.
1526	O9LSG-6	89.50	45.15	Western CAOB	Rhyolite	314	0	314	13.3	13.8	459	435	Su BX et al., 2012, Gondwana Research.
1527	TH05-2	91.80	44.60	Western CAOB	Rhyolite	454	1.7	454	12.5	12.3	620	634	Xu XW et al., 2013, Gondwana Research.
1528	HBQ17-1	132.00	46.00	Eastern CAOB	Rhyolite	392	0	392	3.9	3.9	1067	1064	Meng E et al., 2011, Acta Petrologica Et Mineralogica
1529	HBQ26-2	132.00	46.00	Eastern CAOB	Rhyolite	388	0	388	5.0	4.9	998	1003	Meng E et al., 2011, Acta Petrologica Et Mineralogica
1530	K28-16-1	89.30	46.50	Western CAOB	Rhyolite	278	0	278	12.4	12.0	486	506	Li D et al., 2014, Lithos
1531	DH020	126.74	43.37	North China craton	Rhyolite	300	0	300	6.7	6.7	830	833	Yu Q et al., 2014, Lithos
1532	DH037	126.75	43.36	North China craton	Rhyolite	300	0	300	8.1	8.6	751	722	Yu Q et al., 2014, Lithos
1533	DH041	126.75	43.35	North China craton	Rhyolite	300	0	300	8.5	8.4	730	735	Yu Q et al., 2014, Lithos
1534	MN1	83.50	41.00	Western CAOB	Rhyolite	272	0	272	3.6	3.6	986	991	Liu HQ et al., 2014, Lithos
1535	WQ09-1	81.90	41.80	Western CAOB	Rhyolite	287	0	287	-2.9	-2.8	1376	1371	Liu HQ et al., 2014, Lithos
1536	12HSW4-1	127.14	49.33	Eastern CAOB	Rhyolite	295	0	295	9.4	9.3	669	676	Li Y et al., 2014, Lithos
1537	HSW10-1	127.12	49.98	Eastern CAOB	Rhyolite	351	0	351	9.1	9.3	734	722	Li Y et al., 2014, Lithos
1538	HSW8-1	127.18	49.98	Eastern CAOB	Rhyolite	293	0	293	12.3	12.4	500	496	Li Y et al., 2014, Lithos
1539	11JS3-1	126.20	44.41	Eastern CAOB	Rhyolite	364	0	364	5.8	5.6	936	943	Wang ZW, 2015, Journal of Asian Earth sciencesences
1540	11LK20-1	124.36	42.89	North China craton	Rhyolite	347	0	347	11.7	11.4	580	598	Wang ZW, 2015, Journal of Asian Earth sciencesences
1541	12JS4-1	126.29	44.43	Eastern CAOB	Rhyolite	361	0	361	6.3	6.1	905	914	Wang ZW, 2015, Journal of Asian Earth sciencesences
1542	JS2-1	126.25	44.44	Eastern CAOB	Rhyolite	360	0	360	6.2	6.4	910	896	Wang ZW, 2015, Journal of Asian Earth sciencesences
1543	DB-03-1	125.00	51.00	Eastern CAOB	Rhyolite	134	0	150	5.8	6.0	764	756	Hu X et al., 2015, Acta Geologica Sinica-English Edition.
1544	TW-39-1	125.00	51.00	Eastern CAOB	Rhyolite	116	0	150	6.9	6.9	701	699	Hu X et al., 2015, Acta Geologica Sinica-English Edition.
1545	TW-41-1	125.00	51.00	Eastern CAOB	Rhyolite	118	0	150	5.7	5.6	769	776	Hu X et al., 2015, Acta Geologica Sinica-English Edition.
1546	TW-43	125.00	51.00	Eastern CAOB	Rhyolite	121	0	150	5.6	5.0	779	810	Hu X et al., 2015, Acta Geologica Sinica-English Edition.
1547	YS1104-1	119.25	42.50	North China craton	Rhyolite	405	0	405	-19.0	-19.0	2379	2379	Liu JF et al., 2013, Geological Bulletin of China.
1548	JH4-1	127.00	43.00	North China craton	Rhyolite	279	0	279	8.0	8.2	741	730	Cao HH et al., 2012, Acta Petrologica Sinica
1549	JK11-4	127.00	43.00	North China craton	Rhyolite	286	0	286	3.6	3.8	1002	990	Cao HH et al., 2012, Acta Petrologica Sinica
1550	07057-1	112.85	41.84	North China craton	Rhyolite	276	2	276	-13.8	-13.9	1988	1994	Zhang SH et al., 2016, Lithos
1551	07116-1	110.77	41.95	North China craton	Rhyolite	261	2	261	-6.5	-7.3	1560	1607	Zhang SH et al., 2016, Lithos
1552	07117-1	110.76	41.95	North China craton	Rhyolite	261	3	261	-4.1	-4.0	1424	1419	Zhang SH et al., 2016, Lithos
1553	07D023-1	119.70	42.40	North China craton	Rhyolite	344	3	344	5.6	5.9	930	910	Zhang SH et al., 2016, Lithos
1554	07D037-1	118.65	42.63	North China craton	Rhyolite	273	5	273	-16.0	-15.8	2108	2098	Zhang SH et al., 2016, Lithos
1555	08197-1	118.64	43.30	Eastern CAOB	Rhyolite	314	3	314	1.5	1.4	1142	1149	Zhang SH et al., 2016, Lithos
1556	08485-1	110.73	41.76	North China craton	Rhyolite	264	2	264	5.1	4.7	898	917	Zhang SH et al., 2016, Lithos
1557	08543-2	110.13	41.84	North China craton	Rhyolite	269	3	269	-6.9	-6.8	1585	1580	Zhang SH et al., 2016, Lithos
1558	NM14-50	114.00	44.00	Eastern CAOB	Rhyolite	439	0	439	5.3	4.7	1022	1058	Chen Y et al., 2016, Lithos
1559	NM14-53	114.00	44.00	Eastern CAOB	Rhyolite	441	0	441	2.3	2.2	1194	1199	Chen Y et al., 2016, Lithos
1560	NM14-59	114.00	44.00	Eastern CAOB	Rhyolite	445	0	445	-0.6	0.1	1367	1332	Chen Y et al., 2016, Lithos
1561	A13002	75.55	41.00	Western CAOB	Rhyolite	461	0	461	-3.1	-3.2	1521	1525	Alexeiev DV et al., 2016, Gondwana Research.
1562	13GW118	132.91	47.86	Eastern CAOB	Rhyolite	267	0	267	-3.3	-4.0	1381	1424	Bi JH et al., 2017, Journal of Asian Earth sciencesences
1563	13GW120	132.00	47.00	Eastern CAOB	Rhyolite	269	0	269	-0.1	-0.4	1200	1214	Bi JH et al., 2017, Journal of Asian Earth sciencesences
1564	13GW170	132.00	47.00	Eastern CAOB	Rhyolite	290	0	290	5.0	5.0	924	920	Bi JH et al., 2017, Journal of Asian Earth sciencesences
1565	13GW186	133.02	47.99	Eastern CAOB	Rhyolite	279	0	279	3.2	2.4	1017	1063	Bi JH et al., 2017, Journal of Asian Earth sciencesences
1566	14GW516	133.02	47.99	Eastern CAOB	Rhyolite	279	0	279	6.0	10.7	853	581	Bi JH et al., 2017, Journal of Asian Earth sciencesences
1567	DS005	121.37	46.32	Eastern CAOB	Rhyolite	280	0	280	10.1	10.5	618	598	Yu Q et al., 2017, Gondwana Research
1568	DS019	121.37	46.32	Eastern CAOB	Rhyolite	280	0	280	8.5	8.3	711	723	Yu Q et al., 2017, Gondwana Research
1569	DS042	121.37	46.32	Eastern CAOB	Rhyolite	280	0	280	8.3	8.1	723	736	Yu Q et al., 2017, Gondwana Research
1570	DS070	121.37	46.32	Eastern CAOB	Rhyolite	280	0	280	9.6	10.0	650	623	Yu Q et al., 2017, Gondwana Research
1571	DS079	121.37	46.32	Eastern CAOB	Rhyolite	909	0	600	2.6	5.5	909	909	Yu Q et al., 2017, Gondwana Research
1572	09DDS41_1	96.00	41.00	Western CAOB	Rhyolite	371	0	371	9.5	10.3	727	682	Guo QQ et al., 2017, Lithos
1573	09DDS45	96.00	41.00	Western CAOB	Rhyolite	369	0	369	7.0	7.2	868	860	Guo QQ et al., 2017, Lithos
1574	MO397	101.73	44.86	Eastern CAOB	Rhyolite	319	0	319	0.1	0.5	1226	1206	Kröner A et al., 2017, Gondwana Research
1575	MO62	106.90	48.11	Eastern CAOB	Rhyolite	465	0	465	3.4	6.5	1151	944	Kröner A et al., 2017, Gondwana Research
1576	12XTM01	120.00	41.00	North China craton	Rhyolite	158	0	158	-21.8	-17.0	2445	2078	Wang C et al., 2017, Lithos
1577	HJ13-007	92.00	42.00	Western CAOB	Rhyolite	323	0	323	12.4	11.5	530	568	Zhao LD et al., 2018, Journal of Asian Earth sciencesences
1578	HDY3-1	129.26	45.97	Eastern CAOB	Rhyolite	317	0	317	11.9	11.9	542	544	Guo P et al., 2018, Gondwana Research
1579	P1-26	113.00	44.00	Eastern CAOB	Rhyolite	287	0	287	11.1	11.1	568	566	Chai H et al., 2018, Lithos
1580	TWD15001	123.65	40.98	North China craton	Rhyolite	2179	0	600	-32.4	-32.0	3284	3261	Bi JH et al., 2018, Precambrian Researchearch
1581	TWD15002	123.66	40.97	North China craton	Rhyolite	2127	0	600	-35.4	-36.0	3450	3484	Bi JH et al., 2018, Precambrian Researchearch
1582	TWD15056	123.61	40.99	North China craton	Rhyolite	2184	0	600	-32.3	-32.0	3273	3257	Bi JH et al., 2018, Precambrian Researchearch
1583	DWQ09-6	82.00	42.00	Western CAOB	Rhyolite	274	0	274	-3.0	-3.2	1371	1383	Liu HQ et al., 2019, Lithos
1584	K03-1	83.00	42.20	Western CAOB	Rhyolite	293	0	293	-6.0	-6.0	1554	1557	Liu HQ et al., 2019, Lithos
1585	K07-1	83.00	42.20	Western CAOB	Rhyolite	293	0	293	-6.4	-6.3	1580	1572	Liu HQ et al., 2019, Lithos
1586	HN4-14	110.54	47.62	Eastern CAOB	Rhyolite	461	0	461	0.3	0.2	1329	1330	Tserendash N et al., 2019, Journal of Asian Earth sciencesences
1587	HN48	110.59	47.50	Eastern CAOB	Rhyolite	418	0	418	-3.1	-3.6	1485	1516	Tserendash N et al., 2019, Journal of Asian Earth sciencesences
1588	D9806-2	120.37	46.81	0	Rhyolite	133	1	150	6.2	6.1	743	749	Dong Y et al., 2014, Lithos
1589	PM002-18-1	120.30	46.78	0	Rhyolite	130	4	150	6.8	7.4	710	671	Dong Y et al., 2014, Lithos
1590	BJS-3	106.59	35.06	the southwestern North China Craton	rhyolites	1795	0	600	-29.3	-29.4	3111	3113	Pang L et al., 2021. Lithos
1591	14GW144	133.23	46.40	0	Rhyolitic crystal tuff	174	0	174	7.3	7.1	695	706	Wang ZH et al., 2017, Journal of Asian Earth sciencesences.
1592	14GW152	133.30	46.47	0	Rhyolitic crystal tuff	187	4	187	8.3	8.3	651	649	Wang ZH et al., 2017, Journal of Asian Earth sciencesences.

1593	13GW177	133.30	48.08	0	Rhyolitic crystal tuff	174	2	174	5.3	5.4	811	806	Wang ZH et al., 2017, Journal of Asian Earth sciencesences.
1594	D0570-5	120.37	46.96	0	Rhyolitic tuff	134	1	150	5.3	5.4	796	789	Dong Y et al., 2014, Lithos
1595	D9805	120.37	46.80	0	Rhyolitic tuff	133	2	150	7.8	8.0	649	636	Dong Y et al., 2014, Lithos
1596	D9951	120.29	46.78	0	Rhyolitic tuff	132	2	150	9.0	8.9	578	584	Dong Y et al., 2014, Lithos
1597	D0570-5-2	120.37	46.96	NE China	rhyolitic tuff	134	1	150	5.3	5.4	796	789	Dong Y et al.2014.
1598	11WJ06	90.91	46.06	0	Syenite	279	2.4	279	12.8	13.0	465	453	Tang et al., 2019 Earth and Planetary Science Letters.
1599	11QH06	90.91	46.06	0	Syenite	279	2.4	279	12.0	12.3	511	490	Tang et al., 2019 Earth and Planetary Science Letters.
1600	S-1	85.43	46.70	0	syenite	392	6	392	13.6	13.7	506	502	Chen et al., 2015.
1601	F04-067	119.75	40.05	North China craton	Syenite	120	0	150	-14.2	-13.7	1913	1884	Yang JH et al., 2008, Journal of Petrology.
1602	F04-111	117.55	40.68	North China craton	Syenite	129	0	150	-5.4	-4.7	1409	1370	Yang JH et al., 2008, Journal of Petrology.
1603	FW04-113	117.55	40.68	North China craton	Syenite	130	0	150	-3.9	-3.4	1324	1298	Yang JH et al., 2008, Journal of Petrology.
1604	FW04-114	117.55	40.68	North China craton	Syenite	130	0	150	-3.0	-2.6	1271	1252	Yang JH et al., 2008, Journal of Petrology.
1605	TS-002	90.12	46.46	Western CAOB	Syenite	384	0	384	10.7	10.7	670	665	Liang P et al., 2016, Lithos
1606	09JH50	124.61	40.91	North China craton	Syenite	225	0	225	-1.2	-1.3	1228	1232	Zhu YS et al., 2017, Geology
1607	09JH58	124.61	40.91	North China craton	Syenite	225	0	225	-1.7	-1.6	1255	1250	Zhu YS et al., 2017, Geology
1608	09JH65	124.55	40.91	North China craton	Syenite	226	0	226	-12.4	-12.4	1872	1868	Zhu YS et al., 2017, Geology
1609	09JH79	124.39	40.99	North China craton	Syenite	230	0	230	-12.2	-12.2	1860	1859	Zhu YS et al., 2017, Geology
1610	09JH81	124.39	40.99	North China craton	Syenite	230	0	230	-10.0	-9.9	1736	1731	Zhu YS et al., 2017, Geology
1611	CL13-018	92.00	42.00	Western CAOB	Syenite	252	0	252	4.9	5.9	905	862	Zhao LD et al., 2018 Journal of Asian Earth Sciences
1612	07052-1	112.89	41.56	North China craton	Syenite	423	0	423	-30.6	-30.4	3031	3019	Zhang QQ et al., 2018 Lithos
1613	08587-1	110.42	41.04	North China craton	Syenite	399	0	399	-20.3	-19.4	2451	2396	Zhang QQ et al., 2018 Lithos
1614	14JN16-1	127.32	42.86	North China craton	Syenite	2471	0	600	-37.5	-37.2	3564	3552	Guo, B.R et al., 2018 Gondwana Research
1615	11K46	86.19	41.83	Western CAOB	Syenite	627	0	600	-3.8	-3.7	1667	1664	GE R et al., 2015 Geological Society of America Bulletin
1616	11K48	86.19	41.82	Western CAOB	Syenite	660	0	600	-4.3	-4.6	1701	1714	GE R et al., 2015 Geological Society of America Bulletin
1617	11K51	86.19	41.81	Western CAOB	Syenite	659	0	600	-4.5	-4.5	1707	1712	GE R et al., 2015 Geological Society of America Bulletin
1618	HL-032	90.03	46.60	Western CAOB	Syenite	411	0	411	9.0	8.9	771	774	Wu C et al., 2015 Ore Geology Reviews
1619	JN08-01	112.91	41.57	North China Craton	Syenite	408	0	408	-29.8	-29.8	2994	2992	Zhang XH et al., 2010 Journal of Structural Geology
1620	07235-1	110.22	40.57	North China Craton	Syenite	233	0	233	-13.0	-5.7	1905	1491	Zhang SH et al., 2012 Lithos
1621	07236-1	110.24	40.57	North China Craton	Syenite	224	0	224	-9.4	-9.8	1695	1717	Zhang SH et al., 2012 Lithos
1622	YPD01	115.13	40.93	North China Craton	Syenite	373	0	373	-11.9	-12.0	1956	1958	Li CM, 2014 Acta Petrologica Sinica (ICWEA)
1623	05FW50	119.24	40.89	North China Craton	Syenite	225	0	225	-1.4	-1.5	1237	1242	Yang JH et al., 2012 Chemical Geology
1624	F04-111	117.52	40.60	North China Craton	Syenite	129	0	150	-4.4	-4.2	1354	1340	Yang JH et al., 2008 Journal of Petrology
1625	JS11-1	118.30	40.82	North China Craton	Syenite	109	0	150	0.4	0.2	1079	1087	Liu Y, 2015 Acta Petrologica Et Mineralogica (ICWEA)
1626	FZ1703	114.83	40.50	Trans-North China	syenite	1808	0	600	-33.5	-33.5	3344	3342	Kuang GX et al., 2020 Precambrian Research
1627	71807	117.64	42.92	Northern margin of the North China Craton	Syenite	265	0	265	0.9	2.2	1139	1063	Zhao Pan et al., 2016 Lithos
1628	61001	113.57	42.41	Northern margin of the North China Craton	Syenite	261	0	261	7.8	7.7	739	743	Zhao Pan et al., 2016 Lithos
1629	F04-113	117.52	40.60	North China Craton	Syenite	130	0	150	-4.4	-4.2	1354	1340	Yang JH et al., 2008 Journal of Petrology
1630	F04-114	117.52	40.60	North China Craton	Syenite	130	0	150	-2.8	-2.6	1261	1252	Yang JH et al., 2008 Journal of Petrology
1631	WLS-2	117.48	40.58	North China Craton	Syenite	127	0	150	-4.8	-4.5	1374	1358	Chen CL et al., 2014 Geoscience (ICWEA)
1632	JS11-3	118.30	40.82	North China Craton	Syenite	111	0	150	1.9	2.1	991	979	Liu Y, 2015 Acta Petrologica Et Mineralogica (ICWEA)
1633	WLS-3	117.50	40.55	North China Craton	Syenite	127	0	150	-1.2	-0.8	1167	1146	Chen CL et al., 2014 Geoscience (ICWEA)
1634	JS11-4	118.29	40.82	North China Craton	Syenite	112	0	150	0.2	0.4	1089	1077	Liu Y, 2015 Acta Petrologica Et Mineralogica (ICWEA)
1635	M026-1	116.17	48.84	0	Syenite porphyry	149	2	150	3.3	3.1	909	923	Wang TH et al., 2014 Global Geology (ICWEA)
1636	BJG02	88.77	44.93	Eastern Junggar	Syenite-porphyry	312	3	312	12.2	11.9	522	543	Xiao Y et al., 2011 Gondwana Research
1637	BJG07	88.77	44.93	Eastern Junggar	Syenite-porphyry	307	5	307	11.6	11.9	552	537	Xiao Y et al., 2011 Gondwana Research
1638	05FW	120.00	42.00	the northern North China Craton	syenites	250	0	250	-2.8	-3.0	1338	1350	Yang JH et al., 2012 Chemical Geology
1639	06JH	120.00	42.00	the northern North China Craton	syenites	250	0	250	-12.3	-12.4	1873	1885	Yang JH et al., 2012 Chemical Geology
1640	WJ1126	85.46	46.71	0	Syenitic granite	418	2	418	13.2	13.4	551	537	Yin et al., 2017 Gondwana Research
1641	WJ1136	85.89	46.76	0	Syenitic granite	407	3	407	13.3	13.3	535	534	Yin et al., 2017 Gondwana Research
1642	12WJ03	85.13	46.69	0	Syenitic granite	418	7.2	418	13.0	13.0	560	560	Tang et al., 2019 Earth and Planetary Science Letters
1643	11WJ94	85.46	46.71	0	Syenitic granite	424	5.8	424	13.6	13.8	529	519	Tang et al., 2019 Earth and Planetary Science Letters
1644	12WJ08	85.14	46.69	0	Syenitic porphyry	412	4	412	13.6	13.7	520	518	Tang et al., 2019 Earth and Planetary Science Letters
1645	16DX3-1	120.69	46.33	Eastern CAOB	Syenogranite	135	1	150	7.2	7.0	685	696	Li Y et al., 2018, Lithos
1646	16DX5-1	122.60	47.71	Eastern CAOB	Syenogranite	132	1	150	6.4	6.4	728	733	Li Y et al., 2018, Lithos
1647	M196-1	119.94	49.78	0	Syenogranite	256	3	256	7.7	7.5	742	751	Gou J et al., 2013, Journal of Asian Earth Sciences
1648	15JJW9-1	126.80	43.17	0	Syenogranite	194	2	194	8.2	7.8	663	683	Wang F et al., 2019 Journal of Geophysical Research: Solid Earth
1649	15JJW11-1	126.08	43.60	0	Syenogranite	172	1	172	6.9	7.0	717	711	Wang F et al., 2019 Journal of Geophysical Research: Solid Earth
1650	11ER9-1	119.57	50.71	0	Syenogranite	224	2	224	-0.9	-1.0	1207	1215	Tang J et al., 2016, Gondwana Research
1651	12ER34-1	121.65	52.21	0	Syenogranite	206	1	206	3.4	3.3	949	956	Tang J et al., 2016, Gondwana Research
1652	12ER19-1	120.81	51.49	0	Syenogranite	205	1	205	2.5	2.5	999	999	Tang J et al., 2016, Gondwana Research
1653	13GF-91	129.36	43.70	0	Syenogranite	205	1	205	8.2	8.4	669	660	Guo F et al., 2019, Chemical Geology
1654	13GF-87	130.73	43.09	0	Syenogranite	205	1	205	8.2	8.8	668	636	Guo F et al., 2019, Chemical Geology
1655	13GF-103	129.74	43.72	0	Syenogranite	218	0	218	9.9	9.9	579	582	Guo F et al., 2019, Chemical Geology
1656	13GF-32	128.76	43.27	0	Syenogranite	228	0	228	9.4	9.1	620	634	Guo F et al., 2019, Chemical Geology
1657	13GF-105	129.40	43.49	0	Syenogranite	266	2	266	9.9	10.0	622	616	Guo F et al., 2019, Chemical Geology
1658	12ER2-1	118.83	49.94	0	Syenogranite	152	2	152	5.2	4.8	804	826	Tang J et al., 2015, Tectonophysics
1659	13ER48-1	118.83	49.94	0	Syenogranite	150	2	150	2.2	2.2	974	974	Tang J et al., 2015, Tectonophysics
1660	12ER8-1	119.53	50.78	0	Syenogranite	140	1	150	7.3	7.1	676	688	Tang J et al., 2015, Tectonophysics
1661	DST1	125.13	43.51	0	Syenogranite	257	3	257	4.2	4.6	941	918	Song ZG et al., 2018, Gondwana Research
1662	11ER18-1	120.03	51.19	0	Syenogranite	247	2	247	0.6	0.2	1144	1164	Tang J et al., 2014, Lithos
1663	11ER18	120.03	51.19	0	Syenogranite	247	2	247	0.1	0.1	1168	1168	Tang J et al., 2014, Lithos
1664	13GW222	122.32	47.78	0	Syenogranite	225	2	225	8.1	8.0	689	699	Yang H et al., 2016, Journal of Asian Earth Sciences
1665	13GW234	122.53	47.75	0	Syenogranite	230	2	230	7.0	7.2	761	749	Yang H et al., 2016, Journal of Asian Earth Sciences
1666	13GW237	122.60	47.71	0	Syenogranite	226	3	226	8.2	7.6	689	723	Yang H et al., 2016, Journal of Asian Earth Sciences
1667	13GW348	120.82	48.07	0	Syenogranite	244	3	244	9.4	9.6	631	620	Yang H et al., 2016, Journal of Asian Earth Sciences
1668	M009-1	117.09	48.25	0	Syenogranite	149	1	150	2.4	1.8	964	994	Wang TH et al., 2014 Global Geology (ICWEA)
1669	M011-1	117.06	48.24	0	Syenogranite	142	1	150	8.4	8.3	615	619	Wang TH et al., 2014 Global Geology (ICWEA)
1670	14GW511	121.77	49.46	0	Syenogranite	145	1	150	8.7	8.6	596	600	He Y et al., 2018, Canadian Journal of Earth Sciences
1671	13GW349	120.65	48.05	0	Syenogranite	143	1	150	6.9	7.1	704	692	Ji Z et al., 2016 Global Geology (ICWEA) (ICWEA)
1672	13GW012	131.11	45.01	0	Syenogranite	210	2	210	6.5	6.4	774	779	Yang H et al., 2015, Lithos
1673	12GW016	131.53	45.08	0	Syenogranite	209	2	209	5.3	5.5	838	827	Yang H et al., 2015, Lithos
1674	13HYL3-1	129.78	46.05	0	Syenogranite	204	2	204	1.2	0.8	1073	1095	Guo P et al., 2016, International Geology Review
1675	13HYL8-1	129.60	46.93	0	Syenogranite	205	2	205	0.0	0.3	1144	1126	Guo P et al., 2016, International Geology Review
1676	13HYL9-1	129.60	46.93	0	Syenogranite	204	2	204	1.6	2.0	1050	1027	Guo P et al., 2016, International Geology Review

1677	11GW026	132.41	45.57	0	Syenogranite	212	0	212	4.2	4.2	907	908	Yang H et al., 2015, Gondwana Research
1678	12GW049	132.41	45.57	0	Syenogranite	218	2	218	2.5	2.5	1011	1008	Yang H et al., 2015, Gondwana Research
1679	1629-1	124.70	52.35	0	Syenogranite	193	0	193	1.7	1.5	1035	1050	Gou J et al., 2017, International Geology Review
1680	1630-2	121.24	52.45	0	Syenogranite	207	4	207	2.7	3.1	989	965	Sun DY et al., 2013, International Geology Review
1681	14HYC12-1	129.97	47.39	0	Syenogranite	251	2	251	-0.8	-0.6	1224	1214	Long XY et al., 2020, Gondwana Research
1682	15XH17-1	129.11	44.81	0	Syenogranite	200	2	200	8.5	8.9	648	623	Long XY et al., 2020, Gondwana Research
1683	18XH1-1	127.83	45.21	0	Syenogranite	177	2	177	11.8	11.6	438	450	Long XY et al., 2020, Gondwana Research
1684	H15-34-1	129.49	47.39	0	Syenogranite	210	1	210	5.4	5.1	834	854	Ge MH et al., 2018, Lithos
1685	H15-38-1	129.91	47.40	0	Syenogranite	242	2	242	-1.5	-1.4	1256	1254	Ge MH et al., 2018, Lithos
1686	M220-1	120.15	50.66	0	Syenogranite	241	3	241	-4.8	-4.9	1448	1454	Mao AQ et al., 2021, International Geology Review
1687	M231-2	119.69	50.41	0	Syenogranite	241	5	241	-1.6	-1.2	1260	1242	Mao AQ et al., 2021, International Geology Review
1688	15ER605-2	120.14	51.01	0	Syenogranite	238	4	238	-0.4	-0.7	1189	1207	Mao AQ et al., 2021, International Geology Review
1689	15ER188-7	120.04	51.06	0	Syenogranite	234	3	234	0.4	-0.1	1142	1174	Mao AQ et al., 2021, International Geology Review
1690	14SD012	124.27	51.39	0	Syenogranite	210	3	210	-2.7	-2.3	1300	1276	Mao AQ et al., 2021, International Geology Review
1691	14GW068	130.26	45.86	Eastern CAOB	Syenogranite	299	0	299	-6.1	-6.2	1552	1561	Yang H et al., 2018 Gondwana Research
1692	14GW079	130.00	45.72	Eastern CAOB	Syenogranite	246	0	246	-3.2	-2.3	1357	1307	Yang H et al., 2018 Gondwana Research
1693	H15-70-1	128.42	45.13	Eastern CAOB	Syenogranite	200	0	200	9.8	9.6	570	586	Ge MH et al., 2017, Tectonophysics
1694	A0432	123.05	41.10	North China craton	Syenogranite	2913	0	600	-59.4	-59.6	4776	4783	Dong CY et al., 2017 Lithos
1695	A1104	123.19	41.09	North China craton	Syenogranite	2949	0	600	-56.5	-56.2	4613	4598	Dong CY et al., 2017 Lithos
1696	A1117	123.07	41.12	North China craton	Syenogranite	3024	0	600	-58.5	-58.5	4723	4724	Dong CY et al., 2017 Lithos
1697	A1211	123.28	41.09	North China craton	Syenogranite	2924	0	600	-59.4	-59.9	4773	4801	Dong CY et al., 2017 Lithos
1698	A1212	123.28	41.09	North China craton	Syenogranite	2913	0	600	-56.5	-55.8	4612	4571	Dong CY et al., 2017 Lithos
1699	A1244	123.24	41.09	North China craton	Syenogranite	2912	0	600	-57.1	-57.4	4642	4664	Dong CY et al., 2017 Lithos
1700	14GW372	130.00	45.50	Eastern CAOB	Syenogranite	1155	0	600	-6.1	-6.2	1730	1713	Yang H et al., 2018 Gondwana Research
1701	14RF22-1	132.05	44.23	North China craton	Syenogranite	500	0	500	3.7	3.5	1144	1159	Xu T et al., 2018 Lithos
1702	14RF46-6	133.65	45.69	Eastern CAOB	Syenogranite	492	0	492	-0.9	-1.0	1422	1427	Xu T et al., 2018 Lithos
1703	H15-34	129.49	47.39	Eastern CAOB	Syenogranite	210	0	210	5.4	5.1	834	854	Ge MH et al., 2019 Lithosphere
1704	H15-38	129.91	47.40	Eastern CAOB	Syenogranite	242	0	242	-1.5	-1.4	1256	1254	Ge MH et al., 2019 Lithosphere
1705	06C205	118.97	42.30	North China craton	Syenogranite	376	0	376	-28.5	-24.6	2893	2674	Zhang QQ et al., 2018 Lithos
1706	15054-1	118.97	42.30	North China craton	Syenogranite	396	0	396	-11.9	-11.8	1963	1959	Zhang QQ et al., 2018 Lithos
1707	15057-1	118.97	42.30	North China craton	Syenogranite	401	0	401	-11.5	-11.0	1951	1914	Zhang QQ et al., 2018 Lithos
1708	JD16-007	118.80	40.30	North China craton	Syenogranite	186	0	186	-9.6	-9.1	1682	1643	Jiang SH et al., 2018 Lithos
1709	JD16-107	118.98	40.33	North China craton	Syenogranite	186	0	186	-11.1	-11.2	1765	1771	Jiang SH et al., 2018 Lithos
1710	EL13-9-4	111.46	43.38	Eastern CAOB	Syenogranite	947	0	600	4.9	6.1	947	947	Yang L et al., 2018 Journal of Asian Earth Sciences
1711	13GW351	120.61	48.07	Eastern CAOB	Syenogranite	301	0	301	8.3	8.4	746	738	Ji Z et al., 2018 Lithos
1712	B80628-3	95.78	41.25	Western CAOB	Syenogranite	223	0	223	2.0	2.1	1044	1039	Li S et al., 2012, Lithos
1713	G133	90.35	45.42	Eastern Junggar	syenogranite	309	2.1	309	13.0	13.1	473	471	Liu Wei et al., 2013 Lithos
1714	BL02	90.12	45.19	Eastern Junggar	syenogranite	302	2	302	13.0	13.1	469	464	Liu Wei et al., 2013 Lithos
1715	M1498-3.1	110.43	43.89	Hegenshan ophiolite-arc-accretion complex	Syenogranite	337	0	337	13.3	13.9	482	443	Zhao H, 2015 master's thesis
1716	HLT-1-	113.66	43.57	Baolidao arc-accretion complex	Syenogranite	233	0	233	5.6	5.3	841	860	Hu CS et al., 2015 Journal of Asian Earth Sciences
1717	HLT-2-	113.66	43.57	Baolidao arc-accretion complex	Syenogranite	344	0	344	11.7	11.8	575	573	Hu CS et al., 2015 Journal of Asian Earth Sciences
1718	0024-9	116.08	43.85	Baolidao arc-accretion complex	Syenogranite	330	0	330	13.6	13.7	459	450	Zhou XH, 2012 Doctoral thesis (ICWEA)
1719	TW05.4	121.12	45.28	Baolidao arc-accretion complex	Syenogranite	213	0	213	9.3	10.5	610	543	Chen LL et al., 2016 Geology in China (ICWEA)
1720	TW06.2	120.95	45.30	Baolidao arc-accretion complex	Syenogranite	131	0	150	8.1	8.3	630	622	Chen LL et al., 2016 Geology in China (ICWEA)
1721	HG-1-7	117.44	43.63	Erdaojing accretion complex	Syenogranite	137	0	150	5.0	5.0	809	812	Zhou ZH et al., 2012 Geological Science and Technology Information (ICWEA)
1722	STWH1071	120.68	40.91	North China Craton	Syenogranite	174	0	174	-8.0	-8.0	1578	1579	Cui FH, 2015 Doctoral thesis (ICWEA)
1723	STTW.7	120.50	40.83	North China Craton	Syenogranite	201	0	201	-6.6	-6.7	1519	1522	Cui FH, 2015 Doctoral thesis (ICWEA)
1724	FX09-11	121.95	42.19	North China Craton	Syenogranite	221	0	221	-5.1	-5.3	1450	1461	Zhang XH et al., 2012 Lithos
1725	12GW026	131.18	45.43	NE China	syenogranite	541	7	541	-2.0	-2.0	1523	1522	Yang H et al., 2014 Lithos
1726	11GW034	131.85	46.23	Jiamusi Block	syenogranite	490	3	490	-3.8	-4.0	1584	1597	Bi JH et al., 2014 Journal of Asian Earth Sciences
1727	H15-10-01	128.19	46.92	LXZR	Syenogranite	190	2	190	3.7	3.5	917	930	Ge MH et al., 2019 Lithosphere
1728	H15-55-01	128.65	46.20	LXZR	Syenogranite	196	1	196	5.5	5.6	818	815	Ge MH et al., 2019 Lithosphere
1729	H15-56-01	128.54	46.27	LXZR	Syenogranite	196	1	196	6.0	6.1	792	786	Ge MH et al., 2019 Lithosphere
1730	14HT16–1	129.44	48.13	LXZR	Syenogranite	257	1	257	2.5	2.5	1040	1039	Long X et al., 2019 Geological Journal
1731	15XH20–1	129.34	44.89	LXZR	Syenogranite	260	3	260	3.4	3.3	989	996	Long X et al., 2019 Geological Journal
1732	14RS-7	118.92	43.78	Erdaojing accretion complex	Syenogranite	138	1	150	5.9	5.3	760	797	Li S et al., 2017, Tectonics
1733	18HL-9-1	130.48	45.76	0	syenogranite	507	7	507	-1.1	0.6	1442	1347	Ge MH et al., 2021, Lithos
1734	STWH1043	120.62	40.89	North China Craton	Syenogranite	170	0	170	-9.6	-9.7	1667	1673	Cui FH, 2015 Doctoral thesis (ICWEA)
1735	STTW.7-2	120.50	40.83	North China Craton	Syenogranite	189	0	189	-8.1	-7.9	1597	1586	Cui FH, 2015 Doctoral thesis (ICWEA)
1736	CM95	113.88	40.82	North China Craton	Syenogranite porphyry	140	0	150	-13.9	-13.7	1899	1883	Wu G et al., 2016 Ore Geology Reviews
1737	CM100	113.88	40.82	North China Craton	Syenogranite porphyry	141	0	150	-16.2	-16.0	2029	2016	Wu G et al., 2016 Ore Geology Reviews
1738	CM41	113.88	40.82	North China Craton	Syenogranite porphyry	149	0	150	-15.7	-15.6	2000	1993	Wu G et al., 2016 Ore Geology Reviews
1739	13GW289	123.37	48.68	0	ogranite/Alkali-feldspar gr	171	2	171	9.0	9.3	598	581	Dong Y et al., 2016, Journal of Asian Earth Sciences
1740	13GW297	123.35	48.68	0	ogranite/Alkali-feldspar gr	170	2	170	9.6	9.4	560	570	Dong Y et al., 2016, Journal of Asian Earth Sciences
1741	14TL13	120.52	40.37	North China Craton	Syenogranites	192	0	192	-20.9	-19.3	2325	2235	Li WM et al., 2017 Journal of Asian Earth Sciences
1742	M207-1	120.22	50.10	0	syenogranitic mylonite	360	5	360	12.4	12.4	552	553	Gou J et al., 2013, Journal of Asian Earth Sciences
1743	14XC-2	118.11	43.33	Erdaojing accretion complex	Syneogranite	129	1	150	2.9	3.1	933	921	Li S et al., 2017, Tectonics
1744	KLP-1	89.12	47.91	Chinese Altai	pegmatite	238	0	238	2.0	2.0	1055	1053	Lv ZH et al., 2012 Lithos
1745	KLP-2	89.12	47.91	Chinese Altai	pegmatite	233	0	233	1.6	1.6	1073	1072	Lv ZH et al., 2012 Lithos
1746	KLP-3	89.12	47.91	Chinese Altai	pegmatite	188	0	188	0.6	0.6	1098	1096	Lv ZH et al., 2012 Lithos
1747	KLP-5	89.12	47.91	Chinese Altai	pegmatite	219	0	219	1.4	1.3	1072	1077	Lv ZH et al., 2012 Lithos
1748	KLP-6	89.12	47.91	Chinese Altai	pegmatite	211	0	211	1.4	1.4	1078	1069	Lv ZH et al., 2012 Lithos
1749	W8037	80.84	42.45	Western CAOB	Tonalite	333	3	333	8.9	8.8	729	739	Gou LL et al., 2012, Lithos
1750	11RJ-5	118.36	43.42	Erdaojing accretion complex	Tonalite	134	1	150	3.4	3.3	905	907	Li S et al., 2013, Journal of Asian Earth Sciences
1751	M189-1	119.71	49.66	0	Tonalite	319	4	319	8.4	8.4	748	750	Gou J et al., 2013, Journal of Asian Earth Sciences
1752	13GF-46	129.95	42.48	0	Tonalite	291	3	291	-32.7	-32.1	3064	3034	Guo F et al., 2019, Chemical Geology
1753	13GF-84	130.83	43.14	0	Tonalite	112	0	150	9.4	9.3	559	562	Guo F et al., 2019, Chemical Geology
1754	H15-13-1	128.47	46.94	0	Tonalite	496	3	496	-0.6	-1.0	1405	1426	Ge MH et al., 2021, Lithos
1755	H15-13-2	128.47	46.94	0	Tonalite	502	3	502	-0.6	-0.6	1412	1411	Ge MH et al., 2021, Lithos
1756	14DB18-1-1	125.79	50.25	Eastern CAOB	Tonalite	223	3	223	10.7	11.1	537	518	Zhao C et al., 2019, Lithos
1757	Lzk1–285-1	125.87	50.25	Eastern CAOB	Tonalite	244	2	244	12.4	12.9	458	430	Zhao C et al., 2019, Lithos
1758	01-104	112.80	66.27	Eastern CAOB	Tonalite	2700	0	600	-47.1	-47.1	4097	4098	Moyen JF et al., 2017 Earth and Planetary Science Letters
1759	02-105	112.80	66.27	Eastern CAOB	Tonalite	2724	0	600	-46.5	-46.7	4064	4078	Moyen JF et al., 2017 Earth and Planetary Science Letters
1760	02-137	112.80	66.27	Eastern CAOB	Tonalite	2700	0	600	-47.2	-47.3	4101	4108	Moyen JF et al., 2017 Earth and Planetary Science Letters

1761	14HC19-1	111.35	41.34	North China craton	Tonalite	2404	0	600	-47.4	-47.5	4115	4120	Chen NH et al., 2017 Precambrian Research
1762	14HC28-1	111.12	41.51	North China craton	Tonalite	2526	0	600	-40.1	-40.7	3710	3742	Chen NH et al., 2017 Precambrian Research
1763	V002C	97.61	45.74	Western CAOB	Tonalite	484	0	484	10.7	10.5	754	766	Janoušek, V., et al., 2018 Gondwana Research
1764	V005	97.52	45.81	Western CAOB	Tonalite	513	0	513	4.6	4.5	1126	1134	Janoušek, V., et al., 2018 Gondwana Research
1765	YD4703-10	92.00	42.00	Western CAOB	Tonalite	344	0	344	12.3	11.8	539	566	Wang W et al., 2015 Gondwana Research
1766	XIX	61.68	56.93	Western CAOB	Tonalite	433	0	433	13.5	12.9	545	581	Smirnov VN et al., 2018 Geochemistry International
1767	12k92	86.60	41.53	Western CAOB	Tonalite	1943	0	600	-28.8	-28.8	3080	3083	GE R et al., 2015 Geological Society of America Bulletin
1768	09HS49-5	79.98	46.97	Western CAOB	Tonalite	324	0	324	13.4	13.5	470	462	Shen P et al., 2018 Gondwana Research
1769	12HSB03-3	74.30	51.85	Western CAOB	Tonalite	485	0	485	12.8	13.0	629	623	Shen P et al., 2018 Gondwana Research
1770	12HSB03-4	74.30	51.85	Western CAOB	Tonalite	484	0	484	11.5	10.9	706	749	Shen P et al., 2018 Gondwana Research
1771	9HS76-5	78.47	44.48	Western CAOB	Tonalite	431	0	431	8.4	8.9	844	819	Shen P et al., 2018 Gondwana Research
1772	ME2-5	104.13	49.02	Eastern CAOB	Tonalite	254	0	254	8.7	8.3	671	652	Shen P et al., 2018 Gondwana Research
1773	ME2-7	104.13	49.02	Eastern CAOB	Tonalite	241	0	241	8.1	7.6	706	696	Shen P et al., 2018 Gondwana Research
1774	TZK705-530	92.60	42.12	Western CAOB	Tonalite	334	0	334	12.9	13.0	501	496	Shen P et al., 2018 Gondwana Research
1775	YZK6307-601	92.52	42.10	Western CAOB	Tonalite	331	0	331	13.1	12.9	489	500	Shen P et al., 2018 Gondwana Research
1776	D021-1TN	106.48	41.24	Northern margin of the North China Craton	Tonalite	325	0	325	-4.1	-3.9	1470	1463	Liu M et al., 2016 Journal of Asian Earth Sciences
1777	08502-1	110.12	42.00	Northern margin of the North China Craton	Tonalite	470	0	470	7.2	7.3	937	935	Zhang SH et al., 2014 GSAB
1778	08515-2	110.57	41.84	Northern margin of the North China Craton	Tonalite	433	0	433	-6.3	-5.0	1679	1607	Zhang SH et al., 2014 GSAB
1779	08417-1	112.56	42.24	Northern margin of the North China Craton	Tonalite	430	0	430	1.0	0.9	1265	1266	Zhang SH et al., 2014 GSAB
1780	08429-1	112.63	42.27	Northern margin of the North China Craton	Tonalite	433	0	433	0.9	1.7	1269	1224	Zhang SH et al., 2014 GSAB
1781	GY36	110.05	41.17	North China Craton	Tonalite	277	0	277	-21.2	-20.8	2409	2383	Zhang W et al., 2012 Geology in China (ICWEA)
1782	H15-57-01	128.53	46.42	LXZR	Tonalite	186	1	186	5.1	5.1	837	831	Ge MH et al., 2019 Lithosphere
1783	08500-1	110.12	41.99	Northern margin of the North China Craton	Tonalite	473	0	473	9.6	9.8	800	791	Zhang SH et al., 2014 GSAB
1784	08413-1	112.53	42.22	Northern margin of the North China Craton	Tonalite	439	0	439	-1.0	0.4	1384	1305	Zhang SH et al., 2014 GSAB
1785	71813	117.60	42.93	Northern margin of the North China Craton	Tonalite	267	0	267	-0.3	2.4	1208	1052	Zhao Pan et al., 2016 Lithos
1786	YTS7	82.48	43.73	0	Tonalite porphyry	269	3	269	11.7	11.5	521	528	Li NB et al., 2013
1787	BJXC	121.34	44.98	Erdaojing accretion complex	Tonalite porphyry	152	0	152	5.9	5.9	763	758	Wu CL, 2013 master's thesis
1788	1565-5	124.51	51.89	0	Trachyandensite	122	4	150	3.7	3.6	888	892	Guo HY et al., 2015 Global Geology (ICWEA) (ICWEA)
1789	1575-4	124.52	52.60	0	Trachyandensite	120	2	150	1.8	2.4	995	960	Guo HY et al., 2015 Global Geology (ICWEA) (ICWEA)
1790	NM14-04	112.85	42.21	0	Trachyandesite	131	1	150	-6.3	-5.6	1460	1424	Tang JZ et al., 2019, International Geology Review
1791	NM16-26	112.85	42.21	0	Trachyandesite	131	1	150	-1.5	-0.9	1189	1153	Tang JZ et al., 2019, International Geology Review
1792	16W93-1	121.28	45.94	0	Trachyandesite	152	2	152	8.6	9.2	607	568	Tang ZY et al., 2020, International Geology Review
1793	16W127-1	119.89	45.67	0	Trachyandesite	151	2	151	8.4	8.4	616	614	Tang ZY et al., 2020, International Geology Review
1794	HYL2-1	127.66	46.20	0	Trachyandesite	184	2	184	8.3	8.7	645	623	Tang J et al., 2011 Global Geology (ICWEA)
1795	TH02-3	91.80	44.60	Western CAOB	Trachyandesite	442	1.9	442	12.2	13.3	627	565	Xu XW et al., 2013 Gondwana Research
1796	11PG18-3	117.10	40.24	North China craton	Trachyandesite	1664	0	600	-22.1	-22.3	2706	2716	Wang W et al., 2015 Gondwana Research
1797	11PG44-1	117.23	40.21	North China craton	Trachyandesite	1652	0	600	-22.7	-22.7	2741	2742	Wang W et al., 2015 Gondwana Research
1798	TS04	85.00	43.00	Western CAOB	Trachyandesite	316	0	316	-26.8	-32.2	2748	3057	Zhu YF et al., 2009 Journal of the Geological Society
1799	15XH18-1	129.18	44.90	Eastern CAOB	Trachyandesite	315	0	315	8.2	8.3	763	773	Guo P et al., 2018, Gondwana Research
1800	16LJ4-1	126.99	41.80	0	Trachyandesites	246	2	246	-11.3	-11.0	1823	1804	Wang F et al., 2019 Journal of Geophysical Research: Solid Earth
1801	14JSW7-1	127.08	41.93	0	Trachyandesites	220	1	220	5.3	5.3	847	851	Wang F et al., 2019 Journal of Geophysical Research: Solid Earth
1802	HTW4-1	129.33	48.74	0	Trachyte	108	1	150	5.7	5.2	771	798	Wang F et al., 2019, Lithos
1803	15ER295	119.80	50.72	0	Trachyte	126	2	150	3.0	2.8	929	941	Gou J et al., 2019, International Geology Review
1804	09BGB-12	119.25	40.92	North China craton	Trachyte	220	0	220	-13.8	-6.5	1941	1527	Ma Q et al., 2012 Lithos
1805	TH03-3	91.80	44.60	Western CAOB	Trachyte	442	2	442	12.3	12.3	621	622	Xu XW et al., 2013 Gondwana Research
1806	09JH67	124.55	40.97	North China craton	Trachyte	222	0	222	-13.1	-13.1	1904	1906	Zhu YS et al., 2017 Geology
1807	38-2-705	91.60	40.60	the Central Asian Orogenic Belt	Troctolite	270	0	270	1.1	0.8	1133	1150	Xue SC et al., 2018 Contributions to Mineralogy and Petrology
1808	14HC09-1	111.39	41.54	North China craton	Trondhjemite	2505	0	600	-41.2	-41.3	3771	3779	Chen NH et al., 2017 Precambrian Research
1809	14LS04-1	111.00	41.50	North China craton	Trondhjemite	2530	0	600	-37.9	-38.1	3586	3597	Chen NH et al., 2017 Precambrian Research
1810	14RF38-5	133.26	43.17	North China craton	Trondhjemite	445	0	445	3.0	3.2	1157	1145	Xu T et al., 2018 Lithos
1811	Mo517	92.54	46.05	SW Mongolia	Tuffaceous	511	4	511	-8.2	-8.2	1850	1850	Jiang YD et al., 2012 Lithos
1812	KLW-1	89.12	47.91	CAOB	two mica granite	446	9	446	0.6	0.8	1297	1287	Lv ZH et al., 2012 Lithos
1813	ARJ12	89.24	47.49	Chinese Altai	Two-mica gneissic granite	381	5	381	3.5	3.2	1078	1095	Cai Keda et al., 2011 Journal of Asian Earth Sciences
1814	10LX-66	117.49	43.80	0	Two-mica granite	246	2	246	11.7	10.2	502	416	Zhao L et al., 2019, Journal of Asian Earth Sciences
1815	15JJW3-1	126.50	43.33	0	Volcanic rock	179	2	179	4.9	5.5	840	804	Wang F et al., 2019 Journal of Geophysical Research: Solid Earth
1816	15JJW10-1	126.20	43.17	0	Volcanic rock	181	2	181	5.2	5.0	822	836	Wang F et al., 2019 Journal of Geophysical Research: Solid Earth
1817	Mo519	92.07	46.23	SW Mongolia	Volcanogenic schist	495	5	495	-4.0	1.9	1595	1262	Jiang YD et al., 2012 Lithos
1818	BDB-12	86.84	43.11	0	Weakly gneissic granodiori	432	4	432	2.1	2.4	1201	1184	Huang H et al., 2015 Acta Geologica Sinica (ICWEA)
1819	D4030-1	90.19	47.12	CAOB	Biotite tonalite	396	3.3	396	2.4	2.4	1158	1153	Lin XW et al., 2021 Earth Science (ICWEA)
1820	D4030-2	90.19	47.12	CAOB	Hornblende biotite diorite	392	3.7	392	1.3	1.3	1213	1213	Lin XW et al., 2021 Earth Science (ICWEA)
1821	122A	84.89	43.24	CAOB	granite	282	3.5	282	10.7	10.4	586	589	Huang D et al., 2017 Journal of Earth Sciences and Environment (ICWEA)
1822	14CL126-3	95.64	39.85	CAOB	granite	431	5	431	-9.1	-9.3	1841	1850	Wang N et al., 2016 Acta Petrologica Sinica (ICWEA)
1823	14CL129-3	95.64	39.85	CAOB	granite	360	3	360	-8.7	-8.6	1761	1756	Wang N et al., 2016 Acta Petrologica Sinica (ICWEA)
1824	14CL127-3	95.64	39.85	CAOB	granite	340	4	340	-9.5	-9.6	1794	1796	Wang N et al., 2016 Acta Petrologica Sinica (ICWEA)
1825	14CL126-6	95.64	39.85	CAOB	granite	350	3	350	-9.2	-9.2	1784	1785	Wang N et al., 2016 Acta Petrologica Sinica (ICWEA)
1826	Y28	90.59	41.59	CAOB	Andesite	338	0.9	338	1.5	7.5	1159	816	Wei BY et al., 2020 Southern Metals (ICWEA)
1827	12M06-12	77.22	50.52	COAB	Andesite	459	4.8	459	11.2	11.5	697	678	Pan HD et al., 2015 Acta Petrologica Sinica (ICWEA)
1828	P5.48	106.38	41.33	CAOB	Andesite	285	2.3	285	0.5	-3.3	1177	1393	Guo S et al., 2022 Journal of Geomechanics (ICWEA)
1829	12YX5-7	86.27	42.64	CAOB	diorite	430	1.1	430	-10.1	-10.1	1894	1895	Zhao YY et al., 2015 Geology in China (ICWEA)
1830	DEL4－1	112.02	43.00	CAOB	granite	292	3	292	14.2	14.1	393	400	Liu JL et al., 2020 Geological Bulletin of China (ICWEA)
1831	DTL6－1	114.00	43.02	CAOB	granite	288	2	288	12.8	13.3	470	443	Liu JL et al., 2020 Geological Bulletin of China (ICWEA)
1832	16BYH	85.70	46.78	CAOB	Granite prophyry	394	1.7	394	7.6	8.0	855	832	Liu CY et al., 2019 Journal of Earth Sciences and Environment (ICWEA)
1833	HX-T2	88.47	43.11	CAOB	granite	341	5.7	341	-22.7	-24.0	2540	2612	Liu GP et al., 2021 Bulletin of Mineralogy, Petrology and Geochemistry (ICWEA)
1834	BS-1505-14	95.73	42.61	CAOB	porphyre	230	3.2	230	9.7	9.6	603	607	Liu B et al., 2016 Earth Science Frontiers (ICWEA)
1835	11ND	107.07	41.12	CAOB	Muscovite monzogranite	259	3.3	259	-21.7	-16.1	2490	2106	Wu YF et al., 2013 Geological Science and Technology Information (ICWEA)
1836	百合山	98.17	42.33	CAOB	quartz diorite	351	1.5	351	7.7	7.8	812	808	Duan LF et al., 2020 Geological Bulletin of China (ICWEA)
1837	TW6576-1	106.69	41.14	CAOB	rphyritic biotite monzogran	230	1.5	230	-15.6	-15.2	2054	2034	Wang WL et al., 2018 Acta Geologica Sinica (ICWEA)
1838	XDG21	88.22	47.98	Chinese Altai	porphyritic granite	400	0	400	4.4	4.7	1042	1024	Zheng, 2016
1839	C1609	91.28	41.63	CAOB	orphyritic k-feldspar granit	334	3.6	334	-4.6	-0.9	1497	1295	Chen YR et al., 2019 Bulletin of Mineralogy, Petrology and Geochemistry (ICWEA)
1840	zk02	95.27	43.90	Eastern Junggar	Porphyritic tonalite	419	5	419	13.9	13.6	513	528	Li GF, 2015 master's thesis
1841	北口子	110.37	41.50	CAOB	granodiorite	2573	9	600	-42.8	-43.2	3862	3883	Zhang XW et al., 2022 Journal of Shijiazhuang University of Economics (ICWEA)
1842	X11-109-1	95.90	40.88	CAOB	plagioclase amphibolite	882	4	600	-3.9	-8.1	1671	1912	Jiang HY et al., 2013 Acta Petrologica Sinica (ICWEA)
1843	X10-37-1	95.58	40.80	CAOB	gneiss	881	9	600	6.2	11.4	881	881	Jiang HY et al., 2013 Acta Petrologica Sinica (ICWEA)
1844	X10-37-2	95.58	40.80	CAOB	Felsic veins	295	2	295	-1.5	-1.5	1301	1299	Jiang HY et al., 2013 Acta Petrologica Sinica (ICWEA)

1845	10TW53	113.98	43.68	CAOB	monzonitic granite	1496	20	600	-9.6	-6.7	2001	1836	Sui LX et al., 2013 Geological Bulletin of China (ICWEA)
1846	GW05073	126.28	52.29	CAOB	granite	263	1	263	0.2	0.5	1179	1158	Zhang ZQ et al., 2017 Mineral Exploration (ICWEA)
1847	11DG-1	82.26	43.18	CAOB	monzonitic granite	943	2.6	600	-7.6	-7.6	1885	1885	Li T et al., 2015 Northwestern Geology (ICWEA)
1848	14CL143-3	70.01	30.00	CAOB	granite	452	3.9	452	-2.5	-2.3	1478	1471	Wang N et al., 2016 Acta Petrologica Sinica (ICWEA)
1849	14CL155-6	80.00	30.00	CAOB	granite	462	4.3	462	-1.7	-1.9	1439	1452	Wang N et al., 2016 Acta Petrologica Sinica (ICWEA)
1850	14CL153-3	78.02	30.01	CAOB	granite	441	2.3	441	-2.6	-2.5	1479	1472	Wang N et al., 2016 Acta Petrologica Sinica (ICWEA)
1851	13AX39	103.68	40.43	CAOB	diorite	280	1	280	-10.4	-10.7	1795	1815	Ye K et al., 2016 Acta Petrologica Et Mineralogica (ICWEA)
1852	12LS119	103.09	39.67	CAOB	potassium granite	286	1	286	-2.2	-1.8	1335	1311	Ye K et al., 2016 Acta Petrologica Et Mineralogica (ICWEA)
1853	12LS103	103.18	40.09	CAOB	tonalite	272	1	272	-8.0	-8.0	1654	1653	Ye K et al., 2016 Acta Petrologica Et Mineralogica (ICWEA)
1854	12LS107	104.02	40.10	CAOB	dacite	275	2	275	-9.9	-9.5	1765	1739	Ye K et al., 2016 Acta Petrologica Et Mineralogica (ICWEA)
1855	TW6023-1	106.77	41.14	CAOB	monzonitic granite	247	1.6	247	-9.9	-9.4	1744	1711	Wang WL et al., 2018 Acta Geologica Sinica (ICWEA)
1856	3213	88.16	47.94	Chinese Altai	two-mica granite	220	0	220	5.8	5.5	823	835	Wang Tao et al., 2014
1857	XXX20	95.17	41.83	Tianshan	two-mica granite	445	0	445	-0.4	-0.4	1353	1354	Mao QG et al., 2010, Chinese Journal of Geology (ICWEA)
1858	MQS2-1	85.75	42.50	COAB	two-mica granite	292	2	292	-6.5	-6.4	1585	1578	Yu XH et al., 2020 Acta Geologica Sinica (ICWEA)
1859	BKL1-32	88.58	47.65	CAOB	two-mica granite	282	3.2	282	7.8	7.8	755	753	Chang FM et al., 2021 Acta Petrologica Sinica (ICWEA)
1860	XA7-3-288	123.50	50.70	CAOB	nonzonitic granite porphyr	129	1	150	8.1	7.7	631	653	Zhang C et al., 2013 Acta Petrologica Sinica (ICWEA)
1861	XH08-03	90.38	45.43	Eastern Junggar	monzonitic granite	298	2	298	12.5	12.5	494	494	Gan L et al., 2010 Acta Petrologica Sinica (ICWEA)
1862	XH08-09-1	90.04	45.33	Eastern Junggar	monzonitic granite	300	2	300	12.4	12.3	503	507	Gan L et al., 2010 Acta Petrologica Sinica (ICWEA)
1863	H13630-9.1	95.04	44.19	Eastern Junggar	monzonitic granite	337	2	337	15.3	15.6	365	348	Hou XY, 2015 master's thesis
1864	H13630-4.1	95.00	43.91	Eastern Junggar	monzonitic granite	340	0.84	340	13.4	13.3	474	480	Hou XY, 2015 master's thesis
1865	BL08-2	90.10	45.18	Eastern Junggar	monzonitic granite	300	0	300	12.4	12.9	501	474	Han YJ et al., 2012
1866	BS07-136	97.01	41.79	Beishan	monzonitic granite	402	0	402	-2.8	1.1	1450	1234	Zheng RG et al., 2012 Acta Scientiarum Naturalium Universitatis Pekinensis (ICWEA)
1867	XJ048-3	85.68	42.19	Tianshan	monzonitic granite	405	0	405	7.0	6.9	897	904	Lei RX et al, 2014, International Geology Review
1868	D1207TW	93.13	39.04	CAOB	monzonitic granite	252	3	252	13.2	13.8	419	383	›ng ZC et al., 2015 Earth Science(Journal of China University of Geosciences) (ICWEA)
1869	BS07-136	97.00	41.01	CAOB	monzonitic granite	402	3	402	-2.8	1.1	1450	1234	Zheng RG et al., 2012 Acta Scientiarum Naturalium Universitatis Pekinensis (ICWEA)
1870	HDM-N2	125.01	43.00	CAOB	monzonitic granite	174	1.4	174	7.4	7.2	691	703	Zhang LS et al., 2021 Acta Petrologica Sinica (ICWEA)
1871	M1288-1.7	111.37	43.70	CAOB	monzonitic granite	320	0.9	320	14.9	14.8	372	377	Zhang XW et al., 2021 Acta Petrologica Et Mineralogica (ICWEA)
1872	4XTS-97A	84.00	42.02	CAOB	monzonitic granite	465	3.5	465	-7.5	-7.8	1774	1794	Wei Q et al., 2017 Acta Geologica Sinica (ICWEA)
1873	BL1	116.03	45.04	CAOB	monzonitic granite	300	2.2	300	9.1	9.2	696	685	Yu C et al.,2017 Geoscience (ICWEA)
1874	XHS-02	119.37	44.62	CAOB	monzonitic granite	144	1.1	150	7.3	7.2	680	684	Zhou T et al., 2022 Geoscience (ICWEA)
1875	TW.03.1	121.13	45.33	CAOB	monzonitic granite	131	7.68	150	8.0	8.5	639	607	Chen LL et al., 2016 Geology in China (ICWEA)
1876	FAP-N1	127.32	44.41	CAOB	monzonitic granite	173	0.8	173	7.5	7.4	687	691	Zhang LS et al., 2021 Acta Geologica Sinica (ICWEA)
1877	BKL2-32	88.64	47.61	CAOB	monzonitic granite	285	2.3	285	7.8	8.2	754	733	Chang FM et al., 2021 Acta Petrologica Sinica (ICWEA)
1878	K1607	91.23	41.64	CAOB	monzonitic granite	447	4.9	447	11.4	11.4	679	679	Mu et al., 2020 Acta Geologica Sinica (ICWEA)
1879	K1608	91.23	41.64	CAOB	monzonitic granite	423	5	423	11.4	11.4	659	659	Mu et al., 2020 Acta Geologica Sinica (ICWEA)
1880	EJ1304	90.84	47.08	CAOB	monzonitic granite	372	1.5	372	13.1	12.5	522	552	Xu ZH et al., 2019 Acta Geologica Sinica (ICWEA)
1881	PM2-3	82.68	40.67	CAOB	monzonitic granite	368	4.5	368	1.9	1.8	1160	1166	Zhang YM et al., 2016 Acta Petrologica Sinica (ICWEA)
1882	XM7-75	85.64	46.60	CAOB	monzonitic granite	401	4.3	401	10.4	10.1	699	713	Zhang W et al., 2021 Acta Petrologica Sinica (ICWEA)
1883	ET3-1	100.15	42.28	CAOB	monzonitic granite	306	3	306	11.5	11.6	561	554	Song P et al., 2021 Acta Petrologica Sinica (ICWEA)
1884	C1603	91.28	41.63	CAOB	monzonitic granite	330	3.6	330	2.8	3.0	1078	1066	Chen YR et al., 2019 Bulletin of Mineralogy, Petrology and Geochemistry (ICWEA)
1885	TW4295	114.67	45.17	CAOB	monzonitic granite	1699	6.1	600	-17.6	-18.6	2453	2509	Su MR et al., 2020 Geology in China (ICWEA)
1886	MOHE	121.83	53.17	CAOB	monzonitic granite	485	2	485	-1.2	-1.0	1430	1419	ıg BY et al., 2015 Journal of Heilongjiang University of Science and Technology (ICWEA)
1887	QY	86.38	46.59	CAOB	monzodiorite	364	6	364	9.4	9.3	726	734	She JZ et al.,2019 Geological Bulletin of China (ICWEA)
1888	16AX26	100.09	40.13	CAOB	syenogranite	262	1	262	4.2	4.9	948	907	Huo YJ et al., 2021 Chinese Journal of Geology (ICWEA)
1889	16AX37	100.01	40.04	CAOB	biotite granite	303	2	303	5.8	6.1	885	866	Huo YJ et al., 2021 Chinese Journal of Geology (ICWEA)
1890	14TS53	94.44	42.72	CAOB	Granite prophyry	313	0.63	313	12.6	12.4	504	512	Pang BC et al., 2020 Northwestern Geology (ICWEA)
1891	14TS56	94.57	42.69	CAOB	diorite	309	0.77	309	12.9	12.8	482	484	Pang BC et al., 2020 Northwestern Geology (ICWEA)
1892	15TS11	90.36	42.08	CAOB	diorite	281	0.71	281	9.9	10.0	633	628	Pang BC et al., 2020 Northwestern Geology (ICWEA)
1893	15TS12	90.36	42.08	CAOB	monzonitic granite	268	2.3	268	12.2	12.1	487	496	Pang BC et al., 2020 Northwestern Geology (ICWEA)
1894	15TS33	94.58	42.25	CAOB	monzonitic granite	271	0.92	271	11.8	11.9	515	506	Pang BC et al., 2020 Northwestern Geology (ICWEA)
1895	14TS24-1	92.01	42.49	CAOB	granodiorite	377	0.78	377	10.2	10.0	693	701	Pang BC et al., 2020 Northwestern Geology (ICWEA)
1896	14TS25-1	90.36	42.59	CAOB	diorite	465	1.1	465	14.5	14.6	515	506	Pang BC et al., 2020 Northwestern Geology (ICWEA)
1897	16TS9-1	93.12	42.45	CAOB	monzonitic granite	414	0.91	414	13.2	13.7	544	518	Pang BC et al., 2020 Northwestern Geology (ICWEA)
1898	N1825-1	84.88	46.63	CAOB	alkaline granite	429	2.5	429	13.5	13.6	540	537	Yang G et al., 2015 Earth Science (ICWEA)
1899	CXPC2	118.83	44.49	CAOB	MME	137	1.1	150	7.2	7.4	682	674	Wei W et al., 2017 Mineral Exploration (ICWEA)
1900	MX14-2	95.30	44.20	Eastern Junggar	Granite prophyry	413	3.3	413	12.6	12.8	583	571	Zhang Y et al., 2010 Acta Petrologica Sinica (ICWEA)
1901	15ZB27-2	108.06	41.75	CAOB	biotite hornblende schist	2492	14	600	-38.2	-38.3	3608	3612	Duan RH et al., 2021 Acta Petrologica Sinica (ICWEA)
1902	-	125.86	50.59	CAOB	ᵉ hornblende plagioclase (	246	1	246	12.4	12.3	458	463	Fu JY et al., 2021 Geological Bulletin of China (ICWEA)
1903	13ZB44-1	110.04	41.15	CAOB	ᵉ hornblende plagioclase (	2546	14	600	-42.5	-42.2	3846	3825	Duan RH et al., 2021 Acta Petrologica Sinica (ICWEA)
1904	BLG13-01	77.02	40.09	CAOB	biotite monzonite granite	291	3	291	-0.7	-0.8	1252	1254	Huang H et al., 2015 Acta Geologica Sinica (ICWEA)
1905	H16G07	90.00	42.00	CAOB	biotite monzonite granite	251	3.7	251	7.9	8.0	724	716	Wang XD et al., 2019 Bulletin of Mineralogy, Petrology and Geochemistry (ICWEA)
1906	WL212-1	115.49	42.81	CAOB	biotite monzonite granite	314	3.4	314	4.4	4.5	974	969	Wang XY et al., 2013 Geoscience (ICWEA)
1907	TW3053-1	115.79	42.08	CAOB	biotite monzonite granite	247	4.7	247	-9.9	-10.0	1744	1748	Li YD et al., 2021 Acta Geoscientica Sinica (ICWEA)
1908	H13630-5.1	95.09	44.12	Eastern Junggar	biotite monzonite	346	1.5	346	13.3	13.0	485	502	Zhao JX, 2014 master's thesis
1909	151	96.89	41.06	Beishan	biotite granite	282	0	282	5.7	5.6	876	881	Zhang W et al., 2010 Acta Petrologica Et Mineralogica (ICWEA)
1910	ST7-5	85.04	42.04	COAB	biotite granite	294	3	294	-3.3	-3.5	1400	1411	Yu XH et al., 2020 Acta Geologica Sinica (ICWEA)
1911	WL95-1	115.49	42.81	CAOB	biotite granite	321	4.1	321	6.8	6.7	842	847	Wang XY et al., 2013 Geoscience (ICWEA)
1912	BKL2-7	88.64	47.61	CAOB	biotite granite	284	2.2	284	8.0	8.1	744	740	Chang FM et al., 2021 Acta Petrologica Sinica (ICWEA)
1913	16ZG.23	111.78	41.14	CAOB	biotite potassium granite	125	1	150	-11.4	-10.9	1754	1725	Zhang C et al., 2019 Acta Petrologica Et Mineralogica (ICWEA)
1914	11CL901	81.90	43.22	CAOB	biotite potassium granite	288	2.9	288	1.4	1.1	1126	1143	Liu CH et al., 2014 Acta Petrologica Sinica (ICWEA)
1915	C1605	91.23	41.64	CAOB	biotite diorite	322	2-7.4	322	10.0	10.0	661	661	Mu et al., 2020 Acta Geologica Sinica (ICWEA)
1916	H703-06-8.2	95.04	44.08	Eastern Junggar	biotite quartz monzonite	354	1	354	12.6	12.2	535	556	Zhao JX, 2014 master's thesis
1917	PM008-26-1	84.88	45.89	CAOB	granite	316	2.4	316	15.3	15.2	348	352	ang Y et al., 2015 Earth Science(Journal of China University of Geosciences) (ICWEA)
1918	ZK320	121.51	50.67	CAOB	Granite prophyry	131	0.39	150	0.2	0.2	1088	1088	Chen ZG et al., 2021 Earth Science Frontiers (ICWEA)
1919	桑德乌兰	95.30	44.20	CAOB	Granite prophyry	413	3.3	413	12.6	12.8	583	571	Zhang Y et al., 2010 Acta Petrologica Sinica (ICWEA)
1920	B702-2	120.02	45.02	CAOB	Granite prophyry	151	2.2	151	2.8	2.6	932	957	Guo XG et al., 2020 Acta Geologica Sinica (ICWEA)
1921	TW730b	97.95	42.03	CAOB	Granite prophyry	206	1.7	206	2.6	2.6	992	992	Li M et al., 2020 Geological Bulletin of China (ICWEA)
1922	DS49	110.17	40.12	CAOB	Granite prophyry	220	4	220	-15.9	-14.3	2064	1973	Wu H et al., 2018 Mineral Deposits (ICWEA)
1923	ZK71309-343	121.05	47.73	CAOB	Granite prophyry	128	1.1	150	9.9	9.9	530	530	Zhang B et al.,2021 Acta Petrologica Sinica (ICWEA)
1924	BZY-1	84.00	43.17	CAOB	Granite prophyry	308	1.2	308	8.7	8.7	720	720	Xu SL et al., 2022 Geological Bulletin of China (ICWEA)
1925	Lw2	80.53	44.94	CAOB	Granite prophyry	388	1.3	388	2.1	1.9	1165	1179	Zhan XZ et al., 2018 Journal of Xinjiang University(Natural Science Edition) (ICWEA)
1926	BL17-35	118.96	44.52	0	monzonite	237	0	237	10.2	10.3	577	573	Present study
1927	X10-24-4	95.04	40.97	Beishan	granitic gneiss	908	0	600	-7.6	-6.6	1887	1831	Ye XF et al., 2013, Geological Bulletin of China (ICWEA)
1928	XXX05	94.92	41.83	Tianshan	granitic gneiss	942	0	600	-6.9	-6.4	1848	1820	Wang ZM et al, 2014,Journal of Asian Earth Sciences

1929	XH20171004-1	82.14	45.38	CAOB	granodiorite-porphyry	288	1.5	288	12.8	13.1	470	450	Xu SL et al., 2022 Acta Geoscientica Sinica (ICWEA)
1930	MHSTW-1	83.12	45.09	CAOB	granodiorite-porphyry	317	1.9	317	14.0	13.9	423	428	Duan FH et al.,2021 Acta Petrologica Sinica (ICWEA)
1931	ZK1-79	84.17	45.08	CAOB	granodiorite-porphyry	299	5.3	299	12.8	12.8	481	475	Tao MR et al., 2022 Geological Bulletin of China (ICWEA)
1932	Lw1	80.53	44.94	CAOB	granodiorite-porphyry	386	1.2	386	3.4	3.7	1090	1073	Zhan XZ et al., 2018 Journal of Xinjiang University(Natural Science Edition) (ICWEA)
1933	16A-86	81.85	44.18	CAOB	granodiorite-porphyry	306	5.9	306	4.6	4.4	959	969	ang JB et al., 2019 Earth Science - Journal of China University of Geosciences (ICWEA)
1934	XH08-11	90.47	45.32	Eastern Junggar	granodiorite	297	6	297	11.9	11.6	528	546	Gan L et al., 2010 Acta Petrologica Sinica (ICWEA)
1935	XH08-10	90.47	45.33	Eastern Junggar	granodiorite	297	6	297	12.7	12.6	484	489	Gan L et al., 2010 Acta Petrologica Sinica (ICWEA)
1936	XJ469	94.91	41.89	Tianshan	granodiorite	737	0	600	-2.3	-2.1	1583	1570	Tang GJ et al, 2014, Journal of Asian Earth Sciences
1937	XJ471	94.91	41.89	Tianshan	granodiorite	741	0	600	-5.0	-4.8	1738	1724	Tang GJ et al, 2014, Journal of Asian Earth Sciences
1938	XWQ90	81.84	44.95	Tianshan	granodiorite	465	0	465	-2.0	-2.1	1475	1479	Huang ZY et al,2013, Lithos
1939	761	88.67	42.45	Tianshan	granodiorite	341	0	341	-0.6	-0.1	1291	1269	Ma XX et al, 2014, Gondwana Research
1940	686	86.28	42.63	Tianshan	granodiorite	482	0	482	-8.3	-8.3	1835	1831	Ma XX et al, 2013, International Geology Review
1941	PM02TW1	97.95	41.88	CAOB	granodiorite	437	0.94	437	-11.2	-11.4	1966	1978	Pan ZL et al., 2021 Journal of Shijiazhuang University of Economics (ICWEA)
1942	YD13-31	92.51	42.09	CAOB	granodiorite	421	3.2	421	10.6	10.8	700	690	Xiao B et al., 2015 Earth Science Frontiers (ICWEA)
1943	MZS7-6	96.00	41.00	CAOB	granodiorite	320	2.6	320	-0.7	-0.6	1273	1266	Wang QS et al., 2020 Acta Petrologica Sinica (ICWEA)
1944	HDM-N1	125.01	43.00	CAOB	granodiorite	175	1.6	175	7.6	7.8	680	671	Zhang LS et al., 2021 Acta Petrologica Sinica (ICWEA)
1945	12LS152	103.05	40.08	CAOB	granodiorite	247	1	247	7.2	7.3	758	752	Shi XJ et al., 2015 Acta Petrologica Et Mineralogica (ICWEA)
1946	7218	98.50	42.08	CAOB	granodiorite	306	1.2	306	9.6	9.2	668	691	Li M et al., 2018 Earth Science (ICWEA)
1947	TW730a	97.98	42.02	CAOB	granodiorite	212	1.6	212	1.7	1.7	1050	1050	Li M et al., 2020 Geological Bulletin of China (ICWEA)
1948	ZK086-53	113.52	42.37	CAOB	granodiorite	268	1.8	268	-2.0	5.5	1303	875	Zhu XF et al., 2018 Earth Science Frontiers (ICWEA)
1949	ZKI005-105	113.52	42.37	CAOB	granodiorite	268	4.1	268	5.2	6.5	890	815	Zhu XF et al., 2018 Earth Science Frontiers (ICWEA)
1950	15DX41	116.10	45.32	CAOB	granodiorite	496	6.2	496	8.9	8.9	858	861	Yang ZN et al., 2017 Geological Bulletin of China (ICWEA)
1951	15DX44	116.10	45.32	CAOB	granodiorite	479	2.7	479	6.0	5.9	1013	1021	Yang ZN et al., 2017 Geological Bulletin of China (ICWEA)
1952	WL119-1	115.49	42.81	CAOB	granodiorite	298	2.5	298	1.0	0.8	1157	1169	Wang XY et al., 2013 Geoscience (ICWEA)
1953	NM17-17	113.76	43.74	CAOB	granodiorite	447	2	447	3.9	3.9	1109	1107	Tang JZ et al., 2018 Acta Petrologica Sinica (ICWEA)
1954	XH20171004-2	83.26	45.37	CAOB	granodiorite	288	1.4	288	11.8	11.9	524	520	Xu SL et al., 2022 Acta Geoscientica Sinica (ICWEA)
1955	FAP-N2	127.33	44.43	CAOB	granodiorite	173	1.1	173	7.8	7.9	669	664	Zhang LS et al., 2021 Acta Geologica Sinica (ICWEA)
1956	XM7-70	85.64	46.60	CAOB	granodiorite	382	2.9	382	8.4	8.6	797	784	Zhang W et al., 2021 Acta Petrologica Sinica (ICWEA)
1957	D1-3	100.15	42.28	CAOB	granodiorite	313	3	313	15.4	15.4	338	338	Song P et al., 2021 Acta Petrologica Sinica (ICWEA)
1958	H13630-7.1	95.08	44.15	Eastern Junggar	granite	337	2.3	337	16.1	16.1	337	337	Hou XY, 2015 master's thesis
1959	10JX-51	90.32	45.65	Eastern Junggar	granite	440	2.3	440	9.7	9.4	767	787	Li L, 20102 master's thesis
1960	10JX-214	90.02	45.67	Eastern Junggar	granite	425	2.3	425	13.0	12.8	566	580	Li L, 20102 master's thesis
1961	10JX-95	92.58	44.33	Eastern Junggar	granite	413	3.3	413	11.4	11.2	652	663	Li L, 20102 master's thesis
1962	10JX-107	92.63	43.90	Eastern Junggar	granite	441	3.7	441	11.8	11.7	650	657	Li L, 20102 master's thesis
1963	XDG01	88.23	48.12	Chinese Altai	granite	409	0	409	5.6	5.6	982	983	Zheng, 2016
1964	121	96.64	40.92	Beishan	granite	266	0	266	3.0	3.0	1021	1019	Zhang W et al., 2010 Acta Petrologica Et Mineralogica (ICWEA)
1965	8KT03	77.13	40.25	Tianshan	granite	272	0	272	7.4	7.5	770	762	Zhang CL et al, 2013, Lithos
1966	HLJ III-1 -1	77.13	40.35	Tianshan	granite	272	0	272	4.5	3.8	939	976	Zhang CL et al, 2013, Lithos
1967	HLJ III-1	77.13	40.35	Tianshan	granite	269	0	269	4.0	3.9	960	970	Zhang CL et al, 2013, Lithos
1968	2010HLJ V-2	77.17	40.35	Tianshan	granite	271	0	271	6.0	5.8	847	859	Zhang CL et al, 2013, Lithos
1969	2010 HLJ IV-3	77.15	40.33	Tianshan	granite	269	0	269	4.0	3.9	960	970	Zhang CL et al, 2013, Lithos
1970	L08WQ08	81.16	44.91	Tianshan	granite	454	0	454	-0.3	-0.6	1406	1397	Huang ZY et al,2013, Lithos
1971	753-4	88.47	43.20	Tianshan	granite	268	0	268	12.5	12.5	481	477	Ma XX et al, 2013, Gondwana Research
1972	Z009	97.04	42.24	CAOB	granite	327	2.4	327	7.9	7.9	782	781	Bai RL et al., 2020 Acta Petrologica Sinica (ICWEA)
1973	Z010	96.76	42.11	CAOB	granite	327	2.4	327	4.6	4.8	972	961	Bai RL et al., 2020 Acta Petrologica Sinica (ICWEA)
1974	X12-10-2	96.09	41.06	CAOB	granite	303	1	303	1.8	1.8	1115	1115	Xiao et al., 2015
1975	X12-10-3	96.09	41.06	CAOB	granite	304	2	304	-0.4	3.8	1239	999	Xiao et al., 2015
1976	X12-11-3	96.09	41.06	CAOB	granite	302	2	302	-2.9	-0.3	1385	1237	Xiao et al., 2015
1977	X12-12-3	96.18	41.06	CAOB	granite	291	1	291	8.5	9.1	719	688	Xiao et al., 2015
1978	X12-7-1	95.63	40.71	CAOB	granite	270	1	270	11.4	11.4	536	536	Xiao et al., 2015
1979	X10-98-2	96.08	40.78	CAOB	granite	433	4	433	-13.2	-12.9	2073	2056	He ZY et al., 2014 Acta Petrologica Sinica (ICWEA)
1980	X10-128-1	98.86	40.39	CAOB	granite	435	4	435	-4.2	-3.1	1561	1499	He ZY et al., 2014 Acta Petrologica Sinica (ICWEA)
1981	TS18-08	83.39	44.46	CAOB	granite	303	2	303	11.2	11.3	574	569	Wang M et al., 2022 Acta Geologica Sinica (ICWEA)
1982	TS18-10	83.34	44.49	CAOB	granite	298	2	298	13.2	12.8	457	475	Wang M et al., 2022 Acta Geologica Sinica (ICWEA)
1983	21TW25	124.90	52.38	CAOB	granite	444	1.7	444	0.7	0.9	1288	1281	Ren BZ et al., 2007 Geological Survey and Research (ICWEA)
1984	21TW28	125.47	52.43	CAOB	granite	447	2.1	447	1.3	1.2	1258	1264	Ren BZ et al., 2007 Geological Survey and Research (ICWEA)
1985	13GW332	121.06	48.70	CAOB	granite	311	2	311	10.9	11.4	600	567	He Y et al., 2017 Global Geology (ICWEA)
1986	CDE411-2B	119.20	44.42	CAOB	granite	137	1	150	6.9	7.0	701	698	Zhang ZQ et al., 2017 Mineral Exploration (ICWEA)
1987	CDE411-8	119.20	44.42	CAOB	granite	137	1	150	6.0	5.9	754	758	Zhang ZQ et al., 2017 Mineral Exploration (ICWEA)
1988	XK10-1	127.12	50.17	CAOB	granite	188	1.4	188	3.3	3.4	938	935	Zeng T et al., 2021 Journal of Jilin University(Earth Science Edition) (ICWEA)
1989	XK10-11	126.75	50.27	CAOB	granite	171	1.3	171	3.7	3.3	902	927	Zeng T et al., 2021 Journal of Jilin University(Earth Science Edition) (ICWEA)
1990	XK10-17	126.95	50.29	CAOB	granite	128	1.1	150	4.8	4.8	825	821	Zeng T et al., 2021 Journal of Jilin University(Earth Science Edition) (ICWEA)
1991	XK10-50	126.39	50.05	CAOB	granite	121	0.6	150	5.5	5.4	781	788	Zeng T et al., 2021 Journal of Jilin University(Earth Science Edition) (ICWEA)
1992	L51	96.01	41.01	CAOB	granite	282	2.9	282	5.7	5.6	876	881	Zhang et al., 2010
1993	M1496-4.1	111.09	44.45	CAOB	granite	123	1.4	150	6.1	6.4	750	730	Duan SN et al., 2022 Journal of Jilin University(Earth Science Edition) (ICWEA)
1994	CT1602	86.17	42.08	CAOB	granite	296	1.7	296	-2.3	-2.6	1348	1365	Tao ZL et al., 2019 Earth Science (ICWEA)
1995	100811-2	105.20	39.61	CAOB	granite	1923	28	600	-29.8	-30.0	3139	3150	Bao C et al., 2013 Geological Bulletin of China (ICWEA)
1996	NM17-15	113.79	43.74	CAOB	granite	449	2	449	3.2	2.0	1153	1220	Tang JZ et al., 2018 Acta Petrologica Sinica (ICWEA)
1997	NM17-20	113.72	43.79	CAOB	granite	441	2	441	1.6	1.7	1238	1232	Tang JZ et al., 2018 Acta Petrologica Sinica (ICWEA)
1998	NM17-22	113.72	43.79	CAOB	granite	449	2	449	2.5	2.4	1191	1200	Tang JZ et al., 2018 Acta Petrologica Sinica (ICWEA)
1999	NM13-40	113.01	42.30	CAOB	granite	433	2.6	433	4.0	3.0	1091	1148	an XY et al., 2017 Earth Science(Journal of China University of Geosciences) (ICWEA)
2000	NM13-42	113.00	42.29	CAOB	granite	428	1.8	428	6.1	5.3	970	1012	an XY et al., 2017 Earth Science(Journal of China University of Geosciences) (ICWEA)
2001	MEC2-1	100.06	41.44	CAOB	granite	312	0	312	2.2	2.1	1101	1105	Song P et al., 2021 Acta Petrologica Sinica (ICWEA)
2002	TS28	93.80	43.35	CAOB	granite	294	2.7	294	9.0	8.8	696	707	Chen XX et al., 2016 Acta Geologica Sinica (ICWEA)
2003	TS29	93.80	43.35	CAOB	granite	298	2	298	9.8	9.8	650	654	Chen XX et al., 2016 Acta Geologica Sinica (ICWEA)
2004	吉尔塔斯	89.33	47.42	CAOB	granite	1645	0	600	-4.7	-2.0	1645	1645	Zhang X et al., 2020 Acta Mineralogica Sinica (ICWEA)
2005	大桥东	89.75	47.50	CAOB	granite	992	0	600	-5.5	-2.0	1703	1510	Zhang X et al., 2020 Acta Mineralogica Sinica (ICWEA)
2006	可可托海东	90.13	47.67	CAOB	granite	885	0	600	-8.4	-3.7	1851	1583	Zhang X et al., 2020 Acta Mineralogica Sinica (ICWEA)
2007	B4206-1	110.72	41.30	CAOB	Granitic gneiss	2509	7	600	-39.1	-39.4	3658	3671	Ma MZ et al., 2013 Acta Petrologica Sinica (ICWEA)
2008	HH2704	119.98	48.28	CAOB	monzonitic granite	179	0.6	179	7.5	7.7	688	678	Guo ZJ et al., 2014 Geology in China (ICWEA)
2009	TS126B	86.20	41.78	Tianshan	migmatite	1779	0	600	-30.6	-30.8	3184	3193	Lei RX et al, 2014, International Geology Review
2010	B70823-8	95.23	41.10	Beishan	potassium granite	397	0	397	3.5	3.5	1093	1095	Li S et al., 2011 Acta Petrologica Sinica (ICWEA)
2011	XJ046-1	85.85	42.17	Tianshan	potassium granite	388	0	388	7.4	6.4	863	919	Lei RX et al, 2014, International Geology Review
2012	766	88.48	42.35	Tianshan	potassium granite	405	0	405	-1.1	-1.1	1351	1361	Ma XX et al, 2014, Gondwana Research

2013	4XTS-79A	84.00	43.13	CAOB	potassium granite	430	3.2	430	-3.5	-3.6	1521	1527	Wei Q et al., 2017 Acta Geologica Sinica (ICWEA)
2014	12LS165	103.05	40.08	CAOB	potassium granite	272	1	272	3.5	3.6	997	991	Shi XJ et al., 2015 Acta Petrologica Et Mineralogica (ICWEA)
2015	XA3-3-357	123.50	50.70	CAOB	potassium granite	131	1	150	7.8	7.7	651	655	Zhang C et al., 2013 Acta Petrologica Sinica (ICWEA)
2016	HL-011B	90.00	46.01	CAOB	Alkaline granite porphyry	327	2.1	327	9.8	10.0	676	665	Liang P et al., 2017 Geotectonica Et Metallogenia (ICWEA)
2017	QX-055-1	89.00	46.02	CAOB	Alkaline granite porphyry	331	3.1	331	7.3	7.7	821	800	Liang P et al., 2017 Geotectonica Et Metallogenia (ICWEA)
2018	LS14-070-1	90.00	46.01	CAOB	Alkaline granite porphyry	331	3.5	331	11.3	11.3	588	592	Liang P et al., 2017 Geotectonica Et Metallogenia (ICWEA)
2019	H13911-7.1	93.72	43.04	Eastern Junggar	alkaline granite	310	1	310	8.0	8.0	765	764	Huang W, 2014 master's thesis
2020	H13917-1.1	94.47	42.71	Eastern Junggar	alkaline granite	320	1	320	11.9	12.3	545	523	Huang W, 2014 master's thesis
2021	T90912-2.2	93.15	43.91	Eastern Junggar	alkaline granite	289	1	289	10.4	10.6	609	595	Huang W, 2014 master's thesis
2022	H13916-1.1	95.33	43.02	Eastern Junggar	alkaline granite	312	1	312	10.9	10.6	600	616	Huang W, 2014 master's thesis
2023	XH08-01	90.29	45.38	Eastern Junggar	alkaline granite	304	3	304	11.9	11.7	536	547	Gan L et al., 2010 Acta Petrologica Sinica (ICWEA)
2024	B70822-4	95.51	41.19	Beishan	alkaline granite	221	0	221	4.3	4.1	910	917	Li S, 2009, Doctoral thesis (ICWEA)
2025	13NM22-1	109.58	41.03	CAOB	alkaline granite	1720	12	600	-23.3	-23.2	2775	2767	Kang JL et al., 2020 Acta Petrologica Sinica (ICWEA)
2026	JH-05	90.10	45.13	CAOB	alkaline granite	325	1.5	325	13.3	13.0	468	487	Geng YY et al., 2022 Journal of Earth Sciences and Environment (ICWEA)
2027	WC-03	89.40	45.35	CAOB	alkaline granite	317	1.5	317	13.6	13.9	449	429	Geng YY et al., 2022 Journal of Earth Sciences and Environment (ICWEA)
2028	13ZB66-1	111.33	41.42	CAOB	Hornblende monzogranite	2503	11	600	-39.5	-39.7	3679	3690	Duan RH et al., 2021 Acta Petrologica Sinica (ICWEA)
2029	15ZB28-2	108.06	41.75	CAOB	blende biotite Plagioclase	2491	5	600	-38.5	-38.5	3624	3620	Duan RH et al., 2021 Acta Petrologica Sinica (ICWEA)
2030	BS07-17-152	96.69	40.70	Beishan	hornblende granite	304	0	304	1.3	1.1	1145	1155	Feng JC et al., 2012 Acta Scientiarum Naturalium Universitatis Pekinensis (ICWEA)
2031	13XTS-96	82.89	44.27	CAOB	granite	298	2.1	298	5.3	6.0	912	873	Yang R et al., 2017 Geological Review (ICWEA)
2032	13XTS-94	82.89	44.27	CAOB	granite	303	1.5	303	6.0	6.0	876	876	Yang R et al., 2017 Geological Review (ICWEA)
2033	P31N40-04	113.04	41.82	CAOB	Granite prophyry	270	3	270	-6.3	-4.7	1556	1463	Feng F et al., 2021 Earth Science (ICWEA)
2034	zk230-1	95.27	43.90	CAOB	tonalite	422	2.5	422	13.0	13.0	562	566	Meng XG et al., 2018 Geological Bulletin of China (ICWEA)
2035	B4254-6	110.92	41.09	CAOB	(yanite garnet quartz gneis	2423	0	600	-37.5	-38.7	3566	3635	Ma MZ et al., 2013 Acta Petrologica Sinica (ICWEA)
2036	SQZ78	94.79	41.90	CAOB	rhyolite	322	1.7	322	12.6	13.4	506	462	Xu LL et al., 2014 Geology in China (ICWEA)
2037	NM13-20	112.97	42.27	CAOB	rhyolite	412	1	412	-9.2	-8.6	1831	1798	an XY et al., 2017 Earth Science(Journal of China University of Geosciences) (ICWEA)
2038	KLTQ-E	87.50	43.93	CAOB	rhyolite	294	0.85	294	12.8	13.5	471	432	Chen BX et al., 2018 Chinese Journal of Geology (ICWEA)
2039	Q100	89.59	46.16	CAOB	rhyolite	280	1.3	280	15.3	15.4	315	312	Tang HJ et al., 2018 Geological Review (ICWEA)
2040	SF2-1	81.75	43.23	CAOB	rhyolite	303	3.6	303	5.5	5.7	903	893	Ning WT et al., 2019 Acta Petrologica Et Mineralogica (ICWEA)
2041	T5-1	100.43	42.98	CAOB	ignimbrite	311	2	311	13.9	13.9	423	423	Song P et al., 2021 Acta Petrologica Sinica (ICWEA)
2042	ZK716-68	121.05	47.73	CAOB	granodiorite	506	4.3	506	4.0	4.2	1150	1138	Zhang B et al.,2021 Acta Petrologica Sinica (ICWEA)
2043	ZK71309-243	121.05	47.73	CAOB	diorite	213	2.8	213	11.1	10.6	511	539	Zhang B et al.,2021 Acta Petrologica Sinica (ICWEA)
2044	12TW1-2	113.99	43.67	CAOB	monzonitic granite	1373	7	600	-13.9	-14.0	2244	2247	Sun LX et al., 2018 Acta Geologica Sinica (ICWEA)
2045	12TW2-5	113.98	43.67	CAOB	monzonitic granite	440	3	440	-13.5	-13.8	2095	2116	Sun LX et al., 2018 Acta Geologica Sinica (ICWEA)
2046	12TW4	113.96	43.67	CAOB	syenogranite	1399	11	600	-13.4	-13.7	2214	2231	Sun LX et al., 2018 Acta Geologica Sinica (ICWEA)
2047	XJ11-143	94.01	41.01	CAOB	granite	436	2	436	5.7	5.9	999	988	Li XF et al., 2015 Acta Petrologica Sinica (ICWEA)
2048	XJ11-140	94.01	41.01	CAOB	diorite	437	2	437	6.7	6.1	940	976	Li XF et al., 2015 Acta Petrologica Sinica (ICWEA)
2049	3005	90.23	46.90	CAOB	granite	381	4	381	5.2	5.8	984	946	Ning DX et al., 2017 Acta Geologica Sinica (ICWEA)
2050	A14830-1	90.71	46.83	CAOB	granite	385	5	385	8.4	8.8	801	778	Ning DX et al., 2017 Acta Geologica Sinica (ICWEA)
2051	A14829-2	90.21	47.26	CAOB	granite	382	4	382	6.5	6.3	907	922	Ning DX et al., 2017 Acta Geologica Sinica (ICWEA)
2052	380205	90.84	46.63	CAOB	granite	363	6	363	3.7	6.8	1054	874	Ning DX et al., 2017 Acta Geologica Sinica (ICWEA)
2053	11CL902	81.90	42.22	CAOB	alkali-feldspar granite	281	2.6	281	0.2	0.0	1189	1202	Liu CH et al., 2014 Acta Petrologica Sinica (ICWEA)
2054	11CL903	81.91	42.22	CAOB	alkali-feldspar granite	281	2.2	281	0.5	-0.1	1172	1205	Liu CH et al., 2014 Acta Petrologica Sinica (ICWEA)
2055	11CL904	81.91	43.22	CAOB	syenite	292	2.9	292	2.7	3.3	1057	1023	Liu CH et al., 2014 Acta Petrologica Sinica (ICWEA)
2056	13AX133	102.93	40.61	CAOB	gneiss	1459	11	600	-14.8	-14.8	2293	2296	Shi XJ et al., 2016 Acta Petrologica Sinica (ICWEA)
2057	14AX38	103.16	40.66	CAOB	gneiss	1433	17	600	-13.9	-14.5	2242	2278	Shi XJ et al., 2016 Acta Petrologica Sinica (ICWEA)
2058	NM1132	110.92	41.13	CAOB	gneissic trondhjemite	2692	17	600	-39.0	-39.1	3652	3653	Ma MZ et al., 2013 Acta Petrologica Sinica (ICWEA)
2059	B1001	110.94	41.10	CAOB	gneissic trondhjemite	2697	11	600	-40.2	-40.1	3716	3711	Ma MZ et al., 2013 Acta Petrologica Sinica (ICWEA)
2060	17YH4-10	129.03	48.42	CAOB	gneissic monzonitic granite	479	2.8	479	-8.6	-8.3	1851	1833	Guo XW et al., 2019 Global Geology (ICWEA)
2061	BS041	98.40	41.95	CAOB	gneissic granite	423	4	423	-6.9	-7.0	1708	1712	Liu XM et al., 2010 Geological Bulletin of China (ICWEA)
2062	PM11-22-TW1	80.61	44.91	CAOB	gneissic quartz diorite	468	5.4	468	5.6	5.7	1028	1021	Jia YG et al., 2019 Journal of Jilin University(Earth Science Edition) (ICWEA)
2063	D1073-TW1	80.61	44.91	CAOB	gneissic quartz diorite	455	5	455	5.6	5.9	1018	1001	Jia YG et al., 2019 Journal of Jilin University(Earth Science Edition) (ICWEA)
2064	12TW3	113.97	43.64	CAOB	gneissic syenogranite	1392	16	600	-14.6	-14.7	2284	2291	Sun LX et al., 2018 Acta Geologica Sinica (ICWEA)
2065	17YH4-4	129.03	48.42	CAOB	gneissic syenogranite	499	1.7	499	-7.8	-7.7	1818	1812	Guo XW et al., 2019 Global Geology (ICWEA)
2066	3422.1	99.92	41.18	CAOB	quartz diorite	420	1.8	420	9.6	9.8	757	748	Tian J et al., 2020 Geological Survey and Research (ICWEA)
2067	3424.1	99.92	41.18	CAOB	granodiorite	404	2.6	404	12.4	12.2	586	595	Tian J et al., 2020 Geological Survey and Research (ICWEA)
2068	B12-11	97.12	42.12	CAOB	granodiorite	285	1.5	285	10.9	11.1	577	567	Zheng RG et al., 2016 Acta Geologica Sinica (ICWEA)
2069	B12-17	97.08	42.12	CAOB	potassium granite	328	1.6	328	8.1	8.4	772	757	Zheng RG et al., 2016 Acta Geologica Sinica (ICWEA)
2070	14CL150-9	80.00	30.00	CAOB	monzonitic granite	456	7.2	456	-5.8	-5.9	1673	1678	Wang N et al., 2016 Acta Petrologica Sinica (ICWEA)
2071	HH4309	119.98	48.28	CAOB	monzonitic granite	179	0.5	179	8.1	8.6	656	629	Guo ZJ et al., 2014 Geology in China (ICWEA)
2072	SQZ99	94.79	41.90	CAOB	dioritic porphyrite	323	1.7	323	9.2	10.9	703	607	Xu LL et al., 2014 Geology in China (ICWEA)
2073	IILM84	114.30	45.33	CAOB	dioritic porphyrite	312	1.6	312	4.3	4.2	981	986	Lv YH et al., 2022 Geoscience (ICWEA)
2074	XA3-3-210	123.50	50.70	CAOB	dioritic porphyrite	124	1	150	7.6	7.4	663	671	Zhang C et al., 2013 Acta Petrologica Sinica (ICWEA)
2075	L08WQ14	81.14	44.93	Tianshan	diorite	462	0	462	4.9	4.8	1064	1078	Huang ZY et al,2013, Lithos
2076	798-2	87.20	42.83	Tianshan	diorite	366	0	366	3.8	4.1	1052	1040	Ma XX et al, 2014, Gondwana Research
2077	753	88.47	43.20	Tianshan	diorite	281	0	281	12.5	12.9	484	459	Ma XX et al, 2013, Gondwana Research
2078	769	88.39	42.28	Tianshan	diorite	290	0	290	-0.3	-0.3	1228	1232	Ma XX et al, 2013, Gondwana Research
2079	773	88.39	42.28	Tianshan	diorite	306	0	306	9.5	9.5	669	675	Ma XX et al, 2013, Gondwana Research
2080	12LS160	103.05	40.08	CAOB	diorite	249	1	249	4.6	5.1	914	883	Shi XJ et al., 2015 Acta Petrologica Et Mineralogica (ICWEA)
2081	2P4B40-1	110.92	41.07	CAOB	granite	2558	17	600	-40.4	-40.1	3727	3709	Ma MZ et al., 2013 Acta Petrologica Sinica (ICWEA)
2082	MZS9-1	95.88	41.58	CAOB	quartz porphyry	351	0.6	351	-0.1	0.1	1266	1250	Wang QS et al., 2019 Acta Petrologica Sinica (ICWEA)
2083	DS1	110.17	40.12	CAOB	quartz porphyry	234	3	234	-14.2	-14.9	1977	2016	Wu H et al., 2018 Mineral Deposits (ICWEA)
2084	13DZ06	89.88	45.67	CAOB	quartz monzodiorite	433	1.2	433	12.2	12.2	621	618	Huang G et al., 2016 Acta Petrologica Et Mineralogica (ICWEA)
2085	HH5503	119.98	48.28	CAOB	quartz monzonite	179	0.7	179	9.2	8.8	589	614	Guo ZJ et al., 2014 Geology in China (ICWEA)
2086	JH-04	90.13	45.22	CAOB	quartz monzonite	308	0.85	308	12.9	13.0	477	475	Geng YY et al., 2022 Journal of Earth Sciences and Environment (ICWEA)
2087	BT-03	90.53	45.27	CAOB	quartz monzonite	321	1.6	321	12.5	12.4	514	520	Geng YY et al., 2022 Journal of Earth Sciences and Environment (ICWEA)
2088	SW-4	83.23	45.92	CAOB	quartz diorite porphyrite	322	1.5	322	13.8	13.6	437	452	Hu Y et al., 2018 Acta Petrologica Sinica (ICWEA)
2089	xj373	86.43	41.29	Tianshan	quartz diorite	1944	0	600	-35.3	-35.4	3447	3450	Lei RX et al, 2012, International Geology Review
2090	xj481	86.43	41.29	Tianshan	quartz diorite	1944	0	600	-36.4	-36.7	3508	3525	Lei RX et al, 2012, International Geology Review
2091	L08WQ20	81.14	44.93	Tianshan	quartz diorite	462	0	462	2.2	2.2	1222	1224	Huang ZY et al,2013, Lithos
2092	L08WQ51	80.49	44.90	Tianshan	quartz diorite	463	0	463	1.4	1.6	1272	1251	Huang ZY et al,2013, Lithos
2093	BLT-10	86.31	42.74	Tianshan	quartz diorite	372	0	372	-14.6	-14.4	2157	2146	Huang H et al., 2015 Acta Geologica Sinica (ICWEA)
2094	WL89-1	115.49	42.81	CAOB	quartz diorite	308	4.2	308	4.2	4.0	985	992	Wang XY et al., 2013 Geoscience (ICWEA)
2095	2P3B14-2	110.66	41.29	CAOB	quartz diorite	2495	0	600	-40.1	-40.2	3711	3714	Ma MZ et al., 2013 Acta Petrologica Sinica (ICWEA)
2096	SW-14	83.23	45.92	CAOB	quartz diorite	322	1.5	322	13.8	13.3	436	470	Hu Y et al., 2018 Acta Petrologica Sinica (ICWEA)

2097	PM2	86.93	42.50	CAOB	quartz diorite	453	1.4	453	-12.5	-12.2	2049	2031	Li P et al., 2017 Acta Geologica Sinica (ICWEA)
2098	X10-16-1	94.90	41.80	Beishan	Quartz diorite gneiss	430	0	430	0.0	4.0	1311	1088	He ZY et al., 2012 Acta Petrologica Sinica (ICWEA)
2099	X10-16-3	94.84	41.76	CAOB	Quartz diorite gneiss	425	9	425	3.6	14.5	1100	479	He ZY et al., 2012 Acta Petrologica Sinica (ICWEA)
2100	15SL-YQ	91.91	41.95	CAOB	quartz diorite	301	1.2	301	8.8	13.2	709	457	Guo YX et al., 2019 Xinjiang Geology (ICWEA)
2101	FS-7	96.94	41.19	CAOB	acid volcanic rocks	273	1	273	0.6	1.7	1162	1101	Zheng RG et al., 2016 Geotectonica Et Metallogenia (ICWEA)
2102	YMQ01	90.25	45.59	CAOB	quartz diorite	443	2.5	443	12.4	12.5	618	609	Huang G et al., 2016 Acta Petrologica Et Mineralogica (ICWEA)
2103	TP818	119.92	51.13	CAOB	Granite prophyry	184	2.7	184	-1.1	-1.2	1190	1198	Wang ZL et al., 2010 Acta Petrologica Et Mineralogica (ICWEA)
2104	TP820	119.92	51.13	CAOB	Granite prophyry	194	3.4	194	-1.6	-1.1	1223	1195	Wang ZL et al., 2010 Acta Petrologica Et Mineralogica (ICWEA)
2105	TP848	119.92	51.13	CAOB	Granite prophyry	199	3.4	199	-2.0	-2.5	1253	1279	Wang ZL et al., 2010 Acta Petrologica Et Mineralogica (ICWEA)
2106	Z005	97.21	42.10	CAOB	granite	427	2.5	427	-0.1	0.3	1320	1301	Bai RL et al., 2020 Acta Petrologica Sinica (ICWEA)
2107	ZT001	96.82	41.87	CAOB	granite	410	2.8	410	-1.7	-1.7	1402	1399	Bai RL et al., 2020 Acta Petrologica Sinica (ICWEA)
2108	20TL	91.12	41.83	CAOB	syenogranite	283	1.3	283	11.2	11.3	560	554	Chen BX et al., 2018 Chinese Journal of Geology (ICWEA)
2109	TM05	83.08	43.50	CAOB	andesite	321	2.5	321	10.6	10.7	621	619	Wang DC et al., 2016 Acta Petrologica Sinica (ICWEA)
2110	TM06	83.08	43.50	CAOB	trachyandensite	319	2.3	319	9.8	10.0	665	657	Wang DC et al., 2016 Acta Petrologica Sinica (ICWEA)
2111	TM12	83.08	43.50	CAOB	rhyolite	319	3.5	319	-6.7	-6.7	1618	1618	Wang DC et al., 2016 Acta Petrologica Sinica (ICWEA)
2112	15HTTSM-9	110.33	40.42	CAOB	volcanic rock	305	3.7	305	-6.4	-6.4	1587	1588	Zhang Q et al., 2018 Geological Journal of China Universities (ICWEA) (ICWEA)
2113	ZK005-2	89.09	44.84	CAOB	quartz diorite porphyrite	378	2	378	13.8	13.7	484	486	Zhou HZ et al.,2018 Earth Science (ICWEA)
2114	N2861	85.50	46.68	CAOB	granite	444	2.2	444	11.2	11.3	684	679	Jin S et al., 2015 Geology in China (ICWEA)
2115	J7	95.56	42.53	CAOB	granite	223	1.3	223	9.3	9.1	621	631	Li JB et al., 2020 Geochimica (ICWEA)
2116	J12	95.56	42.53	CAOB	granite	224	1.5	224	9.0	9.2	640	629	Li JB et al., 2020 Geochimica (ICWEA)
2117	J17	95.56	42.53	CAOB	granite	218	1.8	218	11.4	11.4	494	493	Li JB et al., 2020 Geochimica (ICWEA)
2118	JN2013-10	113.12	42.39	CAOB	Granite prophyry	241	1	241	4.2	4.5	931	911	Feng F et al., 2021 Earth Science (ICWEA)
2119	XM3-129	85.59	46.64	CAOB	granite	422	2	422	12.8	12.6	574	589	Wang M et al., 2018 Acta Petrologica Sinica (ICWEA)
2120	XM2-49	85.59	46.64	CAOB	granite	412	1.7	412	10.9	11.8	677	626	Wang M et al., 2018 Acta Petrologica Sinica (ICWEA)
2121	12XL01	89.43	46.89	CAOB	monzonitic granite	324	2.4	324	12.8	13.0	497	485	You J et al., 2017 Mineral Resources and Geology (ICWEA)
2122	JDTW02	97.58	41.48	CAOB	spilite	360	1.4	360	8.5	7.9	776	810	Pan ZL et al., 2017 Geological Survey and Research (ICWEA)
2123	ZJ-010-1	111.01	41.00	CAOB	phyritic alkali feldspar gra	765	5	600	-13.1	-12.5	2200	2162	Mi WT et al., 2020 Science Technology and Engineering (ICWEA)
2124	DA-N2	128.89	49.27	CAOB	syenogranite	184	2	184	1.0	1.1	1071	1060	i BL et al., 2016 Earth Science(Journal of China University of Geosciences) (ICWEA)
2125	14CL132-3	95.64	39.85	CAOB	granite	341	3	341	-7.9	-7.0	1701	1651	Wang N et al., 2016 Acta Petrologica Sinica (ICWEA)
2126	14CL133-3	95.64	39.85	CAOB	granite	340	3	340	-10.3	-10.2	1837	1834	Wang N et al., 2016 Acta Petrologica Sinica (ICWEA)
2127	14CL135-3	95.64	39.85	CAOB	granite	366	3	366	-13.6	-13.5	2046	2039	Wang N et al., 2016 Acta Petrologica Sinica (ICWEA)
2128	AQS07	90.85	41.80	CAOB	Granite prophyry	318	2.1	318	10.2	10.3	643	640	Wang JW et al., 2022 Earth Science (ICWEA)
2129	AQS08	90.85	41.80	CAOB	Granite prophyry	317	1.3	317	11.3	11.1	582	588	Wang JW et al., 2022 Earth Science (ICWEA)
2130	P2121	123.91	45.06	CAOB	trachyandensite	123	1.3	150	6.9	7.1	699	693	Liu C et al., 2017 Journal of Jilin University(Earth Science Edition) (ICWEA)
2131	HBS5309	98.35	41.60	Beishan	plagiogranite	536	0	536	16.9	16.7	536	536	Hou QY et al., 2012 Geoscience (ICWEA)
2132	100811	105.20	39.61	CAOB	plagioclase amphibolite	2082	22	600	-33.7	-33.7	3355	3355	Bao C et al., 2013 Geological Bulletin of China (ICWEA)
2133	BN-Ⅱ08	112.05	42.02	CAOB	plagioclase amphibolite	449	3	449	0.8	2.9	1289	1168	Gao X et al., 2018 Mineral Deposits (ICWEA)
2134	BN-X-A06	112.05	42.02	CAOB	plagioclase amphibolite	493	2.9	493	2.7	2.7	1217	1217	Gao X et al., 2018 Mineral Deposits (ICWEA)
2135	15ZB20-3	107.51	41.17	CAOB	plagioclase amphibolite	2544	22	600	-38.8	-39.0	3641	3648	Duan RH et al., 2021 Acta Petrologica Sinica (ICWEA)
2136	N4814-2	84.88	46.71	CAOB	alkaline granite	428	2.3	428	13.2	13.2	559	558	Yang G et al., 2015 Earth Science (ICWEA)
2137	x640	93.93	41.91	CAOB	granite	228	0.47	228	10.0	10.0	585	585	Lei RX et al., 2011 Acta Petrologica Sinica (ICWEA)
2138	17YMS13	93.50	41.77	CAOB	rhyolite	250	1.4	250	10.3	10.4	581	577	Ding LL et al ., 2021 Mineral Exploration (ICWEA)
2139	17YMS01	93.88	41.88	CAOB	granite	315	3.6	315	5.9	7.3	888	808	Ding LL et al ., 2021 Mineral Exploration (ICWEA)
2140	W100914-8-1	83.76	45.43	CAOB	granodiorite	309	2.6	309	14.0	14.0	419	415	Xu SL et al., 2019 Geology in China (ICWEA)
2141	W100914-14-1	83.78	45.62	CAOB	alkaline granite	303	2.9	303	13.5	13.3	441	452	Xu SL et al., 2019 Geology in China (ICWEA)
2142	HZ-2-89	125.91	50.23	CAOB	dacite porphyry	481	2.6	481	13.3	13.4	594	588	Che HW et al., 2015 Acta Geologica Sinica (ICWEA)
2143	HZ-2-90	125.91	50.23	CAOB	dacite porphyry	481	3.3	481	13.0	14.1	610	550	Che HW et al., 2015 Acta Geologica Sinica (ICWEA)
2144	HZ-2-99	125.91	50.23	CAOB	dacite porphyry	478	3.1	478	13.3	13.3	593	590	Che HW et al., 2015 Acta Geologica Sinica (ICWEA)
2145	BJQ6-4-1	83.93	46.07	CAOB	dacite porphyry	606	1.3	600	23.8	23.8	606	606	Chen Y et al., 2020 Xinjiang Geology (ICWEA)
2146	YMS46	93.86	41.91	CAOB	dacite	334	1.7	334	5.3	5.7	939	919	Wang W et al., 2016 Acta Petrologica Et Mineralogica (ICWEA)
2147	NM10-01	112.97	42.27	CAOB	dacite	434	4.4	434	5.9	5.8	983	990	an XY et al., 2017 Earth Science(Journal of China University of Geosciences) (ICWEA)
2148	c层	87.61	43.14	CAOB	dacite	324	3.4	324	9.8	9.8	671	671	Xing JC et al., 2021 Xinjiang Geology (ICWEA)
2149	WN1402	118.75	43.83	CAOB	dacite	247	0.55	247	13.9	14.2	373	357	Li SC et al., 2020 Acta Petrologica Sinica (ICWEA)
2150	zk230-I	95.27	43.90	Eastern Junggar	tonalite	418	5	418	12.8	12.7	572	576	Li GF, 2015 master's thesis
2151	TW-005	92.60	42.08	CAOB	tonalite	335	3	335	12.3	11.4	534	586	Wang YH et al., 2014 Acta Petrologica Sinica (ICWEA)
2152	13ZB37-3	110.04	41.15	CAOB	tonalite	2546	14	600	-38.3	-37.9	3612	3590	Duan RH et al., 2021 Acta Petrologica Sinica (ICWEA)
2153	13ZB44-2	110.04	41.15	CAOB	tonalite	2503	5	600	-38.8	-39.0	3637	3648	Duan RH et al., 2021 Acta Petrologica Sinica (ICWEA)
2154	15ZB27-1	108.06	41.75	CAOB	plagiogneiss	2523	13	600	-38.5	-38.6	3622	3627	Duan RH et al., 2021 Acta Petrologica Sinica (ICWEA)
2155	S001-2	93.05	42.32	CAOB	porphyre	364	5	364	12.2	12.1	563	573	Wang YH et al., 2018 Acta Petrologica Sinica (ICWEA)
2156	S3601-16	93.05	42.32	CAOB	porphyre	306	5	306	11.7	11.7	548	545	Wang YH et al., 2018 Acta Petrologica Sinica (ICWEA)
2157	13XTS-114	81.90	44.57	CAOB	granite	309	9.3	309	5.4	5.7	914	897	Yang R et al., 2017 Geological Review (ICWEA)
2158	13XTS-108	81.90	44.57	CAOB	granite	301	2	301	6.5	6.4	846	847	Yang R et al., 2017 Geological Review (ICWEA)
2159	10SZ5-1	113.21	44.39	CAOB	rhyolite	300	2.9	300	12.0	12.0	523	528	He SS et al., 2015 Acta Scientiarum Naturalium Universitatis Pekinensis (ICWEA)
2160	10AB9-1	114.59	44.82	CAOB	ignimbrite	159	1.4	159	8.9	11.5	590	441	He SS et al., 2015 Acta Scientiarum Naturalium Universitatis Pekinensis (ICWEA)
2161	02TW1	98.50	42.08	CAOB	tonalite	311	1.4	311	11.6	11.9	559	540	Li M et al., 2018 Earth Science (ICWEA)
2162	14SHSS-Tw1	99.74	40.81	CAOB	rhyolite	295	2.1	295	3.3	6.9	1022	813	Xu W et al., 2018 Acta Petrologica Sinica (ICWEA)
2163	14SHJ-Tw2	97.40	41.04	CAOB	rhyolite	291	2.6	291	2.6	3.5	1062	1011	Xu W et al., 2018 Acta Petrologica Sinica (ICWEA)
2164	14YJZ-Tw2	98.65	40.55	CAOB	rhyolite	273	2.6	273	1.0	2.9	1135	1031	Xu W et al., 2018 Acta Petrologica Sinica (ICWEA)
2165	16DS-Tw5	94.01	40.55	CAOB	rhyolite	281	2.9	281	13.9	14.0	402	391	Xu W et al., 2018 Acta Petrologica Sinica (ICWEA)
2166	13GQ-Tw1	94.33	40.73	CAOB	rhyolite	294	4.8	294	-25.7	5.1	2497	920	Xu W et al., 2018 Acta Petrologica Sinica (ICWEA)
2167	TW6241-1	95.84	41.21	CAOB	syenogranite	289	1.4	289	5.7	5.6	880	886	Sun HR et al., 2020 Journal of Jilin University(Earth Science Edition) (ICWEA)
2168	BL2	90.13	45.14	Eastern Junggar	syenogranite	300	0	300	12.6	12.6	491	490	Han YJ et al., 2012
2169	Z011	96.66	42.23	CAOB	syenogranite	318	2.5	318	8.0	7.9	772	774	Bai RL et al., 2020 Acta Petrologica Sinica (ICWEA)
2170	HDM-N3	125.01	43.00	CAOB	syenogranite	171	1.5	171	7.9	7.9	662	659	Zhang LS et al., 2021 Acta Petrologica Sinica (ICWEA)
2171	H16G01	90.00	42.00	CAOB	syenogranite	272	3.1	272	8.9	9.6	680	644	Wang XD et al., 2019 Bulletin of Mineralogy, Petrology and Geochemistry (ICWEA)
2172	M1498-3.1	110.43	43.89	CAOB	syenogranite	337	4	337	13.3	13.9	481	443	Zhang XW et al., 2021 Acta Petrologica Et Mineralogica (ICWEA)
2173	M1498-1.2	110.42	43.88	CAOB	syenogranite	332	2	332	12.4	12.8	526	505	Zhang XW et al., 2021 Acta Petrologica Et Mineralogica (ICWEA)
2174	DS12	110.17	40.12	CAOB	syenogranite	237	4	237	-10.3	-9.8	1753	1716	Wu H et al., 2018 Mineral Deposits (ICWEA)
2175	TW.05.4	121.13	45.28	CAOB	syenogranite	213	10.11	213	9.3	10.5	610	543	Chen LL et al., 2016 Geology in China (ICWEA)
2176	TW.06.2	120.32	45.28	CAOB	syenogranite	130	7.82	150	8.1	8.4	630	616	Chen LL et al., 2016 Geology in China (ICWEA)
2177	BZY-2	84.00	43.17	CAOB	syenogranite	321	3.6	321	10.6	10.4	623	636	Xu SL et al., 2022 Geological Bulletin of China (ICWEA)
2178	YMS32	93.86	41.91	CAOB	syenite	326	1.7	326	9.1	9.0	715	720	Wang W et al., 2016 Acta Petrologica Et Mineralogica (ICWEA)
2179	BLTIV-9	86.12	42.90	CAOB	granodiorite	306	4.3	306	3.8	4.4	1005	970	Huang H et al., 2015 Acta Geologica Sinica (ICWEA)
2180	WLSTIII-6	86.55	43.88	CAOB	alkaline granite	312	4	312	5.4	5.3	918	925	Huang H et al., 2015 Acta Geologica Sinica (ICWEA)

2181	TH1-1	94.45	41.67	CAOB	orphyry biotite monzogran	244	2.6	244	2.4	2.9	1032	1005	Zhao HG et al., 2017 Acta Geologica Sinica (ICWEA)
2182	TW6240-1	95.01	41.00	CAOB	biotite monzonite granite	368	1.8	368	0.6	0.8	1233	1226	Lv X et al., 2020 Acta Petrologica Sinica (ICWEA)
2183	TW6239-1	95.01	41.00	CAOB	biotite granodiorite	368	1.7	368	3.4	3.7	1074	1055	Lv X et al., 2020 Acta Petrologica Sinica (ICWEA)
2184	KST-CN1	89.25	45.20	CAOB	biotite granite	328	2.3	328	13.1	13.1	484	483	Feng HQ et al., 2019 Bulletin of Mineralogy, Petrology and Geochemistry (ICWEA)
2185	14CL151-3	78.00	30.01	CAOB	monzonitic granite	442	2.6	442	-5.4	-5.4	1636	1635	Wang N et al., 2016 Acta Petrologica Sinica (ICWEA)
2186	H710-4.1	95.02	44.09	Eastern Junggar	potassium granite	309	1.3	309	12.5	12.4	501	508	Zhao JX, 2014 master's thesis
2187	YH1-1	93.93	41.87	CAOB	monzonitic granite	231	2.1	231	9.0	8.7	643	664	Zhao HG et al., 2018 Acta Geologica Sinica (ICWEA)
2188	YH1-4	93.79	42.03	CAOB	monzonitic granite	227	0.9	227	9.4	9.3	619	624	Zhao HG et al., 2018 Acta Geologica Sinica (ICWEA)
2189	YH2-2	93.81	42.04	CAOB	monzonitic granite	229	1.9	229	10.0	9.8	587	597	Zhao HG et al., 2018 Acta Geologica Sinica (ICWEA)
2190	YH3-1	93.76	42.04	CAOB	monzonitic granite	231	2.1	231	9.7	10.0	605	585	Zhao HG et al., 2018 Acta Geologica Sinica (ICWEA)
2191	KB2	91.50	41.63	CAOB	diorite	375	1	375	5.5	5.3	962	974	Ni A et al., 2019 Journal of Jilin University(Earth Science Edition) (ICWEA)
2192	TW6907-1	106.67	41.23	CAOB	granodiorite	278	0.66	278	-2.3	-2.1	1333	1320	Liu Y et al., 2021 Advances in Earth Science (ICWEA)
2193	B9009	110.76	41.05	CAOB	granulite	2507	0	600	-39.6	-40.0	3684	3705	Ma MZ et al., 2013 Acta Petrologica Sinica (ICWEA)
2194	B9007	110.90	40.93	CAOB	granulite	2533	0	600	-39.6	-39.4	3685	3671	Ma MZ et al., 2013 Acta Petrologica Sinica (ICWEA)
2195	TW6237-3	95.01	41.00	CAOB	biotite granodiorite	366	2.2	366	4.5	4.6	1013	1005	Lv X et al., 2020 Acta Petrologica Sinica (ICWEA)
2196	TW6477-1	106.75	41.18	CAOB	granodiorite	278	0.66	278	-5.1	-4.9	1494	1480	Liu Y et al., 2021 Advances in Earth Science (ICWEA)
2197	0029-2	121.18	48.06	CAOB	syenogranite	334	5	334	9.3	9.6	708	689	Zhang J et al., 2022 Global Geology (ICWEA)
2198	TH2-3	94.50	41.65	CAOB	biotite granodiorite	242	2.4	242	3.4	3.4	976	975	Zhao HG et al., 2017 Acta Geologica Sinica (ICWEA)
2199	SJ150731-6-1	83.63	45.79	CAOB	rhyolite	295	1.9	295	13.6	13.7	426	421	Xu SL et al., 2019 Geology in China (ICWEA)
2200	SJ150731-6-2	83.63	45.79	CAOB	rhyolite	311	1.9	311	14.1	14.5	413	389	Xu SL et al., 2019 Geology in China (ICWEA)
2201	SJ150803-1-3	83.58	45.87	CAOB	rhyolite	298	1.6	298	14.3	14.6	387	374	Xu SL et al., 2019 Geology in China (ICWEA)
2202	SJ150803-1-4	83.58	45.87	CAOB	rhyolite	295	1.9	295	14.2	14.4	395	382	Xu SL et al., 2019 Geology in China (ICWEA)
2203	SJ150803-2-1	83.58	45.92	CAOB	rhyolite	304	1.7	304	12.3	12.6	512	495	Xu SL et al., 2019 Geology in China (ICWEA)
2204	SJ150803-3-1	83.59	45.79	CAOB	rhyolite	302	1.9	302	12.4	12.0	504	525	Xu SL et al., 2019 Geology in China (ICWEA)
2205	B9008	110.76	40.93	CAOB	dioritic gneiss	2506	9	600	-38.7	-38.6	3636	3628	Ma MZ et al., 2013 Acta Petrologica Sinica (ICWEA)
2206	SMZG-01	127.92	43.08	in the Songliao Block, NE China	Granodiorite	263	1	263	-0.8	-0.9	1233	1239	Wu T et al., 2016 Lithos
2207	L30-1	106.15	40.57	in the Langshan	Syeno granite	280	1.9	280	-7.3	-7.0	1617	1604	Zheng et al., 2021 Journal of Asian Earth Sciences
2208	Rg50-2	106.37	40.65	in the Langshan	monzogranodiorite	268	1.8	268	-9.1	-8.8	1716	1699	Zheng et al., 2021 Journal of Asian Earth Sciences
2209	D301-TW1	123.50	42.65	north Liaoning, NE China	andesite	370	2	370	11.4	11.1	613	634	Shi Y et al., 2022 Gondwana Research
2210	BJGC-TW1	123.66	42.58	north Liaoning, NE China	Granite	248	1.5	248	5.5	5.0	862	891	Shi Y et al., 2022 Gondwana Research
2211	18KY008	124.21	48.29	southeastern Central Asian Orogenic Belt	Granite	243	3.1	243	-0.5	-0.1	1200	1178	Jing Y et al., 2022 Gondwana Research
2212	18KY011	124.21	48.29	southeastern Central Asian Orogenic Belt	Granite	243	1.6	243	-0.1	-0.1	1179	1179	Jing Y et al., 2022 Gondwana Research
2213	18KY015	124.21	48.29	southeastern Central Asian Orogenic Belt	Granite	243	1.6	243	-1.7	-1.6	1273	1264	Jing Y et al., 2022 Gondwana Research
2214	18KY029	124.21	48.29	southeastern Central Asian Orogenic Belt	Granite	243	1.9	243	-1.3	-1.3	1249	1248	Jing Y et al., 2022 Gondwana Research
2215	16JL08B	126.31	43.68	e southeastern margin of the Centra	Tonalite	486	0	486	11.9	12.1	681	666	Ma HT et al., 2020 Journal of Asian Earth Sciences
2216	11LS88	104.50	41.12	Alxa	Granodiorite	301	3	301	8.6	8.7	721	716	Shi X et al., 2014 Lithos
2217	11LS106	104.25	40.68	Alxa	Granodiorite	264	3	264	10.2	10.1	640	644	Shi X et al., 2014 Lithos
2218	11LS96	104.33	40.68	Alxa	Granodiorite	266	2	266	1.3	1.2	1207	1219	Shi X et al., 2014 Lithos
2219	11LS91	104.42	40.68	Alxa	Granodiorite	254	2	254	6.0	6.0	896	900	Shi X et al., 2014 Lithos
2220	11LS170	104.25	40.68	Alxa	Granodiorite	264	2	264	5.8	5.8	910	913	Shi X et al., 2014 Lithos
2221	11LS175	104.90	40.68	Alxa	Granodiorite	251	2	251	7.1	7.1	821	820	Shi X et al., 2014 Lithos
2222	11LS122	104.45	40.68	Alxa	Granodiorite	250	2	250	6.6	6.3	854	880	Shi X et al., 2014 Lithos
2223	13GW104	132.09	46.04	Jiamusi	granite	272	3	272	2.6	2.4	1046	1055	Bi JH et al., 2016 Lithos
2224	13GW564	132.43	46.26	Jiamusi	granite	267	2	267	4.1	3.9	956	969	Bi JH et al., 2016 Lithos
2225	13GW554	130.68	46.31	Jiamusi	granite	301	2	301	0.7	0.4	1179	1196	Bi JH et al., 2016 Lithos
2226	13GW096	132.67	46.11	Jiamusi	granite	282	2	282	-0.3	-0.2	1218	1212	Bi JH et al., 2016 Lithos
2227	13GW560	132.62	46.17	Jiamusi	granite	302	3	302	0.4	0.2	1193	1208	Bi JH et al., 2016 Lithos
2228	13GW090	132.59	48.49	Jiamusi	granite	281	2	281	-0.8	-1.1	1248	1266	Bi JH et al., 2016 Lithos
2229	13GW039	132.92	46.05	Jiamusi	granite	322	9	322	-0.3	0.0	1253	1234	Bi JH et al., 2016 Lithos
2230	13GW110	131.94	46.10	Jiamusi	granite	275	9	275	5.4	3.7	889	983	Bi JH et al., 2016 Lithos
2231	EL14-18-1	111.21	43.13	Central Inner Mongolia	Granite	141	1	150	6.1	6.3	746	734	Yuan L et al., 2019 Lithos
2232	EL12-1-1	111.23	43.13	Central Inner Mongolia	Granite	141	1	150	6.4	6.6	731	721	Yuan L et al., 2019 Lithos
2233	EL12-1-6	111.23	43.13	Central Inner Mongolia	Granite	143	1	150	6.4	6.3	728	735	Yuan L et al., 2019 Lithos
2234	EL12-3-1	110.22	43.10	Central Inner Mongolia	Granite	141	5	150	6.7	6.7	715	710	Yuan L et al., 2019 Lithos
2235	EL14-15-1	111.24	43.17	Central Inner Mongolia	Granite	142	1	150	5.1	5.2	803	799	Yuan L et al., 2019 Lithos
2236	D0925	105.00	46.15	Mongol-Okhotsk Ocean	Granodiorite	296	3	296	-1.7	-2.0	1311	1332	Ariuntsetseg G et al., 2021 Lithos
2237	D1710	104.13	47.07	Mongol-Okhotsk Ocean	Syenogranite	282	3	282	9.0	8.9	682	688	Ariuntsetseg G et al., 2021 Lithos
2238	D1709	104.77	47.08	Mongol-Okhotsk Ocean	Rhyolite	274	2	274	7.2	7.6	782	757	Ariuntsetseg G et al., 2021 Lithos
2239	D1742	104.62	46.67	Mongol-Okhotsk Ocean	Monzogranite	240	3	240	5.4	5.3	857	864	Ariuntsetseg G et al., 2021 Lithos
2240	D1718	104.04	47.14	Mongol-Okhotsk Ocean	Monzogranite	230	1.6	230	4.0	4.1	934	928	Ariuntsetseg G et al., 2021 Lithos
2241	D1726	104.34	46.94	Mongol-Okhotsk Ocean	Granodiorite	220	1	220	4.5	4.4	894	899	Ariuntsetseg G et al., 2021 Lithos
2242	SDHS-6	96.00	42.63	Xinjiang Province of northwestern China	Granodiorite	427	3	427	14.7	14.4	466	488	He XH et al., 2021 Ore Geology Reviews
2243	SDHS-3	96.00	42.62	Xinjiang Province of northwestern China	Monzogranite	382	4	382	8.4	8.4	796	796	He XH et al., 2021 Ore Geology Reviews
2244	ZK8202-7	96.05	42.50	eastern Tianshan	Monzogranite	238	5	238	10.9	10.9	539	540	He XH et al., 2021 Ore Geology Reviews
2245	ZK8601-3	96.01	42.51	eastern Tianshan	Monzogranite	234	3	234	10.4	10.2	566	577	He XH et al., 2021 Ore Geology Reviews
2246	ZK8202-6	96.05	42.50	eastern Tianshan	Monzogranite porphyry	232	3	232	10.8	10.4	538	566	He XH et al., 2021 Ore Geology Reviews
2247	HL-1-RR	96.06	42.51	eastern Tianshan	Porphyritic granite	232	3	232	10.5	10.4	561	562	He XH et al., 2021 Ore Geology Reviews
2248	DWQ2-TW	115.02	45.07	Xilinhot	Rhyolite	316	2	316	8.2	8.3	757	753	Li J et al., 2021 Lithos
2249	XWQ6-TW	117.02	44.04	Xilinhot	Andesite	262	5	262	-5.6	3.5	1505	986	Li J et al., 2021 Lithos
2250	DWQ5-TW	115.01	45.06	Xilinhot	Alkali-feldspar granite	300	1	300	11.8	11.7	537	542	Li J et al., 2021 Lithos
2251	MD1-TW	116.98	44.02	Xilinhot	Andesite	306	2	306	7.2	15.2	800	344	Li J et al., 2021 Lithos
2252	H19HX7	118.64	44.75	central Inner Mongolia	Pyroxene diorite	281	0.6	281	9.6	9.7	651	646	Zhang X et al., 2021 Journal of Asian Earth Sciences
2253	H19HX15	118.64	44.75	central Inner Mongolia	Alkali feldspar granite	281	0.6	281	9.3	9.3	669	667	Zhang X et al., 2021 Journal of Asian Earth Sciences

Table S2b. Zircon Lu-Hf isotope data of the mafic rocks of the Altaids.

Sample	Longitude	Latitude	Regions	Rock type	Age (Ma)	Error	Recalculated-Age (Ma)	$\varepsilon_{Hf}(t)$_Average	$\varepsilon_{Hf}(t)$_Median	T_{DM2}_Average	T_{DM2}_Median	T_{DM1}_Average	T_{DM1}_Median	Reference
YT	106.66	41.26	North China Craton	Gabbro diorite	274	0	274	-6.6	-6.1	1573	1548	1173	1153	Pi QH et al., 2010 Mineral Deposits
14HS27-1	130.55	42.95	0	Basalt, Andesite	108	1	150	9.3	9.4	560	557	432	429	Wang F et al., 2019 Lithos
HSW11-1	127.08	49.94	0	Basaltic andesite	242	4	242	8.7	8.5	672	681	546	551	Li Y et al., 2017 Gondwana Research
14DX7-1	122.45	48.11	0	Basaltic andesite	228	2	228	14.5	14.6	321	314	293	289	Li Y et al., 2017 Gondwana Research
15DX4-1	122.42	47.51	0	Basaltic andesite	228	2	228	9.6	9.4	610	616	490	493	Li Y et al., 2017 Gondwana Research
P2121-1	123.94	50.20	0	Basaltic ignimbrite	123	1	150	6.9	7.1	699	693	533	529	Liu C et al., 2017 Journal of Jilin University (Earth Science Edition) (ICWEA)
M133-1	116.68	49.60	0	Basaltic trachyandes	164	1	164	1.5	1.2	1023	1041	759	768	Zhao ZH et al., 2011 Journal of Jilin University (Earth Science Edition) (ICWEA)
14HS11-1	130.89	43.74	0	Baslat	105	2	150	9.1	9.9	574	530	446	413	Wang F et al., 2019 Lithos
WJ1122	85.54	46.69	0	Gabbro	420	3	420	13.0	13.1	562	557	522	518	Yin et al., 2017 Gondwana Research
06XJ143	84.45	45.50	0	Gabbro	332	4.3	332	13.1	13.3	488	474	441	432	Tang et al., 2019 Earth and Planetary Science Letters
08AET68	89.18	46.30	0	Gabbro	314	4.2	314	9.0	11.7	712	551	589	480	Tang et al., 2019 Earth and Planetary Science Letters
P18YQ1	86.50	47.50	0	Gabbro	364	1	364	14.1	13.9	456	467	428	435	Jin et al., 2015 Chemical Geology
13ER34-1	120.86	52.04	0	Gabbro	205	1	205	-0.1	0.2	1150	1130	859	848	Tang J et al., 2016 Gondwana Research
13ER8-1	121.54	51.67	0	Gabbro	204	1	204	-0.3	-0.4	1162	1164	877	868	Tang J et al., 2016 Gondwana Research
LK07-1	124.17	42.64	0	Gabbro	175	1	175	6.7	6.2	735	761	561	576	Zhang HH et al., 2016 Lithos
LK11-1	124.34	42.34	0	Gabbro	163	3	163	-19.1	-17.9	2192	2136	1571	1531	Zhang HH et al., 2016 Lithos
15XH7-1	129.47	45.82	0	Gabbro	211	2	211	4.0	4.1	915	912	698	697	Long XY et al., 2020 Gondwana Research
15XH7-5	129.47	45.82	0	Gabbro	210	2	210	4.0	4.3	915	900	698	686	Long XY et al., 2020 Gondwana Research
XW-07	98.65	52.80	East Kunlun Orogenic Belt	Gabbro	251	0.8	251	9.5	9.8	630	615	516	506	Zhang JD et al, 2021 Frontiers in Earth Science
XW-09	98.70	52.70	East Kunlun Orogenic Belt	Gabbro	257	0.8	257	8.3	7.8	704	737	561	584	Zhang JD et al, 2021 Frontiers in Earth Science
PYG	91.59	40.59	NW China	Gabbro	276	0	276	6.4	6.3	831	839	655	661	Xue SC et al., 2018 Contributions to Mineralogy and Petrology
12GW007	131.34	45.03	Jiamusi	Gabbro	501	4	501	4.5	4.4	1119	1122	926	930	Yang H et al., 2014 Lithos
DX03－1	117.94	43.68	0	Gabbro diorite	146	1	150	7.6	7.6	660	659	501	502	Che YW et al., 2021 Geological Bulletin of China (ICWEA)
13ER13-6	122.09	52.06	0	Gabbro-diorite	204	2	204	4.5	5.7	884	813	729	671	Tang J et al., 2016 Gondwana Research
12ER30-5	122.57	52.42	0	Gabbro-diorite	200	1	200	4.2	4.3	895	892	685	683	Tang J et al., 2016 Gondwana Research
14JEW11-1	129.10	42.64	0	Gabbro-diorite	185	1	185	9.1	9.2	600	594	480	477	Wang F et al., 2017 Journal of Asian Earth Sciences
14JEW12-3	129.07	42.59	0	Gabbro-diorite	183	1	183	2.7	2.6	970	975	735	729	Wang F et al., 2017 Journal of Asian Earth Sciences
HWC1-1	127.23	45.14	0	Gabbro-diorite	183	1	183	9.6	9.4	573	583	458	464	Yu JJ et al., 2012 Lithos
12GW019	131.52	45.14	0	Gabbroic diorite	213	2	213	5.2	5.0	851	862	669	667	Yang H et al., 2015 Lithos
NM07-56	110.72	41.03	North China Craton	Gabbroic diorite	277	0	277	-7.9	-7.9	1654	1654	1222	1225	Zhang XH et al., 2011 Lithos
PD-4	91.73	40.64	the Central Asian Orogenic Belt	Gabbronorite	270	0	270	9.3	9.0	659	674	547	559	Xue SC et al., 2018 Contributions to Mineralogy and Petrology
BCD 1	82.09	42.23	Tarim	Gabbro	431	3	431	0.6	0.4	1286	1299	1022	1027	Zhao ZY et al., 2015 Gondwana Research
BCD 2	82.09	42.23	Tarim	Gabbro	430	3	430	2.7	2.4	1165	1180	934	947	Zhao ZY et al., 2015 Gondwana Research
HG-1	85.53	46.67	0	hornblede gabbro	422	5	422	15.3	15.5	429	418	427	419	Chen et al., 2015
15JJW8-1	126.83	43.20	0	Hornblende gabbro	219	3	219	9.1	8.6	629	658	517	522	Wang F et al., 2019 Journal of Geophysical Research: Solid Earth
HTW1-1	129.21	48.54	0	Hornblende gabbro	185	1	185	4.7	4.8	857	848	659	654	Yu JJ et al., 2012 Lithos
15XH11-1	129.42	45.71	0	Hornblende gabbro	215	3	215	3.6	3.5	945	947	716	724	Long XY et al., 2020 Gondwana Research
15XH15-1	129.88	45.70	0	Hornblende gabbro	215	1	215	-2.5	-2.9	1297	1318	970	985	Long XY et al., 2020 Gondwana Research
HTW8-1	129.69	48.22	0	Hornblende gabbro	241	2	241	2.4	1.8	1035	1066	785	809	Long XY et al., 2020 Gondwana Research
15ER186-5	120.05	51.01	0	Hornblende gabbro	256	2	256	5.7	5.3	855	875	678	692	Mao AQ et al., 2021 International Geology Review
WSTGL-2	86.67	49.67	Tianshan	hornblende gabbro	408	0	408	7.8	7.4	861	882	737	746	Qin et al, 2016 International Geology Review
15JLA1	126.29	43.68	CAOB	hornblende gabbro	486	1	486	15.4	15.0	478	497	481	494	Ma HT et al., 2020 Journal of Asian Earth Sciences
HYL1-1	128.02	46.35	0	Hornblendite	186	2	186	3.8	8.0	903	665	698	540	Yu JJ et al., 2012 Lithos
11DB	115.75	31.50	the Hong'an	Mafic dyke	115	0	150	10.1	10.6	519	489	447	420	Dai LQ et al., 2015 Gondwana Research
12YX24-2-2	85.34	42.84	CAOB	Gabbro	317	2.1	317	12.1	12.1	535	532	471	466	Tian YZ et al., 2014 Acta Petrologica Sinica (ICWEA)
BS029	99.40	41.83	CAOB	Diabase	115	3.3	150	-26.7	-23.0	2615	2413	1927	1771	Liu XM et al., 2010 Geological Bulletin of China (ICWEA)
黑白山	90.79	41.75	CAOB	Mafic-ultramafic roc	393	4.5	393	9.4	9.2	747	758	635	642	She JZ et al., 2021 Xinjiang Geology (ICWEA)
H15	128.48	46.82	CAOB	Diabase	187	2	187	2.3	3.7	996	914	748	700	Ge MH et al., 2020 Acta Petrologica Sinica (ICWEA)
辉长岩	110.17	41.53	CAOB	Gabbro	269	1.1	269	-15.2	-15.5	2063	2080	1517	1521	Li ZD et al., 2020 Geological Bulletin of China (ICWEA)
KL1	88.75	46.08	CAOB	Gabbro	278	2.2	278	12.9	12.6	456	471	404	414	Tang HJ et al., 2022 Earth Science (ICWEA)
KP-02	78.92	40.02	CAOB	Basalt	292	2.2	292	-2.8	-2.6	1371	1358	1036	1032	Zhang YD et al., 2010 Acta Petrologica Sinica (ICWEA)
IID	94.60	42.50	Beishan	Mafic-ultramafic roc	274	0	274	15.2	15.1	320	327	306	311	Qian ZZ et al., 2012 Northwestern Geology (ICWEA)
XJ11-150	94.01	41.65	CAOB	Gabbro-diorite	443	1	443	-2.6	0.1	1480	1325	1163	1063	Li XF et al., 2015 Acta Petrologica Sinica (ICWEA)
TY	94.13	41.83	CAOB	Gabbro	290	3.4	290	3.4	3.1	1016	1031	798	805	Tang DM et al., 2009 Acta Petrologica Sinica(ICWEA)
13YA-63-1	84.26	45.67	CAOB	Diabase	358	2.4	358	12.8	12.9	524	521	477	475	Yang HY et al., 2019 Acta Geologica Sinica (ICWEA)
WH2	107.01	41.64	CAOB	Gabbro	320	1.8	320	5.6	5.4	909	924	725	730	Zhang Y et al., 2020 Geoscience (ICWEA)
WH6	106.99	41.68	CAOB	Gabbro	325	1.6	325	0.2	0.9	1223	1184	944	919	Zhang Y et al., 2020 Geoscience (ICWEA)
BEHT01	110.17	42.02	CAOB	Basalt	224	2	224	-3.8	-1.5	1376	1246	1025	929	Zhang W et al., 2010 Geological Bulletin of China(ICWEA)
P7.38.1	106.32	41.38	CAOB	Basaltic andesite	265	1.5	265	-1.5	-1.2	1278	1257	968	956	Guo S et al., 2022 Journal of Geomechanics (ICWEA)
BK-04	85.17	45.75	CAOB	Basaltic andesite	342	1	342	12.9	13.2	508	491	460	445	Xiao Y et al., 2022 Geotectonica et Metallogenia (ICWEA)
SC1	88.50	45.33	0	Basalt	300	0	300	8.8	8.3	749	794	630	687	Su YP et al., 2010 Chinese Science Bulletin (ICWEA)
YS16H05	92.44	41.76	CAOB	Gabbro	312	3.5	312	4.8	3.5	948	1025	764	819	Xie ZH et al., 2018 Acta Mineralogica Sinica (ICWEA)
XDC1	125.27	43.62	0	Alkali basalt	253	5	253	3.4	3.6	984	972	762	757	Song ZG et al., 2018 Gondwana Research
13HYL1-1	129.81	46.02	0	Olivine gabbro	211	2	211	-1.0	-1.4	1203	1225	900	912	Guo P et al., 2016 International Geology Review
HTW6-1	129.42	48.49	0	Olivine gabbro	186	2	186	3.7	3.8	916	909	703	709	Yu JJ et al., 2012 Lithos
15XH14-1	129.90	45.71	0	Olivine gabbro	221	2	221	0.7	0.4	1112	1134	847	865	Long XY et al., 2020 Gondwana Research
SMZM-01	127.92	43.08	in the Songliao Block, NE China	Gabbro	262	0.7	262	-3.1	-2.9	1368	1355	1026	1017	Wu T et al., 2016 Lithos
PM201-3-TW	123.50	42.65	north Liaoning, NE China	Basalt	361	8.1	361	7.0	7.3	823	835	668	680	Shi Y et al,2022 Gondwana Research
13WL	85.00	43.00	Awulale Mountains	Gabbro	313	3	313	7.4	7.6	801	791	657	646	Yan et al., 2015
R-58-1	131.75	50.67	Central Asia Orogenic Belt, Russia	Biotite gneiss	950	2	600	-5.4	-5.0	1759	1740	1414	1399	Sorokin AA et al., 2019 Lithos
S-1278	131.22	51.03	Central Asia Orogenic Belt, Russia	Amphibole–biotite g	923	9	600	-4.7	-5.0	1722	1739	1383	1406	Sorokin AA et al., 2019 Lithos
R-94-1	131.76	50.67	Central Asia Orogenic Belt, Russia	Amphibolite	937	5	600	-3.4	-3.4	1647	1645	1327	1321	Sorokin AA et al., 2019 Lithos
RH02-3	113.02	46.19	Jiamusi Block	Gabbro	274	4	274	5.5	5.4	878	884	693	694	Sun et al.,2015 Tectonophysics
RH02-10	113.02	46.19	Jiamusi Block	Gabbro	276	3	276	6.4	6.1	828	846	659	669	Sun et al., 2015 Tectonophysics
SDHS-18	96.00	42.62	Xinjiang Province of northwestern China	Monzogranite	382	4	382	11.7	11.3	623	648	561	580	He et al., 2021 Ore Geology Reviews
SDHS-1	96.02	42.62	Xinjiang Province of northwestern China	Gabbro	397	4	397	14.6	14.3	453	467	436	446	He et al., 2021 Ore Geology Reviews
Y01	91.75	42.67	eastern Tianshan in NW China	Gabbro	265	1.8	265	15.2	15.2	311	311	297	298	Mao QG et al., 2021

Y02	91.75	42.67	eastern Tianshan in NW China	Olivine gabbro	273	1.3	273	15.2	15.5	316	298	303	291	Mao QG et al., 2021
Y03	91.75	42.67	eastern Tianshan in NW China	Leucogabbro	265	2.2	265	16.8	16.9	219	210	228	223	Mao QG et al., 2021
H19HX1	118.64	44.75	central Inner Mongolia	Gabbro	281	0.6	281	8.8	9.2	696	675	574	557	Zhang X et al., 2021 Journal of Asian Earth Sciences
XS-1	93.66	43.04	CAOB	Mafic-ultramafic roc	285	0.9	285	13.0	13.2	456	441	404	395	Chen JP et al., 2016 Northwestern Geology (ICWEA)
AQS21	90.85	41.80	CAOB	Olivine basalt	318	2.4	318	10.4	11.0	631	596	545	522	Wang JW et al., 2022 Earth Science (ICWEA)
TD1-1	93.66	43.04	CAOB	Mafic-ultramafic roc	298	0.9	298	14.3	14.3	392	390	364	363	Chen JP et al., 2016 Northwestern Geology (ICWEA)
HD	94.68	42.18	CAOB	Mafic-ultramafic roc	274	3	274	15.2	15.1	320	327	306	311	Qian ZZ et al., 2012 Northwestern Geology (ICWEA)
D003-1	93.66	43.04	CAOB	Mafic-ultramafic roc	277	1.3	277	14.3	14.4	375	370	346	342	Chen JP et al., 2016 Northwestern Geology (ICWEA)
D4053-1	90.19	47.12	CAOB	Hornblende gabbro	402	3.3	402	5.7	5.7	970	968	794	793	Lin XW et al., 2021 Earth Science (ICWEA)
XYB14-4	83.31	43.62	CAOB		314	2	314	10.3	10.5			668	659	unpublished data
XYD14-6	83.33	43.57	CAOB		315	3	315	7.4	7.5			856	851	unpublished data
XYE14-8	83.25	43.35	CAOB		323	3	323	5.5	5.5			985	983	unpublished data
TKSE14-15	81.99	43.25	CAOB		304	6	304	12.6	12.6			527	526	unpublished data
TKSA14-6	81.91	42.79	CAOB		412	2	412	8.8	8.9			846	837	unpublished data
KM9802-11	84.82	45.70	CAOB	Dioritic enclave	306	5	306	14.7	14.7			384	379	Geng et al., 2009 Chemical Geology
AK6	84.36	45.76	CAOB	Dioritic enclave	304	2	304	14.1	14.2			415	410	Geng et al., 2009 Chemical Geology

Table S3. Major igneous rock-related deposits of the Altaids.

No.	Longitude	Latitude	Deposit	Type	Scale	Time	Data sources
1	117.683	46.050	Ag	Ag-Pb-Zn deposit	Small		Lv B. et al., 2017. Journal of Jilin University (Earth Science Edition) (In Chinese with English) Abstract)
2	89.642	49.872	Ag	Ag-Pb-Zn deposit	Large	MZ	Ochir Gerel et al., 2021 Mineral Resources of Mongolia (book) and refs therein
3	92.792	46.558	Ag	Ag-Pb-Zn deposit	Small	MZ2	Ochir Gerel et al., 2021 Mineral Resources of Mongolia (book) and refs therein
4	90.478	48.613	Ag	Ag-Pb-Zn deposit	Occurrence	MZ1	Ochir Gerel et al., 2021 Mineral Resources of Mongolia (book) and refs therein
5	90.317	48.400	Ag	Ag-Pb-Zn deposit	Medium	J	Ochir Gerel et al., 2021 Mineral Resources of Mongolia (book) and refs therein
6	116.342	48.745	Ag	Ag-Pb-Zn deposit	Large	K	Li,C.Y., et al., 2021,1995,2019.Development and Research Center, China Geological Survey.
7	70.619	41.069	Ag	Ag-Pb-Zn deposit	Large	P1	Jiang Sihong, et al. 2019. Evaluation and application demonstration of copper, nickel, and uranium resource potential in Central Asia and adjacent regions.
8	89.633	49.867	Ag	Ag-Pb-Zn deposit	Large		Lv B. et al., 2017. Journal of Jilin University (Earth Science Edition) (In Chinese with English) Abstract)
9	95.501	41.203	Ag	Ag-Pb-Zn deposit	Small	T	Li,C.Y., et al., 2021,1995,2019.Development and Research Center, China Geological Survey.
10	119.483	44.542	Ag	Ag-Pb-Zn deposit	Medium	J2	Li,C.Y., et al., 2021,1995,2019.Development and Research Center, China Geological Survey.
11	117.550	44.100	Ag	Ag-Pb-Zn deposit	Large		Lv B. et al., 2017. Journal of Jilin University (Earth Science Edition) (In Chinese with English) Abstract)
12	115.333	48.917	Ag	Ag-Pb-Zn deposit	Large		Lv B. et al., 2017. Journal of Jilin University (Earth Science Edition) (In Chinese with English) Abstract)
13	116.333	48.733	Ag	Ag-Pb-Zn deposit	Large		Lv B. et al., 2017. Journal of Jilin University (Earth Science Edition) (In Chinese with English) Abstract)
14	73.094	40.030	Ag	Ag-Pb-Zn deposit	Small		National Mineral Origin Database of China
15	116.667	48.367	Ag	Ag-Pb-Zn deposit	Large		Lv B. et al., 2017. Journal of Jilin University (Earth Science Edition) (In Chinese with English) Abstract)
16	118.617	44.050	Ag	Ag-Pb-Zn deposit	Small	D-P	Li,C.Y., et al., 2021,1995,2019.Development and Research Center, China Geological Survey.
17	118.527	42.578	Ag	Ag-Pb-Zn deposit	Small	J2-K	Li,C.Y., et al., 2021,1995,2019.Development and Research Center, China Geological Survey.
18	116.558	48.380	Ag	Ag-Pb-Zn deposit	Large	K	Li,C.Y., et al., 2021,1995,2019.Development and Research Center, China Geological Survey.
19	123.319	52.269	Ag	Ag-Pb-Zn deposit	Small	γ5	Li,C.Y., et al., 2021,1995,2019.Development and Research Center, China Geological Survey.
20	117.950	46.067	Ag	Ag-Pb-Zn deposit	Small		Lv B. et al., 2017. Journal of Jilin University (Earth Science Edition) (In Chinese with English) Abstract)
21	116.267	48.783	Ag	Ag-Pb-Zn deposit	Large		Lv B. et al., 2017. Journal of Jilin University (Earth Science Edition) (In Chinese with English) Abstract)
22	71.308	42.416	Ag	Ag-Pb-Zn deposit	Small		Jiang Sihong, et al., 2019.unpublished internal report. 1-517.
23	70.330	40.895	Ag	Ag-Pb-Zn deposit	Large		Jiang Sihong, et al., 2019.unpublished internal report. 1-517.
24	122.824	46.904	Ag	Ag-Pb-Zn deposit	Small	J1	Li,C.Y., et al., 2021,1995,2019.Development and Research Center, China Geological Survey.
25	128.950	42.933	Ag	Ag-Pb-Zn deposit	Large	Pz2-Mz	Li,C.Y., et al., 2021,1995,2019.Development and Research Center, China Geological Survey.
26	121.367	45.200	Ag	Ag-Pb-Zn deposit	Large		Lv B. et al., 2017. Journal of Jilin University (Earth Science Edition) (In Chinese with English) Abstract)
27	70.104	41.301	Ag	Ag-Pb-Zn deposit	Small		Jiang Sihong, et al., 2019.unpublished internal report. 1-517.
28	71.654	42.449	Ag	Ag-Pb-Zn deposit	Small		Jiang Sihong, et al., 2019.unpublished internal report. 1-517.
29	124.528	43.010	Ag	Ag-Pb-Zn deposit	Medium	Mz	Li,C.Y., et al., 2021,1995,2019.Development and Research Center, China Geological Survey.
30	124.480	42.965	Ag	Ag-Pb-Zn deposit	Large	Mz	Li,C.Y., et al., 2021,1995,2019.Development and Research Center, China Geological Survey.
31	128.477	46.969	Ag	Ag-Pb-Zn deposit	Medium	Q	Li,C.Y., et al., 2021,1995,2019.Development and Research Center, China Geological Survey.
32	72.669	40.080	Ag	Ag-Pb-Zn deposit	Small		Jiang Sihong, et al., 2019.unpublished internal report. 1-517.
33	118.026	43.060	Ag	Ag-Pb-Zn deposit	Small	J2-K	Li,C.Y., et al., 2021,1995,2019.Development and Research Center, China Geological Survey.
34	117.508	42.111	Ag	Ag-Pb-Zn deposit	Medium		Li,C.Y., et al., 2021,1995,2019.Development and Research Center, China Geological Survey.
35	117.550	42.125	Ag	Ag-Pb-Zn deposit	Small	Mz	Li,C.Y., et al., 2021,1995,2019.Development and Research Center, China Geological Survey.
36	120.167	45.083	Ag	Ag-Pb-Zn deposit	Small	J2-K	Li,C.Y., et al., 2021,1995,2019.Development and Research Center, China Geological Survey.
37	125.063	43.508	Ag	Ag-Pb-Zn deposit	Medium	Pz2	Li,C.Y., et al., 2021,1995,2019.Development and Research Center, China Geological Survey.
38	126.183	44.150	Ag	Ag-Pb-Zn deposit	Small	Mz	Li,C.Y., et al., 2021,1995,2019.Development and Research Center, China Geological Survey.
39	93.693	41.622	Ag	Ag-Pb-Zn deposit	Small	C	Li,C.Y., et al., 2021,1995,2019.Development and Research Center, China Geological Survey.
40	71.374	42.408	Ag	Ag-Pb-Zn deposit	Small		Jiang Sihong, et al., 2019.unpublished internal report. 1-517.
41	110.183	47.850	Ag,Pb,Zn	Ag-Pb-Zn deposit	Large	MZ2	Ochir Gerel et al., 2021 Mineral Resources of Mongolia (book) and refs therein
42	109.767	47.283	Ag,Pb,Zn	Ag-Pb-Zn deposit	Small	MZ2	Ochir Gerel et al., 2021 Mineral Resources of Mongolia (book) and refs therein
43	111.581	47.231	Ag,Pb,Zn	Ag-Pb-Zn deposit	Small	MZ2	Ochir Gerel et al., 2021 Mineral Resources of Mongolia (book) and refs therein
44	88.264	47.603	Au	Other magmatic-hydrothermal Au deposit	Medium	Qp	Feng, J. et al., 2021. Geological Publishing House
45	88.193	47.453	Au	Other magmatic-hydrothermal Au deposit	Small	Qp	Feng, J. et al., 2021. Geological Publishing House
46	88.884	47.482	Au	Other magmatic-hydrothermal Au deposit	Small	Qp	Feng, J. et al., 2021. Geological Publishing House
47	88.976	47.466	Au	Other magmatic-hydrothermal Au deposit	Small	Qp	Feng, J. et al., 2021. Geological Publishing House
48	89.025	47.412	Au	Other magmatic-hydrothermal Au deposit	Small	D1	Feng, J. et al., 2021. Geological Publishing House
49	89.803	46.734	Au	Other magmatic-hydrothermal Au deposit	Small		National Mineral Origin Database of China
50	89.837	47.085	Au	Other magmatic-hydrothermal Au deposit	Medium		National Mineral Origin Database of China
51	88.500	52.270	Au	Other magmatic-hydrothermal Au deposit	Small		National Mineral Origin Database of China
52	108.493	48.522	Au	Other magmatic-hydrothermal Au deposit	Medium	Q	Ochir Gerel et al., 2021 Mineral Resources of Mongolia (book) and refs therein
53	94.983	47.827	Au	Porphyry/Skarn Cu-Au deposit	Medium	NP3	Ochir Gerel et al., 2021 Mineral Resources of Mongolia (book) and refs therein
54	92.790	51.870	Au	Other magmatic-hydrothermal Au deposit	Small		National Mineral Origin Database of China
55	73.111	42.548	Au	Other magmatic-hydrothermal Au deposit	Large	D2	Jiang Sihong, et al., 2019.unpublished internal report. 1-517.
56	104.526	48.276	Au	Porphyry/Skarn Cu-Au deposit	Small	S	Ochir Gerel et al., 2021 Mineral Resources of Mongolia (book) and refs therein
57	104.506	48.283	Au	Other magmatic-hydrothermal Au deposit	Large	Q	Ochir Gerel et al., 2021 Mineral Resources of Mongolia (book) and refs therein
58	113.250	49.517	Au	Other magmatic-hydrothermal Au deposit	Medium	Q	Ochir Gerel et al., 2021 Mineral Resources of Mongolia (book) and refs therein
59	104.517	48.500	Au	Other magmatic-hydrothermal Au deposit	Small	Q	Ochir Gerel et al., 2021 Mineral Resources of Mongolia (book) and refs therein
60	90.980	51.380	Au	Other magmatic-hydrothermal Au deposit	Small		National Mineral Origin Database of China
61	106.500	48.617	Au	Porphyry/Skarn Cu-Au deposit	Small	MZ1	Ochir Gerel et al., 2021 Mineral Resources of Mongolia (book) and refs therein
62	99.350	46.267	Au	Other magmatic-hydrothermal Au deposit	Small	Q	Ochir Gerel et al., 2021 Mineral Resources of Mongolia (book) and refs therein

63	104.581	48.283	Au	Porphyry/Skarn Cu-Au deposit	Small	S	Ochir Gerel et al., 2021 Mineral Resources of Mongolia (book) and refs therein
64	107.417	47.600	Au	Other magmatic-hydrothermal Au deposit	Small	Q	Ochir Gerel et al., 2021 Mineral Resources of Mongolia (book) and refs therein
65	110.100	48.750	Au	Other magmatic-hydrothermal Au deposit	Small	Q	Ochir Gerel et al., 2021 Mineral Resources of Mongolia (book) and refs therein
66	104.533	48.375	Au	Other magmatic-hydrothermal Au deposit	Medium	Q	Ochir Gerel et al., 2021 Mineral Resources of Mongolia (book) and refs therein
67	104.522	48.392	Au	Other magmatic-hydrothermal Au deposit	Large	Q	Ochir Gerel et al., 2021 Mineral Resources of Mongolia (book) and refs therein
68	104.500	48.383	Au	Other magmatic-hydrothermal Au deposit	Large	Q	Ochir Gerel et al., 2021 Mineral Resources of Mongolia (book) and refs therein
69	100.300	44.743	Au	Porphyry/Skarn Cu-Au deposit	Occurrence	D3	Ochir Gerel et al., 2021 Mineral Resources of Mongolia (book) and refs therein
70	94.450	48.733	Au	Other magmatic-hydrothermal Au deposit	Occurrence	PZ3	Ochir Gerel et al., 2021 Mineral Resources of Mongolia (book) and refs therein
71	112.500	49.017	Au	Porphyry/Skarn Cu-Au deposit	Occurrence	MZ1	Ochir Gerel et al., 2021 Mineral Resources of Mongolia (book) and refs therein
72	115.628	48.850	Au	Porphyry/Skarn Cu-Au deposit	Small	MZ2	Ochir Gerel et al., 2021 Mineral Resources of Mongolia (book) and refs therein
73	106.542	48.708	Au	Other magmatic-hydrothermal Au deposit	Medium	Q	Ochir Gerel et al., 2021 Mineral Resources of Mongolia (book) and refs therein
74	106.150	48.750	Au	Porphyry/Skarn Cu-Au deposit	Medium	MZ1	Ochir Gerel et al., 2021 Mineral Resources of Mongolia (book) and refs therein
75	106.075	48.783	Au	Porphyry/Skarn Cu-Au deposit	Small	MZ1	Ochir Gerel et al., 2021 Mineral Resources of Mongolia (book) and refs therein
76	107.319	49.406	Au	Other magmatic-hydrothermal Au deposit	Large	Q	Ochir Gerel et al., 2021 Mineral Resources of Mongolia (book) and refs therein
77	104.563	48.276	Au	Porphyry/Skarn Cu-Au deposit	Small	S	Ochir Gerel et al., 2021 Mineral Resources of Mongolia (book) and refs therein
78	91.850	49.500	Au	Other magmatic-hydrothermal Au deposit	Medium	Q	Ochir Gerel et al., 2021 Mineral Resources of Mongolia (book) and refs therein
79	112.133	48.867	Au	Porphyry/Skarn Cu-Au deposit	Occurrence	MZ1	Ochir Gerel et al., 2021 Mineral Resources of Mongolia (book) and refs therein
80	113.233	49.817	Au	Other magmatic-hydrothermal Au deposit	Occurrence	Q	Ochir Gerel et al., 2021 Mineral Resources of Mongolia (book) and refs therein
81	100.083	46.367	Au	Other magmatic-hydrothermal Au deposit	Medium	Q	Ochir Gerel et al., 2021 Mineral Resources of Mongolia (book) and refs therein
82	88.280	52.830	Au	Other magmatic-hydrothermal Au deposit	Small		National Mineral Origin Database of China
83	97.975	44.021	Au	Other magmatic-hydrothermal Au deposit	Small	Q	Ochir Gerel et al., 2021 Mineral Resources of Mongolia (book) and refs therein
84	107.039	47.200	Au	Other magmatic-hydrothermal Au deposit	Small	Q	Ochir Gerel et al., 2021 Mineral Resources of Mongolia (book) and refs therein
85	104.600	47.650	Au	Other magmatic-hydrothermal Au deposit	Small	Q	Ochir Gerel et al., 2021 Mineral Resources of Mongolia (book) and refs therein
86	95.739	48.183	Au	Other magmatic-hydrothermal Au deposit	Occurrence	PZ3	Ochir Gerel et al., 2021 Mineral Resources of Mongolia (book) and refs therein
87	102.450	50.117	Au	Porphyry/Skarn Cu-Au deposit	Medium	MZ1	Ochir Gerel et al., 2021 Mineral Resources of Mongolia (book) and refs therein
88	106.500	48.628	Au	Porphyry/Skarn Cu-Au deposit	Large	MZ1	Ochir Gerel et al., 2021 Mineral Resources of Mongolia (book) and refs therein
89	105.583	48.079	Au	Other magmatic-hydrothermal Au deposit	Small	Q	Ochir Gerel et al., 2021 Mineral Resources of Mongolia (book) and refs therein
90	101.867	46.800	Au	Other magmatic-hydrothermal Au deposit	Small	Q	Ochir Gerel et al., 2021 Mineral Resources of Mongolia (book) and refs therein
91	107.667	49.583	Au	Other magmatic-hydrothermal Au deposit	Medium	Q	Ochir Gerel et al., 2021 Mineral Resources of Mongolia (book) and refs therein
92	107.200	49.233	Au	Other magmatic-hydrothermal Au deposit	Large	Q	Ochir Gerel et al., 2021 Mineral Resources of Mongolia (book) and refs therein
93	112.817	49.450	Au	Other magmatic-hydrothermal Au deposit	Occurrence	Q	Ochir Gerel et al., 2021 Mineral Resources of Mongolia (book) and refs therein
94	106.967	49.417	Au	Other magmatic-hydrothermal Au deposit	Medium	Q	Ochir Gerel et al., 2021 Mineral Resources of Mongolia (book) and refs therein
95	104.450	48.350	Au	Other magmatic-hydrothermal Au deposit	Large	Q	Ochir Gerel et al., 2021 Mineral Resources of Mongolia (book) and refs therein
96	106.175	48.750	Au	Other magmatic-hydrothermal Au deposit	Large	Q	Ochir Gerel et al., 2021 Mineral Resources of Mongolia (book) and refs therein
97	96.122	46.308	Au	Porphyry/Skarn Cu-Au deposit	Occurrence	NP3-KE1	Ochir Gerel et al., 2021 Mineral Resources of Mongolia (book) and refs therein
98	90.539	46.126	Au	Other magmatic-hydrothermal Au deposit	Small		National Mineral Origin Database of China
99	90.810	51.340	Au	Other magmatic-hydrothermal Au deposit	Small		National Mineral Origin Database of China
100	103.450	46.583	Au	Other magmatic-hydrothermal Au deposit	Small	Q	Ochir Gerel et al., 2021 Mineral Resources of Mongolia (book) and refs therein
101	107.175	47.483	Au	Other magmatic-hydrothermal Au deposit	Small	Q	Ochir Gerel et al., 2021 Mineral Resources of Mongolia (book) and refs therein
102	99.600	46.483	Au	Other magmatic-hydrothermal Au deposit	Small	PZ3	Ochir Gerel et al., 2021 Mineral Resources of Mongolia (book) and refs therein
103	110.442	49.175	Au	Other magmatic-hydrothermal Au deposit	Occurrence	Q	Ochir Gerel et al., 2021 Mineral Resources of Mongolia (book) and refs therein
104	107.283	47.333	Au	Other magmatic-hydrothermal Au deposit	Small	Q	Ochir Gerel et al., 2021 Mineral Resources of Mongolia (book) and refs therein
105	106.700	49.267	Au	Other magmatic-hydrothermal Au deposit	Large	Q	Ochir Gerel et al., 2021 Mineral Resources of Mongolia (book) and refs therein
106	107.158	47.250	Au	Other magmatic-hydrothermal Au deposit	Small	Q	Ochir Gerel et al., 2021 Mineral Resources of Mongolia (book) and refs therein
107	105.572	48.722	Au	Other magmatic-hydrothermal Au deposit	Small	Q	Ochir Gerel et al., 2021 Mineral Resources of Mongolia (book) and refs therein
108	90.925	47.067	Au	Other magmatic-hydrothermal Au deposit	Small	Q	Ochir Gerel et al., 2021 Mineral Resources of Mongolia (book) and refs therein
109	93.867	49.317	Au	Porphyry/Skarn Cu-Au deposit	Occurrence	E2-3	Ochir Gerel et al., 2021 Mineral Resources of Mongolia (book) and refs therein
110	87.920	52.770	Au	Other magmatic-hydrothermal Au deposit	Small		National Mineral Origin Database of China
111	107.350	47.267	Au	Other magmatic-hydrothermal Au deposit	Small	Q	Ochir Gerel et al., 2021 Mineral Resources of Mongolia (book) and refs therein
112	105.931	48.575	Au	Porphyry/Skarn Cu-Au deposit	Medium	T3	Ochir Gerel et al., 2021 Mineral Resources of Mongolia (book) and refs therein
113	106.708	49.342	Au	Other magmatic-hydrothermal Au deposit	Small	MZ1	Ochir Gerel et al., 2021 Mineral Resources of Mongolia (book) and refs therein
114	104.386	48.099	Au	Porphyry/Skarn Cu-Au deposit	Small	S	Ochir Gerel et al., 2021 Mineral Resources of Mongolia (book) and refs therein
115	100.317	43.700	Au	Other magmatic-hydrothermal Au deposit	Small	Q	Ochir Gerel et al., 2021 Mineral Resources of Mongolia (book) and refs therein
116	104.167	44.367	Au	Porphyry/Skarn Cu-Au deposit	Small	PZ2	Ochir Gerel et al., 2021 Mineral Resources of Mongolia (book) and refs therein
117	102.300	46.550	Au	Other magmatic-hydrothermal Au deposit	Medium	Q	Ochir Gerel et al., 2021 Mineral Resources of Mongolia (book) and refs therein
118	99.817	46.583	Au	Other magmatic-hydrothermal Au deposit	Small	Q	Ochir Gerel et al., 2021 Mineral Resources of Mongolia (book) and refs therein
119	101.667	46.933	Au	Other magmatic-hydrothermal Au deposit	Large	Q	Ochir Gerel et al., 2021 Mineral Resources of Mongolia (book) and refs therein
120	99.317	43.483	Au	Other magmatic-hydrothermal Au deposit	Small	Q	Ochir Gerel et al., 2021 Mineral Resources of Mongolia (book) and refs therein
121	71.682	39.685	Au	Other magmatic-hydrothermal Au deposit	Small		National Mineral Origin Database of China
122	89.020	47.170	Au	Other magmatic-hydrothermal Au deposit	Small		National Mineral Origin Database of China
123	112.367	48.267	Au	Other magmatic-hydrothermal Au deposit	Small	Q	Ochir Gerel et al., 2021 Mineral Resources of Mongolia (book) and refs therein
124	73.037	50.146	Au	Porphyry/Skarn Cu deposit	Large		Jiang Sihong, et al., 2019.unpublished internal report. 1-517.
125	88.250	47.200	Au	Other magmatic-hydrothermal Au deposit	Small		National Mineral Origin Database of China
126	101.894	46.900	Au	Other magmatic-hydrothermal Au deposit	Small	Q	Ochir Gerel et al., 2021 Mineral Resources of Mongolia (book) and refs therein

127	94.100	45.833	Au	Other magmatic-hydrothermal Au deposit	Small	Q	Ochir Gerel et al., 2021 Mineral Resources of Mongolia (book) and refs therein
128	106.517	49.222	Au	Other magmatic-hydrothermal Au deposit	Large	Q	Ochir Gerel et al., 2021 Mineral Resources of Mongolia (book) and refs therein
129	94.617	48.183	Au	Porphyry/Skarn Cu-Au deposit	Occurrence	O2-3	Ochir Gerel et al., 2021 Mineral Resources of Mongolia (book) and refs therein
130	102.536	46.978	Au	Other magmatic-hydrothermal Au deposit	Small	Q	Ochir Gerel et al., 2021 Mineral Resources of Mongolia (book) and refs therein
131	106.517	48.578	Au	Porphyry/Skarn Cu-Au deposit	Small	MZ1	Ochir Gerel et al., 2021 Mineral Resources of Mongolia (book) and refs therein
132	91.017	46.167	Au	Other magmatic-hydrothermal Au deposit	Small		National Mineral Origin Database of China
133	104.534	47.639	Au	Other magmatic-hydrothermal Au deposit	Small	Q	Ochir Gerel et al., 2021 Mineral Resources of Mongolia (book) and refs therein
134	88.670	52.290	Au	Porphyry/Skarn Cu-Au deposit	Small		National Mineral Origin Database of China
135	90.411	48.678	Au	Porphyry/Skarn Cu-Au deposit	Occurrence	MZ1	Ochir Gerel et al., 2021 Mineral Resources of Mongolia (book) and refs therein
136	111.692	49.179	Au	Other magmatic-hydrothermal Au deposit	Occurrence	MZ2	Ochir Gerel et al., 2021 Mineral Resources of Mongolia (book) and refs therein
137	102.482	49.967	Au	Other magmatic-hydrothermal Au deposit	Small	MZ1	Ochir Gerel et al., 2021 Mineral Resources of Mongolia (book) and refs therein
138	107.213	47.367	Au	Other magmatic-hydrothermal Au deposit	Small	Q	Ochir Gerel et al., 2021 Mineral Resources of Mongolia (book) and refs therein
139	99.967	46.283	Au	Other magmatic-hydrothermal Au deposit	Small	Q	Ochir Gerel et al., 2021 Mineral Resources of Mongolia (book) and refs therein
140	107.350	49.500	Au	Other magmatic-hydrothermal Au deposit	Large	Q	Ochir Gerel et al., 2021 Mineral Resources of Mongolia (book) and refs therein
141	104.542	48.433	Au	Other magmatic-hydrothermal Au deposit	Large	Q	Ochir Gerel et al., 2021 Mineral Resources of Mongolia (book) and refs therein
142	103.733	50.117	Au	Other magmatic-hydrothermal Au deposit	Small	Q	Ochir Gerel et al., 2021 Mineral Resources of Mongolia (book) and refs therein
143	104.558	48.400	Au	Other magmatic-hydrothermal Au deposit	Medium	Q	Ochir Gerel et al., 2021 Mineral Resources of Mongolia (book) and refs therein
144	106.183	48.706	Au	Porphyry/Skarn Cu-Au deposit	Small	MZ1	Ochir Gerel et al., 2021 Mineral Resources of Mongolia (book) and refs therein
145	105.183	48.667	Au	Other magmatic-hydrothermal Au deposit	Small	Q	Ochir Gerel et al., 2021 Mineral Resources of Mongolia (book) and refs therein
146	91.792	48.453	Au	Porphyry/Skarn Cu-Au deposit	Medium	D3	Ochir Gerel et al., 2021 Mineral Resources of Mongolia (book) and refs therein
147	100.050	46.086	Au	Porphyry/Skarn Cu-Au deposit	Medium	PZ3	Ochir Gerel et al., 2021 Mineral Resources of Mongolia (book) and refs therein
148	106.733	49.317	Au	Other magmatic-hydrothermal Au deposit	Large	Q	Ochir Gerel et al., 2021 Mineral Resources of Mongolia (book) and refs therein
149	104.400	48.300	Au	Other magmatic-hydrothermal Au deposit	Large	MZ1	Ochir Gerel et al., 2021 Mineral Resources of Mongolia (book) and refs therein
150	104.503	48.388	Au	Other magmatic-hydrothermal Au deposit	Large	Q	Ochir Gerel et al., 2021 Mineral Resources of Mongolia (book) and refs therein
151	104.358	48.188	Au	Other magmatic-hydrothermal Au deposit	Large	Q	Ochir Gerel et al., 2021 Mineral Resources of Mongolia (book) and refs therein
152	113.815	49.269	Au	Other magmatic-hydrothermal Au deposit	Occurrence	MZ2	Ochir Gerel et al., 2021 Mineral Resources of Mongolia (book) and refs therein
153	90.920	51.370	Au	Other magmatic-hydrothermal Au deposit	Small		National Mineral Origin Database of China
154	114.719	49.173	Au	Porphyry/Skarn Cu-Au deposit	Small	MZ2	Ochir Gerel et al., 2021 Mineral Resources of Mongolia (book) and refs therein
155	106.539	48.642	Au	Porphyry/Skarn Cu-Au deposit	Small	MZ1	Ochir Gerel et al., 2021 Mineral Resources of Mongolia (book) and refs therein
156	104.583	48.450	Au	Other magmatic-hydrothermal Au deposit	Medium	Q	Ochir Gerel et al., 2021 Mineral Resources of Mongolia (book) and refs therein
157	104.527	48.270	Au	Porphyry/Skarn Cu-Au deposit	Small	S	Ochir Gerel et al., 2021 Mineral Resources of Mongolia (book) and refs therein
158	89.500	52.270	Au	Other magmatic-hydrothermal Au deposit	Small		National Mineral Origin Database of China
159	106.950	49.067	Au	Other magmatic-hydrothermal Au deposit	Large	Q	Ochir Gerel et al., 2021 Mineral Resources of Mongolia (book) and refs therein
160	90.592	49.017	Au	Porphyry/Skarn Cu-Au deposit	Small	D3	Ochir Gerel et al., 2021 Mineral Resources of Mongolia (book) and refs therein
161	90.151	45.361	Au	Other magmatic-hydrothermal Au deposit	Medium		National Mineral Origin Database of China
162	71.298	48.160	Au	Other magmatic-hydrothermal Au deposit	Small	D3	Jiang Sihong, et al., 2019.unpublished internal report. 1-517.
163	101.894	46.928	Au	Other magmatic-hydrothermal Au deposit	Medium	Q	Ochir Gerel et al., 2021 Mineral Resources of Mongolia (book) and refs therein
164	72.692	45.132	Au	Other magmatic-hydrothermal Au deposit	Medium	D1	Jiang Sihong, et al., 2019.unpublished internal report. 1-517.
165	76.531	49.418	Au	Other magmatic-hydrothermal Au deposit	Medium	D2	Jiang Sihong, et al., 2019.unpublished internal report. 1-517.
166	70.331	53.326	Au	Other magmatic-hydrothermal Au deposit	Small		Jiang Sihong, et al., 2019.unpublished internal report. 1-517.
167	72.419	40.014	Au	Other magmatic-hydrothermal Au deposit	Small		Jiang Sihong, et al., 2019.unpublished internal report. 1-517.
168	70.640	39.718	Au	Other magmatic-hydrothermal Au deposit	Small		Jiang Sihong, et al., 2019.unpublished internal report. 1-517.
169	85.551	48.496	Au	Other magmatic-hydrothermal Au deposit	Small	P-T	Jiang Sihong, et al., 2019.unpublished internal report. 1-517.
170	75.532	51.424	Au	Other magmatic-hydrothermal Au deposit	Small	O2	Jiang Sihong, et al., 2019.unpublished internal report. 1-517.
171	82.266	48.464	Au	Porphyry/Skarn Cu deposit	Medium	C	Jiang Sihong, et al., 2019.unpublished internal report. 1-517.
172	70.864	46.514	Au	Other magmatic-hydrothermal Au deposit	Small	D2	Jiang Sihong, et al., 2019.unpublished internal report. 1-517.
173	82.129	50.344	Au	Other magmatic-hydrothermal Au deposit	Small	P	Jiang Sihong, et al., 2019.unpublished internal report. 1-517.
174	82.109	48.427	Au	Porphyry/Skarn Cu deposit	Medium		Jiang Sihong, et al., 2019.unpublished internal report. 1-517.
175	72.688	45.125	Au	Other magmatic-hydrothermal Au deposit	Large (Super Large		Jiang Sihong, et al., 2019.unpublished internal report. 1-517.
176	77.896	44.214	Au	Other magmatic-hydrothermal Au deposit	Small	P2-T1	Jiang Sihong, et al., 2019.unpublished internal report. 1-517.
177	86.664	48.167	Au	Other magmatic-hydrothermal Au deposit	Small	C3	Feng, J. et al., 2021. Geological Publishing House
178	89.389	47.931	Au	Other magmatic-hydrothermal Au deposit	Small	Qp	Feng, J. et al., 2021. Geological Publishing House
179	86.064	48.348	Au	Other magmatic-hydrothermal Au deposit	Small	C3	Feng, J. et al., 2021. Geological Publishing House
180	74.033	40.780	Au	Other magmatic-hydrothermal Au deposit	Small	P	Jiang Sihong, et al., 2019.unpublished internal report. 1-517.
181	69.696	40.831	Au	Porphyry/Skarn Cu deposit	Large		Jiang Sihong, et al., 2019.unpublished internal report. 1-517.
182	88.756	47.289	Au	Other magmatic-hydrothermal Au deposit	Small	C3	Feng, J. et al., 2021. Geological Publishing House
183	81.435	49.228	Au	Other magmatic-hydrothermal Au deposit	Medium	P	Jiang Sihong, et al., 2019.unpublished internal report. 1-517.
184	70.028	39.763	Au	Other magmatic-hydrothermal Au deposit	Small	T	Jiang Sihong, et al., 2019.unpublished internal report. 1-517.
185	80.350	48.366	Au	Other magmatic-hydrothermal Au deposit	Small		Jiang Sihong, et al., 2019.unpublished internal report. 1-517.
186	77.692	40.750	Au		Small	C3	Feng, J. et al., 2021. Geological Publishing House
187	80.510	48.273	Au	Other magmatic-hydrothermal Au deposit	Small		Jiang Sihong, et al., 2019.unpublished internal report. 1-517.
188	73.795	40.358	Au	Other magmatic-hydrothermal Au deposit	Small		Jiang Sihong, et al., 2019.unpublished internal report. 1-517.
189	75.275	51.546	Au	Other magmatic-hydrothermal Au deposit	Small	O2	Jiang Sihong, et al., 2019.unpublished internal report. 1-517.
190	73.000	42.547	Au	Other magmatic-hydrothermal Au deposit	Large	O2	Jiang Sihong, et al., 2019.unpublished internal report. 1-517.

191	94.888	41.560	Au	Other magmatic-hydrothermal Au deposit	Small	S	Li,C.Y., et al., 2021,1995,2019.Development and Research Center, China Geological Survey.
192	95.569	41.236	Au	Other magmatic-hydrothermal Au deposit	Small	T	Li,C.Y., et al., 2021,1995,2019.Development and Research Center, China Geological Survey.
193	94.822	41.164	Au	Other magmatic-hydrothermal Au deposit	Small		Li,C.Y., et al., 2021,1995,2019.Development and Research Center, China Geological Survey.
194	94.619	41.123	Au	Other magmatic-hydrothermal Au deposit	Small		Li,C.Y., et al., 2021,1995,2019.Development and Research Center, China Geological Survey.
195	94.846	41.137	Au	Other magmatic-hydrothermal Au deposit	Small	C	Li,C.Y., et al., 2021,1995,2019.Development and Research Center, China Geological Survey.
196	95.022	41.178	Au	Other magmatic-hydrothermal Au deposit	Medium		Li,C.Y., et al., 2021,1995,2019.Development and Research Center, China Geological Survey.
197	95.226	41.522	Au	Other magmatic-hydrothermal Au deposit	Small	S	Li,C.Y., et al., 2021,1995,2019.Development and Research Center, China Geological Survey.
198	73.292	42.758	Au	Porphyry/Skarn Cu deposit	Small		Jiang Sihong, et al., 2019.unpublished internal report. 1-517.
199	73.032	44.908	Au	Other magmatic-hydrothermal Au deposit	Small		Jiang Sihong, et al., 2019.unpublished internal report. 1-517.
200	76.272	46.900	Au	Other magmatic-hydrothermal Au deposit	Small		Jiang Sihong, et al., 2019.unpublished internal report. 1-517.
201	78.688	48.334	Au	Other magmatic-hydrothermal Au deposit	Small		Jiang Sihong, et al., 2019.unpublished internal report. 1-517.
202	66.715	52.930	Au	Other magmatic-hydrothermal Au deposit	Small		Jiang Sihong, et al., 2019.unpublished internal report. 1-517.
203	82.250	49.086	Au	Other magmatic-hydrothermal Au deposit	Small		Jiang Sihong, et al., 2019.unpublished internal report. 1-517.
204	85.332	48.544	Au	Other magmatic-hydrothermal Au deposit	Small		Jiang Sihong, et al., 2019.unpublished internal report. 1-517.
205	110.231	41.992	Au	Other magmatic-hydrothermal Au deposit	Small	D-P	Li,C.Y., et al., 2021,1995,2019.Development and Research Center, China Geological Survey.
206	110.240	41.991	Au	Other magmatic-hydrothermal Au deposit	Small	J2	Li,C.Y., et al., 2021,1995,2019.Development and Research Center, China Geological Survey.
207	113.633	43.650	Au	Other magmatic-hydrothermal Au deposit	Small		Lv B. et al., 2017. Journal of Jilin University (Earth Science Edition) (In Chinese with English) Abstract)
208	77.434	50.080	Au	Other magmatic-hydrothermal Au deposit	Small	D	Jiang Sihong, et al., 2019.unpublished internal report. 1-517.
209	125.183	52.917	Au	Other magmatic-hydrothermal Au deposit	Medium		Lv B. et al., 2017. Journal of Jilin University (Earth Science Edition) (In Chinese with English) Abstract)
210	72.679	39.253	Au	Other magmatic-hydrothermal Au deposit	Small	Q	Jiang Sihong, et al., 2019.unpublished internal report. 1-517.
211	80.472	43.308	Au	Other magmatic-hydrothermal Au deposit	Small		Jiang Sihong, et al., 2019.unpublished internal report. 1-517.
212	70.037	43.189	Au	Other magmatic-hydrothermal Au deposit	Small		Jiang Sihong, et al., 2019.unpublished internal report. 1-517.
213	113.550	42.383	Au	Porphyry/Skarn Cu deposit	Large		Lv B. et al., 2017. Journal of Jilin University (Earth Science Edition) (In Chinese with English) Abstract)
214	73.143	52.525	Au	Other magmatic-hydrothermal Au deposit	Large (Super Large		Jiang Sihong, et al., 2019.unpublished internal report. 1-517.
215	84.687	48.836	Au	Other magmatic-hydrothermal Au deposit	Small		Jiang Sihong, et al., 2019.unpublished internal report. 1-517.
216	77.963	40.821	Au		Small	D3	Feng, J. et al., 2021. Geological Publishing House
217	75.643	41.788	Au	Other magmatic-hydrothermal Au deposit	Small		Jiang Sihong, et al., 2019.unpublished internal report. 1-517.
218	80.689	47.694	Au	Other magmatic-hydrothermal Au deposit	Small		Jiang Sihong, et al., 2019.unpublished internal report. 1-517.
219	86.022	48.325	Au	Other magmatic-hydrothermal Au deposit	Small	C3	Feng, J. et al., 2021. Geological Publishing House
220	73.039	39.999	Au	Other magmatic-hydrothermal Au deposit	Medium	P	Jiang Sihong, et al., 2019.unpublished internal report. 1-517.
221	72.516	39.318	Au	Other magmatic-hydrothermal Au deposit	Small		Jiang Sihong, et al., 2019.unpublished internal report. 1-517.
222	70.469	41.104	Au	Other magmatic-hydrothermal Au deposit	Small		Jiang Sihong, et al., 2019.unpublished internal report. 1-517.
223	70.817	41.549	Au	Other magmatic-hydrothermal Au deposit	Small		Jiang Sihong, et al., 2019.unpublished internal report. 1-517.
224	118.483	42.317	Au	Other magmatic-hydrothermal Au deposit	Small		Lv B. et al., 2017. Journal of Jilin University (Earth Science Edition) (In Chinese with English) Abstract)
225	81.833	49.562	Au	Other magmatic-hydrothermal Au deposit	Small		Jiang Sihong, et al., 2019.unpublished internal report. 1-517.
226	118.486	42.328	Au	Other magmatic-hydrothermal Au deposit	Small	J2-K	Li,C.Y., et al., 2021,1995,2019.Development and Research Center, China Geological Survey.
227	129.967	43.000	Au	Other magmatic-hydrothermal Au deposit	Small		Lv B. et al., 2017. Journal of Jilin University (Earth Science Edition) (In Chinese with English) Abstract)
228	81.464	49.751	Au	Other magmatic-hydrothermal Au deposit	Small		Jiang Sihong, et al., 2019.unpublished internal report. 1-517.
229	77.868	44.529	Au	Other magmatic-hydrothermal Au deposit	Small	P2-T1	Jiang Sihong, et al., 2019.unpublished internal report. 1-517.
230	76.532	40.840	Au	Other magmatic-hydrothermal Au deposit	Medium		Jiang Sihong, et al., 2019.unpublished internal report. 1-517.
231	128.467	46.967	Au	Other magmatic-hydrothermal Au deposit	Medium		Lv B. et al., 2017. Journal of Jilin University (Earth Science Edition) (In Chinese with English) Abstract)
232	70.708	52.389	Au	Other magmatic-hydrothermal Au deposit	Small		Jiang Sihong, et al., 2019.unpublished internal report. 1-517.
233	82.062	48.944	Au	Other magmatic-hydrothermal Au deposit	Small	P	Jiang Sihong, et al., 2019.unpublished internal report. 1-517.
234	128.867	49.267	Au	Other magmatic-hydrothermal Au deposit	Large		Lv B. et al., 2017. Journal of Jilin University (Earth Science Edition) (In Chinese with English) Abstract)
235	130.750	44.221	Au	Other magmatic-hydrothermal Au deposit	Small	J3	Li,C.Y., et al., 2021,1995,2019.Development and Research Center, China Geological Survey.
236	130.667	43.000	Au	Other magmatic-hydrothermal Au deposit	Small		Lv B. et al., 2017. Journal of Jilin University (Earth Science Edition) (In Chinese with English) Abstract)
237	86.049	48.330	Au	Other magmatic-hydrothermal Au deposit	Large	C3	Feng, J. et al., 2021. Geological Publishing House
238	86.049	48.336	Au	Other magmatic-hydrothermal Au deposit	Small		Li,C.Y., et al., 2021,1995,2019.Development and Research Center, China Geological Survey.
239	86.049	48.320	Au	Other magmatic-hydrothermal Au deposit	Small	Pz2	Li,C.Y., et al., 2021,1995,2019.Development and Research Center, China Geological Survey.
240	86.049	48.303	Au	Other magmatic-hydrothermal Au deposit	Medium	Pz2	Li,C.Y., et al., 2021,1995,2019.Development and Research Center, China Geological Survey.
241	86.056	48.333	Au	Other magmatic-hydrothermal Au deposit	Medium	P	Li,C.Y., et al., 2021,1995,2019.Development and Research Center, China Geological Survey.
242	76.264	47.000	Au	Other magmatic-hydrothermal Au deposit	Small	P	Jiang Sihong, et al., 2019.unpublished internal report. 1-517.
243	80.099	42.442	Au	Other magmatic-hydrothermal Au deposit	Small		Jiang Sihong, et al., 2019.unpublished internal report. 1-517.
244	116.727	45.110	Au	Other magmatic-hydrothermal Au deposit	Small	P	Li,C.Y., et al., 2021,1995,2019.Development and Research Center, China Geological Survey.
245	82.719	49.113	Au	Other magmatic-hydrothermal Au deposit	Small		Jiang Sihong, et al., 2019.unpublished internal report. 1-517.
246	89.732	41.584	Au		Small	C3	Feng, J. et al., 2021. Geological Publishing House
247	84.933	46.025	Au	Other magmatic-hydrothermal Au deposit	Small	C	Li,C.Y., et al., 2021,1995,2019.Development and Research Center, China Geological Survey.
248	118.518	42.426	Au	Other magmatic-hydrothermal Au deposit	Small	J2-K	Li,C.Y., et al., 2021,1995,2019.Development and Research Center, China Geological Survey.
249	67.594	52.704	Au	Other magmatic-hydrothermal Au deposit	Small		Jiang Sihong, et al., 2019.unpublished internal report. 1-517.
250	70.751	41.034	Au	Other magmatic-hydrothermal Au deposit	Medium		Jiang Sihong, et al., 2019.unpublished internal report. 1-517.
251	118.527	42.578	Au	Other magmatic-hydrothermal Au deposit	Small	J2-K	Li,C.Y., et al., 2021,1995,2019.Development and Research Center, China Geological Survey.
252	72.309	39.936	Au	Other magmatic-hydrothermal Au deposit	Small		Jiang Sihong, et al., 2019.unpublished internal report. 1-517.
253	113.567	42.300	Au	Porphyry/Skarn Cu deposit	Small		Lv B. et al., 2017. Journal of Jilin University (Earth Science Edition) (In Chinese with English) Abstract)
254	90.117	42.192	Au	Other magmatic-hydrothermal Au deposit	Small	C	Li,C.Y., et al., 2021,1995,2019.Development and Research Center, China Geological Survey.

255	113.500	42.292	Au	Other magmatic-hydrothermal Au deposit	Small	J2-K	Li,C.Y., et al., 2021,1995,2019.Development and Research Center, China Geological Survey.
256	126.258	50.175	Au		Medium	Q4	Li,C.Y., et al., 2021,1995,2019.Development and Research Center, China Geological Survey.
257	127.136	50.522	Au	Other magmatic-hydrothermal Au deposit	Medium	J3	Li,C.Y., et al., 2021,1995,2019.Development and Research Center, China Geological Survey.
258	123.624	52.065	Au	Other magmatic-hydrothermal Au deposit	Medium	Q4	Li,C.Y., et al., 2021,1995,2019.Development and Research Center, China Geological Survey.
259	88.211	47.593	Au	Other magmatic-hydrothermal Au deposit	Small	Qp	Feng, J. et al., 2021. Geological Publishing House
260	88.210	47.662	Au	Other magmatic-hydrothermal Au deposit	Small	Qp	Feng, J. et al., 2021. Geological Publishing House
261	118.633	42.217	Au	Other magmatic-hydrothermal Au deposit	Medium	K	Li,C.Y., et al., 2021,1995,2019.Development and Research Center, China Geological Survey.
262	125.415	51.443	Au	Other magmatic-hydrothermal Au deposit	Small		Li,C.Y., et al., 2021,1995,2019.Development and Research Center, China Geological Survey.
263	126.608	51.467	Au	Other magmatic-hydrothermal Au deposit	Medium	K	Li,C.Y., et al., 2021,1995,2019.Development and Research Center, China Geological Survey.
264	126.758	51.132	Au	Other magmatic-hydrothermal Au deposit	Small		Li,C.Y., et al., 2021,1995,2019.Development and Research Center, China Geological Survey.
265	125.843	51.480	Au	Other magmatic-hydrothermal Au deposit	Small	J	Li,C.Y., et al., 2021,1995,2019.Development and Research Center, China Geological Survey.
266	121.350	52.917	Au	Porphyry/Skarn Cu deposit	Medium		Lv B. et al., 2017. Journal of Jilin University (Earth Science Edition) (In Chinese with English) Abstract)
267	130.900	46.100	Au		Medium	N	Li,C.Y., et al., 2021,1995,2019.Development and Research Center, China Geological Survey.
268	84.813	46.092	Au	Other magmatic-hydrothermal Au deposit	Small	C-P	Li,C.Y., et al., 2021,1995,2019.Development and Research Center, China Geological Survey.
269	131.117	43.250	Au		Small	Q	Li,C.Y., et al., 2021,1995,2019.Development and Research Center, China Geological Survey.
270	130.783	42.925	Au		Small	Q	Li,C.Y., et al., 2021,1995,2019.Development and Research Center, China Geological Survey.
271	130.883	43.200	Au	Other magmatic-hydrothermal Au deposit	Large	Mz	Li,C.Y., et al., 2021,1995,2019.Development and Research Center, China Geological Survey.
272	70.988	41.947	Au	Porphyry/Skarn Cu deposit	Medium	P1-2	Jiang Sihong, et al., 2019.unpublished internal report. 1-517.
273	129.667	43.169	Au	Other magmatic-hydrothermal Au deposit	Small	Mz	Li,C.Y., et al., 2021,1995,2019.Development and Research Center, China Geological Survey.
274	78.173	41.870	Au	Other magmatic-hydrothermal Au deposit	Large	P1	Jiang Sihong, et al., 2019.unpublished internal report. 1-517.
275	87.908	48.022	Au	Other magmatic-hydrothermal Au deposit	Small	Qp	Feng, J. et al., 2021. Geological Publishing House
276	129.892	48.447	Au	Other magmatic-hydrothermal Au deposit	Small	J	Li,C.Y., et al., 2021,1995,2019.Development and Research Center, China Geological Survey.
277	73.036	40.089	Au	Other magmatic-hydrothermal Au deposit	Small		Jiang Sihong, et al., 2019.unpublished internal report. 1-517.
278	88.817	47.500	Au	Other magmatic-hydrothermal Au deposit	Small	Qp	Feng, J. et al., 2021. Geological Publishing House
279	72.730	42.305	Au	Other magmatic-hydrothermal Au deposit	Large	O1-2	Jiang Sihong, et al., 2019.unpublished internal report. 1-517.
280	86.604	48.117	Au	Other magmatic-hydrothermal Au deposit	Small	C3	Feng, J. et al., 2021. Geological Publishing House
281	130.750	44.250	Au	Porphyry/Skarn Cu deposit	Large		Lv B. et al., 2017. Journal of Jilin University (Earth Science Edition) (In Chinese with English) Abstract)
282	86.470	48.186	Au	Other magmatic-hydrothermal Au deposit	Small	C3	Feng, J. et al., 2021. Geological Publishing House
283	130.500	43.000	Au	Other magmatic-hydrothermal Au deposit	Small		Lv B. et al., 2017. Journal of Jilin University (Earth Science Edition) (In Chinese with English) Abstract)
284	72.833	45.310	Au	Other magmatic-hydrothermal Au deposit	Small	D3	Jiang Sihong, et al., 2019.unpublished internal report. 1-517.
285	73.164	42.561	Au	Other magmatic-hydrothermal Au deposit	Small		Jiang Sihong, et al., 2019.unpublished internal report. 1-517.
286	70.383	52.254	Au	Other magmatic-hydrothermal Au deposit	Small	S	Jiang Sihong, et al., 2019.unpublished internal report. 1-517.
287	74.735	41.764	Au	Other magmatic-hydrothermal Au deposit	Small		Jiang Sihong, et al., 2019.unpublished internal report. 1-517.
288	87.871	48.008	Au	Other magmatic-hydrothermal Au deposit	Small	Qp	Feng, J. et al., 2021. Geological Publishing House
289	70.788	41.326	Au	Other magmatic-hydrothermal Au deposit	Small		Jiang Sihong, et al., 2019.unpublished internal report. 1-517.
290	68.986	49.668	Au	Other magmatic-hydrothermal Au deposit	Small	T2	Jiang Sihong, et al., 2019.unpublished internal report. 1-517.
291	74.952	43.102	Au	Other magmatic-hydrothermal Au deposit	Small		Jiang Sihong, et al., 2019.unpublished internal report. 1-517.
292	81.785	49.594	Au	Other magmatic-hydrothermal Au deposit	Small		Jiang Sihong, et al., 2019.unpublished internal report. 1-517.
293	126.267	51.433	Au	Other magmatic-hydrothermal Au deposit	Small		Lv B. et al., 2017. Journal of Jilin University (Earth Science Edition) (In Chinese with English) Abstract)
294	67.109	49.172	Au	Other magmatic-hydrothermal Au deposit	Small		Jiang Sihong, et al., 2019.unpublished internal report. 1-517.
295	74.808	51.096	Au	Other magmatic-hydrothermal Au deposit	Small		Jiang Sihong, et al., 2019.unpublished internal report. 1-517.
296	72.802	45.071	Au	Other magmatic-hydrothermal Au deposit	Small		Jiang Sihong, et al., 2019.unpublished internal report. 1-517.
297	73.060	45.286	Au	Other magmatic-hydrothermal Au deposit	Small	D3	Jiang Sihong, et al., 2019.unpublished internal report. 1-517.
298	72.689	39.730	Au	Other magmatic-hydrothermal Au deposit	Small		Jiang Sihong, et al., 2019.unpublished internal report. 1-517.
299	79.336	48.879	Au	Other magmatic-hydrothermal Au deposit	Small	D2-3	Jiang Sihong, et al., 2019.unpublished internal report. 1-517.
300	75.708	41.864	Au	Other magmatic-hydrothermal Au deposit	Small		Jiang Sihong, et al., 2019.unpublished internal report. 1-517.
301	91.679	41.938	Au		Medium	C3	Feng, J. et al., 2021. Geological Publishing House
302	69.679	40.784	Au	Porphyry/Skarn Cu deposit	Medium		Jiang Sihong, et al., 2019.unpublished internal report. 1-517.
303	70.146	40.944	Au	Other magmatic-hydrothermal Au deposit	Large	Pz3	Jiang Sihong, et al., 2019.unpublished internal report. 1-517.
304	71.712	39.726	Au	Other magmatic-hydrothermal Au deposit	Medium		Jiang Sihong, et al., 2019.unpublished internal report. 1-517.
305	81.536	49.005	Au	Other magmatic-hydrothermal Au deposit	Small	P	Jiang Sihong, et al., 2019.unpublished internal report. 1-517.
306	120.212	42.125	Au	Other magmatic-hydrothermal Au deposit	Small	J2-K	Li,C.Y., et al., 2021,1995,2019.Development and Research Center, China Geological Survey.
307	88.917	47.542	Au	Other magmatic-hydrothermal Au deposit	Small	Qp	Feng, J. et al., 2021. Geological Publishing House
308	87.925	48.092	Au	Other magmatic-hydrothermal Au deposit	Small	D2	Feng, J. et al., 2021. Geological Publishing House
309	88.167	47.483	Au	Other magmatic-hydrothermal Au deposit	Small	Qp	Feng, J. et al., 2021. Geological Publishing House
310	73.785	48.275	Au	Other magmatic-hydrothermal Au deposit	Small	P	Jiang Sihong, et al., 2019.unpublished internal report. 1-517.
311	74.740	48.018	Au	Other magmatic-hydrothermal Au deposit	Small	C-P	Jiang Sihong, et al., 2019.unpublished internal report. 1-517.
312	66.618	49.217	Au	Other magmatic-hydrothermal Au deposit	Small	O	Jiang Sihong, et al., 2019.unpublished internal report. 1-517.
313	75.536	42.629	Au	Other magmatic-hydrothermal Au deposit	Medium	D	Jiang Sihong, et al., 2019.unpublished internal report. 1-517.
314	71.448	42.071	Au	Porphyry/Skarn Cu deposit	Medium	C2	Jiang Sihong, et al., 2019.unpublished internal report. 1-517.
315	72.694	39.058	Au	Other magmatic-hydrothermal Au deposit	Small		Jiang Sihong, et al., 2019.unpublished internal report. 1-517.
316	83.062	49.143	Au	Other magmatic-hydrothermal Au deposit	Small		Jiang Sihong, et al., 2019.unpublished internal report. 1-517.
317	84.536	48.692	Au	Other magmatic-hydrothermal Au deposit	Small		Jiang Sihong, et al., 2019.unpublished internal report. 1-517.
318	70.530	52.451	Au	Other magmatic-hydrothermal Au deposit	Small		Jiang Sihong, et al., 2019.unpublished internal report. 1-517.

319	86.439	47.094	Au	Other magmatic-hydrothermal Au deposit	Small		Li,C.Y., et al., 2021,1995,2019.Development and Research Center, China Geological Survey.
320	83.368	49.097	Au	Other magmatic-hydrothermal Au deposit	Small	C-P	Jiang Sihong, et al., 2019.unpublished internal report. 1-517.
321	118.086	42.133	Au	Other magmatic-hydrothermal Au deposit	Small	J2-K	Li,C.Y., et al., 2021,1995,2019.Development and Research Center, China Geological Survey.
322	129.667	42.600	Au		Small	Pz-Mz	Li,C.Y., et al., 2021,1995,2019.Development and Research Center, China Geological Survey.
323	129.306	43.044	Au	Other magmatic-hydrothermal Au deposit	Small	Mz	Li,C.Y., et al., 2021,1995,2019.Development and Research Center, China Geological Survey.
324	74.329	44.428	Au	Other magmatic-hydrothermal Au deposit	Small		Jiang Sihong, et al., 2019.unpublished internal report. 1-517.
325	130.200	48.083	Au	Other magmatic-hydrothermal Au deposit	Small	Y5	Li,C.Y., et al., 2021,1995,2019.Development and Research Center, China Geological Survey.
326	80.464	48.563	Au	Other magmatic-hydrothermal Au deposit	Small	D3	Jiang Sihong, et al., 2019.unpublished internal report. 1-517.
327	84.751	48.795	Au	Other magmatic-hydrothermal Au deposit	Medium	P-T	Jiang Sihong, et al., 2019.unpublished internal report. 1-517.
328	70.409	52.469	Au	Other magmatic-hydrothermal Au deposit	Small	O2	Jiang Sihong, et al., 2019.unpublished internal report. 1-517.
329	96.144	41.958	Au	Porphyry/Skarn Cu deposit	Medium	C1	Feng, J. et al., 2021. Geological Publishing House
330	95.816	41.941	Au	Other magmatic-hydrothermal Au deposit	Medium	P	Li,C.Y., et al., 2021,1995,2019.Development and Research Center, China Geological Survey.
331	73.995	41.202	Au	Other magmatic-hydrothermal Au deposit	Large	P	Jiang Sihong, et al., 2019.unpublished internal report. 1-517.
332	75.748	51.434	Au	Other magmatic-hydrothermal Au deposit	Large	O2	Jiang Sihong, et al., 2019.unpublished internal report. 1-517.
333	67.147	49.236	Au	Other magmatic-hydrothermal Au deposit	Small		Jiang Sihong, et al., 2019.unpublished internal report. 1-517.
334	73.367	50.239	Au	Other magmatic-hydrothermal Au deposit	Small		Jiang Sihong, et al., 2019.unpublished internal report. 1-517.
335	75.105	43.082	Au	Other magmatic-hydrothermal Au deposit	Small		Jiang Sihong, et al., 2019.unpublished internal report. 1-517.
336	78.114	46.935	Au	Other magmatic-hydrothermal Au deposit	Small		Jiang Sihong, et al., 2019.unpublished internal report. 1-517.
337	70.097	53.240	Au	Other magmatic-hydrothermal Au deposit	Small	O2	Jiang Sihong, et al., 2019.unpublished internal report. 1-517.
338	72.639	52.696	Au	Porphyry/Skarn Cu deposit	Medium		Jiang Sihong, et al., 2019.unpublished internal report. 1-517.
339	85.793	48.512	Au	Other magmatic-hydrothermal Au deposit	Small		Jiang Sihong, et al., 2019.unpublished internal report. 1-517.
340	73.618	46.638	Au	Other magmatic-hydrothermal Au deposit	Small	C2	Jiang Sihong, et al., 2019.unpublished internal report. 1-517.
341	81.301	49.803	Au	Other magmatic-hydrothermal Au deposit	Small		Jiang Sihong, et al., 2019.unpublished internal report. 1-517.
342	74.855	42.300	Au	Other magmatic-hydrothermal Au deposit	Small		Jiang Sihong, et al., 2019.unpublished internal report. 1-517.
343	123.397	52.697	Au	Other magmatic-hydrothermal Au deposit	Small		Li,C.Y., et al., 2021,1995,2019.Development and Research Center, China Geological Survey.
344	122.133	53.283	Au	Other magmatic-hydrothermal Au deposit	Medium	K	Li,C.Y., et al., 2021,1995,2019.Development and Research Center, China Geological Survey.
345	123.728	53.214	Au	Other magmatic-hydrothermal Au deposit	Small	K1	Li,C.Y., et al., 2021,1995,2019.Development and Research Center, China Geological Survey.
346	82.098	49.274	Au	Other magmatic-hydrothermal Au deposit	Small		Jiang Sihong, et al., 2019.unpublished internal report. 1-517.
347	74.234	51.736	Au	Other magmatic-hydrothermal Au deposit	Small		Jiang Sihong, et al., 2019.unpublished internal report. 1-517.
348	73.555	42.580	Au	Other magmatic-hydrothermal Au deposit	Small	O2	Jiang Sihong, et al., 2019.unpublished internal report. 1-517.
349	75.126	50.767	Au	Other magmatic-hydrothermal Au deposit	Small	O3	Jiang Sihong, et al., 2019.unpublished internal report. 1-517.
350	70.343	39.357	Au	Other magmatic-hydrothermal Au deposit	Small		Jiang Sihong, et al., 2019.unpublished internal report. 1-517.
351	87.359	41.543	Au		Small		Feng, J. et al., 2021. Geological Publishing House
352	129.667	43.167	Au	Other magmatic-hydrothermal Au deposit	Medium		Lv B. et al., 2017. Journal of Jilin University (Earth Science Edition) (In Chinese with English) Abstract)
353	95.181	43.949	Au	Other magmatic-hydrothermal Au deposit	Small		Li,C.Y., et al., 2021,1995,2019.Development and Research Center, China Geological Survey.
354	82.436	44.011	Au	Other magmatic-hydrothermal Au deposit	Small	C1	Feng, J. et al., 2021. Geological Publishing House
355	130.783	42.933	Au	Other magmatic-hydrothermal Au deposit	Medium		Lv B. et al., 2017. Journal of Jilin University (Earth Science Edition) (In Chinese with English) Abstract)
356	76.077	46.965	Au	Other magmatic-hydrothermal Au deposit	Small	P	Jiang Sihong, et al., 2019.unpublished internal report. 1-517.
357	73.136	52.494	Au	Other magmatic-hydrothermal Au deposit	Small	S	Jiang Sihong, et al., 2019.unpublished internal report. 1-517.
358	71.078	41.495	Au	Other magmatic-hydrothermal Au deposit	Small		Jiang Sihong, et al., 2019.unpublished internal report. 1-517.
359	75.731	41.754	Au	Other magmatic-hydrothermal Au deposit	Medium		Jiang Sihong, et al., 2019.unpublished internal report. 1-517.
360	129.917	48.383	Au	Other magmatic-hydrothermal Au deposit	Medium		Lv B. et al., 2017. Journal of Jilin University (Earth Science Edition) (In Chinese with English) Abstract)
361	73.900	48.652	Au	Other magmatic-hydrothermal Au deposit	Small	C-P	Jiang Sihong, et al., 2019.unpublished internal report. 1-517.
362	131.724	45.974	Au	Other magmatic-hydrothermal Au deposit	Large	Z1	Li,C.Y., et al., 2021,1995,2019.Development and Research Center, China Geological Survey.
363	79.046	45.197	Au	Other magmatic-hydrothermal Au deposit	Small	P	Jiang Sihong, et al., 2019.unpublished internal report. 1-517.
364	84.315	45.903	Au	Other magmatic-hydrothermal Au deposit	Medium		Li,C.Y., et al., 2021,1995,2019.Development and Research Center, China Geological Survey.
365	84.401	45.921	Au	Other magmatic-hydrothermal Au deposit	Small		Li,C.Y., et al., 2021,1995,2019.Development and Research Center, China Geological Survey.
366	71.189	42.058	Au	Other magmatic-hydrothermal Au deposit	Large	P	Jiang Sihong, et al., 2019.unpublished internal report. 1-517.
367	69.769	41.754	Au	Porphyry/Skarn Cu deposit	Medium		Jiang Sihong, et al., 2019.unpublished internal report. 1-517.
368	70.058	41.045	Au	Other magmatic-hydrothermal Au deposit	Small		Jiang Sihong, et al., 2019.unpublished internal report. 1-517.
369	92.173	40.533	Au		Small	C3	Feng, J. et al., 2021. Geological Publishing House
370	91.915	40.465	Au		Small	C3	Feng, J. et al., 2021. Geological Publishing House
371	91.905	40.411	Au		Small	C3	Li,C.Y., et al., 2021,1995,2019.Development and Research Center, China Geological Survey.
372	81.945	50.413	Au	Other magmatic-hydrothermal Au deposit	Small		Jiang Sihong, et al., 2019.unpublished internal report. 1-517.
373	86.363	48.322	Au	Other magmatic-hydrothermal Au deposit	Small	C3	Feng, J. et al., 2021. Geological Publishing House
374	72.349	39.242	Au	Other magmatic-hydrothermal Au deposit	Medium		Jiang Sihong, et al., 2019.unpublished internal report. 1-517.
375	75.181	41.923	Au	Other magmatic-hydrothermal Au deposit	Small	O3	Jiang Sihong, et al., 2019.unpublished internal report. 1-517.
376	70.008	41.035	Au	Other magmatic-hydrothermal Au deposit	Medium		Jiang Sihong, et al., 2019.unpublished internal report. 1-517.
377	74.161	50.416	Au	Porphyry/Skarn Cu deposit	Medium		Jiang Sihong, et al., 2019.unpublished internal report. 1-517.
378	88.232	47.871	Au	Other magmatic-hydrothermal Au deposit	Medium	D1	Feng, J. et al., 2021. Geological Publishing House
379	88.288	47.866	Au	Other magmatic-hydrothermal Au deposit	Small	D1	Feng, J. et al., 2021. Geological Publishing House
380	99.958	41.067	Au		Small		Li,C.Y., et al., 2021,1995,2019.Development and Research Center, China Geological Survey.
381	110.083	41.983	Au	Other magmatic-hydrothermal Au deposit	Medium		Lv B. et al., 2017. Journal of Jilin University (Earth Science Edition) (In Chinese with English) Abstract)

382	126.983	50.350	Au	Other magmatic-hydrothermal Au deposit	Medium		Lv B. et al., 2017. Journal of Jilin University (Earth Science Edition) (In Chinese with English) Abstract)
383	81.626	48.256	Au	Porphyry/Skarn Cu deposit	Medium		Jiang Sihong, et al., 2019.unpublished internal report. 1-517.
384	79.169	44.168	Au	Other magmatic-hydrothermal Au deposit	Small	P2-T1	Jiang Sihong, et al., 2019.unpublished internal report. 1-517.
385	82.467	49.325	Au	Other magmatic-hydrothermal Au deposit	Small		Jiang Sihong, et al., 2019.unpublished internal report. 1-517.
386	77.866	47.691	Au	Other magmatic-hydrothermal Au deposit	Small		Jiang Sihong, et al., 2019.unpublished internal report. 1-517.
387	121.833	53.200	Au		Large		Lv B. et al., 2017. Journal of Jilin University (Earth Science Edition) (In Chinese with English) Abstract)
388	90.117	42.192	Au	Other magmatic-hydrothermal Au deposit	Small	C1	Feng, J. et al., 2021. Geological Publishing House
389	90.856	42.056	Au	Other magmatic-hydrothermal Au deposit	Small	C1	Li,C.Y., et al., 2021,1995,2019.Development and Research Center, China Geological Survey.
390	91.083	42.017	Au	Other magmatic-hydrothermal Au deposit	Medium	C1	Li,C.Y., et al., 2021,1995,2019.Development and Research Center, China Geological Survey.
391	73.928	45.017	Au	Other magmatic-hydrothermal Au deposit	Small	C	Jiang Sihong, et al., 2019.unpublished internal report. 1-517.
392	90.224	42.082	Au	Other magmatic-hydrothermal Au deposit	Medium	C	Li,C.Y., et al., 2021,1995,2019.Development and Research Center, China Geological Survey.
393	90.197	42.086	Au	Other magmatic-hydrothermal Au deposit	Medium	P1	Feng, J. et al., 2021. Geological Publishing House
394	119.900	51.167	Au		Small	Q3-Q4	Li,C.Y., et al., 2021,1995,2019.Development and Research Center, China Geological Survey.
395	84.493	48.769	Au	Other magmatic-hydrothermal Au deposit	Small		Jiang Sihong, et al., 2019.unpublished internal report. 1-517.
396	70.818	52.845	Au	Other magmatic-hydrothermal Au deposit	Medium	O3	Jiang Sihong, et al., 2019.unpublished internal report. 1-517.
397	72.561	45.151	Au	Other magmatic-hydrothermal Au deposit	Medium	D1	Jiang Sihong, et al., 2019.unpublished internal report. 1-517.
398	119.558	42.308	Au	Other magmatic-hydrothermal Au deposit	Medium	K	Li,C.Y., et al., 2021,1995,2019.Development and Research Center, China Geological Survey.
399	133.633	46.483	Au	Other magmatic-hydrothermal Au deposit	Small		Lv B. et al., 2017. Journal of Jilin University (Earth Science Edition) (In Chinese with English) Abstract)
400	131.033	44.917	Au	Other magmatic-hydrothermal Au deposit	Medium		Lv B. et al., 2017. Journal of Jilin University (Earth Science Edition) (In Chinese with English) Abstract)
401	125.503	50.733	Au		Medium	K	Li,C.Y., et al., 2021,1995,2019.Development and Research Center, China Geological Survey.
402	75.318	50.881	Au	Other magmatic-hydrothermal Au deposit	Medium	O3	Jiang Sihong, et al., 2019.unpublished internal report. 1-517.
403	97.550	40.883	Au	Other magmatic-hydrothermal Au deposit	Small	O-S	Li,C.Y., et al., 2021,1995,2019.Development and Research Center, China Geological Survey.
404	97.733	40.865	Au	Other magmatic-hydrothermal Au deposit	Small	C	Li,C.Y., et al., 2021,1995,2019.Development and Research Center, China Geological Survey.
405	97.750	40.667	Au	Other magmatic-hydrothermal Au deposit	Small	Pz2	Li,C.Y., et al., 2021,1995,2019.Development and Research Center, China Geological Survey.
406	96.118	41.948	Au	Other magmatic-hydrothermal Au deposit	Medium	C-P	Li,C.Y., et al., 2021,1995,2019.Development and Research Center, China Geological Survey.
407	96.800	40.800	Au	Other magmatic-hydrothermal Au deposit	Medium	T	Li,C.Y., et al., 2021,1995,2019.Development and Research Center, China Geological Survey.
408	76.248	41.774	Au	Other magmatic-hydrothermal Au deposit	Medium	P	Jiang Sihong, et al., 2019.unpublished internal report. 1-517.
409	92.288	43.646	Au	Other magmatic-hydrothermal Au deposit	Small	C	Feng, J. et al., 2021. Geological Publishing House
410	75.674	42.654	Au	Other magmatic-hydrothermal Au deposit	Large	D-C	Jiang Sihong, et al., 2019.unpublished internal report. 1-517.
411	124.906	52.866	Au	Other magmatic-hydrothermal Au deposit	Small	K	Li,C.Y., et al., 2021,1995,2019.Development and Research Center, China Geological Survey.
412	80.108	50.158	Au	Other magmatic-hydrothermal Au deposit	Small		Jiang Sihong, et al., 2019.unpublished internal report. 1-517.
413	76.635	49.944	Au	Other magmatic-hydrothermal Au deposit	Small	O1-2	Jiang Sihong, et al., 2019.unpublished internal report. 1-517.
414	81.872	49.589	Au	Other magmatic-hydrothermal Au deposit	Small		Jiang Sihong, et al., 2019.unpublished internal report. 1-517.
415	74.945	43.064	Au	Other magmatic-hydrothermal Au deposit	Small		National Mineral Origin Database of China
416	77.771	47.741	Au	Other magmatic-hydrothermal Au deposit	Small	C2-P1	National Mineral Origin Database of China
417	89.267	47.897	Au	Other magmatic-hydrothermal Au deposit	Small	C1	Feng, J. et al., 2021. Geological Publishing House
418	72.413	51.995	Au	Other magmatic-hydrothermal Au deposit	Small	S	National Mineral Origin Database of China
419	85.008	48.874	Au	Other magmatic-hydrothermal Au deposit	Small		National Mineral Origin Database of China
420	71.168	41.536	Au	Other magmatic-hydrothermal Au deposit	Medium	P	National Mineral Origin Database of China
421	128.344	46.969	Au	Other magmatic-hydrothermal Au deposit	Medium	P2	Li,C.Y., et al., 2021,1995,2019.Development and Research Center, China Geological Survey.
422	128.477	46.969	Au	Other magmatic-hydrothermal Au deposit	Medium	Q	Li,C.Y., et al., 2021,1995,2019.Development and Research Center, China Geological Survey.
423	70.984	41.833	Au	Other magmatic-hydrothermal Au deposit	Small	C2	National Mineral Origin Database of China
424	78.304	47.514	Au	Other magmatic-hydrothermal Au deposit	Small		National Mineral Origin Database of China
425	79.425	45.172	Au	Other magmatic-hydrothermal Au deposit	Small		National Mineral Origin Database of China
426	130.267	48.367	Au	Porphyry/Skarn Cu deposit	Large		Lv B. et al., 2017. Journal of Jilin University (Earth Science Edition) (In Chinese with English) Abstract)
427	86.533	48.155	Au	Other magmatic-hydrothermal Au deposit	Medium	C-P	Feng, J. et al., 2021. Geological Publishing House
428	85.250	42.250	Au	Other magmatic-hydrothermal Au deposit	Small	Pz2	Li,C.Y., et al., 2021,1995,2019.Development and Research Center, China Geological Survey.
429	86.531	48.147	Au	Other magmatic-hydrothermal Au deposit	Small	Pz2	Li,C.Y., et al., 2021,1995,2019.Development and Research Center, China Geological Survey.
430	84.645	45.906	Au	Other magmatic-hydrothermal Au deposit	Medium	C3	Feng, J. et al., 2021. Geological Publishing House
431	84.312	45.902	Au	Other magmatic-hydrothermal Au deposit	Medium	C3	Feng, J. et al., 2021. Geological Publishing House
432	84.445	45.400	Au	Other magmatic-hydrothermal Au deposit	Small	C3	Feng, J. et al., 2021. Geological Publishing House
433	84.809	46.093	Au	Other magmatic-hydrothermal Au deposit	Small	C3	Feng, J. et al., 2021. Geological Publishing House
434	86.200	47.200	Au	Other magmatic-hydrothermal Au deposit	Small		Li,C.Y., et al., 2021,1995,2019.Development and Research Center, China Geological Survey.
435	69.291	53.427	Au	Other magmatic-hydrothermal Au deposit	Super Large	D3	National Mineral Origin Database of China
436	81.660	49.054	Au	Other magmatic-hydrothermal Au deposit	Medium	P	National Mineral Origin Database of China
437	130.460	43.321	Au	Other magmatic-hydrothermal Au deposit	Small	Mz	Li,C.Y., et al., 2021,1995,2019.Development and Research Center, China Geological Survey.
438	129.958	43.267	Au	Other magmatic-hydrothermal Au deposit	Medium	Mz	Li,C.Y., et al., 2021,1995,2019.Development and Research Center, China Geological Survey.
439	118.500	42.267	Au	Other magmatic-hydrothermal Au deposit	Medium	K	Li,C.Y., et al., 2021,1995,2019.Development and Research Center, China Geological Survey.
440	117.508	42.111	Au	Other magmatic-hydrothermal Au deposit	Medium		Li,C.Y., et al., 2021,1995,2019.Development and Research Center, China Geological Survey.
441	87.311	41.516	Au		Small	Pz	Feng, J. et al., 2021. Geological Publishing House
442	85.981	48.374	Au	Other magmatic-hydrothermal Au deposit	Medium	C3	Feng, J. et al., 2021. Geological Publishing House
443	83.117	49.113	Au	Other magmatic-hydrothermal Au deposit	Small		National Mineral Origin Database of China
444	74.313	40.088	Au		Large	D2	Feng, J. et al., 2021. Geological Publishing House
445	73.810	52.115	Au	Other magmatic-hydrothermal Au deposit	Small		National Mineral Origin Database of China

446	73.621	44.466	Au	Other magmatic-hydrothermal Au deposit	Small	S	National Mineral Origin Database of China
447	71.038	52.209	Au	Other magmatic-hydrothermal Au deposit	Small	S	National Mineral Origin Database of China
448	69.140	48.341	Au	Other magmatic-hydrothermal Au deposit	Small	D3	National Mineral Origin Database of China
449	75.146	50.881	Au	Other magmatic-hydrothermal Au deposit	Small	O3	National Mineral Origin Database of China
450	74.421	48.278	Au	Other magmatic-hydrothermal Au deposit	Small	C-P	National Mineral Origin Database of China
451	89.601	41.619	Au		Medium	C3	Feng, J. et al., 2021. Geological Publishing House
452	131.367	45.117	Au	Other magmatic-hydrothermal Au deposit	Medium		Lv B. et al., 2017. Journal of Jilin University (Earth Science Edition) (In Chinese with English) Abstract)
453	89.656	41.603	Au		Small	C3	Feng, J. et al., 2021. Geological Publishing House
454	120.017	46.600	Au	Other magmatic-hydrothermal Au deposit	Small		Lv B. et al., 2017. Journal of Jilin University (Earth Science Edition) (In Chinese with English) Abstract)
455	94.533	42.292	Au	Other magmatic-hydrothermal Au deposit	Small		Li,C.Y., et al., 2021,1995,2019.Development and Research Center, China Geological Survey.
456	99.967	41.050	Au		Medium		Lv B. et al., 2017. Journal of Jilin University (Earth Science Edition) (In Chinese with English) Abstract)
457	74.860	43.180	Au	Other magmatic-hydrothermal Au deposit	Small	O3	National Mineral Origin Database of China
458	95.083	42.024	Au	Other magmatic-hydrothermal Au deposit	Medium	C	Li,C.Y., et al., 2021,1995,2019.Development and Research Center, China Geological Survey.
459	91.136	42.027	Au	Other magmatic-hydrothermal Au deposit	Medium	C	Li,C.Y., et al., 2021,1995,2019.Development and Research Center, China Geological Survey.
460	74.250	40.037	Au	Other magmatic-hydrothermal Au deposit	Small	D1	Li,C.Y., et al., 2021,1995,2019.Development and Research Center, China Geological Survey.
461	81.517	44.333	Au	Other magmatic-hydrothermal Au deposit	Small	C1	Feng, J. et al., 2021. Geological Publishing House
462	124.150	51.400	Au	Other magmatic-hydrothermal Au deposit	Small	J3	Li,C.Y., et al., 2021,1995,2019.Development and Research Center, China Geological Survey.
463	69.844	52.642	Au	Other magmatic-hydrothermal Au deposit	Small	S	National Mineral Origin Database of China
464	129.322	43.053	Au	Other magmatic-hydrothermal Au deposit	Small	Mz	Li,C.Y., et al., 2021,1995,2019.Development and Research Center, China Geological Survey.
465	129.675	46.744	Au	Other magmatic-hydrothermal Au deposit	Small	J1	Li,C.Y., et al., 2021,1995,2019.Development and Research Center, China Geological Survey.
466	82.018	49.011	Au	Other magmatic-hydrothermal Au deposit	Small		National Mineral Origin Database of China
467	78.169	44.055	Au	Other magmatic-hydrothermal Au deposit	Small		National Mineral Origin Database of China
468	81.594	44.204	Au	Other magmatic-hydrothermal Au deposit	Small	C1	Feng, J. et al., 2021. Geological Publishing House
469	81.608	44.229	Au	Other magmatic-hydrothermal Au deposit	Large	C	Li,C.Y., et al., 2021,1995,2019.Development and Research Center, China Geological Survey.
470	81.524	44.324	Au	Other magmatic-hydrothermal Au deposit	Small	C1	Feng, J. et al., 2021. Geological Publishing House
471	81.691	44.207	Au	Other magmatic-hydrothermal Au deposit	Small	C1	Feng, J. et al., 2021. Geological Publishing House
472	81.674	44.145	Au	Other magmatic-hydrothermal Au deposit	Small	C1	Feng, J. et al., 2021. Geological Publishing House
473	81.605	44.212	Au	Other magmatic-hydrothermal Au deposit	Small	C1（D3）	Feng, J. et al., 2021. Geological Publishing House
474	81.707	44.214	Au	Other magmatic-hydrothermal Au deposit	Small	C1	Feng, J. et al., 2021. Geological Publishing House
475	70.959	41.497	Au	Other magmatic-hydrothermal Au deposit	Medium		National Mineral Origin Database of China
476	95.181	43.949	Au	Other magmatic-hydrothermal Au deposit	Small	D-C	Feng, J. et al., 2021. Geological Publishing House
477	128.867	44.150	Au	Other magmatic-hydrothermal Au deposit	Medium		Lv B. et al., 2017. Journal of Jilin University (Earth Science Edition) (In Chinese with English) Abstract)
478	126.183	44.150	Au	Other magmatic-hydrothermal Au deposit	Small	Mz	Li,C.Y., et al., 2021,1995,2019.Development and Research Center, China Geological Survey.
479	82.751	49.082	Au	Other magmatic-hydrothermal Au deposit	Small	C-P	National Mineral Origin Database of China
480	86.531	48.151	Au	Other magmatic-hydrothermal Au deposit	Medium		Li,C.Y., et al., 2021,1995,2019.Development and Research Center, China Geological Survey.
481	73.387	48.476	Au	Other magmatic-hydrothermal Au deposit	Small	P	National Mineral Origin Database of China
482	68.573	48.480	Au	Other magmatic-hydrothermal Au deposit	Small	D3	National Mineral Origin Database of China
483	80.133	42.423	Au	Other magmatic-hydrothermal Au deposit	Medium	D3	National Mineral Origin Database of China
484	70.540	46.581	Au	Other magmatic-hydrothermal Au deposit	Small	D2	National Mineral Origin Database of China
485	70.947	53.238	Au	Other magmatic-hydrothermal Au deposit	Small	O2	National Mineral Origin Database of China
486	71.573	52.639	Au	Other magmatic-hydrothermal Au deposit	Small	O3	National Mineral Origin Database of China
487	78.868	41.732	Au	Other magmatic-hydrothermal Au deposit	Small		National Mineral Origin Database of China
488	81.935	48.335	Au	Porphyry/Skarn Cu deposit	Medium		National Mineral Origin Database of China
489	74.408	51.715	Au	Other magmatic-hydrothermal Au deposit	Small	C2	National Mineral Origin Database of China
490	125.667	43.808	Au	Other magmatic-hydrothermal Au deposit	Medium	Mz	Li,C.Y., et al., 2021,1995,2019.Development and Research Center, China Geological Survey.
491	125.867	50.200	Au	Other magmatic-hydrothermal Au deposit	Medium		Lv B. et al., 2017. Journal of Jilin University (Earth Science Edition) (In Chinese with English) Abstract)
492	74.126	48.284	Au	Other magmatic-hydrothermal Au deposit	Small	P	National Mineral Origin Database of China
493	119.600	42.283	Au	Other magmatic-hydrothermal Au deposit	Small		Lv B. et al., 2017. Journal of Jilin University (Earth Science Edition) (In Chinese with English) Abstract)
494	78.283	47.445	Au	Other magmatic-hydrothermal Au deposit	Small		National Mineral Origin Database of China
495	90.290	47.290	Be	Li-Be-Nb-Ta-Zr deposit	Medium		National Mineral Origin Database of China
496	87.943	48.942	Be	Li-Be-Nb-Ta-Zr deposit	Medium	J1	National Mineral Origin Database of China
497	92.593	52.504	Be	Li-Be-Nb-Ta-Zr deposit			Seltmann, R., et al., 2004. Natural History Museum
498	91.383	46.517	Be	Li-Be-Nb-Ta-Zr deposit	Small	P2	Ochir Gerel et al., 2021 Mineral Resources of Mongolia (book) and refs therein
499	84.430	51.170	Be	Li-Be-Nb-Ta-Zr deposit	Small		National Mineral Origin Database of China
500	84.330	51.350	Be	Li-Be-Nb-Ta-Zr deposit	Medium		National Mineral Origin Database of China
501	89.820	47.200	Be	Li-Be-Nb-Ta-Zr deposit	Large		National Mineral Origin Database of China
502	82.670	51.200	Be	Li-Be-Nb-Ta-Zr deposit	Small		National Mineral Origin Database of China
503	88.840	47.910	Be	Li-Be-Nb-Ta-Zr deposit	Small		National Mineral Origin Database of China
504	84.830	51.880	Be	Li-Be-Nb-Ta-Zr deposit	Small		National Mineral Origin Database of China
505	87.921	49.118	Be	Li-Be-Nb-Ta-Zr deposit	Medium	D?	Ochir Gerel et al., 2021 Mineral Resources of Mongolia (book) and refs therein
506	88.830	47.750	Be	Li-Be-Nb-Ta-Zr deposit	Medium		National Mineral Origin Database of China
507	89.676	47.691	Be	Li-Be-Nb-Ta-Zr deposit	Medium	D	Ochir Gerel et al., 2021 Mineral Resources of Mongolia (book) and refs therein
508	83.030	51.030	Be	Li-Be-Nb-Ta-Zr deposit	Small		National Mineral Origin Database of China
509	91.867	46.353	Be	Li-Be-Nb-Ta-Zr deposit	Medium	T	Ochir Gerel et al., 2021 Mineral Resources of Mongolia (book) and refs therein

510	88.363	47.685	Be	Li-Be-Nb-Ta-Zr deposit	Medium	Pz2	National Mineral Origin Database of China
511	88.833	47.850	Be	Li-Be-Nb-Ta-Zr deposit	Small	C	National Mineral Origin Database of China
512	88.800	47.933	Be	Li-Be-Nb-Ta-Zr deposit	Occurrence		National Mineral Origin Database of China
513	88.683	47.650	Be	Li-Be-Nb-Ta-Zr deposit	Occurrence		National Mineral Origin Database of China
514	88.780	47.788	Be	Li-Be-Nb-Ta-Zr deposit	Occurrence		National Mineral Origin Database of China
515	88.883	47.717	Be	Li-Be-Nb-Ta-Zr deposit	Occurrence		National Mineral Origin Database of China
516	89.117	47.800	Be	Li-Be-Nb-Ta-Zr deposit	Occurrence		National Mineral Origin Database of China
517	89.083	47.733	Be	Li-Be-Nb-Ta-Zr deposit	Occurrence		National Mineral Origin Database of China
518	89.717	47.217	Be	Li-Be-Nb-Ta-Zr deposit	Occurrence		National Mineral Origin Database of China
519	89.550	47.483	Be	Li-Be-Nb-Ta-Zr deposit	Occurrence		National Mineral Origin Database of China
520	89.983	47.150	Be	Li-Be-Nb-Ta-Zr deposit	Occurrence		National Mineral Origin Database of China
521	88.367	47.517	Be	Li-Be-Nb-Ta-Zr deposit	Occurrence		National Mineral Origin Database of China
522	88.683	47.767	Be	Li-Be-Nb-Ta-Zr deposit	Small	Pz2	National Mineral Origin Database of China
523	88.167	47.944	Be	Li-Be-Nb-Ta-Zr deposit	Small	C	National Mineral Origin Database of China
524	89.590	47.330	Be	Li-Be-Nb-Ta-Zr deposit	Small	C	National Mineral Origin Database of China
525	89.815	47.207	Be	Li-Be-Nb-Ta-Zr deposit	Large	D	National Mineral Origin Database of China
526	89.810	47.275	Be	Li-Be-Nb-Ta-Zr deposit	Small		National Mineral Origin Database of China
527	89.825	47.257	Be	Li-Be-Nb-Ta-Zr deposit	Small	C	National Mineral Origin Database of China
528	89.067	47.733	Be	Li-Be-Nb-Ta-Zr deposit	Small	C	National Mineral Origin Database of China
529	89.342	47.458	Be	Li-Be-Nb-Ta-Zr deposit	Small	C	National Mineral Origin Database of China
530	90.295	47.296	Be	Li-Be-Nb-Ta-Zr deposit	Medium		Seltmann, R., et al., 2004. Natural History Museum
531	90.300	47.317	Be-Nb-Mo	Li-Be-Nb-Ta-Zr deposit	Occurrence		National Mineral Origin Database of China
532	88.480	47.711	Be-Nb-Ta	Li-Be-Nb-Ta-Zr deposit	Occurrence		National Mineral Origin Database of China
533	88.683	47.700	Be-Nb-Ta	Li-Be-Nb-Ta-Zr deposit	Occurrence		National Mineral Origin Database of China
534	85.970	53.270	Co	Li-Be-Nb-Ta-Zr deposit	Small		National Mineral Origin Database of China
535	85.920	53.130	Co	Li-Be-Nb-Ta-Zr deposit	Medium		National Mineral Origin Database of China
536	91.100	51.040	Co	Li-Be-Nb-Ta-Zr deposit	Small		National Mineral Origin Database of China
537	85.730	53.220	Co	Li-Be-Nb-Ta-Zr deposit	Small		National Mineral Origin Database of China
538	90.550	50.500	Co	Li-Be-Nb-Ta-Zr deposit	Small		National Mineral Origin Database of China
539	82.945	44.080	Cu	Porphyry/Skarn Cu-Au deposit	Medium	C1	Feng, J. et al., 2021. Geological Publishing House
540	90.067	46.544	Cu	Other magmatic-hydrothermal Cu deposit	Small	D2	Feng, J. et al., 2021. Geological Publishing House
541	90.089	46.514	Cu	Other magmatic-hydrothermal Cu deposit	Small	D2	Feng, J. et al., 2021. Geological Publishing House
542	61.820	51.100	Cu	Porphyry/Skarn Cu-Au deposit		C	Wu, C. et al., 2022. Mineralium Deposita
543	76.250	48.330	Cu	Porphyry/Skarn Cu-Au deposit		D	Wu, C. et al., 2022. Mineralium Deposita
544	73.950	47.520	Cu	Porphyry/Skarn Cu-Au deposit		C	Wu, C. et al., 2022. Mineralium Deposita
545	93.950	49.317	Cu	Porphyry/Skarn Cu-Au deposit	Small	NP3-KE1	Ochir Gerel et al., 2021 Mineral Resources of Mongolia (book) and refs therein
546	90.130	52.710	Cu	Porphyry/Skarn Cu-Au deposit	Medium		National Mineral Origin Database of China
547	99.228	42.163	Cu	Porphyry/Skarn Cu-Au deposit	Small		National Mineral Origin Database of China
548	90.070	46.550	Cu	Porphyry/Skarn Cu-Au deposit		D	Wu, C. et al., 2022. Mineralium Deposita
549	90.068	46.567	Cu	Porphyry/Skarn Cu-Au deposit	Medium	D2	National Mineral Origin Database of China
550	90.073	46.538	Cu	Porphyry/Skarn Cu-Au deposit	Medium	D2	National Mineral Origin Database of China
551	90.091	46.518	Cu	Porphyry/Skarn Cu-Au deposit	Medium		National Mineral Origin Database of China
552	95.003	48.250	Cu	Porphyry/Skarn Cu-Au deposit	Occurrence	PZ3	Ochir Gerel et al., 2021 Mineral Resources of Mongolia (book) and refs therein
553	77.640	48.090	Cu	Porphyry/Skarn Cu-Au deposit		C	Wu, C. et al., 2022. Mineralium Deposita
554	73.840	46.660	Cu	Porphyry/Skarn Cu-Au deposit		D	Wu, C. et al., 2022. Mineralium Deposita
555	75.210	47.350	Cu	Porphyry/Skarn Cu-Au deposit		C	Wu, C. et al., 2022. Mineralium Deposita
556	90.120	52.750	Cu	Porphyry/Skarn Cu-Au deposit	Medium		National Mineral Origin Database of China
557	106.144	44.031	Cu	Porphyry/Skarn Cu-Au deposit	Medium	C2	Ochir Gerel et al., 2021 Mineral Resources of Mongolia (book) and refs therein
558	90.374	48.294	Cu	Porphyry/Skarn Cu-Au deposit	Medium		National Mineral Origin Database of China
559	107.835	44.060	Cu	Porphyry/Skarn Cu-Au deposit	Small	C2	Ochir Gerel et al., 2021 Mineral Resources of Mongolia (book) and refs therein
560	62.030	51.670	Cu	Porphyry/Skarn Cu-Au deposit		C	Wu, C. et al., 2022. Mineralium Deposita
561	94.270	52.170	Cu	Porphyry/Skarn Cu-Au deposit		∈	Wu, C. et al., 2022. Mineralium Deposita
562	84.420	47.530	Cu	Porphyry/Skarn Cu-Au deposit		C	Wu, C. et al., 2022. Mineralium Deposita
563	80.000	47.000	Cu	Porphyry/Skarn Cu-Au deposit		C	Wu, C. et al., 2022. Mineralium Deposita
564	91.420	52.970	Cu	Porphyry/Skarn Cu-Au deposit	Small		National Mineral Origin Database of China
565	95.311	44.192	Cu	Porphyry/Skarn Cu-Au deposit	Medium		National Mineral Origin Database of China
566	96.500	46.167	Cu	Porphyry/Skarn Cu-Au deposit	Occurrence	NP3-KE1	Ochir Gerel et al., 2021 Mineral Resources of Mongolia (book) and refs therein
567	108.042	44.242	Cu	Porphyry/Skarn Cu-Au deposit	Small	PZ3	Ochir Gerel et al., 2021 Mineral Resources of Mongolia (book) and refs therein
568	106.875	43.028	Cu	Porphyry/Skarn Cu-Au deposit	Large	D1	Ochir Gerel et al., 2021 Mineral Resources of Mongolia (book) and refs therein
569	109.467	44.583	Cu	Porphyry/Skarn Cu-Au deposit	Small	PZ3	Ochir Gerel et al., 2021 Mineral Resources of Mongolia (book) and refs therein
570	75.940	49.370	Cu	Porphyry/Skarn Cu-Au deposit		C	Wu, C. et al., 2022. Mineralium Deposita
571	95.213	44.168	Cu	Porphyry/Skarn Cu-Au deposit	Small		National Mineral Origin Database of China
572	73.886	47.431	Cu	Porphyry/Skarn Cu-Au deposit	Small	D3	National Mineral Origin Database of China
573	100.600	45.783	Cu	Porphyry/Skarn Cu-Au deposit	Small	PZ3	Ochir Gerel et al., 2021 Mineral Resources of Mongolia (book) and refs therein

574	73.670	46.000	Cu	Porphyry/Skarn Cu-Au deposit		D	Wu, C. et al., 2022. Mineralium Deposita
575	77.431	47.012	Cu	Porphyry/Skarn Cu-Au deposit	Medium	P	National Mineral Origin Database of China
576	90.317	48.392	Cu	Porphyry/Skarn Cu-Au deposit	Medium		National Mineral Origin Database of China
577	108.333	43.872	Cu	Porphyry/Skarn Cu-Au deposit	Large	D1	Ochir Gerel et al., 2021 Mineral Resources of Mongolia (book) and refs therein
578	96.433	46.033	Cu	Porphyry/Skarn Cu-Au deposit	Occurrence	PZ3	Ochir Gerel et al., 2021 Mineral Resources of Mongolia (book) and refs therein
579	94.761	48.299	Cu	Other magmatic-hydrothermal Cu deposit	Occurrence	PZ3	Ochir Gerel et al., 2021 Mineral Resources of Mongolia (book) and refs therein
580	92.228	44.271	Cu	Porphyry/Skarn Cu-Au deposit	Small		National Mineral Origin Database of China
581	89.225	46.827	Cu	Porphyry/Skarn Cu-Au deposit	Medium		National Mineral Origin Database of China
582	90.026	46.606	Cu	Porphyry/Skarn Cu-Au deposit	Small		National Mineral Origin Database of China
583	90.014	46.608	Cu	Porphyry/Skarn Cu-Au deposit	Medium		National Mineral Origin Database of China
584	77.723	48.704	Cu	Other magmatic-hydrothermal Cu deposit	Small	S1	National Mineral Origin Database of China
585	127.244	45.354	Cu	Other magmatic-hydrothermal Cu deposit	Medium	T3	Li,C.Y., et al., 2021,1995,2019.Development and Research Center, China Geological Survey.
586	75.068	42.491	Cu	Other magmatic-hydrothermal Cu deposit	Small		National Mineral Origin Database of China
587	116.268	48.783	Cu	Other magmatic-hydrothermal Cu deposit	Medium	K	Li,C.Y., et al., 2021,1995,2019.Development and Research Center, China Geological Survey.
588	73.999	49.847	Cu	Other magmatic-hydrothermal Cu deposit	Small	D3-C1	National Mineral Origin Database of China
589	73.992	48.657	Cu	Porphyry/Skarn Cu-Au deposit	Medium	C-P	National Mineral Origin Database of China
590	86.555	46.771	Cu	Other magmatic-hydrothermal Cu deposit	Small	C3	Feng, J. et al., 2021. Geological Publishing House
591	74.543	43.528	Cu	Porphyry/Skarn Cu-Au deposit	Medium		National Mineral Origin Database of China
592	74.019	48.093	Cu	Porphyry/Skarn Cu-Au deposit	Small	P	National Mineral Origin Database of China
593	79.998	46.996	Cu	Porphyry/Skarn Cu-Au deposit	Large (Super Large	P	National Mineral Origin Database of China
594	74.573	43.858	Cu	Other magmatic-hydrothermal Cu deposit	Small		National Mineral Origin Database of China
595	70.603	49.246	Cu	Porphyry/Skarn Cu-Au deposit	Small		National Mineral Origin Database of China
596	82.150	45.150	Cu	Other magmatic-hydrothermal Cu deposit	Small	C3（C1）	Feng, J. et al., 2021. Geological Publishing House
597	74.356	43.743	Cu	Porphyry/Skarn Cu-Au deposit	Small		National Mineral Origin Database of China
598	77.733	48.641	Cu	Other magmatic-hydrothermal Cu deposit	Medium	O3	National Mineral Origin Database of China
599	83.009	50.674	Cu	Other magmatic-hydrothermal Cu deposit	Small	D2-3	National Mineral Origin Database of China
600	74.266	43.631	Cu	Porphyry/Skarn Cu-Au deposit	Medium	D3	National Mineral Origin Database of China
601	86.342	48.286	Cu	Other magmatic-hydrothermal Cu deposit	Large	D2	Feng, J. et al., 2021. Geological Publishing House
602	86.366	48.271	Cu	Other magmatic-hydrothermal Cu deposit	Large		Li,C.Y., et al., 2021,1995,2019.Development and Research Center, China Geological Survey.
603	86.344	48.279	Cu	Other magmatic-hydrothermal Cu deposit	Small	D2	Li,C.Y., et al., 2021,1995,2019.Development and Research Center, China Geological Survey.
604	80.548	48.052	Cu	Other magmatic-hydrothermal Cu deposit	Small		National Mineral Origin Database of China
605	74.162	51.055	Cu	Other magmatic-hydrothermal Cu deposit	Small	D	National Mineral Origin Database of China
606	80.389	47.626	Cu	Other magmatic-hydrothermal Cu deposit	Small	P2	National Mineral Origin Database of China
607	75.248	48.588	Cu	Other magmatic-hydrothermal Cu deposit	Small	P	National Mineral Origin Database of China
608	118.967	43.850	Cu	Other magmatic-hydrothermal Cu deposit	Small		Lv B. et al., 2017. Journal of Jilin University (Earth Science Edition) (In Chinese with English) Abstract)
609	120.217	44.533	Cu	Other magmatic-hydrothermal Cu deposit	Medium		Lv B. et al., 2017. Journal of Jilin University (Earth Science Edition) (In Chinese with English) Abstract)
610	79.937	46.999	Cu	Porphyry/Skarn Cu-Au deposit	Large	C2	National Mineral Origin Database of China
611	81.368	50.942	Cu	Other magmatic-hydrothermal Cu deposit	Large	D2-3	National Mineral Origin Database of China
612	75.964	49.369	Cu	Porphyry/Skarn Cu-Au deposit	Medium	C2	National Mineral Origin Database of China
613	71.631	42.903	Cu	Porphyry/Skarn Cu-Au deposit	Small		National Mineral Origin Database of China
614	106.850	43.000	Cu	Porphyry/Skarn Cu-Au deposit	Large		Lv B. et al., 2017. Journal of Jilin University (Earth Science Edition) (In Chinese with English) Abstract)
615	116.083	45.583	Cu	Porphyry/Skarn Cu-Au deposit	Small		Lv B. et al., 2017. Journal of Jilin University (Earth Science Edition) (In Chinese with English) Abstract)
616	116.045	45.572	Cu	Other magmatic-hydrothermal Cu deposit	Small	J2-K	Li,C.Y., et al., 2021,1995,2019.Development and Research Center, China Geological Survey.
617	118.900	49.767	Cu	Porphyry/Skarn Cu-Au deposit	Medium		Lv B. et al., 2017. Journal of Jilin University (Earth Science Edition) (In Chinese with English) Abstract)
618	119.083	49.967	Cu	Porphyry/Skarn Cu-Au deposit	Medium		Lv B. et al., 2017. Journal of Jilin University (Earth Science Edition) (In Chinese with English) Abstract)
619	80.058	48.189	Cu	Porphyry/Skarn Cu-Au deposit	Small	C	National Mineral Origin Database of China
620	122.327	46.992	Cu	Porphyry/Skarn Cu-Au deposit	Small	D-P	Li,C.Y., et al., 2021,1995,2019.Development and Research Center, China Geological Survey.
621	82.892	45.658	Cu	Other magmatic-hydrothermal Cu deposit	Small	C1	Feng, J. et al., 2021. Geological Publishing House
622	122.220	48.284	Cu	Porphyry/Skarn Cu-Au deposit	Small	D-P	Li,C.Y., et al., 2021,1995,2019.Development and Research Center, China Geological Survey.
623	78.991	47.986	Cu	Porphyry/Skarn Cu-Au deposit	Small		National Mineral Origin Database of China
624	70.764	49.173	Cu	Porphyry/Skarn Cu-Au deposit	Medium		National Mineral Origin Database of China
625	119.088	49.971	Cu	Other magmatic-hydrothermal Cu deposit	Small	C1	Li,C.Y., et al., 2021,1995,2019.Development and Research Center, China Geological Survey.
626	99.938	41.157	Cu	Porphyry/Skarn Cu-Au deposit	Small	P	Li,C.Y., et al., 2021,1995,2019.Development and Research Center, China Geological Survey.
627	106.314	41.221	Cu	Other magmatic-hydrothermal Cu deposit	Small	J	Li,C.Y., et al., 2021,1995,2019.Development and Research Center, China Geological Survey.
628	119.767	42.367	Cu	Other magmatic-hydrothermal Cu deposit	Small		Lv B. et al., 2017. Journal of Jilin University (Earth Science Edition) (In Chinese with English) Abstract)
629	112.500	42.250	Cu	Porphyry/Skarn Cu-Au deposit	Medium		Lv B. et al., 2017. Journal of Jilin University (Earth Science Edition) (In Chinese with English) Abstract)
630	109.474	42.441	Cu	Other magmatic-hydrothermal Cu deposit	Small	C2	Li,C.Y., et al., 2021,1995,2019.Development and Research Center, China Geological Survey.
631	121.183	42.667	Cu	Other magmatic-hydrothermal Cu deposit	Small	J2-K	Li,C.Y., et al., 2021,1995,2019.Development and Research Center, China Geological Survey.
632	119.483	44.542	Cu	Other magmatic-hydrothermal Cu deposit	Medium	J2	Li,C.Y., et al., 2021,1995,2019.Development and Research Center, China Geological Survey.
633	75.716	49.202	Cu	Porphyry/Skarn Cu-Au deposit	Medium	C	National Mineral Origin Database of China
634	84.539	45.473	Cu	Porphyry/Skarn Cu-Au deposit	Large	C3	Feng, J. et al., 2021. Geological Publishing House
635	77.013	49.234	Cu	Other magmatic-hydrothermal Cu deposit	Small	P	National Mineral Origin Database of China
636	83.101	49.471	Cu	Other magmatic-hydrothermal Cu deposit	Large	D1	National Mineral Origin Database of China
637	127.444	45.544	Cu	Porphyry/Skarn Cu-Au deposit	Medium	K1	Li,C.Y., et al., 2021,1995,2019.Development and Research Center, China Geological Survey.
638	127.183	45.633	Cu	Porphyry/Skarn Cu-Au deposit	Small	T1	Li,C.Y., et al., 2021,1995,2019.Development and Research Center, China Geological Survey.
639	71.065	41.302	Cu	Porphyry/Skarn Cu-Au deposit	Medium	C2	National Mineral Origin Database of China

640	74.733	47.194	Cu	Porphyry/Skarn Cu-Au deposit	Medium	P	National Mineral Origin Database of China
641	90.583	43.717	Cu	Other magmatic-hydrothermal Cu deposit	Small	C3	Feng, J. et al., 2021. Geological Publishing House
642	74.354	51.859	Cu	Porphyry/Skarn Cu-Au deposit	Large (Super Large	C1	National Mineral Origin Database of China
643	121.325	44.843	Cu	Other magmatic-hydrothermal Cu deposit	Medium	J2	Li,C.Y., et al., 2021,1995,2019.Development and Research Center, China Geological Survey.
644	83.811	49.574	Cu	Other magmatic-hydrothermal Cu deposit	Small		National Mineral Origin Database of China
645	113.374	42.552	Cu	Other magmatic-hydrothermal Cu deposit	Small	T-J1	Li,C.Y., et al., 2021,1995,2019.Development and Research Center, China Geological Survey.
646	88.817	42.150	Cu	Other magmatic-hydrothermal Cu deposit	Small	D1	Feng, J. et al., 2021. Geological Publishing House
647	88.912	42.161	Cu	Other magmatic-hydrothermal Cu deposit	Medium	D1	Feng, J. et al., 2021. Geological Publishing House
648	108.317	43.850	Cu	Porphyry/Skarn Cu-Au deposit	Large		Lv B. et al., 2017. Journal of Jilin University (Earth Science Edition) (In Chinese with English) Abstract)
649	84.917	43.417	Cu	Other magmatic-hydrothermal Cu deposit	Large	C1	Feng, J. et al., 2021. Geological Publishing House
650	84.925	43.336	Cu	Other magmatic-hydrothermal Cu deposit	Small	P	Li,C.Y., et al., 2021,1995,2019.Development and Research Center, China Geological Survey.
651	88.126	43.652	Cu	Other magmatic-hydrothermal Cu deposit	Small	C3	Feng, J. et al., 2021. Geological Publishing House
652	88.471	43.408	Cu	Other magmatic-hydrothermal Cu deposit	Small	C3	Feng, J. et al., 2021. Geological Publishing House
653	118.508	42.419	Cu	Other magmatic-hydrothermal Cu deposit	Small	J2-K	Li,C.Y., et al., 2021,1995,2019.Development and Research Center, China Geological Survey.
654	118.000	44.250	Cu	Other magmatic-hydrothermal Cu deposit	Medium		Lv B. et al., 2017. Journal of Jilin University (Earth Science Edition) (In Chinese with English) Abstract)
655	117.743	45.928	Cu	Other magmatic-hydrothermal Cu deposit	Small	J2-K	Li,C.Y., et al., 2021,1995,2019.Development and Research Center, China Geological Survey.
656	93.771	43.422	Cu	Other magmatic-hydrothermal Cu deposit	Small	C3	Feng, J. et al., 2021. Geological Publishing House
657	93.333	41.917	Cu	Porphyry/Skarn Cu-Au deposit	Small	C1	Feng, J. et al., 2021. Geological Publishing House
658	118.860	49.736	Cu	Other magmatic-hydrothermal Cu deposit	Small	J2-K	Li,C.Y., et al., 2021,1995,2019.Development and Research Center, China Geological Survey.
659	125.783	50.233	Cu	Porphyry/Skarn Cu-Au deposit	Large		Lv B. et al., 2017. Journal of Jilin University (Earth Science Edition) (In Chinese with English) Abstract)
660	73.838	41.483	Cu	Other magmatic-hydrothermal Cu deposit	Small		National Mineral Origin Database of China
661	100.450	40.963	Cu	Other magmatic-hydrothermal Cu deposit	Small	J2-K	Li,C.Y., et al., 2021,1995,2019.Development and Research Center, China Geological Survey.
662	116.698	45.104	Cu	Other magmatic-hydrothermal Cu deposit	Small	C	Li,C.Y., et al., 2021,1995,2019.Development and Research Center, China Geological Survey.
663	74.337	43.577	Cu	Porphyry/Skarn Cu-Au deposit	Medium		National Mineral Origin Database of China
664	128.346	47.178	Cu	Porphyry/Skarn Cu-Au deposit	Small	S2	Li,C.Y., et al., 2021,1995,2019.Development and Research Center, China Geological Survey.
665	128.338	47.169	Cu	Porphyry/Skarn Cu-Au deposit	Medium	P-T	Li,C.Y., et al., 2021,1995,2019.Development and Research Center, China Geological Survey.
666	128.328	47.166	Cu	Porphyry/Skarn Cu-Au deposit	Medium	K2	Li,C.Y., et al., 2021,1995,2019.Development and Research Center, China Geological Survey.
667	74.954	49.919	Cu	Other magmatic-hydrothermal Cu deposit	Small		National Mineral Origin Database of China
668	121.846	45.578	Cu	Other magmatic-hydrothermal Cu deposit	Medium	K1	Li,C.Y., et al., 2021,1995,2019.Development and Research Center, China Geological Survey.
669	81.556	47.600	Cu	Ni-Cu sulfide deposit	Small	D2?	National Mineral Origin Database of China
670	127.433	45.533	Cu	Other magmatic-hydrothermal Cu deposit	Medium		Lv B. et al., 2017. Journal of Jilin University (Earth Science Edition) (In Chinese with English) Abstract)
671	118.261	43.672	Cu	Other magmatic-hydrothermal Cu deposit	Medium	J	Li,C.Y., et al., 2021,1995,2019.Development and Research Center, China Geological Survey.
672	90.062	46.573	Cu	Porphyry/Skarn Cu-Au deposit	Medium	D2	Feng, J. et al., 2021. Geological Publishing House
673	83.058	44.071	Cu	Porphyry/Skarn Cu-Au deposit	Small	C3	Feng, J. et al., 2021. Geological Publishing House
674	81.517	44.667	Cu	Other magmatic-hydrothermal Cu deposit	Small	C3	Feng, J. et al., 2021. Geological Publishing House
675	120.317	43.883	Cu	Porphyry/Skarn Cu-Au deposit	Medium		Lv B. et al., 2017. Journal of Jilin University (Earth Science Edition) (In Chinese with English) Abstract)
676	95.232	43.814	Cu	Porphyry/Skarn Cu-Au deposit	Small	D1	Feng, J. et al., 2021. Geological Publishing House
677	126.867	50.000	Cu	Other magmatic-hydrothermal Cu deposit	Small	P1	Li,C.Y., et al., 2021,1995,2019.Development and Research Center, China Geological Survey.
678	91.566	41.767	Cu	Other magmatic-hydrothermal Cu deposit	Small	C3	Feng, J. et al., 2021. Geological Publishing House
679	91.612	42.172	Cu	Other magmatic-hydrothermal Cu deposit	Small	C1	Feng, J. et al., 2021. Geological Publishing House
680	91.600	41.967	Cu	Other magmatic-hydrothermal Cu deposit	Small	C1	Feng, J. et al., 2021. Geological Publishing House
681	91.917	42.626	Cu	Other magmatic-hydrothermal Cu deposit	Small	O2	Feng, J. et al., 2021. Geological Publishing House
682	123.319	52.269	Cu	Porphyry/Skarn Cu-Au deposit	Small	γ5	Li,C.Y., et al., 2021,1995,2019.Development and Research Center, China Geological Survey.
683	126.704	43.315	Cu	Other magmatic-hydrothermal Cu deposit	Small	Mz	Li,C.Y., et al., 2021,1995,2019.Development and Research Center, China Geological Survey.
684	126.639	43.364	Cu	Other magmatic-hydrothermal Cu deposit	Small	Mz	Li,C.Y., et al., 2021,1995,2019.Development and Research Center, China Geological Survey.
685	86.362	48.273	Cu	Other magmatic-hydrothermal Cu deposit	Small	D2	Li,C.Y., et al., 2021,1995,2019.Development and Research Center, China Geological Survey.
686	94.575	42.304	Cu	Other magmatic-hydrothermal Cu deposit	Medium	P	Li,C.Y., et al., 2021,1995,2019.Development and Research Center, China Geological Survey.
687	94.617	42.850	Cu	Ni-Cu sulfide deposit	Large	C	Li,C.Y., et al., 2021,1995,2019.Development and Research Center, China Geological Survey.
688	94.650	42.221	Cu	Other magmatic-hydrothermal Cu deposit	Large	Pz2	Li,C.Y., et al., 2021,1995,2019.Development and Research Center, China Geological Survey.
689	91.925	42.608	Cu	Other magmatic-hydrothermal Cu deposit	Medium	D1	Feng, J. et al., 2021. Geological Publishing House
690	130.883	43.200	Cu	Other magmatic-hydrothermal Cu deposit	Large	Mz	Li,C.Y., et al., 2021,1995,2019.Development and Research Center, China Geological Survey.
691	125.633	50.250	Cu	Porphyry/Skarn Cu-Au deposit	Small		Lv B. et al., 2017. Journal of Jilin University (Earth Science Edition) (In Chinese with English) Abstract)
692	92.523	41.764	Cu	Other magmatic-hydrothermal Cu deposit	Small	C3	Feng, J. et al., 2021. Geological Publishing House
693	73.618	49.615	Cu	Other magmatic-hydrothermal Cu deposit	Small		National Mineral Origin Database of China
694	121.367	44.917	Cu	Porphyry/Skarn Cu-Au deposit	Medium		Lv B. et al., 2017. Journal of Jilin University (Earth Science Edition) (In Chinese with English) Abstract)
695	89.692	46.756	Cu	Ni-Cu sulfide deposit	Small		Li,C.Y., et al., 2021,1995,2019.Development and Research Center, China Geological Survey.
696	89.692	46.756	Cu	Ni-Cu sulfide deposit	Medium	C-P	Li,C.Y., et al., 2021,1995,2019.Development and Research Center, China Geological Survey.
697	89.693	46.756	Cu	Ni-Cu sulfide deposit	Small	C-P	Li,C.Y., et al., 2021,1995,2019.Development and Research Center, China Geological Survey.
698	89.692	46.756	Cu	Ni-Cu sulfide deposit	Small		Li,C.Y., et al., 2021,1995,2019.Development and Research Center, China Geological Survey.
699	72.927	48.411	Cu	Other magmatic-hydrothermal Cu deposit	Small	p	National Mineral Origin Database of China
700	73.925	46.700	Cu	Porphyry/Skarn Cu-Au deposit	Medium	P	National Mineral Origin Database of China
701	73.866	46.653	Cu	Porphyry/Skarn Cu-Au deposit	Medium		National Mineral Origin Database of China
702	106.150	44.000	Cu	Porphyry/Skarn Cu-Au deposit	Large		Lv B. et al., 2017. Journal of Jilin University (Earth Science Edition) (In Chinese with English) Abstract)
703	69.843	40.812	Cu	Porphyry/Skarn Cu-Au deposit	Medium	Pz2	National Mineral Origin Database of China

704	75.324	47.317	Cu	Porphyry/Skarn Cu-Au deposit	Small	P	National Mineral Origin Database of China
705	74.164	45.389	Cu	Porphyry/Skarn Cu-Au deposit	Small	D3	National Mineral Origin Database of China
706	87.288	47.988	Cu	Other magmatic-hydrothermal Cu deposit	Medium	D1	Feng, J. et al., 2021. Geological Publishing House
707	74.447	49.212	Cu	Ni-Cu sulfide deposit	Small	C2	National Mineral Origin Database of China
708	78.475	44.497	Cu	Porphyry/Skarn Cu-Au deposit	Large (Super Large	P	National Mineral Origin Database of China
709	76.204	50.084	Cu	Porphyry/Skarn Cu-Au deposit	Medium	D3-C1	National Mineral Origin Database of China
710	75.674	50.152	Cu	Other magmatic-hydrothermal Cu deposit	Small	D3-C1	National Mineral Origin Database of China
711	74.992	46.988	Cu	Porphyry/Skarn Cu-Au deposit	Large (Super Large		National Mineral Origin Database of China
712	88.109	42.608	Cu	Other magmatic-hydrothermal Cu deposit	Small	O2	Feng, J. et al., 2021. Geological Publishing House
713	87.383	48.000	Cu	Other magmatic-hydrothermal Cu deposit	Small		Li,C.Y., et al., 2021,1995,2019.Development and Research Center, China Geological Survey.
714	84.475	43.458	Cu	Other magmatic-hydrothermal Cu deposit	Small	P1	Feng, J. et al., 2021. Geological Publishing House
715	83.375	44.175	Cu	Porphyry/Skarn Cu-Au deposit	Small	C1	Feng, J. et al., 2021. Geological Publishing House
716	75.297	47.261	Cu	Porphyry/Skarn Cu-Au deposit	Small	P	National Mineral Origin Database of China
717	85.174	47.315	Cu	Porphyry/Skarn Cu-Au deposit	Small	C1	National Mineral Origin Database of China
718	84.286	47.542	Cu	Porphyry/Skarn Cu-Au deposit	Small	C1	National Mineral Origin Database of China
719	74.948	43.337	Cu	Porphyry/Skarn Cu-Au deposit	Medium	C1	National Mineral Origin Database of China
720	74.374	48.336	Cu	Other magmatic-hydrothermal Cu deposit	Small	C2	National Mineral Origin Database of China
721	119.817	42.417	Cu	Porphyry/Skarn Cu-Au deposit	Small		Lv B. et al., 2017. Journal of Jilin University (Earth Science Edition) (In Chinese with English) Abstract)
722	72.666	41.511	Cu	Other magmatic-hydrothermal Cu deposit	Small		National Mineral Origin Database of China
723	81.578	43.403	Cu	Other magmatic-hydrothermal Cu deposit	Small	P1	Feng, J. et al., 2021. Geological Publishing House
724	77.798	48.581	Cu	Other magmatic-hydrothermal Cu deposit	Medium	O3	National Mineral Origin Database of China
725	74.149	40.446	Cu	Other magmatic-hydrothermal Cu deposit	Small		National Mineral Origin Database of China
726	80.973	44.673	Cu	Porphyry/Skarn Cu-Au deposit	Small	P1	Feng, J. et al., 2021. Geological Publishing House
727	124.584	43.267	Cu	Other magmatic-hydrothermal Cu deposit	Small	Mz	Li,C.Y., et al., 2021,1995,2019.Development and Research Center, China Geological Survey.
728	121.867	45.600	Cu	Porphyry/Skarn Cu-Au deposit	Medium		Lv B. et al., 2017. Journal of Jilin University (Earth Science Edition) (In Chinese with English) Abstract)
729	85.619	42.246	Cu	Other magmatic-hydrothermal Cu deposit	Small	D2	Feng, J. et al., 2021. Geological Publishing House
730	128.950	42.933	Cu	Other magmatic-hydrothermal Cu deposit	Large	Pz2-Mz	Li,C.Y., et al., 2021,1995,2019.Development and Research Center, China Geological Survey.
731	116.517	44.217	Cu	Other magmatic-hydrothermal Cu deposit	Medium		Lv B. et al., 2017. Journal of Jilin University (Earth Science Edition) (In Chinese with English) Abstract)
732	116.562	44.168	Cu	Other magmatic-hydrothermal Cu deposit	Medium	K	Li,C.Y., et al., 2021,1995,2019.Development and Research Center, China Geological Survey.
733	91.897	42.634	Cu	Other magmatic-hydrothermal Cu deposit	Small	O2	Feng, J. et al., 2021. Geological Publishing House
734	77.489	48.867	Cu	Other magmatic-hydrothermal Cu deposit	Medium	O3	National Mineral Origin Database of China
735	76.817	47.440	Cu	Ni-Cu sulfide deposit	Small		National Mineral Origin Database of China
736	95.302	44.202	Cu	Porphyry/Skarn Cu-Au deposit	Medium	P1	Feng, J. et al., 2021. Geological Publishing House
737	73.878	51.420	Cu	Porphyry/Skarn Cu-Au deposit	Small		National Mineral Origin Database of China
738	75.548	50.724	Cu	Porphyry/Skarn Cu-Au deposit	Small		National Mineral Origin Database of China
739	80.163	49.591	Cu	Ni-Cu sulfide deposit	Medium		National Mineral Origin Database of China
740	106.850	43.000	Cu	Porphyry/Skarn Cu-Au deposit	Large		Lv B. et al., 2017. Journal of Jilin University (Earth Science Edition) (In Chinese with English) Abstract)
741	76.277	48.343	Cu	Porphyry/Skarn Cu-Au deposit	Small	C2-P1	National Mineral Origin Database of China
742	120.279	42.309	Cu	Other magmatic-hydrothermal Cu deposit	Small	D-P	Li,C.Y., et al., 2021,1995,2019.Development and Research Center, China Geological Survey.
743	70.394	40.486	Cu	Other magmatic-hydrothermal Cu deposit	Small		National Mineral Origin Database of China
744	121.667	45.750	Cu	Porphyry/Skarn Cu-Au deposit	Small		Lv B. et al., 2017. Journal of Jilin University (Earth Science Edition) (In Chinese with English) Abstract)
745	125.650	50.417	Cu	Porphyry/Skarn Cu-Au deposit	Small	J	Li,C.Y., et al., 2021,1995,2019.Development and Research Center, China Geological Survey.
746	125.824	50.224	Cu	Other magmatic-hydrothermal Cu deposit	Large	J3-K1	Li,C.Y., et al., 2021,1995,2019.Development and Research Center, China Geological Survey.
747	81.879	50.549	Cu	Other magmatic-hydrothermal Cu deposit	Large	D2-3	National Mineral Origin Database of China
748	82.470	43.767	Cu	Other magmatic-hydrothermal Cu deposit	Small	P1	Feng, J. et al., 2021. Geological Publishing House
749	79.174	47.974	Cu	Porphyry/Skarn Cu-Au deposit	Small	C-P	National Mineral Origin Database of China
750	106.300	41.217	Cu	Porphyry/Skarn Cu-Au deposit	Large		Lv B. et al., 2017. Journal of Jilin University (Earth Science Edition) (In Chinese with English) Abstract)
751	126.403	42.903	Cu	Ni-Cu sulfide deposit	Small	Pz	Li,C.Y., et al., 2021,1995,2019.Development and Research Center, China Geological Survey.
752	126.443	42.881	Cu	Ni-Cu sulfide deposit	Small	Pz	Li,C.Y., et al., 2021,1995,2019.Development and Research Center, China Geological Survey.
753	72.357	52.571	Cu	Porphyry/Skarn Cu-Au deposit	Medium		National Mineral Origin Database of China
754	80.038	47.019	Cu	Porphyry/Skarn Cu-Au deposit	Small	P	National Mineral Origin Database of China
755	84.146	47.597	Cu	Porphyry/Skarn Cu-Au deposit	Small	C	National Mineral Origin Database of China
756	72.578	51.874	Cu	Porphyry/Skarn Cu-Au deposit	Small	O3	National Mineral Origin Database of China
757	88.250	47.878	Cu	Porphyry/Skarn Cu-Au deposit	Small	D1	Feng, J. et al., 2021. Geological Publishing House
758	74.145	41.245	Cu	Porphyry/Skarn Cu-Au deposit	Small	P2	National Mineral Origin Database of China
759	80.576	44.652	Cu	Other magmatic-hydrothermal Cu deposit	Medium	C3	Feng, J. et al., 2021. Geological Publishing House
760	73.046	49.598	Cu	Other magmatic-hydrothermal Cu deposit	Small	D	National Mineral Origin Database of China
761	81.288	43.317	Cu	Other magmatic-hydrothermal Cu deposit	Small		Li,C.Y., et al., 2021,1995,2019.Development and Research Center, China Geological Survey.
762	82.067	43.750	Cu	Other magmatic-hydrothermal Cu deposit	Small	P	Li,C.Y., et al., 2021,1995,2019.Development and Research Center, China Geological Survey.
763	95.213	44.168	Cu	Porphyry/Skarn Cu-Au deposit	Small	C1	Feng, J. et al., 2021. Geological Publishing House
764	73.669	47.381	Cu	Porphyry/Skarn Cu-Au deposit	Small	D3	National Mineral Origin Database of China
765	82.451	43.696	Cu	Porphyry/Skarn Cu-Au deposit	Small	P2	Feng, J. et al., 2021. Geological Publishing House
766	82.471	43.684	Cu	Other magmatic-hydrothermal Cu deposit	Small	P	Li,C.Y., et al., 2021,1995,2019.Development and Research Center, China Geological Survey.
767	83.619	49.663	Cu	Other magmatic-hydrothermal Cu deposit	Small	D3	National Mineral Origin Database of China

768	75.582	50.876	Cu	Porphyry/Skarn Cu-Au deposit	Small		National Mineral Origin Database of China
769	74.816	49.021	Cu	Porphyry/Skarn Cu-Au deposit	Small	P	National Mineral Origin Database of China
770	77.558	48.658	Cu	Other magmatic-hydrothermal Cu deposit	Small	D	National Mineral Origin Database of China
771	73.562	51.662	Cu	Porphyry/Skarn Cu-Au deposit	Small		National Mineral Origin Database of China
772	77.255	47.018	Cu	Porphyry/Skarn Cu-Au deposit	Small	P	National Mineral Origin Database of China
773	77.238	46.902	Cu	Porphyry/Skarn Cu-Au deposit	Small	P	National Mineral Origin Database of China
774	94.748	42.386	Cu	Porphyry/Skarn Cu-Au deposit	Small	P2	Feng, J. et al., 2021. Geological Publishing House
775	94.849	42.400	Cu	Ni-Cu sulfide deposit	Small	C	Li,C.Y., et al., 2021,1995,2019.Development and Research Center, China Geological Survey.
776	125.650	50.383	Cu	Other magmatic-hydrothermal Cu deposit	Large		Lv B. et al., 2017. Journal of Jilin University (Earth Science Edition) (In Chinese with English) Abstract)
777	95.303	44.188	Cu	Porphyry/Skarn Cu-Au deposit	Small	S3	Feng, J. et al., 2021. Geological Publishing House
778	82.325	44.234	Cu	Porphyry/Skarn Cu-Au deposit	Small	D1	Feng, J. et al., 2021. Geological Publishing House
779	73.515	46.001	Cu	Porphyry/Skarn Cu-Au deposit	Medium	D3	National Mineral Origin Database of China
780	76.418	48.465	Cu	Porphyry/Skarn Cu-Au deposit	Medium	P	National Mineral Origin Database of China
781	84.917	43.350	Cu	Other magmatic-hydrothermal Cu deposit	Small	C1	Feng, J. et al., 2021. Geological Publishing House
782	72.702	52.691	Cu	Porphyry/Skarn Cu-Au deposit	Medium		National Mineral Origin Database of China
783	95.883	42.097	Cu	Other magmatic-hydrothermal Cu deposit	Small	C3	Feng, J. et al., 2021. Geological Publishing House
784	91.867	41.767	Cu	Porphyry/Skarn Cu-Au deposit	Small	C1	Feng, J. et al., 2021. Geological Publishing House
785	125.650	43.800	Cu	Porphyry/Skarn Cu-Au deposit	Small	Pz	Li,C.Y., et al., 2021,1995,2019.Development and Research Center, China Geological Survey.
786	80.200	42.600	Cu	Other magmatic-hydrothermal Cu deposit	Small	C3	Feng, J. et al., 2021. Geological Publishing House
787	73.353	49.539	Cu	Other magmatic-hydrothermal Cu deposit	Small	P	National Mineral Origin Database of China
788	83.659	50.533	Cu	Other magmatic-hydrothermal Cu deposit	Small	D2-3	National Mineral Origin Database of China
789	127.450	45.550	Cu	Other magmatic-hydrothermal Cu deposit	Small		Lv B. et al., 2017. Journal of Jilin University (Earth Science Edition) (In Chinese with English) Abstract)
790	81.736	50.662	Cu	Other magmatic-hydrothermal Cu deposit	Small	D2	National Mineral Origin Database of China
791	95.999	41.384	Cu	Other magmatic-hydrothermal Cu deposit	Small	Qn	Li,C.Y., et al., 2021,1995,2019.Development and Research Center, China Geological Survey.
792	97.063	41.688	Cu	Other magmatic-hydrothermal Cu deposit	Medium	S2-3	Li,C.Y., et al., 2021,1995,2019.Development and Research Center, China Geological Survey.
793	88.757	46.761	Cu	Porphyry/Skarn Cu-Au deposit	Medium	P1	Feng, J. et al., 2021. Geological Publishing House
794	88.754	46.751	Cu	Other magmatic-hydrothermal Cu deposit	Medium	C	Li,C.Y., et al., 2021,1995,2019.Development and Research Center, China Geological Survey.
795	124.906	52.866	Cu	Other magmatic-hydrothermal Cu deposit	Small	K	Li,C.Y., et al., 2021,1995,2019.Development and Research Center, China Geological Survey.
796	118.933	43.000	Cu	Porphyry/Skarn Cu-Au deposit	Small		Lv B. et al., 2017. Journal of Jilin University (Earth Science Edition) (In Chinese with English) Abstract)
797	120.400	51.500	Cu	Porphyry/Skarn Cu-Au deposit	Medium		Lv B. et al., 2017. Journal of Jilin University (Earth Science Edition) (In Chinese with English) Abstract)
798	70.006	41.417	Cu	Other magmatic-hydrothermal Cu deposit	Small		National Mineral Origin Database of China
799	76.438	47.035	Cu	Ni-Cu sulfide deposit	Small	P	National Mineral Origin Database of China
800	88.041	42.147	Cu	Other magmatic-hydrothermal Cu deposit	Small	D1	Feng, J. et al., 2021. Geological Publishing House
801	95.305	42.813	Cu	Porphyry/Skarn Cu-Au deposit	Small	C1	Feng, J. et al., 2021. Geological Publishing House
802	94.154	42.216	Cu	Ni-Cu sulfide deposit	Medium	C-P	Li,C.Y., et al., 2021,1995,2019.Development and Research Center, China Geological Survey.
803	92.606	42.115	Cu	Porphyry/Skarn Cu-Au deposit	Large	C3	Feng, J. et al., 2021. Geological Publishing House
804	92.613	42.117	Cu	Porphyry/Skarn Cu-Au deposit	Medium	C3	Feng, J. et al., 2021. Geological Publishing House
805	84.473	45.674	Cu	Porphyry/Skarn Cu-Au deposit	Small	C3	Feng, J. et al., 2021. Geological Publishing House
806	73.752	48.246	Cu	Other magmatic-hydrothermal Cu deposit	Small	P	National Mineral Origin Database of China
807	129.556	43.553	Cu	Other magmatic-hydrothermal Cu deposit	Small	Pz2	Li,C.Y., et al., 2021,1995,2019.Development and Research Center, China Geological Survey.
808	88.907	42.158	Cu	Other magmatic-hydrothermal Cu deposit	Small	D1	Feng, J. et al., 2021. Geological Publishing House
809	88.950	42.150	Cu	Other magmatic-hydrothermal Cu deposit	Small	C1	Feng, J. et al., 2021. Geological Publishing House
810	117.483	44.067	Cu	Other magmatic-hydrothermal Cu deposit	Large		Lv B. et al., 2017. Journal of Jilin University (Earth Science Edition) (In Chinese with English) Abstract)
811	91.717	41.883	Cu	Other magmatic-hydrothermal Cu deposit	Small	C1	Feng, J. et al., 2021. Geological Publishing House
812	74.391	45.353	Cu	Porphyry/Skarn Cu-Au deposit	Small	D3	National Mineral Origin Database of China
813	119.806	42.388	Cu	Other magmatic-hydrothermal Cu deposit	Small	J2-K	Li,C.Y., et al., 2021,1995,2019.Development and Research Center, China Geological Survey.
814	99.217	42.150	Cu	Porphyry/Skarn Cu-Au deposit	Small		Lv B. et al., 2017. Journal of Jilin University (Earth Science Edition) (In Chinese with English) Abstract)
815	75.084	42.391	Cu	Other magmatic-hydrothermal Cu deposit	Small		National Mineral Origin Database of China
816	117.283	49.417	Cu	Porphyry/Skarn Cu-Au deposit	Large		Lv B. et al., 2017. Journal of Jilin University (Earth Science Edition) (In Chinese with English) Abstract)
817	83.693	50.410	Cu	Other magmatic-hydrothermal Cu deposit	Small	D3	National Mineral Origin Database of China
818	75.853	48.805	Cu	Porphyry/Skarn Cu-Au deposit	Small	D3	National Mineral Origin Database of China
819	70.985	41.533	Cu	Porphyry/Skarn Cu-Au deposit	Small	P2	National Mineral Origin Database of China
820	118.833	42.750	Cu	Other magmatic-hydrothermal Cu deposit	Small	J2-K	Li,C.Y., et al., 2021,1995,2019.Development and Research Center, China Geological Survey.
821	84.943	43.149	Cu	Other magmatic-hydrothermal Cu deposit	Small	C1	Feng, J. et al., 2021. Geological Publishing House
822	106.833	43.000	Cu	Porphyry/Skarn Cu-Au deposit	Large		Lv B. et al., 2017. Journal of Jilin University (Earth Science Edition) (In Chinese with English) Abstract)
823	73.709	44.372	Cu	Ni-Cu sulfide deposit	Small	C1	National Mineral Origin Database of China
824	116.733	45.100	Cu	Other magmatic-hydrothermal Cu deposit	Medium		Lv B. et al., 2017. Journal of Jilin University (Earth Science Edition) (In Chinese with English) Abstract)
825	125.733	50.283	Cu	Other magmatic-hydrothermal Cu deposit	Small		Lv B. et al., 2017. Journal of Jilin University (Earth Science Edition) (In Chinese with English) Abstract)
826	89.534	42.282	Cu	Other magmatic-hydrothermal Cu deposit	Medium	C1	Feng, J. et al., 2021. Geological Publishing House
827	89.542	42.287	Cu	Other magmatic-hydrothermal Cu deposit	Small	C-P	Li,C.Y., et al., 2021,1995,2019.Development and Research Center, China Geological Survey.
828	130.867	43.200	Cu-Au	Porphyry/Skarn Cu-Au deposit	Large		Lv B. et al., 2017. Journal of Jilin University (Earth Science Edition) (In Chinese with English) Abstract)
829	81.870	50.700	Cu	Other magmatic-hydrothermal Cu deposit	Small	D2-3	National Mineral Origin Database of China
830	92.617	42.117	Cu	Porphyry/Skarn Cu-Au deposit	Large	C-P	Li,C.Y., et al., 2021,1995,2019.Development and Research Center, China Geological Survey.
831	92.533	42.117	Cu	Porphyry/Skarn Cu-Au deposit	Large	C-P	Li,C.Y., et al., 2021,1995,2019.Development and Research Center, China Geological Survey.

832	124.150	51.400	Cu	Other magmatic-hydrothermal Cu deposit	Small	J3	Li,C.Y., et al., 2021,1995,2019.Development and Research Center, China Geological Survey.
833	118.867	43.850	Cu	Other magmatic-hydrothermal Cu deposit	Small	J2-K	Li,C.Y., et al., 2021,1995,2019.Development and Research Center, China Geological Survey.
834	74.003	46.421	Cu	Other magmatic-hydrothermal Cu deposit	Small	P	National Mineral Origin Database of China
835	70.476	40.502	Cu	Other magmatic-hydrothermal Cu deposit	Small		National Mineral Origin Database of China
836	128.908	49.040	Cu	Porphyry/Skarn Cu-Au deposit	Small	C	Li,C.Y., et al., 2021,1995,2019.Development and Research Center, China Geological Survey.
837	74.217	48.524	Cu	Porphyry/Skarn Cu-Au deposit	Small	O2	National Mineral Origin Database of China
838	92.524	42.090	Cu	Porphyry/Skarn Cu-Au deposit	Large	C3	Feng, J. et al., 2021. Geological Publishing House
839	92.958	40.706	Cu	Other magmatic-hydrothermal Cu deposit	Small	P2	Feng, J. et al., 2021. Geological Publishing House
840	131.033	44.600	Cu	Porphyry/Skarn Cu-Au deposit	Small		Lv B. et al., 2017. Journal of Jilin University (Earth Science Edition) (In Chinese with English) Abstract)
841	72.383	51.844	Cu	Porphyry/Skarn Cu-Au deposit	Small	O3	National Mineral Origin Database of China
842	74.282	45.488	Cu	Porphyry/Skarn Cu-Au deposit	Small		National Mineral Origin Database of China
843	125.079	43.469	Cu	Other magmatic-hydrothermal Cu deposit	Small	Pz1	Li,C.Y., et al., 2021,1995,2019.Development and Research Center, China Geological Survey.
844	125.271	43.621	Cu	Other magmatic-hydrothermal Cu deposit	Small	Pz2	Li,C.Y., et al., 2021,1995,2019.Development and Research Center, China Geological Survey.
845	126.478	43.356	Cu	Other magmatic-hydrothermal Cu deposit	Small	Mz	Li,C.Y., et al., 2021,1995,2019.Development and Research Center, China Geological Survey.
846	81.700	44.333	Cu	Porphyry/Skarn Cu-Au deposit	Small	C1	Feng, J. et al., 2021. Geological Publishing House
847	106.850	43.033	Cu	Porphyry/Skarn Cu-Au deposit	Large		Lv B. et al., 2017. Journal of Jilin University (Earth Science Edition) (In Chinese with English) Abstract)
848	90.022	46.603	Cu	Porphyry/Skarn Cu-Au deposit	Medium	P2	Feng, J. et al., 2021. Geological Publishing House
849	84.417	43.483	Cu	Other magmatic-hydrothermal Cu deposit	Small	P1	Feng, J. et al., 2021. Geological Publishing House
850	91.150	42.008	Cu	Other magmatic-hydrothermal Cu deposit	Small	C3	Feng, J. et al., 2021. Geological Publishing House
851	125.683	50.250	Cu	Porphyry/Skarn Cu-Au deposit	Small		Lv B. et al., 2017. Journal of Jilin University (Earth Science Edition) (In Chinese with English) Abstract)
852	74.089	47.833	Cu	Porphyry/Skarn Cu-Au deposit	Small	D3	National Mineral Origin Database of China
853	73.964	43.711	Cu	Porphyry/Skarn Cu-Au deposit	Medium		National Mineral Origin Database of China
854	68.093	53.109	Cu	Ni-Cu sulfide deposit	Small	C	National Mineral Origin Database of China
855	126.950	44.000	Cu	Porphyry/Skarn Cu-Au deposit	Small		Lv B. et al., 2017. Journal of Jilin University (Earth Science Edition) (In Chinese with English) Abstract)
856	92.266	41.883	Cu	Other magmatic-hydrothermal Cu deposit	Small	C3	Feng, J. et al., 2021. Geological Publishing House
857	112.957	42.241	Cu	Other magmatic-hydrothermal Cu deposit	Medium	P	Li,C.Y., et al., 2021,1995,2019.Development and Research Center, China Geological Survey.
858	74.410	43.529	Cu	Porphyry/Skarn Cu-Au deposit	Medium	D3	National Mineral Origin Database of China
859	76.228	50.623	Cu	Other magmatic-hydrothermal Cu deposit	Small	O2-3	National Mineral Origin Database of China
860	104.213	48.979	Cu,Mo	Porphyry/Skarn Cu-Au deposit	Medium	T3	Ochir Gerel et al., 2021 Mineral Resources of Mongolia (book) and refs therein
861	104.133	49.017	Cu,Mo	Porphyry/Skarn Cu-Au deposit	Large	T3	Ochir Gerel et al., 2021 Mineral Resources of Mongolia (book) and refs therein
862	104.217	48.775	Cu,Mo	Porphyry/Skarn Cu-Au deposit	Medium	T3	Ochir Gerel et al., 2021 Mineral Resources of Mongolia (book) and refs therein
863	90.367	48.300	Cu,Pb,Zn	Porphyry/Skarn Cu-Au deposit	Medium	NP3-KE1	Ochir Gerel et al., 2021 Mineral Resources of Mongolia (book) and refs therein
864	96.580	53.430	Cu-Au	Porphyry/Skarn Cu-Au deposit		∈	Wu, C. et al., 2022. Mineralium Deposita
865	112.550	42.720	Cu-Au	Porphyry/Skarn Cu-Au deposit		O	Wu, C. et al., 2022. Mineralium Deposita
866	84.460	45.500	Cu-Au	Porphyry/Skarn Cu-Au deposit		C	Wu, C. et al., 2022. Mineralium Deposita
867	61.910	52.800	Cu-Au	Porphyry/Skarn Cu-Au deposit		C	Wu, C. et al., 2022. Mineralium Deposita
868	61.770	51.220	Cu-Au	Porphyry/Skarn Cu-Au deposit		C	Wu, C. et al., 2022. Mineralium Deposita
869	74.320	51.860	Cu-Au	Porphyry/Skarn Cu-Au deposit		O	Wu, C. et al., 2022. Mineralium Deposita
870	69.630	40.810	Cu-Au	Porphyry/Skarn Cu-Au deposit		C	Wu, C. et al., 2022. Mineralium Deposita
871	69.640	40.810	Cu-Au	Porphyry/Skarn Cu-Au deposit		C	Wu, C. et al., 2022. Mineralium Deposita
872	74.280	45.480	Cu-Au	Porphyry/Skarn Cu-Au deposit		C	Wu, C. et al., 2022. Mineralium Deposita
873	78.510	44.460	Cu-Au	Porphyry/Skarn Cu-Au deposit		C	Wu, C. et al., 2022. Mineralium Deposita
874	76.160	50.100	Cu-Au	Porphyry/Skarn Cu-Au deposit		P	Wu, C. et al., 2022. Mineralium Deposita
875	74.990	46.980	Cu-Au	Porphyry/Skarn Cu-Au deposit		C	Wu, C. et al., 2022. Mineralium Deposita
876	69.730	40.750	Cu-Au	Porphyry/Skarn Cu-Au deposit		C	Wu, C. et al., 2022. Mineralium Deposita
877	73.250	50.170	Cu-Au	Porphyry/Skarn Cu-Au deposit		D	Wu, C. et al., 2022. Mineralium Deposita
878	106.840	43.000	Cu-Au	Porphyry/Skarn Cu-Au deposit		D	Wu, C. et al., 2022. Mineralium Deposita
879	69.780	40.780	Cu-Au	Porphyry/Skarn Cu-Au deposit		C	Wu, C. et al., 2022. Mineralium Deposita
880	77.400	47.000	Cu-Au	Porphyry/Skarn Cu-Au deposit		C	Wu, C. et al., 2022. Mineralium Deposita
881	72.770	42.540	Cu-Au	Porphyry/Skarn Cu-Au deposit		O	Wu, C. et al., 2022. Mineralium Deposita
882	108.330	43.870	Cu-Au	Porphyry/Skarn Cu-Au deposit		D	Wu, C. et al., 2022. Mineralium Deposita
883	92.630	42.120	Cu-Au	Porphyry/Skarn Cu-Au deposit		C	Wu, C. et al., 2022. Mineralium Deposita
884	74.000	48.670	Cu-Mo	Porphyry/Skarn Cu-Au deposit		C	Wu, C. et al., 2022. Mineralium Deposita
885	119.130	49.980	Cu-Mo	Porphyry/Skarn Cu-Au deposit		T	Wu, C. et al., 2022. Mineralium Deposita
886	104.070	48.950	Cu-Mo	Porphyry/Skarn Cu-Au deposit		T	Wu, C. et al., 2022. Mineralium Deposita
887	122.000	52.670	Cu-Mo	Porphyry/Skarn Cu-Au deposit		J	Wu, C. et al., 2022. Mineralium Deposita
888	117.280	49.400	Cu-Mo	Porphyry/Skarn Cu-Au deposit		J	Wu, C. et al., 2022. Mineralium Deposita
889	124.750	51.670	Cu-Mo	Porphyry/Skarn Cu-Au deposit		J	Wu, C. et al., 2022. Mineralium Deposita
890	79.930	46.990	Cu-Mo-Au	Porphyry/Skarn Cu-Au deposit		C	Wu, C. et al., 2022. Mineralium Deposita
891	79.980	46.970	Cu-Mo-Au	Porphyry/Skarn Cu-Au deposit		C	Wu, C. et al., 2022. Mineralium Deposita
892	125.780	50.250	Cu-Mo-Au	Porphyry/Skarn Cu-Au deposit		O	Wu, C. et al., 2022. Mineralium Deposita
893	106.140	44.030	Cu-Mo-Au	Porphyry/Skarn Cu-Au deposit		P	Wu, C. et al., 2022. Mineralium Deposita
894	89.044	47.488	Fe	Fe deposit	Small	D1	Feng, J. et al., 2021. Geological Publishing House
895	89.061	47.505	Fe	Fe deposit	Small	D1	Feng, J. et al., 2021. Geological Publishing House

896	89.052	47.486	Fe	Fe deposit	Small	D1	Feng, J. et al., 2021. Geological Publishing House
897	89.079	47.481	Fe	Fe deposit	Small	D1	Feng, J. et al., 2021. Geological Publishing House
898	89.084	47.473	Fe	Fe deposit	Small	D1	Feng, J. et al., 2021. Geological Publishing House
899	89.113	47.458	Fe	Fe deposit	Small	D1	Feng, J. et al., 2021. Geological Publishing House
900	89.151	47.449	Fe	Fe deposit	Small	D1	Feng, J. et al., 2021. Geological Publishing House
901	91.550	41.804	Fe	Fe deposit	Small	P1	Feng, J. et al., 2021. Geological Publishing House
902	91.860	41.796	Fe	Fe deposit	Small	P1	Feng, J. et al., 2021. Geological Publishing House
903	91.856	41.772	Fe	Fe deposit	Small	P1	Feng, J. et al., 2021. Geological Publishing House
904	91.081	41.807	Fe	Fe deposit	Small	P1	Feng, J. et al., 2021. Geological Publishing House
905	91.645	41.786	Fe	Fe deposit	Small	C3	Feng, J. et al., 2021. Geological Publishing House
906	91.612	41.750	Fe	Fe deposit	Small	C3	Feng, J. et al., 2021. Geological Publishing House
907	94.651	41.844	Fe	Fe deposit	Small	C2	Li,C.Y., et al., 2021,1995,2019.Development and Research Center, China Geological Survey.
908	95.166	43.988	Fe	Fe deposit	Small	D1	Feng, J. et al., 2021. Geological Publishing House
909	98.379	42.302	Fe	Fe deposit	Medium		National Mineral Origin Database of China
910	99.283	46.183	Fe	Fe deposit	Occurrence	PP1	Ochir Gerel et al., 2021 Mineral Resources of Mongolia (book) and refs therein
911	109.272	46.322	Fe	Fe deposit	Small	MZ2	Ochir Gerel et al., 2021 Mineral Resources of Mongolia (book) and refs therein
912	107.017	49.634	Fe	Fe deposit	Medium	NP3	Ochir Gerel et al., 2021 Mineral Resources of Mongolia (book) and refs therein
913	98.139	42.415	Fe	Fe deposit	Small		National Mineral Origin Database of China
914	106.375	48.008	Fe	Fe deposit	Occurrence	S2	Ochir Gerel et al., 2021 Mineral Resources of Mongolia (book) and refs therein
915	98.762	46.090	Fe	Fe deposit	Occurrence	PZ3	Ochir Gerel et al., 2021 Mineral Resources of Mongolia (book) and refs therein
916	111.086	45.708	Fe	Fe deposit	Small	MZ1	Ochir Gerel et al., 2021 Mineral Resources of Mongolia (book) and refs therein
917	108.850	45.918	Fe	Fe deposit	Small	NP	Ochir Gerel et al., 2021 Mineral Resources of Mongolia (book) and refs therein
918	109.308	46.208	Fe	Fe deposit	Small	MZ1	Ochir Gerel et al., 2021 Mineral Resources of Mongolia (book) and refs therein
919	108.217	45.867	Fe	Fe deposit	Medium	NP	Ochir Gerel et al., 2021 Mineral Resources of Mongolia (book) and refs therein
920	92.183	49.617	Fe	Fe deposit	Small	D1	Ochir Gerel et al., 2021 Mineral Resources of Mongolia (book) and refs therein
921	105.883	47.392	Fe	Fe deposit	Occurrence	S2	Ochir Gerel et al., 2021 Mineral Resources of Mongolia (book) and refs therein
922	102.160	47.625	Fe	Fe deposit	Small	S2	Ochir Gerel et al., 2021 Mineral Resources of Mongolia (book) and refs therein
923	97.823	42.439	Fe	Fe deposit	Small		National Mineral Origin Database of China
924	94.117	49.417	Fe	Fe deposit	Small	PP4	Ochir Gerel et al., 2021 Mineral Resources of Mongolia (book) and refs therein
925	107.250	49.683	Fe	Fe deposit	Medium	NP3	Ochir Gerel et al., 2021 Mineral Resources of Mongolia (book) and refs therein
926	106.167	49.300	Fe	Fe deposit	Small	NP3	Ochir Gerel et al., 2021 Mineral Resources of Mongolia (book) and refs therein
927	104.338	48.310	Fe	Fe deposit	Small	NP3	Ochir Gerel et al., 2021 Mineral Resources of Mongolia (book) and refs therein
928	88.439	47.737	Fe	Fe deposit	Small	D1	Feng, J. et al., 2021. Geological Publishing House
929	127.250	45.300	Fe	Fe deposit	Medium	T3	Li,C.Y., et al., 2021,1995,2019.Development and Research Center, China Geological Survey.
930	127.019	45.342	Fe	Fe deposit	Small	T3	Li,C.Y., et al., 2021,1995,2019.Development and Research Center, China Geological Survey.
931	80.808	42.537	Fe	Fe deposit	Small	C1	Feng, J. et al., 2021. Geological Publishing House
932	75.276	41.938	Fe	Fe deposit	Small		National Mineral Origin Database of China
933	92.778	41.506	Fe	Fe deposit	Medium	MP	Feng, J. et al., 2021. Geological Publishing House
934	93.877	41.513	Fe	Fe deposit	Small	C	Li,C.Y., et al., 2021,1995,2019.Development and Research Center, China Geological Survey.
935	88.325	47.729	Fe	Fe deposit	Small	D2	Feng, J. et al., 2021. Geological Publishing House
936	90.821	41.969	Fe	Fe deposit	Small	C	Li,C.Y., et al., 2021,1995,2019.Development and Research Center, China Geological Survey.
937	90.783	41.942	Fe	Fe deposit	Small	C	Li,C.Y., et al., 2021,1995,2019.Development and Research Center, China Geological Survey.
938	91.542	41.774	Fe	Fe deposit	Medium	C3	Feng, J. et al., 2021. Geological Publishing House
939	91.325	41.815	Fe	Fe deposit	Small	C	Li,C.Y., et al., 2021,1995,2019.Development and Research Center, China Geological Survey.
940	91.583	41.742	Fe	Fe deposit	Small	C	Li,C.Y., et al., 2021,1995,2019.Development and Research Center, China Geological Survey.
941	91.083	41.850	Fe	Fe deposit	Small	C	Li,C.Y., et al., 2021,1995,2019.Development and Research Center, China Geological Survey.
942	91.859	41.791	Fe	Fe deposit	Small		Li,C.Y., et al., 2021,1995,2019.Development and Research Center, China Geological Survey.
943	91.012	41.792	Fe	Fe deposit	Small	C	Li,C.Y., et al., 2021,1995,2019.Development and Research Center, China Geological Survey.
944	80.725	42.522	Fe	Fe deposit	Small	C1	Feng, J. et al., 2021. Geological Publishing House
945	84.260	42.920	Fe	Fe deposit	Small	C	Li,C.Y., et al., 2021,1995,2019.Development and Research Center, China Geological Survey.
946	95.299	41.311	Fe	Fe deposit	Small	S-D	Li,C.Y., et al., 2021,1995,2019.Development and Research Center, China Geological Survey.
947	95.139	40.982	Fe	Fe deposit	Small		Li,C.Y., et al., 2021,1995,2019.Development and Research Center, China Geological Survey.
948	74.593	42.043	Fe	Fe deposit	Small		National Mineral Origin Database of China
949	122.327	46.992	Fe	Fe deposit	Small	D-P	Li,C.Y., et al., 2021,1995,2019.Development and Research Center, China Geological Survey.
950	70.091	42.019	Fe	Fe deposit	Small		National Mineral Origin Database of China
951	121.600	46.025	Fe	Fe deposit	Small	J2-K	Li,C.Y., et al., 2021,1995,2019.Development and Research Center, China Geological Survey.
952	72.617	42.474	Fe	Fe deposit	Large	O1-2	National Mineral Origin Database of China
953	120.813	48.361	Fe	Fe deposit	Small	C-P	Li,C.Y., et al., 2021,1995,2019.Development and Research Center, China Geological Survey.
954	110.050	41.917	Fe	Fe deposit	Small	D-P	Li,C.Y., et al., 2021,1995,2019.Development and Research Center, China Geological Survey.
955	99.938	41.157	Fe	Fe deposit	Small	P	Li,C.Y., et al., 2021,1995,2019.Development and Research Center, China Geological Survey.
956	108.150	42.417	Fe	Fe deposit	Small	P	Li,C.Y., et al., 2021,1995,2019.Development and Research Center, China Geological Survey.
957	83.194	46.054	Fe	Fe deposit	Small	D3	Feng, J. et al., 2021. Geological Publishing House
958	96.075	42.179	Fe	Fe deposit	Medium	C1	Feng, J. et al., 2021. Geological Publishing House
959	96.061	42.069	Fe	Fe deposit	Medium	C1	Li,C.Y., et al., 2021,1995,2019.Development and Research Center, China Geological Survey.

960	95.592	41.864	Fe	Fe deposit	Small	C1	Li,C.Y., et al., 2021,1995,2019.Development and Research Center, China Geological Survey.
961	121.600	42.583	Fe	Fe deposit	Small	J2-K	Li,C.Y., et al., 2021,1995,2019.Development and Research Center, China Geological Survey.
962	121.650	42.517	Fe	Fe deposit	Small	D-P	Li,C.Y., et al., 2021,1995,2019.Development and Research Center, China Geological Survey.
963	91.321	41.826	Fe	Fe deposit	Medium	C3	Feng, J. et al., 2021. Geological Publishing House
964	91.383	41.783	Fe	Fe deposit	Small	C	Li,C.Y., et al., 2021,1995,2019.Development and Research Center, China Geological Survey.
965	76.207	41.753	Fe	Fe deposit	Medium	Pz1	National Mineral Origin Database of China
966	76.090	42.213	Fe	Fe deposit	Small	S	National Mineral Origin Database of China
967	76.030	42.248	Fe	Fe deposit	Small	S	National Mineral Origin Database of China
968	113.383	43.400	Fe	Fe deposit	Medium	T	Li,C.Y., et al., 2021,1995,2019.Development and Research Center, China Geological Survey.
969	95.080	44.041	Fe	Fe deposit	Small	D1	Feng, J. et al., 2021. Geological Publishing House
970	85.557	43.250	Fe	Fe deposit	Medium	C1	Feng, J. et al., 2021. Geological Publishing House
971	112.880	42.567	Fe	Fe deposit	Medium	Pt3	Li,C.Y., et al., 2021,1995,2019.Development and Research Center, China Geological Survey.
972	87.676	41.575	Fe	Fe deposit	Small	Pt	Li,C.Y., et al., 2021,1995,2019.Development and Research Center, China Geological Survey.
973	91.203	41.739	Fe	Fe deposit	Medium	C3	Feng, J. et al., 2021. Geological Publishing House
974	84.903	43.348	Fe	Fe deposit	Large	C1	Feng, J. et al., 2021. Geological Publishing House
975	84.911	43.358	Fe	Fe deposit	Medium	C1	Li,C.Y., et al., 2021,1995,2019.Development and Research Center, China Geological Survey.
976	86.950	43.642	Fe	Fe deposit	Small	J	Li,C.Y., et al., 2021,1995,2019.Development and Research Center, China Geological Survey.
977	91.675	41.717	Fe	Fe deposit	Medium	C	Li,C.Y., et al., 2021,1995,2019.Development and Research Center, China Geological Survey.
978	93.328	41.135	Fe	Fe deposit	Large	P1	Feng, J. et al., 2021. Geological Publishing House
979	93.313	41.111	Fe	Fe deposit	Small	P	Li,C.Y., et al., 2021,1995,2019.Development and Research Center, China Geological Survey.
980	128.733	48.483	Fe	Fe deposit	Large		Lv B. et al., 2017. Journal of Jilin University (Earth Science Edition) (In Chinese with English) Abstract)
981	94.109	42.016	Fe	Fe deposit	Small	C	Li,C.Y., et al., 2021,1995,2019.Development and Research Center, China Geological Survey.
982	84.971	43.971	Fe	Fe deposit	Small	P1	Feng, J. et al., 2021. Geological Publishing House
983	74.354	39.917	Fe	Fe deposit	Small	C3	Feng, J. et al., 2021. Geological Publishing House
984	88.092	44.083	Fe	Fe deposit	Small	J	Li,C.Y., et al., 2021,1995,2019.Development and Research Center, China Geological Survey.
985	117.883	43.733	Fe	Fe deposit	Small	J2-K	Li,C.Y., et al., 2021,1995,2019.Development and Research Center, China Geological Survey.
986	91.591	41.764	Fe	Fe deposit	Medium	C3	Feng, J. et al., 2021. Geological Publishing House
987	91.492	41.775	Fe	Fe deposit	Small	C3	Feng, J. et al., 2021. Geological Publishing House
988	101.604	39.574	Fe	Fe deposit	Medium	∈	Li,C.Y., et al., 2021,1995,2019.Development and Research Center, China Geological Survey.
989	118.310	46.000	Fe	Fe deposit	Small	P	Li,C.Y., et al., 2021,1995,2019.Development and Research Center, China Geological Survey.
990	120.117	49.250	Fe	Fe deposit	Medium	C3	Li,C.Y., et al., 2021,1995,2019.Development and Research Center, China Geological Survey.
991	128.358	47.158	Fe	Fe deposit	Small	S2	Li,C.Y., et al., 2021,1995,2019.Development and Research Center, China Geological Survey.
992	128.338	47.169	Fe	Fe deposit	Medium	P-T	Li,C.Y., et al., 2021,1995,2019.Development and Research Center, China Geological Survey.
993	75.931	42.234	Fe	Fe deposit	Small		National Mineral Origin Database of China
994	86.117	42.667	Fe	Fe deposit	Small	C	Li,C.Y., et al., 2021,1995,2019.Development and Research Center, China Geological Survey.
995	86.167	42.808	Fe	Fe deposit	Small	D2	Feng, J. et al., 2021. Geological Publishing House
996	83.042	44.075	Fe	Fe deposit	Medium	C3	Feng, J. et al., 2021. Geological Publishing House
997	86.058	42.979	Fe	Fe deposit	Small	P1	Feng, J. et al., 2021. Geological Publishing House
998	129.500	45.232	Fe	Fe deposit	Small	Pt3-2	Li,C.Y., et al., 2021,1995,2019.Development and Research Center, China Geological Survey.
999	116.183	42.194	Fe	Fe deposit	Small	J2	Li,C.Y., et al., 2021,1995,2019.Development and Research Center, China Geological Survey.
1000	86.033	46.571	Fe	Fe deposit	Small	C1	Feng, J. et al., 2021. Geological Publishing House
1001	83.489	43.569	Fe	Fe deposit	Small	C3	Feng, J. et al., 2021. Geological Publishing House
1002	90.955	41.775	Fe	Fe deposit	Small	C3	Feng, J. et al., 2021. Geological Publishing House
1003	94.583	41.867	Fe	Fe deposit	Small	C3	Feng, J. et al., 2021. Geological Publishing House
1004	89.083	43.500	Fe	Fe deposit	Small	C3	Feng, J. et al., 2021. Geological Publishing House
1005	91.556	41.793	Fe	Fe deposit	Medium	C1（C3）	Feng, J. et al., 2021. Geological Publishing House
1006	91.596	41.775	Fe	Fe deposit	Small	C3	Feng, J. et al., 2021. Geological Publishing House
1007	92.495	41.986	Fe	Fe deposit	Small	C1	Feng, J. et al., 2021. Geological Publishing House
1008	98.367	42.300	Fe	Fe deposit	Medium		Lv B. et al., 2017. Journal of Jilin University (Earth Science Edition) (In Chinese with English) Abstract)
1009	94.047	44.510	Fe	Fe deposit	Small	C1	Feng, J. et al., 2021. Geological Publishing House
1010	74.350	39.904	Fe	Fe deposit	Small		Li,C.Y., et al., 2021,1995,2019.Development and Research Center, China Geological Survey.
1011	90.586	41.785	Fe	Fe deposit	Small	C1	Feng, J. et al., 2021. Geological Publishing House
1012	90.599	41.786	Fe	Fe deposit	Medium	C	Li,C.Y., et al., 2021,1995,2019.Development and Research Center, China Geological Survey.
1013	125.745	51.799	Fe	Fe deposit	Medium	Z1	Li,C.Y., et al., 2021,1995,2019.Development and Research Center, China Geological Survey.
1014	126.796	43.450	Fe	Fe deposit	Small	Mz	Li,C.Y., et al., 2021,1995,2019.Development and Research Center, China Geological Survey.
1015	117.367	43.567	Fe	Fe deposit	Large		Lv B. et al., 2017. Journal of Jilin University (Earth Science Edition) (In Chinese with English) Abstract)
1016	130.733	42.975	Fe	Fe deposit	Small	Pz	Li,C.Y., et al., 2021,1995,2019.Development and Research Center, China Geological Survey.
1017	95.602	41.863	Fe	Fe deposit	Small	C3	Feng, J. et al., 2021. Geological Publishing House
1018	131.333	45.125	Fe	Fe deposit	Medium	Z3	Li,C.Y., et al., 2021,1995,2019.Development and Research Center, China Geological Survey.
1019	131.000	44.883	Fe	Fe deposit	Small	γ5	Li,C.Y., et al., 2021,1995,2019.Development and Research Center, China Geological Survey.
1020	131.050	45.406	Fe	Fe deposit	Medium	S1	Li,C.Y., et al., 2021,1995,2019.Development and Research Center, China Geological Survey.
1021	81.923	47.609	Fe	Fe deposit	Small		National Mineral Origin Database of China
1022	122.872	50.494	Fe	Fe deposit	Small	J2	Li,C.Y., et al., 2021,1995,2019.Development and Research Center, China Geological Survey.

1023	90.117	41.815	Fe	Fe deposit	Small	C	Li,C.Y., et al., 2021,1995,2019.Development and Research Center, China Geological Survey.
1024	89.973	41.827	Fe	Fe deposit	Medium	C	Li,C.Y., et al., 2021,1995,2019.Development and Research Center, China Geological Survey.
1025	88.667	47.583	Fe	Fe deposit	Small	D1	Feng, J. et al., 2021. Geological Publishing House
1026	69.603	41.636	Fe	Fe deposit	Small		National Mineral Origin Database of China
1027	99.333	40.459	Fe	Fe deposit	Small		Li,C.Y., et al., 2021,1995,2019.Development and Research Center, China Geological Survey.
1028	99.412	40.550	Fe	Fe deposit	Small	C1	Li,C.Y., et al., 2021,1995,2019.Development and Research Center, China Geological Survey.
1029	99.654	40.735	Fe	Fe deposit	Small	C1	Li,C.Y., et al., 2021,1995,2019.Development and Research Center, China Geological Survey.
1030	85.042	46.000	Fe	Fe deposit	Small	C	Li,C.Y., et al., 2021,1995,2019.Development and Research Center, China Geological Survey.
1031	71.531	52.719	Fe	Fe deposit	Small	O3	National Mineral Origin Database of China
1032	83.327	43.618	Fe	Fe deposit	Small	C3	Feng, J. et al., 2021. Geological Publishing House
1033	85.558	43.250	Fe	Fe deposit	Medium		Li,C.Y., et al., 2021,1995,2019.Development and Research Center, China Geological Survey.
1034	70.833	48.019	Fe	Fe deposit	Super Large		National Mineral Origin Database of China
1035	75.429	41.940	Fe	Fe deposit	Small		National Mineral Origin Database of China
1036	82.393	45.953	Fe	Fe deposit	Small	C1	Feng, J. et al., 2021. Geological Publishing House
1037	77.233	40.800	Fe	Fe deposit	Small	D1	Feng, J. et al., 2021. Geological Publishing House
1038	70.611	42.358	Fe	Fe deposit	Small	P-T	National Mineral Origin Database of China
1039	89.199	47.329	Fe	Fe deposit	Small	D1	Feng, J. et al., 2021. Geological Publishing House
1040	88.125	43.058	Fe	Fe deposit	Small	J2	Li,C.Y., et al., 2021,1995,2019.Development and Research Center, China Geological Survey.
1041	77.315	40.800	Fe	Fe deposit	Small	D1	Feng, J. et al., 2021. Geological Publishing House
1042	76.026	42.693	Fe	Fe deposit	Small		National Mineral Origin Database of China
1043	88.975	47.533	Fe	Fe deposit	Medium	D3	Feng, J. et al., 2021. Geological Publishing House
1044	69.724	41.636	Fe	Fe deposit	Small		National Mineral Origin Database of China
1045	70.017	41.980	Fe	Fe deposit	Small		National Mineral Origin Database of China
1046	89.468	47.025	Fe	Fe deposit	Small	P2	Feng, J. et al., 2021. Geological Publishing House
1047	74.386	41.356	Fe	Fe deposit	Small		National Mineral Origin Database of China
1048	89.717	41.167	Fe	Fe deposit	Small		Li,C.Y., et al., 2021,1995,2019.Development and Research Center, China Geological Survey.
1049	71.513	45.281	Fe	Fe deposit	Small		National Mineral Origin Database of China
1050	93.200	41.767	Fe	Fe deposit	Medium	C3	Feng, J. et al., 2021. Geological Publishing House
1051	93.000	41.521	Fe	Fe deposit	Small	MP	Feng, J. et al., 2021. Geological Publishing House
1052	71.534	45.314	Fe	Fe deposit	Small		National Mineral Origin Database of China
1053	74.338	39.921	Fe	Fe deposit	Small	C3	Feng, J. et al., 2021. Geological Publishing House
1054	71.423	51.930	Fe	Fe deposit	Small	O3	National Mineral Origin Database of China
1055	73.455	41.804	Fe	Fe deposit	Small	S?	National Mineral Origin Database of China
1056	82.337	43.286	Fe	Fe deposit	Medium	C1	Feng, J. et al., 2021. Geological Publishing House
1057	88.362	47.791	Fe	Fe deposit	Small	D1	Feng, J. et al., 2021. Geological Publishing House
1058	81.250	41.917	Fe	Fe deposit	Small	C3	Li,C.Y., et al., 2021,1995,2019.Development and Research Center, China Geological Survey.
1059	90.100	46.467	Fe	Fe deposit	Small	D2	Feng, J. et al., 2021. Geological Publishing House
1060	93.813	44.574	Fe	Fe deposit	Small	C1	Li,C.Y., et al., 2021,1995,2019.Development and Research Center, China Geological Survey.
1061	70.471	42.374	Fe	Fe deposit	Small	P-T	National Mineral Origin Database of China
1062	91.708	41.733	Fe	Fe deposit	Small	C3	Feng, J. et al., 2021. Geological Publishing House
1063	130.754	47.899	Fe	Fe deposit	Small	Pz1	Li,C.Y., et al., 2021,1995,2019.Development and Research Center, China Geological Survey.
1064	91.733	41.875	Fe	Fe deposit	Small	C1	Feng, J. et al., 2021. Geological Publishing House
1065	98.136	42.413	Fe	Fe deposit	Small	C-P	Li,C.Y., et al., 2021,1995,2019.Development and Research Center, China Geological Survey.
1066	98.376	42.302	Fe	Fe deposit	Medium	C	Li,C.Y., et al., 2021,1995,2019.Development and Research Center, China Geological Survey.
1067	98.333	40.959	Fe	Fe deposit	Small	D	Li,C.Y., et al., 2021,1995,2019.Development and Research Center, China Geological Survey.
1068	97.400	42.467	Fe	Fe deposit	Small	D-P	Li,C.Y., et al., 2021,1995,2019.Development and Research Center, China Geological Survey.
1069	118.500	46.458	Fe	Fe deposit	Large	J	Li,C.Y., et al., 2021,1995,2019.Development and Research Center, China Geological Survey.
1070	83.146	45.644	Fe	Fe deposit	Small	C1	Feng, J. et al., 2021. Geological Publishing House
1071	88.992	47.529	Fe	Fe deposit	Large	D1	Feng, J. et al., 2021. Geological Publishing House
1072	88.930	47.585	Fe	Fe deposit	Large	C-P	Li,C.Y., et al., 2021,1995,2019.Development and Research Center, China Geological Survey.
1073	86.125	42.788	Fe	Fe deposit	Medium	C	Li,C.Y., et al., 2021,1995,2019.Development and Research Center, China Geological Survey.
1074	83.993	43.524	Fe	Fe deposit	Medium	C1	Feng, J. et al., 2021. Geological Publishing House
1075	88.917	43.422	Fe	Fe deposit	Small	C3	Feng, J. et al., 2021. Geological Publishing House
1076	83.603	43.548	Fe	Fe deposit	Small	C3	Feng, J. et al., 2021. Geological Publishing House
1077	70.008	41.758	Fe	Fe deposit	Small		National Mineral Origin Database of China
1078	95.983	41.894	Fe	Fe deposit	Small	C1	Feng, J. et al., 2021. Geological Publishing House
1079	95.968	41.900	Fe	Fe deposit	Small	C1	Li,C.Y., et al., 2021,1995,2019.Development and Research Center, China Geological Survey.
1080	68.871	53.374	Fe	Fe deposit	Small	D2	National Mineral Origin Database of China
1081	69.864	40.497	Fe	Fe deposit	Medium		National Mineral Origin Database of China
1082	89.667	46.808	Fe	Fe deposit	Small	D2	Feng, J. et al., 2021. Geological Publishing House
1083	89.667	46.833	Fe	Fe deposit	Small	Pz2	Li,C.Y., et al., 2021,1995,2019.Development and Research Center, China Geological Survey.
1084	88.250	42.367	Fe	Fe deposit	Small	D3	Feng, J. et al., 2021. Geological Publishing House
1085	95.141	43.981	Fe	Fe deposit	Small	D1	Feng, J. et al., 2021. Geological Publishing House

1086	95.080	44.041	Fe	Fe deposit	Small		Li,C.Y., et al., 2021,1995,2019.Development and Research Center, China Geological Survey.
1087	77.197	40.365	Fe	Fe deposit	Small	P1	Feng, J. et al., 2021. Geological Publishing House
1088	88.833	47.667	Fe	Fe deposit	Small	C3	Feng, J. et al., 2021. Geological Publishing House
1089	88.975	47.480	Fe	Fe deposit	Small		Li,C.Y., et al., 2021,1995,2019.Development and Research Center, China Geological Survey.
1090	91.572	41.765	Fe	Fe deposit	Medium	C3	Feng, J. et al., 2021. Geological Publishing House
1091	84.967	46.017	Fe	Fe deposit	Medium	C	Feng, J. et al., 2021. Geological Publishing House
1092	85.083	46.033	Fe	Fe deposit	Medium	Pz2	Li,C.Y., et al., 2021,1995,2019.Development and Research Center, China Geological Survey.
1093	83.651	43.667	Fe	Fe deposit	Medium	C1	Feng, J. et al., 2021. Geological Publishing House
1094	88.200	47.864	Fe	Fe deposit	Small	D1	Li,C.Y., et al., 2021,1995,2019.Development and Research Center, China Geological Survey.
1095	99.928	42.299	Fe	Fe deposit	Small	C	Li,C.Y., et al., 2021,1995,2019.Development and Research Center, China Geological Survey.
1096	75.093	39.792	Fe	Fe deposit	Small	C3	Feng, J. et al., 2021. Geological Publishing House
1097	94.800	41.906	Fe	Fe deposit	Small	C3	Feng, J. et al., 2021. Geological Publishing House
1098	91.147	42.013	Fe	Fe deposit	Small	C1	Feng, J. et al., 2021. Geological Publishing House
1099	89.020	43.923	Fe	Fe deposit	Small	J1	Li,C.Y., et al., 2021,1995,2019.Development and Research Center, China Geological Survey.
1100	83.645	43.527	Fe	Fe deposit	Medium	C3	Feng, J. et al., 2021. Geological Publishing House
1101	83.617	43.550	Fe	Fe deposit	Medium	C2	Li,C.Y., et al., 2021,1995,2019.Development and Research Center, China Geological Survey.
1102	94.678	41.875	Fe	Fe deposit	Small	C3	Feng, J. et al., 2021. Geological Publishing House
1103	95.750	41.941	Fe	Fe deposit	Small	C	Li,C.Y., et al., 2021,1995,2019.Development and Research Center, China Geological Survey.
1104	125.650	43.800	Fe	Fe deposit	Small	Pz	Li,C.Y., et al., 2021,1995,2019.Development and Research Center, China Geological Survey.
1105	88.858	43.948	Fe	Fe deposit	Small	P2	Feng, J. et al., 2021. Geological Publishing House
1106	83.663	43.608	Fe	Fe deposit	Medium	C1	Feng, J. et al., 2021. Geological Publishing House
1107	119.642	50.606	Fe	Fe deposit	Small	J2-K	Li,C.Y., et al., 2021,1995,2019.Development and Research Center, China Geological Survey.
1108	70.195	42.103	Fe	Fe deposit	Small	C2	National Mineral Origin Database of China
1109	97.317	41.455	Fe	Fe deposit	Small	Pz	Li,C.Y., et al., 2021,1995,2019.Development and Research Center, China Geological Survey.
1110	97.067	42.229	Fe	Fe deposit	Medium	C1	Li,C.Y., et al., 2021,1995,2019.Development and Research Center, China Geological Survey.
1111	74.175	41.637	Fe	Fe deposit	Small		National Mineral Origin Database of China
1112	128.550	43.883	Fe	Fe deposit	Large		Lv B. et al., 2017. Journal of Jilin University (Earth Science Edition) (In Chinese with English) Abstract)
1113	82.009	50.532	Fe	Fe deposit	Small	O?	National Mineral Origin Database of China
1114	73.787	47.630	Fe	Fe deposit	Small		National Mineral Origin Database of China
1115	87.745	43.949	Fe	Fe deposit	Small	J2	Li,C.Y., et al., 2021,1995,2019.Development and Research Center, China Geological Survey.
1116	87.533	42.917	Fe	Fe deposit	Small	C3	Feng, J. et al., 2021. Geological Publishing House
1117	87.542	42.919	Fe	Fe deposit	Small	C	Li,C.Y., et al., 2021,1995,2019.Development and Research Center, China Geological Survey.
1118	87.524	42.914	Fe	Fe deposit	Small	D3	Li,C.Y., et al., 2021,1995,2019.Development and Research Center, China Geological Survey.
1119	91.103	41.775	Fe	Fe deposit	Medium	C	Li,C.Y., et al., 2021,1995,2019.Development and Research Center, China Geological Survey.
1120	83.113	43.654	Fe	Fe deposit	Small	C3	Feng, J. et al., 2021. Geological Publishing House
1121	117.375	43.488	Fe	Fe deposit	Large	K	Li,C.Y., et al., 2021,1995,2019.Development and Research Center, China Geological Survey.
1122	83.605	43.552	Fe	Fe deposit	Small	C3	Feng, J. et al., 2021. Geological Publishing House
1123	90.097	46.467	Fe	Fe deposit	Small	P	Li,C.Y., et al., 2021,1995,2019.Development and Research Center, China Geological Survey.
1124	76.622	48.714	Fe	Fe deposit	Small		National Mineral Origin Database of China
1125	94.317	41.783	Fe	Fe deposit	Medium	Pz2	Li,C.Y., et al., 2021,1995,2019.Development and Research Center, China Geological Survey.
1126	112.833	42.424	Fe	Fe deposit	Medium	∈	Li,C.Y., et al., 2021,1995,2019.Development and Research Center, China Geological Survey.
1127	120.925	46.400	Fe	Fe deposit	Small	J2-K	Li,C.Y., et al., 2021,1995,2019.Development and Research Center, China Geological Survey.
1128	69.918	41.740	Fe	Fe deposit	Small		National Mineral Origin Database of China
1129	86.097	42.676	Fe	Fe deposit	Small	C1	Feng, J. et al., 2021. Geological Publishing House
1130	88.883	41.811	Fe	Fe deposit	Small	D-C	Li,C.Y., et al., 2021,1995,2019.Development and Research Center, China Geological Survey.
1131	89.883	41.758	Fe	Fe deposit	Large	C	Li,C.Y., et al., 2021,1995,2019.Development and Research Center, China Geological Survey.
1132	128.333	47.167	Fe	Fe deposit	Medium		Lv B. et al., 2017. Journal of Jilin University (Earth Science Edition) (In Chinese with English) Abstract)
1133	120.944	48.383	Fe	Fe deposit	Small	C	Li,C.Y., et al., 2021,1995,2019.Development and Research Center, China Geological Survey.
1134	86.353	42.953	Fe	Fe deposit	Small	P1	Feng, J. et al., 2021. Geological Publishing House
1135	86.336	42.811	Fe	Fe deposit	Small		Li,C.Y., et al., 2021,1995,2019.Development and Research Center, China Geological Survey.
1136	88.265	44.100	Fe	Fe deposit	Small	J	Li,C.Y., et al., 2021,1995,2019.Development and Research Center, China Geological Survey.
1137	95.064	44.041	Fe	Fe deposit	Small	D1	Li,C.Y., et al., 2021,1995,2019.Development and Research Center, China Geological Survey.
1138	88.858	43.417	Fe	Fe deposit	Small	C3	Feng, J. et al., 2021. Geological Publishing House
1139	69.850	41.371	Fe	Fe deposit	Small	C2	National Mineral Origin Database of China
1140	85.393	42.816	Fe	Fe deposit	Small	C3	Feng, J. et al., 2021. Geological Publishing House
1141	93.877	41.891	Fe	Fe deposit	Medium	C1	Li,C.Y., et al., 2021,1995,2019.Development and Research Center, China Geological Survey.
1142	86.347	42.947	Fe	Fe deposit	Small	P1	Feng, J. et al., 2021. Geological Publishing House
1143	131.050	46.567	Fe	Fe deposit	Large		Lv B. et al., 2017. Journal of Jilin University (Earth Science Edition) (In Chinese with English) Abstract)
1144	129.032	47.535	Fe	Fe deposit	Small	K1	Li,C.Y., et al., 2021,1995,2019.Development and Research Center, China Geological Survey.
1145	120.342	47.479	Fe	Fe deposit	Small	D-C-P	Li,C.Y., et al., 2021,1995,2019.Development and Research Center, China Geological Survey.
1146	120.813	48.019	Fe	Fe deposit	Small	D	Li,C.Y., et al., 2021,1995,2019.Development and Research Center, China Geological Survey.
1147	126.412	43.494	Fe	Fe deposit	Small	Mz	Li,C.Y., et al., 2021,1995,2019.Development and Research Center, China Geological Survey.
1148	93.821	41.575	Fe	Fe deposit	Small		Li,C.Y., et al., 2021,1995,2019.Development and Research Center, China Geological Survey.
1149	125.672	43.810	Fe	Fe deposit	Small	Pz	Li,C.Y., et al., 2021,1995,2019.Development and Research Center, China Geological Survey.

1150	85.033	43.325	Fe	Fe deposit	Large	C1	Feng, J. et al., 2021. Geological Publishing House
1151	82.782	45.703	Fe	Fe deposit	Small	D3	Feng, J. et al., 2021. Geological Publishing House
1152	95.650	46.881	Fe,Ti	Fe deposit	Occurrence	NP3	Ochir Gerel et al., 2021 Mineral Resources of Mongolia (book) and refs therein
1153	89.438	47.431	Li	Li-Be-Nb-Ta-Zr deposit	Occurrence	C1	National Mineral Origin Database of China
1154	86.950	49.460	Li	Li-Be-Nb-Ta-Zr deposit	Large		National Mineral Origin Database of China
1155	88.813	47.190	Li	Li-Be-Nb-Ta-Zr deposit			Seltmann, R., et al., 2004. Natural History Museum
1156	89.807	47.206	Li	Li-Be-Nb-Ta-Zr deposit	Large		National Mineral Origin Database of China
1157	90.250	46.750	Li	Li-Be-Nb-Ta-Zr deposit	Occurrence	C1	National Mineral Origin Database of China
1158	90.839	46.602	Li	Li-Be-Nb-Ta-Zr deposit	Occurrence	C1	National Mineral Origin Database of China
1159	90.269	46.754	Li	Li-Be-Nb-Ta-Zr deposit	Small	C1	National Mineral Origin Database of China
1160	89.700	47.378	Li	Li-Be-Nb-Ta-Zr deposit	Occurrence	C1	National Mineral Origin Database of China
1161	89.733	47.200	Li	Li-Be-Nb-Ta-Zr deposit	Occurrence		National Mineral Origin Database of China
1162	88.875	47.914	Li	Li-Be-Nb-Ta-Zr deposit	Small	C3-P1	Feng, J. et al., 2021. Geological Publishing House
1163	95.667	42.532	Li	Li-Be-Nb-Ta-Zr deposit	Small	P1	Feng, J. et al., 2021. Geological Publishing House
1164	89.118	47.903	Li	Li-Be-Nb-Ta-Zr deposit	Medium	C3-P1	Feng, J. et al., 2021. Geological Publishing House
1165	89.817	47.207	Li	Li-Be-Nb-Ta-Zr deposit	Medium	C1	National Mineral Origin Database of China
1166	89.815	47.207	Li	Li-Be-Nb-Ta-Zr deposit	Large	D	Li,C.Y., et al., 2021,1995,2019.Development and Research Center, China Geological Survey.
1167	90.698	46.622	Li	Li-Be-Nb-Ta-Zr deposit	Small	C1	National Mineral Origin Database of China
1168	88.842	47.907	Li	Li-Be-Nb-Ta-Zr deposit	Medium	C3-P1	Feng, J. et al., 2021. Geological Publishing House
1169	88.917	47.867	Li	Li-Be-Nb-Ta-Zr deposit	Medium	P	Li,C.Y., et al., 2021,1995,2019.Development and Research Center, China Geological Survey.
1170	88.083	47.833	Li	Li-Be-Nb-Ta-Zr deposit	Medium	C3-P1	Feng, J. et al., 2021. Geological Publishing House
1171	90.230	46.751	Li	Li-Be-Nb-Ta-Zr deposit	Occurrence	C1	National Mineral Origin Database of China
1172	90.500	46.750	Li	Li-Be-Nb-Ta-Zr deposit	Occurrence	C1	National Mineral Origin Database of China
1173	90.480	46.690	Li	Li-Be-Nb-Ta-Zr deposit	Medium	C1	National Mineral Origin Database of China
1174	89.887	47.222	Li	Li-Be-Nb-Ta-Zr deposit	Small	C1	National Mineral Origin Database of China
1175	90.625	46.701	Li	Li-Be-Nb-Ta-Zr deposit	Occurrence	C1	National Mineral Origin Database of China
1176	90.616	46.600	Li	Li-Be-Nb-Ta-Zr deposit	Occurrence	C1	National Mineral Origin Database of China
1177	90.159	47.515	Li	Li-Be-Nb-Ta-Zr deposit	Occurrence	C1	National Mineral Origin Database of China
1178	88.937	48.954	Li-Be	Li-Be-Nb-Ta-Zr deposit	Medium	P	Ochir Gerel et al., 2021 Mineral Resources of Mongolia (book) and refs therein
1179	88.600	47.450	Li-Nb-Ta-Be	Li-Be-Nb-Ta-Zr deposit	Occurrence		National Mineral Origin Database of China
1180	88.483	47.767	Li-Nb-Ta-Be	Li-Be-Nb-Ta-Zr deposit	Occurrence		National Mineral Origin Database of China
1181	88.100	47.833	Li-Nb-Ta-Be	Li-Be-Nb-Ta-Zr deposit	Occurrence		National Mineral Origin Database of China
1182	80.793	42.678	Mn	Mn deposit	Small	C	Li,C.Y., et al., 2021,1995,2019.Development and Research Center, China Geological Survey.
1183	80.750	42.667	Mn	Mn deposit	Small	C	Li,C.Y., et al., 2021,1995,2019.Development and Research Center, China Geological Survey.
1184	80.760	42.678	Mn	Mn deposit	Small	Ꞓ	Feng, J. et al., 2021. Geological Publishing House
1185	70.431	40.158	Mn	Mo deposit	Small		National Mineral Origin Database of China
1186	69.954	41.590	Mn	Mo deposit	Small		National Mineral Origin Database of China
1187	80.957	42.722	Mn	Mn deposit	Medium	C	Li,C.Y., et al., 2021,1995,2019.Development and Research Center, China Geological Survey.
1188	98.334	40.500	Mn	Mn deposit	Small	P1	Li,C.Y., et al., 2021,1995,2019.Development and Research Center, China Geological Survey.
1189	82.950	42.335	Mn	Mn deposit	Small	D1	Feng, J. et al., 2021. Geological Publishing House
1190	113.950	47.225	Mo	Mo deposit	Medium	MZ1	Ochir Gerel et al., 2021 Mineral Resources of Mongolia (book) and refs therein
1191	114.844	49.671	Mo	Mo deposit	Small	MZ2	Ochir Gerel et al., 2021 Mineral Resources of Mongolia (book) and refs therein
1192	101.750	44.850	Mo	Mo deposit	Occurrence	PZ3	Ochir Gerel et al., 2021 Mineral Resources of Mongolia (book) and refs therein
1193	109.250	47.800	Mo	Mo deposit	Medium	MZ2	Ochir Gerel et al., 2021 Mineral Resources of Mongolia (book) and refs therein
1194	101.300	44.767	Mo	Mo deposit	Occurrence	PZ3	Ochir Gerel et al., 2021 Mineral Resources of Mongolia (book) and refs therein
1195	113.442	46.842	Mo	Mo deposit	Large	MZ1	Ochir Gerel et al., 2021 Mineral Resources of Mongolia (book) and refs therein
1196	97.783	48.567	Mo	Mo deposit	Small	P3	Ochir Gerel et al., 2021 Mineral Resources of Mongolia (book) and refs therein
1197	110.183	47.700	Mo	Mo deposit	Medium	MZ2	Ochir Gerel et al., 2021 Mineral Resources of Mongolia (book) and refs therein
1198	98.342	48.708	Mo	Mo deposit	Large	P3	Ochir Gerel et al., 2021 Mineral Resources of Mongolia (book) and refs therein
1199	75.739	50.720	Mo	Mo deposit	Small		National Mineral Origin Database of China
1200	73.283	47.046	Mo	Mo deposit	Medium	P	National Mineral Origin Database of China
1201	73.176	47.013	Mo	Mo deposit	Medium		National Mineral Origin Database of China
1202	113.950	47.217	Mo	Mo deposit	Medium		Lv B. et al., 2017. Journal of Jilin University (Earth Science Edition) (In Chinese with English) Abstract)
1203	95.559	41.217	Mn	Mn deposit	Medium	Z	Li,C.Y., et al., 2021,1995,2019.Development and Research Center, China Geological Survey.
1204	126.417	43.267	Mo	Mo deposit	Medium		Lv B. et al., 2017. Journal of Jilin University (Earth Science Edition) (In Chinese with English) Abstract)
1205	73.893	44.833	Mo	Mo deposit	Medium		National Mineral Origin Database of China
1206	119.088	49.971	Mn	Mn deposit	Small	C1	Li,C.Y., et al., 2021,1995,2019.Development and Research Center, China Geological Survey.
1207	120.000	47.167	Mn	Mn deposit	Small	J2-K	Li,C.Y., et al., 2021,1995,2019.Development and Research Center, China Geological Survey.
1208	95.975	42.525	Mn	Mn deposit	Large	T3	Feng, J. et al., 2021. Geological Publishing House
1209	95.833	42.511	Mn	Mn deposit	Small	C1-2	Li,C.Y., et al., 2021,1995,2019.Development and Research Center, China Geological Survey.
1210	119.800	42.383	Mo	Mo deposit	Small		Lv B. et al., 2017. Journal of Jilin University (Earth Science Edition) (In Chinese with English) Abstract)
1211	120.083	44.033	Mo	Mo deposit	Medium		Lv B. et al., 2017. Journal of Jilin University (Earth Science Edition) (In Chinese with English) Abstract)
1212	114.467	44.083	Mo	Mo deposit	Large		Lv B. et al., 2017. Journal of Jilin University (Earth Science Edition) (In Chinese with English) Abstract)
1213	73.649	47.158	Mo	Mo deposit	Medium		National Mineral Origin Database of China

1214	70.455	47.510	Mo	Mo deposit	Medium	P	National Mineral Origin Database of China
1215	107.500	41.850	Mo	Mo deposit	Small		Lv B. et al., 2017. Journal of Jilin University (Earth Science Edition) (In Chinese with English) Abstract)
1216	107.350	41.900	Mo	Mo deposit	Large		Lv B. et al., 2017. Journal of Jilin University (Earth Science Edition) (In Chinese with English) Abstract)
1217	70.158	41.669	Mo	Mo deposit	Small		National Mineral Origin Database of China
1218	123.917	51.167	Mo	Mo deposit	Large		Lv B. et al., 2017. Journal of Jilin University (Earth Science Edition) (In Chinese with English) Abstract)
1219	118.500	42.417	Mo	Mo deposit	Medium		Lv B. et al., 2017. Journal of Jilin University (Earth Science Edition) (In Chinese with English) Abstract)
1220	93.008	42.163	Mn	Mn deposit	Small	C	Li,C.Y., et al., 2021,1995,2019.Development and Research Center, China Geological Survey.
1221	128.739	48.490	Mn	Mn deposit	Large	C1	Li,C.Y., et al., 2021,1995,2019.Development and Research Center, China Geological Survey.
1222	128.600	48.267	Mo	Mo deposit	Small		Lv B. et al., 2017. Journal of Jilin University (Earth Science Edition) (In Chinese with English) Abstract)
1223	127.617	43.000	Mo	Mo deposit	Small		Lv B. et al., 2017. Journal of Jilin University (Earth Science Edition) (In Chinese with English) Abstract)
1224	126.317	43.483	Mo	Mo deposit	Large		Lv B. et al., 2017. Journal of Jilin University (Earth Science Edition) (In Chinese with English) Abstract)
1225	118.508	42.419	Mn	Mn deposit	Small	J2-K	Li,C.Y., et al., 2021,1995,2019.Development and Research Center, China Geological Survey.
1226	127.650	43.683	Mo	Mo deposit	Large		Lv B. et al., 2017. Journal of Jilin University (Earth Science Edition) (In Chinese with English) Abstract)
1227	118.233	46.000	Mo	Mo deposit	Small		Lv B. et al., 2017. Journal of Jilin University (Earth Science Edition) (In Chinese with English) Abstract)
1228	93.338	41.917	Mn	Mn deposit	Large	C1	Feng, J. et al., 2021. Geological Publishing House
1229	118.860	49.736	Mn	Mn deposit	Small	J2-K	Li,C.Y., et al., 2021,1995,2019.Development and Research Center, China Geological Survey.
1230	127.251	44.400	Mo	Mo deposit	Medium		Lv B. et al., 2017. Journal of Jilin University (Earth Science Edition) (In Chinese with English) Abstract)
1231	117.883	42.950	Mo	Mo deposit	Small		Lv B. et al., 2017. Journal of Jilin University (Earth Science Edition) (In Chinese with English) Abstract)
1232	128.983	48.933	Mo	Mo deposit	Medium		Lv B. et al., 2017. Journal of Jilin University (Earth Science Edition) (In Chinese with English) Abstract)
1233	118.017	44.050	Mo	Mo deposit	Medium		Lv B. et al., 2017. Journal of Jilin University (Earth Science Edition) (In Chinese with English) Abstract)
1234	119.150	44.117	Mo	Mo deposit	Large		Lv B. et al., 2017. Journal of Jilin University (Earth Science Edition) (In Chinese with English) Abstract)
1235	128.933	48.500	Mo	Mo deposit	Large		Lv B. et al., 2017. Journal of Jilin University (Earth Science Edition) (In Chinese with English) Abstract)
1236	119.083	42.417	Mo	Mo deposit	Medium		Lv B. et al., 2017. Journal of Jilin University (Earth Science Edition) (In Chinese with English) Abstract)
1237	126.800	44.100	Mo	Mo deposit	Large		Lv B. et al., 2017. Journal of Jilin University (Earth Science Edition) (In Chinese with English) Abstract)
1238	129.800	43.333	Mo	Mo deposit	Medium		Lv B. et al., 2017. Journal of Jilin University (Earth Science Edition) (In Chinese with English) Abstract)
1239	131.033	44.883	Mo	Mo deposit	Small		Lv B. et al., 2017. Journal of Jilin University (Earth Science Edition) (In Chinese with English) Abstract)
1240	89.042	41.500	Mn	Mn deposit	Small	C3	Feng, J. et al., 2021. Geological Publishing House
1241	78.263	46.799	Mo	Mo deposit	Small	P1	National Mineral Origin Database of China
1242	130.183	44.667	Mo	Mo deposit	Small		Lv B. et al., 2017. Journal of Jilin University (Earth Science Edition) (In Chinese with English) Abstract)
1243	74.207	41.941	Mo	Mo deposit	Small		National Mineral Origin Database of China
1244	72.260	48.621	Mo	Mo deposit	Large	P	National Mineral Origin Database of China
1245	75.003	46.990	Mo	Mo deposit	Large		National Mineral Origin Database of China
1246	76.427	50.660	Mo	Mo deposit	Small	Pz3	National Mineral Origin Database of China
1247	83.333	44.167	Mn	Mn deposit	Small	C	Li,C.Y., et al., 2021,1995,2019.Development and Research Center, China Geological Survey.
1248	67.022	49.246	Mo	Mo deposit	Small		National Mineral Origin Database of China
1249	74.928	43.293	Mo	Mo deposit	Small		National Mineral Origin Database of China
1250	81.864	43.385	Mn	Mn deposit	Large	P1	Feng, J. et al., 2021. Geological Publishing House
1251	93.176	41.676	Mn	Mn deposit	Small	C3	Feng, J. et al., 2021. Geological Publishing House
1252	82.959	44.072	Mn	Mn deposit	Small	C1	Feng, J. et al., 2021. Geological Publishing House
1253	120.067	43.750	Mo	Mo deposit	Medium		Lv B. et al., 2017. Journal of Jilin University (Earth Science Edition) (In Chinese with English) Abstract)
1254	98.367	42.250	Mo	Mo deposit	Medium		Lv B. et al., 2017. Journal of Jilin University (Earth Science Edition) (In Chinese with English) Abstract)
1255	117.733	43.026	Mn	Mn deposit	Medium	J2-K	Li,C.Y., et al., 2021,1995,2019.Development and Research Center, China Geological Survey.
1256	128.550	47.367	Mo	Mo deposit	Large		Lv B. et al., 2017. Journal of Jilin University (Earth Science Edition) (In Chinese with English) Abstract)
1257	125.824	50.224	Mn	Mn deposit	Large	J3-K1	Li,C.Y., et al., 2021,1995,2019.Development and Research Center, China Geological Survey.
1258	118.650	42.417	Mo	Mo deposit	Small		Lv B. et al., 2017. Journal of Jilin University (Earth Science Edition) (In Chinese with English) Abstract)
1259	67.105	49.284	Mo	Mo deposit	Small		National Mineral Origin Database of China
1260	70.070	41.521	Mo	Mo deposit	Small	C2	National Mineral Origin Database of China
1261	128.417	47.292	Mn	Mn deposit	Medium	T1	Li,C.Y., et al., 2021,1995,2019.Development and Research Center, China Geological Survey.
1262	119.810	42.413	Mn	Mn deposit	Small	J2-K	Li,C.Y., et al., 2021,1995,2019.Development and Research Center, China Geological Survey.
1263	70.546	47.498	Mo	Mo deposit	Medium		National Mineral Origin Database of China
1264	99.598	41.386	Mn	Mn deposit	Medium	K	Li,C.Y., et al., 2021,1995,2019.Development and Research Center, China Geological Survey.
1265	119.000	44.133	Mo	Mo deposit	Small		Lv B. et al., 2017. Journal of Jilin University (Earth Science Edition) (In Chinese with English) Abstract)
1266	70.680	47.546	Mo	Mo deposit	Medium	P	National Mineral Origin Database of China
1267	71.157	42.002	Mo	Mo deposit	Small	C2	National Mineral Origin Database of China
1268	129.617	48.467	Mo	Mo deposit	Small		Lv B. et al., 2017. Journal of Jilin University (Earth Science Edition) (In Chinese with English) Abstract)
1269	127.367	48.417	Mo	Mo deposit	Small		Lv B. et al., 2017. Journal of Jilin University (Earth Science Edition) (In Chinese with English) Abstract)
1270	119.108	42.421	Mn	Mn deposit	Medium	J2-K	Li,C.Y., et al., 2021,1995,2019.Development and Research Center, China Geological Survey.
1271	126.400	43.300	Mo	Mo deposit	Medium		Lv B. et al., 2017. Journal of Jilin University (Earth Science Edition) (In Chinese with English) Abstract)
1272	82.468	45.780	Mn	Mn deposit	Medium	C1	Feng, J. et al., 2021. Geological Publishing House
1273	96.800	41.275	Mn	Mn deposit	Small	C2	Li,C.Y., et al., 2021,1995,2019.Development and Research Center, China Geological Survey.
1274	117.900	46.267	Mo	Mo deposit	Medium		Lv B. et al., 2017. Journal of Jilin University (Earth Science Edition) (In Chinese with English) Abstract)
1275	123.683	49.100	Mo	Mo deposit	Medium		Lv B. et al., 2017. Journal of Jilin University (Earth Science Edition) (In Chinese with English) Abstract)
1276	71.413	48.262	Mo	Mo deposit	Small		National Mineral Origin Database of China
1277	69.561	40.754	Mo	Mo deposit	Small	S1?	National Mineral Origin Database of China

1278	112.650	44.517	Mo	Mo deposit	Medium		Lv B. et al., 2017. Journal of Jilin University (Earth Science Edition) (In Chinese with English) Abstract)
1279	112.850	44.800	Mo	Mo deposit	Medium		Lv B. et al., 2017. Journal of Jilin University (Earth Science Edition) (In Chinese with English) Abstract)
1280	111.717	44.633	Mo	Mo deposit	Small		Lv B. et al., 2017. Journal of Jilin University (Earth Science Edition) (In Chinese with English) Abstract)
1281	74.004	48.363	Mo	Mo deposit	Small	P	National Mineral Origin Database of China
1282	127.217	45.300	Mo	Mo deposit	Medium		Lv B. et al., 2017. Journal of Jilin University (Earth Science Edition) (In Chinese with English) Abstract)
1283	117.717	43.017	Mo	Mo deposit	Medium		Lv B. et al., 2017. Journal of Jilin University (Earth Science Edition) (In Chinese with English) Abstract)
1284	100.567	42.517	Mo	Mo deposit	Medium		Lv B. et al., 2017. Journal of Jilin University (Earth Science Edition) (In Chinese with English) Abstract)
1285	121.800	50.133	Mo	Mo deposit	Small		Lv B. et al., 2017. Journal of Jilin University (Earth Science Edition) (In Chinese with English) Abstract)
1286	123.500	50.683	Mo	Mo deposit	Large		Lv B. et al., 2017. Journal of Jilin University (Earth Science Edition) (In Chinese with English) Abstract)
1287	126.267	43.000	Mo	Mo deposit	Small		Lv B. et al., 2017. Journal of Jilin University (Earth Science Edition) (In Chinese with English) Abstract)
1288	119.033	43.500	Mo	Mo deposit	Small		Lv B. et al., 2017. Journal of Jilin University (Earth Science Edition) (In Chinese with English) Abstract)
1289	74.390	43.279	Mo	Mo deposit	Medium		National Mineral Origin Database of China
1290	74.814	48.844	Mo	Mo deposit	Small	C2	National Mineral Origin Database of China
1291	124.267	49.417	Mo	Mo deposit	Medium		Lv B. et al., 2017. Journal of Jilin University (Earth Science Edition) (In Chinese with English) Abstract)
1292	126.412	43.494	Mn	Mn deposit	Small	Mz	Li,C.Y., et al., 2021,1995,2019.Development and Research Center, China Geological Survey.
1293	126.320	43.489	Mn	Mn deposit	Large	Mz	Li,C.Y., et al., 2021,1995,2019.Development and Research Center, China Geological Survey.
1294	77.278	43.161	Mo	Mo deposit	Medium		National Mineral Origin Database of China
1295	118.733	42.267	Mo	Mo deposit	Small		Lv B. et al., 2017. Journal of Jilin University (Earth Science Edition) (In Chinese with English) Abstract)
1296	80.715	48.562	Mo	Mo deposit	Small	P	National Mineral Origin Database of China
1297	74.325	47.529	Mo	Mo deposit	Large	P	National Mineral Origin Database of China
1298	76.412	42.894	Mo	Mo deposit	Small		National Mineral Origin Database of China
1299	112.683	44.517	Mo	Mo deposit	Medium		Lv B. et al., 2017. Journal of Jilin University (Earth Science Edition) (In Chinese with English) Abstract)
1300	88.363	47.685	Nb	Li-Be-Nb-Ta-Zr deposit	Medium	Pz2	National Mineral Origin Database of China
1301	120.967	44.617	Nb-Ta	Li-Be-Nb-Ta-Zr deposit			Schulz, K.J. et al., 2017. U.S. Geological Survey Professional Paper
1302	86.961	49.439	Nb-Ta	Li-Be-Nb-Ta-Zr deposit			Schulz, K.J. et al., 2017. U.S. Geological Survey Professional Paper
1303	82.960	49.700	Ta-Nb	Li-Be-Nb-Ta-Zr deposit	Large		National Mineral Origin Database of China
1304	83.090	49.470	Ta-Nb	Li-Be-Nb-Ta-Zr deposit	Large		National Mineral Origin Database of China
1305	83.710	49.400	Ta-Nb	Li-Be-Nb-Ta-Zr deposit	Small		National Mineral Origin Database of China
1306	88.245	47.790	Ta-Nb	Li-Be-Nb-Ta-Zr deposit	Medium		National Mineral Origin Database of China
1307	83.760	49.320	Ta-Nb	Li-Be-Nb-Ta-Zr deposit	Small		National Mineral Origin Database of China
1308	88.363	47.685	Nb-Ta	Li-Be-Nb-Ta-Zr deposit	Medium	Pz2	Li,C.Y., et al., 2021,1995,2019.Development and Research Center, China Geological Survey.
1309	89.525	47.583	Ta-Nb	Li-Be-Nb-Ta-Zr deposit	Medium	Pz2	National Mineral Origin Database of China
1310	83.717	49.399	Ta-Nb	Li-Be-Nb-Ta-Zr deposit	Small		National Mineral Origin Database of China
[truncated: 65,908 more chars]
